# Integrated analysis of inflammatory mRNAs, miRNAs, and lncRNAs elucidates the molecular interactome behind bovine mastitis

**DOI:** 10.1038/s41598-023-41116-2

**Published:** 2023-08-24

**Authors:** Aliakbar Hasankhani, Maryam Bakherad, Abolfazl Bahrami, Hossein Moradi Shahrbabak, Renzon Daniel Cosme Pecho, Mohammad Moradi Shahrbabak

**Affiliations:** 1 Department of Animal Science, College of Agriculture and Natural Resources, University of Tehran, Karaj, Iran; 2 Department of Animal and Dairy Sciences, University of Wisconsin-Madison, Madison, WI USA; 3 Department of Chemistry and Biology, Universidad San Ignacio de Loyola (USIL), Lima, Peru

**Keywords:** Animal breeding, Computational biology and bioinformatics, Genetics, Immunology, Microbiology, Molecular biology, Systems biology, Biomarkers

## Abstract

Mastitis is known as intramammary inflammation, which has a multifactorial complex phenotype. However, the underlying molecular pathogenesis of mastitis remains poorly understood. In this study, we utilized a combination of RNA-seq and miRNA-seq techniques, along with computational systems biology approaches, to gain a deeper understanding of the molecular interactome involved in mastitis. We retrieved and processed one hundred transcriptomic libraries, consisting of 50 RNA-seq and 50 matched miRNA-seq data, obtained from milk-isolated monocytes of Holstein–Friesian cows, both infected with *Streptococcus uberis* and non-infected controls. Using the weighted gene co-expression network analysis (WGCNA) approach, we constructed co-expressed RNA-seq-based and miRNA-seq-based modules separately. Module-trait relationship analysis was then performed on the RNA-seq-based modules to identify highly-correlated modules associated with clinical traits of mastitis. Functional enrichment analysis was conducted to understand the functional behavior of these modules. Additionally, we assigned the RNA-seq-based modules to the miRNA-seq-based modules and constructed an integrated regulatory network based on the modules of interest. To enhance the reliability of our findings, we conducted further analyses, including hub RNA detection, protein–protein interaction (PPI) network construction, screening of hub-hub RNAs, and target prediction analysis on the detected modules. We identified a total of 17 RNA-seq-based modules and 3 miRNA-seq-based modules. Among the significant highly-correlated RNA-seq-based modules, six modules showed strong associations with clinical characteristics of mastitis. Functional enrichment analysis revealed that the turquoise module was directly related to inflammation persistence and mastitis development. Furthermore, module assignment analysis demonstrated that the blue miRNA-seq-based module post-transcriptionally regulates the turquoise RNA-seq-based module. We also identified a set of different RNAs, including hub-hub genes, hub-hub TFs (transcription factors), hub-hub lncRNAs (long non-coding RNAs), and hub miRNAs within the modules of interest, indicating their central role in the molecular interactome underlying the pathogenic mechanisms of *S. uberis* infection. This study provides a comprehensive insight into the molecular crosstalk between immunoregulatory mRNAs, miRNAs, and lncRNAs during *S. uberis* infection. These findings offer valuable directions for the development of molecular diagnosis and biological therapies for mastitis.

## Introduction

Mastitis, characterized by inflammation of the mammary glands, is a complex and multifactorial disease that poses significant economic losses in the dairy industry^1,2^. In the United States and the European Union, annual economic losses due to bovine mastitis are estimated at approximately $2 billion and €2 billion respectively^3–5^. Various pathogens, including gram-negative coliforms (e.g., *Escherichia coli*), gram-positive streptococci (e.g., *Streptococcus uberis*), and staphylococci (e.g., *Staphylococcus aureus*), can cause mastitis in high-producing dairy cows, leading to a wide range of disease manifestations from subclinical to severe and life-threatening infections^6–8^. Among these pathogens, *S. uberis* is the most prevalent species of mastitis-causing pathogens in Europe and North America^9^.

Evidence suggests that bovine mastitis, as a local bacterial infection, is associated with a robust inflammatory response^10^. Upon infection, pathogens such as *S. uberis* enter the udder through the teat canal and interact with mammary gland epithelial cells and resident immune cells such as monocytes. This interaction involves the recognition of pathogen-associated molecular patterns (PAMPs) by pattern recognition receptors (PRRs) on the cell surfaces of both cell types^11,12^. The over-stimulation of PRRs triggers the secretion of proinflammatory cytokines, including interleukins (ILs), tumor necrosis factors (TNFs), and chemokines, initiating a local pathological inflammatory response in the mammary glands^11,13,14^. Simultaneously, systemic immune cells such as neutrophils and monocytes are recruited to the site of infection/inflammation for antibacterial activities, infection resolution, and inflammation control^15^. The somatic cell count (SCC), which includes immune cells like monocytes and neutrophils, as well as mammary gland epithelial cells, can be measured in milk samples and serves as a potential tool to monitor the inflammatory status of the mammary gland, predict mastitis, and differentiate between chronically infected and non-infected animals^16,17^. A SCC greater than 200,000 cells/ml is considered diagnostic and a hallmark of mastitis^18,19^. Despite extensive research aimed at developing effective diagnostics, prevention, and treatment strategies, mastitis remains an important health concern in both human and veterinary medicine.

High-throughput transcriptome-based techniques such as gene expression microarrays and RNA sequencing (RNA-seq) have been widely used in biological, medical, clinical, and pharmaceutical research to explore gene expression profiles, identify biomarkers, and facilitate drug discovery^20^. Previous studies have employed microarray or RNA-seq techniques to investigate mastitis in different tissues, including blood, mammary epithelial cells, and liver, revealing increased expression of inflammatory mediators in infected samples^21–24^. These findings have been validated using other experimental analytical methods such as quantitative real-time PCR (qRT-PCR)^25^.

Emerging evidence suggests that non-coding regions of the genome, including non-coding RNAs such as microRNAs (miRNAs) and long non-coding RNAs (lncRNAs), play crucial regulatory roles in various aspects of innate and adaptive immunity and inflammation^26^. miRNAs are small non-coding RNAs (~ 22 nucleotides) that post-transcriptionally regulate gene expression by binding to target mRNAs^27^. Several studies have reported differentially expressed (DE) miRNAs in response to *S. uberis* infection, such as *bta-miR-200c*, *bta-miR-182*, *bta-miR-30a-5p*, *bta-miR-146b*, and *bta-miR-125a*, which are key amplifiers of monocyte inflammatory response networks^7,28^. LncRNAs, on the other hand, are longer non-coding RNAs (> 200 bp) that regulate gene expression through diverse mechanisms at the transcriptional, post-transcriptional, and translational levels. They have been implicated in immune responses and inflammation^29,30^. In this regard, Wang et al.^31^ identified 53 DE lncRNAs with inflammatory functions in bovine mammary epithelial cells using a high-throughput infection model of mastitis. However, existing research primarily focuses on individual RNA species (mRNAs, miRNAs, and lncRNAs) and fails to consider the effects of RNA clusters^32^.

Given the complex phenotype of mastitis susceptibility, with genes, proteins, miRNAs, lncRNAs, and other RNA species interacting in intricate molecular networks during infection, there is a need to explore comprehensive regulatory networks to gain insights into the onset and development of mastitis^33^. Weighted gene co-expression network analysis (WGCNA) is an integrated network-based approach widely used in bioinformatics applications for microarray or RNA-seq datasets, providing a systems-level understanding without information loss^34–36^. WGCNA identifies highly correlated nodes (modules) based on expression patterns and calculates intramodular gene connectivity, thereby pinpointing highly connected nodes, or "hubs," that play central functional roles associated with network biological properties^35^. Module-trait relationships, a popular method within WGCNA, examine the correlation between co-expression modules (genome level) and external clinical traits (phenome level), allowing for deep investigations of various diseases^37–40^.

Despite the efficiency of WGCNA's module-trait relationships method, to our knowledge, no study has explored the correlation between functional modules and hallmark clinical features of bovine mastitis, such as SCC and total bacteria count (TBC) in the milk of *S. uberis*-infected animals. Moreover, understanding the crosstalk between immunoregulatory mRNAs, miRNAs, and lncRNAs during mastitis can provide valuable insights into the underlying molecular mechanisms, leading to the development of drug treatment or disease prevention therapies. Therefore, we hypothesized that constructing an integrated regulatory network incorporating various RNA species, including lncRNAs, miRNAs, and mRNAs (genes and transcription factors), and examining their molecular interactome, can provide systemic insights into the regulatory elements involved in the onset and development of mastitis. Our research paper aimed to provide a comprehensive understanding of the interconnectivity of key regulatory elements in bovine mastitis, contributing to novel insights into molecular mechanisms and the development of promising diagnostic biomarkers and therapeutic targets for subclinical and clinical mastitis cases.

## Results

### Transcriptome data preprocessing and analysis

**Figure 1:**
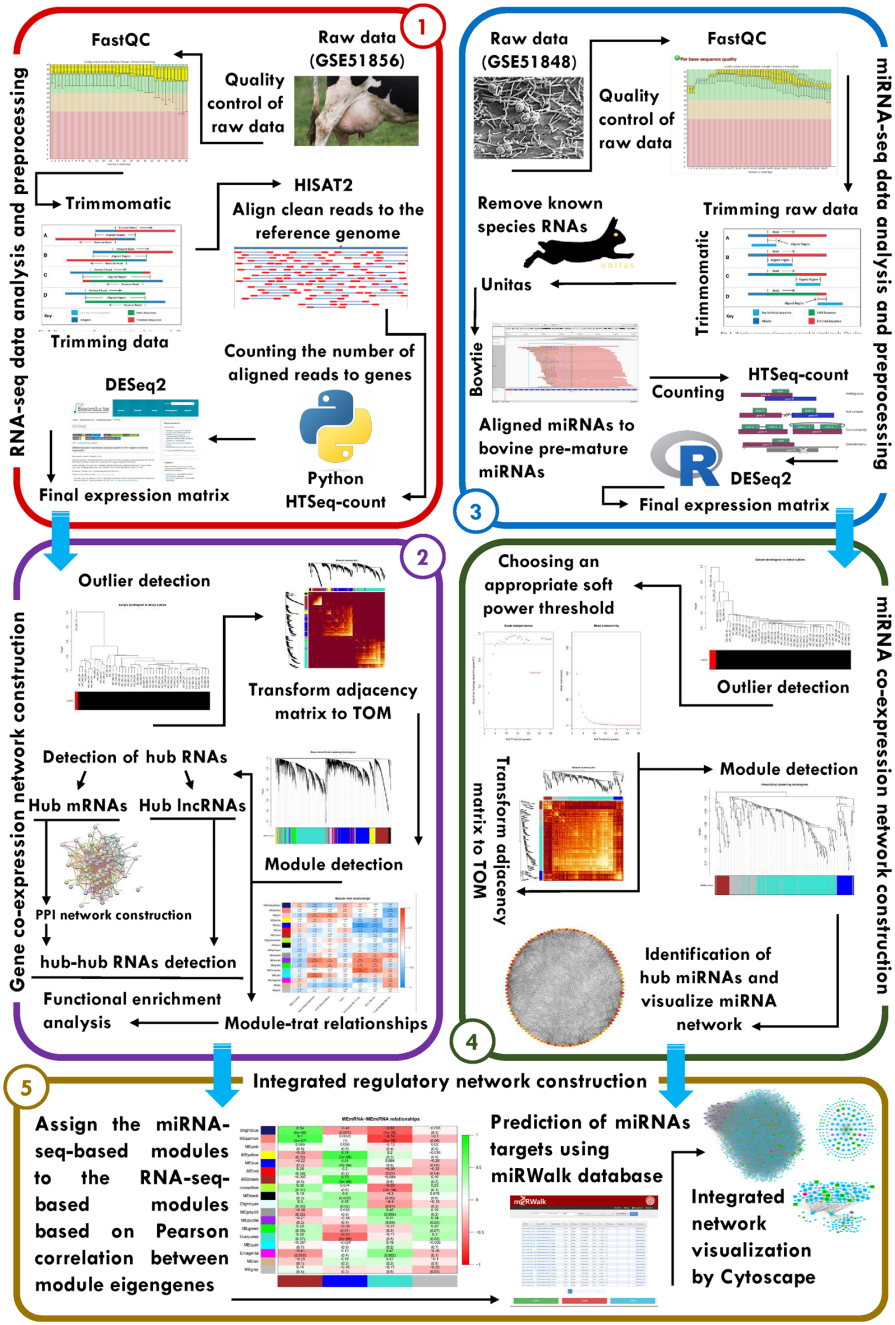
Schematic step-by-step pipeline used for construction an integrated regulatory network.

An overview of the step-by-step pipeline used in this study for the different stages of the analysis is schematically described in Fig. 1. A total of 1,827,194,323 raw RNA-seq reads and 926,360,382 matched raw miRNA-seq reads were retrieved and processed from milk-isolated CD14 + monocytes of 44 Holstein–Friesian cows, including 19 *S. uberis*-infected and 25 non-infected samples. The average raw reads per sample were 42 million for RNA-seq and 21 million for miRNA-seq data. After preprocessing the raw reads, 1,713,184,434 high quality RNA-seq clean reads and 791,307,783 high-quality miRNA-seq clean reads were obtained. The results of reads mapping showed that on average, 93% of RNA-seq clean reads were uniquely aligned to the bovine reference genome, and 88% of miRNA-seq clean reads were aligned to pre-mature miRNA sequences. Detailed information on the preprocessing and analysis steps of RNA-seq and miRNA-seq data can be found in Supplementary File S1. To ensure the minimization of sampling noise and increase the reliability of co-expression network construction, several parameters were applied to remove low-expressed and low-variance RNAs. Consequently, a normalized RNA-seq-based expression matrix comprising 9,263 different RNAs (including 8,564 genes, 571 TFs, and 128 lncRNAs) and a normalized miRNA-seq-based expression matrix comprising 328 miRNAs were generated.

### Weighted co-expression network construction and module detection

**Figure 2:**
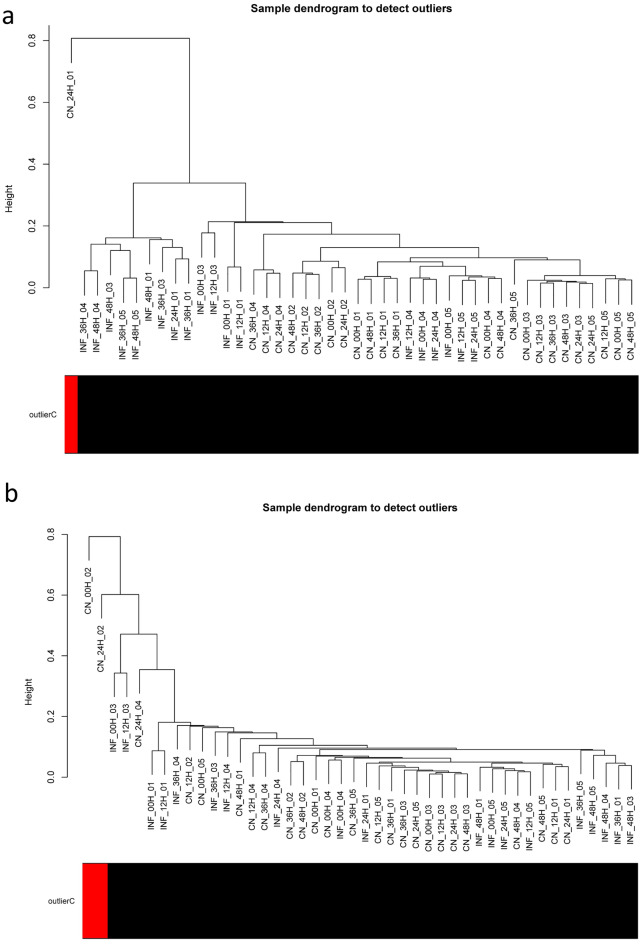
Sample clustering to detect outliers in the (**a**) RNA-seq and (**b**) miRNA-seq samples. The statistics of the adjacency matrices of samples indicated that one RNA-seq sample (GSM1254086) and two miRNA-seq samples (GSM1253778 and GSM1253780) had a standardized connectivity score <  − 2.5 (red color) and were excluded from downstream analyses.

**Figure 3:**
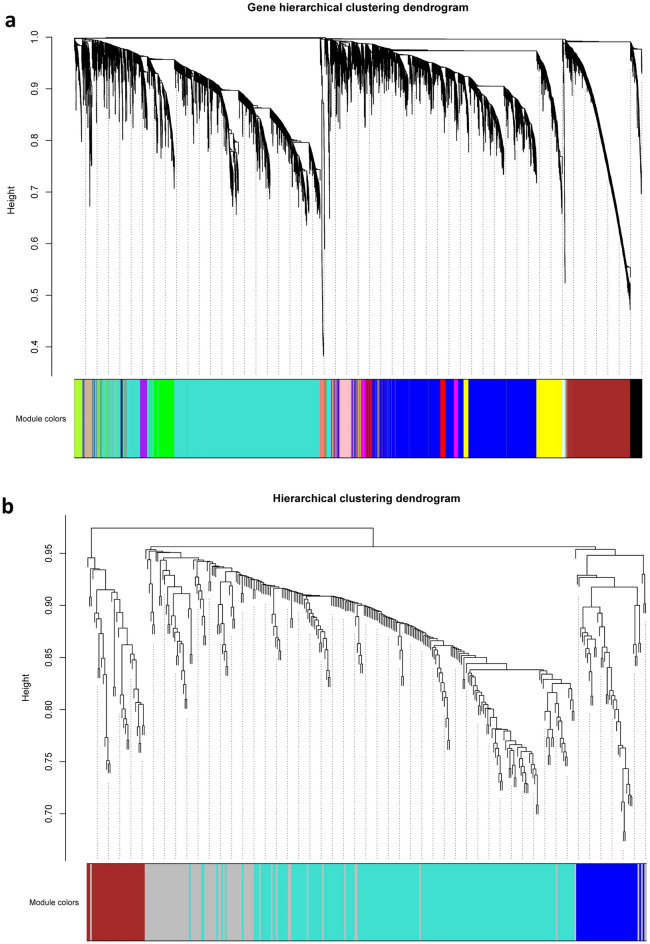
Hierarchical clustering dendrogram of (**a**) mRNAs and lncRNAs and (**b**) miRNAs. A total of 17 and 3 RNA-seq-based and miRNA-seq-based modules were identified based on the TOM dissimilarity (1-TOM) through hierarchical clustering analysis and dynamic hybrid tree cutting algorithms. The x-axis represents the RNAs and the y-axis represents the co-expression distance. The branches indicate the modules which was labeled with a specific color using the static tree cutting method. the grey color indicates uncorrelated RNAs.

To gain a deeper understanding of the molecular regulatory mechanisms underlying bovine mastitis and establish novel insights into the molecular interactome during *S. uberis* infection, separate weighted co-expression networks were constructed using the normalized and filtered RNA-seq-based and miRNA-seq-based expression matrices. Outlier data were identified and excluded using distance-based adjacency metrics. One RNA-seq sample (GSM1254086) had a standardized connectivity score <  − 2.5 and was identified as outlier data and removed (Fig. 2a; Supplementary File S2). Additionally, GSM1253778 and GSM1253780 miRNA-seq samples were also identified as outliers and removed (Fig. 2b; Supplementary File S2). Soft threshold powers (*β*) were determined to ensure the scale-free topology of the networks. *β* values of 17 and 6 were calculated for RNA-seq-based and miRNA-seq-based matrices, respectively, achieving a scale-free topology fitting index (R^2^) ≥ 0.80 (Supplementary File S3). Weighted co-expression networks were constructed, and co-expression modules were identified through hierarchical clustering analysis and dynamic hybrid tree-cutting algorithm based on TOM dissimilarity (1-TOM) and labeled with specific colors as a branch of the hierarchical clustering dendrogram by the WGCNA R package (Fig. 3). Seventeen RNA-seq-based modules with an average size of 531 RNAs were identified. The turquoise module was the largest, containing 2,980 RNAs (2,767 genes, 201 TFs, and 12 lncRNAs), while the grey60 module was the smallest, with 43 genes (without TFs and lncRNAs). Three miRNA-seq-based modules with an average size of 85 miRNAs were identified. The turquoise and brown modules as the largest and smallest miRNA-seq-based modules, containing 183 and 33 miRNAs, respectively. Additionally, 231 uncorrelated RNAs were identified in the RNA-seq-based modules, and 74 uncorrelated RNAs were identified in the miRNA-seq-based modules. Detailed information on RNA-seq and miRNA-seq-based modules is presented in Supplementary File S4.

### Module-trait relationships analysis

**Figure 4:**
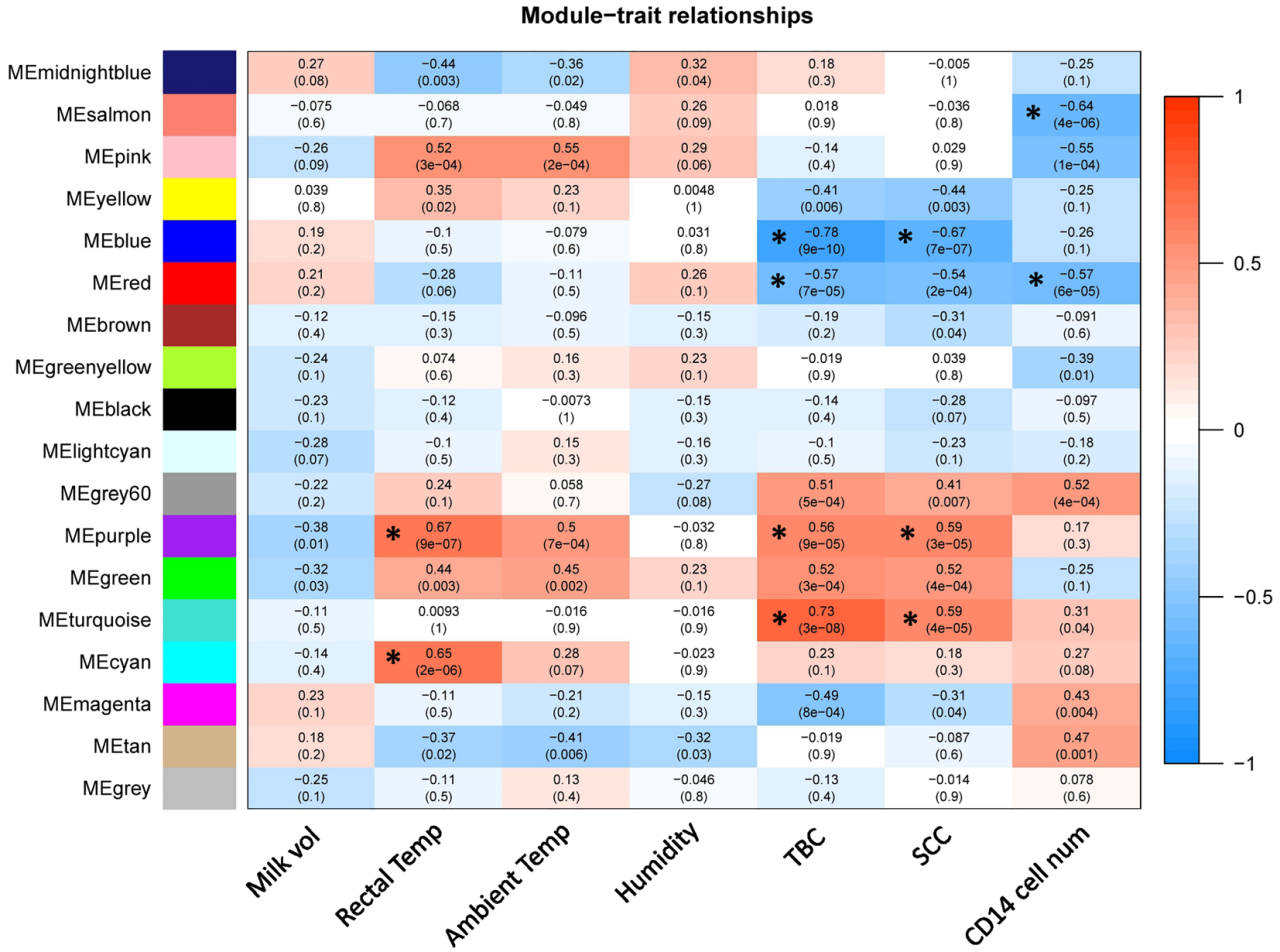
Module–trait relationship analysis between RNA-seq-based modules and clinical traits of mastitis. The blue and red colors indicate strong negative and strong positive correlation, respectively. Rows represent module eigengene and columns indicate clinical traits of mastitis. Asterisks corresponds to significant highly correlated values. Milk volume (in Liters), rectal temperature (in Fahrenheit), ambient temperature (in Celsius), humidity (%), total bacterial counts (TBC; per 10 mL), somatic cell count (SCC; per mL), and CD14-cell-number (per mL).

To explore the association of the genome with phenotypic traits of mastitis and identify key regions regulating the clinical signs of this disorder, module-trait relationships analysis was performed for RNA-seq-based modules. Among the 17 identified RNA-seq-based modules, 2, 4, 3, and 2 modules showed significantly high correlations with rectal temperature, TBC, SCC, and CD14 cell number, respectively (Fig. 4; Supplementary File S5). The MEcyan (*R* = 0.65, *p* = 2e-06) and MEpurple (*R* = 0.67, *p* = 9e-07) modules were significantly highly-correlated with rectal temperature (Fig. 4). Moreover, the MEturquoise (*R* = 0.73, *p* = 3e-08), MEpurple (*R* = 0.56, *p* = 9e-05), MEred (*R* = -0.57, *p* = 7e-05), and MEblue (*R* = -0.78, *p* = 9e-10) modules were significantly highly-correlated with TBC and MEturquoise (*R* = 0.59, *p* = 4e-05), MEpurple (*R* = 0.59, *p* = 3e-05), and MEblue (*R* = -0.67, *p* = 7e-07) modules were also significantly highly-correlated with SCC (Fig. 4). Additionally, the MEred (*R* = -0.57, *p* = 6e-05) and MEsalmon (*R* = -0.64, *p* = 4e-06) modules were significantly highly-correlated with CD14 cell number (Fig. 4). Notably, the turquoise RNA-seq-based module, the largest module identified (2980 RNAs), displayed the highest significant positive correlation with the hallmark features of mastitis, SCC, and TBC (Fig. 4).

### Functional enrichment analysis and identification of RNA-seq-based module of interest

**Figure 5:**
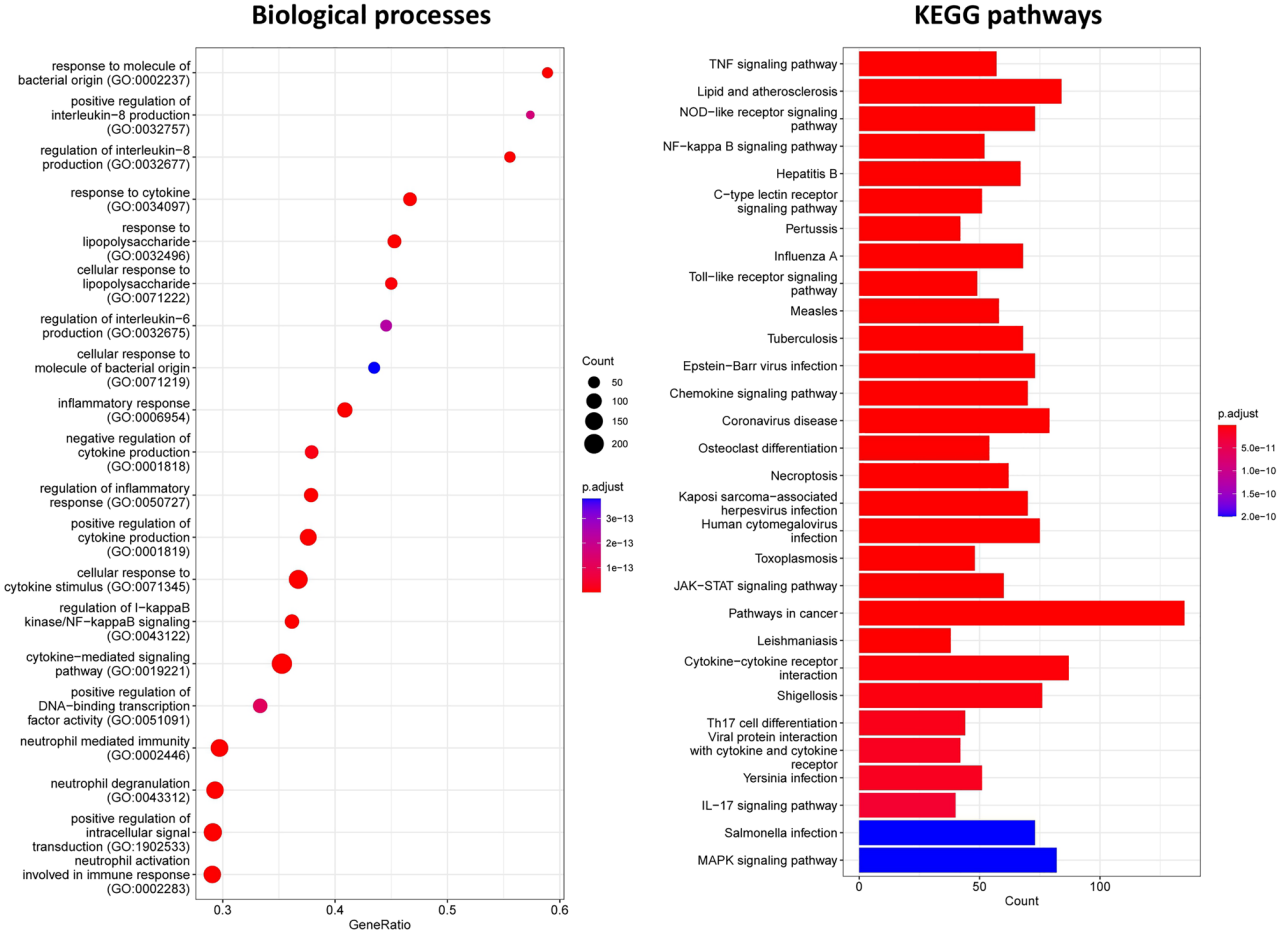
Top significant biological processes and KEGG pathways of the turquoise RNA-seq-based module. The y-axis represent significant terms and x-axis represents enriched genes. Moreover, the color in both plots indicates adjusted *p*-value.

Functional enrichment analysis was performed to describe the biological differences, putative functions, and specific molecular mechanisms of the significant highly-correlated RNA-seq-based modules. A total of 871 biological processes and 153 KEGG pathways were significantly enriched in the cyan, purple, red, blue, and salmon modules. Interestingly, the turquoise module exhibited the highest functional enrichment rate compared to the other modules, with 669 significant biological processes and 111 enriched KEGG pathways. Moreover, the salmon module with two biological processes and one KEGG pathway had the least enriched functional terms compared to others. Comprehensive details of the results of functional enrichment analysis of significant highly-correlated RNA-seq-based modules are provided in Supplementary File S6. Based on the functional enrichment analysis results, most of the significant highly-correlated modules including cyan, purple, blue, and salmon were enriched in common cellular processes such as cell cycle and metabolism, DNA replication, gene expression, rRNA processing, ribosome biogenesis, and translation. Whereas, surprisingly, the largest significant highly-correlated RNA-seq-based module, the turquoise module, which had the highest positive correlation with mastitis phonotypic/clinical measurements and the highest functional enrichment rate, was highly enriched in biological processes and KEGG pathways associated with the host immune response, cell death, inflammatory response, and *S. uberis* pathogenesis. Some of these terms included “Toll-like receptor signaling pathway”, “MAPK signaling pathway”, NF-kappa B signaling pathway, “TNF signaling pathway”, “cytokine-mediated signaling pathway (GO:0,019,221)”, “Chemokine signaling pathway”, “positive regulation of nitric-oxide synthase biosynthetic process (GO:0,051,770)”, “positive regulation of reactive oxygen species metabolic process (GO:2,000,379)”, “positive regulation of acute inflammatory response (GO:0,002,675)”, “Focal adhesion”, “positive regulation of leukocyte cell–cell adhesion (GO:1,903,039)”, “positive regulation of leukocyte chemotaxis (GO:0,002,690)”, “positive regulation of lymphocyte chemotaxis (GO:0,140,131)”, “neutrophil mediated immunity (GO:0,002,446)”, “Neutrophil extracellular trap formation”, “positive regulation of phagocytosis (GO:0,050,766)”, “Necroptosis”, “Apoptosis”, “Ferroptosis”, “positive regulation of programmed cell death (GO:0,043,068)”, “T cell receptor signaling pathway”, “B cell receptor signaling pathway”, “Th17 cell differentiation”, “positive regulation of T cell cytokine production (GO:0,002,726)”, “positive regulation of T-helper 1 type immune response (GO:0,002,827)”, “negative regulation of metabolic process (GO:0,009,892)”, “negative regulation of lipid storage (GO:0,010,888)”, “type I interferon signaling pathway (GO:0,060,337)”, and “interferon-gamma-mediated signaling pathway (GO:0,060,333)” (Supplementary File S6). Figure 5 shows the top significant biological processes and KEGG pathways of the turquoise RNA-seq-based module. These results suggest the potential role of the turquoise RNA-seq-based module in the host-*S. uberis* interactions and the immunopathogenesis of mastitis, so it can be considered a promising essential module to dissect the underlying molecular/pathological regulatory mechanisms of mastitis. Therefore, we narrow down the subsequent analysis to deeply explore the pathological mechanisms and evaluate the molecular interactome of the turquoise RNA-seq-based module as the module of interest in this study.

### Assigning the miRNA-seq-based modules to the RNA-seq-based modules and miRNA target prediction

**Figure 6:**
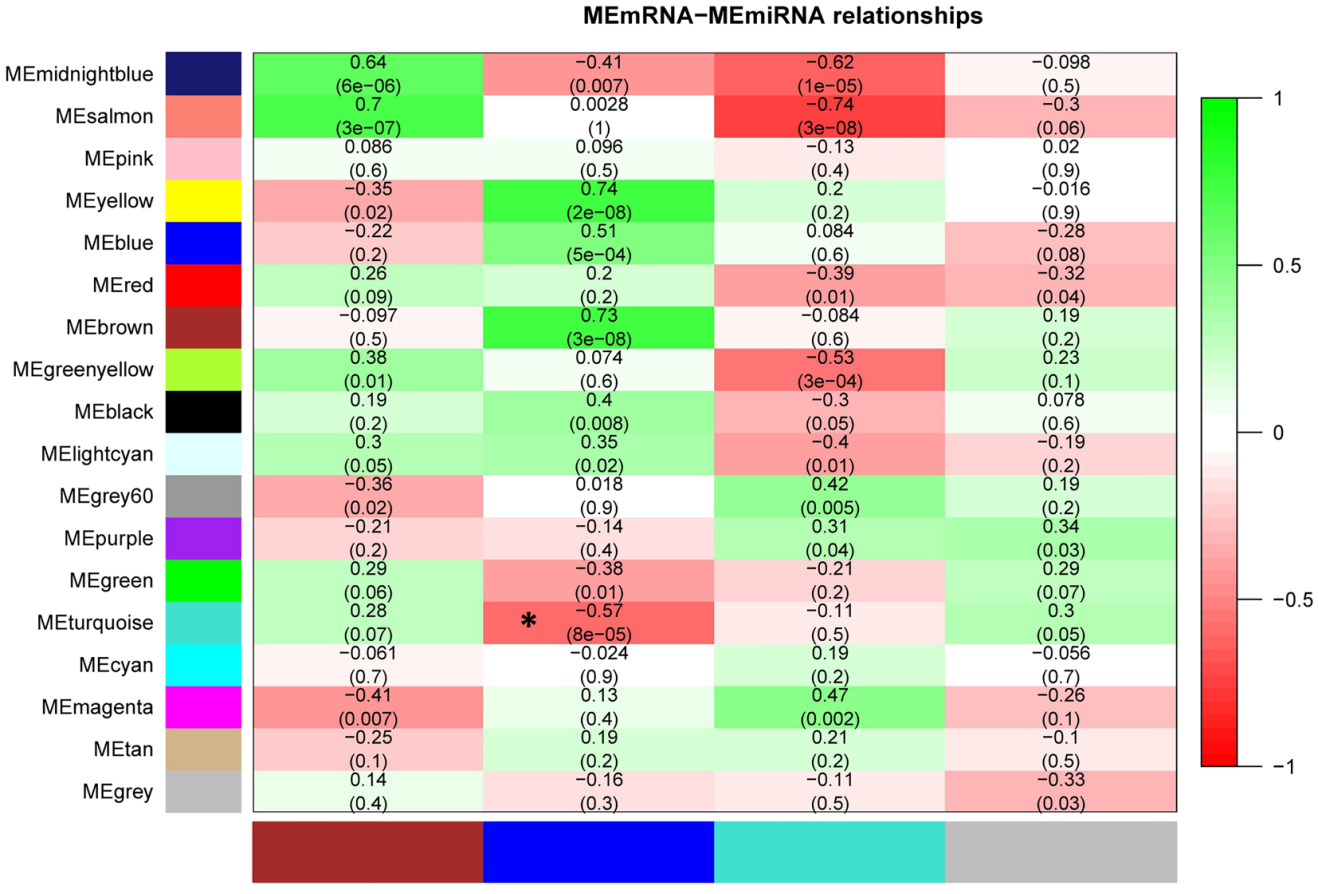
Assigning the miRNA-seq-based modules to the RNA-seq-based modules. Rows represent RNA-seq-based modules and columns represent miRNA-seq-based modules. The green and red colors indicate strong positive and strong negative correlation, respectively. Asterisks corresponds to significant highly-correlated values. As shown, the blue miRNA-seq-based module negatively regulates (*R* = -0.57; *p*-value 8e-05) the turquoise RNA-seq-based module.

Module-trait relationships and functional enrichment analysis revealed that the turquoise RNA-seq-based module as the module of interest in this study, plays a key role during mastitis and is involved in active immunological-inflammatory-pathological networks during *S. uberis* infection. Therefore, to understand which of the miRNA-seq-based modules post-transcriptionally regulates the turquoise RNA-seq-based module and also to obtain an in-depth molecular interactome (mRNAs-miRNAs-lncRNAs) of the underlying immunological-inflammatory-pathological processes of mastitis, miRNA-seq-based modules were assigned to RNA-seq-based modules, and then target prediction analysis of selected miRNAs was performed. Here, the blue miRNA-seq-based module consisting of 38 miRNAs (Supplementary File S4) was found to negatively regulate (*R* = -0.57, *p* = 8e-05) the turquoise RNA-seq-based module and therefore could be a potential regulator of this module (Fig. 6; Supplementary File S7). Furthermore, in agreement with these results, target prediction analysis revealed that miRNAs of the blue miRNA-seq-based module strongly target RNAs (genes, TFs, and lncRNAs) of the turquoise RNA-seq-based module. More information from the results of target prediction analysis and interactions between blue miRNA-seq-based module miRNAs and turquoise RNA-seq-based module RNAs (mRNAs and lncRNAs) are available in Supplementary File S8.

### Detection of hub-hub RNAs in the RNA-seq-based module of interest

**Figure 7:**
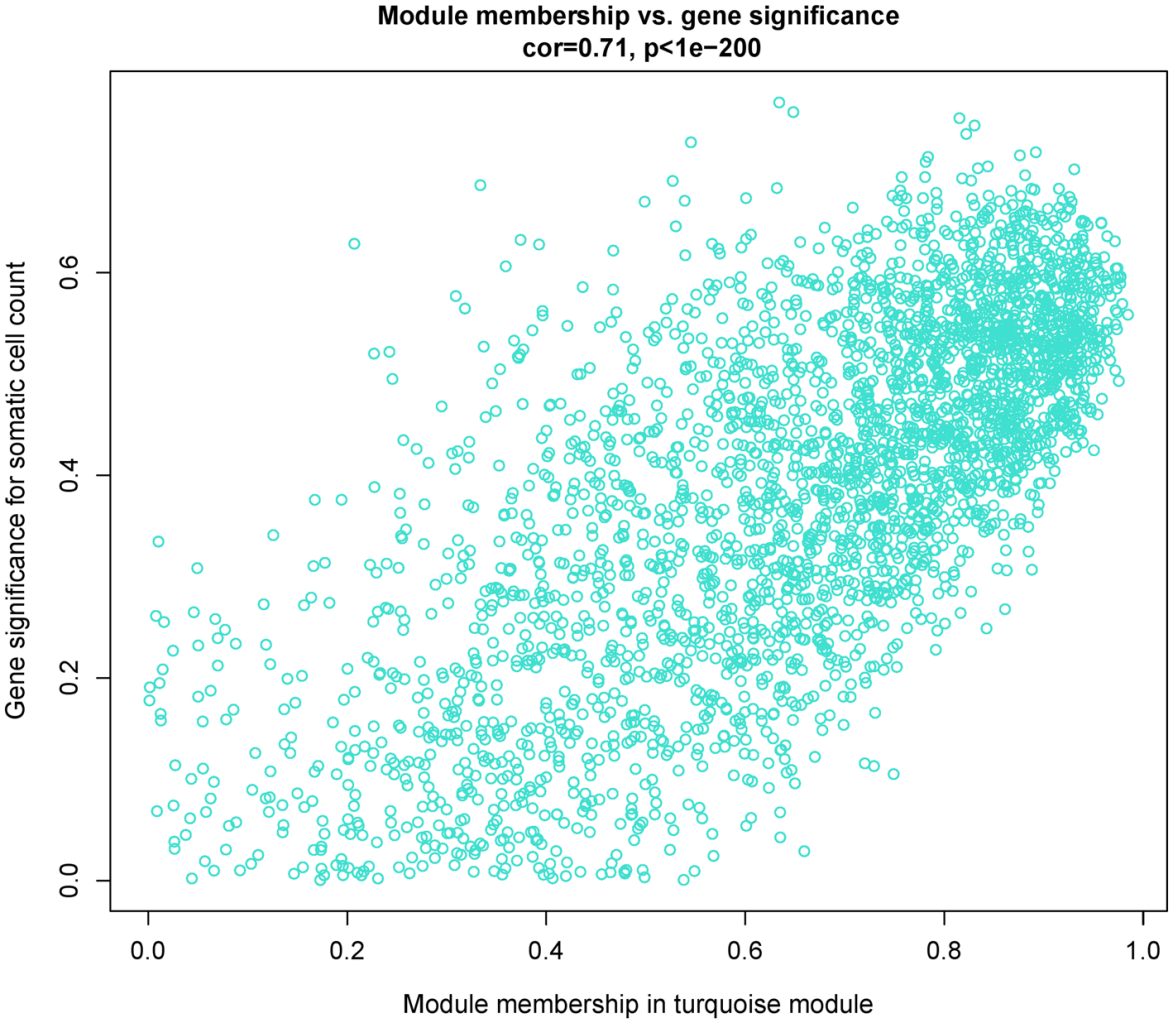
Scatterplots of module membership (MM) versus gene significance (GS) plots for somatic cell counts (SCC) as a hallmark of mastitis in the turquoise RNA-seq-based module. The results indicated a significant strong correlation between GS and MM criteria. These measurements confirm that the significant RNAs with the clinical features of mastitis are often the central and hub RNAs in the turquoise RNA-seq-based module.

To better understand the underlying molecular mechanisms of immunological-inflammatory-pathological processes of mastitis and to identify the key regulators involved in these processes, intramodular hub RNAs (genes, TFs, and lncRNAs) of the turquoise RNA-seq-based module were identified using the MM criterion calculated by WGCNA R package. A total of 1779 hub RNAs including 1637 hub genes, 141 hub TFs, and 1 hub lncRNA were identified in the turquoise RNA-seq-based module (Supplementary File S9). We also calculated the GS criterion for RNAs of the turquoise RNA-seq-based module to investigate and validate the association of hub RNAs (identified by MM criterion) with clinical measurements of SCC as one of the main hallmarks of mastitis. The results indicated a significantly strong correlation (*R* = 0.71, *p* < 1e − 200) between GS and MM criteria (Fig. 7). In other words, these measurements confirm that the significant RNAs with the clinical features of mastitis are often the center and hub RNAs in the turquoise RNA-seq-based module. Comprehensive information from GS for SCC related to the turquoise RNA-seq-based module is available in Supplementary File S10. Moreover, we identified 19 hub miRNAs in the blue miRNA-seq-based module as the main post-transcriptional regulator of the turquoise RNA-seq-based module (Supplementary File S11). Then, to investigate the network density and molecular connections at the translational level and extraction of the PPI network, the co-expressed hub mRNAs (genes and TFs) of the turquoise RNA-seq-based module were subjected to the STRING database. Interestingly, the resulting PPI network of co-expressed hub mRNAs of the turquoise RNA-seq-based module was densely connected (number of nodes: 1542, number of edges: 13,854, average node degree: 18, and PPI *p*-value < 1.0e-16), indicating close interactions of their encoding proteins. Then, the co-expressed hub mRNA-based PPI network was merged with the predicted interactions of the turquoise RNA-seq-based module calculated by WGCNA and assessed to identify hub-hub RNAs. Finally, the top 50 hub-hub RNAs with the highest MCC score were identified in the turquoise RNA-seq-based module, including 37 genes, 12 TFs, and 1 lncRNA (Table 1). Indeed, these RNAs were identified as central RNAs in both WGCNA-calculated co-expression and PPI networks and can be considered as potential candidates for understanding the etiology of complex diseases such as mastitis, promising diagnostic biomarkers, and valuable prognostic and therapeutic targets for the development of effective strategies for the management/control of mastitis.

**Table 1:** List of the hub-hub RNAs including 37 genes, 12 TFs, and 1 lncRNA identified in the turquoise RNA-seq-based module. MM, module memberships; MCC, maximal clique centrality; TF, transcription factor.

Hub-hub RNAs	RNA type	MM	MM *p*-value	MCC score
*CDC42SE1*	lncRNA	0.80	5.57E−11	4.70E + 20
*TNF*	Protein coding	0.67	6.32E−07	4.54E + 20
*IL6*	Protein coding	0.87	2.39E−14	4.52E + 20
*IL10*	Protein coding	0.76	1.71E−09	4.51E + 20
*STAT3*	TF	0.97	3.60E−29	4.39E + 20
*IL15*	Protein coding	0.74	7.22E−09	4.22E + 20
*CD40*	Protein coding	0.89	3.18E−16	3.83E + 20
*STAT1*	TF	0.73	2.10E−08	3.37E + 20
*IL1B*	Protein coding	0.84	7.88E−13	3.31E + 20
*TLR4*	Protein coding	0.85	4.96E−13	3.31E + 20
*CXCL8*	Protein coding	0.68	4.94E−07	3.29E + 20
*ICAM1*	Protein coding	0.83	3.13E−12	3.28E + 20
*TLR2*	Protein coding	0.92	8.20E−19	3.28E + 20
*PTPRC*	Protein coding	0.97	7.46E−28	3.02E + 20
*CD274*	Protein coding	0.87	1.71E−14	2.99E + 20
*CD80*	Protein coding	0.87	3.33E−14	2.99E + 20
*CD44*	Protein coding	0.92	9.88E−19	2.88E + 20
*IL18*	Protein coding	0.87	8.20E−15	2.48E + 20
*CCR2*	Protein coding	0.79	2.57E−10	2.25E + 20
*CXCR4*	Protein coding	0.77	9.73E−10	2.22E + 20
*JAK2*	Protein coding	0.96	2.26E−24	1.90E + 20
*CD69*	Protein coding	0.92	2.80E−18	1.86E + 20
*SELL*	Protein coding	0.94	2.51E−21	1.78E + 20
*STAT2*	TF	0.91	6.13E−18	1.26E + 20
*STAT6*	TF	0.88	4.54E−15	1.23E + 20
*JAK1*	Protein coding	0.84	8.14E−13	1.23E + 20
*TYK2*	Protein coding	0.83	2.80E−12	1.23E + 20
*JAK3*	Protein coding	0.92	9.19E−19	1.23E + 20
*STAT5B*	TF	0.82	7.25E−12	1.22E + 20
*STAT5A*	TF	0.92	3.38E−19	1.22E + 20
*IL4R*	Protein coding	0.94	4.46E−25	1.22E + 20
*IL12A*	Protein coding	0.85	1.88E−13	1.19E + 20
*IL23A*	Protein coding	0.91	4.89E−18	1.19E + 20
*STAT4*	TF	0.68	4.42E−07	1.17E + 20
*IL2RG*	Protein coding	0.94	7.37E−22	1.16E + 20
*CSF1*	Protein coding	0.92	4.96E−19	1.14E + 20
*SOCS1*	Protein coding	0.80	7.79E−11	1.14E + 20
*IL2RA*	Protein coding	0.88	4.95E−15	1.11E + 20
*VEGFA*	Protein coding	0.94	3.00E−21	1.03E + 20
*MYD88*	Protein coding	0.79	2.86E−10	1.01E + 20
*CCR5*	Protein coding	0.84	1.78E−12	6.89E + 19
*SOCS3*	Protein coding	0.95	9.91E−23	6.32E + 19
*RELA*	TF	0.91	1.16E−17	3.09E + 19
*NFKB1*	TF	0.94	5.09E−22	3.07E + 19
*JUN*	TF	0.67	7.48E−07	3.00E + 19
*IL1A*	Protein coding	0.73	2.75E−08	2.79E + 19
*NFKB2*	TF	0.92	3.54E−19	2.28E + 19
*IRF1*	TF	0.80	9.34E−11	2.20E + 19
*PTGS2*	Protein coding	0.86	7.61E−14	2.17E + 19
*DDX58*	Protein coding	0.75	5.87E−09	1.98E + 19

### Integrated regulatory network construction

**Figure 8:**
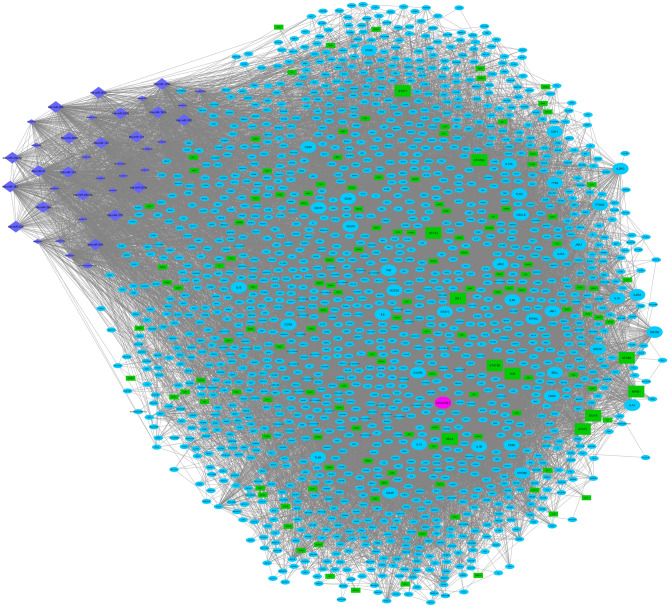
Integrated regulatory network of the turquoise RNA-seq-based and blue miRNA-seq-based modules. Small circles and rectangles represent hub genes and hub transcription factors (TFs) of the turquoise RNA-seq-based module, respectively. Small diamonds represent regulatory miRNAs of the blue miRNA-seq-based module. On the other hand, large circles and rectangles represent hub-hub genes and hub-hub TFs of the turquoise RNA-seq-based module, respectively. large diamonds represent hub miRNAs of the blue miRNA-seq-based module. Additionally, the large pink octagon represents the only hub-hub lncRNA in the turquoise RNA-seq-based module. Cytoscape software (version 3.7.1) (https://cytoscape.org/) was used to generate this figure.

To generate the molecular interactome of RNA species (mRNA-miRNA-lncRNA) involved in the important underlying processes of mastitis, especially inflammatory response, and to construct an integrated immunoregulatory network, the interactions generated from STRING-PPI, WGCNA-calculated co-expressed hub mRNAs, and WGCNA-calculated co-expressed hub lncRNAs of the turquoise RNA-seq-based module were combined with WGCNA-calculated interactions of co-expressed miRNAs of the blue miRNA-seq-based module and target prediction results. The summary of the constructed integrated regulatory network is provided in Supplementary File S12. The molecular interactome of the integrated immunoregulatory network involved in bovine mastitis is shown in Fig. 8. Moreover, the constructed integrated regulatory sub-networks of important inflammatory hub-hub genes during mastitis are shown in Supplementary File S13.

## Discussion

In this study, signed weighted gene co-expression networks were constructed and then, 17 and 3 RNA-seq-based (including genes, TFs, and lncRNAs) and miRNA-seq-based (including miRNAs) modules were identified, respectively through the WGCNA approach. Technically, signed networks distinguish modules based on biological function with high accuracy and obtain more significant terms associated with co-expression patterns^32^. Then, module-trait relationships analysis of the WGCNA was performed between RNA-seq-based modules and clinical hallmarks of mastitis including SCC and TBC in order to extract significant highly-correlated RNA-seq-based modules with the aforementioned clinical measurements. Interestingly, the results indicated that the turquoise RNA-seq-based module, which was the largest co-expression module, had the highest enrichment rate and the highest significant positive correlation with SCC and TBC, and was highly enriched in the pathways related to inflammation and immunopathogenesis of mastitis. Moreover, it was found that the blue miRNA-seq-based module has a negative correlation with the turquoise RNA-seq-based module, indicating the inverse interactions between these two modules. Consequently, an integrated regulatory network comprising immunoregulatory mRNAs-miRNAs-lncRNAs was constructed from the turquoise RNA-seq-based module and its assigned miRNA-seq-based module, the blue module.

### Functional enrichment analysis

Overall, our findings indicated that during *S. uberis* infection, bacterial PAMPs are recognized by surface PRRs, particularly TLRs such as *TLR2* and *TLR4*. This recognition triggers a cascade of downstream inflammatory signals, ultimately leading to the activation of NF-kappa B signaling, MAPK signaling, and JAK-STAT signaling pathways^41,42^. The activation of these pathways results in the secretion of various cytokines and chemokines, especially pro-inflammatory cytokines such as *TNF-α*, *IL6*, and *IL1β* and the recruitment of inflammatory cells to the site of infection^42^. Previous research on clinical and subclinical cases of mastitis has highlighted that *S. ubris* infection induces acute/chronic inflammation in mammary glands through prolonged stimulation of inflammatory signals^43,44^. Furthermore, in agreement with these results, the activation of other types of PRRs, including NOD-like, C-type lectin, and RIG-I-like receptors, has been repeatedly reported in clinical cases of mastitis caused by *S. uberis* infection^7,45,46^.

Functional enrichment analysis revealed several important biological processes enriched in the turquoise RNA-seq-based module. The process “positive regulation of reactive oxygen species metabolic process (GO:2,000,379)” was observed, indicating the involvement of reactive oxygen species (ROS) production in microbial killing during the initiation and recovery of mastitis^47,48^. However, excessive accumulation of ROS can lead to oxidative stress, which plays a major significant role in mediating uncontrolled inflammatory responses and causing tissue damage^49^. Additionally, terms such as “mTOR signaling pathway” and “PI3K-Akt signaling pathway” were enriched in the turquoise RNA-seq-based module. These pathways have been identified as critical in coordinating the inflammatory response mediated by TLRs/NF-κB^50,51^. Recent studies have reported that crosstalk between the PI3K/Akt/mTOR and TLRs/NF-κB axes promotes inflammation in mammary epithelial cells during *S. uberis* infection^52^.

Furthermore, the co-regulated genes in the turquoise module showed high enrichment in processes related to innate immunity. During bacterial infections like mastitis, a massive influx of polymorphonuclear leukocytes occurs at the site of inflammation in infected mammary glands, aiming to control bacterial spread and resolve the infection^53^. Focal adhesion and cell adhesion molecules are crucial for leukocyte cellular migration to the site of inflammation^54^. Neutrophils constitute the majority of immune cells recruited to the site of inflammation during *S. uberis* infection and contribute to the resolution of the inflammation through phagocytosis or the formation of neutrophil extracellular traps (NETs)^15^. Additionally, systemic monocytes are secreted from the bone marrow into the circulatory system and subsequently recruited to the site of infection, where they differentiate into macrophages and dendritic cells (DCs)^55^. However, studies have shown that the massive recruitment of immune cells like neutrophils and monocytes to the site of infection, prolonged phagocytosis, and NET formation are directly associated with increased SCC in the milk of infected animals, mammary gland damage, and an increased risk of clinical mastitis^56–59^.

Functional enrichment analysis also indicated that genes in the turquoise RNA-seq-based module were highly enriched in processes related to different types of cell death. Apoptosis, a type of programmed cell death, has been observed to be induced in response to mastitis-causing pathogens such as *E. coli*^60^ and *S. aureus*^61^. Moreover, in a nonspecific infection model of mastitis, healthy quarters showed a lower percentage of cell apoptosis^55^. Apoptosis after infection with mastitis-causing pathogens is known to be directly associated with bovine mammary epithelial cell damage and subsequent decreased milk production^62^.

Necroptosis, a newly discovered pathway of regulated necrosis associated with inflammation, plays a key role in the pathogenesis of many inflammatory diseases^63^. The occurrence of necroptosis along with apoptosis during mastitis has been reported to exacerbate inflammation and cause severe mammary tissue damage^64^. Additionally, ferroptosis, characterized by lethal iron-dependent lipid peroxidation, is an inflammation-associated cell death mechanism that contributes to mammary epithelial cell dysfunction^65^ and the development of clinical mastitis in dairy cows^66^.

The functional annotation of the turquoise RNA-seq-based module indicated the activation and involvement of the adaptive immune system in host–pathogen interactions during mastitis, consistent with previous transcriptomic studies^67,68^. CD4 T-lymphocytes are stimulated and differentiate into T-helper 1 (Th1) inflammatory phenotypes^69^, secreting cytokines including TNF-α, IFN-γ, and IL2^70^. CD4 T lymphocytes differentiation into Th17 is mediated by transforming growth factor β (TGF-β) and IL6, and Th17 is involved in the secretion of IL17, IL21, and IL22 cytokines^70^. Dysregulation of the Th1/Th2 and Th17/Treg balance has been implicated in the pathogenesis of chronic inflammatory mastitis^70,71^.

We also observed suppression of metabolic processes in the turquoise RNA-seq-based module. This finding is in line with a previous study that demonstrated extensive changes in the host's transcriptional profile during *S. uberis*-induced mastitis, leading to the induction of inflammatory responses and the simultaneous suppression of several metabolic pathways^7^. Metabolic profiling in infected animals has shown that exposure to *S. uberis* leads to inflammation and metabolic dysfunction in mammary glands and mammary epithelial cells^72^. For instance, downregulation of PPAR-γ and PPAR-α, which are anti-inflammatory cores involved in lipid and cholesterol storage and metabolism, has been consistently observed during mastitis^7,73^.

### Identification of hub-hub genes and their regulatory miRNAs

In terms of hub-hub genes, we identified several crucial immune and inflammatory response genes, including *TLR2*, *TLR4*^74^, *TNF*, *IL1β*, *IL1A*, *IL6*^75^, *JAK2*^76,77^, and *IL10*^68^, which play important roles in the pathogen-host interactions during mastitis. *TLR2* and *TLR4* are putative surface receptors of PAMPs from Gram-positive (like *S. uberis* and *S. aureus*) and Gram-negative (like *E. coli*) bacteria, respectively, and they are the first initiators of downstream inflammatory cascades during mastitis^74^. For example, Wu, et al.^78^ reported an induction of inflammatory response and increased secretion of proinflammatory cytokines due to *TLR4*-mediated activation of NF-κB during mastitis^78^. Interestingly, several previous in vivo and in vitro studies have demonstrated that various antagonists, including nuciferine^79^, hederacoside-C^80^, chlorogenic acid^81^, polydatin^82^, curcumin^83^, and indirubin^84^, attenuate the phosphorylation of MAPKs and NF-κB through suppression of *TLR2* and *TLR4*, thereby preventing hyperinflammation and immunopathology induced by LPS and *S. aureus* infection in mastitis.

Furthermore, our target prediction analysis revealed that the *TLR4* hub-hub gene was preferentially targeted by *bta-miR-30a-5p* (hub) and b*ta-miR-486* miRNAs. However, a decrease in the expression of *bta-miR-30a-5p* hub miRNA was observed in response to *S. uberis*^7^ and *S. aureus*^85^ infections, which could indicate a complex strategy of mastitis-causing pathogens to induce inflammation by blocking key suppressors of inflammatory mediators. On the other hand, in vivo evidence from *S. uberis* infection indicates the upregulation of *bta-miR-486* in the milk of infected animals at 48 h post-infection, suggesting the effective role of this miRNA as a brake to control inflammation and the host's immune response in the late stages of infection^7^. Additionally, *bta-miR-204*, which targets *TLR2* and *PTGS2* hub-hub genes, has been identified as one of the key mediators of vascular inflammation, playing an important role in regulating inflammation by attenuating the main inflammatory factors^86^.

As expected, a significant increase in the concentrations of proinflammatory cytokines, including *TNF*, *IL6*, and *IL1β*, in the serum and mammary glands of clinical mastitis cases has been observed in several previous studies^75,87^. These findings indicate that the expression levels of these proinflammatory cytokines are directly related to the progression of infection, the clinical severity, and the pathophysiology of bacterial mastitis^88,89^. Interestingly, the essential role of *IL1β* and *IL1A* in the induction of apoptosis and necroptosis, respectively, has also been discussed^90^. Therefore, targeting proinflammatory cytokines such as *TNF*, *IL1β*, *IL1A*, and *IL6* to reduce their expression or targeting upstream cores that stimulate these cytokines such as NF-κB and MAPKs, has been suggested as a novel therapeutic approach to reduce mammary gland damage and pathology caused by mastitis^91–93^.

Molecular interactome analysis of the turquoise RNA-seq-based module revealed that *TNF* hub-hub gene is a potential target for *bta-miR-193b* and *bta-miR-125a* hub miRNAs. Consistent with our results, a previous study demonstrated that *miR-193b* regulates the inflammatory response in inflamed chondrocytes by inhibition of *TNF* expression^94–96^. Interestingly, *miR-125a* has been found to be negatively correlated with inflammation and could significantly reduce the production of proinflammatory cytokines especially *TNF* in patients with inflammatory bowel diseases^97,98^. However, a decrease in the expression of the *bta-miR-125a* hub miRNA has been observed in response to *S. uberis* infection in the milk of infected animals^7^. Additionally, *miR-125a* potentially targets the *IL1A* hub-hub gene and may play a key role in preventing *IL1A*-induced necrosis in mammary glands, in addition to its anti-inflammatory effects.

*IL6* another hub-hub gene, was predicted to be targeted by *bta-miR-455-5p* and *bta-miR-96* miRNAs. Remarkably, the use of *miR-455-5p* as an important *IL6* suppressor has been recommended as a promising tool to improve disease severity and control inflammation and attacks in patients with multiple sclerosis^99^. Furthermore, the *IL6*-*JAK2*-*STAT3* axis has been found to induce plasma cell mastitis development^76^. On the other hand, *bta-miR-96* has been associated with the risk of mastitis^100^. Moreover, the *IL1β* hub-hub gene was targeted by *bta-miR-375* and *bta-miR-31* hub miRNAs. Surprisingly, *miR-375* is one of the most downregulated miRNAs in bovine mammary tissue infected with *S. aureus*^101^, *E. coli*^67^, and *S. uberis*^7^ indicating its crucial role in regulating immune and inflammatory responses. Thus, the targeting of the *IL1β* hub-hub gene by *miR-375* in the turquoise RNA-seq-based module can be predicted to regulate bovine mammary inflammation and *IL1β*-induced apoptosis. Moreover, recent studies showed that *mir-31* hub miRNA by targeting the *IL1B* gene exerts an inverse relationship with the progression of inflammation in diabetic nephropathy^102^ and apoptosis in mammary cancer cell lines^103^.

*IL10* is one of the most potent anti-inflammatory cytokines that terminates the inflammatory response by suppressing the production of inflammatory cytokines (*TNF-α*, *IL1β*, and *IL6*) and returns the inflammatory system to a resting state when the microbial infection is eradicated^104^. Simultaneously with the increase in the levels of proinflammatory cytokines, a significant decrease in the expression of *IL10* at the early stage of infection has been reported by previous studies^71^. Also, this cytokine has been introduced as one of the key downstream targets of *E. coli* for the elevation of inflammation and establishment of mastitis through expression suppression^105^. In this regard, *IL10* was considered by He, et al.^68^ as key candidate biomarker for anti-*S. aureus* mastitis study and treatment.

In our candidate network, this hub-hub gene was targeted by *bta-miR-143* and *bta-miR-504* hub miRNAs. Surprisingly, unlike other previously miRNAs, *miR-143* was highly expressed in bovine mammary glands in response to various mastitis infections, including *S. aureus* and *E. coli*^101^. Therefore, the increase in the expression of this miRNA during mastitis infection can have a key effect in promoting inflammation in favor of mastitis-causing pathogens by targeting anti-inflammatory factors such as *IL10*. On the other hand, there is no data on the role of *miR-504* in the inflammation caused by mastitis. However, a previous study has shown that in diabetic mice, high glucose leads to increase in inflammation through several mechanisms. Interestingly, one of these mechanisms to exacerbation of inflammation was the upregulation in the expression of *miR-504*^106^.

In addition to these findings, other hub-hub genes in the turquoise RNA-seq-based module including *IL12A*^107^, *CD44*^108^, *CD274*^109^, *SOCS1*^110^, *SOCS3*^111^, *IL18*^112^, *JAK3*^113^, *CXCL8*^114^, *ICAM1*^115^, *IL2RA*, *IL2RG*, *IL4R*^116^, *CCR2*, *IL15*, *IL23A*, *CCR5*^117^, *CXCR4*^118,119^, *VEGFA*^105^, *PTGS2*^120^, *CD40*^121^, and *PTPRC*^122^ have also been reported to play a role in the pathogenesis of mastitis. For instance, Corl, et al.^123^ found that *ICAM1*, which is involved in the development of several inflammatory diseases including atherosclerosis, was among several proinflammatory factors involved in the activation and early migration of leukocytes into the mammary gland during the early stages of coliform mastitis, and reached peak expression between 4 and 12 h following stimulation. Our results showed that *ICAM1* is targeted by *bta-miR-151-3p*, *bta-miR-148a*, and *bta-miR-10b* miRNAs. Interestingly, consistent with our results, the anti-inflammatory properties of *miR-151-3p* and *miR-148a* miRNAs have been revealed previously in *E. coli*, *S. aureus*, and LPS-induced inflammatory processes^85,124^.

The *CXCL8* hub-hub gene, which encodes the *IL8* protein, is one of the essential chemokines for the recruitment of neutrophils to the site of inflammation, and therefore can have a significant correlation with SCC in mastitis animals^125^. Pathogenic strains of *E. coli* have been shown to induce strong expression of proinflammatory cytokines and chemokines such as *IL8* in the udder, leading to acute mastitis^114^. Interestingly, *CXCL8* is preferentially targeted by *bta-miR-183* hub miRNA, which could be considered as a potential therapeutic factor to counteract mastitis-induced inflammation and tissue damage caused by leukocyte influx. Furthermore, the *PTGS2* gene, which is involved in prostaglandin synthesis and regulated during inflammation, was found to be targeted by *bta-miR-429* hub miRNA and *bta-miR-204* in the blue miRNA-seq-based module.

Interestingly, *mir-429* has previously been shown to play a critical role in inducing inflammation caused by LPS challenge in vivo, so it was concluded that targeting this miRNA with anti-miRNAs attenuates the LPS-induced inflammatory response^126^. Eventually, it has been suggested that *VEGFA* hub-hub gene may have key functions in the immune response, inflammation or mastitis development, which could provide a basis for strategies to improve the diagnosis and treatment of mastitis in dairy cattle^105^. In this regard, comprehensive information from the miRWalk database indicated the targeting of *VEGFA* by *bta-miR-205* (hub) and *bta-miR-27b* miRNAs. In this regard, *miR-205* was identified as a mastitis resistance-related miRNA in a recent miRNAomic study^127^. Additionally, *miR-27b*, which is involved in mammary gland development, has been suggested as an early mastitis indicator^128^.

### Identification of hub-hub TFs and their regulatory miRNAs

Several hub-hub TFs were also identified including *STAT1*, *STAT2*, *STAT3*, *STAT5A*, *STAT5B*, *STAT6*^46^, *NFKB1*, *NFKB2*^129^, and *IRF1*^33^, as well as a hub-hub lncRNA including *CDC42SE1*^130^, which had essential immunoregulatory roles in mastitis immunity. It is well clarified that signal transducers, and activators of transcription proteins (STATs) members are involved in cell growth, differentiation, cell survival, apoptosis, inflammation, and mammary gland development. Previous data suggest the effective role of *STAT3* in tumor development in breast cancer^131^. Moreover, this hub-hub TF showed significant upregulation in response to *S. uberis* infection and has also been introduced as an essential mediator for mammary cell apoptosis and inflammation^132^. Interestingly, previous studies have reported that *IL6* expression correlates with *STAT3* phosphorylation levels, thus concluding that the *IL6*-*STAT3* axis is directly related to the chronic inflammatory state of the breast during mastitis^105^. *STAT3* hub-hub TF is regulated by the *bta-miR-30a-5p*, *bta-miR-31*, and *bta-miR-125a* hub miRNAs which were discussed earlier. Moreover, *STAT3* was post-transcriptionally negatively regulated by *bta-mir-127* hub miRNA. Accordingly, this hub miRNA could be a key anti-inflammatory candidate during mastitis. In this regard, previous researches indicate the central role of *mir-127* to promotion the reduction of lung inflammation^133^.

Moreover, it has been reported that *E. coli*-induced mastitis leads to dephosphorylation of *STAT5*, which is one of the lactation-specific genes and one of the main elements for the synthesis of milk components^74^. In other words, it has been concluded that the inactivation of *STAT5* and the activation of *NFKB1* and *STAT3* are directly related to the milk loss in mammary glands after infection^89^. Additionally, *STAT5A/B* and *STAT6* hub-hub TFs were targeted by *bta-miR-200a* and *bta-miR-141* hub miRNAs, respectively. Interestingly, Luoreng, et al.^67^ recently reported that *miR-200a* was significantly upregulated during *E. coli*-induced mastitis, which could be directly related to the reduction of milk production during mastitis by targeting *STAT5* TF.

*NFKB1* and *NFKB2* hub-hub TFs are main members of the NF-κB pathway, which are essential for the transcription of downstream cytokine genes and initiation of inflammatory response^134^. As expected, previous data showed that *NFKB1* and *NFKB2* were upregulated in LPS- and LTA-induced mastitis in mammary epithelial cells, which was directly related to infection-induced inflammation during mastitis^120,129^. Accordingly, *NFKB1* has recently been observed among highly-correlated genes with SCC and other clinical mastitis-related traits^117^. These findings suggested that *NFKB1* hub-hub TF can be considered as the main therapeutic components to manage and eradicate mastitis. Surprisingly, *bta-miR-30a-5p* hub miRNA showed the ability to target *NFKB1* in addition to *TLR4* and *STAT3*. Therefore, this hub miRNA can have a promising potential to develop therapeutic strategies against mastitis-induced inflammation by targeting the *TLR4*-*NFKB1*-*STAT3* axis.

### Identification of hub-hub lncRNAs and their regulatory miRNAs

Furthermore, the precise role of *CDC42SE1* hub-hub lncRNA in mastitis inflammation and immunity is still unclear. However, in relation to inflammation, it has also been highlighted that *CDC42SE1* had a positive correlation with the inflammatory features of clear cell renal cell carcinoma^135^. *CDC42SE1* hub lncRNA was targeted by several miRNAs, including *bta-miR-151-3p*, *bta-miR-486*, *bta-miR-125a*, *bta-miR-504*, and *bta-mir-1388-5p*. Among them, *bta-mir-1388-5p* has been reported to have a potential anti-inflammatory role by interfering with inflammatory signals^136^. These findings provide novel insights into the pathogenesis of mastitis by investigating the molecular interactome involving mRNAs, miRNAs, and lncRNAs. The transcriptional suppression of specific miRNAs can amplify the proinflammatory response, tissue damage, and immunopathogenesis of mastitis. Conversely, targeting anti-inflammatory mediators and their specific miRNAs could potentially be utilized as therapeutic strategies. However, further research is needed to fully understand these mechanisms and their potential applications.

## Conclusion

Mastitis, a prevalent inflammatory condition of the mammary glands, exhibits a complex immunopathology and multifactorial phenotype. In this study, we employed an integrative approach combining RNA-seq and miRNA-seq techniques with systems biology computational algorithms to gain comprehensive insights into the molecular regulatory mechanisms underlying mastitis. Our findings led to the construction of an integrated immunoregulatory network specific to bovine mastitis. The turquoise RNA-seq-based module demonstrated the strongest positive correlation with clinical features of mastitis, including somatic cell count (SCC) and total bacterial count (TBC). Additionally, module assignment analysis revealed that the blue miRNA-seq-based module exerts post-transcriptional regulation on the turquoise RNA-seq-based module. Consequently, several important regulatory elements were identified, including hub-hub genes (*TLR2*, *TLR4*, *TNF*, *IL6*, *IL1B*, *IL1A*, *JAK2*, *SOCS1*, *SOCS3*, *IL10*, *ICAM1*, *CXCL8*, *VEGFA*, and *PTGS2*), hub-hub TFs (*STAT3*, *STAT5*, *NFKB1*, and *NFKB2*), hub-hub lncRNA (*CDC42SE1*), and hub miRNAs (*bta-mir-30a-5p*, *bta-mir-125a*, *bta-mir-205*, *bta-mir-193b*, *bta-mir-455-5p*, *bta-mir-31*, *bta-mir-200a*, *bta-mir-127*, and *bta-mir-143*) were identified. Our results provide compelling evidence suggesting that dysregulation in the interplay between these regulatory elements plays a critical role in the aggravation of inflammation and the pathogenesis of mastitis. As key components of the host immune response, these regulatory elements hold promise as diagnostic tools, prognostic biomarkers, and potential targets for therapeutic interventions, particularly for subclinical mastitis cases. However, further experimental research involving in vitro and in vivo analyses are necessary to validate the findings of this study, thereby elucidating the immunoregulatory roles of miRNAs-mRNAs-lncRNAs in bovine mastitis.

## Materials and methods

### Datasets

Publicly available raw RNA-seq and matched miRNA-seq data were obtained from the Gene Expression Omnibus (GEO) database at the National Center for Biotechnology Information (NCBI) under accession number GSE51858. The dataset consisted of milk-isolated CD14 + monocytes from five *S. uberis*-infected and five non-infected control Holstein–Friesian cows at five time points 0, 12, 24, 36, and 48 h post infection (hpi). Each time point had five biological replicates. Ten primiparous Holstein–Friesian cows in the middle of their first lactation period, aged between 26 and 30 months and between 3 and 5 months postpartum, were selected for an in vivo experiment. Among these, five cows were infected at each time point via the teat canal of the right front quarter with approximately 500 colony-forming units (CFU) of *S. uberis* 0140, a mastitis-causing pathogen, in 10 ml saline. The remaining, five non-infected control cows were inoculated with saline only at the same time points. Milk-derived CD14 + monocytes were isolated using fluorescence-activated cell sorting (FACS) and labeled with monoclonal anti-bovine CD14 and PE-conjugated anti-mouse IgG1 antibody. Labeled cells were then separated based on fluorescence intensity, and cells with more than 95% purity were isolated from the milk of each cow. Infection progression was monitored using recorded milk bacterial counts (CFU/ml) and somatic cell counts (per ml) at each time point^7,46^. An Illumina HiSeq 2000 platform was used to generate 50-bp single-end reads, resulting in a total of 50 RNA-seq and 50 miRNA-seq libraries (25 *S. uberis*-infected vs. 25 non-infected controls) from the milk of both animal groups. Further details about the data can be found in the source paper^7^. According to the source paper^7^, five RNA-seq infected samples (GSM1254114, GSM1254115, GSM1254116, GSM1254117, and GSM1254118) and five matched miRNA-seq infected samples (GSM1253803, GSM1253804, GSM1253805, GSM1253806, and GSM1253807) were excluded due to very low bacterial counts, which indicated an incomplete infection (bacterial count < 200 CFU/ml). Additionally, one RNA-seq infected sample (GSM1254121) and one matched miRNA-seq infected sample (GSM1253810) were excluded due to a low number and poor quality of reads (Q < 20). Finally, a total of 44 RNA-seq samples and 44 matched miRNA-seq samples were retained for downstream analysis (19 *S. uberis*-infected vs. 25 non-infected samples). Clinical traits of bovine mastitis including milk volume (in liters), rectal temperature (in Fahrenheit), ambient temperature (in Celsius), humidity (%), total bacterial counts (TBC) per 10 ml, somatic cell counts (SCC) per ml, and CD14 cell number per ml were obtained from the supplementary material section of the source paper^7^ and filtered for functional measurements.

### RNA-seq and miRNA-seq data analysis and preprocessing

The FastQC software version 0.11.9 (https://www.bioinformatics.babraham.ac.uk/projects/fastqc/) was used to evaluate the sample sequencing protocol and quality control of the raw RNA-seq and miRNA-seq reads. After checking the quality of the raw reads, low-quality reads/bases (Q < 20) and adapter sequences for both RNA-seq and miRNA-seq reads were trimmed using Trimmomatic software^137^ (version 0.39). The trimming parameters for RNA-seq reads were ILLUMINACLIP:Adapter.fa:2:30:10, SLIDINGWINDOW:6:20, TRAILING:20, and MINLEN:30. For miRNA-seq data, the trimming parameters were ILLUMINACLIP:Adapter.fa:2:30:10, SLIDINGWINDOW:6:20, TRAILING:20, and MINLEN:12. After obtaining clean reads, FastQC was used again to assess the quality and confirm the improvements. For RNA-seq data analysis, clean reads were aligned to the latest bovine reference genome (ARS-UCD1.2, release-108 from Ensemble database) using Hisat2^138^ aligner version 2.2.1 with default parameters. The python script HTSeq-count^139^ (version 0.13.5) was used in intersection-strict mode to count uniquely mapped reads to annotated genes based on the Ensembl bovine GTF file (release 108). Then, all counted files were merged into a single table and a raw expression matrix was constructed that contained read counts information of mRNAs and lncRNAs for all samples (infected and non-infected). For miRNA-seq data analysis, putative non-miRNA reads such as ncRNAs, piRNAs, and phasiRNAs were removed using Unitas^140^ (version 1.7.0). The clean miRNA-seq reads were aligned to the bovine pre-mature miRNA sequences (version 22, downloaded from miRBase database) using Bowtie^141^ software (version 1.3.1) allowing one mismatch. The HTSeq-count^139^ version 0.13.5 was then used in intersection-strict mode to assign uniquely mapped miRNA-seq reads to miRBase miRNA annotations (version 22). Finally, all miRNA-seq-based counted files were merged into a table and a raw expression matrix was constructed that contained read counts information of all miRNAs for all samples (infected and non-infected).

To address the issue of low-expression or low-variance RNAs, which can represent sampling noise and result in unreliable correlations for co-expression network analysis, several filtering parameters were applied. First, RNAs were evaluated in both expression matrices, and those with read counts ≥ 5 in at least 10 samples were selected for further analysis. Next, both RNA-seq-based and miRNA-seq-based expression matrices were normalized using the default procedure from the DESeq2^142^ R package version 1.36.0 with correction for the parity number to reduce potential effects from the parity number factor. Then, both expression matrices were transformed using the getVarianceStabilizedData function in DESeq2^142^ R package as recommended in the WGCNA manual (https://horvath.genetics.ucla.edu/html/CoexpressionNetwork/Rpackages/WGCNA/). Finally, RNAs with a standard deviation < 0.25 were excluded from both expression matrices.

### Weighted co-expression network analysis

Weighted co-expression network analysis was performed separately for the RNA-seq-based and miRNA-seq-based matrices using the WGCNA^35^ R package (version 1.71). To ensure the reliability of the network construction and account for outliers, the adjacency matrices of the samples were constructed for both expression matrices using the adjacency function of the WGCNA R package. Sample network connectivity was standardized based on the distances, and samples with a standardized connectivity score < -2.5 were considered outliers and excluded. The goodSamplesGenes function of the WGCNA R package was used to identify samples and genes with > 50% missing entries and genes with zero variance. To construct the scale-free network, an appropriate soft threshold power was calculated using the pickSoftThreshold function of the WGCNA R package for each expression matrix. The soft thresholding power *β* = 17 and *β* = 6 were determined for RNA-seq-based and miRNA-seq-based co-expression module construction, respectively. The weighted adjacency matrix was constructed for each expression matrix based on the respective soft thresholding power using Pearson correlation coefficient. The adjacency matrix was then transformed into a topological overlap matrix (TOM), which describes the interconnectedness between genes in the network. The signed weighted co-expression network was constructed separately, for RNA-seq and miRNA-seq datasets. Modules with different sizes were detected using average linkage hierarchical clustering analysis based on the dissimilarity of the TOM (1-TOM) through a dynamic hybrid tree cutting algorithm. Modules with highly similar expression profiles were merged based on the correlation between the module eigengenes, which represents the first principal component of the expression profile for a given module.

All the above steps were performed in both datasets independently using automatic, one-step network construction and module detection function blockwiseModules of the WGCNA R package. Therefore, for RNA-seq-based module detection, blockwiseModules function of the WGCNA R package was used with the following main parameters: power = 17, networkType = "signed", TOMType = "signed", maxBlockSize = 12,000, minModuleSize = 30, reassignThreshold = 0, mergeCutHeight = 0.25. On the other hand, miRNA-seq-based modules were detected using blockwiseModules function of the WGCNA R package, with the following major parameters: power = 6, networkType = "signed", TOMType = "signed", maxBlockSize = 2000, minModuleSize = 30, reassignThreshold = 0, mergeCutHeight = 0.25.

### Module–trait relationships analysis for RNA-seq-based modules

To investigate the relationship between the genome and the measured phenotypic traits and also to identify significant highly-correlated modules with clinical traits of bovine mastitis such as SCC and TBC, module-trait relationships analysis was performed using the WGCNA^35^ R package. The correlation between the clinical traits of mastitis and module eigengenes of the RNA-seq-based modules was calculated using Pearson correlation coefficient. Then, the cutoff of significant highly-correlated RNA-seq-based modules with clinical traits of mastitis was defined as *p*-value < 0.05 and |*R*|> 0.55. Additionally, the gene significance (GS) criterion was calculated for each gene through the correlation between gene expression profiles and the clinical trait of interest, such as SCC.

### Identification of the RNA-seq-based module of interest and functional annotation

To assess the biological behavior of significant highly-correlated RNA-seq-based modules with clinical traits of mastitis and identify modules involved in immune response, inflammation mechanisms, and *S. uberis-*host interactions, the co-expressed genes in each highly-correlated module were subjected to functional enrichment analysis using the Enrichr^143^ online tool (https://maayanlab.cloud/Enrichr/). Gene Ontology (GO) terms (biological process) and Kyoto Encyclopedia of Genes and Genomes (KEGG) pathway enrichment analyses were performed. The threshold for significant overrepresentation of functional terms was set as adj *p*-value < 0.05 (corrected by the Benjamini–Hochberg method). Additionally, a set of bovine transcriptional regulatory factors were extracted from the AnimalTFDB3.0^144^ database (http://bioinfo.life.hust.edu.cn/AnimalTFDB/#!/) to identify crucial transcription factors (TFs) that regulate the expression of functional genes in the significant highly-correlated RNA-seq-based modules.

### Detection of Hub RNAs

In biological networks, the scale-free topology of the network describes the distribution of interactions among nodes. In other words, one of the main characteristics of a scale-free network is several nodes with low interactions and few nodes with high interactions, which are called hubs^26^. Indeed, these hub nodes have the highest degree of connectivity compared to other nodes in the network and are more closely related to the biological function of that network^145^. In many cases, these central nodes have been evaluated as potential candidates for understanding the molecular mechanisms of many diseases and developing therapeutic/preventive methods^146,147^. The module memberships (MM) or eigengene-based connectivity *k*_*ME*_ criterion of the WGCNA package assesses the relationship of a gene with the corresponding module compared to other genes of that module and explains how the genes of a module are correlated with the characteristics of that module^35^. In this study, to identify intramodular hub RNAs (mRNAs, lncRNAs, and miRNAs) in the selected modules, the MM criterion was calculated by WGCNA R package through the correlation between the expression profiles and module eigengenes. In this regard, RNAs with high MM values play a central role in terms of biological and topological properties in a module^35^. Therefore, RNAs with *k*_*ME*_ > 0.65 were considered as intramodular hubs in the relevant modules.

### Assign the miRNA-seq-based modules to the RNA-seq-based modules and miRNA target prediction

To reveal which of the miRNA-seq-based modules are post-transcriptionally regulates the RNA-seq-based module of interest, Pearson correlation was calculated between module eigengenes of RNA-seq-based and module eigengenes of miRNA-seq-based modules. Negative correlations indicate that miRNA-seq-based modules may inversely regulate the RNA-seq-based modules^26^. The cutoff for assigning miRNA-seq-based modules to RNA-seq-based module of interest was defined as *p*-value < 0.05 and a negative correlation larger than 0.50. miRNA-seq-based modules meeting this threshold were considered important regulators of the RNA-seq-based-module of interest and selected for further investigation.

Moreover, to enhance the reliability of the assignment analysis results and explore molecular interactions more deeply, the target prediction analysis was performed for miRNAs in the selected miRNA-seq-based module that were negatively correlated with the RNA-seq-based module of interest. The miRNAs from the selected miRNA-seq-based module were subjected to target prediction using miRWalk 3.0^148^ database (http://mirwalk.umm.uni-heidelberg.de/). The miRWalk database incorporates predictions from various target prediction tools, including DIANA-microT, miRanda, miRDB, RNA22, miRTarBase, RNAhybrid, PicTar4, PicTar5, PITA, and Targetscan, and provides up-to-date information on mRNA-miRNA and lncRNA-miRNA interactions^148,149^. Additionally, to avoid false positive results, TargetScan, miRDB, and miRTarBase parameters of the miRWalk database were applied with binding score > 0.95, target binding region = 3'UTR, and minimum free energy (ΔG) = -15.

### Identification of hub-hub RNAs in the RNA-seq-based module of interest

To identify the most important intramodular highly connected hub RNAs, including hub-hub mRNAs (genes and TFs) and hub-hub lncRNAs, several steps were followed. First, to evaluate the network density at the translational level and extract protein interactions, co-expressed hub mRNAs (obtained from previous step) were selected from the RNA-seq-based module of interest for protein–protein interaction (PPI) network analysis using Search Tool for the Retrieval of Interacting Genes (STRING)^150^ database (https://string-db.org/). Next, all the generated interactions related to the RNA-seq-based module of interest, including PPI network derived from co-expressed hub mRNAs, WGCNA-calculated co-expressed hub mRNAs, and WGCNA-calculated co-expressed hub lncRNAs were inputted into the Cytoscape^151^ software version 3.7.1 (https://cytoscape.org/) and then were interpreted with the cytoHubba^152^ plugin (version 0.1) for maximal clique centrality (MCC) analysis. Importantly, it is well established that among the topological analytical methods, MCC has a better performance on the precision of predicting featured nodes in the complex biological networks^152,153^. Hence, the top 50 intramodular hub RNAs of the RNA-seq-based module of interest with the highest MCC score were identified as hub-hub RNAs.

### Integrated regulatory network construction

To generate a molecular interactome and subsequently construct an integrated regulatory network, all possible interactions of the RNA-seq-based module of interest (co-expressed hub mRNA-based PPI networks, WGCNA-calculated co-expressed hub mRNAs, and WGCNA-calculated co-expressed hub lncRNAs) were combined with its assigned miRNA-seq-based module interactions (WGCNA-calculated co-expressed miRNAs) and target prediction results. Then, the integrated regulatory network was constructed and visualized in the Cytoscape^151^ software version 3.7.1 (https://cytoscape.org/).

### Supplementary Information

Supplementary Information.

## Data Availability

Raw RNA-seq and their matched miRNA-seq data were obtained from the Gene Expression Omnibus (GEO) database (https://www.ncbi.nlm.nih.gov/geo/) at the National Center for Biotechnology Information (NCBI) under accession number of GSE51858.

## Abbreviations

KEGG: Kyoto encyclopedia of genes and genomes
GO: Gene ontology
GS: Gene significance
MCC: Maximal clique centrality
MM: Module membership
PAMP: Pathogen associated molecular pattern
PPI: Protein-protein interaction
PRR: Pattern recognition receptor
SCC: Somatic cell count
TF: Transcription factor
TOM: Topological overlap matrix
TBC: Total bacterial count
WGCNA: Weighted gene co-expression network analysis
