# Author Correction: Integrated analysis of inflammatory mRNAs, miRNAs, and lncRNAs elucidates the molecular interactome behind bovine mastitis

**DOI:** 10.1038/s41598-024-64764-4

**Published:** 2024-06-14

**Authors:** Aliakbar Hasankhani, Maryam Bakherad, Abolfazl Bahrami, Hossein Moradi Shahrbabak, Renzon Daniel Cosme Pecho, Mohammad Moradi Shahrbabak

**Affiliations:** 1 Department of Animal Science, College of Agriculture and Natural Resources, University of Tehran, Karaj, Iran; 2 Department of Animal and Dairy Sciences, University of Wisconsin-Madison, Madison, WI USA; 3 Department of Chemistry and Biology, Universidad San Ignacio de Loyola (USIL), Lima, Peru

Correction to: *Scientific Reports* 10.1038/s41598-023-41116-2, published online 24 August 2023

In the original version of this Article Abolfazl Bahrami was incorrectly affiliated with ‘Biomedical Center for Systems Biology Science Munich, Ludwig-Maximilians-University, Munich, Germany’. The correct affiliation is listed below.

Department of Animal Science, College of Agriculture and Natural Resources, University of Tehran, Karaj, Iran

The original Article has been corrected.

