# Supplementary material for: Integrated analysis of inflammatory mRNAs, miRNAs, and lncRNAs elucidates the molecular interactome behind bovine mastitis
Source: Sci Rep. 2023 Aug 24;13:13826. doi: 10.1038/s41598-023-41116-2 (PMC10449796; doi:10.1038/s41598-023-41116-2)
Supplement: Supplementary file 1 — Supplementary Information. [file 41598_2023_41116_MOESM1_ESM.pdf]

**Supplementary File S1: Comprehensive details about the different steps of pre-processing and ana**

| Geo_accession_number | Sample_ID | Sample_description | Raw_reads | Clean_reads |
|---|---|---|---|---|
| GSM1254084 | Milk-mRNA-TC-1-001 | 0_hour_control_animal_1 | 20,718,878 | 19111093.07 |
| GSM1254085 | Milk-mRNA-TC-1-002 | 12_hour_control_animal_1 | 19,811,944 | 18514261.67 |
| GSM1254086 | Milk-mRNA-TC-1-003 | 24_hour_control_animal_1 | 25,858,517 | 24257874.8 |
| GSM1254087 | Milk-mRNA-TC-1-004 | 36_hour_control_animal_1 | 18,640,732 | 17559569.54 |
| GSM1254088 | Milk-mRNA-TC-1-005 | 48_hour_control_animal_1 | 17,609,857 | 16202829.43 |
| GSM1254089 | Milk-mRNA-TC-2-001 | 0_hour_control_animal_2 | 33,798,892 | 31879114.93 |
| GSM1254090 | Milk-mRNA-TC-2-002 | 12_hour_control_animal_2 | 17,280,881 | 16356353.87 |
| GSM1254091 | Milk-mRNA-TC-2-003 | 24_hour_control_animal_2 | 52,006,852 | 49130873.08 |
| GSM1254092 | Milk-mRNA-TC-2-004 | 36_hour_control_animal_2 | 27,355,722 | 25252066.98 |
| GSM1254093 | Milk-mRNA-TC-2-005 | 48_hour_control_animal_2 | 9,829,401 | 9306476.867 |
| GSM1254094 | Milk-mRNA-TC-3-001 | 0_hour_control_animal_3 | 17,143,701 | 16283087.21 |
| GSM1254095 | Milk-mRNA-TC-3-002 | 12_hour_control_animal_3 | 18,841,300 | 17742852.21 |
| GSM1254096 | Milk-mRNA-TC-3-003 | 24_hour_control_animal_3 | 12,573,120 | 11782270.75 |
| GSM1254097 | Milk-mRNA-TC-3-004 | 36_hour_control_animal_3 | 15,470,012 | 14275727.07 |
| GSM1254098 | Milk-mRNA-TC-3-005 | 48_hour_control_animal_3 | 36,051,781 | 33373133.67 |
| GSM1254099 | Milk-mRNA-TC-4-001 | 0_hour_control_animal_4 | 17,968,186 | 16785879.36 |
| GSM1254100 | Milk-mRNA-TC-4-002 | 12_hour_control_animal_4 | 12,002,000 | 11182263.4 |
| GSM1254101 | Milk-mRNA-TC-4-003 | 24_hour_control_animal_4 | 19,366,295 | 18080373.01 |
| GSM1254102 | Milk-mRNA-TC-4-004 | 36_hour_control_animal_4 | 45,770,860 | 43395352.37 |
| GSM1254103 | Milk-mRNA-TC-4-005 | 48_hour_control_animal_4 | 43,264,871 | 40141147.31 |
| GSM1254104 | Milk-mRNA-TC-5-001 | 0_hour_control_animal_5 | 24,902,268 | 23490309.4 |
| GSM1254105 | Milk-mRNA-TC-5-002 | 12_hour_control_animal_5 | 38,440,720 | 36334168.54 |
| GSM1254106 | Milk-mRNA-TC-5-003 | 24_hour_control_animal_5 | 70,750,168 | 67134834.42 |
| GSM1254107 | Milk-mRNA-TC-5-004 | 36_hour_control_animal_5 | 66,078,829 | 61552429.21 |
| GSM1254108 | Milk-mRNA-TC-5-005 | 48_hour_control_animal_5 | 77,385,014 | 72502019.62 |
| GSM1254109 | Milk-mRNA-TI-1-001 | 0_hour_infected_animal_1 | 18,370,591 | 17110368.46 |
| GSM1254110 | Milk-mRNA-TI-1-002 | 12_hour_infected_animal_1 | 24,563,421 | 22689231.98 |
| GSM1254111 | Milk-mRNA-TI-1-003 | 24_hour_infected_animal_1 | 70,796,017 | 65606668.95 |
| GSM1254112 | Milk-mRNA-TI-1-004 | 36_hour_infected_animal_1 | 38,934,888 | 36587114.25 |
| GSM1254113 | Milk-mRNA-TI-1-005 | 48_hour_infected_animal_1 | 116,133,346 | 108120145.1 |
| GSM1254119 | Milk-mRNA-TI-3-001 | 0_hour_infected_animal_3 | 55,867,931 | 52258862.66 |
| GSM1254120 | Milk-mRNA-TI-3-002 | 12_hour_infected_animal_3 | 44,902,024 | 42423432.28 |
| GSM1254122 | Milk-mRNA-TI-3-004 | 36_hour_infected_animal_3 | 53,998,739 | 51234003.56 |
| GSM1254123 | Milk-mRNA-TI-3-005 | 48_hour_infected_animal_3 | 39,319,518 | 36810932.75 |
| GSM1254124 | Milk-mRNA-TI-4-001 | 0_hour_infected_animal_4 | 49,159,957 | 45949811.81 |
| GSM1254125 | Milk-mRNA-TI-4-002 | 12_hour_infected_animal_4 | 66,381,407 | 61920576.45 |
| GSM1254126 | Milk-mRNA-TI-4-003 | 24_hour_infected_animal_4 | 38,731,025 | 36267731.81 |
| GSM1254127 | Milk-mRNA-TI-4-004 | 36_hour_infected_animal_4 | 86,666,760 | 80756086.97 |
| GSM1254128 | Milk-mRNA-TI-4-005 | 48_hour_infected_animal_4 | 54,446,900 | 51283535.11 |
| GSM1254129 | Milk-mRNA-TI-5-001 | 0_hour_infected_animal_5 | 83,696,307 | 78875399.72 |
| GSM1254130 | Milk-mRNA-TI-5-002 | 12_hour_infected_animal_5 | 72,428,423 | 68705602.06 |
| GSM1254131 | Milk-mRNA-TI-5-003 | 24_hour_infected_animal_5 | 61,824,286 | 58578510.99 |
| GSM1254132 | Milk-mRNA-TI-5-004 | 36_hour_infected_animal_5 | 41,858,195 | 39489021.16 |
| GSM1254133 | Milk-mRNA-TI-5-005 | 48_hour_infected_animal_5 | 50,193,787 | 46891035.82 |

**lysis of RNA-seq data**

| Clean_reads (%) | uniquely_aligned_reads | uniquely_aligned_reads (%) | Overall_mapping_rate (%) |
|---|---|---|---|
| 92.24% | 17964427.48 | 94.00% | 96.00% |
| 93.45% | 17403405.97 | 94.00% | 98.00% |
| 93.81% | 22559823.56 | 93.00% | 97.00% |
| 94.20% | 16330399.68 | 93.00% | 99.00% |
| 92.01% | 15230659.66 | 94.00% | 99.00% |
| 94.32% | 29328785.74 | 92.00% | 98.00% |
| 94.65% | 15211409.1 | 93.00% | 96.00% |
| 94.47% | 45691711.97 | 93.00% | 97.00% |
| 92.31% | 23484422.29 | 93.00% | 97.00% |
| 94.68% | 8561958.717 | 92.00% | 98.00% |
| 94.98% | 14980440.23 | 92.00% | 97.00% |
| 94.17% | 16678281.08 | 94.00% | 96.00% |
| 93.71% | 11075334.51 | 94.00% | 99.00% |
| 92.28% | 13276426.18 | 93.00% | 98.00% |
| 92.57% | 31370745.65 | 94.00% | 98.00% |
| 93.42% | 15778726.6 | 94.00% | 96.00% |
| 93.17% | 10287682.33 | 92.00% | 96.00% |
| 93.36% | 16814746.9 | 93.00% | 96.00% |
| 94.81% | 40357677.7 | 93.00% | 96.00% |
| 92.78% | 36929855.53 | 92.00% | 97.00% |
| 94.33% | 21845987.75 | 93.00% | 98.00% |
| 94.52% | 33790776.75 | 93.00% | 98.00% |
| 94.89% | 62435396.01 | 93.00% | 99.00% |
| 93.15% | 56628234.88 | 92.00% | 99.00% |
| 93.69% | 67426878.24 | 93.00% | 97.00% |
| 93.14% | 16083746.35 | 94.00% | 96.00% |
| 92.37% | 21327878.06 | 94.00% | 98.00% |
| 92.67% | 60358135.44 | 92.00% | 99.00% |
| 93.97% | 34026016.26 | 93.00% | 99.00% |
| 93.10% | 99470533.52 | 92.00% | 96.00% |
| 93.54% | 49123330.9 | 94.00% | 98.00% |
| 94.48% | 39878026.34 | 94.00% | 97.00% |
| 94.88% | 47135283.28 | 92.00% | 98.00% |
| 93.62% | 34234167.46 | 93.00% | 96.00% |
| 93.47% | 42733324.98 | 93.00% | 98.00% |
| 93.28% | 58205341.86 | 94.00% | 98.00% |
| 93.64% | 33366313.27 | 92.00% | 99.00% |
| 93.18% | 75103160.88 | 93.00% | 97.00% |
| 94.19% | 48206523 | 94.00% | 97.00% |
| 94.24% | 74142875.73 | 94.00% | 97.00% |
| 94.86% | 63896209.91 | 93.00% | 96.00% |
| 94.75% | 54478015.22 | 93.00% | 98.00% |
| 94.34% | 36724789.68 | 93.00% | 98.00% |
| 93.42% | 43139752.95 | 92.00% | 96.00% |

**Supplementary_File_S1: Comprehensive details about the different steps of pre-processing and an**

| Geo_accession_number | Sample_ID | Sample_description | Raw_reads | Clean_reads |
|---|---|---|---|---|
| GSM1253773 | Milk-miRNA-TC-1-001 | 0_hour_control_animal_1 | 17,331,105 | 14,877,021 |
| GSM1253774 | Milk-miRNA-TC-1-002 | 12_hour_control_animal_1 | 28,014,725 | 23,902,163 |
| GSM1253775 | Milk-miRNA-TC-1-003 | 24_hour_control_animal_1 | 20,528,511 | 17,246,002 |
| GSM1253776 | Milk-miRNA-TC-1-004 | 36_hour_control_animal_1 | 26,412,904 | 22,810,184 |
| GSM1253777 | Milk-miRNA-TC-1-005 | 48_hour_control_animal_1 | 17,831,552 | 15,504,534 |
| GSM1253778 | Milk-miRNA-TC-2-001 | 0_hour_control_animal_2 | 25,328,242 | 21,328,913 |
| GSM1253779 | Milk-miRNA-TC-2-002 | 12_hour_control_animal_2 | 14,583,126 | 12,271,701 |
| GSM1253780 | Milk-miRNA-TC-2-003 | 24_hour_control_animal_2 | 11,852,859 | 10,136,565 |
| GSM1253781 | Milk-miRNA-TC-2-004 | 36_hour_control_animal_2 | 7,941,655 | 6,805,204 |
| GSM1253782 | Milk-miRNA-TC-2-005 | 48_hour_control_animal_2 | 18,391,794 | 15,763,607 |
| GSM1253783 | Milk-miRNA-TC-3-001 | 0_hour_control_animal_3 | 40,359,797 | 34,019,273 |
| GSM1253784 | Milk-miRNA-TC-3-002 | 12_hour_control_animal_3 | 22,496,712 | 19,153,701 |
| GSM1253785 | Milk-miRNA-TC-3-003 | 24_hour_control_animal_3 | 22,180,912 | 19,155,436 |
| GSM1253786 | Milk-miRNA-TC-3-004 | 36_hour_control_animal_3 | 11,916,844 | 10,234,186 |
| GSM1253787 | Milk-miRNA-TC-3-005 | 48_hour_control_animal_3 | 16,974,932 | 14,515,264 |
| GSM1253788 | Milk-miRNA-TC-4-001 | 0_hour_control_animal_4 | 19,737,101 | 17,001,539 |
| GSM1253789 | Milk-miRNA-TC-4-002 | 12_hour_control_animal_4 | 53,992,816 | 46,779,376 |
| GSM1253790 | Milk-miRNA-TC-4-003 | 24_hour_control_animal_4 | 42,174,080 | 36,602,884 |
| GSM1253791 | Milk-miRNA-TC-4-004 | 36_hour_control_animal_4 | 19,631,471 | 16,722,087 |
| GSM1253792 | Milk-miRNA-TC-4-005 | 48_hour_control_animal_4 | 24,263,976 | 20,580,704 |
| GSM1253793 | Milk-miRNA-TC-5-001 | 0_hour_control_animal_5 | 16,258,605 | 13,912,488 |
| GSM1253794 | Milk-miRNA-TC-5-002 | 12_hour_control_animal_5 | 14,296,183 | 12,010,223 |
| GSM1253795 | Milk-miRNA-TC-5-003 | 24_hour_control_animal_5 | 17,382,527 | 14,663,900 |
| GSM1253796 | Milk-miRNA-TC-5-004 | 36_hour_control_animal_5 | 12,605,875 | 10,834,750 |
| GSM1253797 | Milk-miRNA-TC-5-005 | 48_hour_control_animal_5 | 17,434,308 | 14,935,972 |
| GSM1253798 | Milk-miRNA-TI-1-001 | 0_hour_infected_animal_1 | 18,912,613 | 15,969,810 |
| GSM1253799 | Milk-miRNA-TI-1-002 | 12_hour_infected_animal_1 | 11,261,766 | 9,766,203 |
| GSM1253800 | Milk-miRNA-TI-1-003 | 24_hour_infected_animal_1 | 36,023,074 | 30,331,428 |
| GSM1253801 | Milk-miRNA-TI-1-004 | 36_hour_infected_animal_1 | 24,112,155 | 20,649,650 |
| GSM1253802 | Milk-miRNA-TI-1-005 | 48_hour_infected_animal_1 | 4,579,416 | 3,979,970 |
| GSM1253808 | Milk-miRNA-TI-3-001 | 0_hour_infected_animal_3 | 651,418 | 547,973 |
| GSM1253809 | Milk-miRNA-TI-3-002 | 12_hour_infected_animal_3 | 532,806 | 462,049 |
| GSM1253811 | Milk-miRNA-TI-3-004 | 36_hour_infected_animal_3 | 25,093,076 | 21,534,878 |
| GSM1253812 | Milk-miRNA-TI-3-005 | 48_hour_infected_animal_3 | 24,657,275 | 21,064,710 |
| GSM1253813 | Milk-miRNA-TI-4-001 | 0_hour_infected_animal_4 | 61,575,773 | 51,920,692 |
| GSM1253814 | Milk-miRNA-TI-4-002 | 12_hour_infected_animal_4 | 30,843,483 | 26,429,781 |
| GSM1253815 | Milk-miRNA-TI-4-003 | 24_hour_infected_animal_4 | 11,136,199 | 9,388,929 |
| GSM1253816 | Milk-miRNA-TI-4-004 | 36_hour_infected_animal_4 | 13,095,699 | 11,208,609 |
| GSM1253817 | Milk-miRNA-TI-4-005 | 48_hour_infected_animal_4 | 19,698,731 | 17,031,523 |
| GSM1253818 | Milk-miRNA-TI-5-001 | 0_hour_infected_animal_5 | 20,994,557 | 18,103,607 |
| GSM1253819 | Milk-miRNA-TI-5-002 | 12_hour_infected_animal_5 | 38,614,345 | 32,656,152 |
| GSM1253820 | Milk-miRNA-TI-5-003 | 24_hour_infected_animal_5 | 12,954,574 | 11,073,570 |
| GSM1253821 | Milk-miRNA-TI-5-004 | 36_hour_infected_animal_5 | 24,631,897 | 21,368,171 |
| GSM1253822 | Milk-miRNA-TI-5-005 | 48_hour_infected_animal_5 | 7,068,913 | 6,052,403 |

**alysis of miRNA-seq data**

| Clean_reads (%) | uniquely_aligned_reads | uniquely_aligned_reads (%) | Overall_mapping_rate (%) |
|---|---|---|---|
| 85.84% | 13091778.07 | 88.00% | 96.00% |
| 85.32% | 21033903.77 | 88.00% | 96.00% |
| 84.01% | 15004021.82 | 87.00% | 97.00% |
| 86.36% | 20072961.83 | 88.00% | 98.00% |
| 86.95% | 13799035.67 | 89.00% | 98.00% |
| 84.21% | 18556153.95 | 87.00% | 95.00% |
| 84.15% | 10799096.47 | 88.00% | 95.00% |
| 85.52% | 9021542.865 | 89.00% | 96.00% |
| 85.69% | 5988579.669 | 88.00% | 97.00% |
| 85.71% | 13871973.84 | 88.00% | 98.00% |
| 84.29% | 30277152.87 | 89.00% | 97.00% |
| 85.14% | 17046793.53 | 89.00% | 97.00% |
| 86.36% | 16665228.97 | 87.00% | 98.00% |
| 85.88% | 9006083.352 | 88.00% | 98.00% |
| 85.51% | 12628279.99 | 87.00% | 98.00% |
| 86.14% | 15131369.53 | 89.00% | 95.00% |
| 86.64% | 41633644.45 | 89.00% | 96.00% |
| 86.79% | 32210537.95 | 88.00% | 95.00% |
| 85.18% | 14548215.69 | 87.00% | 96.00% |
| 84.82% | 18316826.95 | 89.00% | 96.00% |
| 85.57% | 12242989.7 | 88.00% | 97.00% |
| 84.01% | 10568996.54 | 88.00% | 97.00% |
| 84.36% | 12904231.8 | 88.00% | 95.00% |
| 85.95% | 9642927.111 | 89.00% | 98.00% |
| 85.67% | 13293014.78 | 89.00% | 96.00% |
| 84.44% | 13893735.06 | 87.00% | 98.00% |
| 86.72% | 8594259.058 | 88.00% | 95.00% |
| 84.20% | 26994971.19 | 89.00% | 96.00% |
| 85.64% | 18378188.09 | 89.00% | 95.00% |
| 86.91% | 3462574.288 | 87.00% | 95.00% |
| 84.12% | 487695.8112 | 89.00% | 97.00% |
| 86.72% | 401982.946 | 87.00% | 97.00% |
| 85.82% | 18950692.48 | 88.00% | 96.00% |
| 85.43% | 18536944.83 | 88.00% | 98.00% |
| 84.32% | 45690208.78 | 88.00% | 96.00% |
| 85.69% | 23522504.72 | 89.00% | 95.00% |
| 84.31% | 8168368.558 | 87.00% | 98.00% |
| 85.59% | 9863575.721 | 88.00% | 98.00% |
| 86.46% | 14987740.08 | 88.00% | 95.00% |
| 86.23% | 16112209.79 | 89.00% | 95.00% |
| 84.57% | 28410851.86 | 87.00% | 97.00% |
| 85.48% | 9634005.774 | 87.00% | 96.00% |
| 86.75% | 18803990.17 | 88.00% | 96.00% |
| 85.62% | 5326114.913 | 88.00% | 95.00% |

**Supplementary File S2: Information about identifying and removing outliers of RNA-seq**

| Sample | Outlier_color | Standardized_connectivity_score |
|---|---|---|
| CN_00H_01 | black | 0.587585589 |
| CN_00H_02 | black | 0.182584432 |
| CN_00H_03 | black | 0.598669536 |
| CN_00H_04 | black | 0.56761654 |
| CN_00H_05 | black | 0.642335621 |
| CN_12H_01 | black | 0.600621986 |
| CN_12H_02 | black | 0.37379024 |
| CN_12H_03 | black | 0.63376144 |
| CN_12H_04 | black | 0.400496108 |
| CN_12H_05 | black | 0.564408509 |
| CN_24H_01 | red | -4.993607921 |
| CN_24H_02 | black | 0.219212374 |
| CN_24H_03 | black | 0.604887696 |
| CN_24H_04 | black | 0.099484033 |
| CN_24H_05 | black | 0.654066893 |
| CN_36H_01 | black | 0.380765988 |
| CN_36H_02 | black | 0.345497506 |
| CN_36H_03 | black | 0.723367876 |
| CN_36H_04 | black | -0.519991927 |
| CN_36H_05 | black | 0.103122047 |
| CN_48H_01 | black | 0.535926774 |
| CN_48H_02 | black | 0.31397123 |
| CN_48H_03 | black | 0.51140855 |
| CN_48H_04 | black | 0.44339452 |
| CN_48H_05 | black | 0.681020323 |
| INF_00H_01 | black | -0.448461887 |
| INF_00H_03 | black | -0.485562444 |
| INF_00H_04 | black | 0.464510726 |
| INF_00H_05 | black | 0.607730931 |
| INF_12H_01 | black | -0.856200677 |
| INF_12H_03 | black | -0.401676515 |
| INF_12H_04 | black | 0.629825902 |
| INF_12H_05 | black | 0.555584571 |
| INF_24H_01 | black | -0.661985948 |
| INF_24H_04 | black | 0.603374867 |
| INF_24H_05 | black | 0.637767057 |
| INF_36H_01 | black | -1.253586742 |
| INF_36H_03 | black | -1.761281576 |
| INF_36H_04 | black | -1.60290013 |
| INF_36H_05 | black | -0.086094493 |
| INF_48H_01 | black | -0.164702656 |
| INF_48H_03 | black | -0.232846883 |
| INF_48H_04 | black | -0.900073636 |
| INF_48H_05 | black | 0.102183568 |

**data (samples with a standardized connectivity score < −2.5 were highlighted).**

**Supplementary File S2: Information about identifying and removing outliers of miRNA-seq data (s**

| Sample | Outlier_color | Standardized_connectivity_score |
|---|---|---|
| CN_00H_01 | black | 0.342795086 |
| CN_00H_02 | red | -4.299823069 |
| CN_00H_03 | black | 0.504906398 |
| CN_00H_04 | black | 0.475546353 |
| CN_00H_05 | black | -0.241731243 |
| CN_12H_01 | black | 0.468979478 |
| CN_12H_02 | black | -0.120882373 |
| CN_12H_03 | black | 0.573135815 |
| CN_12H_04 | black | 0.227452125 |
| CN_12H_05 | black | 0.521643287 |
| CN_24H_01 | black | 0.446643586 |
| CN_24H_02 | red | -2.952312951 |
| CN_24H_03 | black | 0.486472693 |
| CN_24H_04 | black | -1.231344463 |
| CN_24H_05 | black | 0.568491068 |
| CN_36H_01 | black | 0.480401137 |
| CN_36H_02 | black | 0.455921545 |
| CN_36H_03 | black | 0.537696531 |
| CN_36H_04 | black | 0.145889204 |
| CN_36H_05 | black | 0.370949844 |
| CN_48H_01 | black | 0.087321734 |
| CN_48H_02 | black | 0.425308726 |
| CN_48H_03 | black | 0.452389742 |
| CN_48H_04 | black | 0.4656504 |
| CN_48H_05 | black | 0.447834403 |
| INF_00H_01 | black | -0.234468252 |
| INF_00H_03 | black | -1.949638241 |
| INF_00H_04 | black | 0.405147896 |
| INF_00H_05 | black | 0.519524721 |
| INF_12H_01 | black | -0.183842322 |
| INF_12H_03 | black | -2.072777047 |
| INF_12H_04 | black | 0.071738776 |
| INF_12H_05 | black | 0.450945595 |
| INF_24H_01 | black | 0.523253206 |
| INF_24H_04 | black | 0.32582699 |
| INF_24H_05 | black | 0.52396831 |
| INF_36H_01 | black | 0.322969537 |
| INF_36H_03 | black | -0.039153803 |
| INF_36H_04 | black | -0.105765596 |
| INF_36H_05 | black | 0.355691181 |
| INF_48H_01 | black | 0.500038343 |
| INF_48H_03 | black | 0.353844529 |
| INF_48H_04 | black | 0.279711663 |
| INF_48H_05 | black | 0.313649458 |

**amples with a standardized connectivity score < $-2.5$ were highlighted).**

## Supplementary File S3: appropriate soft threshold powers for RNA-seq-based matrix

| Power | SFT.R.sq | slope | uncated.R.s | mean.k. | median.k. | max.k. |
|---|---|---|---|---|---|---|
| 1 | 0.101854 | 9.143898 | 0.944886 | 4697.058 | 4679.898 | 5090.602 |
| 2 | 0.175488 | 4.971992 | 0.915615 | 2732.366 | 2750.128 | 3188.629 |
| 3 | 0.085777 | 1.350864 | 0.87585 | 1740.856 | 1741.237 | 2349.288 |
| 4 | 0.013307 | 0.291977 | 0.790292 | 1183.296 | 1167.863 | 1855.334 |
| 5 | 0.079213 | -0.51252 | 0.776381 | 844.2439 | 819.6119 | 1549.651 |
| 6 | 0.308867 | -0.89472 | 0.821611 | 625.4011 | 594.7772 | 1325.455 |
| 7 | 0.481716 | -1.08513 | 0.857493 | 477.3329 | 442.6908 | 1152.702 |
| 8 | 0.587929 | -1.17837 | 0.891056 | 373.2521 | 335.5577 | 1016.872 |
| 9 | 0.651775 | -1.25902 | 0.907466 | 297.7516 | 256.8224 | 905.56 |
| 10 | 0.690744 | -1.32064 | 0.919791 | 241.5234 | 200.5027 | 812.5448 |
| 11 | 0.720931 | -1.35379 | 0.931776 | 198.7062 | 157.6355 | 733.6391 |
| 12 | 0.74556 | -1.37787 | 0.942856 | 165.4763 | 125.4166 | 665.8934 |
| 13 | 0.762416 | -1.41504 | 0.949222 | 139.2616 | 100.114 | 607.1533 |
| 14 | 0.778003 | -1.4395 | 0.955717 | 118.2847 | 80.60785 | 555.7985 |
| 15 | 0.787671 | -1.46186 | 0.96152 | 101.2888 | 65.44997 | 510.5816 |
| 16 | 0.798155 | -1.47793 | 0.967343 | 87.36592 | 53.47542 | 470.5232 |
| | | | | | | |
| 18 | 0.807496 | -1.51513 | 0.97065 | 66.23707 | 36.00195 | 402.9059 |
| 19 | 0.810826 | -1.53791 | 0.970439 | 58.15294 | 29.78609 | 374.1977 |
| 20 | 0.815668 | -1.55254 | 0.973513 | 51.30475 | 24.90469 | 348.2898 |
| 21 | 0.824861 | -1.55928 | 0.977894 | 45.46578 | 20.75476 | 324.8253 |
| 22 | 0.830408 | -1.56795 | 0.979832 | 40.4577 | 17.59585 | 303.5041 |
| 23 | 0.833691 | -1.57189 | 0.981647 | 36.13883 | 14.92471 | 284.0721 |
| 24 | 0.836964 | -1.58016 | 0.982806 | 32.39556 | 12.68326 | 266.3125 |
| 25 | 0.839541 | -1.58514 | 0.983845 | 29.13607 | 10.80145 | 250.0395 |
| 26 | 0.839272 | -1.59452 | 0.983276 | 26.28556 | 9.254207 | 235.0926 |
| 27 | 0.840414 | -1.59978 | 0.982982 | 23.78267 | 7.92939 | 221.3331 |
| 28 | 0.841379 | -1.60386 | 0.982483 | 21.57674 | 6.815265 | 208.6401 |
| 29 | 0.841804 | -1.60989 | 0.981738 | 19.62567 | 5.874364 | 196.9077 |
| 30 | 0.845777 | -1.61138 | 0.981928 | 17.89429 | 5.066654 | 186.0431 |

**Supplementary File S3: appropriate soft threshold powers for miRNA-seq-based matrix**

| Power | SFT.R.sq | slope | uncated.R.s | mean.k. | median.k. | max.k. |
|---|---|---|---|---|---|---|
| 1 | 0.740675 | 5.482195 | 0.81684 | 174.3791 | 178.879 | 199.8511 |
| 2 | 0.128873 | 0.785777 | 0.654867 | 99.67238 | 102.6416 | 132.9855 |
| 3 | 0.222015 | -0.76164 | 0.732618 | 60.142 | 60.96055 | 93.50137 |
| 4 | 0.611841 | -1.3123 | 0.843632 | 37.92397 | 37.25778 | 68.067 |
| 5 | 0.767718 | -1.42591 | 0.955712 | 24.81595 | 23.24931 | 50.85509 |
| | | | | | | |
| 7 | 0.86888 | -1.52346 | 0.928604 | 11.64748 | 9.889038 | 30.127 |
| 8 | 0.844447 | -1.46306 | 0.849317 | 8.295858 | 6.693764 | 23.75244 |
| 9 | 0.835298 | -1.41904 | 0.819336 | 6.041869 | 4.564141 | 18.97823 |
| 10 | 0.897584 | -1.38973 | 0.89501 | 4.489613 | 3.318213 | 15.3433 |
| 11 | 0.928728 | -1.35897 | 0.923971 | 3.397388 | 2.45043 | 12.53472 |
| 12 | 0.946152 | -1.33701 | 0.945757 | 2.613652 | 1.79006 | 10.33557 |
| 13 | 0.956992 | -1.34338 | 0.953245 | 2.041101 | 1.334426 | 8.781388 |
| 14 | 0.967039 | -1.37141 | 0.968737 | 1.615884 | 0.999564 | 7.573914 |
| 15 | 0.916614 | -1.43016 | 0.917148 | 1.295268 | 0.773255 | 6.580455 |
| 16 | 0.947355 | -1.4484 | 0.960851 | 1.050123 | 0.595531 | 5.753756 |
| 17 | 0.893118 | -1.49125 | 0.917821 | 0.860256 | 0.462213 | 5.05892 |
| 18 | 0.817353 | -1.55469 | 0.818221 | 0.711444 | 0.375304 | 4.469751 |
| 19 | 0.893672 | -1.53036 | 0.927377 | 0.593523 | 0.307761 | 3.966281 |
| 20 | 0.867478 | -1.55842 | 0.898552 | 0.499126 | 0.242412 | 3.533078 |
| 21 | 0.256888 | -2.87182 | 0.16351 | 0.42285 | 0.198273 | 3.158053 |
| 22 | 0.255675 | -2.8229 | 0.168756 | 0.360676 | 0.160171 | 2.831621 |
| 23 | 0.257 | -2.79572 | 0.170686 | 0.309587 | 0.129662 | 2.5461 |
| 24 | 0.256954 | -2.74545 | 0.17274 | 0.267292 | 0.102721 | 2.295267 |
| 25 | 0.256739 | -2.70682 | 0.170031 | 0.232032 | 0.082876 | 2.074034 |
| 26 | 0.904043 | -1.44425 | 0.93868 | 0.202445 | 0.068348 | 1.878209 |
| 27 | 0.915388 | -1.42744 | 0.953174 | 0.177468 | 0.057258 | 1.704304 |
| 28 | 0.894149 | -1.4045 | 0.915066 | 0.156263 | 0.047482 | 1.549404 |
| 29 | 0.890335 | -1.39136 | 0.899743 | 0.138165 | 0.039113 | 1.411052 |
| 30 | 0.897387 | -1.36778 | 0.908717 | 0.122641 | 0.032177 | 1.287165 |

**Supplementary File S4: Details about RNA-seq modules**

| Gene_id | Gene_symbol | Module_label | Module_color |
|---|---|---|---|
| ENSBTAG00000000144 | LOC100335205 | 7 | black |
| ENSBTAG00000000183 | NA | 7 | black |
| ENSBTAG00000000306 | ITK | 7 | black |
| ENSBTAG00000000431 | NA | 7 | black |
| ENSBTAG00000000432 | NA | 7 | black |
| ENSBTAG00000000641 | SKAP1 | 7 | black |
| ENSBTAG00000000715 | LOC530077 | 7 | black |
| ENSBTAG00000001002 | TCF7 | 7 | black |
| ENSBTAG00000001008 | PRF1 | 7 | black |
| ENSBTAG00000001198 | GIMAP7 | 7 | black |
| ENSBTAG00000001243 | AP3M2 | 7 | black |
| ENSBTAG00000001599 | SV2A | 7 | black |
| ENSBTAG00000001662 | EHD3 | 7 | black |
| ENSBTAG00000001882 | CD79A | 7 | black |
| ENSBTAG00000002100 | GZMM | 7 | black |
| ENSBTAG00000002127 | TCP11L2 | 7 | black |
| ENSBTAG00000002259 | NA | 7 | black |
| ENSBTAG00000002430 | COL17A1 | 7 | black |
| ENSBTAG00000002859 | NA | 7 | black |
| ENSBTAG00000002880 | SORBS1 | 7 | black |
| ENSBTAG00000003255 | CD4 | 7 | black |
| ENSBTAG00000003297 | OASL | 7 | black |
| ENSBTAG00000003408 | NA | 7 | black |
| ENSBTAG00000003668 | RADX | 7 | black |
| ENSBTAG00000003865 | CASR | 7 | black |
| ENSBTAG00000004022 | PLAG1 | 7 | black |
| ENSBTAG00000004273 | CST7 | 7 | black |
| ENSBTAG00000004458 | MORN3 | 7 | black |
| ENSBTAG00000004476 | ADHFE1 | 7 | black |
| ENSBTAG00000004499 | RGS3 | 7 | black |
| ENSBTAG00000004505 | NLRC3 | 7 | black |
| ENSBTAG00000004580 | MS4A1 | 7 | black |
| ENSBTAG00000004750 | VSTM2L | 7 | black |
| ENSBTAG00000004894 | GIMAP5 | 7 | black |
| ENSBTAG00000004899 | ABLIM1 | 7 | black |
| ENSBTAG00000004917 | KLRK1 | 7 | black |
| ENSBTAG00000005049 | BLK | 7 | black |
| ENSBTAG00000005055 | TXK | 7 | black |
| ENSBTAG00000005120 | TESPA1 | 7 | black |
| ENSBTAG00000005170 | ADGRG5 | 7 | black |
| ENSBTAG00000005628 | CD52 | 7 | black |
| ENSBTAG00000005699 | SCML4 | 7 | black |
| ENSBTAG00000005888 | MDGA1 | 7 | black |
| ENSBTAG00000005892 | ZAP70 | 7 | black |

| | | | |
|---|---|---|---|
| ENSBTAG00000006282 | POU2AF1 | 7 | black |
| ENSBTAG00000006452 | CD3D | 7 | black |
| ENSBTAG00000006453 | CD3G | 7 | black |
| ENSBTAG00000006519 | TNFRSF25 | 7 | black |
| ENSBTAG00000006552 | LAMP3 | 7 | black |
| ENSBTAG00000006688 | RPS6KL1 | 7 | black |
| ENSBTAG00000006703 | PTGDR | 7 | black |
| ENSBTAG00000006745 | FGG | 7 | black |
| ENSBTAG00000006844 | LEF1 | 7 | black |
| ENSBTAG00000007378 | CLIP4 | 7 | black |
| ENSBTAG00000007449 | HS3ST1 | 7 | black |
| ENSBTAG00000007855 | NA | 7 | black |
| ENSBTAG00000008074 | C1QTNF6 | 7 | black |
| ENSBTAG00000008154 | LIMD2 | 7 | black |
| ENSBTAG00000008453 | LBR | 7 | black |
| ENSBTAG00000008550 | GIMAP7 | 7 | black |
| ENSBTAG00000008555 | P2RY10 | 7 | black |
| ENSBTAG00000008686 | NA | 7 | black |
| ENSBTAG00000008717 | SERPINE2 | 7 | black |
| ENSBTAG00000008916 | CYFIP2 | 7 | black |
| ENSBTAG00000008956 | CD8B | 7 | black |
| ENSBTAG00000009151 | PYGO1 | 7 | black |
| ENSBTAG00000009216 | HVCN1 | 7 | black |
| ENSBTAG00000009266 | NA | 7 | black |
| ENSBTAG00000009357 | IL34 | 7 | black |
| ENSBTAG00000009850 | GIMAP7 | 7 | black |
| ENSBTAG00000010050 | NA | 7 | black |
| ENSBTAG00000010527 | NA | 7 | black |
| ENSBTAG00000010591 | ZNF365 | 7 | black |
| ENSBTAG00000010620 | ATP6V1B1 | 7 | black |
| ENSBTAG00000010667 | PIK3IP1 | 7 | black |
| ENSBTAG00000010717 | SSBP3 | 7 | black |
| ENSBTAG00000011003 | IKZF3 | 7 | black |
| ENSBTAG00000011019 | LOC618367 | 7 | black |
| ENSBTAG00000011170 | JAKMIP1 | 7 | black |
| ENSBTAG00000011240 | NA | 7 | black |
| ENSBTAG00000011359 | CD7 | 7 | black |
| ENSBTAG00000011411 | SIT1 | 7 | black |
| ENSBTAG00000011481 | IL12RB1 | 7 | black |
| ENSBTAG00000011887 | CD22 | 7 | black |
| ENSBTAG00000011940 | ZNF831 | 7 | black |
| ENSBTAG00000011990 | ALOX15 | 7 | black |
| ENSBTAG00000012029 | KLRB1 | 7 | black |
| ENSBTAG00000012112 | SLC7A6 | 7 | black |
| ENSBTAG00000012205 | CPT1C | 7 | black |
| ENSBTAG00000012695 | LCK | 7 | black |
| ENSBTAG00000012700 | CD247 | 7 | black |

| | | | |
|---|---|---|---|
| ENSBTAG00000012897 | ITGA7 | 7 | black |
| ENSBTAG00000013133 | TBC1D10C | 7 | black |
| ENSBTAG00000013292 | SIDT1 | 7 | black |
| ENSBTAG00000013301 | GGT7 | 7 | black |
| ENSBTAG00000013976 | AGBL3 | 7 | black |
| ENSBTAG00000014048 | SHLD1 | 7 | black |
| ENSBTAG00000014091 | ARHGEF3 | 7 | black |
| ENSBTAG00000014111 | INPP4B | 7 | black |
| ENSBTAG00000014156 | AMN1 | 7 | black |
| ENSBTAG00000014441 | CD96 | 7 | black |
| ENSBTAG00000014698 | CARD11 | 7 | black |
| ENSBTAG00000014725 | CD27 | 7 | black |
| ENSBTAG00000014798 | CXCR3 | 7 | black |
| ENSBTAG00000014809 | ANXA6 | 7 | black |
| ENSBTAG00000014944 | TBXA2R | 7 | black |
| ENSBTAG00000015297 | BANK1 | 7 | black |
| ENSBTAG00000015330 | NA | 7 | black |
| ENSBTAG00000015615 | THEMIS | 7 | black |
| ENSBTAG00000015708 | CXCR6 | 7 | black |
| ENSBTAG00000015710 | CD3E | 7 | black |
| ENSBTAG00000016045 | TRIB2 | 7 | black |
| ENSBTAG00000016124 | PACSIN1 | 7 | black |
| ENSBTAG00000016525 | ITGA1 | 7 | black |
| ENSBTAG00000016534 | BCL11A | 7 | black |
| ENSBTAG00000016920 | EOMES | 7 | black |
| ENSBTAG00000016948 | PTH1R | 7 | black |
| ENSBTAG00000017138 | TMEM71 | 7 | black |
| ENSBTAG00000017256 | CD2 | 7 | black |
| ENSBTAG00000017339 | RUNX1T1 | 7 | black |
| ENSBTAG00000017843 | LOC112445051 | 7 | black |
| ENSBTAG00000018019 | BCL11B | 7 | black |
| ENSBTAG00000018088 | SETBP1 | 7 | black |
| ENSBTAG00000018249 | KCNN3 | 7 | black |
| ENSBTAG00000018343 | NCR3 | 7 | black |
| ENSBTAG00000018367 | CD6 | 7 | black |
| ENSBTAG00000018513 | FHL1 | 7 | black |
| ENSBTAG00000018531 | JCHAIN | 7 | black |
| ENSBTAG00000019282 | CRYBA4 | 7 | black |
| ENSBTAG00000019371 | CNR2 | 7 | black |
| ENSBTAG00000019385 | SCN3A | 7 | black |
| ENSBTAG00000019940 | RASGRF1 | 7 | black |
| ENSBTAG00000020199 | F2R | 7 | black |
| ENSBTAG00000020247 | ADCYAP1R1 | 7 | black |
| ENSBTAG00000020374 | THSD1 | 7 | black |
| ENSBTAG00000020773 | CTSW | 7 | black |
| ENSBTAG00000021141 | CD8A | 7 | black |
| ENSBTAG00000021219 | SEPTIN1 | 7 | black |

| | | | |
|---|---|---|---|
| ENSBTAG00000021437 | GPR174 | 7 | black |
| ENSBTAG00000021596 | ICOS | 7 | black |
| ENSBTAG00000021958 | GZMA | 7 | black |
| ENSBTAG00000023398 | UBASH3A | 7 | black |
| ENSBTAG00000023611 | ZBTB32 | 7 | black |
| ENSBTAG00000024470 | SH2D2A | 7 | black |
| ENSBTAG00000024675 | CYSLTR1 | 7 | black |
| ENSBTAG00000025398 | LOC504548 | 7 | black |
| ENSBTAG00000025942 | HPGD | 7 | black |
| ENSBTAG00000026067 | SH2D1A | 7 | black |
| ENSBTAG00000027204 | LOC100300510 | 7 | black |
| ENSBTAG00000027246 | UBD | 7 | black |
| ENSBTAG00000030426 | LOC100848575 | 7 | black |
| ENSBTAG00000030523 | ZPBP2 | 7 | black |
| ENSBTAG00000030940 | GIMAP7 | 7 | black |
| ENSBTAG00000031160 | NA | 7 | black |
| ENSBTAG00000032125 | NA | 7 | black |
| ENSBTAG00000032534 | RHOF | 7 | black |
| ENSBTAG00000033803 | FABP7 | 7 | black |
| ENSBTAG00000034159 | FCRLA | 7 | black |
| ENSBTAG00000034385 | GPR162 | 7 | black |
| ENSBTAG00000034609 | NA | 7 | black |
| ENSBTAG00000035662 | CAMK4 | 7 | black |
| ENSBTAG00000037510 | LOC615733 | 7 | black |
| ENSBTAG00000038903 | NA | 7 | black |
| ENSBTAG00000039041 | NA | 7 | black |
| ENSBTAG00000039380 | NA | 7 | black |
| ENSBTAG00000039470 | NA | 7 | black |
| ENSBTAG00000039588 | LOC511617 | 7 | black |
| ENSBTAG00000039620 | SAPCD1 | 7 | black |
| ENSBTAG00000039823 | NA | 7 | black |
| ENSBTAG00000039928 | NA | 7 | black |
| ENSBTAG00000040331 | LOC512867 | 7 | black |
| ENSBTAG00000040607 | SLA2 | 7 | black |
| ENSBTAG00000043509 | LOC112444953 | 7 | black |
| ENSBTAG00000043953 | SPIB | 7 | black |
| ENSBTAG00000044204 | CD79B | 7 | black |
| ENSBTAG00000045966 | GPRIN3 | 7 | black |
| ENSBTAG00000046010 | BHLHA15 | 7 | black |
| ENSBTAG00000046076 | NA | 7 | black |
| ENSBTAG00000046257 | GIMAP4 | 7 | black |
| ENSBTAG00000046320 | CXCR5 | 7 | black |
| ENSBTAG00000046744 | PALM3 | 7 | black |
| ENSBTAG00000047121 | LOC100847119 | 7 | black |
| ENSBTAG00000047449 | LOC104968634 | 7 | black |
| ENSBTAG00000047529 | NA | 7 | black |
| ENSBTAG00000047700 | NA | 7 | black |

| | | | |
|---|---|---|---|
| ENSBTAG00000047707 | BOLA-DOB | 7 | black |
| ENSBTAG00000047816 | LOC100300483 | 7 | black |
| ENSBTAG00000047996 | NA | 7 | black |
| ENSBTAG00000048135 | IGCGAMMA | 7 | black |
| ENSBTAG00000048246 | DPP4 | 7 | black |
| ENSBTAG00000000015 | FOXRED2 | 2 | blue |
| ENSBTAG00000000021 | WASHC3 | 2 | blue |
| ENSBTAG00000000022 | NUP37 | 2 | blue |
| ENSBTAG00000000030 | RDM1 | 2 | blue |
| ENSBTAG00000000032 | ABI3 | 2 | blue |
| ENSBTAG00000000033 | PHOSPHO1 | 2 | blue |
| ENSBTAG00000000049 | CCDC77 | 2 | blue |
| ENSBTAG00000000056 | STRADA | 2 | blue |
| ENSBTAG00000000064 | FEN1 | 2 | blue |
| ENSBTAG00000000079 | CCSAP | 2 | blue |
| ENSBTAG00000000084 | MRPS30 | 2 | blue |
| ENSBTAG00000000090 | SEC31B | 2 | blue |
| ENSBTAG00000000105 | RIT1 | 2 | blue |
| ENSBTAG00000000133 | CD68 | 2 | blue |
| ENSBTAG00000000141 | NFU1 | 2 | blue |
| ENSBTAG00000000156 | NA | 2 | blue |
| ENSBTAG00000000170 | GSTT4 | 2 | blue |
| ENSBTAG00000000175 | N4BP2 | 2 | blue |
| ENSBTAG00000000186 | FLAD1 | 2 | blue |
| ENSBTAG00000000191 | SLC25A20 | 2 | blue |
| ENSBTAG00000000205 | MMD | 2 | blue |
| ENSBTAG00000000207 | PUDP | 2 | blue |
| ENSBTAG00000000218 | MAP2K5 | 2 | blue |
| ENSBTAG00000000285 | GUF1 | 2 | blue |
| ENSBTAG00000000286 | PFKM | 2 | blue |
| ENSBTAG00000000287 | GNPDA2 | 2 | blue |
| ENSBTAG00000000295 | CLHC1 | 2 | blue |
| ENSBTAG00000000297 | ZNF330 | 2 | blue |
| ENSBTAG00000000313 | NUDT13 | 2 | blue |
| ENSBTAG00000000321 | TBL2 | 2 | blue |
| ENSBTAG00000000342 | PLOD3 | 2 | blue |
| ENSBTAG00000000343 | ZNHIT1 | 2 | blue |
| ENSBTAG00000000369 | EPS8 | 2 | blue |
| ENSBTAG00000000371 | DIS3L | 2 | blue |
| ENSBTAG00000000376 | P2RX3 | 2 | blue |
| ENSBTAG00000000379 | GOLT1B | 2 | blue |
| ENSBTAG00000000390 | TPST1 | 2 | blue |
| ENSBTAG00000000404 | NAXE | 2 | blue |
| ENSBTAG00000000413 | NRTN | 2 | blue |
| ENSBTAG00000000422 | TSHZ1 | 2 | blue |
| ENSBTAG00000000435 | TARBP2 | 2 | blue |
| ENSBTAG00000000447 | FRA10AC1 | 2 | blue |

| | | | |
|---|---|---|---|
| ENSBTAG00000000454 | TMEM106C | 2 | blue |
| ENSBTAG00000000472 | ZNF570 | 2 | blue |
| ENSBTAG00000000475 | IMMP1L | 2 | blue |
| ENSBTAG00000000477 | TLR5 | 2 | blue |
| ENSBTAG00000000489 | WDR54 | 2 | blue |
| ENSBTAG00000000495 | HAVCR2 | 2 | blue |
| ENSBTAG00000000497 | AKR1A1 | 2 | blue |
| ENSBTAG00000000512 | SNTA1 | 2 | blue |
| ENSBTAG00000000517 | ANKRD29 | 2 | blue |
| ENSBTAG00000000521 | PLEKHA8 | 2 | blue |
| ENSBTAG00000000532 | EXO5 | 2 | blue |
| ENSBTAG00000000533 | ZNF684 | 2 | blue |
| ENSBTAG00000000536 | ZNF395 | 2 | blue |
| ENSBTAG00000000539 | LRRC28 | 2 | blue |
| ENSBTAG00000000542 | CFAP36 | 2 | blue |
| ENSBTAG00000000555 | ACO1 | 2 | blue |
| ENSBTAG00000000565 | RFX7 | 2 | blue |
| ENSBTAG00000000601 | COL11A2 | 2 | blue |
| ENSBTAG00000000604 | GPNMB | 2 | blue |
| ENSBTAG00000000629 | MMS22L | 2 | blue |
| ENSBTAG00000000632 | RAD54B | 2 | blue |
| ENSBTAG00000000666 | GHDC | 2 | blue |
| ENSBTAG00000000668 | SLC22A5 | 2 | blue |
| ENSBTAG00000000679 | PGM3 | 2 | blue |
| ENSBTAG00000000687 | POC1B | 2 | blue |
| ENSBTAG00000000704 | GUSB | 2 | blue |
| ENSBTAG00000000713 | AGFG2 | 2 | blue |
| ENSBTAG00000000758 | PACC1 | 2 | blue |
| ENSBTAG00000000759 | NENF | 2 | blue |
| ENSBTAG00000000764 | GIPC1 | 2 | blue |
| ENSBTAG00000000785 | PASK | 2 | blue |
| ENSBTAG00000000795 | NMNAT2 | 2 | blue |
| ENSBTAG00000000799 | ICA1 | 2 | blue |
| ENSBTAG00000000803 | ZNF667 | 2 | blue |
| ENSBTAG00000000807 | TMED3 | 2 | blue |
| ENSBTAG00000000829 | SHPK | 2 | blue |
| ENSBTAG00000000831 | CTNS | 2 | blue |
| ENSBTAG00000000837 | PARP1 | 2 | blue |
| ENSBTAG00000000838 | CCDC88C | 2 | blue |
| ENSBTAG00000000842 | UBASH3B | 2 | blue |
| ENSBTAG00000000843 | NDRG2 | 2 | blue |
| ENSBTAG00000000851 | CYP20A1 | 2 | blue |
| ENSBTAG00000000855 | NXN | 2 | blue |
| ENSBTAG00000000856 | FBXL6 | 2 | blue |
| ENSBTAG00000000868 | ZFP1 | 2 | blue |
| ENSBTAG00000000875 | CEP131 | 2 | blue |
| ENSBTAG00000000877 | ETFBKMT | 2 | blue |

| | | | |
|---|---|---|---|
| ENSBTAG00000000895 | TAF9B | 2 | blue |
| ENSBTAG00000000918 | ERMARD | 2 | blue |
| ENSBTAG00000000933 | CCDC183 | 2 | blue |
| ENSBTAG00000000942 | SIPA1L2 | 2 | blue |
| ENSBTAG00000000949 | DNAJC9 | 2 | blue |
| ENSBTAG00000000962 | STAP1 | 2 | blue |
| ENSBTAG00000000977 | CADM1 | 2 | blue |
| ENSBTAG00000000988 | BRCA2 | 2 | blue |
| ENSBTAG00000001005 | ZNF280B | 2 | blue |
| ENSBTAG00000001037 | MAP4K2 | 2 | blue |
| ENSBTAG00000001050 | LDLRAP1 | 2 | blue |
| ENSBTAG00000001061 | PRKCA | 2 | blue |
| ENSBTAG00000001077 | OSBPL5 | 2 | blue |
| ENSBTAG00000001078 | SRL | 2 | blue |
| ENSBTAG00000001092 | PHOSPHO2 | 2 | blue |
| ENSBTAG00000001093 | KLHL23 | 2 | blue |
| ENSBTAG00000001097 | FKBP7 | 2 | blue |
| ENSBTAG00000001113 | NDUFAF7 | 2 | blue |
| ENSBTAG00000001114 | PRKD3 | 2 | blue |
| ENSBTAG00000001116 | P4HA2 | 2 | blue |
| ENSBTAG00000001133 | VWA8 | 2 | blue |
| ENSBTAG00000001139 | ACHE | 2 | blue |
| ENSBTAG00000001142 | GNE | 2 | blue |
| ENSBTAG00000001151 | APLP1 | 2 | blue |
| ENSBTAG00000001164 | ACAD10 | 2 | blue |
| ENSBTAG00000001165 | AIFM2 | 2 | blue |
| ENSBTAG00000001181 | RPS6KC1 | 2 | blue |
| ENSBTAG00000001183 | KLHL33 | 2 | blue |
| ENSBTAG00000001187 | RASL10A | 2 | blue |
| ENSBTAG00000001189 | AP1B1 | 2 | blue |
| ENSBTAG00000001192 | STX12 | 2 | blue |
| ENSBTAG00000001219 | NA | 2 | blue |
| ENSBTAG00000001257 | AGTPBP1 | 2 | blue |
| ENSBTAG00000001274 | PPM1L | 2 | blue |
| ENSBTAG00000001282 | RALGAPA1 | 2 | blue |
| ENSBTAG00000001289 | MRPL18 | 2 | blue |
| ENSBTAG00000001305 | ATP2B2 | 2 | blue |
| ENSBTAG00000001333 | PPARG | 2 | blue |
| ENSBTAG00000001354 | INPP5E | 2 | blue |
| ENSBTAG00000001361 | NA | 2 | blue |
| ENSBTAG00000001364 | DPH6 | 2 | blue |
| ENSBTAG00000001408 | CNRIP1 | 2 | blue |
| ENSBTAG00000001410 | GRAMD1B | 2 | blue |
| ENSBTAG00000001422 | NA | 2 | blue |
| ENSBTAG00000001424 | MSH6 | 2 | blue |
| ENSBTAG00000001444 | TNXB | 2 | blue |
| ENSBTAG00000001457 | TMEM267 | 2 | blue |

| | | | |
|---|---|---|---|
| ENSBTAG00000001475 | MCUR1 | 2 | blue |
| ENSBTAG00000001484 | LZTFL1 | 2 | blue |
| ENSBTAG00000001489 | TUBA1A | 2 | blue |
| ENSBTAG00000001497 | MRAS | 2 | blue |
| ENSBTAG00000001512 | ZFP64 | 2 | blue |
| ENSBTAG00000001543 | BRI3BP | 2 | blue |
| ENSBTAG00000001548 | NA | 2 | blue |
| ENSBTAG00000001593 | OGFOD2 | 2 | blue |
| ENSBTAG00000001595 | MT1E | 2 | blue |
| ENSBTAG00000001608 | NA | 2 | blue |
| ENSBTAG00000001609 | MAP2K6 | 2 | blue |
| ENSBTAG00000001614 | ZNF174 | 2 | blue |
| ENSBTAG00000001619 | SERINC5 | 2 | blue |
| ENSBTAG00000001621 | NA | 2 | blue |
| ENSBTAG00000001629 | SCMH1 | 2 | blue |
| ENSBTAG00000001692 | NDST1 | 2 | blue |
| ENSBTAG00000001693 | RPAP1 | 2 | blue |
| ENSBTAG00000001702 | TMEM107 | 2 | blue |
| ENSBTAG00000001714 | MFSD9 | 2 | blue |
| ENSBTAG00000001724 | PTGR3 | 2 | blue |
| ENSBTAG00000001727 | PRXL2B | 2 | blue |
| ENSBTAG00000001748 | TRIM45 | 2 | blue |
| ENSBTAG00000001752 | FUCA2 | 2 | blue |
| ENSBTAG00000001776 | SIRT2 | 2 | blue |
| ENSBTAG00000001786 | HYKK | 2 | blue |
| ENSBTAG00000001796 | CATSPERD | 2 | blue |
| ENSBTAG00000001805 | TWSG1 | 2 | blue |
| ENSBTAG00000001808 | LACTB2 | 2 | blue |
| ENSBTAG00000001817 | TMEM161A | 2 | blue |
| ENSBTAG00000001818 | MEF2B | 2 | blue |
| ENSBTAG00000001826 | SASH1 | 2 | blue |
| ENSBTAG00000001832 | MRPL10 | 2 | blue |
| ENSBTAG00000001843 | WBP1 | 2 | blue |
| ENSBTAG00000001844 | MOGS | 2 | blue |
| ENSBTAG00000001847 | CNTLN | 2 | blue |
| ENSBTAG00000001877 | HMGN5 | 2 | blue |
| ENSBTAG00000001885 | NA | 2 | blue |
| ENSBTAG00000001886 | PABIR2 | 2 | blue |
| ENSBTAG00000001889 | C22H3orf14 | 2 | blue |
| ENSBTAG00000001894 | NEDD1 | 2 | blue |
| ENSBTAG00000001908 | DLD | 2 | blue |
| ENSBTAG00000001932 | CRAT | 2 | blue |
| ENSBTAG00000001938 | CKS2 | 2 | blue |
| ENSBTAG00000001949 | NA | 2 | blue |
| ENSBTAG00000001957 | TRMT11 | 2 | blue |
| ENSBTAG00000002002 | NA | 2 | blue |
| ENSBTAG00000002008 | NA | 2 | blue |

| | | | |
|---|---|---|---|
| ENSBTAG00000002028 | DIPK1A | 2 | blue |
| ENSBTAG00000002037 | ATXN7L1 | 2 | blue |
| ENSBTAG00000002041 | SASH3 | 2 | blue |
| ENSBTAG00000002044 | SIRT3 | 2 | blue |
| ENSBTAG00000002049 | HADH | 2 | blue |
| ENSBTAG00000002058 | ASF1A | 2 | blue |
| ENSBTAG00000002083 | AUTS2 | 2 | blue |
| ENSBTAG00000002087 | IL36A | 2 | blue |
| ENSBTAG00000002101 | GDE1 | 2 | blue |
| ENSBTAG00000002108 | YWHAQ | 2 | blue |
| ENSBTAG00000002113 | NA | 2 | blue |
| ENSBTAG00000002117 | KIF18A | 2 | blue |
| ENSBTAG00000002128 | MPPE1 | 2 | blue |
| ENSBTAG00000002136 | FCHO1 | 2 | blue |
| ENSBTAG00000002169 | NA | 2 | blue |
| ENSBTAG00000002179 | CDIN1 | 2 | blue |
| ENSBTAG00000002184 | NA | 2 | blue |
| ENSBTAG00000002211 | MAPK8IP3 | 2 | blue |
| ENSBTAG00000002223 | POP4 | 2 | blue |
| ENSBTAG00000002260 | NCAPD3 | 2 | blue |
| ENSBTAG00000002266 | NPL | 2 | blue |
| ENSBTAG00000002286 | DNAJC18 | 2 | blue |
| ENSBTAG00000002305 | PCSK4 | 2 | blue |
| ENSBTAG00000002316 | NIPA1 | 2 | blue |
| ENSBTAG00000002321 | AMT | 2 | blue |
| ENSBTAG00000002326 | LGALS3 | 2 | blue |
| ENSBTAG00000002328 | KDM2B | 2 | blue |
| ENSBTAG00000002332 | LSM5 | 2 | blue |
| ENSBTAG00000002350 | PIK3R2 | 2 | blue |
| ENSBTAG00000002352 | GGA2 | 2 | blue |
| ENSBTAG00000002367 | HCFC1R1 | 2 | blue |
| ENSBTAG00000002395 | HPS3 | 2 | blue |
| ENSBTAG00000002398 | NA | 2 | blue |
| ENSBTAG00000002428 | PPA2 | 2 | blue |
| ENSBTAG00000002464 | PLCH2 | 2 | blue |
| ENSBTAG00000002467 | NA | 2 | blue |
| ENSBTAG00000002470 | CCDC78 | 2 | blue |
| ENSBTAG00000002472 | CASP9 | 2 | blue |
| ENSBTAG00000002473 | ANGPTL4 | 2 | blue |
| ENSBTAG00000002481 | HAGHL | 2 | blue |
| ENSBTAG00000002490 | CHPT1 | 2 | blue |
| ENSBTAG00000002492 | SYCP3 | 2 | blue |
| ENSBTAG00000002503 | BSCL2 | 2 | blue |
| ENSBTAG00000002508 | GNG3 | 2 | blue |
| ENSBTAG00000002524 | MRI1 | 2 | blue |
| ENSBTAG00000002526 | BDH2 | 2 | blue |
| ENSBTAG00000002531 | ARHGAP10 | 2 | blue |

| | | | |
|---|---|---|---|
| ENSBTAG00000002557 | MRPL19 | 2 | blue |
| ENSBTAG00000002564 | DLG4 | 2 | blue |
| ENSBTAG00000002566 | PGPEP1 | 2 | blue |
| ENSBTAG00000002570 | PPP1R21 | 2 | blue |
| ENSBTAG00000002580 | MYH14 | 2 | blue |
| ENSBTAG00000002599 | MGARP | 2 | blue |
| ENSBTAG00000002600 | EFCAB7 | 2 | blue |
| ENSBTAG00000002610 | FKBP3 | 2 | blue |
| ENSBTAG00000002612 | FANCM | 2 | blue |
| ENSBTAG00000002614 | RUFY2 | 2 | blue |
| ENSBTAG00000002633 | SEPTIN9 | 2 | blue |
| ENSBTAG00000002640 | KALRN | 2 | blue |
| ENSBTAG00000002660 | NA | 2 | blue |
| ENSBTAG00000002663 | BCAS3 | 2 | blue |
| ENSBTAG00000002689 | NME7 | 2 | blue |
| ENSBTAG00000002703 | ATP6V1A | 2 | blue |
| ENSBTAG00000002706 | GSTZ1 | 2 | blue |
| ENSBTAG00000002708 | MRPL42 | 2 | blue |
| ENSBTAG00000002721 | TUT4 | 2 | blue |
| ENSBTAG00000002736 | DNMT1 | 2 | blue |
| ENSBTAG00000002737 | WFIKKN1 | 2 | blue |
| ENSBTAG00000002747 | ABCA5 | 2 | blue |
| ENSBTAG00000002767 | GNB4 | 2 | blue |
| ENSBTAG00000002782 | ZC3H7B | 2 | blue |
| ENSBTAG00000002783 | PCYOX1 | 2 | blue |
| ENSBTAG00000002786 | LOC781494 | 2 | blue |
| ENSBTAG00000002817 | SLC46A1 | 2 | blue |
| ENSBTAG00000002820 | RBBP7 | 2 | blue |
| ENSBTAG00000002821 | NA | 2 | blue |
| ENSBTAG00000002824 | DPEP3 | 2 | blue |
| ENSBTAG00000002843 | ACOT4 | 2 | blue |
| ENSBTAG00000002885 | MSR1 | 2 | blue |
| ENSBTAG00000002888 | TMTC2 | 2 | blue |
| ENSBTAG00000002894 | TNFSF4 | 2 | blue |
| ENSBTAG00000002918 | RAD51 | 2 | blue |
| ENSBTAG00000002938 | CPED1 | 2 | blue |
| ENSBTAG00000002953 | TXN | 2 | blue |
| ENSBTAG00000002955 | NA | 2 | blue |
| ENSBTAG00000002956 | ZNF674 | 2 | blue |
| ENSBTAG00000002995 | GMPPA | 2 | blue |
| ENSBTAG00000003014 | TRPV2 | 2 | blue |
| ENSBTAG00000003015 | SESN1 | 2 | blue |
| ENSBTAG00000003018 | FSTL3 | 2 | blue |
| ENSBTAG00000003035 | IMPACT | 2 | blue |
| ENSBTAG00000003043 | GNG2 | 2 | blue |
| ENSBTAG00000003069 | MAN1C1 | 2 | blue |
| ENSBTAG00000003072 | ACADVL | 2 | blue |

| | | | |
|---|---|---|---|
| ENSBTAG00000003076 | NT5DC1 | 2 | blue |
| ENSBTAG00000003081 | RWDD4 | 2 | blue |
| ENSBTAG00000003083 | SLC50A1 | 2 | blue |
| ENSBTAG00000003097 | DENND4C | 2 | blue |
| ENSBTAG00000003100 | SMTN | 2 | blue |
| ENSBTAG00000003143 | TBCCD1 | 2 | blue |
| ENSBTAG00000003161 | YBEY | 2 | blue |
| ENSBTAG00000003162 | CRYZ | 2 | blue |
| ENSBTAG00000003174 | NDUFAF4 | 2 | blue |
| ENSBTAG00000003180 | TMEM201 | 2 | blue |
| ENSBTAG00000003189 | CCDC6 | 2 | blue |
| ENSBTAG00000003196 | PAPSS2 | 2 | blue |
| ENSBTAG00000003242 | ACAD9 | 2 | blue |
| ENSBTAG00000003244 | CFAP92 | 2 | blue |
| ENSBTAG00000003265 | ADD3 | 2 | blue |
| ENSBTAG00000003300 | MFGE8 | 2 | blue |
| ENSBTAG00000003328 | TAMM41 | 2 | blue |
| ENSBTAG00000003330 | ATL3 | 2 | blue |
| ENSBTAG00000003358 | MCFD2 | 2 | blue |
| ENSBTAG00000003361 | METTL15 | 2 | blue |
| ENSBTAG00000003405 | TP53I3 | 2 | blue |
| ENSBTAG00000003415 | ESD | 2 | blue |
| ENSBTAG00000003422 | SNX5 | 2 | blue |
| ENSBTAG00000003424 | BET1 | 2 | blue |
| ENSBTAG00000003425 | MGME1 | 2 | blue |
| ENSBTAG00000003443 | USP46 | 2 | blue |
| ENSBTAG00000003447 | NA | 2 | blue |
| ENSBTAG00000003450 | ATP6V1H | 2 | blue |
| ENSBTAG00000003454 | RGS20 | 2 | blue |
| ENSBTAG00000003470 | TTYH1 | 2 | blue |
| ENSBTAG00000003491 | SLC25A23 | 2 | blue |
| ENSBTAG00000003494 | DNAAF9 | 2 | blue |
| ENSBTAG00000003514 | HSF4 | 2 | blue |
| ENSBTAG00000003521 | KIAA0895L | 2 | blue |
| ENSBTAG00000003544 | S1PR5 | 2 | blue |
| ENSBTAG00000003548 | GSTP1 | 2 | blue |
| ENSBTAG00000003550 | C29H11orf54 | 2 | blue |
| ENSBTAG00000003556 | ZFYVE21 | 2 | blue |
| ENSBTAG00000003565 | TRIM3 | 2 | blue |
| ENSBTAG00000003576 | POLL | 2 | blue |
| ENSBTAG00000003578 | DPCD | 2 | blue |
| ENSBTAG00000003579 | FBXW4 | 2 | blue |
| ENSBTAG00000003601 | ZMAT1 | 2 | blue |
| ENSBTAG00000003606 | GLI4 | 2 | blue |
| ENSBTAG00000003607 | FAM20C | 2 | blue |
| ENSBTAG00000003610 | DENND1A | 2 | blue |
| ENSBTAG00000003616 | DCLRE1B | 2 | blue |

| | | | |
|---|---|---|---|
| ENSBTAG00000003619 | SEC24D | 2 | blue |
| ENSBTAG00000003622 | ZKSCAN7 | 2 | blue |
| ENSBTAG00000003629 | ASPA | 2 | blue |
| ENSBTAG00000003634 | CISD1 | 2 | blue |
| ENSBTAG00000003636 | LIPA | 2 | blue |
| ENSBTAG00000003642 | PRDX1 | 2 | blue |
| ENSBTAG00000003699 | ZFP3 | 2 | blue |
| ENSBTAG00000003709 | ITGB8 | 2 | blue |
| ENSBTAG00000003718 | HACL1 | 2 | blue |
| ENSBTAG00000003749 | KCNMB4 | 2 | blue |
| ENSBTAG00000003762 | GNB5 | 2 | blue |
| ENSBTAG00000003779 | ZC4H2 | 2 | blue |
| ENSBTAG00000003800 | LRRC27 | 2 | blue |
| ENSBTAG00000003802 | TEKT3 | 2 | blue |
| ENSBTAG00000003804 | SCCPDH | 2 | blue |
| ENSBTAG00000003815 | EDEM2 | 2 | blue |
| ENSBTAG00000003819 | STK36 | 2 | blue |
| ENSBTAG00000003832 | MFAP2 | 2 | blue |
| ENSBTAG00000003833 | SLC35G2 | 2 | blue |
| ENSBTAG00000003837 | RSPO1 | 2 | blue |
| ENSBTAG00000003855 | FAM120C | 2 | blue |
| ENSBTAG00000003861 | GPATCH11 | 2 | blue |
| ENSBTAG00000003863 | PCED1A | 2 | blue |
| ENSBTAG00000003887 | ECHDC1 | 2 | blue |
| ENSBTAG00000003901 | DNMT3B | 2 | blue |
| ENSBTAG00000003902 | ZNF512 | 2 | blue |
| ENSBTAG00000003914 | MVB12A | 2 | blue |
| ENSBTAG00000003946 | NECAB3 | 2 | blue |
| ENSBTAG00000003952 | FBXL2 | 2 | blue |
| ENSBTAG00000003956 | SGCA | 2 | blue |
| ENSBTAG00000003958 | TMEM220 | 2 | blue |
| ENSBTAG00000003963 | FER1L5 | 2 | blue |
| ENSBTAG00000003965 | LIPT1 | 2 | blue |
| ENSBTAG00000003971 | E2F1 | 2 | blue |
| ENSBTAG00000003975 | LMAN2L | 2 | blue |
| ENSBTAG00000003983 | TOM1L2 | 2 | blue |
| ENSBTAG00000003989 | GSTO1 | 2 | blue |
| ENSBTAG00000003997 | UBE2D4 | 2 | blue |
| ENSBTAG00000004009 | PMM1 | 2 | blue |
| ENSBTAG00000004010 | PAPPA | 2 | blue |
| ENSBTAG00000004011 | GALK2 | 2 | blue |
| ENSBTAG00000004018 | DGKA | 2 | blue |
| ENSBTAG00000004019 | PMEL | 2 | blue |
| ENSBTAG00000004034 | SESN3 | 2 | blue |
| ENSBTAG00000004092 | AK8 | 2 | blue |
| ENSBTAG00000004093 | NA | 2 | blue |
| ENSBTAG00000004097 | A2ML1 | 2 | blue |

| | | | |
|---|---|---|---|
| ENSBTAG00000004100 | TTI1 | 2 | blue |
| ENSBTAG00000004104 | RUNX2 | 2 | blue |
| ENSBTAG00000004108 | CENPX | 2 | blue |
| ENSBTAG00000004111 | LRRC45 | 2 | blue |
| ENSBTAG00000004140 | STKLD1 | 2 | blue |
| ENSBTAG00000004155 | SPATA20 | 2 | blue |
| ENSBTAG00000004165 | NA | 2 | blue |
| ENSBTAG00000004177 | TANC1 | 2 | blue |
| ENSBTAG00000004184 | WDSUB1 | 2 | blue |
| ENSBTAG00000004227 | TBCK | 2 | blue |
| ENSBTAG00000004240 | TMPO | 2 | blue |
| ENSBTAG00000004248 | MLYCD | 2 | blue |
| ENSBTAG00000004249 | TANC2 | 2 | blue |
| ENSBTAG00000004281 | ACSS1 | 2 | blue |
| ENSBTAG00000004288 | GSTA4 | 2 | blue |
| ENSBTAG00000004294 | EVI5L | 2 | blue |
| ENSBTAG00000004318 | ARL3 | 2 | blue |
| ENSBTAG00000004321 | SFXN2 | 2 | blue |
| ENSBTAG00000004328 | CHST10 | 2 | blue |
| ENSBTAG00000004337 | PDE1B | 2 | blue |
| ENSBTAG00000004364 | THNSL2 | 2 | blue |
| ENSBTAG00000004367 | COQ10A | 2 | blue |
| ENSBTAG00000004368 | NFATC3 | 2 | blue |
| ENSBTAG00000004371 | CS | 2 | blue |
| ENSBTAG00000004374 | CNPY2 | 2 | blue |
| ENSBTAG00000004379 | ETHE1 | 2 | blue |
| ENSBTAG00000004394 | PKIB | 2 | blue |
| ENSBTAG00000004398 | IMMP2L | 2 | blue |
| ENSBTAG00000004399 | LRRN3 | 2 | blue |
| ENSBTAG00000004409 | IVD | 2 | blue |
| ENSBTAG00000004421 | CHST14 | 2 | blue |
| ENSBTAG00000004422 | ALG14 | 2 | blue |
| ENSBTAG00000004428 | H2AZ1 | 2 | blue |
| ENSBTAG00000004448 | CKAP5 | 2 | blue |
| ENSBTAG00000004459 | TMEM45A | 2 | blue |
| ENSBTAG00000004460 | RFWD3 | 2 | blue |
| ENSBTAG00000004495 | UBA5 | 2 | blue |
| ENSBTAG00000004502 | CLUAP1 | 2 | blue |
| ENSBTAG00000004509 | SLX4 | 2 | blue |
| ENSBTAG00000004517 | TARBP1 | 2 | blue |
| ENSBTAG00000004524 | SPPL2B | 2 | blue |
| ENSBTAG00000004541 | LPAR5 | 2 | blue |
| ENSBTAG00000004568 | GALNS | 2 | blue |
| ENSBTAG00000004585 | NA | 2 | blue |
| ENSBTAG00000004588 | KCNN4 | 2 | blue |
| ENSBTAG00000004590 | PPIH | 2 | blue |
| ENSBTAG00000004597 | PIM2 | 2 | blue |

| | | | |
|---|---|---|---|
| ENSBTAG00000004601 | ABHD10 | 2 | blue |
| ENSBTAG00000004624 | NA | 2 | blue |
| ENSBTAG00000004631 | KIAA0586 | 2 | blue |
| ENSBTAG00000004639 | HARBI1 | 2 | blue |
| ENSBTAG00000004652 | LPAR2 | 2 | blue |
| ENSBTAG00000004654 | PWP1 | 2 | blue |
| ENSBTAG00000004660 | SPATS2 | 2 | blue |
| ENSBTAG00000004664 | COQ4 | 2 | blue |
| ENSBTAG00000004690 | NA | 2 | blue |
| ENSBTAG00000004706 | SUV39H1 | 2 | blue |
| ENSBTAG00000004722 | THUMPD2 | 2 | blue |
| ENSBTAG00000004727 | PAQR4 | 2 | blue |
| ENSBTAG00000004729 | PKMYT1 | 2 | blue |
| ENSBTAG00000004735 | CCNE1 | 2 | blue |
| ENSBTAG00000004749 | NEK4 | 2 | blue |
| ENSBTAG00000004757 | LTBP4 | 2 | blue |
| ENSBTAG00000004769 | NEIL2 | 2 | blue |
| ENSBTAG00000004786 | TMEM134 | 2 | blue |
| ENSBTAG00000004788 | SDR39U1 | 2 | blue |
| ENSBTAG00000004790 | UBE2T | 2 | blue |
| ENSBTAG00000004806 | PHKB | 2 | blue |
| ENSBTAG00000004826 | ACP5 | 2 | blue |
| ENSBTAG00000004854 | SLC24A5 | 2 | blue |
| ENSBTAG00000004856 | RNF130 | 2 | blue |
| ENSBTAG00000004877 | TRAIP | 2 | blue |
| ENSBTAG00000004925 | NA | 2 | blue |
| ENSBTAG00000004926 | LRR1 | 2 | blue |
| ENSBTAG00000004933 | KLHDC2 | 2 | blue |
| ENSBTAG00000004939 | NA | 2 | blue |
| ENSBTAG00000004941 | TEX35 | 2 | blue |
| ENSBTAG00000004942 | EXOSC9 | 2 | blue |
| ENSBTAG00000004945 | BBS7 | 2 | blue |
| ENSBTAG00000004950 | BRB | 2 | blue |
| ENSBTAG00000004956 | CHEK2 | 2 | blue |
| ENSBTAG00000004991 | ELP1 | 2 | blue |
| ENSBTAG00000004993 | NXT1 | 2 | blue |
| ENSBTAG00000005002 | GALE | 2 | blue |
| ENSBTAG00000005007 | NA | 2 | blue |
| ENSBTAG00000005015 | SFXN3 | 2 | blue |
| ENSBTAG00000005033 | TLCD3B | 2 | blue |
| ENSBTAG00000005078 | UCHL1 | 2 | blue |
| ENSBTAG00000005083 | TULP4 | 2 | blue |
| ENSBTAG00000005090 | CEMIP2 | 2 | blue |
| ENSBTAG00000005093 | CES2 | 2 | blue |
| ENSBTAG00000005119 | PSMD1 | 2 | blue |
| ENSBTAG00000005140 | NBL1 | 2 | blue |
| ENSBTAG00000005144 | PNPLA2 | 2 | blue |

| | | | |
|---|---|---|---|
| ENSBTAG00000005154 | APCDD1 | 2 | blue |
| ENSBTAG00000005193 | CRTAM | 2 | blue |
| ENSBTAG00000005203 | C1H3orf33 | 2 | blue |
| ENSBTAG00000005206 | TMEM14A | 2 | blue |
| ENSBTAG00000005213 | ZWILCH | 2 | blue |
| ENSBTAG00000005219 | KLHL4 | 2 | blue |
| ENSBTAG00000005221 | WNK1 | 2 | blue |
| ENSBTAG00000005239 | NA | 2 | blue |
| ENSBTAG00000005260 | SPP1 | 2 | blue |
| ENSBTAG00000005261 | FCGBP | 2 | blue |
| ENSBTAG00000005275 | PKIG | 2 | blue |
| ENSBTAG00000005288 | GFRA3 | 2 | blue |
| ENSBTAG00000005299 | EDNRB | 2 | blue |
| ENSBTAG00000005300 | TMEM51 | 2 | blue |
| ENSBTAG00000005304 | SRM | 2 | blue |
| ENSBTAG00000005309 | TGDS | 2 | blue |
| ENSBTAG00000005310 | GPR180 | 2 | blue |
| ENSBTAG00000005311 | POLR3H | 2 | blue |
| ENSBTAG00000005328 | ICE2 | 2 | blue |
| ENSBTAG00000005340 | SERGEF | 2 | blue |
| ENSBTAG00000005347 | GPN3 | 2 | blue |
| ENSBTAG00000005351 | RFXAP | 2 | blue |
| ENSBTAG00000005362 | RAB23 | 2 | blue |
| ENSBTAG00000005364 | HMBS | 2 | blue |
| ENSBTAG00000005367 | VRK3 | 2 | blue |
| ENSBTAG00000005370 | TMTC1 | 2 | blue |
| ENSBTAG00000005379 | SLC41A1 | 2 | blue |
| ENSBTAG00000005393 | TIPRL | 2 | blue |
| ENSBTAG00000005403 | ADI1 | 2 | blue |
| ENSBTAG00000005416 | PPIL3 | 2 | blue |
| ENSBTAG00000005431 | LMCD1 | 2 | blue |
| ENSBTAG00000005442 | DNAI4 | 2 | blue |
| ENSBTAG00000005445 | SLC35D1 | 2 | blue |
| ENSBTAG00000005482 | TMEM135 | 2 | blue |
| ENSBTAG00000005490 | P4HTM | 2 | blue |
| ENSBTAG00000005532 | PEX6 | 2 | blue |
| ENSBTAG00000005533 | PPP2R5D | 2 | blue |
| ENSBTAG00000005571 | PHF20 | 2 | blue |
| ENSBTAG00000005572 | ZNF205 | 2 | blue |
| ENSBTAG00000005574 | CLU | 2 | blue |
| ENSBTAG00000005577 | RNASEH2C | 2 | blue |
| ENSBTAG00000005583 | HYI | 2 | blue |
| ENSBTAG00000005586 | GATM | 2 | blue |
| ENSBTAG00000005592 | MCOLN1 | 2 | blue |
| ENSBTAG00000005617 | SLC41A3 | 2 | blue |
| ENSBTAG00000005666 | LRRC20 | 2 | blue |
| ENSBTAG00000005683 | ELAC1 | 2 | blue |

| | | | |
|---|---|---|---|
| ENSBTAG00000005696 | DBF4B | 2 | blue |
| ENSBTAG00000005708 | KIF20B | 2 | blue |
| ENSBTAG00000005718 | PLIN2 | 2 | blue |
| ENSBTAG00000005722 | NA | 2 | blue |
| ENSBTAG00000005729 | FBXL4 | 2 | blue |
| ENSBTAG00000005756 | ZDHHC24 | 2 | blue |
| ENSBTAG00000005760 | TBC1D4 | 2 | blue |
| ENSBTAG00000005765 | SYNGR1 | 2 | blue |
| ENSBTAG00000005785 | DPY19L1 | 2 | blue |
| ENSBTAG00000005787 | IFT57 | 2 | blue |
| ENSBTAG00000005788 | VANGL1 | 2 | blue |
| ENSBTAG00000005791 | UROS | 2 | blue |
| ENSBTAG00000005812 | TP73 | 2 | blue |
| ENSBTAG00000005827 | FIG4 | 2 | blue |
| ENSBTAG00000005828 | MERTK | 2 | blue |
| ENSBTAG00000005845 | MPI | 2 | blue |
| ENSBTAG00000005862 | SMC4 | 2 | blue |
| ENSBTAG00000005868 | HEBP1 | 2 | blue |
| ENSBTAG00000005870 | FANCL | 2 | blue |
| ENSBTAG00000005891 | FCRL1 | 2 | blue |
| ENSBTAG00000005897 | FNTB | 2 | blue |
| ENSBTAG00000005917 | PARD6G | 2 | blue |
| ENSBTAG00000005928 | MINPP1 | 2 | blue |
| ENSBTAG00000005955 | RSPH3 | 2 | blue |
| ENSBTAG00000005957 | CSE1L | 2 | blue |
| ENSBTAG00000005994 | SPESP1 | 2 | blue |
| ENSBTAG00000006005 | MPND | 2 | blue |
| ENSBTAG00000006013 | NT5DC3 | 2 | blue |
| ENSBTAG00000006021 | CEP250 | 2 | blue |
| ENSBTAG00000006022 | ATP6V0E2 | 2 | blue |
| ENSBTAG00000006026 | BEX3 | 2 | blue |
| ENSBTAG00000006034 | RNF113A | 2 | blue |
| ENSBTAG00000006050 | BOLA1 | 2 | blue |
| ENSBTAG00000006054 | ACBD4 | 2 | blue |
| ENSBTAG00000006068 | CC2D1A | 2 | blue |
| ENSBTAG00000006072 | LRRC59 | 2 | blue |
| ENSBTAG00000006073 | PODNL1 | 2 | blue |
| ENSBTAG00000006082 | DNASE2 | 2 | blue |
| ENSBTAG00000006086 | MMP28 | 2 | blue |
| ENSBTAG00000006107 | SLF1 | 2 | blue |
| ENSBTAG00000006117 | FLYWCH1 | 2 | blue |
| ENSBTAG00000006130 | CLEC14A | 2 | blue |
| ENSBTAG00000006162 | RAB11FIP5 | 2 | blue |
| ENSBTAG00000006165 | PRDX4 | 2 | blue |
| ENSBTAG00000006187 | MFAP4 | 2 | blue |
| ENSBTAG00000006201 | DTWD1 | 2 | blue |
| ENSBTAG00000006204 | XPNPEP3 | 2 | blue |

| | | | |
|---|---|---|---|
| ENSBTAG00000006213 | NA | 2 | blue |
| ENSBTAG00000006235 | INTS3 | 2 | blue |
| ENSBTAG00000006246 | CBX5 | 2 | blue |
| ENSBTAG00000006260 | KIAA1614 | 2 | blue |
| ENSBTAG00000006272 | NTHL1 | 2 | blue |
| ENSBTAG00000006276 | C1QTNF1 | 2 | blue |
| ENSBTAG00000006296 | VMA21 | 2 | blue |
| ENSBTAG00000006305 | AK1 | 2 | blue |
| ENSBTAG00000006307 | HSD17B11 | 2 | blue |
| ENSBTAG00000006320 | DBT | 2 | blue |
| ENSBTAG00000006339 | LOC785479 | 2 | blue |
| ENSBTAG00000006349 | EXTL1 | 2 | blue |
| ENSBTAG00000006364 | GLMP | 2 | blue |
| ENSBTAG00000006372 | KLHL42 | 2 | blue |
| ENSBTAG00000006377 | MYO1G | 2 | blue |
| ENSBTAG00000006379 | BPHL | 2 | blue |
| ENSBTAG00000006392 | TTC7B | 2 | blue |
| ENSBTAG00000006393 | FECH | 2 | blue |
| ENSBTAG00000006396 | GPI | 2 | blue |
| ENSBTAG00000006398 | TOMM7 | 2 | blue |
| ENSBTAG00000006405 | PALB2 | 2 | blue |
| ENSBTAG00000006410 | DCTN5 | 2 | blue |
| ENSBTAG00000006411 | NA | 2 | blue |
| ENSBTAG00000006443 | PRDM11 | 2 | blue |
| ENSBTAG00000006448 | ERI2 | 2 | blue |
| ENSBTAG00000006449 | REXO5 | 2 | blue |
| ENSBTAG00000006478 | C8H9orf64 | 2 | blue |
| ENSBTAG00000006485 | TREML1 | 2 | blue |
| ENSBTAG00000006489 | MYO5A | 2 | blue |
| ENSBTAG00000006491 | AGL | 2 | blue |
| ENSBTAG00000006492 | PI4K2B | 2 | blue |
| ENSBTAG00000006504 | TCHP | 2 | blue |
| ENSBTAG00000006532 | CDK5RAP2 | 2 | blue |
| ENSBTAG00000006534 | GTPBP8 | 2 | blue |
| ENSBTAG00000006541 | ATP2A1 | 2 | blue |
| ENSBTAG00000006548 | MCCC1 | 2 | blue |
| ENSBTAG00000006570 | SEC22C | 2 | blue |
| ENSBTAG00000006587 | ZNF367 | 2 | blue |
| ENSBTAG00000006590 | NUDT16L1 | 2 | blue |
| ENSBTAG00000006592 | SGSM1 | 2 | blue |
| ENSBTAG00000006607 | CCNG1 | 2 | blue |
| ENSBTAG00000006613 | METTL17 | 2 | blue |
| ENSBTAG00000006618 | HLF | 2 | blue |
| ENSBTAG00000006625 | NDC1 | 2 | blue |
| ENSBTAG00000006626 | TMEM132B | 2 | blue |
| ENSBTAG00000006662 | NA | 2 | blue |
| ENSBTAG00000006667 | EPB41 | 2 | blue |

| | | | |
|---|---|---|---|
| ENSBTAG00000006713 | ZDHHC6 | 2 | blue |
| ENSBTAG00000006716 | PTGS1 | 2 | blue |
| ENSBTAG00000006748 | DMXL1 | 2 | blue |
| ENSBTAG00000006754 | DBP | 2 | blue |
| ENSBTAG00000006795 | GCSH | 2 | blue |
| ENSBTAG00000006805 | ZNF180 | 2 | blue |
| ENSBTAG00000006828 | RAPGEF3 | 2 | blue |
| ENSBTAG00000006838 | AIFM1 | 2 | blue |
| ENSBTAG00000006852 | ACYP2 | 2 | blue |
| ENSBTAG00000006886 | FAM117A | 2 | blue |
| ENSBTAG00000006901 | NIPSNAP3A | 2 | blue |
| ENSBTAG00000006925 | CENPL | 2 | blue |
| ENSBTAG00000006928 | OAT | 2 | blue |
| ENSBTAG00000006943 | EFCAB3 | 2 | blue |
| ENSBTAG00000006945 | CCDC50 | 2 | blue |
| ENSBTAG00000006954 | ZNF432 | 2 | blue |
| ENSBTAG00000006957 | SLC2A8 | 2 | blue |
| ENSBTAG00000006966 | NRM | 2 | blue |
| ENSBTAG00000006969 | TUBB | 2 | blue |
| ENSBTAG00000006978 | HSD17B4 | 2 | blue |
| ENSBTAG00000006985 | TM7SF3 | 2 | blue |
| ENSBTAG00000007000 | ENOSF1 | 2 | blue |
| ENSBTAG00000007001 | SLC26A1 | 2 | blue |
| ENSBTAG00000007002 | IDUA | 2 | blue |
| ENSBTAG00000007008 | MOK | 2 | blue |
| ENSBTAG00000007012 | ZNF3 | 2 | blue |
| ENSBTAG00000007016 | CNPY4 | 2 | blue |
| ENSBTAG00000007020 | ZNF502 | 2 | blue |
| ENSBTAG00000007031 | OXSM | 2 | blue |
| ENSBTAG00000007053 | ZFHX2 | 2 | blue |
| ENSBTAG00000007096 | GAS8 | 2 | blue |
| ENSBTAG00000007097 | THTPA | 2 | blue |
| ENSBTAG00000007099 | AP1G2 | 2 | blue |
| ENSBTAG00000007102 | GTSE1 | 2 | blue |
| ENSBTAG00000007107 | TMEM143 | 2 | blue |
| ENSBTAG00000007112 | HSPB11 | 2 | blue |
| ENSBTAG00000007115 | GSR | 2 | blue |
| ENSBTAG00000007121 | TK1 | 2 | blue |
| ENSBTAG00000007125 | AFMID | 2 | blue |
| ENSBTAG00000007137 | RFC5 | 2 | blue |
| ENSBTAG00000007142 | CPPED1 | 2 | blue |
| ENSBTAG00000007159 | ESR1 | 2 | blue |
| ENSBTAG00000007160 | CDK4 | 2 | blue |
| ENSBTAG00000007170 | SLC38A7 | 2 | blue |
| ENSBTAG00000007186 | ARHGAP39 | 2 | blue |
| ENSBTAG00000007187 | INF2 | 2 | blue |
| ENSBTAG00000007217 | TMEM94 | 2 | blue |

| | | | |
|---|---|---|---|
| ENSBTAG00000007235 | NTNG2 | 2 | blue |
| ENSBTAG00000007238 | NA | 2 | blue |
| ENSBTAG00000007244 | NID1 | 2 | blue |
| ENSBTAG00000007245 | IFT140 | 2 | blue |
| ENSBTAG00000007246 | TMEM204 | 2 | blue |
| ENSBTAG00000007269 | EIF2B1 | 2 | blue |
| ENSBTAG00000007271 | TCTN2 | 2 | blue |
| ENSBTAG00000007275 | TREM2 | 2 | blue |
| ENSBTAG00000007288 | CDK19 | 2 | blue |
| ENSBTAG00000007312 | CD209 | 2 | blue |
| ENSBTAG00000007315 | LARS2 | 2 | blue |
| ENSBTAG00000007331 | PLOD2 | 2 | blue |
| ENSBTAG00000007333 | NA | 2 | blue |
| ENSBTAG00000007335 | TPD52 | 2 | blue |
| ENSBTAG00000007346 | COMMD4 | 2 | blue |
| ENSBTAG00000007362 | XPC | 2 | blue |
| ENSBTAG00000007363 | LSM3 | 2 | blue |
| ENSBTAG00000007364 | PMS2 | 2 | blue |
| ENSBTAG00000007365 | NEIL1 | 2 | blue |
| ENSBTAG00000007366 | HAUS8 | 2 | blue |
| ENSBTAG00000007368 | MAN2C1 | 2 | blue |
| ENSBTAG00000007374 | LHFPL2 | 2 | blue |
| ENSBTAG00000007375 | MIF | 2 | blue |
| ENSBTAG00000007382 | NA | 2 | blue |
| ENSBTAG00000007390 | VAT1 | 2 | blue |
| ENSBTAG00000007393 | RND2 | 2 | blue |
| ENSBTAG00000007395 | ALS2 | 2 | blue |
| ENSBTAG00000007413 | CEP41 | 2 | blue |
| ENSBTAG00000007438 | STT3B | 2 | blue |
| ENSBTAG00000007439 | TENM4 | 2 | blue |
| ENSBTAG00000007443 | NA | 2 | blue |
| ENSBTAG00000007461 | C20H5orf34 | 2 | blue |
| ENSBTAG00000007480 | CIRBP | 2 | blue |
| ENSBTAG00000007484 | ACADS | 2 | blue |
| ENSBTAG00000007501 | NA | 2 | blue |
| ENSBTAG00000007508 | SRBD1 | 2 | blue |
| ENSBTAG00000007510 | PXMP2 | 2 | blue |
| ENSBTAG00000007512 | PLA2G15 | 2 | blue |
| ENSBTAG00000007534 | GGH | 2 | blue |
| ENSBTAG00000007543 | TAF1B | 2 | blue |
| ENSBTAG00000007564 | BBS12 | 2 | blue |
| ENSBTAG00000007567 | TMX3 | 2 | blue |
| ENSBTAG00000007578 | SHTN1 | 2 | blue |
| ENSBTAG00000007595 | BROX | 2 | blue |
| ENSBTAG00000007608 | BMT2 | 2 | blue |
| ENSBTAG00000007622 | CTSD | 2 | blue |
| ENSBTAG00000007630 | RNF170 | 2 | blue |

| | | | |
|---|---|---|---|
| ENSBTAG00000007632 | FAM151B | 2 | blue |
| ENSBTAG00000007635 | PLCL1 | 2 | blue |
| ENSBTAG00000007642 | NA | 2 | blue |
| ENSBTAG00000007668 | AGPAT4 | 2 | blue |
| ENSBTAG00000007693 | SLC45A3 | 2 | blue |
| ENSBTAG00000007700 | PHYH | 2 | blue |
| ENSBTAG00000007739 | REX1BD | 2 | blue |
| ENSBTAG00000007753 | KIFC2 | 2 | blue |
| ENSBTAG00000007766 | CDK5 | 2 | blue |
| ENSBTAG00000007777 | OGG1 | 2 | blue |
| ENSBTAG00000007814 | WWTR1 | 2 | blue |
| ENSBTAG00000007820 | LOC101905254 | 2 | blue |
| ENSBTAG00000007834 | PPP1R16A | 2 | blue |
| ENSBTAG00000007835 | GPT | 2 | blue |
| ENSBTAG00000007838 | MFSD3 | 2 | blue |
| ENSBTAG00000007842 | GCLM | 2 | blue |
| ENSBTAG00000007844 | CETN2 | 2 | blue |
| ENSBTAG00000007847 | EED | 2 | blue |
| ENSBTAG00000007865 | GNPDA1 | 2 | blue |
| ENSBTAG00000007870 | FKBP14 | 2 | blue |
| ENSBTAG00000007872 | PSIP1 | 2 | blue |
| ENSBTAG00000007893 | SCPEP1 | 2 | blue |
| ENSBTAG00000007916 | FOXJ1 | 2 | blue |
| ENSBTAG00000007931 | SLC38A6 | 2 | blue |
| ENSBTAG00000007937 | PRIM2 | 2 | blue |
| ENSBTAG00000007961 | TADA3 | 2 | blue |
| ENSBTAG00000007979 | TNPO1 | 2 | blue |
| ENSBTAG00000007986 | NSD2 | 2 | blue |
| ENSBTAG00000007992 | NA | 2 | blue |
| ENSBTAG00000007993 | CORO1C | 2 | blue |
| ENSBTAG00000008001 | NUCKS1 | 2 | blue |
| ENSBTAG00000008006 | RASGRP3 | 2 | blue |
| ENSBTAG00000008016 | STX17 | 2 | blue |
| ENSBTAG00000008028 | CHN1 | 2 | blue |
| ENSBTAG00000008032 | ACTR3B | 2 | blue |
| ENSBTAG00000008047 | ZDHHC15 | 2 | blue |
| ENSBTAG00000008057 | PARVG | 2 | blue |
| ENSBTAG00000008061 | RILPL1 | 2 | blue |
| ENSBTAG00000008065 | CDPF1 | 2 | blue |
| ENSBTAG00000008066 | LOC101904413 | 2 | blue |
| ENSBTAG00000008077 | SLC37A4 | 2 | blue |
| ENSBTAG00000008084 | ZNF382 | 2 | blue |
| ENSBTAG00000008093 | NA | 2 | blue |
| ENSBTAG00000008116 | POLDIP2 | 2 | blue |
| ENSBTAG00000008126 | NPHP4 | 2 | blue |
| ENSBTAG00000008167 | TTC3 | 2 | blue |
| ENSBTAG00000008168 | STARD6 | 2 | blue |

| | | | |
|---|---|---|---|
| ENSBTAG00000008169 | C24H18orf54 | 2 | blue |
| ENSBTAG00000008170 | POLA1 | 2 | blue |
| ENSBTAG00000008175 | ZNF713 | 2 | blue |
| ENSBTAG00000008180 | SPDL1 | 2 | blue |
| ENSBTAG00000008190 | SUMF2 | 2 | blue |
| ENSBTAG00000008198 | RGL3 | 2 | blue |
| ENSBTAG00000008201 | ODAD3 | 2 | blue |
| ENSBTAG00000008203 | NA | 2 | blue |
| ENSBTAG00000008224 | PAIP2 | 2 | blue |
| ENSBTAG00000008243 | LRRC40 | 2 | blue |
| ENSBTAG00000008274 | NA | 2 | blue |
| ENSBTAG00000008278 | TMEM187 | 2 | blue |
| ENSBTAG00000008288 | ANKRD26 | 2 | blue |
| ENSBTAG00000008289 | ZBTB4 | 2 | blue |
| ENSBTAG00000008291 | PROCR | 2 | blue |
| ENSBTAG00000008302 | RINT1 | 2 | blue |
| ENSBTAG00000008314 | SDHB | 2 | blue |
| ENSBTAG00000008340 | ATOX1 | 2 | blue |
| ENSBTAG00000008347 | MRPL48 | 2 | blue |
| ENSBTAG00000008349 | ZNF311 | 2 | blue |
| ENSBTAG00000008361 | PON2 | 2 | blue |
| ENSBTAG00000008365 | NA | 2 | blue |
| ENSBTAG00000008367 | NA | 2 | blue |
| ENSBTAG00000008371 | FBXL20 | 2 | blue |
| ENSBTAG00000008373 | ITGA10 | 2 | blue |
| ENSBTAG00000008389 | HTRA1 | 2 | blue |
| ENSBTAG00000008390 | DNAJC10 | 2 | blue |
| ENSBTAG00000008397 | ZNF555 | 2 | blue |
| ENSBTAG00000008416 | ABHD5 | 2 | blue |
| ENSBTAG00000008431 | MOGAT1 | 2 | blue |
| ENSBTAG00000008434 | GCAT | 2 | blue |
| ENSBTAG00000008436 | CDC25B | 2 | blue |
| ENSBTAG00000008438 | ARFIP1 | 2 | blue |
| ENSBTAG00000008448 | MEIS1 | 2 | blue |
| ENSBTAG00000008464 | ABCB9 | 2 | blue |
| ENSBTAG00000008465 | ACCS | 2 | blue |
| ENSBTAG00000008470 | MPHOSPH9 | 2 | blue |
| ENSBTAG00000008483 | CALCRL | 2 | blue |
| ENSBTAG00000008487 | VPS37D | 2 | blue |
| ENSBTAG00000008490 | CRYZL1 | 2 | blue |
| ENSBTAG00000008492 | ZMYM3 | 2 | blue |
| ENSBTAG00000008513 | RHEX | 2 | blue |
| ENSBTAG00000008518 | MED25 | 2 | blue |
| ENSBTAG00000008528 | SLC25A1 | 2 | blue |
| ENSBTAG00000008530 | SLC36A4 | 2 | blue |
| ENSBTAG00000008537 | FAM219A | 2 | blue |
| ENSBTAG00000008543 | GTF2H5 | 2 | blue |

| | | | |
|---|---|---|---|
| ENSBTAG00000008548 | FAM98A | 2 | blue |
| ENSBTAG00000008551 | TUSC1 | 2 | blue |
| ENSBTAG00000008562 | POGLUT1 | 2 | blue |
| ENSBTAG00000008571 | CUX2 | 2 | blue |
| ENSBTAG00000008577 | GRSF1 | 2 | blue |
| ENSBTAG00000008578 | LSM4 | 2 | blue |
| ENSBTAG00000008583 | PALD1 | 2 | blue |
| ENSBTAG00000008584 | CLN8 | 2 | blue |
| ENSBTAG00000008590 | SPAG7 | 2 | blue |
| ENSBTAG00000008592 | FCGR1A | 2 | blue |
| ENSBTAG00000008611 | IGFBP4 | 2 | blue |
| ENSBTAG00000008613 | TCP11L1 | 2 | blue |
| ENSBTAG00000008633 | SLX1A | 2 | blue |
| ENSBTAG00000008674 | FAM136A | 2 | blue |
| ENSBTAG00000008709 | POGLUT3 | 2 | blue |
| ENSBTAG00000008711 | BOK | 2 | blue |
| ENSBTAG00000008721 | MANSC1 | 2 | blue |
| ENSBTAG00000008726 | NCS1 | 2 | blue |
| ENSBTAG00000008733 | MAGED1 | 2 | blue |
| ENSBTAG00000008734 | PM20D1 | 2 | blue |
| ENSBTAG00000008735 | VASH1 | 2 | blue |
| ENSBTAG00000008744 | PDK2 | 2 | blue |
| ENSBTAG00000008747 | DCXR | 2 | blue |
| ENSBTAG00000008759 | CDC23 | 2 | blue |
| ENSBTAG00000008765 | PRADC1 | 2 | blue |
| ENSBTAG00000008766 | SLC9A1 | 2 | blue |
| ENSBTAG00000008787 | FAM171A1 | 2 | blue |
| ENSBTAG00000008793 | RNASE1 | 2 | blue |
| ENSBTAG00000008804 | SAAL1 | 2 | blue |
| ENSBTAG00000008826 | RBBP9 | 2 | blue |
| ENSBTAG00000008836 | NA | 2 | blue |
| ENSBTAG00000008844 | EXTL3 | 2 | blue |
| ENSBTAG00000008852 | SIGLEC10 | 2 | blue |
| ENSBTAG00000008860 | RIPOR1 | 2 | blue |
| ENSBTAG00000008862 | GOLGB1 | 2 | blue |
| ENSBTAG00000008884 | MAPRE3 | 2 | blue |
| ENSBTAG00000008893 | ANO10 | 2 | blue |
| ENSBTAG00000008902 | ZNF345 | 2 | blue |
| ENSBTAG00000008931 | CREG1 | 2 | blue |
| ENSBTAG00000008948 | P2RX7 | 2 | blue |
| ENSBTAG00000009002 | LOC509941 | 2 | blue |
| ENSBTAG00000009023 | TMTC3 | 2 | blue |
| ENSBTAG00000009029 | LOC507443 | 2 | blue |
| ENSBTAG00000009037 | GALNT12 | 2 | blue |
| ENSBTAG00000009049 | LOC507581 | 2 | blue |
| ENSBTAG00000009051 | MMP19 | 2 | blue |
| ENSBTAG00000009072 | ZNF572 | 2 | blue |

| | | | |
|---|---|---|---|
| ENSBTAG00000009097 | FANCI | 2 | blue |
| ENSBTAG00000009107 | CBX8 | 2 | blue |
| ENSBTAG00000009111 | NA | 2 | blue |
| ENSBTAG00000009129 | POLI | 2 | blue |
| ENSBTAG00000009137 | NKG7 | 2 | blue |
| ENSBTAG00000009165 | LPIN3 | 2 | blue |
| ENSBTAG00000009174 | HSD17B14 | 2 | blue |
| ENSBTAG00000009187 | DNAJC28 | 2 | blue |
| ENSBTAG00000009208 | TCTN1 | 2 | blue |
| ENSBTAG00000009245 | PPP2CB | 2 | blue |
| ENSBTAG00000009246 | DPY30 | 2 | blue |
| ENSBTAG00000009263 | MFSD1 | 2 | blue |
| ENSBTAG00000009278 | MSL3 | 2 | blue |
| ENSBTAG00000009290 | NA | 2 | blue |
| ENSBTAG00000009297 | PANX2 | 2 | blue |
| ENSBTAG00000009298 | XYLT2 | 2 | blue |
| ENSBTAG00000009334 | NDUFA5 | 2 | blue |
| ENSBTAG00000009336 | LXN | 2 | blue |
| ENSBTAG00000009358 | MTSS2 | 2 | blue |
| ENSBTAG00000009363 | GINS3 | 2 | blue |
| ENSBTAG00000009366 | ARF5 | 2 | blue |
| ENSBTAG00000009374 | PARP2 | 2 | blue |
| ENSBTAG00000009392 | NA | 2 | blue |
| ENSBTAG00000009412 | SIAE | 2 | blue |
| ENSBTAG00000009417 | ZFAND5 | 2 | blue |
| ENSBTAG00000009461 | STYXL1 | 2 | blue |
| ENSBTAG00000009477 | PIR | 2 | blue |
| ENSBTAG00000009486 | TMEM179B | 2 | blue |
| ENSBTAG00000009519 | TRIM23 | 2 | blue |
| ENSBTAG00000009541 | SUCLG2 | 2 | blue |
| ENSBTAG00000009545 | PPEF1 | 2 | blue |
| ENSBTAG00000009548 | MAD2L1BP | 2 | blue |
| ENSBTAG00000009569 | DOCK6 | 2 | blue |
| ENSBTAG00000009575 | TBC1D32 | 2 | blue |
| ENSBTAG00000009579 | CEP85 | 2 | blue |
| ENSBTAG00000009581 | UBXN11 | 2 | blue |
| ENSBTAG00000009602 | TMEM120B | 2 | blue |
| ENSBTAG00000009639 | SMPD3 | 2 | blue |
| ENSBTAG00000009641 | MTHFD1 | 2 | blue |
| ENSBTAG00000009646 | ALDH7A1 | 2 | blue |
| ENSBTAG00000009649 | DRC1 | 2 | blue |
| ENSBTAG00000009657 | MTMR1 | 2 | blue |
| ENSBTAG00000009661 | RNASEH2A | 2 | blue |
| ENSBTAG00000009683 | PSMA6 | 2 | blue |
| ENSBTAG00000009685 | ERI1 | 2 | blue |
| ENSBTAG00000009698 | ABCD3 | 2 | blue |
| ENSBTAG00000009703 | MYH7 | 2 | blue |

| | | | |
|---|---|---|---|
| ENSBTAG00000009708 | SMYD4 | 2 | blue |
| ENSBTAG00000009711 | RPA1 | 2 | blue |
| ENSBTAG00000009719 | CALHM2 | 2 | blue |
| ENSBTAG00000009734 | XPA | 2 | blue |
| ENSBTAG00000009738 | MGAT4B | 2 | blue |
| ENSBTAG00000009746 | MPHOSPH6 | 2 | blue |
| ENSBTAG00000009762 | BEAN1 | 2 | blue |
| ENSBTAG00000009765 | MTMR14 | 2 | blue |
| ENSBTAG00000009777 | NUP35 | 2 | blue |
| ENSBTAG00000009786 | SCIN | 2 | blue |
| ENSBTAG00000009806 | INVS | 2 | blue |
| ENSBTAG00000009812 | CXCL5 | 2 | blue |
| ENSBTAG00000009826 | GPD1L | 2 | blue |
| ENSBTAG00000009855 | FAN1 | 2 | blue |
| ENSBTAG00000009861 | FRS3 | 2 | blue |
| ENSBTAG00000009867 | GARIN1B | 2 | blue |
| ENSBTAG00000009872 | SPHK2 | 2 | blue |
| ENSBTAG00000009879 | PCGF6 | 2 | blue |
| ENSBTAG00000009886 | KDELR3 | 2 | blue |
| ENSBTAG00000009889 | ZNF181 | 2 | blue |
| ENSBTAG00000009895 | ZNF599 | 2 | blue |
| ENSBTAG00000009902 | AKR1B1 | 2 | blue |
| ENSBTAG00000009904 | SPIC | 2 | blue |
| ENSBTAG00000009941 | MBIP | 2 | blue |
| ENSBTAG00000009956 | CENPM | 2 | blue |
| ENSBTAG00000009965 | ECI1 | 2 | blue |
| ENSBTAG00000009966 | XRCC3 | 2 | blue |
| ENSBTAG00000009975 | PBX4 | 2 | blue |
| ENSBTAG00000009984 | SGPL1 | 2 | blue |
| ENSBTAG00000009987 | ITGB3 | 2 | blue |
| ENSBTAG00000010015 | NMRAL1 | 2 | blue |
| ENSBTAG00000010030 | NA | 2 | blue |
| ENSBTAG00000010048 | SPC25 | 2 | blue |
| ENSBTAG00000010059 | C8H9orf72 | 2 | blue |
| ENSBTAG00000010060 | SLC43A3 | 2 | blue |
| ENSBTAG00000010083 | HADHB | 2 | blue |
| ENSBTAG00000010096 | SLC5A2 | 2 | blue |
| ENSBTAG00000010118 | HAT1 | 2 | blue |
| ENSBTAG00000010119 | ALDH1A2 | 2 | blue |
| ENSBTAG00000010120 | HERC3 | 2 | blue |
| ENSBTAG00000010124 | ATG4C | 2 | blue |
| ENSBTAG00000010135 | PEDS1 | 2 | blue |
| ENSBTAG00000010144 | MTERF2 | 2 | blue |
| ENSBTAG00000010195 | PKN3 | 2 | blue |
| ENSBTAG00000010196 | NUP43 | 2 | blue |
| ENSBTAG00000010208 | ZER1 | 2 | blue |
| ENSBTAG00000010215 | CREB3L3 | 2 | blue |

| | | | |
|---|---|---|---|
| ENSBTAG00000010234 | DHRS1 | 2 | blue |
| ENSBTAG00000010247 | FKTN | 2 | blue |
| ENSBTAG00000010252 | ZNF397 | 2 | blue |
| ENSBTAG00000010256 | TMEM170B | 2 | blue |
| ENSBTAG00000010278 | STPG1 | 2 | blue |
| ENSBTAG00000010286 | NPAT | 2 | blue |
| ENSBTAG00000010318 | FDX2 | 2 | blue |
| ENSBTAG00000010326 | LOC520638 | 2 | blue |
| ENSBTAG00000010338 | FBXO36 | 2 | blue |
| ENSBTAG00000010366 | HCRTR1 | 2 | blue |
| ENSBTAG00000010367 | TTC13 | 2 | blue |
| ENSBTAG00000010370 | CLBA1 | 2 | blue |
| ENSBTAG00000010379 | CRTC1 | 2 | blue |
| ENSBTAG00000010388 | MGAT4A | 2 | blue |
| ENSBTAG00000010403 | FAAP24 | 2 | blue |
| ENSBTAG00000010426 | XRCC5 | 2 | blue |
| ENSBTAG00000010458 | LOC613822 | 2 | blue |
| ENSBTAG00000010460 | MARK1 | 2 | blue |
| ENSBTAG00000010470 | UQCC3 | 2 | blue |
| ENSBTAG00000010484 | LRRN4CL | 2 | blue |
| ENSBTAG00000010496 | STAG3 | 2 | blue |
| ENSBTAG00000010498 | SLC40A1 | 2 | blue |
| ENSBTAG00000010508 | BLVRB | 2 | blue |
| ENSBTAG00000010526 | PLPP1 | 2 | blue |
| ENSBTAG00000010559 | DHRS7B | 2 | blue |
| ENSBTAG00000010568 | ZBED5 | 2 | blue |
| ENSBTAG00000010577 | PAICS | 2 | blue |
| ENSBTAG00000010597 | GGCT | 2 | blue |
| ENSBTAG00000010612 | TMED4 | 2 | blue |
| ENSBTAG00000010641 | APH1B | 2 | blue |
| ENSBTAG00000010653 | MIGA2 | 2 | blue |
| ENSBTAG00000010658 | PFKL | 2 | blue |
| ENSBTAG00000010672 | PCCA | 2 | blue |
| ENSBTAG00000010673 | EFNA4 | 2 | blue |
| ENSBTAG00000010711 | LOC101906280 | 2 | blue |
| ENSBTAG00000010715 | MRPL37 | 2 | blue |
| ENSBTAG00000010718 | RALGPS2 | 2 | blue |
| ENSBTAG00000010720 | MIB2 | 2 | blue |
| ENSBTAG00000010736 | BBS5 | 2 | blue |
| ENSBTAG00000010741 | KLHL41 | 2 | blue |
| ENSBTAG00000010742 | PKD2L1 | 2 | blue |
| ENSBTAG00000010743 | FASTKD1 | 2 | blue |
| ENSBTAG00000010745 | THRA | 2 | blue |
| ENSBTAG00000010760 | REV3L | 2 | blue |
| ENSBTAG00000010772 | TPGS1 | 2 | blue |
| ENSBTAG00000010778 | NDUFAF1 | 2 | blue |
| ENSBTAG00000010787 | RFC3 | 2 | blue |

| | | | |
|---|---|---|---|
| ENSBTAG00000010795 | ATG14 | 2 | blue |
| ENSBTAG00000010801 | CMBL | 2 | blue |
| ENSBTAG00000010805 | POMT1 | 2 | blue |
| ENSBTAG00000010821 | ACER2 | 2 | blue |
| ENSBTAG00000010828 | LOC617313 | 2 | blue |
| ENSBTAG00000010835 | CRELD1 | 2 | blue |
| ENSBTAG00000010841 | FMO5 | 2 | blue |
| ENSBTAG00000010846 | CNNM3 | 2 | blue |
| ENSBTAG00000010849 | ANKRD23 | 2 | blue |
| ENSBTAG00000010884 | NKAP | 2 | blue |
| ENSBTAG00000010899 | TIMP2 | 2 | blue |
| ENSBTAG00000010906 | RPS6KA5 | 2 | blue |
| ENSBTAG00000010932 | DNAJC12 | 2 | blue |
| ENSBTAG00000010943 | SLC22A23 | 2 | blue |
| ENSBTAG00000010948 | CLIC2 | 2 | blue |
| ENSBTAG00000010956 | SCARB2 | 2 | blue |
| ENSBTAG00000010957 | LHPP | 2 | blue |
| ENSBTAG00000010964 | STARD5 | 2 | blue |
| ENSBTAG00000010971 | LIMS1 | 2 | blue |
| ENSBTAG00000010976 | KANK1 | 2 | blue |
| ENSBTAG00000011052 | NA | 2 | blue |
| ENSBTAG00000011063 | CTSO | 2 | blue |
| ENSBTAG00000011072 | ADK | 2 | blue |
| ENSBTAG00000011082 | IGF1 | 2 | blue |
| ENSBTAG00000011102 | TPCN1 | 2 | blue |
| ENSBTAG00000011104 | RTN4 | 2 | blue |
| ENSBTAG00000011136 | NR2F6 | 2 | blue |
| ENSBTAG00000011137 | MORN4 | 2 | blue |
| ENSBTAG00000011140 | FAM162A | 2 | blue |
| ENSBTAG00000011172 | ALMS1 | 2 | blue |
| ENSBTAG00000011193 | C1QC | 2 | blue |
| ENSBTAG00000011195 | NA | 2 | blue |
| ENSBTAG00000011205 | PPP1R42 | 2 | blue |
| ENSBTAG00000011224 | CITED2 | 2 | blue |
| ENSBTAG00000011228 | FASTK | 2 | blue |
| ENSBTAG00000011252 | RAD50 | 2 | blue |
| ENSBTAG00000011262 | NA | 2 | blue |
| ENSBTAG00000011278 | XYLB | 2 | blue |
| ENSBTAG00000011307 | DNM1 | 2 | blue |
| ENSBTAG00000011313 | CASP8AP2 | 2 | blue |
| ENSBTAG00000011324 | EMILIN1 | 2 | blue |
| ENSBTAG00000011334 | NADK2 | 2 | blue |
| ENSBTAG00000011340 | NSL1 | 2 | blue |
| ENSBTAG00000011344 | TMEM110 | 2 | blue |
| ENSBTAG00000011349 | CDH24 | 2 | blue |
| ENSBTAG00000011392 | MYBPC1 | 2 | blue |
| ENSBTAG00000011424 | TPM2 | 2 | blue |

| | | | |
|---|---|---|---|
| ENSBTAG00000011444 | HINT2 | 2 | blue |
| ENSBTAG00000011445 | TMEM8B | 2 | blue |
| ENSBTAG00000011447 | FAM171A2 | 2 | blue |
| ENSBTAG00000011455 | POLA2 | 2 | blue |
| ENSBTAG00000011471 | CARS2 | 2 | blue |
| ENSBTAG00000011473 | MYL9 | 2 | blue |
| ENSBTAG00000011494 | PYGL | 2 | blue |
| ENSBTAG00000011495 | DIP2A | 2 | blue |
| ENSBTAG00000011512 | NA | 2 | blue |
| ENSBTAG00000011528 | SMIM11 | 2 | blue |
| ENSBTAG00000011540 | SPG21 | 2 | blue |
| ENSBTAG00000011541 | RBL1 | 2 | blue |
| ENSBTAG00000011551 | CPNE9 | 2 | blue |
| ENSBTAG00000011553 | FAHD2A | 2 | blue |
| ENSBTAG00000011580 | DAG1 | 2 | blue |
| ENSBTAG00000011583 | APEH | 2 | blue |
| ENSBTAG00000011585 | MST1 | 2 | blue |
| ENSBTAG00000011586 | HABP4 | 2 | blue |
| ENSBTAG00000011589 | CDC14B | 2 | blue |
| ENSBTAG00000011601 | ST3GAL5 | 2 | blue |
| ENSBTAG00000011616 | MPO | 2 | blue |
| ENSBTAG00000011620 | PIGU | 2 | blue |
| ENSBTAG00000011636 | NA | 2 | blue |
| ENSBTAG00000011640 | PLPP3 | 2 | blue |
| ENSBTAG00000011646 | ZNF512B | 2 | blue |
| ENSBTAG00000011647 | SLC25A15 | 2 | blue |
| ENSBTAG00000011648 | TMLHE | 2 | blue |
| ENSBTAG00000011713 | MYO15B | 2 | blue |
| ENSBTAG00000011715 | RECQL5 | 2 | blue |
| ENSBTAG00000011726 | CRYL1 | 2 | blue |
| ENSBTAG00000011727 | ETAA1 | 2 | blue |
| ENSBTAG00000011738 | TFR2 | 2 | blue |
| ENSBTAG00000011758 | NA | 2 | blue |
| ENSBTAG00000011788 | CXHXorf38 | 2 | blue |
| ENSBTAG00000011793 | FDX1 | 2 | blue |
| ENSBTAG00000011795 | TXNRD3 | 2 | blue |
| ENSBTAG00000011805 | PLIN3 | 2 | blue |
| ENSBTAG00000011809 | NA | 2 | blue |
| ENSBTAG00000011819 | NA | 2 | blue |
| ENSBTAG00000011823 | CLSTN1 | 2 | blue |
| ENSBTAG00000011826 | PEX26 | 2 | blue |
| ENSBTAG00000011838 | PCED1B | 2 | blue |
| ENSBTAG00000011866 | PCBD1 | 2 | blue |
| ENSBTAG00000011879 | B4GALT2 | 2 | blue |
| ENSBTAG00000011885 | NNT | 2 | blue |
| ENSBTAG00000011892 | SRGAP1 | 2 | blue |
| ENSBTAG00000011893 | HDHD2 | 2 | blue |

| | | | |
|---|---|---|---|
| ENSBTAG00000011911 | NA | 2 | blue |
| ENSBTAG00000011917 | GPAM | 2 | blue |
| ENSBTAG00000011926 | NA | 2 | blue |
| ENSBTAG00000011928 | NA | 2 | blue |
| ENSBTAG00000011931 | CD63 | 2 | blue |
| ENSBTAG00000011945 | NA | 2 | blue |
| ENSBTAG00000011954 | SEC11C | 2 | blue |
| ENSBTAG00000011961 | PPBP | 2 | blue |
| ENSBTAG00000011966 | LAMC1 | 2 | blue |
| ENSBTAG00000011973 | NA | 2 | blue |
| ENSBTAG00000012005 | IFT43 | 2 | blue |
| ENSBTAG00000012012 | CYB5A | 2 | blue |
| ENSBTAG00000012058 | GMDS | 2 | blue |
| ENSBTAG00000012062 | PRDX2 | 2 | blue |
| ENSBTAG00000012068 | BRIP1 | 2 | blue |
| ENSBTAG00000012069 | CCDC167 | 2 | blue |
| ENSBTAG00000012096 | BCKDHB | 2 | blue |
| ENSBTAG00000012121 | NOXA1 | 2 | blue |
| ENSBTAG00000012126 | PNPLA7 | 2 | blue |
| ENSBTAG00000012142 | RAD18 | 2 | blue |
| ENSBTAG00000012164 | CP | 2 | blue |
| ENSBTAG00000012168 | RANBP6 | 2 | blue |
| ENSBTAG00000012176 | QPCTL | 2 | blue |
| ENSBTAG00000012225 | KPNA2 | 2 | blue |
| ENSBTAG00000012241 | POLD2 | 2 | blue |
| ENSBTAG00000012244 | TUBA1B | 2 | blue |
| ENSBTAG00000012263 | ASAP3 | 2 | blue |
| ENSBTAG00000012265 | VWF | 2 | blue |
| ENSBTAG00000012272 | TMEM19 | 2 | blue |
| ENSBTAG00000012274 | CIZ1 | 2 | blue |
| ENSBTAG00000012275 | ENDOU | 2 | blue |
| ENSBTAG00000012278 | MTMR12 | 2 | blue |
| ENSBTAG00000012338 | FAH | 2 | blue |
| ENSBTAG00000012348 | LRRC71 | 2 | blue |
| ENSBTAG00000012365 | ANP32A | 2 | blue |
| ENSBTAG00000012372 | TMEM53 | 2 | blue |
| ENSBTAG00000012375 | KLHL9 | 2 | blue |
| ENSBTAG00000012377 | ECHDC3 | 2 | blue |
| ENSBTAG00000012382 | KCTD9 | 2 | blue |
| ENSBTAG00000012433 | DENND1B | 2 | blue |
| ENSBTAG00000012442 | CTSB | 2 | blue |
| ENSBTAG00000012446 | MTHFSD | 2 | blue |
| ENSBTAG00000012463 | ZMAT3 | 2 | blue |
| ENSBTAG00000012499 | RPGRIP1L | 2 | blue |
| ENSBTAG00000012509 | DYRK1B | 2 | blue |
| ENSBTAG00000012512 | GPR137 | 2 | blue |
| ENSBTAG00000012522 | ZNF283 | 2 | blue |

| | | | |
|---|---|---|---|
| ENSBTAG00000012543 | EDA | 2 | blue |
| ENSBTAG00000012545 | GOLM2 | 2 | blue |
| ENSBTAG00000012557 | OSGEPL1 | 2 | blue |
| ENSBTAG00000012561 | ORMDL1 | 2 | blue |
| ENSBTAG00000012566 | MAP10 | 2 | blue |
| ENSBTAG00000012621 | RTN4RL2 | 2 | blue |
| ENSBTAG00000012629 | ZNF362 | 2 | blue |
| ENSBTAG00000012667 | CAMK2G | 2 | blue |
| ENSBTAG00000012676 | KRT79 | 2 | blue |
| ENSBTAG00000012684 | CCL19 | 2 | blue |
| ENSBTAG00000012693 | PKD1L2 | 2 | blue |
| ENSBTAG00000012703 | GLO1 | 2 | blue |
| ENSBTAG00000012708 | SERPINI1 | 2 | blue |
| ENSBTAG00000012721 | HOGA1 | 2 | blue |
| ENSBTAG00000012723 | HAUS4 | 2 | blue |
| ENSBTAG00000012725 | C10H14orf93 | 2 | blue |
| ENSBTAG00000012726 | PSMB5 | 2 | blue |
| ENSBTAG00000012738 | ZNF827 | 2 | blue |
| ENSBTAG00000012749 | CUL7 | 2 | blue |
| ENSBTAG00000012784 | RACGAP1 | 2 | blue |
| ENSBTAG00000012800 | MMAA | 2 | blue |
| ENSBTAG00000012804 | REEP5 | 2 | blue |
| ENSBTAG00000012844 | LRSAM1 | 2 | blue |
| ENSBTAG00000012852 | DICER1 | 2 | blue |
| ENSBTAG00000012854 | GSDMB | 2 | blue |
| ENSBTAG00000012885 | ACAT1 | 2 | blue |
| ENSBTAG00000012898 | RPS27L | 2 | blue |
| ENSBTAG00000012915 | ZNF404 | 2 | blue |
| ENSBTAG00000012920 | TMEM70 | 2 | blue |
| ENSBTAG00000012927 | ALDOA | 2 | blue |
| ENSBTAG00000012929 | CEP85L | 2 | blue |
| ENSBTAG00000012936 | THYN1 | 2 | blue |
| ENSBTAG00000012937 | ACAD8 | 2 | blue |
| ENSBTAG00000012941 | IFT46 | 2 | blue |
| ENSBTAG00000012954 | ERAS | 2 | blue |
| ENSBTAG00000012972 | CYP2U1 | 2 | blue |
| ENSBTAG00000012980 | RRAGB | 2 | blue |
| ENSBTAG00000012981 | HEXA | 2 | blue |
| ENSBTAG00000012985 | FCSK | 2 | blue |
| ENSBTAG00000012995 | MCUB | 2 | blue |
| ENSBTAG00000013001 | LOC784914 | 2 | blue |
| ENSBTAG00000013007 | FAM210B | 2 | blue |
| ENSBTAG00000013013 | GMPS | 2 | blue |
| ENSBTAG00000013029 | ARNTL | 2 | blue |
| ENSBTAG00000013031 | TMEM62 | 2 | blue |
| ENSBTAG00000013057 | CBLB | 2 | blue |
| ENSBTAG00000013096 | UNC119 | 2 | blue |

| | | | |
|---|---|---|---|
| ENSBTAG00000013098 | PIGS | 2 | blue |
| ENSBTAG00000013110 | FARP2 | 2 | blue |
| ENSBTAG00000013111 | RRM1 | 2 | blue |
| ENSBTAG00000013114 | ZMYND8 | 2 | blue |
| ENSBTAG00000013118 | CD86 | 2 | blue |
| ENSBTAG00000013132 | MTX3 | 2 | blue |
| ENSBTAG00000013136 | EFCAB11 | 2 | blue |
| ENSBTAG00000013138 | TDP1 | 2 | blue |
| ENSBTAG00000013145 | DDHD2 | 2 | blue |
| ENSBTAG00000013152 | NIPSNAP1 | 2 | blue |
| ENSBTAG00000013168 | RPA3 | 2 | blue |
| ENSBTAG00000013177 | APEX2 | 2 | blue |
| ENSBTAG00000013180 | POT1 | 2 | blue |
| ENSBTAG00000013192 | CEP19 | 2 | blue |
| ENSBTAG00000013197 | PSEN2 | 2 | blue |
| ENSBTAG00000013198 | NA | 2 | blue |
| ENSBTAG00000013208 | SLC25A4 | 2 | blue |
| ENSBTAG00000013221 | RTTN | 2 | blue |
| ENSBTAG00000013224 | RMND1 | 2 | blue |
| ENSBTAG00000013235 | TINAGL1 | 2 | blue |
| ENSBTAG00000013244 | HDAC6 | 2 | blue |
| ENSBTAG00000013245 | ITPR3 | 2 | blue |
| ENSBTAG00000013254 | XPO1 | 2 | blue |
| ENSBTAG00000013288 | SUGP2 | 2 | blue |
| ENSBTAG00000013319 | SMCO1 | 2 | blue |
| ENSBTAG00000013322 | POMGNT1 | 2 | blue |
| ENSBTAG00000013326 | ANAPC10 | 2 | blue |
| ENSBTAG00000013343 | FTL | 2 | blue |
| ENSBTAG00000013367 | PPT1 | 2 | blue |
| ENSBTAG00000013369 | COL14A1 | 2 | blue |
| ENSBTAG00000013391 | ANKH | 2 | blue |
| ENSBTAG00000013401 | ARHGEF40 | 2 | blue |
| ENSBTAG00000013403 | AGBL5 | 2 | blue |
| ENSBTAG00000013405 | CIBAR1 | 2 | blue |
| ENSBTAG00000013413 | CCDC51 | 2 | blue |
| ENSBTAG00000013414 | BLVRA | 2 | blue |
| ENSBTAG00000013419 | HTATIP2 | 2 | blue |
| ENSBTAG00000013420 | FSD1L | 2 | blue |
| ENSBTAG00000013464 | CEP97 | 2 | blue |
| ENSBTAG00000013485 | ZFYVE19 | 2 | blue |
| ENSBTAG00000013486 | KICS2 | 2 | blue |
| ENSBTAG00000013489 | CYP27A1 | 2 | blue |
| ENSBTAG00000013496 | CPM | 2 | blue |
| ENSBTAG00000013505 | NA | 2 | blue |
| ENSBTAG00000013515 | INTS13 | 2 | blue |
| ENSBTAG00000013523 | OSBPL7 | 2 | blue |
| ENSBTAG00000013527 | PGD | 2 | blue |

| | | | |
|---|---|---|---|
| ENSBTAG00000013530 | DDAH2 | 2 | blue |
| ENSBTAG00000013531 | CENPS | 2 | blue |
| ENSBTAG00000013535 | DFFA | 2 | blue |
| ENSBTAG00000013550 | PRKCG | 2 | blue |
| ENSBTAG00000013568 | UEVLD | 2 | blue |
| ENSBTAG00000013592 | ZNF248 | 2 | blue |
| ENSBTAG00000013611 | SCLT1 | 2 | blue |
| ENSBTAG00000013612 | MBLAC2 | 2 | blue |
| ENSBTAG00000013620 | UCHL5 | 2 | blue |
| ENSBTAG00000013629 | GET1 | 2 | blue |
| ENSBTAG00000013678 | OSGIN2 | 2 | blue |
| ENSBTAG00000013693 | NA | 2 | blue |
| ENSBTAG00000013698 | ODAD4 | 2 | blue |
| ENSBTAG00000013717 | PRAP1 | 2 | blue |
| ENSBTAG00000013721 | PSMD10 | 2 | blue |
| ENSBTAG00000013724 | ATG4A | 2 | blue |
| ENSBTAG00000013726 | RNPEP | 2 | blue |
| ENSBTAG00000013755 | ITGB5 | 2 | blue |
| ENSBTAG00000013769 | NECTIN3 | 2 | blue |
| ENSBTAG00000013773 | PKP4 | 2 | blue |
| ENSBTAG00000013774 | CCDC9B | 2 | blue |
| ENSBTAG00000013782 | DNAJB2 | 2 | blue |
| ENSBTAG00000013813 | KLHL17 | 2 | blue |
| ENSBTAG00000013822 | NA | 2 | blue |
| ENSBTAG00000013836 | LOC100297513 | 2 | blue |
| ENSBTAG00000013837 | TUBE1 | 2 | blue |
| ENSBTAG00000013842 | ACTR6 | 2 | blue |
| ENSBTAG00000013856 | SSBP2 | 2 | blue |
| ENSBTAG00000013879 | NA | 2 | blue |
| ENSBTAG00000013901 | PTDSS1 | 2 | blue |
| ENSBTAG00000013905 | SMC3 | 2 | blue |
| ENSBTAG00000013910 | POLR3GL | 2 | blue |
| ENSBTAG00000013912 | TXNRD1 | 2 | blue |
| ENSBTAG00000013915 | DSN1 | 2 | blue |
| ENSBTAG00000013920 | NA | 2 | blue |
| ENSBTAG00000013922 | MOSPD1 | 2 | blue |
| ENSBTAG00000013926 | FCGRT | 2 | blue |
| ENSBTAG00000013927 | GSTCD | 2 | blue |
| ENSBTAG00000013937 | SPICE1 | 2 | blue |
| ENSBTAG00000013949 | AHCTF1 | 2 | blue |
| ENSBTAG00000013953 | CALD1 | 2 | blue |
| ENSBTAG00000013955 | TRAPPC9 | 2 | blue |
| ENSBTAG00000013983 | ZC2HC1C | 2 | blue |
| ENSBTAG00000013992 | ACYP1 | 2 | blue |
| ENSBTAG00000014005 | PPIP5K1 | 2 | blue |
| ENSBTAG00000014006 | TOR3A | 2 | blue |
| ENSBTAG00000014007 | ELMOD2 | 2 | blue |

| | | | |
|---|---|---|---|
| ENSBTAG00000014015 | PLA2G6 | 2 | blue |
| ENSBTAG00000014034 | NA | 2 | blue |
| ENSBTAG00000014054 | CFAP206 | 2 | blue |
| ENSBTAG00000014057 | SLC35A1 | 2 | blue |
| ENSBTAG00000014059 | ATP2B4 | 2 | blue |
| ENSBTAG00000014069 | PDK4 | 2 | blue |
| ENSBTAG00000014089 | PPCDC | 2 | blue |
| ENSBTAG00000014095 | HAUS7 | 2 | blue |
| ENSBTAG00000014112 | EXOC4 | 2 | blue |
| ENSBTAG00000014137 | CEP164 | 2 | blue |
| ENSBTAG00000014138 | MTM1 | 2 | blue |
| ENSBTAG00000014150 | BBLN | 2 | blue |
| ENSBTAG00000014151 | RCSD1 | 2 | blue |
| ENSBTAG00000014159 | TTL | 2 | blue |
| ENSBTAG00000014171 | NAPEPLD | 2 | blue |
| ENSBTAG00000014178 | RALGAPA2 | 2 | blue |
| ENSBTAG00000014220 | LOC518526 | 2 | blue |
| ENSBTAG00000014230 | VRK1 | 2 | blue |
| ENSBTAG00000014246 | CENPH | 2 | blue |
| ENSBTAG00000014272 | MMUT | 2 | blue |
| ENSBTAG00000014313 | PYROXD2 | 2 | blue |
| ENSBTAG00000014322 | FAM234B | 2 | blue |
| ENSBTAG00000014331 | UBL4A | 2 | blue |
| ENSBTAG00000014343 | IFT22 | 2 | blue |
| ENSBTAG00000014351 | EIF2AK4 | 2 | blue |
| ENSBTAG00000014353 | SRP14 | 2 | blue |
| ENSBTAG00000014355 | MYCBPAP | 2 | blue |
| ENSBTAG00000014373 | CETN3 | 2 | blue |
| ENSBTAG00000014401 | SORBS3 | 2 | blue |
| ENSBTAG00000014411 | NA | 2 | blue |
| ENSBTAG00000014448 | VKORC1L1 | 2 | blue |
| ENSBTAG00000014458 | MROH1 | 2 | blue |
| ENSBTAG00000014461 | ANAPC13 | 2 | blue |
| ENSBTAG00000014468 | AP1S1 | 2 | blue |
| ENSBTAG00000014469 | NBEAL1 | 2 | blue |
| ENSBTAG00000014479 | TUBGCP4 | 2 | blue |
| ENSBTAG00000014484 | TMEM26 | 2 | blue |
| ENSBTAG00000014493 | TRIP4 | 2 | blue |
| ENSBTAG00000014502 | BRAT1 | 2 | blue |
| ENSBTAG00000014503 | IQCE | 2 | blue |
| ENSBTAG00000014512 | WDR19 | 2 | blue |
| ENSBTAG00000014521 | UGDH | 2 | blue |
| ENSBTAG00000014550 | GLT8D1 | 2 | blue |
| ENSBTAG00000014564 | XXYLT1 | 2 | blue |
| ENSBTAG00000014587 | NA | 2 | blue |
| ENSBTAG00000014595 | LIG1 | 2 | blue |
| ENSBTAG00000014597 | ZSWIM9 | 2 | blue |

| | | | |
|---|---|---|---|
| ENSBTAG00000014599 | LRRC66 | 2 | blue |
| ENSBTAG00000014601 | SGCB | 2 | blue |
| ENSBTAG00000014608 | KDM1B | 2 | blue |
| ENSBTAG00000014623 | MCM8 | 2 | blue |
| ENSBTAG00000014633 | ABCD4 | 2 | blue |
| ENSBTAG00000014644 | DUS2 | 2 | blue |
| ENSBTAG00000014668 | NAGK | 2 | blue |
| ENSBTAG00000014679 | DHX57 | 2 | blue |
| ENSBTAG00000014682 | NA | 2 | blue |
| ENSBTAG00000014687 | TP53I13 | 2 | blue |
| ENSBTAG00000014689 | CSPP1 | 2 | blue |
| ENSBTAG00000014714 | TUBGCP5 | 2 | blue |
| ENSBTAG00000014726 | NA | 2 | blue |
| ENSBTAG00000014727 | RFC4 | 2 | blue |
| ENSBTAG00000014730 | NCAPD2 | 2 | blue |
| ENSBTAG00000014764 | CD9 | 2 | blue |
| ENSBTAG00000014768 | ZNF786 | 2 | blue |
| ENSBTAG00000014773 | HMMR | 2 | blue |
| ENSBTAG00000014784 | NT5DC2 | 2 | blue |
| ENSBTAG00000014793 | C16H1orf112 | 2 | blue |
| ENSBTAG00000014797 | SEC22A | 2 | blue |
| ENSBTAG00000014824 | MMP14 | 2 | blue |
| ENSBTAG00000014831 | PPP1R3C | 2 | blue |
| ENSBTAG00000014835 | SPARC | 2 | blue |
| ENSBTAG00000014879 | FAM185A | 2 | blue |
| ENSBTAG00000014904 | SIRT5 | 2 | blue |
| ENSBTAG00000014907 | IL11RA | 2 | blue |
| ENSBTAG00000014922 | SHB | 2 | blue |
| ENSBTAG00000014923 | BBS2 | 2 | blue |
| ENSBTAG00000014936 | SLC25A51 | 2 | blue |
| ENSBTAG00000014945 | IQSEC2 | 2 | blue |
| ENSBTAG00000014966 | PLXNB2 | 2 | blue |
| ENSBTAG00000014996 | NA | 2 | blue |
| ENSBTAG00000015006 | NA | 2 | blue |
| ENSBTAG00000015008 | CPA3 | 2 | blue |
| ENSBTAG00000015012 | PTRHD1 | 2 | blue |
| ENSBTAG00000015015 | HINFP | 2 | blue |
| ENSBTAG00000015017 | GAL3ST4 | 2 | blue |
| ENSBTAG00000015019 | STN1 | 2 | blue |
| ENSBTAG00000015038 | HADHA | 2 | blue |
| ENSBTAG00000015050 | HCN2 | 2 | blue |
| ENSBTAG00000015052 | PROX2 | 2 | blue |
| ENSBTAG00000015066 | LANCL1 | 2 | blue |
| ENSBTAG00000015076 | LATS1 | 2 | blue |
| ENSBTAG00000015089 | LGALS1 | 2 | blue |
| ENSBTAG00000015097 | CACFD1 | 2 | blue |
| ENSBTAG00000015104 | CCDC28B | 2 | blue |

| | | | |
|---|---|---|---|
| ENSBTAG00000015108 | USHBP1 | 2 | blue |
| ENSBTAG00000015115 | ACP4 | 2 | blue |
| ENSBTAG00000015131 | SLC29A1 | 2 | blue |
| ENSBTAG00000015142 | MAPKBP1 | 2 | blue |
| ENSBTAG00000015155 | HACD3 | 2 | blue |
| ENSBTAG00000015158 | INTS14 | 2 | blue |
| ENSBTAG00000015164 | SLC27A5 | 2 | blue |
| ENSBTAG00000015169 | UBXN4 | 2 | blue |
| ENSBTAG00000015172 | MCM6 | 2 | blue |
| ENSBTAG00000015178 | ECI2 | 2 | blue |
| ENSBTAG00000015181 | EPM2A | 2 | blue |
| ENSBTAG00000015184 | SYDE1 | 2 | blue |
| ENSBTAG00000015186 | ILVBL | 2 | blue |
| ENSBTAG00000015193 | NA | 2 | blue |
| ENSBTAG00000015200 | L2HGDH | 2 | blue |
| ENSBTAG00000015202 | DMAC2L | 2 | blue |
| ENSBTAG00000015220 | AGO4 | 2 | blue |
| ENSBTAG00000015221 | PCCB | 2 | blue |
| ENSBTAG00000015230 | PLA2G12A | 2 | blue |
| ENSBTAG00000015251 | ALG13 | 2 | blue |
| ENSBTAG00000015254 | GLB1 | 2 | blue |
| ENSBTAG00000015265 | TIGD2 | 2 | blue |
| ENSBTAG00000015268 | PICK1 | 2 | blue |
| ENSBTAG00000015299 | NA | 2 | blue |
| ENSBTAG00000015314 | ASL | 2 | blue |
| ENSBTAG00000015334 | ZHX1 | 2 | blue |
| ENSBTAG00000015338 | DSCC1 | 2 | blue |
| ENSBTAG00000015343 | DYNC2I2 | 2 | blue |
| ENSBTAG00000015346 | NASP | 2 | blue |
| ENSBTAG00000015361 | CNTFR | 2 | blue |
| ENSBTAG00000015366 | SFRP4 | 2 | blue |
| ENSBTAG00000015368 | SEPTIN4 | 2 | blue |
| ENSBTAG00000015369 | MLLT11 | 2 | blue |
| ENSBTAG00000015381 | ARHGAP18 | 2 | blue |
| ENSBTAG00000015390 | FN3KRP | 2 | blue |
| ENSBTAG00000015392 | TTF2 | 2 | blue |
| ENSBTAG00000015394 | NA | 2 | blue |
| ENSBTAG00000015403 | TPP1 | 2 | blue |
| ENSBTAG00000015414 | TBCD | 2 | blue |
| ENSBTAG00000015416 | PEBP4 | 2 | blue |
| ENSBTAG00000015427 | CFDP1 | 2 | blue |
| ENSBTAG00000015432 | TMEM231 | 2 | blue |
| ENSBTAG00000015436 | SLC27A4 | 2 | blue |
| ENSBTAG00000015478 | SRD5A1 | 2 | blue |
| ENSBTAG00000015481 | MTIF2 | 2 | blue |
| ENSBTAG00000015490 | HS1BP3 | 2 | blue |
| ENSBTAG00000015516 | CCDC28A | 2 | blue |

| | | | |
|---|---|---|---|
| ENSBTAG00000015521 | GEMIN6 | 2 | blue |
| ENSBTAG00000015559 | ERI3 | 2 | blue |
| ENSBTAG00000015584 | BMI1 | 2 | blue |
| ENSBTAG00000015588 | TMEM209 | 2 | blue |
| ENSBTAG00000015590 | CILK1 | 2 | blue |
| ENSBTAG00000015595 | MCM5 | 2 | blue |
| ENSBTAG00000015596 | SLCO2B1 | 2 | blue |
| ENSBTAG00000015602 | MRNIP | 2 | blue |
| ENSBTAG00000015607 | ERMP1 | 2 | blue |
| ENSBTAG00000015659 | CIAO1 | 2 | blue |
| ENSBTAG00000015719 | PIGF | 2 | blue |
| ENSBTAG00000015720 | WWP1 | 2 | blue |
| ENSBTAG00000015734 | RMDN1 | 2 | blue |
| ENSBTAG00000015745 | RAB3IL1 | 2 | blue |
| ENSBTAG00000015764 | EML2 | 2 | blue |
| ENSBTAG00000015766 | ZFPM1 | 2 | blue |
| ENSBTAG00000015769 | VSIG4 | 2 | blue |
| ENSBTAG00000015782 | SCAP | 2 | blue |
| ENSBTAG00000015788 | CCDC171 | 2 | blue |
| ENSBTAG00000015793 | RAB38 | 2 | blue |
| ENSBTAG00000015807 | GALT | 2 | blue |
| ENSBTAG00000015860 | FGD1 | 2 | blue |
| ENSBTAG00000015894 | WWOX | 2 | blue |
| ENSBTAG00000015899 | NA | 2 | blue |
| ENSBTAG00000015915 | SBSN | 2 | blue |
| ENSBTAG00000015926 | ABLIM2 | 2 | blue |
| ENSBTAG00000015930 | DNAL1 | 2 | blue |
| ENSBTAG00000015936 | BRINP1 | 2 | blue |
| ENSBTAG00000015950 | ISOC2 | 2 | blue |
| ENSBTAG00000015963 | DMAC1 | 2 | blue |
| ENSBTAG00000015972 | GLRX2 | 2 | blue |
| ENSBTAG00000015996 | GPC1 | 2 | blue |
| ENSBTAG00000016005 | PPP3CA | 2 | blue |
| ENSBTAG00000016010 | KLHL7 | 2 | blue |
| ENSBTAG00000016013 | ASB14 | 2 | blue |
| ENSBTAG00000016028 | ZNF691 | 2 | blue |
| ENSBTAG00000016038 | GCC2 | 2 | blue |
| ENSBTAG00000016048 | CPT1B | 2 | blue |
| ENSBTAG00000016053 | ARSA | 2 | blue |
| ENSBTAG00000016062 | C21H14orf28 | 2 | blue |
| ENSBTAG00000016091 | KLHL25 | 2 | blue |
| ENSBTAG00000016092 | SPATS2L | 2 | blue |
| ENSBTAG00000016096 | SYP | 2 | blue |
| ENSBTAG00000016097 | CACNA1F | 2 | blue |
| ENSBTAG00000016108 | BTBD2 | 2 | blue |
| ENSBTAG00000016131 | NCAPG2 | 2 | blue |
| ENSBTAG00000016152 | DAB2 | 2 | blue |

| | | | |
|---|---|---|---|
| ENSBTAG00000016175 | NA | 2 | blue |
| ENSBTAG00000016191 | ZNF32 | 2 | blue |
| ENSBTAG00000016193 | FASTKD2 | 2 | blue |
| ENSBTAG00000016211 | GCDH | 2 | blue |
| ENSBTAG00000016213 | SYCE2 | 2 | blue |
| ENSBTAG00000016218 | PTGES2 | 2 | blue |
| ENSBTAG00000016221 | AMOT | 2 | blue |
| ENSBTAG00000016231 | POFUT1 | 2 | blue |
| ENSBTAG00000016234 | DUOX2 | 2 | blue |
| ENSBTAG00000016236 | SLC12A6 | 2 | blue |
| ENSBTAG00000016243 | RBL2 | 2 | blue |
| ENSBTAG00000016263 | SLC25A14 | 2 | blue |
| ENSBTAG00000016268 | XRCC1 | 2 | blue |
| ENSBTAG00000016277 | MTARC2 | 2 | blue |
| ENSBTAG00000016290 | MOB1B | 2 | blue |
| ENSBTAG00000016307 | CDC7 | 2 | blue |
| ENSBTAG00000016313 | ECH1 | 2 | blue |
| ENSBTAG00000016319 | CYB5D2 | 2 | blue |
| ENSBTAG00000016343 | KCNIP2 | 2 | blue |
| ENSBTAG00000016352 | STX10 | 2 | blue |
| ENSBTAG00000016369 | TSEN15 | 2 | blue |
| ENSBTAG00000016373 | TTC26 | 2 | blue |
| ENSBTAG00000016415 | LTA4H | 2 | blue |
| ENSBTAG00000016429 | TMEM205 | 2 | blue |
| ENSBTAG00000016430 | METTL8 | 2 | blue |
| ENSBTAG00000016442 | MAP4K3 | 2 | blue |
| ENSBTAG00000016456 | CDC42BPB | 2 | blue |
| ENSBTAG00000016471 | PDPR | 2 | blue |
| ENSBTAG00000016481 | CALU | 2 | blue |
| ENSBTAG00000016495 | LINS1 | 2 | blue |
| ENSBTAG00000016497 | FAM168A | 2 | blue |
| ENSBTAG00000016504 | AGAP1 | 2 | blue |
| ENSBTAG00000016506 | ST3GAL4 | 2 | blue |
| ENSBTAG00000016516 | CYB5R3 | 2 | blue |
| ENSBTAG00000016522 | C21H15orf40 | 2 | blue |
| ENSBTAG00000016541 | CAMK1G | 2 | blue |
| ENSBTAG00000016548 | TEX12 | 2 | blue |
| ENSBTAG00000016549 | CARMIL1 | 2 | blue |
| ENSBTAG00000016559 | NUDT22 | 2 | blue |
| ENSBTAG00000016563 | GOLGA4 | 2 | blue |
| ENSBTAG00000016564 | NPRL3 | 2 | blue |
| ENSBTAG00000016571 | FAM234A | 2 | blue |
| ENSBTAG00000016580 | TEF | 2 | blue |
| ENSBTAG00000016588 | PGAP6 | 2 | blue |
| ENSBTAG00000016589 | PRPF40B | 2 | blue |
| ENSBTAG00000016590 | NME4 | 2 | blue |
| ENSBTAG00000016594 | PIGQ | 2 | blue |

| | | | |
|---|---|---|---|
| ENSBTAG00000016618 | DSTYK | 2 | blue |
| ENSBTAG00000016622 | TCAIM | 2 | blue |
| ENSBTAG00000016640 | HEATR3 | 2 | blue |
| ENSBTAG00000016658 | ABHD4 | 2 | blue |
| ENSBTAG00000016664 | SLC2A4RG | 2 | blue |
| ENSBTAG00000016676 | PTER | 2 | blue |
| ENSBTAG00000016679 | ETFDH | 2 | blue |
| ENSBTAG00000016694 | KIAA0319L | 2 | blue |
| ENSBTAG00000016696 | NCDN | 2 | blue |
| ENSBTAG00000016707 | FKBP9 | 2 | blue |
| ENSBTAG00000016712 | CLCA3 | 2 | blue |
| ENSBTAG00000016724 | NPEPL1 | 2 | blue |
| ENSBTAG00000016731 | TMEM42 | 2 | blue |
| ENSBTAG00000016737 | CDK8 | 2 | blue |
| ENSBTAG00000016742 | WFDC3 | 2 | blue |
| ENSBTAG00000016754 | KLHL38 | 2 | blue |
| ENSBTAG00000016762 | SLC25A12 | 2 | blue |
| ENSBTAG00000016775 | SLC27A1 | 2 | blue |
| ENSBTAG00000016795 | RUFY3 | 2 | blue |
| ENSBTAG00000016819 | FABP3 | 2 | blue |
| ENSBTAG00000016836 | PDK1 | 2 | blue |
| ENSBTAG00000016847 | ARL4D | 2 | blue |
| ENSBTAG00000016854 | AK7 | 2 | blue |
| ENSBTAG00000016876 | LOC101905801 | 2 | blue |
| ENSBTAG00000016943 | NEK7 | 2 | blue |
| ENSBTAG00000016952 | PSMD5 | 2 | blue |
| ENSBTAG00000016963 | ADAMTS6 | 2 | blue |
| ENSBTAG00000016967 | RARS2 | 2 | blue |
| ENSBTAG00000016969 | ORC3 | 2 | blue |
| ENSBTAG00000016975 | H2AZ2 | 2 | blue |
| ENSBTAG00000016982 | MCOLN3 | 2 | blue |
| ENSBTAG00000016995 | HDHD5 | 2 | blue |
| ENSBTAG00000016997 | SIGLEC11 | 2 | blue |
| ENSBTAG00000017011 | CCDC125 | 2 | blue |
| ENSBTAG00000017038 | HPS4 | 2 | blue |
| ENSBTAG00000017051 | ACP6 | 2 | blue |
| ENSBTAG00000017061 | SMARCAD1 | 2 | blue |
| ENSBTAG00000017103 | MAP3K13 | 2 | blue |
| ENSBTAG00000017136 | SSTR2 | 2 | blue |
| ENSBTAG00000017140 | CIP2A | 2 | blue |
| ENSBTAG00000017151 | SH3TC2 | 2 | blue |
| ENSBTAG00000017174 | SCN11A | 2 | blue |
| ENSBTAG00000017179 | USP12 | 2 | blue |
| ENSBTAG00000017195 | FANCC | 2 | blue |
| ENSBTAG00000017215 | WDR76 | 2 | blue |
| ENSBTAG00000017242 | FADS6 | 2 | blue |
| ENSBTAG00000017245 | COPG2 | 2 | blue |

| | | | |
|---|---|---|---|
| ENSBTAG00000017253 | MECR | 2 | blue |
| ENSBTAG00000017268 | PROCA1 | 2 | blue |
| ENSBTAG00000017284 | RHBDL1 | 2 | blue |
| ENSBTAG00000017296 | POLN | 2 | blue |
| ENSBTAG00000017298 | AAMDC | 2 | blue |
| ENSBTAG00000017329 | GMNN | 2 | blue |
| ENSBTAG00000017335 | HGFAC | 2 | blue |
| ENSBTAG00000017349 | PCDHGA8 | 2 | blue |
| ENSBTAG00000017354 | MPP7 | 2 | blue |
| ENSBTAG00000017365 | ZBTB47 | 2 | blue |
| ENSBTAG00000017368 | YBX1 | 2 | blue |
| ENSBTAG00000017388 | SVBP | 2 | blue |
| ENSBTAG00000017412 | SOCS6 | 2 | blue |
| ENSBTAG00000017421 | SLC3A1 | 2 | blue |
| ENSBTAG00000017429 | PREPL | 2 | blue |
| ENSBTAG00000017437 | TMEM237 | 2 | blue |
| ENSBTAG00000017450 | KLHL24 | 2 | blue |
| ENSBTAG00000017452 | RCAN3 | 2 | blue |
| ENSBTAG00000017465 | GNS | 2 | blue |
| ENSBTAG00000017468 | NUCB2 | 2 | blue |
| ENSBTAG00000017469 | CCDC88A | 2 | blue |
| ENSBTAG00000017490 | PLD1 | 2 | blue |
| ENSBTAG00000017502 | RIMKLA | 2 | blue |
| ENSBTAG00000017507 | SV2C | 2 | blue |
| ENSBTAG00000017515 | NA | 2 | blue |
| ENSBTAG00000017530 | PLGRKT | 2 | blue |
| ENSBTAG00000017543 | ATP23 | 2 | blue |
| ENSBTAG00000017551 | DROSHA | 2 | blue |
| ENSBTAG00000017554 | RTN4IP1 | 2 | blue |
| ENSBTAG00000017569 | C20H5orf22 | 2 | blue |
| ENSBTAG00000017582 | CHEK1 | 2 | blue |
| ENSBTAG00000017594 | ZHX3 | 2 | blue |
| ENSBTAG00000017595 | HS2ST1 | 2 | blue |
| ENSBTAG00000017616 | ADSS1 | 2 | blue |
| ENSBTAG00000017660 | LOC100297302 | 2 | blue |
| ENSBTAG00000017662 | EEF2K | 2 | blue |
| ENSBTAG00000017664 | HGF | 2 | blue |
| ENSBTAG00000017666 | ABCC5 | 2 | blue |
| ENSBTAG00000017676 | TPK1 | 2 | blue |
| ENSBTAG00000017681 | RIN1 | 2 | blue |
| ENSBTAG00000017690 | CARNS1 | 2 | blue |
| ENSBTAG00000017704 | ABCG2 | 2 | blue |
| ENSBTAG00000017706 | SLC39A3 | 2 | blue |
| ENSBTAG00000017710 | ECHS1 | 2 | blue |
| ENSBTAG00000017727 | KIFAP3 | 2 | blue |
| ENSBTAG00000017741 | HACD4 | 2 | blue |
| ENSBTAG00000017748 | RNF44 | 2 | blue |

| | | | |
|---|---|---|---|
| ENSBTAG00000017752 | DYNLRB2 | 2 | blue |
| ENSBTAG00000017766 | NA | 2 | blue |
| ENSBTAG00000017775 | SDCCAG8 | 2 | blue |
| ENSBTAG00000017783 | TSPYL2 | 2 | blue |
| ENSBTAG00000017788 | AKT3 | 2 | blue |
| ENSBTAG00000017802 | THRB | 2 | blue |
| ENSBTAG00000017815 | STX8 | 2 | blue |
| ENSBTAG00000017835 | C7H5orf63 | 2 | blue |
| ENSBTAG00000017844 | STIL | 2 | blue |
| ENSBTAG00000017852 | ALDH9A1 | 2 | blue |
| ENSBTAG00000017866 | CD36 | 2 | blue |
| ENSBTAG00000017868 | DHX40 | 2 | blue |
| ENSBTAG00000017879 | C9H6orf163 | 2 | blue |
| ENSBTAG00000017922 | DOLPP1 | 2 | blue |
| ENSBTAG00000017943 | GALNT6 | 2 | blue |
| ENSBTAG00000017958 | AHI1 | 2 | blue |
| ENSBTAG00000018022 | SPINDOC | 2 | blue |
| ENSBTAG00000018031 | PODXL2 | 2 | blue |
| ENSBTAG00000018041 | ACADSB | 2 | blue |
| ENSBTAG00000018061 | LNPK | 2 | blue |
| ENSBTAG00000018067 | S100PBP | 2 | blue |
| ENSBTAG00000018082 | QPRT | 2 | blue |
| ENSBTAG00000018094 | ZNF689 | 2 | blue |
| ENSBTAG00000018103 | HMGB1 | 2 | blue |
| ENSBTAG00000018115 | PEBP1 | 2 | blue |
| ENSBTAG00000018133 | SEMA3A | 2 | blue |
| ENSBTAG00000018151 | HELQ | 2 | blue |
| ENSBTAG00000018157 | NA | 2 | blue |
| ENSBTAG00000018159 | CASP2 | 2 | blue |
| ENSBTAG00000018175 | HEXD | 2 | blue |
| ENSBTAG00000018176 | CYBC1 | 2 | blue |
| ENSBTAG00000018189 | CSTB | 2 | blue |
| ENSBTAG00000018192 | ALG8 | 2 | blue |
| ENSBTAG00000018205 | PHC1 | 2 | blue |
| ENSBTAG00000018213 | TBCEL | 2 | blue |
| ENSBTAG00000018224 | GLP1R | 2 | blue |
| ENSBTAG00000018235 | SLC45A2 | 2 | blue |
| ENSBTAG00000018237 | MYO16 | 2 | blue |
| ENSBTAG00000018245 | SLC1A3 | 2 | blue |
| ENSBTAG00000018256 | SMC6 | 2 | blue |
| ENSBTAG00000018278 | ATP5PO | 2 | blue |
| ENSBTAG00000018279 | SPATA5L1 | 2 | blue |
| ENSBTAG00000018281 | GPX7 | 2 | blue |
| ENSBTAG00000018283 | ACAA1 | 2 | blue |
| ENSBTAG00000018313 | MBNL2 | 2 | blue |
| ENSBTAG00000018316 | ZMYND15 | 2 | blue |
| ENSBTAG00000018362 | TMEM109 | 2 | blue |

| | | | |
|---|---|---|---|
| ENSBTAG00000018372 | STARD7 | 2 | blue |
| ENSBTAG00000018373 | DPYSL2 | 2 | blue |
| ENSBTAG00000018379 | TMEM175 | 2 | blue |
| ENSBTAG00000018383 | ATAD5 | 2 | blue |
| ENSBTAG00000018386 | TEFM | 2 | blue |
| ENSBTAG00000018394 | SDR42E1 | 2 | blue |
| ENSBTAG00000018399 | MYH15 | 2 | blue |
| ENSBTAG00000018400 | BRMS1L | 2 | blue |
| ENSBTAG00000018404 | PRKG1 | 2 | blue |
| ENSBTAG00000018410 | EBPL | 2 | blue |
| ENSBTAG00000018415 | TTLL4 | 2 | blue |
| ENSBTAG00000018420 | POLG2 | 2 | blue |
| ENSBTAG00000018426 | MRRF | 2 | blue |
| ENSBTAG00000018438 | RRAGD | 2 | blue |
| ENSBTAG00000018454 | UFSP2 | 2 | blue |
| ENSBTAG00000018469 | ALDH6A1 | 2 | blue |
| ENSBTAG00000018471 | SSR3 | 2 | blue |
| ENSBTAG00000018479 | PTPMT1 | 2 | blue |
| ENSBTAG00000018502 | ARMC9 | 2 | blue |
| ENSBTAG00000018538 | CEP70 | 2 | blue |
| ENSBTAG00000018548 | INTS7 | 2 | blue |
| ENSBTAG00000018555 | NA | 2 | blue |
| ENSBTAG00000018569 | CUL4B | 2 | blue |
| ENSBTAG00000018578 | LOXL3 | 2 | blue |
| ENSBTAG00000018589 | RFC2 | 2 | blue |
| ENSBTAG00000018600 | VGLL4 | 2 | blue |
| ENSBTAG00000018616 | IPO11 | 2 | blue |
| ENSBTAG00000018633 | RABAC1 | 2 | blue |
| ENSBTAG00000018637 | HDDC3 | 2 | blue |
| ENSBTAG00000018642 | RCCD1 | 2 | blue |
| ENSBTAG00000018682 | SETD4 | 2 | blue |
| ENSBTAG00000018688 | CBR3 | 2 | blue |
| ENSBTAG00000018694 | ACSS3 | 2 | blue |
| ENSBTAG00000018737 | GLMN | 2 | blue |
| ENSBTAG00000018745 | CEP290 | 2 | blue |
| ENSBTAG00000018746 | NA | 2 | blue |
| ENSBTAG00000018768 | HMGN1 | 2 | blue |
| ENSBTAG00000018770 | HSDL1 | 2 | blue |
| ENSBTAG00000018795 | PMS1 | 2 | blue |
| ENSBTAG00000018808 | SLC25A35 | 2 | blue |
| ENSBTAG00000018809 | RANGRF | 2 | blue |
| ENSBTAG00000018823 | GRN | 2 | blue |
| ENSBTAG00000018837 | SLC39A11 | 2 | blue |
| ENSBTAG00000018854 | SOD1 | 2 | blue |
| ENSBTAG00000018880 | TRPV1 | 2 | blue |
| ENSBTAG00000018894 | PEX11G | 2 | blue |
| ENSBTAG00000018902 | ZC2HC1A | 2 | blue |

| | | | |
|---|---|---|---|
| ENSBTAG00000018904 | ENKD1 | 2 | blue |
| ENSBTAG00000018920 | TRIM47 | 2 | blue |
| ENSBTAG00000018937 | HMOX2 | 2 | blue |
| ENSBTAG00000018938 | CDIP1 | 2 | blue |
| ENSBTAG00000018948 | HIF3A | 2 | blue |
| ENSBTAG00000018960 | NA | 2 | blue |
| ENSBTAG00000018971 | RAD51B | 2 | blue |
| ENSBTAG00000018981 | NA | 2 | blue |
| ENSBTAG00000019011 | PGM1 | 2 | blue |
| ENSBTAG00000019014 | MAGEF1 | 2 | blue |
| ENSBTAG00000019025 | NDUFA11 | 2 | blue |
| ENSBTAG00000019033 | CD84 | 2 | blue |
| ENSBTAG00000019044 | BAIAP2 | 2 | blue |
| ENSBTAG00000019049 | AATK | 2 | blue |
| ENSBTAG00000019065 | EEPD1 | 2 | blue |
| ENSBTAG00000019070 | PMP22 | 2 | blue |
| ENSBTAG00000019071 | COMMD10 | 2 | blue |
| ENSBTAG00000019079 | PLCB2 | 2 | blue |
| ENSBTAG00000019086 | TELO2 | 2 | blue |
| ENSBTAG00000019090 | PLEKHH3 | 2 | blue |
| ENSBTAG00000019093 | AMY2B | 2 | blue |
| ENSBTAG00000019097 | CNTNAP1 | 2 | blue |
| ENSBTAG00000019104 | FAAP100 | 2 | blue |
| ENSBTAG00000019120 | WDHD1 | 2 | blue |
| ENSBTAG00000019121 | IFT122 | 2 | blue |
| ENSBTAG00000019124 | NA | 2 | blue |
| ENSBTAG00000019129 | BTD | 2 | blue |
| ENSBTAG00000019146 | OSBP2 | 2 | blue |
| ENSBTAG00000019150 | PLD3 | 2 | blue |
| ENSBTAG00000019161 | NPRL2 | 2 | blue |
| ENSBTAG00000019166 | NSMCE4A | 2 | blue |
| ENSBTAG00000019167 | INPPL1 | 2 | blue |
| ENSBTAG00000019194 | SHLD2 | 2 | blue |
| ENSBTAG00000019209 | RAD1 | 2 | blue |
| ENSBTAG00000019214 | USP14 | 2 | blue |
| ENSBTAG00000019218 | ATP6V0A1 | 2 | blue |
| ENSBTAG00000019229 | TMEM144 | 2 | blue |
| ENSBTAG00000019232 | TYW5 | 2 | blue |
| ENSBTAG00000019235 | MAGOHB | 2 | blue |
| ENSBTAG00000019237 | TXNDC5 | 2 | blue |
| ENSBTAG00000019242 | CHRNB1 | 2 | blue |
| ENSBTAG00000019256 | GLA | 2 | blue |
| ENSBTAG00000019266 | NA | 2 | blue |
| ENSBTAG00000019280 | CRYBB1 | 2 | blue |
| ENSBTAG00000019283 | FAM186B | 2 | blue |
| ENSBTAG00000019295 | MDH1 | 2 | blue |
| ENSBTAG00000019309 | NA | 2 | blue |

| | | | |
|---|---|---|---|
| ENSBTAG00000019315 | PLA2G7 | 2 | blue |
| ENSBTAG00000019321 | CCDC57 | 2 | blue |
| ENSBTAG00000019337 | ACVR1C | 2 | blue |
| ENSBTAG00000019347 | PLXDC1 | 2 | blue |
| ENSBTAG00000019358 | RNF214 | 2 | blue |
| ENSBTAG00000019362 | NOA1A | 2 | blue |
| ENSBTAG00000019365 | BACE1 | 2 | blue |
| ENSBTAG00000019369 | TSPAN4 | 2 | blue |
| ENSBTAG00000019406 | IGF2BP3 | 2 | blue |
| ENSBTAG00000019407 | NA | 2 | blue |
| ENSBTAG00000019417 | ARMCX2 | 2 | blue |
| ENSBTAG00000019418 | UBE3D | 2 | blue |
| ENSBTAG00000019434 | PDCD4 | 2 | blue |
| ENSBTAG00000019457 | MRPL22 | 2 | blue |
| ENSBTAG00000019478 | NAAA | 2 | blue |
| ENSBTAG00000019480 | PDE6D | 2 | blue |
| ENSBTAG00000019486 | CLDN12 | 2 | blue |
| ENSBTAG00000019492 | FANCE | 2 | blue |
| ENSBTAG00000019516 | COMT | 2 | blue |
| ENSBTAG00000019533 | ADTRP | 2 | blue |
| ENSBTAG00000019540 | NA | 2 | blue |
| ENSBTAG00000019542 | MRPL39 | 2 | blue |
| ENSBTAG00000019568 | IFT74 | 2 | blue |
| ENSBTAG00000019579 | NA | 2 | blue |
| ENSBTAG00000019589 | CFAP410 | 2 | blue |
| ENSBTAG00000019614 | FAM76A | 2 | blue |
| ENSBTAG00000019615 | PHTF1 | 2 | blue |
| ENSBTAG00000019621 | NDRG3 | 2 | blue |
| ENSBTAG00000019625 | EHHADH | 2 | blue |
| ENSBTAG00000019630 | RGL1 | 2 | blue |
| ENSBTAG00000019634 | PIDD1 | 2 | blue |
| ENSBTAG00000019663 | DECR1 | 2 | blue |
| ENSBTAG00000019667 | RNF187 | 2 | blue |
| ENSBTAG00000019670 | NA | 2 | blue |
| ENSBTAG00000019674 | CLIP3 | 2 | blue |
| ENSBTAG00000019678 | ALKBH6 | 2 | blue |
| ENSBTAG00000019687 | NMRK1 | 2 | blue |
| ENSBTAG00000019692 | BCL2L2 | 2 | blue |
| ENSBTAG00000019704 | HLTF | 2 | blue |
| ENSBTAG00000019722 | GUCY2C | 2 | blue |
| ENSBTAG00000019728 | CD164L2 | 2 | blue |
| ENSBTAG00000019729 | COQ3 | 2 | blue |
| ENSBTAG00000019738 | MDM1 | 2 | blue |
| ENSBTAG00000019745 | LMF1 | 2 | blue |
| ENSBTAG00000019746 | CCDC66 | 2 | blue |
| ENSBTAG00000019750 | TMEM106B | 2 | blue |
| ENSBTAG00000019770 | APOA4 | 2 | blue |

| | | | |
|---|---|---|---|
| ENSBTAG00000019771 | PALM | 2 | blue |
| ENSBTAG00000019781 | CLASP1 | 2 | blue |
| ENSBTAG00000019790 | MSH5 | 2 | blue |
| ENSBTAG00000019819 | FUT8 | 2 | blue |
| ENSBTAG00000019828 | TAB1 | 2 | blue |
| ENSBTAG00000019836 | GSKIP | 2 | blue |
| ENSBTAG00000019843 | HAUS6 | 2 | blue |
| ENSBTAG00000019848 | NA | 2 | blue |
| ENSBTAG00000019859 | B3GALT2 | 2 | blue |
| ENSBTAG00000019864 | MAPK15 | 2 | blue |
| ENSBTAG00000019867 | COMMD1 | 2 | blue |
| ENSBTAG00000019887 | DHODH | 2 | blue |
| ENSBTAG00000019907 | WNK4 | 2 | blue |
| ENSBTAG00000019952 | ARMC8 | 2 | blue |
| ENSBTAG00000019956 | CMPK1 | 2 | blue |
| ENSBTAG00000019964 | GAS6 | 2 | blue |
| ENSBTAG00000019966 | TICAM1 | 2 | blue |
| ENSBTAG00000019984 | GINS1 | 2 | blue |
| ENSBTAG00000019993 | APLN | 2 | blue |
| ENSBTAG00000020030 | FITM2 | 2 | blue |
| ENSBTAG00000020031 | PKD2 | 2 | blue |
| ENSBTAG00000020050 | MLEC | 2 | blue |
| ENSBTAG00000020055 | CEP44 | 2 | blue |
| ENSBTAG00000020059 | GEN1 | 2 | blue |
| ENSBTAG00000020066 | ZW10 | 2 | blue |
| ENSBTAG00000020073 | NCEH1 | 2 | blue |
| ENSBTAG00000020074 | NKPD1 | 2 | blue |
| ENSBTAG00000020087 | CAMK2A | 2 | blue |
| ENSBTAG00000020090 | EPHX3 | 2 | blue |
| ENSBTAG00000020119 | RNF43 | 2 | blue |
| ENSBTAG00000020132 | HPF1 | 2 | blue |
| ENSBTAG00000020133 | PPP3CC | 2 | blue |
| ENSBTAG00000020144 | GPR34 | 2 | blue |
| ENSBTAG00000020147 | BBS1 | 2 | blue |
| ENSBTAG00000020148 | TEK | 2 | blue |
| ENSBTAG00000020179 | AAAS | 2 | blue |
| ENSBTAG00000020185 | ZNF275 | 2 | blue |
| ENSBTAG00000020193 | DCLRE1A | 2 | blue |
| ENSBTAG00000020214 | LRRC8B | 2 | blue |
| ENSBTAG00000020218 | ANXA7 | 2 | blue |
| ENSBTAG00000020236 | NA | 2 | blue |
| ENSBTAG00000020279 | ALG9 | 2 | blue |
| ENSBTAG00000020282 | DCTD | 2 | blue |
| ENSBTAG00000020292 | NA | 2 | blue |
| ENSBTAG00000020301 | BLM | 2 | blue |
| ENSBTAG00000020314 | COG2 | 2 | blue |
| ENSBTAG00000020319 | ALOX5 | 2 | blue |

| | | | |
|---|---|---|---|
| ENSBTAG00000020331 | COQ6 | 2 | blue |
| ENSBTAG00000020334 | ENTPD5 | 2 | blue |
| ENSBTAG00000020338 | CCT6B | 2 | blue |
| ENSBTAG00000020346 | CCDC92 | 2 | blue |
| ENSBTAG00000020357 | GOLPH3L | 2 | blue |
| ENSBTAG00000020359 | PLEKHM1 | 2 | blue |
| ENSBTAG00000020371 | ACOT8 | 2 | blue |
| ENSBTAG00000020385 | NA | 2 | blue |
| ENSBTAG00000020412 | RAP2A | 2 | blue |
| ENSBTAG00000020416 | PGAP1 | 2 | blue |
| ENSBTAG00000020434 | C18H19orf12 | 2 | blue |
| ENSBTAG00000020436 | KIF27 | 2 | blue |
| ENSBTAG00000020445 | ZNF398 | 2 | blue |
| ENSBTAG00000020446 | THOP1 | 2 | blue |
| ENSBTAG00000020448 | NA | 2 | blue |
| ENSBTAG00000020450 | CLDND2 | 2 | blue |
| ENSBTAG00000020481 | GXYLT2 | 2 | blue |
| ENSBTAG00000020498 | ACP1 | 2 | blue |
| ENSBTAG00000020503 | MTRFR | 2 | blue |
| ENSBTAG00000020504 | CDK2AP1 | 2 | blue |
| ENSBTAG00000020510 | EPHA10 | 2 | blue |
| ENSBTAG00000020518 | PARK7 | 2 | blue |
| ENSBTAG00000020527 | IDH1 | 2 | blue |
| ENSBTAG00000020539 | CHPF | 2 | blue |
| ENSBTAG00000020541 | PIGY | 2 | blue |
| ENSBTAG00000020583 | AASDH | 2 | blue |
| ENSBTAG00000020591 | VAMP4 | 2 | blue |
| ENSBTAG00000020597 | FMO3 | 2 | blue |
| ENSBTAG00000020598 | PPP2R2C | 2 | blue |
| ENSBTAG00000020605 | SMTNL2 | 2 | blue |
| ENSBTAG00000020612 | NEK3 | 2 | blue |
| ENSBTAG00000020626 | ERCC6L2 | 2 | blue |
| ENSBTAG00000020635 | VPS45 | 2 | blue |
| ENSBTAG00000020646 | NA | 2 | blue |
| ENSBTAG00000020665 | GFRA2 | 2 | blue |
| ENSBTAG00000020676 | MMP9 | 2 | blue |
| ENSBTAG00000020685 | NA | 2 | blue |
| ENSBTAG00000020699 | TSPAN11 | 2 | blue |
| ENSBTAG00000020749 | PRXL2C | 2 | blue |
| ENSBTAG00000020754 | ZNF526 | 2 | blue |
| ENSBTAG00000020802 | DNAH12 | 2 | blue |
| ENSBTAG00000020824 | KRT10 | 2 | blue |
| ENSBTAG00000020825 | IRAK1BP1 | 2 | blue |
| ENSBTAG00000020828 | NA | 2 | blue |
| ENSBTAG00000020855 | PTPN4 | 2 | blue |
| ENSBTAG00000020890 | TRIM66 | 2 | blue |
| ENSBTAG00000020894 | LAPTM4A | 2 | blue |

| | | | |
|---|---|---|---|
| ENSBTAG00000020923 | GDF1 | 2 | blue |
| ENSBTAG00000020924 | FBXW9 | 2 | blue |
| ENSBTAG00000020927 | HSD17B8 | 2 | blue |
| ENSBTAG00000020930 | GEMIN2 | 2 | blue |
| ENSBTAG00000020954 | TRIM7 | 2 | blue |
| ENSBTAG00000020957 | FZD5 | 2 | blue |
| ENSBTAG00000020961 | DUSP19 | 2 | blue |
| ENSBTAG00000020980 | CAT | 2 | blue |
| ENSBTAG00000020988 | KIZ | 2 | blue |
| ENSBTAG00000020994 | C19H17orf75 | 2 | blue |
| ENSBTAG00000020999 | DDB2 | 2 | blue |
| ENSBTAG00000021002 | ACP2 | 2 | blue |
| ENSBTAG00000021007 | FOLR1 | 2 | blue |
| ENSBTAG00000021008 | ZNF219 | 2 | blue |
| ENSBTAG00000021013 | TUBB4A | 2 | blue |
| ENSBTAG00000021023 | ZMYM4 | 2 | blue |
| ENSBTAG00000021035 | CTSK | 2 | blue |
| ENSBTAG00000021036 | MARCHF2 | 2 | blue |
| ENSBTAG00000021066 | NA | 2 | blue |
| ENSBTAG00000021068 | SUFU | 2 | blue |
| ENSBTAG00000021072 | DTNB | 2 | blue |
| ENSBTAG00000021078 | PYROXD1 | 2 | blue |
| ENSBTAG00000021096 | RTEL1 | 2 | blue |
| ENSBTAG00000021097 | PELI3 | 2 | blue |
| ENSBTAG00000021102 | GALM | 2 | blue |
| ENSBTAG00000021135 | RMI1 | 2 | blue |
| ENSBTAG00000021156 | TBC1D31 | 2 | blue |
| ENSBTAG00000021183 | MAP3K21 | 2 | blue |
| ENSBTAG00000021205 | NA | 2 | blue |
| ENSBTAG00000021208 | RRM2B | 2 | blue |
| ENSBTAG00000021215 | VWA5A | 2 | blue |
| ENSBTAG00000021222 | ZNF771 | 2 | blue |
| ENSBTAG00000021249 | LAT | 2 | blue |
| ENSBTAG00000021250 | RALBP1 | 2 | blue |
| ENSBTAG00000021254 | CENPI | 2 | blue |
| ENSBTAG00000021275 | CIB1 | 2 | blue |
| ENSBTAG00000021291 | STXBP6 | 2 | blue |
| ENSBTAG00000021301 | ACSF2 | 2 | blue |
| ENSBTAG00000021302 | CHAD | 2 | blue |
| ENSBTAG00000021318 | SCAMP5 | 2 | blue |
| ENSBTAG00000021321 | SDCBP2 | 2 | blue |
| ENSBTAG00000021357 | ZNF518A | 2 | blue |
| ENSBTAG00000021359 | NA | 2 | blue |
| ENSBTAG00000021368 | LOC782884 | 2 | blue |
| ENSBTAG00000021398 | MTRES1 | 2 | blue |
| ENSBTAG00000021423 | LKAAEAR1 | 2 | blue |
| ENSBTAG00000021427 | HOXB3 | 2 | blue |

| | | | |
|---|---|---|---|
| ENSBTAG00000021433 | LOC508131 | 2 | blue |
| ENSBTAG00000021438 | ZNF821 | 2 | blue |
| ENSBTAG00000021442 | CRTAP | 2 | blue |
| ENSBTAG00000021468 | MED24 | 2 | blue |
| ENSBTAG00000021469 | CTTNBP2 | 2 | blue |
| ENSBTAG00000021506 | ARL6IP5 | 2 | blue |
| ENSBTAG00000021525 | RAB42 | 2 | blue |
| ENSBTAG00000021535 | CROT | 2 | blue |
| ENSBTAG00000021538 | MYO1E | 2 | blue |
| ENSBTAG00000021540 | WDR11 | 2 | blue |
| ENSBTAG00000021543 | MDFIC | 2 | blue |
| ENSBTAG00000021549 | NA | 2 | blue |
| ENSBTAG00000021569 | DDB1 | 2 | blue |
| ENSBTAG00000021581 | FHOD3 | 2 | blue |
| ENSBTAG00000021592 | WRN | 2 | blue |
| ENSBTAG00000021606 | ERCC8 | 2 | blue |
| ENSBTAG00000021607 | NDUFAF2 | 2 | blue |
| ENSBTAG00000021611 | IFTAP | 2 | blue |
| ENSBTAG00000021620 | PITHD1 | 2 | blue |
| ENSBTAG00000021630 | MTFMT | 2 | blue |
| ENSBTAG00000021632 | MSRA | 2 | blue |
| ENSBTAG00000021645 | MBD4 | 2 | blue |
| ENSBTAG00000021656 | SPECC1L | 2 | blue |
| ENSBTAG00000021664 | TACR2 | 2 | blue |
| ENSBTAG00000021668 | METTL4 | 2 | blue |
| ENSBTAG00000021671 | METTL18 | 2 | blue |
| ENSBTAG00000021677 | TRIM37 | 2 | blue |
| ENSBTAG00000021678 | SLC31A1 | 2 | blue |
| ENSBTAG00000021680 | SKA2 | 2 | blue |
| ENSBTAG00000021686 | MELK | 2 | blue |
| ENSBTAG00000021694 | SPAST | 2 | blue |
| ENSBTAG00000021697 | PDGFB | 2 | blue |
| ENSBTAG00000021700 | MADD | 2 | blue |
| ENSBTAG00000021707 | MYBPC3 | 2 | blue |
| ENSBTAG00000021708 | CEP128 | 2 | blue |
| ENSBTAG00000021741 | RPS6KA2 | 2 | blue |
| ENSBTAG00000021742 | ZNF568 | 2 | blue |
| ENSBTAG00000021743 | ZNF704 | 2 | blue |
| ENSBTAG00000021752 | DNAJB4 | 2 | blue |
| ENSBTAG00000021772 | NA | 2 | blue |
| ENSBTAG00000021777 | DOC2G | 2 | blue |
| ENSBTAG00000021779 | MGST2 | 2 | blue |
| ENSBTAG00000021790 | APOOL | 2 | blue |
| ENSBTAG00000021809 | DEPDC7 | 2 | blue |
| ENSBTAG00000021813 | AMACR | 2 | blue |
| ENSBTAG00000021822 | ARV1 | 2 | blue |
| ENSBTAG00000021829 | GM2A | 2 | blue |

| | | | |
|---|---|---|---|
| ENSBTAG00000021831 | NA | 2 | blue |
| ENSBTAG00000021836 | TCEA2 | 2 | blue |
| ENSBTAG00000021843 | UBQLN2 | 2 | blue |
| ENSBTAG00000021846 | CELSR3 | 2 | blue |
| ENSBTAG00000021850 | NCKIPSD | 2 | blue |
| ENSBTAG00000021859 | PDSS2 | 2 | blue |
| ENSBTAG00000021870 | FAM98B | 2 | blue |
| ENSBTAG00000021872 | RASGRP1 | 2 | blue |
| ENSBTAG00000021874 | KIF14 | 2 | blue |
| ENSBTAG00000021880 | COQ8A | 2 | blue |
| ENSBTAG00000021899 | PDE9A | 2 | blue |
| ENSBTAG00000021901 | TRPM2 | 2 | blue |
| ENSBTAG00000021902 | ALDH5A1 | 2 | blue |
| ENSBTAG00000021903 | KIAA0319 | 2 | blue |
| ENSBTAG00000021920 | SEMA4C | 2 | blue |
| ENSBTAG00000021934 | RETSAT | 2 | blue |
| ENSBTAG00000021940 | CCDC149 | 2 | blue |
| ENSBTAG00000021944 | RTRAF | 2 | blue |
| ENSBTAG00000021953 | LARGE1 | 2 | blue |
| ENSBTAG00000021955 | NPC2 | 2 | blue |
| ENSBTAG00000021957 | LTBP2 | 2 | blue |
| ENSBTAG00000021961 | SPR | 2 | blue |
| ENSBTAG00000021978 | PARVB | 2 | blue |
| ENSBTAG00000021996 | GATAD1 | 2 | blue |
| ENSBTAG00000021997 | ITSN1 | 2 | blue |
| ENSBTAG00000022003 | NSUN6 | 2 | blue |
| ENSBTAG00000022004 | FLNB | 2 | blue |
| ENSBTAG00000022027 | DDT | 2 | blue |
| ENSBTAG00000022058 | UNG | 2 | blue |
| ENSBTAG00000022084 | NA | 2 | blue |
| ENSBTAG00000022109 | SCOC | 2 | blue |
| ENSBTAG00000022114 | TMEM65 | 2 | blue |
| ENSBTAG00000022209 | CABP2 | 2 | blue |
| ENSBTAG00000022238 | B4GAT1 | 2 | blue |
| ENSBTAG00000022244 | ACTN3 | 2 | blue |
| ENSBTAG00000022294 | FADS1 | 2 | blue |
| ENSBTAG00000022461 | SEPTIN10 | 2 | blue |
| ENSBTAG00000022509 | DNAH9 | 2 | blue |
| ENSBTAG00000022514 | CD5L | 2 | blue |
| ENSBTAG00000022564 | AKR1C4 | 2 | blue |
| ENSBTAG00000022632 | TCTA | 2 | blue |
| ENSBTAG00000022751 | ZCWPW2 | 2 | blue |
| ENSBTAG00000022777 | CDC42BPA | 2 | blue |
| ENSBTAG00000022847 | HEATR5A | 2 | blue |
| ENSBTAG00000022893 | LOC781726 | 2 | blue |
| ENSBTAG00000022920 | RNF128 | 2 | blue |
| ENSBTAG00000022991 | NBEA | 2 | blue |

| | | | |
|---|---|---|---|
| ENSBTAG00000023078 | NA | 2 | blue |
| ENSBTAG00000023106 | LOC534913 | 2 | blue |
| ENSBTAG00000023182 | WNK3 | 2 | blue |
| ENSBTAG00000023259 | OSBPL1A | 2 | blue |
| ENSBTAG00000023367 | LOC101906366 | 2 | blue |
| ENSBTAG00000023384 | CBR1 | 2 | blue |
| ENSBTAG00000023453 | C18H19orf54 | 2 | blue |
| ENSBTAG00000023464 | NA | 2 | blue |
| ENSBTAG00000023523 | GKAP1 | 2 | blue |
| ENSBTAG00000023614 | NUDT19 | 2 | blue |
| ENSBTAG00000023632 | FBXL8 | 2 | blue |
| ENSBTAG00000023652 | PROS1 | 2 | blue |
| ENSBTAG00000023730 | TUBB3 | 2 | blue |
| ENSBTAG00000023776 | NA | 2 | blue |
| ENSBTAG00000023814 | ECT2 | 2 | blue |
| ENSBTAG00000023831 | ELP4 | 2 | blue |
| ENSBTAG00000023843 | RALGPS1 | 2 | blue |
| ENSBTAG00000023860 | NA | 2 | blue |
| ENSBTAG00000023947 | AVPI1 | 2 | blue |
| ENSBTAG00000024042 | MORN2 | 2 | blue |
| ENSBTAG00000024096 | LSM10 | 2 | blue |
| ENSBTAG00000024157 | DYNC2I1 | 2 | blue |
| ENSBTAG00000024162 | SLC19A1 | 2 | blue |
| ENSBTAG00000024240 | ACADM | 2 | blue |
| ENSBTAG00000024291 | GARIN1A | 2 | blue |
| ENSBTAG00000024378 | MRPS21 | 2 | blue |
| ENSBTAG00000024426 | PPP1R9A | 2 | blue |
| ENSBTAG00000024431 | PEX1 | 2 | blue |
| ENSBTAG00000024578 | NA | 2 | blue |
| ENSBTAG00000024585 | NA | 2 | blue |
| ENSBTAG00000024623 | LOC782120 | 2 | blue |
| ENSBTAG00000024708 | CABIN1 | 2 | blue |
| ENSBTAG00000024803 | ENDOD1 | 2 | blue |
| ENSBTAG00000024878 | ANKRD31 | 2 | blue |
| ENSBTAG00000024884 | CCNF | 2 | blue |
| ENSBTAG00000024958 | FAM214A | 2 | blue |
| ENSBTAG00000024984 | FSBP | 2 | blue |
| ENSBTAG00000025028 | NA | 2 | blue |
| ENSBTAG00000025101 | ASGR2 | 2 | blue |
| ENSBTAG00000025126 | NA | 2 | blue |
| ENSBTAG00000025140 | DOC2B | 2 | blue |
| ENSBTAG00000025182 | FBXL21 | 2 | blue |
| ENSBTAG00000025274 | NA | 2 | blue |
| ENSBTAG00000025458 | DPF3 | 2 | blue |
| ENSBTAG00000025477 | KIF3A | 2 | blue |
| ENSBTAG00000025496 | SORD | 2 | blue |
| ENSBTAG00000025526 | MDC1 | 2 | blue |

| | | | |
|---|---|---|---|
| ENSBTAG00000025540 | ALKBH7 | 2 | blue |
| ENSBTAG00000025622 | DEF6 | 2 | blue |
| ENSBTAG00000025659 | ZNF618 | 2 | blue |
| ENSBTAG00000025756 | LOC528919 | 2 | blue |
| ENSBTAG00000025778 | EMC9 | 2 | blue |
| ENSBTAG00000025809 | ABHD8 | 2 | blue |
| ENSBTAG00000025822 | CALML4 | 2 | blue |
| ENSBTAG00000025830 | R3HCC1 | 2 | blue |
| ENSBTAG00000025931 | NEU3 | 2 | blue |
| ENSBTAG00000026008 | METTL5 | 2 | blue |
| ENSBTAG00000026114 | UBE2F | 2 | blue |
| ENSBTAG00000026180 | NA | 2 | blue |
| ENSBTAG00000026181 | UGT1A6 | 2 | blue |
| ENSBTAG00000026232 | NA | 2 | blue |
| ENSBTAG00000026248 | POP7 | 2 | blue |
| ENSBTAG00000026283 | ASPH | 2 | blue |
| ENSBTAG00000026307 | ZNF629 | 2 | blue |
| ENSBTAG00000026371 | SNX29 | 2 | blue |
| ENSBTAG00000026429 | DECR2 | 2 | blue |
| ENSBTAG00000026527 | NA | 2 | blue |
| ENSBTAG00000026604 | LYRM2 | 2 | blue |
| ENSBTAG00000026624 | ETFRF1 | 2 | blue |
| ENSBTAG00000026657 | IFT27 | 2 | blue |
| ENSBTAG00000026673 | KPNA5 | 2 | blue |
| ENSBTAG00000026758 | LOC788425 | 2 | blue |
| ENSBTAG00000026812 | AOX2 | 2 | blue |
| ENSBTAG00000026825 | TMEM37 | 2 | blue |
| ENSBTAG00000026842 | ZRANB3 | 2 | blue |
| ENSBTAG00000026882 | NA | 2 | blue |
| ENSBTAG00000026915 | NEK1 | 2 | blue |
| ENSBTAG00000026919 | PXDC1 | 2 | blue |
| ENSBTAG00000026936 | ZGRF1 | 2 | blue |
| ENSBTAG00000026962 | XPOT | 2 | blue |
| ENSBTAG00000027049 | SDHAF4 | 2 | blue |
| ENSBTAG00000027059 | ARPIN | 2 | blue |
| ENSBTAG00000027181 | LAMA3 | 2 | blue |
| ENSBTAG00000027321 | CCDC18 | 2 | blue |
| ENSBTAG00000027412 | NA | 2 | blue |
| ENSBTAG00000027446 | RSU1 | 2 | blue |
| ENSBTAG00000027464 | FGFRL1 | 2 | blue |
| ENSBTAG00000027684 | FOLR2 | 2 | blue |
| ENSBTAG00000027694 | TTC30A | 2 | blue |
| ENSBTAG00000027764 | EID1 | 2 | blue |
| ENSBTAG00000027843 | TCEAL8 | 2 | blue |
| ENSBTAG00000027875 | CCDC141 | 2 | blue |
| ENSBTAG00000027879 | MPC1 | 2 | blue |
| ENSBTAG00000029237 | NA | 2 | blue |

| | | | |
|---|---|---|---|
| ENSBTAG00000029683 | LOC112449179 | 2 | blue |
| ENSBTAG00000030179 | MAPK8IP2 | 2 | blue |
| ENSBTAG00000030198 | TMEM232 | 2 | blue |
| ENSBTAG00000030246 | ENTPD8 | 2 | blue |
| ENSBTAG00000030255 | PDXP | 2 | blue |
| ENSBTAG00000030282 | ZBED8 | 2 | blue |
| ENSBTAG00000030317 | PPP1R26 | 2 | blue |
| ENSBTAG00000030347 | TUBG2 | 2 | blue |
| ENSBTAG00000030432 | TTC8 | 2 | blue |
| ENSBTAG00000030434 | FUCA1 | 2 | blue |
| ENSBTAG00000030435 | PNRC2 | 2 | blue |
| ENSBTAG00000030456 | SPATA7 | 2 | blue |
| ENSBTAG00000030507 | LOC783993 | 2 | blue |
| ENSBTAG00000030563 | PRRG2 | 2 | blue |
| ENSBTAG00000030566 | GLE1 | 2 | blue |
| ENSBTAG00000030578 | PTOV1 | 2 | blue |
| ENSBTAG00000030608 | NA | 2 | blue |
| ENSBTAG00000030632 | ALG10 | 2 | blue |
| ENSBTAG00000030650 | TST | 2 | blue |
| ENSBTAG00000030667 | ODAD1 | 2 | blue |
| ENSBTAG00000030706 | ALG6 | 2 | blue |
| ENSBTAG00000030710 | ITGB3BP | 2 | blue |
| ENSBTAG00000030769 | KPTN | 2 | blue |
| ENSBTAG00000030775 | PCBP3 | 2 | blue |
| ENSBTAG00000030820 | NT5M | 2 | blue |
| ENSBTAG00000030824 | L3HYPDH | 2 | blue |
| ENSBTAG00000030864 | FAM228B | 2 | blue |
| ENSBTAG00000030898 | MS4A13 | 2 | blue |
| ENSBTAG00000030939 | ZNF575 | 2 | blue |
| ENSBTAG00000030962 | EEFSEC | 2 | blue |
| ENSBTAG00000031001 | MEGF8 | 2 | blue |
| ENSBTAG00000031010 | ACAD11 | 2 | blue |
| ENSBTAG00000031012 | PIGK | 2 | blue |
| ENSBTAG00000031014 | LDAH | 2 | blue |
| ENSBTAG00000031044 | MIGA1 | 2 | blue |
| ENSBTAG00000031059 | LRRC61 | 2 | blue |
| ENSBTAG00000031069 | SNX24 | 2 | blue |
| ENSBTAG00000031071 | ZNF624 | 2 | blue |
| ENSBTAG00000031076 | NA | 2 | blue |
| ENSBTAG00000031178 | SLC9A9 | 2 | blue |
| ENSBTAG00000031185 | ATRAID | 2 | blue |
| ENSBTAG00000031242 | NA | 2 | blue |
| ENSBTAG00000031249 | PSMG4 | 2 | blue |
| ENSBTAG00000031261 | REPS2 | 2 | blue |
| ENSBTAG00000031295 | HSDL2 | 2 | blue |
| ENSBTAG00000031348 | CCR9 | 2 | blue |
| ENSBTAG00000031352 | ZNF554 | 2 | blue |

| | | | |
|---|---|---|---|
| ENSBTAG00000031363 | TLE5 | 2 | blue |
| ENSBTAG00000031433 | TEX9 | 2 | blue |
| ENSBTAG00000031500 | SHMT2 | 2 | blue |
| ENSBTAG00000031503 | NDUFA4L2 | 2 | blue |
| ENSBTAG00000031509 | PFAS | 2 | blue |
| ENSBTAG00000031553 | SLC49A3 | 2 | blue |
| ENSBTAG00000031561 | RSRC1 | 2 | blue |
| ENSBTAG00000031569 | GOLM1 | 2 | blue |
| ENSBTAG00000031572 | IFT80 | 2 | blue |
| ENSBTAG00000031575 | VAV3 | 2 | blue |
| ENSBTAG00000031579 | SGO2 | 2 | blue |
| ENSBTAG00000031595 | FTCDNL1 | 2 | blue |
| ENSBTAG00000031598 | NIPSNAP2 | 2 | blue |
| ENSBTAG00000031655 | NA | 2 | blue |
| ENSBTAG00000031682 | NA | 2 | blue |
| ENSBTAG00000031697 | KLHDC1 | 2 | blue |
| ENSBTAG00000031715 | PCMTD2 | 2 | blue |
| ENSBTAG00000031725 | TNFSF12 | 2 | blue |
| ENSBTAG00000031749 | SLC35E2 | 2 | blue |
| ENSBTAG00000031752 | TMEM256 | 2 | blue |
| ENSBTAG00000031777 | GDPD5 | 2 | blue |
| ENSBTAG00000031800 | PPDPF | 2 | blue |
| ENSBTAG00000031806 | ZNF48 | 2 | blue |
| ENSBTAG00000031863 | NA | 2 | blue |
| ENSBTAG00000031869 | ZSCAN26 | 2 | blue |
| ENSBTAG00000031873 | ZSCAN16 | 2 | blue |
| ENSBTAG00000031916 | NA | 2 | blue |
| ENSBTAG00000031941 | PEPD | 2 | blue |
| ENSBTAG00000032018 | ZNF19 | 2 | blue |
| ENSBTAG00000032047 | LOC100296211 | 2 | blue |
| ENSBTAG00000032051 | SULT1C4 | 2 | blue |
| ENSBTAG00000032121 | SUGCT | 2 | blue |
| ENSBTAG00000032122 | CD19 | 2 | blue |
| ENSBTAG00000032140 | SBK1 | 2 | blue |
| ENSBTAG00000032166 | GTPBP10 | 2 | blue |
| ENSBTAG00000032200 | NA | 2 | blue |
| ENSBTAG00000032331 | SLC48A1 | 2 | blue |
| ENSBTAG00000032364 | NA | 2 | blue |
| ENSBTAG00000032425 | NA | 2 | blue |
| ENSBTAG00000032455 | H2BC18 | 2 | blue |
| ENSBTAG00000032515 | PLA2R1 | 2 | blue |
| ENSBTAG00000032558 | TTC7A | 2 | blue |
| ENSBTAG00000032609 | NA | 2 | blue |
| ENSBTAG00000032680 | TMEM63B | 2 | blue |
| ENSBTAG00000032719 | TFRC | 2 | blue |
| ENSBTAG00000032859 | NA | 2 | blue |
| ENSBTAG00000032893 | NA | 2 | blue |

| | | | |
|---|---|---|---|
| ENSBTAG00000032899 | NA | 2 | blue |
| ENSBTAG00000032933 | TTC23L | 2 | blue |
| ENSBTAG00000032961 | IER3IP1 | 2 | blue |
| ENSBTAG00000033089 | HACD1 | 2 | blue |
| ENSBTAG00000033160 | FAM114A1 | 2 | blue |
| ENSBTAG00000033174 | TFAP4 | 2 | blue |
| ENSBTAG00000033180 | SMYD3 | 2 | blue |
| ENSBTAG00000033186 | OXCT1 | 2 | blue |
| ENSBTAG00000033214 | TBC1D19 | 2 | blue |
| ENSBTAG00000033221 | CCDC152 | 2 | blue |
| ENSBTAG00000033222 | CALR3 | 2 | blue |
| ENSBTAG00000033248 | CDH3 | 2 | blue |
| ENSBTAG00000033267 | LIN9 | 2 | blue |
| ENSBTAG00000033313 | SLC38A9 | 2 | blue |
| ENSBTAG00000033333 | CDK10 | 2 | blue |
| ENSBTAG00000033345 | STAR | 2 | blue |
| ENSBTAG00000033351 | PACRGL | 2 | blue |
| ENSBTAG00000033412 | NA | 2 | blue |
| ENSBTAG00000033429 | FAM229B | 2 | blue |
| ENSBTAG00000033464 | SPA17 | 2 | blue |
| ENSBTAG00000033486 | C17H4orf46 | 2 | blue |
| ENSBTAG00000033563 | NA | 2 | blue |
| ENSBTAG00000033603 | IP6K3 | 2 | blue |
| ENSBTAG00000033677 | NA | 2 | blue |
| ENSBTAG00000033679 | HLCS | 2 | blue |
| ENSBTAG00000033680 | NA | 2 | blue |
| ENSBTAG00000033685 | CCNJ | 2 | blue |
| ENSBTAG00000033727 | RBPMS | 2 | blue |
| ENSBTAG00000033740 | NA | 2 | blue |
| ENSBTAG00000033747 | SIL1 | 2 | blue |
| ENSBTAG00000033801 | VRK2 | 2 | blue |
| ENSBTAG00000033806 | LOC613401 | 2 | blue |
| ENSBTAG00000034090 | NA | 2 | blue |
| ENSBTAG00000034182 | LOC782966 | 2 | blue |
| ENSBTAG00000034222 | CAB39L | 2 | blue |
| ENSBTAG00000034295 | LAG3 | 2 | blue |
| ENSBTAG00000034347 | RBM11 | 2 | blue |
| ENSBTAG00000034435 | NKD2 | 2 | blue |
| ENSBTAG00000034442 | LNP1 | 2 | blue |
| ENSBTAG00000034785 | DNAJC15 | 2 | blue |
| ENSBTAG00000034827 | PDGFD | 2 | blue |
| ENSBTAG00000034871 | NA | 2 | blue |
| ENSBTAG00000034883 | CCR4 | 2 | blue |
| ENSBTAG00000034963 | MKS1 | 2 | blue |
| ENSBTAG00000034998 | SIMC1 | 2 | blue |
| ENSBTAG00000035175 | DONSON | 2 | blue |
| ENSBTAG00000035244 | ANKRD39 | 2 | blue |

| | | | |
|---|---|---|---|
| ENSBTAG00000035247 | MCEE | 2 | blue |
| ENSBTAG00000035572 | NA | 2 | blue |
| ENSBTAG00000035615 | UPF3B | 2 | blue |
| ENSBTAG00000035726 | NA | 2 | blue |
| ENSBTAG00000035782 | METTL22 | 2 | blue |
| ENSBTAG00000035844 | PLAAT1 | 2 | blue |
| ENSBTAG00000035958 | RHOBTB2 | 2 | blue |
| ENSBTAG00000036087 | ARMC2 | 2 | blue |
| ENSBTAG00000036101 | KYAT1 | 2 | blue |
| ENSBTAG00000036260 | LPXN | 2 | blue |
| ENSBTAG00000036262 | GBE1 | 2 | blue |
| ENSBTAG00000037375 | LOC512684 | 2 | blue |
| ENSBTAG00000037377 | ABHD14B | 2 | blue |
| ENSBTAG00000037440 | NA | 2 | blue |
| ENSBTAG00000037456 | AHDC1 | 2 | blue |
| ENSBTAG00000037489 | CRCP | 2 | blue |
| ENSBTAG00000037549 | NA | 2 | blue |
| ENSBTAG00000037565 | LOC788142 | 2 | blue |
| ENSBTAG00000037566 | ZNF500 | 2 | blue |
| ENSBTAG00000037571 | EBAG9 | 2 | blue |
| ENSBTAG00000037661 | NA | 2 | blue |
| ENSBTAG00000037673 | GSTM1 | 2 | blue |
| ENSBTAG00000037726 | PLCD1 | 2 | blue |
| ENSBTAG00000037729 | CERS1 | 2 | blue |
| ENSBTAG00000037768 | MMP3 | 2 | blue |
| ENSBTAG00000037775 | LOC100141266 | 2 | blue |
| ENSBTAG00000037803 | ZNF197 | 2 | blue |
| ENSBTAG00000037819 | SFT2D2 | 2 | blue |
| ENSBTAG00000037882 | NA | 2 | blue |
| ENSBTAG00000037888 | LYPD5 | 2 | blue |
| ENSBTAG00000037971 | NA | 2 | blue |
| ENSBTAG00000037981 | LOC530973 | 2 | blue |
| ENSBTAG00000038011 | SH3RF3 | 2 | blue |
| ENSBTAG00000038025 | NA | 2 | blue |
| ENSBTAG00000038048 | MRC1 | 2 | blue |
| ENSBTAG00000038050 | ZNF420 | 2 | blue |
| ENSBTAG00000038051 | LOC532875 | 2 | blue |
| ENSBTAG00000038063 | LIPJ | 2 | blue |
| ENSBTAG00000038079 | C16H1orf21 | 2 | blue |
| ENSBTAG00000038088 | NA | 2 | blue |
| ENSBTAG00000038111 | NA | 2 | blue |
| ENSBTAG00000038117 | ATG10 | 2 | blue |
| ENSBTAG00000038131 | NA | 2 | blue |
| ENSBTAG00000038134 | NA | 2 | blue |
| ENSBTAG00000038156 | DYNLT3 | 2 | blue |
| ENSBTAG00000038178 | ZNF25 | 2 | blue |
| ENSBTAG00000038181 | FOCAD | 2 | blue |

| | | | |
|---|---|---|---|
| ENSBTAG00000038251 | NAA16 | 2 | blue |
| ENSBTAG00000038262 | NA | 2 | blue |
| ENSBTAG00000038283 | TMEM243 | 2 | blue |
| ENSBTAG00000038284 | NA | 2 | blue |
| ENSBTAG00000038306 | CBX2 | 2 | blue |
| ENSBTAG00000038366 | CD1B5 | 2 | blue |
| ENSBTAG00000038407 | NA | 2 | blue |
| ENSBTAG00000038415 | SLC6A12 | 2 | blue |
| ENSBTAG00000038422 | NA | 2 | blue |
| ENSBTAG00000038426 | CD1B | 2 | blue |
| ENSBTAG00000038430 | LOC788801 | 2 | blue |
| ENSBTAG00000038439 | NA | 2 | blue |
| ENSBTAG00000038477 | GGCX | 2 | blue |
| ENSBTAG00000038480 | FPGT | 2 | blue |
| ENSBTAG00000038540 | LOC785216 | 2 | blue |
| ENSBTAG00000038604 | NA | 2 | blue |
| ENSBTAG00000038620 | CIR1 | 2 | blue |
| ENSBTAG00000038680 | LOC538435 | 2 | blue |
| ENSBTAG00000038682 | FAM83H | 2 | blue |
| ENSBTAG00000038706 | MT1E | 2 | blue |
| ENSBTAG00000038715 | NA | 2 | blue |
| ENSBTAG00000038756 | GLB1L3 | 2 | blue |
| ENSBTAG00000038794 | TMEM245 | 2 | blue |
| ENSBTAG00000038845 | TYSND1 | 2 | blue |
| ENSBTAG00000038869 | ZNF383 | 2 | blue |
| ENSBTAG00000038878 | AOX4 | 2 | blue |
| ENSBTAG00000038888 | ZBTB9 | 2 | blue |
| ENSBTAG00000038889 | RILP | 2 | blue |
| ENSBTAG00000038913 | NA | 2 | blue |
| ENSBTAG00000038931 | TSTD3 | 2 | blue |
| ENSBTAG00000038945 | PADI6 | 2 | blue |
| ENSBTAG00000038949 | MRPL30 | 2 | blue |
| ENSBTAG00000038951 | NA | 2 | blue |
| ENSBTAG00000039023 | NA | 2 | blue |
| ENSBTAG00000039075 | ZNF527 | 2 | blue |
| ENSBTAG00000039090 | MAGEH1 | 2 | blue |
| ENSBTAG00000039117 | TOGARAM1 | 2 | blue |
| ENSBTAG00000039119 | NA | 2 | blue |
| ENSBTAG00000039129 | LOC510798 | 2 | blue |
| ENSBTAG00000039132 | NA | 2 | blue |
| ENSBTAG00000039161 | ALDH3A2 | 2 | blue |
| ENSBTAG00000039196 | LOC510860 | 2 | blue |
| ENSBTAG00000039231 | NA | 2 | blue |
| ENSBTAG00000039242 | ZNF10 | 2 | blue |
| ENSBTAG00000039260 | RPTN | 2 | blue |
| ENSBTAG00000039287 | ZFP69 | 2 | blue |
| ENSBTAG00000039316 | NA | 2 | blue |

| | | | |
|---|---|---|---|
| ENSBTAG00000039341 | LOC100296121 | 2 | blue |
| ENSBTAG00000039366 | LOC515418 | 2 | blue |
| ENSBTAG00000039406 | LOC613570 | 2 | blue |
| ENSBTAG00000039426 | DPEP2 | 2 | blue |
| ENSBTAG00000039531 | LCN12 | 2 | blue |
| ENSBTAG00000039552 | PLK4 | 2 | blue |
| ENSBTAG00000039582 | NDUFC1 | 2 | blue |
| ENSBTAG00000039593 | FAM98C | 2 | blue |
| ENSBTAG00000039599 | HOXB4 | 2 | blue |
| ENSBTAG00000039664 | LOC101906101 | 2 | blue |
| ENSBTAG00000039671 | LOC539876 | 2 | blue |
| ENSBTAG00000039682 | MTUS1 | 2 | blue |
| ENSBTAG00000039711 | ZFP62 | 2 | blue |
| ENSBTAG00000039738 | ADORA3 | 2 | blue |
| ENSBTAG00000039875 | NA | 2 | blue |
| ENSBTAG00000039886 | LOC509184 | 2 | blue |
| ENSBTAG00000039888 | MAGED2 | 2 | blue |
| ENSBTAG00000039922 | ARAP2 | 2 | blue |
| ENSBTAG00000039954 | COPZ2 | 2 | blue |
| ENSBTAG00000039962 | DCTPP1 | 2 | blue |
| ENSBTAG00000039967 | KRT78 | 2 | blue |
| ENSBTAG00000039971 | LOC514658 | 2 | blue |
| ENSBTAG00000039992 | TUBA1C | 2 | blue |
| ENSBTAG00000040042 | GRIN2C | 2 | blue |
| ENSBTAG00000040043 | NA | 2 | blue |
| ENSBTAG00000040055 | CAB39 | 2 | blue |
| ENSBTAG00000040067 | HIPK4 | 2 | blue |
| ENSBTAG00000040072 | ZNF140 | 2 | blue |
| ENSBTAG00000040078 | FLVCR2 | 2 | blue |
| ENSBTAG00000040108 | NA | 2 | blue |
| ENSBTAG00000040116 | H1-10 | 2 | blue |
| ENSBTAG00000040161 | TM4SF19 | 2 | blue |
| ENSBTAG00000040169 | LOC509810 | 2 | blue |
| ENSBTAG00000040206 | ZNF770 | 2 | blue |
| ENSBTAG00000040304 | LOC616094 | 2 | blue |
| ENSBTAG00000040333 | QDPR | 2 | blue |
| ENSBTAG00000040338 | OBSL1 | 2 | blue |
| ENSBTAG00000040398 | CRACD | 2 | blue |
| ENSBTAG00000040427 | ARHGAP19 | 2 | blue |
| ENSBTAG00000040435 | NA | 2 | blue |
| ENSBTAG00000040473 | NA | 2 | blue |
| ENSBTAG00000040477 | CHD5 | 2 | blue |
| ENSBTAG00000040518 | LOC112443216 | 2 | blue |
| ENSBTAG00000040564 | NA | 2 | blue |
| ENSBTAG00000040569 | NLRX1 | 2 | blue |
| ENSBTAG00000043547 | NA | 2 | blue |
| ENSBTAG00000043557 | NA | 2 | blue |

| | | | |
|---|---|---|---|
| ENSBTAG00000043573 | NA | 2 | blue |
| ENSBTAG00000043580 | NA | 2 | blue |
| ENSBTAG00000043951 | IMPA2 | 2 | blue |
| ENSBTAG00000043981 | SBF2 | 2 | blue |
| ENSBTAG00000044038 | TEN1 | 2 | blue |
| ENSBTAG00000044044 | CLOCK | 2 | blue |
| ENSBTAG00000044053 | SLC17A5 | 2 | blue |
| ENSBTAG00000044058 | MFSD8 | 2 | blue |
| ENSBTAG00000044065 | PITPNC1 | 2 | blue |
| ENSBTAG00000044077 | POLR3G | 2 | blue |
| ENSBTAG00000044079 | SMIM4 | 2 | blue |
| ENSBTAG00000044087 | NA | 2 | blue |
| ENSBTAG00000044106 | SPIDR | 2 | blue |
| ENSBTAG00000044112 | MSRB2 | 2 | blue |
| ENSBTAG00000044121 | NUBPL | 2 | blue |
| ENSBTAG00000044126 | SNTB1 | 2 | blue |
| ENSBTAG00000044160 | MCCC2 | 2 | blue |
| ENSBTAG00000044172 | LMLN | 2 | blue |
| ENSBTAG00000044178 | ZMPSTE24 | 2 | blue |
| ENSBTAG00000045523 | LOC509118 | 2 | blue |
| ENSBTAG00000045538 | NA | 2 | blue |
| ENSBTAG00000045581 | NA | 2 | blue |
| ENSBTAG00000045622 | NA | 2 | blue |
| ENSBTAG00000045630 | NA | 2 | blue |
| ENSBTAG00000045658 | NA | 2 | blue |
| ENSBTAG00000045703 | COX15 | 2 | blue |
| ENSBTAG00000045728 | NA | 2 | blue |
| ENSBTAG00000045754 | SMIM1 | 2 | blue |
| ENSBTAG00000045794 | NA | 2 | blue |
| ENSBTAG00000045836 | NA | 2 | blue |
| ENSBTAG00000045857 | MCRIP2 | 2 | blue |
| ENSBTAG00000045868 | NA | 2 | blue |
| ENSBTAG00000045888 | NA | 2 | blue |
| ENSBTAG00000045909 | FAM174A | 2 | blue |
| ENSBTAG00000045955 | TSSK1B | 2 | blue |
| ENSBTAG00000045971 | NA | 2 | blue |
| ENSBTAG00000045981 | DIRAS1 | 2 | blue |
| ENSBTAG00000045987 | NA | 2 | blue |
| ENSBTAG00000046003 | NA | 2 | blue |
| ENSBTAG00000046049 | NA | 2 | blue |
| ENSBTAG00000046052 | ANKRD63 | 2 | blue |
| ENSBTAG00000046088 | LOC101902204 | 2 | blue |
| ENSBTAG00000046155 | RGN | 2 | blue |
| ENSBTAG00000046156 | NA | 2 | blue |
| ENSBTAG00000046162 | ZNF385C | 2 | blue |
| ENSBTAG00000046189 | RNASE13 | 2 | blue |
| ENSBTAG00000046192 | AK6 | 2 | blue |

| | | | |
|---|---|---|---|
| ENSBTAG00000046218 | KLF11 | 2 | blue |
| ENSBTAG00000046263 | ADAMTSL5 | 2 | blue |
| ENSBTAG00000046286 | CCNB3 | 2 | blue |
| ENSBTAG00000046321 | NA | 2 | blue |
| ENSBTAG00000046325 | NA | 2 | blue |
| ENSBTAG00000046337 | TUBB6 | 2 | blue |
| ENSBTAG00000046338 | NA | 2 | blue |
| ENSBTAG00000046339 | VASN | 2 | blue |
| ENSBTAG00000046357 | SLC29A3 | 2 | blue |
| ENSBTAG00000046362 | BBIP1 | 2 | blue |
| ENSBTAG00000046452 | NA | 2 | blue |
| ENSBTAG00000046502 | ACBD6 | 2 | blue |
| ENSBTAG00000046503 | NCOA7 | 2 | blue |
| ENSBTAG00000046547 | CIDEA | 2 | blue |
| ENSBTAG00000046552 | GGACT | 2 | blue |
| ENSBTAG00000046569 | CISD3 | 2 | blue |
| ENSBTAG00000046570 | NA | 2 | blue |
| ENSBTAG00000046573 | PGBD1 | 2 | blue |
| ENSBTAG00000046633 | NA | 2 | blue |
| ENSBTAG00000046668 | ANG2 | 2 | blue |
| ENSBTAG00000046671 | WFS1 | 2 | blue |
| ENSBTAG00000046771 | FNDC7 | 2 | blue |
| ENSBTAG00000046794 | NA | 2 | blue |
| ENSBTAG00000046803 | GAS1 | 2 | blue |
| ENSBTAG00000046814 | ACOT2 | 2 | blue |
| ENSBTAG00000046837 | ZNF358 | 2 | blue |
| ENSBTAG00000046839 | NA | 2 | blue |
| ENSBTAG00000046840 | DEXI | 2 | blue |
| ENSBTAG00000046871 | NA | 2 | blue |
| ENSBTAG00000046886 | NA | 2 | blue |
| ENSBTAG00000046901 | NA | 2 | blue |
| ENSBTAG00000046922 | TWIST1 | 2 | blue |
| ENSBTAG00000046926 | NA | 2 | blue |
| ENSBTAG00000046979 | LGMN | 2 | blue |
| ENSBTAG00000046980 | KLRF2 | 2 | blue |
| ENSBTAG00000047002 | NA | 2 | blue |
| ENSBTAG00000047009 | ANKRD66 | 2 | blue |
| ENSBTAG00000047053 | NA | 2 | blue |
| ENSBTAG00000047098 | NA | 2 | blue |
| ENSBTAG00000047119 | NA | 2 | blue |
| ENSBTAG00000047135 | GORASP1 | 2 | blue |
| ENSBTAG00000047150 | NA | 2 | blue |
| ENSBTAG00000047196 | NA | 2 | blue |
| ENSBTAG00000047210 | NA | 2 | blue |
| ENSBTAG00000047214 | TRAPPC1 | 2 | blue |
| ENSBTAG00000047225 | BCL2L14 | 2 | blue |
| ENSBTAG00000047226 | NA | 2 | blue |

| | | | |
|---|---|---|---|
| ENSBTAG00000047227 | NA | 2 | blue |
| ENSBTAG00000047254 | ABCA2 | 2 | blue |
| ENSBTAG00000047277 | NA | 2 | blue |
| ENSBTAG00000047299 | DGCR6L | 2 | blue |
| ENSBTAG00000047302 | LOC514978 | 2 | blue |
| ENSBTAG00000047330 | FABP5 | 2 | blue |
| ENSBTAG00000047343 | NA | 2 | blue |
| ENSBTAG00000047344 | OR2AT2C | 2 | blue |
| ENSBTAG00000047357 | RNF208 | 2 | blue |
| ENSBTAG00000047380 | CALCB | 2 | blue |
| ENSBTAG00000047412 | TNFSF18 | 2 | blue |
| ENSBTAG00000047416 | HEPH | 2 | blue |
| ENSBTAG00000047450 | SFR1 | 2 | blue |
| ENSBTAG00000047532 | TRIQK | 2 | blue |
| ENSBTAG00000047545 | RTL5 | 2 | blue |
| ENSBTAG00000047567 | VEGFB | 2 | blue |
| ENSBTAG00000047572 | ZNF789 | 2 | blue |
| ENSBTAG00000047579 | NA | 2 | blue |
| ENSBTAG00000047591 | C23H6orf141 | 2 | blue |
| ENSBTAG00000047608 | MRPS22 | 2 | blue |
| ENSBTAG00000047609 | NA | 2 | blue |
| ENSBTAG00000047621 | NA | 2 | blue |
| ENSBTAG00000047646 | NA | 2 | blue |
| ENSBTAG00000047648 | SYT2 | 2 | blue |
| ENSBTAG00000047667 | NA | 2 | blue |
| ENSBTAG00000047673 | THUMPD1 | 2 | blue |
| ENSBTAG00000047734 | RUSF1 | 2 | blue |
| ENSBTAG00000047752 | OTUD1 | 2 | blue |
| ENSBTAG00000047756 | CNTROB | 2 | blue |
| ENSBTAG00000047788 | NA | 2 | blue |
| ENSBTAG00000047809 | NA | 2 | blue |
| ENSBTAG00000047957 | NA | 2 | blue |
| ENSBTAG00000047958 | DPP7 | 2 | blue |
| ENSBTAG00000047998 | COL5A1 | 2 | blue |
| ENSBTAG00000048010 | NUDT14 | 2 | blue |
| ENSBTAG00000048041 | ACTR1B | 2 | blue |
| ENSBTAG00000048042 | NA | 2 | blue |
| ENSBTAG00000048049 | NA | 2 | blue |
| ENSBTAG00000048054 | UAP1L1 | 2 | blue |
| ENSBTAG00000048114 | LOC100848331 | 2 | blue |
| ENSBTAG00000048140 | DNAAF5 | 2 | blue |
| ENSBTAG00000048152 | TUBG1 | 2 | blue |
| ENSBTAG00000048234 | NA | 2 | blue |
| ENSBTAG00000048265 | NA | 2 | blue |
| ENSBTAG00000000031 | TRPV4 | 3 | brown |
| ENSBTAG00000000042 | PYCR1 | 3 | brown |
| ENSBTAG00000000074 | NFIA | 3 | brown |

| | | | |
|---|---|---|---|
| ENSBTAG00000000078 | GLIPR2 | 3 | brown |
| ENSBTAG00000000120 | USP43 | 3 | brown |
| ENSBTAG00000000146 | FARP1 | 3 | brown |
| ENSBTAG00000000160 | CBS | 3 | brown |
| ENSBTAG00000000182 | SPINT2 | 3 | brown |
| ENSBTAG00000000185 | SLC6A14 | 3 | brown |
| ENSBTAG00000000211 | SHROOM2 | 3 | brown |
| ENSBTAG00000000220 | NA | 3 | brown |
| ENSBTAG00000000231 | BMPR1A | 3 | brown |
| ENSBTAG00000000243 | HEBP2 | 3 | brown |
| ENSBTAG00000000245 | NHSL1 | 3 | brown |
| ENSBTAG00000000253 | PTPRF | 3 | brown |
| ENSBTAG00000000305 | LMO4 | 3 | brown |
| ENSBTAG00000000394 | BACE2 | 3 | brown |
| ENSBTAG00000000418 | TOX3 | 3 | brown |
| ENSBTAG00000000434 | CRYAB | 3 | brown |
| ENSBTAG00000000440 | HSPB2 | 3 | brown |
| ENSBTAG00000000448 | BDH1 | 3 | brown |
| ENSBTAG00000000460 | SYTL2 | 3 | brown |
| ENSBTAG00000000462 | CCDC102A | 3 | brown |
| ENSBTAG00000000490 | RTKN | 3 | brown |
| ENSBTAG00000000496 | SLC12A8 | 3 | brown |
| ENSBTAG00000000561 | OCLN | 3 | brown |
| ENSBTAG00000000575 | TNC | 3 | brown |
| ENSBTAG00000000576 | ADGRG2 | 3 | brown |
| ENSBTAG00000000582 | LY6G6E | 3 | brown |
| ENSBTAG00000000585 | LY6G6C | 3 | brown |
| ENSBTAG00000000598 | CST3 | 3 | brown |
| ENSBTAG00000000603 | JAM2 | 3 | brown |
| ENSBTAG00000000628 | NA | 3 | brown |
| ENSBTAG00000000634 | MLPH | 3 | brown |
| ENSBTAG00000000655 | MIPOL1 | 3 | brown |
| ENSBTAG00000000664 | SLC22A15 | 3 | brown |
| ENSBTAG00000000667 | APOL3 | 3 | brown |
| ENSBTAG00000000700 | PARVA | 3 | brown |
| ENSBTAG00000000706 | ADAMTS1 | 3 | brown |
| ENSBTAG00000000720 | CTSL | 3 | brown |
| ENSBTAG00000000738 | DAPK1 | 3 | brown |
| ENSBTAG00000000742 | AMOTL2 | 3 | brown |
| ENSBTAG00000000782 | KDR | 3 | brown |
| ENSBTAG00000000793 | LAMC2 | 3 | brown |
| ENSBTAG00000000809 | IRX1 | 3 | brown |
| ENSBTAG00000000815 | EPHA2 | 3 | brown |
| ENSBTAG00000000817 | SYNJ2 | 3 | brown |
| ENSBTAG00000000828 | CAPN6 | 3 | brown |
| ENSBTAG00000000835 | NA | 3 | brown |
| ENSBTAG00000000836 | KRT8 | 3 | brown |

| | | | |
|---|---|---|---|
| ENSBTAG00000000859 | SLC38A1 | 3 | brown |
| ENSBTAG00000000943 | ZNF286A | 3 | brown |
| ENSBTAG00000000945 | ATP2C2 | 3 | brown |
| ENSBTAG00000000951 | JAKMIP3 | 3 | brown |
| ENSBTAG00000001081 | PALLD | 3 | brown |
| ENSBTAG00000001086 | FHL2 | 3 | brown |
| ENSBTAG00000001112 | SMOX | 3 | brown |
| ENSBTAG00000001119 | ANKRD13B | 3 | brown |
| ENSBTAG00000001153 | TLE2 | 3 | brown |
| ENSBTAG00000001161 | SINHCAF | 3 | brown |
| ENSBTAG00000001186 | SH2D4A | 3 | brown |
| ENSBTAG00000001204 | JCAD | 3 | brown |
| ENSBTAG00000001207 | SERPINB8 | 3 | brown |
| ENSBTAG00000001209 | PHLDB2 | 3 | brown |
| ENSBTAG00000001233 | MPZL3 | 3 | brown |
| ENSBTAG00000001242 | ADRB1 | 3 | brown |
| ENSBTAG00000001244 | PLAT | 3 | brown |
| ENSBTAG00000001250 | TFAP2A | 3 | brown |
| ENSBTAG00000001286 | ELMO3 | 3 | brown |
| ENSBTAG00000001303 | HSPB8 | 3 | brown |
| ENSBTAG00000001335 | GHR | 3 | brown |
| ENSBTAG00000001341 | NA | 3 | brown |
| ENSBTAG00000001349 | KIF1C | 3 | brown |
| ENSBTAG00000001350 | LOC101906045 | 3 | brown |
| ENSBTAG00000001359 | B3GNT7 | 3 | brown |
| ENSBTAG00000001374 | METTL27 | 3 | brown |
| ENSBTAG00000001481 | IGSF9 | 3 | brown |
| ENSBTAG00000001517 | KRT18 | 3 | brown |
| ENSBTAG00000001523 | YES1 | 3 | brown |
| ENSBTAG00000001535 | LIPH | 3 | brown |
| ENSBTAG00000001568 | PPIC | 3 | brown |
| ENSBTAG00000001572 | NSUN7 | 3 | brown |
| ENSBTAG00000001586 | OTUD7B | 3 | brown |
| ENSBTAG00000001598 | MAP7 | 3 | brown |
| ENSBTAG00000001610 | SLC4A1 | 3 | brown |
| ENSBTAG00000001640 | EPB41L1 | 3 | brown |
| ENSBTAG00000001675 | ITPKA | 3 | brown |
| ENSBTAG00000001710 | CMTM8 | 3 | brown |
| ENSBTAG00000001793 | HSD11B1L | 3 | brown |
| ENSBTAG00000001815 | ARHGEF5 | 3 | brown |
| ENSBTAG00000001823 | STC2 | 3 | brown |
| ENSBTAG00000001912 | TRPV5 | 3 | brown |
| ENSBTAG00000001927 | ATP6V1C2 | 3 | brown |
| ENSBTAG00000001950 | RDH11 | 3 | brown |
| ENSBTAG00000001952 | PROM2 | 3 | brown |
| ENSBTAG00000001969 | CCDC39 | 3 | brown |
| ENSBTAG00000002006 | THBS1 | 3 | brown |

| | | | |
|---|---|---|---|
| ENSBTAG00000002009 | NA | 3 | brown |
| ENSBTAG00000002015 | RPS6KA6 | 3 | brown |
| ENSBTAG00000002019 | SERPINB5 | 3 | brown |
| ENSBTAG00000002029 | IGSF5 | 3 | brown |
| ENSBTAG00000002070 | KLC3 | 3 | brown |
| ENSBTAG00000002080 | CCN3 | 3 | brown |
| ENSBTAG00000002089 | NA | 3 | brown |
| ENSBTAG00000002123 | MYO3A | 3 | brown |
| ENSBTAG00000002126 | PFKFB2 | 3 | brown |
| ENSBTAG00000002178 | PPL | 3 | brown |
| ENSBTAG00000002188 | C25H16orf89 | 3 | brown |
| ENSBTAG00000002194 | MYZAP | 3 | brown |
| ENSBTAG00000002215 | GFPT2 | 3 | brown |
| ENSBTAG00000002227 | LRIG3 | 3 | brown |
| ENSBTAG00000002283 | FZD7 | 3 | brown |
| ENSBTAG00000002299 | SEL1L3 | 3 | brown |
| ENSBTAG00000002302 | CD59 | 3 | brown |
| ENSBTAG00000002341 | ETS1 | 3 | brown |
| ENSBTAG00000002361 | TMCC2 | 3 | brown |
| ENSBTAG00000002394 | TMEM44 | 3 | brown |
| ENSBTAG00000002408 | FHAD1 | 3 | brown |
| ENSBTAG00000002422 | BCL9 | 3 | brown |
| ENSBTAG00000002440 | KIF7 | 3 | brown |
| ENSBTAG00000002554 | TCAF1 | 3 | brown |
| ENSBTAG00000002561 | CCDC120 | 3 | brown |
| ENSBTAG00000002594 | ZNF436 | 3 | brown |
| ENSBTAG00000002606 | LGR4 | 3 | brown |
| ENSBTAG00000002631 | SCNN1A | 3 | brown |
| ENSBTAG00000002651 | PKP2 | 3 | brown |
| ENSBTAG00000002668 | ZNF711 | 3 | brown |
| ENSBTAG00000002670 | DEPP1 | 3 | brown |
| ENSBTAG00000002688 | ATP1B1 | 3 | brown |
| ENSBTAG00000002699 | KIT | 3 | brown |
| ENSBTAG00000002779 | GABRP | 3 | brown |
| ENSBTAG00000002788 | RAB27B | 3 | brown |
| ENSBTAG00000002835 | SMAD1 | 3 | brown |
| ENSBTAG00000002849 | IRF6 | 3 | brown |
| ENSBTAG00000002929 | IRF4 | 3 | brown |
| ENSBTAG00000002980 | GPT2 | 3 | brown |
| ENSBTAG00000003045 | BAMBI | 3 | brown |
| ENSBTAG00000003049 | MICALL2 | 3 | brown |
| ENSBTAG00000003061 | NA | 3 | brown |
| ENSBTAG00000003089 | RHPN2 | 3 | brown |
| ENSBTAG00000003171 | SHANK2 | 3 | brown |
| ENSBTAG00000003183 | NTN4 | 3 | brown |
| ENSBTAG00000003215 | KIF3B | 3 | brown |
| ENSBTAG00000003222 | ASNS | 3 | brown |

| | | | |
|---|---|---|---|
| ENSBTAG00000003231 | LIM2 | 3 | brown |
| ENSBTAG00000003240 | C8H9orf152 | 3 | brown |
| ENSBTAG00000003275 | NA | 3 | brown |
| ENSBTAG00000003291 | RIMKLB | 3 | brown |
| ENSBTAG00000003313 | TNFRSF9 | 3 | brown |
| ENSBTAG00000003329 | FST | 3 | brown |
| ENSBTAG00000003336 | TPD52L1 | 3 | brown |
| ENSBTAG00000003339 | ZBTB10 | 3 | brown |
| ENSBTAG00000003375 | TC2N | 3 | brown |
| ENSBTAG00000003444 | SYDE2 | 3 | brown |
| ENSBTAG00000003446 | EPHB4 | 3 | brown |
| ENSBTAG00000003455 | ANKRD6 | 3 | brown |
| ENSBTAG00000003457 | ATF5 | 3 | brown |
| ENSBTAG00000003474 | RAB15 | 3 | brown |
| ENSBTAG00000003505 | DCN | 3 | brown |
| ENSBTAG00000003511 | SLAIN1 | 3 | brown |
| ENSBTAG00000003581 | SETD7 | 3 | brown |
| ENSBTAG00000003651 | OTOP1 | 3 | brown |
| ENSBTAG00000003675 | ADGRL1 | 3 | brown |
| ENSBTAG00000003700 | SLC5A10 | 3 | brown |
| ENSBTAG00000003705 | FAM83G | 3 | brown |
| ENSBTAG00000003711 | EPAS1 | 3 | brown |
| ENSBTAG00000003751 | MACC1 | 3 | brown |
| ENSBTAG00000003763 | MYO5C | 3 | brown |
| ENSBTAG00000003777 | TIE1 | 3 | brown |
| ENSBTAG00000003794 | GNAO1 | 3 | brown |
| ENSBTAG00000003826 | SCN1B | 3 | brown |
| ENSBTAG00000003840 | GUCY1B1 | 3 | brown |
| ENSBTAG00000003871 | CYP2B6 | 3 | brown |
| ENSBTAG00000003894 | NDRG4 | 3 | brown |
| ENSBTAG00000003915 | LARP6 | 3 | brown |
| ENSBTAG00000003942 | PIP4K2C | 3 | brown |
| ENSBTAG00000003943 | TTC39B | 3 | brown |
| ENSBTAG00000003960 | CCP110 | 3 | brown |
| ENSBTAG00000003994 | IGFBP3 | 3 | brown |
| ENSBTAG00000004035 | SDK1 | 3 | brown |
| ENSBTAG00000004046 | DTX4 | 3 | brown |
| ENSBTAG00000004086 | SPINT1 | 3 | brown |
| ENSBTAG00000004168 | STON1 | 3 | brown |
| ENSBTAG00000004178 | ACOX2 | 3 | brown |
| ENSBTAG00000004190 | ARHGAP29 | 3 | brown |
| ENSBTAG00000004226 | SHBG | 3 | brown |
| ENSBTAG00000004237 | BTC | 3 | brown |
| ENSBTAG00000004283 | PPFIBP1 | 3 | brown |
| ENSBTAG00000004300 | LRRC8E | 3 | brown |
| ENSBTAG00000004344 | ACSL1 | 3 | brown |
| ENSBTAG00000004375 | ESRP2 | 3 | brown |

| | | | |
|---|---|---|---|
| ENSBTAG00000004381 | TACSTD2 | 3 | brown |
| ENSBTAG00000004383 | FNBP1L | 3 | brown |
| ENSBTAG00000004388 | SLITRK6 | 3 | brown |
| ENSBTAG00000004423 | ARHGAP42 | 3 | brown |
| ENSBTAG00000004440 | SLC15A1 | 3 | brown |
| ENSBTAG00000004511 | VAV2 | 3 | brown |
| ENSBTAG00000004515 | KCNK1 | 3 | brown |
| ENSBTAG00000004518 | GRHL2 | 3 | brown |
| ENSBTAG00000004557 | DHRS9 | 3 | brown |
| ENSBTAG00000004572 | RNF183 | 3 | brown |
| ENSBTAG00000004575 | FAM126A | 3 | brown |
| ENSBTAG00000004641 | PLEKHA6 | 3 | brown |
| ENSBTAG00000004645 | ADAMTSL3 | 3 | brown |
| ENSBTAG00000004653 | ADGRA3 | 3 | brown |
| ENSBTAG00000004777 | S100B | 3 | brown |
| ENSBTAG00000004792 | CHMP4C | 3 | brown |
| ENSBTAG00000004822 | SOX10 | 3 | brown |
| ENSBTAG00000004832 | KIF21A | 3 | brown |
| ENSBTAG00000004838 | IRX5 | 3 | brown |
| ENSBTAG00000004848 | OLFML2A | 3 | brown |
| ENSBTAG00000004869 | CXADR | 3 | brown |
| ENSBTAG00000004884 | SLC4A5 | 3 | brown |
| ENSBTAG00000004895 | AFAP1L2 | 3 | brown |
| ENSBTAG00000004896 | ABLIM3 | 3 | brown |
| ENSBTAG00000004905 | KRT19 | 3 | brown |
| ENSBTAG00000004948 | LRATD1 | 3 | brown |
| ENSBTAG00000004990 | NEO1 | 3 | brown |
| ENSBTAG00000005110 | CADPS2 | 3 | brown |
| ENSBTAG00000005111 | BMP2 | 3 | brown |
| ENSBTAG00000005162 | STX19 | 3 | brown |
| ENSBTAG00000005164 | GZMK | 3 | brown |
| ENSBTAG00000005186 | DBNDD2 | 3 | brown |
| ENSBTAG00000005198 | FGF1 | 3 | brown |
| ENSBTAG00000005234 | LYPD1 | 3 | brown |
| ENSBTAG00000005240 | NA | 3 | brown |
| ENSBTAG00000005278 | WEE1 | 3 | brown |
| ENSBTAG00000005313 | EPHB3 | 3 | brown |
| ENSBTAG00000005356 | DENND2B | 3 | brown |
| ENSBTAG00000005359 | TGFB2 | 3 | brown |
| ENSBTAG00000005373 | TPM1 | 3 | brown |
| ENSBTAG00000005412 | NEDD4L | 3 | brown |
| ENSBTAG00000005464 | ADCY6 | 3 | brown |
| ENSBTAG00000005475 | TCAF2 | 3 | brown |
| ENSBTAG00000005501 | COBLL1 | 3 | brown |
| ENSBTAG00000005530 | NA | 3 | brown |
| ENSBTAG00000005629 | CRYBG2 | 3 | brown |
| ENSBTAG00000005630 | VWA7 | 3 | brown |

| | | | |
|---|---|---|---|
| ENSBTAG00000005633 | ARHGEF28 | 3 | brown |
| ENSBTAG00000005675 | SLC44A4 | 3 | brown |
| ENSBTAG00000005681 | ME1 | 3 | brown |
| ENSBTAG00000005691 | FGF2 | 3 | brown |
| ENSBTAG00000005700 | MAP3K9 | 3 | brown |
| ENSBTAG00000005716 | CRABP2 | 3 | brown |
| ENSBTAG00000005738 | ATP8B1 | 3 | brown |
| ENSBTAG00000005766 | IL4I1 | 3 | brown |
| ENSBTAG00000005776 | ZNF185 | 3 | brown |
| ENSBTAG00000005850 | DLK2 | 3 | brown |
| ENSBTAG00000005929 | NA | 3 | brown |
| ENSBTAG00000005984 | DAAM1 | 3 | brown |
| ENSBTAG00000006001 | STAP2 | 3 | brown |
| ENSBTAG00000006052 | PLCD3 | 3 | brown |
| ENSBTAG00000006064 | BRME1 | 3 | brown |
| ENSBTAG00000006071 | CTTN | 3 | brown |
| ENSBTAG00000006139 | TRPM4 | 3 | brown |
| ENSBTAG00000006143 | CREB3L1 | 3 | brown |
| ENSBTAG00000006161 | MET | 3 | brown |
| ENSBTAG00000006214 | LOXL2 | 3 | brown |
| ENSBTAG00000006225 | RPA2 | 3 | brown |
| ENSBTAG00000006326 | ALDH1L2 | 3 | brown |
| ENSBTAG00000006359 | EPHA1 | 3 | brown |
| ENSBTAG00000006367 | CCN2 | 3 | brown |
| ENSBTAG00000006434 | SYNPO2 | 3 | brown |
| ENSBTAG00000006447 | ACSM3 | 3 | brown |
| ENSBTAG00000006474 | EPCAM | 3 | brown |
| ENSBTAG00000006515 | ESPN | 3 | brown |
| ENSBTAG00000006528 | BBS9 | 3 | brown |
| ENSBTAG00000006538 | PTHLH | 3 | brown |
| ENSBTAG00000006563 | KLHL40 | 3 | brown |
| ENSBTAG00000006609 | ERRFI1 | 3 | brown |
| ENSBTAG00000006673 | TMEM179 | 3 | brown |
| ENSBTAG00000006720 | GJA5 | 3 | brown |
| ENSBTAG00000006734 | BICD1 | 3 | brown |
| ENSBTAG00000006747 | LTBP3 | 3 | brown |
| ENSBTAG00000006762 | SH3RF2 | 3 | brown |
| ENSBTAG00000006777 | TLCD1 | 3 | brown |
| ENSBTAG00000006806 | KRT17 | 3 | brown |
| ENSBTAG00000006862 | MEIS3 | 3 | brown |
| ENSBTAG00000006870 | RASGEF1A | 3 | brown |
| ENSBTAG00000006904 | TNS2 | 3 | brown |
| ENSBTAG00000006934 | CYP11A1 | 3 | brown |
| ENSBTAG00000006958 | C23H6orf136 | 3 | brown |
| ENSBTAG00000006974 | PLEKHA7 | 3 | brown |
| ENSBTAG00000006989 | SEMA4F | 3 | brown |
| ENSBTAG00000006995 | SPTBN1 | 3 | brown |

| | | | |
|---|---|---|---|
| ENSBTAG00000007013 | TOM1L1 | 3 | brown |
| ENSBTAG00000007036 | SLC17A7 | 3 | brown |
| ENSBTAG00000007051 | CLDN23 | 3 | brown |
| ENSBTAG00000007071 | RAI14 | 3 | brown |
| ENSBTAG00000007147 | CUEDC1 | 3 | brown |
| ENSBTAG00000007177 | EMP2 | 3 | brown |
| ENSBTAG00000007196 | TAGLN | 3 | brown |
| ENSBTAG00000007204 | KRT80 | 3 | brown |
| ENSBTAG00000007220 | CASKIN2 | 3 | brown |
| ENSBTAG00000007369 | PDLIM2 | 3 | brown |
| ENSBTAG00000007415 | SLC7A8 | 3 | brown |
| ENSBTAG00000007429 | SPNS2 | 3 | brown |
| ENSBTAG00000007436 | GGT6 | 3 | brown |
| ENSBTAG00000007485 | GRHL1 | 3 | brown |
| ENSBTAG00000007490 | SULF2 | 3 | brown |
| ENSBTAG00000007502 | CKMT1A | 3 | brown |
| ENSBTAG00000007532 | TRIM15 | 3 | brown |
| ENSBTAG00000007544 | MARVELD3 | 3 | brown |
| ENSBTAG00000007545 | PHLPP2 | 3 | brown |
| ENSBTAG00000007623 | CPTP | 3 | brown |
| ENSBTAG00000007651 | TARS3 | 3 | brown |
| ENSBTAG00000007657 | SCARA3 | 3 | brown |
| ENSBTAG00000007666 | IGF2BP2 | 3 | brown |
| ENSBTAG00000007678 | MKX | 3 | brown |
| ENSBTAG00000007704 | ELOVL7 | 3 | brown |
| ENSBTAG00000007762 | ASIC3 | 3 | brown |
| ENSBTAG00000007763 | SLC1A4 | 3 | brown |
| ENSBTAG00000007841 | WTIP | 3 | brown |
| ENSBTAG00000007958 | TNS4 | 3 | brown |
| ENSBTAG00000007962 | ATP9A | 3 | brown |
| ENSBTAG00000008023 | RHBDL2 | 3 | brown |
| ENSBTAG00000008036 | CELSR1 | 3 | brown |
| ENSBTAG00000008079 | NRBP2 | 3 | brown |
| ENSBTAG00000008111 | ESYT3 | 3 | brown |
| ENSBTAG00000008232 | DAB2IP | 3 | brown |
| ENSBTAG00000008275 | GREB1L | 3 | brown |
| ENSBTAG00000008280 | HNF4G | 3 | brown |
| ENSBTAG00000008301 | WNK2 | 3 | brown |
| ENSBTAG00000008331 | TMEM54 | 3 | brown |
| ENSBTAG00000008333 | ETV4 | 3 | brown |
| ENSBTAG00000008493 | AQP3 | 3 | brown |
| ENSBTAG00000008494 | TMEM171 | 3 | brown |
| ENSBTAG00000008533 | GLCCI1 | 3 | brown |
| ENSBTAG00000008541 | MGST1 | 3 | brown |
| ENSBTAG00000008553 | B4GALNT3 | 3 | brown |
| ENSBTAG00000008575 | CGNL1 | 3 | brown |
| ENSBTAG00000008596 | PRICKLE1 | 3 | brown |

| | | | |
|---|---|---|---|
| ENSBTAG00000008609 | SEPTIN8 | 3 | brown |
| ENSBTAG00000008654 | HOOK2 | 3 | brown |
| ENSBTAG00000008656 | KBTBD6 | 3 | brown |
| ENSBTAG00000008661 | C15H11orf52 | 3 | brown |
| ENSBTAG00000008700 | CRCT1 | 3 | brown |
| ENSBTAG00000008704 | FIGN | 3 | brown |
| ENSBTAG00000008728 | MRTFB | 3 | brown |
| ENSBTAG00000008756 | ELF3 | 3 | brown |
| ENSBTAG00000008797 | ARHGEF38 | 3 | brown |
| ENSBTAG00000008816 | TRIM2 | 3 | brown |
| ENSBTAG00000008817 | LAMA4 | 3 | brown |
| ENSBTAG00000008842 | JPH1 | 3 | brown |
| ENSBTAG00000008849 | SORT1 | 3 | brown |
| ENSBTAG00000008858 | ARHGAP8 | 3 | brown |
| ENSBTAG00000008877 | APBA1 | 3 | brown |
| ENSBTAG00000008921 | NEXN | 3 | brown |
| ENSBTAG00000009020 | CRIM1 | 3 | brown |
| ENSBTAG00000009058 | TMEM177 | 3 | brown |
| ENSBTAG00000009075 | RNF207 | 3 | brown |
| ENSBTAG00000009080 | ITGB6 | 3 | brown |
| ENSBTAG00000009125 | ALDH1A3 | 3 | brown |
| ENSBTAG00000009126 | YBX2 | 3 | brown |
| ENSBTAG00000009127 | TSPYL4 | 3 | brown |
| ENSBTAG00000009132 | TMPRSS2 | 3 | brown |
| ENSBTAG00000009145 | SLC7A4 | 3 | brown |
| ENSBTAG00000009150 | SPON1 | 3 | brown |
| ENSBTAG00000009153 | MLXIPL | 3 | brown |
| ENSBTAG00000009191 | TUSC3 | 3 | brown |
| ENSBTAG00000009194 | OSBPL10 | 3 | brown |
| ENSBTAG00000009209 | BEND5 | 3 | brown |
| ENSBTAG00000009258 | NEK11 | 3 | brown |
| ENSBTAG00000009294 | DEGS2 | 3 | brown |
| ENSBTAG00000009299 | AFTPH | 3 | brown |
| ENSBTAG00000009390 | MINDY4 | 3 | brown |
| ENSBTAG00000009407 | MBTD1 | 3 | brown |
| ENSBTAG00000009419 | TRIM29 | 3 | brown |
| ENSBTAG00000009423 | NIPAL1 | 3 | brown |
| ENSBTAG00000009494 | CBLC | 3 | brown |
| ENSBTAG00000009495 | BCAM | 3 | brown |
| ENSBTAG00000009512 | EPS8L1 | 3 | brown |
| ENSBTAG00000009543 | ESYT1 | 3 | brown |
| ENSBTAG00000009576 | PACSIN3 | 3 | brown |
| ENSBTAG00000009607 | LARGE2 | 3 | brown |
| ENSBTAG00000009637 | SLC12A2 | 3 | brown |
| ENSBTAG00000009663 | YBX3 | 3 | brown |
| ENSBTAG00000009665 | UTRN | 3 | brown |
| ENSBTAG00000009679 | PLEKHG1 | 3 | brown |

| | | | |
|---|---|---|---|
| ENSBTAG00000009704 | CECR2 | 3 | brown |
| ENSBTAG00000009725 | AOX1 | 3 | brown |
| ENSBTAG00000009733 | FBP1 | 3 | brown |
| ENSBTAG00000009736 | CDC42BPG | 3 | brown |
| ENSBTAG00000009830 | PLEKHB1 | 3 | brown |
| ENSBTAG00000009835 | CACNA1G | 3 | brown |
| ENSBTAG00000009844 | CCN1 | 3 | brown |
| ENSBTAG00000009873 | NRN1 | 3 | brown |
| ENSBTAG00000009943 | XCL2 | 3 | brown |
| ENSBTAG00000009994 | EML5 | 3 | brown |
| ENSBTAG00000009997 | ANO1 | 3 | brown |
| ENSBTAG00000009998 | GALNT16 | 3 | brown |
| ENSBTAG00000010113 | ZBTB24 | 3 | brown |
| ENSBTAG00000010146 | PLXNB3 | 3 | brown |
| ENSBTAG00000010152 | MID1 | 3 | brown |
| ENSBTAG00000010219 | KRBA1 | 3 | brown |
| ENSBTAG00000010228 | NA | 3 | brown |
| ENSBTAG00000010270 | LOC512541 | 3 | brown |
| ENSBTAG00000010279 | DUSP14 | 3 | brown |
| ENSBTAG00000010293 | PARD3B | 3 | brown |
| ENSBTAG00000010300 | NA | 3 | brown |
| ENSBTAG00000010357 | ST6GAL1 | 3 | brown |
| ENSBTAG00000010360 | LRIG1 | 3 | brown |
| ENSBTAG00000010362 | NOSTRIN | 3 | brown |
| ENSBTAG00000010371 | CHAC1 | 3 | brown |
| ENSBTAG00000010389 | STBD1 | 3 | brown |
| ENSBTAG00000010392 | ESRRG | 3 | brown |
| ENSBTAG00000010395 | DOCK9 | 3 | brown |
| ENSBTAG00000010423 | LIFR | 3 | brown |
| ENSBTAG00000010431 | LURAP1L | 3 | brown |
| ENSBTAG00000010444 | ERBB3 | 3 | brown |
| ENSBTAG00000010452 | PODXL | 3 | brown |
| ENSBTAG00000010517 | EVPL | 3 | brown |
| ENSBTAG00000010529 | FZD6 | 3 | brown |
| ENSBTAG00000010531 | CYP1B1 | 3 | brown |
| ENSBTAG00000010581 | MAGI1 | 3 | brown |
| ENSBTAG00000010609 | FAM221A | 3 | brown |
| ENSBTAG00000010634 | NDNF | 3 | brown |
| ENSBTAG00000010640 | NA | 3 | brown |
| ENSBTAG00000010660 | CACNA1C | 3 | brown |
| ENSBTAG00000010661 | DPYSL3 | 3 | brown |
| ENSBTAG00000010671 | GLI3 | 3 | brown |
| ENSBTAG00000010677 | LIMCH1 | 3 | brown |
| ENSBTAG00000010682 | DDR1 | 3 | brown |
| ENSBTAG00000010693 | LMO7 | 3 | brown |
| ENSBTAG00000010756 | ST7 | 3 | brown |
| ENSBTAG00000010786 | TACC2 | 3 | brown |

| | | | |
|---|---|---|---|
| ENSBTAG00000010798 | SLC23A1 | 3 | brown |
| ENSBTAG00000010809 | HID1 | 3 | brown |
| ENSBTAG00000010820 | WNT11 | 3 | brown |
| ENSBTAG00000010850 | SERTAD4 | 3 | brown |
| ENSBTAG00000010981 | CIB2 | 3 | brown |
| ENSBTAG00000010986 | KCNQ1 | 3 | brown |
| ENSBTAG00000011108 | USP54 | 3 | brown |
| ENSBTAG00000011112 | KCNK5 | 3 | brown |
| ENSBTAG00000011133 | AP1S3 | 3 | brown |
| ENSBTAG00000011149 | SIGIRR | 3 | brown |
| ENSBTAG00000011150 | PFN2 | 3 | brown |
| ENSBTAG00000011154 | SDC1 | 3 | brown |
| ENSBTAG00000011156 | ATP8A1 | 3 | brown |
| ENSBTAG00000011171 | NA | 3 | brown |
| ENSBTAG00000011182 | PDLIM1 | 3 | brown |
| ENSBTAG00000011187 | FAM13A | 3 | brown |
| ENSBTAG00000011229 | TMUB1 | 3 | brown |
| ENSBTAG00000011256 | MYO1B | 3 | brown |
| ENSBTAG00000011266 | ZBTB16 | 3 | brown |
| ENSBTAG00000011274 | ENO2 | 3 | brown |
| ENSBTAG00000011291 | DLG3 | 3 | brown |
| ENSBTAG00000011330 | NA | 3 | brown |
| ENSBTAG00000011356 | CAMSAP3 | 3 | brown |
| ENSBTAG00000011397 | UNC13B | 3 | brown |
| ENSBTAG00000011400 | DBN1 | 3 | brown |
| ENSBTAG00000011413 | CCDC107 | 3 | brown |
| ENSBTAG00000011476 | NA | 3 | brown |
| ENSBTAG00000011518 | RARB | 3 | brown |
| ENSBTAG00000011538 | KIF1A | 3 | brown |
| ENSBTAG00000011558 | GPAT2 | 3 | brown |
| ENSBTAG00000011602 | RASAL3 | 3 | brown |
| ENSBTAG00000011613 | PLS3 | 3 | brown |
| ENSBTAG00000011628 | EGFR | 3 | brown |
| ENSBTAG00000011668 | KCTD14 | 3 | brown |
| ENSBTAG00000011723 | GRIK5 | 3 | brown |
| ENSBTAG00000011734 | ANKRD1 | 3 | brown |
| ENSBTAG00000011752 | SYNM | 3 | brown |
| ENSBTAG00000011770 | TJP2 | 3 | brown |
| ENSBTAG00000011779 | MAL2 | 3 | brown |
| ENSBTAG00000011833 | GRIA3 | 3 | brown |
| ENSBTAG00000011854 | SLC38A5 | 3 | brown |
| ENSBTAG00000011864 | NA | 3 | brown |
| ENSBTAG00000011880 | CHAF1B | 3 | brown |
| ENSBTAG00000011905 | RRAS2 | 3 | brown |
| ENSBTAG00000011935 | RASSF6 | 3 | brown |
| ENSBTAG00000012004 | TGFB3 | 3 | brown |
| ENSBTAG00000012007 | SOCS2 | 3 | brown |

| | | | |
|---|---|---|---|
| ENSBTAG00000012020 | TRAF5 | 3 | brown |
| ENSBTAG00000012031 | ATG9B | 3 | brown |
| ENSBTAG00000012036 | HR | 3 | brown |
| ENSBTAG00000012054 | LOC508879 | 3 | brown |
| ENSBTAG00000012082 | TNFRSF12A | 3 | brown |
| ENSBTAG00000012083 | IRX3 | 3 | brown |
| ENSBTAG00000012111 | PRDM5 | 3 | brown |
| ENSBTAG00000012128 | AASS | 3 | brown |
| ENSBTAG00000012137 | NTN1 | 3 | brown |
| ENSBTAG00000012157 | CCDC158 | 3 | brown |
| ENSBTAG00000012194 | DENND2C | 3 | brown |
| ENSBTAG00000012211 | HOXA5 | 3 | brown |
| ENSBTAG00000012249 | PRR15L | 3 | brown |
| ENSBTAG00000012280 | PGLYRP2 | 3 | brown |
| ENSBTAG00000012305 | ADGRL2 | 3 | brown |
| ENSBTAG00000012307 | DTNA | 3 | brown |
| ENSBTAG00000012405 | PEAR1 | 3 | brown |
| ENSBTAG00000012449 | AFF3 | 3 | brown |
| ENSBTAG00000012464 | AFAP1 | 3 | brown |
| ENSBTAG00000012482 | CDCP1 | 3 | brown |
| ENSBTAG00000012518 | PCDH1 | 3 | brown |
| ENSBTAG00000012584 | GJB3 | 3 | brown |
| ENSBTAG00000012646 | PLCXD2 | 3 | brown |
| ENSBTAG00000012656 | OVOL1 | 3 | brown |
| ENSBTAG00000012673 | CDK18 | 3 | brown |
| ENSBTAG00000012674 | CNKSR3 | 3 | brown |
| ENSBTAG00000012715 | KIF26B | 3 | brown |
| ENSBTAG00000012718 | NA | 3 | brown |
| ENSBTAG00000012724 | AJUBA | 3 | brown |
| ENSBTAG00000012781 | TP53BP2 | 3 | brown |
| ENSBTAG00000012818 | PDLIM5 | 3 | brown |
| ENSBTAG00000012829 | EBI3 | 3 | brown |
| ENSBTAG00000012848 | PTPRU | 3 | brown |
| ENSBTAG00000012851 | SLC5A1 | 3 | brown |
| ENSBTAG00000012919 | MMP15 | 3 | brown |
| ENSBTAG00000013081 | PSPH | 3 | brown |
| ENSBTAG00000013107 | SHANK1 | 3 | brown |
| ENSBTAG00000013143 | BAG4 | 3 | brown |
| ENSBTAG00000013148 | CLDN1 | 3 | brown |
| ENSBTAG00000013187 | DLG5 | 3 | brown |
| ENSBTAG00000013191 | AGRN | 3 | brown |
| ENSBTAG00000013222 | CD109 | 3 | brown |
| ENSBTAG00000013287 | SMO | 3 | brown |
| ENSBTAG00000013306 | ZBTB7C | 3 | brown |
| ENSBTAG00000013320 | TSPAN1 | 3 | brown |
| ENSBTAG00000013341 | GYG2 | 3 | brown |
| ENSBTAG00000013406 | CSRP2 | 3 | brown |

| | | | |
|---|---|---|---|
| ENSBTAG00000013407 | PLXNB1 | 3 | brown |
| ENSBTAG00000013439 | ARHGEF26 | 3 | brown |
| ENSBTAG00000013462 | L1CAM | 3 | brown |
| ENSBTAG00000013533 | CLIC1 | 3 | brown |
| ENSBTAG00000013537 | FER1L6 | 3 | brown |
| ENSBTAG00000013548 | GABRE | 3 | brown |
| ENSBTAG00000013664 | FILIP1L | 3 | brown |
| ENSBTAG00000013736 | PROM1 | 3 | brown |
| ENSBTAG00000013744 | SYNPO | 3 | brown |
| ENSBTAG00000013869 | SH3BP4 | 3 | brown |
| ENSBTAG00000013880 | WWC1 | 3 | brown |
| ENSBTAG00000013888 | FOXA1 | 3 | brown |
| ENSBTAG00000013928 | WFDC2 | 3 | brown |
| ENSBTAG00000014053 | MAK | 3 | brown |
| ENSBTAG00000014064 | FGFR2 | 3 | brown |
| ENSBTAG00000014083 | AMIGO1 | 3 | brown |
| ENSBTAG00000014092 | CELF6 | 3 | brown |
| ENSBTAG00000014119 | PRKCZ | 3 | brown |
| ENSBTAG00000014124 | MLLT3 | 3 | brown |
| ENSBTAG00000014147 | PRSS54 | 3 | brown |
| ENSBTAG00000014191 | QSOX1 | 3 | brown |
| ENSBTAG00000014204 | S100A16 | 3 | brown |
| ENSBTAG00000014207 | ADAMTS10 | 3 | brown |
| ENSBTAG00000014224 | IGSF9B | 3 | brown |
| ENSBTAG00000014225 | SLC5A9 | 3 | brown |
| ENSBTAG00000014234 | CCDC196 | 3 | brown |
| ENSBTAG00000014270 | UNC5B | 3 | brown |
| ENSBTAG00000014299 | RHOC | 3 | brown |
| ENSBTAG00000014304 | TMEM47 | 3 | brown |
| ENSBTAG00000014329 | LOC512548 | 3 | brown |
| ENSBTAG00000014375 | TMCC3 | 3 | brown |
| ENSBTAG00000014405 | OSBPL6 | 3 | brown |
| ENSBTAG00000014455 | PDZD2 | 3 | brown |
| ENSBTAG00000014485 | POF1B | 3 | brown |
| ENSBTAG00000014501 | FERMT2 | 3 | brown |
| ENSBTAG00000014515 | RFC1 | 3 | brown |
| ENSBTAG00000014537 | NA | 3 | brown |
| ENSBTAG00000014538 | HPCAL4 | 3 | brown |
| ENSBTAG00000014540 | PERM1 | 3 | brown |
| ENSBTAG00000014576 | GUCY1A1 | 3 | brown |
| ENSBTAG00000014596 | EFHD1 | 3 | brown |
| ENSBTAG00000014602 | CAMTA1 | 3 | brown |
| ENSBTAG00000014676 | SHROOM1 | 3 | brown |
| ENSBTAG00000014718 | NA | 3 | brown |
| ENSBTAG00000014750 | EPB41L4A | 3 | brown |
| ENSBTAG00000014791 | CTH | 3 | brown |
| ENSBTAG00000014885 | MYOM3 | 3 | brown |

| | | | |
|---|---|---|---|
| ENSBTAG00000014889 | DCBLD2 | 3 | brown |
| ENSBTAG00000014915 | ETV5 | 3 | brown |
| ENSBTAG00000014932 | ARSL | 3 | brown |
| ENSBTAG00000014947 | PTPN13 | 3 | brown |
| ENSBTAG00000014960 | SCHIP1 | 3 | brown |
| ENSBTAG00000015044 | CARD10 | 3 | brown |
| ENSBTAG00000015053 | CFL2 | 3 | brown |
| ENSBTAG00000015085 | CLIC3 | 3 | brown |
| ENSBTAG00000015106 | DSP | 3 | brown |
| ENSBTAG00000015113 | CITED4 | 3 | brown |
| ENSBTAG00000015163 | TM4SF1 | 3 | brown |
| ENSBTAG00000015177 | PRSS23 | 3 | brown |
| ENSBTAG00000015218 | NA | 3 | brown |
| ENSBTAG00000015311 | PTPRR | 3 | brown |
| ENSBTAG00000015398 | TJP1 | 3 | brown |
| ENSBTAG00000015406 | ZNF750 | 3 | brown |
| ENSBTAG00000015419 | ARHGEF37 | 3 | brown |
| ENSBTAG00000015434 | DSTN | 3 | brown |
| ENSBTAG00000015457 | FGFR1 | 3 | brown |
| ENSBTAG00000015511 | FADS3 | 3 | brown |
| ENSBTAG00000015513 | DOCK5 | 3 | brown |
| ENSBTAG00000015527 | MYO1D | 3 | brown |
| ENSBTAG00000015537 | RASAL2 | 3 | brown |
| ENSBTAG00000015565 | NA | 3 | brown |
| ENSBTAG00000015606 | NA | 3 | brown |
| ENSBTAG00000015618 | GDF15 | 3 | brown |
| ENSBTAG00000015655 | APBB1 | 3 | brown |
| ENSBTAG00000015794 | NES | 3 | brown |
| ENSBTAG00000015893 | EPN3 | 3 | brown |
| ENSBTAG00000015896 | CC2D1B | 3 | brown |
| ENSBTAG00000015904 | RORA | 3 | brown |
| ENSBTAG00000015905 | ARHGAP32 | 3 | brown |
| ENSBTAG00000015913 | MFHAS1 | 3 | brown |
| ENSBTAG00000015991 | CDH1 | 3 | brown |
| ENSBTAG00000016041 | B3GNT8 | 3 | brown |
| ENSBTAG00000016070 | LINGO2 | 3 | brown |
| ENSBTAG00000016137 | ZNF608 | 3 | brown |
| ENSBTAG00000016140 | PPFIBP2 | 3 | brown |
| ENSBTAG00000016165 | KRT7 | 3 | brown |
| ENSBTAG00000016185 | ENAH | 3 | brown |
| ENSBTAG00000016194 | FBXO32 | 3 | brown |
| ENSBTAG00000016228 | NMD3 | 3 | brown |
| ENSBTAG00000016250 | AKTIP | 3 | brown |
| ENSBTAG00000016283 | TMEM63C | 3 | brown |
| ENSBTAG00000016349 | TEAD2 | 3 | brown |
| ENSBTAG00000016407 | IRX6 | 3 | brown |
| ENSBTAG00000016412 | NA | 3 | brown |

| | | | |
|---|---|---|---|
| ENSBTAG00000016444 | RETREG1 | 3 | brown |
| ENSBTAG00000016469 | TNKS1BP1 | 3 | brown |
| ENSBTAG00000016486 | SORBS2 | 3 | brown |
| ENSBTAG00000016496 | ZNF641 | 3 | brown |
| ENSBTAG00000016554 | C1H3orf52 | 3 | brown |
| ENSBTAG00000016713 | TMEM30B | 3 | brown |
| ENSBTAG00000016714 | NA | 3 | brown |
| ENSBTAG00000016728 | PLEKHG5 | 3 | brown |
| ENSBTAG00000016738 | DTX1 | 3 | brown |
| ENSBTAG00000016739 | RASAL1 | 3 | brown |
| ENSBTAG00000016751 | MYO6 | 3 | brown |
| ENSBTAG00000016770 | GAPT | 3 | brown |
| ENSBTAG00000016771 | PLK2 | 3 | brown |
| ENSBTAG00000016784 | DNAH7 | 3 | brown |
| ENSBTAG00000016800 | SEMA6D | 3 | brown |
| ENSBTAG00000016813 | SH3D19 | 3 | brown |
| ENSBTAG00000016904 | MARCHF9 | 3 | brown |
| ENSBTAG00000016918 | MYOF | 3 | brown |
| ENSBTAG00000016936 | MISP3 | 3 | brown |
| ENSBTAG00000016973 | PRSS8 | 3 | brown |
| ENSBTAG00000017007 | TRIB3 | 3 | brown |
| ENSBTAG00000017024 | PPARGC1A | 3 | brown |
| ENSBTAG00000017033 | LSR | 3 | brown |
| ENSBTAG00000017040 | LY6E | 3 | brown |
| ENSBTAG00000017062 | NA | 3 | brown |
| ENSBTAG00000017063 | EPB41L4B | 3 | brown |
| ENSBTAG00000017075 | STX1A | 3 | brown |
| ENSBTAG00000017077 | CTSV | 3 | brown |
| ENSBTAG00000017086 | GRB10 | 3 | brown |
| ENSBTAG00000017122 | HSPG2 | 3 | brown |
| ENSBTAG00000017123 | ZNF93 | 3 | brown |
| ENSBTAG00000017132 | TTC22 | 3 | brown |
| ENSBTAG00000017150 | EHF | 3 | brown |
| ENSBTAG00000017205 | CCN6 | 3 | brown |
| ENSBTAG00000017239 | GABBR1 | 3 | brown |
| ENSBTAG00000017243 | GATA3 | 3 | brown |
| ENSBTAG00000017281 | OPLAH | 3 | brown |
| ENSBTAG00000017325 | ATG13 | 3 | brown |
| ENSBTAG00000017366 | GRB7 | 3 | brown |
| ENSBTAG00000017370 | ARFGEF3 | 3 | brown |
| ENSBTAG00000017405 | RORC | 3 | brown |
| ENSBTAG00000017409 | DLX3 | 3 | brown |
| ENSBTAG00000017456 | NYAP1 | 3 | brown |
| ENSBTAG00000017512 | MAPT | 3 | brown |
| ENSBTAG00000017514 | CCND1 | 3 | brown |
| ENSBTAG00000017529 | CA8 | 3 | brown |
| ENSBTAG00000017533 | LRRC1 | 3 | brown |

| | | | |
|---|---|---|---|
| ENSBTAG00000017564 | CGN | 3 | brown |
| ENSBTAG00000017566 | TUFT1 | 3 | brown |
| ENSBTAG00000017602 | TMEM45B | 3 | brown |
| ENSBTAG00000017604 | RAB13 | 3 | brown |
| ENSBTAG00000017655 | PALMD | 3 | brown |
| ENSBTAG00000017685 | JUP | 3 | brown |
| ENSBTAG00000017691 | KIAA0040 | 3 | brown |
| ENSBTAG00000017715 | HOMER2 | 3 | brown |
| ENSBTAG00000017718 | CCL22 | 3 | brown |
| ENSBTAG00000017739 | TNK1 | 3 | brown |
| ENSBTAG00000017747 | PLSCR3 | 3 | brown |
| ENSBTAG00000017753 | APP | 3 | brown |
| ENSBTAG00000017771 | EPN2 | 3 | brown |
| ENSBTAG00000017797 | LY75 | 3 | brown |
| ENSBTAG00000017812 | ALS2CL | 3 | brown |
| ENSBTAG00000017818 | MOG | 3 | brown |
| ENSBTAG00000017830 | RBMS2 | 3 | brown |
| ENSBTAG00000017836 | ZFP57 | 3 | brown |
| ENSBTAG00000017846 | F11R | 3 | brown |
| ENSBTAG00000017869 | CAV1 | 3 | brown |
| ENSBTAG00000017877 | NECTIN4 | 3 | brown |
| ENSBTAG00000017956 | KCTD15 | 3 | brown |
| ENSBTAG00000017983 | PRELID3A | 3 | brown |
| ENSBTAG00000018002 | DTX3 | 3 | brown |
| ENSBTAG00000018007 | NR2F2 | 3 | brown |
| ENSBTAG00000018062 | KIAA1522 | 3 | brown |
| ENSBTAG00000018064 | FAM221A | 3 | brown |
| ENSBTAG00000018077 | LYPD3 | 3 | brown |
| ENSBTAG00000018085 | GTSF1 | 3 | brown |
| ENSBTAG00000018143 | C16H1orf116 | 3 | brown |
| ENSBTAG00000018156 | KRTCAP3 | 3 | brown |
| ENSBTAG00000018164 | FNDC4 | 3 | brown |
| ENSBTAG00000018169 | ITGB4 | 3 | brown |
| ENSBTAG00000018199 | ZBTB8A | 3 | brown |
| ENSBTAG00000018202 | CYB561A3 | 3 | brown |
| ENSBTAG00000018206 | NIPAL2 | 3 | brown |
| ENSBTAG00000018225 | SIPA1L3 | 3 | brown |
| ENSBTAG00000018236 | EPB41L5 | 3 | brown |
| ENSBTAG00000018257 | APIP | 3 | brown |
| ENSBTAG00000018297 | PLEKHH1 | 3 | brown |
| ENSBTAG00000018307 | SEMA3F | 3 | brown |
| ENSBTAG00000018342 | TJP3 | 3 | brown |
| ENSBTAG00000018395 | KIAA1217 | 3 | brown |
| ENSBTAG00000018431 | NAV2 | 3 | brown |
| ENSBTAG00000018467 | BBOF1 | 3 | brown |
| ENSBTAG00000018499 | ANXA8L1 | 3 | brown |
| ENSBTAG00000018517 | VLDLR | 3 | brown |

| | | | |
|---|---|---|---|
| ENSBTAG00000018546 | LRBA | 3 | brown |
| ENSBTAG00000018596 | PTPN21 | 3 | brown |
| ENSBTAG00000018644 | PDZRN3 | 3 | brown |
| ENSBTAG00000018663 | GPRC5C | 3 | brown |
| ENSBTAG00000018691 | RHOU | 3 | brown |
| ENSBTAG00000018705 | ADCY3 | 3 | brown |
| ENSBTAG00000018725 | FBLIM1 | 3 | brown |
| ENSBTAG00000018749 | NUDT16 | 3 | brown |
| ENSBTAG00000018774 | NUAK2 | 3 | brown |
| ENSBTAG00000018799 | GPSM1 | 3 | brown |
| ENSBTAG00000018810 | THBS2 | 3 | brown |
| ENSBTAG00000018841 | PTPN3 | 3 | brown |
| ENSBTAG00000018843 | SERPINA1 | 3 | brown |
| ENSBTAG00000018908 | GFOD2 | 3 | brown |
| ENSBTAG00000018909 | CREB5 | 3 | brown |
| ENSBTAG00000018914 | RAB25 | 3 | brown |
| ENSBTAG00000018940 | CRACR2A | 3 | brown |
| ENSBTAG00000018966 | PLCE1 | 3 | brown |
| ENSBTAG00000018972 | GARS1 | 3 | brown |
| ENSBTAG00000019024 | JAZF1 | 3 | brown |
| ENSBTAG00000019026 | EXTL2 | 3 | brown |
| ENSBTAG00000019036 | ARHGAP28 | 3 | brown |
| ENSBTAG00000019052 | ANK3 | 3 | brown |
| ENSBTAG00000019076 | PAK6 | 3 | brown |
| ENSBTAG00000019125 | SLC1A1 | 3 | brown |
| ENSBTAG00000019138 | KIF3C | 3 | brown |
| ENSBTAG00000019164 | RHOBTB1 | 3 | brown |
| ENSBTAG00000019181 | BAIAP2L1 | 3 | brown |
| ENSBTAG00000019208 | ZFTA | 3 | brown |
| ENSBTAG00000019217 | KIF13A | 3 | brown |
| ENSBTAG00000019252 | KDF1 | 3 | brown |
| ENSBTAG00000019289 | ITGA2 | 3 | brown |
| ENSBTAG00000019334 | KLC2 | 3 | brown |
| ENSBTAG00000019340 | NA | 3 | brown |
| ENSBTAG00000019342 | OSBPL3 | 3 | brown |
| ENSBTAG00000019343 | MDFI | 3 | brown |
| ENSBTAG00000019353 | STIM2 | 3 | brown |
| ENSBTAG00000019373 | CAPRIN2 | 3 | brown |
| ENSBTAG00000019382 | PKDCC | 3 | brown |
| ENSBTAG00000019399 | CNKSR1 | 3 | brown |
| ENSBTAG00000019448 | CLDN7 | 3 | brown |
| ENSBTAG00000019453 | PTGES | 3 | brown |
| ENSBTAG00000019455 | MYO5B | 3 | brown |
| ENSBTAG00000019459 | CHAC2 | 3 | brown |
| ENSBTAG00000019495 | TEAD3 | 3 | brown |
| ENSBTAG00000019498 | ITGA3 | 3 | brown |
| ENSBTAG00000019529 | ATP8A2 | 3 | brown |

| | | | |
|---|---|---|---|
| ENSBTAG00000019596 | GPRC5B | 3 | brown |
| ENSBTAG00000019633 | SHROOM3 | 3 | brown |
| ENSBTAG00000019689 | NA | 3 | brown |
| ENSBTAG00000019700 | PC | 3 | brown |
| ENSBTAG00000019754 | CAVIN3 | 3 | brown |
| ENSBTAG00000019761 | MANBA | 3 | brown |
| ENSBTAG00000019788 | TEAD4 | 3 | brown |
| ENSBTAG00000019791 | GRHL3 | 3 | brown |
| ENSBTAG00000019805 | RHBDF1 | 3 | brown |
| ENSBTAG00000019806 | YPEL2 | 3 | brown |
| ENSBTAG00000019810 | AARS1 | 3 | brown |
| ENSBTAG00000019818 | CASZ1 | 3 | brown |
| ENSBTAG00000019839 | LTBP1 | 3 | brown |
| ENSBTAG00000019916 | CLMN | 3 | brown |
| ENSBTAG00000019920 | BICDL2 | 3 | brown |
| ENSBTAG00000019947 | SYT7 | 3 | brown |
| ENSBTAG00000020018 | FRK | 3 | brown |
| ENSBTAG00000020046 | CLMP | 3 | brown |
| ENSBTAG00000020053 | ZEB1 | 3 | brown |
| ENSBTAG00000020056 | COL12A1 | 3 | brown |
| ENSBTAG00000020067 | LLGL2 | 3 | brown |
| ENSBTAG00000020070 | ABCC3 | 3 | brown |
| ENSBTAG00000020097 | PERP | 3 | brown |
| ENSBTAG00000020125 | RBFOX2 | 3 | brown |
| ENSBTAG00000020205 | PDE8B | 3 | brown |
| ENSBTAG00000020244 | EFNA1 | 3 | brown |
| ENSBTAG00000020266 | SLC19A2 | 3 | brown |
| ENSBTAG00000020269 | MISP | 3 | brown |
| ENSBTAG00000020286 | GAREM1 | 3 | brown |
| ENSBTAG00000020426 | TFAP2B | 3 | brown |
| ENSBTAG00000020454 | LZTS3 | 3 | brown |
| ENSBTAG00000020469 | TMED8 | 3 | brown |
| ENSBTAG00000020471 | SAMD15 | 3 | brown |
| ENSBTAG00000020495 | SH3YL1 | 3 | brown |
| ENSBTAG00000020569 | CACNA2D1 | 3 | brown |
| ENSBTAG00000020582 | PRSS22 | 3 | brown |
| ENSBTAG00000020600 | RHOD | 3 | brown |
| ENSBTAG00000020638 | TIMP3 | 3 | brown |
| ENSBTAG00000020643 | BARX2 | 3 | brown |
| ENSBTAG00000020689 | PPP1R13L | 3 | brown |
| ENSBTAG00000020707 | ADGRG6 | 3 | brown |
| ENSBTAG00000020731 | PRRG4 | 3 | brown |
| ENSBTAG00000020800 | BEND7 | 3 | brown |
| ENSBTAG00000020810 | CYB561 | 3 | brown |
| ENSBTAG00000020829 | PTPRK | 3 | brown |
| ENSBTAG00000020837 | ARHGEF16 | 3 | brown |
| ENSBTAG00000020852 | SLC16A11 | 3 | brown |

| | | | |
|---|---|---|---|
| ENSBTAG00000020895 | LOXL4 | 3 | brown |
| ENSBTAG00000020914 | CPNE8 | 3 | brown |
| ENSBTAG00000020934 | SHF | 3 | brown |
| ENSBTAG00000021025 | P3H2 | 3 | brown |
| ENSBTAG00000021073 | KIAA1549 | 3 | brown |
| ENSBTAG00000021079 | C3H1orf210 | 3 | brown |
| ENSBTAG00000021082 | TMEM125 | 3 | brown |
| ENSBTAG00000021133 | TENM3 | 3 | brown |
| ENSBTAG00000021140 | RMND5A | 3 | brown |
| ENSBTAG00000021151 | MYH10 | 3 | brown |
| ENSBTAG00000021160 | AP1M2 | 3 | brown |
| ENSBTAG00000021191 | EHD2 | 3 | brown |
| ENSBTAG00000021204 | TES | 3 | brown |
| ENSBTAG00000021210 | HES2 | 3 | brown |
| ENSBTAG00000021237 | DST | 3 | brown |
| ENSBTAG00000021251 | RIPK4 | 3 | brown |
| ENSBTAG00000021259 | C2CD2 | 3 | brown |
| ENSBTAG00000021294 | VWA1 | 3 | brown |
| ENSBTAG00000021308 | NA | 3 | brown |
| ENSBTAG00000021343 | ARHGEF12 | 3 | brown |
| ENSBTAG00000021372 | SEPTIN11 | 3 | brown |
| ENSBTAG00000021377 | S100A14 | 3 | brown |
| ENSBTAG00000021378 | S100A13 | 3 | brown |
| ENSBTAG00000021417 | DMWD | 3 | brown |
| ENSBTAG00000021434 | EDN2 | 3 | brown |
| ENSBTAG00000021518 | TMPRSS4 | 3 | brown |
| ENSBTAG00000021553 | PTPN14 | 3 | brown |
| ENSBTAG00000021594 | NA | 3 | brown |
| ENSBTAG00000021601 | PARD6B | 3 | brown |
| ENSBTAG00000021609 | ADGRF1 | 3 | brown |
| ENSBTAG00000021706 | TBX3 | 3 | brown |
| ENSBTAG00000021714 | TEKT2 | 3 | brown |
| ENSBTAG00000021751 | RASEF | 3 | brown |
| ENSBTAG00000021798 | ERBB2 | 3 | brown |
| ENSBTAG00000021808 | CACNB3 | 3 | brown |
| ENSBTAG00000021815 | STARD13 | 3 | brown |
| ENSBTAG00000021838 | ESRP1 | 3 | brown |
| ENSBTAG00000021867 | BFSP1 | 3 | brown |
| ENSBTAG00000021876 | WDR72 | 3 | brown |
| ENSBTAG00000021897 | B4GALT3 | 3 | brown |
| ENSBTAG00000021921 | STRBP | 3 | brown |
| ENSBTAG00000021923 | DSG2 | 3 | brown |
| ENSBTAG00000021975 | PATJ | 3 | brown |
| ENSBTAG00000021980 | RASSF8 | 3 | brown |
| ENSBTAG00000021992 | CAVIN4 | 3 | brown |
| ENSBTAG00000022019 | LOC101905136 | 3 | brown |
| ENSBTAG00000022147 | EPS8L2 | 3 | brown |

| | | | |
|---|---|---|---|
| ENSBTAG00000022155 | FSTL1 | 3 | brown |
| ENSBTAG00000022635 | LAMB2 | 3 | brown |
| ENSBTAG00000022989 | FAM174B | 3 | brown |
| ENSBTAG00000023338 | NA | 3 | brown |
| ENSBTAG00000023407 | NA | 3 | brown |
| ENSBTAG00000023411 | INHCA | 3 | brown |
| ENSBTAG00000023416 | PPP2R3A | 3 | brown |
| ENSBTAG00000023426 | FAIM | 3 | brown |
| ENSBTAG00000023472 | PPP1R14A | 3 | brown |
| ENSBTAG00000023736 | CDC42EP3 | 3 | brown |
| ENSBTAG00000023806 | COBL | 3 | brown |
| ENSBTAG00000023967 | NA | 3 | brown |
| ENSBTAG00000024015 | PTPRM | 3 | brown |
| ENSBTAG00000024091 | MALL | 3 | brown |
| ENSBTAG00000024219 | TRPV6 | 3 | brown |
| ENSBTAG00000024269 | TGFBR3 | 3 | brown |
| ENSBTAG00000024482 | RABGAP1L | 3 | brown |
| ENSBTAG00000024633 | PIK3C2B | 3 | brown |
| ENSBTAG00000024643 | VEPH1 | 3 | brown |
| ENSBTAG00000024663 | LAD1 | 3 | brown |
| ENSBTAG00000024869 | CX3CL1 | 3 | brown |
| ENSBTAG00000025035 | PRLR | 3 | brown |
| ENSBTAG00000025078 | LRRC75A | 3 | brown |
| ENSBTAG00000025161 | AGPAT2 | 3 | brown |
| ENSBTAG00000025181 | CCDC85C | 3 | brown |
| ENSBTAG00000025221 | TEX30 | 3 | brown |
| ENSBTAG00000025263 | FHIP1A | 3 | brown |
| ENSBTAG00000025856 | JMY | 3 | brown |
| ENSBTAG00000025964 | MAST4 | 3 | brown |
| ENSBTAG00000026156 | VGLL3 | 3 | brown |
| ENSBTAG00000026172 | LRATD2 | 3 | brown |
| ENSBTAG00000026275 | CCL24 | 3 | brown |
| ENSBTAG00000026278 | CLDN4 | 3 | brown |
| ENSBTAG00000026461 | CACNA1H | 3 | brown |
| ENSBTAG00000026497 | A4GALT | 3 | brown |
| ENSBTAG00000026716 | IBTK | 3 | brown |
| ENSBTAG00000026893 | EXOC3L4 | 3 | brown |
| ENSBTAG00000027024 | ARHGAP5 | 3 | brown |
| ENSBTAG00000027081 | ATP10A | 3 | brown |
| ENSBTAG00000027182 | NR3C2 | 3 | brown |
| ENSBTAG00000027225 | NA | 3 | brown |
| ENSBTAG00000027442 | NFIB | 3 | brown |
| ENSBTAG00000027569 | APBB2 | 3 | brown |
| ENSBTAG00000027722 | IPO7 | 3 | brown |
| ENSBTAG00000027766 | C1QTNF5 | 3 | brown |
| ENSBTAG00000030180 | SHANK3 | 3 | brown |
| ENSBTAG00000030227 | NA | 3 | brown |

| | | | |
|---|---|---|---|
| ENSBTAG00000030258 | CDC42EP1 | 3 | brown |
| ENSBTAG00000030322 | NA | 3 | brown |
| ENSBTAG00000030340 | IFFO2 | 3 | brown |
| ENSBTAG00000030671 | HOOK1 | 3 | brown |
| ENSBTAG00000030690 | RADIL | 3 | brown |
| ENSBTAG00000030749 | DNAJC6 | 3 | brown |
| ENSBTAG00000030805 | NA | 3 | brown |
| ENSBTAG00000030999 | NA | 3 | brown |
| ENSBTAG00000031134 | YWHAH | 3 | brown |
| ENSBTAG00000031184 | CDKN1C | 3 | brown |
| ENSBTAG00000031209 | SLC22A4 | 3 | brown |
| ENSBTAG00000031279 | SELENOM | 3 | brown |
| ENSBTAG00000031648 | NA | 3 | brown |
| ENSBTAG00000031707 | FRMD6 | 3 | brown |
| ENSBTAG00000031874 | ZNF165 | 3 | brown |
| ENSBTAG00000031933 | ALOX12E | 3 | brown |
| ENSBTAG00000032007 | LRP11 | 3 | brown |
| ENSBTAG00000032017 | NA | 3 | brown |
| ENSBTAG00000032068 | PLA2G4F | 3 | brown |
| ENSBTAG00000032152 | TMPRSS6 | 3 | brown |
| ENSBTAG00000032531 | MUSTN1 | 3 | brown |
| ENSBTAG00000032548 | SYBU | 3 | brown |
| ENSBTAG00000032657 | TEAD1 | 3 | brown |
| ENSBTAG00000032821 | SCEL | 3 | brown |
| ENSBTAG00000032951 | ABHD17C | 3 | brown |
| ENSBTAG00000033015 | FAHD1 | 3 | brown |
| ENSBTAG00000033107 | NA | 3 | brown |
| ENSBTAG00000033190 | PGM5 | 3 | brown |
| ENSBTAG00000033304 | TCIM | 3 | brown |
| ENSBTAG00000033398 | HKDC1 | 3 | brown |
| ENSBTAG00000033510 | MPZL2 | 3 | brown |
| ENSBTAG00000033621 | SMCO4 | 3 | brown |
| ENSBTAG00000033662 | NCKAP1 | 3 | brown |
| ENSBTAG00000033886 | CIST1 | 3 | brown |
| ENSBTAG00000034075 | SLC7A11 | 3 | brown |
| ENSBTAG00000034192 | KIAA2012 | 3 | brown |
| ENSBTAG00000034501 | CFI | 3 | brown |
| ENSBTAG00000034645 | PON3 | 3 | brown |
| ENSBTAG00000034693 | SYT1 | 3 | brown |
| ENSBTAG00000034776 | DDAH1 | 3 | brown |
| ENSBTAG00000034848 | F2RL1 | 3 | brown |
| ENSBTAG00000034954 | NA | 3 | brown |
| ENSBTAG00000035945 | NA | 3 | brown |
| ENSBTAG00000036028 | BEX2 | 3 | brown |
| ENSBTAG00000037651 | S100A2 | 3 | brown |
| ENSBTAG00000037693 | NA | 3 | brown |
| ENSBTAG00000037929 | ADAMDEC1 | 3 | brown |

| | | | |
|---|---|---|---|
| ENSBTAG00000038033 | KRT6B | 3 | brown |
| ENSBTAG00000038112 | LOC100850276 | 3 | brown |
| ENSBTAG00000038141 | ADGRG1 | 3 | brown |
| ENSBTAG00000038148 | SOWAHB | 3 | brown |
| ENSBTAG00000038263 | LOC782529 | 3 | brown |
| ENSBTAG00000038495 | DCLK1 | 3 | brown |
| ENSBTAG00000038502 | NA | 3 | brown |
| ENSBTAG00000038600 | LRRC15 | 3 | brown |
| ENSBTAG00000038617 | SH3D21 | 3 | brown |
| ENSBTAG00000038698 | NA | 3 | brown |
| ENSBTAG00000038733 | EFS | 3 | brown |
| ENSBTAG00000038990 | TRAF4 | 3 | brown |
| ENSBTAG00000039195 | NHS | 3 | brown |
| ENSBTAG00000039307 | YAP1 | 3 | brown |
| ENSBTAG00000039355 | MADCAM1 | 3 | brown |
| ENSBTAG00000039425 | KRT6A | 3 | brown |
| ENSBTAG00000039477 | TPBG | 3 | brown |
| ENSBTAG00000039509 | GPRC5A | 3 | brown |
| ENSBTAG00000039571 | PROSER2 | 3 | brown |
| ENSBTAG00000039684 | CAVIN1 | 3 | brown |
| ENSBTAG00000039714 | LOC523126 | 3 | brown |
| ENSBTAG00000039719 | PHGDH | 3 | brown |
| ENSBTAG00000039731 | RND3 | 3 | brown |
| ENSBTAG00000039820 | CLDN8 | 3 | brown |
| ENSBTAG00000040001 | MARVELD2 | 3 | brown |
| ENSBTAG00000040088 | SLC9B2 | 3 | brown |
| ENSBTAG00000040209 | ZNF112 | 3 | brown |
| ENSBTAG00000040432 | CLDN3 | 3 | brown |
| ENSBTAG00000040460 | KRT23 | 3 | brown |
| ENSBTAG00000040584 | DSC2 | 3 | brown |
| ENSBTAG00000043949 | PAWR | 3 | brown |
| ENSBTAG00000043959 | PDGFC | 3 | brown |
| ENSBTAG00000043961 | MPDZ | 3 | brown |
| ENSBTAG00000043963 | FHDC1 | 3 | brown |
| ENSBTAG00000044007 | KLF12 | 3 | brown |
| ENSBTAG00000044022 | KIAA1671 | 3 | brown |
| ENSBTAG00000044061 | FAXC | 3 | brown |
| ENSBTAG00000044064 | RNF144A | 3 | brown |
| ENSBTAG00000044153 | SH3PXD2A | 3 | brown |
| ENSBTAG00000044155 | BSPRY | 3 | brown |
| ENSBTAG00000044161 | NMU | 3 | brown |
| ENSBTAG00000044173 | EHBP1 | 3 | brown |
| ENSBTAG00000044240 | NA | 3 | brown |
| ENSBTAG00000045500 | SGSM2 | 3 | brown |
| ENSBTAG00000045507 | NA | 3 | brown |
| ENSBTAG00000045550 | TSPAN6 | 3 | brown |
| ENSBTAG00000045567 | PRR15 | 3 | brown |

| | | | |
|---|---|---|---|
| ENSBTAG00000045584 | NA | 3 | brown |
| ENSBTAG00000045727 | EPPK1 | 3 | brown |
| ENSBTAG00000045787 | CDS1 | 3 | brown |
| ENSBTAG00000045824 | SOX9 | 3 | brown |
| ENSBTAG00000045834 | RASSF7 | 3 | brown |
| ENSBTAG00000045859 | NA | 3 | brown |
| ENSBTAG00000045879 | CALHM5 | 3 | brown |
| ENSBTAG00000045886 | NA | 3 | brown |
| ENSBTAG00000045901 | CLDN6 | 3 | brown |
| ENSBTAG00000045976 | NA | 3 | brown |
| ENSBTAG00000045989 | CDC42EP5 | 3 | brown |
| ENSBTAG00000046029 | NA | 3 | brown |
| ENSBTAG00000046110 | CLCF1 | 3 | brown |
| ENSBTAG00000046121 | LOC100196897 | 3 | brown |
| ENSBTAG00000046129 | RAB34 | 3 | brown |
| ENSBTAG00000046140 | NPDC1 | 3 | brown |
| ENSBTAG00000046176 | SPEG | 3 | brown |
| ENSBTAG00000046323 | NA | 3 | brown |
| ENSBTAG00000046350 | PKP1 | 3 | brown |
| ENSBTAG00000046364 | NA | 3 | brown |
| ENSBTAG00000046389 | KLRD1 | 3 | brown |
| ENSBTAG00000046467 | PTP4A3 | 3 | brown |
| ENSBTAG00000046482 | LOC538679 | 3 | brown |
| ENSBTAG00000046587 | CST6 | 3 | brown |
| ENSBTAG00000046758 | RNF223 | 3 | brown |
| ENSBTAG00000046852 | NA | 3 | brown |
| ENSBTAG00000046934 | NA | 3 | brown |
| ENSBTAG00000046957 | DOK6 | 3 | brown |
| ENSBTAG00000046971 | NA | 3 | brown |
| ENSBTAG00000047000 | NA | 3 | brown |
| ENSBTAG00000047014 | NA | 3 | brown |
| ENSBTAG00000047036 | GPR39 | 3 | brown |
| ENSBTAG00000047116 | NA | 3 | brown |
| ENSBTAG00000047148 | NA | 3 | brown |
| ENSBTAG00000047229 | CRIP1 | 3 | brown |
| ENSBTAG00000047363 | NA | 3 | brown |
| ENSBTAG00000047406 | NA | 3 | brown |
| ENSBTAG00000047421 | NA | 3 | brown |
| ENSBTAG00000047499 | IRX2 | 3 | brown |
| ENSBTAG00000047547 | NA | 3 | brown |
| ENSBTAG00000047560 | C2CD4C | 3 | brown |
| ENSBTAG00000047685 | NA | 3 | brown |
| ENSBTAG00000047697 | NA | 3 | brown |
| ENSBTAG00000047727 | NA | 3 | brown |
| ENSBTAG00000047879 | KCNA3 | 3 | brown |
| ENSBTAG00000047880 | NA | 3 | brown |
| ENSBTAG00000047894 | SLC32A1 | 3 | brown |

| | | | |
|---|---|---|---|
| ENSBTAG00000047902 | ULBP21 | 3 | brown |
| ENSBTAG00000048098 | PKP3 | 3 | brown |
| ENSBTAG00000048112 | NA | 3 | brown |
| ENSBTAG00000048125 | ENTPD2 | 3 | brown |
| ENSBTAG00000048171 | TAP | 3 | brown |
| ENSBTAG00000048188 | NA | 3 | brown |
| ENSBTAG00000048228 | SNHG12 | 3 | brown |
| ENSBTAG00000048230 | NA | 3 | brown |
| ENSBTAG00000000778 | HSP90AB1 | 14 | cyan |
| ENSBTAG00000001107 | CACYBP | 14 | cyan |
| ENSBTAG00000003557 | CCDC117 | 14 | cyan |
| ENSBTAG00000005012 | HSPH1 | 14 | cyan |
| ENSBTAG00000005793 | PEA15 | 14 | cyan |
| ENSBTAG00000006025 | AHSA2 | 14 | cyan |
| ENSBTAG00000006270 | HSP90AA1 | 14 | cyan |
| ENSBTAG00000007127 | NA | 14 | cyan |
| ENSBTAG00000007605 | FKBP4 | 14 | cyan |
| ENSBTAG00000007784 | NAA50 | 14 | cyan |
| ENSBTAG00000008105 | RBM38 | 14 | cyan |
| ENSBTAG00000008184 | CCT6A | 14 | cyan |
| ENSBTAG00000008346 | NA | 14 | cyan |
| ENSBTAG00000008705 | CABLES1 | 14 | cyan |
| ENSBTAG00000009459 | MTCL1 | 14 | cyan |
| ENSBTAG00000012508 | NECAB2 | 14 | cyan |
| ENSBTAG00000012586 | HSPD1 | 14 | cyan |
| ENSBTAG00000012589 | HSPE1 | 14 | cyan |
| ENSBTAG00000013162 | HSPA8 | 14 | cyan |
| ENSBTAG00000013615 | CHORDC1 | 14 | cyan |
| ENSBTAG00000013641 | BAG3 | 14 | cyan |
| ENSBTAG00000014233 | CCT8 | 14 | cyan |
| ENSBTAG00000015205 | NCK2 | 14 | cyan |
| ENSBTAG00000015582 | HMOX1 | 14 | cyan |
| ENSBTAG00000015663 | STIP1 | 14 | cyan |
| ENSBTAG00000015683 | HSPA4 | 14 | cyan |
| ENSBTAG00000015692 | HSPA4L | 14 | cyan |
| ENSBTAG00000015942 | DNAJA4 | 14 | cyan |
| ENSBTAG00000016265 | DNAJA1 | 14 | cyan |
| ENSBTAG00000016650 | TIGAR | 14 | cyan |
| ENSBTAG00000016680 | PPID | 14 | cyan |
| ENSBTAG00000016874 | DNAJB1 | 14 | cyan |
| ENSBTAG00000017143 | PDIA4 | 14 | cyan |
| ENSBTAG00000017967 | PTGES3 | 14 | cyan |
| ENSBTAG00000019156 | CCT2 | 14 | cyan |
| ENSBTAG00000019422 | NA | 14 | cyan |
| ENSBTAG00000019464 | NA | 14 | cyan |
| ENSBTAG00000020477 | AHSA1 | 14 | cyan |
| ENSBTAG00000021202 | ACOT7 | 14 | cyan |

| | | | |
|---|---|---|---|
| ENSBTAG00000022161 | TLR7 | 14 | cyan |
| ENSBTAG00000025441 | HSPA1A | 14 | cyan |
| ENSBTAG00000025442 | HSPA1L | 14 | cyan |
| ENSBTAG00000027803 | NA | 14 | cyan |
| ENSBTAG00000032996 | P4HA1 | 14 | cyan |
| ENSBTAG00000038916 | NA | 14 | cyan |
| ENSBTAG00000039035 | NA | 14 | cyan |
| ENSBTAG00000039130 | LOC613956 | 14 | cyan |
| ENSBTAG00000045858 | LOC504207 | 14 | cyan |
| ENSBTAG00000046509 | TENT5C | 14 | cyan |
| ENSBTAG00000046677 | RTN4R | 14 | cyan |
| ENSBTAG00000047008 | NA | 14 | cyan |
| ENSBTAG00000047441 | NA | 14 | cyan |
| ENSBTAG00000047699 | NA | 14 | cyan |
| ENSBTAG00000000040 | MAFG | 5 | green |
| ENSBTAG00000000250 | HDHD3 | 5 | green |
| ENSBTAG00000000332 | ERF | 5 | green |
| ENSBTAG00000000429 | DGCR2 | 5 | green |
| ENSBTAG00000000625 | SMAD6 | 5 | green |
| ENSBTAG00000000658 | NA | 5 | green |
| ENSBTAG00000000662 | TCF20 | 5 | green |
| ENSBTAG00000000683 | WDR37 | 5 | green |
| ENSBTAG00000000710 | PIP5K1C | 5 | green |
| ENSBTAG00000000781 | HIP1 | 5 | green |
| ENSBTAG00000000944 | LPCAT1 | 5 | green |
| ENSBTAG00000001022 | AMDHD2 | 5 | green |
| ENSBTAG00000001023 | ZNF598 | 5 | green |
| ENSBTAG00000001110 | SLC16A5 | 5 | green |
| ENSBTAG00000001301 | LRRC32 | 5 | green |
| ENSBTAG00000001414 | KCTD12 | 5 | green |
| ENSBTAG00000001465 | P2RY1 | 5 | green |
| ENSBTAG00000001470 | DTX2 | 5 | green |
| ENSBTAG00000001529 | NSD3 | 5 | green |
| ENSBTAG00000001551 | PLEKHG3 | 5 | green |
| ENSBTAG00000001567 | LRRK1 | 5 | green |
| ENSBTAG00000001616 | ZNF592 | 5 | green |
| ENSBTAG00000001618 | ALPK3 | 5 | green |
| ENSBTAG00000001647 | PLEKHJ1 | 5 | green |
| ENSBTAG00000001712 | CMTM7 | 5 | green |
| ENSBTAG00000001834 | PNKP | 5 | green |
| ENSBTAG00000001867 | SACS | 5 | green |
| ENSBTAG00000001888 | MED13L | 5 | green |
| ENSBTAG00000001904 | ZNF276 | 5 | green |
| ENSBTAG00000001931 | TRABD | 5 | green |
| ENSBTAG00000002202 | CRAMP1 | 5 | green |
| ENSBTAG00000002249 | NAALADL1 | 5 | green |
| ENSBTAG00000002345 | SETD1A | 5 | green |

| | | | |
|---|---|---|---|
| ENSBTAG00000002347 | MAST3 | 5 | green |
| ENSBTAG00000002381 | ZDHHC5 | 5 | green |
| ENSBTAG00000002385 | SLC35E4 | 5 | green |
| ENSBTAG00000002445 | NA | 5 | green |
| ENSBTAG00000002495 | BRD4 | 5 | green |
| ENSBTAG00000002630 | MRTFA | 5 | green |
| ENSBTAG00000002682 | MARK4 | 5 | green |
| ENSBTAG00000002683 | PFKP | 5 | green |
| ENSBTAG00000002702 | TSPAN32 | 5 | green |
| ENSBTAG00000002763 | KMT2B | 5 | green |
| ENSBTAG00000002774 | PTPN23 | 5 | green |
| ENSBTAG00000002809 | CAPN10 | 5 | green |
| ENSBTAG00000002830 | U2AF2 | 5 | green |
| ENSBTAG00000002904 | ZNF787 | 5 | green |
| ENSBTAG00000002923 | CX3CR1 | 5 | green |
| ENSBTAG00000002939 | FURIN | 5 | green |
| ENSBTAG00000003148 | MCM3AP | 5 | green |
| ENSBTAG00000003160 | LRCH4 | 5 | green |
| ENSBTAG00000003169 | FBXO24 | 5 | green |
| ENSBTAG00000003298 | LZTS2 | 5 | green |
| ENSBTAG00000003390 | SF3A1 | 5 | green |
| ENSBTAG00000003396 | MAFB | 5 | green |
| ENSBTAG00000003682 | DNAJB13 | 5 | green |
| ENSBTAG00000003712 | FAM89B | 5 | green |
| ENSBTAG00000003741 | NEURL1 | 5 | green |
| ENSBTAG00000003754 | NA | 5 | green |
| ENSBTAG00000003759 | PCNX3 | 5 | green |
| ENSBTAG00000003889 | PER1 | 5 | green |
| ENSBTAG00000003891 | NA | 5 | green |
| ENSBTAG00000003953 | ZNF316 | 5 | green |
| ENSBTAG00000004015 | KAT6A | 5 | green |
| ENSBTAG00000004017 | PLEKHG2 | 5 | green |
| ENSBTAG00000004029 | NA | 5 | green |
| ENSBTAG00000004091 | GTF3C4 | 5 | green |
| ENSBTAG00000004114 | MAP3K11 | 5 | green |
| ENSBTAG00000004138 | BICRA | 5 | green |
| ENSBTAG00000004172 | SETD1B | 5 | green |
| ENSBTAG00000004316 | BOD1L1 | 5 | green |
| ENSBTAG00000004343 | CCS | 5 | green |
| ENSBTAG00000004400 | PRRC2B | 5 | green |
| ENSBTAG00000004475 | CDK5R1 | 5 | green |
| ENSBTAG00000004650 | ZNF516 | 5 | green |
| ENSBTAG00000004742 | RUNX1 | 5 | green |
| ENSBTAG00000004746 | HELZ | 5 | green |
| ENSBTAG00000004768 | RAI1 | 5 | green |
| ENSBTAG00000004797 | TRMT44 | 5 | green |
| ENSBTAG00000004802 | CEP170B | 5 | green |

| | | | |
|---|---|---|---|
| ENSBTAG00000004901 | PIK3C2A | 5 | green |
| ENSBTAG00000004962 | BCORL1 | 5 | green |
| ENSBTAG00000005042 | SYN1 | 5 | green |
| ENSBTAG00000005138 | BCL9L | 5 | green |
| ENSBTAG00000005145 | PEAK1 | 5 | green |
| ENSBTAG00000005259 | UCP3 | 5 | green |
| ENSBTAG00000005262 | NA | 5 | green |
| ENSBTAG00000005289 | CHERP | 5 | green |
| ENSBTAG00000005382 | NA | 5 | green |
| ENSBTAG00000005496 | RP2 | 5 | green |
| ENSBTAG00000005580 | SZT2 | 5 | green |
| ENSBTAG00000005615 | CEACAM1 | 5 | green |
| ENSBTAG00000005688 | MRPS2 | 5 | green |
| ENSBTAG00000005934 | TTYH3 | 5 | green |
| ENSBTAG00000005951 | HIC2 | 5 | green |
| ENSBTAG00000005980 | RREB1 | 5 | green |
| ENSBTAG00000006122 | HUWE1 | 5 | green |
| ENSBTAG00000006124 | MAU2 | 5 | green |
| ENSBTAG00000006126 | GTF3C1 | 5 | green |
| ENSBTAG00000006500 | RBM15B | 5 | green |
| ENSBTAG00000006511 | MTF1 | 5 | green |
| ENSBTAG00000006612 | SCAF1 | 5 | green |
| ENSBTAG00000006740 | SCYL1 | 5 | green |
| ENSBTAG00000006784 | PLA2G4B | 5 | green |
| ENSBTAG00000006817 | CBL | 5 | green |
| ENSBTAG00000006848 | DHX34 | 5 | green |
| ENSBTAG00000006896 | ABCA9 | 5 | green |
| ENSBTAG00000006905 | KLHDC4 | 5 | green |
| ENSBTAG00000006938 | PPM1F | 5 | green |
| ENSBTAG00000007058 | ATG2A | 5 | green |
| ENSBTAG00000007070 | BLOC1S3 | 5 | green |
| ENSBTAG00000007084 | MAP3K14 | 5 | green |
| ENSBTAG00000007318 | ZNF687 | 5 | green |
| ENSBTAG00000007402 | ZFAND2A | 5 | green |
| ENSBTAG00000007498 | ZGPAT | 5 | green |
| ENSBTAG00000007507 | FAAH | 5 | green |
| ENSBTAG00000007546 | NA | 5 | green |
| ENSBTAG00000007609 | ITFG2 | 5 | green |
| ENSBTAG00000007634 | HOOK3 | 5 | green |
| ENSBTAG00000007793 | NA | 5 | green |
| ENSBTAG00000007825 | MARK2 | 5 | green |
| ENSBTAG00000007829 | CSAD | 5 | green |
| ENSBTAG00000007884 | SREBF1 | 5 | green |
| ENSBTAG00000007909 | NOTCH2 | 5 | green |
| ENSBTAG00000007966 | TTLL3 | 5 | green |
| ENSBTAG00000007998 | TMCO4 | 5 | green |
| ENSBTAG00000008125 | KCNAB2 | 5 | green |

| | | | |
|---|---|---|---|
| ENSBTAG00000008151 | MAP3K3 | 5 | green |
| ENSBTAG00000008237 | TOLLIP | 5 | green |
| ENSBTAG00000008309 | ATP13A2 | 5 | green |
| ENSBTAG00000008313 | KLF15 | 5 | green |
| ENSBTAG00000008348 | NOL4L | 5 | green |
| ENSBTAG00000008350 | KLHL21 | 5 | green |
| ENSBTAG00000008362 | CTDSP1 | 5 | green |
| ENSBTAG00000008366 | STAC2 | 5 | green |
| ENSBTAG00000008385 | KLHL36 | 5 | green |
| ENSBTAG00000008395 | APBA3 | 5 | green |
| ENSBTAG00000008443 | LARP1 | 5 | green |
| ENSBTAG00000008507 | SPHK1 | 5 | green |
| ENSBTAG00000008535 | SOCS7 | 5 | green |
| ENSBTAG00000008579 | RCC2 | 5 | green |
| ENSBTAG00000008591 | CAMTA2 | 5 | green |
| ENSBTAG00000008607 | ARID3A | 5 | green |
| ENSBTAG00000008695 | TCF3 | 5 | green |
| ENSBTAG00000008794 | ATF6B | 5 | green |
| ENSBTAG00000008854 | RFX1 | 5 | green |
| ENSBTAG00000009006 | PISD | 5 | green |
| ENSBTAG00000009078 | MIA | 5 | green |
| ENSBTAG00000009101 | SH2B3 | 5 | green |
| ENSBTAG00000009112 | TRAM2 | 5 | green |
| ENSBTAG00000009167 | SLC15A4 | 5 | green |
| ENSBTAG00000009284 | GLS2 | 5 | green |
| ENSBTAG00000009286 | ZC3H4 | 5 | green |
| ENSBTAG00000009376 | DPH1 | 5 | green |
| ENSBTAG00000009428 | GAN | 5 | green |
| ENSBTAG00000009668 | TMEM184B | 5 | green |
| ENSBTAG00000009918 | GSE1 | 5 | green |
| ENSBTAG00000009996 | DOT1L | 5 | green |
| ENSBTAG00000010052 | EXD2 | 5 | green |
| ENSBTAG00000010126 | PCIF1 | 5 | green |
| ENSBTAG00000010130 | ZNF335 | 5 | green |
| ENSBTAG00000010164 | ESPNL | 5 | green |
| ENSBTAG00000010243 | RBM33 | 5 | green |
| ENSBTAG00000010254 | MAP3K6 | 5 | green |
| ENSBTAG00000010264 | FBXL18 | 5 | green |
| ENSBTAG00000010344 | SLC16A14 | 5 | green |
| ENSBTAG00000010373 | CACTIN | 5 | green |
| ENSBTAG00000010402 | MYH9 | 5 | green |
| ENSBTAG00000010416 | RIN3 | 5 | green |
| ENSBTAG00000010442 | PANK1 | 5 | green |
| ENSBTAG00000010467 | SUPT5H | 5 | green |
| ENSBTAG00000010482 | PITPNM1 | 5 | green |
| ENSBTAG00000010515 | FBXW5 | 5 | green |
| ENSBTAG00000010616 | MBD6 | 5 | green |

| | | | |
|---|---|---|---|
| ENSBTAG00000010639 | MAP2K7 | 5 | green |
| ENSBTAG00000010659 | CUX1 | 5 | green |
| ENSBTAG00000010668 | GEMIN7 | 5 | green |
| ENSBTAG00000010729 | KRI1 | 5 | green |
| ENSBTAG00000010802 | KCNK7 | 5 | green |
| ENSBTAG00000010882 | ANKRD9 | 5 | green |
| ENSBTAG00000010915 | CRTC3 | 5 | green |
| ENSBTAG00000010949 | PROSER1 | 5 | green |
| ENSBTAG00000011064 | ADCK5 | 5 | green |
| ENSBTAG00000011067 | IL17RA | 5 | green |
| ENSBTAG00000011125 | MYO9B | 5 | green |
| ENSBTAG00000011189 | TJAP1 | 5 | green |
| ENSBTAG00000011190 | FLNA | 5 | green |
| ENSBTAG00000011214 | THAP3 | 5 | green |
| ENSBTAG00000011234 | FOXO3 | 5 | green |
| ENSBTAG00000011322 | HIPK1 | 5 | green |
| ENSBTAG00000011387 | NA | 5 | green |
| ENSBTAG00000011466 | NAXD | 5 | green |
| ENSBTAG00000011482 | SLC43A2 | 5 | green |
| ENSBTAG00000011491 | WDR81 | 5 | green |
| ENSBTAG00000011517 | CCDC106 | 5 | green |
| ENSBTAG00000011532 | MLLT6 | 5 | green |
| ENSBTAG00000011543 | PDCD1 | 5 | green |
| ENSBTAG00000011639 | STK11 | 5 | green |
| ENSBTAG00000011693 | LENG9 | 5 | green |
| ENSBTAG00000011730 | TCAP | 5 | green |
| ENSBTAG00000011744 | USP31 | 5 | green |
| ENSBTAG00000011904 | HCFC1 | 5 | green |
| ENSBTAG00000011912 | SLC25A25 | 5 | green |
| ENSBTAG00000011922 | PLEC | 5 | green |
| ENSBTAG00000012101 | SYMPK | 5 | green |
| ENSBTAG00000012107 | SLC25A28 | 5 | green |
| ENSBTAG00000012197 | AP5Z1 | 5 | green |
| ENSBTAG00000012284 | CLCN2 | 5 | green |
| ENSBTAG00000012384 | TFEB | 5 | green |
| ENSBTAG00000012504 | GAS2L1 | 5 | green |
| ENSBTAG00000012505 | ARHGEF17 | 5 | green |
| ENSBTAG00000012577 | UVSSA | 5 | green |
| ENSBTAG00000012657 | ZSWIM8 | 5 | green |
| ENSBTAG00000012719 | UBTD1 | 5 | green |
| ENSBTAG00000012753 | BRF1 | 5 | green |
| ENSBTAG00000012777 | SRF | 5 | green |
| ENSBTAG00000012944 | USP35 | 5 | green |
| ENSBTAG00000013271 | NCOR1 | 5 | green |
| ENSBTAG00000013281 | SPATA21 | 5 | green |
| ENSBTAG00000013468 | AHNAK | 5 | green |
| ENSBTAG00000013495 | WDFY3 | 5 | green |

| | | | |
|---|---|---|---|
| ENSBTAG00000013556 | UNC13D | 5 | green |
| ENSBTAG00000013645 | RFFL | 5 | green |
| ENSBTAG00000013697 | CLASRP | 5 | green |
| ENSBTAG00000013716 | NR2C2 | 5 | green |
| ENSBTAG00000013725 | RBSN | 5 | green |
| ENSBTAG00000013792 | UBALD2 | 5 | green |
| ENSBTAG00000013860 | GADD45A | 5 | green |
| ENSBTAG00000013884 | SIPA1 | 5 | green |
| ENSBTAG00000013917 | NA | 5 | green |
| ENSBTAG00000013943 | ZNF703 | 5 | green |
| ENSBTAG00000013958 | PAK4 | 5 | green |
| ENSBTAG00000013971 | PI4KA | 5 | green |
| ENSBTAG00000014117 | CCDC9 | 5 | green |
| ENSBTAG00000014265 | SREBF2 | 5 | green |
| ENSBTAG00000014267 | ZCCHC14 | 5 | green |
| ENSBTAG00000014407 | INO80D | 5 | green |
| ENSBTAG00000014429 | KMT2D | 5 | green |
| ENSBTAG00000014447 | RPS6KA1 | 5 | green |
| ENSBTAG00000014769 | GMEB2 | 5 | green |
| ENSBTAG00000014806 | ATP2A3 | 5 | green |
| ENSBTAG00000014900 | CNOT3 | 5 | green |
| ENSBTAG00000014943 | KDM5C | 5 | green |
| ENSBTAG00000014982 | TEKTIP1 | 5 | green |
| ENSBTAG00000015013 | C2CD2L | 5 | green |
| ENSBTAG00000015026 | ABL2 | 5 | green |
| ENSBTAG00000015208 | CARMIL3 | 5 | green |
| ENSBTAG00000015240 | UBR4 | 5 | green |
| ENSBTAG00000015272 | TSC22D4 | 5 | green |
| ENSBTAG00000015401 | ATG4B | 5 | green |
| ENSBTAG00000015604 | ZNF385A | 5 | green |
| ENSBTAG00000015667 | TMEM127 | 5 | green |
| ENSBTAG00000015721 | RAVER1 | 5 | green |
| ENSBTAG00000015739 | MRC2 | 5 | green |
| ENSBTAG00000015801 | EFNB1 | 5 | green |
| ENSBTAG00000015802 | CREB3L2 | 5 | green |
| ENSBTAG00000015808 | ZNF609 | 5 | green |
| ENSBTAG00000015856 | CFAP126 | 5 | green |
| ENSBTAG00000015908 | MBOAT7 | 5 | green |
| ENSBTAG00000015982 | KCTD13 | 5 | green |
| ENSBTAG00000015984 | CADM4 | 5 | green |
| ENSBTAG00000015988 | MYH11 | 5 | green |
| ENSBTAG00000016006 | ANKRD11 | 5 | green |
| ENSBTAG00000016080 | VPS13D | 5 | green |
| ENSBTAG00000016085 | IRAK1 | 5 | green |
| ENSBTAG00000016128 | GGA3 | 5 | green |
| ENSBTAG00000016155 | GDPD3 | 5 | green |
| ENSBTAG00000016198 | EP300 | 5 | green |

| | | | |
|---|---|---|---|
| ENSBTAG00000016254 | HDAC5 | 5 | green |
| ENSBTAG00000016316 | ZZEF1 | 5 | green |
| ENSBTAG00000016448 | ZBTB40 | 5 | green |
| ENSBTAG00000016553 | EME2 | 5 | green |
| ENSBTAG00000016558 | SPSB3 | 5 | green |
| ENSBTAG00000016577 | AXIN1 | 5 | green |
| ENSBTAG00000016592 | CAPN15 | 5 | green |
| ENSBTAG00000016722 | PGGHG | 5 | green |
| ENSBTAG00000016757 | ZKSCAN1 | 5 | green |
| ENSBTAG00000016776 | BAHCC1 | 5 | green |
| ENSBTAG00000016797 | CSNK1G2 | 5 | green |
| ENSBTAG00000016799 | ERN1 | 5 | green |
| ENSBTAG00000016890 | ANKS1A | 5 | green |
| ENSBTAG00000017090 | SMCR8 | 5 | green |
| ENSBTAG00000017306 | AKNA | 5 | green |
| ENSBTAG00000017371 | RELL2 | 5 | green |
| ENSBTAG00000017459 | DUSP18 | 5 | green |
| ENSBTAG00000017486 | ADARB1 | 5 | green |
| ENSBTAG00000017517 | ZNF236 | 5 | green |
| ENSBTAG00000017746 | SLIT3 | 5 | green |
| ENSBTAG00000017851 | RXRA | 5 | green |
| ENSBTAG00000017921 | NELFA | 5 | green |
| ENSBTAG00000018048 | MED15 | 5 | green |
| ENSBTAG00000018093 | KMT2A | 5 | green |
| ENSBTAG00000018105 | ACVR2B | 5 | green |
| ENSBTAG00000018270 | NFATC2 | 5 | green |
| ENSBTAG00000018324 | AGO3 | 5 | green |
| ENSBTAG00000018449 | NUMA1 | 5 | green |
| ENSBTAG00000018488 | AFF1 | 5 | green |
| ENSBTAG00000018524 | AMHR2 | 5 | green |
| ENSBTAG00000018534 | ESS2 | 5 | green |
| ENSBTAG00000018658 | TNRC6C | 5 | green |
| ENSBTAG00000018660 | PPP6R2 | 5 | green |
| ENSBTAG00000018661 | TMC6 | 5 | green |
| ENSBTAG00000018711 | DENND4B | 5 | green |
| ENSBTAG00000018729 | SPEN | 5 | green |
| ENSBTAG00000018732 | HSPA12B | 5 | green |
| ENSBTAG00000018744 | MGAT5 | 5 | green |
| ENSBTAG00000018761 | SLC35F6 | 5 | green |
| ENSBTAG00000018828 | ATN1 | 5 | green |
| ENSBTAG00000018905 | MAN2A2 | 5 | green |
| ENSBTAG00000018946 | NIBAN3 | 5 | green |
| ENSBTAG00000018999 | MGRN1 | 5 | green |
| ENSBTAG00000019040 | PLBD2 | 5 | green |
| ENSBTAG00000019061 | ELF4 | 5 | green |
| ENSBTAG00000019174 | ZNF710 | 5 | green |
| ENSBTAG00000019188 | GAK | 5 | green |

| | | | |
|---|---|---|---|
| ENSBTAG00000019193 | SETD5 | 5 | green |
| ENSBTAG00000019197 | TASOR2 | 5 | green |
| ENSBTAG00000019310 | FOXK1 | 5 | green |
| ENSBTAG00000019329 | LYL1 | 5 | green |
| ENSBTAG00000019336 | DVL3 | 5 | green |
| ENSBTAG00000019462 | COQ8B | 5 | green |
| ENSBTAG00000019519 | PWWP2B | 5 | green |
| ENSBTAG00000019524 | ITGAD | 5 | green |
| ENSBTAG00000019550 | TBC1D2 | 5 | green |
| ENSBTAG00000019569 | CD151 | 5 | green |
| ENSBTAG00000019767 | MIER2 | 5 | green |
| ENSBTAG00000019783 | NA | 5 | green |
| ENSBTAG00000019785 | CIC | 5 | green |
| ENSBTAG00000019844 | ZNF467 | 5 | green |
| ENSBTAG00000019900 | LNPEP | 5 | green |
| ENSBTAG00000019988 | GNA15 | 5 | green |
| ENSBTAG00000020117 | ZBTB7A | 5 | green |
| ENSBTAG00000020232 | REXO1 | 5 | green |
| ENSBTAG00000020312 | NCOA2 | 5 | green |
| ENSBTAG00000020414 | DHX37 | 5 | green |
| ENSBTAG00000020423 | EP400 | 5 | green |
| ENSBTAG00000020433 | NLRP1 | 5 | green |
| ENSBTAG00000020543 | UPK1A | 5 | green |
| ENSBTAG00000020552 | PRDM2 | 5 | green |
| ENSBTAG00000020700 | CLCN6 | 5 | green |
| ENSBTAG00000020835 | KIAA0513 | 5 | green |
| ENSBTAG00000021000 | USF3 | 5 | green |
| ENSBTAG00000021052 | GNB1L | 5 | green |
| ENSBTAG00000021064 | NA | 5 | green |
| ENSBTAG00000021090 | ABCC1 | 5 | green |
| ENSBTAG00000021111 | POU5F1 | 5 | green |
| ENSBTAG00000021115 | ZNF692 | 5 | green |
| ENSBTAG00000021121 | PPIL2 | 5 | green |
| ENSBTAG00000021130 | MFSD10 | 5 | green |
| ENSBTAG00000021134 | DPP9 | 5 | green |
| ENSBTAG00000021157 | NA | 5 | green |
| ENSBTAG00000021253 | PRDM15 | 5 | green |
| ENSBTAG00000021337 | SBF1 | 5 | green |
| ENSBTAG00000021351 | MED12 | 5 | green |
| ENSBTAG00000021364 | TNK2 | 5 | green |
| ENSBTAG00000021424 | CSK | 5 | green |
| ENSBTAG00000021430 | UPK3B | 5 | green |
| ENSBTAG00000021639 | ARC | 5 | green |
| ENSBTAG00000021709 | SPI1 | 5 | green |
| ENSBTAG00000021811 | ANGPT4 | 5 | green |
| ENSBTAG00000021837 | RGS19 | 5 | green |
| ENSBTAG00000021857 | ARHGAP21 | 5 | green |

| | | | |
|---|---|---|---|
| ENSBTAG00000021884 | CXXC1 | 5 | green |
| ENSBTAG00000021919 | NAV1 | 5 | green |
| ENSBTAG00000022039 | CMKLR1 | 5 | green |
| ENSBTAG00000022185 | IGHMBP2 | 5 | green |
| ENSBTAG00000022381 | TET3 | 5 | green |
| ENSBTAG00000022799 | NOTCH1 | 5 | green |
| ENSBTAG00000023562 | KCNK6 | 5 | green |
| ENSBTAG00000023788 | EXD3 | 5 | green |
| ENSBTAG00000023832 | ADAM8 | 5 | green |
| ENSBTAG00000023933 | SEC16A | 5 | green |
| ENSBTAG00000024199 | KMT2C | 5 | green |
| ENSBTAG00000024450 | MAP2K2 | 5 | green |
| ENSBTAG00000024534 | POU2F1 | 5 | green |
| ENSBTAG00000024603 | NCOR2 | 5 | green |
| ENSBTAG00000024960 | LOC512286 | 5 | green |
| ENSBTAG00000025277 | ABHD17A | 5 | green |
| ENSBTAG00000025329 | IRF2BPL | 5 | green |
| ENSBTAG00000025452 | SF3A2 | 5 | green |
| ENSBTAG00000025666 | LOC112448430 | 5 | green |
| ENSBTAG00000025868 | TLN1 | 5 | green |
| ENSBTAG00000026356 | DGAT1 | 5 | green |
| ENSBTAG00000026769 | ASCL2 | 5 | green |
| ENSBTAG00000026986 | NA | 5 | green |
| ENSBTAG00000030319 | GBGT1 | 5 | green |
| ENSBTAG00000030334 | AOC2 | 5 | green |
| ENSBTAG00000030369 | IFFO1 | 5 | green |
| ENSBTAG00000030517 | SMIM33 | 5 | green |
| ENSBTAG00000030556 | ZNF217 | 5 | green |
| ENSBTAG00000030704 | NA | 5 | green |
| ENSBTAG00000031402 | ASXL1 | 5 | green |
| ENSBTAG00000031524 | NA | 5 | green |
| ENSBTAG00000031673 | ARRDC5 | 5 | green |
| ENSBTAG00000031852 | TBC1D10B | 5 | green |
| ENSBTAG00000032253 | RAD54L2 | 5 | green |
| ENSBTAG00000032427 | FHOD1 | 5 | green |
| ENSBTAG00000032503 | ITPRIPL2 | 5 | green |
| ENSBTAG00000034113 | GALNT10 | 5 | green |
| ENSBTAG00000035144 | PLCXD1 | 5 | green |
| ENSBTAG00000035587 | ARRB2 | 5 | green |
| ENSBTAG00000037383 | AKAP13 | 5 | green |
| ENSBTAG00000037400 | TNRC18 | 5 | green |
| ENSBTAG00000037581 | MZF1 | 5 | green |
| ENSBTAG00000038034 | ZNF628 | 5 | green |
| ENSBTAG00000038055 | KLF16 | 5 | green |
| ENSBTAG00000038316 | GPATCH8 | 5 | green |
| ENSBTAG00000038381 | SLC26A6 | 5 | green |
| ENSBTAG00000038613 | NA | 5 | green |

| | | | |
|---|---|---|---|
| ENSBTAG00000038716 | SKI | 5 | green |
| ENSBTAG00000038992 | STK11IP | 5 | green |
| ENSBTAG00000039453 | LOC100847773 | 5 | green |
| ENSBTAG00000039766 | FBRSL1 | 5 | green |
| ENSBTAG00000039784 | PRR5 | 5 | green |
| ENSBTAG00000039789 | NA | 5 | green |
| ENSBTAG00000040334 | MEX3D | 5 | green |
| ENSBTAG00000044492 | NA | 5 | green |
| ENSBTAG00000045547 | NA | 5 | green |
| ENSBTAG00000045656 | NA | 5 | green |
| ENSBTAG00000045862 | FERMT3 | 5 | green |
| ENSBTAG00000046012 | SOWAHD | 5 | green |
| ENSBTAG00000046054 | NA | 5 | green |
| ENSBTAG00000046062 | NA | 5 | green |
| ENSBTAG00000046174 | GNA13 | 5 | green |
| ENSBTAG00000046223 | ANAPC2 | 5 | green |
| ENSBTAG00000046239 | LCOR | 5 | green |
| ENSBTAG00000046255 | ATXN1L | 5 | green |
| ENSBTAG00000046295 | CCER2 | 5 | green |
| ENSBTAG00000046332 | ACTA1 | 5 | green |
| ENSBTAG00000046359 | PHRF1 | 5 | green |
| ENSBTAG00000046416 | NA | 5 | green |
| ENSBTAG00000046545 | MEF2D | 5 | green |
| ENSBTAG00000046555 | WAS | 5 | green |
| ENSBTAG00000046640 | NA | 5 | green |
| ENSBTAG00000046655 | NA | 5 | green |
| ENSBTAG00000046664 | TSC22D1 | 5 | green |
| ENSBTAG00000046760 | BRD1 | 5 | green |
| ENSBTAG00000046875 | AP5B1 | 5 | green |
| ENSBTAG00000046996 | EPN1 | 5 | green |
| ENSBTAG00000047061 | NDOR1 | 5 | green |
| ENSBTAG00000047147 | CRYBG3 | 5 | green |
| ENSBTAG00000047274 | KIF21B | 5 | green |
| ENSBTAG00000047531 | ARHGAP4 | 5 | green |
| ENSBTAG00000047633 | NA | 5 | green |
| ENSBTAG00000047675 | LOC618268 | 5 | green |
| ENSBTAG00000047876 | SH3TC1 | 5 | green |
| ENSBTAG00000047883 | NA | 5 | green |
| ENSBTAG00000047991 | TAF4 | 5 | green |
| ENSBTAG00000000552 | DISP2 | 11 | greenyellow |
| ENSBTAG00000000595 | FBN3 | 11 | greenyellow |
| ENSBTAG00000000987 | OTOG | 11 | greenyellow |
| ENSBTAG00000001084 | OR10H1 | 11 | greenyellow |
| ENSBTAG00000002296 | STING1 | 11 | greenyellow |
| ENSBTAG00000002796 | KLHL3 | 11 | greenyellow |
| ENSBTAG00000003088 | LANCL3 | 11 | greenyellow |
| ENSBTAG00000003345 | FAT4 | 11 | greenyellow |

| | | | |
|---|---|---|---|
| ENSBTAG00000003512 | MYH7B | 11 | greenyellow |
| ENSBTAG00000003667 | TLN2 | 11 | greenyellow |
| ENSBTAG00000003877 | ZCCHC24 | 11 | greenyellow |
| ENSBTAG00000003977 | SLC52A3 | 11 | greenyellow |
| ENSBTAG00000004406 | MSI2 | 11 | greenyellow |
| ENSBTAG00000004555 | LRP2 | 11 | greenyellow |
| ENSBTAG00000005019 | PRSS50 | 11 | greenyellow |
| ENSBTAG00000005143 | NA | 11 | greenyellow |
| ENSBTAG00000005953 | ESX1 | 11 | greenyellow |
| ENSBTAG00000006280 | RBFOX3 | 11 | greenyellow |
| ENSBTAG00000006371 | RMND5B | 11 | greenyellow |
| ENSBTAG00000006520 | NA | 11 | greenyellow |
| ENSBTAG00000006621 | NA | 11 | greenyellow |
| ENSBTAG00000006907 | NEB | 11 | greenyellow |
| ENSBTAG00000006961 | NLRP13 | 11 | greenyellow |
| ENSBTAG00000006965 | NLRP8 | 11 | greenyellow |
| ENSBTAG00000007131 | GADL1 | 11 | greenyellow |
| ENSBTAG00000007148 | F2 | 11 | greenyellow |
| ENSBTAG00000007550 | OLFM2 | 11 | greenyellow |
| ENSBTAG00000007558 | IL9R | 11 | greenyellow |
| ENSBTAG00000007882 | CACNA1B | 11 | greenyellow |
| ENSBTAG00000008129 | CLSTN3 | 11 | greenyellow |
| ENSBTAG00000008542 | SSPO | 11 | greenyellow |
| ENSBTAG00000008544 | SALL1 | 11 | greenyellow |
| ENSBTAG00000008718 | GALNT17 | 11 | greenyellow |
| ENSBTAG00000009047 | YPEL3 | 11 | greenyellow |
| ENSBTAG00000009834 | NA | 11 | greenyellow |
| ENSBTAG00000010027 | CHDH | 11 | greenyellow |
| ENSBTAG00000010161 | CCL21 | 11 | greenyellow |
| ENSBTAG00000010353 | ATP7B | 11 | greenyellow |
| ENSBTAG00000010878 | NA | 11 | greenyellow |
| ENSBTAG00000010927 | CBFA2T3 | 11 | greenyellow |
| ENSBTAG00000010978 | PROX1 | 11 | greenyellow |
| ENSBTAG00000011327 | OLFML3 | 11 | greenyellow |
| ENSBTAG00000011706 | TNR | 11 | greenyellow |
| ENSBTAG00000011756 | NA | 11 | greenyellow |
| ENSBTAG00000011803 | MYH3 | 11 | greenyellow |
| ENSBTAG00000011976 | CYP4B1 | 11 | greenyellow |
| ENSBTAG00000012193 | TTC28 | 11 | greenyellow |
| ENSBTAG00000012837 | NA | 11 | greenyellow |
| ENSBTAG00000012987 | ULK1 | 11 | greenyellow |
| ENSBTAG00000013004 | ITIH5 | 11 | greenyellow |
| ENSBTAG00000013105 | SYT3 | 11 | greenyellow |
| ENSBTAG00000013715 | BRINP2 | 11 | greenyellow |
| ENSBTAG00000014042 | TLR10 | 11 | greenyellow |
| ENSBTAG00000014179 | SLC12A5 | 11 | greenyellow |
| ENSBTAG00000014335 | LOC112441470 | 11 | greenyellow |

| | | | |
|---|---|---|---|
| ENSBTAG00000014412 | ELAVL3 | 11 | greenyellow |
| ENSBTAG00000015058 | CDH4 | 11 | greenyellow |
| ENSBTAG00000015255 | ST6GALNAC2 | 11 | greenyellow |
| ENSBTAG00000015980 | FASN | 11 | greenyellow |
| ENSBTAG00000016523 | DNAH10 | 11 | greenyellow |
| ENSBTAG00000016761 | NOX1 | 11 | greenyellow |
| ENSBTAG00000016878 | WDR59 | 11 | greenyellow |
| ENSBTAG00000017785 | FRMPD4 | 11 | greenyellow |
| ENSBTAG00000018052 | PTPRS | 11 | greenyellow |
| ENSBTAG00000018560 | NA | 11 | greenyellow |
| ENSBTAG00000018807 | MYBPHL | 11 | greenyellow |
| ENSBTAG00000019327 | NRAP | 11 | greenyellow |
| ENSBTAG00000019958 | NXNL2 | 11 | greenyellow |
| ENSBTAG00000019977 | PCDH10 | 11 | greenyellow |
| ENSBTAG00000020127 | TKTL1 | 11 | greenyellow |
| ENSBTAG00000020793 | KCNA4 | 11 | greenyellow |
| ENSBTAG00000021177 | ADAMTS14 | 11 | greenyellow |
| ENSBTAG00000022744 | TRIM71 | 11 | greenyellow |
| ENSBTAG00000022807 | KIR3DS1 | 11 | greenyellow |
| ENSBTAG00000023891 | RBM20 | 11 | greenyellow |
| ENSBTAG00000024874 | NA | 11 | greenyellow |
| ENSBTAG00000025021 | LOC614741 | 11 | greenyellow |
| ENSBTAG00000025071 | TENM2 | 11 | greenyellow |
| ENSBTAG00000025597 | GRIN3B | 11 | greenyellow |
| ENSBTAG00000025642 | NA | 11 | greenyellow |
| ENSBTAG00000026032 | ZYG11A | 11 | greenyellow |
| ENSBTAG00000026141 | NA | 11 | greenyellow |
| ENSBTAG00000026531 | NA | 11 | greenyellow |
| ENSBTAG00000028443 | LOC112443528 | 11 | greenyellow |
| ENSBTAG00000028704 | NA | 11 | greenyellow |
| ENSBTAG00000030543 | SLC6A16 | 11 | greenyellow |
| ENSBTAG00000030838 | LOC112446467 | 11 | greenyellow |
| ENSBTAG00000030892 | LOC790134 | 11 | greenyellow |
| ENSBTAG00000031031 | OR10P1 | 11 | greenyellow |
| ENSBTAG00000031359 | NA | 11 | greenyellow |
| ENSBTAG00000031447 | PRSS45 | 11 | greenyellow |
| ENSBTAG00000031810 | WDR88 | 11 | greenyellow |
| ENSBTAG00000032014 | NA | 11 | greenyellow |
| ENSBTAG00000033268 | ZFHX4 | 11 | greenyellow |
| ENSBTAG00000033562 | LHX1 | 11 | greenyellow |
| ENSBTAG00000034613 | MROH6 | 11 | greenyellow |
| ENSBTAG00000036222 | NA | 11 | greenyellow |
| ENSBTAG00000037650 | RTL9 | 11 | greenyellow |
| ENSBTAG00000037821 | LOC112443487 | 11 | greenyellow |
| ENSBTAG00000037826 | LOC407148 | 11 | greenyellow |
| ENSBTAG00000037885 | PCDHB1 | 11 | greenyellow |
| ENSBTAG00000039292 | ADRA2A | 11 | greenyellow |

| | | | |
|---|---|---|---|
| ENSBTAG00000039601 | SAP25 | 11 | greenyellow |
| ENSBTAG00000039803 | MYO7B | 11 | greenyellow |
| ENSBTAG00000040279 | KRT24 | 11 | greenyellow |
| ENSBTAG00000044055 | THSD7B | 11 | greenyellow |
| ENSBTAG00000045518 | NA | 11 | greenyellow |
| ENSBTAG00000045577 | NA | 11 | greenyellow |
| ENSBTAG00000045642 | NA | 11 | greenyellow |
| ENSBTAG00000046150 | NA | 11 | greenyellow |
| ENSBTAG00000046210 | NA | 11 | greenyellow |
| ENSBTAG00000046227 | NA | 11 | greenyellow |
| ENSBTAG00000046333 | C6H4orf54 | 11 | greenyellow |
| ENSBTAG00000047003 | ASXL3 | 11 | greenyellow |
| ENSBTAG00000047122 | NA | 11 | greenyellow |
| ENSBTAG00000047190 | NA | 11 | greenyellow |
| ENSBTAG00000047216 | NA | 11 | greenyellow |
| ENSBTAG00000047282 | NA | 11 | greenyellow |
| ENSBTAG00000047373 | LOC112447652 | 11 | greenyellow |
| ENSBTAG00000047411 | NA | 11 | greenyellow |
| ENSBTAG00000047794 | DNAH1 | 11 | greenyellow |
| ENSBTAG00000048077 | MAGED4B | 11 | greenyellow |
| ENSBTAG00000048245 | NA | 11 | greenyellow |
| ENSBTAG00000000067 | SDF2L1 | 0 | grey |
| ENSBTAG00000000130 | TNFSF13 | 0 | grey |
| ENSBTAG00000000524 | CSTB | 0 | grey |
| ENSBTAG00000000559 | PLTP | 0 | grey |
| ENSBTAG00000000581 | DOCK10 | 0 | grey |
| ENSBTAG00000000718 | TMPRSS9 | 0 | grey |
| ENSBTAG00000000833 | TAX1BP3 | 0 | grey |
| ENSBTAG00000000897 | IQGAP2 | 0 | grey |
| ENSBTAG00000000959 | ZSWIM1 | 0 | grey |
| ENSBTAG00000001066 | PILRA | 0 | grey |
| ENSBTAG00000001083 | MAP3K20 | 0 | grey |
| ENSBTAG00000001191 | CCL17 | 0 | grey |
| ENSBTAG00000001228 | CEND1 | 0 | grey |
| ENSBTAG00000001441 | NA | 0 | grey |
| ENSBTAG00000001570 | CSF2 | 0 | grey |
| ENSBTAG00000001835 | GJA1 | 0 | grey |
| ENSBTAG00000002034 | MFSD2B | 0 | grey |
| ENSBTAG00000002065 | TFAP2C | 0 | grey |
| ENSBTAG00000002199 | CORIN | 0 | grey |
| ENSBTAG00000002219 | ATAD3A | 0 | grey |
| ENSBTAG00000002348 | SLC4A4 | 0 | grey |
| ENSBTAG00000002976 | CD177 | 0 | grey |
| ENSBTAG00000003152 | IFI27 | 0 | grey |
| ENSBTAG00000003200 | CSPG4B | 0 | grey |
| ENSBTAG00000003261 | AURKC | 0 | grey |
| ENSBTAG00000003692 | UCP2 | 0 | grey |

| | | | |
|---|---|---|---|
| ENSBTAG00000003892 | LOC537017 | 0 | grey |
| ENSBTAG00000004038 | ABAT | 0 | grey |
| ENSBTAG00000004297 | ACOXL | 0 | grey |
| ENSBTAG00000004361 | ZBTB37 | 0 | grey |
| ENSBTAG00000004640 | SPAM1 | 0 | grey |
| ENSBTAG00000004657 | NA | 0 | grey |
| ENSBTAG00000004815 | LOC509006 | 0 | grey |
| ENSBTAG00000005115 | SLC31A2 | 0 | grey |
| ENSBTAG00000005244 | RASL11A | 0 | grey |
| ENSBTAG00000005432 | CAPG | 0 | grey |
| ENSBTAG00000005527 | GIPR | 0 | grey |
| ENSBTAG00000005734 | GATA6 | 0 | grey |
| ENSBTAG00000005769 | NPHP3 | 0 | grey |
| ENSBTAG00000005866 | NA | 0 | grey |
| ENSBTAG00000005945 | NXPE4 | 0 | grey |
| ENSBTAG00000005997 | ABCB1 | 0 | grey |
| ENSBTAG00000005998 | NOCT | 0 | grey |
| ENSBTAG00000006036 | ERP27 | 0 | grey |
| ENSBTAG00000006080 | MAST1 | 0 | grey |
| ENSBTAG00000006138 | SEMA3C | 0 | grey |
| ENSBTAG00000006173 | CCDC116 | 0 | grey |
| ENSBTAG00000006233 | NIM1K | 0 | grey |
| ENSBTAG00000006719 | ZFAND4 | 0 | grey |
| ENSBTAG00000006893 | BBX | 0 | grey |
| ENSBTAG00000007169 | P2RX1 | 0 | grey |
| ENSBTAG00000007371 | SCAMP1 | 0 | grey |
| ENSBTAG00000007482 | NA | 0 | grey |
| ENSBTAG00000007654 | CHST7 | 0 | grey |
| ENSBTAG00000007746 | AHR | 0 | grey |
| ENSBTAG00000007996 | HTR6 | 0 | grey |
| ENSBTAG00000008118 | RPF1 | 0 | grey |
| ENSBTAG00000008250 | SPRY4 | 0 | grey |
| ENSBTAG00000008271 | MEDAG | 0 | grey |
| ENSBTAG00000008419 | PDE2A | 0 | grey |
| ENSBTAG00000008614 | KASH5 | 0 | grey |
| ENSBTAG00000008792 | RNASE6 | 0 | grey |
| ENSBTAG00000009315 | CDON | 0 | grey |
| ENSBTAG00000009337 | PNMA8A | 0 | grey |
| ENSBTAG00000009527 | TCEANC2 | 0 | grey |
| ENSBTAG00000009642 | AKAP5 | 0 | grey |
| ENSBTAG00000009841 | CNDP2 | 0 | grey |
| ENSBTAG00000010128 | NAP1L5 | 0 | grey |
| ENSBTAG00000010167 | TEX14 | 0 | grey |
| ENSBTAG00000010174 | SPATA46 | 0 | grey |
| ENSBTAG00000010253 | SYTL1 | 0 | grey |
| ENSBTAG00000010351 | SNX25 | 0 | grey |
| ENSBTAG00000010387 | MINDY3 | 0 | grey |

| | | | |
|---|---|---|---|
| ENSBTAG00000010923 | RAB9A | 0 | grey |
| ENSBTAG00000010999 | IL17RD | 0 | grey |
| ENSBTAG00000011002 | CCDC136 | 0 | grey |
| ENSBTAG00000011017 | ABI2 | 0 | grey |
| ENSBTAG00000011027 | PTGR1 | 0 | grey |
| ENSBTAG00000011091 | SGIP1 | 0 | grey |
| ENSBTAG00000011132 | CCDC83 | 0 | grey |
| ENSBTAG00000011178 | NA | 0 | grey |
| ENSBTAG00000011186 | BEST1 | 0 | grey |
| ENSBTAG00000011470 | NA | 0 | grey |
| ENSBTAG00000011504 | ZP2 | 0 | grey |
| ENSBTAG00000011680 | PAPLN | 0 | grey |
| ENSBTAG00000011782 | CHGB | 0 | grey |
| ENSBTAG00000011985 | NA | 0 | grey |
| ENSBTAG00000012024 | SLC29A2 | 0 | grey |
| ENSBTAG00000012215 | CPNE7 | 0 | grey |
| ENSBTAG00000012239 | SLC37A3 | 0 | grey |
| ENSBTAG00000012558 | ADAMTS12 | 0 | grey |
| ENSBTAG00000012827 | NA | 0 | grey |
| ENSBTAG00000013294 | DYRK3 | 0 | grey |
| ENSBTAG00000013478 | MARVELD1 | 0 | grey |
| ENSBTAG00000013596 | NR1H4 | 0 | grey |
| ENSBTAG00000014334 | ZFYVE26 | 0 | grey |
| ENSBTAG00000014372 | SMARCD3 | 0 | grey |
| ENSBTAG00000014374 | CARD6 | 0 | grey |
| ENSBTAG00000014377 | CHD3 | 0 | grey |
| ENSBTAG00000014580 | FCRLB | 0 | grey |
| ENSBTAG00000014611 | SORL1 | 0 | grey |
| ENSBTAG00000014616 | SEPSECS | 0 | grey |
| ENSBTAG00000014788 | AKAP12 | 0 | grey |
| ENSBTAG00000014804 | HNRNPDL | 0 | grey |
| ENSBTAG00000014912 | FMOD | 0 | grey |
| ENSBTAG00000014972 | PTGER4 | 0 | grey |
| ENSBTAG00000015261 | CARD14 | 0 | grey |
| ENSBTAG00000015304 | ANXA9 | 0 | grey |
| ENSBTAG00000015553 | SCAI | 0 | grey |
| ENSBTAG00000015879 | TULP3 | 0 | grey |
| ENSBTAG00000015909 | PDE8A | 0 | grey |
| ENSBTAG00000016103 | CBFB | 0 | grey |
| ENSBTAG00000016109 | DHRS12 | 0 | grey |
| ENSBTAG00000016305 | ATP13A4 | 0 | grey |
| ENSBTAG00000016615 | ABHD6 | 0 | grey |
| ENSBTAG00000016644 | LVRN | 0 | grey |
| ENSBTAG00000016883 | SGF29 | 0 | grey |
| ENSBTAG00000016891 | TCP11 | 0 | grey |
| ENSBTAG00000016991 | EFNB2 | 0 | grey |
| ENSBTAG00000016998 | ARL11 | 0 | grey |

| | | | |
|---|---|---|---|
| ENSBTAG00000017095 | DENND4A | 0 | grey |
| ENSBTAG00000017112 | CDYL2 | 0 | grey |
| ENSBTAG00000017158 | VSNL1 | 0 | grey |
| ENSBTAG00000017455 | ADAM9 | 0 | grey |
| ENSBTAG00000017644 | KIF17 | 0 | grey |
| ENSBTAG00000017738 | EMID1 | 0 | grey |
| ENSBTAG00000017804 | BNIP3 | 0 | grey |
| ENSBTAG00000017831 | ERLIN2 | 0 | grey |
| ENSBTAG00000018069 | TNFSF15 | 0 | grey |
| ENSBTAG00000018179 | PCDH8 | 0 | grey |
| ENSBTAG00000018246 | RAB39A | 0 | grey |
| ENSBTAG00000018248 | MGLL | 0 | grey |
| ENSBTAG00000018289 | TMEM150A | 0 | grey |
| ENSBTAG00000018326 | FXYD7 | 0 | grey |
| ENSBTAG00000018473 | MARCO | 0 | grey |
| ENSBTAG00000018562 | LDAF1 | 0 | grey |
| ENSBTAG00000018724 | TMEM82 | 0 | grey |
| ENSBTAG00000018942 | SSX2IP | 0 | grey |
| ENSBTAG00000019066 | FAM167A | 0 | grey |
| ENSBTAG00000019251 | EPB41L3 | 0 | grey |
| ENSBTAG00000019403 | MALSU1 | 0 | grey |
| ENSBTAG00000019612 | RNASE4 | 0 | grey |
| ENSBTAG00000020035 | RCAN1 | 0 | grey |
| ENSBTAG00000020048 | MAPK10 | 0 | grey |
| ENSBTAG00000020468 | MICU3 | 0 | grey |
| ENSBTAG00000020523 | SLC10A5 | 0 | grey |
| ENSBTAG00000020584 | IFT81 | 0 | grey |
| ENSBTAG00000020657 | FAT1 | 0 | grey |
| ENSBTAG00000020658 | LNX1 | 0 | grey |
| ENSBTAG00000020826 | CHIC1 | 0 | grey |
| ENSBTAG00000021313 | RUNDC3B | 0 | grey |
| ENSBTAG00000021484 | TSPAN18 | 0 | grey |
| ENSBTAG00000021508 | LMOD3 | 0 | grey |
| ENSBTAG00000021636 | GPR155 | 0 | grey |
| ENSBTAG00000021672 | RGS1 | 0 | grey |
| ENSBTAG00000021693 | RPP25 | 0 | grey |
| ENSBTAG00000021696 | CCIN | 0 | grey |
| ENSBTAG00000021835 | PRICKLE2 | 0 | grey |
| ENSBTAG00000021879 | VCL | 0 | grey |
| ENSBTAG00000021932 | RNASEK | 0 | grey |
| ENSBTAG00000021969 | KLHL29 | 0 | grey |
| ENSBTAG00000021993 | SPDEF | 0 | grey |
| ENSBTAG00000021999 | CPT1A | 0 | grey |
| ENSBTAG00000022007 | SAMHD1 | 0 | grey |
| ENSBTAG00000022534 | NA | 0 | grey |
| ENSBTAG00000023146 | ADAM32 | 0 | grey |
| ENSBTAG00000024492 | LOC526769 | 0 | grey |

| | | | |
|---|---|---|---|
| ENSBTAG00000024688 | PHIP | 0 | grey |
| ENSBTAG00000024751 | ULBP17 | 0 | grey |
| ENSBTAG00000024950 | ACE | 0 | grey |
| ENSBTAG00000024974 | HEXIM2 | 0 | grey |
| ENSBTAG00000024979 | HSPB9 | 0 | grey |
| ENSBTAG00000025023 | NA | 0 | grey |
| ENSBTAG00000025213 | OMG | 0 | grey |
| ENSBTAG00000025803 | INSYN1 | 0 | grey |
| ENSBTAG00000026501 | MGC127055 | 0 | grey |
| ENSBTAG00000027074 | SV2B | 0 | grey |
| ENSBTAG00000027337 | CTSA | 0 | grey |
| ENSBTAG00000027630 | CISD2 | 0 | grey |
| ENSBTAG00000030335 | ALDH4A1 | 0 | grey |
| ENSBTAG00000030885 | SSC5D | 0 | grey |
| ENSBTAG00000030930 | HMCES | 0 | grey |
| ENSBTAG00000031439 | CDC14A | 0 | grey |
| ENSBTAG00000031785 | NA | 0 | grey |
| ENSBTAG00000031885 | TMEM251 | 0 | grey |
| ENSBTAG00000032366 | SLC23A2 | 0 | grey |
| ENSBTAG00000033284 | NA | 0 | grey |
| ENSBTAG00000034138 | LOC786974 | 0 | grey |
| ENSBTAG00000035777 | NA | 0 | grey |
| ENSBTAG00000037679 | PPP1R3E | 0 | grey |
| ENSBTAG00000037756 | TET1 | 0 | grey |
| ENSBTAG00000037765 | TNFAIP8L1 | 0 | grey |
| ENSBTAG00000037778 | CXCL3 | 0 | grey |
| ENSBTAG00000037832 | ASPHD2 | 0 | grey |
| ENSBTAG00000038490 | CLEC4A | 0 | grey |
| ENSBTAG00000038584 | OLFM1 | 0 | grey |
| ENSBTAG00000038896 | RPS26 | 0 | grey |
| ENSBTAG00000039028 | PI3 | 0 | grey |
| ENSBTAG00000039513 | GCNT2 | 0 | grey |
| ENSBTAG00000039618 | ARL14EPL | 0 | grey |
| ENSBTAG00000040063 | NA | 0 | grey |
| ENSBTAG00000040585 | NR6A1 | 0 | grey |
| ENSBTAG00000042479 | LOC112444954 | 0 | grey |
| ENSBTAG00000042711 | LOC112444971 | 0 | grey |
| ENSBTAG00000042871 | LOC112443652 | 0 | grey |
| ENSBTAG00000043729 | NA | 0 | grey |
| ENSBTAG00000043914 | NA | 0 | grey |
| ENSBTAG00000044023 | CDK6 | 0 | grey |
| ENSBTAG00000044138 | METTL23 | 0 | grey |
| ENSBTAG00000044179 | CERS6 | 0 | grey |
| ENSBTAG00000044208 | DUSP4 | 0 | grey |
| ENSBTAG00000045674 | NA | 0 | grey |
| ENSBTAG00000045832 | PHLPP1 | 0 | grey |
| ENSBTAG00000046057 | NA | 0 | grey |

| | | | |
|---|---|---|---|
| ENSBTAG00000046118 | NA | 0 | grey |
| ENSBTAG00000046328 | XG | 0 | grey |
| ENSBTAG00000046429 | NA | 0 | grey |
| ENSBTAG00000046444 | NA | 0 | grey |
| ENSBTAG00000046456 | NA | 0 | grey |
| ENSBTAG00000046678 | NA | 0 | grey |
| ENSBTAG00000047024 | NA | 0 | grey |
| ENSBTAG00000047062 | LRP3 | 0 | grey |
| ENSBTAG00000047284 | NA | 0 | grey |
| ENSBTAG00000047405 | NA | 0 | grey |
| ENSBTAG00000047582 | CSNK1E | 0 | grey |
| ENSBTAG00000047650 | NXPH4 | 0 | grey |
| ENSBTAG00000047764 | LOC100848700 | 0 | grey |
| ENSBTAG00000047827 | RENBP | 0 | grey |
| ENSBTAG00000047848 | NA | 0 | grey |
| ENSBTAG00000048059 | TMEM86A | 0 | grey |
| ENSBTAG00000048067 | EPOP | 0 | grey |
| ENSBTAG00000000359 | EIF3J | 17 | grey60 |
| ENSBTAG00000000974 | IARS1 | 17 | grey60 |
| ENSBTAG00000002579 | FBL | 17 | grey60 |
| ENSBTAG00000002745 | APEX1 | 17 | grey60 |
| ENSBTAG00000003239 | UTP20 | 17 | grey60 |
| ENSBTAG00000003838 | NA | 17 | grey60 |
| ENSBTAG00000004257 | TAF4B | 17 | grey60 |
| ENSBTAG00000004329 | RELL1 | 17 | grey60 |
| ENSBTAG00000005517 | NOP58 | 17 | grey60 |
| ENSBTAG00000006883 | EIF4B | 17 | grey60 |
| ENSBTAG00000007616 | TMED6 | 17 | grey60 |
| ENSBTAG00000007743 | PUS7 | 17 | grey60 |
| ENSBTAG00000009393 | GAL | 17 | grey60 |
| ENSBTAG00000009838 | RRP15 | 17 | grey60 |
| ENSBTAG00000009846 | MTHFD1L | 17 | grey60 |
| ENSBTAG00000010604 | PNO1 | 17 | grey60 |
| ENSBTAG00000014541 | PDGFA | 17 | grey60 |
| ENSBTAG00000014549 | GNL3 | 17 | grey60 |
| ENSBTAG00000014558 | DDX21 | 17 | grey60 |
| ENSBTAG00000014588 | RSL1D1 | 17 | grey60 |
| ENSBTAG00000015303 | PALS2 | 17 | grey60 |
| ENSBTAG00000015316 | NPM1 | 17 | grey60 |
| ENSBTAG00000015424 | WDR12 | 17 | grey60 |
| ENSBTAG00000016034 | EXOSC5 | 17 | grey60 |
| ENSBTAG00000016347 | LOC784385 | 17 | grey60 |
| ENSBTAG00000016584 | RPF2 | 17 | grey60 |
| ENSBTAG00000016805 | SGMS2 | 17 | grey60 |
| ENSBTAG00000017889 | CCT5 | 17 | grey60 |
| ENSBTAG00000018065 | YARS1 | 17 | grey60 |
| ENSBTAG00000019225 | SLC39A14 | 17 | grey60 |

| | | | |
|---|---|---|---|
| ENSBTAG00000019387 | NIFK | 17 | grey60 |
| ENSBTAG00000019648 | RACK1 | 17 | grey60 |
| ENSBTAG00000020931 | CHN2 | 17 | grey60 |
| ENSBTAG00000021979 | EEF1B2 | 17 | grey60 |
| ENSBTAG00000022292 | TIMM8A | 17 | grey60 |
| ENSBTAG00000023507 | NA | 17 | grey60 |
| ENSBTAG00000025664 | RNASE2 | 17 | grey60 |
| ENSBTAG00000027319 | NA | 17 | grey60 |
| ENSBTAG00000027407 | TNFRSF6B | 17 | grey60 |
| ENSBTAG00000033504 | MAK16 | 17 | grey60 |
| ENSBTAG00000034396 | PIGW | 17 | grey60 |
| ENSBTAG00000035379 | LOC511386 | 17 | grey60 |
| ENSBTAG00000038527 | LOC783382 | 17 | grey60 |
| ENSBTAG00000000140 | EPHX1 | 16 | lightcyan |
| ENSBTAG00000000819 | RFX3 | 16 | lightcyan |
| ENSBTAG00000000905 | RAB6B | 16 | lightcyan |
| ENSBTAG00000001292 | LTF | 16 | lightcyan |
| ENSBTAG00000001545 | SLC34A2 | 16 | lightcyan |
| ENSBTAG00000002025 | AMIGO2 | 16 | lightcyan |
| ENSBTAG00000002632 | CSN2 | 16 | lightcyan |
| ENSBTAG00000002844 | PPFIA3 | 16 | lightcyan |
| ENSBTAG00000003608 | SYCP2 | 16 | lightcyan |
| ENSBTAG00000003696 | BICDL1 | 16 | lightcyan |
| ENSBTAG00000003829 | HPN | 16 | lightcyan |
| ENSBTAG00000005005 | CSN1S2 | 16 | lightcyan |
| ENSBTAG00000005859 | LALBA | 16 | lightcyan |
| ENSBTAG00000006222 | TFDP2 | 16 | lightcyan |
| ENSBTAG00000006263 | GP2 | 16 | lightcyan |
| ENSBTAG00000006645 | LPAR6 | 16 | lightcyan |
| ENSBTAG00000007695 | CSN1S1 | 16 | lightcyan |
| ENSBTAG00000009877 | MCMBP | 16 | lightcyan |
| ENSBTAG00000010990 | LHX2 | 16 | lightcyan |
| ENSBTAG00000012460 | ELF5 | 16 | lightcyan |
| ENSBTAG00000012780 | LPO | 16 | lightcyan |
| ENSBTAG00000012794 | PAH | 16 | lightcyan |
| ENSBTAG00000012918 | CRISP3 | 16 | lightcyan |
| ENSBTAG00000013417 | GLYCAM1 | 16 | lightcyan |
| ENSBTAG00000014354 | FXYD6 | 16 | lightcyan |
| ENSBTAG00000014357 | SDC2 | 16 | lightcyan |
| ENSBTAG00000014678 | PAEP | 16 | lightcyan |
| ENSBTAG00000015182 | STARD10 | 16 | lightcyan |
| ENSBTAG00000015222 | RSRP1 | 16 | lightcyan |
| ENSBTAG00000015450 | CPEB3 | 16 | lightcyan |
| ENSBTAG00000016502 | LOC786562 | 16 | lightcyan |
| ENSBTAG00000017104 | MUC1 | 16 | lightcyan |
| ENSBTAG00000017561 | HHIPL2 | 16 | lightcyan |
| ENSBTAG00000017597 | ESCO1 | 16 | lightcyan |

| | | | |
|---|---|---|---|
| ENSBTAG00000019421 | DACT1 | 16 | lightcyan |
| ENSBTAG00000019798 | PIGR | 16 | lightcyan |
| ENSBTAG00000024107 | INPP5B | 16 | lightcyan |
| ENSBTAG00000027126 | MUC15 | 16 | lightcyan |
| ENSBTAG00000031497 | FGFBP1 | 16 | lightcyan |
| ENSBTAG00000031753 | BTN1A1 | 16 | lightcyan |
| ENSBTAG00000032183 | NELL2 | 16 | lightcyan |
| ENSBTAG00000032288 | GCK | 16 | lightcyan |
| ENSBTAG00000039787 | CSN3 | 16 | lightcyan |
| ENSBTAG00000040070 | SCGB1D | 16 | lightcyan |
| ENSBTAG00000043567 | NA | 16 | lightcyan |
| ENSBTAG00000043570 | NA | 16 | lightcyan |
| ENSBTAG00000046481 | ZNF740 | 16 | lightcyan |
| ENSBTAG00000047287 | MTARC1 | 16 | lightcyan |
| ENSBTAG00000048250 | HSTN | 16 | lightcyan |
| ENSBTAG00000000296 | IMPDH1 | 9 | magenta |
| ENSBTAG00000000406 | GPATCH4 | 9 | magenta |
| ENSBTAG00000000451 | TMEM126A | 9 | magenta |
| ENSBTAG00000000605 | ATP5PF | 9 | magenta |
| ENSBTAG00000000879 | C14H8orf33 | 9 | magenta |
| ENSBTAG00000000913 | UQCR10 | 9 | magenta |
| ENSBTAG00000001036 | HIBADH | 9 | magenta |
| ENSBTAG00000001302 | MRPS28 | 9 | magenta |
| ENSBTAG00000001520 | MRPL24 | 9 | magenta |
| ENSBTAG00000001521 | UQCRB | 9 | magenta |
| ENSBTAG00000001836 | NA | 9 | magenta |
| ENSBTAG00000001839 | OCIAD2 | 9 | magenta |
| ENSBTAG00000001858 | NA | 9 | magenta |
| ENSBTAG00000002085 | IL36G | 9 | magenta |
| ENSBTAG00000002808 | PSMA3 | 9 | magenta |
| ENSBTAG00000002944 | ATP5MC3 | 9 | magenta |
| ENSBTAG00000003155 | IFI27L2 | 9 | magenta |
| ENSBTAG00000003263 | TBCA | 9 | magenta |
| ENSBTAG00000003279 | NDUFA10 | 9 | magenta |
| ENSBTAG00000004295 | NDUFA8 | 9 | magenta |
| ENSBTAG00000004538 | PRDM9 | 9 | magenta |
| ENSBTAG00000004544 | TXNDC9 | 9 | magenta |
| ENSBTAG00000004651 | NME1 | 9 | magenta |
| ENSBTAG00000004772 | THEM4 | 9 | magenta |
| ENSBTAG00000004862 | TUB | 9 | magenta |
| ENSBTAG00000004922 | AGPAT5 | 9 | magenta |
| ENSBTAG00000005096 | COX7A2 | 9 | magenta |
| ENSBTAG00000005907 | NDUFB6 | 9 | magenta |
| ENSBTAG00000006075 | SUCLG1 | 9 | magenta |
| ENSBTAG00000006342 | ATP5IF1 | 9 | magenta |
| ENSBTAG00000006391 | NDUFAB1 | 9 | magenta |
| ENSBTAG00000006414 | NUTF2 | 9 | magenta |

| | | | |
|---|---|---|---|
| ENSBTAG00000006429 | ACO2 | 9 | magenta |
| ENSBTAG00000006530 | COX7ALP1 | 9 | magenta |
| ENSBTAG00000006812 | FAM181B | 9 | magenta |
| ENSBTAG00000006821 | PECR | 9 | magenta |
| ENSBTAG00000006936 | MRPS18B | 9 | magenta |
| ENSBTAG00000007172 | GOT2 | 9 | magenta |
| ENSBTAG00000007434 | CD300LG | 9 | magenta |
| ENSBTAG00000007554 | IFI6 | 9 | magenta |
| ENSBTAG00000007755 | APOBEC3H | 9 | magenta |
| ENSBTAG00000007773 | VCAM1 | 9 | magenta |
| ENSBTAG00000007812 | NDUFA13 | 9 | magenta |
| ENSBTAG00000007817 | BRCC3 | 9 | magenta |
| ENSBTAG00000008103 | ALDH1A1 | 9 | magenta |
| ENSBTAG00000008292 | SNRPD1 | 9 | magenta |
| ENSBTAG00000008323 | SNAP25 | 9 | magenta |
| ENSBTAG00000008692 | UQCR11 | 9 | magenta |
| ENSBTAG00000008730 | SFXN4 | 9 | magenta |
| ENSBTAG00000008812 | PFDN4 | 9 | magenta |
| ENSBTAG00000008880 | POGLUT2 | 9 | magenta |
| ENSBTAG00000009172 | BCAT2 | 9 | magenta |
| ENSBTAG00000009182 | CLCN1 | 9 | magenta |
| ENSBTAG00000009462 | MDH2 | 9 | magenta |
| ENSBTAG00000009479 | UQCRQ | 9 | magenta |
| ENSBTAG00000009517 | DBI | 9 | magenta |
| ENSBTAG00000009603 | UQCRH | 9 | magenta |
| ENSBTAG00000009713 | ATP5MK | 9 | magenta |
| ENSBTAG00000009881 | ENTPD6 | 9 | magenta |
| ENSBTAG00000010265 | MGST3 | 9 | magenta |
| ENSBTAG00000010463 | NA | 9 | magenta |
| ENSBTAG00000010503 | TIMM9 | 9 | magenta |
| ENSBTAG00000010550 | EIF5A2 | 9 | magenta |
| ENSBTAG00000010630 | ABHD18 | 9 | magenta |
| ENSBTAG00000010709 | DLAT | 9 | magenta |
| ENSBTAG00000010937 | APOO | 9 | magenta |
| ENSBTAG00000010961 | LYRM7 | 9 | magenta |
| ENSBTAG00000011045 | MRPS36 | 9 | magenta |
| ENSBTAG00000011138 | NA | 9 | magenta |
| ENSBTAG00000011145 | NDUFA4 | 9 | magenta |
| ENSBTAG00000011754 | LAMTOR2 | 9 | magenta |
| ENSBTAG00000011841 | TCTN3 | 9 | magenta |
| ENSBTAG00000012366 | MRPS23 | 9 | magenta |
| ENSBTAG00000012490 | ETFA | 9 | magenta |
| ENSBTAG00000012739 | C1QBP | 9 | magenta |
| ENSBTAG00000012752 | MRPL2 | 9 | magenta |
| ENSBTAG00000012757 | GCNT1 | 9 | magenta |
| ENSBTAG00000012760 | NDUFB3 | 9 | magenta |
| ENSBTAG00000012798 | KCNH8 | 9 | magenta |

| | | | |
|---|---|---|---|
| ENSBTAG00000012907 | ODF2L | 9 | magenta |
| ENSBTAG00000012975 | VDAC2 | 9 | magenta |
| ENSBTAG00000012977 | COMTD1 | 9 | magenta |
| ENSBTAG00000013273 | PIGL | 9 | magenta |
| ENSBTAG00000013600 | PSMG1 | 9 | magenta |
| ENSBTAG00000014432 | HNMT | 9 | magenta |
| ENSBTAG00000014440 | PSMA4 | 9 | magenta |
| ENSBTAG00000014520 | LIAS | 9 | magenta |
| ENSBTAG00000014536 | SFXN1 | 9 | magenta |
| ENSBTAG00000014669 | NDUFA12 | 9 | magenta |
| ENSBTAG00000014729 | MRPL51 | 9 | magenta |
| ENSBTAG00000014805 | ENOPH1 | 9 | magenta |
| ENSBTAG00000014841 | GBA | 9 | magenta |
| ENSBTAG00000014958 | PRKAR2B | 9 | magenta |
| ENSBTAG00000015024 | GXYLT1 | 9 | magenta |
| ENSBTAG00000015296 | PTPRB | 9 | magenta |
| ENSBTAG00000015512 | HEXB | 9 | magenta |
| ENSBTAG00000015522 | MRPS31 | 9 | magenta |
| ENSBTAG00000015567 | TIMM44 | 9 | magenta |
| ENSBTAG00000015644 | EID2 | 9 | magenta |
| ENSBTAG00000015761 | COQ5 | 9 | magenta |
| ENSBTAG00000015835 | PIWIL2 | 9 | magenta |
| ENSBTAG00000016032 | NA | 9 | magenta |
| ENSBTAG00000016042 | TM6SF2 | 9 | magenta |
| ENSBTAG00000016266 | SDHD | 9 | magenta |
| ENSBTAG00000016284 | IL7 | 9 | magenta |
| ENSBTAG00000016337 | ACOT13 | 9 | magenta |
| ENSBTAG00000016886 | MT3 | 9 | magenta |
| ENSBTAG00000017085 | AGA | 9 | magenta |
| ENSBTAG00000017135 | CTSS | 9 | magenta |
| ENSBTAG00000017267 | COX5A | 9 | magenta |
| ENSBTAG00000017326 | OMA1 | 9 | magenta |
| ENSBTAG00000017330 | NA | 9 | magenta |
| ENSBTAG00000017557 | QRSL1 | 9 | magenta |
| ENSBTAG00000017665 | DHRS4 | 9 | magenta |
| ENSBTAG00000017733 | CA2 | 9 | magenta |
| ENSBTAG00000018101 | AHCY | 9 | magenta |
| ENSBTAG00000018188 | NDUFC2 | 9 | magenta |
| ENSBTAG00000018261 | PDHX | 9 | magenta |
| ENSBTAG00000018291 | C11H2orf68 | 9 | magenta |
| ENSBTAG00000018339 | ATP5MC1 | 9 | magenta |
| ENSBTAG00000018382 | SMS | 9 | magenta |
| ENSBTAG00000018397 | HHLA2 | 9 | magenta |
| ENSBTAG00000018483 | NDUFS3 | 9 | magenta |
| ENSBTAG00000018813 | IDH3B | 9 | magenta |
| ENSBTAG00000019010 | EIF2B3 | 9 | magenta |
| ENSBTAG00000019274 | ATIC | 9 | magenta |

| | | | |
|---|---|---|---|
| ENSBTAG00000019298 | STRADB | 9 | magenta |
| ENSBTAG00000020190 | KREMEN2 | 9 | magenta |
| ENSBTAG00000020596 | TMEM126B | 9 | magenta |
| ENSBTAG00000020607 | ABCB6 | 9 | magenta |
| ENSBTAG00000020632 | NQO1 | 9 | magenta |
| ENSBTAG00000020774 | NA | 9 | magenta |
| ENSBTAG00000020777 | ERCC3 | 9 | magenta |
| ENSBTAG00000021048 | ADM | 9 | magenta |
| ENSBTAG00000021199 | COA8 | 9 | magenta |
| ENSBTAG00000021517 | TMEM216 | 9 | magenta |
| ENSBTAG00000021651 | UQCRC2 | 9 | magenta |
| ENSBTAG00000021663 | SMIM8 | 9 | magenta |
| ENSBTAG00000021776 | NDUFV1 | 9 | magenta |
| ENSBTAG00000021976 | NDUFS1 | 9 | magenta |
| ENSBTAG00000022005 | TLDC2 | 9 | magenta |
| ENSBTAG00000022032 | CHCHD10 | 9 | magenta |
| ENSBTAG00000022613 | CYCS | 9 | magenta |
| ENSBTAG00000023419 | SULT2A1 | 9 | magenta |
| ENSBTAG00000023823 | LOC100847700 | 9 | magenta |
| ENSBTAG00000024027 | TRMT10C | 9 | magenta |
| ENSBTAG00000024490 | NA | 9 | magenta |
| ENSBTAG00000025001 | MRPL45 | 9 | magenta |
| ENSBTAG00000025005 | METTL7A | 9 | magenta |
| ENSBTAG00000025310 | NA | 9 | magenta |
| ENSBTAG00000025853 | HOMER1 | 9 | magenta |
| ENSBTAG00000025903 | GLDC | 9 | magenta |
| ENSBTAG00000026286 | GTF2IRD2 | 9 | magenta |
| ENSBTAG00000026417 | NA | 9 | magenta |
| ENSBTAG00000026418 | NA | 9 | magenta |
| ENSBTAG00000027795 | GFM1 | 9 | magenta |
| ENSBTAG00000030575 | BHLHE41 | 9 | magenta |
| ENSBTAG00000030674 | AK4 | 9 | magenta |
| ENSBTAG00000030735 | NA | 9 | magenta |
| ENSBTAG00000031461 | SNX7 | 9 | magenta |
| ENSBTAG00000032156 | NA | 9 | magenta |
| ENSBTAG00000033197 | EPDR1 | 9 | magenta |
| ENSBTAG00000033731 | PRCP | 9 | magenta |
| ENSBTAG00000034174 | NA | 9 | magenta |
| ENSBTAG00000034498 | LY6D | 9 | magenta |
| ENSBTAG00000034992 | MCTS1 | 9 | magenta |
| ENSBTAG00000037415 | LOC100295347 | 9 | magenta |
| ENSBTAG00000037539 | LOC534578 | 9 | magenta |
| ENSBTAG00000038067 | NA | 9 | magenta |
| ENSBTAG00000039555 | COX7C | 9 | magenta |
| ENSBTAG00000039932 | NA | 9 | magenta |
| ENSBTAG00000039980 | CLECL1 | 9 | magenta |
| ENSBTAG00000040058 | ORC5 | 9 | magenta |

| | | | |
|---|---|---|---|
| ENSBTAG00000045783 | NA | 9 | magenta |
| ENSBTAG00000046037 | SLC25A5 | 9 | magenta |
| ENSBTAG00000047319 | NA | 9 | magenta |
| ENSBTAG00000047362 | RCN1 | 9 | magenta |
| ENSBTAG00000047768 | COX7B | 9 | magenta |
| ENSBTAG00000000087 | HSD17B12 | 15 | midnightblue |
| ENSBTAG00000000754 | PPP2R5A | 15 | midnightblue |
| ENSBTAG00000000970 | ASIC1 | 15 | midnightblue |
| ENSBTAG00000001607 | TMEM41B | 15 | midnightblue |
| ENSBTAG00000001992 | CYP51A1 | 15 | midnightblue |
| ENSBTAG00000002714 | GNAI1 | 15 | midnightblue |
| ENSBTAG00000002827 | ACAT2 | 15 | midnightblue |
| ENSBTAG00000003068 | MSMO1 | 15 | midnightblue |
| ENSBTAG00000003948 | FDPS | 15 | midnightblue |
| ENSBTAG00000004075 | IDI1 | 15 | midnightblue |
| ENSBTAG00000004688 | DHCR24 | 15 | midnightblue |
| ENSBTAG00000005498 | SQLE | 15 | midnightblue |
| ENSBTAG00000005678 | AACS | 15 | midnightblue |
| ENSBTAG00000006209 | EXT1 | 15 | midnightblue |
| ENSBTAG00000006679 | MITF | 15 | midnightblue |
| ENSBTAG00000006680 | NKIRAS1 | 15 | midnightblue |
| ENSBTAG00000007689 | LPIN1 | 15 | midnightblue |
| ENSBTAG00000007840 | HMGCR | 15 | midnightblue |
| ENSBTAG00000008004 | NCF2 | 15 | midnightblue |
| ENSBTAG00000008329 | CYTIP | 15 | midnightblue |
| ENSBTAG00000008621 | PRKAR1A | 15 | midnightblue |
| ENSBTAG00000008993 | INTS6L | 15 | midnightblue |
| ENSBTAG00000009231 | NSDHL | 15 | midnightblue |
| ENSBTAG00000009441 | RBBP6 | 15 | midnightblue |
| ENSBTAG00000009539 | NA | 15 | midnightblue |
| ENSBTAG00000010376 | TMEM184C | 15 | midnightblue |
| ENSBTAG00000010637 | ARSG | 15 | midnightblue |
| ENSBTAG00000011839 | HMGCS1 | 15 | midnightblue |
| ENSBTAG00000012059 | MVD | 15 | midnightblue |
| ENSBTAG00000012432 | FDFT1 | 15 | midnightblue |
| ENSBTAG00000012817 | JAG1 | 15 | midnightblue |
| ENSBTAG00000013452 | AQP9 | 15 | midnightblue |
| ENSBTAG00000013909 | BOC | 15 | midnightblue |
| ENSBTAG00000014038 | CDKL2 | 15 | midnightblue |
| ENSBTAG00000014420 | AGER | 15 | midnightblue |
| ENSBTAG00000015277 | PCF11 | 15 | midnightblue |
| ENSBTAG00000016465 | DHCR7 | 15 | midnightblue |
| ENSBTAG00000016595 | CYSTM1 | 15 | midnightblue |
| ENSBTAG00000016625 | TMEM131L | 15 | midnightblue |
| ENSBTAG00000018936 | LSS | 15 | midnightblue |
| ENSBTAG00000019246 | SC5D | 15 | midnightblue |
| ENSBTAG00000020219 | MSS51 | 15 | midnightblue |

| | | | |
|---|---|---|---|
| ENSBTAG00000021965 | SUB1 | 15 | midnightblue |
| ENSBTAG00000021994 | CACNA2D4 | 15 | midnightblue |
| ENSBTAG00000024904 | NA | 15 | midnightblue |
| ENSBTAG00000039740 | LY6G5B | 15 | midnightblue |
| ENSBTAG00000042183 | LOC112446498 | 15 | midnightblue |
| ENSBTAG00000042757 | LOC112448166 | 15 | midnightblue |
| ENSBTAG00000043229 | NA | 15 | midnightblue |
| ENSBTAG00000044048 | LMBR1L | 15 | midnightblue |
| ENSBTAG00000047837 | NA | 15 | midnightblue |
| ENSBTAG00000047954 | ZNF75D | 15 | midnightblue |
| ENSBTAG00000047970 | NA | 15 | midnightblue |
| ENSBTAG00000000054 | SNAPC4 | 8 | pink |
| ENSBTAG00000000153 | LRFN3 | 8 | pink |
| ENSBTAG00000000621 | CATSPERG | 8 | pink |
| ENSBTAG00000000656 | NFATC1 | 8 | pink |
| ENSBTAG00000000813 | HOMER3 | 8 | pink |
| ENSBTAG00000000946 | MEAK7 | 8 | pink |
| ENSBTAG00000001024 | ARID1A | 8 | pink |
| ENSBTAG00000001156 | ST3GAL1 | 8 | pink |
| ENSBTAG00000001179 | TEP1 | 8 | pink |
| ENSBTAG00000001195 | KCNH4 | 8 | pink |
| ENSBTAG00000001473 | ARVCF | 8 | pink |
| ENSBTAG00000001597 | PITPNM2 | 8 | pink |
| ENSBTAG00000001814 | PLXND1 | 8 | pink |
| ENSBTAG00000001829 | SLC17A9 | 8 | pink |
| ENSBTAG00000001890 | RUBCN | 8 | pink |
| ENSBTAG00000002177 | PIKFYVE | 8 | pink |
| ENSBTAG00000002226 | FBXO31 | 8 | pink |
| ENSBTAG00000002303 | GLG1 | 8 | pink |
| ENSBTAG00000002539 | TRIOBP | 8 | pink |
| ENSBTAG00000002639 | ZDHHC7 | 8 | pink |
| ENSBTAG00000002770 | TCN2 | 8 | pink |
| ENSBTAG00000003553 | ZFP36L2 | 8 | pink |
| ENSBTAG00000003604 | ADAMTSL4 | 8 | pink |
| ENSBTAG00000003822 | CROCC | 8 | pink |
| ENSBTAG00000004246 | SPSB1 | 8 | pink |
| ENSBTAG00000004315 | HDLBP | 8 | pink |
| ENSBTAG00000004377 | PHLDB3 | 8 | pink |
| ENSBTAG00000004457 | ORAI1 | 8 | pink |
| ENSBTAG00000004625 | PYGB | 8 | pink |
| ENSBTAG00000004782 | PTPRCAP | 8 | pink |
| ENSBTAG00000004970 | LRRC24 | 8 | pink |
| ENSBTAG00000005069 | TM7SF2 | 8 | pink |
| ENSBTAG00000005514 | TRIO | 8 | pink |
| ENSBTAG00000005733 | CHD6 | 8 | pink |
| ENSBTAG00000005780 | LDB1 | 8 | pink |
| ENSBTAG00000005842 | ABCC10 | 8 | pink |

| | | | |
|---|---|---|---|
| ENSBTAG00000005882 | NA | 8 | pink |
| ENSBTAG00000006572 | CARD9 | 8 | pink |
| ENSBTAG00000006657 | ZDHHC8 | 8 | pink |
| ENSBTAG00000006775 | SLC12A7 | 8 | pink |
| ENSBTAG00000006832 | SLC5A6 | 8 | pink |
| ENSBTAG00000007388 | ZC3H12D | 8 | pink |
| ENSBTAG00000008283 | FLI1 | 8 | pink |
| ENSBTAG00000008421 | SCRIB | 8 | pink |
| ENSBTAG00000008510 | FBXO42 | 8 | pink |
| ENSBTAG00000008520 | NFIC | 8 | pink |
| ENSBTAG00000008762 | HECTD4 | 8 | pink |
| ENSBTAG00000009267 | UHRF1BP1 | 8 | pink |
| ENSBTAG00000009271 | PNMA1 | 8 | pink |
| ENSBTAG00000009272 | MIDEAS | 8 | pink |
| ENSBTAG00000009343 | AAK1 | 8 | pink |
| ENSBTAG00000009386 | NA | 8 | pink |
| ENSBTAG00000009856 | ACAP3 | 8 | pink |
| ENSBTAG00000009915 | AP2A2 | 8 | pink |
| ENSBTAG00000010107 | WHRN | 8 | pink |
| ENSBTAG00000010348 | GATD1 | 8 | pink |
| ENSBTAG00000010450 | WIZ | 8 | pink |
| ENSBTAG00000010698 | VARS2 | 8 | pink |
| ENSBTAG00000010830 | LRP1 | 8 | pink |
| ENSBTAG00000010958 | NA | 8 | pink |
| ENSBTAG00000011000 | HDAC10 | 8 | pink |
| ENSBTAG00000011129 | QSOX2 | 8 | pink |
| ENSBTAG00000011310 | NALF2 | 8 | pink |
| ENSBTAG00000011762 | ANKRD52 | 8 | pink |
| ENSBTAG00000011875 | CLIP2 | 8 | pink |
| ENSBTAG00000011969 | HSPB1 | 8 | pink |
| ENSBTAG00000011984 | RSKR | 8 | pink |
| ENSBTAG00000011988 | KIAA0100 | 8 | pink |
| ENSBTAG00000012032 | PDE4A | 8 | pink |
| ENSBTAG00000012040 | GIT1 | 8 | pink |
| ENSBTAG00000012189 | NA | 8 | pink |
| ENSBTAG00000012200 | ANKRD13D | 8 | pink |
| ENSBTAG00000012675 | MYO19 | 8 | pink |
| ENSBTAG00000012768 | HMGXB3 | 8 | pink |
| ENSBTAG00000012922 | PHF2 | 8 | pink |
| ENSBTAG00000012970 | FAM53B | 8 | pink |
| ENSBTAG00000013109 | STIM1 | 8 | pink |
| ENSBTAG00000013624 | LMNB2 | 8 | pink |
| ENSBTAG00000013636 | DGKZ | 8 | pink |
| ENSBTAG00000013722 | AARS2 | 8 | pink |
| ENSBTAG00000013916 | HERC2 | 8 | pink |
| ENSBTAG00000013938 | PHC3 | 8 | pink |
| ENSBTAG00000013942 | DAGLA | 8 | pink |

| | | | |
|---|---|---|---|
| ENSBTAG00000014016 | IKZF1 | 8 | pink |
| ENSBTAG00000014021 | UBN2 | 8 | pink |
| ENSBTAG00000014093 | IDH2 | 8 | pink |
| ENSBTAG00000014454 | NA | 8 | pink |
| ENSBTAG00000014636 | ZFHX3 | 8 | pink |
| ENSBTAG00000014646 | MZT2B | 8 | pink |
| ENSBTAG00000014779 | NISCH | 8 | pink |
| ENSBTAG00000014782 | STAB1 | 8 | pink |
| ENSBTAG00000015037 | GAREM2 | 8 | pink |
| ENSBTAG00000015165 | DPY19L3 | 8 | pink |
| ENSBTAG00000015192 | FAM193B | 8 | pink |
| ENSBTAG00000015321 | TRERF1 | 8 | pink |
| ENSBTAG00000015438 | RRBP1 | 8 | pink |
| ENSBTAG00000015538 | LEMD2 | 8 | pink |
| ENSBTAG00000015698 | FYCO1 | 8 | pink |
| ENSBTAG00000015939 | NECTIN1 | 8 | pink |
| ENSBTAG00000016094 | SLC36A1 | 8 | pink |
| ENSBTAG00000016362 | POU6F1 | 8 | pink |
| ENSBTAG00000016470 | NADSYN1 | 8 | pink |
| ENSBTAG00000016609 | DQX1 | 8 | pink |
| ENSBTAG00000016629 | ADCY9 | 8 | pink |
| ENSBTAG00000016630 | NA | 8 | pink |
| ENSBTAG00000016827 | RGL2 | 8 | pink |
| ENSBTAG00000016958 | PHF19 | 8 | pink |
| ENSBTAG00000017240 | DGKQ | 8 | pink |
| ENSBTAG00000017380 | NA | 8 | pink |
| ENSBTAG00000017488 | KLF3 | 8 | pink |
| ENSBTAG00000017761 | SMC1A | 8 | pink |
| ENSBTAG00000017860 | HIPK2 | 8 | pink |
| ENSBTAG00000017871 | GNA12 | 8 | pink |
| ENSBTAG00000017894 | CAD | 8 | pink |
| ENSBTAG00000017925 | LMF2 | 8 | pink |
| ENSBTAG00000017950 | SLC4A8 | 8 | pink |
| ENSBTAG00000018272 | RERE | 8 | pink |
| ENSBTAG00000018321 | PAOX | 8 | pink |
| ENSBTAG00000018772 | TAF1C | 8 | pink |
| ENSBTAG00000018825 | SSH1 | 8 | pink |
| ENSBTAG00000019072 | PSD4 | 8 | pink |
| ENSBTAG00000019177 | BIN1 | 8 | pink |
| ENSBTAG00000019556 | MIIP | 8 | pink |
| ENSBTAG00000019807 | COL27A1 | 8 | pink |
| ENSBTAG00000019908 | CUL9 | 8 | pink |
| ENSBTAG00000019954 | ABHD2 | 8 | pink |
| ENSBTAG00000020551 | ABCD1 | 8 | pink |
| ENSBTAG00000020619 | PKD1 | 8 | pink |
| ENSBTAG00000020709 | MAP1S | 8 | pink |
| ENSBTAG00000021024 | MACF1 | 8 | pink |

| | | | |
|---|---|---|---|
| ENSBTAG00000021065 | MFSD13A | 8 | pink |
| ENSBTAG00000021076 | TNRC6B | 8 | pink |
| ENSBTAG00000021143 | DNMT3A | 8 | pink |
| ENSBTAG00000021454 | NA | 8 | pink |
| ENSBTAG00000021472 | ZC3H3 | 8 | pink |
| ENSBTAG00000021512 | ZBTB7B | 8 | pink |
| ENSBTAG00000021550 | MAMDC4 | 8 | pink |
| ENSBTAG00000021669 | SOGA1 | 8 | pink |
| ENSBTAG00000021695 | GLYCTK | 8 | pink |
| ENSBTAG00000021799 | RCN3 | 8 | pink |
| ENSBTAG00000021862 | SLC27A3 | 8 | pink |
| ENSBTAG00000022006 | TRIM65 | 8 | pink |
| ENSBTAG00000023028 | WDFY4 | 8 | pink |
| ENSBTAG00000023787 | TOR4A | 8 | pink |
| ENSBTAG00000024756 | DENND6B | 8 | pink |
| ENSBTAG00000026097 | LIMD1 | 8 | pink |
| ENSBTAG00000026340 | LOC101905222 | 8 | pink |
| ENSBTAG00000026772 | ZNF142 | 8 | pink |
| ENSBTAG00000026819 | HDAC7 | 8 | pink |
| ENSBTAG00000027205 | PGBD5 | 8 | pink |
| ENSBTAG00000030175 | FBF1 | 8 | pink |
| ENSBTAG00000030257 | SH3BP1 | 8 | pink |
| ENSBTAG00000031340 | SLC6A20 | 8 | pink |
| ENSBTAG00000031590 | SSH3 | 8 | pink |
| ENSBTAG00000031809 | DENND1C | 8 | pink |
| ENSBTAG00000032137 | PNPLA6 | 8 | pink |
| ENSBTAG00000033182 | HGSNAT | 8 | pink |
| ENSBTAG00000034985 | PHLDA1 | 8 | pink |
| ENSBTAG00000037404 | LRFN4 | 8 | pink |
| ENSBTAG00000037595 | NA | 8 | pink |
| ENSBTAG00000038047 | NA | 8 | pink |
| ENSBTAG00000038241 | REPIN1 | 8 | pink |
| ENSBTAG00000039594 | ZNF862 | 8 | pink |
| ENSBTAG00000039708 | AJM1 | 8 | pink |
| ENSBTAG00000039935 | KCNC3 | 8 | pink |
| ENSBTAG00000040128 | FZD4 | 8 | pink |
| ENSBTAG00000040507 | ARHGEF18 | 8 | pink |
| ENSBTAG00000040555 | FBXL19 | 8 | pink |
| ENSBTAG00000044019 | KAT2A | 8 | pink |
| ENSBTAG00000044070 | SNX30 | 8 | pink |
| ENSBTAG00000044192 | MAF | 8 | pink |
| ENSBTAG00000045625 | NTN3 | 8 | pink |
| ENSBTAG00000045762 | SCAMP4 | 8 | pink |
| ENSBTAG00000046514 | ASMTL | 8 | pink |
| ENSBTAG00000046516 | FUT7 | 8 | pink |
| ENSBTAG00000046730 | NA | 8 | pink |
| ENSBTAG00000047606 | NA | 8 | pink |

| | | | |
|---|---|---|---|
| ENSBTAG00000047822 | NA | 8 | pink |
| ENSBTAG00000048160 | TIGD5 | 8 | pink |
| ENSBTAG00000000080 | GRWD1 | 10 | purple |
| ENSBTAG00000000480 | IFRD2 | 10 | purple |
| ENSBTAG00000000670 | RRP9 | 10 | purple |
| ENSBTAG00000000917 | BMP1 | 10 | purple |
| ENSBTAG00000001082 | SH2D5 | 10 | purple |
| ENSBTAG00000001229 | SLC25A22 | 10 | purple |
| ENSBTAG00000001468 | SAMD4A | 10 | purple |
| ENSBTAG00000001504 | NOP14 | 10 | purple |
| ENSBTAG00000001537 | TSKU | 10 | purple |
| ENSBTAG00000001644 | MDN1 | 10 | purple |
| ENSBTAG00000001781 | TOMM40 | 10 | purple |
| ENSBTAG00000001795 | LONP1 | 10 | purple |
| ENSBTAG00000001879 | PER2 | 10 | purple |
| ENSBTAG00000002050 | COA7 | 10 | purple |
| ENSBTAG00000002052 | PLOD1 | 10 | purple |
| ENSBTAG00000002056 | MARS2 | 10 | purple |
| ENSBTAG00000002411 | CTNND1 | 10 | purple |
| ENSBTAG00000002669 | RASSF4 | 10 | purple |
| ENSBTAG00000002685 | PITRM1 | 10 | purple |
| ENSBTAG00000002707 | IPO4 | 10 | purple |
| ENSBTAG00000002941 | FES | 10 | purple |
| ENSBTAG00000003323 | NOL6 | 10 | purple |
| ENSBTAG00000003598 | NA | 10 | purple |
| ENSBTAG00000003644 | HSPBP1 | 10 | purple |
| ENSBTAG00000003707 | GRAP | 10 | purple |
| ENSBTAG00000003806 | ECM1 | 10 | purple |
| ENSBTAG00000003813 | BCS1L | 10 | purple |
| ENSBTAG00000003981 | RRP1 | 10 | purple |
| ENSBTAG00000004225 | TSR1 | 10 | purple |
| ENSBTAG00000004392 | C29H11orf24 | 10 | purple |
| ENSBTAG00000004403 | CD320 | 10 | purple |
| ENSBTAG00000005355 | BAG2 | 10 | purple |
| ENSBTAG00000005631 | VARS1 | 10 | purple |
| ENSBTAG00000005773 | PGM2 | 10 | purple |
| ENSBTAG00000005819 | CCDC61 | 10 | purple |
| ENSBTAG00000006157 | NA | 10 | purple |
| ENSBTAG00000006347 | UTP4 | 10 | purple |
| ENSBTAG00000006525 | FDXR | 10 | purple |
| ENSBTAG00000006526 | BCL2L1 | 10 | purple |
| ENSBTAG00000006665 | ERP29 | 10 | purple |
| ENSBTAG00000007105 | PWP2 | 10 | purple |
| ENSBTAG00000007166 | CAMKK1 | 10 | purple |
| ENSBTAG00000007427 | PPRC1 | 10 | purple |
| ENSBTAG00000007430 | MYBBP1A | 10 | purple |
| ENSBTAG00000007435 | NOLC1 | 10 | purple |

| | | | |
|---|---|---|---|
| ENSBTAG00000007619 | PUSL1 | 10 | purple |
| ENSBTAG00000007728 | PES1 | 10 | purple |
| ENSBTAG00000008022 | PTGFRN | 10 | purple |
| ENSBTAG00000008115 | TNFAIP1 | 10 | purple |
| ENSBTAG00000008399 | ATRIP | 10 | purple |
| ENSBTAG00000008409 | MYC | 10 | purple |
| ENSBTAG00000008713 | DCAF4 | 10 | purple |
| ENSBTAG00000008764 | SMYD5 | 10 | purple |
| ENSBTAG00000008994 | FPGS | 10 | purple |
| ENSBTAG00000008997 | ENG | 10 | purple |
| ENSBTAG00000009562 | ZNF593 | 10 | purple |
| ENSBTAG00000009655 | TNS3 | 10 | purple |
| ENSBTAG00000009811 | BOP1 | 10 | purple |
| ENSBTAG00000010101 | BYSL | 10 | purple |
| ENSBTAG00000010180 | URB2 | 10 | purple |
| ENSBTAG00000010602 | DDX56 | 10 | purple |
| ENSBTAG00000010890 | PRMT5 | 10 | purple |
| ENSBTAG00000010904 | PAGR1 | 10 | purple |
| ENSBTAG00000011079 | C18H19orf48 | 10 | purple |
| ENSBTAG00000011280 | TBKBP1 | 10 | purple |
| ENSBTAG00000011554 | SURF6 | 10 | purple |
| ENSBTAG00000011786 | CLUH | 10 | purple |
| ENSBTAG00000011889 | NOC4L | 10 | purple |
| ENSBTAG00000011930 | DDX54 | 10 | purple |
| ENSBTAG00000011951 | MRTO4 | 10 | purple |
| ENSBTAG00000012087 | NA | 10 | purple |
| ENSBTAG00000012234 | RHBG | 10 | purple |
| ENSBTAG00000012412 | URB1 | 10 | purple |
| ENSBTAG00000012525 | ABO | 10 | purple |
| ENSBTAG00000012678 | COLGALT1 | 10 | purple |
| ENSBTAG00000012745 | PPAN | 10 | purple |
| ENSBTAG00000012880 | DNLZ | 10 | purple |
| ENSBTAG00000012990 | PUS1 | 10 | purple |
| ENSBTAG00000013018 | AMPD2 | 10 | purple |
| ENSBTAG00000013045 | DKC1 | 10 | purple |
| ENSBTAG00000013126 | EMG1 | 10 | purple |
| ENSBTAG00000014136 | FAAP20 | 10 | purple |
| ENSBTAG00000014186 | NCOA5 | 10 | purple |
| ENSBTAG00000014733 | NOP2 | 10 | purple |
| ENSBTAG00000014742 | LRWD1 | 10 | purple |
| ENSBTAG00000014975 | SLC4A3 | 10 | purple |
| ENSBTAG00000014979 | MFSD12 | 10 | purple |
| ENSBTAG00000015148 | METTL3 | 10 | purple |
| ENSBTAG00000015529 | CHCHD4 | 10 | purple |
| ENSBTAG00000016220 | WIPF3 | 10 | purple |
| ENSBTAG00000016335 | NPM3 | 10 | purple |
| ENSBTAG00000016528 | NOC2L | 10 | purple |

| | | | |
|---|---|---|---|
| ENSBTAG00000016561 | NUBP2 | 10 | purple |
| ENSBTAG00000016716 | RRS1 | 10 | purple |
| ENSBTAG00000016733 | NOL9 | 10 | purple |
| ENSBTAG00000016824 | WDR46 | 10 | purple |
| ENSBTAG00000017027 | MMACHC | 10 | purple |
| ENSBTAG00000017074 | ANO8 | 10 | purple |
| ENSBTAG00000017219 | DDX51 | 10 | purple |
| ENSBTAG00000017273 | LRRC25 | 10 | purple |
| ENSBTAG00000017632 | AEN | 10 | purple |
| ENSBTAG00000018084 | CASTOR1 | 10 | purple |
| ENSBTAG00000018274 | FADD | 10 | purple |
| ENSBTAG00000018387 | EIPR1 | 10 | purple |
| ENSBTAG00000018655 | MYDGF | 10 | purple |
| ENSBTAG00000018784 | CTSZ | 10 | purple |
| ENSBTAG00000018969 | POLR1C | 10 | purple |
| ENSBTAG00000019162 | MRM3 | 10 | purple |
| ENSBTAG00000019325 | TRMT1 | 10 | purple |
| ENSBTAG00000019721 | REXO4 | 10 | purple |
| ENSBTAG00000019782 | TPI1 | 10 | purple |
| ENSBTAG00000019871 | TRMT2A | 10 | purple |
| ENSBTAG00000019888 | HIRA | 10 | purple |
| ENSBTAG00000020751 | HSF1 | 10 | purple |
| ENSBTAG00000020944 | PIEZO1 | 10 | purple |
| ENSBTAG00000020983 | RRAS | 10 | purple |
| ENSBTAG00000020998 | RUVBL1 | 10 | purple |
| ENSBTAG00000021060 | FTSJ3 | 10 | purple |
| ENSBTAG00000021374 | FARSA | 10 | purple |
| ENSBTAG00000021467 | IGFBP6 | 10 | purple |
| ENSBTAG00000021939 | ELAC2 | 10 | purple |
| ENSBTAG00000022715 | NA | 10 | purple |
| ENSBTAG00000025320 | AKAP1 | 10 | purple |
| ENSBTAG00000030247 | NSMF | 10 | purple |
| ENSBTAG00000031327 | TRMT61A | 10 | purple |
| ENSBTAG00000032089 | CDC42EP2 | 10 | purple |
| ENSBTAG00000032702 | LOC783144 | 10 | purple |
| ENSBTAG00000034529 | HMGA1 | 10 | purple |
| ENSBTAG00000035998 | CKB | 10 | purple |
| ENSBTAG00000038330 | LOC516108 | 10 | purple |
| ENSBTAG00000039050 | P2RY2 | 10 | purple |
| ENSBTAG00000039958 | GYS1 | 10 | purple |
| ENSBTAG00000044198 | THAP7 | 10 | purple |
| ENSBTAG00000046303 | NA | 10 | purple |
| ENSBTAG00000046644 | HRAS | 10 | purple |
| ENSBTAG00000046708 | PDP2 | 10 | purple |
| ENSBTAG00000046977 | PLA2G2D4 | 10 | purple |
| ENSBTAG00000047793 | TSSC4 | 10 | purple |
| ENSBTAG00000047956 | NAGLU | 10 | purple |

| | | | |
|---|---|---|---|
| ENSBTAG00000000009 | FOXF1 | 6 | red |
| ENSBTAG00000000053 | FILIP1 | 6 | red |
| ENSBTAG00000000494 | PDE4D | 6 | red |
| ENSBTAG00000000501 | KLHL13 | 6 | red |
| ENSBTAG00000000736 | LRP8 | 6 | red |
| ENSBTAG00000000928 | SPECC1 | 6 | red |
| ENSBTAG00000001117 | ANKRD50 | 6 | red |
| ENSBTAG00000001140 | IAH1 | 6 | red |
| ENSBTAG00000001505 | GRK4 | 6 | red |
| ENSBTAG00000001770 | ST8SIA5 | 6 | red |
| ENSBTAG00000001801 | SYT4 | 6 | red |
| ENSBTAG00000001848 | KATNB1 | 6 | red |
| ENSBTAG00000001861 | SESTD1 | 6 | red |
| ENSBTAG00000001868 | PCYT2 | 6 | red |
| ENSBTAG00000001906 | FANCA | 6 | red |
| ENSBTAG00000001919 | ZNF652 | 6 | red |
| ENSBTAG00000002655 | ZWINT | 6 | red |
| ENSBTAG00000002823 | MPZL1 | 6 | red |
| ENSBTAG00000002847 | D2HGDH | 6 | red |
| ENSBTAG00000002952 | TBX19 | 6 | red |
| ENSBTAG00000003176 | JAM3 | 6 | red |
| ENSBTAG00000003496 | AIG1 | 6 | red |
| ENSBTAG00000003519 | NOL3 | 6 | red |
| ENSBTAG00000003632 | CYP39A1 | 6 | red |
| ENSBTAG00000003701 | NA | 6 | red |
| ENSBTAG00000003788 | RCC1 | 6 | red |
| ENSBTAG00000004218 | NA | 6 | red |
| ENSBTAG00000004490 | TRIM31 | 6 | red |
| ENSBTAG00000004613 | CSGALNACT1 | 6 | red |
| ENSBTAG00000004705 | ILDR1 | 6 | red |
| ENSBTAG00000004751 | SPTBN4 | 6 | red |
| ENSBTAG00000004879 | FOXO4 | 6 | red |
| ENSBTAG00000004907 | MINK1 | 6 | red |
| ENSBTAG00000004966 | NA | 6 | red |
| ENSBTAG00000005072 | CUBN | 6 | red |
| ENSBTAG00000005183 | MVK | 6 | red |
| ENSBTAG00000005268 | PLA2G4D | 6 | red |
| ENSBTAG00000005280 | ADA | 6 | red |
| ENSBTAG00000005670 | ARHGEF19 | 6 | red |
| ENSBTAG00000005796 | DCAF8 | 6 | red |
| ENSBTAG00000005810 | NA | 6 | red |
| ENSBTAG00000005884 | WDR31 | 6 | red |
| ENSBTAG00000005915 | SFMBT2 | 6 | red |
| ENSBTAG00000006067 | VMAC | 6 | red |
| ENSBTAG00000006069 | CAPS | 6 | red |
| ENSBTAG00000006160 | SUOX | 6 | red |
| ENSBTAG00000006338 | NA | 6 | red |

| | | | |
|---|---|---|---|
| ENSBTAG00000006470 | TTBK2 | 6 | red |
| ENSBTAG00000006531 | DIP2C | 6 | red |
| ENSBTAG00000006790 | SPTBN5 | 6 | red |
| ENSBTAG00000006801 | TMEM106A | 6 | red |
| ENSBTAG00000007074 | ZKSCAN8 | 6 | red |
| ENSBTAG00000007116 | ARRDC3 | 6 | red |
| ENSBTAG00000007428 | CDKL5 | 6 | red |
| ENSBTAG00000007494 | SMARCA2 | 6 | red |
| ENSBTAG00000007665 | NPR3 | 6 | red |
| ENSBTAG00000007705 | COLEC12 | 6 | red |
| ENSBTAG00000007749 | TONSL | 6 | red |
| ENSBTAG00000007758 | PDE10A | 6 | red |
| ENSBTAG00000007830 | PLEKHM3 | 6 | red |
| ENSBTAG00000008063 | PPARA | 6 | red |
| ENSBTAG00000008567 | DLEC1 | 6 | red |
| ENSBTAG00000008789 | HMG20B | 6 | red |
| ENSBTAG00000008814 | ADGRA2 | 6 | red |
| ENSBTAG00000008827 | SPOCK2 | 6 | red |
| ENSBTAG00000009026 | PACS1 | 6 | red |
| ENSBTAG00000009050 | ADIPOR2 | 6 | red |
| ENSBTAG00000009181 | INPP5A | 6 | red |
| ENSBTAG00000009287 | EBP | 6 | red |
| ENSBTAG00000009293 | EVL | 6 | red |
| ENSBTAG00000009421 | CD1E | 6 | red |
| ENSBTAG00000009573 | CSTPP1 | 6 | red |
| ENSBTAG00000009772 | LIX1 | 6 | red |
| ENSBTAG00000009782 | NA | 6 | red |
| ENSBTAG00000009797 | RIMBP2 | 6 | red |
| ENSBTAG00000009832 | NOBOX | 6 | red |
| ENSBTAG00000009837 | ANKS4B | 6 | red |
| ENSBTAG00000009972 | INHA | 6 | red |
| ENSBTAG00000010020 | LOC533921 | 6 | red |
| ENSBTAG00000010217 | ZNF318 | 6 | red |
| ENSBTAG00000010239 | GARIN2 | 6 | red |
| ENSBTAG00000010413 | TMEM169 | 6 | red |
| ENSBTAG00000010509 | CDK3 | 6 | red |
| ENSBTAG00000010521 | PVRIG | 6 | red |
| ENSBTAG00000010815 | CAMKK2 | 6 | red |
| ENSBTAG00000010836 | NA | 6 | red |
| ENSBTAG00000010916 | LHFPL4 | 6 | red |
| ENSBTAG00000011162 | ZKSCAN2 | 6 | red |
| ENSBTAG00000011244 | TESK2 | 6 | red |
| ENSBTAG00000011389 | TMEM104 | 6 | red |
| ENSBTAG00000011403 | RUSC2 | 6 | red |
| ENSBTAG00000011562 | STK32C | 6 | red |
| ENSBTAG00000011581 | BSN | 6 | red |
| ENSBTAG00000011759 | C1QL1 | 6 | red |

| | | | |
|---|---|---|---|
| ENSBTAG00000011971 | NRP2 | 6 | red |
| ENSBTAG00000011987 | ERG28 | 6 | red |
| ENSBTAG00000012253 | AGO1 | 6 | red |
| ENSBTAG00000012349 | LAX1 | 6 | red |
| ENSBTAG00000012605 | GPR157 | 6 | red |
| ENSBTAG00000012687 | INSR | 6 | red |
| ENSBTAG00000012761 | PTK7 | 6 | red |
| ENSBTAG00000012774 | RAB7B | 6 | red |
| ENSBTAG00000013099 | ALDOC | 6 | red |
| ENSBTAG00000013127 | LPCAT3 | 6 | red |
| ENSBTAG00000013166 | SMYD2 | 6 | red |
| ENSBTAG00000013283 | PRR19 | 6 | red |
| ENSBTAG00000013303 | ACSS2 | 6 | red |
| ENSBTAG00000013436 | HAUS5 | 6 | red |
| ENSBTAG00000013686 | WSCD2 | 6 | red |
| ENSBTAG00000013851 | BCL10 | 6 | red |
| ENSBTAG00000013855 | ORMDL3 | 6 | red |
| ENSBTAG00000013974 | TMEM215 | 6 | red |
| ENSBTAG00000014026 | PAG1 | 6 | red |
| ENSBTAG00000014417 | CASTOR2 | 6 | red |
| ENSBTAG00000014459 | MAP6D1 | 6 | red |
| ENSBTAG00000014655 | MYO1A | 6 | red |
| ENSBTAG00000015016 | CALCOCO1 | 6 | red |
| ENSBTAG00000015094 | VNN1 | 6 | red |
| ENSBTAG00000015101 | HMGB2 | 6 | red |
| ENSBTAG00000015133 | CCR7 | 6 | red |
| ENSBTAG00000015242 | SLC18B1 | 6 | red |
| ENSBTAG00000015286 | GOLGA1 | 6 | red |
| ENSBTAG00000015505 | FADS2 | 6 | red |
| ENSBTAG00000015611 | TBC1D9B | 6 | red |
| ENSBTAG00000015614 | LNX2 | 6 | red |
| ENSBTAG00000015637 | BPNT2 | 6 | red |
| ENSBTAG00000015651 | NA | 6 | red |
| ENSBTAG00000015888 | PDE7B | 6 | red |
| ENSBTAG00000016158 | ACAN | 6 | red |
| ENSBTAG00000016296 | GPD1 | 6 | red |
| ENSBTAG00000016302 | SLC25A27 | 6 | red |
| ENSBTAG00000016312 | LGALS4 | 6 | red |
| ENSBTAG00000016529 | SLC25A30 | 6 | red |
| ENSBTAG00000016740 | ACLY | 6 | red |
| ENSBTAG00000016785 | LOC523484 | 6 | red |
| ENSBTAG00000016959 | LAPTM4B | 6 | red |
| ENSBTAG00000017019 | PRKDC | 6 | red |
| ENSBTAG00000017116 | RASD2 | 6 | red |
| ENSBTAG00000017493 | JADE1 | 6 | red |
| ENSBTAG00000017527 | CRYBG1 | 6 | red |
| ENSBTAG00000017626 | GFPT1 | 6 | red |

| | | | |
|---|---|---|---|
| ENSBTAG00000017740 | TUBAL3 | 6 | red |
| ENSBTAG00000017751 | RGS9 | 6 | red |
| ENSBTAG00000018358 | STAC3 | 6 | red |
| ENSBTAG00000018364 | TMEM132A | 6 | red |
| ENSBTAG00000018413 | TENT5B | 6 | red |
| ENSBTAG00000018706 | PCTP | 6 | red |
| ENSBTAG00000018900 | CARMIL2 | 6 | red |
| ENSBTAG00000018984 | PIK3CD | 6 | red |
| ENSBTAG00000019461 | NUMBL | 6 | red |
| ENSBTAG00000019465 | ITPKC | 6 | red |
| ENSBTAG00000019574 | MAPK12 | 6 | red |
| ENSBTAG00000019646 | TRIM41 | 6 | red |
| ENSBTAG00000019847 | CPEB2 | 6 | red |
| ENSBTAG00000020054 | TNFRSF21 | 6 | red |
| ENSBTAG00000020252 | NLGN2 | 6 | red |
| ENSBTAG00000020253 | SPEM1 | 6 | red |
| ENSBTAG00000020260 | NA | 6 | red |
| ENSBTAG00000020311 | USP20 | 6 | red |
| ENSBTAG00000020369 | NOXO1 | 6 | red |
| ENSBTAG00000020701 | MEF2C | 6 | red |
| ENSBTAG00000020729 | DHRS7 | 6 | red |
| ENSBTAG00000021100 | PPP1R3F | 6 | red |
| ENSBTAG00000021107 | STXBP5 | 6 | red |
| ENSBTAG00000021158 | SATB1 | 6 | red |
| ENSBTAG00000021425 | LMAN1L | 6 | red |
| ENSBTAG00000021445 | ST8SIA4 | 6 | red |
| ENSBTAG00000021497 | CDH23 | 6 | red |
| ENSBTAG00000021713 | MAMLD1 | 6 | red |
| ENSBTAG00000021787 | PAQR7 | 6 | red |
| ENSBTAG00000021964 | CDH17 | 6 | red |
| ENSBTAG00000022000 | CACNB2 | 6 | red |
| ENSBTAG00000022150 | NA | 6 | red |
| ENSBTAG00000022733 | EPM2AIP1 | 6 | red |
| ENSBTAG00000022837 | LOC100337093 | 6 | red |
| ENSBTAG00000024379 | LRRC17 | 6 | red |
| ENSBTAG00000026916 | PRR13 | 6 | red |
| ENSBTAG00000027151 | ARHGAP26 | 6 | red |
| ENSBTAG00000027438 | NA | 6 | red |
| ENSBTAG00000030162 | AXIN2 | 6 | red |
| ENSBTAG00000030670 | PCNT | 6 | red |
| ENSBTAG00000030977 | ULK2 | 6 | red |
| ENSBTAG00000031335 | BABAM2 | 6 | red |
| ENSBTAG00000031993 | NA | 6 | red |
| ENSBTAG00000033076 | NA | 6 | red |
| ENSBTAG00000033137 | PSD3 | 6 | red |
| ENSBTAG00000034393 | FAM149A | 6 | red |
| ENSBTAG00000034712 | RAB33A | 6 | red |

| | | | |
|---|---|---|---|
| ENSBTAG00000034991 | NA | 6 | red |
| ENSBTAG00000035129 | NA | 6 | red |
| ENSBTAG00000036257 | LOC107131241 | 6 | red |
| ENSBTAG00000038221 | EFNA2 | 6 | red |
| ENSBTAG00000038487 | ZNF613 | 6 | red |
| ENSBTAG00000038770 | LOC513767 | 6 | red |
| ENSBTAG00000038893 | LOC101904477 | 6 | red |
| ENSBTAG00000038970 | ZBTB14 | 6 | red |
| ENSBTAG00000039015 | TMEM145 | 6 | red |
| ENSBTAG00000039347 | CLEC4D | 6 | red |
| ENSBTAG00000039466 | CDR2L | 6 | red |
| ENSBTAG00000039563 | LOC528282 | 6 | red |
| ENSBTAG00000040061 | ZBTB38 | 6 | red |
| ENSBTAG00000040298 | GSTT1 | 6 | red |
| ENSBTAG00000040356 | SPIRE2 | 6 | red |
| ENSBTAG00000040360 | CCDC166 | 6 | red |
| ENSBTAG00000040485 | NA | 6 | red |
| ENSBTAG00000040559 | NA | 6 | red |
| ENSBTAG00000044056 | C19H17orf98 | 6 | red |
| ENSBTAG00000044105 | FOXO1 | 6 | red |
| ENSBTAG00000045492 | NA | 6 | red |
| ENSBTAG00000045926 | NA | 6 | red |
| ENSBTAG00000046047 | LOC100851913 | 6 | red |
| ENSBTAG00000046273 | TLCD3A | 6 | red |
| ENSBTAG00000046974 | NA | 6 | red |
| ENSBTAG00000047668 | NA | 6 | red |
| ENSBTAG00000047783 | NA | 6 | red |
| ENSBTAG00000047939 | ZNF75D | 6 | red |
| ENSBTAG00000000169 | ASZ1 | 13 | salmon |
| ENSBTAG00000005976 | HSD17B7 | 13 | salmon |
| ENSBTAG00000006497 | NA | 13 | salmon |
| ENSBTAG00000006804 | KIRREL3 | 13 | salmon |
| ENSBTAG00000007878 | ARHGAP22 | 13 | salmon |
| ENSBTAG00000008466 | CCNB1IP1 | 13 | salmon |
| ENSBTAG00000009239 | SLC16A10 | 13 | salmon |
| ENSBTAG00000010193 | RAB7A | 13 | salmon |
| ENSBTAG00000012016 | LMTK3 | 13 | salmon |
| ENSBTAG00000012030 | TTLL1 | 13 | salmon |
| ENSBTAG00000012991 | NA | 13 | salmon |
| ENSBTAG00000015340 | ANGPTL7 | 13 | salmon |
| ENSBTAG00000015341 | DEPTOR | 13 | salmon |
| ENSBTAG00000016867 | HSD17B3 | 13 | salmon |
| ENSBTAG00000017964 | LRRC51 | 13 | salmon |
| ENSBTAG00000020315 | DENND5B | 13 | salmon |
| ENSBTAG00000023147 | LINGO1 | 13 | salmon |
| ENSBTAG00000025062 | MAGEE2 | 13 | salmon |
| ENSBTAG00000028359 | LOC112442867 | 13 | salmon |

| | | | |
|---|---|---|---|
| ENSBTAG00000030124 | NA | 13 | salmon |
| ENSBTAG00000030533 | NA | 13 | salmon |
| ENSBTAG00000037649 | VIPR2 | 13 | salmon |
| ENSBTAG00000043250 | NA | 13 | salmon |
| ENSBTAG00000043482 | NA | 13 | salmon |
| ENSBTAG00000043639 | NA | 13 | salmon |
| ENSBTAG00000044427 | NA | 13 | salmon |
| ENSBTAG00000045494 | NA | 13 | salmon |
| ENSBTAG00000045532 | NA | 13 | salmon |
| ENSBTAG00000045613 | NA | 13 | salmon |
| ENSBTAG00000045632 | NA | 13 | salmon |
| ENSBTAG00000045657 | NA | 13 | salmon |
| ENSBTAG00000045661 | NA | 13 | salmon |
| ENSBTAG00000045710 | NA | 13 | salmon |
| ENSBTAG00000045747 | NA | 13 | salmon |
| ENSBTAG00000045781 | NA | 13 | salmon |
| ENSBTAG00000045818 | NA | 13 | salmon |
| ENSBTAG00000045825 | NA | 13 | salmon |
| ENSBTAG00000045895 | NFASC | 13 | salmon |
| ENSBTAG00000045952 | NA | 13 | salmon |
| ENSBTAG00000045965 | NA | 13 | salmon |
| ENSBTAG00000046133 | NA | 13 | salmon |
| ENSBTAG00000046351 | NA | 13 | salmon |
| ENSBTAG00000046537 | NA | 13 | salmon |
| ENSBTAG00000046680 | NA | 13 | salmon |
| ENSBTAG00000046695 | NA | 13 | salmon |
| ENSBTAG00000046874 | NA | 13 | salmon |
| ENSBTAG00000046882 | NA | 13 | salmon |
| ENSBTAG00000046888 | NA | 13 | salmon |
| ENSBTAG00000046912 | NA | 13 | salmon |
| ENSBTAG00000047042 | NA | 13 | salmon |
| ENSBTAG00000047046 | NA | 13 | salmon |
| ENSBTAG00000047154 | NA | 13 | salmon |
| ENSBTAG00000047157 | NA | 13 | salmon |
| ENSBTAG00000047242 | NA | 13 | salmon |
| ENSBTAG00000047359 | NA | 13 | salmon |
| ENSBTAG00000047370 | NA | 13 | salmon |
| ENSBTAG00000047381 | NA | 13 | salmon |
| ENSBTAG00000047401 | NA | 13 | salmon |
| ENSBTAG00000047481 | NA | 13 | salmon |
| ENSBTAG00000047501 | NA | 13 | salmon |
| ENSBTAG00000047663 | NA | 13 | salmon |
| ENSBTAG00000047754 | NA | 13 | salmon |
| ENSBTAG00000047790 | NA | 13 | salmon |
| ENSBTAG00000047838 | NA | 13 | salmon |
| ENSBTAG00000047852 | NA | 13 | salmon |
| ENSBTAG00000047908 | NA | 13 | salmon |

| | | | |
|---|---|---|---|
| ENSBTAG00000047918 | NA | 13 | salmon |
| ENSBTAG00000047947 | NA | 13 | salmon |
| ENSBTAG00000048000 | NA | 13 | salmon |
| ENSBTAG00000048148 | NA | 13 | salmon |
| ENSBTAG00000048174 | NA | 13 | salmon |
| ENSBTAG00000048185 | NA | 13 | salmon |
| ENSBTAG00000048191 | NA | 13 | salmon |
| ENSBTAG00000048205 | NA | 13 | salmon |
| ENSBTAG00000048255 | NA | 13 | salmon |
| ENSBTAG00000000301 | LOC538702 | 12 | tan |
| ENSBTAG00000000562 | TOMM20 | 12 | tan |
| ENSBTAG00000000609 | LOC509829 | 12 | tan |
| ENSBTAG00000000894 | PGK1 | 12 | tan |
| ENSBTAG00000001197 | SLAMF7 | 12 | tan |
| ENSBTAG00000001656 | TMEM254 | 12 | tan |
| ENSBTAG00000001898 | CCDC112 | 12 | tan |
| ENSBTAG00000002256 | YEATS4 | 12 | tan |
| ENSBTAG00000002382 | DDX10 | 12 | tan |
| ENSBTAG00000003229 | RPL23 | 12 | tan |
| ENSBTAG00000003276 | PRKCH | 12 | tan |
| ENSBTAG00000003545 | TAF1D | 12 | tan |
| ENSBTAG00000003609 | PBDC1 | 12 | tan |
| ENSBTAG00000004471 | ST3GAL6 | 12 | tan |
| ENSBTAG00000005142 | RPL37 | 12 | tan |
| ENSBTAG00000006152 | NA | 12 | tan |
| ENSBTAG00000006167 | BTBD8 | 12 | tan |
| ENSBTAG00000006397 | NA | 12 | tan |
| ENSBTAG00000006702 | EIF3E | 12 | tan |
| ENSBTAG00000006743 | RBBP8 | 12 | tan |
| ENSBTAG00000007321 | SREK1 | 12 | tan |
| ENSBTAG00000007692 | NA | 12 | tan |
| ENSBTAG00000007939 | CHMP2B | 12 | tan |
| ENSBTAG00000008124 | GK | 12 | tan |
| ENSBTAG00000008683 | LDHA | 12 | tan |
| ENSBTAG00000008716 | SSB | 12 | tan |
| ENSBTAG00000009368 | RRAGC | 12 | tan |
| ENSBTAG00000009469 | SREK1IP1 | 12 | tan |
| ENSBTAG00000009632 | LARP7 | 12 | tan |
| ENSBTAG00000009689 | RWDD1 | 12 | tan |
| ENSBTAG00000009949 | DARS1 | 12 | tan |
| ENSBTAG00000010006 | EMC2 | 12 | tan |
| ENSBTAG00000011299 | NA | 12 | tan |
| ENSBTAG00000011419 | HSPA9 | 12 | tan |
| ENSBTAG00000012003 | PPIA | 12 | tan |
| ENSBTAG00000012074 | MYB | 12 | tan |
| ENSBTAG00000012276 | LOC784446 | 12 | tan |
| ENSBTAG00000012448 | CCDC91 | 12 | tan |

| | | | |
|---|---|---|---|
| ENSBTAG00000012649 | LSM8 | 12 | tan |
| ENSBTAG00000012658 | TMA16 | 12 | tan |
| ENSBTAG00000012828 | TBC1D15 | 12 | tan |
| ENSBTAG00000012902 | ARL8B | 12 | tan |
| ENSBTAG00000013264 | RPS24 | 12 | tan |
| ENSBTAG00000013793 | NEDD4 | 12 | tan |
| ENSBTAG00000014027 | TRIM36 | 12 | tan |
| ENSBTAG00000014208 | RPL35A | 12 | tan |
| ENSBTAG00000014294 | ST7L | 12 | tan |
| ENSBTAG00000015497 | NA | 12 | tan |
| ENSBTAG00000016391 | LOC100295568 | 12 | tan |
| ENSBTAG00000016451 | HSPA14 | 12 | tan |
| ENSBTAG00000016688 | NA | 12 | tan |
| ENSBTAG00000016720 | RAB1A | 12 | tan |
| ENSBTAG00000016895 | NIPA2 | 12 | tan |
| ENSBTAG00000017124 | GPSM2 | 12 | tan |
| ENSBTAG00000017170 | NA | 12 | tan |
| ENSBTAG00000017248 | LOC787187 | 12 | tan |
| ENSBTAG00000017633 | EIF1AX | 12 | tan |
| ENSBTAG00000017801 | ATP6AP2 | 12 | tan |
| ENSBTAG00000018275 | LOC507930 | 12 | tan |
| ENSBTAG00000018922 | TRMT10B | 12 | tan |
| ENSBTAG00000019794 | SYPL1 | 12 | tan |
| ENSBTAG00000019892 | HAS2 | 12 | tan |
| ENSBTAG00000020368 | NA | 12 | tan |
| ENSBTAG00000020432 | MIS12 | 12 | tan |
| ENSBTAG00000020733 | RPS15A | 12 | tan |
| ENSBTAG00000020739 | NXT2 | 12 | tan |
| ENSBTAG00000021116 | EXOC6 | 12 | tan |
| ENSBTAG00000021485 | C3H1orf54 | 12 | tan |
| ENSBTAG00000021801 | INPP5F | 12 | tan |
| ENSBTAG00000024125 | LOC100295881 | 12 | tan |
| ENSBTAG00000025604 | NA | 12 | tan |
| ENSBTAG00000031082 | NA | 12 | tan |
| ENSBTAG00000034255 | EIF5B | 12 | tan |
| ENSBTAG00000034503 | NA | 12 | tan |
| ENSBTAG00000034656 | LOC781416 | 12 | tan |
| ENSBTAG00000034844 | LOC100295757 | 12 | tan |
| ENSBTAG00000035392 | NA | 12 | tan |
| ENSBTAG00000035660 | LOC514680 | 12 | tan |
| ENSBTAG00000037600 | NA | 12 | tan |
| ENSBTAG00000037931 | NA | 12 | tan |
| ENSBTAG00000038379 | LOC101902561 | 12 | tan |
| ENSBTAG00000039121 | PTP4A2 | 12 | tan |
| ENSBTAG00000039245 | GK2 | 12 | tan |
| ENSBTAG00000042524 | NA | 12 | tan |
| ENSBTAG00000043546 | ND6 | 12 | tan |

| | | | |
|---|---|---|---|
| ENSBTAG00000043549 | NA | 12 | tan |
| ENSBTAG00000043550 | CYTB | 12 | tan |
| ENSBTAG00000043555 | NA | 12 | tan |
| ENSBTAG00000043556 | COX2 | 12 | tan |
| ENSBTAG00000043558 | ND1 | 12 | tan |
| ENSBTAG00000043559 | ND4L | 12 | tan |
| ENSBTAG00000043561 | COX1 | 12 | tan |
| ENSBTAG00000043564 | ATP8 | 12 | tan |
| ENSBTAG00000043565 | NA | 12 | tan |
| ENSBTAG00000043568 | ND3 | 12 | tan |
| ENSBTAG00000043571 | ND2 | 12 | tan |
| ENSBTAG00000043572 | NA | 12 | tan |
| ENSBTAG00000043577 | ND4 | 12 | tan |
| ENSBTAG00000043582 | NA | 12 | tan |
| ENSBTAG00000043584 | ATP6 | 12 | tan |
| ENSBTAG00000045772 | NA | 12 | tan |
| ENSBTAG00000045867 | RPGR | 12 | tan |
| ENSBTAG00000045937 | NA | 12 | tan |
| ENSBTAG00000046100 | NA | 12 | tan |
| ENSBTAG00000046120 | NA | 12 | tan |
| ENSBTAG00000046623 | LOC782136 | 12 | tan |
| ENSBTAG00000046850 | NA | 12 | tan |
| ENSBTAG00000046952 | LOC784931 | 12 | tan |
| ENSBTAG00000047064 | NA | 12 | tan |
| ENSBTAG00000047136 | LOC101902490 | 12 | tan |
| ENSBTAG00000047354 | NA | 12 | tan |
| ENSBTAG00000047537 | NA | 12 | tan |
| ENSBTAG00000047548 | NA | 12 | tan |
| ENSBTAG00000047647 | LOC784731 | 12 | tan |
| ENSBTAG00000047744 | NA | 12 | tan |
| ENSBTAG00000047787 | LOC619131 | 12 | tan |
| ENSBTAG00000048179 | LOC782835 | 12 | tan |
| ENSBTAG00000000012 | TTC33 | 1 | turquoise |
| ENSBTAG00000000013 | PRKAA1 | 1 | turquoise |
| ENSBTAG00000000016 | LTA | 1 | turquoise |
| ENSBTAG00000000065 | CRLS1 | 1 | turquoise |
| ENSBTAG00000000088 | ALCAM | 1 | turquoise |
| ENSBTAG00000000095 | CD274 | 1 | turquoise |
| ENSBTAG00000000098 | SETDB1 | 1 | turquoise |
| ENSBTAG00000000113 | ARHGEF2 | 1 | turquoise |
| ENSBTAG00000000115 | VPS54 | 1 | turquoise |
| ENSBTAG00000000128 | FGF18 | 1 | turquoise |
| ENSBTAG00000000131 | SENP3 | 1 | turquoise |
| ENSBTAG00000000132 | EIF4A1 | 1 | turquoise |
| ENSBTAG00000000137 | FRYL | 1 | turquoise |
| ENSBTAG00000000152 | RANBP2 | 1 | turquoise |
| ENSBTAG00000000163 | DDIT4 | 1 | turquoise |

| | | | |
|---|---|---|---|
| ENSBTAG00000000176 | SP3 | 1 | turquoise |
| ENSBTAG00000000181 | SUSD3 | 1 | turquoise |
| ENSBTAG00000000199 | PDP1 | 1 | turquoise |
| ENSBTAG00000000202 | SLC25A19 | 1 | turquoise |
| ENSBTAG00000000203 | ATP6V1G1 | 1 | turquoise |
| ENSBTAG00000000204 | TMEM268 | 1 | turquoise |
| ENSBTAG00000000210 | ARHGAP31 | 1 | turquoise |
| ENSBTAG00000000222 | ARID4B | 1 | turquoise |
| ENSBTAG00000000225 | POLB | 1 | turquoise |
| ENSBTAG00000000226 | DKK4 | 1 | turquoise |
| ENSBTAG00000000240 | AKAP7 | 1 | turquoise |
| ENSBTAG00000000241 | STAU1 | 1 | turquoise |
| ENSBTAG00000000242 | NA | 1 | turquoise |
| ENSBTAG00000000244 | NRXN2 | 1 | turquoise |
| ENSBTAG00000000256 | NA | 1 | turquoise |
| ENSBTAG00000000266 | NAB1 | 1 | turquoise |
| ENSBTAG00000000275 | PARP11 | 1 | turquoise |
| ENSBTAG00000000277 | IL18 | 1 | turquoise |
| ENSBTAG00000000283 | CSF1 | 1 | turquoise |
| ENSBTAG00000000288 | UPF2 | 1 | turquoise |
| ENSBTAG00000000290 | ATP11B | 1 | turquoise |
| ENSBTAG00000000292 | TCIRG1 | 1 | turquoise |
| ENSBTAG00000000308 | ITCH | 1 | turquoise |
| ENSBTAG00000000312 | GRINA | 1 | turquoise |
| ENSBTAG00000000320 | UBAP1 | 1 | turquoise |
| ENSBTAG00000000333 | ARHGAP20 | 1 | turquoise |
| ENSBTAG00000000336 | LOC112442378 | 1 | turquoise |
| ENSBTAG00000000347 | RHOG | 1 | turquoise |
| ENSBTAG00000000356 | CCDC137 | 1 | turquoise |
| ENSBTAG00000000377 | BMX | 1 | turquoise |
| ENSBTAG00000000380 | NA | 1 | turquoise |
| ENSBTAG00000000381 | S1PR4 | 1 | turquoise |
| ENSBTAG00000000386 | NA | 1 | turquoise |
| ENSBTAG00000000396 | PIM1 | 1 | turquoise |
| ENSBTAG00000000415 | ERBIN | 1 | turquoise |
| ENSBTAG00000000428 | BAK1 | 1 | turquoise |
| ENSBTAG00000000436 | TNFAIP3 | 1 | turquoise |
| ENSBTAG00000000446 | ATP11A | 1 | turquoise |
| ENSBTAG00000000483 | HYAL1 | 1 | turquoise |
| ENSBTAG00000000484 | HYAL2 | 1 | turquoise |
| ENSBTAG00000000504 | GTF2B | 1 | turquoise |
| ENSBTAG00000000505 | KYAT3 | 1 | turquoise |
| ENSBTAG00000000507 | NR4A1 | 1 | turquoise |
| ENSBTAG00000000510 | ATG101 | 1 | turquoise |
| ENSBTAG00000000527 | ZFAND3 | 1 | turquoise |
| ENSBTAG00000000551 | SMNDC1 | 1 | turquoise |
| ENSBTAG00000000563 | MYD88 | 1 | turquoise |

| | | | |
|---|---|---|---|
| ENSBTAG00000000569 | HES1 | 1 | turquoise |
| ENSBTAG00000000570 | RET | 1 | turquoise |
| ENSBTAG00000000571 | ARHGAP27 | 1 | turquoise |
| ENSBTAG00000000579 | NIPBL | 1 | turquoise |
| ENSBTAG00000000639 | APRT | 1 | turquoise |
| ENSBTAG00000000653 | PPP1R16B | 1 | turquoise |
| ENSBTAG00000000671 | PARP3 | 1 | turquoise |
| ENSBTAG00000000672 | OTULINL | 1 | turquoise |
| ENSBTAG00000000675 | PGS1 | 1 | turquoise |
| ENSBTAG00000000682 | RAPGEF6 | 1 | turquoise |
| ENSBTAG00000000684 | ARHGEF10L | 1 | turquoise |
| ENSBTAG00000000697 | RRP8 | 1 | turquoise |
| ENSBTAG00000000711 | NDRG1 | 1 | turquoise |
| ENSBTAG00000000725 | USP42 | 1 | turquoise |
| ENSBTAG00000000731 | WFIKKN2 | 1 | turquoise |
| ENSBTAG00000000735 | BCL2A1 | 1 | turquoise |
| ENSBTAG00000000737 | DMXL2 | 1 | turquoise |
| ENSBTAG00000000746 | KAT2B | 1 | turquoise |
| ENSBTAG00000000748 | GAR1 | 1 | turquoise |
| ENSBTAG00000000771 | DNAJB6 | 1 | turquoise |
| ENSBTAG00000000783 | TGFA | 1 | turquoise |
| ENSBTAG00000000786 | NQO2 | 1 | turquoise |
| ENSBTAG00000000806 | ATAD1 | 1 | turquoise |
| ENSBTAG00000000812 | CD300A | 1 | turquoise |
| ENSBTAG00000000816 | PRDM1 | 1 | turquoise |
| ENSBTAG00000000820 | GNG11 | 1 | turquoise |
| ENSBTAG00000000854 | SEC16B | 1 | turquoise |
| ENSBTAG00000000873 | SLC13A3 | 1 | turquoise |
| ENSBTAG00000000874 | CAST | 1 | turquoise |
| ENSBTAG00000000878 | RB1CC1 | 1 | turquoise |
| ENSBTAG00000000892 | CGAS | 1 | turquoise |
| ENSBTAG00000000900 | LRRC8C | 1 | turquoise |
| ENSBTAG00000000920 | DNAH17 | 1 | turquoise |
| ENSBTAG00000000930 | NA | 1 | turquoise |
| ENSBTAG00000000936 | SLC39A2 | 1 | turquoise |
| ENSBTAG00000000937 | ITPRID2 | 1 | turquoise |
| ENSBTAG00000000957 | CDKN2AIP | 1 | turquoise |
| ENSBTAG00000000961 | NEURL2 | 1 | turquoise |
| ENSBTAG00000000964 | FCRL5 | 1 | turquoise |
| ENSBTAG00000000973 | KCNJ15 | 1 | turquoise |
| ENSBTAG00000000990 | PSMA2 | 1 | turquoise |
| ENSBTAG00000000993 | N4BP2L1 | 1 | turquoise |
| ENSBTAG00000000995 | TENT5A | 1 | turquoise |
| ENSBTAG00000001009 | HCLS1 | 1 | turquoise |
| ENSBTAG00000001015 | TM9SF4 | 1 | turquoise |
| ENSBTAG00000001029 | RASGRP2 | 1 | turquoise |
| ENSBTAG00000001030 | MTMR3 | 1 | turquoise |

| | | | |
|---|---|---|---|
| ENSBTAG00000001032 | PYGM | 1 | turquoise |
| ENSBTAG00000001034 | IL18R1 | 1 | turquoise |
| ENSBTAG00000001042 | MXD1 | 1 | turquoise |
| ENSBTAG00000001051 | NA | 1 | turquoise |
| ENSBTAG00000001060 | CXCR4 | 1 | turquoise |
| ENSBTAG00000001085 | FAM20A | 1 | turquoise |
| ENSBTAG00000001102 | SERPINB10 | 1 | turquoise |
| ENSBTAG00000001104 | CMTM4 | 1 | turquoise |
| ENSBTAG00000001120 | CORO6 | 1 | turquoise |
| ENSBTAG00000001124 | GALC | 1 | turquoise |
| ENSBTAG00000001125 | ADAMDEC1 | 1 | turquoise |
| ENSBTAG00000001126 | BMP2K | 1 | turquoise |
| ENSBTAG00000001131 | SLC12A9 | 1 | turquoise |
| ENSBTAG00000001136 | NA | 1 | turquoise |
| ENSBTAG00000001141 | ADAM17 | 1 | turquoise |
| ENSBTAG00000001143 | LOC512486 | 1 | turquoise |
| ENSBTAG00000001146 | HIVEP2 | 1 | turquoise |
| ENSBTAG00000001154 | NA | 1 | turquoise |
| ENSBTAG00000001160 | GMEB1 | 1 | turquoise |
| ENSBTAG00000001173 | PLXNA2 | 1 | turquoise |
| ENSBTAG00000001178 | USPL1 | 1 | turquoise |
| ENSBTAG00000001185 | KMT5B | 1 | turquoise |
| ENSBTAG00000001246 | ATP1A1 | 1 | turquoise |
| ENSBTAG00000001252 | HIVEP1 | 1 | turquoise |
| ENSBTAG00000001255 | CRYBB3 | 1 | turquoise |
| ENSBTAG00000001258 | TMEM50B | 1 | turquoise |
| ENSBTAG00000001269 | TMEM165 | 1 | turquoise |
| ENSBTAG00000001273 | NLRP3 | 1 | turquoise |
| ENSBTAG00000001283 | RIC1 | 1 | turquoise |
| ENSBTAG00000001288 | MAOB | 1 | turquoise |
| ENSBTAG00000001294 | PPP1R15A | 1 | turquoise |
| ENSBTAG00000001295 | DEPDC5 | 1 | turquoise |
| ENSBTAG00000001308 | LOC522763 | 1 | turquoise |
| ENSBTAG00000001311 | MORF4L2 | 1 | turquoise |
| ENSBTAG00000001314 | TRMT6 | 1 | turquoise |
| ENSBTAG00000001321 | IL1B | 1 | turquoise |
| ENSBTAG00000001325 | UPB1 | 1 | turquoise |
| ENSBTAG00000001338 | CCR3 | 1 | turquoise |
| ENSBTAG00000001346 | STRA8 | 1 | turquoise |
| ENSBTAG00000001368 | LGALS3BP | 1 | turquoise |
| ENSBTAG00000001388 | NA | 1 | turquoise |
| ENSBTAG00000001390 | B4GALT4 | 1 | turquoise |
| ENSBTAG00000001395 | C1H21orf91 | 1 | turquoise |
| ENSBTAG00000001396 | ADAMTS7 | 1 | turquoise |
| ENSBTAG00000001446 | ABRACL | 1 | turquoise |
| ENSBTAG00000001447 | NA | 1 | turquoise |
| ENSBTAG00000001462 | FLT4 | 1 | turquoise |

| | | | |
|---|---|---|---|
| ENSBTAG00000001476 | NA | 1 | turquoise |
| ENSBTAG00000001483 | SRGAP2 | 1 | turquoise |
| ENSBTAG00000001492 | FYB1 | 1 | turquoise |
| ENSBTAG00000001509 | ELK3 | 1 | turquoise |
| ENSBTAG00000001510 | CDK17 | 1 | turquoise |
| ENSBTAG00000001511 | BCL6 | 1 | turquoise |
| ENSBTAG00000001518 | ISG20L2 | 1 | turquoise |
| ENSBTAG00000001519 | METTL25B | 1 | turquoise |
| ENSBTAG00000001533 | HACE1 | 1 | turquoise |
| ENSBTAG00000001552 | EPC1 | 1 | turquoise |
| ENSBTAG00000001562 | NFE2 | 1 | turquoise |
| ENSBTAG00000001573 | JMJD1C | 1 | turquoise |
| ENSBTAG00000001576 | TMEM140 | 1 | turquoise |
| ENSBTAG00000001578 | ADPGK | 1 | turquoise |
| ENSBTAG00000001579 | AGO2 | 1 | turquoise |
| ENSBTAG00000001592 | INSIG1 | 1 | turquoise |
| ENSBTAG00000001602 | IL4R | 1 | turquoise |
| ENSBTAG00000001634 | PHF1 | 1 | turquoise |
| ENSBTAG00000001637 | FUNDC1 | 1 | turquoise |
| ENSBTAG00000001652 | SLCO3A1 | 1 | turquoise |
| ENSBTAG00000001657 | PICALM | 1 | turquoise |
| ENSBTAG00000001683 | NA | 1 | turquoise |
| ENSBTAG00000001725 | CXCL10 | 1 | turquoise |
| ENSBTAG00000001728 | IGSF10 | 1 | turquoise |
| ENSBTAG00000001729 | DUSP10 | 1 | turquoise |
| ENSBTAG00000001737 | TUT7 | 1 | turquoise |
| ENSBTAG00000001749 | SENP2 | 1 | turquoise |
| ENSBTAG00000001763 | NR2C2AP | 1 | turquoise |
| ENSBTAG00000001775 | RINL | 1 | turquoise |
| ENSBTAG00000001778 | NFKBIB | 1 | turquoise |
| ENSBTAG00000001785 | TGM3 | 1 | turquoise |
| ENSBTAG00000001788 | PDPN | 1 | turquoise |
| ENSBTAG00000001790 | SAFB2 | 1 | turquoise |
| ENSBTAG00000001821 | PRRC2C | 1 | turquoise |
| ENSBTAG00000001822 | SNX10 | 1 | turquoise |
| ENSBTAG00000001824 | SLC2A6 | 1 | turquoise |
| ENSBTAG00000001856 | POLR2C | 1 | turquoise |
| ENSBTAG00000001864 | NR4A3 | 1 | turquoise |
| ENSBTAG00000001874 | DOCK3 | 1 | turquoise |
| ENSBTAG00000001881 | SLC10A1 | 1 | turquoise |
| ENSBTAG00000001887 | STAM2 | 1 | turquoise |
| ENSBTAG00000001928 | PDIA6 | 1 | turquoise |
| ENSBTAG00000001941 | SEMA4D | 1 | turquoise |
| ENSBTAG00000001945 | ARG2 | 1 | turquoise |
| ENSBTAG00000001961 | MAP1B | 1 | turquoise |
| ENSBTAG00000002010 | PI4K2A | 1 | turquoise |
| ENSBTAG00000002020 | CREBRF | 1 | turquoise |

| | | | |
|---|---|---|---|
| ENSBTAG00000002027 | FAM167B | 1 | turquoise |
| ENSBTAG00000002035 | NA | 1 | turquoise |
| ENSBTAG00000002051 | ARAP1 | 1 | turquoise |
| ENSBTAG00000002068 | TAGLN2 | 1 | turquoise |
| ENSBTAG00000002069 | BOLA | 1 | turquoise |
| ENSBTAG00000002078 | KDM4A | 1 | turquoise |
| ENSBTAG00000002082 | MARCKS | 1 | turquoise |
| ENSBTAG00000002092 | PI16 | 1 | turquoise |
| ENSBTAG00000002096 | FCGR3A | 1 | turquoise |
| ENSBTAG00000002103 | PLCG2 | 1 | turquoise |
| ENSBTAG00000002107 | FZD1 | 1 | turquoise |
| ENSBTAG00000002125 | GNAL | 1 | turquoise |
| ENSBTAG00000002129 | KLF5 | 1 | turquoise |
| ENSBTAG00000002135 | CD69 | 1 | turquoise |
| ENSBTAG00000002147 | PPP4R2 | 1 | turquoise |
| ENSBTAG00000002148 | RAB3D | 1 | turquoise |
| ENSBTAG00000002154 | GPR141 | 1 | turquoise |
| ENSBTAG00000002163 | PTGER2 | 1 | turquoise |
| ENSBTAG00000002164 | AXDND1 | 1 | turquoise |
| ENSBTAG00000002182 | COX17 | 1 | turquoise |
| ENSBTAG00000002190 | DOCK8 | 1 | turquoise |
| ENSBTAG00000002191 | RRP12 | 1 | turquoise |
| ENSBTAG00000002233 | CPNE2 | 1 | turquoise |
| ENSBTAG00000002240 | GPR18 | 1 | turquoise |
| ENSBTAG00000002243 | SNX15 | 1 | turquoise |
| ENSBTAG00000002251 | NA | 1 | turquoise |
| ENSBTAG00000002271 | CDADC1 | 1 | turquoise |
| ENSBTAG00000002279 | LUC7L3 | 1 | turquoise |
| ENSBTAG00000002282 | EIF1 | 1 | turquoise |
| ENSBTAG00000002290 | NA | 1 | turquoise |
| ENSBTAG00000002313 | ITPR2 | 1 | turquoise |
| ENSBTAG00000002333 | HOPX | 1 | turquoise |
| ENSBTAG00000002340 | STEAP4 | 1 | turquoise |
| ENSBTAG00000002346 | HSD3B7 | 1 | turquoise |
| ENSBTAG00000002349 | STX1B | 1 | turquoise |
| ENSBTAG00000002355 | LOC112441543 | 1 | turquoise |
| ENSBTAG00000002356 | RBM47 | 1 | turquoise |
| ENSBTAG00000002357 | TICAM2 | 1 | turquoise |
| ENSBTAG00000002362 | APOLD1 | 1 | turquoise |
| ENSBTAG00000002363 | SESN2 | 1 | turquoise |
| ENSBTAG00000002402 | IGF2R | 1 | turquoise |
| ENSBTAG00000002416 | LOC783604 | 1 | turquoise |
| ENSBTAG00000002425 | SCN9A | 1 | turquoise |
| ENSBTAG00000002432 | ABHD13 | 1 | turquoise |
| ENSBTAG00000002435 | PTPRE | 1 | turquoise |
| ENSBTAG00000002450 | NA | 1 | turquoise |
| ENSBTAG00000002455 | KLHL8 | 1 | turquoise |

| | | | |
|---|---|---|---|
| ENSBTAG00000002477 | MRM2 | 1 | turquoise |
| ENSBTAG00000002478 | AGMAT | 1 | turquoise |
| ENSBTAG00000002480 | NUDT1 | 1 | turquoise |
| ENSBTAG00000002485 | TNS1 | 1 | turquoise |
| ENSBTAG00000002527 | ZSWIM4 | 1 | turquoise |
| ENSBTAG00000002562 | PRAF2 | 1 | turquoise |
| ENSBTAG00000002563 | WDR45 | 1 | turquoise |
| ENSBTAG00000002596 | VSTM1 | 1 | turquoise |
| ENSBTAG00000002615 | LONRF3 | 1 | turquoise |
| ENSBTAG00000002623 | SAMSN1 | 1 | turquoise |
| ENSBTAG00000002624 | PSTPIP2 | 1 | turquoise |
| ENSBTAG00000002628 | SLC44A2 | 1 | turquoise |
| ENSBTAG00000002641 | NA | 1 | turquoise |
| ENSBTAG00000002647 | RGS10 | 1 | turquoise |
| ENSBTAG00000002650 | ZPR1 | 1 | turquoise |
| ENSBTAG00000002690 | BLZF1 | 1 | turquoise |
| ENSBTAG00000002705 | REC8 | 1 | turquoise |
| ENSBTAG00000002724 | HORMAD1 | 1 | turquoise |
| ENSBTAG00000002738 | S1PR2 | 1 | turquoise |
| ENSBTAG00000002743 | OSGEP | 1 | turquoise |
| ENSBTAG00000002755 | ANKRD12 | 1 | turquoise |
| ENSBTAG00000002758 | NA | 1 | turquoise |
| ENSBTAG00000002764 | IGFLR1 | 1 | turquoise |
| ENSBTAG00000002765 | CYP24A1 | 1 | turquoise |
| ENSBTAG00000002773 | CD300LF | 1 | turquoise |
| ENSBTAG00000002798 | NABP2 | 1 | turquoise |
| ENSBTAG00000002816 | SARM1 | 1 | turquoise |
| ENSBTAG00000002834 | CCDC69 | 1 | turquoise |
| ENSBTAG00000002842 | HEATR4 | 1 | turquoise |
| ENSBTAG00000002845 | CDH10 | 1 | turquoise |
| ENSBTAG00000002846 | TRAF3IP3 | 1 | turquoise |
| ENSBTAG00000002850 | HEATR9 | 1 | turquoise |
| ENSBTAG00000002866 | RPIA | 1 | turquoise |
| ENSBTAG00000002869 | ARL4A | 1 | turquoise |
| ENSBTAG00000002882 | LMNB1 | 1 | turquoise |
| ENSBTAG00000002900 | LOC511898 | 1 | turquoise |
| ENSBTAG00000002908 | GNG4 | 1 | turquoise |
| ENSBTAG00000002912 | NA | 1 | turquoise |
| ENSBTAG00000002917 | PRKAG2 | 1 | turquoise |
| ENSBTAG00000002920 | KAT6B | 1 | turquoise |
| ENSBTAG00000002922 | GAB2 | 1 | turquoise |
| ENSBTAG00000002937 | ADGRE3 | 1 | turquoise |
| ENSBTAG00000002942 | SLC2A10 | 1 | turquoise |
| ENSBTAG00000002947 | LY9 | 1 | turquoise |
| ENSBTAG00000002951 | CD244 | 1 | turquoise |
| ENSBTAG00000002959 | LRCH1 | 1 | turquoise |
| ENSBTAG00000002962 | PPM1M | 1 | turquoise |

| | | | |
|---|---|---|---|
| ENSBTAG00000002970 | INTS6 | 1 | turquoise |
| ENSBTAG00000002974 | FMO2 | 1 | turquoise |
| ENSBTAG00000002977 | ECE1 | 1 | turquoise |
| ENSBTAG00000002996 | SHROOM4 | 1 | turquoise |
| ENSBTAG00000003012 | TRAF1 | 1 | turquoise |
| ENSBTAG00000003016 | PTPN7 | 1 | turquoise |
| ENSBTAG00000003033 | GADD45G | 1 | turquoise |
| ENSBTAG00000003037 | NA | 1 | turquoise |
| ENSBTAG00000003038 | TAP2 | 1 | turquoise |
| ENSBTAG00000003039 | PSMB8 | 1 | turquoise |
| ENSBTAG00000003063 | SYNJ1 | 1 | turquoise |
| ENSBTAG00000003074 | SLC16A6 | 1 | turquoise |
| ENSBTAG00000003075 | DVL2 | 1 | turquoise |
| ENSBTAG00000003109 | ITM2B | 1 | turquoise |
| ENSBTAG00000003113 | NA | 1 | turquoise |
| ENSBTAG00000003128 | SPATA6 | 1 | turquoise |
| ENSBTAG00000003147 | JAK1 | 1 | turquoise |
| ENSBTAG00000003151 | DNAJB11 | 1 | turquoise |
| ENSBTAG00000003191 | FSCN1 | 1 | turquoise |
| ENSBTAG00000003197 | NA | 1 | turquoise |
| ENSBTAG00000003221 | ATF7IP | 1 | turquoise |
| ENSBTAG00000003237 | IQSEC1 | 1 | turquoise |
| ENSBTAG00000003245 | BCAR3 | 1 | turquoise |
| ENSBTAG00000003257 | STK4 | 1 | turquoise |
| ENSBTAG00000003264 | NR1H2 | 1 | turquoise |
| ENSBTAG00000003289 | LOC616364 | 1 | turquoise |
| ENSBTAG00000003305 | NCF1 | 1 | turquoise |
| ENSBTAG00000003312 | CHST15 | 1 | turquoise |
| ENSBTAG00000003314 | SKA3 | 1 | turquoise |
| ENSBTAG00000003316 | SYAP1 | 1 | turquoise |
| ENSBTAG00000003322 | TTLL7 | 1 | turquoise |
| ENSBTAG00000003325 | SNRK | 1 | turquoise |
| ENSBTAG00000003326 | NAIP | 1 | turquoise |
| ENSBTAG00000003327 | RUFY4 | 1 | turquoise |
| ENSBTAG00000003341 | DOK2 | 1 | turquoise |
| ENSBTAG00000003352 | NA | 1 | turquoise |
| ENSBTAG00000003353 | SLC6A2 | 1 | turquoise |
| ENSBTAG00000003359 | ELOVL5 | 1 | turquoise |
| ENSBTAG00000003362 | HSP90B1 | 1 | turquoise |
| ENSBTAG00000003366 | DDX58 | 1 | turquoise |
| ENSBTAG00000003386 | NA | 1 | turquoise |
| ENSBTAG00000003397 | CTBP2 | 1 | turquoise |
| ENSBTAG00000003417 | PHKG2 | 1 | turquoise |
| ENSBTAG00000003436 | RHBDF2 | 1 | turquoise |
| ENSBTAG00000003449 | KCP | 1 | turquoise |
| ENSBTAG00000003489 | IPCEF1 | 1 | turquoise |
| ENSBTAG00000003495 | KDM7A | 1 | turquoise |

| | | | |
|---|---|---|---|
| ENSBTAG00000003499 | ENPP4 | 1 | turquoise |
| ENSBTAG00000003506 | STEAP2 | 1 | turquoise |
| ENSBTAG00000003508 | CFAP69 | 1 | turquoise |
| ENSBTAG00000003510 | MMRN2 | 1 | turquoise |
| ENSBTAG00000003525 | STXBP1 | 1 | turquoise |
| ENSBTAG00000003526 | SYNE3 | 1 | turquoise |
| ENSBTAG00000003528 | NA | 1 | turquoise |
| ENSBTAG00000003531 | ABCA13 | 1 | turquoise |
| ENSBTAG00000003532 | TLE4 | 1 | turquoise |
| ENSBTAG00000003536 | MARCHF6 | 1 | turquoise |
| ENSBTAG00000003541 | ZNF614 | 1 | turquoise |
| ENSBTAG00000003572 | F11 | 1 | turquoise |
| ENSBTAG00000003585 | CD47 | 1 | turquoise |
| ENSBTAG00000003586 | MAN1A2 | 1 | turquoise |
| ENSBTAG00000003587 | ELL | 1 | turquoise |
| ENSBTAG00000003588 | ZFAND1 | 1 | turquoise |
| ENSBTAG00000003602 | RBPJ | 1 | turquoise |
| ENSBTAG00000003639 | ELMO2 | 1 | turquoise |
| ENSBTAG00000003650 | NR4A2 | 1 | turquoise |
| ENSBTAG00000003665 | NUP210 | 1 | turquoise |
| ENSBTAG00000003693 | SUN2 | 1 | turquoise |
| ENSBTAG00000003719 | TDRD7 | 1 | turquoise |
| ENSBTAG00000003743 | NA | 1 | turquoise |
| ENSBTAG00000003748 | CNOT2 | 1 | turquoise |
| ENSBTAG00000003758 | TKT | 1 | turquoise |
| ENSBTAG00000003760 | NA | 1 | turquoise |
| ENSBTAG00000003764 | AKIRIN2 | 1 | turquoise |
| ENSBTAG00000003791 | LPAR3 | 1 | turquoise |
| ENSBTAG00000003793 | NGLY1 | 1 | turquoise |
| ENSBTAG00000003820 | GRAMD1A | 1 | turquoise |
| ENSBTAG00000003825 | PTPN12 | 1 | turquoise |
| ENSBTAG00000003836 | ADAM19 | 1 | turquoise |
| ENSBTAG00000003842 | RABGEF1 | 1 | turquoise |
| ENSBTAG00000003845 | CSRNP1 | 1 | turquoise |
| ENSBTAG00000003851 | CCNL1 | 1 | turquoise |
| ENSBTAG00000003857 | SUSD6 | 1 | turquoise |
| ENSBTAG00000003876 | MCU | 1 | turquoise |
| ENSBTAG00000003878 | ZUP1 | 1 | turquoise |
| ENSBTAG00000003880 | EMILIN2 | 1 | turquoise |
| ENSBTAG00000003895 | NA | 1 | turquoise |
| ENSBTAG00000003907 | TSPAN14 | 1 | turquoise |
| ENSBTAG00000003908 | MAPRE1 | 1 | turquoise |
| ENSBTAG00000003916 | DPH3 | 1 | turquoise |
| ENSBTAG00000003920 | TGM1 | 1 | turquoise |
| ENSBTAG00000003922 | MPHOSPH8 | 1 | turquoise |
| ENSBTAG00000003923 | GPBAR1 | 1 | turquoise |
| ENSBTAG00000003928 | ATG16L1 | 1 | turquoise |

| | | | |
|---|---|---|---|
| ENSBTAG00000003934 | PSPC1 | 1 | turquoise |
| ENSBTAG00000003935 | TMBIM1 | 1 | turquoise |
| ENSBTAG00000003936 | PNKD | 1 | turquoise |
| ENSBTAG00000003947 | SSBP4 | 1 | turquoise |
| ENSBTAG00000003957 | NA | 1 | turquoise |
| ENSBTAG00000003961 | SNX20 | 1 | turquoise |
| ENSBTAG00000003970 | MITD1 | 1 | turquoise |
| ENSBTAG00000003990 | ITPRIP | 1 | turquoise |
| ENSBTAG00000004005 | WDR7 | 1 | turquoise |
| ENSBTAG00000004037 | JUN | 1 | turquoise |
| ENSBTAG00000004043 | LOC407171 | 1 | turquoise |
| ENSBTAG00000004051 | SBDS | 1 | turquoise |
| ENSBTAG00000004064 | BPNT1 | 1 | turquoise |
| ENSBTAG00000004066 | PARP8 | 1 | turquoise |
| ENSBTAG00000004099 | CCDC170 | 1 | turquoise |
| ENSBTAG00000004115 | MYLIP | 1 | turquoise |
| ENSBTAG00000004118 | ALAS1 | 1 | turquoise |
| ENSBTAG00000004124 | CPOX | 1 | turquoise |
| ENSBTAG00000004135 | LMTK2 | 1 | turquoise |
| ENSBTAG00000004136 | NFE2L3 | 1 | turquoise |
| ENSBTAG00000004139 | BACH1 | 1 | turquoise |
| ENSBTAG00000004150 | NRG1 | 1 | turquoise |
| ENSBTAG00000004154 | SKIL | 1 | turquoise |
| ENSBTAG00000004189 | MLXIP | 1 | turquoise |
| ENSBTAG00000004193 | VEZF1 | 1 | turquoise |
| ENSBTAG00000004194 | RALGDS | 1 | turquoise |
| ENSBTAG00000004196 | FMNL1 | 1 | turquoise |
| ENSBTAG00000004208 | PLEKHG6 | 1 | turquoise |
| ENSBTAG00000004211 | TNFRSF1A | 1 | turquoise |
| ENSBTAG00000004216 | MAP3K2 | 1 | turquoise |
| ENSBTAG00000004224 | C7H19orf38 | 1 | turquoise |
| ENSBTAG00000004243 | LYPLA1 | 1 | turquoise |
| ENSBTAG00000004247 | RPH3A | 1 | turquoise |
| ENSBTAG00000004256 | ODC1 | 1 | turquoise |
| ENSBTAG00000004259 | HPCAL1 | 1 | turquoise |
| ENSBTAG00000004269 | SGK1 | 1 | turquoise |
| ENSBTAG00000004270 | FCER2 | 1 | turquoise |
| ENSBTAG00000004305 | RGS16 | 1 | turquoise |
| ENSBTAG00000004322 | FOS | 1 | turquoise |
| ENSBTAG00000004339 | LOC508153 | 1 | turquoise |
| ENSBTAG00000004349 | DAZAP2 | 1 | turquoise |
| ENSBTAG00000004353 | NA | 1 | turquoise |
| ENSBTAG00000004378 | IL23A | 1 | turquoise |
| ENSBTAG00000004380 | STAT2 | 1 | turquoise |
| ENSBTAG00000004386 | SOCS1 | 1 | turquoise |
| ENSBTAG00000004430 | SGPP1 | 1 | turquoise |
| ENSBTAG00000004443 | NA | 1 | turquoise |

| | | | |
|---|---|---|---|
| ENSBTAG00000004472 | NA | 1 | turquoise |
| ENSBTAG00000004484 | CEP83 | 1 | turquoise |
| ENSBTAG00000004492 | SWT1 | 1 | turquoise |
| ENSBTAG00000004494 | B4GALNT1 | 1 | turquoise |
| ENSBTAG00000004496 | NA | 1 | turquoise |
| ENSBTAG00000004514 | NA | 1 | turquoise |
| ENSBTAG00000004526 | NA | 1 | turquoise |
| ENSBTAG00000004532 | FGFR1OP2 | 1 | turquoise |
| ENSBTAG00000004540 | NUB1 | 1 | turquoise |
| ENSBTAG00000004547 | OLR1 | 1 | turquoise |
| ENSBTAG00000004552 | PBXIP1 | 1 | turquoise |
| ENSBTAG00000004553 | TPM4 | 1 | turquoise |
| ENSBTAG00000004556 | SLC2A3 | 1 | turquoise |
| ENSBTAG00000004558 | C10H15orf48 | 1 | turquoise |
| ENSBTAG00000004560 | CLEC4F | 1 | turquoise |
| ENSBTAG00000004562 | CD207 | 1 | turquoise |
| ENSBTAG00000004587 | DUSP6 | 1 | turquoise |
| ENSBTAG00000004603 | PHC2 | 1 | turquoise |
| ENSBTAG00000004608 | LOC100138951 | 1 | turquoise |
| ENSBTAG00000004635 | LLGL1 | 1 | turquoise |
| ENSBTAG00000004659 | COQ10B | 1 | turquoise |
| ENSBTAG00000004662 | SLC16A12 | 1 | turquoise |
| ENSBTAG00000004674 | HAAO | 1 | turquoise |
| ENSBTAG00000004679 | WARS1 | 1 | turquoise |
| ENSBTAG00000004680 | SLC13A5 | 1 | turquoise |
| ENSBTAG00000004710 | HNF1B | 1 | turquoise |
| ENSBTAG00000004714 | MCEMP1 | 1 | turquoise |
| ENSBTAG00000004716 | RETN | 1 | turquoise |
| ENSBTAG00000004723 | TEX2 | 1 | turquoise |
| ENSBTAG00000004732 | SPTB | 1 | turquoise |
| ENSBTAG00000004736 | GRB2 | 1 | turquoise |
| ENSBTAG00000004741 | IL12B | 1 | turquoise |
| ENSBTAG00000004770 | SCN4A | 1 | turquoise |
| ENSBTAG00000004787 | METRNL | 1 | turquoise |
| ENSBTAG00000004789 | LGR6 | 1 | turquoise |
| ENSBTAG00000004813 | SUSD1 | 1 | turquoise |
| ENSBTAG00000004814 | INPP4A | 1 | turquoise |
| ENSBTAG00000004840 | C1S | 1 | turquoise |
| ENSBTAG00000004842 | RSPRY1 | 1 | turquoise |
| ENSBTAG00000004849 | IGSF3 | 1 | turquoise |
| ENSBTAG00000004850 | KPNA3 | 1 | turquoise |
| ENSBTAG00000004852 | PLEKHO1 | 1 | turquoise |
| ENSBTAG00000004873 | CCNL2 | 1 | turquoise |
| ENSBTAG00000004881 | MTHFD2 | 1 | turquoise |
| ENSBTAG00000004885 | DDR2 | 1 | turquoise |
| ENSBTAG00000004908 | CHRNE | 1 | turquoise |
| ENSBTAG00000004953 | ELK4 | 1 | turquoise |

| | | | |
|---|---|---|---|
| ENSBTAG00000004954 | TOX | 1 | turquoise |
| ENSBTAG00000004961 | GOSR1 | 1 | turquoise |
| ENSBTAG00000004964 | PCGF5 | 1 | turquoise |
| ENSBTAG00000004971 | GRAMD1C | 1 | turquoise |
| ENSBTAG00000004974 | ZDHHC23 | 1 | turquoise |
| ENSBTAG00000004987 | NUP210L | 1 | turquoise |
| ENSBTAG00000004988 | CCKAR | 1 | turquoise |
| ENSBTAG00000004989 | IRF5 | 1 | turquoise |
| ENSBTAG00000004994 | NA | 1 | turquoise |
| ENSBTAG00000004999 | RESF1 | 1 | turquoise |
| ENSBTAG00000005008 | WSB1 | 1 | turquoise |
| ENSBTAG00000005018 | NFAM1 | 1 | turquoise |
| ENSBTAG00000005029 | TAL1 | 1 | turquoise |
| ENSBTAG00000005038 | ZBTB42 | 1 | turquoise |
| ENSBTAG00000005043 | NA | 1 | turquoise |
| ENSBTAG00000005062 | TEC | 1 | turquoise |
| ENSBTAG00000005066 | HSPBAP1 | 1 | turquoise |
| ENSBTAG00000005067 | UBTF | 1 | turquoise |
| ENSBTAG00000005089 | RHBDD2 | 1 | turquoise |
| ENSBTAG00000005091 | DGKG | 1 | turquoise |
| ENSBTAG00000005092 | ROR2 | 1 | turquoise |
| ENSBTAG00000005124 | JADE3 | 1 | turquoise |
| ENSBTAG00000005136 | ABI1 | 1 | turquoise |
| ENSBTAG00000005141 | PELI2 | 1 | turquoise |
| ENSBTAG00000005146 | NA | 1 | turquoise |
| ENSBTAG00000005147 | HPS5 | 1 | turquoise |
| ENSBTAG00000005155 | ARL13B | 1 | turquoise |
| ENSBTAG00000005160 | GTF2H1 | 1 | turquoise |
| ENSBTAG00000005165 | CSGALNACT2 | 1 | turquoise |
| ENSBTAG00000005180 | RBMS1 | 1 | turquoise |
| ENSBTAG00000005181 | SYS1 | 1 | turquoise |
| ENSBTAG00000005182 | BoLA | 1 | turquoise |
| ENSBTAG00000005215 | IL10RA | 1 | turquoise |
| ENSBTAG00000005218 | PDE3B | 1 | turquoise |
| ENSBTAG00000005251 | LOC516599 | 1 | turquoise |
| ENSBTAG00000005273 | IL1R1 | 1 | turquoise |
| ENSBTAG00000005286 | NA | 1 | turquoise |
| ENSBTAG00000005339 | VEGFA | 1 | turquoise |
| ENSBTAG00000005354 | LRRFIP1 | 1 | turquoise |
| ENSBTAG00000005384 | COPZ1 | 1 | turquoise |
| ENSBTAG00000005397 | CD46 | 1 | turquoise |
| ENSBTAG00000005408 | CLK1 | 1 | turquoise |
| ENSBTAG00000005413 | NLRC5 | 1 | turquoise |
| ENSBTAG00000005414 | NA | 1 | turquoise |
| ENSBTAG00000005427 | NRDE2 | 1 | turquoise |
| ENSBTAG00000005430 | MB21D2 | 1 | turquoise |
| ENSBTAG00000005443 | MIER1 | 1 | turquoise |

| | | | |
|---|---|---|---|
| ENSBTAG00000005444 | UGGT1 | 1 | turquoise |
| ENSBTAG00000005448 | LOC521224 | 1 | turquoise |
| ENSBTAG00000005450 | NA | 1 | turquoise |
| ENSBTAG00000005461 | SOX15 | 1 | turquoise |
| ENSBTAG00000005462 | FXR2 | 1 | turquoise |
| ENSBTAG00000005474 | CREB1 | 1 | turquoise |
| ENSBTAG00000005476 | NA | 1 | turquoise |
| ENSBTAG00000005477 | LAPTM5 | 1 | turquoise |
| ENSBTAG00000005483 | ESYT2 | 1 | turquoise |
| ENSBTAG00000005488 | ARIH2 | 1 | turquoise |
| ENSBTAG00000005491 | NA | 1 | turquoise |
| ENSBTAG00000005493 | TBC1D14 | 1 | turquoise |
| ENSBTAG00000005497 | RPRD1A | 1 | turquoise |
| ENSBTAG00000005504 | TP53INP2 | 1 | turquoise |
| ENSBTAG00000005523 | XYLT1 | 1 | turquoise |
| ENSBTAG00000005524 | HBP1 | 1 | turquoise |
| ENSBTAG00000005534 | ENO3 | 1 | turquoise |
| ENSBTAG00000005547 | VCPKMT | 1 | turquoise |
| ENSBTAG00000005564 | PIAS1 | 1 | turquoise |
| ENSBTAG00000005578 | KAT5 | 1 | turquoise |
| ENSBTAG00000005588 | DXO | 1 | turquoise |
| ENSBTAG00000005589 | STK19 | 1 | turquoise |
| ENSBTAG00000005603 | CXCL11 | 1 | turquoise |
| ENSBTAG00000005606 | HOXC9 | 1 | turquoise |
| ENSBTAG00000005622 | LITAF | 1 | turquoise |
| ENSBTAG00000005635 | TTC39C | 1 | turquoise |
| ENSBTAG00000005638 | CABYR | 1 | turquoise |
| ENSBTAG00000005647 | GPR132 | 1 | turquoise |
| ENSBTAG00000005650 | SKAP2 | 1 | turquoise |
| ENSBTAG00000005668 | SLC39A8 | 1 | turquoise |
| ENSBTAG00000005674 | NEU1 | 1 | turquoise |
| ENSBTAG00000005682 | UBAC2 | 1 | turquoise |
| ENSBTAG00000005723 | NEPRO | 1 | turquoise |
| ENSBTAG00000005725 | IDO2 | 1 | turquoise |
| ENSBTAG00000005730 | GPAT4 | 1 | turquoise |
| ENSBTAG00000005745 | HPSE | 1 | turquoise |
| ENSBTAG00000005757 | FUS | 1 | turquoise |
| ENSBTAG00000005761 | DEDD2 | 1 | turquoise |
| ENSBTAG00000005786 | ATXN3 | 1 | turquoise |
| ENSBTAG00000005807 | MICU1 | 1 | turquoise |
| ENSBTAG00000005814 | PSME2 | 1 | turquoise |
| ENSBTAG00000005815 | RNF31 | 1 | turquoise |
| ENSBTAG00000005816 | IRF9 | 1 | turquoise |
| ENSBTAG00000005832 | GRK2 | 1 | turquoise |
| ENSBTAG00000005833 | ETNK1 | 1 | turquoise |
| ENSBTAG00000005841 | NA | 1 | turquoise |
| ENSBTAG00000005861 | CCNT1 | 1 | turquoise |

| | | | |
|---|---|---|---|
| ENSBTAG00000005865 | MAPK6 | 1 | turquoise |
| ENSBTAG00000005903 | LRP5 | 1 | turquoise |
| ENSBTAG00000005923 | ABTB2 | 1 | turquoise |
| ENSBTAG00000005940 | PRKAB1 | 1 | turquoise |
| ENSBTAG00000005947 | PLAU | 1 | turquoise |
| ENSBTAG00000005952 | CEBPE | 1 | turquoise |
| ENSBTAG00000005958 | PTK2B | 1 | turquoise |
| ENSBTAG00000005960 | EPB41L2 | 1 | turquoise |
| ENSBTAG00000005961 | CDC42SE2 | 1 | turquoise |
| ENSBTAG00000005970 | XBP1 | 1 | turquoise |
| ENSBTAG00000005973 | OVGP1 | 1 | turquoise |
| ENSBTAG00000005978 | PIK3R5 | 1 | turquoise |
| ENSBTAG00000005989 | LAP3 | 1 | turquoise |
| ENSBTAG00000005990 | S1PR1 | 1 | turquoise |
| ENSBTAG00000006016 | GTPBP2 | 1 | turquoise |
| ENSBTAG00000006017 | NFKB2 | 1 | turquoise |
| ENSBTAG00000006027 | USP34 | 1 | turquoise |
| ENSBTAG00000006039 | ARHGDIB | 1 | turquoise |
| ENSBTAG00000006043 | LOC515755 | 1 | turquoise |
| ENSBTAG00000006044 | KLHL5 | 1 | turquoise |
| ENSBTAG00000006056 | HEXIM1 | 1 | turquoise |
| ENSBTAG00000006066 | CDS2 | 1 | turquoise |
| ENSBTAG00000006078 | IL15RA | 1 | turquoise |
| ENSBTAG00000006088 | FAM104A | 1 | turquoise |
| ENSBTAG00000006089 | C19H17orf80 | 1 | turquoise |
| ENSBTAG00000006110 | NA | 1 | turquoise |
| ENSBTAG00000006118 | RSRC2 | 1 | turquoise |
| ENSBTAG00000006125 | PLK3 | 1 | turquoise |
| ENSBTAG00000006128 | DYNLT4 | 1 | turquoise |
| ENSBTAG00000006132 | DENND3 | 1 | turquoise |
| ENSBTAG00000006135 | MAP1LC3A | 1 | turquoise |
| ENSBTAG00000006136 | NA | 1 | turquoise |
| ENSBTAG00000006155 | CCRL2 | 1 | turquoise |
| ENSBTAG00000006156 | BST1 | 1 | turquoise |
| ENSBTAG00000006189 | ACTG1 | 1 | turquoise |
| ENSBTAG00000006193 | IRAK2 | 1 | turquoise |
| ENSBTAG00000006194 | FOSL1 | 1 | turquoise |
| ENSBTAG00000006208 | ADCY7 | 1 | turquoise |
| ENSBTAG00000006221 | ADGRG3 | 1 | turquoise |
| ENSBTAG00000006223 | THEMIS2 | 1 | turquoise |
| ENSBTAG00000006227 | IDH3A | 1 | turquoise |
| ENSBTAG00000006236 | NA | 1 | turquoise |
| ENSBTAG00000006240 | TLR4 | 1 | turquoise |
| ENSBTAG00000006243 | GCNT7 | 1 | turquoise |
| ENSBTAG00000006255 | MDM4 | 1 | turquoise |
| ENSBTAG00000006256 | PTPRO | 1 | turquoise |
| ENSBTAG00000006287 | NEDD9 | 1 | turquoise |

| | | | |
|---|---|---|---|
| ENSBTAG00000006291 | CYLD | 1 | turquoise |
| ENSBTAG00000006322 | DENND5A | 1 | turquoise |
| ENSBTAG00000006323 | SNX9 | 1 | turquoise |
| ENSBTAG00000006324 | NAB2 | 1 | turquoise |
| ENSBTAG00000006335 | STAT6 | 1 | turquoise |
| ENSBTAG00000006343 | IL1R2 | 1 | turquoise |
| ENSBTAG00000006345 | ART1 | 1 | turquoise |
| ENSBTAG00000006353 | TXNL4B | 1 | turquoise |
| ENSBTAG00000006354 | HP | 1 | turquoise |
| ENSBTAG00000006360 | CD180 | 1 | turquoise |
| ENSBTAG00000006368 | N4BP3 | 1 | turquoise |
| ENSBTAG00000006378 | RIPK1 | 1 | turquoise |
| ENSBTAG00000006403 | NA | 1 | turquoise |
| ENSBTAG00000006439 | DOCK11 | 1 | turquoise |
| ENSBTAG00000006441 | ATP5PB | 1 | turquoise |
| ENSBTAG00000006466 | CD53 | 1 | turquoise |
| ENSBTAG00000006471 | OSBPL11 | 1 | turquoise |
| ENSBTAG00000006487 | RPS9 | 1 | turquoise |
| ENSBTAG00000006490 | BOLA-DOA | 1 | turquoise |
| ENSBTAG00000006505 | S100A9 | 1 | turquoise |
| ENSBTAG00000006510 | YRDC | 1 | turquoise |
| ENSBTAG00000006523 | SOD2 | 1 | turquoise |
| ENSBTAG00000006539 | SIRPB2 | 1 | turquoise |
| ENSBTAG00000006549 | CALHM6 | 1 | turquoise |
| ENSBTAG00000006558 | NA | 1 | turquoise |
| ENSBTAG00000006561 | RASGEF1B | 1 | turquoise |
| ENSBTAG00000006566 | FFAR2 | 1 | turquoise |
| ENSBTAG00000006573 | KLHL18 | 1 | turquoise |
| ENSBTAG00000006581 | CCDC82 | 1 | turquoise |
| ENSBTAG00000006589 | CFTR | 1 | turquoise |
| ENSBTAG00000006601 | KSR1 | 1 | turquoise |
| ENSBTAG00000006606 | RFLNB | 1 | turquoise |
| ENSBTAG00000006608 | GGT5 | 1 | turquoise |
| ENSBTAG00000006615 | CASP7 | 1 | turquoise |
| ENSBTAG00000006616 | PTPN1 | 1 | turquoise |
| ENSBTAG00000006631 | GLI1 | 1 | turquoise |
| ENSBTAG00000006633 | IRF3 | 1 | turquoise |
| ENSBTAG00000006638 | BCL2L12 | 1 | turquoise |
| ENSBTAG00000006650 | ZNF668 | 1 | turquoise |
| ENSBTAG00000006654 | TANK | 1 | turquoise |
| ENSBTAG00000006663 | CYTH1 | 1 | turquoise |
| ENSBTAG00000006677 | NA | 1 | turquoise |
| ENSBTAG00000006683 | ZFYVE28 | 1 | turquoise |
| ENSBTAG00000006685 | IL10 | 1 | turquoise |
| ENSBTAG00000006695 | VCPIP1 | 1 | turquoise |
| ENSBTAG00000006697 | RICTOR | 1 | turquoise |
| ENSBTAG00000006707 | ACSL5 | 1 | turquoise |

| | | | |
|---|---|---|---|
| ENSBTAG00000006721 | POLR1F | 1 | turquoise |
| ENSBTAG00000006729 | NA | 1 | turquoise |
| ENSBTAG00000006730 | SUSD2 | 1 | turquoise |
| ENSBTAG00000006731 | SLC7A5 | 1 | turquoise |
| ENSBTAG00000006751 | TENT2 | 1 | turquoise |
| ENSBTAG00000006752 | PFKFB4 | 1 | turquoise |
| ENSBTAG00000006771 | FRY | 1 | turquoise |
| ENSBTAG00000006780 | NAPSA | 1 | turquoise |
| ENSBTAG00000006792 | EHD4 | 1 | turquoise |
| ENSBTAG00000006797 | MARCHF3 | 1 | turquoise |
| ENSBTAG00000006823 | CMYA5 | 1 | turquoise |
| ENSBTAG00000006843 | UTP15 | 1 | turquoise |
| ENSBTAG00000006846 | LGALS9 | 1 | turquoise |
| ENSBTAG00000006851 | LOC617565 | 1 | turquoise |
| ENSBTAG00000006859 | LOC100336941 | 1 | turquoise |
| ENSBTAG00000006864 | LOC107131209 | 1 | turquoise |
| ENSBTAG00000006894 | NOS2 | 1 | turquoise |
| ENSBTAG00000006910 | ASRGL1 | 1 | turquoise |
| ENSBTAG00000006921 | ABCA6 | 1 | turquoise |
| ENSBTAG00000006933 | PPP1R10 | 1 | turquoise |
| ENSBTAG00000006937 | NA | 1 | turquoise |
| ENSBTAG00000006939 | FGD3 | 1 | turquoise |
| ENSBTAG00000006951 | LMO2 | 1 | turquoise |
| ENSBTAG00000006984 | CD55 | 1 | turquoise |
| ENSBTAG00000006998 | RASGRP4 | 1 | turquoise |
| ENSBTAG00000006999 | RYR1 | 1 | turquoise |
| ENSBTAG00000007039 | FBXO25 | 1 | turquoise |
| ENSBTAG00000007073 | CPB2 | 1 | turquoise |
| ENSBTAG00000007075 | LOC616942 | 1 | turquoise |
| ENSBTAG00000007077 | ABHD1 | 1 | turquoise |
| ENSBTAG00000007089 | FRG1 | 1 | turquoise |
| ENSBTAG00000007101 | F3 | 1 | turquoise |
| ENSBTAG00000007103 | ITGAL | 1 | turquoise |
| ENSBTAG00000007111 | STEAP3 | 1 | turquoise |
| ENSBTAG00000007118 | SMURF1 | 1 | turquoise |
| ENSBTAG00000007130 | ESF1 | 1 | turquoise |
| ENSBTAG00000007156 | AGAP2 | 1 | turquoise |
| ENSBTAG00000007158 | TSPAN31 | 1 | turquoise |
| ENSBTAG00000007176 | NA | 1 | turquoise |
| ENSBTAG00000007181 | KIFC3 | 1 | turquoise |
| ENSBTAG00000007184 | NUDT7 | 1 | turquoise |
| ENSBTAG00000007191 | NA | 1 | turquoise |
| ENSBTAG00000007192 | SIDT2 | 1 | turquoise |
| ENSBTAG00000007193 | NA | 1 | turquoise |
| ENSBTAG00000007199 | NA | 1 | turquoise |
| ENSBTAG00000007208 | HDAC11 | 1 | turquoise |
| ENSBTAG00000007213 | SIRPA | 1 | turquoise |

| | | | |
|---|---|---|---|
| ENSBTAG00000007214 | ELL2 | 1 | turquoise |
| ENSBTAG00000007228 | CBX7 | 1 | turquoise |
| ENSBTAG00000007239 | TNFAIP6 | 1 | turquoise |
| ENSBTAG00000007268 | F13A1 | 1 | turquoise |
| ENSBTAG00000007280 | NA | 1 | turquoise |
| ENSBTAG00000007300 | FHL3 | 1 | turquoise |
| ENSBTAG00000007304 | CLK3 | 1 | turquoise |
| ENSBTAG00000007307 | SELE | 1 | turquoise |
| ENSBTAG00000007330 | STXBP3 | 1 | turquoise |
| ENSBTAG00000007334 | NFKBID | 1 | turquoise |
| ENSBTAG00000007336 | HCST | 1 | turquoise |
| ENSBTAG00000007343 | NA | 1 | turquoise |
| ENSBTAG00000007348 | STRA6 | 1 | turquoise |
| ENSBTAG00000007356 | ELF1 | 1 | turquoise |
| ENSBTAG00000007357 | CHSY1 | 1 | turquoise |
| ENSBTAG00000007361 | NA | 1 | turquoise |
| ENSBTAG00000007389 | IFI35 | 1 | turquoise |
| ENSBTAG00000007397 | FOLH1B | 1 | turquoise |
| ENSBTAG00000007399 | LAMP2 | 1 | turquoise |
| ENSBTAG00000007409 | NA | 1 | turquoise |
| ENSBTAG00000007414 | PAPOLG | 1 | turquoise |
| ENSBTAG00000007424 | LIF | 1 | turquoise |
| ENSBTAG00000007433 | MYO9A | 1 | turquoise |
| ENSBTAG00000007446 | NGF | 1 | turquoise |
| ENSBTAG00000007447 | LOC531462 | 1 | turquoise |
| ENSBTAG00000007450 | C2 | 1 | turquoise |
| ENSBTAG00000007454 | RPL10 | 1 | turquoise |
| ENSBTAG00000007455 | DNASE1L1 | 1 | turquoise |
| ENSBTAG00000007513 | PGAM5 | 1 | turquoise |
| ENSBTAG00000007519 | ADAR | 1 | turquoise |
| ENSBTAG00000007523 | STX4 | 1 | turquoise |
| ENSBTAG00000007530 | TRIM10 | 1 | turquoise |
| ENSBTAG00000007531 | NCF4 | 1 | turquoise |
| ENSBTAG00000007559 | URI1 | 1 | turquoise |
| ENSBTAG00000007577 | UBXN7 | 1 | turquoise |
| ENSBTAG00000007581 | ADORA2B | 1 | turquoise |
| ENSBTAG00000007584 | INPP1 | 1 | turquoise |
| ENSBTAG00000007592 | RARG | 1 | turquoise |
| ENSBTAG00000007593 | AIDA | 1 | turquoise |
| ENSBTAG00000007599 | IKBKB | 1 | turquoise |
| ENSBTAG00000007624 | FRMD4B | 1 | turquoise |
| ENSBTAG00000007626 | IL2RG | 1 | turquoise |
| ENSBTAG00000007636 | IRAK3 | 1 | turquoise |
| ENSBTAG00000007659 | CORO2A | 1 | turquoise |
| ENSBTAG00000007661 | MYO1F | 1 | turquoise |
| ENSBTAG00000007662 | HSPA5 | 1 | turquoise |
| ENSBTAG00000007675 | NPHP1 | 1 | turquoise |

| | | | |
|---|---|---|---|
| ENSBTAG00000007708 | NLGN3 | 1 | turquoise |
| ENSBTAG00000007709 | SHOC2 | 1 | turquoise |
| ENSBTAG00000007718 | TGIF1 | 1 | turquoise |
| ENSBTAG00000007719 | MFSD6 | 1 | turquoise |
| ENSBTAG00000007788 | GRIPAP1 | 1 | turquoise |
| ENSBTAG00000007804 | RILPL2 | 1 | turquoise |
| ENSBTAG00000007807 | HSPA2 | 1 | turquoise |
| ENSBTAG00000007816 | LOC787045 | 1 | turquoise |
| ENSBTAG00000007823 | TG | 1 | turquoise |
| ENSBTAG00000007828 | SLA | 1 | turquoise |
| ENSBTAG00000007833 | PIAS2 | 1 | turquoise |
| ENSBTAG00000007836 | PPA1 | 1 | turquoise |
| ENSBTAG00000007863 | GLS | 1 | turquoise |
| ENSBTAG00000007867 | STAT1 | 1 | turquoise |
| ENSBTAG00000007875 | SAFB | 1 | turquoise |
| ENSBTAG00000007876 | MAPK8 | 1 | turquoise |
| ENSBTAG00000007881 | LOC100139670 | 1 | turquoise |
| ENSBTAG00000007883 | NA | 1 | turquoise |
| ENSBTAG00000007895 | SLC20A1 | 1 | turquoise |
| ENSBTAG00000007901 | LOC508459 | 1 | turquoise |
| ENSBTAG00000007921 | DAPP1 | 1 | turquoise |
| ENSBTAG00000007922 | RNF24 | 1 | turquoise |
| ENSBTAG00000007923 | LAMTOR3 | 1 | turquoise |
| ENSBTAG00000007927 | SLAMF1 | 1 | turquoise |
| ENSBTAG00000007932 | HCK | 1 | turquoise |
| ENSBTAG00000007935 | CALCOCO2 | 1 | turquoise |
| ENSBTAG00000007942 | TENT4B | 1 | turquoise |
| ENSBTAG00000007956 | FRAT1 | 1 | turquoise |
| ENSBTAG00000007960 | TOP1 | 1 | turquoise |
| ENSBTAG00000007970 | C1H3orf38 | 1 | turquoise |
| ENSBTAG00000007974 | WTAP | 1 | turquoise |
| ENSBTAG00000007981 | GRK5 | 1 | turquoise |
| ENSBTAG00000007994 | OSCP1 | 1 | turquoise |
| ENSBTAG00000008008 | TLR2 | 1 | turquoise |
| ENSBTAG00000008010 | FLCN | 1 | turquoise |
| ENSBTAG00000008021 | LOC112441507 | 1 | turquoise |
| ENSBTAG00000008040 | SPART | 1 | turquoise |
| ENSBTAG00000008048 | GCFC2 | 1 | turquoise |
| ENSBTAG00000008082 | CEP350 | 1 | turquoise |
| ENSBTAG00000008092 | RAB37 | 1 | turquoise |
| ENSBTAG00000008096 | EDN1 | 1 | turquoise |
| ENSBTAG00000008099 | CCR5 | 1 | turquoise |
| ENSBTAG00000008100 | GOLGA7B | 1 | turquoise |
| ENSBTAG00000008132 | SOX13 | 1 | turquoise |
| ENSBTAG00000008133 | ALKBH1 | 1 | turquoise |
| ENSBTAG00000008140 | FAP | 1 | turquoise |
| ENSBTAG00000008142 | IFIH1 | 1 | turquoise |

| | | | |
|---|---|---|---|
| ENSBTAG00000008143 | STK17A | 1 | turquoise |
| ENSBTAG00000008165 | ITGA2B | 1 | turquoise |
| ENSBTAG00000008172 | EGLN3 | 1 | turquoise |
| ENSBTAG00000008182 | FOSB | 1 | turquoise |
| ENSBTAG00000008195 | PHKG1 | 1 | turquoise |
| ENSBTAG00000008223 | NLRC4 | 1 | turquoise |
| ENSBTAG00000008238 | S100A7 | 1 | turquoise |
| ENSBTAG00000008259 | ACOD1 | 1 | turquoise |
| ENSBTAG00000008260 | NA | 1 | turquoise |
| ENSBTAG00000008267 | CNOT11 | 1 | turquoise |
| ENSBTAG00000008269 | NA | 1 | turquoise |
| ENSBTAG00000008279 | FRMD4A | 1 | turquoise |
| ENSBTAG00000008293 | GPCPD1 | 1 | turquoise |
| ENSBTAG00000008294 | KCNJ2 | 1 | turquoise |
| ENSBTAG00000008320 | SEPTIN5 | 1 | turquoise |
| ENSBTAG00000008327 | NA | 1 | turquoise |
| ENSBTAG00000008328 | LOC526163 | 1 | turquoise |
| ENSBTAG00000008330 | RNF19B | 1 | turquoise |
| ENSBTAG00000008339 | DHX8 | 1 | turquoise |
| ENSBTAG00000008353 | CDKN1A | 1 | turquoise |
| ENSBTAG00000008388 | THOC2 | 1 | turquoise |
| ENSBTAG00000008401 | PFKFB3 | 1 | turquoise |
| ENSBTAG00000008403 | ROCK1 | 1 | turquoise |
| ENSBTAG00000008406 | TREX1 | 1 | turquoise |
| ENSBTAG00000008411 | PLEKHO2 | 1 | turquoise |
| ENSBTAG00000008428 | UPP1 | 1 | turquoise |
| ENSBTAG00000008429 | LRP4 | 1 | turquoise |
| ENSBTAG00000008432 | NUP98 | 1 | turquoise |
| ENSBTAG00000008441 | SOCS3 | 1 | turquoise |
| ENSBTAG00000008462 | FAM43A | 1 | turquoise |
| ENSBTAG00000008463 | NA | 1 | turquoise |
| ENSBTAG00000008471 | MX2 | 1 | turquoise |
| ENSBTAG00000008479 | CXCL13 | 1 | turquoise |
| ENSBTAG00000008485 | NA | 1 | turquoise |
| ENSBTAG00000008497 | RGS14 | 1 | turquoise |
| ENSBTAG00000008498 | ALYREF | 1 | turquoise |
| ENSBTAG00000008540 | SLC22A1 | 1 | turquoise |
| ENSBTAG00000008545 | ATF3 | 1 | turquoise |
| ENSBTAG00000008556 | POU2F2 | 1 | turquoise |
| ENSBTAG00000008573 | ZFP36 | 1 | turquoise |
| ENSBTAG00000008597 | SLC45A4 | 1 | turquoise |
| ENSBTAG00000008600 | TRAF3IP2 | 1 | turquoise |
| ENSBTAG00000008603 | TASL | 1 | turquoise |
| ENSBTAG00000008612 | C1R | 1 | turquoise |
| ENSBTAG00000008624 | NA | 1 | turquoise |
| ENSBTAG00000008631 | CORO1A | 1 | turquoise |
| ENSBTAG00000008636 | PDE4B | 1 | turquoise |

| | | | |
|---|---|---|---|
| ENSBTAG00000008638 | FAM110A | 1 | turquoise |
| ENSBTAG00000008648 | PRDX5 | 1 | turquoise |
| ENSBTAG00000008649 | ETV3 | 1 | turquoise |
| ENSBTAG00000008650 | CAMK1D | 1 | turquoise |
| ENSBTAG00000008665 | DIXDC1 | 1 | turquoise |
| ENSBTAG00000008682 | TLR3 | 1 | turquoise |
| ENSBTAG00000008690 | SLC6A9 | 1 | turquoise |
| ENSBTAG00000008699 | FEM1C | 1 | turquoise |
| ENSBTAG00000008703 | EIF2AK2 | 1 | turquoise |
| ENSBTAG00000008719 | PRKCD | 1 | turquoise |
| ENSBTAG00000008729 | DENND10 | 1 | turquoise |
| ENSBTAG00000008732 | ZC3H12C | 1 | turquoise |
| ENSBTAG00000008743 | ALDH2 | 1 | turquoise |
| ENSBTAG00000008755 | MARCHF7 | 1 | turquoise |
| ENSBTAG00000008761 | TRAFD1 | 1 | turquoise |
| ENSBTAG00000008783 | HTR7 | 1 | turquoise |
| ENSBTAG00000008800 | XKR8 | 1 | turquoise |
| ENSBTAG00000008805 | USP3 | 1 | turquoise |
| ENSBTAG00000008808 | CCDC17 | 1 | turquoise |
| ENSBTAG00000008809 | PTBP2 | 1 | turquoise |
| ENSBTAG00000008839 | AMER1 | 1 | turquoise |
| ENSBTAG00000008840 | HM13 | 1 | turquoise |
| ENSBTAG00000008851 | SIGLEC8 | 1 | turquoise |
| ENSBTAG00000008853 | HNRNPF | 1 | turquoise |
| ENSBTAG00000008864 | LY96 | 1 | turquoise |
| ENSBTAG00000008866 | GANC | 1 | turquoise |
| ENSBTAG00000008870 | KCNS1 | 1 | turquoise |
| ENSBTAG00000008883 | ZNF207 | 1 | turquoise |
| ENSBTAG00000008897 | BIN2 | 1 | turquoise |
| ENSBTAG00000008908 | GZF1 | 1 | turquoise |
| ENSBTAG00000008909 | PNPT1 | 1 | turquoise |
| ENSBTAG00000008915 | SF3B1 | 1 | turquoise |
| ENSBTAG00000008937 | NA | 1 | turquoise |
| ENSBTAG00000008938 | SRC | 1 | turquoise |
| ENSBTAG00000008939 | DNMBP | 1 | turquoise |
| ENSBTAG00000008945 | SDSL | 1 | turquoise |
| ENSBTAG00000008951 | ALPL | 1 | turquoise |
| ENSBTAG00000008952 | RAP1GAP | 1 | turquoise |
| ENSBTAG00000008953 | TAP1 | 1 | turquoise |
| ENSBTAG00000008954 | PSMB9 | 1 | turquoise |
| ENSBTAG00000008958 | NSMAF | 1 | turquoise |
| ENSBTAG00000008959 | NA | 1 | turquoise |
| ENSBTAG00000008967 | RAP1B | 1 | turquoise |
| ENSBTAG00000008996 | SFI1 | 1 | turquoise |
| ENSBTAG00000008998 | NA | 1 | turquoise |
| ENSBTAG00000009012 | PTX3 | 1 | turquoise |
| ENSBTAG00000009013 | NA | 1 | turquoise |

| | | | |
|---|---|---|---|
| ENSBTAG00000009019 | SH3PXD2B | 1 | turquoise |
| ENSBTAG00000009024 | KCND1 | 1 | turquoise |
| ENSBTAG00000009036 | SUPT6H | 1 | turquoise |
| ENSBTAG00000009048 | EFHD2 | 1 | turquoise |
| ENSBTAG00000009055 | RNF144B | 1 | turquoise |
| ENSBTAG00000009064 | CSF2RB | 1 | turquoise |
| ENSBTAG00000009065 | MAP3K5 | 1 | turquoise |
| ENSBTAG00000009085 | SLC35A5 | 1 | turquoise |
| ENSBTAG00000009086 | LOXL1 | 1 | turquoise |
| ENSBTAG00000009091 | RNASEL | 1 | turquoise |
| ENSBTAG00000009123 | ING5 | 1 | turquoise |
| ENSBTAG00000009139 | LDLRAD4 | 1 | turquoise |
| ENSBTAG00000009159 | PLXNA1 | 1 | turquoise |
| ENSBTAG00000009171 | LOC527385 | 1 | turquoise |
| ENSBTAG00000009177 | PLEKHA4 | 1 | turquoise |
| ENSBTAG00000009183 | SHISA5 | 1 | turquoise |
| ENSBTAG00000009201 | CORO7 | 1 | turquoise |
| ENSBTAG00000009206 | FOXS1 | 1 | turquoise |
| ENSBTAG00000009207 | KMT2E | 1 | turquoise |
| ENSBTAG00000009210 | ZBTB44 | 1 | turquoise |
| ENSBTAG00000009211 | UBE2S | 1 | turquoise |
| ENSBTAG00000009212 | APOA2 | 1 | turquoise |
| ENSBTAG00000009214 | ETS2 | 1 | turquoise |
| ENSBTAG00000009215 | NR1I3 | 1 | turquoise |
| ENSBTAG00000009223 | SFN | 1 | turquoise |
| ENSBTAG00000009226 | SCUBE3 | 1 | turquoise |
| ENSBTAG00000009232 | PIK3CA | 1 | turquoise |
| ENSBTAG00000009233 | NA | 1 | turquoise |
| ENSBTAG00000009236 | DAGLB | 1 | turquoise |
| ENSBTAG00000009255 | NA | 1 | turquoise |
| ENSBTAG00000009289 | MS4A8 | 1 | turquoise |
| ENSBTAG00000009340 | KATNAL1 | 1 | turquoise |
| ENSBTAG00000009341 | CCDC146 | 1 | turquoise |
| ENSBTAG00000009353 | EVI2B | 1 | turquoise |
| ENSBTAG00000009354 | EVI2A | 1 | turquoise |
| ENSBTAG00000009359 | LOC782705 | 1 | turquoise |
| ENSBTAG00000009362 | SYNE1 | 1 | turquoise |
| ENSBTAG00000009381 | LCP2 | 1 | turquoise |
| ENSBTAG00000009384 | AGTRAP | 1 | turquoise |
| ENSBTAG00000009389 | HNRNPH1 | 1 | turquoise |
| ENSBTAG00000009394 | NSMCE2 | 1 | turquoise |
| ENSBTAG00000009426 | EZH2 | 1 | turquoise |
| ENSBTAG00000009427 | PPM1D | 1 | turquoise |
| ENSBTAG00000009434 | PAQR6 | 1 | turquoise |
| ENSBTAG00000009436 | TMEM79 | 1 | turquoise |
| ENSBTAG00000009470 | CLIC4 | 1 | turquoise |
| ENSBTAG00000009482 | AFF4 | 1 | turquoise |

| | | | |
|---|---|---|---|
| ENSBTAG00000009488 | NXF1 | 1 | turquoise |
| ENSBTAG00000009493 | BCL3 | 1 | turquoise |
| ENSBTAG00000009496 | STAT5A | 1 | turquoise |
| ENSBTAG00000009502 | LUZP1 | 1 | turquoise |
| ENSBTAG00000009513 | TGFBI | 1 | turquoise |
| ENSBTAG00000009522 | EIF4E | 1 | turquoise |
| ENSBTAG00000009552 | ATP2B1 | 1 | turquoise |
| ENSBTAG00000009554 | EHBP1L1 | 1 | turquoise |
| ENSBTAG00000009578 | PTK2 | 1 | turquoise |
| ENSBTAG00000009598 | CHM | 1 | turquoise |
| ENSBTAG00000009611 | PHF21A | 1 | turquoise |
| ENSBTAG00000009614 | NRROS | 1 | turquoise |
| ENSBTAG00000009615 | ANXA2 | 1 | turquoise |
| ENSBTAG00000009617 | SLC2A1 | 1 | turquoise |
| ENSBTAG00000009656 | BOLA-DQA2 | 1 | turquoise |
| ENSBTAG00000009658 | PLEK | 1 | turquoise |
| ENSBTAG00000009664 | NA | 1 | turquoise |
| ENSBTAG00000009677 | PARP10 | 1 | turquoise |
| ENSBTAG00000009681 | PPP2R3C | 1 | turquoise |
| ENSBTAG00000009691 | SH2B2 | 1 | turquoise |
| ENSBTAG00000009694 | PUS10 | 1 | turquoise |
| ENSBTAG00000009696 | ACTN2 | 1 | turquoise |
| ENSBTAG00000009717 | FGL2 | 1 | turquoise |
| ENSBTAG00000009749 | USP2 | 1 | turquoise |
| ENSBTAG00000009760 | LOC515736 | 1 | turquoise |
| ENSBTAG00000009768 | IFIT3 | 1 | turquoise |
| ENSBTAG00000009770 | GPD2 | 1 | turquoise |
| ENSBTAG00000009773 | KREMEN1 | 1 | turquoise |
| ENSBTAG00000009778 | KRAS | 1 | turquoise |
| ENSBTAG00000009785 | SAMD4B | 1 | turquoise |
| ENSBTAG00000009845 | ITPK1 | 1 | turquoise |
| ENSBTAG00000009848 | GPR171 | 1 | turquoise |
| ENSBTAG00000009849 | PLAC8A | 1 | turquoise |
| ENSBTAG00000009863 | BHLHE40 | 1 | turquoise |
| ENSBTAG00000009868 | VGF | 1 | turquoise |
| ENSBTAG00000009887 | DDX17 | 1 | turquoise |
| ENSBTAG00000009888 | DRAM2 | 1 | turquoise |
| ENSBTAG00000009912 | PAN3 | 1 | turquoise |
| ENSBTAG00000009917 | PIP5K1B | 1 | turquoise |
| ENSBTAG00000009924 | MR1 | 1 | turquoise |
| ENSBTAG00000009933 | DTX3L | 1 | turquoise |
| ENSBTAG00000009942 | PLCL2 | 1 | turquoise |
| ENSBTAG00000009945 | BRICD5 | 1 | turquoise |
| ENSBTAG00000009948 | TRIM25 | 1 | turquoise |
| ENSBTAG00000009951 | PGP | 1 | turquoise |
| ENSBTAG00000009958 | TRIM35 | 1 | turquoise |
| ENSBTAG00000009959 | SLC6A8 | 1 | turquoise |

| | | | |
|---|---|---|---|
| ENSBTAG00000009960 | FLOT1 | 1 | turquoise |
| ENSBTAG00000009982 | IGSF8 | 1 | turquoise |
| ENSBTAG00000010002 | IRF2 | 1 | turquoise |
| ENSBTAG00000010007 | MAPK13 | 1 | turquoise |
| ENSBTAG00000010018 | ATP7A | 1 | turquoise |
| ENSBTAG00000010023 | FAM107B | 1 | turquoise |
| ENSBTAG00000010033 | NA | 1 | turquoise |
| ENSBTAG00000010042 | RIOK3 | 1 | turquoise |
| ENSBTAG00000010047 | TIAM2 | 1 | turquoise |
| ENSBTAG00000010063 | KDM3B | 1 | turquoise |
| ENSBTAG00000010065 | TRPC5 | 1 | turquoise |
| ENSBTAG00000010069 | EGR1 | 1 | turquoise |
| ENSBTAG00000010073 | FLOT2 | 1 | turquoise |
| ENSBTAG00000010125 | STAT5B | 1 | turquoise |
| ENSBTAG00000010134 | OSTM1 | 1 | turquoise |
| ENSBTAG00000010148 | LOC505856 | 1 | turquoise |
| ENSBTAG00000010153 | ANXA3 | 1 | turquoise |
| ENSBTAG00000010155 | LOC504773 | 1 | turquoise |
| ENSBTAG00000010156 | TPT1 | 1 | turquoise |
| ENSBTAG00000010166 | MIC2 | 1 | turquoise |
| ENSBTAG00000010171 | NA | 1 | turquoise |
| ENSBTAG00000010191 | PAK1 | 1 | turquoise |
| ENSBTAG00000010206 | UAP1 | 1 | turquoise |
| ENSBTAG00000010235 | NOP9 | 1 | turquoise |
| ENSBTAG00000010236 | CIDEB | 1 | turquoise |
| ENSBTAG00000010238 | LTB4R | 1 | turquoise |
| ENSBTAG00000010242 | LAMP1 | 1 | turquoise |
| ENSBTAG00000010244 | CLIC5 | 1 | turquoise |
| ENSBTAG00000010246 | GRTP1 | 1 | turquoise |
| ENSBTAG00000010273 | EREG | 1 | turquoise |
| ENSBTAG00000010285 | MMRN1 | 1 | turquoise |
| ENSBTAG00000010303 | ICAM1 | 1 | turquoise |
| ENSBTAG00000010304 | CHKA | 1 | turquoise |
| ENSBTAG00000010309 | XPO6 | 1 | turquoise |
| ENSBTAG00000010314 | ICAM4 | 1 | turquoise |
| ENSBTAG00000010316 | ICAM5 | 1 | turquoise |
| ENSBTAG00000010322 | HYOU1 | 1 | turquoise |
| ENSBTAG00000010324 | GTPBP1 | 1 | turquoise |
| ENSBTAG00000010328 | KCNK17 | 1 | turquoise |
| ENSBTAG00000010334 | SYTL3 | 1 | turquoise |
| ENSBTAG00000010347 | EZR | 1 | turquoise |
| ENSBTAG00000010349 | IL1A | 1 | turquoise |
| ENSBTAG00000010350 | USP53 | 1 | turquoise |
| ENSBTAG00000010355 | VPS37A | 1 | turquoise |
| ENSBTAG00000010356 | RAB12 | 1 | turquoise |
| ENSBTAG00000010365 | SQOR | 1 | turquoise |
| ENSBTAG00000010372 | MTMR7 | 1 | turquoise |

| | | | |
|---|---|---|---|
| ENSBTAG00000010390 | SRI | 1 | turquoise |
| ENSBTAG00000010394 | MCF2L2 | 1 | turquoise |
| ENSBTAG00000010399 | TXNDC16 | 1 | turquoise |
| ENSBTAG00000010408 | IKBKE | 1 | turquoise |
| ENSBTAG00000010411 | ADAMTS8 | 1 | turquoise |
| ENSBTAG00000010420 | PCDH17 | 1 | turquoise |
| ENSBTAG00000010422 | MDM2 | 1 | turquoise |
| ENSBTAG00000010427 | RASSF5 | 1 | turquoise |
| ENSBTAG00000010428 | USP15 | 1 | turquoise |
| ENSBTAG00000010433 | NA | 1 | turquoise |
| ENSBTAG00000010447 | LSP1 | 1 | turquoise |
| ENSBTAG00000010449 | MOCS1 | 1 | turquoise |
| ENSBTAG00000010464 | MN1 | 1 | turquoise |
| ENSBTAG00000010472 | TNFAIP8L3 | 1 | turquoise |
| ENSBTAG00000010487 | SMIM3 | 1 | turquoise |
| ENSBTAG00000010492 | VPS4B | 1 | turquoise |
| ENSBTAG00000010493 | SERTAD1 | 1 | turquoise |
| ENSBTAG00000010497 | TRAF2 | 1 | turquoise |
| ENSBTAG00000010511 | KAT8 | 1 | turquoise |
| ENSBTAG00000010520 | NA | 1 | turquoise |
| ENSBTAG00000010547 | CEP76 | 1 | turquoise |
| ENSBTAG00000010549 | IFRD1 | 1 | turquoise |
| ENSBTAG00000010555 | LSMEM1 | 1 | turquoise |
| ENSBTAG00000010563 | PTPN2 | 1 | turquoise |
| ENSBTAG00000010576 | MAP2K3 | 1 | turquoise |
| ENSBTAG00000010582 | BRD3 | 1 | turquoise |
| ENSBTAG00000010587 | SH3BGRL | 1 | turquoise |
| ENSBTAG00000010590 | FAM118A | 1 | turquoise |
| ENSBTAG00000010606 | PPP1R3B | 1 | turquoise |
| ENSBTAG00000010644 | CHST11 | 1 | turquoise |
| ENSBTAG00000010645 | BOLA-DRA | 1 | turquoise |
| ENSBTAG00000010649 | MEF2A | 1 | turquoise |
| ENSBTAG00000010662 | LIMK2 | 1 | turquoise |
| ENSBTAG00000010663 | ADAM15 | 1 | turquoise |
| ENSBTAG00000010664 | PRKCQ | 1 | turquoise |
| ENSBTAG00000010665 | CBLN3 | 1 | turquoise |
| ENSBTAG00000010666 | KHNYN | 1 | turquoise |
| ENSBTAG00000010670 | VSIG8 | 1 | turquoise |
| ENSBTAG00000010681 | NR1H3 | 1 | turquoise |
| ENSBTAG00000010722 | NA | 1 | turquoise |
| ENSBTAG00000010731 | CDKN2D | 1 | turquoise |
| ENSBTAG00000010738 | CCL14 | 1 | turquoise |
| ENSBTAG00000010763 | DUSP16 | 1 | turquoise |
| ENSBTAG00000010766 | OIP5 | 1 | turquoise |
| ENSBTAG00000010774 | NUSAP1 | 1 | turquoise |
| ENSBTAG00000010777 | CARS1 | 1 | turquoise |
| ENSBTAG00000010784 | ABT1 | 1 | turquoise |

| | | | |
|---|---|---|---|
| ENSBTAG00000010785 | FAS | 1 | turquoise |
| ENSBTAG00000010788 | PPM1E | 1 | turquoise |
| ENSBTAG00000010792 | SEH1L | 1 | turquoise |
| ENSBTAG00000010813 | NA | 1 | turquoise |
| ENSBTAG00000010818 | AEBP2 | 1 | turquoise |
| ENSBTAG00000010852 | LOC107131749 | 1 | turquoise |
| ENSBTAG00000010859 | HYLS1 | 1 | turquoise |
| ENSBTAG00000010871 | NA | 1 | turquoise |
| ENSBTAG00000010874 | GHSR | 1 | turquoise |
| ENSBTAG00000010877 | ARMC12 | 1 | turquoise |
| ENSBTAG00000010888 | VSIR | 1 | turquoise |
| ENSBTAG00000010909 | SLC30A1 | 1 | turquoise |
| ENSBTAG00000010913 | SRXN1 | 1 | turquoise |
| ENSBTAG00000010928 | CSRNP2 | 1 | turquoise |
| ENSBTAG00000010935 | EML4 | 1 | turquoise |
| ENSBTAG00000010945 | SEC24B | 1 | turquoise |
| ENSBTAG00000010954 | ART3 | 1 | turquoise |
| ENSBTAG00000010963 | IL16 | 1 | turquoise |
| ENSBTAG00000010982 | UBE2B | 1 | turquoise |
| ENSBTAG00000010987 | NFKBIZ | 1 | turquoise |
| ENSBTAG00000010992 | CTSH | 1 | turquoise |
| ENSBTAG00000010993 | ZNF839 | 1 | turquoise |
| ENSBTAG00000010998 | CFLAR | 1 | turquoise |
| ENSBTAG00000011001 | ERG | 1 | turquoise |
| ENSBTAG00000011007 | TTYH2 | 1 | turquoise |
| ENSBTAG00000011011 | SSH2 | 1 | turquoise |
| ENSBTAG00000011014 | CHST3 | 1 | turquoise |
| ENSBTAG00000011042 | TMEM129 | 1 | turquoise |
| ENSBTAG00000011043 | RAC2 | 1 | turquoise |
| ENSBTAG00000011044 | TACC3 | 1 | turquoise |
| ENSBTAG00000011075 | PLPBP | 1 | turquoise |
| ENSBTAG00000011088 | SLC6A6 | 1 | turquoise |
| ENSBTAG00000011095 | FAR2 | 1 | turquoise |
| ENSBTAG00000011100 | CTSC | 1 | turquoise |
| ENSBTAG00000011103 | SLC8B1 | 1 | turquoise |
| ENSBTAG00000011105 | SLC38A2 | 1 | turquoise |
| ENSBTAG00000011126 | STK38 | 1 | turquoise |
| ENSBTAG00000011131 | NMUR2 | 1 | turquoise |
| ENSBTAG00000011143 | KPNA1 | 1 | turquoise |
| ENSBTAG00000011146 | RAB8B | 1 | turquoise |
| ENSBTAG00000011147 | RNF13 | 1 | turquoise |
| ENSBTAG00000011184 | FTH1 | 1 | turquoise |
| ENSBTAG00000011192 | GPR87 | 1 | turquoise |
| ENSBTAG00000011197 | IPMK | 1 | turquoise |
| ENSBTAG00000011202 | CARD19 | 1 | turquoise |
| ENSBTAG00000011204 | NINJ1 | 1 | turquoise |
| ENSBTAG00000011206 | GALNT1 | 1 | turquoise |

| | | | |
|---|---|---|---|
| ENSBTAG00000011215 | ACTN4 | 1 | turquoise |
| ENSBTAG00000011226 | SLC4A2 | 1 | turquoise |
| ENSBTAG00000011227 | TRPC6 | 1 | turquoise |
| ENSBTAG00000011236 | STX11 | 1 | turquoise |
| ENSBTAG00000011238 | CD48 | 1 | turquoise |
| ENSBTAG00000011257 | ASAH1 | 1 | turquoise |
| ENSBTAG00000011258 | ACOT9 | 1 | turquoise |
| ENSBTAG00000011263 | EIF6 | 1 | turquoise |
| ENSBTAG00000011268 | TMEM64 | 1 | turquoise |
| ENSBTAG00000011283 | RBPJL | 1 | turquoise |
| ENSBTAG00000011287 | ATXN7 | 1 | turquoise |
| ENSBTAG00000011304 | XRN2 | 1 | turquoise |
| ENSBTAG00000011316 | ZC3H12A | 1 | turquoise |
| ENSBTAG00000011321 | NUGGC | 1 | turquoise |
| ENSBTAG00000011325 | KHK | 1 | turquoise |
| ENSBTAG00000011337 | ANKRD33B | 1 | turquoise |
| ENSBTAG00000011343 | XAF1 | 1 | turquoise |
| ENSBTAG00000011345 | SFMBT1 | 1 | turquoise |
| ENSBTAG00000011358 | IER3 | 1 | turquoise |
| ENSBTAG00000011373 | MAML3 | 1 | turquoise |
| ENSBTAG00000011382 | SLC9A3R1 | 1 | turquoise |
| ENSBTAG00000011398 | PRR7 | 1 | turquoise |
| ENSBTAG00000011406 | TESK1 | 1 | turquoise |
| ENSBTAG00000011409 | CD72 | 1 | turquoise |
| ENSBTAG00000011415 | ETF1 | 1 | turquoise |
| ENSBTAG00000011417 | NA | 1 | turquoise |
| ENSBTAG00000011420 | CA9 | 1 | turquoise |
| ENSBTAG00000011421 | CD37 | 1 | turquoise |
| ENSBTAG00000011435 | NPEPPS | 1 | turquoise |
| ENSBTAG00000011437 | SIK1 | 1 | turquoise |
| ENSBTAG00000011446 | SEMA7A | 1 | turquoise |
| ENSBTAG00000011463 | MID1IP1 | 1 | turquoise |
| ENSBTAG00000011465 | MYBPH | 1 | turquoise |
| ENSBTAG00000011467 | BATF2 | 1 | turquoise |
| ENSBTAG00000011479 | INPP5K | 1 | turquoise |
| ENSBTAG00000011483 | SCARF1 | 1 | turquoise |
| ENSBTAG00000011508 | H3-3D | 1 | turquoise |
| ENSBTAG00000011511 | IFI16 | 1 | turquoise |
| ENSBTAG00000011515 | SELL | 1 | turquoise |
| ENSBTAG00000011524 | XRN1 | 1 | turquoise |
| ENSBTAG00000011545 | GDA | 1 | turquoise |
| ENSBTAG00000011548 | AMPD1 | 1 | turquoise |
| ENSBTAG00000011563 | TNFSF13B | 1 | turquoise |
| ENSBTAG00000011578 | CD44 | 1 | turquoise |
| ENSBTAG00000011582 | NA | 1 | turquoise |
| ENSBTAG00000011590 | GLIPR1 | 1 | turquoise |
| ENSBTAG00000011591 | KRR1 | 1 | turquoise |

| | | | |
|---|---|---|---|
| ENSBTAG00000011593 | QKI | 1 | turquoise |
| ENSBTAG00000011596 | SRSF5 | 1 | turquoise |
| ENSBTAG00000011600 | MAP3K8 | 1 | turquoise |
| ENSBTAG00000011608 | KIAA1191 | 1 | turquoise |
| ENSBTAG00000011614 | PDE7A | 1 | turquoise |
| ENSBTAG00000011623 | VMP1 | 1 | turquoise |
| ENSBTAG00000011638 | NA | 1 | turquoise |
| ENSBTAG00000011667 | PM20D2 | 1 | turquoise |
| ENSBTAG00000011671 | NA | 1 | turquoise |
| ENSBTAG00000011672 | GABRR2 | 1 | turquoise |
| ENSBTAG00000011677 | NA | 1 | turquoise |
| ENSBTAG00000011683 | NUMB | 1 | turquoise |
| ENSBTAG00000011684 | RAB11FIP1 | 1 | turquoise |
| ENSBTAG00000011689 | LENG8 | 1 | turquoise |
| ENSBTAG00000011698 | TYK2 | 1 | turquoise |
| ENSBTAG00000011700 | PLLP | 1 | turquoise |
| ENSBTAG00000011721 | MED13 | 1 | turquoise |
| ENSBTAG00000011729 | STARD3 | 1 | turquoise |
| ENSBTAG00000011742 | NA | 1 | turquoise |
| ENSBTAG00000011757 | PSEN1 | 1 | turquoise |
| ENSBTAG00000011765 | GABARAPL1 | 1 | turquoise |
| ENSBTAG00000011766 | C7 | 1 | turquoise |
| ENSBTAG00000011771 | FICD | 1 | turquoise |
| ENSBTAG00000011784 | FGR | 1 | turquoise |
| ENSBTAG00000011798 | STK38L | 1 | turquoise |
| ENSBTAG00000011815 | SMG9 | 1 | turquoise |
| ENSBTAG00000011825 | TRIR | 1 | turquoise |
| ENSBTAG00000011828 | ARID3B | 1 | turquoise |
| ENSBTAG00000011829 | NF1 | 1 | turquoise |
| ENSBTAG00000011831 | SPPL2A | 1 | turquoise |
| ENSBTAG00000011851 | FYN | 1 | turquoise |
| ENSBTAG00000011861 | CFAP161 | 1 | turquoise |
| ENSBTAG00000011876 | MORC3 | 1 | turquoise |
| ENSBTAG00000011881 | TDRD10 | 1 | turquoise |
| ENSBTAG00000011882 | SLC24A3 | 1 | turquoise |
| ENSBTAG00000011894 | C22H3orf62 | 1 | turquoise |
| ENSBTAG00000011898 | LTO1 | 1 | turquoise |
| ENSBTAG00000011936 | ATP8B4 | 1 | turquoise |
| ENSBTAG00000011957 | CREBL2 | 1 | turquoise |
| ENSBTAG00000011970 | FNIP2 | 1 | turquoise |
| ENSBTAG00000011975 | SERPINB1 | 1 | turquoise |
| ENSBTAG00000011981 | MYBL1 | 1 | turquoise |
| ENSBTAG00000011982 | BATF3 | 1 | turquoise |
| ENSBTAG00000011986 | PLSCR4 | 1 | turquoise |
| ENSBTAG00000011994 | NDEL1 | 1 | turquoise |
| ENSBTAG00000011997 | ZMIZ2 | 1 | turquoise |
| ENSBTAG00000012002 | BCAR1 | 1 | turquoise |

| | | | |
|---|---|---|---|
| ENSBTAG00000012010 | PANX1 | 1 | turquoise |
| ENSBTAG00000012019 | NA | 1 | turquoise |
| ENSBTAG00000012022 | TBC1D2B | 1 | turquoise |
| ENSBTAG00000012038 | NA | 1 | turquoise |
| ENSBTAG00000012039 | PHACTR1 | 1 | turquoise |
| ENSBTAG00000012043 | PADI3 | 1 | turquoise |
| ENSBTAG00000012046 | JUNB | 1 | turquoise |
| ENSBTAG00000012047 | JAK2 | 1 | turquoise |
| ENSBTAG00000012050 | ORAI2 | 1 | turquoise |
| ENSBTAG00000012052 | PADI4 | 1 | turquoise |
| ENSBTAG00000012057 | CBLIF | 1 | turquoise |
| ENSBTAG00000012060 | LPIN2 | 1 | turquoise |
| ENSBTAG00000012064 | ZNF473 | 1 | turquoise |
| ENSBTAG00000012066 | PECAM1 | 1 | turquoise |
| ENSBTAG00000012067 | EPG5 | 1 | turquoise |
| ENSBTAG00000012090 | GGTA1 | 1 | turquoise |
| ENSBTAG00000012120 | TIPARP | 1 | turquoise |
| ENSBTAG00000012124 | GPBP1 | 1 | turquoise |
| ENSBTAG00000012125 | GDI1 | 1 | turquoise |
| ENSBTAG00000012156 | NAIF1 | 1 | turquoise |
| ENSBTAG00000012172 | MIDN | 1 | turquoise |
| ENSBTAG00000012180 | BMF | 1 | turquoise |
| ENSBTAG00000012182 | DIRAS3 | 1 | turquoise |
| ENSBTAG00000012185 | CLEC4E | 1 | turquoise |
| ENSBTAG00000012191 | WWC2 | 1 | turquoise |
| ENSBTAG00000012201 | NA | 1 | turquoise |
| ENSBTAG00000012208 | BOLA | 1 | turquoise |
| ENSBTAG00000012216 | MLKL | 1 | turquoise |
| ENSBTAG00000012223 | TNFSF14 | 1 | turquoise |
| ENSBTAG00000012228 | SEMA4A | 1 | turquoise |
| ENSBTAG00000012252 | MOCOS | 1 | turquoise |
| ENSBTAG00000012266 | NA | 1 | turquoise |
| ENSBTAG00000012267 | ZFC3H1 | 1 | turquoise |
| ENSBTAG00000012271 | NA | 1 | turquoise |
| ENSBTAG00000012273 | FAM131A | 1 | turquoise |
| ENSBTAG00000012277 | SRRD | 1 | turquoise |
| ENSBTAG00000012288 | TFIP11 | 1 | turquoise |
| ENSBTAG00000012291 | TBC1D22A | 1 | turquoise |
| ENSBTAG00000012297 | RIPOR3 | 1 | turquoise |
| ENSBTAG00000012314 | LDLR | 1 | turquoise |
| ENSBTAG00000012317 | PNP | 1 | turquoise |
| ENSBTAG00000012321 | ZFAND6 | 1 | turquoise |
| ENSBTAG00000012322 | INKA1 | 1 | turquoise |
| ENSBTAG00000012330 | B2M | 1 | turquoise |
| ENSBTAG00000012335 | UBA7 | 1 | turquoise |
| ENSBTAG00000012343 | TSPAN5 | 1 | turquoise |
| ENSBTAG00000012357 | VPS13B | 1 | turquoise |

| | | | |
|---|---|---|---|
| ENSBTAG00000012361 | ARHGEF11 | 1 | turquoise |
| ENSBTAG00000012371 | CPD | 1 | turquoise |
| ENSBTAG00000012383 | CHMP5 | 1 | turquoise |
| ENSBTAG00000012387 | PAM | 1 | turquoise |
| ENSBTAG00000012397 | DCK | 1 | turquoise |
| ENSBTAG00000012406 | ZBP1 | 1 | turquoise |
| ENSBTAG00000012408 | RAB27A | 1 | turquoise |
| ENSBTAG00000012417 | ARL14EP | 1 | turquoise |
| ENSBTAG00000012419 | BCL2L15 | 1 | turquoise |
| ENSBTAG00000012441 | SMAD7 | 1 | turquoise |
| ENSBTAG00000012451 | BOLA-DMB | 1 | turquoise |
| ENSBTAG00000012456 | HEPACAM2 | 1 | turquoise |
| ENSBTAG00000012500 | RARA | 1 | turquoise |
| ENSBTAG00000012519 | XDH | 1 | turquoise |
| ENSBTAG00000012526 | APBB1IP | 1 | turquoise |
| ENSBTAG00000012529 | IFNG | 1 | turquoise |
| ENSBTAG00000012537 | CASP6 | 1 | turquoise |
| ENSBTAG00000012544 | IFNGR1 | 1 | turquoise |
| ENSBTAG00000012552 | FMR1 | 1 | turquoise |
| ENSBTAG00000012575 | MAEA | 1 | turquoise |
| ENSBTAG00000012595 | RIDA | 1 | turquoise |
| ENSBTAG00000012599 | SMAD3 | 1 | turquoise |
| ENSBTAG00000012615 | NA | 1 | turquoise |
| ENSBTAG00000012619 | CNPPD1 | 1 | turquoise |
| ENSBTAG00000012622 | TRA2A | 1 | turquoise |
| ENSBTAG00000012626 | B4GALT5 | 1 | turquoise |
| ENSBTAG00000012628 | SLC1A2 | 1 | turquoise |
| ENSBTAG00000012638 | S100A12 | 1 | turquoise |
| ENSBTAG00000012640 | S100A8 | 1 | turquoise |
| ENSBTAG00000012659 | LRIF1 | 1 | turquoise |
| ENSBTAG00000012668 | NA | 1 | turquoise |
| ENSBTAG00000012671 | TNIP1 | 1 | turquoise |
| ENSBTAG00000012683 | SRRM2 | 1 | turquoise |
| ENSBTAG00000012720 | ANKRD2 | 1 | turquoise |
| ENSBTAG00000012737 | GPR65 | 1 | turquoise |
| ENSBTAG00000012741 | CCPG1 | 1 | turquoise |
| ENSBTAG00000012778 | CAPN2 | 1 | turquoise |
| ENSBTAG00000012779 | CYRIA | 1 | turquoise |
| ENSBTAG00000012787 | SDE2 | 1 | turquoise |
| ENSBTAG00000012805 | TSPAN13 | 1 | turquoise |
| ENSBTAG00000012808 | MASP2 | 1 | turquoise |
| ENSBTAG00000012815 | CLEC12A | 1 | turquoise |
| ENSBTAG00000012820 | STX3 | 1 | turquoise |
| ENSBTAG00000012822 | MGC137036 | 1 | turquoise |
| ENSBTAG00000012847 | NIBAN2 | 1 | turquoise |
| ENSBTAG00000012887 | FCER1A | 1 | turquoise |
| ENSBTAG00000012894 | NA | 1 | turquoise |

| | | | |
|---|---|---|---|
| ENSBTAG00000012899 | IFNGR2 | 1 | turquoise |
| ENSBTAG00000012900 | TMEM229B | 1 | turquoise |
| ENSBTAG00000012904 | EDEM1 | 1 | turquoise |
| ENSBTAG00000012916 | NA | 1 | turquoise |
| ENSBTAG00000012926 | TM6SF1 | 1 | turquoise |
| ENSBTAG00000012928 | PPP4C | 1 | turquoise |
| ENSBTAG00000012938 | JARID2 | 1 | turquoise |
| ENSBTAG00000012943 | PPARGC1B | 1 | turquoise |
| ENSBTAG00000012962 | SARS1 | 1 | turquoise |
| ENSBTAG00000012976 | WLS | 1 | turquoise |
| ENSBTAG00000012989 | UBE2L6 | 1 | turquoise |
| ENSBTAG00000012997 | SMPDL3B | 1 | turquoise |
| ENSBTAG00000013017 | GNAT2 | 1 | turquoise |
| ENSBTAG00000013023 | MAP4K4 | 1 | turquoise |
| ENSBTAG00000013044 | GAB3 | 1 | turquoise |
| ENSBTAG00000013064 | RGS18 | 1 | turquoise |
| ENSBTAG00000013074 | PPP1R15B | 1 | turquoise |
| ENSBTAG00000013093 | ALDH3B1 | 1 | turquoise |
| ENSBTAG00000013108 | HK2 | 1 | turquoise |
| ENSBTAG00000013125 | PLAUR | 1 | turquoise |
| ENSBTAG00000013159 | PHF11 | 1 | turquoise |
| ENSBTAG00000013165 | ENPP2 | 1 | turquoise |
| ENSBTAG00000013167 | SIGLEC1 | 1 | turquoise |
| ENSBTAG00000013184 | CEP120 | 1 | turquoise |
| ENSBTAG00000013196 | STK17B | 1 | turquoise |
| ENSBTAG00000013201 | ALOX5AP | 1 | turquoise |
| ENSBTAG00000013204 | ATP13A3 | 1 | turquoise |
| ENSBTAG00000013205 | IL1RAP | 1 | turquoise |
| ENSBTAG00000013215 | MREG | 1 | turquoise |
| ENSBTAG00000013240 | SLC3A2 | 1 | turquoise |
| ENSBTAG00000013259 | POLR3A | 1 | turquoise |
| ENSBTAG00000013270 | TTC19 | 1 | turquoise |
| ENSBTAG00000013275 | MAD2L2 | 1 | turquoise |
| ENSBTAG00000013284 | SGK3 | 1 | turquoise |
| ENSBTAG00000013290 | DYSF | 1 | turquoise |
| ENSBTAG00000013298 | PLA2G4A | 1 | turquoise |
| ENSBTAG00000013305 | NA | 1 | turquoise |
| ENSBTAG00000013316 | BRPF3 | 1 | turquoise |
| ENSBTAG00000013334 | NA | 1 | turquoise |
| ENSBTAG00000013362 | DNM2 | 1 | turquoise |
| ENSBTAG00000013364 | LIPM | 1 | turquoise |
| ENSBTAG00000013366 | NRIP3 | 1 | turquoise |
| ENSBTAG00000013368 | ANKRD22 | 1 | turquoise |
| ENSBTAG00000013371 | RUBCNL | 1 | turquoise |
| ENSBTAG00000013380 | CEP192 | 1 | turquoise |
| ENSBTAG00000013402 | NA | 1 | turquoise |
| ENSBTAG00000013410 | MARK3 | 1 | turquoise |

| | | | |
|---|---|---|---|
| ENSBTAG00000013411 | ENO1 | 1 | turquoise |
| ENSBTAG00000013412 | NFAT5 | 1 | turquoise |
| ENSBTAG00000013449 | RAB3GAP1 | 1 | turquoise |
| ENSBTAG00000013451 | RHOH | 1 | turquoise |
| ENSBTAG00000013454 | TAB2 | 1 | turquoise |
| ENSBTAG00000013465 | NXPE3 | 1 | turquoise |
| ENSBTAG00000013475 | TRAF3 | 1 | turquoise |
| ENSBTAG00000013483 | TAF1A | 1 | turquoise |
| ENSBTAG00000013501 | DHRS13 | 1 | turquoise |
| ENSBTAG00000013524 | CMIP | 1 | turquoise |
| ENSBTAG00000013536 | P2RY11 | 1 | turquoise |
| ENSBTAG00000013544 | LGALS8 | 1 | turquoise |
| ENSBTAG00000013555 | ACVR1B | 1 | turquoise |
| ENSBTAG00000013557 | ERAP1 | 1 | turquoise |
| ENSBTAG00000013569 | CD38 | 1 | turquoise |
| ENSBTAG00000013577 | SLC1A5 | 1 | turquoise |
| ENSBTAG00000013578 | CHI3L2 | 1 | turquoise |
| ENSBTAG00000013580 | KDM3A | 1 | turquoise |
| ENSBTAG00000013586 | CCR2 | 1 | turquoise |
| ENSBTAG00000013614 | TMEM38A | 1 | turquoise |
| ENSBTAG00000013631 | GLUL | 1 | turquoise |
| ENSBTAG00000013650 | SLC66A1 | 1 | turquoise |
| ENSBTAG00000013651 | DNAJB12 | 1 | turquoise |
| ENSBTAG00000013666 | SLC25A29 | 1 | turquoise |
| ENSBTAG00000013670 | SLC46A2 | 1 | turquoise |
| ENSBTAG00000013671 | UTP14A | 1 | turquoise |
| ENSBTAG00000013689 | MCTP2 | 1 | turquoise |
| ENSBTAG00000013702 | ZNF296 | 1 | turquoise |
| ENSBTAG00000013705 | NFKBIE | 1 | turquoise |
| ENSBTAG00000013706 | MEGF9 | 1 | turquoise |
| ENSBTAG00000013708 | SNX8 | 1 | turquoise |
| ENSBTAG00000013711 | NA | 1 | turquoise |
| ENSBTAG00000013730 | CD5 | 1 | turquoise |
| ENSBTAG00000013740 | SP2 | 1 | turquoise |
| ENSBTAG00000013745 | ITGA5 | 1 | turquoise |
| ENSBTAG00000013790 | MAP3K1 | 1 | turquoise |
| ENSBTAG00000013791 | FFAR3 | 1 | turquoise |
| ENSBTAG00000013806 | EFHB | 1 | turquoise |
| ENSBTAG00000013820 | NA | 1 | turquoise |
| ENSBTAG00000013821 | RIOK1 | 1 | turquoise |
| ENSBTAG00000013824 | WWC3 | 1 | turquoise |
| ENSBTAG00000013834 | NA | 1 | turquoise |
| ENSBTAG00000013843 | ACVRL1 | 1 | turquoise |
| ENSBTAG00000013857 | SH2D3A | 1 | turquoise |
| ENSBTAG00000013861 | SLC8A1 | 1 | turquoise |
| ENSBTAG00000013863 | DUSP1 | 1 | turquoise |
| ENSBTAG00000013882 | CHMP2A | 1 | turquoise |

| | | | |
|---|---|---|---|
| ENSBTAG00000013885 | ADSS2 | 1 | turquoise |
| ENSBTAG00000013895 | RELA | 1 | turquoise |
| ENSBTAG00000013900 | TRIM21 | 1 | turquoise |
| ENSBTAG00000013919 | BOLA-DRB3 | 1 | turquoise |
| ENSBTAG00000013929 | RRAD | 1 | turquoise |
| ENSBTAG00000013957 | GALNT3 | 1 | turquoise |
| ENSBTAG00000013960 | PSAT1 | 1 | turquoise |
| ENSBTAG00000013961 | MYSM1 | 1 | turquoise |
| ENSBTAG00000013973 | SERPIND1 | 1 | turquoise |
| ENSBTAG00000013981 | SPTSSA | 1 | turquoise |
| ENSBTAG00000013988 | BID | 1 | turquoise |
| ENSBTAG00000013996 | SH3BP2 | 1 | turquoise |
| ENSBTAG00000014002 | CEP95 | 1 | turquoise |
| ENSBTAG00000014023 | SIRT1 | 1 | turquoise |
| ENSBTAG00000014030 | FGD6 | 1 | turquoise |
| ENSBTAG00000014031 | TLR6 | 1 | turquoise |
| ENSBTAG00000014046 | BPI | 1 | turquoise |
| ENSBTAG00000014068 | VDAC3 | 1 | turquoise |
| ENSBTAG00000014087 | KMT5C | 1 | turquoise |
| ENSBTAG00000014090 | ARID5A | 1 | turquoise |
| ENSBTAG00000014099 | YTHDC2 | 1 | turquoise |
| ENSBTAG00000014102 | WDR77 | 1 | turquoise |
| ENSBTAG00000014105 | SRPRA | 1 | turquoise |
| ENSBTAG00000014113 | CCL8 | 1 | turquoise |
| ENSBTAG00000014122 | FOXRED1 | 1 | turquoise |
| ENSBTAG00000014123 | ZNF507 | 1 | turquoise |
| ENSBTAG00000014126 | UNKL | 1 | turquoise |
| ENSBTAG00000014127 | PTGS2 | 1 | turquoise |
| ENSBTAG00000014140 | ATP1B3 | 1 | turquoise |
| ENSBTAG00000014149 | LCN2 | 1 | turquoise |
| ENSBTAG00000014153 | PKNOX1 | 1 | turquoise |
| ENSBTAG00000014169 | PCDH19 | 1 | turquoise |
| ENSBTAG00000014172 | FGD2 | 1 | turquoise |
| ENSBTAG00000014199 | TBX21 | 1 | turquoise |
| ENSBTAG00000014211 | FOXP4 | 1 | turquoise |
| ENSBTAG00000014215 | CCDC86 | 1 | turquoise |
| ENSBTAG00000014217 | HHEX | 1 | turquoise |
| ENSBTAG00000014227 | NDFIP2 | 1 | turquoise |
| ENSBTAG00000014228 | TFEC | 1 | turquoise |
| ENSBTAG00000014237 | CYTH4 | 1 | turquoise |
| ENSBTAG00000014238 | ATP6V1E1 | 1 | turquoise |
| ENSBTAG00000014248 | MIER3 | 1 | turquoise |
| ENSBTAG00000014249 | IVNS1ABP | 1 | turquoise |
| ENSBTAG00000014259 | NA | 1 | turquoise |
| ENSBTAG00000014261 | TARS1 | 1 | turquoise |
| ENSBTAG00000014291 | WNT2B | 1 | turquoise |
| ENSBTAG00000014297 | MOV10 | 1 | turquoise |

| | | | |
|---|---|---|---|
| ENSBTAG00000014306 | DOP1B | 1 | turquoise |
| ENSBTAG00000014310 | HEATR5B | 1 | turquoise |
| ENSBTAG00000014324 | ANTXR2 | 1 | turquoise |
| ENSBTAG00000014328 | MGC138914 | 1 | turquoise |
| ENSBTAG00000014349 | RNF126 | 1 | turquoise |
| ENSBTAG00000014358 | EVA1B | 1 | turquoise |
| ENSBTAG00000014359 | DRAXIN | 1 | turquoise |
| ENSBTAG00000014365 | LOC112447333 | 1 | turquoise |
| ENSBTAG00000014367 | PRKX | 1 | turquoise |
| ENSBTAG00000014368 | SLAMF6 | 1 | turquoise |
| ENSBTAG00000014371 | CHPF2 | 1 | turquoise |
| ENSBTAG00000014376 | ANKRD13A | 1 | turquoise |
| ENSBTAG00000014389 | SP4 | 1 | turquoise |
| ENSBTAG00000014390 | MTMR9 | 1 | turquoise |
| ENSBTAG00000014393 | CLK2 | 1 | turquoise |
| ENSBTAG00000014396 | KLF10 | 1 | turquoise |
| ENSBTAG00000014400 | E2F2 | 1 | turquoise |
| ENSBTAG00000014402 | GIMAP8 | 1 | turquoise |
| ENSBTAG00000014421 | NA | 1 | turquoise |
| ENSBTAG00000014424 | DDN | 1 | turquoise |
| ENSBTAG00000014433 | UBE2G1 | 1 | turquoise |
| ENSBTAG00000014439 | KMO | 1 | turquoise |
| ENSBTAG00000014465 | SERPINE1 | 1 | turquoise |
| ENSBTAG00000014467 | GLIS3 | 1 | turquoise |
| ENSBTAG00000014471 | PALM2 | 1 | turquoise |
| ENSBTAG00000014490 | DDX39B | 1 | turquoise |
| ENSBTAG00000014496 | CCR6 | 1 | turquoise |
| ENSBTAG00000014497 | GPR31 | 1 | turquoise |
| ENSBTAG00000014506 | ZNF503 | 1 | turquoise |
| ENSBTAG00000014508 | FBXO40 | 1 | turquoise |
| ENSBTAG00000014511 | C16H1orf159 | 1 | turquoise |
| ENSBTAG00000014526 | MOB3A | 1 | turquoise |
| ENSBTAG00000014529 | GBP4 | 1 | turquoise |
| ENSBTAG00000014543 | PLXNA4 | 1 | turquoise |
| ENSBTAG00000014546 | CLEC7A | 1 | turquoise |
| ENSBTAG00000014548 | NIBAN1 | 1 | turquoise |
| ENSBTAG00000014551 | TNFAIP8 | 1 | turquoise |
| ENSBTAG00000014553 | ATP6V0D1 | 1 | turquoise |
| ENSBTAG00000014554 | SNAI1 | 1 | turquoise |
| ENSBTAG00000014555 | CASS4 | 1 | turquoise |
| ENSBTAG00000014560 | HLX | 1 | turquoise |
| ENSBTAG00000014585 | PTGIR | 1 | turquoise |
| ENSBTAG00000014593 | NA | 1 | turquoise |
| ENSBTAG00000014600 | ADCY8 | 1 | turquoise |
| ENSBTAG00000014605 | ETV6 | 1 | turquoise |
| ENSBTAG00000014612 | DOCK2 | 1 | turquoise |
| ENSBTAG00000014614 | ACTA2 | 1 | turquoise |

| | | | |
|---|---|---|---|
| ENSBTAG00000014628 | OAS2 | 1 | turquoise |
| ENSBTAG00000014642 | NAPRT | 1 | turquoise |
| ENSBTAG00000014665 | ADAMTS2 | 1 | turquoise |
| ENSBTAG00000014680 | ZBTB49 | 1 | turquoise |
| ENSBTAG00000014693 | TMEM88 | 1 | turquoise |
| ENSBTAG00000014705 | HES4 | 1 | turquoise |
| ENSBTAG00000014707 | ISG15 | 1 | turquoise |
| ENSBTAG00000014728 | TAPBPL | 1 | turquoise |
| ENSBTAG00000014737 | VEGFC | 1 | turquoise |
| ENSBTAG00000014761 | PGA5 | 1 | turquoise |
| ENSBTAG00000014762 | ISG20 | 1 | turquoise |
| ENSBTAG00000014790 | ZBTB2 | 1 | turquoise |
| ENSBTAG00000014794 | SCYL3 | 1 | turquoise |
| ENSBTAG00000014799 | ARMC7 | 1 | turquoise |
| ENSBTAG00000014814 | BICRAL | 1 | turquoise |
| ENSBTAG00000014818 | MYLK3 | 1 | turquoise |
| ENSBTAG00000014825 | NLK | 1 | turquoise |
| ENSBTAG00000014828 | CACNA1A | 1 | turquoise |
| ENSBTAG00000014829 | MYRFL | 1 | turquoise |
| ENSBTAG00000014857 | LOC107131333 | 1 | turquoise |
| ENSBTAG00000014863 | GYPC | 1 | turquoise |
| ENSBTAG00000014878 | COX7A1 | 1 | turquoise |
| ENSBTAG00000014880 | ICOSLG | 1 | turquoise |
| ENSBTAG00000014881 | PHF23 | 1 | turquoise |
| ENSBTAG00000014882 | DNMT3L | 1 | turquoise |
| ENSBTAG00000014884 | HIPK3 | 1 | turquoise |
| ENSBTAG00000014897 | UNC5A | 1 | turquoise |
| ENSBTAG00000014898 | HK3 | 1 | turquoise |
| ENSBTAG00000014903 | ABCA3 | 1 | turquoise |
| ENSBTAG00000014906 | VCAN | 1 | turquoise |
| ENSBTAG00000014917 | GTPBP4 | 1 | turquoise |
| ENSBTAG00000014921 | IL6 | 1 | turquoise |
| ENSBTAG00000014953 | LOC104968422 | 1 | turquoise |
| ENSBTAG00000014970 | LAMTOR5 | 1 | turquoise |
| ENSBTAG00000014971 | SEC61G | 1 | turquoise |
| ENSBTAG00000014974 | GRAMD2B | 1 | turquoise |
| ENSBTAG00000014981 | OTUB2 | 1 | turquoise |
| ENSBTAG00000014983 | IL23R | 1 | turquoise |
| ENSBTAG00000014991 | PARD3 | 1 | turquoise |
| ENSBTAG00000015007 | NCOA1 | 1 | turquoise |
| ENSBTAG00000015010 | TBC1D23 | 1 | turquoise |
| ENSBTAG00000015018 | FSD2 | 1 | turquoise |
| ENSBTAG00000015032 | CD14 | 1 | turquoise |
| ENSBTAG00000015034 | IK | 1 | turquoise |
| ENSBTAG00000015042 | WDR55 | 1 | turquoise |
| ENSBTAG00000015043 | MFNG | 1 | turquoise |
| ENSBTAG00000015046 | MST1R | 1 | turquoise |

| | | | |
|---|---|---|---|
| ENSBTAG00000015060 | GBP5 | 1 | turquoise |
| ENSBTAG00000015061 | LOC617141 | 1 | turquoise |
| ENSBTAG00000015083 | FBXO9 | 1 | turquoise |
| ENSBTAG00000015086 | HSD11B1 | 1 | turquoise |
| ENSBTAG00000015092 | NA | 1 | turquoise |
| ENSBTAG00000015107 | SLC16A1 | 1 | turquoise |
| ENSBTAG00000015109 | TOB2 | 1 | turquoise |
| ENSBTAG00000015114 | CALR | 1 | turquoise |
| ENSBTAG00000015119 | SIK3 | 1 | turquoise |
| ENSBTAG00000015124 | ARGLU1 | 1 | turquoise |
| ENSBTAG00000015126 | TNIP2 | 1 | turquoise |
| ENSBTAG00000015127 | SDC4 | 1 | turquoise |
| ENSBTAG00000015150 | IL12A | 1 | turquoise |
| ENSBTAG00000015154 | MCL1 | 1 | turquoise |
| ENSBTAG00000015160 | PLD4 | 1 | turquoise |
| ENSBTAG00000015183 | TPCN2 | 1 | turquoise |
| ENSBTAG00000015188 | KLF6 | 1 | turquoise |
| ENSBTAG00000015190 | RMC1 | 1 | turquoise |
| ENSBTAG00000015195 | NPC1 | 1 | turquoise |
| ENSBTAG00000015209 | MXD4 | 1 | turquoise |
| ENSBTAG00000015212 | IFNAR2 | 1 | turquoise |
| ENSBTAG00000015228 | CD74 | 1 | turquoise |
| ENSBTAG00000015241 | EPHX2 | 1 | turquoise |
| ENSBTAG00000015258 | P2RX5 | 1 | turquoise |
| ENSBTAG00000015266 | SDHAF2 | 1 | turquoise |
| ENSBTAG00000015271 | RIPK2 | 1 | turquoise |
| ENSBTAG00000015276 | SLC26A11 | 1 | turquoise |
| ENSBTAG00000015312 | LTBR | 1 | turquoise |
| ENSBTAG00000015318 | NECTIN2 | 1 | turquoise |
| ENSBTAG00000015326 | GSDME | 1 | turquoise |
| ENSBTAG00000015363 | CDC42SE1 | 1 | turquoise |
| ENSBTAG00000015375 | SH2D3C | 1 | turquoise |
| ENSBTAG00000015386 | REL | 1 | turquoise |
| ENSBTAG00000015388 | RPL18 | 1 | turquoise |
| ENSBTAG00000015405 | DCHS1 | 1 | turquoise |
| ENSBTAG00000015413 | RASA2 | 1 | turquoise |
| ENSBTAG00000015467 | FAM184A | 1 | turquoise |
| ENSBTAG00000015474 | SRSF11 | 1 | turquoise |
| ENSBTAG00000015498 | ELOVL4 | 1 | turquoise |
| ENSBTAG00000015509 | NAMPT | 1 | turquoise |
| ENSBTAG00000015520 | SLC11A1 | 1 | turquoise |
| ENSBTAG00000015541 | DLC1 | 1 | turquoise |
| ENSBTAG00000015543 | ARHGAP35 | 1 | turquoise |
| ENSBTAG00000015557 | LOC528040 | 1 | turquoise |
| ENSBTAG00000015562 | PLBD1 | 1 | turquoise |
| ENSBTAG00000015569 | ENKUR | 1 | turquoise |
| ENSBTAG00000015571 | GCLC | 1 | turquoise |

| | | | |
|---|---|---|---|
| ENSBTAG00000015576 | NPAS1 | 1 | turquoise |
| ENSBTAG00000015580 | TLE3 | 1 | turquoise |
| ENSBTAG00000015591 | SQSTM1 | 1 | turquoise |
| ENSBTAG00000015592 | GPR84 | 1 | turquoise |
| ENSBTAG00000015599 | PRKD2 | 1 | turquoise |
| ENSBTAG00000015609 | PREX1 | 1 | turquoise |
| ENSBTAG00000015632 | TNFRSF18 | 1 | turquoise |
| ENSBTAG00000015635 | TNFRSF4 | 1 | turquoise |
| ENSBTAG00000015636 | SHFL | 1 | turquoise |
| ENSBTAG00000015638 | IL20RA | 1 | turquoise |
| ENSBTAG00000015649 | TMEM156 | 1 | turquoise |
| ENSBTAG00000015662 | IMPG2 | 1 | turquoise |
| ENSBTAG00000015678 | NEMP2 | 1 | turquoise |
| ENSBTAG00000015694 | NA | 1 | turquoise |
| ENSBTAG00000015707 | TREML2 | 1 | turquoise |
| ENSBTAG00000015711 | BTG2 | 1 | turquoise |
| ENSBTAG00000015716 | ERO1A | 1 | turquoise |
| ENSBTAG00000015718 | CASP8 | 1 | turquoise |
| ENSBTAG00000015727 | IFI47 | 1 | turquoise |
| ENSBTAG00000015730 | BOLA-DMA | 1 | turquoise |
| ENSBTAG00000015742 | ICAM3 | 1 | turquoise |
| ENSBTAG00000015749 | STEAP1 | 1 | turquoise |
| ENSBTAG00000015752 | SP110 | 1 | turquoise |
| ENSBTAG00000015758 | USB1 | 1 | turquoise |
| ENSBTAG00000015772 | STOML1 | 1 | turquoise |
| ENSBTAG00000015776 | MFSD14A | 1 | turquoise |
| ENSBTAG00000015778 | SASS6 | 1 | turquoise |
| ENSBTAG00000015779 | PML | 1 | turquoise |
| ENSBTAG00000015792 | CFAP54 | 1 | turquoise |
| ENSBTAG00000015805 | SIPA1L1 | 1 | turquoise |
| ENSBTAG00000015815 | CFP | 1 | turquoise |
| ENSBTAG00000015818 | LOC112441463 | 1 | turquoise |
| ENSBTAG00000015821 | AMPD3 | 1 | turquoise |
| ENSBTAG00000015825 | CCDC65 | 1 | turquoise |
| ENSBTAG00000015828 | FKBP11 | 1 | turquoise |
| ENSBTAG00000015829 | ENPP5 | 1 | turquoise |
| ENSBTAG00000015837 | P2RY12 | 1 | turquoise |
| ENSBTAG00000015844 | TFPI2 | 1 | turquoise |
| ENSBTAG00000015865 | CAPN14 | 1 | turquoise |
| ENSBTAG00000015868 | LIG4 | 1 | turquoise |
| ENSBTAG00000015872 | PROKR2 | 1 | turquoise |
| ENSBTAG00000015874 | CASP3 | 1 | turquoise |
| ENSBTAG00000015882 | UBE2J2 | 1 | turquoise |
| ENSBTAG00000015889 | CLCN7 | 1 | turquoise |
| ENSBTAG00000015902 | PTGFR | 1 | turquoise |
| ENSBTAG00000015912 | DMKN | 1 | turquoise |
| ENSBTAG00000015919 | PARM1 | 1 | turquoise |

| | | | |
|---|---|---|---|
| ENSBTAG00000015938 | ARAP3 | 1 | turquoise |
| ENSBTAG00000015952 | ADAP1 | 1 | turquoise |
| ENSBTAG00000015961 | TSPAN2 | 1 | turquoise |
| ENSBTAG00000015968 | ACSF3 | 1 | turquoise |
| ENSBTAG00000015969 | STK40 | 1 | turquoise |
| ENSBTAG00000015974 | FLVCR1 | 1 | turquoise |
| ENSBTAG00000015976 | PHF12 | 1 | turquoise |
| ENSBTAG00000015978 | ANXA1 | 1 | turquoise |
| ENSBTAG00000015979 | SEZ6 | 1 | turquoise |
| ENSBTAG00000015986 | NDE1 | 1 | turquoise |
| ENSBTAG00000015994 | LRRC63 | 1 | turquoise |
| ENSBTAG00000016060 | CREM | 1 | turquoise |
| ENSBTAG00000016061 | RSAD2 | 1 | turquoise |
| ENSBTAG00000016073 | UGCG | 1 | turquoise |
| ENSBTAG00000016074 | ZNF143 | 1 | turquoise |
| ENSBTAG00000016081 | NA | 1 | turquoise |
| ENSBTAG00000016095 | PRICKLE3 | 1 | turquoise |
| ENSBTAG00000016116 | NA | 1 | turquoise |
| ENSBTAG00000016139 | TMEM120A | 1 | turquoise |
| ENSBTAG00000016163 | OSM | 1 | turquoise |
| ENSBTAG00000016168 | APLP2 | 1 | turquoise |
| ENSBTAG00000016169 | ID1 | 1 | turquoise |
| ENSBTAG00000016197 | PHLDB1 | 1 | turquoise |
| ENSBTAG00000016204 | C1RL | 1 | turquoise |
| ENSBTAG00000016206 | MAOA | 1 | turquoise |
| ENSBTAG00000016208 | TGM2 | 1 | turquoise |
| ENSBTAG00000016217 | RBM43 | 1 | turquoise |
| ENSBTAG00000016224 | RPS7 | 1 | turquoise |
| ENSBTAG00000016229 | KLF9 | 1 | turquoise |
| ENSBTAG00000016230 | BRWD3 | 1 | turquoise |
| ENSBTAG00000016260 | LRRK2 | 1 | turquoise |
| ENSBTAG00000016267 | SERPING1 | 1 | turquoise |
| ENSBTAG00000016269 | ME2 | 1 | turquoise |
| ENSBTAG00000016276 | HAL | 1 | turquoise |
| ENSBTAG00000016282 | EIF5 | 1 | turquoise |
| ENSBTAG00000016297 | CCNYL1 | 1 | turquoise |
| ENSBTAG00000016315 | COTL1 | 1 | turquoise |
| ENSBTAG00000016328 | SFPQ | 1 | turquoise |
| ENSBTAG00000016344 | PIK3R6 | 1 | turquoise |
| ENSBTAG00000016345 | IL2RB | 1 | turquoise |
| ENSBTAG00000016354 | IER2 | 1 | turquoise |
| ENSBTAG00000016355 | DGAT2 | 1 | turquoise |
| ENSBTAG00000016357 | VNN2 | 1 | turquoise |
| ENSBTAG00000016359 | SLCO4C1 | 1 | turquoise |
| ENSBTAG00000016367 | RBM18 | 1 | turquoise |
| ENSBTAG00000016374 | OGFRL1 | 1 | turquoise |
| ENSBTAG00000016378 | LRP10 | 1 | turquoise |

| | | | |
|---|---|---|---|
| ENSBTAG00000016381 | REM2 | 1 | turquoise |
| ENSBTAG00000016388 | SLCO4A1 | 1 | turquoise |
| ENSBTAG00000016401 | OPTN | 1 | turquoise |
| ENSBTAG00000016411 | RNF122 | 1 | turquoise |
| ENSBTAG00000016414 | VDR | 1 | turquoise |
| ENSBTAG00000016424 | MMP25 | 1 | turquoise |
| ENSBTAG00000016435 | NOM1 | 1 | turquoise |
| ENSBTAG00000016455 | CACHD1 | 1 | turquoise |
| ENSBTAG00000016462 | TCF4 | 1 | turquoise |
| ENSBTAG00000016477 | PEAK3 | 1 | turquoise |
| ENSBTAG00000016494 | RELT | 1 | turquoise |
| ENSBTAG00000016498 | PHACTR2 | 1 | turquoise |
| ENSBTAG00000016508 | CTDSP2 | 1 | turquoise |
| ENSBTAG00000016513 | ZNF331 | 1 | turquoise |
| ENSBTAG00000016519 | MBOAT1 | 1 | turquoise |
| ENSBTAG00000016526 | PIH1D1 | 1 | turquoise |
| ENSBTAG00000016532 | MAPKAPK3 | 1 | turquoise |
| ENSBTAG00000016542 | LAMB3 | 1 | turquoise |
| ENSBTAG00000016546 | PARP12 | 1 | turquoise |
| ENSBTAG00000016566 | ITGA9 | 1 | turquoise |
| ENSBTAG00000016593 | FMNL3 | 1 | turquoise |
| ENSBTAG00000016598 | DYNC1H1 | 1 | turquoise |
| ENSBTAG00000016603 | LOC786914 | 1 | turquoise |
| ENSBTAG00000016649 | CCND2 | 1 | turquoise |
| ENSBTAG00000016656 | PARP14 | 1 | turquoise |
| ENSBTAG00000016661 | USP18 | 1 | turquoise |
| ENSBTAG00000016683 | NFKBIA | 1 | turquoise |
| ENSBTAG00000016698 | SLAMF8 | 1 | turquoise |
| ENSBTAG00000016709 | NT5C3A | 1 | turquoise |
| ENSBTAG00000016711 | PPIF | 1 | turquoise |
| ENSBTAG00000016723 | GFI1 | 1 | turquoise |
| ENSBTAG00000016750 | PTBP3 | 1 | turquoise |
| ENSBTAG00000016779 | CLIP1 | 1 | turquoise |
| ENSBTAG00000016804 | LYST | 1 | turquoise |
| ENSBTAG00000016828 | TAPBP | 1 | turquoise |
| ENSBTAG00000016830 | DAXX | 1 | turquoise |
| ENSBTAG00000016841 | ATP10B | 1 | turquoise |
| ENSBTAG00000016844 | ADGB | 1 | turquoise |
| ENSBTAG00000016852 | SNIP1 | 1 | turquoise |
| ENSBTAG00000016864 | LBP | 1 | turquoise |
| ENSBTAG00000016879 | DENND11 | 1 | turquoise |
| ENSBTAG00000016896 | HERPUD1 | 1 | turquoise |
| ENSBTAG00000016906 | CYP27B1 | 1 | turquoise |
| ENSBTAG00000016908 | METTL1 | 1 | turquoise |
| ENSBTAG00000016910 | EEF1AKMT3 | 1 | turquoise |
| ENSBTAG00000016911 | ARHGAP23 | 1 | turquoise |
| ENSBTAG00000016912 | TSFM | 1 | turquoise |

| | | | |
|---|---|---|---|
| ENSBTAG00000016913 | AVIL | 1 | turquoise |
| ENSBTAG00000016915 | FLT1 | 1 | turquoise |
| ENSBTAG00000016932 | SENP7 | 1 | turquoise |
| ENSBTAG00000016933 | IL27RA | 1 | turquoise |
| ENSBTAG00000016939 | C11H2orf42 | 1 | turquoise |
| ENSBTAG00000016944 | ADORA2A | 1 | turquoise |
| ENSBTAG00000016972 | RAB11FIP4 | 1 | turquoise |
| ENSBTAG00000016981 | FBXO4 | 1 | turquoise |
| ENSBTAG00000016984 | PTPN9 | 1 | turquoise |
| ENSBTAG00000017000 | IL22RA2 | 1 | turquoise |
| ENSBTAG00000017002 | RBCK1 | 1 | turquoise |
| ENSBTAG00000017016 | H3-5 | 1 | turquoise |
| ENSBTAG00000017054 | NA | 1 | turquoise |
| ENSBTAG00000017056 | PKLR | 1 | turquoise |
| ENSBTAG00000017068 | DDA1 | 1 | turquoise |
| ENSBTAG00000017082 | POR | 1 | turquoise |
| ENSBTAG00000017091 | CMTR1 | 1 | turquoise |
| ENSBTAG00000017118 | FAM8A1 | 1 | turquoise |
| ENSBTAG00000017187 | TMEM255A | 1 | turquoise |
| ENSBTAG00000017196 | PDIA3 | 1 | turquoise |
| ENSBTAG00000017211 | SERINC4 | 1 | turquoise |
| ENSBTAG00000017212 | HYPK | 1 | turquoise |
| ENSBTAG00000017218 | ACER3 | 1 | turquoise |
| ENSBTAG00000017228 | TP53I11 | 1 | turquoise |
| ENSBTAG00000017232 | KIF13B | 1 | turquoise |
| ENSBTAG00000017233 | NA | 1 | turquoise |
| ENSBTAG00000017255 | ATAD2B | 1 | turquoise |
| ENSBTAG00000017263 | MXI1 | 1 | turquoise |
| ENSBTAG00000017280 | C3 | 1 | turquoise |
| ENSBTAG00000017287 | DRAM1 | 1 | turquoise |
| ENSBTAG00000017289 | MCF2L | 1 | turquoise |
| ENSBTAG00000017310 | EVA1C | 1 | turquoise |
| ENSBTAG00000017313 | ADA2 | 1 | turquoise |
| ENSBTAG00000017346 | RASSF2 | 1 | turquoise |
| ENSBTAG00000017363 | SAT1 | 1 | turquoise |
| ENSBTAG00000017367 | IFIT5 | 1 | turquoise |
| ENSBTAG00000017375 | FAM241A | 1 | turquoise |
| ENSBTAG00000017393 | L3MBTL3 | 1 | turquoise |
| ENSBTAG00000017402 | OSBPL2 | 1 | turquoise |
| ENSBTAG00000017425 | GJB2 | 1 | turquoise |
| ENSBTAG00000017435 | KCTD7 | 1 | turquoise |
| ENSBTAG00000017448 | EFEMP1 | 1 | turquoise |
| ENSBTAG00000017457 | STK10 | 1 | turquoise |
| ENSBTAG00000017461 | SLC16A3 | 1 | turquoise |
| ENSBTAG00000017462 | ATF4 | 1 | turquoise |
| ENSBTAG00000017463 | RPS19BP1 | 1 | turquoise |
| ENSBTAG00000017482 | ISYNA1 | 1 | turquoise |

| | | | |
|---|---|---|---|
| ENSBTAG00000017492 | PCMTD1 | 1 | turquoise |
| ENSBTAG00000017528 | SNAI3 | 1 | turquoise |
| ENSBTAG00000017542 | PPARD | 1 | turquoise |
| ENSBTAG00000017560 | CEBPG | 1 | turquoise |
| ENSBTAG00000017571 | PLEKHA5 | 1 | turquoise |
| ENSBTAG00000017580 | RFX5 | 1 | turquoise |
| ENSBTAG00000017592 | GPAT3 | 1 | turquoise |
| ENSBTAG00000017593 | TREM1 | 1 | turquoise |
| ENSBTAG00000017648 | TIFAB | 1 | turquoise |
| ENSBTAG00000017656 | ST3GAL3 | 1 | turquoise |
| ENSBTAG00000017659 | BFSP2 | 1 | turquoise |
| ENSBTAG00000017661 | RFX2 | 1 | turquoise |
| ENSBTAG00000017670 | LOC511531 | 1 | turquoise |
| ENSBTAG00000017672 | HAS1 | 1 | turquoise |
| ENSBTAG00000017674 | SCNN1D | 1 | turquoise |
| ENSBTAG00000017677 | SCG3 | 1 | turquoise |
| ENSBTAG00000017682 | TET2 | 1 | turquoise |
| ENSBTAG00000017701 | LOC618939 | 1 | turquoise |
| ENSBTAG00000017711 | NA | 1 | turquoise |
| ENSBTAG00000017713 | KTN1 | 1 | turquoise |
| ENSBTAG00000017721 | METTL13 | 1 | turquoise |
| ENSBTAG00000017722 | F5 | 1 | turquoise |
| ENSBTAG00000017731 | ZFAT | 1 | turquoise |
| ENSBTAG00000017745 | IL6ST | 1 | turquoise |
| ENSBTAG00000017763 | NA | 1 | turquoise |
| ENSBTAG00000017764 | HDAC4 | 1 | turquoise |
| ENSBTAG00000017786 | FCRL6 | 1 | turquoise |
| ENSBTAG00000017793 | KLRG2 | 1 | turquoise |
| ENSBTAG00000017805 | CRACR2B | 1 | turquoise |
| ENSBTAG00000017819 | PMVK | 1 | turquoise |
| ENSBTAG00000017824 | IRF8 | 1 | turquoise |
| ENSBTAG00000017826 | SCLY | 1 | turquoise |
| ENSBTAG00000017833 | RNF19A | 1 | turquoise |
| ENSBTAG00000017839 | NA | 1 | turquoise |
| ENSBTAG00000017840 | BAZ2A | 1 | turquoise |
| ENSBTAG00000017850 | ARHGEF6 | 1 | turquoise |
| ENSBTAG00000017855 | ITM2C | 1 | turquoise |
| ENSBTAG00000017856 | TSTD1 | 1 | turquoise |
| ENSBTAG00000017863 | SRGN | 1 | turquoise |
| ENSBTAG00000017867 | YPEL1 | 1 | turquoise |
| ENSBTAG00000017872 | ZNF304 | 1 | turquoise |
| ENSBTAG00000017895 | CLEC10A | 1 | turquoise |
| ENSBTAG00000017942 | CRTC2 | 1 | turquoise |
| ENSBTAG00000017949 | NA | 1 | turquoise |
| ENSBTAG00000017969 | CA4 | 1 | turquoise |
| ENSBTAG00000017970 | ZYX | 1 | turquoise |
| ENSBTAG00000017981 | ADRB3 | 1 | turquoise |

| | | | |
|---|---|---|---|
| ENSBTAG00000017992 | CD164 | 1 | turquoise |
| ENSBTAG00000017994 | MAX | 1 | turquoise |
| ENSBTAG00000018000 | CLN3 | 1 | turquoise |
| ENSBTAG00000018011 | NA | 1 | turquoise |
| ENSBTAG00000018013 | EMP3 | 1 | turquoise |
| ENSBTAG00000018015 | IL27 | 1 | turquoise |
| ENSBTAG00000018016 | NUPR1 | 1 | turquoise |
| ENSBTAG00000018024 | NR1D2 | 1 | turquoise |
| ENSBTAG00000018025 | IQCG | 1 | turquoise |
| ENSBTAG00000018035 | TGFBR1 | 1 | turquoise |
| ENSBTAG00000018036 | CNOT7 | 1 | turquoise |
| ENSBTAG00000018040 | PSMB10 | 1 | turquoise |
| ENSBTAG00000018046 | B3GNT2 | 1 | turquoise |
| ENSBTAG00000018050 | CDR2 | 1 | turquoise |
| ENSBTAG00000018059 | CD80 | 1 | turquoise |
| ENSBTAG00000018073 | TSPO | 1 | turquoise |
| ENSBTAG00000018086 | TBC1D10A | 1 | turquoise |
| ENSBTAG00000018108 | PXN | 1 | turquoise |
| ENSBTAG00000018112 | TBL1X | 1 | turquoise |
| ENSBTAG00000018116 | MTFP1 | 1 | turquoise |
| ENSBTAG00000018119 | AOAH | 1 | turquoise |
| ENSBTAG00000018125 | KIF5C | 1 | turquoise |
| ENSBTAG00000018134 | AREG | 1 | turquoise |
| ENSBTAG00000018137 | NA | 1 | turquoise |
| ENSBTAG00000018138 | GK5 | 1 | turquoise |
| ENSBTAG00000018146 | VAT1L | 1 | turquoise |
| ENSBTAG00000018152 | MYADM | 1 | turquoise |
| ENSBTAG00000018165 | RIPOR2 | 1 | turquoise |
| ENSBTAG00000018186 | PDXK | 1 | turquoise |
| ENSBTAG00000018198 | TLR9 | 1 | turquoise |
| ENSBTAG00000018200 | IL15 | 1 | turquoise |
| ENSBTAG00000018203 | CD101 | 1 | turquoise |
| ENSBTAG00000018223 | CHI3L1 | 1 | turquoise |
| ENSBTAG00000018227 | SLC4A7 | 1 | turquoise |
| ENSBTAG00000018229 | NFIX | 1 | turquoise |
| ENSBTAG00000018232 | STOML3 | 1 | turquoise |
| ENSBTAG00000018238 | ABTB1 | 1 | turquoise |
| ENSBTAG00000018240 | CYP2S1 | 1 | turquoise |
| ENSBTAG00000018252 | ARRDC4 | 1 | turquoise |
| ENSBTAG00000018254 | CDKN1B | 1 | turquoise |
| ENSBTAG00000018255 | ACTN1 | 1 | turquoise |
| ENSBTAG00000018263 | WAC | 1 | turquoise |
| ENSBTAG00000018280 | SLC28A3 | 1 | turquoise |
| ENSBTAG00000018285 | VAMP5 | 1 | turquoise |
| ENSBTAG00000018290 | IL9 | 1 | turquoise |
| ENSBTAG00000018299 | PIGH | 1 | turquoise |
| ENSBTAG00000018309 | NA | 1 | turquoise |

| | | | |
|---|---|---|---|
| ENSBTAG00000018314 | MED11 | 1 | turquoise |
| ENSBTAG00000018315 | PELP1 | 1 | turquoise |
| ENSBTAG00000018320 | RPLP1 | 1 | turquoise |
| ENSBTAG00000018331 | CLEC3B | 1 | turquoise |
| ENSBTAG00000018332 | PPTC7 | 1 | turquoise |
| ENSBTAG00000018334 | PLAGL2 | 1 | turquoise |
| ENSBTAG00000018366 | SLC15A3 | 1 | turquoise |
| ENSBTAG00000018381 | CRLF3 | 1 | turquoise |
| ENSBTAG00000018398 | GPR4 | 1 | turquoise |
| ENSBTAG00000018403 | ARHGAP9 | 1 | turquoise |
| ENSBTAG00000018416 | CCDC42 | 1 | turquoise |
| ENSBTAG00000018419 | ADCY4 | 1 | turquoise |
| ENSBTAG00000018422 | RIPK3 | 1 | turquoise |
| ENSBTAG00000018423 | DDX5 | 1 | turquoise |
| ENSBTAG00000018424 | ACKR3 | 1 | turquoise |
| ENSBTAG00000018430 | GSAP | 1 | turquoise |
| ENSBTAG00000018436 | UBE2J1 | 1 | turquoise |
| ENSBTAG00000018445 | GRIN3A | 1 | turquoise |
| ENSBTAG00000018446 | GCA | 1 | turquoise |
| ENSBTAG00000018447 | RABGGTB | 1 | turquoise |
| ENSBTAG00000018451 | PTMS | 1 | turquoise |
| ENSBTAG00000018453 | ANKRD37 | 1 | turquoise |
| ENSBTAG00000018464 | ZDHHC14 | 1 | turquoise |
| ENSBTAG00000018474 | IL6R | 1 | turquoise |
| ENSBTAG00000018487 | CFAP45 | 1 | turquoise |
| ENSBTAG00000018498 | PQBP1 | 1 | turquoise |
| ENSBTAG00000018523 | TRIM38 | 1 | turquoise |
| ENSBTAG00000018577 | SLC7A1 | 1 | turquoise |
| ENSBTAG00000018625 | ZNF263 | 1 | turquoise |
| ENSBTAG00000018629 | CWF19L2 | 1 | turquoise |
| ENSBTAG00000018635 | ATP1A3 | 1 | turquoise |
| ENSBTAG00000018638 | CC2D2A | 1 | turquoise |
| ENSBTAG00000018653 | NABP1 | 1 | turquoise |
| ENSBTAG00000018667 | RCL1 | 1 | turquoise |
| ENSBTAG00000018669 | NA | 1 | turquoise |
| ENSBTAG00000018697 | WDR44 | 1 | turquoise |
| ENSBTAG00000018704 | BTG3 | 1 | turquoise |
| ENSBTAG00000018708 | NA | 1 | turquoise |
| ENSBTAG00000018710 | TTF1 | 1 | turquoise |
| ENSBTAG00000018718 | NA | 1 | turquoise |
| ENSBTAG00000018722 | PLEKHM2 | 1 | turquoise |
| ENSBTAG00000018723 | SLC25A34 | 1 | turquoise |
| ENSBTAG00000018730 | ZBTB17 | 1 | turquoise |
| ENSBTAG00000018731 | LOC524181 | 1 | turquoise |
| ENSBTAG00000018773 | RND1 | 1 | turquoise |
| ENSBTAG00000018785 | TUBB1 | 1 | turquoise |
| ENSBTAG00000018804 | CELSR2 | 1 | turquoise |

| | | | |
|---|---|---|---|
| ENSBTAG00000018819 | TENM1 | 1 | turquoise |
| ENSBTAG00000018848 | FBXL3 | 1 | turquoise |
| ENSBTAG00000018851 | MYCBP2 | 1 | turquoise |
| ENSBTAG00000018862 | EIF4E3 | 1 | turquoise |
| ENSBTAG00000018868 | METTL9 | 1 | turquoise |
| ENSBTAG00000018869 | IGSF6 | 1 | turquoise |
| ENSBTAG00000018870 | OTOA | 1 | turquoise |
| ENSBTAG00000018872 | F12 | 1 | turquoise |
| ENSBTAG00000018898 | ARID4A | 1 | turquoise |
| ENSBTAG00000018901 | CTIF | 1 | turquoise |
| ENSBTAG00000018912 | ARHGEF1 | 1 | turquoise |
| ENSBTAG00000018926 | LEAP2 | 1 | turquoise |
| ENSBTAG00000018951 | TBC1D9 | 1 | turquoise |
| ENSBTAG00000018975 | KCNT1 | 1 | turquoise |
| ENSBTAG00000018986 | ACSL4 | 1 | turquoise |
| ENSBTAG00000018991 | TSGA10 | 1 | turquoise |
| ENSBTAG00000018993 | ITGB7 | 1 | turquoise |
| ENSBTAG00000018994 | TNFSF10 | 1 | turquoise |
| ENSBTAG00000019001 | PRDM10 | 1 | turquoise |
| ENSBTAG00000019015 | IFITM3 | 1 | turquoise |
| ENSBTAG00000019017 | LOC112444847 | 1 | turquoise |
| ENSBTAG00000019018 | LOC112441484 | 1 | turquoise |
| ENSBTAG00000019028 | STOX1 | 1 | turquoise |
| ENSBTAG00000019054 | EPSTI1 | 1 | turquoise |
| ENSBTAG00000019058 | IQSEC3 | 1 | turquoise |
| ENSBTAG00000019059 | ATG16L2 | 1 | turquoise |
| ENSBTAG00000019067 | TMC8 | 1 | turquoise |
| ENSBTAG00000019069 | SYNGR2 | 1 | turquoise |
| ENSBTAG00000019073 | TRAF7 | 1 | turquoise |
| ENSBTAG00000019081 | NA | 1 | turquoise |
| ENSBTAG00000019091 | RNPC3 | 1 | turquoise |
| ENSBTAG00000019099 | SLC25A37 | 1 | turquoise |
| ENSBTAG00000019107 | GAS7 | 1 | turquoise |
| ENSBTAG00000019110 | PNRC1 | 1 | turquoise |
| ENSBTAG00000019115 | NA | 1 | turquoise |
| ENSBTAG00000019123 | MEFV | 1 | turquoise |
| ENSBTAG00000019131 | CELF3 | 1 | turquoise |
| ENSBTAG00000019153 | JMJD6 | 1 | turquoise |
| ENSBTAG00000019175 | SOBP | 1 | turquoise |
| ENSBTAG00000019203 | S100A4 | 1 | turquoise |
| ENSBTAG00000019227 | LOC789748 | 1 | turquoise |
| ENSBTAG00000019249 | HS3ST6 | 1 | turquoise |
| ENSBTAG00000019255 | NFE2L2 | 1 | turquoise |
| ENSBTAG00000019272 | LPCAT2 | 1 | turquoise |
| ENSBTAG00000019293 | EGR3 | 1 | turquoise |
| ENSBTAG00000019303 | NA | 1 | turquoise |
| ENSBTAG00000019313 | ZMIZ1 | 1 | turquoise |

| | | | |
|---|---|---|---|
| ENSBTAG00000019314 | USP25 | 1 | turquoise |
| ENSBTAG00000019330 | PROK2 | 1 | turquoise |
| ENSBTAG00000019338 | IL13RA1 | 1 | turquoise |
| ENSBTAG00000019339 | NA | 1 | turquoise |
| ENSBTAG00000019345 | RFK | 1 | turquoise |
| ENSBTAG00000019348 | NA | 1 | turquoise |
| ENSBTAG00000019370 | LOC781001 | 1 | turquoise |
| ENSBTAG00000019375 | SCARF2 | 1 | turquoise |
| ENSBTAG00000019376 | FNDC3B | 1 | turquoise |
| ENSBTAG00000019386 | BOLA-NC1 | 1 | turquoise |
| ENSBTAG00000019390 | CA12 | 1 | turquoise |
| ENSBTAG00000019404 | IL10RB | 1 | turquoise |
| ENSBTAG00000019414 | CLK4 | 1 | turquoise |
| ENSBTAG00000019426 | RNF175 | 1 | turquoise |
| ENSBTAG00000019427 | KIAA0232 | 1 | turquoise |
| ENSBTAG00000019428 | CCR1 | 1 | turquoise |
| ENSBTAG00000019432 | LOC616254 | 1 | turquoise |
| ENSBTAG00000019436 | SLC39A13 | 1 | turquoise |
| ENSBTAG00000019437 | SLFN11 | 1 | turquoise |
| ENSBTAG00000019451 | TAPT1 | 1 | turquoise |
| ENSBTAG00000019456 | SPRED2 | 1 | turquoise |
| ENSBTAG00000019470 | NEK6 | 1 | turquoise |
| ENSBTAG00000019474 | NA | 1 | turquoise |
| ENSBTAG00000019479 | TATDN2 | 1 | turquoise |
| ENSBTAG00000019517 | ELN | 1 | turquoise |
| ENSBTAG00000019530 | KDM8 | 1 | turquoise |
| ENSBTAG00000019537 | MARCHF8 | 1 | turquoise |
| ENSBTAG00000019545 | SPATA13 | 1 | turquoise |
| ENSBTAG00000019547 | NA | 1 | turquoise |
| ENSBTAG00000019565 | NCOA4 | 1 | turquoise |
| ENSBTAG00000019567 | IL21R | 1 | turquoise |
| ENSBTAG00000019581 | PLEKHF2 | 1 | turquoise |
| ENSBTAG00000019588 | BLA-DQB | 1 | turquoise |
| ENSBTAG00000019604 | VASP | 1 | turquoise |
| ENSBTAG00000019606 | IFT52 | 1 | turquoise |
| ENSBTAG00000019617 | PTPN22 | 1 | turquoise |
| ENSBTAG00000019636 | SCARA5 | 1 | turquoise |
| ENSBTAG00000019652 | STK26 | 1 | turquoise |
| ENSBTAG00000019662 | ATXN7L3 | 1 | turquoise |
| ENSBTAG00000019665 | IL1RN | 1 | turquoise |
| ENSBTAG00000019669 | CD163 | 1 | turquoise |
| ENSBTAG00000019672 | LOC574091 | 1 | turquoise |
| ENSBTAG00000019675 | ATXN1 | 1 | turquoise |
| ENSBTAG00000019684 | NUDT3 | 1 | turquoise |
| ENSBTAG00000019695 | RLF | 1 | turquoise |
| ENSBTAG00000019708 | ACSL6 | 1 | turquoise |
| ENSBTAG00000019711 | IRAG2 | 1 | turquoise |

| | | | |
|---|---|---|---|
| ENSBTAG00000019716 | CXCL8 | 1 | turquoise |
| ENSBTAG00000019730 | SFRS18 | 1 | turquoise |
| ENSBTAG00000019741 | C3AR1 | 1 | turquoise |
| ENSBTAG00000019742 | FOXJ2 | 1 | turquoise |
| ENSBTAG00000019796 | FCMR | 1 | turquoise |
| ENSBTAG00000019800 | NA | 1 | turquoise |
| ENSBTAG00000019808 | CCSER1 | 1 | turquoise |
| ENSBTAG00000019814 | PGBD2 | 1 | turquoise |
| ENSBTAG00000019822 | TPPP3 | 1 | turquoise |
| ENSBTAG00000019857 | OTUD4 | 1 | turquoise |
| ENSBTAG00000019866 | NRP1 | 1 | turquoise |
| ENSBTAG00000019872 | PIK3AP1 | 1 | turquoise |
| ENSBTAG00000019884 | PKD1L3 | 1 | turquoise |
| ENSBTAG00000019903 | RAMP2 | 1 | turquoise |
| ENSBTAG00000019910 | SDCBP | 1 | turquoise |
| ENSBTAG00000019915 | GSN | 1 | turquoise |
| ENSBTAG00000019929 | ITGAV | 1 | turquoise |
| ENSBTAG00000019933 | MKNK1 | 1 | turquoise |
| ENSBTAG00000019938 | N4BP1 | 1 | turquoise |
| ENSBTAG00000019953 | CYBB | 1 | turquoise |
| ENSBTAG00000019967 | RASA3 | 1 | turquoise |
| ENSBTAG00000019975 | IL7R | 1 | turquoise |
| ENSBTAG00000019979 | CMPK2 | 1 | turquoise |
| ENSBTAG00000019989 | PXK | 1 | turquoise |
| ENSBTAG00000020000 | RAP1GAP2 | 1 | turquoise |
| ENSBTAG00000020015 | DFFB | 1 | turquoise |
| ENSBTAG00000020019 | XCR1 | 1 | turquoise |
| ENSBTAG00000020023 | MTHFS | 1 | turquoise |
| ENSBTAG00000020034 | LYN | 1 | turquoise |
| ENSBTAG00000020041 | FAM13B | 1 | turquoise |
| ENSBTAG00000020043 | ARHGAP17 | 1 | turquoise |
| ENSBTAG00000020060 | TXNIP | 1 | turquoise |
| ENSBTAG00000020061 | KCNJ12 | 1 | turquoise |
| ENSBTAG00000020062 | TMC4 | 1 | turquoise |
| ENSBTAG00000020080 | MYBPC2 | 1 | turquoise |
| ENSBTAG00000020089 | USP32 | 1 | turquoise |
| ENSBTAG00000020093 | CUL4A | 1 | turquoise |
| ENSBTAG00000020095 | MS4A3 | 1 | turquoise |
| ENSBTAG00000020116 | JSP.1 | 1 | turquoise |
| ENSBTAG00000020141 | FREM1 | 1 | turquoise |
| ENSBTAG00000020149 | RNASEH2B | 1 | turquoise |
| ENSBTAG00000020161 | KLHL22 | 1 | turquoise |
| ENSBTAG00000020164 | BAZ1A | 1 | turquoise |
| ENSBTAG00000020166 | ZNFX1 | 1 | turquoise |
| ENSBTAG00000020172 | DENND2D | 1 | turquoise |
| ENSBTAG00000020173 | INPP5D | 1 | turquoise |
| ENSBTAG00000020213 | NIPAL4 | 1 | turquoise |

| | | | |
|---|---|---|---|
| ENSBTAG00000020225 | TBXAS1 | 1 | turquoise |
| ENSBTAG00000020233 | CCDC186 | 1 | turquoise |
| ENSBTAG00000020237 | NECAP1 | 1 | turquoise |
| ENSBTAG00000020242 | LOC529792 | 1 | turquoise |
| ENSBTAG00000020257 | PTPN5 | 1 | turquoise |
| ENSBTAG00000020261 | CDH26 | 1 | turquoise |
| ENSBTAG00000020270 | NFKB1 | 1 | turquoise |
| ENSBTAG00000020281 | NIN | 1 | turquoise |
| ENSBTAG00000020283 | DUSP5 | 1 | turquoise |
| ENSBTAG00000020294 | PTPN6 | 1 | turquoise |
| ENSBTAG00000020297 | SLC66A3 | 1 | turquoise |
| ENSBTAG00000020303 | FBXO38 | 1 | turquoise |
| ENSBTAG00000020313 | FNBP1 | 1 | turquoise |
| ENSBTAG00000020329 | FAM161B | 1 | turquoise |
| ENSBTAG00000020330 | BTG1 | 1 | turquoise |
| ENSBTAG00000020344 | SLC44A3 | 1 | turquoise |
| ENSBTAG00000020345 | CNN3 | 1 | turquoise |
| ENSBTAG00000020348 | ASTL | 1 | turquoise |
| ENSBTAG00000020350 | DUSP2 | 1 | turquoise |
| ENSBTAG00000020355 | KLF4 | 1 | turquoise |
| ENSBTAG00000020382 | RIN2 | 1 | turquoise |
| ENSBTAG00000020395 | LYSMD1 | 1 | turquoise |
| ENSBTAG00000020418 | CA5B | 1 | turquoise |
| ENSBTAG00000020428 | PAF1 | 1 | turquoise |
| ENSBTAG00000020430 | GLT1D1 | 1 | turquoise |
| ENSBTAG00000020453 | CLEC5A | 1 | turquoise |
| ENSBTAG00000020455 | ITPR1 | 1 | turquoise |
| ENSBTAG00000020458 | STX6 | 1 | turquoise |
| ENSBTAG00000020472 | KDM5A | 1 | turquoise |
| ENSBTAG00000020480 | SPTLC2 | 1 | turquoise |
| ENSBTAG00000020489 | SEMA6A | 1 | turquoise |
| ENSBTAG00000020490 | NACC2 | 1 | turquoise |
| ENSBTAG00000020499 | LOC112443425 | 1 | turquoise |
| ENSBTAG00000020515 | FHIP1B | 1 | turquoise |
| ENSBTAG00000020520 | RASD1 | 1 | turquoise |
| ENSBTAG00000020524 | UPK3BL1 | 1 | turquoise |
| ENSBTAG00000020535 | PYCARD | 1 | turquoise |
| ENSBTAG00000020536 | HERC6 | 1 | turquoise |
| ENSBTAG00000020538 | HERC5 | 1 | turquoise |
| ENSBTAG00000020540 | TMEM198 | 1 | turquoise |
| ENSBTAG00000020542 | SHE | 1 | turquoise |
| ENSBTAG00000020554 | AIF1 | 1 | turquoise |
| ENSBTAG00000020566 | BCR | 1 | turquoise |
| ENSBTAG00000020573 | SCUBE2 | 1 | turquoise |
| ENSBTAG00000020575 | IFITM5 | 1 | turquoise |
| ENSBTAG00000020580 | TCN1 | 1 | turquoise |
| ENSBTAG00000020589 | ENTPD1 | 1 | turquoise |

| | | | |
|---|---|---|---|
| ENSBTAG00000020602 | IDO1 | 1 | turquoise |
| ENSBTAG00000020611 | GLB1L | 1 | turquoise |
| ENSBTAG00000020614 | PRKCE | 1 | turquoise |
| ENSBTAG00000020616 | DGKD | 1 | turquoise |
| ENSBTAG00000020620 | RGS2 | 1 | turquoise |
| ENSBTAG00000020634 | FBXO7 | 1 | turquoise |
| ENSBTAG00000020642 | ZDHHC13 | 1 | turquoise |
| ENSBTAG00000020649 | CIITA | 1 | turquoise |
| ENSBTAG00000020654 | BAZ2B | 1 | turquoise |
| ENSBTAG00000020661 | ABCA1 | 1 | turquoise |
| ENSBTAG00000020669 | NA | 1 | turquoise |
| ENSBTAG00000020674 | LTB | 1 | turquoise |
| ENSBTAG00000020696 | HECW2 | 1 | turquoise |
| ENSBTAG00000020705 | PRSS12 | 1 | turquoise |
| ENSBTAG00000020713 | BACH2 | 1 | turquoise |
| ENSBTAG00000020714 | DDX19B | 1 | turquoise |
| ENSBTAG00000020735 | SMG1 | 1 | turquoise |
| ENSBTAG00000020736 | CD40 | 1 | turquoise |
| ENSBTAG00000020742 | NA | 1 | turquoise |
| ENSBTAG00000020745 | HIVEP3 | 1 | turquoise |
| ENSBTAG00000020747 | ASS1 | 1 | turquoise |
| ENSBTAG00000020755 | SELP | 1 | turquoise |
| ENSBTAG00000020759 | NCLN | 1 | turquoise |
| ENSBTAG00000020760 | SNAP29 | 1 | turquoise |
| ENSBTAG00000020764 | CNN2 | 1 | turquoise |
| ENSBTAG00000020766 | ABCA7 | 1 | turquoise |
| ENSBTAG00000020767 | NA | 1 | turquoise |
| ENSBTAG00000020772 | ARHGAP45 | 1 | turquoise |
| ENSBTAG00000020780 | SBNO2 | 1 | turquoise |
| ENSBTAG00000020783 | MAPK14 | 1 | turquoise |
| ENSBTAG00000020791 | RAPGEF1 | 1 | turquoise |
| ENSBTAG00000020796 | UBE2D1 | 1 | turquoise |
| ENSBTAG00000020801 | CYRIB | 1 | turquoise |
| ENSBTAG00000020807 | FAU | 1 | turquoise |
| ENSBTAG00000020848 | PIK3CG | 1 | turquoise |
| ENSBTAG00000020860 | CHD1 | 1 | turquoise |
| ENSBTAG00000020865 | PELI1 | 1 | turquoise |
| ENSBTAG00000020872 | C5AR1 | 1 | turquoise |
| ENSBTAG00000020884 | CASP4 | 1 | turquoise |
| ENSBTAG00000020892 | IL2RA | 1 | turquoise |
| ENSBTAG00000020893 | MATN3 | 1 | turquoise |
| ENSBTAG00000020900 | RPUSD4 | 1 | turquoise |
| ENSBTAG00000020904 | JAK3 | 1 | turquoise |
| ENSBTAG00000020907 | PSMC3IP | 1 | turquoise |
| ENSBTAG00000020911 | FNBP4 | 1 | turquoise |
| ENSBTAG00000020921 | PRKCB | 1 | turquoise |
| ENSBTAG00000020935 | HIF1A | 1 | turquoise |

| | | | |
|---|---|---|---|
| ENSBTAG00000020936 | NOD2 | 1 | turquoise |
| ENSBTAG00000020937 | SNAPC1 | 1 | turquoise |
| ENSBTAG00000020942 | RNF166 | 1 | turquoise |
| ENSBTAG00000020963 | RASSF1 | 1 | turquoise |
| ENSBTAG00000020965 | ZMYND10 | 1 | turquoise |
| ENSBTAG00000020969 | DCAF6 | 1 | turquoise |
| ENSBTAG00000020990 | P2RY14 | 1 | turquoise |
| ENSBTAG00000021021 | C21H15orf39 | 1 | turquoise |
| ENSBTAG00000021029 | PRR5L | 1 | turquoise |
| ENSBTAG00000021041 | PRAM1 | 1 | turquoise |
| ENSBTAG00000021045 | E2F3 | 1 | turquoise |
| ENSBTAG00000021071 | TRIM8 | 1 | turquoise |
| ENSBTAG00000021077 | BOLA-DQB | 1 | turquoise |
| ENSBTAG00000021087 | UBR1 | 1 | turquoise |
| ENSBTAG00000021091 | ANKFY1 | 1 | turquoise |
| ENSBTAG00000021105 | IRAK4 | 1 | turquoise |
| ENSBTAG00000021144 | PSTPIP1 | 1 | turquoise |
| ENSBTAG00000021145 | MAP4K1 | 1 | turquoise |
| ENSBTAG00000021165 | VPS9D1 | 1 | turquoise |
| ENSBTAG00000021166 | NA | 1 | turquoise |
| ENSBTAG00000021176 | CRISPLD2 | 1 | turquoise |
| ENSBTAG00000021182 | DOCK4 | 1 | turquoise |
| ENSBTAG00000021187 | ID2 | 1 | turquoise |
| ENSBTAG00000021214 | FBXL5 | 1 | turquoise |
| ENSBTAG00000021223 | CRY2 | 1 | turquoise |
| ENSBTAG00000021224 | ZSWIM6 | 1 | turquoise |
| ENSBTAG00000021240 | DCSTAMP | 1 | turquoise |
| ENSBTAG00000021245 | SPRY1 | 1 | turquoise |
| ENSBTAG00000021260 | GALNT11 | 1 | turquoise |
| ENSBTAG00000021263 | CYP4V2 | 1 | turquoise |
| ENSBTAG00000021272 | ABCG1 | 1 | turquoise |
| ENSBTAG00000021273 | SEMA4B | 1 | turquoise |
| ENSBTAG00000021286 | SLC37A1 | 1 | turquoise |
| ENSBTAG00000021287 | SLC16A7 | 1 | turquoise |
| ENSBTAG00000021289 | POGZ | 1 | turquoise |
| ENSBTAG00000021326 | CCL20 | 1 | turquoise |
| ENSBTAG00000021338 | OAF | 1 | turquoise |
| ENSBTAG00000021341 | BTBD1 | 1 | turquoise |
| ENSBTAG00000021381 | DAAM2 | 1 | turquoise |
| ENSBTAG00000021392 | DCAF11 | 1 | turquoise |
| ENSBTAG00000021394 | FITM1 | 1 | turquoise |
| ENSBTAG00000021395 | PSME1 | 1 | turquoise |
| ENSBTAG00000021396 | HEG1 | 1 | turquoise |
| ENSBTAG00000021435 | MAFF | 1 | turquoise |
| ENSBTAG00000021452 | TRANK1 | 1 | turquoise |
| ENSBTAG00000021462 | CSF3 | 1 | turquoise |
| ENSBTAG00000021464 | SPRYD3 | 1 | turquoise |

| | | | |
|---|---|---|---|
| ENSBTAG00000021474 | GSDMD | 1 | turquoise |
| ENSBTAG00000021480 | SAG | 1 | turquoise |
| ENSBTAG00000021487 | CIART | 1 | turquoise |
| ENSBTAG00000021490 | WDR4 | 1 | turquoise |
| ENSBTAG00000021501 | LOC784768 | 1 | turquoise |
| ENSBTAG00000021504 | TIRAP | 1 | turquoise |
| ENSBTAG00000021505 | USP36 | 1 | turquoise |
| ENSBTAG00000021514 | DNPEP | 1 | turquoise |
| ENSBTAG00000021516 | GSTA1 | 1 | turquoise |
| ENSBTAG00000021521 | BFAR | 1 | turquoise |
| ENSBTAG00000021523 | STAT3 | 1 | turquoise |
| ENSBTAG00000021573 | DCAF12 | 1 | turquoise |
| ENSBTAG00000021574 | TCF7L2 | 1 | turquoise |
| ENSBTAG00000021577 | ZFYVE16 | 1 | turquoise |
| ENSBTAG00000021587 | SMPDL3A | 1 | turquoise |
| ENSBTAG00000021595 | HIP1R | 1 | turquoise |
| ENSBTAG00000021602 | CTTNBP2NL | 1 | turquoise |
| ENSBTAG00000021617 | ZC3HAV1 | 1 | turquoise |
| ENSBTAG00000021647 | FCAR | 1 | turquoise |
| ENSBTAG00000021658 | TAMALIN | 1 | turquoise |
| ENSBTAG00000021687 | JADE2 | 1 | turquoise |
| ENSBTAG00000021703 | MED12L | 1 | turquoise |
| ENSBTAG00000021726 | CWC22 | 1 | turquoise |
| ENSBTAG00000021761 | BRAF | 1 | turquoise |
| ENSBTAG00000021766 | HBEGF | 1 | turquoise |
| ENSBTAG00000021768 | CCNG2 | 1 | turquoise |
| ENSBTAG00000021775 | SLC4A9 | 1 | turquoise |
| ENSBTAG00000021791 | PARP9 | 1 | turquoise |
| ENSBTAG00000021803 | GRAMD4 | 1 | turquoise |
| ENSBTAG00000021818 | ADGRE5 | 1 | turquoise |
| ENSBTAG00000021819 | IFNAR1 | 1 | turquoise |
| ENSBTAG00000021820 | DDX39A | 1 | turquoise |
| ENSBTAG00000021842 | FCGR2B | 1 | turquoise |
| ENSBTAG00000021856 | R3HCC1L | 1 | turquoise |
| ENSBTAG00000021887 | DPYS | 1 | turquoise |
| ENSBTAG00000021911 | PTPRG | 1 | turquoise |
| ENSBTAG00000021918 | EZH1 | 1 | turquoise |
| ENSBTAG00000021933 | ALOX12 | 1 | turquoise |
| ENSBTAG00000021962 | BCLAF3 | 1 | turquoise |
| ENSBTAG00000021967 | TMEM168 | 1 | turquoise |
| ENSBTAG00000021968 | MID2 | 1 | turquoise |
| ENSBTAG00000022022 | GUCD1 | 1 | turquoise |
| ENSBTAG00000022137 | NA | 1 | turquoise |
| ENSBTAG00000022227 | PLSCR2 | 1 | turquoise |
| ENSBTAG00000022275 | LOC100337136 | 1 | turquoise |
| ENSBTAG00000022288 | AMMECR1 | 1 | turquoise |
| ENSBTAG00000022373 | LOC112441455 | 1 | turquoise |

| | | | |
|---|---|---|---|
| ENSBTAG00000022394 | SAA2 | 1 | turquoise |
| ENSBTAG00000022395 | LOC104968478 | 1 | turquoise |
| ENSBTAG00000022396 | NA | 1 | turquoise |
| ENSBTAG00000022489 | LOC509283 | 1 | turquoise |
| ENSBTAG00000022539 | POLR2K | 1 | turquoise |
| ENSBTAG00000022590 | NA | 1 | turquoise |
| ENSBTAG00000022598 | NRADD | 1 | turquoise |
| ENSBTAG00000022622 | CISH | 1 | turquoise |
| ENSBTAG00000022681 | EOGT | 1 | turquoise |
| ENSBTAG00000022689 | RYBP | 1 | turquoise |
| ENSBTAG00000022779 | OLFM4 | 1 | turquoise |
| ENSBTAG00000022813 | TMEM150B | 1 | turquoise |
| ENSBTAG00000022825 | BRI3 | 1 | turquoise |
| ENSBTAG00000022890 | MBP | 1 | turquoise |
| ENSBTAG00000022895 | NA | 1 | turquoise |
| ENSBTAG00000023026 | LOC281376 | 1 | turquoise |
| ENSBTAG00000023054 | SLC25A16 | 1 | turquoise |
| ENSBTAG00000023064 | NA | 1 | turquoise |
| ENSBTAG00000023073 | FAM89A | 1 | turquoise |
| ENSBTAG00000023144 | PTPRC | 1 | turquoise |
| ENSBTAG00000023169 | NINL | 1 | turquoise |
| ENSBTAG00000023179 | TRIB1 | 1 | turquoise |
| ENSBTAG00000023198 | SERPINB2 | 1 | turquoise |
| ENSBTAG00000023279 | N4BP2L2 | 1 | turquoise |
| ENSBTAG00000023283 | JAML | 1 | turquoise |
| ENSBTAG00000023309 | LOC100337390 | 1 | turquoise |
| ENSBTAG00000023375 | METTL6 | 1 | turquoise |
| ENSBTAG00000023377 | SH3BP5 | 1 | turquoise |
| ENSBTAG00000023551 | MOB3C | 1 | turquoise |
| ENSBTAG00000023607 | HACD2 | 1 | turquoise |
| ENSBTAG00000023610 | U2AF1L4 | 1 | turquoise |
| ENSBTAG00000023635 | CLEC6A | 1 | turquoise |
| ENSBTAG00000023648 | LOC618664 | 1 | turquoise |
| ENSBTAG00000023659 | MT2A | 1 | turquoise |
| ENSBTAG00000023675 | ZNF654 | 1 | turquoise |
| ENSBTAG00000023745 | BANP | 1 | turquoise |
| ENSBTAG00000023847 | IKZF5 | 1 | turquoise |
| ENSBTAG00000023851 | FAM102A | 1 | turquoise |
| ENSBTAG00000023867 | RC3H2 | 1 | turquoise |
| ENSBTAG00000023929 | FOSL2 | 1 | turquoise |
| ENSBTAG00000023963 | RHBDD1 | 1 | turquoise |
| ENSBTAG00000023970 | LOC783508 | 1 | turquoise |
| ENSBTAG00000023976 | ARHGAP25 | 1 | turquoise |
| ENSBTAG00000024097 | MRPS15 | 1 | turquoise |
| ENSBTAG00000024105 | BCL2L11 | 1 | turquoise |
| ENSBTAG00000024177 | NA | 1 | turquoise |
| ENSBTAG00000024272 | NA | 1 | turquoise |

| | | | |
|---|---|---|---|
| ENSBTAG00000024387 | PNPLA8 | 1 | turquoise |
| ENSBTAG00000024407 | LOC787530 | 1 | turquoise |
| ENSBTAG00000024493 | DHRS3 | 1 | turquoise |
| ENSBTAG00000024496 | CELA2A | 1 | turquoise |
| ENSBTAG00000024503 | FCER1G | 1 | turquoise |
| ENSBTAG00000024561 | H3-3A | 1 | turquoise |
| ENSBTAG00000024595 | HECA | 1 | turquoise |
| ENSBTAG00000024641 | NA | 1 | turquoise |
| ENSBTAG00000024715 | PTPRJ | 1 | turquoise |
| ENSBTAG00000024815 | ANKRD28 | 1 | turquoise |
| ENSBTAG00000024822 | SETX | 1 | turquoise |
| ENSBTAG00000024849 | LOC100138271 | 1 | turquoise |
| ENSBTAG00000024851 | TRIM14 | 1 | turquoise |
| ENSBTAG00000024901 | LOC509881 | 1 | turquoise |
| ENSBTAG00000024903 | KCNJ16 | 1 | turquoise |
| ENSBTAG00000024909 | H3-3B | 1 | turquoise |
| ENSBTAG00000024915 | SECTM1A | 1 | turquoise |
| ENSBTAG00000024918 | BIRC3 | 1 | turquoise |
| ENSBTAG00000024928 | TNFRSF1B | 1 | turquoise |
| ENSBTAG00000024934 | NA | 1 | turquoise |
| ENSBTAG00000025099 | RNF145 | 1 | turquoise |
| ENSBTAG00000025124 | ZNF300 | 1 | turquoise |
| ENSBTAG00000025129 | MNT | 1 | turquoise |
| ENSBTAG00000025130 | HIC1 | 1 | turquoise |
| ENSBTAG00000025219 | LOC100848263 | 1 | turquoise |
| ENSBTAG00000025233 | CBARP | 1 | turquoise |
| ENSBTAG00000025250 | CCL3 | 1 | turquoise |
| ENSBTAG00000025257 | CCL4 | 1 | turquoise |
| ENSBTAG00000025258 | LOC515676 | 1 | turquoise |
| ENSBTAG00000025280 | NA | 1 | turquoise |
| ENSBTAG00000025391 | NA | 1 | turquoise |
| ENSBTAG00000025401 | DOK3 | 1 | turquoise |
| ENSBTAG00000025405 | BATF | 1 | turquoise |
| ENSBTAG00000025424 | NOTCH4 | 1 | turquoise |
| ENSBTAG00000025425 | RAB24 | 1 | turquoise |
| ENSBTAG00000025443 | FNIP1 | 1 | turquoise |
| ENSBTAG00000025445 | LOC789148 | 1 | turquoise |
| ENSBTAG00000025450 | SYNE2 | 1 | turquoise |
| ENSBTAG00000025462 | GADD45B | 1 | turquoise |
| ENSBTAG00000025471 | TNF | 1 | turquoise |
| ENSBTAG00000025494 | PAQR8 | 1 | turquoise |
| ENSBTAG00000025516 | GNL1 | 1 | turquoise |
| ENSBTAG00000025644 | CALM1 | 1 | turquoise |
| ENSBTAG00000025762 | CNP | 1 | turquoise |
| ENSBTAG00000025775 | INSL3 | 1 | turquoise |
| ENSBTAG00000025782 | TNFSF8 | 1 | turquoise |
| ENSBTAG00000025812 | NA | 1 | turquoise |

| | | | |
|---|---|---|---|
| ENSBTAG00000025826 | SLC24A1 | 1 | turquoise |
| ENSBTAG00000025848 | RBM3 | 1 | turquoise |
| ENSBTAG00000025898 | TBC1D8 | 1 | turquoise |
| ENSBTAG00000025953 | NA | 1 | turquoise |
| ENSBTAG00000026070 | LOC107131273 | 1 | turquoise |
| ENSBTAG00000026080 | LAIR1 | 1 | turquoise |
| ENSBTAG00000026148 | LOC100300051 | 1 | turquoise |
| ENSBTAG00000026192 | NA | 1 | turquoise |
| ENSBTAG00000026199 | ACTB | 1 | turquoise |
| ENSBTAG00000026233 | NA | 1 | turquoise |
| ENSBTAG00000026234 | STK3 | 1 | turquoise |
| ENSBTAG00000026254 | MED30 | 1 | turquoise |
| ENSBTAG00000026309 | ZHX2 | 1 | turquoise |
| ENSBTAG00000026326 | SPN | 1 | turquoise |
| ENSBTAG00000026376 | NA | 1 | turquoise |
| ENSBTAG00000026394 | UBALD1 | 1 | turquoise |
| ENSBTAG00000026403 | CREBBP | 1 | turquoise |
| ENSBTAG00000026437 | NA | 1 | turquoise |
| ENSBTAG00000026502 | CYP2D14 | 1 | turquoise |
| ENSBTAG00000026637 | CCDC162P | 1 | turquoise |
| ENSBTAG00000026645 | LOC526787 | 1 | turquoise |
| ENSBTAG00000026676 | SDC3 | 1 | turquoise |
| ENSBTAG00000026753 | CXCR1 | 1 | turquoise |
| ENSBTAG00000026754 | RNF41 | 1 | turquoise |
| ENSBTAG00000026779 | LYZ | 1 | turquoise |
| ENSBTAG00000026796 | LOC507756 | 1 | turquoise |
| ENSBTAG00000026944 | NA | 1 | turquoise |
| ENSBTAG00000026993 | TMBIM4 | 1 | turquoise |
| ENSBTAG00000027051 | PTAFR | 1 | turquoise |
| ENSBTAG00000027064 | BTBD11 | 1 | turquoise |
| ENSBTAG00000027173 | CHP1 | 1 | turquoise |
| ENSBTAG00000027201 | RAPH1 | 1 | turquoise |
| ENSBTAG00000027317 | RNF114 | 1 | turquoise |
| ENSBTAG00000027326 | TP53RK | 1 | turquoise |
| ENSBTAG00000027327 | OCSTAMP | 1 | turquoise |
| ENSBTAG00000027348 | OOSP2 | 1 | turquoise |
| ENSBTAG00000027397 | PPP1R3D | 1 | turquoise |
| ENSBTAG00000027444 | SVIL | 1 | turquoise |
| ENSBTAG00000027513 | CXCL2 | 1 | turquoise |
| ENSBTAG00000027557 | ZNF408 | 1 | turquoise |
| ENSBTAG00000027626 | GOLGA7 | 1 | turquoise |
| ENSBTAG00000027642 | C15H11orf42 | 1 | turquoise |
| ENSBTAG00000027655 | NA | 1 | turquoise |
| ENSBTAG00000027665 | BRF2 | 1 | turquoise |
| ENSBTAG00000027676 | IL18BP | 1 | turquoise |
| ENSBTAG00000027713 | LYSMD3 | 1 | turquoise |
| ENSBTAG00000027760 | NA | 1 | turquoise |

| | | | |
|---|---|---|---|
| ENSBTAG00000027809 | RABGAP1L | 1 | turquoise |
| ENSBTAG00000027916 | MALT1 | 1 | turquoise |
| ENSBTAG00000027924 | SNX18 | 1 | turquoise |
| ENSBTAG00000027937 | NA | 1 | turquoise |
| ENSBTAG00000028421 | LOC112442826 | 1 | turquoise |
| ENSBTAG00000029765 | NA | 1 | turquoise |
| ENSBTAG00000029803 | MIR221 | 1 | turquoise |
| ENSBTAG00000029828 | MIR421 | 1 | turquoise |
| ENSBTAG00000029830 | MIR146A | 1 | turquoise |
| ENSBTAG00000029855 | MIR101-1 | 1 | turquoise |
| ENSBTAG00000029897 | MIR21 | 1 | turquoise |
| ENSBTAG00000029901 | MIR23A | 1 | turquoise |
| ENSBTAG00000029904 | MIR222 | 1 | turquoise |
| ENSBTAG00000029905 | NA | 1 | turquoise |
| ENSBTAG00000029930 | MIR22 | 1 | turquoise |
| ENSBTAG00000029942 | NA | 1 | turquoise |
| ENSBTAG00000029982 | MIR142 | 1 | turquoise |
| ENSBTAG00000029994 | MIRLET7I | 1 | turquoise |
| ENSBTAG00000029996 | MIR27A | 1 | turquoise |
| ENSBTAG00000030071 | MIR24-2 | 1 | turquoise |
| ENSBTAG00000030168 | MIF4GD | 1 | turquoise |
| ENSBTAG00000030172 | PRPSAP1 | 1 | turquoise |
| ENSBTAG00000030173 | QRICH2 | 1 | turquoise |
| ENSBTAG00000030182 | MAPK11 | 1 | turquoise |
| ENSBTAG00000030190 | COL6A3 | 1 | turquoise |
| ENSBTAG00000030193 | GPR35 | 1 | turquoise |
| ENSBTAG00000030230 | NA | 1 | turquoise |
| ENSBTAG00000030416 | LOC538810 | 1 | turquoise |
| ENSBTAG00000030424 | CLEC1A | 1 | turquoise |
| ENSBTAG00000030425 | ID3 | 1 | turquoise |
| ENSBTAG00000030439 | NA | 1 | turquoise |
| ENSBTAG00000030503 | H2AJ | 1 | turquoise |
| ENSBTAG00000030518 | ECSCR | 1 | turquoise |
| ENSBTAG00000030521 | IER5L | 1 | turquoise |
| ENSBTAG00000030589 | TMCO6 | 1 | turquoise |
| ENSBTAG00000030675 | PDCL | 1 | turquoise |
| ENSBTAG00000030836 | ZBTB21 | 1 | turquoise |
| ENSBTAG00000030845 | MPEG1 | 1 | turquoise |
| ENSBTAG00000030913 | MX1 | 1 | turquoise |
| ENSBTAG00000030921 | FAM3B | 1 | turquoise |
| ENSBTAG00000030932 | IFI44L | 1 | turquoise |
| ENSBTAG00000030951 | ZNF655 | 1 | turquoise |
| ENSBTAG00000030973 | TUBA1D | 1 | turquoise |
| ENSBTAG00000030974 | TUBA4A | 1 | turquoise |
| ENSBTAG00000031020 | TLR8 | 1 | turquoise |
| ENSBTAG00000031166 | ZDBF2 | 1 | turquoise |
| ENSBTAG00000031214 | LOC618409 | 1 | turquoise |

| | | | |
|---|---|---|---|
| ENSBTAG00000031231 | IRF1 | 1 | turquoise |
| ENSBTAG00000031252 | CD82 | 1 | turquoise |
| ENSBTAG00000031256 | NA | 1 | turquoise |
| ENSBTAG00000031265 | SVOPL | 1 | turquoise |
| ENSBTAG00000031287 | TMEM158 | 1 | turquoise |
| ENSBTAG00000031332 | RBKS | 1 | turquoise |
| ENSBTAG00000031355 | LOC529196 | 1 | turquoise |
| ENSBTAG00000031358 | DPYD | 1 | turquoise |
| ENSBTAG00000031385 | COP1 | 1 | turquoise |
| ENSBTAG00000031387 | FZR1 | 1 | turquoise |
| ENSBTAG00000031396 | NA | 1 | turquoise |
| ENSBTAG00000031397 | NA | 1 | turquoise |
| ENSBTAG00000031430 | NA | 1 | turquoise |
| ENSBTAG00000031441 | FXYD5 | 1 | turquoise |
| ENSBTAG00000031444 | LY86 | 1 | turquoise |
| ENSBTAG00000031453 | CCM2L | 1 | turquoise |
| ENSBTAG00000031462 | FAM209A | 1 | turquoise |
| ENSBTAG00000031468 | LOC614881 | 1 | turquoise |
| ENSBTAG00000031476 | DLL1 | 1 | turquoise |
| ENSBTAG00000031483 | NA | 1 | turquoise |
| ENSBTAG00000031519 | NGP | 1 | turquoise |
| ENSBTAG00000031544 | DDIT3 | 1 | turquoise |
| ENSBTAG00000031548 | LOC514257 | 1 | turquoise |
| ENSBTAG00000031558 | NA | 1 | turquoise |
| ENSBTAG00000031696 | ANKRD44 | 1 | turquoise |
| ENSBTAG00000031701 | TAGAP | 1 | turquoise |
| ENSBTAG00000031718 | OGFR | 1 | turquoise |
| ENSBTAG00000031731 | HIGD1A | 1 | turquoise |
| ENSBTAG00000031746 | NA | 1 | turquoise |
| ENSBTAG00000031750 | PLAC8B | 1 | turquoise |
| ENSBTAG00000031756 | NA | 1 | turquoise |
| ENSBTAG00000031773 | NA | 1 | turquoise |
| ENSBTAG00000031778 | H2BC5 | 1 | turquoise |
| ENSBTAG00000031789 | FBRS | 1 | turquoise |
| ENSBTAG00000031795 | NA | 1 | turquoise |
| ENSBTAG00000031797 | MANF | 1 | turquoise |
| ENSBTAG00000031814 | SDS | 1 | turquoise |
| ENSBTAG00000031849 | TMEM119 | 1 | turquoise |
| ENSBTAG00000031851 | SELPLG | 1 | turquoise |
| ENSBTAG00000031886 | H2BC15 | 1 | turquoise |
| ENSBTAG00000031889 | NA | 1 | turquoise |
| ENSBTAG00000031895 | TFB1M | 1 | turquoise |
| ENSBTAG00000031905 | LOC107131731 | 1 | turquoise |
| ENSBTAG00000031940 | RSBN1 | 1 | turquoise |
| ENSBTAG00000031950 | RAB3IP | 1 | turquoise |
| ENSBTAG00000031962 | NA | 1 | turquoise |
| ENSBTAG00000031998 | CXCL16 | 1 | turquoise |

| | | | |
|---|---|---|---|
| ENSBTAG00000032031 | ZBTB12 | 1 | turquoise |
| ENSBTAG00000032071 | SYCP1 | 1 | turquoise |
| ENSBTAG00000032084 | GPR183 | 1 | turquoise |
| ENSBTAG00000032163 | LTV1 | 1 | turquoise |
| ENSBTAG00000032209 | CXHXorf65 | 1 | turquoise |
| ENSBTAG00000032260 | DCUN1D3 | 1 | turquoise |
| ENSBTAG00000032265 | RTP4 | 1 | turquoise |
| ENSBTAG00000032277 | KYNU | 1 | turquoise |
| ENSBTAG00000032281 | LOC515547 | 1 | turquoise |
| ENSBTAG00000032289 | ARHGAP15 | 1 | turquoise |
| ENSBTAG00000032369 | NMI | 1 | turquoise |
| ENSBTAG00000032373 | LOC101905757 | 1 | turquoise |
| ENSBTAG00000032374 | SELENOK | 1 | turquoise |
| ENSBTAG00000032433 | DENR | 1 | turquoise |
| ENSBTAG00000032493 | RETREG3 | 1 | turquoise |
| ENSBTAG00000032518 | SPRED1 | 1 | turquoise |
| ENSBTAG00000032544 | NA | 1 | turquoise |
| ENSBTAG00000032603 | LOC515333 | 1 | turquoise |
| ENSBTAG00000032660 | CHIC2 | 1 | turquoise |
| ENSBTAG00000032686 | HOMEZ | 1 | turquoise |
| ENSBTAG00000032764 | NA | 1 | turquoise |
| ENSBTAG00000032808 | FASLG | 1 | turquoise |
| ENSBTAG00000032869 | RNASE10 | 1 | turquoise |
| ENSBTAG00000032914 | SLC11A2 | 1 | turquoise |
| ENSBTAG00000033041 | YIPF4 | 1 | turquoise |
| ENSBTAG00000033078 | CNEP1R1 | 1 | turquoise |
| ENSBTAG00000033136 | COX20 | 1 | turquoise |
| ENSBTAG00000033218 | NA | 1 | turquoise |
| ENSBTAG00000033255 | DUSP11 | 1 | turquoise |
| ENSBTAG00000033299 | IGFALS | 1 | turquoise |
| ENSBTAG00000033315 | DNAJC1 | 1 | turquoise |
| ENSBTAG00000033316 | PDCD1LG2 | 1 | turquoise |
| ENSBTAG00000033334 | SPATA2L | 1 | turquoise |
| ENSBTAG00000033395 | C25H16orf91 | 1 | turquoise |
| ENSBTAG00000033529 | NA | 1 | turquoise |
| ENSBTAG00000033547 | C17H4orf45 | 1 | turquoise |
| ENSBTAG00000033565 | NA | 1 | turquoise |
| ENSBTAG00000033669 | IL17RC | 1 | turquoise |
| ENSBTAG00000033702 | STARD8 | 1 | turquoise |
| ENSBTAG00000033735 | WASHC4 | 1 | turquoise |
| ENSBTAG00000033748 | IL18RAP | 1 | turquoise |
| ENSBTAG00000034225 | TAF5L | 1 | turquoise |
| ENSBTAG00000034302 | NA | 1 | turquoise |
| ENSBTAG00000034349 | IFI44 | 1 | turquoise |
| ENSBTAG00000034366 | RGS2 | 1 | turquoise |
| ENSBTAG00000034384 | VWA3B | 1 | turquoise |
| ENSBTAG00000034449 | NA | 1 | turquoise |

| | | | |
|---|---|---|---|
| ENSBTAG00000034493 | ILRUN | 1 | turquoise |
| ENSBTAG00000034580 | NA | 1 | turquoise |
| ENSBTAG00000034633 | ERO1B | 1 | turquoise |
| ENSBTAG00000034643 | NA | 1 | turquoise |
| ENSBTAG00000034659 | UBTD2 | 1 | turquoise |
| ENSBTAG00000034676 | HIGD1A | 1 | turquoise |
| ENSBTAG00000034689 | ZNRF1 | 1 | turquoise |
| ENSBTAG00000034850 | NA | 1 | turquoise |
| ENSBTAG00000034875 | ALPK1 | 1 | turquoise |
| ENSBTAG00000034885 | RGCC | 1 | turquoise |
| ENSBTAG00000034918 | IFIT2 | 1 | turquoise |
| ENSBTAG00000035081 | SERINC2 | 1 | turquoise |
| ENSBTAG00000035183 | NA | 1 | turquoise |
| ENSBTAG00000035206 | NA | 1 | turquoise |
| ENSBTAG00000035224 | NA | 1 | turquoise |
| ENSBTAG00000035226 | NA | 1 | turquoise |
| ENSBTAG00000035584 | NA | 1 | turquoise |
| ENSBTAG00000035641 | NA | 1 | turquoise |
| ENSBTAG00000035735 | BIRC2 | 1 | turquoise |
| ENSBTAG00000035744 | LOC785873 | 1 | turquoise |
| ENSBTAG00000035827 | ATG7 | 1 | turquoise |
| ENSBTAG00000035858 | MCC | 1 | turquoise |
| ENSBTAG00000035868 | LOC614923 | 1 | turquoise |
| ENSBTAG00000035907 | DDX3X | 1 | turquoise |
| ENSBTAG00000035975 | NA | 1 | turquoise |
| ENSBTAG00000035995 | TNFAIP2 | 1 | turquoise |
| ENSBTAG00000036016 | FAM111B | 1 | turquoise |
| ENSBTAG00000036061 | LOC100336795 | 1 | turquoise |
| ENSBTAG00000036078 | EMP1 | 1 | turquoise |
| ENSBTAG00000036127 | AS3MT | 1 | turquoise |
| ENSBTAG00000036224 | MGC157082 | 1 | turquoise |
| ENSBTAG00000036298 | LOC515578 | 1 | turquoise |
| ENSBTAG00000036367 | MIR374B | 1 | turquoise |
| ENSBTAG00000036391 | MIR212 | 1 | turquoise |
| ENSBTAG00000036392 | MIR371 | 1 | turquoise |
| ENSBTAG00000036418 | MIR223 | 1 | turquoise |
| ENSBTAG00000036437 | MIR33B | 1 | turquoise |
| ENSBTAG00000037179 | LOC112443347 | 1 | turquoise |
| ENSBTAG00000037397 | PRSS36 | 1 | turquoise |
| ENSBTAG00000037413 | TMEM164 | 1 | turquoise |
| ENSBTAG00000037421 | NA | 1 | turquoise |
| ENSBTAG00000037452 | LOC790312 | 1 | turquoise |
| ENSBTAG00000037457 | MBD2 | 1 | turquoise |
| ENSBTAG00000037465 | TRIM34 | 1 | turquoise |
| ENSBTAG00000037470 | NA | 1 | turquoise |
| ENSBTAG00000037490 | NA | 1 | turquoise |
| ENSBTAG00000037496 | NA | 1 | turquoise |

| | | | |
|---|---|---|---|
| ENSBTAG00000037523 | B3GALT6 | 1 | turquoise |
| ENSBTAG00000037527 | OAS1Z | 1 | turquoise |
| ENSBTAG00000037533 | C4A | 1 | turquoise |
| ENSBTAG00000037537 | LOC618463 | 1 | turquoise |
| ENSBTAG00000037554 | NA | 1 | turquoise |
| ENSBTAG00000037558 | GRO1 | 1 | turquoise |
| ENSBTAG00000037605 | LOC100848815 | 1 | turquoise |
| ENSBTAG00000037608 | NA | 1 | turquoise |
| ENSBTAG00000037634 | GBP4 | 1 | turquoise |
| ENSBTAG00000037702 | SP140L | 1 | turquoise |
| ENSBTAG00000037735 | C5AR2 | 1 | turquoise |
| ENSBTAG00000037743 | LOC112446756 | 1 | turquoise |
| ENSBTAG00000037799 | LOC515697 | 1 | turquoise |
| ENSBTAG00000037800 | APOBEC3Z1 | 1 | turquoise |
| ENSBTAG00000037811 | CCL2 | 1 | turquoise |
| ENSBTAG00000037830 | NA | 1 | turquoise |
| ENSBTAG00000037902 | FAM229A | 1 | turquoise |
| ENSBTAG00000037937 | LOC101903126 | 1 | turquoise |
| ENSBTAG00000037962 | MAML2 | 1 | turquoise |
| ENSBTAG00000037964 | H2AW | 1 | turquoise |
| ENSBTAG00000037989 | NA | 1 | turquoise |
| ENSBTAG00000038042 | CXCR2 | 1 | turquoise |
| ENSBTAG00000038062 | FUT4 | 1 | turquoise |
| ENSBTAG00000038084 | NA | 1 | turquoise |
| ENSBTAG00000038107 | MAPKAPK2 | 1 | turquoise |
| ENSBTAG00000038124 | FCGR2A | 1 | turquoise |
| ENSBTAG00000038128 | BOLA-DQA5 | 1 | turquoise |
| ENSBTAG00000038149 | NLRP12 | 1 | turquoise |
| ENSBTAG00000038154 | HSH2D | 1 | turquoise |
| ENSBTAG00000038186 | GLRX | 1 | turquoise |
| ENSBTAG00000038233 | LOC507055 | 1 | turquoise |
| ENSBTAG00000038235 | NOD1 | 1 | turquoise |
| ENSBTAG00000038238 | LOC101902340 | 1 | turquoise |
| ENSBTAG00000038321 | LIPE | 1 | turquoise |
| ENSBTAG00000038335 | LOC534155 | 1 | turquoise |
| ENSBTAG00000038375 | STOM | 1 | turquoise |
| ENSBTAG00000038397 | BOLA-DRB2 | 1 | turquoise |
| ENSBTAG00000038428 | RELB | 1 | turquoise |
| ENSBTAG00000038476 | NA | 1 | turquoise |
| ENSBTAG00000038488 | TMSB4X | 1 | turquoise |
| ENSBTAG00000038494 | NA | 1 | turquoise |
| ENSBTAG00000038500 | NA | 1 | turquoise |
| ENSBTAG00000038536 | LOC616948 | 1 | turquoise |
| ENSBTAG00000038576 | GBP6 | 1 | turquoise |
| ENSBTAG00000038619 | LOC512672 | 1 | turquoise |
| ENSBTAG00000038625 | LOC513659 | 1 | turquoise |
| ENSBTAG00000038639 | NA | 1 | turquoise |

| | | | |
|---|---|---|---|
| ENSBTAG00000038640 | NA | 1 | turquoise |
| ENSBTAG00000038650 | MS4A7 | 1 | turquoise |
| ENSBTAG00000038662 | GJB6 | 1 | turquoise |
| ENSBTAG00000038710 | NA | 1 | turquoise |
| ENSBTAG00000038737 | P2RY6 | 1 | turquoise |
| ENSBTAG00000038797 | NA | 1 | turquoise |
| ENSBTAG00000038815 | DIAPH2 | 1 | turquoise |
| ENSBTAG00000038910 | SECTM1 | 1 | turquoise |
| ENSBTAG00000038929 | BCAS4 | 1 | turquoise |
| ENSBTAG00000038938 | LOC781710 | 1 | turquoise |
| ENSBTAG00000038981 | LOC518134 | 1 | turquoise |
| ENSBTAG00000039012 | MFSD6L | 1 | turquoise |
| ENSBTAG00000039014 | DEF8 | 1 | turquoise |
| ENSBTAG00000039016 | NA | 1 | turquoise |
| ENSBTAG00000039037 | SERPINB4 | 1 | turquoise |
| ENSBTAG00000039046 | NA | 1 | turquoise |
| ENSBTAG00000039080 | SLC22A3 | 1 | turquoise |
| ENSBTAG00000039086 | NA | 1 | turquoise |
| ENSBTAG00000039122 | PLA2G5 | 1 | turquoise |
| ENSBTAG00000039160 | VAV1 | 1 | turquoise |
| ENSBTAG00000039197 | SLC2A9 | 1 | turquoise |
| ENSBTAG00000039275 | ERAP2 | 1 | turquoise |
| ENSBTAG00000039283 | NA | 1 | turquoise |
| ENSBTAG00000039310 | NA | 1 | turquoise |
| ENSBTAG00000039319 | CYP4F2 | 1 | turquoise |
| ENSBTAG00000039326 | LOC520336 | 1 | turquoise |
| ENSBTAG00000039335 | ARRDC2 | 1 | turquoise |
| ENSBTAG00000039340 | SCN4B | 1 | turquoise |
| ENSBTAG00000039353 | NA | 1 | turquoise |
| ENSBTAG00000039413 | NA | 1 | turquoise |
| ENSBTAG00000039442 | SEC62 | 1 | turquoise |
| ENSBTAG00000039446 | LOC100850808 | 1 | turquoise |
| ENSBTAG00000039486 | NA | 1 | turquoise |
| ENSBTAG00000039512 | NA | 1 | turquoise |
| ENSBTAG00000039520 | SIRPB1 | 1 | turquoise |
| ENSBTAG00000039524 | NA | 1 | turquoise |
| ENSBTAG00000039529 | BTBD19 | 1 | turquoise |
| ENSBTAG00000039634 | NA | 1 | turquoise |
| ENSBTAG00000039657 | H2AC6 | 1 | turquoise |
| ENSBTAG00000039691 | NA | 1 | turquoise |
| ENSBTAG00000039720 | TMEM225B | 1 | turquoise |
| ENSBTAG00000039728 | LOC100295775 | 1 | turquoise |
| ENSBTAG00000039764 | NA | 1 | turquoise |
| ENSBTAG00000039793 | HDGF | 1 | turquoise |
| ENSBTAG00000039812 | NA | 1 | turquoise |
| ENSBTAG00000039819 | RPH3AL | 1 | turquoise |
| ENSBTAG00000039856 | NA | 1 | turquoise |

| | | | |
|---|---|---|---|
| ENSBTAG00000039861 | OAS1Y | 1 | turquoise |
| ENSBTAG00000039892 | NA | 1 | turquoise |
| ENSBTAG00000039937 | NA | 1 | turquoise |
| ENSBTAG00000039950 | NA | 1 | turquoise |
| ENSBTAG00000039993 | CD1D | 1 | turquoise |
| ENSBTAG00000040006 | SRSF3 | 1 | turquoise |
| ENSBTAG00000040056 | LTB4R2 | 1 | turquoise |
| ENSBTAG00000040151 | GCH1 | 1 | turquoise |
| ENSBTAG00000040190 | CCDC88B | 1 | turquoise |
| ENSBTAG00000040242 | NA | 1 | turquoise |
| ENSBTAG00000040244 | APOL3 | 1 | turquoise |
| ENSBTAG00000040323 | NA | 1 | turquoise |
| ENSBTAG00000040350 | XIAP | 1 | turquoise |
| ENSBTAG00000040361 | LFNG | 1 | turquoise |
| ENSBTAG00000040392 | MGC139164 | 1 | turquoise |
| ENSBTAG00000040575 | KCTD5 | 1 | turquoise |
| ENSBTAG00000040580 | NA | 1 | turquoise |
| ENSBTAG00000040586 | NA | 1 | turquoise |
| ENSBTAG00000040595 | NA | 1 | turquoise |
| ENSBTAG00000042134 | NA | 1 | turquoise |
| ENSBTAG00000042135 | LOC112448925 | 1 | turquoise |
| ENSBTAG00000042180 | LOC112441892 | 1 | turquoise |
| ENSBTAG00000042217 | LOC112441903 | 1 | turquoise |
| ENSBTAG00000042258 | NA | 1 | turquoise |
| ENSBTAG00000042264 | LOC112444803 | 1 | turquoise |
| ENSBTAG00000042339 | LOC112441895 | 1 | turquoise |
| ENSBTAG00000042370 | NA | 1 | turquoise |
| ENSBTAG00000042475 | LOC112448304 | 1 | turquoise |
| ENSBTAG00000042553 | LOC112443669 | 1 | turquoise |
| ENSBTAG00000042616 | NA | 1 | turquoise |
| ENSBTAG00000042625 | LOC112444675 | 1 | turquoise |
| ENSBTAG00000042644 | LOC112449391 | 1 | turquoise |
| ENSBTAG00000042645 | LOC112446104 | 1 | turquoise |
| ENSBTAG00000042713 | LOC112443329 | 1 | turquoise |
| ENSBTAG00000042833 | LOC112448169 | 1 | turquoise |
| ENSBTAG00000042836 | LOC112442823 | 1 | turquoise |
| ENSBTAG00000042837 | LOC112444676 | 1 | turquoise |
| ENSBTAG00000042868 | LOC112442805 | 1 | turquoise |
| ENSBTAG00000042883 | LOC112442425 | 1 | turquoise |
| ENSBTAG00000042974 | LOC112441942 | 1 | turquoise |
| ENSBTAG00000042986 | LOC112443648 | 1 | turquoise |
| ENSBTAG00000042989 | LOC112443901 | 1 | turquoise |
| ENSBTAG00000043036 | LOC112449405 | 1 | turquoise |
| ENSBTAG00000043122 | LOC112441672 | 1 | turquoise |
| ENSBTAG00000043151 | LOC112443902 | 1 | turquoise |
| ENSBTAG00000043166 | LOC112443650 | 1 | turquoise |
| ENSBTAG00000043240 | LOC112442436 | 1 | turquoise |

| | | | |
|---|---|---|---|
| ENSBTAG00000043243 | LOC112449172 | 1 | turquoise |
| ENSBTAG00000043264 | LOC112449400 | 1 | turquoise |
| ENSBTAG00000043268 | LOC112441945 | 1 | turquoise |
| ENSBTAG00000043347 | NA | 1 | turquoise |
| ENSBTAG00000043350 | LOC112449403 | 1 | turquoise |
| ENSBTAG00000043369 | LOC112446907 | 1 | turquoise |
| ENSBTAG00000043376 | NA | 1 | turquoise |
| ENSBTAG00000043378 | LOC112441944 | 1 | turquoise |
| ENSBTAG00000043414 | NA | 1 | turquoise |
| ENSBTAG00000043432 | LOC112441894 | 1 | turquoise |
| ENSBTAG00000043453 | LOC112442485 | 1 | turquoise |
| ENSBTAG00000043462 | LOC112441682 | 1 | turquoise |
| ENSBTAG00000043480 | LOC112441683 | 1 | turquoise |
| ENSBTAG00000043486 | LOC112441934 | 1 | turquoise |
| ENSBTAG00000043530 | LOC112447190 | 1 | turquoise |
| ENSBTAG00000043553 | GPX3 | 1 | turquoise |
| ENSBTAG00000043602 | NA | 1 | turquoise |
| ENSBTAG00000043615 | LOC112447901 | 1 | turquoise |
| ENSBTAG00000043683 | NA | 1 | turquoise |
| ENSBTAG00000043706 | NA | 1 | turquoise |
| ENSBTAG00000043876 | LOC112446144 | 1 | turquoise |
| ENSBTAG00000043907 | NA | 1 | turquoise |
| ENSBTAG00000043964 | ARL5B | 1 | turquoise |
| ENSBTAG00000043971 | NOTCH3 | 1 | turquoise |
| ENSBTAG00000043994 | GRK6 | 1 | turquoise |
| ENSBTAG00000044010 | NA | 1 | turquoise |
| ENSBTAG00000044017 | MSRB3 | 1 | turquoise |
| ENSBTAG00000044059 | NA | 1 | turquoise |
| ENSBTAG00000044071 | HRH2 | 1 | turquoise |
| ENSBTAG00000044078 | TESC | 1 | turquoise |
| ENSBTAG00000044082 | MARCHF1 | 1 | turquoise |
| ENSBTAG00000044083 | LIMK1 | 1 | turquoise |
| ENSBTAG00000044097 | KLF7 | 1 | turquoise |
| ENSBTAG00000044099 | ZSWIM7 | 1 | turquoise |
| ENSBTAG00000044129 | ST6GALNAC6 | 1 | turquoise |
| ENSBTAG00000044158 | LDLRAD3 | 1 | turquoise |
| ENSBTAG00000044194 | ZDHHC2 | 1 | turquoise |
| ENSBTAG00000044197 | EDARADD | 1 | turquoise |
| ENSBTAG00000044284 | MIR2357 | 1 | turquoise |
| ENSBTAG00000044419 | NA | 1 | turquoise |
| ENSBTAG00000044526 | MIR1584 | 1 | turquoise |
| ENSBTAG00000044676 | LOC112446843 | 1 | turquoise |
| ENSBTAG00000044681 | NA | 1 | turquoise |
| ENSBTAG00000044741 | LOC112441677 | 1 | turquoise |
| ENSBTAG00000044830 | MIR339A | 1 | turquoise |
| ENSBTAG00000044856 | LOC112442840 | 1 | turquoise |
| ENSBTAG00000044861 | LOC112446116 | 1 | turquoise |

| | | | |
|---|---|---|---|
| ENSBTAG00000045039 | LOC112444215 | 1 | turquoise |
| ENSBTAG00000045156 | LOC112449177 | 1 | turquoise |
| ENSBTAG00000045172 | MIR2358 | 1 | turquoise |
| ENSBTAG00000045293 | LOC112442824 | 1 | turquoise |
| ENSBTAG00000045327 | LOC112444391 | 1 | turquoise |
| ENSBTAG00000045337 | MIR2376 | 1 | turquoise |
| ENSBTAG00000045449 | MIR2349 | 1 | turquoise |
| ENSBTAG00000045475 | NA | 1 | turquoise |
| ENSBTAG00000045509 | FAM217B | 1 | turquoise |
| ENSBTAG00000045515 | NA | 1 | turquoise |
| ENSBTAG00000045529 | NCR1 | 1 | turquoise |
| ENSBTAG00000045543 | NA | 1 | turquoise |
| ENSBTAG00000045548 | CRKL | 1 | turquoise |
| ENSBTAG00000045565 | NHSL2 | 1 | turquoise |
| ENSBTAG00000045588 | LOC100298356 | 1 | turquoise |
| ENSBTAG00000045592 | NA | 1 | turquoise |
| ENSBTAG00000045608 | NA | 1 | turquoise |
| ENSBTAG00000045609 | NA | 1 | turquoise |
| ENSBTAG00000045647 | NA | 1 | turquoise |
| ENSBTAG00000045673 | LOC613345 | 1 | turquoise |
| ENSBTAG00000045685 | NA | 1 | turquoise |
| ENSBTAG00000045702 | NA | 1 | turquoise |
| ENSBTAG00000045742 | NA | 1 | turquoise |
| ENSBTAG00000045744 | NKRF | 1 | turquoise |
| ENSBTAG00000045748 | RAF1 | 1 | turquoise |
| ENSBTAG00000045751 | LOC520016 | 1 | turquoise |
| ENSBTAG00000045767 | LAT2 | 1 | turquoise |
| ENSBTAG00000045788 | NA | 1 | turquoise |
| ENSBTAG00000045808 | IL17REL | 1 | turquoise |
| ENSBTAG00000045814 | NA | 1 | turquoise |
| ENSBTAG00000045822 | C15H11orf96 | 1 | turquoise |
| ENSBTAG00000045849 | KCNK13 | 1 | turquoise |
| ENSBTAG00000045854 | LOC100852077 | 1 | turquoise |
| ENSBTAG00000045861 | TARM1 | 1 | turquoise |
| ENSBTAG00000045877 | TSC22D3 | 1 | turquoise |
| ENSBTAG00000045880 | SIGLEC5 | 1 | turquoise |
| ENSBTAG00000045881 | MIR1260B | 1 | turquoise |
| ENSBTAG00000045885 | MIR3064 | 1 | turquoise |
| ENSBTAG00000045904 | PIM3 | 1 | turquoise |
| ENSBTAG00000045907 | NA | 1 | turquoise |
| ENSBTAG00000045914 | LOC100297725 | 1 | turquoise |
| ENSBTAG00000045929 | LOC512440 | 1 | turquoise |
| ENSBTAG00000045936 | NA | 1 | turquoise |
| ENSBTAG00000045948 | NA | 1 | turquoise |
| ENSBTAG00000045980 | ARF6 | 1 | turquoise |
| ENSBTAG00000046014 | GPR146 | 1 | turquoise |
| ENSBTAG00000046030 | NA | 1 | turquoise |

| | | | |
|---|---|---|---|
| ENSBTAG00000046041 | NA | 1 | turquoise |
| ENSBTAG00000046050 | NA | 1 | turquoise |
| ENSBTAG00000046063 | NA | 1 | turquoise |
| ENSBTAG00000046090 | LOC507247 | 1 | turquoise |
| ENSBTAG00000046117 | NA | 1 | turquoise |
| ENSBTAG00000046144 | NA | 1 | turquoise |
| ENSBTAG00000046152 | MGAM | 1 | turquoise |
| ENSBTAG00000046153 | NA | 1 | turquoise |
| ENSBTAG00000046158 | CFB | 1 | turquoise |
| ENSBTAG00000046188 | ELANE | 1 | turquoise |
| ENSBTAG00000046211 | NA | 1 | turquoise |
| ENSBTAG00000046235 | SRSF12 | 1 | turquoise |
| ENSBTAG00000046245 | NA | 1 | turquoise |
| ENSBTAG00000046266 | TNFSF9 | 1 | turquoise |
| ENSBTAG00000046288 | NA | 1 | turquoise |
| ENSBTAG00000046294 | NA | 1 | turquoise |
| ENSBTAG00000046307 | CEBPD | 1 | turquoise |
| ENSBTAG00000046324 | LOC100297676 | 1 | turquoise |
| ENSBTAG00000046343 | CCNJL | 1 | turquoise |
| ENSBTAG00000046356 | NA | 1 | turquoise |
| ENSBTAG00000046375 | LOC407163 | 1 | turquoise |
| ENSBTAG00000046383 | NA | 1 | turquoise |
| ENSBTAG00000046406 | R3HDM4 | 1 | turquoise |
| ENSBTAG00000046407 | NYX | 1 | turquoise |
| ENSBTAG00000046409 | EGR2 | 1 | turquoise |
| ENSBTAG00000046412 | NA | 1 | turquoise |
| ENSBTAG00000046419 | TPC3 | 1 | turquoise |
| ENSBTAG00000046431 | NA | 1 | turquoise |
| ENSBTAG00000046449 | NA | 1 | turquoise |
| ENSBTAG00000046450 | NA | 1 | turquoise |
| ENSBTAG00000046461 | NA | 1 | turquoise |
| ENSBTAG00000046484 | NA | 1 | turquoise |
| ENSBTAG00000046533 | RAB31 | 1 | turquoise |
| ENSBTAG00000046548 | ST6GALNAC4 | 1 | turquoise |
| ENSBTAG00000046566 | NA | 1 | turquoise |
| ENSBTAG00000046580 | DHX58 | 1 | turquoise |
| ENSBTAG00000046588 | DNAJB9 | 1 | turquoise |
| ENSBTAG00000046595 | NA | 1 | turquoise |
| ENSBTAG00000046607 | JMJD7 | 1 | turquoise |
| ENSBTAG00000046611 | NA | 1 | turquoise |
| ENSBTAG00000046612 | ZNF292 | 1 | turquoise |
| ENSBTAG00000046616 | NA | 1 | turquoise |
| ENSBTAG00000046622 | NA | 1 | turquoise |
| ENSBTAG00000046662 | TRPM5 | 1 | turquoise |
| ENSBTAG00000046672 | ZDHHC18 | 1 | turquoise |
| ENSBTAG00000046684 | FOXN3 | 1 | turquoise |
| ENSBTAG00000046690 | LOC107133338 | 1 | turquoise |

| | | | |
|---|---|---|---|
| ENSBTAG00000046693 | NA | 1 | turquoise |
| ENSBTAG00000046699 | STAT4 | 1 | turquoise |
| ENSBTAG00000046700 | NA | 1 | turquoise |
| ENSBTAG00000046701 | FRAT2 | 1 | turquoise |
| ENSBTAG00000046721 | NA | 1 | turquoise |
| ENSBTAG00000046724 | CBX6 | 1 | turquoise |
| ENSBTAG00000046750 | B3GNT3 | 1 | turquoise |
| ENSBTAG00000046765 | POLR2A | 1 | turquoise |
| ENSBTAG00000046773 | MCOLN2 | 1 | turquoise |
| ENSBTAG00000046841 | IRF2BP2 | 1 | turquoise |
| ENSBTAG00000046862 | MARCKSL1 | 1 | turquoise |
| ENSBTAG00000046885 | NA | 1 | turquoise |
| ENSBTAG00000046900 | GGT1 | 1 | turquoise |
| ENSBTAG00000046939 | NA | 1 | turquoise |
| ENSBTAG00000046941 | LOC786015 | 1 | turquoise |
| ENSBTAG00000046956 | STYX | 1 | turquoise |
| ENSBTAG00000046981 | NA | 1 | turquoise |
| ENSBTAG00000047030 | LOC515276 | 1 | turquoise |
| ENSBTAG00000047055 | NA | 1 | turquoise |
| ENSBTAG00000047059 | TIMM17A | 1 | turquoise |
| ENSBTAG00000047077 | ENTPD7 | 1 | turquoise |
| ENSBTAG00000047088 | NA | 1 | turquoise |
| ENSBTAG00000047093 | NA | 1 | turquoise |
| ENSBTAG00000047107 | TNIP3 | 1 | turquoise |
| ENSBTAG00000047128 | NA | 1 | turquoise |
| ENSBTAG00000047133 | NA | 1 | turquoise |
| ENSBTAG00000047158 | LRFN1 | 1 | turquoise |
| ENSBTAG00000047166 | SH2D7 | 1 | turquoise |
| ENSBTAG00000047200 | NA | 1 | turquoise |
| ENSBTAG00000047206 | H1-4 | 1 | turquoise |
| ENSBTAG00000047213 | GPRASP1 | 1 | turquoise |
| ENSBTAG00000047238 | ITGAM | 1 | turquoise |
| ENSBTAG00000047275 | NA | 1 | turquoise |
| ENSBTAG00000047293 | NRIP1 | 1 | turquoise |
| ENSBTAG00000047336 | LOC100847115 | 1 | turquoise |
| ENSBTAG00000047338 | DCBLD1 | 1 | turquoise |
| ENSBTAG00000047339 | BCOR | 1 | turquoise |
| ENSBTAG00000047341 | LOC100299503 | 1 | turquoise |
| ENSBTAG00000047345 | NA | 1 | turquoise |
| ENSBTAG00000047367 | CMTR2 | 1 | turquoise |
| ENSBTAG00000047379 | NA | 1 | turquoise |
| ENSBTAG00000047383 | LOC784305 | 1 | turquoise |
| ENSBTAG00000047389 | NA | 1 | turquoise |
| ENSBTAG00000047424 | TRIP10 | 1 | turquoise |
| ENSBTAG00000047432 | LOC781224 | 1 | turquoise |
| ENSBTAG00000047448 | C25H16orf54 | 1 | turquoise |
| ENSBTAG00000047473 | LOC100301263 | 1 | turquoise |

| | | | |
|---|---|---|---|
| ENSBTAG00000047488 | KCNK9 | 1 | turquoise |
| ENSBTAG00000047495 | CD81 | 1 | turquoise |
| ENSBTAG00000047502 | FKBP5 | 1 | turquoise |
| ENSBTAG00000047520 | MPV17L | 1 | turquoise |
| ENSBTAG00000047534 | NA | 1 | turquoise |
| ENSBTAG00000047538 | RIMBP3B | 1 | turquoise |
| ENSBTAG00000047561 | NA | 1 | turquoise |
| ENSBTAG00000047570 | NA | 1 | turquoise |
| ENSBTAG00000047575 | NA | 1 | turquoise |
| ENSBTAG00000047604 | HSD17B1 | 1 | turquoise |
| ENSBTAG00000047605 | LOC100337457 | 1 | turquoise |
| ENSBTAG00000047654 | NA | 1 | turquoise |
| ENSBTAG00000047659 | NA | 1 | turquoise |
| ENSBTAG00000047676 | PRODH | 1 | turquoise |
| ENSBTAG00000047680 | IRF7 | 1 | turquoise |
| ENSBTAG00000047703 | NA | 1 | turquoise |
| ENSBTAG00000047710 | NA | 1 | turquoise |
| ENSBTAG00000047740 | NA | 1 | turquoise |
| ENSBTAG00000047766 | NA | 1 | turquoise |
| ENSBTAG00000047776 | NA | 1 | turquoise |
| ENSBTAG00000047801 | CRELD2 | 1 | turquoise |
| ENSBTAG00000047805 | NA | 1 | turquoise |
| ENSBTAG00000047839 | P2RY8 | 1 | turquoise |
| ENSBTAG00000047871 | KLF17 | 1 | turquoise |
| ENSBTAG00000047875 | NA | 1 | turquoise |
| ENSBTAG00000047904 | LOC527744 | 1 | turquoise |
| ENSBTAG00000047914 | CDHR5 | 1 | turquoise |
| ENSBTAG00000047926 | OVCA2 | 1 | turquoise |
| ENSBTAG00000047942 | DESI1 | 1 | turquoise |
| ENSBTAG00000047953 | NLRP2 | 1 | turquoise |
| ENSBTAG00000047972 | INAVA | 1 | turquoise |
| ENSBTAG00000047988 | NA | 1 | turquoise |
| ENSBTAG00000048004 | NA | 1 | turquoise |
| ENSBTAG00000048011 | GPR160 | 1 | turquoise |
| ENSBTAG00000048023 | NA | 1 | turquoise |
| ENSBTAG00000048029 | MMP1 | 1 | turquoise |
| ENSBTAG00000048062 | KDM6B | 1 | turquoise |
| ENSBTAG00000048107 | CANX | 1 | turquoise |
| ENSBTAG00000048122 | CFD | 1 | turquoise |
| ENSBTAG00000048137 | NA | 1 | turquoise |
| ENSBTAG00000048145 | NA | 1 | turquoise |
| ENSBTAG00000048154 | NA | 1 | turquoise |
| ENSBTAG00000048155 | FCN1 | 1 | turquoise |
| ENSBTAG00000048184 | NA | 1 | turquoise |
| ENSBTAG00000048226 | NA | 1 | turquoise |
| ENSBTAG00000048229 | NA | 1 | turquoise |
| ENSBTAG00000048249 | NA | 1 | turquoise |

| | | | |
|---|---|---|---|
| ENSBTAG00000048272 | NA | 1 | turquoise |
| ENSBTAG00000048273 | C6H4orf3 | 1 | turquoise |
| ENSBTAG00000048306 | NA | 1 | turquoise |
| ENSBTAG00000048314 | NA | 1 | turquoise |
| ENSBTAG00000000020 | TRPV3 | 4 | yellow |
| ENSBTAG00000000224 | TSPAN15 | 4 | yellow |
| ENSBTAG00000000251 | ALAD | 4 | yellow |
| ENSBTAG00000000281 | MND1 | 4 | yellow |
| ENSBTAG00000000329 | MEGF10 | 4 | yellow |
| ENSBTAG00000000357 | PRAG1 | 4 | yellow |
| ENSBTAG00000000442 | RBP4 | 4 | yellow |
| ENSBTAG00000000566 | CCDC14 | 4 | yellow |
| ENSBTAG00000000586 | CPLANE1 | 4 | yellow |
| ENSBTAG00000000590 | POLE | 4 | yellow |
| ENSBTAG00000000638 | CDT1 | 4 | yellow |
| ENSBTAG00000000660 | FAM83D | 4 | yellow |
| ENSBTAG00000000717 | PLPP2 | 4 | yellow |
| ENSBTAG00000000752 | SGO1 | 4 | yellow |
| ENSBTAG00000000770 | PGM2L1 | 4 | yellow |
| ENSBTAG00000000802 | LYVE1 | 4 | yellow |
| ENSBTAG00000000804 | MOB3B | 4 | yellow |
| ENSBTAG00000000824 | TSEN54 | 4 | yellow |
| ENSBTAG00000000825 | MRPS26 | 4 | yellow |
| ENSBTAG00000000853 | NA | 4 | yellow |
| ENSBTAG00000000898 | F2RL2 | 4 | yellow |
| ENSBTAG00000000899 | TMEM17 | 4 | yellow |
| ENSBTAG00000000986 | USH1C | 4 | yellow |
| ENSBTAG00000001027 | SERPINH1 | 4 | yellow |
| ENSBTAG00000001068 | ZCWPW1 | 4 | yellow |
| ENSBTAG00000001094 | MTUS2 | 4 | yellow |
| ENSBTAG00000001101 | IFNLR1 | 4 | yellow |
| ENSBTAG00000001138 | NA | 4 | yellow |
| ENSBTAG00000001174 | MRPL15 | 4 | yellow |
| ENSBTAG00000001231 | CDHR1 | 4 | yellow |
| ENSBTAG00000001235 | CD200R1L | 4 | yellow |
| ENSBTAG00000001298 | STAMBPL1 | 4 | yellow |
| ENSBTAG00000001343 | DEPDC1 | 4 | yellow |
| ENSBTAG00000001385 | ZSCAN2 | 4 | yellow |
| ENSBTAG00000001471 | POC1A | 4 | yellow |
| ENSBTAG00000001500 | FIGNL1 | 4 | yellow |
| ENSBTAG00000001564 | PDE4DIP | 4 | yellow |
| ENSBTAG00000001604 | NA | 4 | yellow |
| ENSBTAG00000001626 | CTPS1 | 4 | yellow |
| ENSBTAG00000001631 | KIFC1 | 4 | yellow |
| ENSBTAG00000001635 | CUTA | 4 | yellow |
| ENSBTAG00000001694 | TYRO3 | 4 | yellow |
| ENSBTAG00000001717 | AURKB | 4 | yellow |

| | | | |
|---|---|---|---|
| ENSBTAG00000001774 | SPRY2 | 4 | yellow |
| ENSBTAG00000001828 | SCRN2 | 4 | yellow |
| ENSBTAG00000001838 | LRRC36 | 4 | yellow |
| ENSBTAG00000001842 | GSTM3 | 4 | yellow |
| ENSBTAG00000001872 | ORC6 | 4 | yellow |
| ENSBTAG00000001920 | POLQ | 4 | yellow |
| ENSBTAG00000002144 | ADRB2 | 4 | yellow |
| ENSBTAG00000002224 | UHRF1 | 4 | yellow |
| ENSBTAG00000002278 | FBN1 | 4 | yellow |
| ENSBTAG00000002292 | SMARCA1 | 4 | yellow |
| ENSBTAG00000002298 | CKAP2L | 4 | yellow |
| ENSBTAG00000002311 | REEP6 | 4 | yellow |
| ENSBTAG00000002329 | ASAP2 | 4 | yellow |
| ENSBTAG00000002331 | DLGAP5 | 4 | yellow |
| ENSBTAG00000002417 | RAB30 | 4 | yellow |
| ENSBTAG00000002418 | TICRR | 4 | yellow |
| ENSBTAG00000002444 | MKI67 | 4 | yellow |
| ENSBTAG00000002451 | TTC39A | 4 | yellow |
| ENSBTAG00000002522 | SYT5 | 4 | yellow |
| ENSBTAG00000002576 | GLDN | 4 | yellow |
| ENSBTAG00000002613 | MIS18BP1 | 4 | yellow |
| ENSBTAG00000002620 | DNA2 | 4 | yellow |
| ENSBTAG00000002626 | ARHGAP6 | 4 | yellow |
| ENSBTAG00000002719 | ORC1 | 4 | yellow |
| ENSBTAG00000002742 | MSH2 | 4 | yellow |
| ENSBTAG00000002756 | CDCA2 | 4 | yellow |
| ENSBTAG00000002781 | NA | 4 | yellow |
| ENSBTAG00000002826 | CLSPN | 4 | yellow |
| ENSBTAG00000002858 | ACBD7 | 4 | yellow |
| ENSBTAG00000002910 | ECHDC2 | 4 | yellow |
| ENSBTAG00000002964 | TXLNB | 4 | yellow |
| ENSBTAG00000002981 | PIMREG | 4 | yellow |
| ENSBTAG00000003140 | CRYGS | 4 | yellow |
| ENSBTAG00000003144 | KIAA0825 | 4 | yellow |
| ENSBTAG00000003166 | AXL | 4 | yellow |
| ENSBTAG00000003192 | TBC1D16 | 4 | yellow |
| ENSBTAG00000003212 | NNAT | 4 | yellow |
| ENSBTAG00000003256 | TOMM34 | 4 | yellow |
| ENSBTAG00000003290 | SLC9A7 | 4 | yellow |
| ENSBTAG00000003403 | PADI2 | 4 | yellow |
| ENSBTAG00000003458 | CDCA7 | 4 | yellow |
| ENSBTAG00000003559 | PPP1R13B | 4 | yellow |
| ENSBTAG00000003580 | PSD | 4 | yellow |
| ENSBTAG00000003661 | SLC35F2 | 4 | yellow |
| ENSBTAG00000003669 | BNC2 | 4 | yellow |
| ENSBTAG00000003733 | TM4SF5 | 4 | yellow |
| ENSBTAG00000003773 | SLC25A13 | 4 | yellow |

| | | | |
|---|---|---|---|
| ENSBTAG00000003808 | HDAC9 | 4 | yellow |
| ENSBTAG00000003950 | PROC | 4 | yellow |
| ENSBTAG00000003955 | MYO7A | 4 | yellow |
| ENSBTAG00000003959 | ARHGAP24 | 4 | yellow |
| ENSBTAG00000003966 | DUSP3 | 4 | yellow |
| ENSBTAG00000003986 | CXXC5 | 4 | yellow |
| ENSBTAG00000004041 | ANKRD10 | 4 | yellow |
| ENSBTAG00000004073 | MTA3 | 4 | yellow |
| ENSBTAG00000004085 | ASF1B | 4 | yellow |
| ENSBTAG00000004112 | GAMT | 4 | yellow |
| ENSBTAG00000004222 | SAT2 | 4 | yellow |
| ENSBTAG00000004272 | ISG12(B) | 4 | yellow |
| ENSBTAG00000004275 | DKK3 | 4 | yellow |
| ENSBTAG00000004277 | EVC2 | 4 | yellow |
| ENSBTAG00000004280 | SRPX | 4 | yellow |
| ENSBTAG00000004286 | CDC45 | 4 | yellow |
| ENSBTAG00000004287 | EVC | 4 | yellow |
| ENSBTAG00000004347 | ADGRF5 | 4 | yellow |
| ENSBTAG00000004358 | DARS2 | 4 | yellow |
| ENSBTAG00000004413 | RHOBTB3 | 4 | yellow |
| ENSBTAG00000004565 | NA | 4 | yellow |
| ENSBTAG00000004685 | SRGAP3 | 4 | yellow |
| ENSBTAG00000004851 | NCKAP5L | 4 | yellow |
| ENSBTAG00000004903 | KRT15 | 4 | yellow |
| ENSBTAG00000004906 | CCNE2 | 4 | yellow |
| ENSBTAG00000004931 | POLE2 | 4 | yellow |
| ENSBTAG00000004943 | CCNA2 | 4 | yellow |
| ENSBTAG00000004976 | CDCA7L | 4 | yellow |
| ENSBTAG00000004997 | CTNNAL1 | 4 | yellow |
| ENSBTAG00000005010 | MXD3 | 4 | yellow |
| ENSBTAG00000005028 | HIRIP3 | 4 | yellow |
| ENSBTAG00000005031 | DOC2A | 4 | yellow |
| ENSBTAG00000005034 | CAPN5 | 4 | yellow |
| ENSBTAG00000005104 | MGAT5B | 4 | yellow |
| ENSBTAG00000005105 | PAFAH2 | 4 | yellow |
| ENSBTAG00000005129 | CEP55 | 4 | yellow |
| ENSBTAG00000005150 | HMGB3 | 4 | yellow |
| ENSBTAG00000005161 | ATP8B2 | 4 | yellow |
| ENSBTAG00000005205 | DSE | 4 | yellow |
| ENSBTAG00000005250 | BGN | 4 | yellow |
| ENSBTAG00000005269 | CCNB2 | 4 | yellow |
| ENSBTAG00000005293 | CDC25C | 4 | yellow |
| ENSBTAG00000005294 | NA | 4 | yellow |
| ENSBTAG00000005371 | DPAGT1 | 4 | yellow |
| ENSBTAG00000005385 | POP5 | 4 | yellow |
| ENSBTAG00000005424 | KCNC1 | 4 | yellow |
| ENSBTAG00000005456 | TTK | 4 | yellow |

| | | | |
|---|---|---|---|
| ENSBTAG00000005536 | MEA1 | 4 | yellow |
| ENSBTAG00000005557 | AFDN | 4 | yellow |
| ENSBTAG00000005560 | ST18 | 4 | yellow |
| ENSBTAG00000005607 | ERCC6L | 4 | yellow |
| ENSBTAG00000005704 | IL1RL2 | 4 | yellow |
| ENSBTAG00000005754 | PPM1K | 4 | yellow |
| ENSBTAG00000005762 | LYNX1 | 4 | yellow |
| ENSBTAG00000005825 | NEIL3 | 4 | yellow |
| ENSBTAG00000005838 | ULK3 | 4 | yellow |
| ENSBTAG00000005843 | REXO2 | 4 | yellow |
| ENSBTAG00000005846 | FAM219B | 4 | yellow |
| ENSBTAG00000005979 | HELLS | 4 | yellow |
| ENSBTAG00000006133 | NA | 4 | yellow |
| ENSBTAG00000006158 | DZIP3 | 4 | yellow |
| ENSBTAG00000006185 | SPC24 | 4 | yellow |
| ENSBTAG00000006212 | GTF2IRD1 | 4 | yellow |
| ENSBTAG00000006253 | FLNC | 4 | yellow |
| ENSBTAG00000006261 | GPR17 | 4 | yellow |
| ENSBTAG00000006262 | LIMS2 | 4 | yellow |
| ENSBTAG00000006373 | NA | 4 | yellow |
| ENSBTAG00000006374 | NHP2 | 4 | yellow |
| ENSBTAG00000006404 | CENPT | 4 | yellow |
| ENSBTAG00000006551 | ESCO2 | 4 | yellow |
| ENSBTAG00000006579 | P4HA3 | 4 | yellow |
| ENSBTAG00000006591 | NA | 4 | yellow |
| ENSBTAG00000006619 | MATK | 4 | yellow |
| ENSBTAG00000006738 | GPR68 | 4 | yellow |
| ENSBTAG00000006758 | NA | 4 | yellow |
| ENSBTAG00000006770 | MTBP | 4 | yellow |
| ENSBTAG00000006835 | MCAM | 4 | yellow |
| ENSBTAG00000006882 | IQGAP3 | 4 | yellow |
| ENSBTAG00000006948 | GARNL3 | 4 | yellow |
| ENSBTAG00000006963 | RPL12 | 4 | yellow |
| ENSBTAG00000006972 | TRIP13 | 4 | yellow |
| ENSBTAG00000007003 | TYMS | 4 | yellow |
| ENSBTAG00000007093 | NA | 4 | yellow |
| ENSBTAG00000007149 | NYNRIN | 4 | yellow |
| ENSBTAG00000007153 | C1QA | 4 | yellow |
| ENSBTAG00000007206 | STRIP2 | 4 | yellow |
| ENSBTAG00000007237 | BUB1B | 4 | yellow |
| ENSBTAG00000007247 | NUF2 | 4 | yellow |
| ENSBTAG00000007253 | TSPAN33 | 4 | yellow |
| ENSBTAG00000007302 | SLC2A5 | 4 | yellow |
| ENSBTAG00000007306 | RAB3C | 4 | yellow |
| ENSBTAG00000007352 | KCNC4 | 4 | yellow |
| ENSBTAG00000007569 | TNFRSF11A | 4 | yellow |
| ENSBTAG00000007596 | GEM | 4 | yellow |

| | | | |
|---|---|---|---|
| ENSBTAG00000007615 | CNTN3 | 4 | yellow |
| ENSBTAG00000007644 | GNG7 | 4 | yellow |
| ENSBTAG00000007652 | RFTN2 | 4 | yellow |
| ENSBTAG00000007681 | DHFR | 4 | yellow |
| ENSBTAG00000007702 | RPP38 | 4 | yellow |
| ENSBTAG00000007780 | CAMK1 | 4 | yellow |
| ENSBTAG00000007783 | MYBL2 | 4 | yellow |
| ENSBTAG00000007791 | KCNQ5 | 4 | yellow |
| ENSBTAG00000007799 | MTFR2 | 4 | yellow |
| ENSBTAG00000007837 | SELENOI | 4 | yellow |
| ENSBTAG00000007850 | ITIH4 | 4 | yellow |
| ENSBTAG00000007860 | ASPM | 4 | yellow |
| ENSBTAG00000007888 | EFR3B | 4 | yellow |
| ENSBTAG00000007898 | CYBRD1 | 4 | yellow |
| ENSBTAG00000008026 | OXT | 4 | yellow |
| ENSBTAG00000008078 | TRIM59 | 4 | yellow |
| ENSBTAG00000008088 | KIF18B | 4 | yellow |
| ENSBTAG00000008091 | SELENBP1 | 4 | yellow |
| ENSBTAG00000008098 | DCLK2 | 4 | yellow |
| ENSBTAG00000008147 | MICAL1 | 4 | yellow |
| ENSBTAG00000008150 | PKIA | 4 | yellow |
| ENSBTAG00000008181 | CHAF1A | 4 | yellow |
| ENSBTAG00000008193 | NA | 4 | yellow |
| ENSBTAG00000008216 | RRM2 | 4 | yellow |
| ENSBTAG00000008248 | DMD | 4 | yellow |
| ENSBTAG00000008300 | FN1 | 4 | yellow |
| ENSBTAG00000008468 | NA | 4 | yellow |
| ENSBTAG00000008499 | TROAP | 4 | yellow |
| ENSBTAG00000008554 | NINJ2 | 4 | yellow |
| ENSBTAG00000008585 | ARHGEF10 | 4 | yellow |
| ENSBTAG00000008625 | PLEKHF1 | 4 | yellow |
| ENSBTAG00000008758 | KIF20A | 4 | yellow |
| ENSBTAG00000008771 | MYEF2 | 4 | yellow |
| ENSBTAG00000008772 | SMC2 | 4 | yellow |
| ENSBTAG00000008778 | CHST13 | 4 | yellow |
| ENSBTAG00000008868 | CAPN3 | 4 | yellow |
| ENSBTAG00000008934 | ESPL1 | 4 | yellow |
| ENSBTAG00000008947 | TKTL2 | 4 | yellow |
| ENSBTAG00000008963 | CIT | 4 | yellow |
| ENSBTAG00000008966 | TSPAN7 | 4 | yellow |
| ENSBTAG00000009005 | NA | 4 | yellow |
| ENSBTAG00000009035 | CENPE | 4 | yellow |
| ENSBTAG00000009046 | TBX6 | 4 | yellow |
| ENSBTAG00000009218 | ANLN | 4 | yellow |
| ENSBTAG00000009256 | ITGA4 | 4 | yellow |
| ENSBTAG00000009257 | CERKL | 4 | yellow |
| ENSBTAG00000009331 | CPAMD8 | 4 | yellow |

| | | | |
|---|---|---|---|
| ENSBTAG00000009351 | ATXN10 | 4 | yellow |
| ENSBTAG00000009383 | KIF11 | 4 | yellow |
| ENSBTAG00000009396 | EXO1 | 4 | yellow |
| ENSBTAG00000009455 | IL12RB2 | 4 | yellow |
| ENSBTAG00000009568 | KANK2 | 4 | yellow |
| ENSBTAG00000009586 | CDC25A | 4 | yellow |
| ENSBTAG00000009618 | NEK2 | 4 | yellow |
| ENSBTAG00000009654 | LPAR1 | 4 | yellow |
| ENSBTAG00000009714 | ARHGAP33 | 4 | yellow |
| ENSBTAG00000009737 | LTC4S | 4 | yellow |
| ENSBTAG00000009780 | GTF2I | 4 | yellow |
| ENSBTAG00000009803 | SNCB | 4 | yellow |
| ENSBTAG00000009819 | CDC20 | 4 | yellow |
| ENSBTAG00000009839 | GSTK1 | 4 | yellow |
| ENSBTAG00000009852 | NAV3 | 4 | yellow |
| ENSBTAG00000009983 | KIF23 | 4 | yellow |
| ENSBTAG00000010077 | FANCD2 | 4 | yellow |
| ENSBTAG00000010091 | ARL6 | 4 | yellow |
| ENSBTAG00000010106 | CCND3 | 4 | yellow |
| ENSBTAG00000010109 | CDK1 | 4 | yellow |
| ENSBTAG00000010123 | APOE | 4 | yellow |
| ENSBTAG00000010158 | NA | 4 | yellow |
| ENSBTAG00000010175 | COL8A2 | 4 | yellow |
| ENSBTAG00000010179 | COL5A3 | 4 | yellow |
| ENSBTAG00000010276 | RECQL4 | 4 | yellow |
| ENSBTAG00000010339 | ABHD11 | 4 | yellow |
| ENSBTAG00000010345 | XPNPEP2 | 4 | yellow |
| ENSBTAG00000010384 | CDC6 | 4 | yellow |
| ENSBTAG00000010419 | CYP2R1 | 4 | yellow |
| ENSBTAG00000010457 | NUAK1 | 4 | yellow |
| ENSBTAG00000010542 | SPIRE1 | 4 | yellow |
| ENSBTAG00000010564 | ELOVL6 | 4 | yellow |
| ENSBTAG00000010578 | IL17B | 4 | yellow |
| ENSBTAG00000010626 | SH3GLB2 | 4 | yellow |
| ENSBTAG00000010721 | MCM3 | 4 | yellow |
| ENSBTAG00000010793 | CCDC80 | 4 | yellow |
| ENSBTAG00000010989 | PIK3R1 | 4 | yellow |
| ENSBTAG00000010991 | TTR | 4 | yellow |
| ENSBTAG00000011051 | NA | 4 | yellow |
| ENSBTAG00000011056 | IDS | 4 | yellow |
| ENSBTAG00000011059 | CDKN2C | 4 | yellow |
| ENSBTAG00000011083 | POLD1 | 4 | yellow |
| ENSBTAG00000011115 | NA | 4 | yellow |
| ENSBTAG00000011121 | CLCN4 | 4 | yellow |
| ENSBTAG00000011196 | C1QB | 4 | yellow |
| ENSBTAG00000011209 | SYT11 | 4 | yellow |
| ENSBTAG00000011284 | SLC39A1 | 4 | yellow |

| | | | |
|---|---|---|---|
| ENSBTAG00000011328 | NA | 4 | yellow |
| ENSBTAG00000011338 | NREP | 4 | yellow |
| ENSBTAG00000011354 | NUDT9 | 4 | yellow |
| ENSBTAG00000011405 | CEP72 | 4 | yellow |
| ENSBTAG00000011412 | LAMB1 | 4 | yellow |
| ENSBTAG00000011416 | ARHGEF39 | 4 | yellow |
| ENSBTAG00000011434 | NPR2 | 4 | yellow |
| ENSBTAG00000011442 | SPAG8 | 4 | yellow |
| ENSBTAG00000011454 | FKBP10 | 4 | yellow |
| ENSBTAG00000011584 | CHST2 | 4 | yellow |
| ENSBTAG00000011635 | CENPN | 4 | yellow |
| ENSBTAG00000011651 | HECTD2 | 4 | yellow |
| ENSBTAG00000011802 | COL6A1 | 4 | yellow |
| ENSBTAG00000011818 | COL26A1 | 4 | yellow |
| ENSBTAG00000011873 | KCNE3 | 4 | yellow |
| ENSBTAG00000011934 | PCK2 | 4 | yellow |
| ENSBTAG00000011998 | DUT | 4 | yellow |
| ENSBTAG00000012061 | POU3F1 | 4 | yellow |
| ENSBTAG00000012073 | VOPP1 | 4 | yellow |
| ENSBTAG00000012184 | PTTG1 | 4 | yellow |
| ENSBTAG00000012186 | DKKL1 | 4 | yellow |
| ENSBTAG00000012219 | CSPG4 | 4 | yellow |
| ENSBTAG00000012251 | MTR | 4 | yellow |
| ENSBTAG00000012260 | MTRF1 | 4 | yellow |
| ENSBTAG00000012342 | LIMA1 | 4 | yellow |
| ENSBTAG00000012409 | POSTN | 4 | yellow |
| ENSBTAG00000012443 | DIAPH3 | 4 | yellow |
| ENSBTAG00000012496 | MTMR11 | 4 | yellow |
| ENSBTAG00000012507 | PDZD3 | 4 | yellow |
| ENSBTAG00000012736 | PTRH1 | 4 | yellow |
| ENSBTAG00000012742 | SLC22A18 | 4 | yellow |
| ENSBTAG00000012834 | ARSI | 4 | yellow |
| ENSBTAG00000012849 | COL4A1 | 4 | yellow |
| ENSBTAG00000012855 | LPL | 4 | yellow |
| ENSBTAG00000012861 | KIF4A | 4 | yellow |
| ENSBTAG00000012921 | KIF24 | 4 | yellow |
| ENSBTAG00000012925 | NCAPH | 4 | yellow |
| ENSBTAG00000012940 | TMEM25 | 4 | yellow |
| ENSBTAG00000012966 | SCRN3 | 4 | yellow |
| ENSBTAG00000013008 | PMCH | 4 | yellow |
| ENSBTAG00000013009 | AURKA | 4 | yellow |
| ENSBTAG00000013036 | YDJC | 4 | yellow |
| ENSBTAG00000013054 | MFSD2A | 4 | yellow |
| ENSBTAG00000013070 | EAF2 | 4 | yellow |
| ENSBTAG00000013071 | GPR153 | 4 | yellow |
| ENSBTAG00000013078 | LOC104968411 | 4 | yellow |
| ENSBTAG00000013100 | SPAG5 | 4 | yellow |

| | | | |
|---|---|---|---|
| ENSBTAG00000013116 | PLCB4 | 4 | yellow |
| ENSBTAG00000013141 | ACTRT3 | 4 | yellow |
| ENSBTAG00000013185 | TIMD4 | 4 | yellow |
| ENSBTAG00000013274 | CENPV | 4 | yellow |
| ENSBTAG00000013278 | MAGIX | 4 | yellow |
| ENSBTAG00000013337 | DHDH | 4 | yellow |
| ENSBTAG00000013392 | PLD2 | 4 | yellow |
| ENSBTAG00000013429 | CLEC4G | 4 | yellow |
| ENSBTAG00000013492 | PRKAG3 | 4 | yellow |
| ENSBTAG00000013562 | SKP2 | 4 | yellow |
| ENSBTAG00000013573 | BIRC5 | 4 | yellow |
| ENSBTAG00000013588 | ZNF532 | 4 | yellow |
| ENSBTAG00000013663 | CMSS1 | 4 | yellow |
| ENSBTAG00000013669 | KIF22 | 4 | yellow |
| ENSBTAG00000013676 | DYNC2LI1 | 4 | yellow |
| ENSBTAG00000013735 | TBCE | 4 | yellow |
| ENSBTAG00000013750 | B3GALNT2 | 4 | yellow |
| ENSBTAG00000013761 | STMN1 | 4 | yellow |
| ENSBTAG00000013825 | BCAT1 | 4 | yellow |
| ENSBTAG00000013923 | QPCT | 4 | yellow |
| ENSBTAG00000013982 | UACA | 4 | yellow |
| ENSBTAG00000014001 | MAP1A | 4 | yellow |
| ENSBTAG00000014088 | MBNL3 | 4 | yellow |
| ENSBTAG00000014103 | SH3GL2 | 4 | yellow |
| ENSBTAG00000014194 | ADARB2 | 4 | yellow |
| ENSBTAG00000014239 | CCNB1 | 4 | yellow |
| ENSBTAG00000014247 | FAM83F | 4 | yellow |
| ENSBTAG00000014250 | ZC3HAV1L | 4 | yellow |
| ENSBTAG00000014252 | SPTLC3 | 4 | yellow |
| ENSBTAG00000014326 | CDCA8 | 4 | yellow |
| ENSBTAG00000014380 | MCM2 | 4 | yellow |
| ENSBTAG00000014434 | CCHCR1 | 4 | yellow |
| ENSBTAG00000014435 | TCF19 | 4 | yellow |
| ENSBTAG00000014453 | PLK1 | 4 | yellow |
| ENSBTAG00000014472 | LCMT2 | 4 | yellow |
| ENSBTAG00000014494 | RNASET2 | 4 | yellow |
| ENSBTAG00000014498 | UNC93A | 4 | yellow |
| ENSBTAG00000014567 | MYLK | 4 | yellow |
| ENSBTAG00000014615 | SLC26A2 | 4 | yellow |
| ENSBTAG00000014713 | RARRES1 | 4 | yellow |
| ENSBTAG00000014855 | MICAL2 | 4 | yellow |
| ENSBTAG00000014956 | JSRP1 | 4 | yellow |
| ENSBTAG00000015014 | CENPO | 4 | yellow |
| ENSBTAG00000015074 | PTGDS | 4 | yellow |
| ENSBTAG00000015098 | RABEPK | 4 | yellow |
| ENSBTAG00000015129 | KLK10 | 4 | yellow |
| ENSBTAG00000015157 | GRAP2 | 4 | yellow |

| | | | |
|---|---|---|---|
| ENSBTAG00000015224 | TCOF1 | 4 | yellow |
| ENSBTAG00000015235 | HMCN1 | 4 | yellow |
| ENSBTAG00000015280 | KIF2C | 4 | yellow |
| ENSBTAG00000015293 | NXPH3 | 4 | yellow |
| ENSBTAG00000015337 | TSPAN12 | 4 | yellow |
| ENSBTAG00000015395 | NA | 4 | yellow |
| ENSBTAG00000015402 | GREB1 | 4 | yellow |
| ENSBTAG00000015408 | DTYMK | 4 | yellow |
| ENSBTAG00000015426 | PDLIM4 | 4 | yellow |
| ENSBTAG00000015449 | PPEF2 | 4 | yellow |
| ENSBTAG00000015534 | LYPLAL1 | 4 | yellow |
| ENSBTAG00000015546 | RIOX2 | 4 | yellow |
| ENSBTAG00000015669 | ADAT1 | 4 | yellow |
| ENSBTAG00000015804 | SIGMAR1 | 4 | yellow |
| ENSBTAG00000015875 | FOXM1 | 4 | yellow |
| ENSBTAG00000015981 | ETV1 | 4 | yellow |
| ENSBTAG00000016007 | ADH5 | 4 | yellow |
| ENSBTAG00000016072 | SHC2 | 4 | yellow |
| ENSBTAG00000016147 | CETN4 | 4 | yellow |
| ENSBTAG00000016240 | RNF157 | 4 | yellow |
| ENSBTAG00000016255 | PLEK2 | 4 | yellow |
| ENSBTAG00000016406 | MCM10 | 4 | yellow |
| ENSBTAG00000016467 | SLC22A17 | 4 | yellow |
| ENSBTAG00000016472 | NA | 4 | yellow |
| ENSBTAG00000016512 | TTC21B | 4 | yellow |
| ENSBTAG00000016619 | MIS18A | 4 | yellow |
| ENSBTAG00000016620 | SIGLEC15 | 4 | yellow |
| ENSBTAG00000016704 | SLC37A2 | 4 | yellow |
| ENSBTAG00000016717 | CEP68 | 4 | yellow |
| ENSBTAG00000016726 | KIF15 | 4 | yellow |
| ENSBTAG00000016746 | UBE2C | 4 | yellow |
| ENSBTAG00000016782 | SLC39A10 | 4 | yellow |
| ENSBTAG00000016857 | ADAMTS15 | 4 | yellow |
| ENSBTAG00000016881 | ANPEP | 4 | yellow |
| ENSBTAG00000016931 | E2F7 | 4 | yellow |
| ENSBTAG00000016957 | NA | 4 | yellow |
| ENSBTAG00000017021 | MCM4 | 4 | yellow |
| ENSBTAG00000017026 | DEPDC1B | 4 | yellow |
| ENSBTAG00000017069 | GASK1B | 4 | yellow |
| ENSBTAG00000017120 | PHB1 | 4 | yellow |
| ENSBTAG00000017125 | PABPC4 | 4 | yellow |
| ENSBTAG00000017133 | GINS4 | 4 | yellow |
| ENSBTAG00000017181 | MACROD1 | 4 | yellow |
| ENSBTAG00000017223 | MEST | 4 | yellow |
| ENSBTAG00000017266 | ITGA6 | 4 | yellow |
| ENSBTAG00000017271 | MASTL | 4 | yellow |
| ENSBTAG00000017446 | E2F8 | 4 | yellow |

| | | | |
|---|---|---|---|
| ENSBTAG00000017574 | LMNA | 4 | yellow |
| ENSBTAG00000017584 | PLCG1 | 4 | yellow |
| ENSBTAG00000017680 | NOS3 | 4 | yellow |
| ENSBTAG00000017719 | AKAP6 | 4 | yellow |
| ENSBTAG00000017765 | GSTM1 | 4 | yellow |
| ENSBTAG00000017790 | B9D1 | 4 | yellow |
| ENSBTAG00000018049 | MKNK2 | 4 | yellow |
| ENSBTAG00000018130 | MAP2 | 4 | yellow |
| ENSBTAG00000018142 | DTL | 4 | yellow |
| ENSBTAG00000018181 | CCDC8 | 4 | yellow |
| ENSBTAG00000018201 | TKFC | 4 | yellow |
| ENSBTAG00000018216 | SKA1 | 4 | yellow |
| ENSBTAG00000018220 | RGSL1 | 4 | yellow |
| ENSBTAG00000018284 | KBTBD11 | 4 | yellow |
| ENSBTAG00000018572 | FLT3 | 4 | yellow |
| ENSBTAG00000018599 | PROSER3 | 4 | yellow |
| ENSBTAG00000018622 | PCBP4 | 4 | yellow |
| ENSBTAG00000018643 | PRC1 | 4 | yellow |
| ENSBTAG00000018775 | TPX2 | 4 | yellow |
| ENSBTAG00000018806 | PSRC1 | 4 | yellow |
| ENSBTAG00000018863 | PRIMPOL | 4 | yellow |
| ENSBTAG00000018864 | CENPU | 4 | yellow |
| ENSBTAG00000018945 | AMOTL1 | 4 | yellow |
| ENSBTAG00000019002 | SLC2A12 | 4 | yellow |
| ENSBTAG00000019023 | NANS | 4 | yellow |
| ENSBTAG00000019075 | METAP1D | 4 | yellow |
| ENSBTAG00000019132 | DMP1 | 4 | yellow |
| ENSBTAG00000019152 | NYAP2 | 4 | yellow |
| ENSBTAG00000019187 | ZNF462 | 4 | yellow |
| ENSBTAG00000019247 | NA | 4 | yellow |
| ENSBTAG00000019262 | TOP2A | 4 | yellow |
| ENSBTAG00000019269 | COL6A2 | 4 | yellow |
| ENSBTAG00000019276 | NA | 4 | yellow |
| ENSBTAG00000019278 | KNTC1 | 4 | yellow |
| ENSBTAG00000019302 | BCL2 | 4 | yellow |
| ENSBTAG00000019354 | PAX8 | 4 | yellow |
| ENSBTAG00000019496 | CDKN3 | 4 | yellow |
| ENSBTAG00000019585 | MYOM1 | 4 | yellow |
| ENSBTAG00000019600 | OOEP | 4 | yellow |
| ENSBTAG00000019712 | ST14 | 4 | yellow |
| ENSBTAG00000019734 | CD276 | 4 | yellow |
| ENSBTAG00000019743 | CHTF18 | 4 | yellow |
| ENSBTAG00000019777 | CDCA3 | 4 | yellow |
| ENSBTAG00000019824 | NA | 4 | yellow |
| ENSBTAG00000019901 | RAD54L | 4 | yellow |
| ENSBTAG00000019951 | PCYOX1L | 4 | yellow |
| ENSBTAG00000019960 | TM4SF18 | 4 | yellow |

| | | | |
|---|---|---|---|
| ENSBTAG00000020040 | LPCAT4 | 4 | yellow |
| ENSBTAG00000020100 | ARHGAP11A | 4 | yellow |
| ENSBTAG00000020126 | MYO10 | 4 | yellow |
| ENSBTAG00000020153 | NIT1 | 4 | yellow |
| ENSBTAG00000020178 | ARSK | 4 | yellow |
| ENSBTAG00000020227 | FAM72A | 4 | yellow |
| ENSBTAG00000020352 | PAQR5 | 4 | yellow |
| ENSBTAG00000020394 | TNFAIP8L2 | 4 | yellow |
| ENSBTAG00000020465 | FERMT1 | 4 | yellow |
| ENSBTAG00000020601 | ZNF366 | 4 | yellow |
| ENSBTAG00000020710 | CENPQ | 4 | yellow |
| ENSBTAG00000020758 | MAP3K12 | 4 | yellow |
| ENSBTAG00000020789 | STS | 4 | yellow |
| ENSBTAG00000020814 | C2CD4B | 4 | yellow |
| ENSBTAG00000020853 | SLC16A13 | 4 | yellow |
| ENSBTAG00000020880 | ARMH4 | 4 | yellow |
| ENSBTAG00000020975 | SYNGAP1 | 4 | yellow |
| ENSBTAG00000021019 | SLC30A4 | 4 | yellow |
| ENSBTAG00000021069 | PBK | 4 | yellow |
| ENSBTAG00000021092 | ATP6V0D2 | 4 | yellow |
| ENSBTAG00000021162 | CKAP2 | 4 | yellow |
| ENSBTAG00000021181 | BUB1 | 4 | yellow |
| ENSBTAG00000021193 | FBXO5 | 4 | yellow |
| ENSBTAG00000021339 | SCN8A | 4 | yellow |
| ENSBTAG00000021346 | CCDC34 | 4 | yellow |
| ENSBTAG00000021358 | BLNK | 4 | yellow |
| ENSBTAG00000021416 | PRXL2A | 4 | yellow |
| ENSBTAG00000021568 | VWCE | 4 | yellow |
| ENSBTAG00000021582 | NCAPG | 4 | yellow |
| ENSBTAG00000021583 | TPGS2 | 4 | yellow |
| ENSBTAG00000021614 | TASP1 | 4 | yellow |
| ENSBTAG00000021635 | NUDT17 | 4 | yellow |
| ENSBTAG00000021673 | NDC80 | 4 | yellow |
| ENSBTAG00000021681 | PRR11 | 4 | yellow |
| ENSBTAG00000021685 | EEF1A2 | 4 | yellow |
| ENSBTAG00000021830 | ENPP1 | 4 | yellow |
| ENSBTAG00000021841 | CHD7 | 4 | yellow |
| ENSBTAG00000021875 | SLC41A2 | 4 | yellow |
| ENSBTAG00000021912 | DUSP7 | 4 | yellow |
| ENSBTAG00000022028 | DERL3 | 4 | yellow |
| ENSBTAG00000022520 | BRCA1 | 4 | yellow |
| ENSBTAG00000022570 | LOC782922 | 4 | yellow |
| ENSBTAG00000022927 | RAC3 | 4 | yellow |
| ENSBTAG00000023601 | POLR1G | 4 | yellow |
| ENSBTAG00000023938 | CENPA | 4 | yellow |
| ENSBTAG00000024449 | CENPF | 4 | yellow |
| ENSBTAG00000024476 | CKS1B | 4 | yellow |

| | | | |
|---|---|---|---|
| ENSBTAG00000024539 | SPSB1 | 4 | yellow |
| ENSBTAG00000024726 | HJURP | 4 | yellow |
| ENSBTAG00000025009 | NA | 4 | yellow |
| ENSBTAG00000025167 | ABHD15 | 4 | yellow |
| ENSBTAG00000025210 | COL4A2 | 4 | yellow |
| ENSBTAG00000025308 | STON2 | 4 | yellow |
| ENSBTAG00000025345 | ARL10 | 4 | yellow |
| ENSBTAG00000025752 | P3H4 | 4 | yellow |
| ENSBTAG00000026111 | LBH | 4 | yellow |
| ENSBTAG00000026236 | AZGP1 | 4 | yellow |
| ENSBTAG00000026369 | ENC1 | 4 | yellow |
| ENSBTAG00000026375 | RMI2 | 4 | yellow |
| ENSBTAG00000026505 | LOC539789 | 4 | yellow |
| ENSBTAG00000026580 | HTR1E | 4 | yellow |
| ENSBTAG00000026586 | PPP1R14C | 4 | yellow |
| ENSBTAG00000026613 | MSTO1 | 4 | yellow |
| ENSBTAG00000026704 | CENPW | 4 | yellow |
| ENSBTAG00000026768 | TH | 4 | yellow |
| ENSBTAG00000026851 | FMNL2 | 4 | yellow |
| ENSBTAG00000026963 | NCALD | 4 | yellow |
| ENSBTAG00000026971 | CDCA5 | 4 | yellow |
| ENSBTAG00000027386 | SOX12 | 4 | yellow |
| ENSBTAG00000027715 | LOC101906134 | 4 | yellow |
| ENSBTAG00000027728 | NUDT12 | 4 | yellow |
| ENSBTAG00000027923 | NA | 4 | yellow |
| ENSBTAG00000030393 | RDH13 | 4 | yellow |
| ENSBTAG00000030593 | RFTN1 | 4 | yellow |
| ENSBTAG00000030646 | KCTD17 | 4 | yellow |
| ENSBTAG00000030757 | ELFN1 | 4 | yellow |
| ENSBTAG00000030855 | CHCHD6 | 4 | yellow |
| ENSBTAG00000030897 | XRCC2 | 4 | yellow |
| ENSBTAG00000030965 | MCM7 | 4 | yellow |
| ENSBTAG00000031217 | MYL6B | 4 | yellow |
| ENSBTAG00000031711 | NA | 4 | yellow |
| ENSBTAG00000031837 | IMPDH2 | 4 | yellow |
| ENSBTAG00000032304 | ACY1 | 4 | yellow |
| ENSBTAG00000032340 | KNSTRN | 4 | yellow |
| ENSBTAG00000032372 | HASPIN | 4 | yellow |
| ENSBTAG00000032396 | KCTD19 | 4 | yellow |
| ENSBTAG00000032450 | NA | 4 | yellow |
| ENSBTAG00000032887 | SUPT3H | 4 | yellow |
| ENSBTAG00000032902 | LOC512464 | 4 | yellow |
| ENSBTAG00000033008 | MYOZ1 | 4 | yellow |
| ENSBTAG00000033441 | SHCBP1 | 4 | yellow |
| ENSBTAG00000033545 | EBD | 4 | yellow |
| ENSBTAG00000033690 | BARD1 | 4 | yellow |
| ENSBTAG00000034193 | BCO2 | 4 | yellow |

| | | | |
|---|---|---|---|
| ENSBTAG00000034246 | ACRBP | 4 | yellow |
| ENSBTAG00000034323 | LOC107131287 | 4 | yellow |
| ENSBTAG00000035319 | NA | 4 | yellow |
| ENSBTAG00000037389 | TRIM44 | 4 | yellow |
| ENSBTAG00000037526 | FABP4 | 4 | yellow |
| ENSBTAG00000037640 | PID1 | 4 | yellow |
| ENSBTAG00000037757 | EBF4 | 4 | yellow |
| ENSBTAG00000037856 | MGC152281 | 4 | yellow |
| ENSBTAG00000037951 | KLHL6 | 4 | yellow |
| ENSBTAG00000038085 | NA | 4 | yellow |
| ENSBTAG00000038322 | LOC510613 | 4 | yellow |
| ENSBTAG00000038384 | KRT5 | 4 | yellow |
| ENSBTAG00000038496 | CR2 | 4 | yellow |
| ENSBTAG00000038532 | FOLR3 | 4 | yellow |
| ENSBTAG00000039071 | ANKRD16 | 4 | yellow |
| ENSBTAG00000039163 | NA | 4 | yellow |
| ENSBTAG00000039190 | SLC9A5 | 4 | yellow |
| ENSBTAG00000039234 | LIPK | 4 | yellow |
| ENSBTAG00000039329 | RAET1G | 4 | yellow |
| ENSBTAG00000039462 | PCLAF | 4 | yellow |
| ENSBTAG00000039686 | NA | 4 | yellow |
| ENSBTAG00000039709 | PARPBP | 4 | yellow |
| ENSBTAG00000040028 | OBSCN | 4 | yellow |
| ENSBTAG00000040065 | RAD51AP1 | 4 | yellow |
| ENSBTAG00000040512 | PCP4L1 | 4 | yellow |
| ENSBTAG00000042753 | LOC112443378 | 4 | yellow |
| ENSBTAG00000043545 | NA | 4 | yellow |
| ENSBTAG00000044000 | PRIM1 | 4 | yellow |
| ENSBTAG00000044006 | GINS2 | 4 | yellow |
| ENSBTAG00000044033 | EDIL3 | 4 | yellow |
| ENSBTAG00000044063 | B4GALT6 | 4 | yellow |
| ENSBTAG00000044150 | UCK2 | 4 | yellow |
| ENSBTAG00000044175 | CENPK | 4 | yellow |
| ENSBTAG00000045604 | TTC9 | 4 | yellow |
| ENSBTAG00000045770 | KRT77 | 4 | yellow |
| ENSBTAG00000045795 | KIR2DS1 | 4 | yellow |
| ENSBTAG00000045892 | NA | 4 | yellow |
| ENSBTAG00000046264 | NA | 4 | yellow |
| ENSBTAG00000046556 | NA | 4 | yellow |
| ENSBTAG00000046594 | NA | 4 | yellow |
| ENSBTAG00000046766 | NA | 4 | yellow |
| ENSBTAG00000046866 | NA | 4 | yellow |
| ENSBTAG00000046910 | NA | 4 | yellow |
| ENSBTAG00000047103 | IDNK | 4 | yellow |
| ENSBTAG00000047265 | PPP1R14B | 4 | yellow |
| ENSBTAG00000047322 | NA | 4 | yellow |
| ENSBTAG00000047347 | NA | 4 | yellow |

| | | | |
|---|---|---|---|
| ENSBTAG00000047462 | NA | 4 | yellow |
| ENSBTAG00000047536 | NA | 4 | yellow |
| ENSBTAG00000047598 | NA | 4 | yellow |
| ENSBTAG00000047739 | TSC22D1 | 4 | yellow |
| ENSBTAG00000047755 | FNDC10 | 4 | yellow |
| ENSBTAG00000047807 | SAPCD2 | 4 | yellow |
| ENSBTAG00000048271 | MICAL3 | 4 | yellow |

| | | |
|---|---|---|
| black | 7 | 190 |
| blue | 2 | 2530 |
| brown | 3 | 1045 |
| cyan | 14 | 53 |
| green | 5 | 448 |
| greenyellow | 11 | 123 |
| grey | 0 | 231 |
| grey60 | 17 | 43 |
| lightcyan | 16 | 49 |
| magenta | 9 | 178 |
| midnightblue | 15 | 53 |
| pink | 8 | 179 |
| purple | 10 | 139 |
| red | 6 | 216 |
| salmon | 13 | 75 |
| tan | 12 | 117 |
| turquoise | 1 | 2980 |
| yellow | 4 | 614 |

**Supplementary File S4: Details about miRNA-seq-based modules**

| miRNA_id | Module_label | Module_color |
|---|---|---|
| bta-miR-100 | 2 | blue |
| bta-miR-10b | 2 | blue |
| bta-miR-125a | 2 | blue |
| bta-miR-127 | 2 | blue |
| bta-miR-1388-5p | 2 | blue |
| bta-miR-141 | 2 | blue |
| bta-miR-143 | 2 | blue |
| bta-miR-148a | 2 | blue |
| bta-miR-150 | 2 | blue |
| bta-miR-151-3p | 2 | blue |
| bta-miR-152/bta-miR-2957 | 2 | blue |
| bta-miR-182 | 2 | blue |
| bta-miR-183 | 2 | blue |
| bta-miR-193b | 2 | blue |
| bta-miR-200a | 2 | blue |
| bta-miR-200b | 2 | blue |
| bta-miR-200c | 2 | blue |
| bta-miR-204 | 2 | blue |
| bta-miR-205 | 2 | blue |
| bta-miR-224 | 2 | blue |
| bta-miR-2285t | 2 | blue |
| bta-miR-27b | 2 | blue |
| bta-miR-29a | 2 | blue |
| bta-miR-30a-5p | 2 | blue |
| bta-miR-31 | 2 | blue |
| bta-miR-335 | 2 | blue |
| bta-miR-3431 | 2 | blue |
| bta-miR-375 | 2 | blue |
| bta-miR-429 | 2 | blue |
| bta-miR-450b | 2 | blue |
| bta-miR-451 | 2 | blue |
| bta-miR-455-5p | 2 | blue |
| bta-miR-486 | 2 | blue |
| bta-miR-504 | 2 | blue |
| bta-miR-615 | 2 | blue |
| bta-miR-96 | 2 | blue |
| bta-miR-99a-3p | 2 | blue |
| bta-miR-99a-5p | 2 | blue |
| bta-miR-10a | 3 | brown |
| bta-miR-126-5p | 3 | brown |
| bta-miR-142-3p | 3 | brown |
| bta-miR-142-5p | 3 | brown |
| bta-miR-15a | 3 | brown |
| bta-miR-16a | 3 | brown |

| | | |
|---|---|---|
| bta-miR-16b | 3 | brown |
| bta-miR-181c | 3 | brown |
| bta-miR-181d | 3 | brown |
| bta-miR-186 | 3 | brown |
| bta-miR-191 | 3 | brown |
| bta-miR-192 | 3 | brown |
| bta-miR-19a | 3 | brown |
| bta-miR-19b | 3 | brown |
| bta-miR-223 | 3 | brown |
| bta-miR-2284w | 3 | brown |
| bta-miR-2284x | 3 | brown |
| bta-miR-23a | 3 | brown |
| bta-miR-25 | 3 | brown |
| bta-miR-29c | 3 | brown |
| bta-miR-301a | 3 | brown |
| bta-miR-30b-5p | 3 | brown |
| bta-miR-32 | 3 | brown |
| bta-miR-338 | 3 | brown |
| bta-miR-342 | 3 | brown |
| bta-miR-361 | 3 | brown |
| bta-miR-362-5p | 3 | brown |
| bta-miR-363 | 3 | brown |
| bta-miR-374a | 3 | brown |
| bta-miR-374b | 3 | brown |
| bta-miR-410 | 3 | brown |
| bta-miR-425-5p | 3 | brown |
| bta-miR-660 | 3 | brown |
| bta-miR-101 | 0 | grey |
| bta-miR-106b | 0 | grey |
| bta-miR-107 | 0 | grey |
| bta-miR-125b | 0 | grey |
| bta-miR-1296 | 0 | grey |
| bta-miR-1388-3p | 0 | grey |
| bta-miR-145 | 0 | grey |
| bta-miR-1468 | 0 | grey |
| bta-miR-146a | 0 | grey |
| bta-miR-15b | 0 | grey |
| bta-miR-188 | 0 | grey |
| bta-miR-194 | 0 | grey |
| bta-miR-196b | 0 | grey |
| bta-miR-208b | 0 | grey |
| bta-miR-21-3p | 0 | grey |
| bta-miR-21-5p | 0 | grey |
| bta-miR-215 | 0 | grey |
| bta-miR-22-3p/bta-miR-3600 | 0 | grey |
| bta-miR-2284i | 0 | grey |
| bta-miR-2285b | 0 | grey |

| | | |
|---|---|---|
| bta-miR-2285h | 0 | grey |
| bta-miR-2285k | 0 | grey |
| bta-miR-2285q | 0 | grey |
| bta-miR-2285v | 0 | grey |
| bta-miR-2299-5p | 0 | grey |
| bta-miR-2313-3p | 0 | grey |
| bta-miR-2313-5p | 0 | grey |
| bta-miR-2316 | 0 | grey |
| bta-miR-2319a | 0 | grey |
| bta-miR-2336 | 0 | grey |
| bta-miR-2339 | 0 | grey |
| bta-miR-2357 | 0 | grey |
| bta-miR-2388-3p | 0 | grey |
| bta-miR-2397-5p | 0 | grey |
| bta-miR-2411-3p | 0 | grey |
| bta-miR-2424 | 0 | grey |
| bta-miR-2450a | 0 | grey |
| bta-miR-2478 | 0 | grey |
| bta-miR-2483-5p | 0 | grey |
| bta-miR-26a | 0 | grey |
| bta-miR-26a/bta-miR-26c | 0 | grey |
| bta-miR-26b | 0 | grey |
| bta-miR-27a-3p | 0 | grey |
| bta-miR-2898 | 0 | grey |
| bta-miR-292 | 0 | grey |
| bta-miR-30e-5p | 0 | grey |
| bta-miR-30f | 0 | grey |
| bta-miR-331 | 0 | grey |
| bta-miR-339a | 0 | grey |
| bta-miR-33a | 0 | grey |
| bta-miR-33b | 0 | grey |
| bta-miR-340 | 0 | grey |
| bta-miR-345-3p | 0 | grey |
| bta-miR-345-5p | 0 | grey |
| bta-miR-34c | 0 | grey |
| bta-miR-3613 | 0 | grey |
| bta-miR-362-3p | 0 | grey |
| bta-miR-369-3p | 0 | grey |
| bta-miR-4286 | 0 | grey |
| bta-miR-454 | 0 | grey |
| bta-miR-505 | 0 | grey |
| bta-miR-532 | 0 | grey |
| bta-miR-6119-3p | 0 | grey |
| bta-miR-6119-5p | 0 | grey |
| bta-miR-6120-3p | 0 | grey |
| bta-miR-6520 | 0 | grey |
| bta-miR-6524 | 0 | grey |

| | | |
|---|---|---|
| bta-miR-6530 | 0 | grey |
| bta-miR-6533 | 0 | grey |
| bta-miR-664 | 0 | grey |
| bta-miR-769 | 0 | grey |
| bta-miR-874 | 0 | grey |
| bta-miR-935 | 0 | grey |
| bta-miR-98 | 0 | grey |
| bta-let-7a | 1 | turquoise |
| bta-let-7a-5p | 1 | turquoise |
| bta-let-7b/bta-miR-3596 | 1 | turquoise |
| bta-let-7c | 1 | turquoise |
| bta-let-7d | 1 | turquoise |
| bta-let-7e | 1 | turquoise |
| bta-let-7f | 1 | turquoise |
| bta-let-7g | 1 | turquoise |
| bta-let-7i | 1 | turquoise |
| bta-miR-103 | 1 | turquoise |
| bta-miR-106a | 1 | turquoise |
| bta-miR-1246 | 1 | turquoise |
| bta-miR-1249 | 1 | turquoise |
| bta-miR-126-3p | 1 | turquoise |
| bta-miR-1271 | 1 | turquoise |
| bta-miR-1277 | 1 | turquoise |
| bta-miR-128 | 1 | turquoise |
| bta-miR-129-3p | 1 | turquoise |
| bta-miR-1291 | 1 | turquoise |
| bta-miR-1306 | 1 | turquoise |
| bta-miR-1307 | 1 | turquoise |
| bta-miR-130a | 1 | turquoise |
| bta-miR-130b | 1 | turquoise |
| bta-miR-132 | 1 | turquoise |
| bta-miR-1343-3p | 1 | turquoise |
| bta-miR-1343-5p | 1 | turquoise |
| bta-miR-138 | 1 | turquoise |
| bta-miR-139 | 1 | turquoise |
| bta-miR-140 | 1 | turquoise |
| bta-miR-1434-3p | 1 | turquoise |
| bta-miR-1434-5p | 1 | turquoise |
| bta-miR-146b | 1 | turquoise |
| bta-miR-147 | 1 | turquoise |
| bta-miR-148b | 1 | turquoise |
| bta-miR-149-3p | 1 | turquoise |
| bta-miR-149-5p | 1 | turquoise |
| bta-miR-151-5p | 1 | turquoise |
| bta-miR-155 | 1 | turquoise |
| bta-miR-17-3p | 1 | turquoise |
| bta-miR-17-5p | 1 | turquoise |

| | | |
|---|---|---|
| bta-miR-181a | 1 | turquoise |
| bta-miR-181b | 1 | turquoise |
| bta-miR-1839 | 1 | turquoise |
| bta-miR-184 | 1 | turquoise |
| bta-miR-185 | 1 | turquoise |
| bta-miR-187 | 1 | turquoise |
| bta-miR-18a | 1 | turquoise |
| bta-miR-18b | 1 | turquoise |
| bta-miR-190a | 1 | turquoise |
| bta-miR-193a-3p | 1 | turquoise |
| bta-miR-193a-5p | 1 | turquoise |
| bta-miR-1940 | 1 | turquoise |
| bta-miR-195 | 1 | turquoise |
| bta-miR-197 | 1 | turquoise |
| bta-miR-20a | 1 | turquoise |
| bta-miR-20b | 1 | turquoise |
| bta-miR-210 | 1 | turquoise |
| bta-miR-212 | 1 | turquoise |
| bta-miR-219-5p | 1 | turquoise |
| bta-miR-22-5p | 1 | turquoise |
| bta-miR-221 | 1 | turquoise |
| bta-miR-222 | 1 | turquoise |
| bta-miR-2284z | 1 | turquoise |
| bta-miR-2289 | 1 | turquoise |
| bta-miR-2299-3p | 1 | turquoise |
| bta-miR-2318 | 1 | turquoise |
| bta-miR-2320-3p | 1 | turquoise |
| bta-miR-2320-5p | 1 | turquoise |
| bta-miR-2323 | 1 | turquoise |
| bta-miR-2328-5p | 1 | turquoise |
| bta-miR-2331-5p | 1 | turquoise |
| bta-miR-2332 | 1 | turquoise |
| bta-miR-2344 | 1 | turquoise |
| bta-miR-2346 | 1 | turquoise |
| bta-miR-2347 | 1 | turquoise |
| bta-miR-2349 | 1 | turquoise |
| bta-miR-2350 | 1 | turquoise |
| bta-miR-2353 | 1 | turquoise |
| bta-miR-2355-5p | 1 | turquoise |
| bta-miR-2368-3p | 1 | turquoise |
| bta-miR-2370-3p | 1 | turquoise |
| bta-miR-2376 | 1 | turquoise |
| bta-miR-2388-5p | 1 | turquoise |
| bta-miR-2389 | 1 | turquoise |
| bta-miR-2397-3p | 1 | turquoise |
| bta-miR-2398 | 1 | turquoise |
| bta-miR-23b-3p | 1 | turquoise |

| | | |
|---|---|---|
| bta-miR-23b-5p | 1 | turquoise |
| bta-miR-24 | 1 | turquoise |
| bta-miR-2400 | 1 | turquoise |
| bta-miR-2402 | 1 | turquoise |
| bta-miR-2409 | 1 | turquoise |
| bta-miR-2410 | 1 | turquoise |
| bta-miR-2411-5p | 1 | turquoise |
| bta-miR-2415-3p | 1 | turquoise |
| bta-miR-2417 | 1 | turquoise |
| bta-miR-2419-3p | 1 | turquoise |
| bta-miR-2419-5p | 1 | turquoise |
| bta-miR-2425-3p | 1 | turquoise |
| bta-miR-2425-5p | 1 | turquoise |
| bta-miR-2431-3p | 1 | turquoise |
| bta-miR-2431-5p | 1 | turquoise |
| bta-miR-2436-3p | 1 | turquoise |
| bta-miR-2440 | 1 | turquoise |
| bta-miR-2443 | 1 | turquoise |
| bta-miR-2446 | 1 | turquoise |
| bta-miR-2447 | 1 | turquoise |
| bta-miR-2448-3p | 1 | turquoise |
| bta-miR-2448-5p | 1 | turquoise |
| bta-miR-2449 | 1 | turquoise |
| bta-miR-2454-3p | 1 | turquoise |
| bta-miR-2454-5p | 1 | turquoise |
| bta-miR-2457 | 1 | turquoise |
| bta-miR-2465 | 1 | turquoise |
| bta-miR-2468 | 1 | turquoise |
| bta-miR-2469 | 1 | turquoise |
| bta-miR-2483-3p | 1 | turquoise |
| bta-miR-2484 | 1 | turquoise |
| bta-miR-27a-5p | 1 | turquoise |
| bta-miR-28 | 1 | turquoise |
| bta-miR-2903 | 1 | turquoise |
| bta-miR-296 | 1 | turquoise |
| bta-miR-29b | 1 | turquoise |
| bta-miR-301b | 1 | turquoise |
| bta-miR-30b-3p | 1 | turquoise |
| bta-miR-30c | 1 | turquoise |
| bta-miR-30d | 1 | turquoise |
| bta-miR-324 | 1 | turquoise |
| bta-miR-326 | 1 | turquoise |
| bta-miR-328 | 1 | turquoise |
| bta-miR-330 | 1 | turquoise |
| bta-miR-3432 | 1 | turquoise |
| bta-miR-34a | 1 | turquoise |
| bta-miR-34b | 1 | turquoise |

| | | |
|---|---|---|
| bta-miR-378 | 1 | turquoise |
| bta-miR-378b | 1 | turquoise |
| bta-miR-378c | 1 | turquoise |
| bta-miR-380-3p | 1 | turquoise |
| bta-miR-411a | 1 | turquoise |
| bta-miR-421 | 1 | turquoise |
| bta-miR-423-3p | 1 | turquoise |
| bta-miR-423-5p | 1 | turquoise |
| bta-miR-424-3p | 1 | turquoise |
| bta-miR-424-5p | 1 | turquoise |
| bta-miR-425-3p | 1 | turquoise |
| bta-miR-449a | 1 | turquoise |
| bta-miR-449b | 1 | turquoise |
| bta-miR-449c | 1 | turquoise |
| bta-miR-452 | 1 | turquoise |
| bta-miR-455-3p | 1 | turquoise |
| bta-miR-484 | 1 | turquoise |
| bta-miR-491 | 1 | turquoise |
| bta-miR-497 | 1 | turquoise |
| bta-miR-500 | 1 | turquoise |
| bta-miR-502b | 1 | turquoise |
| bta-miR-503-3p | 1 | turquoise |
| bta-miR-503-5p | 1 | turquoise |
| bta-miR-542-5p/bta-miR-3601 | 1 | turquoise |
| bta-miR-545-3p | 1 | turquoise |
| bta-miR-545-5p | 1 | turquoise |
| bta-miR-6123 | 1 | turquoise |
| bta-miR-628 | 1 | turquoise |
| bta-miR-6517 | 1 | turquoise |
| bta-miR-6518 | 1 | turquoise |
| bta-miR-652 | 1 | turquoise |
| bta-miR-6521 | 1 | turquoise |
| bta-miR-6522 | 1 | turquoise |
| bta-miR-6523 | 1 | turquoise |
| bta-miR-6525 | 1 | turquoise |
| bta-miR-6527 | 1 | turquoise |
| bta-miR-6529 | 1 | turquoise |
| bta-miR-6531 | 1 | turquoise |
| bta-miR-671 | 1 | turquoise |
| bta-miR-677 | 1 | turquoise |
| bta-miR-744 | 1 | turquoise |
| bta-miR-760-3p | 1 | turquoise |
| bta-miR-760-5p | 1 | turquoise |
| bta-miR-877 | 1 | turquoise |
| bta-miR-885 | 1 | turquoise |
| bta-miR-92a | 1 | turquoise |
| bta-miR-92b | 1 | turquoise |

| | | |
|---|---|---|
| bta-miR-93 | 1 | turquoise |
| bta-miR-99b | 1 | turquoise |

| | | |
|---|---|---|
| blue | 2 | 38 |
| brown | 3 | 33 |
| turquoise | 1 | 183 |
| grey | 0 | 74 |

**Supplementary File S5: Comprehensive information from the module-trai**

| RNA-seq-based Modules | R_Milk_volume (in Litres) | P_Milk_volume (in Litres) |
|---|---|---|
| MEmidnightblue | 0.266826804 | 0.083691788 |
| MEsalmon | -0.075499909 | 0.630384984 |
| MEpink | -0.263818804 | 0.087371242 |
| MEyellow | 0.038674102 | 0.805510269 |
| MEblue | 0.192266291 | 0.216761239 |
| MEred | 0.209245812 | 0.17809405 |
| MEbrown | -0.123424523 | 0.430391768 |
| MEgreenyellow | -0.238218238 | 0.123972471 |
| MEblack | -0.233585543 | 0.131674988 |
| MElightcyan | -0.281852798 | 0.067086922 |
| MEgrey60 | -0.215957931 | 0.164262696 |
| MEpurple | -0.379037561 | 0.01219027 |
| MEgreen | -0.324236179 | 0.033906212 |
| MEturquoise | -0.107651539 | 0.492008434 |
| MEcyan | -0.137323203 | 0.379876321 |
| MEmagenta | 0.23178803 | 0.134757851 |
| MEtan | 0.183698931 | 0.238332263 |
| MEgrey | -0.251371792 | 0.10394731 |

**t relationships analysis result (R = correlation; P = p-value)**

| R_Rectal_tempreature (in Farenheit) | P_Rectal_tempreature (in Farenheit) |
|---|---|
| -0.442496997 | 0.002966311 |
| -0.068481731 | 0.662586431 |
| 0.520484855 | 0.000346411 |
| 0.347039559 | 0.022607361 |
| -0.101892669 | 0.51558771 |
| -0.284088612 | 0.064856823 |
| -0.152079722 | 0.330282267 |
| 0.073803399 | 0.638110219 |
| -0.120735778 | 0.440579251 |
| -0.104549072 | 0.504641585 |
| 0.244011579 | 0.114822667 |
| 0.670228647 | 8.82E-07 |
| 0.43633481 | 0.003443629 |
| 0.00928522 | 0.952876996 |
| 0.654898615 | 1.89E-06 |
| -0.11316074 | 0.469984409 |
| -0.367793542 | 0.015247753 |
| -0.112175956 | 0.473882327 |

| R_Ambient_temperature (in Celsius) | P_Ambient_temperature (in Celsius) | R_Humidity (%) | P_Humidity (%) |
|---|---|---|---|
| -0.358663929 | 0.018186873 | 0.321584632 | 0.035478908 |
| -0.049271645 | 0.753698972 | 0.263469087 | 0.08780705 |
| 0.545594118 | 0.000154447 | 0.28525189 | 0.063720033 |
| 0.227728963 | 0.141916565 | 0.004837921 | 0.97543726 |
| -0.078709749 | 0.61587511 | 0.031233299 | 0.842400676 |
| -0.109354446 | 0.485144382 | 0.256427062 | 0.096946798 |
| -0.095593134 | 0.542011664 | -0.150138389 | 0.336565267 |
| 0.157010573 | 0.314655091 | 0.233063692 | 0.132564527 |
| -0.007294893 | 0.962970073 | -0.149958729 | 0.337150442 |
| 0.148254414 | 0.342732898 | -0.155316625 | 0.319970003 |
| 0.057665441 | 0.713394605 | -0.269788223 | 0.080188443 |
| 0.498655722 | 0.000665583 | -0.031851223 | 0.839323241 |
| 0.451418866 | 0.002378291 | 0.228190722 | 0.141088301 |
| -0.015537596 | 0.921225291 | -0.016228056 | 0.917736412 |
| 0.280762802 | 0.068195946 | -0.023097485 | 0.883118804 |
| -0.205280081 | 0.186648067 | -0.147846788 | 0.344076452 |
| -0.410358334 | 0.006273411 | -0.323081834 | 0.03458368 |
| 0.132387003 | 0.397400674 | -0.046091256 | 0.769147321 |

| R_Total_bacterial_counts (TBC; Per 10mL) | P_Total_bacterial_counts (TBC; Per 10mL) |
|---|---|
| 0.175623718 | 0.259958677 |
| 0.018190078 | 0.907830902 |
| -0.141322082 | 0.366020958 |
| -0.413970312 | 0.005787278 |
| -0.777108635 | 8.93E-10 |
| -0.569640981 | 6.69E-05 |
| -0.188775006 | 0.2253822 |
| -0.019029971 | 0.903594722 |
| -0.138850221 | 0.374549268 |
| -0.102594571 | 0.512683927 |
| 0.505936669 | 0.000537905 |
| 0.561080297 | 9.08E-05 |
| 0.521197772 | 0.000338848 |
| 0.72688445 | 3.40E-08 |
| 0.22954044 | 0.138687808 |
| -0.491822602 | 0.0008094 |
| -0.018575946 | 0.905884376 |
| -0.1309517 | 0.402582928 |

| R_Somatic_cell_counts (SCC; Per mL) | P_Somatic_cell_counts (SCC; Per mL) | R_CD14_cell_number (Per mL) |
|---|---|---|
| -0.005036622 | 0.974428757 | -0.251603113 |
| -0.035585914 | 0.820775315 | -0.639160425 |
| 0.029199986 | 0.852543288 | -0.553401627 |
| -0.443348346 | 0.002905163 | -0.249221376 |
| -0.673523696 | 7.44E-07 | -0.257210283 |
| -0.54437482 | 0.000160864 | -0.573593276 |
| -0.312723784 | 0.041174754 | -0.090947223 |
| 0.039157009 | 0.803129529 | -0.39010217 |
| -0.275153919 | 0.074134086 | -0.09688273 |
| -0.229420417 | 0.138900034 | -0.182912468 |
| 0.40560248 | 0.006967192 | 0.518114994 |
| 0.589130206 | 3.24E-05 | 0.170097421 |
| 0.518846066 | 0.000364376 | -0.252727102 |
| 0.586031746 | 3.64E-05 | 0.308495358 |
| 0.177461306 | 0.254926169 | 0.270261933 |
| -0.313456016 | 0.040677447 | 0.428011859 |
| -0.086821106 | 0.579856137 | 0.469801662 |
| -0.014228759 | 0.927842763 | 0.077611988 |

| P_CD14_cell_number (Per mL) |
| --- |
| 0.103618763 |
| 3.97E-06 |
| 0.00011856 |
| 0.10703982 |
| 0.09589548 |
| 5.80E-05 |
| 0.561906941 |
| 0.009708307 |
| 0.536549861 |
| 0.240383017 |
| 0.000372658 |
| 0.275490095 |
| 0.102033649 |
| 0.044144413 |
| 0.079638848 |
| 0.004194445 |
| 0.001480192 |
| 0.620821586 |

**Supplementary File S6: Comprehensive details of the results of functional enrichment analysis of si**

| BP | Term | Overlap | P-value | Adjusted P-value |
|---|---|---|---|---|
| | cytokine-mediated signaling pathway (GO:0019221) | 219/621 | 8.06E-46 | 4.19E-42 |
| | cellular response to cytokine stimulus (GO:0071345) | 177/482 | 9.01E-40 | 2.34E-36 |
| | positive regulation of cytokine production (GO:0001819) | 126/335 | 1.03E-29 | 1.79E-26 |
| | inflammatory response (GO:0006954) | 94/230 | 1.38E-25 | 1.80E-22 |
| | response to cytokine (GO:0034097) | 70/150 | 2.06E-23 | 2.14E-20 |
| | response to lipopolysaccharide (GO:0032496) | 72/159 | 4.55E-23 | 3.51E-20 |
| | positive regulation of intracellular signal transduction (G( | 159/546 | 4.73E-23 | 3.51E-20 |
| | neutrophil mediated immunity (GO:0002446) | 145/488 | 5.01E-22 | 3.25E-19 |
| | neutrophil degranulation (GO:0043312) | 141/481 | 7.51E-21 | 4.34E-18 |
| | neutrophil activation involved in immune response (GO:( | 141/485 | 1.73E-20 | 8.99E-18 |
| | response to molecule of bacterial origin (GO:0002237) | 43/73 | 6.48E-20 | 3.06E-17 |
| | regulation of interleukin-8 production (GO:0032677) | 45/81 | 2.28E-19 | 9.85E-17 |
| | regulation of inflammatory response (GO:0050727) | 78/206 | 4.44E-19 | 1.77E-16 |
| | regulation of I-kappaB kinase/NF-kappaB signaling (GO:0( | 81/224 | 2.42E-18 | 8.97E-16 |
| | cellular response to lipopolysaccharide (GO:0071222) | 54/120 | 1.68E-17 | 5.81E-15 |
| | negative regulation of cytokine production (GO:0001818) | 69/182 | 4.50E-17 | 1.46E-14 |
| | positive regulation of DNA-binding transcription factor ac | 82/246 | 3.83E-16 | 1.17E-13 |
| | positive regulation of interleukin-8 production (GO:0032 | 35/61 | 5.71E-16 | 1.65E-13 |
| | regulation of interleukin-6 production (GO:0032675) | 49/110 | 8.55E-16 | 2.34E-13 |
| | cellular response to molecule of bacterial origin (GO:007 | 50/115 | 1.46E-15 | 3.80E-13 |
| | positive regulation of NF-kappaB transcription factor acti | 59/155 | 6.76E-15 | 1.67E-12 |
| | positive regulation of I-kappaB kinase/NF-kappaB signalir | 62/171 | 1.97E-14 | 4.64E-12 |
| | innate immune response (GO:0045087) | 90/302 | 3.08E-14 | 6.79E-12 |
| | regulation of cytokine-mediated signaling pathway (GO:0 | 37/74 | 3.13E-14 | 6.79E-12 |
| | defense response to symbiont (GO:0140546) | 50/124 | 5.49E-14 | 1.10E-11 |
| | regulation of tumor necrosis factor production (GO:0032 | 50/124 | 5.49E-14 | 1.10E-11 |
| | positive regulation of phagocytosis (GO:0050766) | 30/53 | 1.14E-13 | 2.20E-11 |
| | regulation of innate immune response (GO:0045088) | 33/64 | 2.47E-13 | 4.58E-11 |
| | defense response to virus (GO:0051607) | 51/133 | 3.23E-13 | 5.78E-11 |
| | positive regulation of response to external stimulus (GO:( | 52/139 | 5.95E-13 | 1.03E-10 |
| | regulation of type I interferon production (GO:0032479) | 39/89 | 1.39E-12 | 2.33E-10 |
| | positive regulation of T cell activation (GO:0050870) | 35/75 | 2.06E-12 | 3.34E-10 |
| | positive regulation of defense response (GO:0031349) | 41/98 | 2.38E-12 | 3.66E-10 |
| | response to lipid (GO:0033993) | 45/114 | 2.40E-12 | 3.66E-10 |
| | regulation of phagocytosis (GO:0050764) | 30/58 | 2.70E-12 | 3.97E-10 |
| | regulation of defense response (GO:0031347) | 37/83 | 2.75E-12 | 3.97E-10 |
| | toll-like receptor signaling pathway (GO:0002224) | 39/91 | 3.27E-12 | 4.55E-10 |
| | positive regulation of interleukin-6 production (GO:0032 | 35/76 | 3.33E-12 | 4.55E-10 |
| | positive regulation of cellular process (GO:0048522) | 145/625 | 4.52E-12 | 6.02E-10 |
| | positive regulation of inflammatory response (GO:00507 | 38/89 | 7.10E-12 | 9.22E-10 |
| | regulation of interferon-gamma production (GO:0032649 | 37/86 | 1.02E-11 | 1.29E-09 |
| | positive regulation of transcription, DNA-templated (GO:( | 237/1183 | 1.43E-11 | 1.77E-09 |
| | cellular response to lipid (GO:0071396) | 67/219 | 1.61E-11 | 1.93E-09 |
| | regulation of interleukin-12 production (GO:0032655) | 27/51 | 1.63E-11 | 1.93E-09 |

| | | | |
|---|---|---|---|
| regulation of cell adhesion (GO:0030155) | 48/133 | 2.05E-11 | 2.36E-09 |
| positive regulation of type I interferon production (GO:0( | 34/77 | 2.90E-11 | 3.27E-09 |
| regulation of cell population proliferation (GO:0042127) | 166/764 | 3.60E-11 | 3.96E-09 |
| cellular response to tumor necrosis factor (GO:0071356) | 61/194 | 3.66E-11 | 3.96E-09 |
| MyD88-independent toll-like receptor signaling pathway | 20/31 | 5.19E-11 | 5.50E-09 |
| regulation of cytokine production (GO:0001817) | 51/150 | 6.19E-11 | 6.44E-09 |
| positive regulation of transport (GO:0051050) | 37/91 | 7.57E-11 | 7.71E-09 |
| positive regulation of peptidyl-tyrosine phosphorylation ( | 47/134 | 1.03E-10 | 1.02E-08 |
| cellular response to interferon-gamma (GO:0071346) | 44/121 | 1.04E-10 | 1.02E-08 |
| cellular response to type I interferon (GO:0071357) | 30/65 | 1.09E-10 | 1.02E-08 |
| type I interferon signaling pathway (GO:0060337) | 30/65 | 1.09E-10 | 1.02E-08 |
| regulation of peptidyl-tyrosine phosphorylation (GO:005( | 37/92 | 1.10E-10 | 1.02E-08 |
| cellular response to oxygen-containing compound (GO:1 | 85/323 | 2.16E-10 | 1.97E-08 |
| positive regulation of transcription by RNA polymerase II | 187/908 | 2.34E-10 | 2.06E-08 |
| response to interleukin-1 (GO:0070555) | 35/86 | 2.37E-10 | 2.06E-08 |
| cellular response to interleukin-1 (GO:0071347) | 51/155 | 2.38E-10 | 2.06E-08 |
| TRIF-dependent toll-like receptor signaling pathway (GO: | 19/30 | 2.55E-10 | 2.15E-08 |
| cellular protein modification process (GO:0006464) | 206/1025 | 2.57E-10 | 2.15E-08 |
| regulation of interferon-beta production (GO:0032648) | 25/49 | 2.62E-10 | 2.16E-08 |
| positive regulation of interleukin-1 beta production (GO:( | 27/56 | 2.68E-10 | 2.17E-08 |
| negative regulation of intracellular signal transduction (G | 60/198 | 2.78E-10 | 2.22E-08 |
| positive regulation of interferon-gamma production (GO: | 27/57 | 4.45E-10 | 3.50E-08 |
| response to interferon-gamma (GO:0034341) | 33/80 | 5.05E-10 | 3.88E-08 |
| positive regulation of protein phosphorylation (GO:0001 | 93/371 | 5.08E-10 | 3.88E-08 |
| positive regulation of interleukin-12 production (GO:003 | 20/34 | 5.71E-10 | 4.30E-08 |
| response to tumor necrosis factor (GO:0034612) | 40/110 | 7.28E-10 | 5.37E-08 |
| positive regulation of tumor necrosis factor production ( | 32/77 | 7.34E-10 | 5.37E-08 |
| positive regulation of interleukin-1 production (GO:0032 | 28/62 | 8.45E-10 | 6.09E-08 |
| programmed necrotic cell death (GO:0097300) | 14/18 | 9.17E-10 | 6.53E-08 |
| necroptotic process (GO:0070266) | 13/16 | 1.45E-09 | 1.02E-07 |
| regulation of interleukin-1 beta production (GO:0032651 | 33/83 | 1.58E-09 | 1.08E-07 |
| positive regulation of lymphocyte proliferation (GO:0050 | 31/75 | 1.58E-09 | 1.08E-07 |
| regulation of apoptotic process (GO:0042981) | 156/742 | 1.72E-09 | 1.16E-07 |
| regulation of tyrosine phosphorylation of STAT protein (G | 29/68 | 2.21E-09 | 1.47E-07 |
| regulation of NIK/NF-kappaB signaling (GO:1901222) | 32/80 | 2.32E-09 | 1.53E-07 |
| pattern recognition receptor signaling pathway (GO:0002 | 27/61 | 2.92E-09 | 1.90E-07 |
| positive regulation of tumor necrosis factor superfamily c | 32/81 | 3.35E-09 | 2.15E-07 |
| I-kappaB kinase/NF-kappaB signaling (GO:0007249) | 27/62 | 4.52E-09 | 2.86E-07 |
| regulation of response to external stimulus (GO:0032101 | 43/130 | 4.97E-09 | 3.11E-07 |
| positive regulation of immune response (GO:0050778) | 30/75 | 7.30E-09 | 4.51E-07 |
| regulation of programmed necrotic cell death (GO:00620 | 14/20 | 8.94E-09 | 5.46E-07 |
| interferon-gamma-mediated signaling pathway (GO:006( | 28/68 | 1.07E-08 | 6.48E-07 |
| regulation of interferon-gamma-mediated signaling path | 15/23 | 1.14E-08 | 6.82E-07 |
| positive regulation of chemokine production (GO:003272 | 24/53 | 1.26E-08 | 7.40E-07 |
| protein phosphorylation (GO:0006468) | 111/496 | 1.27E-08 | 7.40E-07 |
| positive regulation of interferon-beta production (GO:00 | 19/36 | 1.80E-08 | 1.04E-06 |
| negative regulation of type I interferon production (GO:0 | 21/43 | 1.94E-08 | 1.11E-06 |

| | | | |
|---|---|---|---|
| regulation of interleukin-17 production (GO:0032660) | 18/33 | 2.17E-08 | 1.22E-06 |
| positive regulation of tyrosine phosphorylation of STAT p | 25/59 | 3.28E-08 | 1.83E-06 |
| regulation of interleukin-10 production (GO:0032653) | 22/48 | 3.88E-08 | 2.14E-06 |
| receptor signaling pathway via JAK-STAT (GO:0007259) | 17/31 | 4.83E-08 | 2.64E-06 |
| regulation of response to interferon-gamma (GO:006033 | 44879 | 5.43E-08 | 2.94E-06 |
| phosphorylation (GO:0016310) | 92/400 | 5.81E-08 | 3.11E-06 |
| receptor signaling pathway via STAT (GO:0097696) | 15/25 | 5.86E-08 | 3.11E-06 |
| regulation of chemokine production (GO:0032642) | 20/42 | 7.26E-08 | 3.81E-06 |
| positive regulation of cell migration (GO:0030335) | 68/269 | 7.41E-08 | 3.85E-06 |
| regulation of intracellular signal transduction (GO:19025 | 98/437 | 8.05E-08 | 4.14E-06 |
| negative regulation of viral process (GO:0048525) | 27/70 | 1.00E-07 | 5.10E-06 |
| positive regulation of cell population proliferation (GO:0 | 104/474 | 1.04E-07 | 5.24E-06 |
| negative regulation of DNA-binding transcription factor a | 43/143 | 1.14E-07 | 5.71E-06 |
| lipopolysaccharide-mediated signaling pathway (GO:003 | 13/20 | 1.19E-07 | 5.90E-06 |
| regulation of necroptotic process (GO:0060544) | 14/23 | 1.27E-07 | 6.23E-06 |
| regulation of MAP kinase activity (GO:0043405) | 33/97 | 1.39E-07 | 6.73E-06 |
| apoptotic process (GO:0006915) | 60/231 | 1.55E-07 | 7.44E-06 |
| interleukin-27-mediated signaling pathway (GO:0070106 | 44880 | 1.79E-07 | 8.53E-06 |
| regulation of T cell proliferation (GO:0042129) | 28/76 | 1.82E-07 | 8.62E-06 |
| defense response to bacterium (GO:0042742) | 49/176 | 2.21E-07 | 1.03E-05 |
| positive regulation of macromolecule metabolic process | 87/384 | 2.66E-07 | 1.23E-05 |
| cellular response to interleukin-15 (GO:0071350) | 44847 | 3.24E-07 | 1.47E-05 |
| interleukin-15-mediated signaling pathway (GO:0035723 | 44847 | 3.24E-07 | 1.47E-05 |
| cellular defense response (GO:0006968) | 21/49 | 3.24E-07 | 1.47E-05 |
| positive regulation of NIK/NF-kappaB signaling (GO:1901 | 22/53 | 3.36E-07 | 1.51E-05 |
| regulation of immune response (GO:0050776) | 49/179 | 3.85E-07 | 1.71E-05 |
| positive regulation of T cell proliferation (GO:0042102) | 25/66 | 4.44E-07 | 1.96E-05 |
| cellular response to virus (GO:0098586) | 17/35 | 4.87E-07 | 2.11E-05 |
| positive regulation of lymphocyte activation (GO:005125 | 17/35 | 4.87E-07 | 2.11E-05 |
| tumor necrosis factor-mediated signaling pathway (GO:0 | 36/116 | 5.12E-07 | 2.20E-05 |
| interleukin-35-mediated signaling pathway (GO:0070757 | 44815 | 5.36E-07 | 2.27E-05 |
| positive regulation of nitric-oxide synthase biosynthetic p | 44815 | 5.36E-07 | 2.27E-05 |
| negative regulation of cell population proliferation (GO:0 | 85/379 | 5.72E-07 | 2.39E-05 |
| vascular endothelial growth factor receptor signaling pat | 25/67 | 6.20E-07 | 2.56E-05 |
| negative regulation of apoptotic process (GO:0043066) | 103/485 | 6.21E-07 | 2.56E-05 |
| positive regulation of immune effector process (GO:0002 | 16/32 | 6.42E-07 | 2.62E-05 |
| positive regulation of apoptotic signaling pathway (GO:2 | 23/59 | 7.02E-07 | 2.85E-05 |
| regulation of calcidiol 1-monooxygenase activity (GO:006 | 44749 | 7.18E-07 | 2.89E-05 |
| cellular response to interleukin-9 (GO:0071355) | 44782 | 7.54E-07 | 2.98E-05 |
| interleukin-9-mediated signaling pathway (GO:0038113) | 44782 | 7.54E-07 | 2.98E-05 |
| positive regulation of MAPK cascade (GO:0043410) | 66/274 | 7.57E-07 | 2.98E-05 |
| extrinsic apoptotic signaling pathway (GO:0097191) | 26/72 | 7.79E-07 | 3.04E-05 |
| negative regulation of toll-like receptor signaling pathwa | 17/36 | 8.08E-07 | 3.11E-05 |
| positive regulation of myeloid leukocyte differentiation ( | 17/36 | 8.08E-07 | 3.11E-05 |
| regulation of response to biotic stimulus (GO:0002831) | 15/29 | 8.22E-07 | 3.14E-05 |
| positive regulation of gene expression (GO:0010628) | 102/482 | 8.36E-07 | 3.17E-05 |
| MAPK cascade (GO:0000165) | 71/303 | 8.86E-07 | 3.33E-05 |

| | | | |
|---|---|---|---|
| regulation of extrinsic apoptotic signaling pathway (GO:2 | 24/64 | 9.31E-07 | 3.48E-05 |
| regulation of nitric-oxide synthase biosynthetic process (( | 44848 | 9.98E-07 | 3.70E-05 |
| positive regulation of cysteine-type endopeptidase activi | 36/119 | 1.02E-06 | 3.75E-05 |
| positive regulation of interleukin-17 production (GO:003 | 13/23 | 1.19E-06 | 4.36E-05 |
| positive regulation of interferon-alpha production (GO:0( | 44915 | 1.30E-06 | 4.68E-05 |
| toll-like receptor 4 signaling pathway (GO:0034142) | 44915 | 1.30E-06 | 4.68E-05 |
| negative regulation of transcription, DNA-templated (GO | 176/948 | 1.51E-06 | 5.41E-05 |
| regulation of angiogenesis (GO:0045765) | 52/203 | 1.59E-06 | 5.66E-05 |
| negative regulation of transcription by RNA polymerase I | 134/684 | 1.62E-06 | 5.71E-05 |
| negative regulation of NF-kappaB transcription factor act | 27/79 | 1.67E-06 | 5.88E-05 |
| positive regulation of cell death (GO:0010942) | 24/66 | 1.78E-06 | 6.19E-05 |
| cellular response to vascular endothelial growth factor st | 16/34 | 1.81E-06 | 6.26E-05 |
| positive regulation of toll-like receptor signaling pathway | 14/27 | 1.85E-06 | 6.37E-05 |
| positive regulation of T-helper 1 type immune response ( | 44816 | 1.89E-06 | 6.42E-05 |
| positive regulation of T-helper 17 type immune response | 44816 | 1.89E-06 | 6.42E-05 |
| stress-activated MAPK cascade (GO:0051403) | 23/62 | 1.95E-06 | 6.56E-05 |
| negative regulation of innate immune response (GO:004! | 17/38 | 2.08E-06 | 6.97E-05 |
| negative regulation of viral genome replication (GO:0045 | 21/54 | 2.22E-06 | 7.40E-05 |
| interleukin-1-mediated signaling pathway (GO:0070498) | 30/94 | 2.33E-06 | 7.71E-05 |
| negative regulation of interleukin-6 production (GO:0032 | 15/31 | 2.45E-06 | 8.01E-05 |
| positive regulation of leukocyte cell-cell adhesion (GO:19 | 15/31 | 2.45E-06 | 8.01E-05 |
| negative regulation of defense response (GO:0031348) | 28/85 | 2.49E-06 | 8.09E-05 |
| regulation of gene expression (GO:0010468) | 195/1079 | 2.64E-06 | 8.47E-05 |
| negative regulation of programmed cell death (GO:0043( | 83/381 | 2.64E-06 | 8.47E-05 |
| positive regulation of T cell cytokine production (GO:000 | 44916 | 2.66E-06 | 8.47E-05 |
| negative regulation of inflammatory response (GO:00507 | 53/212 | 2.79E-06 | 8.84E-05 |
| regulation of cytokine production involved in inflammato | 18/43 | 3.38E-06 | 1.06E-04 |
| positive regulation of neuron death (GO:1901216) | 19/47 | 3.40E-06 | 1.06E-04 |
| negative regulation of multicellular organismal process (( | 53/214 | 3.76E-06 | 1.17E-04 |
| regulation of cell migration (GO:0030334) | 87/408 | 3.82E-06 | 1.18E-04 |
| positive regulation of interleukin-10 production (GO:003 | 15/32 | 4.05E-06 | 1.25E-04 |
| regulation of interferon-alpha production (GO:0032647) | 13/25 | 4.18E-06 | 1.28E-04 |
| negative regulation of cellular process (GO:0048523) | 113/566 | 4.25E-06 | 1.29E-04 |
| positive regulation of cellular catabolic process (GO:0031 | 39/141 | 4.34E-06 | 1.30E-04 |
| regulation of small GTPase mediated signal transduction | 39/141 | 4.34E-06 | 1.30E-04 |
| cell chemotaxis (GO:0060326) | 24/69 | 4.39E-06 | 1.30E-04 |
| regulation of protein-containing complex assembly (GO:( | 34/116 | 4.39E-06 | 1.30E-04 |
| negative regulation of gene expression (GO:0010629) | 72/322 | 4.55E-06 | 1.34E-04 |
| positive regulation of cytokine-mediated signaling pathw | 16/36 | 4.61E-06 | 1.35E-04 |
| negative regulation of immune response (GO:0050777) | 46/178 | 4.81E-06 | 1.40E-04 |
| negative regulation of MAP kinase activity (GO:0043407) | 19/48 | 4.93E-06 | 1.42E-04 |
| negative regulation of tumor necrosis factor superfamily | 19/48 | 4.93E-06 | 1.42E-04 |
| regulation of interleukin-2 production (GO:0032663) | 19/48 | 4.93E-06 | 1.42E-04 |
| calcium-mediated signaling (GO:0019722) | 31/102 | 5.02E-06 | 1.43E-04 |
| cellular response to interleukin-21 (GO:0098757) | 44750 | 5.08E-06 | 1.43E-04 |
| interleukin-21-mediated signaling pathway (GO:0038114 | 44750 | 5.08E-06 | 1.43E-04 |
| regulation of MyD88-independent toll-like receptor signa | 44750 | 5.08E-06 | 1.43E-04 |

| | | | |
|---|---|---|---|
| positive regulation of adaptive immune response based c | 44817 | 5.42E-06 | 1.49E-04 |
| response to muramyl dipeptide (GO:0032495) | 44817 | 5.42E-06 | 1.49E-04 |
| cellular response to lipoteichoic acid (GO:0071223) | 44718 | 5.42E-06 | 1.49E-04 |
| response to lipoteichoic acid (GO:0070391) | 44718 | 5.42E-06 | 1.49E-04 |
| myeloid cell activation involved in immune response (GO | 44884 | 5.93E-06 | 1.62E-04 |
| positive regulation of protein kinase activity (GO:004586( | 41/154 | 7.05E-06 | 1.91E-04 |
| regulation of smooth muscle cell proliferation (GO:00486 | 19/49 | 7.06E-06 | 1.91E-04 |
| negative regulation of MAPK cascade (GO:0043409) | 29/94 | 7.24E-06 | 1.95E-04 |
| positive regulation of interleukin-2 production (GO:0032 | 13/26 | 7.34E-06 | 1.96E-04 |
| positive regulation of signal transduction (GO:0009967) | 59/252 | 7.37E-06 | 1.96E-04 |
| positive regulation of programmed cell death (GO:00430 | 65/286 | 7.42E-06 | 1.97E-04 |
| positive regulation of apoptotic process (GO:0043065) | 69/310 | 8.39E-06 | 2.21E-04 |
| positive regulation of small GTPase mediated signal trans | 14/30 | 9.08E-06 | 2.38E-04 |
| negative regulation of tumor necrosis factor production ( | 18/46 | 1.06E-05 | 2.76E-04 |
| cellular response to interleukin-2 (GO:0071352) | 44784 | 1.08E-05 | 2.76E-04 |
| interleukin-2-mediated signaling pathway (GO:0038110) | 44784 | 1.08E-05 | 2.76E-04 |
| negative regulation of programmed necrotic cell death (G | 44784 | 1.08E-05 | 2.76E-04 |
| positive regulation of production of molecular mediator c | 16/38 | 1.08E-05 | 2.76E-04 |
| regulation of toll-like receptor signaling pathway (GO:003 | 13/27 | 1.24E-05 | 3.17E-04 |
| positive regulation of cytokine production involved in infl | 44851 | 1.32E-05 | 3.35E-04 |
| defense response to protozoan (GO:0042832) | 44818 | 1.34E-05 | 3.37E-04 |
| cellular response to chemokine (GO:1990869) | 21/60 | 1.54E-05 | 3.86E-04 |
| peptidyl-tyrosine dephosphorylation (GO:0035335) | 15/35 | 1.57E-05 | 3.93E-04 |
| protein dephosphorylation (GO:0006470) | 37/139 | 1.94E-05 | 4.82E-04 |
| interleukin-23-mediated signaling pathway (GO:0038155 | 44751 | 2.02E-05 | 4.97E-04 |
| negative regulation of lipid localization (GO:1905953) | 44751 | 2.02E-05 | 4.97E-04 |
| cellular response to interleukin-6 (GO:0071354) | 13/28 | 2.04E-05 | 5.00E-04 |
| regulation of protein phosphorylation (GO:0001932) | 60/266 | 2.08E-05 | 5.07E-04 |
| cellular response to interleukin-12 (GO:0071349) | 18/48 | 2.10E-05 | 5.11E-04 |
| regulation of ERK1 and ERK2 cascade (GO:0070372) | 55/238 | 2.20E-05 | 5.31E-04 |
| macrophage activation (GO:0042116) | 15/36 | 2.36E-05 | 5.69E-04 |
| regulation of GTPase activity (GO:0043087) | 46/189 | 2.57E-05 | 6.14E-04 |
| myeloid leukocyte differentiation (GO:0002573) | 23/71 | 2.61E-05 | 6.21E-04 |
| negative regulation of interleukin-8 production (GO:0032 | 44852 | 2.62E-05 | 6.21E-04 |
| positive regulation of B cell activation (GO:0050871) | 19/53 | 2.63E-05 | 6.21E-04 |
| positive regulation of cysteine-type endopeptidase activi | 21/62 | 2.72E-05 | 6.39E-04 |
| protein mono-ADP-ribosylation (GO:0140289) | 44785 | 2.85E-05 | 6.68E-04 |
| cellular response to mechanical stimulus (GO:0071260) | 18/49 | 2.92E-05 | 6.79E-04 |
| activation of cysteine-type endopeptidase activity involve | 25/81 | 3.00E-05 | 6.96E-04 |
| regulation of viral genome replication (GO:0045069) | 22/67 | 3.06E-05 | 7.07E-04 |
| regulation of tumor necrosis factor-mediated signaling pa | 20/58 | 3.13E-05 | 7.18E-04 |
| zymogen activation (GO:0031638) | 17/45 | 3.18E-05 | 7.27E-04 |
| positive regulation of angiogenesis (GO:0045766) | 32/116 | 3.21E-05 | 7.32E-04 |
| MyD88-dependent toll-like receptor signaling pathway (G | 13/29 | 3.25E-05 | 7.37E-04 |
| negative regulation of MyD88-independent toll-like recep | 44719 | 3.36E-05 | 7.53E-04 |
| positive regulation of T-helper 17 cell differentiation (GO | 44719 | 3.36E-05 | 7.53E-04 |
| regulation of protein deubiquitination (GO:0090085) | 44719 | 3.36E-05 | 7.53E-04 |

| | | | |
|---|---|---|---|
| positive regulation of signaling receptor activity (GO:200( | 15/37 | 3.49E-05 | 7.78E-04 |
| positive regulation of protein modification process (GO:0 | 50/214 | 3.75E-05 | 8.32E-04 |
| osteoclast differentiation (GO:0030316) | 44887 | 3.77E-05 | 8.34E-04 |
| intrinsic apoptotic signaling pathway (GO:0097193) | 29/102 | 4.04E-05 | 8.89E-04 |
| monocyte extravasation (GO:0035696) | 44686 | 4.09E-05 | 8.97E-04 |
| cellular response to organic substance (GO:0071310) | 33/123 | 4.50E-05 | 9.82E-04 |
| negative regulation of protein serine/threonine kinase ac | 24/78 | 4.54E-05 | 9.87E-04 |
| metal ion transport (GO:0030001) | 26/88 | 4.86E-05 | 0.00105119 |
| regulation of peptidyl-serine phosphorylation (GO:00331 | 28/98 | 4.95E-05 | 0.001065956 |
| positive regulation of MAP kinase activity (GO:0043406) | 22/69 | 5.10E-05 | 0.001091459 |
| negative regulation of macromolecule metabolic process | 46/194 | 5.13E-05 | 0.001091459 |
| regulation of programmed cell death (GO:0043067) | 46/194 | 5.13E-05 | 0.001091459 |
| Fc receptor mediated stimulatory signaling pathway (GO: | 23/74 | 5.45E-05 | 0.001154643 |
| dendritic cell chemotaxis (GO:0002407) | 44820 | 5.94E-05 | 0.001242686 |
| positive regulation of toll-like receptor 4 signaling pathwa | 44752 | 5.96E-05 | 0.001242686 |
| regulation of T-helper 17 cell differentiation (GO:200031! | 44752 | 5.96E-05 | 0.001242686 |
| regulation of T-helper 17 type immune response (GO:200( | 44752 | 5.96E-05 | 0.001242686 |
| regulation of cysteine-type endopeptidase activity involv | 26/89 | 6.00E-05 | 0.001242686 |
| second-messenger-mediated signaling (GO:0019932) | 26/89 | 6.00E-05 | 0.001242686 |
| hemopoiesis (GO:0030097) | 27/94 | 6.05E-05 | 0.001246689 |
| regulation of T cell activation (GO:0050863) | 17/47 | 6.07E-05 | 0.001246689 |
| regulation of toll-like receptor 4 signaling pathway (GO:0 | 44888 | 6.36E-05 | 0.001301622 |
| mature B cell differentiation involved in immune respons | 44786 | 6.55E-05 | 0.001335247 |
| integrin-mediated signaling pathway (GO:0007229) | 23/75 | 6.88E-05 | 0.001395703 |
| myeloid cell differentiation (GO:0030099) | 18/52 | 7.26E-05 | 0.001451666 |
| regulation of intrinsic apoptotic signaling pathway (GO:2( | 18/52 | 7.26E-05 | 0.001451666 |
| cytoplasmic pattern recognition receptor signaling pathw | 44922 | 7.27E-05 | 0.001451666 |
| regulation of vascular endothelial growth factor producti | 44922 | 7.27E-05 | 0.001451666 |
| cellular response to growth factor stimulus (GO:0071363 | 39/158 | 7.36E-05 | 0.001465249 |
| cellular response to hydrogen peroxide (GO:0070301) | 14/35 | 7.57E-05 | 0.001501427 |
| Fc-epsilon receptor signaling pathway (GO:0038095) | 30/111 | 8.45E-05 | 0.00166878 |
| growth hormone receptor signaling pathway (GO:00603! | 44854 | 8.59E-05 | 0.001684714 |
| negative regulation of lipid storage (GO:0010888) | 44854 | 8.59E-05 | 0.001684714 |
| positive regulation of cell motility (GO:2000147) | 50/221 | 8.99E-05 | 0.001754987 |
| positive regulation of nucleic acid-templated transcriptio | 98/511 | 9.17E-05 | 0.001784575 |
| regulation of nitric oxide biosynthetic process (GO:00454 | 15/40 | 1.02E-04 | 0.001967529 |
| regulation of cytokine production involved in immune re: | 44889 | 1.03E-04 | 0.001997466 |
| Fc-gamma receptor signaling pathway (GO:0038094) | 22/72 | 1.04E-04 | 0.002005203 |
| positive regulation of vasculature development (GO:190⁄ | 28/102 | 1.07E-04 | 0.002051766 |
| neutrophil migration (GO:1990266) | 23/77 | 1.08E-04 | 0.00206015 |
| positive regulation of cellular component biogenesis (GO | 26/92 | 1.10E-04 | 0.002097564 |
| response to hydrogen peroxide (GO:0042542) | 17/49 | 1.11E-04 | 0.002097564 |
| negative regulation of interleukin-10 production (GO:003 | 44821 | 1.11E-04 | 0.002097564 |
| positive regulation of defense response to virus by host ( | 44923 | 1.12E-04 | 0.002097564 |
| regulation of B cell activation (GO:0050864) | 44923 | 1.12E-04 | 0.002097564 |
| positive regulation of protein-containing complex assemb | 31/118 | 1.15E-04 | 0.002155633 |
| cellular response to reactive oxygen species (GO:003461⁄ | 20/63 | 1.18E-04 | 0.002182959 |

| | | | |
|---|---|---|---|
| leukocyte aggregation (GO:0070486) | 44720 | 1.19E-04 | 0.002182959 |
| positive regulation of apoptotic cell clearance (GO:20004 | 44720 | 1.19E-04 | 0.002182959 |
| positive regulation of cysteine-type endopeptidase activi | 44720 | 1.19E-04 | 0.002182959 |
| regulation of apoptotic cell clearance (GO:2000425) | 44720 | 1.19E-04 | 0.002182959 |
| T-helper cell lineage commitment (GO:0002295) | 44720 | 1.19E-04 | 0.002182959 |
| positive regulation of macromolecule biosynthetic proces | 33/129 | 1.23E-04 | 0.002247627 |
| positive regulation of Notch signaling pathway (GO:0045 | 16/45 | 1.26E-04 | 0.002284719 |
| regulation of humoral immune response (GO:0002920) | 18/54 | 1.27E-04 | 0.002295807 |
| granulocyte chemotaxis (GO:0071621) | 22/73 | 1.31E-04 | 0.002357322 |
| growth hormone receptor signaling pathway via JAK-STAT | 44787 | 1.35E-04 | 0.002403545 |
| positive regulation of granulocyte macrophage colony-sti | 44787 | 1.35E-04 | 0.002403545 |
| positive regulation of T-helper cell differentiation (GO:00 | 44787 | 1.35E-04 | 0.002403545 |
| positive regulation of type 2 immune response (GO:0002 | 44787 | 1.35E-04 | 0.002403545 |
| purinergic nucleotide receptor signaling pathway (GO:00 | 44855 | 1.45E-04 | 0.002553451 |
| PERK-mediated unfolded protein response (GO:0036499) | 44753 | 1.45E-04 | 0.002553451 |
| T cell chemotaxis (GO:0010818) | 44753 | 1.45E-04 | 0.002553451 |
| negative regulation of ERK1 and ERK2 cascade (GO:0070 | 17/50 | 1.47E-04 | 0.002570988 |
| regulation of complement activation (GO:0030449) | 17/50 | 1.47E-04 | 0.002570988 |
| regulation of autophagy (GO:0010506) | 51/231 | 1.47E-04 | 0.002570988 |
| negative regulation of intrinsic apoptotic signaling pathw | 20/64 | 1.50E-04 | 0.002613699 |
| platelet degranulation (GO:0002576) | 32/125 | 1.53E-04 | 0.002641089 |
| positive regulation of GTPase activity (GO:0043547) | 48/214 | 1.53E-04 | 0.002641089 |
| antigen processing and presentation of peptide antigen v | 13/33 | 1.64E-04 | 0.002818356 |
| negative regulation of cysteine-type endopeptidase activ | 18/55 | 1.65E-04 | 0.002835348 |
| positive regulation of extrinsic apoptotic signaling pathwa | 44924 | 1.68E-04 | 0.002859056 |
| protein ADP-ribosylation (GO:0006471) | 44924 | 1.68E-04 | 0.002859056 |
| interleukin-12-mediated signaling pathway (GO:0035722 | 16/46 | 1.69E-04 | 0.00287425 |
| response to organic cyclic compound (GO:0014070) | 19/60 | 1.80E-04 | 0.003041746 |
| regulation of extrinsic apoptotic signaling pathway via de | 15/42 | 1.92E-04 | 0.003238996 |
| dendritic cell migration (GO:0036336) | 44822 | 1.97E-04 | 0.003273682 |
| interleukin-6-mediated signaling pathway (GO:0070102) | 44822 | 1.97E-04 | 0.003273682 |
| regulation of adaptive immune response (GO:0002819) | 44822 | 1.97E-04 | 0.003273682 |
| T cell migration (GO:0072678) | 44822 | 1.97E-04 | 0.003273682 |
| neutrophil chemotaxis (GO:0030593) | 21/70 | 1.98E-04 | 0.003282891 |
| receptor-mediated endocytosis (GO:0006898) | 35/143 | 2.00E-04 | 0.003316188 |
| positive regulation of hydrolase activity (GO:0051345) | 36/149 | 2.16E-04 | 0.003537951 |
| regulation of NK T cell activation (GO:0051133) | 44687 | 2.19E-04 | 0.003537951 |
| regulation of vitamin D biosynthetic process (GO:006055 | 44687 | 2.19E-04 | 0.003537951 |
| response to peptidoglycan (GO:0032494) | 44687 | 2.19E-04 | 0.003537951 |
| T-helper 17 cell lineage commitment (GO:0072540) | 44687 | 2.19E-04 | 0.003537951 |
| vesicle fusion with endoplasmic reticulum-Golgi intermed | 44687 | 2.19E-04 | 0.003537951 |
| positive regulation of protein serine/threonine kinase act | 28/106 | 2.19E-04 | 0.003537951 |
| cellular response to chemical stress (GO:0062197) | 27/101 | 2.27E-04 | 0.003667672 |
| negative regulation of extrinsic apoptotic signaling pathw | 13/34 | 2.33E-04 | 0.003734465 |
| regulation of activated T cell proliferation (GO:0046006) | 13/34 | 2.33E-04 | 0.003734465 |
| regulation of lipid storage (GO:0010883) | 44856 | 2.34E-04 | 0.003734465 |
| positive regulation of phosphorylation (GO:0042327) | 54/253 | 2.39E-04 | 0.00380571 |

| | | | |
|---|---|---|---|
| Fc-gamma receptor signaling pathway involved in phagoc | 21/71 | 2.46E-04 | 0.003910869 |
| Fc receptor signaling pathway (GO:0038093) | 29/112 | 2.47E-04 | 0.003912636 |
| positive regulation of acute inflammatory response (GO:( | 44788 | 2.56E-04 | 0.004026938 |
| positive regulation of osteoclast differentiation (GO:0045 | 44788 | 2.56E-04 | 0.004026938 |
| cellular response to peptide (GO:1901653) | 18/57 | 2.74E-04 | 0.004302193 |
| negative regulation of response to external stimulus (GO: | 30/118 | 2.75E-04 | 0.004306374 |
| antigen receptor-mediated signaling pathway (GO:00508 | 42/185 | 2.92E-04 | 0.004547806 |
| regulation of viral entry into host cell (GO:0046596) | 14/39 | 2.94E-04 | 0.004551847 |
| response to peptide (GO:1901652) | 14/39 | 2.94E-04 | 0.004551847 |
| positive regulation of phosphatidylinositol 3-kinase signa | 22/77 | 3.06E-04 | 0.004711699 |
| positive regulation of protein modification by small prote | 22/77 | 3.06E-04 | 0.004711699 |
| cellular response to muramyl dipeptide (GO:0071225) | 44721 | 3.18E-04 | 0.004813168 |
| negative regulation of glycoprotein biosynthetic process ( | 44721 | 3.18E-04 | 0.004813168 |
| negative regulation of necroptotic process (GO:0060546) | 44721 | 3.18E-04 | 0.004813168 |
| positive regulation of protein polyubiquitination (GO:190 | 44721 | 3.18E-04 | 0.004813168 |
| regulation of T-helper 1 type immune response (GO:0002 | 44721 | 3.18E-04 | 0.004813168 |
| positive regulation of cellular biosynthetic process (GO:0( | 41/180 | 3.18E-04 | 0.004813168 |
| negative regulation of cysteine-type endopeptidase activ | 17/53 | 3.27E-04 | 0.004918656 |
| regulation of immune effector process (GO:0002697) | 17/53 | 3.27E-04 | 0.004918656 |
| cellular response to interleukin-7 (GO:0098761) | 44823 | 3.30E-04 | 0.004921342 |
| interleukin-7-mediated signaling pathway (GO:0038111) | 44823 | 3.30E-04 | 0.004921342 |
| leukocyte adhesion to vascular endothelial cell (GO:0061 | 44823 | 3.30E-04 | 0.004921342 |
| positive regulation of peptidyl-serine phosphorylation (G | 25/93 | 3.48E-04 | 0.00517847 |
| negative regulation of peptidyl-tyrosine phosphorylation | 44926 | 3.53E-04 | 0.005246353 |
| negative regulation of inflammatory response to antigeni | 33/136 | 3.56E-04 | 0.005261845 |
| regulation of Notch signaling pathway (GO:0008593) | 23/83 | 3.68E-04 | 0.005423795 |
| positive regulation of nitric oxide biosynthetic process (G | 44892 | 3.69E-04 | 0.005429344 |
| dephosphorylation (GO:0016311) | 36/153 | 3.73E-04 | 0.005472526 |
| defense response to Gram-negative bacterium (GO:0050 | 21/73 | 3.75E-04 | 0.005485714 |
| protein autophosphorylation (GO:0046777) | 37/159 | 3.92E-04 | 0.005723792 |
| negative regulation of I-kappaB kinase/NF-kappaB signali | 14/40 | 3.97E-04 | 0.00577598 |
| calcium ion transport (GO:0006816) | 29/115 | 3.99E-04 | 0.005796975 |
| regulation of cytosolic calcium ion concentration (GO:005 | 35/148 | 4.04E-04 | 0.005840093 |
| regulation of inflammatory response to antigenic stimulu | 33/137 | 4.10E-04 | 0.005911627 |
| positive regulation of leukocyte chemotaxis (GO:000269( | 17/54 | 4.18E-04 | 0.006021815 |
| regulation of protein kinase activity (GO:0045859) | 28/110 | 4.24E-04 | 0.006061192 |
| response to endoplasmic reticulum stress (GO:0034976) | 28/110 | 4.24E-04 | 0.006061192 |
| negative regulation of autophagy (GO:0010507) | 18/59 | 4.40E-04 | 0.00628436 |
| positive regulation of innate immune response (GO:0045 | 13/36 | 4.49E-04 | 0.006376629 |
| regulation of defense response to virus by host (GO:005( | 13/36 | 4.49E-04 | 0.006376629 |
| positive regulation of alpha-beta T cell proliferation (GO:( | 44789 | 4.52E-04 | 0.00638319 |
| regulation of granulocyte macrophage colony-stimulating | 44789 | 4.52E-04 | 0.00638319 |
| positive regulation of response to biotic stimulus (GO:00( | 15/45 | 4.55E-04 | 0.006405658 |
| protein polyubiquitination (GO:0000209) | 63/314 | 4.60E-04 | 0.006464644 |
| negative regulation of nucleic acid-templated transcriptic | 87/464 | 4.74E-04 | 0.006636169 |
| regulation of protein serine/threonine kinase activity (GC | 28/111 | 4.96E-04 | 0.006923082 |
| regulation of chemotaxis (GO:0050920) | 12024 | 4.98E-04 | 0.006923082 |

| | | | |
|---|---|---|---|
| regulation of protein kinase B signaling (GO:0051896) | 45/207 | 4.99E-04 | 0.006923082 |
| regulation of cellular component movement (GO:005127 | 16/50 | 5.00E-04 | 0.006923082 |
| positive regulation of protein kinase B signaling (GO:0051 | 37/161 | 5.07E-04 | 0.006998137 |
| cellular response to organic cyclic compound (GO:00714( | 35/150 | 5.26E-04 | 0.007243679 |
| adaptive immune response based on somatic recombinat | 44824 | 5.29E-04 | 0.007243679 |
| regulation of T cell migration (GO:2000404) | 44824 | 5.29E-04 | 0.007243679 |
| cortical cytoskeleton organization (GO:0030865) | 14/41 | 5.30E-04 | 0.007243679 |
| nucleotide-binding oligomerization domain containing sig | 44893 | 5.35E-04 | 0.0072794 |
| positive regulation of nitric oxide metabolic process (GO: | 44893 | 5.35E-04 | 0.0072794 |
| negative regulation of extrinsic apoptotic signaling pathw | 22/80 | 5.48E-04 | 0.007431382 |
| negative regulation of NIK/NF-kappaB signaling (GO:1901 | 44858 | 5.51E-04 | 0.007448473 |
| Notch signaling pathway (GO:0007219) | 18/60 | 5.52E-04 | 0.007448473 |
| macrophage activation involved in immune response (GC | 44755 | 5.91E-04 | 0.007881934 |
| negative regulation of endoplasmic reticulum unfolded p | 44755 | 5.91E-04 | 0.007881934 |
| positive regulation of mRNA metabolic process (GO:1903 | 44755 | 5.91E-04 | 0.007881934 |
| toll-like receptor 9 signaling pathway (GO:0034162) | 44755 | 5.91E-04 | 0.007881934 |
| positive regulation of smooth muscle cell proliferation (G | 15/46 | 5.93E-04 | 0.007881934 |
| regulation of B cell proliferation (GO:0030888) | 15/46 | 5.93E-04 | 0.007881934 |
| regulation of p38MAPK cascade (GO:1900744) | 13/37 | 6.09E-04 | 0.008074049 |
| regulation of Ras protein signal transduction (GO:004657 | 23/86 | 6.37E-04 | 0.008411456 |
| intrinsic apoptotic signaling pathway in response to DNA | 16/51 | 6.40E-04 | 0.008411456 |
| regulation of protein modification process (GO:0031399) | 16/51 | 6.40E-04 | 0.008411456 |
| death-inducing signaling complex assembly (GO:0071550 | 44688 | 6.81E-04 | 0.008774451 |
| marginal zone B cell differentiation (GO:0002315) | 44688 | 6.81E-04 | 0.008774451 |
| negative regulation of p38MAPK cascade (GO:1903753) | 44688 | 6.81E-04 | 0.008774451 |
| positive regulation of chemokine (C-X-C motif) ligand 2 pı | 44688 | 6.81E-04 | 0.008774451 |
| positive regulation of NK T cell activation (GO:0051135) | 44688 | 6.81E-04 | 0.008774451 |
| regulation of natural killer cell proliferation (GO:0032817 | 44688 | 6.81E-04 | 0.008774451 |
| regulation of prostaglandin biosynthetic process (GO:003 | 44688 | 6.81E-04 | 0.008774451 |
| T-helper 17 cell differentiation (GO:0072539) | 44688 | 6.81E-04 | 0.008774451 |
| positive regulation of cytokine production involved in imı | 12389 | 6.89E-04 | 0.008854533 |
| granulocyte activation (GO:0036230) | 44722 | 7.05E-04 | 0.008994136 |
| positive regulation of tissue remodeling (GO:0034105) | 44722 | 7.05E-04 | 0.008994136 |
| regulation of neutrophil migration (GO:1902622) | 44722 | 7.05E-04 | 0.008994136 |
| T cell receptor signaling pathway (GO:0050852) | 36/158 | 7.08E-04 | 0.009019137 |
| innate immune response activating cell surface receptor | 29/119 | 7.29E-04 | 0.009256443 |
| positive regulation of transcription of Notch receptor tar | 44790 | 7.55E-04 | 0.009543891 |
| regulation of leukocyte migration (GO:0002685) | 44790 | 7.55E-04 | 0.009543891 |
| epidermal growth factor receptor signaling pathway (GO: | 15/47 | 7.66E-04 | 0.009654603 |
| regulation of cell growth (GO:0001558) | 46/217 | 7.73E-04 | 0.009718419 |
| regulation of phosphorylation (GO:0042325) | 30/125 | 7.81E-04 | 0.009797173 |
| positive regulation of T cell migration (GO:2000406) | 44859 | 8.10E-04 | 0.01008499 |
| positive regulation of vascular endothelial growth factor | 44859 | 8.10E-04 | 0.01008499 |
| regulation of heterotypic cell-cell adhesion (GO:0034114 | 44859 | 8.10E-04 | 0.01008499 |
| response to peptide hormone (GO:0043434) | 16/52 | 8.11E-04 | 0.01008499 |
| positive regulation of protein metabolic process (GO:005 | 28/115 | 9.05E-04 | 0.01121854 |
| positive regulation of pri-miRNA transcription by RNA pol | 12754 | 9.37E-04 | 0.011589326 |

| | | | |
|---|---|---|---|
| transmembrane receptor protein tyrosine kinase signalin | 76/404 | 9.47E-04 | 0.011684899 |
| positive regulation of stress-activated MAPK cascade (GC | 25/99 | 9.51E-04 | 0.011701612 |
| protein homooligomerization (GO:0051260) | 29/121 | 9.69E-04 | 0.011896567 |
| positive regulation of epithelial cell migration (GO:00106 | 24/94 | 0.001008061 | 0.012292612 |
| negative regulation of cell adhesion (GO:0007162) | 20/73 | 0.001010384 | 0.012292612 |
| positive regulation of JNK cascade (GO:0046330) | 20/73 | 0.001010384 | 0.012292612 |
| regulation of actin filament-based process (GO:0032970) | 20/73 | 0.001010384 | 0.012292612 |
| regulation of JNK cascade (GO:0046328) | 26/105 | 0.001039865 | 0.012530394 |
| actin cytoskeleton reorganization (GO:0031532) | 18/63 | 0.001044496 | 0.012530394 |
| positive regulation of Ras protein signal transduction (GC | 18/63 | 0.001044496 | 0.012530394 |
| activation of cysteine-type endopeptidase activity (GO:0( | 44756 | 0.001046812 | 0.012530394 |
| microvillus organization (GO:0032528) | 44756 | 0.001046812 | 0.012530394 |
| myeloid dendritic cell activation (GO:0001773) | 44756 | 0.001046812 | 0.012530394 |
| regulation of IRE1-mediated unfolded protein response (( | 44756 | 0.001046812 | 0.012530394 |
| positive regulation of B cell proliferation (GO:0030890) | 44895 | 0.001057173 | 0.012596359 |
| regulation of type I interferon-mediated signaling pathwa | 44895 | 0.001057173 | 0.012596359 |
| negative regulation of cellular macromolecule biosynthet | 98/547 | 0.001068245 | 0.01269916 |
| membrane protein proteolysis (GO:0033619) | 13/39 | 0.001075325 | 0.012754143 |
| peptidyl-serine phosphorylation (GO:0018105) | 35/156 | 0.001114018 | 0.013182967 |
| leukocyte tethering or rolling (GO:0050901) | 44791 | 0.001201823 | 0.014157528 |
| positive regulation of amyloid-beta formation (GO:19020 | 44791 | 0.001201823 | 0.014157528 |
| regulation of phosphatidylinositol 3-kinase signaling (GO: | 26/106 | 0.00120697 | 0.014185991 |
| negative regulation of B cell activation (GO:0050869) | 44826 | 0.00122166 | 0.014229875 |
| peptidyl-threonine dephosphorylation (GO:0035970) | 44826 | 0.00122166 | 0.014229875 |
| positive regulation of amyloid precursor protein catabolic | 44826 | 0.00122166 | 0.014229875 |
| regulation of platelet activation (GO:0010543) | 44826 | 0.00122166 | 0.014229875 |
| positive regulation of reactive oxygen species metabolic | | 15/49 | 0.001239925 | 0.014378144 |
| receptor internalization (GO:0031623) | 15/49 | 0.001239925 | 0.014378144 |
| negative regulation of protein kinase activity (GO:000646 | 23/90 | 0.001253687 | 0.014462528 |
| positive regulation of cell cycle G1/S phase transition (GC | 13119 | 0.001255553 | 0.014462528 |
| regulation of JUN kinase activity (GO:0043506) | 13119 | 0.001255553 | 0.014462528 |
| regulation of cytoskeleton organization (GO:0051493) | 27/112 | 0.001297108 | 0.014908132 |
| copper ion transport (GO:0006825) | 44723 | 0.001376217 | 0.015138789 |
| negative regulation of interleukin-17 production (GO:003 | 44723 | 0.001376217 | 0.015138789 |
| negative regulation of T-helper cell differentiation (GO:0( | 44723 | 0.001376217 | 0.015138789 |
| positive regulation of epidermal growth factor-activated | 44723 | 0.001376217 | 0.015138789 |
| regulation of cysteine-type endopeptidase activity involv | 44723 | 0.001376217 | 0.015138789 |
| tryptophan metabolic process (GO:0006568) | 44723 | 0.001376217 | 0.015138789 |
| cadmium ion transmembrane transport (GO:0070574) | 44656 | 0.001381287 | 0.015138789 |
| cadmium ion transport (GO:0015691) | 44656 | 0.001381287 | 0.015138789 |
| cellular response to nicotine (GO:0071316) | 44656 | 0.001381287 | 0.015138789 |
| copper ion import (GO:0015677) | 44656 | 0.001381287 | 0.015138789 |
| endothelin receptor signaling pathway (GO:0086100) | 44656 | 0.001381287 | 0.015138789 |
| negative regulation of PERK-mediated unfolded protein r | 44656 | 0.001381287 | 0.015138789 |
| negative regulation of vitamin D biosynthetic process (GC | 44656 | 0.001381287 | 0.015138789 |
| p38MAPK cascade (GO:0038066) | 44656 | 0.001381287 | 0.015138789 |
| positive regulation of natural killer cell proliferation (GO:( | 44656 | 0.001381287 | 0.015138789 |

| | | | |
|---|---|---|---|
| positive regulation of prostaglandin biosynthetic process | 44656 | 0.001381287 | 0.015138789 |
| positive regulation of T-helper 1 cell cytokine production | 44656 | 0.001381287 | 0.015138789 |
| positive regulation of transcription from RNA polymerase | 44656 | 0.001381287 | 0.015138789 |
| regulation of receptor catabolic process (GO:2000644) | 44656 | 0.001381287 | 0.015138789 |
| regulation of sodium:proton antiporter activity (GO:0032 | 44656 | 0.001381287 | 0.015138789 |
| regulation of T-helper 1 cell cytokine production (GO:200 | 44656 | 0.001381287 | 0.015138789 |
| toll-like receptor 2 signaling pathway (GO:0034134) | 44656 | 0.001381287 | 0.015138789 |
| cellular response to starvation (GO:0009267) | 35/158 | 0.001409532 | 0.015415828 |
| negative regulation of response to biotic stimulus (GO:00 | 11628 | 0.001444216 | 0.015728938 |
| positive regulation of mononuclear cell migration (GO:00 | 11628 | 0.001444216 | 0.015728938 |
| positive regulation of RNA metabolic process (GO:00512! | 14/45 | 0.001500297 | 0.01622914 |
| regulation of pri-miRNA transcription by RNA polymerase | 14/45 | 0.001500297 | 0.01622914 |
| regulation of receptor signaling pathway via JAK-STAT (G | 14/45 | 0.001500297 | 0.01622914 |
| positive regulation of cytosolic calcium ion concentration | 33/147 | 0.00150264 | 0.01622914 |
| establishment of protein localization to membrane (GO:0 | 19/70 | 0.001506937 | 0.016241775 |
| cellular calcium ion homeostasis (GO:0006874) | 31/136 | 0.001588285 | 0.017065799 |
| actin filament reorganization (GO:0090527) | 44689 | 0.001616241 | 0.017065799 |
| antigen processing and presentation of endogenous pept | 44689 | 0.001616241 | 0.017065799 |
| positive regulation of glial cell migration (GO:1903977) | 44689 | 0.001616241 | 0.017065799 |
| positive regulation of MHC class II biosynthetic process (( | 44689 | 0.001616241 | 0.017065799 |
| regulation of activation of Janus kinase activity (GO:0010 | 44689 | 0.001616241 | 0.017065799 |
| regulation of dendritic cell chemotaxis (GO:2000508) | 44689 | 0.001616241 | 0.017065799 |
| regulation of interleukin-18 production (GO:0032661) | 44689 | 0.001616241 | 0.017065799 |
| regulation of protein K63-linked ubiquitination (GO:1900 | 44689 | 0.001616241 | 0.017065799 |
| regulation of toll-like receptor 3 signaling pathway (GO:0 | 44689 | 0.001616241 | 0.017065799 |
| positive regulation of T cell mediated immunity (GO:0002 | 13485 | 0.001658707 | 0.017431891 |
| regulation of cell cycle G1/S phase transition (GO:19028C | 13485 | 0.001658707 | 0.017431891 |
| cellular response to oxidative stress (GO:0034599) | 29/125 | 0.001660979 | 0.017431891 |
| cellular response to arsenic-containing substance (GO:00 | 44757 | 0.00173895 | 0.017995708 |
| integrated stress response signaling (GO:0140467) | 44757 | 0.00173895 | 0.017995708 |
| membrane raft organization (GO:0031579) | 44757 | 0.00173895 | 0.017995708 |
| microvillus assembly (GO:0030033) | 44757 | 0.00173895 | 0.017995708 |
| prostanoid biosynthetic process (GO:0046457) | 44757 | 0.00173895 | 0.017995708 |
| protein homotrimerization (GO:0070207) | 44757 | 0.00173895 | 0.017995708 |
| protein trimerization (GO:0070206) | 44757 | 0.00173895 | 0.017995708 |
| artery development (GO:0060840) | 44827 | 0.001772765 | 0.018272842 |
| regulation of cysteine-type endopeptidase activity (GO:2( | 44827 | 0.001772765 | 0.018272842 |
| regulation of G1/S transition of mitotic cell cycle (GO:200 | 19/71 | 0.001805949 | 0.018514201 |
| JNK cascade (GO:0007254) | 13/41 | 0.001806872 | 0.018514201 |
| negative regulation of cell-cell adhesion (GO:0022408) | 13/41 | 0.001806872 | 0.018514201 |
| regulation of glucose transmembrane transport (GO:001( | 44792 | 0.00183612 | 0.018776853 |
| regulation of protein ubiquitination (GO:0031396) | 26/109 | 0.001854511 | 0.018927668 |
| positive regulation of response to stimulus (GO:0048584) | 14/46 | 0.001897139 | 0.019324778 |
| regulation of cellular biosynthetic process (GO:0031326) | 11994 | 0.001939974 | 0.019681782 |
| chemokine-mediated signaling pathway (GO:0070098) | 16/56 | 0.001943552 | 0.019681782 |
| peptidyl-tyrosine modification (GO:0018212) | 16/56 | 0.001943552 | 0.019681782 |
| cellular response to lectin (GO:1990858) | 27/115 | 0.001961665 | 0.019788056 |

| | | | |
|---|---|---|---|
| stimulatory C-type lectin receptor signaling pathway (GO | 27/115 | 0.001961665 | 0.019788056 |
| protein modification by small protein removal (GO:0070€ | 54/276 | 0.002017868 | 0.020315549 |
| RNA metabolic process (GO:0016070) | 30/133 | 0.002220237 | 0.022170324 |
| leukocyte cell-cell adhesion (GO:0007159) | 44862 | 0.002231969 | 0.022170324 |
| positive regulation of monooxygenase activity (GO:0032 | 44862 | 0.002231969 | 0.022170324 |
| regulation of calcium-mediated signaling (GO:0050848) | 44862 | 0.002231969 | 0.022170324 |
| regulation of DNA damage response, signal transduction | 44862 | 0.002231969 | 0.022170324 |
| regulation of immunoglobulin production (GO:0002637) | 44862 | 0.002231969 | 0.022170324 |
| response to interferon-beta (GO:0035456) | 44862 | 0.002231969 | 0.022170324 |
| regulation of epidermal growth factor receptor signaling | 18/67 | 0.002245456 | 0.022261721 |
| regulated exocytosis (GO:0045055) | 38/180 | 0.002273632 | 0.022498128 |
| positive regulation of cellular metabolic process (GO:003 | 25/105 | 0.002304917 | 0.022764337 |
| carboxylic acid transport (GO:0046942) | 16/57 | 0.002374753 | 0.02334464 |
| positive regulation of proteolysis (GO:0045862) | 16/57 | 0.002374753 | 0.02334464 |
| negative regulation of cold-induced thermogenesis (GO:( | 14/47 | 0.002377154 | 0.02334464 |
| antigen processing and presentation of exogenous peptic | 20/78 | 0.002420848 | 0.023691525 |
| adipose tissue development (GO:0060612) | 44724 | 0.0024444 | 0.023691525 |
| mRNA transcription (GO:0009299) | 44724 | 0.0024444 | 0.023691525 |
| positive regulation of leukocyte proliferation (GO:00706€ | 44724 | 0.0024444 | 0.023691525 |
| positive regulation of neuroinflammatory response (GO:( | 44724 | 0.0024444 | 0.023691525 |
| protein auto-ADP-ribosylation (GO:0070213) | 44724 | 0.0024444 | 0.023691525 |
| regulation of MHC class II biosynthetic process (GO:0045 | 44724 | 0.0024444 | 0.023691525 |
| regulation of transcription by RNA polymerase II (GO:000 | 336/2206 | 0.002471123 | 0.023905928 |
| nucleotide-binding domain, leucine rich repeat containin | 44828 | 0.002505952 | 0.024108183 |
| positive regulation of transcription from RNA polymerase | 44828 | 0.002505952 | 0.024108183 |
| regulation of vascular endothelial growth factor receptor | 44828 | 0.002505952 | 0.024108183 |
| negative regulation of smooth muscle cell proliferation (( | 12359 | 0.002565668 | 0.024637051 |
| negative regulation of protein phosphorylation (GO:0001 | 33/152 | 0.002673682 | 0.025626896 |
| cortical actin cytoskeleton organization (GO:0030866) | 44793 | 0.002707479 | 0.02585543 |
| positive regulation of activated T cell proliferation (GO:0( | 44793 | 0.002707479 | 0.02585543 |
| filopodium assembly (GO:0046847) | 44758 | 0.002739784 | 0.025878506 |
| negative regulation of epithelial cell differentiation (GO:( | 44758 | 0.002739784 | 0.025878506 |
| neutrophil activation (GO:0042119) | 44758 | 0.002739784 | 0.025878506 |
| positive regulation of leukocyte adhesion to vascular end | 44758 | 0.002739784 | 0.025878506 |
| positive regulation of myeloid leukocyte cytokine produc | 44758 | 0.002739784 | 0.025878506 |
| regulation of hormone secretion (GO:0046883) | 44758 | 0.002739784 | 0.025878506 |
| fat cell differentiation (GO:0045444) | 16/58 | 0.002882576 | 0.027128591 |
| receptor metabolic process (GO:0043112) | 16/58 | 0.002882576 | 0.027128591 |
| positive regulation of T cell differentiation (GO:0045582) | 13/43 | 0.002907053 | 0.027309481 |
| intrinsic apoptotic signaling pathway in response to endo | 44863 | 0.003006796 | 0.028195495 |
| peptidyl-tyrosine phosphorylation (GO:0018108) | 18/69 | 0.003186985 | 0.029629276 |
| ATF6-mediated unfolded protein response (GO:0036500) | 44690 | 0.003239544 | 0.029629276 |
| endothelial cell chemotaxis (GO:0035767) | 44690 | 0.003239544 | 0.029629276 |
| kynurenine metabolic process (GO:0070189) | 44690 | 0.003239544 | 0.029629276 |
| positive regulation of macrophage cytokine production (( | 44690 | 0.003239544 | 0.029629276 |
| positive regulation of NLRP3 inflammasome complex ass( | 44690 | 0.003239544 | 0.029629276 |
| positive regulation of smooth muscle cell apoptotic proc€ | 44690 | 0.003239544 | 0.029629276 |

| | | | |
|---|---|---|---|
| regulation of endothelial cell development (GO:1901550) | 44690 | 0.003239544 | 0.029629276 |
| regulation of endothelial cell differentiation (GO:004560: | 44690 | 0.003239544 | 0.029629276 |
| regulation of interleukin-23 production (GO:0032667) | 44690 | 0.003239544 | 0.029629276 |
| regulation of leukocyte mediated cytotoxicity (GO:00019 | 44690 | 0.003239544 | 0.029629276 |
| regulation of PERK-mediated unfolded protein response ( | 44690 | 0.003239544 | 0.029629276 |
| regulation of tumor necrosis factor superfamily cytokine | 44690 | 0.003239544 | 0.029629276 |
| response to vitamin (GO:0033273) | 44690 | 0.003239544 | 0.029629276 |
| positive regulation of cell adhesion (GO:0045785) | 20/80 | 0.003329452 | 0.030398074 |
| positive regulation of epidermal growth factor receptor s | 12724 | 0.003344616 | 0.03048295 |
| regulation of neutrophil chemotaxis (GO:0090022) | 44829 | 0.003460168 | 0.031480865 |
| response to reactive oxygen species (GO:0000302) | 16/59 | 0.003476971 | 0.031578431 |
| negative regulation of secretion by cell (GO:1903531) | 14580 | 0.003544105 | 0.032076004 |
| regulation of peptidyl-threonine phosphorylation (GO:00 | 14580 | 0.003544105 | 0.032076004 |
| regulation of cyclin-dependent protein kinase activity (GC | 15/54 | 0.003580179 | 0.032346139 |
| negative regulation of phosphorylation (GO:0042326) | 21/86 | 0.003611181 | 0.032513145 |
| positive regulation of endothelial cell migration (GO:001( | 21/86 | 0.003611181 | 0.032513145 |
| lymphocyte chemotaxis (GO:0048247) | 13/44 | 0.003633362 | 0.03254365 |
| protein K63-linked ubiquitination (GO:0070534) | 13/44 | 0.003633362 | 0.03254365 |
| regulation of endothelial cell apoptotic process (GO:2000 | 13/44 | 0.003633362 | 0.03254365 |
| cellular response to bacterial lipopeptide (GO:0071221) | 44657 | 0.003706861 | 0.032583995 |
| ISG15-protein conjugation (GO:0032020) | 44657 | 0.003706861 | 0.032583995 |
| negative regulation of amyloid precursor protein biosynth | 44657 | 0.003706861 | 0.032583995 |
| negative regulation of toll-like receptor 2 signaling pathw | 44657 | 0.003706861 | 0.032583995 |
| positive regulation of killing of cells of other organism (G( | 44657 | 0.003706861 | 0.032583995 |
| positive regulation of toll-like receptor 7 signaling pathwa | 44657 | 0.003706861 | 0.032583995 |
| purine ribonucleoside catabolic process (GO:0046130) | 44657 | 0.003706861 | 0.032583995 |
| regulation of interleukin-1-mediated signaling pathway (( | 44657 | 0.003706861 | 0.032583995 |
| regulation of T-helper 17 cell lineage commitment (GO:2( | 44657 | 0.003706861 | 0.032583995 |
| regulation of type 2 immune response (GO:0002828) | 44657 | 0.003706861 | 0.032583995 |
| response to angiotensin (GO:1990776) | 44657 | 0.003706861 | 0.032583995 |
| protein stabilization (GO:0050821) | 37/179 | 0.003741757 | 0.032835185 |
| negative regulation of growth (GO:0045926) | 28/126 | 0.003819284 | 0.033458991 |
| positive regulation of phosphatase activity (GO:0010922) | 44794 | 0.003870569 | 0.033794293 |
| positive regulation of transcription from RNA polymerase | 44794 | 0.003870569 | 0.033794293 |
| positive regulation of endopeptidase activity (GO:001095 | 44864 | 0.00398161 | 0.034647342 |
| regulation of macrophage activation (GO:0043030) | 44864 | 0.00398161 | 0.034647342 |
| negative regulation of macrophage derived foam cell diff | 44725 | 0.004033074 | 0.034803683 |
| regulation of chemokine (C-X-C motif) ligand 2 productio | 44725 | 0.004033074 | 0.034803683 |
| regulation of receptor signaling pathway via STAT (GO:19 | 44725 | 0.004033074 | 0.034803683 |
| relaxation of cardiac muscle (GO:0055119) | 44725 | 0.004033074 | 0.034803683 |
| response to UV-B (GO:0010224) | 44725 | 0.004033074 | 0.034803683 |
| negative regulation of chemokine production (GO:00326 | 44759 | 0.004129095 | 0.035338797 |
| negative regulation of peptidyl-threonine phosphorylatio | 44759 | 0.004129095 | 0.035338797 |
| negative regulation of phagocytosis (GO:0050765) | 44759 | 0.004129095 | 0.035338797 |
| regulation of establishment of endothelial barrier (GO:19 | 44759 | 0.004129095 | 0.035338797 |
| regulation of NLRP3 inflammasome complex assembly (G | 44759 | 0.004129095 | 0.035338797 |
| cellular response to hormone stimulus (GO:0032870) | 19/76 | 0.004157063 | 0.035519639 |

| | | | |
|---|---|---|---|
| positive regulation of immunoglobulin production (GO:0( | 13089 | 0.004302106 | 0.036698587 |
| regulation of blood vessel endothelial cell migration (GO: | 15/55 | 0.004330342 | 0.036878892 |
| negative regulation of ERBB signaling pathway (GO:1901: | 14946 | 0.004462168 | 0.037815599 |
| positive regulation of intrinsic apoptotic signaling pathwa | 14946 | 0.004462168 | 0.037815599 |
| positive regulation of wound healing (GO:0090303) | 14946 | 0.004462168 | 0.037815599 |
| positive regulation of chemotaxis (GO:0050921) | 13/45 | 0.004499922 | 0.038073445 |
| peptidyl-serine modification (GO:0018209) | 35/169 | 0.004562848 | 0.038543079 |
| regulation of monocyte chemotaxis (GO:0090025) | 44830 | 0.004677686 | 0.039385058 |
| regulation of regulatory T cell differentiation (GO:004558 | 44830 | 0.004677686 | 0.039385058 |
| ERBB signaling pathway (GO:0038127) | 17/66 | 0.004708621 | 0.039581365 |
| positive regulation of multicellular organismal process (G | 63/345 | 0.004944946 | 0.041500796 |
| enzyme linked receptor protein signaling pathway (GO:0( | 30/140 | 0.004953683 | 0.04150707 |
| B cell differentiation (GO:0030183) | 16/61 | 0.004968821 | 0.041566872 |
| negative regulation of response to DNA damage stimulus | 44865 | 0.005189895 | 0.043276897 |
| regulation of macrophage derived foam cell differentiatic | 44865 | 0.005189895 | 0.043276897 |
| negative regulation of metabolic process (GO:0009892) | 15/56 | 0.005203559 | 0.043321294 |
| protein localization to membrane (GO:0072657) | 39/195 | 0.005357799 | 0.044534028 |
| negative regulation of phosphate metabolic process (GO: | 44795 | 0.005384235 | 0.04456957 |
| positive regulation of granulocyte chemotaxis (GO:00716 | 44795 | 0.005384235 | 0.04456957 |
| positive regulation of fat cell differentiation (GO:004560( | 14/51 | 0.005396393 | 0.04456957 |
| regulation of epithelial cell migration (GO:0010632) | 14/51 | 0.005396393 | 0.04456957 |
| membrane assembly (GO:0071709) | 13455 | 0.005465204 | 0.044994823 |
| regulation of osteoclast differentiation (GO:0045670) | 13455 | 0.005465204 | 0.044994823 |
| erythrocyte differentiation (GO:0030218) | 13/46 | 0.005525263 | 0.045376696 |
| proteasome-mediated ubiquitin-dependent protein catal | 59/321 | 0.005529057 | 0.045376696 |
| T cell differentiation (GO:0030217) | 15311 | 0.005560913 | 0.04556616 |
| cellular response to interleukin-4 (GO:0071353) | 44691 | 0.005774455 | 0.046436987 |
| cytoplasmic pattern recognition receptor signaling pathw | 44691 | 0.005774455 | 0.046436987 |
| immunoglobulin mediated immune response (GO:00160( | 44691 | 0.005774455 | 0.046436987 |
| indolalkylamine catabolic process (GO:0046218) | 44691 | 0.005774455 | 0.046436987 |
| negative regulation of cholesterol storage (GO:0010887) | 44691 | 0.005774455 | 0.046436987 |
| negative regulation of heterotypic cell-cell adhesion (GO: | 44691 | 0.005774455 | 0.046436987 |
| positive regulation of cell activation (GO:0050867) | 44691 | 0.005774455 | 0.046436987 |
| positive regulation of vascular endothelial growth factor | 44691 | 0.005774455 | 0.046436987 |
| protein poly-ADP-ribosylation (GO:0070212) | 44691 | 0.005774455 | 0.046436987 |
| regulation of axon guidance (GO:1902667) | 44691 | 0.005774455 | 0.046436987 |
| regulation of cell-cell adhesion involved in gastrulation (G | 44691 | 0.005774455 | 0.046436987 |
| response to laminar fluid shear stress (GO:0034616) | 44691 | 0.005774455 | 0.046436987 |
| regulation of protein binding (GO:0043393) | 26/118 | 0.005825327 | 0.046773687 |
| establishment of vesicle localization (GO:0051650) | 44760 | 0.00599191 | 0.047962979 |
| positive regulation of lymphocyte chemotaxis (GO:01401 | 44760 | 0.00599191 | 0.047962979 |
| antigen processing and presentation of exogenous peptic | 18/73 | 0.006059521 | 0.04842956 |
| protein localization to plasma membrane (GO:0072659) | 29/136 | 0.006081906 | 0.048486743 |
| positive regulation of ERK1 and ERK2 cascade (GO:00703 | 35/172 | 0.006085343 | 0.048486743 |
| regulation of MAPK cascade (GO:0043408) | 34/166 | 0.006119885 | 0.048639015 |
| Ras protein signal transduction (GO:0007265) | 30/142 | 0.006123179 | 0.048639015 |
| positive regulation of intracellular protein transport (GO: | 31/148 | 0.006145802 | 0.048744186 |

| | | | |
|---|---|---|---|
| negative regulation of insulin receptor signaling pathway | 44831 | 0.006203531 | 0.048783043 |
| amyloid precursor protein catabolic process (GO:004298 | 44726 | 0.006272776 | 0.048783043 |
| manganese ion transport (GO:0006828) | 44726 | 0.006272776 | 0.048783043 |
| negative regulation of bone mineralization (GO:0030502) | 44726 | 0.006272776 | 0.048783043 |
| negative regulation of DNA damage response, signal tran | 44726 | 0.006272776 | 0.048783043 |
| negative regulation of interleukin-12 production (GO:003 | 44726 | 0.006272776 | 0.048783043 |
| negative regulation of viral transcription (GO:0032897) | 44726 | 0.006272776 | 0.048783043 |
| nuclear migration (GO:0007097) | 44726 | 0.006272776 | 0.048783043 |
| positive regulation of lymphocyte migration (GO:200040 | 44726 | 0.006272776 | 0.048783043 |
| regulation of macrophage cytokine production (GO:0010 | 44726 | 0.006272776 | 0.048783043 |
| regulation of protein kinase C signaling (GO:0090036) | 44726 | 0.006272776 | 0.048783043 |
| relaxation of muscle (GO:0090075) | 44726 | 0.006272776 | 0.048783043 |
| T cell differentiation in thymus (GO:0033077) | 44726 | 0.006272776 | 0.048783043 |
| positive regulation of autophagy (GO:0010508) | 21/90 | 0.006334832 | 0.049192005 |
| superoxide metabolic process (GO:0006801) | 11963 | 0.006667135 | 0.051695177 |
| monocyte chemotaxis (GO:0002548) | 15676 | 0.006863837 | 0.053061957 |
| positive regulation of protein tyrosine kinase activity (GO | 15676 | 0.006863837 | 0.053061957 |
| B cell activation (GO:0042113) | 20/85 | 0.006898547 | 0.053251041 |
| negative regulation of signal transduction (GO:0009968) | 50/267 | 0.007007681 | 0.053933193 |
| protein deubiquitination (GO:0016579) | 50/267 | 0.007007681 | 0.053933193 |
| NIK/NF-kappaB signaling (GO:0038061) | 18/74 | 0.007036799 | 0.053997295 |
| proteolysis involved in cellular protein catabolic process ( | 18/74 | 0.007036799 | 0.053997295 |
| regulation of actin cytoskeleton organization (GO:003295 | 21/91 | 0.007230006 | 0.055398057 |
| positive regulation of JUN kinase activity (GO:0043507) | 44796 | 0.007310357 | 0.055848976 |
| regulation of B cell receptor signaling pathway (GO:0050 | 44796 | 0.007310357 | 0.055848976 |
| negative regulation of cellular catabolic process (GO:003 | 17/69 | 0.007584189 | 0.05696687 |
| cellular response to interleukin-18 (GO:0071351) | 44658 | 0.007741792 | 0.05696687 |
| chemical homeostasis within a tissue (GO:0048875) | 44658 | 0.007741792 | 0.05696687 |
| complement activation, alternative pathway (GO:000695 | 44658 | 0.007741792 | 0.05696687 |
| interleukin-18-mediated signaling pathway (GO:0035655 | 44658 | 0.007741792 | 0.05696687 |
| macropinocytosis (GO:0044351) | 44658 | 0.007741792 | 0.05696687 |
| negative regulation of interferon-gamma-mediated signa | 44658 | 0.007741792 | 0.05696687 |
| negative regulation of neurotransmitter secretion (GO:0 | 44658 | 0.007741792 | 0.05696687 |
| negative regulation of response to interferon-gamma (G | 44658 | 0.007741792 | 0.05696687 |
| negative regulation of T-helper 17 type immune respons | 44658 | 0.007741792 | 0.05696687 |
| nucleotide-binding oligomerization domain containing 2 | 44658 | 0.007741792 | 0.05696687 |
| positive regulation of activation of Janus kinase activity ( | 44658 | 0.007741792 | 0.05696687 |
| positive regulation of fibroblast apoptotic process (GO:2 | 44658 | 0.007741792 | 0.05696687 |
| positive regulation of interleukin-23 production (GO:003 | 44658 | 0.007741792 | 0.05696687 |
| positive regulation of miRNA metabolic process (GO:200 | 44658 | 0.007741792 | 0.05696687 |
| positive regulation of oxidative stress-induced cell death | 44658 | 0.007741792 | 0.05696687 |
| positive regulation of receptor catabolic process (GO:200 | 44658 | 0.007741792 | 0.05696687 |
| positive regulation of unsaturated fatty acid biosynthetic | 44658 | 0.007741792 | 0.05696687 |
| protein desumoylation (GO:0016926) | 44658 | 0.007741792 | 0.05696687 |
| regulation of necrotic cell death (GO:0010939) | 44658 | 0.007741792 | 0.05696687 |
| regulation of T-helper 1 cell differentiation (GO:0045625) | 44658 | 0.007741792 | 0.05696687 |
| regulation of toll-like receptor 7 signaling pathway (GO:0 | 44658 | 0.007741792 | 0.05696687 |

| | | | |
|---|---|---|---|
| release of sequestered calcium ion into cytosol by endop | 44658 | 0.007741792 | 0.05696687 |
| sphingosine-1-phosphate receptor signaling pathway (GC | 44658 | 0.007741792 | 0.05696687 |
| surfactant homeostasis (GO:0043129) | 44658 | 0.007741792 | 0.05696687 |
| synaptic vesicle budding from presynaptic endocytic zone | 44658 | 0.007741792 | 0.05696687 |
| negative regulation of cellular response to insulin stimulu | 44832 | 0.008084801 | 0.059406709 |
| negative regulation of mitotic cell cycle (GO:0045930) | 13/48 | 0.008131475 | 0.059581116 |
| positive regulation of blood vessel endothelial cell migrat | 13/48 | 0.008131475 | 0.059581116 |
| positive regulation of neutrophil chemotaxis (GO:009002 | 44761 | 0.008416127 | 0.060978776 |
| positive regulation of oxidoreductase activity (GO:00513! | 44761 | 0.008416127 | 0.060978776 |
| prostaglandin biosynthetic process (GO:0001516) | 44761 | 0.008416127 | 0.060978776 |
| pyroptosis (GO:0070269) | 44761 | 0.008416127 | 0.060978776 |
| regulation of leukocyte chemotaxis (GO:0002688) | 44761 | 0.008416127 | 0.060978776 |
| regulation of lymphocyte proliferation (GO:0050670) | 44761 | 0.008416127 | 0.060978776 |
| regulation of neuroinflammatory response (GO:0150077) | 44761 | 0.008416127 | 0.060978776 |
| regulation of T cell cytokine production (GO:0002724) | 44761 | 0.008416127 | 0.060978776 |
| apoptotic cell clearance (GO:0043277) | 12328 | 0.008450386 | 0.061056681 |
| regulation of sprouting angiogenesis (GO:1903670) | 12328 | 0.008450386 | 0.061056681 |
| connective tissue development (GO:0061448) | 14185 | 0.008523955 | 0.061417399 |
| regulation of cell communication (GO:0010646) | 14185 | 0.008523955 | 0.061417399 |
| positive regulation of cellular amide metabolic process (G | 19/81 | 0.008609164 | 0.061945438 |
| cellular metal ion homeostasis (GO:0006875) | 23/104 | 0.008717488 | 0.062638102 |
| regulation of cell cycle (GO:0051726) | 54/296 | 0.008906919 | 0.063910835 |
| protein localization to cell periphery (GO:1990778) | 29/140 | 0.009183471 | 0.065648 |
| macrophage differentiation (GO:0030225) | 44727 | 0.009295335 | 0.065648 |
| negative regulation of leukocyte activation (GO:0002695) | 44727 | 0.009295335 | 0.065648 |
| positive regulation of cell killing (GO:0031343) | 44727 | 0.009295335 | 0.065648 |
| positive regulation of natural killer cell activation (GO:00: | 44727 | 0.009295335 | 0.065648 |
| regulation of macrophage differentiation (GO:0045649) | 44727 | 0.009295335 | 0.065648 |
| regulation of triglyceride biosynthetic process (GO:00108 | 44727 | 0.009295335 | 0.065648 |
| regulation of RNA splicing (GO:0043484) | 18/76 | 0.00937316 | 0.065648 |
| negative regulation of oxidative stress-induced neuron de | 44692 | 0.009439664 | 0.065648 |
| negative regulation of platelet-derived growth factor rec | 44692 | 0.009439664 | 0.065648 |
| nucleus localization (GO:0051647) | 44692 | 0.009439664 | 0.065648 |
| positive regulation of DNA methylation-dependent heter | 44692 | 0.009439664 | 0.065648 |
| positive regulation of integrin activation (GO:0033625) | 44692 | 0.009439664 | 0.065648 |
| positive regulation of macrophage differentiation (GO:00 | 44692 | 0.009439664 | 0.065648 |
| pyrimidine nucleoside catabolic process (GO:0046135) | 44692 | 0.009439664 | 0.065648 |
| rDNA heterochromatin assembly (GO:0000183) | 44692 | 0.009439664 | 0.065648 |
| regulation of CD4-positive, alpha-beta T cell proliferation | 44692 | 0.009439664 | 0.065648 |
| regulation of T cell apoptotic process (GO:0070232) | 44692 | 0.009439664 | 0.065648 |
| regulation of T-helper 2 cell cytokine production (GO:200 | 44692 | 0.009439664 | 0.065648 |
| regulation of toll-like receptor 2 signaling pathway (GO:0 | 44692 | 0.009439664 | 0.065648 |
| response to sterol (GO:0036314) | 44692 | 0.009439664 | 0.065648 |
| T cell homeostasis (GO:0043029) | 44692 | 0.009439664 | 0.065648 |
| tryptophan catabolic process (GO:0006569) | 44692 | 0.009439664 | 0.065648 |
| heterochromatin assembly (GO:0031507) | 44797 | 0.009712565 | 0.067007667 |
| leukotriene metabolic process (GO:0006691) | 44797 | 0.009712565 | 0.067007667 |

| | | | |
|---|---|---|---|
| long-chain fatty-acyl-CoA metabolic process (GO:003533( | 44797 | 0.009712565 | 0.067007667 |
| negative regulation of ossification (GO:0030279) | 44797 | 0.009712565 | 0.067007667 |
| positive regulation of neuron apoptotic process (GO:004: | 44797 | 0.009712565 | 0.067007667 |
| regulation of cell size (GO:0008361) | 44797 | 0.009712565 | 0.067007667 |
| positive regulation of protein ubiquitination (GO:003139 | 19/82 | 0.009846577 | 0.067752277 |
| regulation of cyclin-dependent protein serine/threonine | 19/82 | 0.009846577 | 0.067752277 |
| positive regulation of mRNA catabolic process (GO:0061C | 16407 | 0.010181302 | 0.069962786 |
| cellular protein catabolic process (GO:0044257) | 15/60 | 0.010217284 | 0.070117295 |
| negative regulation of interferon-gamma production (GO | 44833 | 0.010369908 | 0.070977172 |
| positive regulation of leukocyte migration (GO:0002687) | 44833 | 0.010369908 | 0.070977172 |
| endothelial cell migration (GO:0043542) | 14550 | 0.010480328 | 0.071544417 |
| protein acylation (GO:0043543) | 14550 | 0.010480328 | 0.071544417 |
| B cell receptor signaling pathway (GO:0050853) | 12693 | 0.010577794 | 0.071926227 |
| regulation of amyloid-beta formation (GO:1902003) | 12693 | 0.010577794 | 0.071926227 |
| regulation of cell adhesion mediated by integrin (GO:003 | 12693 | 0.010577794 | 0.071926227 |
| negative regulation of cytokine-mediated signaling pathw | 14/55 | 0.010929946 | 0.07422362 |
| small GTPase mediated signal transduction (GO:0007264 | 23/106 | 0.010981793 | 0.074478345 |
| protein-containing complex subunit organization (GO:004 | 24/112 | 0.011113499 | 0.075273305 |
| modulation by symbiont of entry into host (GO:0052372) | 44762 | 0.011490022 | 0.077419796 |
| positive regulation of alpha-beta T cell activation (GO:004 | 44762 | 0.011490022 | 0.077419796 |
| positive regulation of protein processing (GO:0010954) | 44762 | 0.011490022 | 0.077419796 |
| regulation of protein modification by small protein conju | 44762 | 0.011490022 | 0.077419796 |
| transforming growth factor beta receptor signaling pathw | 20/89 | 0.01159966 | 0.0780573 |
| transcription by RNA polymerase II (GO:0006366) | 57/320 | 0.011939845 | 0.080242554 |
| proteasomal protein catabolic process (GO:0010498) | 39/205 | 0.012301853 | 0.082568636 |
| negative regulation of cell junction assembly (GO:190188 | 44798 | 0.012654875 | 0.084719166 |
| positive regulation of release of cytochrome c from mito | 44798 | 0.012654875 | 0.084719166 |
| lymphocyte differentiation (GO:0030098) | 19/84 | 0.012746783 | 0.085005824 |
| nuclear export (GO:0051168) | 19/84 | 0.012746783 | 0.085005824 |
| vascular transport (GO:0010232) | 19/84 | 0.012746783 | 0.085005824 |
| ubiquitin-dependent protein catabolic process (GO:0006! | 62/354 | 0.012890963 | 0.085857121 |
| regulation of T cell receptor signaling pathway (GO:0050 | 13058 | 0.013088073 | 0.086443981 |
| positive regulation of binding (GO:0051099) | 20/90 | 0.013103899 | 0.086443981 |
| extrinsic apoptotic signaling pathway via death domain re | 44834 | 0.013107755 | 0.086443981 |
| activation of NF-kappaB-inducing kinase activity (GO:000 | 44728 | 0.013228675 | 0.086443981 |
| cellular polysaccharide catabolic process (GO:0044247) | 44728 | 0.013228675 | 0.086443981 |
| negative regulation of anoikis (GO:2000811) | 44728 | 0.013228675 | 0.086443981 |
| negative regulation of macrophage activation (GO:00430 | 44728 | 0.013228675 | 0.086443981 |
| negative regulation of type I interferon-mediated signalir | 44728 | 0.013228675 | 0.086443981 |
| platelet-derived growth factor receptor signaling pathwa | 44728 | 0.013228675 | 0.086443981 |
| positive regulation of double-strand break repair via nonl | 44728 | 0.013228675 | 0.086443981 |
| positive regulation of TORC1 signaling (GO:1904263) | 44728 | 0.013228675 | 0.086443981 |
| protein localization to endoplasmic reticulum (GO:00709 | 44728 | 0.013228675 | 0.086443981 |
| regulation of establishment of cell polarity (GO:2000114) | 44728 | 0.013228675 | 0.086443981 |
| regulation of interleukin-1 production (GO:0032652) | 44728 | 0.013228675 | 0.086443981 |
| regulation of isotype switching (GO:0045191) | 44728 | 0.013228675 | 0.086443981 |
| regulation of hydrolase activity (GO:0051336) | 17/73 | 0.01344732 | 0.08765223 |

| | | | |
|---|---|---|---|
| regulation of Rho protein signal transduction (GO:00350: | 17/73 | 0.01344732 | 0.08765223 |
| blood circulation (GO:0008015) | 13/51 | 0.013747201 | 0.08785481 |
| negative regulation of protein ubiquitination (GO:00313 | 13/51 | 0.013747201 | 0.08785481 |
| negative regulation of transport (GO:0051051) | 13/51 | 0.013747201 | 0.08785481 |
| macroautophagy (GO:0016236) | 25/120 | 0.013785473 | 0.08785481 |
| coronary vasculature morphogenesis (GO:0060977) | 44659 | 0.013867362 | 0.08785481 |
| cytoplasmic sequestering of NF-kappaB (GO:0007253) | 44659 | 0.013867362 | 0.08785481 |
| fusion of virus membrane with host plasma membrane (( | 44659 | 0.013867362 | 0.08785481 |
| membrane fusion involved in viral entry into host cell (G( | 44659 | 0.013867362 | 0.08785481 |
| myeloid leukocyte mediated immunity (GO:0002444) | 44659 | 0.013867362 | 0.08785481 |
| negative regulation of regulated secretory pathway (GO:: | 44659 | 0.013867362 | 0.08785481 |
| negative regulation of T-helper 17 cell differentiation (GC | 44659 | 0.013867362 | 0.08785481 |
| positive regulation of adaptive immune response (GO:00 | 44659 | 0.013867362 | 0.08785481 |
| positive regulation of dendritic cell chemotaxis (GO:2000 | 44659 | 0.013867362 | 0.08785481 |
| positive regulation of extracellular matrix disassembly (G | 44659 | 0.013867362 | 0.08785481 |
| positive regulation of T-helper 2 cell cytokine production | 44659 | 0.013867362 | 0.08785481 |
| regulation of extracellular matrix constituent secretion (( | 44659 | 0.013867362 | 0.08785481 |
| regulation of nitrogen compound metabolic process (GO: | 44659 | 0.013867362 | 0.08785481 |
| regulation of oxidative stress-induced neuron death (GO: | 44659 | 0.013867362 | 0.08785481 |
| regulation of pattern recognition receptor signaling path\ | 44659 | 0.013867362 | 0.08785481 |
| regulation of T-helper 2 cell differentiation (GO:0045628) | 44659 | 0.013867362 | 0.08785481 |
| regulation of transcription from RNA polymerase II prom( | 44659 | 0.013867362 | 0.08785481 |
| regulation of vascular wound healing (GO:0061043) | 44659 | 0.013867362 | 0.08785481 |
| synaptic vesicle budding (GO:0070142) | 44659 | 0.013867362 | 0.08785481 |
| histone H3-K9 demethylation (GO:0033169) | 44693 | 0.014436294 | 0.090794852 |
| nucleoside catabolic process (GO:0009164) | 44693 | 0.014436294 | 0.090794852 |
| regulation of glycogen metabolic process (GO:0070873) | 44693 | 0.014436294 | 0.090794852 |
| regulation of myoblast fusion (GO:1901739) | 44693 | 0.014436294 | 0.090794852 |
| regulation of natural killer cell activation (GO:0032814) | 44693 | 0.014436294 | 0.090794852 |
| synaptic vesicle fusion to presynaptic active zone membr | 44693 | 0.014436294 | 0.090794852 |
| calcium ion homeostasis (GO:0055074) | 21/97 | 0.014995535 | 0.094085279 |
| protein K48-linked ubiquitination (GO:0070936) | 14/57 | 0.01499569 | 0.094085279 |
| chondroitin sulfate biosynthetic process (GO:0030206) | 44763 | 0.015299756 | 0.095302434 |
| NAD biosynthetic process (GO:0009435) | 44763 | 0.015299756 | 0.095302434 |
| nuclear-transcribed mRNA poly(A) tail shortening (GO:00 | 44763 | 0.015299756 | 0.095302434 |
| positive regulation of antigen receptor-mediated signalin | 44763 | 0.015299756 | 0.095302434 |
| regulation of anoikis (GO:2000209) | 44763 | 0.015299756 | 0.095302434 |
| regulation of fibroblast migration (GO:0010762) | 44763 | 0.015299756 | 0.095302434 |
| DNA damage response, signal transduction by p53 class r | 17/74 | 0.015359232 | 0.095558336 |
| keratinocyte differentiation (GO:0030216) | 15281 | 0.01540388 | 0.095607117 |
| regulation of filopodium assembly (GO:0051489) | 15281 | 0.01540388 | 0.095607117 |
| skin development (GO:0043588) | 18/80 | 0.01588458 | 0.098473025 |
| actin filament organization (GO:0007015) | 30/152 | 0.015926303 | 0.098613999 |
| membrane fusion (GO:0061025) | 13424 | 0.01601995 | 0.098957955 |
| transition metal ion transport (GO:0000041) | 13424 | 0.01601995 | 0.098957955 |
| histone lysine demethylation (GO:0070076) | 44799 | 0.016200298 | 0.099598279 |
| negative regulation of intrinsic apoptotic signaling pathw | 44799 | 0.016200298 | 0.099598279 |

| | | | |
|---|---|---|---|
| positive regulation of G1/S transition of mitotic cell cycle | 44799 | 0.016200298 | 0.099598279 |
| positive regulation of T cell mediated cytotoxicity (GO:00 | 44799 | 0.016200298 | 0.099598279 |
| transport across blood-brain barrier (GO:0150104) | 19/86 | 0.016283763 | 0.099993082 |
| prostaglandin metabolic process (GO:0006693) | 11567 | 0.016346885 | 0.100143948 |
| protein palmitoylation (GO:0018345) | 11567 | 0.016346885 | 0.100143948 |
| cellular response to fibroblast growth factor stimulus (GC | 20/92 | 0.016573202 | 0.101410819 |
| positive regulation of cell-cell adhesion (GO:0022409) | 17502 | 0.01732515 | 0.105887242 |
| membrane organization (GO:0061024) | 44/242 | 0.017810975 | 0.108535905 |
| negative regulation of cytokine production involved in im | 44729 | 0.018192091 | 0.108535905 |
| negative regulation of viral entry into host cell (GO:00465 | 44729 | 0.018192091 | 0.108535905 |
| pyrimidine-containing compound catabolic process (GO:( | 44729 | 0.018192091 | 0.108535905 |
| synaptic vesicle membrane organization (GO:0048499) | 44729 | 0.018192091 | 0.108535905 |
| negative regulation of angiogenesis (GO:0016525) | 19/87 | 0.018317725 | 0.108535905 |
| regulation of transcription from RNA polymerase II prom | 19/87 | 0.018317725 | 0.108535905 |
| nucleic acid-templated transcription (GO:0097659) | 15646 | 0.018434222 | 0.108535905 |
| regulation of transcription, DNA-templated (GO:0006355 | 330/2244 | 0.018657623 | 0.108535905 |
| cellular divalent inorganic cation homeostasis (GO:00725 | 23/111 | 0.01874298 | 0.108535905 |
| regulation of signal transduction (GO:0009966) | 37/198 | 0.018858469 | 0.108535905 |
| IRE1-mediated unfolded protein response (GO:0036498) | 13/53 | 0.018886702 | 0.108535905 |
| alpha-beta T cell differentiation (GO:0046632) | 44625 | 0.018928495 | 0.108535905 |
| bone marrow development (GO:0048539) | 44625 | 0.018928495 | 0.108535905 |
| cellular response to sodium arsenite (GO:1903936) | 44625 | 0.018928495 | 0.108535905 |
| cellular response to vitamin D (GO:0071305) | 44625 | 0.018928495 | 0.108535905 |
| chronic inflammatory response (GO:0002544) | 44625 | 0.018928495 | 0.108535905 |
| DNA catabolic process, exonucleolytic (GO:0000738) | 44625 | 0.018928495 | 0.108535905 |
| glucose import across plasma membrane (GO:0098708) | 44625 | 0.018928495 | 0.108535905 |
| inosine metabolic process (GO:0046102) | 44625 | 0.018928495 | 0.108535905 |
| mature ribosome assembly (GO:0042256) | 44625 | 0.018928495 | 0.108535905 |
| negative regulation of CREB transcription factor activity ( | 44625 | 0.018928495 | 0.108535905 |
| negative regulation of hydrogen peroxide metabolic proc | 44625 | 0.018928495 | 0.108535905 |
| negative regulation of IRE1-mediated unfolded protein re | 44625 | 0.018928495 | 0.108535905 |
| negative regulation of lipid transport (GO:0032369) | 44625 | 0.018928495 | 0.108535905 |
| negative regulation of myosin-light-chain-phosphatase ac | 44625 | 0.018928495 | 0.108535905 |
| negative regulation of pinocytosis (GO:0048550) | 44625 | 0.018928495 | 0.108535905 |
| peptidyl-cysteine S-nitrosylation (GO:0018119) | 44625 | 0.018928495 | 0.108535905 |
| positive regulation of CD4-positive, alpha-beta T cell prol | 44625 | 0.018928495 | 0.108535905 |
| positive regulation of cell fate commitment (GO:0010455 | 44625 | 0.018928495 | 0.108535905 |
| positive regulation of glial cell proliferation (GO:0060252 | 44625 | 0.018928495 | 0.108535905 |
| positive regulation of heat generation (GO:0031652) | 44625 | 0.018928495 | 0.108535905 |
| positive regulation of interleukin-18 production (GO:003. | 44625 | 0.018928495 | 0.108535905 |
| positive regulation of leukocyte mediated immunity (GO: | 44625 | 0.018928495 | 0.108535905 |
| positive regulation of leukocyte tethering or rolling (GO:1 | 44625 | 0.018928495 | 0.108535905 |
| positive regulation of MHC class I biosynthetic process (G | 44625 | 0.018928495 | 0.108535905 |
| positive regulation of mononuclear cell proliferation (GO | 44625 | 0.018928495 | 0.108535905 |
| positive regulation of oxidative stress-induced neuron de | 44625 | 0.018928495 | 0.108535905 |
| postsynaptic endocytosis (GO:0140239) | 44625 | 0.018928495 | 0.108535905 |
| postsynaptic neurotransmitter receptor internalization (( | 44625 | 0.018928495 | 0.108535905 |

| | | | |
|---|---|---|---|
| regulation of core promoter binding (GO:1904796) | 44625 | 0.018928495 | 0.108535905 |
| regulation of dendritic cell cytokine production (GO:0002 | 44625 | 0.018928495 | 0.108535905 |
| regulation of Fc-gamma receptor signaling pathway invol | 44625 | 0.018928495 | 0.108535905 |
| regulation of fever generation (GO:0031620) | 44625 | 0.018928495 | 0.108535905 |
| regulation of glial cell migration (GO:1903975) | 44625 | 0.018928495 | 0.108535905 |
| regulation of miRNA catabolic process (GO:2000625) | 44625 | 0.018928495 | 0.108535905 |
| regulation of miRNA metabolic process (GO:2000628) | 44625 | 0.018928495 | 0.108535905 |
| regulation of mononuclear cell proliferation (GO:003294 | 44625 | 0.018928495 | 0.108535905 |
| regulation of NK T cell proliferation (GO:0051140) | 44625 | 0.018928495 | 0.108535905 |
| regulation of nucleotide-binding oligomerization domain | 44625 | 0.018928495 | 0.108535905 |
| regulation of platelet-derived growth factor receptor-bet | 44625 | 0.018928495 | 0.108535905 |
| regulation of retinal ganglion cell axon guidance (GO:009 | 44625 | 0.018928495 | 0.108535905 |
| regulation of type B pancreatic cell apoptotic process (GC | 44625 | 0.018928495 | 0.108535905 |
| response to sodium arsenite (GO:1903935) | 44625 | 0.018928495 | 0.108535905 |
| Toll signaling pathway (GO:0008063) | 44625 | 0.018928495 | 0.108535905 |
| tryptophan catabolic process to kynurenine (GO:0019441 | 44625 | 0.018928495 | 0.108535905 |
| negative regulation of epithelial cell apoptotic process (G | 13789 | 0.019411593 | 0.110695091 |
| phagocytosis, engulfment (GO:0006911) | 13789 | 0.019411593 | 0.110695091 |
| positive regulation of myeloid cell differentiation (GO:00 | 13789 | 0.019411593 | 0.110695091 |
| regulation of erythrocyte differentiation (GO:0045646) | 13789 | 0.019411593 | 0.110695091 |
| regulation of intracellular pH (GO:0051453) | 13789 | 0.019411593 | 0.110695091 |
| regulation of epithelial to mesenchymal transition (GO:0( | 17/76 | 0.019810553 | 0.112767753 |
| membrane protein intracellular domain proteolysis (GO:( | 44764 | 0.019927006 | 0.112767753 |
| microglial cell activation (GO:0001774) | 44764 | 0.019927006 | 0.112767753 |
| negative regulation of interleukin-2 production (GO:0032 | 44764 | 0.019927006 | 0.112767753 |
| positive regulation of neutrophil migration (GO:1902624) | 44764 | 0.019927006 | 0.112767753 |
| regulation of membrane protein ectodomain proteolysis | 44764 | 0.019927006 | 0.112767753 |
| regulation of viral release from host cell (GO:1902186) | 44764 | 0.019927006 | 0.112767753 |
| cellular response to topologically incorrect protein (GO:0 | 11933 | 0.020134619 | 0.113818655 |
| regulation of gene expression, epigenetic (GO:0040029) | 18/82 | 0.020246096 | 0.114253438 |
| regulation of protein metabolic process (GO:0051246) | 17868 | 0.020389713 | 0.114253438 |
| cellular senescence (GO:0090398) | 44800 | 0.020409469 | 0.114253438 |
| chondroitin sulfate proteoglycan biosynthetic process (G( | 44800 | 0.020409469 | 0.114253438 |
| positive regulation of erythrocyte differentiation (GO:004 | 44800 | 0.020409469 | 0.114253438 |
| positive regulation of nuclear division (GO:0051785) | 44800 | 0.020409469 | 0.114253438 |
| protein export from nucleus (GO:0006611) | 44800 | 0.020409469 | 0.114253438 |
| release of sequestered calcium ion into cytosol (GO:0051 | 44800 | 0.020409469 | 0.114253438 |
| stress-activated protein kinase signaling cascade (GO:003 | 44800 | 0.020409469 | 0.114253438 |
| negative regulation of binding (GO:0051100) | 19/88 | 0.020543059 | 0.114877492 |
| DNA catabolic process (GO:0006308) | 44694 | 0.02093832 | 0.115471947 |
| entry into host (GO:0044409) | 44694 | 0.02093832 | 0.115471947 |
| icosanoid secretion (GO:0032309) | 44694 | 0.02093832 | 0.115471947 |
| negative regulation of hippo signaling (GO:0035331) | 44694 | 0.02093832 | 0.115471947 |
| negative regulation of viral release from host cell (GO:19( | 44694 | 0.02093832 | 0.115471947 |
| positive regulation of calcium-mediated signaling (GO:00 | 44694 | 0.02093832 | 0.115471947 |
| positive regulation of CD4-positive, alpha-beta T cell activ | 44694 | 0.02093832 | 0.115471947 |
| positive regulation of heterochromatin assembly (GO:003 | 44694 | 0.02093832 | 0.115471947 |

| | | | |
|---|---|---|---|
| positive regulation of protein localization to cell surface ( | 44694 | 0.02093832 | 0.115471947 |
| purine-containing compound salvage (GO:0043101) | 44694 | 0.02093832 | 0.115471947 |
| regulation of antigen receptor-mediated signaling pathwa | 44694 | 0.02093832 | 0.115471947 |
| regulation of homotypic cell-cell adhesion (GO:0034110) | 44694 | 0.02093832 | 0.115471947 |
| response to interleukin-6 (GO:0070741) | 44694 | 0.02093832 | 0.115471947 |
| DNA-templated transcription, initiation (GO:0006352) | 32/168 | 0.02135201 | 0.117628519 |
| cellular response to DNA damage stimulus (GO:0006974) | 60/350 | 0.021501842 | 0.118328465 |
| cellular response to glucose starvation (GO:0042149) | 16011 | 0.021885037 | 0.120055723 |
| positive regulation of leukocyte mediated cytotoxicity (G | 16011 | 0.021885037 | 0.120055723 |
| regulation of cell-cell adhesion (GO:0022407) | 16011 | 0.021885037 | 0.120055723 |
| regulation of cell differentiation (GO:0045595) | 30/156 | 0.022329713 | 0.120550061 |
| CD40 signaling pathway (GO:0023035) | 44660 | 0.022369636 | 0.120550061 |
| cellular response to leptin stimulus (GO:0044320) | 44660 | 0.022369636 | 0.120550061 |
| IMP metabolic process (GO:0046040) | 44660 | 0.022369636 | 0.120550061 |
| negative regulation of leukocyte degranulation (GO:0043 | 44660 | 0.022369636 | 0.120550061 |
| negative regulation of production of molecular mediator | 44660 | 0.022369636 | 0.120550061 |
| positive regulation of memory T cell differentiation (GO:( | 44660 | 0.022369636 | 0.120550061 |
| protein O-linked glycosylation via threonine (GO:001824 | 44660 | 0.022369636 | 0.120550061 |
| regulation of cell adhesion molecule production (GO:006 | 44660 | 0.022369636 | 0.120550061 |
| regulation of dendritic cell antigen processing and preser | 44660 | 0.022369636 | 0.120550061 |
| regulation of epidermis development (GO:0045682) | 44660 | 0.022369636 | 0.120550061 |
| regulation of fibroblast apoptotic process (GO:2000269) | 44660 | 0.022369636 | 0.120550061 |
| regulation of glial cell proliferation (GO:0060251) | 44660 | 0.022369636 | 0.120550061 |
| regulation of pinocytosis (GO:0048548) | 44660 | 0.022369636 | 0.120550061 |
| regulation of smooth muscle cell apoptotic process (GO:( | 44660 | 0.022369636 | 0.120550061 |
| response to vitamin D (GO:0033280) | 44660 | 0.022369636 | 0.120550061 |
| T-helper 1 type immune response (GO:0042088) | 44660 | 0.022369636 | 0.120550061 |
| regulation of apoptotic signaling pathway (GO:2001233) | 14154 | 0.023300041 | 0.125433898 |
| negative regulation of catabolic process (GO:0009895) | 18233 | 0.023839115 | 0.12820311 |
| positive regulation of cell cycle (GO:0045787) | 15/66 | 0.024062254 | 0.128773405 |
| cellular response to alkaloid (GO:0071312) | 44730 | 0.024292192 | 0.128773405 |
| cellular response to dsRNA (GO:0071359) | 44730 | 0.024292192 | 0.128773405 |
| long-chain fatty-acyl-CoA biosynthetic process (GO:0035 | 44730 | 0.024292192 | 0.128773405 |
| negative regulation of signal transduction by p53 class m | 44730 | 0.024292192 | 0.128773405 |
| positive regulation of extracellular matrix organization (G | 44730 | 0.024292192 | 0.128773405 |
| positive regulation of fatty acid metabolic process (GO:0( | 44730 | 0.024292192 | 0.128773405 |
| positive regulation of interleukin-4 production (GO:0032 | 44730 | 0.024292192 | 0.128773405 |
| positive regulation of response to endoplasmic reticulum | 44730 | 0.024292192 | 0.128773405 |
| regulation of brown fat cell differentiation (GO:0090335) | 44730 | 0.024292192 | 0.128773405 |
| regulation of establishment or maintenance of cell polari | 44730 | 0.024292192 | 0.128773405 |
| regulation of platelet aggregation (GO:0090330) | 44730 | 0.024292192 | 0.128773405 |
| regulation of transforming growth factor beta productior | 44730 | 0.024292192 | 0.128773405 |
| response to interferon-alpha (GO:0035455) | 44730 | 0.024292192 | 0.128773405 |
| negative regulation of cell growth (GO:0030308) | 25/126 | 0.024498067 | 0.129432642 |
| positive regulation of ATP metabolic process (GO:190358 | 12298 | 0.024516212 | 0.129432642 |
| positive regulation of receptor signaling pathway via JAK- | 12298 | 0.024516212 | 0.129432642 |
| signal transduction by p53 class mediator (GO:0072331) | 12298 | 0.024516212 | 0.129432642 |

| | | | |
|---|---|---|---|
| cellular response to transforming growth factor beta stim | 23/114 | 0.025138059 | 0.132312278 |
| positive regulation of cellular component organization (G | 23/114 | 0.025138059 | 0.132312278 |
| positive regulation of kinase activity (GO:0033674) | 23/114 | 0.025138059 | 0.132312278 |
| negative regulation of apoptotic signaling pathway (GO:2 | 17/78 | 0.025187069 | 0.132436055 |
| protein-containing complex assembly (GO:0065003) | 47/267 | 0.025231498 | 0.132535524 |
| positive regulation of phosphatidylinositol 3-kinase activi | 44801 | 0.025339342 | 0.132565845 |
| positive regulation of protein import into nucleus (GO:00 | 44801 | 0.025339342 | 0.132565845 |
| positive regulation of response to wounding (GO:190303 | 44801 | 0.025339342 | 0.132565845 |
| regulation of protein transport (GO:0051223) | 44801 | 0.025339342 | 0.132565845 |
| membrane protein ectodomain proteolysis (GO:0006509 | 44765 | 0.025446798 | 0.132727026 |
| positive regulation of epithelial cell apoptotic process (G( | 44765 | 0.025446798 | 0.132727026 |
| regulation of interleukin-4 production (GO:0032673) | 44765 | 0.025446798 | 0.132727026 |
| negative regulation of epidermal growth factor receptor : | 16377 | 0.025786287 | 0.134228217 |
| positive regulation of stress fiber assembly (GO:0051496) | 16377 | 0.025786287 | 0.134228217 |
| glycerophospholipid biosynthetic process (GO:0046474) | 33/177 | 0.026189068 | 0.136188395 |
| axon guidance (GO:0007411) | 37/203 | 0.02691506 | 0.139823735 |
| anion transmembrane transport (GO:0098656) | 15/67 | 0.027321711 | 0.141794494 |
| actin polymerization or depolymerization (GO:0008154) | 18598 | 0.027698242 | 0.143292246 |
| regulation of generation of precursor metabolites and en | 18598 | 0.027698242 | 0.143292246 |
| cellular response to ketone (GO:1901655) | 14519 | 0.027720637 | 0.143292246 |
| protein monoubiquitination (GO:0006513) | 14519 | 0.027720637 | 0.143292246 |
| regulation of actin filament polymerization (GO:0030833 | 16/73 | 0.027895075 | 0.144050609 |
| transcription initiation from RNA polymerase II promoter | 27/140 | 0.028069866 | 0.144809287 |
| glycosaminoglycan biosynthetic process (GO:0006024) | 20/97 | 0.02839031 | 0.146317124 |
| positive regulation of protein catabolic process (GO:0045 | 18/85 | 0.028438113 | 0.146418233 |
| negative regulation of cell differentiation (GO:0045596) | 35/191 | 0.028573466 | 0.146969462 |
| protein ubiquitination (GO:0016567) | 85/525 | 0.028720837 | 0.147271963 |
| antigen processing and presentation of endogenous pept | 44695 | 0.029085858 | 0.147271963 |
| cardiolipin metabolic process (GO:0032048) | 44695 | 0.029085858 | 0.147271963 |
| cytoplasmic sequestering of transcription factor (GO:004 | 44695 | 0.029085858 | 0.147271963 |
| detection of bacterium (GO:0016045) | 44695 | 0.029085858 | 0.147271963 |
| NAD biosynthesis via nicotinamide riboside salvage pathv | 44695 | 0.029085858 | 0.147271963 |
| negative regulation of secretion (GO:0051048) | 44695 | 0.029085858 | 0.147271963 |
| positive regulation of leukocyte degranulation (GO:0043: | 44695 | 0.029085858 | 0.147271963 |
| positive regulation of regulatory T cell differentiation (GC | 44695 | 0.029085858 | 0.147271963 |
| regulation of DNA methylation-dependent heterochroma | 44695 | 0.029085858 | 0.147271963 |
| regulation of response to endoplasmic reticulum stress (( | 44695 | 0.029085858 | 0.147271963 |
| regulation of trophoblast cell migration (GO:1901163) | 44695 | 0.029085858 | 0.147271963 |
| response to chemokine (GO:1990868) | 44695 | 0.029085858 | 0.147271963 |
| substrate-dependent cell migration (GO:0006929) | 44695 | 0.029085858 | 0.147271963 |
| T-helper cell differentiation (GO:0042093) | 44695 | 0.029085858 | 0.147271963 |
| vascular endothelial growth factor signaling pathway (GC | 44695 | 0.029085858 | 0.147271963 |
| blood vessel morphogenesis (GO:0048514) | 13/56 | 0.029133516 | 0.147369635 |
| autophagosome maturation (GO:0097352) | 12663 | 0.029534048 | 0.148960562 |
| protein destabilization (GO:0031648) | 12663 | 0.029534048 | 0.148960562 |
| regulation of transmembrane transport (GO:0034762) | 12663 | 0.029534048 | 0.148960562 |
| negative regulation of T cell activation (GO:0050868) | 16742 | 0.030166564 | 0.151855912 |

| | | | |
|---|---|---|---|
| regulation of insulin receptor signaling pathway (GO:004( | 16742 | 0.030166564 | 0.151855912 |
| positive regulation of protein localization to nucleus (GO: | 15/68 | 0.030896844 | 0.154538414 |
| regulation of myeloid cell differentiation (GO:0045637) | 15/68 | 0.030896844 | 0.154538414 |
| actin filament polymerization (GO:0030041) | 44802 | 0.031041991 | 0.154538414 |
| establishment of endothelial barrier (GO:0061028) | 44802 | 0.031041991 | 0.154538414 |
| positive regulation of dephosphorylation (GO:0035306) | 44802 | 0.031041991 | 0.154538414 |
| regulation of T cell mediated cytotoxicity (GO:0001914) | 44802 | 0.031041991 | 0.154538414 |
| mRNA transport (GO:0051028) | 21/104 | 0.03107154 | 0.154538414 |
| cellular response to amino acid starvation (GO:0034198) | 16/74 | 0.031353241 | 0.154538414 |
| regulation of stress fiber assembly (GO:0051492) | 16/74 | 0.031353241 | 0.154538414 |
| regulation of endothelial cell proliferation (GO:0001936) | 19/92 | 0.031572432 | 0.154538414 |
| negative regulation of cellular component organization (( | 17/80 | 0.031593083 | 0.154538414 |
| DNA alkylation (GO:0006305) | 44731 | 0.031619617 | 0.154538414 |
| DNA methylation or demethylation (GO:0044728) | 44731 | 0.031619617 | 0.154538414 |
| leukotriene biosynthetic process (GO:0019370) | 44731 | 0.031619617 | 0.154538414 |
| maintenance of protein localization in organelle (GO:007 | 44731 | 0.031619617 | 0.154538414 |
| negative regulation of RNA metabolic process (GO:00512 | 44731 | 0.031619617 | 0.154538414 |
| positive regulation of leukocyte activation (GO:0002696) | 44731 | 0.031619617 | 0.154538414 |
| positive regulation of monocyte chemotaxis (GO:090020 | 44731 | 0.031619617 | 0.154538414 |
| regulation of cell-cell adhesion mediated by cadherin (GC | 44731 | 0.031619617 | 0.154538414 |
| regulation of epithelial cell differentiation (GO:0030856) | 44731 | 0.031619617 | 0.154538414 |
| response to cholesterol (GO:0070723) | 44731 | 0.031619617 | 0.154538414 |
| phosphate-containing compound metabolic process (GO: | 38/212 | 0.031864143 | 0.154538414 |
| amino acid import across plasma membrane (GO:008971 | 44766 | 0.031925609 | 0.154538414 |
| defense response to fungus (GO:0050832) | 44766 | 0.031925609 | 0.154538414 |
| dendritic cell differentiation (GO:0097028) | 44766 | 0.031925609 | 0.154538414 |
| immune response-activating cell surface receptor signalir | 44766 | 0.031925609 | 0.154538414 |
| negative regulation of synaptic transmission (GO:005080 | 44766 | 0.031925609 | 0.154538414 |
| positive regulation of carbohydrate metabolic process (G | 44766 | 0.031925609 | 0.154538414 |
| positive regulation of cell-substrate junction organization | 44766 | 0.031925609 | 0.154538414 |
| positive regulation of p38MAPK cascade (GO:1900745) | 44766 | 0.031925609 | 0.154538414 |
| protein K48-linked deubiquitination (GO:0071108) | 44766 | 0.031925609 | 0.154538414 |
| regulation of axon extension involved in axon guidance (( | 44766 | 0.031925609 | 0.154538414 |
| regulation of monooxygenase activity (GO:0032768) | 44766 | 0.031925609 | 0.154538414 |
| positive regulation of actin filament bundle assembly (GC | 18963 | 0.031990874 | 0.154538414 |
| positive regulation of epithelial cell proliferation (GO:005 | 24/123 | 0.032478735 | 0.154538414 |
| lymphocyte migration (GO:0072676) | 14885 | 0.032706494 | 0.154538414 |
| negative regulation of blood coagulation (GO:0030195) | 14885 | 0.032706494 | 0.154538414 |
| chromatin organization (GO:0006325) | 27/142 | 0.033030888 | 0.154538414 |
| regulation of bone mineralization (GO:0030500) | 13/57 | 0.033310605 | 0.154538414 |
| arachidonate transport (GO:1903963) | 44661 | 0.03343311 | 0.154538414 |
| arachidonic acid secretion (GO:0050482) | 44661 | 0.03343311 | 0.154538414 |
| B cell homeostasis (GO:0001782) | 44661 | 0.03343311 | 0.154538414 |
| I-kappaB phosphorylation (GO:0007252) | 44661 | 0.03343311 | 0.154538414 |
| iron ion transmembrane transport (GO:0034755) | 44661 | 0.03343311 | 0.154538414 |
| myeloid dendritic cell differentiation (GO:0043011) | 44661 | 0.03343311 | 0.154538414 |
| negative regulation of cell-cell adhesion mediated by cad | 44661 | 0.03343311 | 0.154538414 |

| | | | |
|---|---|---|---|
| negative regulation of hormone secretion (GO:0046888) | 44661 | 0.03343311 | 0.154538414 |
| negative regulation of interferon-beta production (GO:0C | 44661 | 0.03343311 | 0.154538414 |
| negative regulation of transcription by competitive prom | 44661 | 0.03343311 | 0.154538414 |
| negative regulation of transmembrane transport (GO:003 | 44661 | 0.03343311 | 0.154538414 |
| negative regulation of tyrosine phosphorylation of STAT p | 44661 | 0.03343311 | 0.154538414 |
| negative regulation of vascular endothelial growth factor | 44661 | 0.03343311 | 0.154538414 |
| negative regulation of voltage-gated calcium channel acti | 44661 | 0.03343311 | 0.154538414 |
| peptidyl-lysine acetylation (GO:0018394) | 44661 | 0.03343311 | 0.154538414 |
| phosphatidylcholine catabolic process (GO:0034638) | 44661 | 0.03343311 | 0.154538414 |
| pinocytosis (GO:0006907) | 44661 | 0.03343311 | 0.154538414 |
| positive regulation of coagulation (GO:0050820) | 44661 | 0.03343311 | 0.154538414 |
| positive regulation of hemostasis (GO:1900048) | 44661 | 0.03343311 | 0.154538414 |
| positive regulation of myoblast fusion (GO:1901741) | 44661 | 0.03343311 | 0.154538414 |
| regulation of endoplasmic reticulum unfolded protein res | 44661 | 0.03343311 | 0.154538414 |
| regulation of granulocyte differentiation (GO:0030852) | 44661 | 0.03343311 | 0.154538414 |
| regulation of hydrogen peroxide-induced cell death (GO:1 | 44661 | 0.03343311 | 0.154538414 |
| regulation of macrophage migration (GO:1905521) | 44661 | 0.03343311 | 0.154538414 |
| regulation of memory T cell differentiation (GO:0043380) | 44661 | 0.03343311 | 0.154538414 |
| regulation of podosome assembly (GO:0071801) | 44661 | 0.03343311 | 0.154538414 |
| regulation of tau-protein kinase activity (GO:1902947) | 44661 | 0.03343311 | 0.154538414 |
| response to interleukin-4 (GO:0070670) | 44661 | 0.03343311 | 0.154538414 |
| astrocyte development (GO:0014002) | 44626 | 0.034150164 | 0.154538414 |
| cellular response to salt (GO:1902075) | 44626 | 0.034150164 | 0.154538414 |
| cellular triglyceride homeostasis (GO:0035356) | 44626 | 0.034150164 | 0.154538414 |
| chitin catabolic process (GO:0006032) | 44626 | 0.034150164 | 0.154538414 |
| chitin metabolic process (GO:0006030) | 44626 | 0.034150164 | 0.154538414 |
| contact inhibition (GO:0060242) | 44626 | 0.034150164 | 0.154538414 |
| detection of virus (GO:0009597) | 44626 | 0.034150164 | 0.154538414 |
| ear development (GO:0043583) | 44626 | 0.034150164 | 0.154538414 |
| hexose import across plasma membrane (GO:0140271) | 44626 | 0.034150164 | 0.154538414 |
| leukocyte chemotaxis involved in inflammatory response | 44626 | 0.034150164 | 0.154538414 |
| membrane raft assembly (GO:0001765) | 44626 | 0.034150164 | 0.154538414 |
| multinuclear osteoclast differentiation (GO:0072674) | 44626 | 0.034150164 | 0.154538414 |
| negative regulation of CD8-positive, alpha-beta T cell acti | 44626 | 0.034150164 | 0.154538414 |
| negative regulation of cytosolic calcium ion concentratio | 44626 | 0.034150164 | 0.154538414 |
| negative regulation of lens fiber cell differentiation (GO:1 | 44626 | 0.034150164 | 0.154538414 |
| negative regulation of myeloid cell apoptotic process (GC | 44626 | 0.034150164 | 0.154538414 |
| negative regulation of myeloid leukocyte mediated immu | 44626 | 0.034150164 | 0.154538414 |
| negative regulation of relaxation of muscle (GO:1901078 | 44626 | 0.034150164 | 0.154538414 |
| negative regulation of transporter activity (GO:0032410) | 44626 | 0.034150164 | 0.154538414 |
| negative regulation of vitamin metabolic process (GO:004 | 44626 | 0.034150164 | 0.154538414 |
| neutrophil extravasation (GO:0072672) | 44626 | 0.034150164 | 0.154538414 |
| norepinephrine transport (GO:0015874) | 44626 | 0.034150164 | 0.154538414 |
| positive regulation of antigen processing and presentatio | 44626 | 0.034150164 | 0.154538414 |
| positive regulation of deacetylase activity (GO:0090045) | 44626 | 0.034150164 | 0.154538414 |
| positive regulation of dendritic cell antigen processing an | 44626 | 0.034150164 | 0.154538414 |
| positive regulation of fever generation (GO:0031622) | 44626 | 0.034150164 | 0.154538414 |

| | | | |
|---|---|---|---|
| positive regulation of focal adhesion disassembly (GO:01: | 44626 | 0.034150164 | 0.154538414 |
| positive regulation of granulocyte differentiation (GO:003 | 44626 | 0.034150164 | 0.154538414 |
| positive regulation of hemoglobin biosynthetic process (( | 44626 | 0.034150164 | 0.154538414 |
| positive regulation of microglial cell migration (GO:19041 | 44626 | 0.034150164 | 0.154538414 |
| positive regulation of natural killer cell mediated cytotoxi | 44626 | 0.034150164 | 0.154538414 |
| positive regulation of natural killer cell mediated immune | 44626 | 0.034150164 | 0.154538414 |
| positive regulation of non-membrane spanning protein ty | 44626 | 0.034150164 | 0.154538414 |
| positive regulation of plasminogen activation (GO:00107! | 44626 | 0.034150164 | 0.154538414 |
| positive regulation of receptor binding (GO:1900122) | 44626 | 0.034150164 | 0.154538414 |
| positive regulation of sterol transport (GO:0032373) | 44626 | 0.034150164 | 0.154538414 |
| positive regulation of T-helper 2 cell differentiation (GO:( | 44626 | 0.034150164 | 0.154538414 |
| positive regulation of toll-like receptor 3 signaling pathwa | 44626 | 0.034150164 | 0.154538414 |
| regulation of centriole elongation (GO:1903722) | 44626 | 0.034150164 | 0.154538414 |
| regulation of focal adhesion disassembly (GO:0120182) | 44626 | 0.034150164 | 0.154538414 |
| regulation of hormone biosynthetic process (GO:004688! | 44626 | 0.034150164 | 0.154538414 |
| regulation of immunoglobulin mediated immune respons | 44626 | 0.034150164 | 0.154538414 |
| regulation of macrophage apoptotic process (GO:200010 | 44626 | 0.034150164 | 0.154538414 |
| regulation of nucleotide-binding oligomerization domain | 44626 | 0.034150164 | 0.154538414 |
| regulation of pro-B cell differentiation (GO:2000973) | 44626 | 0.034150164 | 0.154538414 |
| regulation of type B pancreatic cell proliferation (GO:006 | 44626 | 0.034150164 | 0.154538414 |
| release of sequestered calcium ion into cytosol by sarcop | 44626 | 0.034150164 | 0.154538414 |
| ruffle assembly (GO:0097178) | 44626 | 0.034150164 | 0.154538414 |
| TORC2 signaling (GO:0038203) | 44626 | 0.034150164 | 0.154538414 |
| positive regulation of nucleocytoplasmic transport (GO:0 | 17107 | 0.035052715 | 0.158170874 |
| regulation of transcription from RNA polymerase II prom | 16/75 | 0.035116599 | 0.158170874 |
| response to amino acid starvation (GO:1990928) | 16/75 | 0.035116599 | 0.158170874 |
| protein targeting to membrane (GO:0006612) | 18/87 | 0.035134851 | 0.158170874 |
| negative regulation of protein modification process (GO:( | 17/81 | 0.035213504 | 0.158170874 |
| phospholipase C-activating G protein-coupled receptor si | 17/81 | 0.035213504 | 0.158170874 |
| bicarbonate transport (GO:0015701) | 13028 | 0.035226891 | 0.158170874 |
| cellular response to amyloid-beta (GO:1904646) | 13028 | 0.035226891 | 0.158170874 |
| positive regulation of lipid biosynthetic process (GO:0046 | 13028 | 0.035226891 | 0.158170874 |
| regulation of canonical Wnt signaling pathway (GO:00608 | 44/253 | 0.035370538 | 0.15867871 |
| nitrogen compound transport (GO:0071705) | 27/143 | 0.035747488 | 0.160231408 |
| negative regulation of protein metabolic process (GO:005 | 19329 | 0.03673941 | 0.164535548 |
| transcription, DNA-templated (GO:0006351) | 39/221 | 0.03726422 | 0.16674214 |
| cellular response to peptide hormone stimulus (GO:0071 | 21/106 | 0.037450002 | 0.166788531 |
| peptidyl-lysine modification (GO:0018205) | 21/106 | 0.037450002 | 0.166788531 |
| cell projection organization (GO:0030030) | 44803 | 0.037563538 | 0.166788531 |
| chondroitin sulfate metabolic process (GO:0030204) | 44803 | 0.037563538 | 0.166788531 |
| negative regulation of gene expression, epigenetic (GO:0 | 44803 | 0.037563538 | 0.166788531 |
| negative regulation of interleukin-1 production (GO:0032 | 44803 | 0.037563538 | 0.166788531 |
| plasma membrane invagination (GO:0099024) | 44803 | 0.037563538 | 0.166788531 |
| positive regulation of lipid kinase activity (GO:0090218) | 44803 | 0.037563538 | 0.166788531 |
| positive regulation of protein import (GO:1904591) | 44803 | 0.037563538 | 0.166788531 |
| positive regulation of protein binding (GO:0032092) | 13/58 | 0.03790118 | 0.168144004 |
| nucleobase-containing compound catabolic process (GO: | 15250 | 0.038287987 | 0.169178771 |

| | | | |
|---|---|---|---|
| positive regulation of TOR signaling (GO:0032008) | 15250 | 0.038287987 | 0.169178771 |
| regulation of viral transcription (GO:0046782) | 15250 | 0.038287987 | 0.169178771 |
| androgen receptor signaling pathway (GO:0030521) | 44696 | 0.038981134 | 0.169178771 |
| cellular response to estrogen stimulus (GO:0071391) | 44696 | 0.038981134 | 0.169178771 |
| cellular response to exogenous dsRNA (GO:0071360) | 44696 | 0.038981134 | 0.169178771 |
| fibrinolysis (GO:0042730) | 44696 | 0.038981134 | 0.169178771 |
| glucan catabolic process (GO:0009251) | 44696 | 0.038981134 | 0.169178771 |
| glycogen catabolic process (GO:0005980) | 44696 | 0.038981134 | 0.169178771 |
| intracellular cholesterol transport (GO:0032367) | 44696 | 0.038981134 | 0.169178771 |
| myoblast differentiation (GO:0045445) | 44696 | 0.038981134 | 0.169178771 |
| negative regulation of ubiquitin-protein transferase activ | 44696 | 0.038981134 | 0.169178771 |
| neurotrophin TRK receptor signaling pathway (GO:00480 | 44696 | 0.038981134 | 0.169178771 |
| positive regulation of exosomal secretion (GO:1903543) | 44696 | 0.038981134 | 0.169178771 |
| positive regulation of heterotypic cell-cell adhesion (GO:( | 44696 | 0.038981134 | 0.169178771 |
| positive regulation of membrane protein ectodomain prc | 44696 | 0.038981134 | 0.169178771 |
| positive regulation of pattern recognition receptor signal | 44696 | 0.038981134 | 0.169178771 |
| positive regulation of sprouting angiogenesis (GO:190367 | 44696 | 0.038981134 | 0.169178771 |
| pyrimidine nucleoside metabolic process (GO:0006213) | 44696 | 0.038981134 | 0.169178771 |
| regulation of dephosphorylation (GO:0035303) | 44696 | 0.038981134 | 0.169178771 |
| regulation of extracellular matrix disassembly (GO:00107 | 44696 | 0.038981134 | 0.169178771 |
| regulation of integrin activation (GO:0033623) | 44696 | 0.038981134 | 0.169178771 |
| regulation of nuclear-transcribed mRNA poly(A) tail short | 44696 | 0.038981134 | 0.169178771 |
| regulation of T cell chemotaxis (GO:0010819) | 44696 | 0.038981134 | 0.169178771 |
| response to acidic pH (GO:0010447) | 44696 | 0.038981134 | 0.169178771 |
| T cell proliferation (GO:0042098) | 44696 | 0.038981134 | 0.169178771 |
| carbohydrate homeostasis (GO:0033500) | 15/70 | 0.039054893 | 0.169357402 |
| RNA transport (GO:0050658) | 16/76 | 0.039198214 | 0.169837132 |
| negative regulation of actin filament polymerization (GO: | 44767 | 0.039419792 | 0.170087889 |
| negative regulation of antigen receptor-mediated signalir | 44767 | 0.039419792 | 0.170087889 |
| peptide catabolic process (GO:0043171) | 44767 | 0.039419792 | 0.170087889 |
| phosphatidylglycerol metabolic process (GO:0046471) | 44767 | 0.039419792 | 0.170087889 |
| regulation of epidermal cell differentiation (GO:0045604) | 44767 | 0.039419792 | 0.170087889 |
| endoplasmic reticulum calcium ion homeostasis (GO:003 | 44732 | 0.040246596 | 0.172509132 |
| eye morphogenesis (GO:0048592) | 44732 | 0.040246596 | 0.172509132 |
| glucose transmembrane transport (GO:1904659) | 44732 | 0.040246596 | 0.172509132 |
| granulocyte differentiation (GO:0030851) | 44732 | 0.040246596 | 0.172509132 |
| myeloid cell development (GO:0061515) | 44732 | 0.040246596 | 0.172509132 |
| negative regulation of viral life cycle (GO:1903901) | 44732 | 0.040246596 | 0.172509132 |
| regulation of acute inflammatory response (GO:0002673 | 44732 | 0.040246596 | 0.172509132 |
| regulation of tissue remodeling (GO:0034103) | 44732 | 0.040246596 | 0.172509132 |
| polyol metabolic process (GO:0019751) | 17472 | 0.040469475 | 0.173178686 |
| regulation of DNA binding (GO:0051101) | 17472 | 0.040469475 | 0.173178686 |
| positive regulation of mitotic nuclear division (GO:00458 | 13394 | 0.041629211 | 0.177848481 |
| regulation of protein import into nucleus (GO:0042306) | 13394 | 0.041629211 | 0.177848481 |
| cellular macromolecule catabolic process (GO:0044265) | 19694 | 0.041964597 | 0.178986931 |
| epidermal cell differentiation (GO:0009913) | 19694 | 0.041964597 | 0.178986931 |
| regulation of cellular component organization (GO:00511 | 26/139 | 0.042866621 | 0.182625075 |

| | | | |
|---|---|---|---|
| protein polymerization (GO:0051258) | 13/59 | 0.042923045 | 0.182625075 |
| regulation of secretion by cell (GO:1903530) | 13/59 | 0.042923045 | 0.182625075 |
| mitotic G1 DNA damage checkpoint signaling (GO:003157 | 14/65 | 0.043460399 | 0.184277925 |
| regulation of cellular catabolic process (GO:0031329) | 14/65 | 0.043460399 | 0.184277925 |
| regulation of RNA metabolic process (GO:0051252) | 14/65 | 0.043460399 | 0.184277925 |
| cellular response to UV (GO:0034644) | 16/77 | 0.043610453 | 0.184277925 |
| phosphatidylcholine metabolic process (GO:0046470) | 16/77 | 0.043610453 | 0.184277925 |
| gland development (GO:0048732) | 15/71 | 0.043666242 | 0.184277925 |
| regulation of endopeptidase activity (GO:0052548) | 15/71 | 0.043666242 | 0.184277925 |
| regulation of glycolytic process (GO:0006110) | 15/71 | 0.043666242 | 0.184277925 |
| regulation of TOR signaling (GO:0032006) | 15/71 | 0.043666242 | 0.184277925 |
| snRNA transcription (GO:0009301) | 15/71 | 0.043666242 | 0.184277925 |
| activation of protein kinase activity (GO:0032147) | 22/114 | 0.043783746 | 0.184474096 |
| histone modification (GO:0016570) | 22/114 | 0.043783746 | 0.184474096 |
| positive regulation of epithelial to mesenchymal transitio | 15615 | 0.044492294 | 0.18730751 |
| phospholipid translocation (GO:0045332) | 44804 | 0.044943251 | 0.18874712 |
| positive regulation of RNA splicing (GO:0033120) | 44804 | 0.044943251 | 0.18874712 |
| regulation of cellular component size (GO:0032535) | 44804 | 0.044943251 | 0.18874712 |
| inositol phosphate metabolic process (GO:0043647) | 17838 | 0.046439139 | 0.194374098 |
| B cell mediated immunity (GO:0019724) | 44662 | 0.04714367 | 0.194374098 |
| cellular response to increased oxygen levels (GO:003629! | 44662 | 0.04714367 | 0.194374098 |
| fatty acid homeostasis (GO:0055089) | 44662 | 0.04714367 | 0.194374098 |
| Golgi to lysosome transport (GO:0090160) | 44662 | 0.04714367 | 0.194374098 |
| hepatocyte growth factor receptor signaling pathway (GC | 44662 | 0.04714367 | 0.194374098 |
| induction of positive chemotaxis (GO:0050930) | 44662 | 0.04714367 | 0.194374098 |
| miRNA mediated inhibition of translation (GO:0035278) | 44662 | 0.04714367 | 0.194374098 |
| negative regulation of translation, ncRNA-mediated (GO:( | 44662 | 0.04714367 | 0.194374098 |
| positive regulation of cellular respiration (GO:1901857) | 44662 | 0.04714367 | 0.194374098 |
| positive regulation of ceramide biosynthetic process (GO | 44662 | 0.04714367 | 0.194374098 |
| positive regulation of leukocyte apoptotic process (GO:2( | 44662 | 0.04714367 | 0.194374098 |
| positive regulation of lipid localization (GO:1905954) | 44662 | 0.04714367 | 0.194374098 |
| positive regulation of sphingolipid biosynthetic process (( | 44662 | 0.04714367 | 0.194374098 |
| positive regulation of transcription from RNA polymerase | 44662 | 0.04714367 | 0.194374098 |
| positive regulation of triglyceride biosynthetic process (G | 44662 | 0.04714367 | 0.194374098 |
| regulation of amyloid precursor protein biosynthetic proc | 44662 | 0.04714367 | 0.194374098 |
| regulation of lamellipodium morphogenesis (GO:200039. | 44662 | 0.04714367 | 0.194374098 |
| regulation of lymphocyte migration (GO:2000401) | 44662 | 0.04714367 | 0.194374098 |
| regulation of T cell mediated immunity (GO:0002709) | 44662 | 0.04714367 | 0.194374098 |
| regulation of toll-like receptor 9 signaling pathway (GO:0 | 44662 | 0.04714367 | 0.194374098 |
| response to corticosteroid (GO:0031960) | 44662 | 0.04714367 | 0.194374098 |
| synaptic vesicle transport (GO:0048489) | 44662 | 0.04714367 | 0.194374098 |
| endothelial cell development (GO:0001885) | 44768 | 0.047974351 | 0.197329178 |
| positive regulation of filopodium assembly (GO:0051491) | 44768 | 0.047974351 | 0.197329178 |
| positive regulation of ion transmembrane transporter act | 44768 | 0.047974351 | 0.197329178 |
| negative regulation of blood vessel morphogenesis (GO:2 | 16/78 | 0.048364878 | 0.198778118 |
| response to ionizing radiation (GO:0010212) | 15/72 | 0.048649877 | 0.199791391 |
| negative regulation of G1/S transition of mitotic cell cycle | 13759 | 0.048770595 | 0.20012894 |

| | | | |
|---|---|---|---|
| extrinsic apoptotic signaling pathway in absence of ligand | 44733 | 0.050225343 | 0.204804284 |
| intrinsic apoptotic signaling pathway in response to DNA | 44733 | 0.050225343 | 0.204804284 |
| neurotrophin signaling pathway (GO:0038179) | 44733 | 0.050225343 | 0.204804284 |
| positive regulation of glycolytic process (GO:0045821) | 44733 | 0.050225343 | 0.204804284 |
| positive regulation of immune system process (GO:00026 | 44733 | 0.050225343 | 0.204804284 |
| positive regulation of lipid storage (GO:0010884) | 44733 | 0.050225343 | 0.204804284 |
| positive regulation of purine nucleotide metabolic proces | 44733 | 0.050225343 | 0.204804284 |
| regulation of transcription by RNA polymerase III (GO:00( | 44733 | 0.050225343 | 0.204804284 |
| aromatic amino acid family catabolic process (GO:000907 | 44697 | 0.05068681 | 0.205076308 |
| B cell activation involved in immune response (GO:00023 | 44697 | 0.05068681 | 0.205076308 |
| negative regulation of receptor signaling pathway via JAK | 44697 | 0.05068681 | 0.205076308 |
| positive regulation of endothelial cell apoptotic process ( | 44697 | 0.05068681 | 0.205076308 |
| positive regulation of syncytium formation by plasma me | 44697 | 0.05068681 | 0.205076308 |
| regulation of amyloid-beta clearance (GO:1900221) | 44697 | 0.05068681 | 0.205076308 |
| regulation of cholesterol storage (GO:0010885) | 44697 | 0.05068681 | 0.205076308 |
| regulation of defense response to virus (GO:0050688) | 44697 | 0.05068681 | 0.205076308 |
| regulation of intrinsic apoptotic signaling pathway in resp | 44697 | 0.05068681 | 0.205076308 |
| regulation of mast cell degranulation (GO:0043304) | 44697 | 0.05068681 | 0.205076308 |
| regulation of cellular protein metabolic process (GO:0032 | 18203 | 0.052981256 | 0.210438348 |
| ERBB2 signaling pathway (GO:0038128) | 11902 | 0.053212802 | 0.210438348 |
| gene silencing by miRNA (GO:0035195) | 11902 | 0.053212802 | 0.210438348 |
| hematopoietic progenitor cell differentiation (GO:000224 | 11902 | 0.053212802 | 0.210438348 |
| negative regulation of chemotaxis (GO:0050922) | 11902 | 0.053212802 | 0.210438348 |
| positive regulation of receptor signaling pathway via STA | 11902 | 0.053212802 | 0.210438348 |
| positive regulation of proteasomal ubiquitin-dependent p | 16/79 | 0.053472145 | 0.210438348 |
| negative regulation of protein modification by small prot | 20424 | 0.05391816 | 0.210438348 |
| astrocyte differentiation (GO:0048708) | 44627 | 0.053956473 | 0.210438348 |
| autophagosome membrane docking (GO:0016240) | 44627 | 0.053956473 | 0.210438348 |
| catecholamine uptake (GO:0090493) | 44627 | 0.053956473 | 0.210438348 |
| cellular response to laminar fluid shear stress (GO:00714 | 44627 | 0.053956473 | 0.210438348 |
| cellular response to platelet-derived growth factor stimul | 44627 | 0.053956473 | 0.210438348 |
| detection of molecule of bacterial origin (GO:0032490) | 44627 | 0.053956473 | 0.210438348 |
| glial cell migration (GO:0008347) | 44627 | 0.053956473 | 0.210438348 |
| hyaluronan biosynthetic process (GO:0030213) | 44627 | 0.053956473 | 0.210438348 |
| hyperosmotic response (GO:0006972) | 44627 | 0.053956473 | 0.210438348 |
| mRNA transcription by RNA polymerase II (GO:0042789) | 44627 | 0.053956473 | 0.210438348 |
| negative regulation of oligodendrocyte differentiation (G | 44627 | 0.053956473 | 0.210438348 |
| negative regulation of type 2 immune response (GO:0002 | 44627 | 0.053956473 | 0.210438348 |
| positive regulation of B cell receptor signaling pathway (( | 44627 | 0.053956473 | 0.210438348 |
| positive regulation of gliogenesis (GO:0014015) | 44627 | 0.053956473 | 0.210438348 |
| positive regulation of histone H3-K9 methylation (GO:005 | 44627 | 0.053956473 | 0.210438348 |
| positive regulation of humoral immune response (GO:00( | 44627 | 0.053956473 | 0.210438348 |
| positive regulation of IRE1-mediated unfolded protein re | 44627 | 0.053956473 | 0.210438348 |
| positive regulation of lipopolysaccharide-mediated signal | 44627 | 0.053956473 | 0.210438348 |
| positive regulation of lymphocyte apoptotic process (GO: | 44627 | 0.053956473 | 0.210438348 |
| positive regulation of protein deubiquitination (GO:1903( | 44627 | 0.053956473 | 0.210438348 |
| positive regulation of respiratory burst (GO:0060267) | 44627 | 0.053956473 | 0.210438348 |

| | | | |
|---|---|---|---|
| positive regulation of trophoblast cell migration (GO:190 | 44627 | 0.053956473 | 0.210438348 |
| posttranslational protein targeting to membrane, translo | 44627 | 0.053956473 | 0.210438348 |
| protein catabolic process in the vacuole (GO:0007039) | 44627 | 0.053956473 | 0.210438348 |
| regulation of glial cell apoptotic process (GO:0034350) | 44627 | 0.053956473 | 0.210438348 |
| regulation of hemoglobin biosynthetic process (GO:0046 | 44627 | 0.053956473 | 0.210438348 |
| regulation of histone deacetylase activity (GO:1901725) | 44627 | 0.053956473 | 0.210438348 |
| regulation of I-kappaB phosphorylation (GO:1903719) | 44627 | 0.053956473 | 0.210438348 |
| regulation of inositol phosphate biosynthetic process (GC | 44627 | 0.053956473 | 0.210438348 |
| regulation of lens fiber cell differentiation (GO:1902746) | 44627 | 0.053956473 | 0.210438348 |
| regulation of MHC class I biosynthetic process (GO:0045: | 44627 | 0.053956473 | 0.210438348 |
| regulation of microglial cell migration (GO:1904139) | 44627 | 0.053956473 | 0.210438348 |
| regulation of myosin-light-chain-phosphatase activity (GC | 44627 | 0.053956473 | 0.210438348 |
| regulation of natural killer cell chemotaxis (GO:2000501) | 44627 | 0.053956473 | 0.210438348 |
| regulation of natural killer cell mediated cytotoxicity dire | 44627 | 0.053956473 | 0.210438348 |
| regulation of non-membrane spanning protein tyrosine k | 44627 | 0.053956473 | 0.210438348 |
| ribonucleoprotein complex disassembly (GO:0032988) | 44627 | 0.053956473 | 0.210438348 |
| substrate-dependent cell migration, cell extension (GO:0 | 44627 | 0.053956473 | 0.210438348 |
| T cell apoptotic process (GO:0070231) | 44627 | 0.053956473 | 0.210438348 |
| vascular associated smooth muscle cell development (GC | 44627 | 0.053956473 | 0.210438348 |
| positive regulation of catabolic process (GO:0009896) | 14/67 | 0.054325583 | 0.211718983 |
| cellular component assembly (GO:0022607) | 44/261 | 0.054889604 | 0.213756741 |
| acute inflammatory response (GO:0002526) | 44769 | 0.057622068 | 0.222728156 |
| cellular lipid catabolic process (GO:0044242) | 44769 | 0.057622068 | 0.222728156 |
| cellular response to unfolded protein (GO:0034620) | 44769 | 0.057622068 | 0.222728156 |
| entrainment of circadian clock by photoperiod (GO:0043: | 44769 | 0.057622068 | 0.222728156 |
| L-alpha-amino acid transmembrane transport (GO:19024 | 44769 | 0.057622068 | 0.222728156 |
| negative regulation of endothelial cell apoptotic process | 44769 | 0.057622068 | 0.222728156 |
| positive regulation of gene silencing by miRNA (GO:2000 | 44769 | 0.057622068 | 0.222728156 |
| positive regulation of mitotic cell cycle (GO:0045931) | 44769 | 0.057622068 | 0.222728156 |
| regulation of epidermal growth factor-activated receptor | 44769 | 0.057622068 | 0.222728156 |
| response to glucocorticoid (GO:0051384) | 44769 | 0.057622068 | 0.222728156 |
| glucose homeostasis (GO:0042593) | 17/86 | 0.0579411 | 0.223628541 |
| regulation of neuron death (GO:1901214) | 17/86 | 0.0579411 | 0.223628541 |
| negative regulation of protein binding (GO:0032091) | 15/74 | 0.059779604 | 0.230553115 |
| iron ion transport (GO:0006826) | 18568 | 0.060112367 | 0.231664501 |
| negative regulation of transforming growth factor beta re | 14/68 | 0.060399704 | 0.232599305 |
| adenylate cyclase-activating G protein-coupled receptor : | 22/118 | 0.060530017 | 0.232928475 |
| response to UV (GO:0009411) | 19/99 | 0.060603115 | 0.232978711 |
| DNA damage response, signal transduction by p53 class r | 20790 | 0.060677612 | 0.232978711 |
| protein-containing complex disassembly (GO:0032984) | 20790 | 0.060677612 | 0.232978711 |
| glutamate metabolic process (GO:0006536) | 44734 | 0.061587229 | 0.235427267 |
| negative regulation of biomineral tissue development (G( | 44734 | 0.061587229 | 0.235427267 |
| negative regulation of lymphocyte activation (GO:005125 | 44734 | 0.061587229 | 0.235427267 |
| positive regulation of DNA-templated transcription, initia | 44734 | 0.061587229 | 0.235427267 |
| positive regulation of signal transduction by p53 class me | 44734 | 0.061587229 | 0.235427267 |
| regulation of response to cytokine stimulus (GO:0060759 | 44734 | 0.061587229 | 0.235427267 |
| regulation of macroautophagy (GO:0016241) | 21/112 | 0.062320964 | 0.238056918 |

| | | | |
|---|---|---|---|
| protein targeting (GO:0006605) | 23/125 | 0.063324829 | 0.239556183 |
| regulation of protein secretion (GO:0050708) | 23/125 | 0.063324829 | 0.239556183 |
| ERAD pathway (GO:0036503) | 17/87 | 0.063479764 | 0.239556183 |
| cellular response to growth hormone stimulus (GO:0071: | 44663 | 0.063497375 | 0.239556183 |
| inositol phosphate catabolic process (GO:0071545) | 44663 | 0.063497375 | 0.239556183 |
| negative regulation of B cell proliferation (GO:0030889) | 44663 | 0.063497375 | 0.239556183 |
| negative regulation of cellular response to vascular endo | 44663 | 0.063497375 | 0.239556183 |
| negative regulation of heart contraction (GO:0045822) | 44663 | 0.063497375 | 0.239556183 |
| negative regulation of JUN kinase activity (GO:0043508) | 44663 | 0.063497375 | 0.239556183 |
| negative regulation of production of miRNAs involved in ; | 44663 | 0.063497375 | 0.239556183 |
| phospholipid efflux (GO:0033700) | 44663 | 0.063497375 | 0.239556183 |
| positive regulation of cellular senescence (GO:2000774) | 44663 | 0.063497375 | 0.239556183 |
| positive regulation of endoplasmic reticulum unfolded pr | 44663 | 0.063497375 | 0.239556183 |
| regulation of amyloid precursor protein catabolic process | 44663 | 0.063497375 | 0.239556183 |
| regulation of B cell apoptotic process (GO:0002902) | 44663 | 0.063497375 | 0.239556183 |
| regulation of plasminogen activation (GO:0010755) | 44663 | 0.063497375 | 0.239556183 |
| toll-like receptor 3 signaling pathway (GO:0034138) | 44663 | 0.063497375 | 0.239556183 |
| protein processing (GO:0016485) | 20/106 | 0.064115159 | 0.240385859 |
| negative regulation of ATPase activity (GO:0032780) | 44698 | 0.064226289 | 0.240385859 |
| negative regulation of intrinsic apoptotic signaling pathw | 44698 | 0.064226289 | 0.240385859 |
| positive regulation of blood coagulation (GO:0030194) | 44698 | 0.064226289 | 0.240385859 |
| positive regulation of macrophage activation (GO:0430: | 44698 | 0.064226289 | 0.240385859 |
| positive regulation of muscle hypertrophy (GO:0014742) | 44698 | 0.064226289 | 0.240385859 |
| positive regulation of positive chemotaxis (GO:0050927) | 44698 | 0.064226289 | 0.240385859 |
| regulation of cell killing (GO:0031341) | 44698 | 0.064226289 | 0.240385859 |
| regulation of cellular respiration (GO:0043457) | 44698 | 0.064226289 | 0.240385859 |
| retrograde protein transport, ER to cytosol (GO:0030970) | 44698 | 0.064226289 | 0.240385859 |
| vasculature development (GO:0001944) | 44698 | 0.064226289 | 0.240385859 |
| negative regulation of lymphocyte proliferation (GO:005( | 14489 | 0.065361646 | 0.243932294 |
| negative regulation of protein secretion (GO:0050709) | 14489 | 0.065361646 | 0.243932294 |
| regulation of nitric-oxide synthase activity (GO:0050999) | 14489 | 0.065361646 | 0.243932294 |
| regulation of T cell differentiation (GO:0045580) | 14489 | 0.065361646 | 0.243932294 |
| negative regulation of canonical Wnt signaling pathway (( | 29/165 | 0.067705382 | 0.252316684 |
| regulation of neuron projection development (GO:00109 | 29/165 | 0.067705382 | 0.252316684 |
| regulation of axonogenesis (GO:0050770) | 18933 | 0.067845782 | 0.252477679 |
| regulation of substrate adhesion-dependent cell spreadir | 18933 | 0.067845782 | 0.252477679 |
| icosanoid metabolic process (GO:0006690) | 21155 | 0.067975485 | 0.252598458 |
| regulation of reactive oxygen species metabolic process ( | 21155 | 0.067975485 | 0.252598458 |
| negative regulation of oxidative stress-induced cell death | 44770 | 0.068382984 | 0.253749715 |
| photoperiodism (GO:0009648) | 44770 | 0.068382984 | 0.253749715 |
| transmembrane receptor protein serine/threonine kinase | 24/133 | 0.07075088 | 0.26234891 |
| positive regulation of cytoskeleton organization (GO:005: | 16/82 | 0.071002079 | 0.263092583 |
| protein transport (GO:0015031) | 59/369 | 0.072091267 | 0.266938085 |
| cellular response to amino acid stimulus (GO:0071230) | 12632 | 0.072507006 | 0.268095301 |
| positive regulation of protein dephosphorylation (GO:00: | 12632 | 0.072507006 | 0.268095301 |
| defense response to Gram-positive bacterium (GO:00508 | 14/70 | 0.073880313 | 0.272747351 |
| snRNA transcription by RNA polymerase II (GO:0042795) | 14/70 | 0.073880313 | 0.272747351 |

| | | | |
|---|---|---|---|
| diacylglycerol metabolic process (GO:0046339) | 44735 | 0.074342685 | 0.272747351 |
| DNA methylation (GO:0006306) | 44735 | 0.074342685 | 0.272747351 |
| hexose transmembrane transport (GO:0008645) | 44735 | 0.074342685 | 0.272747351 |
| hyaluronan metabolic process (GO:0030212) | 44735 | 0.074342685 | 0.272747351 |
| negative regulation of G protein-coupled receptor signali | 44735 | 0.074342685 | 0.272747351 |
| peptidyl-L-cysteine S-palmitoylation (GO:0018230) | 44735 | 0.074342685 | 0.272747351 |
| peptidyl-S-diacylglycerol-L-cysteine biosynthetic process | 44735 | 0.074342685 | 0.272747351 |
| positive regulation of cholesterol efflux (GO:0010875) | 44735 | 0.074342685 | 0.272747351 |
| regulation of mononuclear cell migration (GO:0071675) | 44735 | 0.074342685 | 0.272747351 |
| gene expression (GO:0010467) | 57/356 | 0.074406549 | 0.272789007 |
| plasma membrane organization (GO:0007009) | 13/64 | 0.075009566 | 0.274805849 |
| autophagosome assembly (GO:0000045) | 21520 | 0.07582096 | 0.277192039 |
| positive regulation of mitochondrion organization (GO:0( | 21520 | 0.07582096 | 0.277192039 |
| positive regulation of mitotic cell cycle phase transition ( | 21520 | 0.07582096 | 0.277192039 |
| positive regulation of organelle assembly (GO:1902117) | 17076 | 0.075947975 | 0.27719684 |
| regulation of fibroblast proliferation (GO:0048145) | 17076 | 0.075947975 | 0.27719684 |
| negative regulation of cellular biosynthetic process (GO:( | 19299 | 0.076191399 | 0.27719684 |
| positive regulation of protein localization to plasma mem | 19299 | 0.076191399 | 0.27719684 |
| sprouting angiogenesis (GO:0002040) | 19299 | 0.076191399 | 0.27719684 |
| positive regulation of cellular protein metabolic process ( | 19/102 | 0.077421024 | 0.27719684 |
| regulation of protein catabolic process (GO:0042176) | 19/102 | 0.077421024 | 0.27719684 |
| positive regulation of plasma membrane bounded cell pr | 16/83 | 0.077606054 | 0.27719684 |
| alpha-beta T cell activation (GO:0046631) | 44628 | 0.078009967 | 0.27719684 |
| basic amino acid transport (GO:0015802) | 44628 | 0.078009967 | 0.27719684 |
| benzene-containing compound metabolic process (GO:0( | 44628 | 0.078009967 | 0.27719684 |
| cellular response to angiotensin (GO:1904385) | 44628 | 0.078009967 | 0.27719684 |
| cellular response to UV-B (GO:0071493) | 44628 | 0.078009967 | 0.27719684 |
| dopamine uptake (GO:0090494) | 44628 | 0.078009967 | 0.27719684 |
| dynamin family protein polymerization involved in memb | 44628 | 0.078009967 | 0.27719684 |
| dynamin family protein polymerization involved in mitoc | 44628 | 0.078009967 | 0.27719684 |
| negative regulation of amyloid-beta clearance (GO:19002 | 44628 | 0.078009967 | 0.27719684 |
| negative regulation of CD4-positive, alpha-beta T cell pro | 44628 | 0.078009967 | 0.27719684 |
| negative regulation of glial cell apoptotic process (GO:00 | 44628 | 0.078009967 | 0.27719684 |
| negative regulation of monooxygenase activity (GO:0032 | 44628 | 0.078009967 | 0.27719684 |
| peptidyl-tyrosine dephosphorylation involved in inactivat | 44628 | 0.078009967 | 0.27719684 |
| positive regulation of dendritic cell cytokine production ( | 44628 | 0.078009967 | 0.27719684 |
| positive regulation of execution phase of apoptosis (GO:1 | 44628 | 0.078009967 | 0.27719684 |
| positive regulation of fibroblast migration (GO:0010763) | 44628 | 0.078009967 | 0.27719684 |
| positive regulation of homotypic cell-cell adhesion (GO:0 | 44628 | 0.078009967 | 0.27719684 |
| positive regulation of monocyte chemotactic protein-1 pr | 44628 | 0.078009967 | 0.27719684 |
| purine ribonucleoside monophosphate catabolic process | 44628 | 0.078009967 | 0.27719684 |
| regulation of CD8-positive, alpha-beta T cell proliferation | 44628 | 0.078009967 | 0.27719684 |
| regulation of chemokine (C-C motif) ligand 5 production ( | 44628 | 0.078009967 | 0.27719684 |
| regulation of lymphocyte apoptotic process (GO:007022{ | 44628 | 0.078009967 | 0.27719684 |
| regulation of nitric oxide metabolic process (GO:0080164 | 44628 | 0.078009967 | 0.27719684 |
| regulation of protein ADP-ribosylation (GO:0010835) | 44628 | 0.078009967 | 0.27719684 |
| regulation of RNA biosynthetic process (GO:2001141) | 44628 | 0.078009967 | 0.27719684 |

| | | | |
|---|---|---|---|
| RNA secondary structure unwinding (GO:0010501) | 44628 | 0.078009967 | 0.27719684 |
| stress-induced premature senescence (GO:0090400) | 44628 | 0.078009967 | 0.27719684 |
| translocation of molecules into host (GO:0044417) | 44628 | 0.078009967 | 0.27719684 |
| tyrosine phosphorylation of STAT protein (GO:0007260) | 44628 | 0.078009967 | 0.27719684 |
| vascular associated smooth muscle contraction (GO:0014 | 44628 | 0.078009967 | 0.27719684 |
| ventricular trabecula myocardium morphogenesis (GO:0( | 44628 | 0.078009967 | 0.27719684 |
| viral mRNA export from host cell nucleus (GO:0046784) | 44628 | 0.078009967 | 0.27719684 |
| wound healing, spreading of epidermal cells (GO:003531 | 44628 | 0.078009967 | 0.27719684 |
| cation transport (GO:0006812) | 30/174 | 0.078332699 | 0.278153365 |
| 3'-UTR-mediated mRNA stabilization (GO:0070935) | 44699 | 0.079585569 | 0.279356103 |
| amyloid precursor protein metabolic process (GO:00429 | 44699 | 0.079585569 | 0.279356103 |
| carbohydrate derivative transport (GO:1901264) | 44699 | 0.079585569 | 0.279356103 |
| intracellular estrogen receptor signaling pathway (GO:00 | 44699 | 0.079585569 | 0.279356103 |
| negative regulation of fibroblast proliferation (GO:00481 | 44699 | 0.079585569 | 0.279356103 |
| negative regulation of lipid catabolic process (GO:00509 | 44699 | 0.079585569 | 0.279356103 |
| negative regulation of Rho protein signal transduction (G | 44699 | 0.079585569 | 0.279356103 |
| positive regulation of cardiac muscle hypertrophy (GO:0( | 44699 | 0.079585569 | 0.279356103 |
| positive regulation of cell cycle phase transition (GO:190 | 44699 | 0.079585569 | 0.279356103 |
| positive regulation of CREB transcription factor activity ( | 44699 | 0.079585569 | 0.279356103 |
| positive regulation of glial cell differentiation (GO:00456 | 44699 | 0.079585569 | 0.279356103 |
| positive regulation of phosphoprotein phosphatase activi | 44699 | 0.079585569 | 0.279356103 |
| positive regulation of stress-activated protein kinase sign | 44699 | 0.079585569 | 0.279356103 |
| regulation of B cell differentiation (GO:0045577) | 44699 | 0.079585569 | 0.279356103 |
| regulation of production of miRNAs involved in gene siler | 44699 | 0.079585569 | 0.279356103 |
| regulation of protein-containing complex disassembly (G | 44699 | 0.079585569 | 0.279356103 |
| regulation of single stranded viral RNA replication via dou | 44699 | 0.079585569 | 0.279356103 |
| regulation of vesicle-mediated transport (GO:0060627) | 18/96 | 0.079817218 | 0.279980045 |
| negative regulation of epithelial to mesenchymal transiti | 44771 | 0.080264216 | 0.280789633 |
| negative regulation of transferase activity (GO:0051348) | 44771 | 0.080264216 | 0.280789633 |
| regulation of double-strand break repair via nonhomolog | 44771 | 0.080264216 | 0.280789633 |
| response to cadmium ion (GO:0046686) | 44771 | 0.080264216 | 0.280789633 |
| cholesterol homeostasis (GO:0042632) | 14/71 | 0.081301316 | 0.282409682 |
| positive regulation of cellular protein localization (GO:19 | 14/71 | 0.081301316 | 0.282409682 |
| protein complex oligomerization (GO:0051259) | 17/90 | 0.082217292 | 0.282409682 |
| AMP metabolic process (GO:0046033) | 44664 | 0.082412527 | 0.282409682 |
| antifungal innate immune response (GO:0061760) | 44664 | 0.082412527 | 0.282409682 |
| cellular biogenic amine metabolic process (GO:0006576) | 44664 | 0.082412527 | 0.282409682 |
| cellular potassium ion homeostasis (GO:0030007) | 44664 | 0.082412527 | 0.282409682 |
| intracellular sterol transport (GO:0032366) | 44664 | 0.082412527 | 0.282409682 |
| negative regulation by host of viral transcription (GO:004 | 44664 | 0.082412527 | 0.282409682 |
| negative regulation of alcohol biosynthetic process (GO:1 | 44664 | 0.082412527 | 0.282409682 |
| negative regulation of CD4-positive, alpha-beta T cell acti | 44664 | 0.082412527 | 0.282409682 |
| negative regulation of histone methylation (GO:0031061 | 44664 | 0.082412527 | 0.282409682 |
| negative regulation of hydrogen peroxide-induced cell de | 44664 | 0.082412527 | 0.282409682 |
| negative regulation of intrinsic apoptotic signaling pathw | 44664 | 0.082412527 | 0.282409682 |
| negative regulation of vascular endothelial growth factor | 44664 | 0.082412527 | 0.282409682 |
| positive regulation of cell aging (GO:0090343) | 44664 | 0.082412527 | 0.282409682 |

| | | | |
|---|---|---|---|
| positive regulation of fatty acid biosynthetic process (GO | 44664 | 0.082412527 | 0.282409682 |
| positive regulation of nuclear-transcribed mRNA poly(A) t | 44664 | 0.082412527 | 0.282409682 |
| positive regulation of protein exit from endoplasmic retic | 44664 | 0.082412527 | 0.282409682 |
| positive regulation of reactive oxygen species biosyntheti | 44664 | 0.082412527 | 0.282409682 |
| positive regulation of transporter activity (GO:0032411) | 44664 | 0.082412527 | 0.282409682 |
| purine-containing compound catabolic process (GO:0072 | 44664 | 0.082412527 | 0.282409682 |
| regulation of fibrinolysis (GO:0051917) | 44664 | 0.082412527 | 0.282409682 |
| regulation of leukocyte adhesion to vascular endothelial | 44664 | 0.082412527 | 0.282409682 |
| regulation of lysosomal lumen pH (GO:0035751) | 44664 | 0.082412527 | 0.282409682 |
| regulation of oxidoreductase activity (GO:0051341) | 44664 | 0.082412527 | 0.282409682 |
| regulation of platelet-derived growth factor receptor sigr | 44664 | 0.082412527 | 0.282409682 |
| regulation of protein exit from endoplasmic reticulum (G | 44664 | 0.082412527 | 0.282409682 |
| regulation of response to stress (GO:0080134) | 44664 | 0.082412527 | 0.282409682 |
| semaphorin-plexin signaling pathway involved in neuron | 44664 | 0.082412527 | 0.282409682 |
| synaptic vesicle localization (GO:0097479) | 44664 | 0.082412527 | 0.282409682 |
| negative regulation of cell cycle G1/S phase transition (G | 12997 | 0.083552973 | 0.285376524 |
| regulation of lipid biosynthetic process (GO:0046890) | 12997 | 0.083552973 | 0.285376524 |
| regulation of TORC1 signaling (GO:1903432) | 12997 | 0.083552973 | 0.285376524 |
| response to exogenous dsRNA (GO:0043330) | 12997 | 0.083552973 | 0.285376524 |
| semaphorin-plexin signaling pathway (GO:0071526) | 12997 | 0.083552973 | 0.285376524 |
| chromatin remodeling (GO:0006338) | 19/103 | 0.083654841 | 0.285536726 |
| phospholipid transport (GO:0015914) | 21885 | 0.084220432 | 0.287278491 |
| histone deacetylation (GO:0016575) | 17441 | 0.085536763 | 0.291194943 |
| regulation of protein localization to nucleus (GO:190018 | 17441 | 0.085536763 | 0.291194943 |
| regulation of wound healing (GO:0061041) | 17441 | 0.085536763 | 0.291194943 |
| histone monoubiquitination (GO:0010390) | 44736 | 0.088481719 | 0.299845095 |
| lipid translocation (GO:0034204) | 44736 | 0.088481719 | 0.299845095 |
| mammary gland development (GO:0030879) | 44736 | 0.088481719 | 0.299845095 |
| phospholipid catabolic process (GO:0009395) | 44736 | 0.088481719 | 0.299845095 |
| protein kinase B signaling (GO:0043491) | 44736 | 0.088481719 | 0.299845095 |
| regulation of cardiac muscle hypertrophy (GO:0010611) | 44736 | 0.088481719 | 0.299845095 |
| wound healing, spreading of cells (GO:0044319) | 44736 | 0.088481719 | 0.299845095 |
| sterol homeostasis (GO:0055092) | 14/72 | 0.089182701 | 0.301840602 |
| positive regulation of supramolecular fiber organization ( | 17/91 | 0.08918678 | 0.301840602 |
| plasma membrane bounded cell projection assembly (GC | 45/278 | 0.089817462 | 0.303777157 |
| artery morphogenesis (GO:0048844) | 44772 | 0.093260082 | 0.314397227 |
| negative regulation of cytoskeleton organization (GO:005 | 44772 | 0.093260082 | 0.314397227 |
| phosphatidylcholine acyl-chain remodeling (GO:0036151 | 44772 | 0.093260082 | 0.314397227 |
| positive regulation of phosphate metabolic process (GO:( | 44772 | 0.093260082 | 0.314397227 |
| regulation of natural killer cell mediated cytotoxicity (GO | 44772 | 0.093260082 | 0.314397227 |
| positive regulation of ubiquitin-dependent protein catabo | 15/79 | 0.094777436 | 0.319305304 |
| intrinsic apoptotic signaling pathway by p53 class mediat | 13363 | 0.095531196 | 0.320638839 |
| mitochondrion disassembly (GO:0061726) | 13363 | 0.095531196 | 0.320638839 |
| platelet aggregation (GO:0070527) | 13363 | 0.095531196 | 0.320638839 |
| positive regulation of substrate adhesion-dependent cell | 13363 | 0.095531196 | 0.320638839 |
| regulation of cardiac muscle contraction (GO:0055117) | 13363 | 0.095531196 | 0.320638839 |
| cellular response to alcohol (GO:0097306) | 17807 | 0.095825616 | 0.320638839 |

| | | | |
|---|---|---|---|
| nucleus organization (GO:0006997) | 17807 | 0.095825616 | 0.320638839 |
| cellular response to hypoxia (GO:0071456) | 23/131 | 0.095979966 | 0.320638839 |
| heterophilic cell-cell adhesion via plasma membrane cell | 15585 | 0.096204173 | 0.320638839 |
| potassium ion import across plasma membrane (GO:199( | 15585 | 0.096204173 | 0.320638839 |
| regulation of ubiquitin-dependent protein catabolic proc | 15585 | 0.096204173 | 0.320638839 |
| cardiac muscle cell contraction (GO:0086003) | 44700 | 0.096716277 | 0.320638839 |
| histone deubiquitination (GO:0016578) | 44700 | 0.096716277 | 0.320638839 |
| negative regulation of cytokine production involved in inf | 44700 | 0.096716277 | 0.320638839 |
| negative regulation of nervous system development (GO: | 44700 | 0.096716277 | 0.320638839 |
| negative regulation of T cell receptor signaling pathway (( | 44700 | 0.096716277 | 0.320638839 |
| neuromuscular process (GO:0050905) | 44700 | 0.096716277 | 0.320638839 |
| positive regulation of focal adhesion assembly (GO:0051{ | 44700 | 0.096716277 | 0.320638839 |
| positive regulation of triglyceride metabolic process (GO: | 44700 | 0.096716277 | 0.320638839 |
| Rac protein signal transduction (GO:0016601) | 44700 | 0.096716277 | 0.320638839 |
| regulation of bicellular tight junction assembly (GO:2000{ | 44700 | 0.096716277 | 0.320638839 |
| regulation of exosomal secretion (GO:1903541) | 44700 | 0.096716277 | 0.320638839 |
| regulation of oligodendrocyte differentiation (GO:004871 | 44700 | 0.096716277 | 0.320638839 |
| regulation of synaptic vesicle exocytosis (GO:2000300) | 44700 | 0.096716277 | 0.320638839 |
| regulation of vascular endothelial growth factor signaling | 44700 | 0.096716277 | 0.320638839 |
| positive regulation of synaptic transmission (GO:005080€ | 14/73 | 0.097526988 | 0.322914407 |
| ubiquitin-dependent ERAD pathway (GO:0030433) | 14/73 | 0.097526988 | 0.322914407 |
| organonitrogen compound biosynthetic process (GO:190 | 27/158 | 0.098803784 | 0.326933542 |
| positive regulation of proteasomal protein catabolic proc | 16/86 | 0.099767811 | 0.32991329 |
| peptidyl-threonine modification (GO:0018210) | 13/67 | 0.10018754 | 0.331090501 |
| phosphatidylinositol metabolic process (GO:0046488) | 20/112 | 0.100485657 | 0.331629784 |
| adenylate cyclase-modulating G protein-coupled recepto | 28/165 | 0.100604457 | 0.331629784 |
| mRNA-containing ribonucleoprotein complex export fron | 18/99 | 0.100606071 | 0.331629784 |
| organic anion transport (GO:0015711) | 18/99 | 0.100606071 | 0.331629784 |
| regulation of DNA-templated transcription, initiation (GO | 22616 | 0.102692514 | 0.335032088 |
| negative regulation of cell cycle (GO:0045786) | 15/80 | 0.103047174 | 0.335032088 |
| regulation of protein localization to plasma membrane (( | 15/80 | 0.103047174 | 0.335032088 |
| cellular response to low-density lipoprotein particle stimu | 44665 | 0.103743209 | 0.335032088 |
| dicarboxylic acid catabolic process (GO:0043649) | 44665 | 0.103743209 | 0.335032088 |
| heterochromatin organization (GO:0070828) | 44665 | 0.103743209 | 0.335032088 |
| positive regulation of brown fat cell differentiation (GO:0 | 44665 | 0.103743209 | 0.335032088 |
| positive regulation of cyclase activity (GO:0031281) | 44665 | 0.103743209 | 0.335032088 |
| positive regulation of sodium ion transmembrane transp( | 44665 | 0.103743209 | 0.335032088 |
| positive regulation of T cell chemotaxis (GO:0010820) | 44665 | 0.103743209 | 0.335032088 |
| positive regulation of T cell receptor signaling pathway (( | 44665 | 0.103743209 | 0.335032088 |
| positive regulation of transmembrane transport (GO:003 | 44665 | 0.103743209 | 0.335032088 |
| regulation of intrinsic apoptotic signaling pathway in resp | 44665 | 0.103743209 | 0.335032088 |
| regulation of retinoic acid receptor signaling pathway (G( | 44665 | 0.103743209 | 0.335032088 |
| renal absorption (GO:0070293) | 44665 | 0.103743209 | 0.335032088 |
| smooth muscle contraction (GO:0006939) | 44665 | 0.103743209 | 0.335032088 |
| endosome transport via multivesicular body sorting path | 44737 | 0.103974967 | 0.335032088 |
| glycolytic process through glucose-6-phosphate (GO:006: | 44737 | 0.103974967 | 0.335032088 |
| negative regulation of cellular carbohydrate metabolic pr | 44737 | 0.103974967 | 0.335032088 |

| | | | |
|---|---|---|---|
| negative regulation of endocytosis (GO:0045806) | 44737 | 0.103974967 | 0.335032088 |
| negative regulation of response to endoplasmic reticulun | 44737 | 0.103974967 | 0.335032088 |
| negative regulation of sequestering of calcium ion (GO:0( | 44737 | 0.103974967 | 0.335032088 |
| negative regulation of signaling (GO:0023057) | 44737 | 0.103974967 | 0.335032088 |
| organophosphate ester transport (GO:0015748) | 44737 | 0.103974967 | 0.335032088 |
| positive regulation of ERBB signaling pathway (GO:19011 | 44737 | 0.103974967 | 0.335032088 |
| positive regulation of peptidyl-threonine phosphorylatior | 44737 | 0.103974967 | 0.335032088 |
| regulation of cholesterol transport (GO:0032374) | 44737 | 0.103974967 | 0.335032088 |
| regulation of myotube differentiation (GO:0010830) | 44737 | 0.103974967 | 0.335032088 |
| regulation of epithelial cell proliferation (GO:0050678) | 17/93 | 0.104228744 | 0.335032088 |
| actin-mediated cell contraction (GO:0070252) | 44629 | 0.105830155 | 0.335032088 |
| bone resorption (GO:0045453) | 44629 | 0.105830155 | 0.335032088 |
| glucosamine-containing compound catabolic process (GC | 44629 | 0.105830155 | 0.335032088 |
| leukotriene D4 biosynthetic process (GO:1901750) | 44629 | 0.105830155 | 0.335032088 |
| leukotriene D4 metabolic process (GO:1901748) | 44629 | 0.105830155 | 0.335032088 |
| negative regulation of alpha-beta T cell activation (GO:00 | 44629 | 0.105830155 | 0.335032088 |
| negative regulation of bone resorption (GO:0045779) | 44629 | 0.105830155 | 0.335032088 |
| negative regulation of glial cell differentiation (GO:00456 | 44629 | 0.105830155 | 0.335032088 |
| negative regulation of protein activation cascade (GO:20( | 44629 | 0.105830155 | 0.335032088 |
| negative regulation of protein-containing complex disass( | 44629 | 0.105830155 | 0.335032088 |
| negative regulation of RNA catabolic process (GO:190236 | 44629 | 0.105830155 | 0.335032088 |
| neurotransmitter receptor transport to postsynaptic mer | 44629 | 0.105830155 | 0.335032088 |
| Notch receptor processing (GO:0007220) | 44629 | 0.105830155 | 0.335032088 |
| peptidyl-cysteine modification (GO:0018198) | 44629 | 0.105830155 | 0.335032088 |
| phosphatidylglycerol biosynthetic process (GO:0006655) | 44629 | 0.105830155 | 0.335032088 |
| positive regulation of B cell differentiation (GO:0045579) | 44629 | 0.105830155 | 0.335032088 |
| positive regulation of extracellular matrix assembly (GO:1 | 44629 | 0.105830155 | 0.335032088 |
| positive regulation of mitochondrial calcium ion concentr | 44629 | 0.105830155 | 0.335032088 |
| positive regulation of phagocytosis, engulfment (GO:006( | 44629 | 0.105830155 | 0.335032088 |
| positive regulation of protein kinase C signaling (GO:009( | 44629 | 0.105830155 | 0.335032088 |
| positive regulation of tumor necrosis factor-mediated sig | 44629 | 0.105830155 | 0.335032088 |
| postsynaptic membrane assembly (GO:0097104) | 44629 | 0.105830155 | 0.335032088 |
| quaternary ammonium group transport (GO:0015697) | 44629 | 0.105830155 | 0.335032088 |
| regulation of cellular extravasation (GO:0002691) | 44629 | 0.105830155 | 0.335032088 |
| regulation of DNA-templated transcription in response tc | 44629 | 0.105830155 | 0.335032088 |
| regulation of glucan biosynthetic process (GO:0010962) | 44629 | 0.105830155 | 0.335032088 |
| regulation of lipid transport (GO:0032368) | 44629 | 0.105830155 | 0.335032088 |
| regulation of protein deacetylation (GO:0090311) | 44629 | 0.105830155 | 0.335032088 |
| regulation of respiratory burst (GO:0060263) | 44629 | 0.105830155 | 0.335032088 |
| regulation of RNA polymerase II regulatory region sequer | 44629 | 0.105830155 | 0.335032088 |
| regulation of skeletal muscle cell differentiation (GO:2001 | 44629 | 0.105830155 | 0.335032088 |
| regulation of vesicle fusion (GO:0031338) | 44629 | 0.105830155 | 0.335032088 |
| response to leptin (GO:0044321) | 44629 | 0.105830155 | 0.335032088 |
| response to misfolded protein (GO:0051788) | 44629 | 0.105830155 | 0.335032088 |
| response to type I interferon (GO:0034340) | 44629 | 0.105830155 | 0.335032088 |
| semaphorin-plexin signaling pathway involved in axon gu | 44629 | 0.105830155 | 0.335032088 |
| circulatory system development (GO:0072359) | 22/126 | 0.10637321 | 0.336546179 |

| | | | |
|---|---|---|---|
| positive regulation of actin filament polymerization (GO:( | 18172 | 0.106811353 | 0.337726707 |
| aminoglycan catabolic process (GO:0006026) | 44773 | 0.107352498 | 0.338819092 |
| peptide cross-linking (GO:0018149) | 44773 | 0.107352498 | 0.338819092 |
| positive regulation of proteolysis involved in cellular prot | 44773 | 0.107352498 | 0.338819092 |
| regulation of cell-substrate adhesion (GO:0010810) | 15950 | 0.108079199 | 0.340905549 |
| negative regulation of endothelial cell proliferation (GO:( | 13728 | 0.108430678 | 0.340979038 |
| negative regulation of stress-activated MAPK cascade (G( | 13728 | 0.108430678 | 0.340979038 |
| phagosome maturation (GO:0090382) | 13728 | 0.108430678 | 0.340979038 |
| positive regulation of exocytosis (GO:0045921) | 13728 | 0.108430678 | 0.340979038 |
| regulation of vascular associated smooth muscle cell prol | 13728 | 0.108430678 | 0.340979038 |
| regulation of neurogenesis (GO:0050767) | 22981 | 0.11276335 | 0.354175092 |
| response to retinoic acid (GO:0032526) | 22981 | 0.11276335 | 0.354175092 |
| modification-dependent protein catabolic process (GO:0( | 33/201 | 0.113156563 | 0.355195376 |
| regulation of nucleic acid-templated transcription (GO:19 | 66/430 | 0.113345219 | 0.355572713 |
| cardiac epithelial to mesenchymal transition (GO:006031 | 44701 | 0.115539514 | 0.358559005 |
| cellular monovalent inorganic cation homeostasis (GO:00 | 44701 | 0.115539514 | 0.358559005 |
| cellular response to interferon-beta (GO:0035458) | 44701 | 0.115539514 | 0.358559005 |
| embryonic digestive tract development (GO:0048566) | 44701 | 0.115539514 | 0.358559005 |
| establishment of epithelial cell polarity (GO:0090162) | 44701 | 0.115539514 | 0.358559005 |
| maintenance of protein location in nucleus (GO:0051457 | 44701 | 0.115539514 | 0.358559005 |
| mesoderm development (GO:0007498) | 44701 | 0.115539514 | 0.358559005 |
| negative regulation of axon extension involved in axon gu | 44701 | 0.115539514 | 0.358559005 |
| negative regulation of dephosphorylation (GO:0035305) | 44701 | 0.115539514 | 0.358559005 |
| nucleobase-containing compound biosynthetic process (( | 44701 | 0.115539514 | 0.358559005 |
| organic cation transport (GO:0015695) | 44701 | 0.115539514 | 0.358559005 |
| positive regulation of cyclin-dependent protein kinase ac | 44701 | 0.115539514 | 0.358559005 |
| positive regulation of natural killer cell mediated cytotoxi | 44701 | 0.115539514 | 0.358559005 |
| positive regulation of nitrogen compound metabolic proc | 44701 | 0.115539514 | 0.358559005 |
| post-transcriptional gene silencing by RNA (GO:0035194) | 44701 | 0.115539514 | 0.358559005 |
| regulation of granulocyte chemotaxis (GO:0071622) | 44701 | 0.115539514 | 0.358559005 |
| regulation of hippo signaling (GO:0035330) | 44701 | 0.115539514 | 0.358559005 |
| regulation of lymphocyte differentiation (GO:0045619) | 44701 | 0.115539514 | 0.358559005 |
| autophagosome organization (GO:1905037) | 20760 | 0.115768041 | 0.359053715 |
| positive regulation of protein polymerization (GO:003227 | 13/69 | 0.119499037 | 0.370183362 |
| RNA 3'-end processing (GO:0031123) | 13/69 | 0.119499037 | 0.370183362 |
| intracellular steroid hormone receptor signaling pathway | 16316 | 0.120735655 | 0.371478362 |
| positive regulation of receptor-mediated endocytosis (G( | 16316 | 0.120735655 | 0.371478362 |
| response to amyloid-beta (GO:1904645) | 16316 | 0.120735655 | 0.371478362 |
| lamellipodium assembly (GO:0030032) | 44738 | 0.120775159 | 0.371478362 |
| mononuclear cell differentiation (GO:1903131) | 44738 | 0.120775159 | 0.371478362 |
| negative regulation of interleukin-1 beta production (GO: | 44738 | 0.120775159 | 0.371478362 |
| negative regulation of macroautophagy (GO:0016242) | 44738 | 0.120775159 | 0.371478362 |
| negative regulation of protein transport (GO:0051224) | 44738 | 0.120775159 | 0.371478362 |
| negative regulation of receptor-mediated endocytosis (G | 44738 | 0.120775159 | 0.371478362 |
| purine-containing compound biosynthetic process (GO:0( | 44738 | 0.120775159 | 0.371478362 |
| regulation of viral life cycle (GO:1903900) | 44738 | 0.120775159 | 0.371478362 |
| TOR signaling (GO:0031929) | 44738 | 0.120775159 | 0.371478362 |

| | | | |
|---|---|---|---|
| lysosome organization (GO:0007040) | 14093 | 0.122232129 | 0.375515025 |
| negative regulation of TOR signaling (GO:0032007) | 14093 | 0.122232129 | 0.375515025 |
| amyloid fibril formation (GO:1990000) | 23346 | 0.123384616 | 0.377515428 |
| positive regulation of cell growth (GO:0030307) | 18/102 | 0.124495244 | 0.377515428 |
| regulation of proteasomal ubiquitin-dependent protein c | 18/102 | 0.124495244 | 0.377515428 |
| regulation of endothelial cell migration (GO:0010594) | 16/89 | 0.125483771 | 0.377515428 |
| regulation of mRNA catabolic process (GO:0061013) | 21/122 | 0.125549407 | 0.377515428 |
| external encapsulating structure organization (GO:00452 | 35/217 | 0.126349171 | 0.377515428 |
| positive regulation of gene expression, epigenetic (GO:00 | 21125 | 0.127196699 | 0.377515428 |
| cellular sodium ion homeostasis (GO:0006883) | 44666 | 0.127293066 | 0.377515428 |
| engulfment of apoptotic cell (GO:0043652) | 44666 | 0.127293066 | 0.377515428 |
| establishment of apical/basal cell polarity (GO:0035089) | 44666 | 0.127293066 | 0.377515428 |
| ganglioside biosynthetic process (GO:0001574) | 44666 | 0.127293066 | 0.377515428 |
| histone H3 deacetylation (GO:0070932) | 44666 | 0.127293066 | 0.377515428 |
| histone mRNA catabolic process (GO:0071044) | 44666 | 0.127293066 | 0.377515428 |
| macrophage migration (GO:1905517) | 44666 | 0.127293066 | 0.377515428 |
| modulation by host of viral genome replication (GO:0044 | 44666 | 0.127293066 | 0.377515428 |
| negative regulation of chondrocyte differentiation (GO:0( | 44666 | 0.127293066 | 0.377515428 |
| negative regulation of single stranded viral RNA replicatic | 44666 | 0.127293066 | 0.377515428 |
| peptidyl-arginine modification (GO:0018195) | 44666 | 0.127293066 | 0.377515428 |
| positive regulation of chromatin organization (GO:19052( | 44666 | 0.127293066 | 0.377515428 |
| positive regulation of DNA damage response, signal trans | 44666 | 0.127293066 | 0.377515428 |
| positive regulation of endothelial cell chemotaxis (GO:20( | 44666 | 0.127293066 | 0.377515428 |
| regulation of extracellular matrix organization (GO:19030 | 44666 | 0.127293066 | 0.377515428 |
| regulation of microglial cell activation (GO:1903978) | 44666 | 0.127293066 | 0.377515428 |
| response to muscle stretch (GO:0035994) | 44666 | 0.127293066 | 0.377515428 |
| superoxide anion generation (GO:0042554) | 44666 | 0.127293066 | 0.377515428 |
| proteolysis (GO:0006508) | 45/287 | 0.13083209 | 0.377515428 |
| cellular response to organonitrogen compound (GO:0071 | 18/103 | 0.133143802 | 0.377515428 |
| regulation of anatomical structure morphogenesis (GO:0( | 21/123 | 0.133451625 | 0.377515428 |
| AMP biosynthetic process (GO:0006167) | 44597 | 0.13373725 | 0.377515428 |
| anion homeostasis (GO:0055081) | 44597 | 0.13373725 | 0.377515428 |
| cardiac muscle cell myoblast differentiation (GO:006037$ | 44597 | 0.13373725 | 0.377515428 |
| CD4-positive, alpha-beta T cell activation (GO:0035710) | 44597 | 0.13373725 | 0.377515428 |
| chromatin maintenance (GO:0070827) | 44597 | 0.13373725 | 0.377515428 |
| ciliary neurotrophic factor-mediated signaling pathway (( | 44597 | 0.13373725 | 0.377515428 |
| coronary artery morphogenesis (GO:0060982) | 44597 | 0.13373725 | 0.377515428 |
| DNA ligation involved in DNA repair (GO:0051103) | 44597 | 0.13373725 | 0.377515428 |
| dopamine catabolic process (GO:0042420) | 44597 | 0.13373725 | 0.377515428 |
| endonucleolytic cleavage in 5'-ETS of tricistronic rRNA tra | 44597 | 0.13373725 | 0.377515428 |
| endonucleolytic cleavage to generate mature 5'-end of S! | 44597 | 0.13373725 | 0.377515428 |
| fatty acid omega-oxidation (GO:0010430) | 44597 | 0.13373725 | 0.377515428 |
| fructose 2,6-bisphosphate metabolic process (GO:00060( | 44597 | 0.13373725 | 0.377515428 |
| glomerular visceral epithelial cell migration (GO:0090521 | 44597 | 0.13373725 | 0.377515428 |
| glutathione transport (GO:0034635) | 44597 | 0.13373725 | 0.377515428 |
| His-Purkinje system development (GO:0003164) | 44597 | 0.13373725 | 0.377515428 |
| histone citrullination (GO:0036414) | 44597 | 0.13373725 | 0.377515428 |

| | | | |
|---|---|---|---|
| histone H3-K27 demethylation (GO:0071557) | 44597 | 0.13373725 | 0.377515428 |
| histone H4-K20 methylation (GO:0034770) | 44597 | 0.13373725 | 0.377515428 |
| histone H4-K20 trimethylation (GO:0034773) | 44597 | 0.13373725 | 0.377515428 |
| immature B cell differentiation (GO:0002327) | 44597 | 0.13373725 | 0.377515428 |
| intracellular sequestering of iron ion (GO:0006880) | 44597 | 0.13373725 | 0.377515428 |
| kidney morphogenesis (GO:0060993) | 44597 | 0.13373725 | 0.377515428 |
| L-aspartate transmembrane transport (GO:0070778) | 44597 | 0.13373725 | 0.377515428 |
| lymph node development (GO:0048535) | 44597 | 0.13373725 | 0.377515428 |
| lysosomal protein catabolic process (GO:1905146) | 44597 | 0.13373725 | 0.377515428 |
| meiotic sister chromatid segregation (GO:0045144) | 44597 | 0.13373725 | 0.377515428 |
| microtubule nucleation by microtubule organizing center | 44597 | 0.13373725 | 0.377515428 |
| mRNA cleavage involved in gene silencing by miRNA (GO: | 44597 | 0.13373725 | 0.377515428 |
| negative regulation of blood coagulation, intrinsic pathwa | 44597 | 0.13373725 | 0.377515428 |
| negative regulation of calcium ion-dependent exocytosis | 44597 | 0.13373725 | 0.377515428 |
| negative regulation of cysteine-type endopeptidase activ | 44597 | 0.13373725 | 0.377515428 |
| negative regulation of histone H3-K27 methylation (GO:0 | 44597 | 0.13373725 | 0.377515428 |
| negative regulation of histone H4 acetylation (GO:00902 | 44597 | 0.13373725 | 0.377515428 |
| negative regulation of hydrogen peroxide-induced neuro | 44597 | 0.13373725 | 0.377515428 |
| negative regulation of immunoglobulin production (GO:0 | 44597 | 0.13373725 | 0.377515428 |
| negative regulation of mast cell activation involved in imı | 44597 | 0.13373725 | 0.377515428 |
| negative regulation of organ growth (GO:0046621) | 44597 | 0.13373725 | 0.377515428 |
| negative regulation of RNA polymerase II regulatory regic | 44597 | 0.13373725 | 0.377515428 |
| negative regulation of T cell migration (GO:2000405) | 44597 | 0.13373725 | 0.377515428 |
| negative regulation of vascular endothelial cell proliferati | 44597 | 0.13373725 | 0.377515428 |
| neuroinflammatory response (GO:0150076) | 44597 | 0.13373725 | 0.377515428 |
| ornithine transport (GO:0015822) | 44597 | 0.13373725 | 0.377515428 |
| oxidative RNA demethylation (GO:0035513) | 44597 | 0.13373725 | 0.377515428 |
| positive regulation of axon extension involved in axon gu | 44597 | 0.13373725 | 0.377515428 |
| positive regulation of CD4-positive, CD25-positive, alpha- | 44597 | 0.13373725 | 0.377515428 |
| positive regulation of cell proliferation involved in heart r | 44597 | 0.13373725 | 0.377515428 |
| positive regulation of DNA demethylation (GO:1901537) | 44597 | 0.13373725 | 0.377515428 |
| positive regulation of endothelial cell chemotaxis by VEG | 44597 | 0.13373725 | 0.377515428 |
| positive regulation of histone H3-K27 methylation (GO:0( | 44597 | 0.13373725 | 0.377515428 |
| positive regulation of mast cell activation involved in imn | 44597 | 0.13373725 | 0.377515428 |
| positive regulation of mast cell chemotaxis (GO:0060754) | 44597 | 0.13373725 | 0.377515428 |
| positive regulation of metallopeptidase activity (GO:1905 | 44597 | 0.13373725 | 0.377515428 |
| positive regulation of natural killer cell chemotaxis (GO:2 | 44597 | 0.13373725 | 0.377515428 |
| positive regulation of necrotic cell death (GO:0010940) | 44597 | 0.13373725 | 0.377515428 |
| positive regulation of nucleotide-binding oligomerization | 44597 | 0.13373725 | 0.377515428 |
| positive regulation of protein catabolic process in the vac | 44597 | 0.13373725 | 0.377515428 |
| positive regulation of toll-like receptor 9 signaling pathwa | 44597 | 0.13373725 | 0.377515428 |
| positive regulation of vascular associated smooth muscle | 44597 | 0.13373725 | 0.377515428 |
| prostaglandin transport (GO:0015732) | 44597 | 0.13373725 | 0.377515428 |
| protein citrullination (GO:0018101) | 44597 | 0.13373725 | 0.377515428 |
| protein localization to membrane raft (GO:1903044) | 44597 | 0.13373725 | 0.377515428 |
| pyridine-containing compound metabolic process (GO:00 | 44597 | 0.13373725 | 0.377515428 |
| quinone catabolic process (GO:1901662) | 44597 | 0.13373725 | 0.377515428 |

| | | | |
|---|---|---|---|
| receptor-mediated virion attachment to host cell (GO:00⁴ | 44597 | 0.13373725 | 0.377515428 |
| regulation of antifungal innate immune response (GO:19( | 44597 | 0.13373725 | 0.377515428 |
| regulation of astrocyte activation (GO:0061888) | 44597 | 0.13373725 | 0.377515428 |
| regulation of blood coagulation, intrinsic pathway (GO:2( | 44597 | 0.13373725 | 0.377515428 |
| regulation of caveolin-mediated endocytosis (GO:200128 | 44597 | 0.13373725 | 0.377515428 |
| regulation of cell maturation (GO:1903429) | 44597 | 0.13373725 | 0.377515428 |
| regulation of cellular response to osmotic stress (GO:010 | 44597 | 0.13373725 | 0.377515428 |
| regulation of cyclase activity (GO:0031279) | 44597 | 0.13373725 | 0.377515428 |
| regulation of germinal center formation (GO:0002634) | 44597 | 0.13373725 | 0.377515428 |
| regulation of guanyl-nucleotide exchange factor activity ( | 44597 | 0.13373725 | 0.377515428 |
| regulation of histone H3-K9 trimethylation (GO:1900112) | 44597 | 0.13373725 | 0.377515428 |
| regulation of histone H4-K16 acetylation (GO:2000618) | 44597 | 0.13373725 | 0.377515428 |
| regulation of hormone metabolic process (GO:0032350) | 44597 | 0.13373725 | 0.377515428 |
| regulation of hydrogen peroxide biosynthetic process (G( | 44597 | 0.13373725 | 0.377515428 |
| regulation of inositol trisphosphate biosynthetic process | 44597 | 0.13373725 | 0.377515428 |
| regulation of microvillus assembly (GO:0032534) | 44597 | 0.13373725 | 0.377515428 |
| regulation of natural killer cell mediated immunity (GO:0( | 44597 | 0.13373725 | 0.377515428 |
| regulation of neutrophil activation (GO:1902563) | 44597 | 0.13373725 | 0.377515428 |
| regulation of phosphatidylinositol dephosphorylation (G( | 44597 | 0.13373725 | 0.377515428 |
| regulation of respiratory burst involved in inflammatory r | 44597 | 0.13373725 | 0.377515428 |
| regulation of synaptic vesicle priming (GO:0010807) | 44597 | 0.13373725 | 0.377515428 |
| regulation of synaptic vesicle recycling (GO:1903421) | 44597 | 0.13373725 | 0.377515428 |
| regulation of systemic arterial blood pressure by endothe | 44597 | 0.13373725 | 0.377515428 |
| regulation of vascular associated smooth muscle contract | 44597 | 0.13373725 | 0.377515428 |
| regulation of vitamin D receptor signaling pathway (GO:0 | 44597 | 0.13373725 | 0.377515428 |
| response to fructose (GO:0009750) | 44597 | 0.13373725 | 0.377515428 |
| response to salt stress (GO:0009651) | 44597 | 0.13373725 | 0.377515428 |
| ribose phosphate metabolic process (GO:0019693) | 44597 | 0.13373725 | 0.377515428 |
| RNA phosphodiester bond hydrolysis, exonucleolytic (GO | 44597 | 0.13373725 | 0.377515428 |
| rRNA 5'-end processing (GO:0000967) | 44597 | 0.13373725 | 0.377515428 |
| Schwann cell differentiation (GO:0014037) | 44597 | 0.13373725 | 0.377515428 |
| serotonin transport (GO:0006837) | 44597 | 0.13373725 | 0.377515428 |
| serotonin uptake (GO:0051610) | 44597 | 0.13373725 | 0.377515428 |
| sulfide oxidation (GO:0019418) | 44597 | 0.13373725 | 0.377515428 |
| sulfide oxidation, using sulfide:quinone oxidoreductase (( | 44597 | 0.13373725 | 0.377515428 |
| T cell extravasation (GO:0072683) | 44597 | 0.13373725 | 0.377515428 |
| negative regulation of Wnt signaling pathway (GO:00301 | 31/191 | 0.135101031 | 0.377515428 |
| positive regulation of endothelial cell proliferation (GO:0( | 14/77 | 0.135513727 | 0.377515428 |
| beta-catenin destruction complex disassembly (GO:1904{ | 44702 | 0.1359502 | 0.377515428 |
| calcium-mediated signaling using intracellular calcium so | 44702 | 0.1359502 | 0.377515428 |
| cellular response to steroid hormone stimulus (GO:0071 | 44702 | 0.1359502 | 0.377515428 |
| focal adhesion assembly (GO:0048041) | 44702 | 0.1359502 | 0.377515428 |
| histone ubiquitination (GO:0016574) | 44702 | 0.1359502 | 0.377515428 |
| negative regulation of cell communication (GO:0010648) | 44702 | 0.1359502 | 0.377515428 |
| negative regulation of reproductive process (GO:200024 | 44702 | 0.1359502 | 0.377515428 |
| positive regulation of vascular associated smooth muscle | 44702 | 0.1359502 | 0.377515428 |
| regulation of lipid catabolic process (GO:0050994) | 44702 | 0.1359502 | 0.377515428 |

| | | | |
|---|---|---|---|
| regulation of lipopolysaccharide-mediated signaling path | 44702 | 0.1359502 | 0.377515428 |
| sterol transport (GO:0015918) | 44702 | 0.1359502 | 0.377515428 |
| vacuolar acidification (GO:0007035) | 44702 | 0.1359502 | 0.377515428 |
| adherens junction assembly (GO:0034333) | 44630 | 0.136857477 | 0.377515428 |
| arginine metabolic process (GO:0006525) | 44630 | 0.136857477 | 0.377515428 |
| aromatic amino acid family metabolic process (GO:0009C | 44630 | 0.136857477 | 0.377515428 |
| cAMP biosynthetic process (GO:0006171) | 44630 | 0.136857477 | 0.377515428 |
| cellular response to dopamine (GO:1903351) | 44630 | 0.136857477 | 0.377515428 |
| cellular response to leucine (GO:0071233) | 44630 | 0.136857477 | 0.377515428 |
| cellular response to oxidised low-density lipoprotein part | 44630 | 0.136857477 | 0.377515428 |
| cellular response to purine-containing compound (GO:00 | 44630 | 0.136857477 | 0.377515428 |
| dicarboxylic acid biosynthetic process (GO:0043650) | 44630 | 0.136857477 | 0.377515428 |
| endothelial tube morphogenesis (GO:0061154) | 44630 | 0.136857477 | 0.377515428 |
| ESCRT complex disassembly (GO:1904896) | 44630 | 0.136857477 | 0.377515428 |
| ESCRT III complex disassembly (GO:1904903) | 44630 | 0.136857477 | 0.377515428 |
| granulocyte migration (GO:0097530) | 44630 | 0.136857477 | 0.377515428 |
| histone H2B ubiquitination (GO:0033523) | 44630 | 0.136857477 | 0.377515428 |
| histone H3-K36 demethylation (GO:0070544) | 44630 | 0.136857477 | 0.377515428 |
| ionotropic glutamate receptor signaling pathway (GO:003 | 44630 | 0.136857477 | 0.377515428 |
| leptin-mediated signaling pathway (GO:0033210) | 44630 | 0.136857477 | 0.377515428 |
| modulation by symbiont of host process (GO:0044003) | 44630 | 0.136857477 | 0.377515428 |
| monocyte activation (GO:0042117) | 44630 | 0.136857477 | 0.377515428 |
| negative regulation of alpha-beta T cell proliferation (GO: | 44630 | 0.136857477 | 0.377515428 |
| negative regulation of extracellular matrix organization (( | 44630 | 0.136857477 | 0.377515428 |
| negative regulation of humoral immune response mediat | 44630 | 0.136857477 | 0.377515428 |
| negative regulation of protein autophosphorylation (GO:( | 44630 | 0.136857477 | 0.377515428 |
| negative regulation of protein exit from endoplasmic reti | 44630 | 0.136857477 | 0.377515428 |
| negative regulation of receptor binding (GO:1900121) | 44630 | 0.136857477 | 0.377515428 |
| negative regulation of toll-like receptor 4 signaling pathw | 44630 | 0.136857477 | 0.377515428 |
| neuron remodeling (GO:0016322) | 44630 | 0.136857477 | 0.377515428 |
| positive regulation by symbiont of entry into host (GO:00 | 44630 | 0.136857477 | 0.377515428 |
| positive regulation of keratinocyte migration (GO:005154 | 44630 | 0.136857477 | 0.377515428 |
| positive regulation of monocyte differentiation (GO:0045 | 44630 | 0.136857477 | 0.377515428 |
| positive regulation of skeletal muscle tissue development | 44630 | 0.136857477 | 0.377515428 |
| positive regulation of T cell apoptotic process (GO:00702 | 44630 | 0.136857477 | 0.377515428 |
| positive regulation of viral entry into host cell (GO:00465 | 44630 | 0.136857477 | 0.377515428 |
| protein K11-linked deubiquitination (GO:0035871) | 44630 | 0.136857477 | 0.377515428 |
| regulation of actin filament-based movement (GO:19031 | 44630 | 0.136857477 | 0.377515428 |
| regulation of cell activation (GO:0050865) | 44630 | 0.136857477 | 0.377515428 |
| regulation of epithelial cell apoptotic process (GO:19040: | 44630 | 0.136857477 | 0.377515428 |
| regulation of high voltage-gated calcium channel activity | 44630 | 0.136857477 | 0.377515428 |
| regulation of leukocyte tethering or rolling (GO:1903236) | 44630 | 0.136857477 | 0.377515428 |
| regulation of mast cell activation (GO:0033003) | 44630 | 0.136857477 | 0.377515428 |
| regulation of organ growth (GO:0046620) | 44630 | 0.136857477 | 0.377515428 |
| regulation of peptidyl-lysine acetylation (GO:2000756) | 44630 | 0.136857477 | 0.377515428 |
| regulation of phagocytosis, engulfment (GO:0060099) | 44630 | 0.136857477 | 0.377515428 |
| regulation of phospholipid metabolic process (GO:19037: | 44630 | 0.136857477 | 0.377515428 |

| | | | |
|---|---|---|---|
| response to dopamine (GO:1903350) | 44630 | 0.136857477 | 0.377515428 |
| response to leucine (GO:0043201) | 44630 | 0.136857477 | 0.377515428 |
| retinoic acid receptor signaling pathway (GO:0048384) | 44630 | 0.136857477 | 0.377515428 |
| sarcoplasmic reticulum calcium ion transport (GO:00702( | 44630 | 0.136857477 | 0.377515428 |
| secretory granule organization (GO:0033363) | 44630 | 0.136857477 | 0.377515428 |
| toxin transport (GO:1901998) | 44630 | 0.136857477 | 0.377515428 |
| regulation of receptor-mediated endocytosis (GO:004825 | 14458 | 0.136908386 | 0.377515428 |
| apoptotic mitochondrial changes (GO:0008637) | 12236 | 0.138696674 | 0.380160367 |
| negative chemotaxis (GO:0050919) | 12236 | 0.138696674 | 0.380160367 |
| negative regulation of JNK cascade (GO:0046329) | 12236 | 0.138696674 | 0.380160367 |
| positive regulation of cholesterol transport (GO:0032376 | 12236 | 0.138696674 | 0.380160367 |
| positive regulation of organelle organization (GO:001063 | 12236 | 0.138696674 | 0.380160367 |
| regulation of neurotransmitter secretion (GO:0046928) | 12236 | 0.138696674 | 0.380160367 |
| regulation of protein localization to cell surface (GO:2000 | 12236 | 0.138696674 | 0.380160367 |
| cellular response to cadmium ion (GO:0071276) | 44739 | 0.138818906 | 0.380160367 |
| negative regulation of cation channel activity (GO:20012! | 44739 | 0.138818906 | 0.380160367 |
| negative regulation of protein polymerization (GO:00322 | 44739 | 0.138818906 | 0.380160367 |
| positive regulation of cellular component movement (GC | 44739 | 0.138818906 | 0.380160367 |
| positive regulation of striated muscle cell differentiation | 44739 | 0.138818906 | 0.380160367 |
| regulation of blood coagulation (GO:0030193) | 44739 | 0.138818906 | 0.380160367 |
| positive regulation of cell projection organization (GO:00 | 20/117 | 0.139139186 | 0.380836707 |
| regulation of mitotic cell cycle (GO:0007346) | 29/178 | 0.139668664 | 0.38208463 |
| response to insulin (GO:0032868) | 15/84 | 0.140365474 | 0.383788757 |
| negative regulation of protein-containing complex assem | 19268 | 0.143848361 | 0.392511969 |
| positive regulation of protein localization to cell peripher | 19268 | 0.143848361 | 0.392511969 |
| protein homotetramerization (GO:0051289) | 19268 | 0.143848361 | 0.392511969 |
| cellular response to metal ion (GO:0071248) | 22/131 | 0.143858092 | 0.392511969 |
| protein autoubiquitination (GO:0051865) | 24077 | 0.146242713 | 0.398808868 |
| RNA processing (GO:0006396) | 29/179 | 0.146605573 | 0.399588643 |
| regulation of cellular response to stress (GO:0080135) | 20/118 | 0.147775071 | 0.40256502 |
| activation of GTPase activity (GO:0090630) | 18/105 | 0.151449873 | 0.411583216 |
| autophagy of mitochondrion (GO:0000422) | 14824 | 0.152424941 | 0.411583216 |
| negative regulation of protein kinase B signaling (GO:005 | 14824 | 0.152424941 | 0.411583216 |
| positive regulation of cation channel activity (GO:200125 | 14824 | 0.152424941 | 0.411583216 |
| response to organonitrogen compound (GO:0010243) | 14824 | 0.152424941 | 0.411583216 |
| digestive tract morphogenesis (GO:0048546) | 44667 | 0.152828494 | 0.411583216 |
| eosinophil chemotaxis (GO:0048245) | 44667 | 0.152828494 | 0.411583216 |
| eosinophil migration (GO:0072677) | 44667 | 0.152828494 | 0.411583216 |
| glutamine family amino acid catabolic process (GO:0009( | 44667 | 0.152828494 | 0.411583216 |
| negative regulation of immune effector process (GO:000: | 44667 | 0.152828494 | 0.411583216 |
| negative regulation of transcription regulatory region DN | 44667 | 0.152828494 | 0.411583216 |
| nucleotide catabolic process (GO:0009166) | 44667 | 0.152828494 | 0.411583216 |
| polyol biosynthetic process (GO:0046173) | 44667 | 0.152828494 | 0.411583216 |
| positive regulation of immunoglobulin mediated immune | 44667 | 0.152828494 | 0.411583216 |
| positive regulation of isotype switching (GO:0045830) | 44667 | 0.152828494 | 0.411583216 |
| regulation of blood vessel endothelial cell proliferation in | 44667 | 0.152828494 | 0.411583216 |
| regulation of ceramide biosynthetic process (GO:200030: | 44667 | 0.152828494 | 0.411583216 |

| | | | |
|---|---|---|---|
| regulation of monocyte differentiation (GO:0045655) | 44667 | 0.152828494 | 0.411583216 |
| regulation of oxidative phosphorylation (GO:0002082) | 44667 | 0.152828494 | 0.411583216 |
| regulation of rRNA processing (GO:2000232) | 44667 | 0.152828494 | 0.411583216 |
| sphingolipid catabolic process (GO:0030149) | 44667 | 0.152828494 | 0.411583216 |
| T cell mediated immunity (GO:0002456) | 44667 | 0.152828494 | 0.411583216 |
| T cell activation (GO:0042110) | 16/92 | 0.154694461 | 0.416176967 |
| Wnt signaling pathway (GO:0016055) | 16/92 | 0.154694461 | 0.416176967 |
| regulation of myoblast differentiation (GO:0045661) | 12601 | 0.155856962 | 0.419087431 |
| mRNA 3'-end processing (GO:0031124) | 14/79 | 0.157203195 | 0.421436928 |
| cell projection assembly (GO:0030031) | 44703 | 0.157821668 | 0.421436928 |
| positive regulation of peptidase activity (GO:0010952) | 44703 | 0.157821668 | 0.421436928 |
| proteasomal ubiquitin-independent protein catabolic pro | 44703 | 0.157821668 | 0.421436928 |
| regulation of endoplasmic reticulum stress-induced intrir | 44703 | 0.157821668 | 0.421436928 |
| regulation of vacuole organization (GO:0044088) | 44703 | 0.157821668 | 0.421436928 |
| regulation of voltage-gated calcium channel activity (GO: | 44703 | 0.157821668 | 0.421436928 |
| cellular response to acid chemical (GO:0071229) | 44740 | 0.158028708 | 0.421436928 |
| cellular response to epidermal growth factor stimulus (G( | 44740 | 0.158028708 | 0.421436928 |
| internal peptidyl-lysine acetylation (GO:0018393) | 44740 | 0.158028708 | 0.421436928 |
| L-amino acid transport (GO:0015807) | 44740 | 0.158028708 | 0.421436928 |
| negative regulation of calcium ion transmembrane transp | 44740 | 0.158028708 | 0.421436928 |
| neutral amino acid transport (GO:0015804) | 44740 | 0.158028708 | 0.421436928 |
| positive regulation of cytosolic calcium ion concentration | 44740 | 0.158028708 | 0.421436928 |
| regulation of insulin secretion involved in cellular respons | 44740 | 0.158028708 | 0.421436928 |
| regulation of neuron migration (GO:2001222) | 44740 | 0.158028708 | 0.421436928 |
| cellular response to nutrient levels (GO:0031669) | 24442 | 0.158454663 | 0.422139473 |
| regulation of protein localization (GO:0032880) | 24442 | 0.158454663 | 0.422139473 |
| phosphatidylinositol biosynthetic process (GO:0006661) | 21/126 | 0.158854484 | 0.422987721 |
| RNA export from nucleus (GO:0006405) | 18/106 | 0.161097131 | 0.428739546 |
| epithelial to mesenchymal transition (GO:0001837) | 17411 | 0.163181867 | 0.434065439 |
| extracellular structure organization (GO:0043062) | 34/216 | 0.163653216 | 0.434945165 |
| positive regulation of metabolic process (GO:0009893) | 19/113 | 0.163680038 | 0.434945165 |
| positive regulation of cell differentiation (GO:0045597) | 40/258 | 0.164412546 | 0.436668292 |
| cytoskeleton organization (GO:0007010) | 20/120 | 0.165924648 | 0.440459145 |
| regulation of fat cell differentiation (GO:0045598) | 14/80 | 0.168690837 | 0.444429687 |
| epithelial cell migration (GO:0010631) | 15189 | 0.168740554 | 0.444429687 |
| regulation of release of cytochrome c from mitochondria | 15189 | 0.168740554 | 0.444429687 |
| cellular glucan metabolic process (GO:0006073) | 44631 | 0.170500167 | 0.444429687 |
| chromatin-mediated maintenance of transcription (GO:0( | 44631 | 0.170500167 | 0.444429687 |
| complement receptor mediated signaling pathway (GO:0 | 44631 | 0.170500167 | 0.444429687 |
| desensitization of G protein-coupled receptor signaling p. | 44631 | 0.170500167 | 0.444429687 |
| establishment of epithelial cell apical/basal polarity (GO:( | 44631 | 0.170500167 | 0.444429687 |
| glutamine family amino acid biosynthetic process (GO:00 | 44631 | 0.170500167 | 0.444429687 |
| hematopoietic stem cell proliferation (GO:0071425) | 44631 | 0.170500167 | 0.444429687 |
| humoral immune response mediated by circulating immu | 44631 | 0.170500167 | 0.444429687 |
| inositol phosphate dephosphorylation (GO:0046855) | 44631 | 0.170500167 | 0.444429687 |
| isotype switching (GO:0045190) | 44631 | 0.170500167 | 0.444429687 |
| lipoprotein biosynthetic process (GO:0042158) | 44631 | 0.170500167 | 0.444429687 |

| | | | |
|---|---|---|---|
| negative regulation of activated T cell proliferation (GO:0 | 44631 | 0.170500167 | 0.444429687 |
| negative regulation of bone remodeling (GO:0046851) | 44631 | 0.170500167 | 0.444429687 |
| negative regulation of receptor signaling pathway via STA | 44631 | 0.170500167 | 0.444429687 |
| negative regulation of response to cytokine stimulus (GO | 44631 | 0.170500167 | 0.444429687 |
| negative regulation of steroid biosynthetic process (GO:0 | 44631 | 0.170500167 | 0.444429687 |
| Notch signaling involved in heart development (GO:0061 | 44631 | 0.170500167 | 0.444429687 |
| organophosphate catabolic process (GO:0046434) | 44631 | 0.170500167 | 0.444429687 |
| phosphorylated carbohydrate dephosphorylation (GO:00 | 44631 | 0.170500167 | 0.444429687 |
| positive regulation of alcohol biosynthetic process (GO:1 | 44631 | 0.170500167 | 0.444429687 |
| positive regulation of cellular extravasation (GO:0002693 | 44631 | 0.170500167 | 0.444429687 |
| positive regulation of interleukin-13 production (GO:003 | 44631 | 0.170500167 | 0.444429687 |
| positive regulation of myelination (GO:0031643) | 44631 | 0.170500167 | 0.444429687 |
| positive regulation of posttranscriptional gene silencing ( | 44631 | 0.170500167 | 0.444429687 |
| positive regulation of production of miRNAs involved in g | 44631 | 0.170500167 | 0.444429687 |
| positive regulation of transforming growth factor beta pr | 44631 | 0.170500167 | 0.444429687 |
| postsynapse assembly (GO:0099068) | 44631 | 0.170500167 | 0.444429687 |
| regulation of cytoplasmic transport (GO:1903649) | 44631 | 0.170500167 | 0.444429687 |
| regulation of ERAD pathway (GO:1904292) | 44631 | 0.170500167 | 0.444429687 |
| regulation of gene expression by genetic imprinting (GO: | 44631 | 0.170500167 | 0.444429687 |
| regulation of monocyte chemotactic protein-1 productio | 44631 | 0.170500167 | 0.444429687 |
| regulation of stress-activated protein kinase signaling cas | 44631 | 0.170500167 | 0.444429687 |
| vasoconstriction (GO:0042310) | 44631 | 0.170500167 | 0.444429687 |
| mRNA export from nucleus (GO:0006406) | 18/107 | 0.171065251 | 0.445679027 |
| regulation of gene silencing by miRNA (GO:0060964) | 24807 | 0.171167491 | 0.445721863 |
| regulation of focal adhesion assembly (GO:0051893) | 19998 | 0.171760776 | 0.446595211 |
| regulation of megakaryocyte differentiation (GO:004565 | 19998 | 0.171760776 | 0.446595211 |
| regulation of proteolysis (GO:0030162) | 19998 | 0.171760776 | 0.446595211 |
| intracellular pH reduction (GO:0051452) | 12966 | 0.173932962 | 0.451114198 |
| negative regulation of neurogenesis (GO:0050768) | 12966 | 0.173932962 | 0.451114198 |
| negative regulation of T cell proliferation (GO:0042130) | 12966 | 0.173932962 | 0.451114198 |
| regulation of phosphatidylinositol 3-kinase activity (GO:0 | 12966 | 0.173932962 | 0.451114198 |
| response to estrogen (GO:0043627) | 12966 | 0.173932962 | 0.451114198 |
| regulation of peptide hormone secretion (GO:0090276) | 13/74 | 0.17629986 | 0.451226966 |
| protein modification by small protein conjugation (GO:0C | 61/409 | 0.178016587 | 0.451226966 |
| production of miRNAs involved in gene silencing by miRN | 44741 | 0.1783151 | 0.451226966 |
| regulation of DNA biosynthetic process (GO:2000278) | 44741 | 0.1783151 | 0.451226966 |
| regulation of fatty acid biosynthetic process (GO:004230 | 44741 | 0.1783151 | 0.451226966 |
| transition metal ion homeostasis (GO:0055076) | 44741 | 0.1783151 | 0.451226966 |
| iron ion homeostasis (GO:0055072) | 22586 | 0.178696506 | 0.451226966 |
| protein lipidation (GO:0006497) | 22586 | 0.178696506 | 0.451226966 |
| cellular response to ionizing radiation (GO:0071479) | 17777 | 0.17872562 | 0.451226966 |
| positive regulation of endocytosis (GO:0045807) | 17777 | 0.17872562 | 0.451226966 |
| positive regulation of protein localization to membrane ( | 17/101 | 0.178898729 | 0.451226966 |
| cellular response to fatty acid (GO:0071398) | 44668 | 0.180090717 | 0.451226966 |
| diol biosynthetic process (GO:0034312) | 44668 | 0.180090717 | 0.451226966 |
| establishment of protein localization to endoplasmic reti | 44668 | 0.180090717 | 0.451226966 |
| histone H2A ubiquitination (GO:0033522) | 44668 | 0.180090717 | 0.451226966 |

| | | | |
|---|---|---|---|
| midbody abscission (GO:0061952) | 44668 | 0.180090717 | 0.451226966 |
| modified amino acid transport (GO:0072337) | 44668 | 0.180090717 | 0.451226966 |
| negative regulation of ATP metabolic process (GO:19035 | 44668 | 0.180090717 | 0.451226966 |
| negative regulation of vascular associated smooth muscle | 44668 | 0.180090717 | 0.451226966 |
| positive regulation of cardiac muscle tissue growth (GO:0 | 44668 | 0.180090717 | 0.451226966 |
| positive regulation of cyclin-dependent protein serine/th | 44668 | 0.180090717 | 0.451226966 |
| positive regulation of DNA replication (GO:0045740) | 44668 | 0.180090717 | 0.451226966 |
| positive regulation of smooth muscle cell migration (GO:( | 44668 | 0.180090717 | 0.451226966 |
| regulation of osteoblast proliferation (GO:0033688) | 44668 | 0.180090717 | 0.451226966 |
| response to purine-containing compound (GO:0014074) | 44668 | 0.180090717 | 0.451226966 |
| ruffle organization (GO:0031529) | 44668 | 0.180090717 | 0.451226966 |
| acylglycerol biosynthetic process (GO:0046463) | 44704 | 0.181010279 | 0.451226966 |
| blood vessel endothelial cell migration (GO:0043534) | 44704 | 0.181010279 | 0.451226966 |
| glucose 6-phosphate metabolic process (GO:0051156) | 44704 | 0.181010279 | 0.451226966 |
| negative regulation of intracellular protein transport (GO | 44704 | 0.181010279 | 0.451226966 |
| negative regulation of phosphoprotein phosphatase activ | 44704 | 0.181010279 | 0.451226966 |
| positive regulation of mRNA processing (GO:0050685) | 44704 | 0.181010279 | 0.451226966 |
| activation of Janus kinase activity (GO:0042976) | 44598 | 0.183530814 | 0.451226966 |
| adherens junction maintenance (GO:0034334) | 44598 | 0.183530814 | 0.451226966 |
| aromatic amino acid transport (GO:0015801) | 44598 | 0.183530814 | 0.451226966 |
| B cell chemotaxis (GO:0035754) | 44598 | 0.183530814 | 0.451226966 |
| chromatin organization involved in negative regulation of | 44598 | 0.183530814 | 0.451226966 |
| cobalamin transport (GO:0015889) | 44598 | 0.183530814 | 0.451226966 |
| dehydroascorbic acid transport (GO:0070837) | 44598 | 0.183530814 | 0.451226966 |
| gamma-aminobutyric acid transport (GO:0015812) | 44598 | 0.183530814 | 0.451226966 |
| histone H4-K12 acetylation (GO:0043983) | 44598 | 0.183530814 | 0.451226966 |
| imitative learning (GO:0098596) | 44598 | 0.183530814 | 0.451226966 |
| IMP biosynthetic process (GO:0006188) | 44598 | 0.183530814 | 0.451226966 |
| intracellular pH elevation (GO:0051454) | 44598 | 0.183530814 | 0.451226966 |
| isoleucine metabolic process (GO:0006549) | 44598 | 0.183530814 | 0.451226966 |
| L-arginine transmembrane transport (GO:1903400) | 44598 | 0.183530814 | 0.451226966 |
| L-ornithine transmembrane transport (GO:1903352) | 44598 | 0.183530814 | 0.451226966 |
| leucine transport (GO:0015820) | 44598 | 0.183530814 | 0.451226966 |
| lipoxin metabolic process (GO:2001300) | 44598 | 0.183530814 | 0.451226966 |
| lymphocyte apoptotic process (GO:0070227) | 44598 | 0.183530814 | 0.451226966 |
| molybdopterin cofactor biosynthetic process (GO:003232 | 44598 | 0.183530814 | 0.451226966 |
| monoubiquitinated protein deubiquitination (GO:003552 | 44598 | 0.183530814 | 0.451226966 |
| morphogenesis of an endothelium (GO:0003159) | 44598 | 0.183530814 | 0.451226966 |
| negative regulation of actin filament depolymerization (G | 44598 | 0.183530814 | 0.451226966 |
| negative regulation of B cell receptor signaling pathway ( | 44598 | 0.183530814 | 0.451226966 |
| negative regulation of blood vessel endothelial cell prolif | 44598 | 0.183530814 | 0.451226966 |
| negative regulation of cardiac muscle cell apoptotic proce | 44598 | 0.183530814 | 0.451226966 |
| negative regulation of cellular response to hypoxia (GO:1 | 44598 | 0.183530814 | 0.451226966 |
| negative regulation of fibroblast migration (GO:0010764) | 44598 | 0.183530814 | 0.451226966 |
| negative regulation of mast cell activation (GO:0033004) | 44598 | 0.183530814 | 0.451226966 |
| negative regulation of monocyte chemotaxis (GO:009002 | 44598 | 0.183530814 | 0.451226966 |
| negative regulation of protein targeting to membrane (G | 44598 | 0.183530814 | 0.451226966 |

| | | | |
|---|---|---|---|
| negative regulation of trophoblast cell migration (GO:190 | 44598 | 0.183530814 | 0.451226966 |
| nucleotide transport (GO:0006862) | 44598 | 0.183530814 | 0.451226966 |
| oxygen homeostasis (GO:0032364) | 44598 | 0.183530814 | 0.451226966 |
| pentose-phosphate shunt, non-oxidative branch (GO:000 | 44598 | 0.183530814 | 0.451226966 |
| peptide antigen assembly with MHC protein complex (GC | 44598 | 0.183530814 | 0.451226966 |
| positive regulation of centriole replication (GO:0046601) | 44598 | 0.183530814 | 0.451226966 |
| positive regulation of cholesterol metabolic process (GO: | 44598 | 0.183530814 | 0.451226966 |
| positive regulation of endoplasmic reticulum stress-induc | 44598 | 0.183530814 | 0.451226966 |
| positive regulation of gluconeogenesis (GO:0045722) | 44598 | 0.183530814 | 0.451226966 |
| positive regulation of histone phosphorylation (GO:0033 | 44598 | 0.183530814 | 0.451226966 |
| positive regulation of I-kappaB phosphorylation (GO:190 | 44598 | 0.183530814 | 0.451226966 |
| positive regulation of immune response to tumor cell (G | 44598 | 0.183530814 | 0.451226966 |
| positive regulation of osteoblast proliferation (GO:00336 | 44598 | 0.183530814 | 0.451226966 |
| positive regulation of T cell mediated immune response t | 44598 | 0.183530814 | 0.451226966 |
| positive regulation of tau-protein kinase activity (GO:190 | 44598 | 0.183530814 | 0.451226966 |
| protein geranylgeranylation (GO:0018344) | 44598 | 0.183530814 | 0.451226966 |
| protein insertion into mitochondrial membrane involved | 44598 | 0.183530814 | 0.451226966 |
| protein K29-linked ubiquitination (GO:0035519) | 44598 | 0.183530814 | 0.451226966 |
| protein-DNA complex subunit organization (GO:0071824) | 44598 | 0.183530814 | 0.451226966 |
| pteridine-containing compound biosynthetic process (GC | 44598 | 0.183530814 | 0.451226966 |
| purine nucleobase transport (GO:0006863) | 44598 | 0.183530814 | 0.451226966 |
| radial glial cell differentiation (GO:0060019) | 44598 | 0.183530814 | 0.451226966 |
| regulation of adiponectin secretion (GO:0070163) | 44598 | 0.183530814 | 0.451226966 |
| regulation of adipose tissue development (GO:1904177) | 44598 | 0.183530814 | 0.451226966 |
| regulation of blood vessel remodeling (GO:0060312) | 44598 | 0.183530814 | 0.451226966 |
| regulation of DNA demethylation (GO:1901535) | 44598 | 0.183530814 | 0.451226966 |
| regulation of extracellular exosome assembly (GO:19035 | 44598 | 0.183530814 | 0.451226966 |
| regulation of hematopoietic stem cell proliferation (GO:1 | 44598 | 0.183530814 | 0.451226966 |
| regulation of immature T cell proliferation in thymus (GO | 44598 | 0.183530814 | 0.451226966 |
| regulation of keratinocyte apoptotic process (GO:190217 | 44598 | 0.183530814 | 0.451226966 |
| regulation of macromolecule biosynthetic process (GO:0 | 44598 | 0.183530814 | 0.451226966 |
| regulation of mast cell chemotaxis (GO:0060753) | 44598 | 0.183530814 | 0.451226966 |
| regulation of membrane tubulation (GO:1903525) | 44598 | 0.183530814 | 0.451226966 |
| regulation of neutrophil extravasation (GO:2000389) | 44598 | 0.183530814 | 0.451226966 |
| regulation of protein autoubiquitination (GO:1902498) | 44598 | 0.183530814 | 0.451226966 |
| regulation of response to reactive oxygen species (GO:19 | 44598 | 0.183530814 | 0.451226966 |
| regulation of resting membrane potential (GO:0060075) | 44598 | 0.183530814 | 0.451226966 |
| regulation of sarcomere organization (GO:0060297) | 44598 | 0.183530814 | 0.451226966 |
| regulation of superoxide metabolic process (GO:0090322 | 44598 | 0.183530814 | 0.451226966 |
| regulation of T cell activation via T cell receptor contact v | 44598 | 0.183530814 | 0.451226966 |
| renal system process involved in regulation of blood volu | 44598 | 0.183530814 | 0.451226966 |
| replication-born double-strand break repair via sister chr | 44598 | 0.183530814 | 0.451226966 |
| retinal ganglion cell axon guidance (GO:0031290) | 44598 | 0.183530814 | 0.451226966 |
| signal complex assembly (GO:0007172) | 44598 | 0.183530814 | 0.451226966 |
| taurine metabolic process (GO:0019530) | 44598 | 0.183530814 | 0.451226966 |
| tRNA pseudouridine synthesis (GO:0031119) | 44598 | 0.183530814 | 0.451226966 |
| vesicle localization (GO:0051648) | 44598 | 0.183530814 | 0.451226966 |

| | | | |
|---|---|---|---|
| vocal learning (GO:0042297) | 44598 | 0.183530814 | 0.451226966 |
| canonical Wnt signaling pathway (GO:0060070) | 25173 | 0.184362254 | 0.45305672 |
| calcium ion transmembrane import into cytosol (GO:009 | 15554 | 0.18580794 | 0.455962327 |
| negative regulation of protein catabolic process (GO:004 | 15554 | 0.18580794 | 0.455962327 |
| regulation of DNA recombination (GO:0000018) | 15554 | 0.18580794 | 0.455962327 |
| positive regulation of response to DNA damage stimulus | 20363 | 0.186608349 | 0.457062883 |
| pyruvate metabolic process (GO:0006090) | 20363 | 0.186608349 | 0.457062883 |
| regulation of gene silencing by RNA (GO:0060966) | 20363 | 0.186608349 | 0.457062883 |
| regulation of posttranscriptional gene silencing (GO:0060 | 20363 | 0.186608349 | 0.457062883 |
| cellular response to insulin stimulus (GO:0032869) | 21/129 | 0.186713524 | 0.457104975 |
| organic substance transport (GO:0071702) | 22/136 | 0.187984786 | 0.460000453 |
| regulation of cell death (GO:0010941) | 17/102 | 0.189779426 | 0.464173314 |
| activation of adenylate cyclase activity (GO:0007190) | 13332 | 0.192857529 | 0.470077773 |
| establishment of protein localization to plasma membrar | 13332 | 0.192857529 | 0.470077773 |
| regulation of mRNA processing (GO:0050684) | 13332 | 0.192857529 | 0.470077773 |
| regulation of ossification (GO:0030278) | 13332 | 0.192857529 | 0.470077773 |
| regulation of receptor internalization (GO:0002090) | 13332 | 0.192857529 | 0.470077773 |
| transferrin transport (GO:0033572) | 13332 | 0.192857529 | 0.470077773 |
| regulation of G protein-coupled receptor signaling pathw | 14/82 | 0.192886654 | 0.470077773 |
| negative regulation of kinase activity (GO:0033673) | 22951 | 0.192917384 | 0.470077773 |
| sphingolipid metabolic process (GO:0006665) | 19/116 | 0.193741801 | 0.471865287 |
| response to unfolded protein (GO:0006986) | 18142 | 0.194906332 | 0.474256859 |
| RNA catabolic process (GO:0006401) | 18142 | 0.194906332 | 0.474256859 |
| positive regulation of canonical Wnt signaling pathway (G | 21/130 | 0.196519182 | 0.477957468 |
| positive regulation of protein transport (GO:0051222) | 25538 | 0.198017668 | 0.481376597 |
| negative regulation of cell migration (GO:0030336) | 23/144 | 0.19840888 | 0.482102026 |
| alpha-amino acid catabolic process (GO:1901606) | 44742 | 0.199578858 | 0.483362315 |
| fatty-acyl-CoA biosynthetic process (GO:0046949) | 44742 | 0.199578858 | 0.483362315 |
| Golgi to plasma membrane protein transport (GO:00430( | 44742 | 0.199578858 | 0.483362315 |
| heterotypic cell-cell adhesion (GO:0034113) | 44742 | 0.199578858 | 0.483362315 |
| maintenance of blood-brain barrier (GO:0035633) | 44742 | 0.199578858 | 0.483362315 |
| respiratory tube development (GO:0030323) | 44742 | 0.199578858 | 0.483362315 |
| termination of RNA polymerase I transcription (GO:0006 | 44742 | 0.199578858 | 0.483362315 |
| regulation of macromolecule metabolic process (GO:006( | 31/200 | 0.200490765 | 0.485344606 |
| positive regulation of establishment of protein localizatio | 20729 | 0.202008418 | 0.488391048 |
| regulation of ATP metabolic process (GO:1903578) | 20729 | 0.202008418 | 0.488391048 |
| positive regulation of growth (GO:0045927) | 13/76 | 0.202169484 | 0.488391048 |
| positive regulation of axonogenesis (GO:0050772) | 15919 | 0.20357451 | 0.488391048 |
| regulation of carbohydrate catabolic process (GO:004347 | 15919 | 0.20357451 | 0.488391048 |
| regulation of purine nucleotide metabolic process (GO:19 | 15919 | 0.20357451 | 0.488391048 |
| canonical glycolysis (GO:0061621) | 44705 | 0.205359904 | 0.488391048 |
| cardiac muscle cell differentiation (GO:0055007) | 44705 | 0.205359904 | 0.488391048 |
| cellular response to catecholamine stimulus (GO:007187( | 44705 | 0.205359904 | 0.488391048 |
| cholesterol efflux (GO:0033344) | 44705 | 0.205359904 | 0.488391048 |
| glucose catabolic process to pyruvate (GO:0061718) | 44705 | 0.205359904 | 0.488391048 |
| negative regulation of lipid metabolic process (GO:00458 | 44705 | 0.205359904 | 0.488391048 |
| negative regulation of neuron differentiation (GO:00456( | 44705 | 0.205359904 | 0.488391048 |

| | | | |
|---|---|---|---|
| positive regulation of cellular response to transforming g | 44705 | 0.205359904 | 0.488391048 |
| positive regulation of transforming growth factor beta re | 44705 | 0.205359904 | 0.488391048 |
| positive regulation of viral life cycle (GO:1903902) | 44705 | 0.205359904 | 0.488391048 |
| potassium ion homeostasis (GO:0055075) | 44705 | 0.205359904 | 0.488391048 |
| purine ribonucleotide catabolic process (GO:0009154) | 44705 | 0.205359904 | 0.488391048 |
| regulation of keratinocyte proliferation (GO:0010837) | 44705 | 0.205359904 | 0.488391048 |
| cardiocyte differentiation (GO:0035051) | 44632 | 0.206167771 | 0.488391048 |
| cellular response to fluid shear stress (GO:0071498) | 44632 | 0.206167771 | 0.488391048 |
| cellular response to nitrogen levels (GO:0043562) | 44632 | 0.206167771 | 0.488391048 |
| cellular response to nitrogen starvation (GO:0006995) | 44632 | 0.206167771 | 0.488391048 |
| cellular response to thyroid hormone stimulus (GO:0097( | 44632 | 0.206167771 | 0.488391048 |
| fat-soluble vitamin catabolic process (GO:0042363) | 44632 | 0.206167771 | 0.488391048 |
| glycine metabolic process (GO:0006544) | 44632 | 0.206167771 | 0.488391048 |
| intrinsic apoptotic signaling pathway in response to oxida | 44632 | 0.206167771 | 0.488391048 |
| maintenance of protein localization in endoplasmic reticu | 44632 | 0.206167771 | 0.488391048 |
| mitotic G1/S transition checkpoint signaling (GO:004481( | 44632 | 0.206167771 | 0.488391048 |
| mitotic nuclear membrane disassembly (GO:0007077) | 44632 | 0.206167771 | 0.488391048 |
| negative regulation by host of viral process (GO:0044793 | 44632 | 0.206167771 | 0.488391048 |
| negative regulation of complement activation (GO:00459 | 44632 | 0.206167771 | 0.488391048 |
| negative regulation of glucose transmembrane transport | 44632 | 0.206167771 | 0.488391048 |
| negative regulation of leukocyte apoptotic process (GO:2 | 44632 | 0.206167771 | 0.488391048 |
| negative regulation of vascular permeability (GO:004311( | 44632 | 0.206167771 | 0.488391048 |
| nuclear body organization (GO:0030575) | 44632 | 0.206167771 | 0.488391048 |
| organic hydroxy compound catabolic process (GO:19016: | 44632 | 0.206167771 | 0.488391048 |
| phagolysosome assembly (GO:0001845) | 44632 | 0.206167771 | 0.488391048 |
| positive regulation of myeloid leukocyte mediated immu | 44632 | 0.206167771 | 0.488391048 |
| positive regulation of protein maturation (GO:1903319) | 44632 | 0.206167771 | 0.488391048 |
| posttranslational protein targeting to endoplasmic reticu | 44632 | 0.206167771 | 0.488391048 |
| presynapse organization (GO:0099172) | 44632 | 0.206167771 | 0.488391048 |
| pyrimidine-containing compound metabolic process (GO: | 44632 | 0.206167771 | 0.488391048 |
| regulation of astrocyte differentiation (GO:0048710) | 44632 | 0.206167771 | 0.488391048 |
| regulation of chromatin binding (GO:0035561) | 44632 | 0.206167771 | 0.488391048 |
| regulation of hemopoiesis (GO:1903706) | 44632 | 0.206167771 | 0.488391048 |
| regulation of keratinocyte migration (GO:0051547) | 44632 | 0.206167771 | 0.488391048 |
| anterior/posterior pattern specification (GO:0009952) | 23316 | 0.20762624 | 0.489066569 |
| cell cycle G1/S phase transition (GO:0044843) | 23316 | 0.20762624 | 0.489066569 |
| acidic amino acid transport (GO:0015800) | 44669 | 0.208806477 | 0.489066569 |
| alternative mRNA splicing, via spliceosome (GO:0000380) | 44669 | 0.208806477 | 0.489066569 |
| cell migration involved in sprouting angiogenesis (GO:00( | 44669 | 0.208806477 | 0.489066569 |
| cellular response to glucocorticoid stimulus (GO:0071385 | 44669 | 0.208806477 | 0.489066569 |
| hyaluronan catabolic process (GO:0030214) | 44669 | 0.208806477 | 0.489066569 |
| maintenance of gastrointestinal epithelium (GO:0030277 | 44669 | 0.208806477 | 0.489066569 |
| mitochondrial fission (GO:0000266) | 44669 | 0.208806477 | 0.489066569 |
| negative regulation of leukocyte cell-cell adhesion (GO:1( | 44669 | 0.208806477 | 0.489066569 |
| neuron maturation (GO:0042551) | 44669 | 0.208806477 | 0.489066569 |
| positive chemotaxis (GO:0050918) | 44669 | 0.208806477 | 0.489066569 |
| positive regulation of cell adhesion mediated by integrin | 44669 | 0.208806477 | 0.489066569 |

| | | | |
|---|---|---|---|
| positive regulation of macrophage derived foam cell diffe | 44669 | 0.208806477 | 0.489066569 |
| positive regulation of mRNA splicing, via spliceosome (GC | 44669 | 0.208806477 | 0.489066569 |
| positive regulation of myoblast differentiation (GO:0045€ | 44669 | 0.208806477 | 0.489066569 |
| positive regulation of nitric-oxide synthase activity (GO:0 | 44669 | 0.208806477 | 0.489066569 |
| positive regulation of synaptic transmission, glutamaterg | 44669 | 0.208806477 | 0.489066569 |
| regulation of catabolic process (GO:0009894) | 44669 | 0.208806477 | 0.489066569 |
| regulation of mitochondrial outer membrane permeabiliz | 44669 | 0.208806477 | 0.489066569 |
| regulation of oxidative stress-induced intrinsic apoptotic | 44669 | 0.208806477 | 0.489066569 |
| regulation of protein sumoylation (GO:0033233) | 44669 | 0.208806477 | 0.489066569 |
| regulation of transcription by RNA polymerase I (GO:000( | 44669 | 0.208806477 | 0.489066569 |
| release of cytochrome c from mitochondria (GO:00183€ | 44669 | 0.208806477 | 0.489066569 |
| triglyceride biosynthetic process (GO:0019432) | 44669 | 0.208806477 | 0.489066569 |
| wound healing (GO:0042060) | 25903 | 0.212110295 | 0.496580884 |
| glutamine family amino acid metabolic process (GO:0009 | 13697 | 0.212557129 | 0.496955124 |
| positive regulation of calcium ion transport (GO:0051928 | 13697 | 0.212557129 | 0.496955124 |
| regulation of protein autophosphorylation (GO:0031952) | 13697 | 0.212557129 | 0.496955124 |
| supramolecular fiber organization (GO:0097435) | 52/351 | 0.214301289 | 0.500807555 |
| monocarboxylic acid transport (GO:0015718) | 21094 | 0.217925805 | 0.509048812 |
| cellular nitrogen compound biosynthetic process (GO:00 | 11475 | 0.221713185 | 0.515512937 |
| multivesicular body organization (GO:0036257) | 11475 | 0.221713185 | 0.515512937 |
| negative regulation of DNA binding (GO:0043392) | 11475 | 0.221713185 | 0.515512937 |
| positive regulation of protein targeting to mitochondrion | 11475 | 0.221713185 | 0.515512937 |
| protein acetylation (GO:0006473) | 11475 | 0.221713185 | 0.515512937 |
| regulation of cellular pH (GO:0030641) | 11475 | 0.221713185 | 0.515512937 |
| regulation of cellular senescence (GO:2000772) | 11475 | 0.221713185 | 0.515512937 |
| triglyceride homeostasis (GO:0070328) | 11475 | 0.221713185 | 0.515512937 |
| homotypic cell-cell adhesion (GO:0034109) | 16285 | 0.221983146 | 0.515512937 |
| mRNA catabolic process (GO:0006402) | 16285 | 0.221983146 | 0.515512937 |
| mRNA stabilization (GO:0048255) | 16285 | 0.221983146 | 0.515512937 |
| positive regulation of biosynthetic process (GO:0009891) | 16285 | 0.221983146 | 0.515512937 |
| positive regulation of cell-matrix adhesion (GO:0001954) | 16285 | 0.221983146 | 0.515512937 |
| regulation of cilium assembly (GO:1902017) | 23682 | 0.222793403 | 0.516932438 |
| regulation of transcription initiation from RNA polymeras | 23682 | 0.222793403 | 0.516932438 |
| histone acetylation (GO:0016573) | 26268 | 0.226614722 | 0.516997084 |
| regulation of hematopoietic stem cell differentiation (GO | 26268 | 0.226614722 | 0.516997084 |
| mesenchymal cell differentiation (GO:0048762) | 18872 | 0.229005241 | 0.516997084 |
| protein modification process (GO:0036211) | 13/78 | 0.229622642 | 0.516997084 |
| phosphatidylinositol dephosphorylation (GO:0046856) | 44706 | 0.230706139 | 0.516997084 |
| positive regulation of DNA-templated transcription, elon | 44706 | 0.230706139 | 0.516997084 |
| positive regulation of lipid metabolic process (GO:00458 | 44706 | 0.230706139 | 0.516997084 |
| regulation of protein processing (GO:0070613) | 44706 | 0.230706139 | 0.516997084 |
| regulation of proteolysis involved in cellular protein catal | 44706 | 0.230706139 | 0.516997084 |
| response to dsRNA (GO:0043331) | 44706 | 0.230706139 | 0.516997084 |
| sodium ion homeostasis (GO:0055078) | 44706 | 0.230706139 | 0.516997084 |
| G1/S transition of mitotic cell cycle (GO:0000082) | 14/85 | 0.231986233 | 0.516997084 |
| mRNA destabilization (GO:0061157) | 14062 | 0.232953097 | 0.516997084 |
| negative regulation of ubiquitin-dependent protein catab | 14062 | 0.232953097 | 0.516997084 |

| | | | |
|---|---|---|---|
| positive regulation of cell development (GO:0010720) | 14062 | 0.232953097 | 0.516997084 |
| positive regulation of signaling (GO:0023056) | 14062 | 0.232953097 | 0.516997084 |
| response to cAMP (GO:0051591) | 14062 | 0.232953097 | 0.516997084 |
| response to estradiol (GO:0032355) | 14062 | 0.232953097 | 0.516997084 |
| synaptic vesicle endocytosis (GO:0048488) | 14062 | 0.232953097 | 0.516997084 |
| negative regulation of mitotic cell cycle phase transition ( | 15/92 | 0.233831325 | 0.516997084 |
| muscle organ development (GO:0007517) | 21459 | 0.234322679 | 0.516997084 |
| regulation of calcium ion transport (GO:0051924) | 21459 | 0.234322679 | 0.516997084 |
| aldehyde biosynthetic process (GO:0046184) | 44599 | 0.235360559 | 0.516997084 |
| angiotensin-activated signaling pathway (GO:0038166) | 44599 | 0.235360559 | 0.516997084 |
| arginine catabolic process (GO:0006527) | 44599 | 0.235360559 | 0.516997084 |
| bleb assembly (GO:0032060) | 44599 | 0.235360559 | 0.516997084 |
| calcium ion export (GO:1901660) | 44599 | 0.235360559 | 0.516997084 |
| cardiolipin acyl-chain remodeling (GO:0035965) | 44599 | 0.235360559 | 0.516997084 |
| cardiolipin biosynthetic process (GO:0032049) | 44599 | 0.235360559 | 0.516997084 |
| catecholamine catabolic process (GO:0042424) | 44599 | 0.235360559 | 0.516997084 |
| cellular protein complex disassembly (GO:0043624) | 44599 | 0.235360559 | 0.516997084 |
| cellular response to caffeine (GO:0071313) | 44599 | 0.235360559 | 0.516997084 |
| cellular response to monoamine stimulus (GO:0071868) | 44599 | 0.235360559 | 0.516997084 |
| cleavage furrow formation (GO:0036089) | 44599 | 0.235360559 | 0.516997084 |
| dendrite arborization (GO:0140059) | 44599 | 0.235360559 | 0.516997084 |
| dopamine transport (GO:0015872) | 44599 | 0.235360559 | 0.516997084 |
| dorsal aorta development (GO:0035907) | 44599 | 0.235360559 | 0.516997084 |
| dorsal aorta morphogenesis (GO:0035912) | 44599 | 0.235360559 | 0.516997084 |
| endosome to lysosome transport via multivesicular body | 44599 | 0.235360559 | 0.516997084 |
| establishment of centrosome localization (GO:0051660) | 44599 | 0.235360559 | 0.516997084 |
| fatty acid elongation, monounsaturated fatty acid (GO:0( | 44599 | 0.235360559 | 0.516997084 |
| fatty acid elongation, polyunsaturated fatty acid (GO:003 | 44599 | 0.235360559 | 0.516997084 |
| fatty acid elongation, saturated fatty acid (GO:0019367) | 44599 | 0.235360559 | 0.516997084 |
| fatty acid elongation, unsaturated fatty acid (GO:001936ł | 44599 | 0.235360559 | 0.516997084 |
| gamma-delta T cell activation (GO:0046629) | 44599 | 0.235360559 | 0.516997084 |
| gap junction assembly (GO:0016264) | 44599 | 0.235360559 | 0.516997084 |
| gas homeostasis (GO:0033483) | 44599 | 0.235360559 | 0.516997084 |
| gland morphogenesis (GO:0022612) | 44599 | 0.235360559 | 0.516997084 |
| glutamine transport (GO:0006868) | 44599 | 0.235360559 | 0.516997084 |
| glutathione catabolic process (GO:0006751) | 44599 | 0.235360559 | 0.516997084 |
| glyceraldehyde-3-phosphate metabolic process (GO:001S | 44599 | 0.235360559 | 0.516997084 |
| hemostasis (GO:0007599) | 44599 | 0.235360559 | 0.516997084 |
| histone H3-K14 acetylation (GO:0044154) | 44599 | 0.235360559 | 0.516997084 |
| inhibition of cysteine-type endopeptidase activity (GO:00 | 44599 | 0.235360559 | 0.516997084 |
| iron coordination entity transport (GO:1901678) | 44599 | 0.235360559 | 0.516997084 |
| iron import into cell (GO:0033212) | 44599 | 0.235360559 | 0.516997084 |
| lipoxin biosynthetic process (GO:2001301) | 44599 | 0.235360559 | 0.516997084 |
| lymphoid progenitor cell differentiation (GO:0002320) | 44599 | 0.235360559 | 0.516997084 |
| lysosomal microautophagy (GO:0016237) | 44599 | 0.235360559 | 0.516997084 |
| membrane invagination (GO:0010324) | 44599 | 0.235360559 | 0.516997084 |
| miRNA loading onto RISC involved in gene silencing by mi | 44599 | 0.235360559 | 0.516997084 |

| | | | |
|---|---|---|---|
| mitochondrion-endoplasmic reticulum membrane tether | 44599 | 0.235360559 | 0.516997084 |
| modulation by symbiont of host defense response (GO:0 | 44599 | 0.235360559 | 0.516997084 |
| monoacylglycerol biosynthetic process (GO:0006640) | 44599 | 0.235360559 | 0.516997084 |
| negative regulation by host of viral genome replication (( | 44599 | 0.235360559 | 0.516997084 |
| negative regulation of acute inflammatory response (GO: | 44599 | 0.235360559 | 0.516997084 |
| negative regulation of cell cycle phase transition (GO:190 | 44599 | 0.235360559 | 0.516997084 |
| negative regulation of digestive system process (GO:006( | 44599 | 0.235360559 | 0.516997084 |
| negative regulation of low-density lipoprotein receptor a | 44599 | 0.235360559 | 0.516997084 |
| negative regulation of membrane protein ectodomain pr | 44599 | 0.235360559 | 0.516997084 |
| negative regulation of protein K63-linked ubiquitination ( | 44599 | 0.235360559 | 0.516997084 |
| negative regulation of retinoic acid receptor signaling pat | 44599 | 0.235360559 | 0.516997084 |
| negative regulation of T cell apoptotic process (GO:0070 | 44599 | 0.235360559 | 0.516997084 |
| neuron projection arborization (GO:0140058) | 44599 | 0.235360559 | 0.516997084 |
| nicotinamide nucleotide biosynthetic process (GO:00193 | 44599 | 0.235360559 | 0.516997084 |
| nodal signaling pathway (GO:0038092) | 44599 | 0.235360559 | 0.516997084 |
| nucleobase catabolic process (GO:0046113) | 44599 | 0.235360559 | 0.516997084 |
| parallel actin filament bundle assembly (GO:0030046) | 44599 | 0.235360559 | 0.516997084 |
| peptide transport (GO:0015833) | 44599 | 0.235360559 | 0.516997084 |
| piecemeal microautophagy of the nucleus (GO:0034727) | 44599 | 0.235360559 | 0.516997084 |
| positive regulation of amyloid-beta clearance (GO:19002 | 44599 | 0.235360559 | 0.516997084 |
| positive regulation of aspartic-type endopeptidase activit | 44599 | 0.235360559 | 0.516997084 |
| positive regulation of cilium movement (GO:0003353) | 44599 | 0.235360559 | 0.516997084 |
| positive regulation of cilium-dependent cell motility (GO: | 44599 | 0.235360559 | 0.516997084 |
| positive regulation of ER-associated ubiquitin-dependent | 44599 | 0.235360559 | 0.516997084 |
| positive regulation of flagellated sperm motility (GO:190 | 44599 | 0.235360559 | 0.516997084 |
| positive regulation of hormone metabolic process (GO:0( | 44599 | 0.235360559 | 0.516997084 |
| positive regulation of icosanoid secretion (GO:0032305) | 44599 | 0.235360559 | 0.516997084 |
| positive regulation of interleukin-5 production (GO:0032 | 44599 | 0.235360559 | 0.516997084 |
| positive regulation of lamellipodium morphogenesis (GO: | 44599 | 0.235360559 | 0.516997084 |
| positive regulation of mast cell activation (GO:0033005) | 44599 | 0.235360559 | 0.516997084 |
| positive regulation of miRNA mediated inhibition of trans | 44599 | 0.235360559 | 0.516997084 |
| positive regulation of pinocytosis (GO:0048549) | 44599 | 0.235360559 | 0.516997084 |
| positive regulation of podosome assembly (GO:0071803) | 44599 | 0.235360559 | 0.516997084 |
| positive regulation of type I interferon-mediated signalin | 44599 | 0.235360559 | 0.516997084 |
| positive regulation of urine volume (GO:0035810) | 44599 | 0.235360559 | 0.516997084 |
| positive regulation of vesicle fusion (GO:0031340) | 44599 | 0.235360559 | 0.516997084 |
| protein localization to endoplasmic reticulum exit site (G | 44599 | 0.235360559 | 0.516997084 |
| receptor-mediated endocytosis of virus by host cell (GO:( | 44599 | 0.235360559 | 0.516997084 |
| regulation by virus of viral protein levels in host cell (GO: | 44599 | 0.235360559 | 0.516997084 |
| regulation of chromatin assembly or disassembly (GO:00( | 44599 | 0.235360559 | 0.516997084 |
| regulation of endothelial cell chemotaxis to fibroblast grc | 44599 | 0.235360559 | 0.516997084 |
| regulation of entry of bacterium into host cell (GO:20005 | 44599 | 0.235360559 | 0.516997084 |
| regulation of extracellular matrix assembly (GO:1901201) | 44599 | 0.235360559 | 0.516997084 |
| regulation of histone H3-K27 methylation (GO:0061085) | 44599 | 0.235360559 | 0.516997084 |
| regulation of ketone biosynthetic process (GO:0010566) | 44599 | 0.235360559 | 0.516997084 |
| regulation of MDA-5 signaling pathway (GO:0039533) | 44599 | 0.235360559 | 0.516997084 |
| regulation of miRNA mediated inhibition of translation (G | 44599 | 0.235360559 | 0.516997084 |

| | | | |
|---|---|---|---|
| regulation of muscle organ development (GO:0048634) | 44599 | 0.235360559 | 0.516997084 |
| regulation of NAD(P)H oxidase activity (GO:0033860) | 44599 | 0.235360559 | 0.516997084 |
| regulation of oxidative stress-induced neuron intrinsic ap | 44599 | 0.235360559 | 0.516997084 |
| regulation of relaxation of muscle (GO:1901077) | 44599 | 0.235360559 | 0.516997084 |
| regulation of skeletal muscle contraction (GO:0014819) | 44599 | 0.235360559 | 0.516997084 |
| regulation of striated muscle cell differentiation (GO:005 | 44599 | 0.235360559 | 0.516997084 |
| regulation of transforming growth factor beta2 productic | 44599 | 0.235360559 | 0.516997084 |
| regulation of vascular associated smooth muscle cell apo | 44599 | 0.235360559 | 0.516997084 |
| response to caffeine (GO:0031000) | 44599 | 0.235360559 | 0.516997084 |
| response to host defenses (GO:0052200) | 44599 | 0.235360559 | 0.516997084 |
| response to increased oxygen levels (GO:0036296) | 44599 | 0.235360559 | 0.516997084 |
| ribose phosphate biosynthetic process (GO:0046390) | 44599 | 0.235360559 | 0.516997084 |
| ribosomal small subunit export from nucleus (GO:000005 | 44599 | 0.235360559 | 0.516997084 |
| RIG-I signaling pathway (GO:0039529) | 44599 | 0.235360559 | 0.516997084 |
| succinate transport (GO:0015744) | 44599 | 0.235360559 | 0.516997084 |
| tachykinin receptor signaling pathway (GO:0007217) | 44599 | 0.235360559 | 0.516997084 |
| tRNA threonylcarbamoyladenosine metabolic process (G( | 44599 | 0.235360559 | 0.516997084 |
| white fat cell differentiation (GO:0050872) | 44599 | 0.235360559 | 0.516997084 |
| axonogenesis (GO:0007409) | 36/240 | 0.23787727 | 0.521240836 |
| dopamine metabolic process (GO:0042417) | 44670 | 0.238697199 | 0.521240836 |
| establishment of skin barrier (GO:0061436) | 44670 | 0.238697199 | 0.521240836 |
| establishment or maintenance of apical/basal cell polarit | 44670 | 0.238697199 | 0.521240836 |
| heart trabecula morphogenesis (GO:0061384) | 44670 | 0.238697199 | 0.521240836 |
| negative regulation of amyloid precursor protein cataboli | 44670 | 0.238697199 | 0.521240836 |
| negative regulation of oxidative stress-induced intrinsic a | 44670 | 0.238697199 | 0.521240836 |
| positive regulation of actin cytoskeleton reorganization (( | 44670 | 0.238697199 | 0.521240836 |
| positive regulation of cellular protein catabolic process (( | 44670 | 0.238697199 | 0.521240836 |
| positive regulation of cilium assembly (GO:0045724) | 44670 | 0.238697199 | 0.521240836 |
| positive regulation of macrophage migration (GO:190552 | 44670 | 0.238697199 | 0.521240836 |
| regulation of RIG-I signaling pathway (GO:0039535) | 44670 | 0.238697199 | 0.521240836 |
| regulation of water loss via skin (GO:0033561) | 44670 | 0.238697199 | 0.521240836 |
| sphingosine metabolic process (GO:0006670) | 44670 | 0.238697199 | 0.521240836 |
| protein deacetylation (GO:0006476) | 16650 | 0.240972983 | 0.522494473 |
| negative regulation of epithelial cell proliferation (GO:00! | 26634 | 0.241503771 | 0.522494473 |
| negative regulation of supramolecular fiber organization | 26634 | 0.241503771 | 0.522494473 |
| positive regulation of secretion by cell (GO:1903532) | 26634 | 0.241503771 | 0.522494473 |
| central nervous system neuron axonogenesis (GO:00219! | 44633 | 0.243294347 | 0.522494473 |
| chromatin organization involved in regulation of transcrip | 44633 | 0.243294347 | 0.522494473 |
| endonucleolytic cleavage of tricistronic rRNA transcript (! | 44633 | 0.243294347 | 0.522494473 |
| establishment of protein localization to chromosome (GC | 44633 | 0.243294347 | 0.522494473 |
| fatty acid elongation (GO:0030497) | 44633 | 0.243294347 | 0.522494473 |
| glucose import (GO:0046323) | 44633 | 0.243294347 | 0.522494473 |
| high-density lipoprotein particle assembly (GO:0034380) | 44633 | 0.243294347 | 0.522494473 |
| lymphocyte mediated immunity (GO:0002449) | 44633 | 0.243294347 | 0.522494473 |
| negative regulation of autophagosome assembly (GO:19( | 44633 | 0.243294347 | 0.522494473 |
| negative regulation of gluconeogenesis (GO:0045721) | 44633 | 0.243294347 | 0.522494473 |
| negative regulation of myotube differentiation (GO:0010 | 44633 | 0.243294347 | 0.522494473 |

| | | | |
|---|---|---|---|
| pathway-restricted SMAD protein phosphorylation (GO:0 | 44633 | 0.243294347 | 0.522494473 |
| pentose-phosphate shunt (GO:0006098) | 44633 | 0.243294347 | 0.522494473 |
| positive regulation of ERAD pathway (GO:1904294) | 44633 | 0.243294347 | 0.522494473 |
| positive regulation of histone deacetylation (GO:003106! | 44633 | 0.243294347 | 0.522494473 |
| positive regulation of neuron migration (GO:2001224) | 44633 | 0.243294347 | 0.522494473 |
| positive regulation of protein acetylation (GO:1901985) | 44633 | 0.243294347 | 0.522494473 |
| positive regulation of purine nucleotide biosynthetic pro( | 44633 | 0.243294347 | 0.522494473 |
| positive regulation of ruffle assembly (GO:1900029) | 44633 | 0.243294347 | 0.522494473 |
| protein localization to mitochondrion (GO:0070585) | 44633 | 0.243294347 | 0.522494473 |
| purine nucleotide catabolic process (GO:0006195) | 44633 | 0.243294347 | 0.522494473 |
| purine ribonucleoside triphosphate metabolic process (G | 44633 | 0.243294347 | 0.522494473 |
| regulation of autophagosome maturation (GO:1901096) | 44633 | 0.243294347 | 0.522494473 |
| regulation of cell communication by electrical coupling (( | 44633 | 0.243294347 | 0.522494473 |
| regulation of interleukin-5 production (GO:0032674) | 44633 | 0.243294347 | 0.522494473 |
| regulation of mRNA metabolic process (GO:1903311) | 44633 | 0.243294347 | 0.522494473 |
| regulation of protein acetylation (GO:1901983) | 44633 | 0.243294347 | 0.522494473 |
| regulation of retrograde protein transport, ER to cytosol ( | 44633 | 0.243294347 | 0.522494473 |
| response to iron ion (GO:0010039) | 44633 | 0.243294347 | 0.522494473 |
| response to thyroid hormone (GO:0097066) | 44633 | 0.243294347 | 0.522494473 |
| ribosomal subunit export from nucleus (GO:0000054) | 44633 | 0.243294347 | 0.522494473 |
| sodium ion export across plasma membrane (GO:003637 | 44633 | 0.243294347 | 0.522494473 |
| sphingoid biosynthetic process (GO:0046520) | 44633 | 0.243294347 | 0.522494473 |
| sphingosine biosynthetic process (GO:0046512) | 44633 | 0.243294347 | 0.522494473 |
| spinal cord development (GO:0021510) | 44633 | 0.243294347 | 0.522494473 |
| very long-chain fatty acid biosynthetic process (GO:0042 | 44633 | 0.243294347 | 0.522494473 |
| 7-methylguanosine mRNA capping (GO:0006370) | 11841 | 0.244605855 | 0.522717983 |
| 7-methylguanosine RNA capping (GO:0009452) | 11841 | 0.244605855 | 0.522717983 |
| activation of phospholipase C activity (GO:0007202) | 11841 | 0.244605855 | 0.522717983 |
| modulation by host of symbiont process (GO:0051851) | 11841 | 0.244605855 | 0.522717983 |
| negative regulation of cell-matrix adhesion (GO:0001953 | 11841 | 0.244605855 | 0.522717983 |
| negative regulation of DNA biosynthetic process (GO:200 | 11841 | 0.244605855 | 0.522717983 |
| positive regulation of lamellipodium organization (GO:19 | 11841 | 0.244605855 | 0.522717983 |
| protein K63-linked deubiquitination (GO:0070536) | 11841 | 0.244605855 | 0.522717983 |
| regulation of cellular amide metabolic process (GO:0034 | 11841 | 0.244605855 | 0.522717983 |
| regulation of centrosome duplication (GO:0010824) | 11841 | 0.244605855 | 0.522717983 |
| regulation of intracellular protein transport (GO:0033157 | 11841 | 0.244605855 | 0.522717983 |
| regulation of NMDA receptor activity (GO:2000310) | 11841 | 0.244605855 | 0.522717983 |
| plasma membrane bounded cell projection morphogenes | 19238 | 0.246828773 | 0.527034722 |
| signal transduction in response to DNA damage (GO:004 | 19238 | 0.246828773 | 0.527034722 |
| regulation of transforming growth factor beta receptor si | 16/100 | 0.247998346 | 0.529314465 |
| protein localization to organelle (GO:0033365) | 22/142 | 0.248813665 | 0.530836545 |
| negative regulation of cellular metabolic process (GO:003 | 14427 | 0.253962878 | 0.540490434 |
| organophosphate biosynthetic process (GO:0090407) | 14427 | 0.253962878 | 0.540490434 |
| phospholipid dephosphorylation (GO:0046839) | 14427 | 0.253962878 | 0.540490434 |
| regulation of cellular component biogenesis (GO:004408 | 14427 | 0.253962878 | 0.540490434 |
| regulation of intracellular transport (GO:0032386) | 14427 | 0.253962878 | 0.540490434 |
| regulation of protein targeting to mitochondrion (GO:190 | 14427 | 0.253962878 | 0.540490434 |

| | | | |
|---|---|---|---|
| glial cell development (GO:0021782) | 44707 | 0.256880182 | 0.544246551 |
| hippo signaling (GO:0035329) | 44707 | 0.256880182 | 0.544246551 |
| membrane depolarization during action potential (GO:00 | 44707 | 0.256880182 | 0.544246551 |
| mRNA polyadenylation (GO:0006378) | 44707 | 0.256880182 | 0.544246551 |
| negative regulation of cell development (GO:0010721) | 44707 | 0.256880182 | 0.544246551 |
| negative regulation of protein localization to membrane ( | 44707 | 0.256880182 | 0.544246551 |
| organelle fission (GO:0048285) | 44707 | 0.256880182 | 0.544246551 |
| phosphatidylethanolamine acyl-chain remodeling (GO:00 | 44707 | 0.256880182 | 0.544246551 |
| proteoglycan metabolic process (GO:0006029) | 44707 | 0.256880182 | 0.544246551 |
| regulation of biomineral tissue development (GO:007016 | 44707 | 0.256880182 | 0.544246551 |
| RNA polyadenylation (GO:0043631) | 44707 | 0.256880182 | 0.544246551 |
| calcium ion transmembrane transport (GO:0070588) | 14/87 | 0.259698691 | 0.549769642 |
| sodium ion transmembrane transport (GO:0035725) | 14/87 | 0.259698691 | 0.549769642 |
| regulation of synaptic transmission, glutamatergic (GO:0( | 17015 | 0.260480209 | 0.551199465 |
| cellular protein metabolic process (GO:0044267) | 60/417 | 0.267209464 | 0.565208943 |
| regulation of cellular macromolecule biosynthetic proces | 67/468 | 0.267771658 | 0.566167589 |
| phosphatidylcholine biosynthetic process (GO:0006656) | 12206 | 0.268141228 | 0.566487872 |
| regulation of cholesterol efflux (GO:0010874) | 12206 | 0.268141228 | 0.566487872 |
| peptidyl-threonine phosphorylation (GO:0018107) | 22190 | 0.268392537 | 0.566565354 |
| cellular response to misfolded protein (GO:0071218) | 44671 | 0.26948662 | 0.566565354 |
| cellular response to nitrogen compound (GO:1901699) | 44671 | 0.26948662 | 0.566565354 |
| energy reserve metabolic process (GO:0006112) | 44671 | 0.26948662 | 0.566565354 |
| ganglioside metabolic process (GO:0001573) | 44671 | 0.26948662 | 0.566565354 |
| hepaticobiliary system development (GO:0061008) | 44671 | 0.26948662 | 0.566565354 |
| miRNA metabolic process (GO:0010586) | 44671 | 0.26948662 | 0.566565354 |
| mitotic cytokinetic process (GO:1902410) | 44671 | 0.26948662 | 0.566565354 |
| positive regulation of lymphocyte differentiation (GO:004 | 44671 | 0.26948662 | 0.566565354 |
| protein targeting to lysosome (GO:0006622) | 44671 | 0.26948662 | 0.566565354 |
| regulation of smooth muscle cell migration (GO:0014910 | 44671 | 0.26948662 | 0.566565354 |
| xenobiotic transport (GO:0042908) | 44671 | 0.26948662 | 0.566565354 |
| positive regulation of neuron projection development (G | 14/88 | 0.273983709 | 0.575657443 |
| lipid transport (GO:0006869) | 17/109 | 0.274032889 | 0.575657443 |
| fatty acid derivative biosynthetic process (GO:1901570) | 14793 | 0.275501226 | 0.576398178 |
| positive regulation of catalytic activity (GO:0043085) | 14793 | 0.275501226 | 0.576398178 |
| positive regulation of double-strand break repair (GO:20( | 14793 | 0.275501226 | 0.576398178 |
| antiviral innate immune response (GO:0140374) | 44634 | 0.281353746 | 0.576398178 |
| branching involved in blood vessel morphogenesis (GO:0( | 44634 | 0.281353746 | 0.576398178 |
| cAMP metabolic process (GO:0046058) | 44634 | 0.281353746 | 0.576398178 |
| covalent chromatin modification (GO:0016569) | 44634 | 0.281353746 | 0.576398178 |
| immune response-regulating cell surface receptor signali | 44634 | 0.281353746 | 0.576398178 |
| low-density lipoprotein particle remodeling (GO:0034374 | 44634 | 0.281353746 | 0.576398178 |
| negative regulation of cartilage development (GO:00610: | 44634 | 0.281353746 | 0.576398178 |
| negative regulation of neuroinflammatory response (GO: | 44634 | 0.281353746 | 0.576398178 |
| negative regulation of smooth muscle cell migration (GO: | 44634 | 0.281353746 | 0.576398178 |
| neuron fate commitment (GO:0048663) | 44634 | 0.281353746 | 0.576398178 |
| nuclear membrane disassembly (GO:0051081) | 44634 | 0.281353746 | 0.576398178 |
| nucleoside phosphate catabolic process (GO:1901292) | 44634 | 0.281353746 | 0.576398178 |

| | | | |
|---|---|---|---|
| peptidyl-serine dephosphorylation (GO:0070262) | 44634 | 0.281353746 | 0.576398178 |
| polarized epithelial cell differentiation (GO:0030859) | 44634 | 0.281353746 | 0.576398178 |
| positive regulation of cardiac muscle cell proliferation (G( | 44634 | 0.281353746 | 0.576398178 |
| positive regulation of insulin secretion involved in cellula | 44634 | 0.281353746 | 0.576398178 |
| positive regulation of long-term synaptic potentiation (G( | 44634 | 0.281353746 | 0.576398178 |
| positive regulation of protein-containing complex disasse | 44634 | 0.281353746 | 0.576398178 |
| positive regulation of superoxide anion generation (GO:0 | 44634 | 0.281353746 | 0.576398178 |
| pre-miRNA processing (GO:0031054) | 44634 | 0.281353746 | 0.576398178 |
| protein modification by small protein conjugation or rem | 44634 | 0.281353746 | 0.576398178 |
| regulation of cell projection organization (GO:0031344) | 44634 | 0.281353746 | 0.576398178 |
| regulation of histone methylation (GO:0031060) | 44634 | 0.281353746 | 0.576398178 |
| response to X-ray (GO:0010165) | 44634 | 0.281353746 | 0.576398178 |
| sodium-independent organic anion transport (GO:00432! | 44634 | 0.281353746 | 0.576398178 |
| steroid catabolic process (GO:0006706) | 44634 | 0.281353746 | 0.576398178 |
| thymus development (GO:0048538) | 44634 | 0.281353746 | 0.576398178 |
| camera-type eye development (GO:0043010) | 44708 | 0.283712288 | 0.576398178 |
| negative regulation of catalytic activity (GO:0043086) | 44708 | 0.283712288 | 0.576398178 |
| peptide hormone secretion (GO:0030072) | 44708 | 0.283712288 | 0.576398178 |
| regulation of cell junction assembly (GO:1901888) | 44708 | 0.283712288 | 0.576398178 |
| regulation of vasculature development (GO:1901342) | 44708 | 0.283712288 | 0.576398178 |
| cytoplasmic microtubule organization (GO:0031122) | 19968 | 0.283769051 | 0.576398178 |
| negative regulation of response to stimulus (GO:004858! | 22555 | 0.285979993 | 0.576398178 |
| regulation of translational initiation (GO:0006446) | 25143 | 0.287382649 | 0.576398178 |
| aromatic compound biosynthetic process (GO:0019438) | 44600 | 0.287810755 | 0.576398178 |
| aromatic compound catabolic process (GO:0019439) | 44600 | 0.287810755 | 0.576398178 |
| autophagosome-lysosome fusion (GO:0061909) | 44600 | 0.287810755 | 0.576398178 |
| axon extension involved in axon guidance (GO:0048846) | 44600 | 0.287810755 | 0.576398178 |
| cellular response to hepatocyte growth factor stimulus (( | 44600 | 0.287810755 | 0.576398178 |
| chromatin silencing at telomere (GO:0006348) | 44600 | 0.287810755 | 0.576398178 |
| commissural neuron axon guidance (GO:0071679) | 44600 | 0.287810755 | 0.576398178 |
| cysteine metabolic process (GO:0006534) | 44600 | 0.287810755 | 0.576398178 |
| endonucleolytic cleavage in ITS1 to separate SSU-rRNA fr | 44600 | 0.287810755 | 0.576398178 |
| entry of bacterium into host cell (GO:0035635) | 44600 | 0.287810755 | 0.576398178 |
| establishment of protein localization to chromatin (GO:0( | 44600 | 0.287810755 | 0.576398178 |
| immunological synapse formation (GO:0001771) | 44600 | 0.287810755 | 0.576398178 |
| lymphatic endothelial cell differentiation (GO:0060836) | 44600 | 0.287810755 | 0.576398178 |
| mast cell activation involved in immune response (GO:00 | 44600 | 0.287810755 | 0.576398178 |
| mast cell degranulation (GO:0043303) | 44600 | 0.287810755 | 0.576398178 |
| meiotic sister chromatid cohesion (GO:0051177) | 44600 | 0.287810755 | 0.576398178 |
| monoamine transport (GO:0015844) | 44600 | 0.287810755 | 0.576398178 |
| myoblast migration (GO:0051451) | 44600 | 0.287810755 | 0.576398178 |
| natural killer cell differentiation (GO:0001779) | 44600 | 0.287810755 | 0.576398178 |
| negative regulation of complement activation, classical p | 44600 | 0.287810755 | 0.576398178 |
| negative regulation of keratinocyte proliferation (GO:001 | 44600 | 0.287810755 | 0.576398178 |
| negative regulation of microglial cell activation (GO:1903 | 44600 | 0.287810755 | 0.576398178 |
| negative regulation of muscle cell apoptotic process (GO: | 44600 | 0.287810755 | 0.576398178 |
| negative regulation of muscle cell differentiation (GO:00! | 44600 | 0.287810755 | 0.576398178 |

| | | | |
|---|---|---|---|
| negative regulation of osteoblast proliferation (GO:0033( | 44600 | 0.287810755 | 0.576398178 |
| negative regulation of phospholipase activity (GO:001051 | 44600 | 0.287810755 | 0.576398178 |
| negative regulation of response to reactive oxygen specie | 44600 | 0.287810755 | 0.576398178 |
| negative regulation of striated muscle cell apoptotic proc | 44600 | 0.287810755 | 0.576398178 |
| negative regulation of T cell mediated cytotoxicity (GO:0( | 44600 | 0.287810755 | 0.576398178 |
| neuron projection extension involved in neuron projectic | 44600 | 0.287810755 | 0.576398178 |
| neurotransmitter reuptake (GO:0098810) | 44600 | 0.287810755 | 0.576398178 |
| osteoblast development (GO:0002076) | 44600 | 0.287810755 | 0.576398178 |
| peptidyl-lysine deacetylation (GO:0034983) | 44600 | 0.287810755 | 0.576398178 |
| peptidyl-serine autophosphorylation (GO:0036289) | 44600 | 0.287810755 | 0.576398178 |
| phosphatidylinositol-3-phosphate biosynthetic process (( | 44600 | 0.287810755 | 0.576398178 |
| plasma membrane phospholipid scrambling (GO:001712: | 44600 | 0.287810755 | 0.576398178 |
| platelet activating factor metabolic process (GO:0046469 | 44600 | 0.287810755 | 0.576398178 |
| positive regulation of aspartic-type peptidase activity (GC | 44600 | 0.287810755 | 0.576398178 |
| positive regulation of astrocyte differentiation (GO:0048: | 44600 | 0.287810755 | 0.576398178 |
| positive regulation of cell migration by vascular endothel | 44600 | 0.287810755 | 0.576398178 |
| positive regulation of endocytic recycling (GO:2001137) | 44600 | 0.287810755 | 0.576398178 |
| positive regulation of isomerase activity (GO:0010912) | 44600 | 0.287810755 | 0.576398178 |
| positive regulation of lipoprotein lipase activity (GO:0051 | 44600 | 0.287810755 | 0.576398178 |
| positive regulation of membrane invagination (GO:19051 | 44600 | 0.287810755 | 0.576398178 |
| positive regulation of muscle contraction (GO:0045933) | 44600 | 0.287810755 | 0.576398178 |
| positive regulation of myeloid cell apoptotic process (GO | 44600 | 0.287810755 | 0.576398178 |
| positive regulation of myotube differentiation (GO:0010& | 44600 | 0.287810755 | 0.576398178 |
| positive regulation of oligodendrocyte differentiation (GC | 44600 | 0.287810755 | 0.576398178 |
| positive regulation of protein localization to early endosc | 44600 | 0.287810755 | 0.576398178 |
| positive regulation of protein localization to endosome (( | 44600 | 0.287810755 | 0.576398178 |
| positive regulation of steroid biosynthetic process (GO:0( | 44600 | 0.287810755 | 0.576398178 |
| positive regulation of vascular associated smooth muscle | 44600 | 0.287810755 | 0.576398178 |
| protein localization to microtubule (GO:0035372) | 44600 | 0.287810755 | 0.576398178 |
| protein localization to microtubule cytoskeleton (GO:007 | 44600 | 0.287810755 | 0.576398178 |
| proximal/distal pattern formation (GO:0009954) | 44600 | 0.287810755 | 0.576398178 |
| pyrimidine nucleobase catabolic process (GO:0006208) | 44600 | 0.287810755 | 0.576398178 |
| regulation of alpha-beta T cell differentiation (GO:00466: | 44600 | 0.287810755 | 0.576398178 |
| regulation of alpha-beta T cell proliferation (GO:0046640 | 44600 | 0.287810755 | 0.576398178 |
| regulation of ATPase-coupled calcium transmembrane tr | 44600 | 0.287810755 | 0.576398178 |
| regulation of CD4-positive, alpha-beta T cell activation (G | 44600 | 0.287810755 | 0.576398178 |
| regulation of Cdc42 protein signal transduction (GO:0032 | 44600 | 0.287810755 | 0.576398178 |
| regulation of chemokine-mediated signaling pathway (GC | 44600 | 0.287810755 | 0.576398178 |
| regulation of collateral sprouting (GO:0048670) | 44600 | 0.287810755 | 0.576398178 |
| regulation of dendritic spine maintenance (GO:1902950) | 44600 | 0.287810755 | 0.576398178 |
| regulation of endodeoxyribonuclease activity (GO:00320: | 44600 | 0.287810755 | 0.576398178 |
| regulation of intrinsic apoptotic signaling pathway by p53 | 44600 | 0.287810755 | 0.576398178 |
| regulation of metaphase/anaphase transition of cell cycle | 44600 | 0.287810755 | 0.576398178 |
| regulation of microvillus organization (GO:0032530) | 44600 | 0.287810755 | 0.576398178 |
| regulation of mitochondrial gene expression (GO:006212 | 44600 | 0.287810755 | 0.576398178 |
| regulation of positive chemotaxis (GO:0050926) | 44600 | 0.287810755 | 0.576398178 |
| regulation of protein localization to early endosome (GO: | 44600 | 0.287810755 | 0.576398178 |

| | | | |
|---|---|---|---|
| regulation of proton transport (GO:0010155) | 44600 | 0.287810755 | 0.576398178 |
| regulation of response to wounding (GO:1903034) | 44600 | 0.287810755 | 0.576398178 |
| regulation of translational termination (GO:0006449) | 44600 | 0.287810755 | 0.576398178 |
| response to hepatocyte growth factor (GO:0035728) | 44600 | 0.287810755 | 0.576398178 |
| response to hydroperoxide (GO:0033194) | 44600 | 0.287810755 | 0.576398178 |
| single strand break repair (GO:0000012) | 44600 | 0.287810755 | 0.576398178 |
| skeletal muscle tissue regeneration (GO:0043403) | 44600 | 0.287810755 | 0.576398178 |
| snRNA transcription by RNA polymerase III (GO:0042796) | 44600 | 0.287810755 | 0.576398178 |
| spleen development (GO:0048536) | 44600 | 0.287810755 | 0.576398178 |
| SREBP signaling pathway (GO:0032933) | 44600 | 0.287810755 | 0.576398178 |
| store-operated calcium entry (GO:0002115) | 44600 | 0.287810755 | 0.576398178 |
| vascular associated smooth muscle cell differentiation (G | 44600 | 0.287810755 | 0.576398178 |
| fibroblast growth factor receptor signaling pathway (GO:( | 27729 | 0.288184804 | 0.576924876 |
| positive regulation of Wnt signaling pathway (GO:003017 | 23/153 | 0.291806057 | 0.583949332 |
| cell-substrate junction assembly (GO:0007044) | 12571 | 0.29220214 | 0.584066993 |
| positive regulation of mitochondrial outer membrane pe | 12571 | 0.29220214 | 0.584066993 |
| transcription by RNA polymerase I (GO:0006360) | 12571 | 0.29220214 | 0.584066993 |
| import into cell (GO:0098657) | 15158 | 0.297481363 | 0.594162122 |
| regulation of protein dephosphorylation (GO:0035304) | 15158 | 0.297481363 | 0.594162122 |
| cognition (GO:0050890) | 17746 | 0.300781484 | 0.596645652 |
| Golgi to plasma membrane transport (GO:0006893) | 17746 | 0.300781484 | 0.596645652 |
| actin filament-based transport (GO:0099515) | 44672 | 0.300906951 | 0.596645652 |
| epithelial cell development (GO:0002064) | 44672 | 0.300906951 | 0.596645652 |
| linoleic acid metabolic process (GO:0043651) | 44672 | 0.300906951 | 0.596645652 |
| long-term synaptic potentiation (GO:0060291) | 44672 | 0.300906951 | 0.596645652 |
| mitotic spindle assembly checkpoint signaling (GO:00070 | 44672 | 0.300906951 | 0.596645652 |
| mitotic spindle checkpoint signaling (GO:0071174) | 44672 | 0.300906951 | 0.596645652 |
| negative regulation of peptidyl-serine phosphorylation (G | 44672 | 0.300906951 | 0.596645652 |
| neuron apoptotic process (GO:0051402) | 44672 | 0.300906951 | 0.596645652 |
| poly(A)+ mRNA export from nucleus (GO:0016973) | 44672 | 0.300906951 | 0.596645652 |
| positive regulation of transcription regulatory region DN/ | 44672 | 0.300906951 | 0.596645652 |
| regulation of cardiac muscle cell proliferation (GO:00600 | 44672 | 0.300906951 | 0.596645652 |
| regulation of dendritic spine development (GO:0060998) | 44672 | 0.300906951 | 0.596645652 |
| regulation of ubiquitin protein ligase activity (GO:190466 | 44672 | 0.300906951 | 0.596645652 |
| response to glucagon (GO:0033762) | 44672 | 0.300906951 | 0.596645652 |
| sensory perception of pain (GO:0019233) | 44672 | 0.300906951 | 0.596645652 |
| spindle assembly checkpoint signaling (GO:0071173) | 44672 | 0.300906951 | 0.596645652 |
| zinc ion transport (GO:0006829) | 44672 | 0.300906951 | 0.596645652 |
| regulation of insulin secretion (GO:0050796) | 16/104 | 0.301417515 | 0.597429985 |
| positive regulation of cold-induced thermogenesis (GO:0: | 15/97 | 0.302437748 | 0.599223531 |
| protein sumoylation (GO:0016925) | 20333 | 0.302778752 | 0.599670459 |
| regulation of mRNA splicing, via spliceosome (GO:004802 | 14/90 | 0.303293419 | 0.600460866 |
| cellular response to calcium ion (GO:0071277) | 25508 | 0.304331178 | 0.602056538 |
| cellular response to decreased oxygen levels (GO:003629 | 25508 | 0.304331178 | 0.602056538 |
| glycerophospholipid catabolic process (GO:0046475) | 44709 | 0.311034802 | 0.611360498 |
| glycogen metabolic process (GO:0005977) | 44709 | 0.311034802 | 0.611360498 |
| histone mRNA metabolic process (GO:0008334) | 44709 | 0.311034802 | 0.611360498 |

| | | | |
|---|---|---|---|
| keratan sulfate biosynthetic process (GO:0018146) | 44709 | 0.311034802 | 0.611360498 |
| negative regulation of phosphatase activity (GO:0010923 | 44709 | 0.311034802 | 0.611360498 |
| phagosome acidification (GO:0090383) | 44709 | 0.311034802 | 0.611360498 |
| positive regulation of cell communication (GO:0010647) | 44709 | 0.311034802 | 0.611360498 |
| positive regulation of fibroblast proliferation (GO:004814 | 44709 | 0.311034802 | 0.611360498 |
| protein localization to lysosome (GO:0061462) | 44709 | 0.311034802 | 0.611360498 |
| regulation of calcineurin-NFAT signaling cascade (GO:007 | 44709 | 0.311034802 | 0.611360498 |
| regulation of DNA-templated transcription, elongation (G | 44709 | 0.311034802 | 0.611360498 |
| regulation of extrinsic apoptotic signaling pathway in abs | 44709 | 0.311034802 | 0.611360498 |
| regulation of multicellular organismal development (GO:: | 44709 | 0.311034802 | 0.611360498 |
| regulation of phosphatase activity (GO:0010921) | 44709 | 0.311034802 | 0.611360498 |
| regulation of plasma membrane bounded cell projection | 44709 | 0.311034802 | 0.611360498 |
| RNA phosphodiester bond hydrolysis (GO:0090501) | 44709 | 0.311034802 | 0.611360498 |
| T cell activation involved in immune response (GO:00022 | 44709 | 0.311034802 | 0.611360498 |
| negative regulation of organelle assembly (GO:1902116) | 12936 | 0.316671621 | 0.619120341 |
| negative regulation of Ras protein signal transduction (G( | 12936 | 0.316671621 | 0.619120341 |
| regulation of autophagosome assembly (GO:2000785) | 12936 | 0.316671621 | 0.619120341 |
| regulation of mitotic spindle organization (GO:0060236) | 12936 | 0.316671621 | 0.619120341 |
| regulation of neuron apoptotic process (GO:0043523) | 15/98 | 0.316832268 | 0.619120341 |
| histone H3 acetylation (GO:0043966) | 15523 | 0.319816044 | 0.619120341 |
| phosphatidylinositol phosphate biosynthetic process (GO | 15523 | 0.319816044 | 0.619120341 |
| acute-phase response (GO:0006953) | 44635 | 0.319868912 | 0.619120341 |
| cellular response to ATP (GO:0071318) | 44635 | 0.319868912 | 0.619120341 |
| central nervous system neuron development (GO:00219! | 44635 | 0.319868912 | 0.619120341 |
| contractile actin filament bundle assembly (GO:0030038) | 44635 | 0.319868912 | 0.619120341 |
| endoderm development (GO:0007492) | 44635 | 0.319868912 | 0.619120341 |
| endoplasmic reticulum to cytosol transport (GO:1903513 | 44635 | 0.319868912 | 0.619120341 |
| establishment of spindle localization (GO:0051293) | 44635 | 0.319868912 | 0.619120341 |
| GTP metabolic process (GO:0046039) | 44635 | 0.319868912 | 0.619120341 |
| heterocycle biosynthetic process (GO:0018130) | 44635 | 0.319868912 | 0.619120341 |
| histone H4-K5 acetylation (GO:0043981) | 44635 | 0.319868912 | 0.619120341 |
| histone H4-K8 acetylation (GO:0043982) | 44635 | 0.319868912 | 0.619120341 |
| icosanoid biosynthetic process (GO:0046456) | 44635 | 0.319868912 | 0.619120341 |
| lipoxygenase pathway (GO:0019372) | 44635 | 0.319868912 | 0.619120341 |
| mRNA methylation (GO:0080009) | 44635 | 0.319868912 | 0.619120341 |
| negative regulation of endoplasmic reticulum stress-indu | 44635 | 0.319868912 | 0.619120341 |
| negative regulation of leukocyte mediated cytotoxicity (( | 44635 | 0.319868912 | 0.619120341 |
| nitric oxide biosynthetic process (GO:0006809) | 44635 | 0.319868912 | 0.619120341 |
| nucleobase-containing small molecule catabolic process ( | 44635 | 0.319868912 | 0.619120341 |
| peptidyl-tyrosine autophosphorylation (GO:0038083) | 44635 | 0.319868912 | 0.619120341 |
| phosphatidylethanolamine biosynthetic process (GO:000 | 44635 | 0.319868912 | 0.619120341 |
| positive regulation of chromosome organization (GO:200 | 44635 | 0.319868912 | 0.619120341 |
| positive regulation of nuclear-transcribed mRNA cataboli | 44635 | 0.319868912 | 0.619120341 |
| positive regulation of response to cytokine stimulus (GO: | 44635 | 0.319868912 | 0.619120341 |
| protein localization to cell-cell junction (GO:0150105) | 44635 | 0.319868912 | 0.619120341 |
| regulation of clathrin-dependent endocytosis (GO:20003( | 44635 | 0.319868912 | 0.619120341 |
| regulation of neuron projection arborization (GO:015001 | 44635 | 0.319868912 | 0.619120341 |

| | | | |
|---|---|---|---|
| regulation of nuclear-transcribed mRNA catabolic proces: | 44635 | 0.319868912 | 0.619120341 |
| regulation of secretion (GO:0051046) | 44635 | 0.319868912 | 0.619120341 |
| regulation of superoxide anion generation (GO:0032928) | 44635 | 0.319868912 | 0.619120341 |
| RNA phosphodiester bond hydrolysis, endonucleolytic (G | 44635 | 0.319868912 | 0.619120341 |
| stress fiber assembly (GO:0043149) | 44635 | 0.319868912 | 0.619120341 |
| synaptonemal complex assembly (GO:0007130) | 44635 | 0.319868912 | 0.619120341 |
| synaptonemal complex organization (GO:0070193) | 44635 | 0.319868912 | 0.619120341 |
| vitamin D metabolic process (GO:0042359) | 44635 | 0.319868912 | 0.619120341 |
| inorganic cation import across plasma membrane (GO:00 | 28460 | 0.320670285 | 0.620440273 |
| aminoglycan biosynthetic process (GO:0006023) | 18111 | 0.321440047 | 0.620937491 |
| negative regulation of cell projection organization (GO:0( | 18111 | 0.321440047 | 0.620937491 |
| cellular response to light stimulus (GO:0071482) | 25873 | 0.321524899 | 0.620937491 |
| positive regulation of cell-substrate adhesion (GO:00108: | 25873 | 0.321524899 | 0.620937491 |
| regulation of plasma membrane bounded cell projection | 25873 | 0.321524899 | 0.620937491 |
| positive regulation of DNA metabolic process (GO:00510! | 23285 | 0.322037098 | 0.621695549 |
| post-translational protein modification (GO:0043687) | 49/345 | 0.325199867 | 0.627568094 |
| sulfur compound biosynthetic process (GO:0044272) | 17/113 | 0.327218677 | 0.631229494 |
| nuclear-transcribed mRNA catabolic process (GO:000095 | 25/171 | 0.332208729 | 0.634311188 |
| amino acid import (GO:0043090) | 44673 | 0.332703675 | 0.634311188 |
| heme biosynthetic process (GO:0006783) | 44673 | 0.332703675 | 0.634311188 |
| membrane lipid metabolic process (GO:0006643) | 44673 | 0.332703675 | 0.634311188 |
| mitochondrial calcium ion homeostasis (GO:0051560) | 44673 | 0.332703675 | 0.634311188 |
| natural killer cell mediated cytotoxicity (GO:0042267) | 44673 | 0.332703675 | 0.634311188 |
| negative regulation of cyclin-dependent protein kinase a( | 44673 | 0.332703675 | 0.634311188 |
| negative regulation of DNA recombination at telomere (( | 44673 | 0.332703675 | 0.634311188 |
| negative regulation of mitotic metaphase/anaphase tran: | 44673 | 0.332703675 | 0.634311188 |
| positive regulation of axon extension (GO:0045773) | 44673 | 0.332703675 | 0.634311188 |
| positive regulation of lipid catabolic process (GO:005099( | 44673 | 0.332703675 | 0.634311188 |
| regulation of centrosome cycle (GO:0046605) | 44673 | 0.332703675 | 0.634311188 |
| regulation of DNA recombination at telomere (GO:00726 | 44673 | 0.332703675 | 0.634311188 |
| regulation of macrophage chemotaxis (GO:0010758) | 44673 | 0.332703675 | 0.634311188 |
| ubiquitin-dependent protein catabolic process via the mu | 44673 | 0.332703675 | 0.634311188 |
| zinc ion transmembrane transport (GO:0071577) | 44673 | 0.332703675 | 0.634311188 |
| beta-catenin-TCF complex assembly (GO:1904837) | 44710 | 0.338684735 | 0.634311188 |
| dicarboxylic acid transport (GO:0006835) | 44710 | 0.338684735 | 0.634311188 |
| glycolytic process (GO:0006096) | 44710 | 0.338684735 | 0.634311188 |
| lamellipodium organization (GO:0097581) | 44710 | 0.338684735 | 0.634311188 |
| negative regulation of myeloid leukocyte differentiation ( | 44710 | 0.338684735 | 0.634311188 |
| regulation of gluconeogenesis (GO:0006111) | 44710 | 0.338684735 | 0.634311188 |
| regulation of protein targeting to membrane (GO:009031 | 44710 | 0.338684735 | 0.634311188 |
| regulation of ubiquitin-protein transferase activity (GO:0( | 44710 | 0.338684735 | 0.634311188 |
| nucleic acid metabolic process (GO:0090304) | 26238 | 0.338925095 | 0.634311188 |
| adenosine metabolic process (GO:0046085) | 44601 | 0.339805204 | 0.634311188 |
| alpha-amino acid biosynthetic process (GO:1901607) | 44601 | 0.339805204 | 0.634311188 |
| amyloid-beta formation (GO:0034205) | 44601 | 0.339805204 | 0.634311188 |
| anoikis (GO:0043276) | 44601 | 0.339805204 | 0.634311188 |
| astral microtubule organization (GO:0030953) | 44601 | 0.339805204 | 0.634311188 |

| | | | |
|---|---|---|---|
| basement membrane assembly (GO:0070831) | 44601 | 0.339805204 | 0.634311188 |
| bone cell development (GO:0098751) | 44601 | 0.339805204 | 0.634311188 |
| cellular response to forskolin (GO:1904322) | 44601 | 0.339805204 | 0.634311188 |
| cellular response to interleukin-17 (GO:0097398) | 44601 | 0.339805204 | 0.634311188 |
| cellular response to sterol depletion (GO:0071501) | 44601 | 0.339805204 | 0.634311188 |
| complement activation, classical pathway (GO:0006958) | 44601 | 0.339805204 | 0.634311188 |
| embryonic digestive tract morphogenesis (GO:0048557) | 44601 | 0.339805204 | 0.634311188 |
| epithelial cell apoptotic process (GO:1904019) | 44601 | 0.339805204 | 0.634311188 |
| epithelial to mesenchymal transition involved in endocar | 44601 | 0.339805204 | 0.634311188 |
| glycerolipid biosynthetic process (GO:0045017) | 44601 | 0.339805204 | 0.634311188 |
| inositol metabolic process (GO:0006020) | 44601 | 0.339805204 | 0.634311188 |
| interleukin-17-mediated signaling pathway (GO:0097400 | 44601 | 0.339805204 | 0.634311188 |
| intestinal epithelial cell differentiation (GO:0060575) | 44601 | 0.339805204 | 0.634311188 |
| L-ascorbic acid metabolic process (GO:0019852) | 44601 | 0.339805204 | 0.634311188 |
| lipoprotein metabolic process (GO:0042157) | 44601 | 0.339805204 | 0.634311188 |
| mast cell mediated immunity (GO:0002448) | 44601 | 0.339805204 | 0.634311188 |
| mitochondrial respirasome assembly (GO:0097250) | 44601 | 0.339805204 | 0.634311188 |
| negative regulation of exocytosis (GO:0045920) | 44601 | 0.339805204 | 0.634311188 |
| negative regulation of leukocyte proliferation (GO:00706 | 44601 | 0.339805204 | 0.634311188 |
| negative regulation of lipase activity (GO:0060192) | 44601 | 0.339805204 | 0.634311188 |
| negative regulation of nitric oxide biosynthetic process (G | 44601 | 0.339805204 | 0.634311188 |
| negative regulation of nitric oxide metabolic process (GO | 44601 | 0.339805204 | 0.634311188 |
| negative regulation of pathway-restricted SMAD protein | 44601 | 0.339805204 | 0.634311188 |
| negative regulation of platelet aggregation (GO:0090331) | 44601 | 0.339805204 | 0.634311188 |
| negative regulation of retrograde protein transport, ER to | 44601 | 0.339805204 | 0.634311188 |
| negative regulation of systemic arterial blood pressure (G | 44601 | 0.339805204 | 0.634311188 |
| negative regulation of T cell cytokine production (GO:000 | 44601 | 0.339805204 | 0.634311188 |
| negative regulation of ubiquitin protein ligase activity (GO | 44601 | 0.339805204 | 0.634311188 |
| nuclear RNA surveillance (GO:0071027) | 44601 | 0.339805204 | 0.634311188 |
| nucleoside diphosphate catabolic process (GO:0009134) | 44601 | 0.339805204 | 0.634311188 |
| organic cyclic compound catabolic process (GO:1901361) | 44601 | 0.339805204 | 0.634311188 |
| ornithine metabolic process (GO:0006591) | 44601 | 0.339805204 | 0.634311188 |
| peripheral nervous system axon ensheathment (GO:0032 | 44601 | 0.339805204 | 0.634311188 |
| plasminogen activation (GO:0031639) | 44601 | 0.339805204 | 0.634311188 |
| positive regulation of ATP biosynthetic process (GO:2001 | 44601 | 0.339805204 | 0.634311188 |
| positive regulation of blood vessel endothelial cell prolife | 44601 | 0.339805204 | 0.634311188 |
| positive regulation of cell-cell adhesion mediated by cadh | 44601 | 0.339805204 | 0.634311188 |
| positive regulation of chromatin binding (GO:0035563) | 44601 | 0.339805204 | 0.634311188 |
| positive regulation of inositol phosphate biosynthetic pro | 44601 | 0.339805204 | 0.634311188 |
| positive regulation of ligase activity (GO:0051351) | 44601 | 0.339805204 | 0.634311188 |
| positive regulation of mast cell degranulation (GO:00433 | 44601 | 0.339805204 | 0.634311188 |
| positive regulation of sequestering of triglyceride (GO:00 | 44601 | 0.339805204 | 0.634311188 |
| positive regulation of triglyceride lipase activity (GO:0061 | 44601 | 0.339805204 | 0.634311188 |
| purine nucleobase metabolic process (GO:0006144) | 44601 | 0.339805204 | 0.634311188 |
| regulation of actin filament organization (GO:0110053) | 44601 | 0.339805204 | 0.634311188 |
| regulation of aspartic-type endopeptidase activity involve | 44601 | 0.339805204 | 0.634311188 |
| regulation of CD8-positive, alpha-beta T cell activation (G | 44601 | 0.339805204 | 0.634311188 |

| | | | |
|---|---|---|---|
| regulation of complement activation, classical pathway (( | 44601 | 0.339805204 | 0.634311188 |
| regulation of complement-dependent cytotoxicity (GO:19 | 44601 | 0.339805204 | 0.634311188 |
| regulation of ER-associated ubiquitin-dependent protein | 44601 | 0.339805204 | 0.634311188 |
| regulation of fertilization (GO:0080154) | 44601 | 0.339805204 | 0.634311188 |
| regulation of transmembrane transporter activity (GO:00 | 44601 | 0.339805204 | 0.634311188 |
| response to forskolin (GO:1904321) | 44601 | 0.339805204 | 0.634311188 |
| response to UV-C (GO:0010225) | 44601 | 0.339805204 | 0.634311188 |
| Schwann cell development (GO:0014044) | 44601 | 0.339805204 | 0.634311188 |
| sensory organ morphogenesis (GO:0090596) | 44601 | 0.339805204 | 0.634311188 |
| small RNA loading onto RISC (GO:0070922) | 44601 | 0.339805204 | 0.634311188 |
| somatic recombination of immunoglobulin genes involve | 44601 | 0.339805204 | 0.634311188 |
| synapse pruning (GO:0098883) | 44601 | 0.339805204 | 0.634311188 |
| tetrahydrofolate interconversion (GO:0035999) | 44601 | 0.339805204 | 0.634311188 |
| lipid homeostasis (GO:0055088) | 23651 | 0.340415557 | 0.634994189 |
| protein catabolic process (GO:0030163) | 23651 | 0.340415557 | 0.634994189 |
| endoderm formation (GO:0001706) | 13302 | 0.341434435 | 0.635081907 |
| negative regulation of cellular protein localization (GO:19 | 13302 | 0.341434435 | 0.635081907 |
| Wnt signaling pathway, calcium modulating pathway (GC | 13302 | 0.341434435 | 0.635081907 |
| arachidonic acid metabolic process (GO:0019369) | 21064 | 0.341603203 | 0.635081907 |
| positive regulation of cell junction assembly (GO:190189( | 21064 | 0.341603203 | 0.635081907 |
| regulation of mitotic nuclear division (GO:0007088) | 21064 | 0.341603203 | 0.635081907 |
| amino acid transport (GO:0006865) | 18476 | 0.342346433 | 0.635081907 |
| organic hydroxy compound biosynthetic process (GO:190 | 18476 | 0.342346433 | 0.635081907 |
| positive regulation of cell morphogenesis involved in diffe | 18476 | 0.342346433 | 0.635081907 |
| cellular glucose homeostasis (GO:0001678) | 15888 | 0.34241856 | 0.635081907 |
| cellular response to retinoic acid (GO:0071300) | 15888 | 0.34241856 | 0.635081907 |
| DNA synthesis involved in DNA repair (GO:0000731) | 15888 | 0.34241856 | 0.635081907 |
| natural killer cell activation (GO:0030101) | 15888 | 0.34241856 | 0.635081907 |
| negative regulation of proteolysis (GO:0045861) | 15888 | 0.34241856 | 0.635081907 |
| positive regulation of phospholipase C activity (GO:0010{ | 15888 | 0.34241856 | 0.635081907 |
| substantia nigra development (GO:0021762) | 15888 | 0.34241856 | 0.635081907 |
| cell morphogenesis involved in differentiation (GO:00009 | 26604 | 0.356492872 | 0.657476948 |
| positive regulation of neurogenesis (GO:0050769) | 26604 | 0.356492872 | 0.657476948 |
| positive regulation of protein secretion (GO:0050714) | 26604 | 0.356492872 | 0.657476948 |
| 3'-UTR-mediated mRNA destabilization (GO:0061158) | 44636 | 0.358416692 | 0.657476948 |
| apoptotic DNA fragmentation (GO:0006309) | 44636 | 0.358416692 | 0.657476948 |
| autophagy of nucleus (GO:0044804) | 44636 | 0.358416692 | 0.657476948 |
| cellular response to corticosteroid stimulus (GO:0071384 | 44636 | 0.358416692 | 0.657476948 |
| cellular response to sterol (GO:0036315) | 44636 | 0.358416692 | 0.657476948 |
| ceramide catabolic process (GO:0046514) | 44636 | 0.358416692 | 0.657476948 |
| epiboly involved in wound healing (GO:0090505) | 44636 | 0.358416692 | 0.657476948 |
| glycosphingolipid biosynthetic process (GO:0006688) | 44636 | 0.358416692 | 0.657476948 |
| Golgi to endosome transport (GO:0006895) | 44636 | 0.358416692 | 0.657476948 |
| male meiosis I (GO:0007141) | 44636 | 0.358416692 | 0.657476948 |
| membrane repolarization (GO:0086009) | 44636 | 0.358416692 | 0.657476948 |
| mitotic sister chromatid cohesion (GO:0007064) | 44636 | 0.358416692 | 0.657476948 |
| motor neuron axon guidance (GO:0008045) | 44636 | 0.358416692 | 0.657476948 |

| | | | |
|---|---|---|---|
| negative regulation of cell-substrate junction organizatio | 44636 | 0.358416692 | 0.657476948 |
| negative regulation of focal adhesion assembly (GO:0051 | 44636 | 0.358416692 | 0.657476948 |
| neuron cell-cell adhesion (GO:0007158) | 44636 | 0.358416692 | 0.657476948 |
| nitric oxide metabolic process (GO:0046209) | 44636 | 0.358416692 | 0.657476948 |
| nucleoside diphosphate metabolic process (GO:0009132) | 44636 | 0.358416692 | 0.657476948 |
| positive regulation by host of viral transcription (GO:004 | 44636 | 0.358416692 | 0.657476948 |
| protein localization to microtubule organizing center (GO | 44636 | 0.358416692 | 0.657476948 |
| purine ribonucleoside monophosphate biosynthetic proc | 44636 | 0.358416692 | 0.657476948 |
| regulation of ATP biosynthetic process (GO:2001169) | 44636 | 0.358416692 | 0.657476948 |
| regulation of chromatin silencing (GO:0031935) | 44636 | 0.358416692 | 0.657476948 |
| regulation of ERBB signaling pathway (GO:1901184) | 44636 | 0.358416692 | 0.657476948 |
| regulation of interleukin-13 production (GO:0032656) | 44636 | 0.358416692 | 0.657476948 |
| regulation of mitotic sister chromatid separation (GO:001 | 44636 | 0.358416692 | 0.657476948 |
| regulation of neurotransmitter transport (GO:0051588) | 44636 | 0.358416692 | 0.657476948 |
| regulation of Rac protein signal transduction (GO:003502 | 44636 | 0.358416692 | 0.657476948 |
| chemical homeostasis (GO:0048878) | 24016 | 0.358966188 | 0.658020236 |
| regulation of cell-matrix adhesion (GO:0001952) | 24016 | 0.358966188 | 0.658020236 |
| cellular iron ion homeostasis (GO:0006879) | 21429 | 0.361307785 | 0.661612246 |
| membrane lipid biosynthetic process (GO:0046467) | 21429 | 0.361307785 | 0.661612246 |
| negative regulation of neuron projection development (G | 21429 | 0.361307785 | 0.661612246 |
| regulation of primary metabolic process (GO:0080090) | 19/130 | 0.361845566 | 0.662363537 |
| cholesterol transport (GO:0030301) | 18841 | 0.363433161 | 0.664666745 |
| inositol lipid-mediated signaling (GO:0048017) | 18841 | 0.363433161 | 0.664666745 |
| negative regulation of cellular protein metabolic process | 14/94 | 0.364172339 | 0.664666745 |
| DNA catabolic process, endonucleolytic (GO:0000737) | 44674 | 0.364639119 | 0.664666745 |
| negative regulation of extrinsic apoptotic signaling pathw | 44674 | 0.364639119 | 0.664666745 |
| negative regulation of signal transduction in absence of li | 44674 | 0.364639119 | 0.664666745 |
| nucleotide metabolic process (GO:0009117) | 44674 | 0.364639119 | 0.664666745 |
| positive regulation of natural killer cell mediated immuni | 44674 | 0.364639119 | 0.664666745 |
| positive regulation of protein autophosphorylation (GO:0 | 44674 | 0.364639119 | 0.664666745 |
| positive regulation of receptor internalization (GO:00020 | 44674 | 0.364639119 | 0.664666745 |
| positive regulation of transcription by RNA polymerase I ( | 44674 | 0.364639119 | 0.664666745 |
| vesicle transport along microtubule (GO:0047496) | 44674 | 0.364639119 | 0.664666745 |
| cation transmembrane transport (GO:0098655) | 16254 | 0.365203634 | 0.665462251 |
| lysosome localization (GO:0032418) | 13667 | 0.366378424 | 0.665500934 |
| neural crest cell migration (GO:0001755) | 13667 | 0.366378424 | 0.665500934 |
| regulation of actin cytoskeleton reorganization (GO:2000 | 13667 | 0.366378424 | 0.665500934 |
| regulation of microtubule cytoskeleton organization (GO: | 13667 | 0.366378424 | 0.665500934 |
| mRNA splice site selection (GO:0006376) | 44711 | 0.366505904 | 0.665500934 |
| multivesicular body assembly (GO:0036258) | 44711 | 0.366505904 | 0.665500934 |
| regulation of protein localization to membrane (GO:1905 | 44711 | 0.366505904 | 0.665500934 |
| regulation of striated muscle contraction (GO:0006942) | 44711 | 0.366505904 | 0.665500934 |
| response to alcohol (GO:0097305) | 44711 | 0.366505904 | 0.665500934 |
| zinc ion homeostasis (GO:0055069) | 44711 | 0.366505904 | 0.665500934 |
| cytosolic transport (GO:0016482) | 17/116 | 0.368669676 | 0.669196006 |
| insulin receptor signaling pathway (GO:0008286) | 26969 | 0.374189414 | 0.678740923 |
| neurotransmitter transport (GO:0006836) | 26969 | 0.374189414 | 0.678740923 |

| | | | |
|---|---|---|---|
| cell-cell junction assembly (GO:0007043) | 24381 | 0.377643442 | 0.68476708 |
| glycolipid metabolic process (GO:0006664) | 19207 | 0.384633954 | 0.690090587 |
| regulation of exocytosis (GO:0017157) | 19207 | 0.384633954 | 0.690090587 |
| cellular response to radiation (GO:0071478) | 16619 | 0.388088222 | 0.690090587 |
| neural crest cell development (GO:0014032) | 16619 | 0.388088222 | 0.690090587 |
| positive regulation of nervous system development (GO:( | 16619 | 0.388088222 | 0.690090587 |
| adrenal gland development (GO:0030325) | 44602 | 0.390542122 | 0.690090587 |
| calcium import into the mitochondrion (GO:0036444) | 44602 | 0.390542122 | 0.690090587 |
| cellular response to acidic pH (GO:0071468) | 44602 | 0.390542122 | 0.690090587 |
| cellular response to oxygen levels (GO:0071453) | 44602 | 0.390542122 | 0.690090587 |
| cellular response to prostaglandin stimulus (GO:0071379 | 44602 | 0.390542122 | 0.690090587 |
| chloride ion homeostasis (GO:0055064) | 44602 | 0.390542122 | 0.690090587 |
| chondroitin sulfate proteoglycan metabolic process (GO:( | 44602 | 0.390542122 | 0.690090587 |
| complement activation, lectin pathway (GO:0001867) | 44602 | 0.390542122 | 0.690090587 |
| defense response to tumor cell (GO:0002357) | 44602 | 0.390542122 | 0.690090587 |
| ectoderm development (GO:0007398) | 44602 | 0.390542122 | 0.690090587 |
| endothelium development (GO:0003158) | 44602 | 0.390542122 | 0.690090587 |
| ether lipid biosynthetic process (GO:0008611) | 44602 | 0.390542122 | 0.690090587 |
| formation of translation preinitiation complex (GO:00017 | 44602 | 0.390542122 | 0.690090587 |
| fructose 6-phosphate metabolic process (GO:0006002) | 44602 | 0.390542122 | 0.690090587 |
| genetic imprinting (GO:0071514) | 44602 | 0.390542122 | 0.690090587 |
| L-glutamate transmembrane transport (GO:0015813) | 44602 | 0.390542122 | 0.690090587 |
| L-serine metabolic process (GO:0006563) | 44602 | 0.390542122 | 0.690090587 |
| lactation (GO:0007595) | 44602 | 0.390542122 | 0.690090587 |
| lipid droplet formation (GO:0140042) | 44602 | 0.390542122 | 0.690090587 |
| lipid phosphorylation (GO:0046834) | 44602 | 0.390542122 | 0.690090587 |
| lipoprotein transport (GO:0042953) | 44602 | 0.390542122 | 0.690090587 |
| membrane lipid catabolic process (GO:0046466) | 44602 | 0.390542122 | 0.690090587 |
| microtubule anchoring at centrosome (GO:0034454) | 44602 | 0.390542122 | 0.690090587 |
| modification-dependent macromolecule catabolic proces | 44602 | 0.390542122 | 0.690090587 |
| monovalent inorganic anion homeostasis (GO:0055083) | 44602 | 0.390542122 | 0.690090587 |
| negative regulation of cAMP-mediated signaling (GO:004 | 44602 | 0.390542122 | 0.690090587 |
| negative regulation of cell adhesion mediated by integrin | 44602 | 0.390542122 | 0.690090587 |
| negative regulation of cellular protein catabolic process ( | 44602 | 0.390542122 | 0.690090587 |
| negative regulation of circadian rhythm (GO:0042754) | 44602 | 0.390542122 | 0.690090587 |
| negative regulation of glycolytic process (GO:0045820) | 44602 | 0.390542122 | 0.690090587 |
| negative regulation of inclusion body assembly (GO:0090 | 44602 | 0.390542122 | 0.690090587 |
| negative regulation of leukocyte migration (GO:0002686) | 44602 | 0.390542122 | 0.690090587 |
| negative regulation of lymphocyte apoptotic process (GO | 44602 | 0.390542122 | 0.690090587 |
| negative regulation of purine nucleotide metabolic proce | 44602 | 0.390542122 | 0.690090587 |
| negative regulation of sodium ion transmembrane transp | 44602 | 0.390542122 | 0.690090587 |
| negative regulation of sodium ion transmembrane transp | 44602 | 0.390542122 | 0.690090587 |
| negative regulation of telomerase activity (GO:0051974) | 44602 | 0.390542122 | 0.690090587 |
| negative regulation of viral-induced cytoplasmic pattern r | 44602 | 0.390542122 | 0.690090587 |
| nerve growth factor signaling pathway (GO:0038180) | 44602 | 0.390542122 | 0.690090587 |
| neuron projection maintenance (GO:1990535) | 44602 | 0.390542122 | 0.690090587 |
| nuclear mRNA surveillance (GO:0071028) | 44602 | 0.390542122 | 0.690090587 |

| | | | |
|---|---|---|---|
| peptidyl-amino acid modification (GO:0018193) | 44602 | 0.390542122 | 0.690090587 |
| phagosome-lysosome fusion (GO:0090385) | 44602 | 0.390542122 | 0.690090587 |
| positive regulation of behavior (GO:0048520) | 44602 | 0.390542122 | 0.690090587 |
| positive regulation of epidermis development (GO:00456 | 44602 | 0.390542122 | 0.690090587 |
| positive regulation of mesenchymal cell proliferation (GC | 44602 | 0.390542122 | 0.690090587 |
| positive regulation of protein sumoylation (GO:0033235) | 44602 | 0.390542122 | 0.690090587 |
| positive regulation of receptor recycling (GO:0001921) | 44602 | 0.390542122 | 0.690090587 |
| positive regulation of RIG-I signaling pathway (GO:19002 | 44602 | 0.390542122 | 0.690090587 |
| positive regulation of rRNA processing (GO:2000234) | 44602 | 0.390542122 | 0.690090587 |
| positive regulation of transcription by RNA polymerase II | 44602 | 0.390542122 | 0.690090587 |
| presynaptic membrane assembly (GO:0097105) | 44602 | 0.390542122 | 0.690090587 |
| primary miRNA processing (GO:0031053) | 44602 | 0.390542122 | 0.690090587 |
| protein localization to endosome (GO:0036010) | 44602 | 0.390542122 | 0.690090587 |
| protein O-linked glycosylation via serine (GO:0018242) | 44602 | 0.390542122 | 0.690090587 |
| protein prenylation (GO:0018342) | 44602 | 0.390542122 | 0.690090587 |
| Rap protein signal transduction (GO:0032486) | 44602 | 0.390542122 | 0.690090587 |
| regulation of DNA methylation (GO:0044030) | 44602 | 0.390542122 | 0.690090587 |
| regulation of hair cycle (GO:0042634) | 44602 | 0.390542122 | 0.690090587 |
| regulation of histone phosphorylation (GO:0033127) | 44602 | 0.390542122 | 0.690090587 |
| regulation of long-term synaptic depression (GO:190045 | 44602 | 0.390542122 | 0.690090587 |
| regulation of protein neddylation (GO:2000434) | 44602 | 0.390542122 | 0.690090587 |
| regulation of receptor binding (GO:1900120) | 44602 | 0.390542122 | 0.690090587 |
| regulation of store-operated calcium entry (GO:2001256) | 44602 | 0.390542122 | 0.690090587 |
| regulation of urine volume (GO:0035809) | 44602 | 0.390542122 | 0.690090587 |
| removal of superoxide radicals (GO:0019430) | 44602 | 0.390542122 | 0.690090587 |
| rRNA pseudouridine synthesis (GO:0031118) | 44602 | 0.390542122 | 0.690090587 |
| sequestering of actin monomers (GO:0042989) | 44602 | 0.390542122 | 0.690090587 |
| smooth muscle tissue development (GO:0048745) | 44602 | 0.390542122 | 0.690090587 |
| urea cycle (GO:0000050) | 44602 | 0.390542122 | 0.690090587 |
| lymphocyte proliferation (GO:0046651) | 14032 | 0.391395666 | 0.690659133 |
| negative regulation of proteasomal protein catabolic pro | 14032 | 0.391395666 | 0.690659133 |
| positive regulation of insulin secretion (GO:0032024) | 14032 | 0.391395666 | 0.690659133 |
| regulation of phosphoprotein phosphatase activity (GO:0 | 14032 | 0.391395666 | 0.690659133 |
| 'de novo' posttranslational protein folding (GO:0051084) | 44712 | 0.39435065 | 0.691439491 |
| cellular response to cAMP (GO:0071320) | 44712 | 0.39435065 | 0.691439491 |
| negative regulation of axon extension (GO:0030517) | 44712 | 0.39435065 | 0.691439491 |
| negative regulation of proteasomal ubiquitin-dependent | 44712 | 0.39435065 | 0.691439491 |
| positive regulation of Rho protein signal transduction (GC | 44712 | 0.39435065 | 0.691439491 |
| regulation of developmental growth (GO:0048638) | 44712 | 0.39435065 | 0.691439491 |
| vitamin transport (GO:0051180) | 44712 | 0.39435065 | 0.691439491 |
| cellular transition metal ion homeostasis (GO:0046916) | 14/96 | 0.39533073 | 0.691439491 |
| apoptotic nuclear changes (GO:0030262) | 44675 | 0.396494953 | 0.691439491 |
| axon extension (GO:0048675) | 44675 | 0.396494953 | 0.691439491 |
| endocrine system development (GO:0035270) | 44675 | 0.396494953 | 0.691439491 |
| extracellular matrix assembly (GO:0085029) | 44675 | 0.396494953 | 0.691439491 |
| negative regulation of protein depolymerization (GO:190 | 44675 | 0.396494953 | 0.691439491 |
| negative regulation of protein tyrosine kinase activity (GC | 44675 | 0.396494953 | 0.691439491 |

| | | | |
|---|---|---|---|
| porphyrin-containing compound biosynthetic process (GO | 44675 | 0.396494953 | 0.691439491 |
| positive regulation of lamellipodium assembly (GO:0010 | 44675 | 0.396494953 | 0.691439491 |
| protein alpha-1,2-demannosylation (GO:0036508) | 44675 | 0.396494953 | 0.691439491 |
| purine nucleotide metabolic process (GO:0006163) | 44675 | 0.396494953 | 0.691439491 |
| response to gamma radiation (GO:0010332) | 44675 | 0.396494953 | 0.691439491 |
| activation of protein kinase A activity (GO:0034199) | 44637 | 0.39662939 | 0.691439491 |
| cell communication by electrical coupling involved in carc | 44637 | 0.39662939 | 0.691439491 |
| cell surface receptor signaling pathway involved in heart | 44637 | 0.39662939 | 0.691439491 |
| cellular modified amino acid catabolic process (GO:0042 | 44637 | 0.39662939 | 0.691439491 |
| copper ion homeostasis (GO:0055070) | 44637 | 0.39662939 | 0.691439491 |
| histone H2A monoubiquitination (GO:0035518) | 44637 | 0.39662939 | 0.691439491 |
| membrane depolarization during cardiac muscle cell actio | 44637 | 0.39662939 | 0.691439491 |
| monosaccharide transmembrane transport (GO:0015749 | 44637 | 0.39662939 | 0.691439491 |
| negative regulation of insulin secretion (GO:0046676) | 44637 | 0.39662939 | 0.691439491 |
| protein autoprocessing (GO:0016540) | 44637 | 0.39662939 | 0.691439491 |
| regulation of neuroblast proliferation (GO:1902692) | 44637 | 0.39662939 | 0.691439491 |
| regulation of receptor recycling (GO:0001919) | 44637 | 0.39662939 | 0.691439491 |
| regulation of steroid biosynthetic process (GO:0050810) | 44637 | 0.39662939 | 0.691439491 |
| regulation of transferase activity (GO:0051338) | 44637 | 0.39662939 | 0.691439491 |
| reverse cholesterol transport (GO:0043691) | 44637 | 0.39662939 | 0.691439491 |
| stabilization of membrane potential (GO:0030322) | 44637 | 0.39662939 | 0.691439491 |
| stem cell development (GO:0048864) | 44637 | 0.39662939 | 0.691439491 |
| neuron projection morphogenesis (GO:0048812) | 20/140 | 0.397145089 | 0.692106253 |
| regulation of cell motility (GO:2000145) | 19/133 | 0.401543528 | 0.699536764 |
| heart morphogenesis (GO:0003007) | 19572 | 0.40588425 | 0.706624892 |
| positive regulation of macroautophagy (GO:0016239) | 19572 | 0.40588425 | 0.706624892 |
| regulation of hematopoietic progenitor cell differentiatio | 27699 | 0.409815375 | 0.713229773 |
| alpha-amino acid metabolic process (GO:1901605) | 16984 | 0.410992217 | 0.714333221 |
| spliceosomal complex assembly (GO:0000245) | 16984 | 0.410992217 | 0.714333221 |
| vesicle organization (GO:0016050) | 16984 | 0.410992217 | 0.714333221 |
| protein maturation (GO:0051604) | 14/97 | 0.410999422 | 0.714333221 |
| sodium ion transport (GO:0006814) | 13/90 | 0.416233125 | 0.722723676 |
| positive regulation of intracellular transport (GO:003238 | 14397 | 0.416383438 | 0.722723676 |
| regulation of protein tyrosine kinase activity (GO:006109 | 14397 | 0.416383438 | 0.722723676 |
| vesicle cytoskeletal trafficking (GO:0099518) | 14397 | 0.416383438 | 0.722723676 |
| regulation of endocytosis (GO:0030100) | 22525 | 0.420942757 | 0.729202401 |
| heart development (GO:0007507) | 23/164 | 0.421020885 | 0.729202401 |
| glycoprotein biosynthetic process (GO:0009101) | 30651 | 0.421772819 | 0.729202401 |
| long-chain fatty acid metabolic process (GO:0001676) | 30651 | 0.421772819 | 0.729202401 |
| establishment or maintenance of epithelial cell apical/ba | 11810 | 0.422081159 | 0.729202401 |
| liver development (GO:0001889) | 11810 | 0.422081159 | 0.729202401 |
| metal ion export (GO:0070839) | 11810 | 0.422081159 | 0.729202401 |
| negative regulation of developmental growth (GO:00486 | 11810 | 0.422081159 | 0.729202401 |
| negative regulation of intracellular steroid hormone rece | 11810 | 0.422081159 | 0.729202401 |
| regulation of cell migration involved in sprouting angioge | 11810 | 0.422081159 | 0.729202401 |
| regulation of transcription involved in G1/S transition of | 11810 | 0.422081159 | 0.729202401 |
| response to amino acid (GO:0043200) | 11810 | 0.422081159 | 0.729202401 |

| | | | |
|---|---|---|---|
| RNA biosynthetic process (GO:0032774) | 11810 | 0.422081159 | 0.729202401 |
| transcription initiation from RNA polymerase I promoter | 11810 | 0.422081159 | 0.729202401 |
| negative regulation of neuron death (GO:1901215) | 14/98 | 0.426693943 | 0.736615835 |
| glycosaminoglycan metabolic process (GO:0030203) | 19937 | 0.427121669 | 0.736615835 |
| mitotic cell cycle phase transition (GO:0044772) | 29/209 | 0.427385276 | 0.736615835 |
| cellular polysaccharide biosynthetic process (GO:003369: | 44676 | 0.428073764 | 0.736615835 |
| divalent inorganic cation homeostasis (GO:0072507) | 44676 | 0.428073764 | 0.736615835 |
| fat-soluble vitamin metabolic process (GO:0006775) | 44676 | 0.428073764 | 0.736615835 |
| in utero embryonic development (GO:0001701) | 44676 | 0.428073764 | 0.736615835 |
| negative regulation of ion transport (GO:0043271) | 44676 | 0.428073764 | 0.736615835 |
| regulation of calcium ion import (GO:0090279) | 44676 | 0.428073764 | 0.736615835 |
| regulation of microtubule depolymerization (GO:003111 | 44676 | 0.428073764 | 0.736615835 |
| regulation of mitochondrial fission (GO:0090140) | 44676 | 0.428073764 | 0.736615835 |
| regulation of spindle assembly (GO:0090169) | 44676 | 0.428073764 | 0.736615835 |
| glycosphingolipid metabolic process (GO:0006687) | 17349 | 0.43383905 | 0.738800143 |
| cellular response to pH (GO:0071467) | 44638 | 0.434193959 | 0.738800143 |
| endothelial cell proliferation (GO:0001935) | 44638 | 0.434193959 | 0.738800143 |
| phosphatidylglycerol acyl-chain remodeling (GO:003614٤ | 44638 | 0.434193959 | 0.738800143 |
| phosphatidylinositol acyl-chain remodeling (GO:0036149 | 44638 | 0.434193959 | 0.738800143 |
| positive regulation of cell migration involved in sprouting | 44638 | 0.434193959 | 0.738800143 |
| positive regulation of excitatory postsynaptic potential (( | 44638 | 0.434193959 | 0.738800143 |
| positive regulation of nervous system process (GO:00316 | 44638 | 0.434193959 | 0.738800143 |
| presynapse assembly (GO:0099054) | 44638 | 0.434193959 | 0.738800143 |
| protein localization to centrosome (GO:0071539) | 44638 | 0.434193959 | 0.738800143 |
| pulmonary valve development (GO:0003177) | 44638 | 0.434193959 | 0.738800143 |
| regulation of cellular protein catabolic process (GO:1903: | 44638 | 0.434193959 | 0.738800143 |
| regulation of early endosome to late endosome transpor | 44638 | 0.434193959 | 0.738800143 |
| regulation of fatty acid metabolic process (GO:0019217) | 44638 | 0.434193959 | 0.738800143 |
| regulation of multicellular organism growth (GO:004001 | 44638 | 0.434193959 | 0.738800143 |
| regulation of spindle organization (GO:0090224) | 44638 | 0.434193959 | 0.738800143 |
| regulation of vascular endothelial cell proliferation (GO:1 | 44638 | 0.434193959 | 0.738800143 |
| RNA destabilization (GO:0050779) | 44638 | 0.434193959 | 0.738800143 |
| rRNA catabolic process (GO:0016075) | 44638 | 0.434193959 | 0.738800143 |
| aminoglycan metabolic process (GO:0006022) | 44603 | 0.439440316 | 0.738800143 |
| angiogenesis involved in wound healing (GO:0060055) | 44603 | 0.439440316 | 0.738800143 |
| camera-type eye morphogenesis (GO:0048593) | 44603 | 0.439440316 | 0.738800143 |
| cardiac muscle cell membrane repolarization (GO:00996: | 44603 | 0.439440316 | 0.738800143 |
| carnitine shuttle (GO:0006853) | 44603 | 0.439440316 | 0.738800143 |
| cell differentiation in spinal cord (GO:0021515) | 44603 | 0.439440316 | 0.738800143 |
| cell proliferation in forebrain (GO:0021846) | 44603 | 0.439440316 | 0.738800143 |
| cell-cell junction maintenance (GO:0045217) | 44603 | 0.439440316 | 0.738800143 |
| cellular response to cholesterol (GO:0071397) | 44603 | 0.439440316 | 0.738800143 |
| cellular response to superoxide (GO:0071451) | 44603 | 0.439440316 | 0.738800143 |
| cyclooxygenase pathway (GO:0019371) | 44603 | 0.439440316 | 0.738800143 |
| DNA damage response, signal transduction resulting in tr | 44603 | 0.439440316 | 0.738800143 |
| embryonic eye morphogenesis (GO:0048048) | 44603 | 0.439440316 | 0.738800143 |
| glutathione biosynthetic process (GO:0006750) | 44603 | 0.439440316 | 0.738800143 |

| | | | |
|---|---|---|---|
| glycerol metabolic process (GO:0006071) | 44603 | 0.439440316 | 0.738800143 |
| glycerolipid metabolic process (GO:0046486) | 44603 | 0.439440316 | 0.738800143 |
| Golgi inheritance (GO:0048313) | 44603 | 0.439440316 | 0.738800143 |
| intestinal lipid absorption (GO:0098856) | 44603 | 0.439440316 | 0.738800143 |
| lipoprotein localization (GO:0044872) | 44603 | 0.439440316 | 0.738800143 |
| midbrain dopaminergic neuron differentiation (GO:1904 | 44603 | 0.439440316 | 0.738800143 |
| monocyte differentiation (GO:0030224) | 44603 | 0.439440316 | 0.738800143 |
| negative regulation of cholesterol transport (GO:003237 | 44603 | 0.439440316 | 0.738800143 |
| negative regulation of defense response to virus (GO:005 | 44603 | 0.439440316 | 0.738800143 |
| negative regulation of homotypic cell-cell adhesion (GO:( | 44603 | 0.439440316 | 0.738800143 |
| negative regulation of lymphocyte migration (GO:20004C | 44603 | 0.439440316 | 0.738800143 |
| negative regulation of protein localization to cell surface | 44603 | 0.439440316 | 0.738800143 |
| negative regulation of sprouting angiogenesis (GO:19036 | 44603 | 0.439440316 | 0.738800143 |
| positive regulation by host of viral process (GO:0044794) | 44603 | 0.439440316 | 0.738800143 |
| positive regulation of bone resorption (GO:0045780) | 44603 | 0.439440316 | 0.738800143 |
| positive regulation of histone exchange (GO:1900051) | 44603 | 0.439440316 | 0.738800143 |
| positive regulation of keratinocyte proliferation (GO:001( | 44603 | 0.439440316 | 0.738800143 |
| positive regulation of phospholipid transport (GO:20011 | 44603 | 0.439440316 | 0.738800143 |
| positive regulation of transcription elongation from RNA | 44603 | 0.439440316 | 0.738800143 |
| positive regulation of vacuole organization (GO:0044090) | 44603 | 0.439440316 | 0.738800143 |
| presynaptic membrane organization (GO:0097090) | 44603 | 0.439440316 | 0.738800143 |
| protein kinase C signaling (GO:0070528) | 44603 | 0.439440316 | 0.738800143 |
| protein kinase C-activating G protein-coupled receptor si | 44603 | 0.439440316 | 0.738800143 |
| protein oxidation (GO:0018158) | 44603 | 0.439440316 | 0.738800143 |
| pyrimidine nucleoside salvage (GO:0043097) | 44603 | 0.439440316 | 0.738800143 |
| pyrimidine-containing compound salvage (GO:0008655) | 44603 | 0.439440316 | 0.738800143 |
| regulation of cardiac muscle cell apoptotic process (GO:0 | 44603 | 0.439440316 | 0.738800143 |
| regulation of dendritic cell apoptotic process (GO:20006( | 44603 | 0.439440316 | 0.738800143 |
| regulation of histone exchange (GO:1900049) | 44603 | 0.439440316 | 0.738800143 |
| regulation of inclusion body assembly (GO:0090083) | 44603 | 0.439440316 | 0.738800143 |
| regulation of mast cell activation involved in immune res | 44603 | 0.439440316 | 0.738800143 |
| regulation of mesenchymal cell proliferation (GO:001046 | 44603 | 0.439440316 | 0.738800143 |
| regulation of microtubule-based movement (GO:006063. | 44603 | 0.439440316 | 0.738800143 |
| regulation of mitochondrial depolarization (GO:0051900) | 44603 | 0.439440316 | 0.738800143 |
| regulation of phospholipase activity (GO:0010517) | 44603 | 0.439440316 | 0.738800143 |
| tetrahydrobiopterin metabolic process (GO:0046146) | 44603 | 0.439440316 | 0.738800143 |
| tetrahydrofolate metabolic process (GO:0046653) | 44603 | 0.439440316 | 0.738800143 |
| urate metabolic process (GO:0046415) | 44603 | 0.439440316 | 0.738800143 |
| bone development (GO:0060348) | 14763 | 0.441244997 | 0.741114697 |
| carboxylic acid metabolic process (GO:0019752) | 14763 | 0.441244997 | 0.741114697 |
| regulation of sodium ion transmembrane transporter act | 14763 | 0.441244997 | 0.741114697 |
| extracellular matrix organization (GO:0030198) | 41/300 | 0.443945671 | 0.745409748 |
| negative regulation of cell motility (GO:2000146) | 16/114 | 0.445160893 | 0.747208672 |
| regulation of organelle assembly (GO:1902115) | 28430 | 0.445502899 | 0.747541201 |
| muscle contraction (GO:0006936) | 18/129 | 0.447368582 | 0.750429379 |
| glycoprotein metabolic process (GO:0009100) | 20302 | 0.448286414 | 0.751483679 |
| regulation of cardiac conduction (GO:1903779) | 20302 | 0.448286414 | 0.751483679 |

| | | | |
|---|---|---|---|
| regulation of lipid metabolic process (GO:0019216) | 13/92 | 0.448874049 | 0.75217982 |
| actin filament bundle assembly (GO:0051017) | 12175 | 0.449570422 | 0.75217982 |
| actin filament bundle organization (GO:0061572) | 12175 | 0.449570422 | 0.75217982 |
| keratan sulfate metabolic process (GO:0042339) | 12175 | 0.449570422 | 0.75217982 |
| muscle cell development (GO:0055001) | 12175 | 0.449570422 | 0.75217982 |
| very long-chain fatty acid metabolic process (GO:000003 | 12175 | 0.449570422 | 0.75217982 |
| kidney development (GO:0001822) | 25842 | 0.452734157 | 0.757229216 |
| myelination (GO:0042552) | 17715 | 0.456556187 | 0.762493641 |
| purine ribonucleotide biosynthetic process (GO:0009152 | 17715 | 0.456556187 | 0.762493641 |
| regulation of mitochondrion organization (GO:0010821) | 17715 | 0.456556187 | 0.762493641 |
| substrate adhesion-dependent cell spreading (GO:00344 | 17715 | 0.456556187 | 0.762493641 |
| cell-matrix adhesion (GO:0007160) | 14/100 | 0.458061549 | 0.762493641 |
| cellular response to drug (GO:0035690) | 44677 | 0.459199858 | 0.762493641 |
| chaperone cofactor-dependent protein refolding (GO:005 | 44677 | 0.459199858 | 0.762493641 |
| clathrin-dependent endocytosis (GO:0072583) | 44677 | 0.459199858 | 0.762493641 |
| meiosis I (GO:0007127) | 44677 | 0.459199858 | 0.762493641 |
| negative regulation of proteolysis involved in cellular pro | 44677 | 0.459199858 | 0.762493641 |
| positive regulation of histone methylation (GO:0031062) | 44677 | 0.459199858 | 0.762493641 |
| positive regulation of protein insertion into mitochondria | 44677 | 0.459199858 | 0.762493641 |
| positive regulation of viral transcription (GO:0050434) | 44677 | 0.459199858 | 0.762493641 |
| regulation of actin filament bundle assembly (GO:003223 | 44677 | 0.459199858 | 0.762493641 |
| regulation of chondrocyte differentiation (GO:0032330) | 44677 | 0.459199858 | 0.762493641 |
| regulation of mitotic metaphase/anaphase transition (GC | 44677 | 0.459199858 | 0.762493641 |
| regulation of myelination (GO:0031641) | 44677 | 0.459199858 | 0.762493641 |
| regulation of protein insertion into mitochondrial membr | 44677 | 0.459199858 | 0.762493641 |
| organonitrogen compound catabolic process (GO:19015€ | 23255 | 0.460619361 | 0.762493641 |
| acylglycerol metabolic process (GO:0006639) | 15128 | 0.465890181 | 0.762493641 |
| carbohydrate catabolic process (GO:0016052) | 15128 | 0.465890181 | 0.762493641 |
| regulation of response to DNA damage stimulus (GO:200 | 15128 | 0.465890181 | 0.762493641 |
| response to growth factor (GO:0070848) | 15128 | 0.465890181 | 0.762493641 |
| negative regulation of transmembrane receptor protein s | 15/108 | 0.466516173 | 0.762493641 |
| glycosaminoglycan catabolic process (GO:0006027) | 20668 | 0.469321609 | 0.762493641 |
| mitochondrial transport (GO:0006839) | 20668 | 0.469321609 | 0.762493641 |
| nuclear-transcribed mRNA catabolic process, deadenylati | 20668 | 0.469321609 | 0.762493641 |
| actin filament capping (GO:0051693) | 44639 | 0.470849586 | 0.762493641 |
| activin receptor signaling pathway (GO:0032924) | 44639 | 0.470849586 | 0.762493641 |
| barbed-end actin filament capping (GO:0051016) | 44639 | 0.470849586 | 0.762493641 |
| cellular response to glucagon stimulus (GO:0071377) | 44639 | 0.470849586 | 0.762493641 |
| cytoplasmic translational initiation (GO:0002183) | 44639 | 0.470849586 | 0.762493641 |
| diol metabolic process (GO:0034311) | 44639 | 0.470849586 | 0.762493641 |
| epoxygenase P450 pathway (GO:0019373) | 44639 | 0.470849586 | 0.762493641 |
| fatty acid transmembrane transport (GO:1902001) | 44639 | 0.470849586 | 0.762493641 |
| folic acid metabolic process (GO:0046655) | 44639 | 0.470849586 | 0.762493641 |
| histone H4-K16 acetylation (GO:0043984) | 44639 | 0.470849586 | 0.762493641 |
| long-term memory (GO:0007616) | 44639 | 0.470849586 | 0.762493641 |
| mitochondrion localization (GO:0051646) | 44639 | 0.470849586 | 0.762493641 |
| negative regulation of calcium ion transport (GO:005192( | 44639 | 0.470849586 | 0.762493641 |

| | | | |
|---|---|---|---|
| negative regulation of protein localization to nucleus (GO | 44639 | 0.470849586 | 0.762493641 |
| negative regulation of RNA splicing (GO:0033119) | 44639 | 0.470849586 | 0.762493641 |
| negative regulation of striated muscle cell differentiation | 44639 | 0.470849586 | 0.762493641 |
| NLS-bearing protein import into nucleus (GO:0006607) | 44639 | 0.470849586 | 0.762493641 |
| positive regulation of establishment of protein localizatio | 44639 | 0.470849586 | 0.762493641 |
| postsynaptic membrane organization (GO:0001941) | 44639 | 0.470849586 | 0.762493641 |
| regulation of collagen biosynthetic process (GO:0032965 | 44639 | 0.470849586 | 0.762493641 |
| regulation of mitochondrial membrane permeability (GO | 44639 | 0.470849586 | 0.762493641 |
| regulation of mitotic spindle assembly (GO:1901673) | 44639 | 0.470849586 | 0.762493641 |
| response to ATP (GO:0033198) | 44639 | 0.470849586 | 0.762493641 |
| striated muscle cell differentiation (GO:0051146) | 44639 | 0.470849586 | 0.762493641 |
| negative regulation of neuron apoptotic process (GO:004 | 26207 | 0.471391373 | 0.762493641 |
| phosphatidylinositol-mediated signaling (GO:0048015) | 26207 | 0.471391373 | 0.762493641 |
| protein tetramerization (GO:0051262) | 26207 | 0.471391373 | 0.762493641 |
| cardiac muscle contraction (GO:0060048) | 12540 | 0.476702873 | 0.762493641 |
| epithelial tube morphogenesis (GO:0060562) | 12540 | 0.476702873 | 0.762493641 |
| negative regulation of blood vessel endothelial cell migra | 12540 | 0.476702873 | 0.762493641 |
| phosphatidylinositol 3-kinase signaling (GO:0014065) | 12540 | 0.476702873 | 0.762493641 |
| positive regulation of DNA binding (GO:0043388) | 12540 | 0.476702873 | 0.762493641 |
| regulation of keratinocyte differentiation (GO:0045616) | 12540 | 0.476702873 | 0.762493641 |
| response to steroid hormone (GO:0048545) | 12540 | 0.476702873 | 0.762493641 |
| adherens junction organization (GO:0034332) | 18080 | 0.479075535 | 0.762493641 |
| protein secretion (GO:0009306) | 23621 | 0.480289067 | 0.762493641 |
| regulation of cellular ketone metabolic process (GO:0010 | 23621 | 0.480289067 | 0.762493641 |
| inorganic cation transmembrane transport (GO:0098662) | 37/274 | 0.48045763 | 0.762493641 |
| regulation of kinase activity (GO:0043549) | 13/94 | 0.481352558 | 0.762493641 |
| non-canonical Wnt signaling pathway (GO:0035567) | 17/124 | 0.481654965 | 0.762493641 |
| autophagy of peroxisome (GO:0030242) | 44604 | 0.486094803 | 0.762493641 |
| C-terminal protein amino acid modification (GO:0018410 | 44604 | 0.486094803 | 0.762493641 |
| cell cycle checkpoint signaling (GO:0000075) | 44604 | 0.486094803 | 0.762493641 |
| dosage compensation by inactivation of X chromosome ( | 44604 | 0.486094803 | 0.762493641 |
| energy coupled proton transport, down electrochemical ; | 44604 | 0.486094803 | 0.762493641 |
| exit from mitosis (GO:0010458) | 44604 | 0.486094803 | 0.762493641 |
| eye photoreceptor cell differentiation (GO:0001754) | 44604 | 0.486094803 | 0.762493641 |
| glutamine metabolic process (GO:0006541) | 44604 | 0.486094803 | 0.762493641 |
| macrophage chemotaxis (GO:0048246) | 44604 | 0.486094803 | 0.762493641 |
| microtubule anchoring at microtubule organizing center ( | 44604 | 0.486094803 | 0.762493641 |
| mitochondrion distribution (GO:0048311) | 44604 | 0.486094803 | 0.762493641 |
| modulation by host of viral process (GO:0044788) | 44604 | 0.486094803 | 0.762493641 |
| morphogenesis of a polarized epithelium (GO:0001738) | 44604 | 0.486094803 | 0.762493641 |
| motile cilium assembly (GO:0044458) | 44604 | 0.486094803 | 0.762493641 |
| negative regulation of cell division (GO:0051782) | 44604 | 0.486094803 | 0.762493641 |
| negative regulation of cilium assembly (GO:1902018) | 44604 | 0.486094803 | 0.762493641 |
| negative regulation of gene silencing by miRNA (GO:0060 | 44604 | 0.486094803 | 0.762493641 |
| negative regulation of hemostasis (GO:1900047) | 44604 | 0.486094803 | 0.762493641 |
| negative regulation of leukocyte chemotaxis (GO:000268 | 44604 | 0.486094803 | 0.762493641 |
| negative regulation of receptor internalization (GO:0002( | 44604 | 0.486094803 | 0.762493641 |

| | | | |
|---|---|---|---|
| negative regulation of release of sequestered calcium ior | 44604 | 0.486094803 | 0.762493641 |
| negative regulation of ryanodine-sensitive calcium-releas | 44604 | 0.486094803 | 0.762493641 |
| neurotransmitter uptake (GO:0001504) | 44604 | 0.486094803 | 0.762493641 |
| nonribosomal peptide biosynthetic process (GO:0019184 | 44604 | 0.486094803 | 0.762493641 |
| nucleoside salvage (GO:0043174) | 44604 | 0.486094803 | 0.762493641 |
| phasic smooth muscle contraction (GO:0014821) | 44604 | 0.486094803 | 0.762493641 |
| phosphate ion transmembrane transport (GO:0035435) | 44604 | 0.486094803 | 0.762493641 |
| phospholipid homeostasis (GO:0055091) | 44604 | 0.486094803 | 0.762493641 |
| poly-N-acetyllactosamine biosynthetic process (GO:0030: | 44604 | 0.486094803 | 0.762493641 |
| positive regulation of actin filament depolymerization (G( | 44604 | 0.486094803 | 0.762493641 |
| positive regulation of sodium ion transmembrane transp( | 44604 | 0.486094803 | 0.762493641 |
| positive regulation of ubiquitin protein ligase activity (GO | 44604 | 0.486094803 | 0.762493641 |
| pyrimidine nucleobase metabolic process (GO:0006206) | 44604 | 0.486094803 | 0.762493641 |
| regulation of actin filament depolymerization (GO:00308 | 44604 | 0.486094803 | 0.762493641 |
| regulation of actin nucleation (GO:0051125) | 44604 | 0.486094803 | 0.762493641 |
| regulation of cell communication by electrical coupling in | 44604 | 0.486094803 | 0.762493641 |
| regulation of cholesterol esterification (GO:0010872) | 44604 | 0.486094803 | 0.762493641 |
| regulation of dendritic cell differentiation (GO:2001198) | 44604 | 0.486094803 | 0.762493641 |
| regulation of feeding behavior (GO:0060259) | 44604 | 0.486094803 | 0.762493641 |
| regulation of flagellated sperm motility (GO:1901317) | 44604 | 0.486094803 | 0.762493641 |
| regulation of histone H3-K9 methylation (GO:0051570) | 44604 | 0.486094803 | 0.762493641 |
| regulation of leukocyte cell-cell adhesion (GO:1903037) | 44604 | 0.486094803 | 0.762493641 |
| regulation of oxidative stress-induced cell death (GO:190 | 44604 | 0.486094803 | 0.762493641 |
| regulation of RNA export from nucleus (GO:0046831) | 44604 | 0.486094803 | 0.762493641 |
| regulation of vascular associated smooth muscle cell mig | 44604 | 0.486094803 | 0.762493641 |
| serine family amino acid biosynthetic process (GO:00090 | 44604 | 0.486094803 | 0.762493641 |
| tricarboxylic acid metabolic process (GO:0072350) | 44604 | 0.486094803 | 0.762493641 |
| modulation of excitatory postsynaptic potential (GO:009{ | 44678 | 0.489719421 | 0.762493641 |
| negative regulation of plasma membrane bounded cell pı | 44678 | 0.489719421 | 0.762493641 |
| positive regulation of muscle cell differentiation (GO:005 | 44678 | 0.489719421 | 0.762493641 |
| cell junction organization (GO:0034330) | 26573 | 0.489923383 | 0.762493641 |
| negative regulation of DNA recombination (GO:0045910) | 21033 | 0.490173585 | 0.762493641 |
| ceramide biosynthetic process (GO:0046513) | 15493 | 0.490235861 | 0.762493641 |
| regulation of nervous system development (GO:0051960 | 15493 | 0.490235861 | 0.762493641 |
| lipid biosynthetic process (GO:0008610) | 29526 | 0.4985318 | 0.762493641 |
| regulation of translation (GO:0006417) | 24/178 | 0.499173098 | 0.762493641 |
| G protein-coupled receptor signaling pathway, coupled t( | 23986 | 0.499787379 | 0.762493641 |
| positive regulation of ion transport (GO:0043270) | 18445 | 0.501333751 | 0.762493641 |
| lung development (GO:0030324) | 12905 | 0.503374736 | 0.762493641 |
| positive regulation of reproductive process (GO:2000243 | 12905 | 0.503374736 | 0.762493641 |
| regulation of muscle cell differentiation (GO:0051147) | 12905 | 0.503374736 | 0.762493641 |
| regulation of trans-synaptic signaling (GO:0099177) | 12905 | 0.503374736 | 0.762493641 |
| respiratory system development (GO:0060541) | 12905 | 0.503374736 | 0.762493641 |
| retrograde transport, endosome to Golgi (GO:0042147) | 32478 | 0.506327298 | 0.762493641 |
| cardiac ventricle development (GO:0003231) | 44640 | 0.50638421 | 0.762493641 |
| cellular modified amino acid biosynthetic process (GO:00 | 44640 | 0.50638421 | 0.762493641 |
| dopaminergic neuron differentiation (GO:0071542) | 44640 | 0.50638421 | 0.762493641 |

| | | | |
|---|---|---|---|
| inositol phosphate-mediated signaling (GO:0048016) | 44640 | 0.50638421 | 0.762493641 |
| natural killer cell mediated immunity (GO:0002228) | 44640 | 0.50638421 | 0.762493641 |
| negative regulation of cyclin-dependent protein serine/tl | 44640 | 0.50638421 | 0.762493641 |
| negative regulation of osteoclast differentiation (GO:004 | 44640 | 0.50638421 | 0.762493641 |
| negative regulation of telomere maintenance via telomeı | 44640 | 0.50638421 | 0.762493641 |
| positive regulation of epidermal cell differentiation (GO:( | 44640 | 0.50638421 | 0.762493641 |
| positive regulation of epithelial cell differentiation (GO:0( | 44640 | 0.50638421 | 0.762493641 |
| purine ribonucleoside monophosphate metabolic proces: | 44640 | 0.50638421 | 0.762493641 |
| regulation of protein localization to cell periphery (GO:19 | 44640 | 0.50638421 | 0.762493641 |
| regulation of smooth muscle contraction (GO:0006940) | 44640 | 0.50638421 | 0.762493641 |
| chromatin assembly (GO:0031497) | 26938 | 0.508293846 | 0.762493641 |
| 5-phosphoribose 1-diphosphate biosynthetic process (GC | 44566 | 0.509160816 | 0.762493641 |
| 5-phosphoribose 1-diphosphate metabolic process (GO:0 | 44566 | 0.509160816 | 0.762493641 |
| adenosine 5'-(hexahydrogen pentaphosphate) catabolic | 44566 | 0.509160816 | 0.762493641 |
| adenosine 5'-(hexahydrogen pentaphosphate) metabolic | 44566 | 0.509160816 | 0.762493641 |
| anterograde dendritic transport (GO:0098937) | 44566 | 0.509160816 | 0.762493641 |
| anterograde dendritic transport of neurotransmitter rece | 44566 | 0.509160816 | 0.762493641 |
| antigen processing and presentation of lipid antigen via N | 44566 | 0.509160816 | 0.762493641 |
| antigen processing and presentation, endogenous lipid aı | 44566 | 0.509160816 | 0.762493641 |
| antigen processing and presentation, exogenous lipid ant | 44566 | 0.509160816 | 0.762493641 |
| apoptotic chromosome condensation (GO:0030263) | 44566 | 0.509160816 | 0.762493641 |
| beta-catenin destruction complex assembly (GO:190488! | 44566 | 0.509160816 | 0.762493641 |
| bile acid secretion (GO:0032782) | 44566 | 0.509160816 | 0.762493641 |
| C-terminal protein deglutamylation (GO:0035609) | 44566 | 0.509160816 | 0.762493641 |
| C-terminal protein lipidation (GO:0006501) | 44566 | 0.509160816 | 0.762493641 |
| cannabinoid signaling pathway (GO:0038171) | 44566 | 0.509160816 | 0.762493641 |
| cardiac endothelial cell differentiation (GO:0003348) | 44566 | 0.509160816 | 0.762493641 |
| cell fate commitment involved in formation of primary ge | 44566 | 0.509160816 | 0.762493641 |
| cellular response to antibiotic (GO:0071236) | 44566 | 0.509160816 | 0.762493641 |
| cellular response to erythropoietin (GO:0036018) | 44566 | 0.509160816 | 0.762493641 |
| cellular response to heparin (GO:0071504) | 44566 | 0.509160816 | 0.762493641 |
| cellular response to macrophage colony-stimulating facto | 44566 | 0.509160816 | 0.762493641 |
| cellular response to nutrient (GO:0031670) | 44566 | 0.509160816 | 0.762493641 |
| cellular response to testosterone stimulus (GO:0071394) | 44566 | 0.509160816 | 0.762493641 |
| cerebellar Purkinje cell differentiation (GO:0021702) | 44566 | 0.509160816 | 0.762493641 |
| cerebral cortex neuron differentiation (GO:0021895) | 44566 | 0.509160816 | 0.762493641 |
| chemokine (C-X-C motif) ligand 12 signaling pathway (GO | 44566 | 0.509160816 | 0.762493641 |
| cyclic-GMP-AMP transmembrane import across plasma n | 44566 | 0.509160816 | 0.762493641 |
| dephosphorylation of RNA polymerase II C-terminal dom | 44566 | 0.509160816 | 0.762493641 |
| determination of dorsal/ventral asymmetry (GO:0048262 | 44566 | 0.509160816 | 0.762493641 |
| diadenosine hexaphosphate catabolic process (GO:19019 | 44566 | 0.509160816 | 0.762493641 |
| diadenosine hexaphosphate metabolic process (GO:1901 | 44566 | 0.509160816 | 0.762493641 |
| diadenosine pentaphosphate catabolic process (GO:1901 | 44566 | 0.509160816 | 0.762493641 |
| diadenosine pentaphosphate metabolic process (GO:190 | 44566 | 0.509160816 | 0.762493641 |
| diaphragm development (GO:0060539) | 44566 | 0.509160816 | 0.762493641 |
| diphosphoinositol polyphosphate metabolic process (GO: | 44566 | 0.509160816 | 0.762493641 |
| disaccharide biosynthetic process (GO:0046351) | 44566 | 0.509160816 | 0.762493641 |

| | | | |
|---|---|---|---|
| DNA protection (GO:0042262) | 44566 | 0.509160816 | 0.762493641 |
| dopamine biosynthetic process (GO:0042416) | 44566 | 0.509160816 | 0.762493641 |
| drug transport (GO:0015893) | 44566 | 0.509160816 | 0.762493641 |
| embryonic viscerocranium morphogenesis (GO:0048703) | 44566 | 0.509160816 | 0.762493641 |
| enamel mineralization (GO:0070166) | 44566 | 0.509160816 | 0.762493641 |
| endocardial cell differentiation (GO:0060956) | 44566 | 0.509160816 | 0.762493641 |
| endothelial cell apoptotic process (GO:0072577) | 44566 | 0.509160816 | 0.762493641 |
| establishment of monopolar cell polarity (GO:0061162) | 44566 | 0.509160816 | 0.762493641 |
| establishment of protein localization to telomere (GO:00 | 44566 | 0.509160816 | 0.762493641 |
| exocyst localization (GO:0051601) | 44566 | 0.509160816 | 0.762493641 |
| extracellular exosome biogenesis (GO:0097734) | 44566 | 0.509160816 | 0.762493641 |
| female genitalia development (GO:0030540) | 44566 | 0.509160816 | 0.762493641 |
| forebrain regionalization (GO:0021871) | 44566 | 0.509160816 | 0.762493641 |
| fructose catabolic process (GO:0006001) | 44566 | 0.509160816 | 0.762493641 |
| fructose catabolic process to hydroxyacetone phosphate | 44566 | 0.509160816 | 0.762493641 |
| fructose transmembrane transport (GO:0015755) | 44566 | 0.509160816 | 0.762493641 |
| G protein-coupled opioid receptor signaling pathway (GC | 44566 | 0.509160816 | 0.762493641 |
| glial cell-derived neurotrophic factor receptor signaling p | 44566 | 0.509160816 | 0.762493641 |
| glucose-6-phosphate transport (GO:0015760) | 44566 | 0.509160816 | 0.762493641 |
| glucosylceramide metabolic process (GO:0006678) | 44566 | 0.509160816 | 0.762493641 |
| glutamate catabolic process (GO:0006538) | 44566 | 0.509160816 | 0.762493641 |
| glycerol biosynthetic process (GO:0006114) | 44566 | 0.509160816 | 0.762493641 |
| glycerol ether metabolic process (GO:0006662) | 44566 | 0.509160816 | 0.762493641 |
| hemoglobin metabolic process (GO:0020027) | 44566 | 0.509160816 | 0.762493641 |
| hexose phosphate transport (GO:0015712) | 44566 | 0.509160816 | 0.762493641 |
| histone-threonine phosphorylation (GO:0035405) | 44566 | 0.509160816 | 0.762493641 |
| insulin metabolic process (GO:1901142) | 44566 | 0.509160816 | 0.762493641 |
| intracellular distribution of mitochondria (GO:0048312) | 44566 | 0.509160816 | 0.762493641 |
| isocitrate metabolic process (GO:0006102) | 44566 | 0.509160816 | 0.762493641 |
| isopentenyl diphosphate biosynthetic process (GO:00092 | 44566 | 0.509160816 | 0.762493641 |
| L-arginine import across plasma membrane (GO:009763 | 44566 | 0.509160816 | 0.762493641 |
| leukotriene B4 metabolic process (GO:0036102) | 44566 | 0.509160816 | 0.762493641 |
| mammary gland duct morphogenesis (GO:0060603) | 44566 | 0.509160816 | 0.762493641 |
| mannose trimming involved in glycoprotein ERAD pathwa | 44566 | 0.509160816 | 0.762493641 |
| mature conventional dendritic cell differentiation (GO:00 | 44566 | 0.509160816 | 0.762493641 |
| medium-chain fatty acid catabolic process (GO:0051793) | 44566 | 0.509160816 | 0.762493641 |
| membrane raft distribution (GO:0031580) | 44566 | 0.509160816 | 0.762493641 |
| menaquinone metabolic process (GO:0009233) | 44566 | 0.509160816 | 0.762493641 |
| metanephric mesenchymal cell differentiation (GO:0072 | 44566 | 0.509160816 | 0.762493641 |
| mitochondrial RNA 3'-end processing (GO:0000965) | 44566 | 0.509160816 | 0.762493641 |
| modulation by symbiont of host autophagy (GO:0075071 | 44566 | 0.509160816 | 0.762493641 |
| mRNA 3'-end processing by stem-loop binding and cleava | 44566 | 0.509160816 | 0.762493641 |
| negative regulation of adaptive immune response (GO:0 | 44566 | 0.509160816 | 0.762493641 |
| negative regulation of autophagosome maturation (GO:1 | 44566 | 0.509160816 | 0.762493641 |
| negative regulation of B cell differentiation (GO:0045578 | 44566 | 0.509160816 | 0.762493641 |
| negative regulation of cellular respiration (GO:1901856) | 44566 | 0.509160816 | 0.762493641 |
| negative regulation of ceramide biosynthetic process (GC | 44566 | 0.509160816 | 0.762493641 |

| | | | |
|---|---|---|---|
| negative regulation of dendritic cell differentiation (GO:2 | 44566 | 0.509160816 | 0.762493641 |
| negative regulation of histone deacetylation (GO:003106 | 44566 | 0.509160816 | 0.762493641 |
| negative regulation of interferon-alpha production (GO:0 | 44566 | 0.509160816 | 0.762493641 |
| negative regulation of leukocyte adhesion to vascular en | 44566 | 0.509160816 | 0.762493641 |
| negative regulation of leukocyte differentiation (GO:1902 | 44566 | 0.509160816 | 0.762493641 |
| negative regulation of macrophage chemotaxis (GO:0010 | 44566 | 0.509160816 | 0.762493641 |
| negative regulation of macrophage cytokine production ( | 44566 | 0.509160816 | 0.762493641 |
| negative regulation of macrophage differentiation (GO:0 | 44566 | 0.509160816 | 0.762493641 |
| negative regulation of organelle organization (GO:00106 | 44566 | 0.509160816 | 0.762493641 |
| negative regulation of oxidative stress-induced neuron in | 44566 | 0.509160816 | 0.762493641 |
| negative regulation of plasminogen activation (GO:00107 | 44566 | 0.509160816 | 0.762493641 |
| negative regulation of protein acetylation (GO:1901984) | 44566 | 0.509160816 | 0.762493641 |
| negative regulation of protein polyubiquitination (GO:19 | 44566 | 0.509160816 | 0.762493641 |
| negative regulation of regulatory T cell differentiation (G | 44566 | 0.509160816 | 0.762493641 |
| negative regulation of smooth muscle contraction (GO:0 | 44566 | 0.509160816 | 0.762493641 |
| negative regulation of synapse organization (GO:190580 | 44566 | 0.509160816 | 0.762493641 |
| negative regulation of tubulin deacetylation (GO:190442 | 44566 | 0.509160816 | 0.762493641 |
| nucleoside transport (GO:0015858) | 44566 | 0.509160816 | 0.762493641 |
| nucleoside triphosphate catabolic process (GO:0009143) | 44566 | 0.509160816 | 0.762493641 |
| oocyte development (GO:0048599) | 44566 | 0.509160816 | 0.762493641 |
| oxaloacetate transport (GO:0015729) | 44566 | 0.509160816 | 0.762493641 |
| oxidative DNA demethylation (GO:0035511) | 44566 | 0.509160816 | 0.762493641 |
| peptidyl-lysine oxidation (GO:0018057) | 44566 | 0.509160816 | 0.762493641 |
| peristalsis (GO:0030432) | 44566 | 0.509160816 | 0.762493641 |
| phenol-containing compound metabolic process (GO:001 | 44566 | 0.509160816 | 0.762493641 |
| PML body organization (GO:0030578) | 44566 | 0.509160816 | 0.762493641 |
| positive regulation by host of viral genome replication (G | 44566 | 0.509160816 | 0.762493641 |
| positive regulation of 3'-UTR-mediated mRNA stabilizatio | 44566 | 0.509160816 | 0.762493641 |
| positive regulation of amine transport (GO:0051954) | 44566 | 0.509160816 | 0.762493641 |
| positive regulation of AMPA receptor activity (GO:20009 | 44566 | 0.509160816 | 0.762493641 |
| positive regulation of anoikis (GO:2000210) | 44566 | 0.509160816 | 0.762493641 |
| positive regulation of basement membrane assembly inv | 44566 | 0.509160816 | 0.762493641 |
| positive regulation of bicellular tight junction assembly ( | 44566 | 0.509160816 | 0.762493641 |
| positive regulation of cAMP-dependent protein kinase ac | 44566 | 0.509160816 | 0.762493641 |
| positive regulation of cardiac muscle contraction (GO:00 | 44566 | 0.509160816 | 0.762493641 |
| positive regulation of dendritic cell differentiation (GO:2 | 44566 | 0.509160816 | 0.762493641 |
| positive regulation of deoxyribonuclease activity (GO:003 | 44566 | 0.509160816 | 0.762493641 |
| positive regulation of DNA ligation (GO:0051106) | 44566 | 0.509160816 | 0.762493641 |
| positive regulation of dopamine receptor signaling pathw | 44566 | 0.509160816 | 0.762493641 |
| positive regulation of endothelial cell-matrix adhesion vi | 44566 | 0.509160816 | 0.762493641 |
| positive regulation of epithelial to mesenchymal transitio | 44566 | 0.509160816 | 0.762493641 |
| positive regulation of feeding behavior (GO:2000253) | 44566 | 0.509160816 | 0.762493641 |
| positive regulation of fertilization (GO:1905516) | 44566 | 0.509160816 | 0.762493641 |
| positive regulation of fibroblast growth factor receptor si | 44566 | 0.509160816 | 0.762493641 |
| positive regulation of gamma-delta T cell activation (GO: | 44566 | 0.509160816 | 0.762493641 |
| positive regulation of Golgi to plasma membrane protein | 44566 | 0.509160816 | 0.762493641 |
| positive regulation of histone H3-K9 acetylation (GO:200 | 44566 | 0.509160816 | 0.762493641 |

| | | | |
|---|---|---|---|
| positive regulation of intrinsic apoptotic signaling pathwa | 44566 | 0.509160816 | 0.762493641 |
| positive regulation of long-term synaptic depression (GO: | 44566 | 0.509160816 | 0.762493641 |
| positive regulation of low-density lipoprotein receptor ac | 44566 | 0.509160816 | 0.762493641 |
| positive regulation of macrophage proliferation (GO:012( | 44566 | 0.509160816 | 0.762493641 |
| positive regulation of maintenance of mitotic sister chror | 44566 | 0.509160816 | 0.762493641 |
| positive regulation of maintenance of sister chromatid cc | 44566 | 0.509160816 | 0.762493641 |
| positive regulation of membrane permeability (GO:19057 | 44566 | 0.509160816 | 0.762493641 |
| positive regulation of myoblast proliferation (GO:200028 | 44566 | 0.509160816 | 0.762493641 |
| positive regulation of neurotransmitter transport (GO:00 | 44566 | 0.509160816 | 0.762493641 |
| positive regulation of neutrophil extravasation (GO:2000: | 44566 | 0.509160816 | 0.762493641 |
| positive regulation of nucleobase-containing compound t | 44566 | 0.509160816 | 0.762493641 |
| positive regulation of prostaglandin secretion (GO:00323 | 44566 | 0.509160816 | 0.762493641 |
| positive regulation of protein localization to cell cortex (G | 44566 | 0.509160816 | 0.762493641 |
| positive regulation of protein monoubiquitination (GO:19 | 44566 | 0.509160816 | 0.762493641 |
| positive regulation of protein neddylation (GO:2000436) | 44566 | 0.509160816 | 0.762493641 |
| positive regulation of RNA export from nucleus (GO:0046 | 44566 | 0.509160816 | 0.762493641 |
| positive regulation of the force of heart contraction (GO: | 44566 | 0.509160816 | 0.762493641 |
| positive regulation of translation in response to stress (G | 44566 | 0.509160816 | 0.762493641 |
| postsynaptic density protein 95 clustering (GO:0097119) | 44566 | 0.509160816 | 0.762493641 |
| protein deglycosylation involved in glycoprotein catabolic | 44566 | 0.509160816 | 0.762493641 |
| protein K29-linked deubiquitination (GO:0035523) | 44566 | 0.509160816 | 0.762493641 |
| protein localization to microtubule end (GO:1905725) | 44566 | 0.509160816 | 0.762493641 |
| protein localization to microtubule plus-end (GO:190482! | 44566 | 0.509160816 | 0.762493641 |
| protein localization to site of double-strand break (GO:19 | 44566 | 0.509160816 | 0.762493641 |
| pulmonary artery morphogenesis (GO:0061156) | 44566 | 0.509160816 | 0.762493641 |
| pyrimidine deoxyribonucleotide biosynthetic process (GC | 44566 | 0.509160816 | 0.762493641 |
| pyrimidine nucleoside transport (GO:0015864) | 44566 | 0.509160816 | 0.762493641 |
| pyrimidine nucleotide metabolic process (GO:0006220) | 44566 | 0.509160816 | 0.762493641 |
| receptor transactivation (GO:0035624) | 44566 | 0.509160816 | 0.762493641 |
| reelin-mediated signaling pathway (GO:0038026) | 44566 | 0.509160816 | 0.762493641 |
| regulation of adherens junction organization (GO:190339 | 44566 | 0.509160816 | 0.762493641 |
| regulation of basement membrane assembly involved in | 44566 | 0.509160816 | 0.762493641 |
| regulation of cell proliferation involved in heart morphog | 44566 | 0.509160816 | 0.762493641 |
| regulation of cellular response to vascular endothelial grc | 44566 | 0.509160816 | 0.762493641 |
| regulation of cytochrome-c oxidase activity (GO:1904959 | 44566 | 0.509160816 | 0.762493641 |
| regulation of endothelial tube morphogenesis (GO:19015 | 44566 | 0.509160816 | 0.762493641 |
| regulation of glomerular mesangial cell proliferation (GO: | 44566 | 0.509160816 | 0.762493641 |
| regulation of glycoprotein metabolic process (GO:190301 | 44566 | 0.509160816 | 0.762493641 |
| regulation of growth hormone receptor signaling pathwa | 44566 | 0.509160816 | 0.762493641 |
| regulation of guanylate cyclase activity (GO:0031282) | 44566 | 0.509160816 | 0.762493641 |
| regulation of heart growth (GO:0060420) | 44566 | 0.509160816 | 0.762493641 |
| regulation of hippocampal neuron apoptotic process (GO | 44566 | 0.509160816 | 0.762493641 |
| regulation of homophilic cell adhesion (GO:1903385) | 44566 | 0.509160816 | 0.762493641 |
| regulation of integrin biosynthetic process (GO:0045113) | 44566 | 0.509160816 | 0.762493641 |
| regulation of interleukin-1 alpha production (GO:003265( | 44566 | 0.509160816 | 0.762493641 |
| regulation of low-density lipoprotein particle receptor ca | 44566 | 0.509160816 | 0.762493641 |
| regulation of maintenance of mitotic sister chromatid col | 44566 | 0.509160816 | 0.762493641 |

| | | | |
|---|---|---|---|
| regulation of metaphase plate congression (GO:0090235) | 44566 | 0.509160816 | 0.762493641 |
| regulation of microglial cell mediated cytotoxicity (GO:19 | 44566 | 0.509160816 | 0.762493641 |
| regulation of morphogenesis of a branching structure (G( | 44566 | 0.509160816 | 0.762493641 |
| regulation of mRNA binding (GO:1902415) | 44566 | 0.509160816 | 0.762493641 |
| regulation of myoblast proliferation (GO:2000291) | 44566 | 0.509160816 | 0.762493641 |
| regulation of natural killer cell differentiation (GO:00328: | 44566 | 0.509160816 | 0.762493641 |
| regulation of neutrophil degranulation (GO:0043313) | 44566 | 0.509160816 | 0.762493641 |
| regulation of plasma membrane organization (GO:19037: | 44566 | 0.509160816 | 0.762493641 |
| regulation of protein monoubiquitination (GO:1902525) | 44566 | 0.509160816 | 0.762493641 |
| regulation of respiratory gaseous exchange by nervous sy | 44566 | 0.509160816 | 0.762493641 |
| regulation of ribonuclease activity (GO:0060700) | 44566 | 0.509160816 | 0.762493641 |
| regulation of skeletal muscle tissue development (GO:00 | 44566 | 0.509160816 | 0.762493641 |
| regulation of snRNA transcription by RNA polymerase II ( | 44566 | 0.509160816 | 0.762493641 |
| regulation of sterol transport (GO:0032371) | 44566 | 0.509160816 | 0.762493641 |
| regulation of triglyceride metabolic process (GO:0090207 | 44566 | 0.509160816 | 0.762493641 |
| renal tubule development (GO:0061326) | 44566 | 0.509160816 | 0.762493641 |
| response to cocaine (GO:0042220) | 44566 | 0.509160816 | 0.762493641 |
| response to denervation involved in regulation of muscle | 44566 | 0.509160816 | 0.762493641 |
| response to macrophage colony-stimulating factor (GO:0 | 44566 | 0.509160816 | 0.762493641 |
| response to mitochondrial depolarisation (GO:0098780) | 44566 | 0.509160816 | 0.762493641 |
| response to morphine (GO:0043278) | 44566 | 0.509160816 | 0.762493641 |
| retina vasculature development in camera-type eye (GO: | 44566 | 0.509160816 | 0.762493641 |
| ribosome localization (GO:0033750) | 44566 | 0.509160816 | 0.762493641 |
| RNA import into mitochondrion (GO:0035927) | 44566 | 0.509160816 | 0.762493641 |
| rRNA transport (GO:0051029) | 44566 | 0.509160816 | 0.762493641 |
| sensory perception of sour taste (GO:0050915) | 44566 | 0.509160816 | 0.762493641 |
| serotonin metabolic process (GO:0042428) | 44566 | 0.509160816 | 0.762493641 |
| stress granule disassembly (GO:0035617) | 44566 | 0.509160816 | 0.762493641 |
| thorax and anterior abdomen determination (GO:000735 | 44566 | 0.509160816 | 0.762493641 |
| tight junction organization (GO:0120193) | 44566 | 0.509160816 | 0.762493641 |
| tonic smooth muscle contraction (GO:0014820) | 44566 | 0.509160816 | 0.762493641 |
| trophoblast giant cell differentiation (GO:0060707) | 44566 | 0.509160816 | 0.762493641 |
| ubiquitin-dependent glycoprotein ERAD pathway (GO:00! | 44566 | 0.509160816 | 0.762493641 |
| ubiquitin-dependent SMAD protein catabolic process (GC | 44566 | 0.509160816 | 0.762493641 |
| ubiquitin-independent protein catabolic process via the r | 44566 | 0.509160816 | 0.762493641 |
| uridine transport (GO:0015862) | 44566 | 0.509160816 | 0.762493641 |
| vascular endothelial growth factor receptor-2 signaling p | 44566 | 0.509160816 | 0.762493641 |
| vesicle tethering to Golgi (GO:0099041) | 44566 | 0.509160816 | 0.762493641 |
| vitamin D3 metabolic process (GO:0070640) | 44566 | 0.509160816 | 0.762493641 |
| zygotic determination of anterior/posterior axis, embryo | 44566 | 0.509160816 | 0.762493641 |
| zymogen inhibition (GO:0097341) | 44566 | 0.509160816 | 0.762493641 |
| negative regulation of hydrolase activity (GO:0051346) | 21398 | 0.510792104 | 0.764495817 |
| RNA modification (GO:0009451) | 21398 | 0.510792104 | 0.764495817 |
| anion transport (GO:0006820) | 15858 | 0.514206222 | 0.768941084 |
| lipid modification (GO:0030258) | 15858 | 0.514206222 | 0.768941084 |
| spermatid development (GO:0007286) | 15858 | 0.514206222 | 0.768941084 |
| cellular component disassembly (GO:0022411) | 24351 | 0.519073255 | 0.773517746 |

| | | | |
|---|---|---|---|
| extracellular matrix disassembly (GO:0022617) | 24351 | 0.519073255 | 0.773517746 |
| negative regulation of G2/M transition of mitotic cell cycl | 24351 | 0.519073255 | 0.773517746 |
| amide transport (GO:0042886) | 44679 | 0.519500176 | 0.773517746 |
| calcium ion import (GO:0070509) | 44679 | 0.519500176 | 0.773517746 |
| carbohydrate derivative biosynthetic process (GO:19011: | 44679 | 0.519500176 | 0.773517746 |
| cellular zinc ion homeostasis (GO:0006882) | 44679 | 0.519500176 | 0.773517746 |
| cytosolic calcium ion transport (GO:0060401) | 44679 | 0.519500176 | 0.773517746 |
| positive regulation of synapse assembly (GO:0051965) | 44679 | 0.519500176 | 0.773517746 |
| protein localization to Golgi apparatus (GO:0034067) | 44679 | 0.519500176 | 0.773517746 |
| receptor clustering (GO:0043113) | 44679 | 0.519500176 | 0.773517746 |
| regulation of muscle contraction (GO:0006937) | 44679 | 0.519500176 | 0.773517746 |
| regulation of reactive oxygen species biosynthetic proces | 44679 | 0.519500176 | 0.773517746 |
| sulfur compound transport (GO:0072348) | 44679 | 0.519500176 | 0.773517746 |
| transcription by RNA polymerase III (GO:0006383) | 44679 | 0.519500176 | 0.773517746 |
| SCF-dependent proteasomal ubiquitin-dependent protein | 32843 | 0.522910307 | 0.778372219 |
| regulation of cellular amine metabolic process (GO:0033: | 18810 | 0.523272471 | 0.778465203 |
| sensory perception (GO:0007600) | 18810 | 0.523272471 | 0.778465203 |
| sphingolipid biosynthetic process (GO:0030148) | 27303 | 0.526468466 | 0.780118505 |
| aortic valve development (GO:0003176) | 13271 | 0.529494126 | 0.780118505 |
| dendrite morphogenesis (GO:0048813) | 13271 | 0.529494126 | 0.780118505 |
| negative regulation of DNA metabolic process (GO:00510 | 13271 | 0.529494126 | 0.780118505 |
| negative regulation of fat cell differentiation (GO:004559 | 13271 | 0.529494126 | 0.780118505 |
| regulation of androgen receptor signaling pathway (GO:0 | 13271 | 0.529494126 | 0.780118505 |
| cardiac cell development (GO:0055006) | 44605 | 0.530240316 | 0.780118505 |
| cellular component maintenance (GO:0043954) | 44605 | 0.530240316 | 0.780118505 |
| chondroitin sulfate catabolic process (GO:0030207) | 44605 | 0.530240316 | 0.780118505 |
| cytoplasmic sequestering of protein (GO:0051220) | 44605 | 0.530240316 | 0.780118505 |
| embryonic hemopoiesis (GO:0035162) | 44605 | 0.530240316 | 0.780118505 |
| Golgi ribbon formation (GO:0090161) | 44605 | 0.530240316 | 0.780118505 |
| mitochondrial outer membrane permeabilization (GO:00 | 44605 | 0.530240316 | 0.780118505 |
| muscle tissue development (GO:0060537) | 44605 | 0.530240316 | 0.780118505 |
| myelination in peripheral nervous system (GO:0022011) | 44605 | 0.530240316 | 0.780118505 |
| negative regulation of adenylate cyclase activity (GO:000 | 44605 | 0.530240316 | 0.780118505 |
| negative regulation of chromatin organization (GO:19052 | 44605 | 0.530240316 | 0.780118505 |
| negative regulation of chromatin silencing (GO:0031936) | 44605 | 0.530240316 | 0.780118505 |
| negative regulation of gene silencing (GO:0060969) | 44605 | 0.530240316 | 0.780118505 |
| negative regulation of locomotion (GO:0040013) | 44605 | 0.530240316 | 0.780118505 |
| negative regulation of macromolecule biosynthetic proce | 44605 | 0.530240316 | 0.780118505 |
| negative regulation of mononuclear cell migration (GO:0( | 44605 | 0.530240316 | 0.780118505 |
| negative regulation of myoblast differentiation (GO:0045 | 44605 | 0.530240316 | 0.780118505 |
| negative regulation of potassium ion transport (GO:0043 | 44605 | 0.530240316 | 0.780118505 |
| negative regulation of reactive oxygen species biosynthet | 44605 | 0.530240316 | 0.780118505 |
| negative regulation of stem cell differentiation (GO:2000 | 44605 | 0.530240316 | 0.780118505 |
| plasma membrane tubulation (GO:0097320) | 44605 | 0.530240316 | 0.780118505 |
| poly-N-acetyllactosamine metabolic process (GO:003030 | 44605 | 0.530240316 | 0.780118505 |
| polyamine metabolic process (GO:0006595) | 44605 | 0.530240316 | 0.780118505 |
| positive regulation of calcium ion import (GO:0090280) | 44605 | 0.530240316 | 0.780118505 |

| | | | |
|---|---|---|---|
| positive regulation of cellular carbohydrate metabolic pro | 44605 | 0.530240316 | 0.780118505 |
| pseudouridine synthesis (GO:0001522) | 44605 | 0.530240316 | 0.780118505 |
| pteridine-containing compound metabolic process (GO:0 | 44605 | 0.530240316 | 0.780118505 |
| regulation of actomyosin structure organization (GO:011( | 44605 | 0.530240316 | 0.780118505 |
| regulation of cell aging (GO:0090342) | 44605 | 0.530240316 | 0.780118505 |
| regulation of short-term neuronal synaptic plasticity (GO: | 44605 | 0.530240316 | 0.780118505 |
| regulation of tube diameter (GO:0035296) | 44605 | 0.530240316 | 0.780118505 |
| UV-damage excision repair (GO:0070914) | 44605 | 0.530240316 | 0.780118505 |
| vascular process in circulatory system (GO:0003018) | 44605 | 0.530240316 | 0.780118505 |
| ephrin receptor signaling pathway (GO:0048013) | 30256 | 0.533172586 | 0.784210527 |
| embryonic organ morphogenesis (GO:0048562) | 16224 | 0.537732935 | 0.789264492 |
| neurogenesis (GO:0022008) | 16224 | 0.537732935 | 0.789264492 |
| positive regulation of osteoblast differentiation (GO:004 | 16224 | 0.537732935 | 0.789264492 |
| positive regulation of potassium ion transmembrane tran | 16224 | 0.537732935 | 0.789264492 |
| transcription elongation from RNA polymerase II promote | 24716 | 0.538108385 | 0.789264492 |
| cellular amino acid catabolic process (GO:0009063) | 33208 | 0.539322173 | 0.789264492 |
| cyclic purine nucleotide metabolic process (GO:0052652) | 44641 | 0.5406304 | 0.789264492 |
| intracellular lipid transport (GO:0032365) | 44641 | 0.5406304 | 0.789264492 |
| mitochondrial calcium ion transmembrane transport (GO | 44641 | 0.5406304 | 0.789264492 |
| negative regulation of ion transmembrane transporter ac | 44641 | 0.5406304 | 0.789264492 |
| negative regulation of microtubule depolymerization (GC | 44641 | 0.5406304 | 0.789264492 |
| negative regulation of protein localization to cell periphe | 44641 | 0.5406304 | 0.789264492 |
| negative regulation of protein localization to plasma men | 44641 | 0.5406304 | 0.789264492 |
| negative regulation of small GTPase mediated signal tran | 44641 | 0.5406304 | 0.789264492 |
| regulation of cell-substrate junction assembly (GO:00901 | 44641 | 0.5406304 | 0.789264492 |
| regulation of mitochondrial translation (GO:0070129) | 44641 | 0.5406304 | 0.789264492 |
| response to progesterone (GO:0032570) | 44641 | 0.5406304 | 0.789264492 |
| rRNA modification (GO:0000154) | 44641 | 0.5406304 | 0.789264492 |
| stress granule assembly (GO:0034063) | 44641 | 0.5406304 | 0.789264492 |
| ameboidal-type cell migration (GO:0001667) | 19176 | 0.544838452 | 0.789264492 |
| positive regulation of developmental growth (GO:004863 | 19176 | 0.544838452 | 0.789264492 |
| RNA splicing (GO:0008380) | 13/98 | 0.545016552 | 0.789264492 |
| aggrephagy (GO:0035973) | 44680 | 0.54843063 | 0.789264492 |
| negative regulation of mRNA catabolic process (GO:1902 | 44680 | 0.54843063 | 0.789264492 |
| positive regulation of viral genome replication (GO:0045( | 44680 | 0.54843063 | 0.789264492 |
| response to zinc ion (GO:0010043) | 44680 | 0.54843063 | 0.789264492 |
| regulation of osteoblast differentiation (GO:0045667) | 30621 | 0.550191986 | 0.789264492 |
| chromosome organization (GO:0051276) | 14/106 | 0.550330184 | 0.789264492 |
| glutamate receptor signaling pathway (GO:0007215) | 13636 | 0.55498094 | 0.789264492 |
| regulation of transport (GO:0051049) | 13636 | 0.55498094 | 0.789264492 |
| skeletal muscle tissue development (GO:0007519) | 13636 | 0.55498094 | 0.789264492 |
| regulation of stem cell differentiation (GO:2000736) | 33573 | 0.555539129 | 0.789264492 |
| regulation of cell division (GO:0051302) | 28034 | 0.562103777 | 0.789264492 |
| water-soluble vitamin metabolic process (GO:0006767) | 28034 | 0.562103777 | 0.789264492 |
| regulation of DNA replication (GO:0006275) | 19541 | 0.565983645 | 0.789264492 |
| cellular protein localization (GO:0034613) | 43/329 | 0.566797621 | 0.789264492 |
| O-glycan processing (GO:0016266) | 22494 | 0.570799254 | 0.789264492 |

| | | | |
|---|---|---|---|
| bone morphogenesis (GO:0060349) | 44606 | 0.571721384 | 0.789264492 |
| C4-dicarboxylate transport (GO:0015740) | 44606 | 0.571721384 | 0.789264492 |
| calcium ion import across plasma membrane (GO:00987( | 44606 | 0.571721384 | 0.789264492 |
| cellular copper ion homeostasis (GO:0006878) | 44606 | 0.571721384 | 0.789264492 |
| ceramide transport (GO:0035627) | 44606 | 0.571721384 | 0.789264492 |
| coenzyme A metabolic process (GO:0015936) | 44606 | 0.571721384 | 0.789264492 |
| endothelial cell differentiation (GO:0045446) | 44606 | 0.571721384 | 0.789264492 |
| genitalia development (GO:0048806) | 44606 | 0.571721384 | 0.789264492 |
| membrane repolarization during action potential (GO:00 | 44606 | 0.571721384 | 0.789264492 |
| monoacylglycerol metabolic process (GO:0046462) | 44606 | 0.571721384 | 0.789264492 |
| negative regulation of cell migration involved in sproutin | 44606 | 0.571721384 | 0.789264492 |
| negative regulation of coagulation (GO:0050819) | 44606 | 0.571721384 | 0.789264492 |
| negative regulation of cyclase activity (GO:0031280) | 44606 | 0.571721384 | 0.789264492 |
| negative regulation of pri-miRNA transcription by RNA pc | 44606 | 0.571721384 | 0.789264492 |
| negative regulation of T cell mediated immunity (GO:000 | 44606 | 0.571721384 | 0.789264492 |
| negative regulation of TORC1 signaling (GO:1904262) | 44606 | 0.571721384 | 0.789264492 |
| negative regulation of translational initiation (GO:00459 | 44606 | 0.571721384 | 0.789264492 |
| neuromuscular process controlling balance (GO:0050885 | 44606 | 0.571721384 | 0.789264492 |
| nuclear pore organization (GO:0006999) | 44606 | 0.571721384 | 0.789264492 |
| nucleoside phosphate biosynthetic process (GO:1901293 | 44606 | 0.571721384 | 0.789264492 |
| nucleoside triphosphate metabolic process (GO:0009141 | 44606 | 0.571721384 | 0.789264492 |
| positive regulation of alpha-beta T cell differentiation (G( | 44606 | 0.571721384 | 0.789264492 |
| positive regulation of glucose metabolic process (GO:001 | 44606 | 0.571721384 | 0.789264492 |
| positive regulation of glycoprotein biosynthetic process ( | 44606 | 0.571721384 | 0.789264492 |
| positive regulation of macrophage chemotaxis (GO:0010 | 44606 | 0.571721384 | 0.789264492 |
| positive regulation of potassium ion transmembrane trar | 44606 | 0.571721384 | 0.789264492 |
| positive regulation of protein deacetylation (GO:0090312 | 44606 | 0.571721384 | 0.789264492 |
| positive regulation of SMAD protein signal transduction ( | 44606 | 0.571721384 | 0.789264492 |
| protein localization to vacuole (GO:0072665) | 44606 | 0.571721384 | 0.789264492 |
| pyrimidine nucleoside biosynthetic process (GO:0046134 | 44606 | 0.571721384 | 0.789264492 |
| regulation of integrin-mediated signaling pathway (GO:2( | 44606 | 0.571721384 | 0.789264492 |
| regulation of lamellipodium organization (GO:1902743) | 44606 | 0.571721384 | 0.789264492 |
| regulation of vasculogenesis (GO:2001212) | 44606 | 0.571721384 | 0.789264492 |
| ribonucleoside monophosphate biosynthetic process (GC | 44606 | 0.571721384 | 0.789264492 |
| sequestering of extracellular ligand from receptor (GO:0( | 44606 | 0.571721384 | 0.789264492 |
| tRNA wobble base modification (GO:0002097) | 44606 | 0.571721384 | 0.789264492 |
| vasodilation (GO:0042311) | 44606 | 0.571721384 | 0.789264492 |
| ribosome biogenesis (GO:0042254) | 25/192 | 0.572281691 | 0.789264492 |
| regulation of mRNA stability (GO:0043488) | 19/146 | 0.572822863 | 0.789264492 |
| cellular response to osmotic stress (GO:0071470) | 44642 | 0.573460914 | 0.789264492 |
| fatty-acyl-CoA metabolic process (GO:0035337) | 44642 | 0.573460914 | 0.789264492 |
| lipid droplet organization (GO:0034389) | 44642 | 0.573460914 | 0.789264492 |
| mRNA modification (GO:0016556) | 44642 | 0.573460914 | 0.789264492 |
| negative regulation of myeloid cell differentiation (GO:0( | 44642 | 0.573460914 | 0.789264492 |
| negative regulation of protein dephosphorylation (GO:00 | 44642 | 0.573460914 | 0.789264492 |
| negative regulation of small molecule metabolic process | 44642 | 0.573460914 | 0.789264492 |
| phosphatidylserine acyl-chain remodeling (GO:0036150) | 44642 | 0.573460914 | 0.789264492 |

| | | | |
|---|---|---|---|
| positive regulation of calcium ion transmembrane transp | 44642 | 0.573460914 | 0.789264492 |
| regulation of adenylate cyclase activity (GO:0045761) | 44642 | 0.573460914 | 0.789264492 |
| regulation of execution phase of apoptosis (GO:1900117) | 44642 | 0.573460914 | 0.789264492 |
| activation of transmembrane receptor protein tyrosine ki | 44567 | 0.574286772 | 0.789264492 |
| alditol metabolic process (GO:0019400) | 44567 | 0.574286772 | 0.789264492 |
| amino-acid betaine transport (GO:0015838) | 44567 | 0.574286772 | 0.789264492 |
| aminophospholipid transport (GO:0015917) | 44567 | 0.574286772 | 0.789264492 |
| amylin receptor signaling pathway (GO:0097647) | 44567 | 0.574286772 | 0.789264492 |
| aspartate metabolic process (GO:0006531) | 44567 | 0.574286772 | 0.789264492 |
| blood vessel endothelial cell proliferation involved in spro | 44567 | 0.574286772 | 0.789264492 |
| carnitine transport (GO:0015879) | 44567 | 0.574286772 | 0.789264492 |
| cellular hyperosmotic response (GO:0071474) | 44567 | 0.574286772 | 0.789264492 |
| cellular response to cold (GO:0070417) | 44567 | 0.574286772 | 0.789264492 |
| cellular response to granulocyte macrophage colony-stim | 44567 | 0.574286772 | 0.789264492 |
| cellular response to iron ion (GO:0071281) | 44567 | 0.574286772 | 0.789264492 |
| cellular response to X-ray (GO:0071481) | 44567 | 0.574286772 | 0.789264492 |
| cellular water homeostasis (GO:0009992) | 44567 | 0.574286772 | 0.789264492 |
| cholesterol import (GO:0070508) | 44567 | 0.574286772 | 0.789264492 |
| choline transport (GO:0015871) | 44567 | 0.574286772 | 0.789264492 |
| dermatan sulfate proteoglycan metabolic process (GO:00 | 44567 | 0.574286772 | 0.789264492 |
| diadenosine polyphosphate catabolic process (GO:00159 | 44567 | 0.574286772 | 0.789264492 |
| double-strand break repair via classical nonhomologous e | 44567 | 0.574286772 | 0.789264492 |
| embryonic hindlimb morphogenesis (GO:0035116) | 44567 | 0.574286772 | 0.789264492 |
| embryonic skeletal joint development (GO:0072498) | 44567 | 0.574286772 | 0.789264492 |
| enzyme-directed rRNA pseudouridine synthesis (GO:0000 | 44567 | 0.574286772 | 0.789264492 |
| epithelial tube branching involved in lung morphogenesis | 44567 | 0.574286772 | 0.789264492 |
| erythrose 4-phosphate/phosphoenolpyruvate family ami | 44567 | 0.574286772 | 0.789264492 |
| establishment of planar polarity (GO:0001736) | 44567 | 0.574286772 | 0.789264492 |
| foam cell differentiation (GO:0090077) | 44567 | 0.574286772 | 0.789264492 |
| free ubiquitin chain polymerization (GO:0010994) | 44567 | 0.574286772 | 0.789264492 |
| gastrulation with mouth forming second (GO:0001702) | 44567 | 0.574286772 | 0.789264492 |
| glandular epithelial cell differentiation (GO:0002067) | 44567 | 0.574286772 | 0.789264492 |
| glomerular epithelial cell differentiation (GO:0072311) | 44567 | 0.574286772 | 0.789264492 |
| glomerulus vasculature development (GO:0072012) | 44567 | 0.574286772 | 0.789264492 |
| glycerol-3-phosphate metabolic process (GO:0006072) | 44567 | 0.574286772 | 0.789264492 |
| glycolipid transport (GO:0046836) | 44567 | 0.574286772 | 0.789264492 |
| glycosylceramide catabolic process (GO:0046477) | 44567 | 0.574286772 | 0.789264492 |
| glycosylceramide metabolic process (GO:0006677) | 44567 | 0.574286772 | 0.789264492 |
| growth hormone secretion (GO:0030252) | 44567 | 0.574286772 | 0.789264492 |
| guanine nucleotide transmembrane transport (GO:19037 | 44567 | 0.574286772 | 0.789264492 |
| hepatocyte differentiation (GO:0070365) | 44567 | 0.574286772 | 0.789264492 |
| histamine metabolic process (GO:0001692) | 44567 | 0.574286772 | 0.789264492 |
| hydrogen peroxide biosynthetic process (GO:0050665) | 44567 | 0.574286772 | 0.789264492 |
| hydrogen sulfide metabolic process (GO:0070813) | 44567 | 0.574286772 | 0.789264492 |
| indolalkylamine metabolic process (GO:0006586) | 44567 | 0.574286772 | 0.789264492 |
| inner ear auditory receptor cell differentiation (GO:0042 | 44567 | 0.574286772 | 0.789264492 |
| L-serine biosynthetic process (GO:0006564) | 44567 | 0.574286772 | 0.789264492 |

| | | | |
|---|---|---|---|
| macromolecule biosynthetic process (GO:0009059) | 44567 | 0.574286772 | 0.789264492 |
| macrophage derived foam cell differentiation (GO:00107 | 44567 | 0.574286772 | 0.789264492 |
| maintenance of DNA repeat elements (GO:0043570) | 44567 | 0.574286772 | 0.789264492 |
| medium-chain fatty acid biosynthetic process (GO:00517 | 44567 | 0.574286772 | 0.789264492 |
| meiotic DNA double-strand break formation (GO:004213 | 44567 | 0.574286772 | 0.789264492 |
| mesenchymal cell differentiation involved in kidney deve | 44567 | 0.574286772 | 0.789264492 |
| mesodermal cell fate commitment (GO:0001710) | 44567 | 0.574286772 | 0.789264492 |
| metaphase/anaphase transition of cell cycle (GO:004478 | 44567 | 0.574286772 | 0.789264492 |
| metaphase/anaphase transition of mitotic cell cycle (GO: | 44567 | 0.574286772 | 0.789264492 |
| mRNA 5'-splice site recognition (GO:0000395) | 44567 | 0.574286772 | 0.789264492 |
| negative regulation of anion transport (GO:1903792) | 44567 | 0.574286772 | 0.789264492 |
| negative regulation of astrocyte differentiation (GO:0048 | 44567 | 0.574286772 | 0.789264492 |
| negative regulation of endoplasmic reticulum calcium ior | 44567 | 0.574286772 | 0.789264492 |
| negative regulation of ERAD pathway (GO:1904293) | 44567 | 0.574286772 | 0.789264492 |
| negative regulation of erythrocyte differentiation (GO:00 | 44567 | 0.574286772 | 0.789264492 |
| negative regulation of establishment of protein localizati | 44567 | 0.574286772 | 0.789264492 |
| negative regulation of excitatory postsynaptic potential ( | 44567 | 0.574286772 | 0.789264492 |
| negative regulation of fat cell proliferation (GO:0070345) | 44567 | 0.574286772 | 0.789264492 |
| negative regulation of glial cell proliferation (GO:006025 | 44567 | 0.574286772 | 0.789264492 |
| negative regulation of glucocorticoid receptor signaling p | 44567 | 0.574286772 | 0.789264492 |
| negative regulation of glycogen biosynthetic process (GO | 44567 | 0.574286772 | 0.789264492 |
| negative regulation of hematopoietic progenitor cell diffe | 44567 | 0.574286772 | 0.789264492 |
| negative regulation of histone H3-K9 methylation (GO:00 | 44567 | 0.574286772 | 0.789264492 |
| negative regulation of meiotic cell cycle (GO:0051447) | 44567 | 0.574286772 | 0.789264492 |
| negative regulation of morphogenesis of an epithelium (( | 44567 | 0.574286772 | 0.789264492 |
| negative regulation of neuron migration (GO:2001223) | 44567 | 0.574286772 | 0.789264492 |
| negative regulation of SMAD protein signal transduction | 44567 | 0.574286772 | 0.789264492 |
| negative regulation of T cell differentiation in thymus (GC | 44567 | 0.574286772 | 0.789264492 |
| negative regulation of triglyceride metabolic process (GO | 44567 | 0.574286772 | 0.789264492 |
| nephron tubule development (GO:0072080) | 44567 | 0.574286772 | 0.789264492 |
| neurotransmitter receptor transport, endosome to posts | 44567 | 0.574286772 | 0.789264492 |
| peptidyl-diphthamide biosynthetic process from peptidyl | 44567 | 0.574286772 | 0.789264492 |
| peptidyl-diphthamide metabolic process (GO:0017182) | 44567 | 0.574286772 | 0.789264492 |
| peptidyl-glutamine methylation (GO:0018364) | 44567 | 0.574286772 | 0.789264492 |
| peroxisome proliferator activated receptor signaling path | 44567 | 0.574286772 | 0.789264492 |
| piRNA biosynthetic process (GO:1990511) | 44567 | 0.574286772 | 0.789264492 |
| plasma membrane raft assembly (GO:0044854) | 44567 | 0.574286772 | 0.789264492 |
| positive regulation of activin receptor signaling pathway ( | 44567 | 0.574286772 | 0.789264492 |
| positive regulation of anion transport (GO:1903793) | 44567 | 0.574286772 | 0.789264492 |
| positive regulation of autophagosome maturation (GO:19 | 44567 | 0.574286772 | 0.789264492 |
| positive regulation of autophagy of mitochondrion in res | 44567 | 0.574286772 | 0.789264492 |
| positive regulation of cell-cell adhesion mediated by inte | 44567 | 0.574286772 | 0.789264492 |
| positive regulation of endothelial cell development (GO:1 | 44567 | 0.574286772 | 0.789264492 |
| positive regulation of epithelial cell differentiation involv | 44567 | 0.574286772 | 0.789264492 |
| positive regulation of establishment of endothelial barrie | 44567 | 0.574286772 | 0.789264492 |
| positive regulation of extrinsic apoptotic signaling pathwa | 44567 | 0.574286772 | 0.789264492 |
| positive regulation of ion transmembrane transport (GO: | 44567 | 0.574286772 | 0.789264492 |

| | | | |
|---|---|---|---|
| positive regulation of lipoprotein particle clearance (GO:( | 44567 | 0.574286772 | 0.789264492 |
| positive regulation of microglial cell activation (GO:19039 | 44567 | 0.574286772 | 0.789264492 |
| positive regulation of neurotransmitter secretion (GO:00( | 44567 | 0.574286772 | 0.789264492 |
| positive regulation of protein glycosylation (GO:0060050) | 44567 | 0.574286772 | 0.789264492 |
| positive regulation of RNA binding (GO:1905216) | 44567 | 0.574286772 | 0.789264492 |
| positive regulation of skeletal muscle fiber development | 44567 | 0.574286772 | 0.789264492 |
| positive regulation of toll-like receptor 2 signaling pathwa | 44567 | 0.574286772 | 0.789264492 |
| positive regulation of Wnt signaling pathway, planar cell | | 44567 | 0.574286772 | 0.789264492 |
| postsynaptic density assembly (GO:0097107) | 44567 | 0.574286772 | 0.789264492 |
| presynaptic active zone organization (GO:1990709) | 44567 | 0.574286772 | 0.789264492 |
| pronephros development (GO:0048793) | 44567 | 0.574286772 | 0.789264492 |
| protection from natural killer cell mediated cytotoxicity ( | 44567 | 0.574286772 | 0.789264492 |
| protein deubiquitination involved in ubiquitin-dependent | 44567 | 0.574286772 | 0.789264492 |
| protein K27-linked ubiquitination (GO:0044314) | 44567 | 0.574286772 | 0.789264492 |
| protein localization to non-motile cilium (GO:0097499) | 44567 | 0.574286772 | 0.789264492 |
| protein localization to perinuclear region of cytoplasm (G | 44567 | 0.574286772 | 0.789264492 |
| protein repair (GO:0030091) | 44567 | 0.574286772 | 0.789264492 |
| protein-DNA covalent cross-linking repair (GO:0106300) | 44567 | 0.574286772 | 0.789264492 |
| proton-transporting V-type ATPase complex assembly (G | 44567 | 0.574286772 | 0.789264492 |
| purine deoxyribonucleotide catabolic process (GO:00091 | 44567 | 0.574286772 | 0.789264492 |
| regulation of 3'-UTR-mediated mRNA stabilization (GO:19 | 44567 | 0.574286772 | 0.789264492 |
| regulation of digestive system process (GO:0044058) | 44567 | 0.574286772 | 0.789264492 |
| regulation of endoplasmic reticulum tubular network org | 44567 | 0.574286772 | 0.789264492 |
| regulation of endothelial cell-matrix adhesion via fibrone | 44567 | 0.574286772 | 0.789264492 |
| regulation of excitatory synapse assembly (GO:1904889) | 44567 | 0.574286772 | 0.789264492 |
| regulation of glomerular filtration (GO:0003093) | 44567 | 0.574286772 | 0.789264492 |
| regulation of glucagon secretion (GO:0070092) | 44567 | 0.574286772 | 0.789264492 |
| regulation of glucokinase activity (GO:0033131) | 44567 | 0.574286772 | 0.789264492 |
| regulation of glutamate receptor signaling pathway (GO:1 | 44567 | 0.574286772 | 0.789264492 |
| regulation of hepatocyte proliferation (GO:2000345) | 44567 | 0.574286772 | 0.789264492 |
| regulation of hyaluronan biosynthetic process (GO:19001 | 44567 | 0.574286772 | 0.789264492 |
| regulation of hydrogen peroxide metabolic process (GO:( | 44567 | 0.574286772 | 0.789264492 |
| regulation of long-chain fatty acid import across plasma r | 44567 | 0.574286772 | 0.789264492 |
| regulation of macrophage proliferation (GO:0120040) | 44567 | 0.574286772 | 0.789264492 |
| regulation of mitotic sister chromatid segregation (GO:0( | 44567 | 0.574286772 | 0.789264492 |
| regulation of mitotic spindle checkpoint (GO:1903504) | 44567 | 0.574286772 | 0.789264492 |
| regulation of mRNA export from nucleus (GO:0010793) | 44567 | 0.574286772 | 0.789264492 |
| regulation of nitric oxide mediated signal transduction (G | 44567 | 0.574286772 | 0.789264492 |
| regulation of nuclease activity (GO:0032069) | 44567 | 0.574286772 | 0.789264492 |
| regulation of protein depolymerization (GO:1901879) | 44567 | 0.574286772 | 0.789264492 |
| regulation of protein lipidation (GO:1903059) | 44567 | 0.574286772 | 0.789264492 |
| regulation of relaxation of cardiac muscle (GO:1901897) | 44567 | 0.574286772 | 0.789264492 |
| regulation of retrograde transport, endosome to Golgi (G | 44567 | 0.574286772 | 0.789264492 |
| regulation of ribonucleoprotein complex localization (GO | 44567 | 0.574286772 | 0.789264492 |
| regulation of saliva secretion (GO:0046877) | 44567 | 0.574286772 | 0.789264492 |
| regulation of SNARE complex assembly (GO:0035542) | 44567 | 0.574286772 | 0.789264492 |
| regulation of T-helper cell differentiation (GO:0045622) | 44567 | 0.574286772 | 0.789264492 |

| | | | |
|---|---|---|---|
| regulation of thymocyte apoptotic process (GO:0070243) | 44567 | 0.574286772 | 0.789264492 |
| regulation of tooth mineralization (GO:0070170) | 44567 | 0.574286772 | 0.789264492 |
| regulation of translational elongation (GO:0006448) | 44567 | 0.574286772 | 0.789264492 |
| regulation of transmission of nerve impulse (GO:005196 | 44567 | 0.574286772 | 0.789264492 |
| regulation of ventricular cardiac muscle cell membrane d | 44567 | 0.574286772 | 0.789264492 |
| regulation of very-low-density lipoprotein particle remod | 44567 | 0.574286772 | 0.789264492 |
| renal filtration cell differentiation (GO:0061318) | 44567 | 0.574286772 | 0.789264492 |
| response to catecholamine (GO:0071869) | 44567 | 0.574286772 | 0.789264492 |
| response to granulocyte macrophage colony-stimulating | 44567 | 0.574286772 | 0.789264492 |
| response to heparin (GO:0071503) | 44567 | 0.574286772 | 0.789264492 |
| response to manganese ion (GO:0010042) | 44567 | 0.574286772 | 0.789264492 |
| response to testosterone (GO:0033574) | 44567 | 0.574286772 | 0.789264492 |
| snoRNA localization (GO:0048254) | 44567 | 0.574286772 | 0.789264492 |
| sterol import (GO:0035376) | 44567 | 0.574286772 | 0.789264492 |
| succinate transmembrane transport (GO:0071422) | 44567 | 0.574286772 | 0.789264492 |
| T-tubule organization (GO:0033292) | 44567 | 0.574286772 | 0.789264492 |
| telencephalon cell migration (GO:0022029) | 44567 | 0.574286772 | 0.789264492 |
| trigeminal nerve development (GO:0021559) | 44567 | 0.574286772 | 0.789264492 |
| type B pancreatic cell proliferation (GO:0044342) | 44567 | 0.574286772 | 0.789264492 |
| venous blood vessel morphogenesis (GO:0048845) | 44567 | 0.574286772 | 0.789264492 |
| ventricular cardiac muscle cell development (GO:005501 | 44567 | 0.574286772 | 0.789264492 |
| ventricular compact myocardium morphogenesis (GO:00 | 44567 | 0.574286772 | 0.789264492 |
| xenobiotic transport across blood-brain barrier (GO:1990 | 44567 | 0.574286772 | 0.789264492 |
| DNA-templated transcription, elongation (GO:0006354) | 25447 | 0.575287372 | 0.790313242 |
| organic acid transport (GO:0015849) | 13/100 | 0.57584131 | 0.790313242 |
| cardiac muscle cell action potential (GO:0086001) | 44681 | 0.576419032 | 0.790313242 |
| microtubule polymerization (GO:0046785) | 44681 | 0.576419032 | 0.790313242 |
| morphogenesis of an epithelium (GO:0002009) | 44681 | 0.576419032 | 0.790313242 |
| ncRNA catabolic process (GO:0034661) | 44681 | 0.576419032 | 0.790313242 |
| negative regulation of Notch signaling pathway (GO:0045 | 44681 | 0.576419032 | 0.790313242 |
| peptidyl-lysine trimethylation (GO:0018023) | 44681 | 0.576419032 | 0.790313242 |
| positive regulation of DNA recombination (GO:0045911) | 44681 | 0.576419032 | 0.790313242 |
| purine ribonucleotide metabolic process (GO:0009150) | 28399 | 0.579506883 | 0.794064683 |
| positive regulation of potassium ion transport (GO:00432 | 14001 | 0.579766572 | 0.794064683 |
| tRNA aminoacylation (GO:0043039) | 14001 | 0.579766572 | 0.794064683 |
| ventricular septum development (GO:0003281) | 14001 | 0.579766572 | 0.794064683 |
| rRNA metabolic process (GO:0016072) | 21/162 | 0.580464654 | 0.794811249 |
| establishment of protein localization to extracellular regi | 16954 | 0.583219617 | 0.79816278 |
| regulation of DNA metabolic process (GO:0051052) | 16954 | 0.583219617 | 0.79816278 |
| Wnt signaling pathway, planar cell polarity pathway (GO: | 31352 | 0.583500553 | 0.798336943 |
| regulation of alternative mRNA splicing, via spliceosome | 19906 | 0.586665203 | 0.801822081 |
| regulation of cellular amino acid metabolic process (GO:0 | 19906 | 0.586665203 | 0.801822081 |
| regulation of proteasomal protein catabolic process (GO: | 19906 | 0.586665203 | 0.801822081 |
| unsaturated fatty acid metabolic process (GO:0033559) | 19906 | 0.586665203 | 0.801822081 |
| regulation of heart contraction (GO:0008016) | 34304 | 0.587300666 | 0.802268462 |
| visual perception (GO:0007601) | 34304 | 0.587300666 | 0.802268462 |
| fatty acid metabolic process (GO:0006631) | 16/124 | 0.587520885 | 0.802358306 |

| | | | |
|---|---|---|---|
| positive regulation of cell cycle process (GO:0090068) | 13/101 | 0.590932339 | 0.806805125 |
| DNA-templated transcription, termination (GO:0006353) | 25812 | 0.593368984 | 0.809706296 |
| sterol metabolic process (GO:0016125) | 25812 | 0.593368984 | 0.809706296 |
| negative regulation of cellular amide metabolic process (( | 31717 | 0.599743958 | 0.815751343 |
| phosphatidylethanolamine metabolic process (GO:00463 | 11414 | 0.603392107 | 0.815751343 |
| positive regulation of locomotion (GO:0040017) | 11414 | 0.603392107 | 0.815751343 |
| positive regulation of ubiquitin-protein transferase activit | 11414 | 0.603392107 | 0.815751343 |
| proteoglycan biosynthetic process (GO:0030166) | 11414 | 0.603392107 | 0.815751343 |
| regulation of calcium ion-dependent exocytosis (GO:0017 | 11414 | 0.603392107 | 0.815751343 |
| renal water homeostasis (GO:0003091) | 11414 | 0.603392107 | 0.815751343 |
| regulation of ion transmembrane transporter activity (GC | 14366 | 0.603793488 | 0.815751343 |
| cilium-dependent cell motility (GO:0060285) | 44643 | 0.604784161 | 0.815751343 |
| cytoskeleton-dependent intracellular transport (GO:0030 | 44643 | 0.604784161 | 0.815751343 |
| ERK1 and ERK2 cascade (GO:0070371) | 44643 | 0.604784161 | 0.815751343 |
| mitotic cell cycle checkpoint signaling (GO:0007093) | 44643 | 0.604784161 | 0.815751343 |
| negative regulation of GTPase activity (GO:0034260) | 44643 | 0.604784161 | 0.815751343 |
| oligosaccharide biosynthetic process (GO:0009312) | 44643 | 0.604784161 | 0.815751343 |
| positive regulation of histone acetylation (GO:0035066) | 44643 | 0.604784161 | 0.815751343 |
| positive regulation of transcription initiation from RNA po | 44643 | 0.604784161 | 0.815751343 |
| protein deacylation (GO:0035601) | 44643 | 0.604784161 | 0.815751343 |
| regulation of calcium ion transmembrane transport (GO:1 | 44643 | 0.604784161 | 0.815751343 |
| regulation of neural precursor cell proliferation (GO:2000 | 44643 | 0.604784161 | 0.815751343 |
| regulation of peptidase activity (GO:0052547) | 44643 | 0.604784161 | 0.815751343 |
| regulation of release of sequestered calcium ion into cyto | 44643 | 0.604784161 | 0.815751343 |
| regulation of SMAD protein signal transduction (GO:0060 | 44643 | 0.604784161 | 0.815751343 |
| regulation of steroid metabolic process (GO:0019218) | 44643 | 0.604784161 | 0.815751343 |
| response to copper ion (GO:0046688) | 44643 | 0.604784161 | 0.815751343 |
| regulation of cellular metabolic process (GO:0031323) | 17319 | 0.605080174 | 0.815751343 |
| triglyceride metabolic process (GO:0006641) | 20271 | 0.606845431 | 0.815751343 |
| negative regulation of endopeptidase activity (GO:00109 | 23224 | 0.608882159 | 0.815751343 |
| positive regulation of viral process (GO:0048524) | 23224 | 0.608882159 | 0.815751343 |
| acetyl-CoA metabolic process (GO:0006084) | 44607 | 0.610467888 | 0.815751343 |
| C21-steroid hormone biosynthetic process (GO:0006700) | 44607 | 0.610467888 | 0.815751343 |
| calcium ion import into cytosol (GO:1902656) | 44607 | 0.610467888 | 0.815751343 |
| cardiac myofibril assembly (GO:0055003) | 44607 | 0.610467888 | 0.815751343 |
| clathrin coat assembly (GO:0048268) | 44607 | 0.610467888 | 0.815751343 |
| dermatan sulfate proteoglycan biosynthetic process (GO: | 44607 | 0.610467888 | 0.815751343 |
| embryonic cranial skeleton morphogenesis (GO:0048701 | 44607 | 0.610467888 | 0.815751343 |
| mammary gland epithelium development (GO:0061180) | 44607 | 0.610467888 | 0.815751343 |
| membrane repolarization during cardiac muscle cell actic | 44607 | 0.610467888 | 0.815751343 |
| mitochondrial RNA metabolic process (GO:0000959) | 44607 | 0.610467888 | 0.815751343 |
| negative regulation of androgen receptor signaling pathw | 44607 | 0.610467888 | 0.815751343 |
| negative regulation of blood pressure (GO:0045776) | 44607 | 0.610467888 | 0.815751343 |
| negative regulation of protein processing (GO:0010955) | 44607 | 0.610467888 | 0.815751343 |
| nucleosome positioning (GO:0016584) | 44607 | 0.610467888 | 0.815751343 |
| placenta development (GO:0001890) | 44607 | 0.610467888 | 0.815751343 |
| positive regulation of keratinocyte differentiation (GO:00 | 44607 | 0.610467888 | 0.815751343 |

| | | | |
|---|---|---|---|
| positive regulation of lyase activity (GO:0051349) | 44607 | 0.610467888 | 0.815751343 |
| positive regulation of protein export from nucleus (GO:0( | 44607 | 0.610467888 | 0.815751343 |
| positive regulation of secretion (GO:0051047) | 44607 | 0.610467888 | 0.815751343 |
| protein hydroxylation (GO:0018126) | 44607 | 0.610467888 | 0.815751343 |
| regulation of aerobic respiration (GO:1903715) | 44607 | 0.610467888 | 0.815751343 |
| regulation of amyloid fibril formation (GO:1905906) | 44607 | 0.610467888 | 0.815751343 |
| regulation of endothelial cell chemotaxis (GO:2001026) | 44607 | 0.610467888 | 0.815751343 |
| regulation of lymphocyte activation (GO:0051249) | 44607 | 0.610467888 | 0.815751343 |
| regulation of sequestering of triglyceride (GO:0010889) | 44607 | 0.610467888 | 0.815751343 |
| regulation of synaptic transmission, GABAergic (GO:0032 | 44607 | 0.610467888 | 0.815751343 |
| response to ethanol (GO:0045471) | 44607 | 0.610467888 | 0.815751343 |
| cellular response to BMP stimulus (GO:0071773) | 26177 | 0.6110754 | 0.815751343 |
| fatty acid biosynthetic process (GO:0006633) | 26177 | 0.6110754 | 0.815751343 |
| response to metal ion (GO:0010038) | 26177 | 0.6110754 | 0.815751343 |
| regulation of neuron differentiation (GO:0045664) | 29129 | 0.613357321 | 0.815751343 |
| ciliary basal body-plasma membrane docking (GO:00977: | 35034 | 0.618035005 | 0.815751343 |
| regulation of cell cycle phase transition (GO:1901987) | 35034 | 0.618035005 | 0.815751343 |
| sensory perception of light stimulus (GO:0050953) | 35034 | 0.618035005 | 0.815751343 |
| rRNA processing (GO:0006364) | 22/173 | 0.618429881 | 0.815751343 |
| regulation of Wnt signaling pathway (GO:0030111) | 14/111 | 0.622729728 | 0.815751343 |
| histone H4 acetylation (GO:0043967) | 17685 | 0.626297871 | 0.815751343 |
| phosphatidic acid biosynthetic process (GO:0006654) | 17685 | 0.626297871 | 0.815751343 |
| Rho protein signal transduction (GO:0007266) | 20637 | 0.626491678 | 0.815751343 |
| digestive tract development (GO:0048565) | 14732 | 0.627014684 | 0.815751343 |
| nucleocytoplasmic transport (GO:0006913) | 14732 | 0.627014684 | 0.815751343 |
| ribosomal small subunit biogenesis (GO:0042274) | 14732 | 0.627014684 | 0.815751343 |
| sulfur compound catabolic process (GO:0044273) | 14732 | 0.627014684 | 0.815751343 |
| synaptic vesicle exocytosis (GO:0016079) | 14732 | 0.627014684 | 0.815751343 |
| antimicrobial humoral immune response mediated by an | 23590 | 0.627252588 | 0.815751343 |
| B cell proliferation (GO:0042100) | 11780 | 0.629293652 | 0.815751343 |
| endodermal cell differentiation (GO:0035987) | 11780 | 0.629293652 | 0.815751343 |
| negative regulation of double-strand break repair (GO:20 | 11780 | 0.629293652 | 0.815751343 |
| negative regulation of microtubule polymerization or dep | 11780 | 0.629293652 | 0.815751343 |
| positive regulation of glucose transmembrane transport ( | 11780 | 0.629293652 | 0.815751343 |
| positive regulation of telomere maintenance (GO:00322( | 11780 | 0.629293652 | 0.815751343 |
| regulation of cell morphogenesis (GO:0022604) | 11780 | 0.629293652 | 0.815751343 |
| skeletal muscle organ development (GO:0060538) | 11780 | 0.629293652 | 0.815751343 |
| negative regulation of cellular response to growth factor | 29495 | 0.629760957 | 0.815751343 |
| acylglycerol acyl-chain remodeling (GO:0036155) | 44568 | 0.630774426 | 0.815751343 |
| adenylate cyclase-activating dopamine receptor signaling | 44568 | 0.630774426 | 0.815751343 |
| aldehyde catabolic process (GO:0046185) | 44568 | 0.630774426 | 0.815751343 |
| atrioventricular valve formation (GO:0003190) | 44568 | 0.630774426 | 0.815751343 |
| AV node cell action potential (GO:0086016) | 44568 | 0.630774426 | 0.815751343 |
| axon ensheathment in central nervous system (GO:0032: | 44568 | 0.630774426 | 0.815751343 |
| calcitonin family receptor signaling pathway (GO:009764( | 44568 | 0.630774426 | 0.815751343 |
| cellular response to prostaglandin E stimulus (GO:00713{ | 44568 | 0.630774426 | 0.815751343 |
| chylomicron remnant clearance (GO:0034382) | 44568 | 0.630774426 | 0.815751343 |

| | | | |
|---|---|---|---|
| citrulline metabolic process (GO:0000052) | 44568 | 0.630774426 | 0.815751343 |
| collagen-activated tyrosine kinase receptor signaling path | 44568 | 0.630774426 | 0.815751343 |
| creatine metabolic process (GO:0006600) | 44568 | 0.630774426 | 0.815751343 |
| cyclic nucleotide transport (GO:0070729) | 44568 | 0.630774426 | 0.815751343 |
| detection of muscle stretch (GO:0035995) | 44568 | 0.630774426 | 0.815751343 |
| detection of stimulus involved in sensory perception of p | 44568 | 0.630774426 | 0.815751343 |
| DNA ligation (GO:0006266) | 44568 | 0.630774426 | 0.815751343 |
| exocrine system development (GO:0035272) | 44568 | 0.630774426 | 0.815751343 |
| flavin-containing compound metabolic process (GO:0042 | 44568 | 0.630774426 | 0.815751343 |
| G protein-coupled receptor internalization (GO:0002031) | 44568 | 0.630774426 | 0.815751343 |
| ganglioside catabolic process (GO:0006689) | 44568 | 0.630774426 | 0.815751343 |
| glandular epithelial cell development (GO:0002068) | 44568 | 0.630774426 | 0.815751343 |
| glycosylceramide biosynthetic process (GO:0046476) | 44568 | 0.630774426 | 0.815751343 |
| Golgi disassembly (GO:0090166) | 44568 | 0.630774426 | 0.815751343 |
| heme transport (GO:0015886) | 44568 | 0.630774426 | 0.815751343 |
| hexose catabolic process (GO:0019320) | 44568 | 0.630774426 | 0.815751343 |
| hindlimb morphogenesis (GO:0035137) | 44568 | 0.630774426 | 0.815751343 |
| histone H3-K9 methylation (GO:0051567) | 44568 | 0.630774426 | 0.815751343 |
| inhibitory postsynaptic potential (GO:0060080) | 44568 | 0.630774426 | 0.815751343 |
| keratinocyte proliferation (GO:0043616) | 44568 | 0.630774426 | 0.815751343 |
| late nucleophagy (GO:0044805) | 44568 | 0.630774426 | 0.815751343 |
| long-term synaptic depression (GO:0060292) | 44568 | 0.630774426 | 0.815751343 |
| maintenance of protein location in extracellular region (G | 44568 | 0.630774426 | 0.815751343 |
| malate metabolic process (GO:0006108) | 44568 | 0.630774426 | 0.815751343 |
| miRNA catabolic process (GO:0010587) | 44568 | 0.630774426 | 0.815751343 |
| modulation by symbiont of host apoptotic process (GO:0 | 44568 | 0.630774426 | 0.815751343 |
| negative regulation of centriole replication (GO:0046600) | 44568 | 0.630774426 | 0.815751343 |
| negative regulation of cytoplasmic translation (GO:20007 | 44568 | 0.630774426 | 0.815751343 |
| negative regulation of dendritic spine development (GO:( | 44568 | 0.630774426 | 0.815751343 |
| negative regulation of DNA repair (GO:0045738) | 44568 | 0.630774426 | 0.815751343 |
| negative regulation of double-strand break repair via nor | 44568 | 0.630774426 | 0.815751343 |
| negative regulation of epidermal cell differentiation (GO: | 44568 | 0.630774426 | 0.815751343 |
| negative regulation of glycogen metabolic process (GO:0( | 44568 | 0.630774426 | 0.815751343 |
| negative regulation of heart rate (GO:0010459) | 44568 | 0.630774426 | 0.815751343 |
| negative regulation of intracellular transport (GO:003238 | 44568 | 0.630774426 | 0.815751343 |
| negative regulation of lymphocyte differentiation (GO:00 | 44568 | 0.630774426 | 0.815751343 |
| negative regulation of meiotic nuclear division (GO:0045{ | 44568 | 0.630774426 | 0.815751343 |
| negative regulation of mitochondrial outer membrane pe | 44568 | 0.630774426 | 0.815751343 |
| negative regulation of NLRP3 inflammasome complex ass | 44568 | 0.630774426 | 0.815751343 |
| negative regulation of protein import (GO:1904590) | 44568 | 0.630774426 | 0.815751343 |
| negative regulation of protein import into nucleus (GO:0( | 44568 | 0.630774426 | 0.815751343 |
| negative regulation of protein kinase activity by regulatio | 44568 | 0.630774426 | 0.815751343 |
| negative regulation of RIG-I signaling pathway (GO:00395 | 44568 | 0.630774426 | 0.815751343 |
| negative regulation of striated muscle tissue developmer | 44568 | 0.630774426 | 0.815751343 |
| neurotransmitter receptor transport, endosome to plasm | 44568 | 0.630774426 | 0.815751343 |
| norepinephrine metabolic process (GO:0042415) | 44568 | 0.630774426 | 0.815751343 |
| nose development (GO:0043584) | 44568 | 0.630774426 | 0.815751343 |

| | | | |
|---|---|---|---|
| Notch receptor processing, ligand-dependent (GO:00353 | 44568 | 0.630774426 | 0.815751343 |
| nuclear-transcribed mRNA catabolic process, deadenylati | 44568 | 0.630774426 | 0.815751343 |
| oxygen transport (GO:0015671) | 44568 | 0.630774426 | 0.815751343 |
| pantothenate metabolic process (GO:0015939) | 44568 | 0.630774426 | 0.815751343 |
| pentose metabolic process (GO:0019321) | 44568 | 0.630774426 | 0.815751343 |
| pexophagy (GO:0000425) | 44568 | 0.630774426 | 0.815751343 |
| pharyngeal arch artery morphogenesis (GO:0061626) | 44568 | 0.630774426 | 0.815751343 |
| photoreceptor cell differentiation (GO:0046530) | 44568 | 0.630774426 | 0.815751343 |
| platelet dense granule organization (GO:0060155) | 44568 | 0.630774426 | 0.815751343 |
| polyamine biosynthetic process (GO:0006596) | 44568 | 0.630774426 | 0.815751343 |
| positive regulation by symbiont of host autophagy (GO:0 | 44568 | 0.630774426 | 0.815751343 |
| positive regulation of adenylate cyclase activity (GO:004 | 44568 | 0.630774426 | 0.815751343 |
| positive regulation of cAMP-mediated signaling (GO:004 | 44568 | 0.630774426 | 0.815751343 |
| positive regulation of cardiac epithelial to mesenchymal | 44568 | 0.630774426 | 0.815751343 |
| positive regulation of catecholamine secretion (GO:0033 | 44568 | 0.630774426 | 0.815751343 |
| positive regulation of histone H4 acetylation (GO:009024 | 44568 | 0.630774426 | 0.815751343 |
| positive regulation of intracellular estrogen receptor sign | 44568 | 0.630774426 | 0.815751343 |
| positive regulation of microtubule nucleation (GO:00900 | 44568 | 0.630774426 | 0.815751343 |
| positive regulation of mitophagy in response to mitochor | 44568 | 0.630774426 | 0.815751343 |
| positive regulation of mRNA binding (GO:1902416) | 44568 | 0.630774426 | 0.815751343 |
| positive regulation of multicellular organism growth (GO: | 44568 | 0.630774426 | 0.815751343 |
| positive regulation of phospholipid biosynthetic process ( | 44568 | 0.630774426 | 0.815751343 |
| positive regulation of potassium ion import across plasm | 44568 | 0.630774426 | 0.815751343 |
| positive regulation of striated muscle contraction (GO:00 | 44568 | 0.630774426 | 0.815751343 |
| positive regulation of synaptic plasticity (GO:0031915) | 44568 | 0.630774426 | 0.815751343 |
| positive regulation of vascular associated smooth muscle | 44568 | 0.630774426 | 0.815751343 |
| proline metabolic process (GO:0006560) | 44568 | 0.630774426 | 0.815751343 |
| protection from non-homologous end joining at telomere | 44568 | 0.630774426 | 0.815751343 |
| protein localization to ciliary membrane (GO:1903441) | 44568 | 0.630774426 | 0.815751343 |
| protein localization to cytoplasmic stress granule (GO:19 | 44568 | 0.630774426 | 0.815751343 |
| purine ribonucleoside metabolic process (GO:0046128) | 44568 | 0.630774426 | 0.815751343 |
| regulation of bile acid metabolic process (GO:1904251) | 44568 | 0.630774426 | 0.815751343 |
| regulation of blood-brain barrier permeability (GO:19056 | 44568 | 0.630774426 | 0.815751343 |
| regulation of cardiac muscle cell membrane potential (G | 44568 | 0.630774426 | 0.815751343 |
| regulation of CD8-positive, alpha-beta T cell differentiatic | 44568 | 0.630774426 | 0.815751343 |
| regulation of cGMP-mediated signaling (GO:0010752) | 44568 | 0.630774426 | 0.815751343 |
| regulation of cytoplasmic mRNA processing body assemb | 44568 | 0.630774426 | 0.815751343 |
| regulation of dopamine metabolic process (GO:0042053) | 44568 | 0.630774426 | 0.815751343 |
| regulation of fat cell proliferation (GO:0070344) | 44568 | 0.630774426 | 0.815751343 |
| regulation of glial cell differentiation (GO:0045685) | 44568 | 0.630774426 | 0.815751343 |
| regulation of glucocorticoid receptor signaling pathway ( | 44568 | 0.630774426 | 0.815751343 |
| regulation of glycogen (starch) synthase activity (GO:200 | 44568 | 0.630774426 | 0.815751343 |
| regulation of lysosomal protein catabolic process (GO:19 | 44568 | 0.630774426 | 0.815751343 |
| regulation of mitochondrial membrane permeability invo | 44568 | 0.630774426 | 0.815751343 |
| regulation of morphogenesis of an epithelium (GO:19053 | 44568 | 0.630774426 | 0.815751343 |
| regulation of norepinephrine secretion (GO:0014061) | 44568 | 0.630774426 | 0.815751343 |
| regulation of phospholipid transport (GO:2001138) | 44568 | 0.630774426 | 0.815751343 |

| | | | |
|---|---|---|---|
| regulation of Rab protein signal transduction (GO:00324ε | 44568 | 0.630774426 | 0.815751343 |
| regulation of respiratory system process (GO:0044065) | 44568 | 0.630774426 | 0.815751343 |
| regulation of skeletal muscle satellite cell proliferation (G | 44568 | 0.630774426 | 0.815751343 |
| regulation of smooth muscle cell differentiation (GO:005: | 44568 | 0.630774426 | 0.815751343 |
| regulation of T cell mediated immune response to tumor | 44568 | 0.630774426 | 0.815751343 |
| regulation of T cell tolerance induction (GO:0002664) | 44568 | 0.630774426 | 0.815751343 |
| regulation of transcription from RNA polymerase II prom | 44568 | 0.630774426 | 0.815751343 |
| regulation of transforming growth factor beta activation | 44568 | 0.630774426 | 0.815751343 |
| regulation of tubulin deacetylation (GO:0090043) | 44568 | 0.630774426 | 0.815751343 |
| regulation of vesicle size (GO:0097494) | 44568 | 0.630774426 | 0.815751343 |
| response to alkaloid (GO:0043279) | 44568 | 0.630774426 | 0.815751343 |
| riboflavin metabolic process (GO:0006771) | 44568 | 0.630774426 | 0.815751343 |
| rRNA-containing ribonucleoprotein complex export from | 44568 | 0.630774426 | 0.815751343 |
| serine transport (GO:0032329) | 44568 | 0.630774426 | 0.815751343 |
| sinoatrial node development (GO:0003163) | 44568 | 0.630774426 | 0.815751343 |
| SMAD protein complex assembly (GO:0007183) | 44568 | 0.630774426 | 0.815751343 |
| somatic recombination of immunoglobulin gene segment | 44568 | 0.630774426 | 0.815751343 |
| synaptic transmission, dopaminergic (GO:0001963) | 44568 | 0.630774426 | 0.815751343 |
| synaptic vesicle docking (GO:0016081) | 44568 | 0.630774426 | 0.815751343 |
| thiamine-containing compound metabolic process (GO:0( | 44568 | 0.630774426 | 0.815751343 |
| thyroid hormone transport (GO:0070327) | 44568 | 0.630774426 | 0.815751343 |
| TRAIL-activated apoptotic signaling pathway (GO:003646 | 44568 | 0.630774426 | 0.815751343 |
| type B pancreatic cell development (GO:0003323) | 44568 | 0.630774426 | 0.815751343 |
| vascular wound healing (GO:0061042) | 44568 | 0.630774426 | 0.815751343 |
| zinc ion import across plasma membrane (GO:0071578) | 44568 | 0.630774426 | 0.815751343 |
| regulation of establishment of planar polarity (GO:00901 | 32448 | 0.63130875 | 0.816239163 |
| carbohydrate biosynthetic process (GO:0016051) | 44644 | 0.634539739 | 0.818989799 |
| cardiac muscle cell development (GO:0055013) | 44644 | 0.634539739 | 0.818989799 |
| lymphocyte activation involved in immune response (GO | 44644 | 0.634539739 | 0.818989799 |
| negative regulation of stress fiber assembly (GO:0051497 | 44644 | 0.634539739 | 0.818989799 |
| positive regulation of protein targeting to membrane (GC | 44644 | 0.634539739 | 0.818989799 |
| protein exit from endoplasmic reticulum (GO:0032527) | 44644 | 0.634539739 | 0.818989799 |
| regulation of ruffle assembly (GO:1900027) | 44644 | 0.634539739 | 0.818989799 |
| BMP signaling pathway (GO:0030509) | 23955 | 0.645142429 | 0.828689627 |
| dicarboxylic acid metabolic process (GO:0043648) | 21002 | 0.645576197 | 0.828689627 |
| negative regulation of cell cycle G2/M phase transition (G | 21002 | 0.645576197 | 0.828689627 |
| renal system development (GO:0072001) | 21002 | 0.645576197 | 0.828689627 |
| basement membrane organization (GO:0071711) | 44608 | 0.646475171 | 0.828689627 |
| body fluid secretion (GO:0007589) | 44608 | 0.646475171 | 0.828689627 |
| histone H2A acetylation (GO:0043968) | 44608 | 0.646475171 | 0.828689627 |
| late endosome to vacuole transport via multivesicular bo | 44608 | 0.646475171 | 0.828689627 |
| N-terminal protein amino acid acetylation (GO:0006474) | 44608 | 0.646475171 | 0.828689627 |
| negative regulation of DNA replication (GO:0008156) | 44608 | 0.646475171 | 0.828689627 |
| negative regulation of lyase activity (GO:0051350) | 44608 | 0.646475171 | 0.828689627 |
| negative regulation of natural killer cell mediated cytoto› | 44608 | 0.646475171 | 0.828689627 |
| negative regulation of platelet activation (GO:0010544) | 44608 | 0.646475171 | 0.828689627 |
| neuron projection organization (GO:0106027) | 44608 | 0.646475171 | 0.828689627 |

| | | | |
|---|---|---|---|
| nuclear transport (GO:0051169) | 44608 | 0.646475171 | 0.828689627 |
| oogenesis (GO:0048477) | 44608 | 0.646475171 | 0.828689627 |
| positive regulation of small molecule metabolic process ( | 44608 | 0.646475171 | 0.828689627 |
| regulation of activin receptor signaling pathway (GO:003: | 44608 | 0.646475171 | 0.828689627 |
| regulation of fatty acid oxidation (GO:0046320) | 44608 | 0.646475171 | 0.828689627 |
| regulation of leukocyte activation (GO:0002694) | 44608 | 0.646475171 | 0.828689627 |
| regulation of phosphate metabolic process (GO:0019220 | 44608 | 0.646475171 | 0.828689627 |
| regulation of systemic arterial blood pressure by hormon | 44608 | 0.646475171 | 0.828689627 |
| response to organophosphorus (GO:0046683) | 44608 | 0.646475171 | 0.828689627 |
| serine family amino acid metabolic process (GO:0009069 | 44608 | 0.646475171 | 0.828689627 |
| sympathetic nervous system development (GO:0048485) | 44608 | 0.646475171 | 0.828689627 |
| mitotic cytokinesis (GO:0000281) | 18050 | 0.646840507 | 0.828689627 |
| regulation of cellular localization (GO:0060341) | 18050 | 0.646840507 | 0.828689627 |
| regulation of stress-activated MAPK cascade (GO:003287 | 18050 | 0.646840507 | 0.828689627 |
| response to glucose (GO:0009749) | 18050 | 0.646840507 | 0.828689627 |
| secondary alcohol metabolic process (GO:1902652) | 18050 | 0.646840507 | 0.828689627 |
| carboxylic acid transmembrane transport (GO:1905039) | 15097 | 0.64939307 | 0.830730607 |
| endosome organization (GO:0007032) | 15097 | 0.64939307 | 0.830730607 |
| positive regulation of cation transmembrane transport (G | 15097 | 0.64939307 | 0.830730607 |
| regulation of cell development (GO:0060284) | 15097 | 0.64939307 | 0.830730607 |
| regulation of G0 to G1 transition (GO:0070316) | 15097 | 0.64939307 | 0.830730607 |
| regulation of mitochondrial membrane potential (GO:005 | 15097 | 0.64939307 | 0.830730607 |
| lytic vacuole organization (GO:0080171) | 12145 | 0.654083043 | 0.835906865 |
| neuron projection extension (GO:1990138) | 12145 | 0.654083043 | 0.835906865 |
| positive regulation of histone modification (GO:0031058) | 12145 | 0.654083043 | 0.835906865 |
| RNA stabilization (GO:0043489) | 12145 | 0.654083043 | 0.835906865 |
| negative regulation of translation (GO:0017148) | 33178 | 0.66153251 | 0.838039437 |
| positive regulation of developmental process (GO:00510! | 22/177 | 0.662174195 | 0.838039437 |
| embryonic organ development (GO:0048568) | 24320 | 0.662531268 | 0.838039437 |
| protein localization to nucleus (GO:0034504) | 13/106 | 0.662533774 | 0.838039437 |
| acylglycerol homeostasis (GO:0055090) | 44645 | 0.662694165 | 0.838039437 |
| cellular response to hexose stimulus (GO:0071331) | 44645 | 0.662694165 | 0.838039437 |
| chemical synaptic transmission, postsynaptic (GO:00995€ | 44645 | 0.662694165 | 0.838039437 |
| energy derivation by oxidation of organic compounds (G( | 44645 | 0.662694165 | 0.838039437 |
| peripheral nervous system development (GO:0007422) | 44645 | 0.662694165 | 0.838039437 |
| protein quality control for misfolded or incompletely synt | 44645 | 0.662694165 | 0.838039437 |
| receptor catabolic process (GO:0032801) | 44645 | 0.662694165 | 0.838039437 |
| regulation of cAMP-mediated signaling (GO:0043949) | 44645 | 0.662694165 | 0.838039437 |
| response to hexose (GO:0009746) | 44645 | 0.662694165 | 0.838039437 |
| ventricular cardiac muscle tissue development (GO:0003: | 44645 | 0.662694165 | 0.838039437 |
| phosphatidic acid metabolic process (GO:0046473) | 18415 | 0.666682322 | 0.838039437 |
| ncRNA processing (GO:0034470) | 25/201 | 0.666979978 | 0.838039437 |
| skeletal system morphogenesis (GO:0048705) | 15462 | 0.670900782 | 0.838039437 |
| translesion synthesis (GO:0019985) | 15462 | 0.670900782 | 0.838039437 |
| nucleic acid phosphodiester bond hydrolysis (GO:009030 | 27638 | 0.67771588 | 0.838039437 |
| central nervous system neuron differentiation (GO:0021! | 12510 | 0.677733711 | 0.838039437 |
| female gamete generation (GO:0007292) | 12510 | 0.677733711 | 0.838039437 |

| | | | |
|---|---|---|---|
| organelle disassembly (GO:1903008) | 12510 | 0.677733711 | 0.838039437 |
| organic cyclic compound biosynthetic process (GO:19013 | 12510 | 0.677733711 | 0.838039437 |
| positive regulation of release of sequestered calcium ion | 12510 | 0.677733711 | 0.838039437 |
| regulation of lamellipodium assembly (GO:0010591) | 12510 | 0.677733711 | 0.838039437 |
| tRNA export from nucleus (GO:0006409) | 12510 | 0.677733711 | 0.838039437 |
| tRNA-containing ribonucleoprotein complex export from | 12510 | 0.677733711 | 0.838039437 |
| ventricular cardiac muscle tissue morphogenesis (GO:005 | 12510 | 0.677733711 | 0.838039437 |
| adenosine to inosine editing (GO:0006382) | 44569 | 0.679769188 | 0.838039437 |
| anterograde axonal protein transport (GO:0099641) | 44569 | 0.679769188 | 0.838039437 |
| antigen processing and presentation of exogenous peptic | 44569 | 0.679769188 | 0.838039437 |
| axo-dendritic protein transport (GO:0099640) | 44569 | 0.679769188 | 0.838039437 |
| box C/D snoRNP assembly (GO:0000492) | 44569 | 0.679769188 | 0.838039437 |
| brown fat cell differentiation (GO:0050873) | 44569 | 0.679769188 | 0.838039437 |
| carbohydrate phosphorylation (GO:0046835) | 44569 | 0.679769188 | 0.838039437 |
| cardiac atrium development (GO:0003230) | 44569 | 0.679769188 | 0.838039437 |
| cardiac muscle hypertrophy (GO:0003300) | 44569 | 0.679769188 | 0.838039437 |
| cell migration involved in heart development (GO:006097 | 44569 | 0.679769188 | 0.838039437 |
| cellular response to epinephrine stimulus (GO:0071872) | 44569 | 0.679769188 | 0.838039437 |
| cellular response to interferon-alpha (GO:0035457) | 44569 | 0.679769188 | 0.838039437 |
| dendritic spine maintenance (GO:0097062) | 44569 | 0.679769188 | 0.838039437 |
| diacylglycerol biosynthetic process (GO:0006651) | 44569 | 0.679769188 | 0.838039437 |
| elastic fiber assembly (GO:0048251) | 44569 | 0.679769188 | 0.838039437 |
| endosomal vesicle fusion (GO:0034058) | 44569 | 0.679769188 | 0.838039437 |
| ER overload response (GO:0006983) | 44569 | 0.679769188 | 0.838039437 |
| establishment of Golgi localization (GO:0051683) | 44569 | 0.679769188 | 0.838039437 |
| establishment of protein localization to peroxisome (GO: | 44569 | 0.679769188 | 0.838039437 |
| folic acid transport (GO:0015884) | 44569 | 0.679769188 | 0.838039437 |
| forebrain neuron development (GO:0021884) | 44569 | 0.679769188 | 0.838039437 |
| forebrain neuron differentiation (GO:0021879) | 44569 | 0.679769188 | 0.838039437 |
| fructose metabolic process (GO:0006000) | 44569 | 0.679769188 | 0.838039437 |
| galactosylceramide metabolic process (GO:0006681) | 44569 | 0.679769188 | 0.838039437 |
| histidine catabolic process (GO:0006548) | 44569 | 0.679769188 | 0.838039437 |
| histidine metabolic process (GO:0006547) | 44569 | 0.679769188 | 0.838039437 |
| histone H2A-K119 monoubiquitination (GO:0036353) | 44569 | 0.679769188 | 0.838039437 |
| histone H3-K4 demethylation (GO:0034720) | 44569 | 0.679769188 | 0.838039437 |
| immune response-inhibiting cell surface receptor signalin | 44569 | 0.679769188 | 0.838039437 |
| internal protein amino acid acetylation (GO:0006475) | 44569 | 0.679769188 | 0.838039437 |
| lens fiber cell development (GO:0070307) | 44569 | 0.679769188 | 0.838039437 |
| lens fiber cell differentiation (GO:0070306) | 44569 | 0.679769188 | 0.838039437 |
| macromolecule depalmitoylation (GO:0098734) | 44569 | 0.679769188 | 0.838039437 |
| macromolecule modification (GO:0043412) | 44569 | 0.679769188 | 0.838039437 |
| mammary gland epithelial cell differentiation (GO:00606 | 44569 | 0.679769188 | 0.838039437 |
| mitochondrial respiratory chain complex III assembly (GC | 44569 | 0.679769188 | 0.838039437 |
| mitochondrial RNA processing (GO:0000963) | 44569 | 0.679769188 | 0.838039437 |
| mitophagy (GO:0000423) | 44569 | 0.679769188 | 0.838039437 |
| negative regulation by symbiont of host apoptotic proces | 44569 | 0.679769188 | 0.838039437 |
| negative regulation by symbiont of host programmed cel | 44569 | 0.679769188 | 0.838039437 |

| | | | |
|---|---|---|---|
| negative regulation of actin nucleation (GO:0051126) | 44569 | 0.679769188 | 0.838039437 |
| negative regulation of alpha-beta T cell differentiation (G | 44569 | 0.679769188 | 0.838039437 |
| negative regulation of B cell apoptotic process (GO:0002 | 44569 | 0.679769188 | 0.838039437 |
| negative regulation of calcium ion import (GO:0090281) | 44569 | 0.679769188 | 0.838039437 |
| negative regulation of cAMP-dependent protein kinase a | 44569 | 0.679769188 | 0.838039437 |
| negative regulation of macrophage migration (GO:19055 | 44569 | 0.679769188 | 0.838039437 |
| negative regulation of mitochondrial membrane potentia | 44569 | 0.679769188 | 0.838039437 |
| negative regulation of mRNA polyadenylation (GO:19003 | 44569 | 0.679769188 | 0.838039437 |
| negative regulation of muscle hypertrophy (GO:0014741) | 44569 | 0.679769188 | 0.838039437 |
| negative regulation of neural precursor cell proliferation | 44569 | 0.679769188 | 0.838039437 |
| negative regulation of nitrogen compound metabolic pro | 44569 | 0.679769188 | 0.838039437 |
| negative regulation of potassium ion transmembrane tra | 44569 | 0.679769188 | 0.838039437 |
| neuron fate specification (GO:0048665) | 44569 | 0.679769188 | 0.838039437 |
| peptidyl-lysine hydroxylation (GO:0017185) | 44569 | 0.679769188 | 0.838039437 |
| peptidyl-proline hydroxylation to 4-hydroxy-L-proline (GC | 44569 | 0.679769188 | 0.838039437 |
| positive regulation of CD4-positive, alpha-beta T cell diffe | 44569 | 0.679769188 | 0.838039437 |
| positive regulation of early endosome to late endosome | 44569 | 0.679769188 | 0.838039437 |
| positive regulation of epithelial cell proliferation involved | 44569 | 0.679769188 | 0.838039437 |
| positive regulation of intracellular steroid hormone recep | 44569 | 0.679769188 | 0.838039437 |
| positive regulation of leukocyte differentiation (GO:1902 | 44569 | 0.679769188 | 0.838039437 |
| positive regulation of meiotic nuclear division (GO:00458 | 44569 | 0.679769188 | 0.838039437 |
| positive regulation of melanin biosynthetic process (GO:C | 44569 | 0.679769188 | 0.838039437 |
| positive regulation of neuron projection arborization (GO | 44569 | 0.679769188 | 0.838039437 |
| positive regulation of ryanodine-sensitive calcium-releas | 44569 | 0.679769188 | 0.838039437 |
| positive regulation of secondary metabolite biosynthetic | 44569 | 0.679769188 | 0.838039437 |
| positive regulation of triglyceride catabolic process (GO:C | 44569 | 0.679769188 | 0.838039437 |
| postsynaptic density organization (GO:0097106) | 44569 | 0.679769188 | 0.838039437 |
| progesterone receptor signaling pathway (GO:0050847) | 44569 | 0.679769188 | 0.838039437 |
| protein deglutamylation (GO:0035608) | 44569 | 0.679769188 | 0.838039437 |
| protein deglycosylation (GO:0006517) | 44569 | 0.679769188 | 0.838039437 |
| protein depalmitoylation (GO:0002084) | 44569 | 0.679769188 | 0.838039437 |
| protein localization to presynapse (GO:1905383) | 44569 | 0.679769188 | 0.838039437 |
| regulation of acetyl-CoA biosynthetic process from pyruv | 44569 | 0.679769188 | 0.838039437 |
| regulation of actin filament length (GO:0030832) | 44569 | 0.679769188 | 0.838039437 |
| regulation of acyl-CoA biosynthetic process (GO:0050812 | 44569 | 0.679769188 | 0.838039437 |
| regulation of chaperone-mediated autophagy (GO:19047 | 44569 | 0.679769188 | 0.838039437 |
| regulation of cyclic-nucleotide phosphodiesterase activity | 44569 | 0.679769188 | 0.838039437 |
| regulation of cytoplasmic translational elongation (GO:19 | 44569 | 0.679769188 | 0.838039437 |
| regulation of dopamine receptor signaling pathway (GO:( | 44569 | 0.679769188 | 0.838039437 |
| regulation of epithelial to mesenchymal transition involv | 44569 | 0.679769188 | 0.838039437 |
| regulation of extent of cell growth (GO:0061387) | 44569 | 0.679769188 | 0.838039437 |
| regulation of Golgi to plasma membrane protein transpo | 44569 | 0.679769188 | 0.838039437 |
| regulation of myeloid leukocyte differentiation (GO:0002 | 44569 | 0.679769188 | 0.838039437 |
| regulation of phospholipase A2 activity (GO:0032429) | 44569 | 0.679769188 | 0.838039437 |
| regulation of potassium ion import (GO:1903286) | 44569 | 0.679769188 | 0.838039437 |
| regulation of protein glycosylation (GO:0060049) | 44569 | 0.679769188 | 0.838039437 |
| regulation of protein localization to cell cortex (GO:1904 | 44569 | 0.679769188 | 0.838039437 |

| | | | |
|---|---|---|---|
| regulation of protein localization to chromatin (GO:1905( | 44569 | 0.679769188 | 0.838039437 |
| regulation of protein localization to nucleolus (GO:19047 | 44569 | 0.679769188 | 0.838039437 |
| regulation of skeletal muscle fiber development (GO:004 | 44569 | 0.679769188 | 0.838039437 |
| regulation of stem cell division (GO:2000035) | 44569 | 0.679769188 | 0.838039437 |
| regulation of striated muscle tissue development (GO:00 | 44569 | 0.679769188 | 0.838039437 |
| respiratory chain complex III assembly (GO:0017062) | 44569 | 0.679769188 | 0.838039437 |
| response to epinephrine (GO:0071871) | 44569 | 0.679769188 | 0.838039437 |
| ribosome disassembly (GO:0032790) | 44569 | 0.679769188 | 0.838039437 |
| self proteolysis (GO:0097264) | 44569 | 0.679769188 | 0.838039437 |
| skeletal myofibril assembly (GO:0014866) | 44569 | 0.679769188 | 0.838039437 |
| spermidine metabolic process (GO:0008216) | 44569 | 0.679769188 | 0.838039437 |
| sulfur amino acid biosynthetic process (GO:0000097) | 44569 | 0.679769188 | 0.838039437 |
| T cell mediated cytotoxicity (GO:0001913) | 44569 | 0.679769188 | 0.838039437 |
| thyroid gland development (GO:0030878) | 44569 | 0.679769188 | 0.838039437 |
| TORC1 signaling (GO:0038202) | 44569 | 0.679769188 | 0.838039437 |
| venous blood vessel development (GO:0060841) | 44569 | 0.679769188 | 0.838039437 |
| vitamin K metabolic process (GO:0042373) | 44569 | 0.679769188 | 0.838039437 |
| water homeostasis (GO:0030104) | 44569 | 0.679769188 | 0.838039437 |
| amyloid-beta metabolic process (GO:0050435) | 44609 | 0.679787909 | 0.838039437 |
| aorta morphogenesis (GO:0035909) | 44609 | 0.679787909 | 0.838039437 |
| DNA biosynthetic process (GO:0071897) | 44609 | 0.679787909 | 0.838039437 |
| mesenchyme morphogenesis (GO:0072132) | 44609 | 0.679787909 | 0.838039437 |
| negative regulation of amyloid-beta formation (GO:1902 | 44609 | 0.679787909 | 0.838039437 |
| pharyngeal system development (GO:0060037) | 44609 | 0.679787909 | 0.838039437 |
| phosphate ion transport (GO:0006817) | 44609 | 0.679787909 | 0.838039437 |
| positive regulation of collagen metabolic process (GO:00 | 44609 | 0.679787909 | 0.838039437 |
| positive regulation of regulated secretory pathway (GO:1 | 44609 | 0.679787909 | 0.838039437 |
| positive regulation of stem cell proliferation (GO:200064 | 44609 | 0.679787909 | 0.838039437 |
| positive regulation of telomere capping (GO:1904355) | 44609 | 0.679787909 | 0.838039437 |
| regulation of cardiac muscle contraction by regulation of | 44609 | 0.679787909 | 0.838039437 |
| regulation of cholesterol biosynthetic process (GO:00455 | 44609 | 0.679787909 | 0.838039437 |
| regulation of metal ion transport (GO:0010959) | 44609 | 0.679787909 | 0.838039437 |
| regulation of stem cell proliferation (GO:0072091) | 44609 | 0.679787909 | 0.838039437 |
| RNA interference (GO:0016246) | 44609 | 0.679787909 | 0.838039437 |
| neuron projection development (GO:0031175) | 21/171 | 0.681960435 | 0.840333693 |
| selective autophagy (GO:0061912) | 21732 | 0.681972445 | 0.840333693 |
| intracellular protein transport (GO:0006886) | 42/336 | 0.685576646 | 0.844574502 |
| ion transport (GO:0006811) | 14/116 | 0.68899611 | 0.847074891 |
| metanephros development (GO:0001656) | 44646 | 0.689236875 | 0.847074891 |
| negative regulation of signaling receptor activity (GO:200 | 44646 | 0.689236875 | 0.847074891 |
| negative regulation of telomere maintenance via telomei | 44646 | 0.689236875 | 0.847074891 |
| negative regulation of transposition (GO:0010529) | 44646 | 0.689236875 | 0.847074891 |
| positive regulation of microtubule polymerization or dep | 44646 | 0.689236875 | 0.847074891 |
| regulation of glycogen biosynthetic process (GO:000597 | 44646 | 0.689236875 | 0.847074891 |
| regulation of potassium ion transmembrane transporter | 44646 | 0.689236875 | 0.847074891 |
| regulation of synapse organization (GO:0050807) | 44646 | 0.689236875 | 0.847074891 |
| regulation of telomere maintenance (GO:0032204) | 44646 | 0.689236875 | 0.847074891 |

| | | | |
|---|---|---|---|
| regulation of signal transduction by p53 class mediator (( | 19/156 | 0.690877801 | 0.84889077 |
| tRNA aminoacylation for protein translation (GO:000641 | 15827 | 0.691518469 | 0.84947705 |
| cell morphogenesis involved in neuron differentiation (G( | 28004 | 0.693245852 | 0.851196455 |
| phospholipid metabolic process (GO:0006644) | 28004 | 0.693245852 | 0.851196455 |
| RNA splicing, via transesterification reactions with bulgec | 31/251 | 0.693878306 | 0.851771692 |
| regulation of mitotic cell cycle phase transition (GO:1901 | 23/188 | 0.694105678 | 0.851849514 |
| adenylate cyclase-inhibiting G protein-coupled receptor s | 22098 | 0.699251401 | 0.857556899 |
| ribonucleoprotein complex biogenesis (GO:0022613) | 22098 | 0.699251401 | 0.857556899 |
| RNA methylation (GO:0001510) | 22098 | 0.699251401 | 0.857556899 |
| blood vessel diameter maintenance (GO:0097746) | 12875 | 0.700231621 | 0.857948885 |
| negative regulation of epithelial cell migration (GO:00106 | 12875 | 0.700231621 | 0.857948885 |
| regulation of ion transmembrane transport (GO:0034765 | 12875 | 0.700231621 | 0.857948885 |
| regulation of regulated secretory pathway (GO:1903305) | 12875 | 0.700231621 | 0.857948885 |
| amide biosynthetic process (GO:0043604) | 19146 | 0.704190363 | 0.862392488 |
| cartilage development (GO:0051216) | 19146 | 0.704190363 | 0.862392488 |
| negative regulation of developmental process (GO:00510 | 28369 | 0.708299526 | 0.862963381 |
| chromatin disassembly (GO:0031498) | 44610 | 0.710487119 | 0.862963381 |
| DNA conformation change (GO:0071103) | 44610 | 0.710487119 | 0.862963381 |
| embryonic digit morphogenesis (GO:0042733) | 44610 | 0.710487119 | 0.862963381 |
| epithelial structure maintenance (GO:0010669) | 44610 | 0.710487119 | 0.862963381 |
| G protein-coupled acetylcholine receptor signaling pathw | 44610 | 0.710487119 | 0.862963381 |
| glycoprotein catabolic process (GO:0006516) | 44610 | 0.710487119 | 0.862963381 |
| high-density lipoprotein particle remodeling (GO:003437 | 44610 | 0.710487119 | 0.862963381 |
| intermediate filament organization (GO:0045109) | 44610 | 0.710487119 | 0.862963381 |
| melanosome transport (GO:0032402) | 44610 | 0.710487119 | 0.862963381 |
| negative regulation of biosynthetic process (GO:0009890 | 44610 | 0.710487119 | 0.862963381 |
| negative regulation of cation transmembrane transport (( | 44610 | 0.710487119 | 0.862963381 |
| negative regulation of double-strand break repair via hon | 44610 | 0.710487119 | 0.862963381 |
| nucleosome disassembly (GO:0006337) | 44610 | 0.710487119 | 0.862963381 |
| nucleotide biosynthetic process (GO:0009165) | 44610 | 0.710487119 | 0.862963381 |
| outer dynein arm assembly (GO:0036158) | 44610 | 0.710487119 | 0.862963381 |
| pigment granule transport (GO:0051904) | 44610 | 0.710487119 | 0.862963381 |
| positive regulation of cartilage development (GO:006103 | 44610 | 0.710487119 | 0.862963381 |
| positive regulation of peptidyl-serine phosphorylation of | 44610 | 0.710487119 | 0.862963381 |
| prostanoid metabolic process (GO:0006692) | 44610 | 0.710487119 | 0.862963381 |
| regulation of chromosome segregation (GO:0051983) | 44610 | 0.710487119 | 0.862963381 |
| regulation of membrane depolarization (GO:0003254) | 44610 | 0.710487119 | 0.862963381 |
| response to axon injury (GO:0048678) | 44610 | 0.710487119 | 0.862963381 |
| water transport (GO:0006833) | 44610 | 0.710487119 | 0.862963381 |
| branching morphogenesis of an epithelial tube (GO:0048 | 16193 | 0.711234544 | 0.862963381 |
| hexose biosynthetic process (GO:0019319) | 16193 | 0.711234544 | 0.862963381 |
| metal ion homeostasis (GO:0055065) | 16193 | 0.711234544 | 0.862963381 |
| monocarboxylic acid catabolic process (GO:0072329) | 44647 | 0.71417653 | 0.862963381 |
| negative regulation of actin filament bundle assembly (G( | 44647 | 0.71417653 | 0.862963381 |
| presynaptic endocytosis (GO:0140238) | 44647 | 0.71417653 | 0.862963381 |
| regulation of transcription elongation from RNA polymer | 44647 | 0.71417653 | 0.862963381 |
| nucleosome organization (GO:0034728) | 34639 | 0.717552974 | 0.862963381 |

| | | | |
|---|---|---|---|
| calcium-dependent cell-cell adhesion via plasma membra | 13241 | 0.721573785 | 0.862963381 |
| cellular response to heat (GO:0034605) | 13241 | 0.721573785 | 0.862963381 |
| embryonic limb morphogenesis (GO:0030326) | 13241 | 0.721573785 | 0.862963381 |
| positive regulation of bone mineralization (GO:0030501) | 13241 | 0.721573785 | 0.862963381 |
| regulation of embryonic development (GO:0045995) | 13241 | 0.721573785 | 0.862963381 |
| regulation of microtubule-based process (GO:0032886) | 13241 | 0.721573785 | 0.862963381 |
| retina homeostasis (GO:0001895) | 13241 | 0.721573785 | 0.862963381 |
| acid secretion (GO:0046717) | 44570 | 0.722264636 | 0.862963381 |
| associative learning (GO:0008306) | 44570 | 0.722264636 | 0.862963381 |
| cholesterol catabolic process (GO:0006707) | 44570 | 0.722264636 | 0.862963381 |
| chylomicron remodeling (GO:0034371) | 44570 | 0.722264636 | 0.862963381 |
| cilium movement involved in cell motility (GO:0060294) | 44570 | 0.722264636 | 0.862963381 |
| collagen-activated signaling pathway (GO:0038065) | 44570 | 0.722264636 | 0.862963381 |
| DNA cytosine deamination (GO:0070383) | 44570 | 0.722264636 | 0.862963381 |
| DNA topological change (GO:0006265) | 44570 | 0.722264636 | 0.862963381 |
| embryonic forelimb morphogenesis (GO:0035115) | 44570 | 0.722264636 | 0.862963381 |
| endochondral ossification (GO:0001958) | 44570 | 0.722264636 | 0.862963381 |
| estrogen biosynthetic process (GO:0006703) | 44570 | 0.722264636 | 0.862963381 |
| fucose catabolic process (GO:0019317) | 44570 | 0.722264636 | 0.862963381 |
| hepoxilin biosynthetic process (GO:0051122) | 44570 | 0.722264636 | 0.862963381 |
| hepoxilin metabolic process (GO:0051121) | 44570 | 0.722264636 | 0.862963381 |
| histone phosphorylation (GO:0016572) | 44570 | 0.722264636 | 0.862963381 |
| hormone transport (GO:0009914) | 44570 | 0.722264636 | 0.862963381 |
| icosanoid transport (GO:0071715) | 44570 | 0.722264636 | 0.862963381 |
| inner ear receptor cell development (GO:0060119) | 44570 | 0.722264636 | 0.862963381 |
| intestinal cholesterol absorption (GO:0030299) | 44570 | 0.722264636 | 0.862963381 |
| ketone biosynthetic process (GO:0042181) | 44570 | 0.722264636 | 0.862963381 |
| kinetochore assembly (GO:0051382) | 44570 | 0.722264636 | 0.862963381 |
| L-fucose catabolic process (GO:0042355) | 44570 | 0.722264636 | 0.862963381 |
| L-fucose metabolic process (GO:0042354) | 44570 | 0.722264636 | 0.862963381 |
| L-phenylalanine metabolic process (GO:0006558) | 44570 | 0.722264636 | 0.862963381 |
| lysosomal lumen acidification (GO:0007042) | 44570 | 0.722264636 | 0.862963381 |
| maintenance of apical/basal cell polarity (GO:0035090) | 44570 | 0.722264636 | 0.862963381 |
| maintenance of epithelial cell apical/basal polarity (GO:0( | 44570 | 0.722264636 | 0.862963381 |
| mesodermal cell differentiation (GO:0048333) | 44570 | 0.722264636 | 0.862963381 |
| mitochondrial tRNA modification (GO:0070900) | 44570 | 0.722264636 | 0.862963381 |
| mitral valve morphogenesis (GO:0003183) | 44570 | 0.722264636 | 0.862963381 |
| negative regulation of biomineralization (GO:0110150) | 44570 | 0.722264636 | 0.862963381 |
| negative regulation of fibrinolysis (GO:0051918) | 44570 | 0.722264636 | 0.862963381 |
| negative regulation of membrane potential (GO:0045837 | 44570 | 0.722264636 | 0.862963381 |
| negative regulation of mRNA 3'-end processing (GO:0031 | 44570 | 0.722264636 | 0.862963381 |
| negative regulation of smooth muscle cell differentiation | 44570 | 0.722264636 | 0.862963381 |
| nucleoside diphosphate phosphorylation (GO:0006165) | 44570 | 0.722264636 | 0.862963381 |
| nucleoside triphosphate biosynthetic process (GO:00091 | 44570 | 0.722264636 | 0.862963381 |
| nucleotide phosphorylation (GO:0046939) | 44570 | 0.722264636 | 0.862963381 |
| oligosaccharide catabolic process (GO:0009313) | 44570 | 0.722264636 | 0.862963381 |
| peroxisomal transport (GO:0043574) | 44570 | 0.722264636 | 0.862963381 |

| | | | |
|---|---|---|---|
| peroxisome fission (GO:0016559) | 44570 | 0.722264636 | 0.862963381 |
| phosphorylation of RNA polymerase II C-terminal domain | 44570 | 0.722264636 | 0.862963381 |
| positive regulation of autophagosome assembly (GO:200 | 44570 | 0.722264636 | 0.862963381 |
| positive regulation of autophagy of mitochondrion (GO:1 | 44570 | 0.722264636 | 0.862963381 |
| positive regulation of blood pressure (GO:0045777) | 44570 | 0.722264636 | 0.862963381 |
| positive regulation of CD8-positive, alpha-beta T cell activ | 44570 | 0.722264636 | 0.862963381 |
| positive regulation of cholesterol esterification (GO:0010 | 44570 | 0.722264636 | 0.862963381 |
| positive regulation of megakaryocyte differentiation (GO | 44570 | 0.722264636 | 0.862963381 |
| positive regulation of membrane potential (GO:0045838) | 44570 | 0.722264636 | 0.862963381 |
| positive regulation of smooth muscle contraction (GO:00 | 44570 | 0.722264636 | 0.862963381 |
| protein hexamerization (GO:0034214) | 44570 | 0.722264636 | 0.862963381 |
| protein K6-linked ubiquitination (GO:0085020) | 44570 | 0.722264636 | 0.862963381 |
| regulation of cellular response to oxidative stress (GO:19 | 44570 | 0.722264636 | 0.862963381 |
| regulation of ER to Golgi vesicle-mediated transport (GO: | 44570 | 0.722264636 | 0.862963381 |
| regulation of gap junction assembly (GO:1903596) | 44570 | 0.722264636 | 0.862963381 |
| regulation of helicase activity (GO:0051095) | 44570 | 0.722264636 | 0.862963381 |
| regulation of histone H4 acetylation (GO:0090239) | 44570 | 0.722264636 | 0.862963381 |
| regulation of leukocyte degranulation (GO:0043300) | 44570 | 0.722264636 | 0.862963381 |
| regulation of nephron tubule epithelial cell differentiatio | 44570 | 0.722264636 | 0.862963381 |
| regulation of nuclear cell cycle DNA replication (GO:0033 | 44570 | 0.722264636 | 0.862963381 |
| regulation of peroxisome proliferator activated receptor | 44570 | 0.722264636 | 0.862963381 |
| regulation of Wnt signaling pathway, planar cell polarity | 44570 | 0.722264636 | 0.862963381 |
| skeletal muscle fiber development (GO:0048741) | 44570 | 0.722264636 | 0.862963381 |
| sterol catabolic process (GO:0016127) | 44570 | 0.722264636 | 0.862963381 |
| sympathetic ganglion development (GO:0061549) | 44570 | 0.722264636 | 0.862963381 |
| type B pancreatic cell differentiation (GO:0003309) | 44570 | 0.722264636 | 0.862963381 |
| unsaturated fatty acid biosynthetic process (GO:0006636 | 44570 | 0.722264636 | 0.862963381 |
| monocarboxylic acid metabolic process (GO:0032787) | 17/143 | 0.722950392 | 0.863385583 |
| neuron development (GO:0048666) | 17/143 | 0.722950392 | 0.863385583 |
| action potential (GO:0001508) | 16558 | 0.730044444 | 0.871256808 |
| amino acid transmembrane transport (GO:0003333) | 16558 | 0.730044444 | 0.871256808 |
| mitotic G2/M transition checkpoint (GO:0044818) | 16558 | 0.730044444 | 0.871256808 |
| mRNA metabolic process (GO:0016071) | 35004 | 0.730585077 | 0.871701763 |
| glucose metabolic process (GO:0006006) | 22828 | 0.73191952 | 0.873093435 |
| cellular protein-containing complex assembly (GO:00346 | 20/168 | 0.732181704 | 0.873205682 |
| plasma membrane bounded cell projection organization ( | 15/128 | 0.735411221 | 0.874929601 |
| mucosal immune response (GO:0002385) | 44648 | 0.737537665 | 0.874929601 |
| positive regulation of microtubule polymerization (GO:0( | 44648 | 0.737537665 | 0.874929601 |
| protein localization to cell surface (GO:0034394) | 44648 | 0.737537665 | 0.874929601 |
| regulation of microtubule polymerization or depolymeriz | 44648 | 0.737537665 | 0.874929601 |
| regulation of postsynapse organization (GO:0099175) | 44648 | 0.737537665 | 0.874929601 |
| ventricular septum morphogenesis (GO:0060412) | 44648 | 0.737537665 | 0.874929601 |
| aspartate family amino acid catabolic process (GO:00090 | 44611 | 0.738679736 | 0.874929601 |
| aspartate family amino acid metabolic process (GO:0009( | 44611 | 0.738679736 | 0.874929601 |
| ATP synthesis coupled proton transport (GO:0015986) | 44611 | 0.738679736 | 0.874929601 |
| carbohydrate derivative catabolic process (GO:1901136) | 44611 | 0.738679736 | 0.874929601 |
| cellular response to copper ion (GO:0071280) | 44611 | 0.738679736 | 0.874929601 |

| | | | |
|---|---|---|---|
| detection of mechanical stimulus (GO:0050982) | 44611 | 0.738679736 | 0.874929601 |
| endocardial cushion morphogenesis (GO:0003203) | 44611 | 0.738679736 | 0.874929601 |
| endoplasmic reticulum mannose trimming (GO:1904380) | 44611 | 0.738679736 | 0.874929601 |
| establishment of melanosome localization (GO:0032401) | 44611 | 0.738679736 | 0.874929601 |
| excitatory postsynaptic potential (GO:0060079) | 44611 | 0.738679736 | 0.874929601 |
| glycerolipid catabolic process (GO:0046503) | 44611 | 0.738679736 | 0.874929601 |
| mesenchymal cell development (GO:0014031) | 44611 | 0.738679736 | 0.874929601 |
| N-glycan processing (GO:0006491) | 44611 | 0.738679736 | 0.874929601 |
| negative regulation of immune system process (GO:0002 | 44611 | 0.738679736 | 0.874929601 |
| neural crest cell differentiation (GO:0014033) | 44611 | 0.738679736 | 0.874929601 |
| pore complex assembly (GO:0046931) | 44611 | 0.738679736 | 0.874929601 |
| regulation of autophagy of mitochondrion (GO:1903146) | 44611 | 0.738679736 | 0.874929601 |
| regulation of centriole replication (GO:0046599) | 44611 | 0.738679736 | 0.874929601 |
| regulation of histone H3-K4 methylation (GO:0051569) | 44611 | 0.738679736 | 0.874929601 |
| regulation of peptidyl-serine phosphorylation of STAT prc | 44611 | 0.738679736 | 0.874929601 |
| regulation of stem cell population maintenance (GO:200( | 44611 | 0.738679736 | 0.874929601 |
| response to fatty acid (GO:0070542) | 44611 | 0.738679736 | 0.874929601 |
| vesicle transport along actin filament (GO:0030050) | 44611 | 0.738679736 | 0.874929601 |
| ncRNA metabolic process (GO:0034660) | 13606 | 0.741766835 | 0.877785582 |
| phospholipid biosynthetic process (GO:0008654) | 13606 | 0.741766835 | 0.877785582 |
| regulation of intracellular steroid hormone receptor signa | 13606 | 0.741766835 | 0.877785582 |
| tRNA transport (GO:0051031) | 13606 | 0.741766835 | 0.877785582 |
| COPII vesicle coating (GO:0048208) | 23193 | 0.747299175 | 0.883310154 |
| negative regulation of peptidase activity (GO:0010466) | 23193 | 0.747299175 | 0.883310154 |
| vesicle coating (GO:0006901) | 23193 | 0.747299175 | 0.883310154 |
| vesicle targeting, rough ER to cis-Golgi (GO:0048207) | 23193 | 0.747299175 | 0.883310154 |
| postreplication repair (GO:0006301) | 16923 | 0.747949891 | 0.883310154 |
| posttranscriptional regulation of gene expression (GO:00 | 16923 | 0.747949891 | 0.883310154 |
| regulation of double-strand break repair via homologous | 16923 | 0.747949891 | 0.883310154 |
| regulation of telomerase activity (GO:0051972) | 16923 | 0.747949891 | 0.883310154 |
| glycerophospholipid metabolic process (GO:0006650) | 29465 | 0.750536445 | 0.883310154 |
| regulation of organelle organization (GO:0033043) | 29465 | 0.750536445 | 0.883310154 |
| mRNA splicing, via spliceosome (GO:0000398) | 33/274 | 0.751240615 | 0.883310154 |
| ceramide metabolic process (GO:0006672) | 20241 | 0.754878897 | 0.883310154 |
| G2/M transition of mitotic cell cycle (GO:0000086) | 15/130 | 0.756840064 | 0.883310154 |
| Golgi organization (GO:0007030) | 15/130 | 0.756840064 | 0.883310154 |
| post-Golgi vesicle-mediated transport (GO:0006892) | 32782 | 0.759121977 | 0.883310154 |
| alanine transport (GO:0032328) | 44571 | 0.759122642 | 0.883310154 |
| apical protein localization (GO:0045176) | 44571 | 0.759122642 | 0.883310154 |
| cell differentiation involved in metanephros developmen | 44571 | 0.759122642 | 0.883310154 |
| cellular response to hydroxyurea (GO:0072711) | 44571 | 0.759122642 | 0.883310154 |
| cellular response to leucine starvation (GO:1990253) | 44571 | 0.759122642 | 0.883310154 |
| chylomicron assembly (GO:0034378) | 44571 | 0.759122642 | 0.883310154 |
| DNA damage response, signal transduction by p53 class r | 44571 | 0.759122642 | 0.883310154 |
| DNA dealkylation (GO:0035510) | 44571 | 0.759122642 | 0.883310154 |
| DNA dealkylation involved in DNA repair (GO:0006307) | 44571 | 0.759122642 | 0.883310154 |
| dosage compensation (GO:0007549) | 44571 | 0.759122642 | 0.883310154 |

| | | | |
|---|---|---|---|
| endodermal cell fate commitment (GO:0001711) | 44571 | 0.759122642 | 0.883310154 |
| forelimb morphogenesis (GO:0035136) | 44571 | 0.759122642 | 0.883310154 |
| glycine transport (GO:0015816) | 44571 | 0.759122642 | 0.883310154 |
| glycosphingolipid catabolic process (GO:0046479) | 44571 | 0.759122642 | 0.883310154 |
| hematopoietic stem cell differentiation (GO:0060218) | 44571 | 0.759122642 | 0.883310154 |
| histone H4 deacetylation (GO:0070933) | 44571 | 0.759122642 | 0.883310154 |
| long-chain fatty acid import into cell (GO:0044539) | 44571 | 0.759122642 | 0.883310154 |
| low-density lipoprotein particle receptor catabolic proces | 44571 | 0.759122642 | 0.883310154 |
| low-density lipoprotein receptor particle metabolic proce | 44571 | 0.759122642 | 0.883310154 |
| lung epithelium development (GO:0060428) | 44571 | 0.759122642 | 0.883310154 |
| muscle cell migration (GO:0014812) | 44571 | 0.759122642 | 0.883310154 |
| myelin maintenance (GO:0043217) | 44571 | 0.759122642 | 0.883310154 |
| negative regulation of establishment of protein localizati | 44571 | 0.759122642 | 0.883310154 |
| negative regulation of lipopolysaccharide-mediated signa | 44571 | 0.759122642 | 0.883310154 |
| negative regulation of peptidyl-lysine acetylation (GO:20( | 44571 | 0.759122642 | 0.883310154 |
| negative regulation of phosphatidylinositol 3-kinase signa | 44571 | 0.759122642 | 0.883310154 |
| negative regulation of transcription from RNA polymeras | 44571 | 0.759122642 | 0.883310154 |
| neuron projection fasciculation (GO:0106030) | 44571 | 0.759122642 | 0.883310154 |
| nuclear pore complex assembly (GO:0051292) | 44571 | 0.759122642 | 0.883310154 |
| nucleic acid transport (GO:0050657) | 44571 | 0.759122642 | 0.883310154 |
| nucleoside metabolic process (GO:0009116) | 44571 | 0.759122642 | 0.883310154 |
| omega-hydroxylase P450 pathway (GO:0097267) | 44571 | 0.759122642 | 0.883310154 |
| positive regulation of lipase activity (GO:0060193) | 44571 | 0.759122642 | 0.883310154 |
| positive regulation of mRNA 3'-end processing (GO:0031 | 44571 | 0.759122642 | 0.883310154 |
| positive regulation of transcription of nucleolar large rRN | 44571 | 0.759122642 | 0.883310154 |
| positive regulation of vasculogenesis (GO:2001214) | 44571 | 0.759122642 | 0.883310154 |
| production of siRNA involved in RNA interference (GO:00 | 44571 | 0.759122642 | 0.883310154 |
| receptor recycling (GO:0001881) | 44571 | 0.759122642 | 0.883310154 |
| regulation of bile acid biosynthetic process (GO:0070857 | 44571 | 0.759122642 | 0.883310154 |
| regulation of cell-cell adhesion mediated by integrin (GO: | 44571 | 0.759122642 | 0.883310154 |
| regulation of cilium movement (GO:0003352) | 44571 | 0.759122642 | 0.883310154 |
| regulation of DNA damage checkpoint (GO:2000001) | 44571 | 0.759122642 | 0.883310154 |
| regulation of low-density lipoprotein particle clearance ( | 44571 | 0.759122642 | 0.883310154 |
| regulation of microtubule nucleation (GO:0010968) | 44571 | 0.759122642 | 0.883310154 |
| regulation of RNA polymerase II transcription preinitiatio | 44571 | 0.759122642 | 0.883310154 |
| RNA polymerase II preinitiation complex assembly (GO:0( | 44571 | 0.759122642 | 0.883310154 |
| serine family amino acid catabolic process (GO:0009071) | 44571 | 0.759122642 | 0.883310154 |
| small nucleolar ribonucleoprotein complex assembly (GO | 44571 | 0.759122642 | 0.883310154 |
| SNARE complex assembly (GO:0035493) | 44571 | 0.759122642 | 0.883310154 |
| telomere maintenance in response to DNA damage (GO:( | 44571 | 0.759122642 | 0.883310154 |
| Wnt signaling pathway involved in midbrain dopaminergi | 44571 | 0.759122642 | 0.883310154 |
| establishment of mitotic spindle localization (GO:004000 | 44649 | 0.75935768 | 0.883310154 |
| homologous chromosome pairing at meiosis (GO:000712 | 44649 | 0.75935768 | 0.883310154 |
| hydrogen peroxide metabolic process (GO:0042743) | 44649 | 0.75935768 | 0.883310154 |
| limb development (GO:0060173) | 44649 | 0.75935768 | 0.883310154 |
| lipid catabolic process (GO:0016042) | 44649 | 0.75935768 | 0.883310154 |
| negative regulation of wound healing (GO:0061045) | 44649 | 0.75935768 | 0.883310154 |

| | | | |
|---|---|---|---|
| protein K11-linked ubiquitination (GO:0070979) | 44649 | 0.75935768 | 0.883310154 |
| protein targeting to peroxisome (GO:0006625) | 44649 | 0.75935768 | 0.883310154 |
| regulation of actin polymerization or depolymerization (G | 44649 | 0.75935768 | 0.883310154 |
| regulation of neuronal synaptic plasticity (GO:0048168) | 44649 | 0.75935768 | 0.883310154 |
| ncRNA export from nucleus (GO:0097064) | 13971 | 0.760825666 | 0.884621605 |
| negative regulation of G0 to G1 transition (GO:0070317) | 13971 | 0.760825666 | 0.884621605 |
| epithelial cilium movement involved in extracellular fluid | 44612 | 0.764490315 | 0.885908361 |
| folic acid-containing compound metabolic process (GO:0( | 44612 | 0.764490315 | 0.885908361 |
| gastrulation (GO:0007369) | 44612 | 0.764490315 | 0.885908361 |
| insulin secretion (GO:0030073) | 44612 | 0.764490315 | 0.885908361 |
| mesoderm formation (GO:0001707) | 44612 | 0.764490315 | 0.885908361 |
| microtubule nucleation (GO:0007020) | 44612 | 0.764490315 | 0.885908361 |
| mitochondrial cytochrome c oxidase assembly (GO:0033( | 44612 | 0.764490315 | 0.885908361 |
| positive regulation of embryonic development (GO:0040( | 44612 | 0.764490315 | 0.885908361 |
| positive regulation of G2/M transition of mitotic cell cycl( | 44612 | 0.764490315 | 0.885908361 |
| positive regulation of heart contraction (GO:0045823) | 44612 | 0.764490315 | 0.885908361 |
| positive regulation of peptidyl-lysine acetylation (GO:200 | 44612 | 0.764490315 | 0.885908361 |
| production of small RNA involved in gene silencing by RN | 44612 | 0.764490315 | 0.885908361 |
| protein-DNA complex disassembly (GO:0032986) | 44612 | 0.764490315 | 0.885908361 |
| regulation of fibroblast growth factor receptor signaling p | 44612 | 0.764490315 | 0.885908361 |
| regulation of protein kinase A signaling (GO:0010738) | 44612 | 0.764490315 | 0.885908361 |
| mRNA processing (GO:0006397) | 36/300 | 0.766452823 | 0.887984482 |
| response to calcium ion (GO:0051592) | 12/107 | 0.775017366 | 0.897706848 |
| steroid biosynthetic process (GO:0006694) | 23924 | 0.77615024 | 0.898618341 |
| tRNA modification (GO:0006400) | 23924 | 0.77615024 | 0.898618341 |
| regulation of dendrite development (GO:0050773) | 14336 | 0.778772176 | 0.900069388 |
| establishment of spindle orientation (GO:0051294) | 44650 | 0.779684168 | 0.900069388 |
| ion homeostasis (GO:0050801) | 44650 | 0.779684168 | 0.900069388 |
| long-chain fatty acid biosynthetic process (GO:0042759) | 44650 | 0.779684168 | 0.900069388 |
| primary neural tube formation (GO:0014020) | 44650 | 0.779684168 | 0.900069388 |
| regulation of long-term synaptic potentiation (GO:19002' | 44650 | 0.779684168 | 0.900069388 |
| mitotic nuclear division (GO:0140014) | 27242 | 0.782225705 | 0.900069388 |
| regulation of G2/M transition of mitotic cell cycle (GO:00 | 17/149 | 0.782472147 | 0.900069388 |
| acetylcholine receptor signaling pathway (GO:0095500) | 44613 | 0.78805448 | 0.900069388 |
| anterior/posterior axis specification (GO:0009948) | 44613 | 0.78805448 | 0.900069388 |
| autonomic nervous system development (GO:0048483) | 44613 | 0.78805448 | 0.900069388 |
| cellular response to estradiol stimulus (GO:0071392) | 44613 | 0.78805448 | 0.900069388 |
| cobalamin metabolic process (GO:0009235) | 44613 | 0.78805448 | 0.900069388 |
| endocardial cushion development (GO:0003197) | 44613 | 0.78805448 | 0.900069388 |
| heparan sulfate proteoglycan metabolic process (GO:003 | 44613 | 0.78805448 | 0.900069388 |
| hydrogen peroxide catabolic process (GO:0042744) | 44613 | 0.78805448 | 0.900069388 |
| melanosome localization (GO:0032400) | 44613 | 0.78805448 | 0.900069388 |
| natural killer cell activation involved in immune response | 44613 | 0.78805448 | 0.900069388 |
| nucleotide-excision repair, DNA incision, 3'-to lesion (GO: | 44613 | 0.78805448 | 0.900069388 |
| nucleotide-excision repair, preincision complex stabilizati | 44613 | 0.78805448 | 0.900069388 |
| positive regulation of smoothened signaling pathway (GC | 44613 | 0.78805448 | 0.900069388 |
| protein localization to chromatin (GO:0071168) | 44613 | 0.78805448 | 0.900069388 |

| | | | |
|---|---|---|---|
| regulation of cardiac muscle contraction by calcium ion si | 44613 | 0.78805448 | 0.900069388 |
| regulation of lipoprotein lipase activity (GO:0051004) | 44613 | 0.78805448 | 0.900069388 |
| peptide metabolic process (GO:0006518) | 30560 | 0.78833741 | 0.900069388 |
| positive regulation of translation (GO:0045727) | 11/100 | 0.789708786 | 0.900069388 |
| actin filament depolymerization (GO:0030042) | 44572 | 0.791090823 | 0.900069388 |
| actin filament network formation (GO:0051639) | 44572 | 0.791090823 | 0.900069388 |
| alcohol catabolic process (GO:0046164) | 44572 | 0.791090823 | 0.900069388 |
| axonal transport of mitochondrion (GO:0019896) | 44572 | 0.791090823 | 0.900069388 |
| calcineurin-NFAT signaling cascade (GO:0033173) | 44572 | 0.791090823 | 0.900069388 |
| cellular response to UV-A (GO:0071492) | 44572 | 0.791090823 | 0.900069388 |
| central nervous system myelination (GO:0022010) | 44572 | 0.791090823 | 0.900069388 |
| coenzyme A biosynthetic process (GO:0015937) | 44572 | 0.791090823 | 0.900069388 |
| detection of light stimulus involved in sensory perception | 44572 | 0.791090823 | 0.900069388 |
| detection of light stimulus involved in visual perception ( | 44572 | 0.791090823 | 0.900069388 |
| ER-associated misfolded protein catabolic process (GO:0( | 44572 | 0.791090823 | 0.900069388 |
| erythrocyte development (GO:0048821) | 44572 | 0.791090823 | 0.900069388 |
| establishment of endothelial intestinal barrier (GO:00905 | 44572 | 0.791090823 | 0.900069388 |
| glucocorticoid biosynthetic process (GO:0006704) | 44572 | 0.791090823 | 0.900069388 |
| glucocorticoid metabolic process (GO:0008211) | 44572 | 0.791090823 | 0.900069388 |
| inositol phosphate biosynthetic process (GO:0032958) | 44572 | 0.791090823 | 0.900069388 |
| lipoprotein catabolic process (GO:0042159) | 44572 | 0.791090823 | 0.900069388 |
| metanephric mesenchyme development (GO:0072075) | 44572 | 0.791090823 | 0.900069388 |
| mitochondrial transcription (GO:0006390) | 44572 | 0.791090823 | 0.900069388 |
| mitochondrion morphogenesis (GO:0070584) | 44572 | 0.791090823 | 0.900069388 |
| myotube cell development (GO:0014904) | 44572 | 0.791090823 | 0.900069388 |
| negative regulation of calcium ion transmembrane transp | 44572 | 0.791090823 | 0.900069388 |
| negative regulation of centrosome duplication (GO:0010 | 44572 | 0.791090823 | 0.900069388 |
| negative regulation of intracellular estrogen receptor sig | 44572 | 0.791090823 | 0.900069388 |
| negative regulation of natural killer cell mediated immun | 44572 | 0.791090823 | 0.900069388 |
| negative regulation of potassium ion transmembrane tra | 44572 | 0.791090823 | 0.900069388 |
| negative regulation of protein maturation (GO:1903318) | 44572 | 0.791090823 | 0.900069388 |
| peptidyl-proline hydroxylation (GO:0019511) | 44572 | 0.791090823 | 0.900069388 |
| positive regulation of actin nucleation (GO:0051127) | 44572 | 0.791090823 | 0.900069388 |
| positive regulation of chondrocyte differentiation (GO:00 | 44572 | 0.791090823 | 0.900069388 |
| positive regulation of cytoplasmic transport (GO:190365 | 44572 | 0.791090823 | 0.900069388 |
| positive regulation of neuroblast proliferation (GO:0002C | 44572 | 0.791090823 | 0.900069388 |
| positive regulation of non-canonical Wnt signaling pathw | 44572 | 0.791090823 | 0.900069388 |
| positive regulation of viral release from host cell (GO:190 | 44572 | 0.791090823 | 0.900069388 |
| protein localization to phagophore assembly site (GO:003 | 44572 | 0.791090823 | 0.900069388 |
| protein polyglutamylation (GO:0018095) | 44572 | 0.791090823 | 0.900069388 |
| purine nucleoside bisphosphate biosynthetic process (GC | 44572 | 0.791090823 | 0.900069388 |
| regulation of biosynthetic process (GO:0009889) | 44572 | 0.791090823 | 0.900069388 |
| regulation of endocytic recycling (GO:2001135) | 44572 | 0.791090823 | 0.900069388 |
| regulation of histone H3-K9 acetylation (GO:2000615) | 44572 | 0.791090823 | 0.900069388 |
| regulation of melanin biosynthetic process (GO:0048021) | 44572 | 0.791090823 | 0.900069388 |
| regulation of mRNA 3'-end processing (GO:0031440) | 44572 | 0.791090823 | 0.900069388 |
| regulation of myeloid leukocyte mediated immunity (GO: | 44572 | 0.791090823 | 0.900069388 |

| | | | |
|---|---|---|---|
| response to hydroxyurea (GO:0072710) | 44572 | 0.791090823 | 0.900069388 |
| response to prostaglandin E (GO:0034695) | 44572 | 0.791090823 | 0.900069388 |
| rhodopsin mediated signaling pathway (GO:0016056) | 44572 | 0.791090823 | 0.900069388 |
| ribonucleoside bisphosphate biosynthetic process (GO:0( | 44572 | 0.791090823 | 0.900069388 |
| signal peptide processing (GO:0006465) | 44572 | 0.791090823 | 0.900069388 |
| skeletal muscle cell differentiation (GO:0035914) | 44572 | 0.791090823 | 0.900069388 |
| sphingomyelin metabolic process (GO:0006684) | 44572 | 0.791090823 | 0.900069388 |
| telomere maintenance via recombination (GO:0000722) | 44572 | 0.791090823 | 0.900069388 |
| UDP-N-acetylglucosamine biosynthetic process (GO:0006 | 44572 | 0.791090823 | 0.900069388 |
| V(D)J recombination (GO:0033151) | 44572 | 0.791090823 | 0.900069388 |
| positive regulation of transmembrane receptor protein s | 33878 | 0.794388653 | 0.903623616 |
| negative regulation of cell-substrate adhesion (GO:00108 | 14702 | 0.795634096 | 0.904248332 |
| organic hydroxy compound transport (GO:0015850) | 14702 | 0.795634096 | 0.904248332 |
| positive regulation of ossification (GO:0045778) | 14702 | 0.795634096 | 0.904248332 |
| stem cell differentiation (GO:0048863) | 14702 | 0.795634096 | 0.904248332 |
| modulation of chemical synaptic transmission (GO:00508 | 12/109 | 0.796117511 | 0.90459984 |
| oligosaccharide metabolic process (GO:0009311) | 18019 | 0.796336069 | 0.904650313 |
| activation of protein kinase B activity (GO:0032148) | 44651 | 0.798572574 | 0.905390564 |
| cardiac muscle cell action potential involved in contractic | 44651 | 0.798572574 | 0.905390564 |
| negative regulation of osteoblast differentiation (GO:004 | 44651 | 0.798572574 | 0.905390564 |
| organelle transport along microtubule (GO:0072384) | 44651 | 0.798572574 | 0.905390564 |
| purine nucleoside bisphosphate metabolic process (GO:0 | 44651 | 0.798572574 | 0.905390564 |
| regulation of transmembrane receptor protein serine/thr | 44651 | 0.798572574 | 0.905390564 |
| ribonucleoside bisphosphate metabolic process (GO:003: | 44651 | 0.798572574 | 0.905390564 |
| steroid hormone biosynthetic process (GO:0120178) | 44651 | 0.798572574 | 0.905390564 |
| tube closure (GO:0060606) | 44651 | 0.798572574 | 0.905390564 |
| eye development (GO:0001654) | 21337 | 0.798904783 | 0.905390564 |
| nucleosome assembly (GO:0006334) | 21337 | 0.798904783 | 0.905390564 |
| anaphase-promoting complex-dependent catabolic proce | 30926 | 0.799958444 | 0.90638694 |
| regulation of BMP signaling pathway (GO:0030510) | 27973 | 0.806640768 | 0.909894729 |
| cellular response to nerve growth factor stimulus (GO:19 | 44614 | 0.8095138 | 0.909894729 |
| maturation of 5.8S rRNA from tricistronic rRNA transcript | 44614 | 0.8095138 | 0.909894729 |
| neuronal action potential (GO:0019228) | 44614 | 0.8095138 | 0.909894729 |
| nucleotide-excision repair, DNA duplex unwinding (GO:0( | 44614 | 0.8095138 | 0.909894729 |
| positive regulation of dendritic spine development (GO:0 | 44614 | 0.8095138 | 0.909894729 |
| positive regulation of neural precursor cell proliferation ( | 44614 | 0.8095138 | 0.909894729 |
| protein import into peroxisome membrane (GO:0045046 | 44614 | 0.8095138 | 0.909894729 |
| protein localization to cell junction (GO:1902414) | 44614 | 0.8095138 | 0.909894729 |
| regulation of chromosome organization (GO:0033044) | 44614 | 0.8095138 | 0.909894729 |
| rRNA methylation (GO:0031167) | 44614 | 0.8095138 | 0.909894729 |
| tetrapyrrole metabolic process (GO:0033013) | 44614 | 0.8095138 | 0.909894729 |
| cell junction assembly (GO:0034329) | 11/102 | 0.81054318 | 0.909894729 |
| mitotic sister chromatid segregation (GO:0000070) | 11/102 | 0.81054318 | 0.909894729 |
| neuron migration (GO:0001764) | 18384 | 0.81074202 | 0.909894729 |
| ribosome assembly (GO:0042255) | 18384 | 0.81074202 | 0.909894729 |
| gluconeogenesis (GO:0006094) | 15067 | 0.811443912 | 0.909894729 |
| heart contraction (GO:0060047) | 15067 | 0.811443912 | 0.909894729 |

| | | | |
|---|---|---|---|
| hexose metabolic process (GO:0019318) | 15067 | 0.811443912 | 0.909894729 |
| positive regulation of biomineral tissue development (GC | 15067 | 0.811443912 | 0.909894729 |
| protein methylation (GO:0006479) | 15067 | 0.811443912 | 0.909894729 |
| visual system development (GO:0150063) | 15067 | 0.811443912 | 0.909894729 |
| aortic valve morphogenesis (GO:0003180) | 11749 | 0.816084158 | 0.909894729 |
| axonemal dynein complex assembly (GO:0070286) | 11749 | 0.816084158 | 0.909894729 |
| cellular response to glucose stimulus (GO:0071333) | 11749 | 0.816084158 | 0.909894729 |
| membrane depolarization (GO:0051899) | 11749 | 0.816084158 | 0.909894729 |
| regulation of dendrite morphogenesis (GO:0048814) | 11749 | 0.816084158 | 0.909894729 |
| microtubule cytoskeleton organization involved in mitosi | 14/128 | 0.817203705 | 0.909894729 |
| cholesterol metabolic process (GO:0008203) | 28338 | 0.818042635 | 0.909894729 |
| actin crosslink formation (GO:0051764) | 44573 | 0.818817682 | 0.909894729 |
| amino sugar biosynthetic process (GO:0046349) | 44573 | 0.818817682 | 0.909894729 |
| ATP transport (GO:0015867) | 44573 | 0.818817682 | 0.909894729 |
| attachment of mitotic spindle microtubules to kinetochoı | 44573 | 0.818817682 | 0.909894729 |
| axonal fasciculation (GO:0007413) | 44573 | 0.818817682 | 0.909894729 |
| calcineurin-mediated signaling (GO:0097720) | 44573 | 0.818817682 | 0.909894729 |
| catecholamine biosynthetic process (GO:0042423) | 44573 | 0.818817682 | 0.909894729 |
| cell differentiation in hindbrain (GO:0021533) | 44573 | 0.818817682 | 0.909894729 |
| cochlea morphogenesis (GO:0090103) | 44573 | 0.818817682 | 0.909894729 |
| dermatan sulfate biosynthetic process (GO:0030208) | 44573 | 0.818817682 | 0.909894729 |
| digestive system development (GO:0055123) | 44573 | 0.818817682 | 0.909894729 |
| early endosome to Golgi transport (GO:0034498) | 44573 | 0.818817682 | 0.909894729 |
| endoplasmic reticulum membrane organization (GO:009( | 44573 | 0.818817682 | 0.909894729 |
| epithelial cell-cell adhesion (GO:0090136) | 44573 | 0.818817682 | 0.909894729 |
| ethanol oxidation (GO:0006069) | 44573 | 0.818817682 | 0.909894729 |
| ganglion development (GO:0061548) | 44573 | 0.818817682 | 0.909894729 |
| gas transport (GO:0015669) | 44573 | 0.818817682 | 0.909894729 |
| glomerular visceral epithelial cell differentiation (GO:007 | 44573 | 0.818817682 | 0.909894729 |
| Golgi localization (GO:0051645) | 44573 | 0.818817682 | 0.909894729 |
| hemidesmosome assembly (GO:0031581) | 44573 | 0.818817682 | 0.909894729 |
| magnesium ion transport (GO:0015693) | 44573 | 0.818817682 | 0.909894729 |
| megakaryocyte differentiation (GO:0030219) | 44573 | 0.818817682 | 0.909894729 |
| melanocyte differentiation (GO:0030318) | 44573 | 0.818817682 | 0.909894729 |
| mesoderm morphogenesis (GO:0048332) | 44573 | 0.818817682 | 0.909894729 |
| mitochondrial genome maintenance (GO:0000002) | 44573 | 0.818817682 | 0.909894729 |
| negative regulation of amyloid fibril formation (GO:1905! | 44573 | 0.818817682 | 0.909894729 |
| negative regulation of calcineurin-mediated signaling (GC | 44573 | 0.818817682 | 0.909894729 |
| negative regulation of calcineurin-NFAT signaling cascade | 44573 | 0.818817682 | 0.909894729 |
| negative regulation of cardiac muscle hypertrophy (GO:0 | 44573 | 0.818817682 | 0.909894729 |
| negative regulation of fatty acid biosynthetic process (GC | 44573 | 0.818817682 | 0.909894729 |
| negative regulation of histone acetylation (GO:0035067) | 44573 | 0.818817682 | 0.909894729 |
| negative regulation of microtubule polymerization (GO:0 | 44573 | 0.818817682 | 0.909894729 |
| negative regulation of oxidoreductase activity (GO:00513 | 44573 | 0.818817682 | 0.909894729 |
| negative regulation of transcription elongation from RNA | 44573 | 0.818817682 | 0.909894729 |
| P-body assembly (GO:0033962) | 44573 | 0.818817682 | 0.909894729 |
| peptidyl-methionine modification (GO:0018206) | 44573 | 0.818817682 | 0.909894729 |

| | | | |
|---|---|---|---|
| positive regulation of branching involved in ureteric bud r | 44573 | 0.818817682 | 0.909894729 |
| positive regulation of fatty acid oxidation (GO:0046321) | 44573 | 0.818817682 | 0.909894729 |
| positive regulation of metaphase/anaphase transition of | 44573 | 0.818817682 | 0.909894729 |
| positive regulation of mitotic metaphase/anaphase trans | 44573 | 0.818817682 | 0.909894729 |
| positive regulation of mitotic sister chromatid separation | 44573 | 0.818817682 | 0.909894729 |
| positive regulation of protein depolymerization (GO:1901 | 44573 | 0.818817682 | 0.909894729 |
| positive regulation of vascular permeability (GO:0043117 | 44573 | 0.818817682 | 0.909894729 |
| protein localization to condensed chromosome (GO:1903 | 44573 | 0.818817682 | 0.909894729 |
| protein localization to synapse (GO:0035418) | 44573 | 0.818817682 | 0.909894729 |
| purine nucleoside monophosphate biosynthetic process ( | 44573 | 0.818817682 | 0.909894729 |
| purine nucleotide biosynthetic process (GO:0006164) | 44573 | 0.818817682 | 0.909894729 |
| regulation of axon regeneration (GO:0048679) | 44573 | 0.818817682 | 0.909894729 |
| regulation of CD4-positive, alpha-beta T cell differentiatic | 44573 | 0.818817682 | 0.909894729 |
| regulation of DNA-dependent DNA replication (GO:00903 | 44573 | 0.818817682 | 0.909894729 |
| regulation of muscle system process (GO:0090257) | 44573 | 0.818817682 | 0.909894729 |
| regulation of response to oxidative stress (GO:1902882) | 44573 | 0.818817682 | 0.909894729 |
| regulation of sensory perception (GO:0051931) | 44573 | 0.818817682 | 0.909894729 |
| regulation of sequestering of calcium ion (GO:0051282) | 44573 | 0.818817682 | 0.909894729 |
| regulation of triglyceride catabolic process (GO:0010896) | 44573 | 0.818817682 | 0.909894729 |
| replicative senescence (GO:0090399) | 44573 | 0.818817682 | 0.909894729 |
| sensory perception of taste (GO:0050909) | 44573 | 0.818817682 | 0.909894729 |
| sno(s)RNA 3'-end processing (GO:0031126) | 44573 | 0.818817682 | 0.909894729 |
| sphingoid metabolic process (GO:0046519) | 44573 | 0.818817682 | 0.909894729 |
| thyroid hormone generation (GO:0006590) | 44573 | 0.818817682 | 0.909894729 |
| urogenital system development (GO:0001655) | 44573 | 0.818817682 | 0.909894729 |
| mitotic metaphase plate congression (GO:0007080) | 18749 | 0.824322259 | 0.915815684 |
| regulation of double-strand break repair (GO:2000779) | 15432 | 0.8262379 | 0.917747679 |
| ear morphogenesis (GO:0042471) | 44615 | 0.829011825 | 0.919060271 |
| positive regulation of cell cycle G2/M phase transition (G | 44615 | 0.829011825 | 0.919060271 |
| positive regulation of morphogenesis of an epithelium (G | 44615 | 0.829011825 | 0.919060271 |
| protein alkylation (GO:0008213) | 44615 | 0.829011825 | 0.919060271 |
| regulation of protein export from nucleus (GO:0046825) | 44615 | 0.829011825 | 0.919060271 |
| regulation of supramolecular fiber organization (GO:1902 | 44615 | 0.829011825 | 0.919060271 |
| regulation of telomere capping (GO:1904353) | 44615 | 0.829011825 | 0.919060271 |
| respiratory chain complex IV assembly (GO:0008535) | 44615 | 0.829011825 | 0.919060271 |
| response to nutrient levels (GO:0031667) | 44615 | 0.829011825 | 0.919060271 |
| bile acid metabolic process (GO:0008206) | 12114 | 0.83228424 | 0.922097809 |
| mitotic G2 DNA damage checkpoint signaling (GO:00070S | 12114 | 0.83228424 | 0.922097809 |
| vasculogenesis (GO:0001570) | 12114 | 0.83228424 | 0.922097809 |
| positive regulation of DNA biosynthetic process (GO:200( | 22433 | 0.836553051 | 0.923189446 |
| response to light stimulus (GO:0009416) | 22433 | 0.836553051 | 0.923189446 |
| endocytic recycling (GO:0032456) | 19115 | 0.837102227 | 0.923189446 |
| negative regulation of BMP signaling pathway (GO:00305 | 19115 | 0.837102227 | 0.923189446 |
| regulation of release of sequestered calcium ion into cytc | 19115 | 0.837102227 | 0.923189446 |
| regulation of telomere maintenance via telomerase (GO:! | 19115 | 0.837102227 | 0.923189446 |
| COPII-coated vesicle budding (GO:0090114) | 25750 | 0.837459294 | 0.923189446 |
| potassium ion transport (GO:0006813) | 13/122 | 0.838080885 | 0.923189446 |

| | | | |
|---|---|---|---|
| potassium ion transmembrane transport (GO:0071805) | 15/139 | 0.838611243 | 0.923189446 |
| axon development (GO:0061564) | 29068 | 0.839282433 | 0.923189446 |
| regulation of cellular response to heat (GO:1900034) | 29068 | 0.839282433 | 0.923189446 |
| regulation of ion transport (GO:0043269) | 29068 | 0.839282433 | 0.923189446 |
| glutathione metabolic process (GO:0006749) | 15797 | 0.840055253 | 0.923189446 |
| cell cycle G2/M phase transition (GO:0044839) | 14/131 | 0.842189917 | 0.923189446 |
| alpha-linolenic acid metabolic process (GO:0036109) | 44574 | 0.842865744 | 0.923189446 |
| cell part morphogenesis (GO:0032990) | 44574 | 0.842865744 | 0.923189446 |
| central nervous system projection neuron axonogenesis ( | 44574 | 0.842865744 | 0.923189446 |
| cranial nerve development (GO:0021545) | 44574 | 0.842865744 | 0.923189446 |
| dermatan sulfate metabolic process (GO:0030205) | 44574 | 0.842865744 | 0.923189446 |
| detection of calcium ion (GO:0005513) | 44574 | 0.842865744 | 0.923189446 |
| DNA deamination (GO:0045006) | 44574 | 0.842865744 | 0.923189446 |
| DNA replication-independent nucleosome organization (( | 44574 | 0.842865744 | 0.923189446 |
| endochondral bone morphogenesis (GO:0060350) | 44574 | 0.842865744 | 0.923189446 |
| extracellular transport (GO:0006858) | 44574 | 0.842865744 | 0.923189446 |
| inner dynein arm assembly (GO:0036159) | 44574 | 0.842865744 | 0.923189446 |
| kinetochore organization (GO:0051383) | 44574 | 0.842865744 | 0.923189446 |
| lipid oxidation (GO:0034440) | 44574 | 0.842865744 | 0.923189446 |
| microtubule-based transport (GO:0099111) | 44574 | 0.842865744 | 0.923189446 |
| mitochondrion transport along microtubule (GO:004749) | 44574 | 0.842865744 | 0.923189446 |
| negative regulation of cell morphogenesis involved in diff | 44574 | 0.842865744 | 0.923189446 |
| negative regulation of cellular senescence (GO:2000773) | 44574 | 0.842865744 | 0.923189446 |
| negative regulation of DNA-dependent DNA replication (( | 44574 | 0.842865744 | 0.923189446 |
| negative regulation of epidermal growth factor-activated | 44574 | 0.842865744 | 0.923189446 |
| negative regulation of nucleocytoplasmic transport (GO:( | 44574 | 0.842865744 | 0.923189446 |
| negative regulation of substrate adhesion-dependent cel | 44574 | 0.842865744 | 0.923189446 |
| plasma membrane repair (GO:0001778) | 44574 | 0.842865744 | 0.923189446 |
| positive regulation of calcineurin-mediated signaling (GO | 44574 | 0.842865744 | 0.923189446 |
| positive regulation of calcineurin-NFAT signaling cascade | 44574 | 0.842865744 | 0.923189446 |
| positive regulation of insulin receptor signaling pathway ( | 44574 | 0.842865744 | 0.923189446 |
| positive regulation of lipid transport (GO:0032370) | 44574 | 0.842865744 | 0.923189446 |
| positive regulation of sodium ion transport (GO:0010765 | 44574 | 0.842865744 | 0.923189446 |
| positive regulation of steroid metabolic process (GO:004! | 44574 | 0.842865744 | 0.923189446 |
| positive regulation of vascular endothelial cell proliferatic | 44574 | 0.842865744 | 0.923189446 |
| protein neddylation (GO:0045116) | 44574 | 0.842865744 | 0.923189446 |
| regulation of histone modification (GO:0031056) | 44574 | 0.842865744 | 0.923189446 |
| regulation of membrane repolarization (GO:0060306) | 44574 | 0.842865744 | 0.923189446 |
| regulation of mitotic cell cycle spindle assembly checkpoi | 44574 | 0.842865744 | 0.923189446 |
| regulation of nucleocytoplasmic transport (GO:0046822) | 44574 | 0.842865744 | 0.923189446 |
| regulation of T cell differentiation in thymus (GO:003308 | 44574 | 0.842865744 | 0.923189446 |
| reticulophagy (GO:0061709) | 44574 | 0.842865744 | 0.923189446 |
| spliceosomal tri-snRNP complex assembly (GO:0000244) | 44574 | 0.842865744 | 0.923189446 |
| transcription preinitiation complex assembly (GO:00708! | 44574 | 0.842865744 | 0.923189446 |
| triglyceride-rich lipoprotein particle remodeling (GO:003 | 44574 | 0.842865744 | 0.923189446 |
| UDP-N-acetylglucosamine metabolic process (GO:000604 | 44574 | 0.842865744 | 0.923189446 |
| C21-steroid hormone metabolic process (GO:0008207) | 44616 | 0.846691068 | 0.92427877 |

| | | | |
|---|---|---|---|
| dorsal/ventral pattern formation (GO:0009953) | 44616 | 0.846691068 | 0.92427877 |
| establishment of mitotic spindle orientation (GO:000013 | 44616 | 0.846691068 | 0.92427877 |
| inner ear morphogenesis (GO:0042472) | 44616 | 0.846691068 | 0.92427877 |
| neuromuscular junction development (GO:0007528) | 44616 | 0.846691068 | 0.92427877 |
| nucleotide-excision repair, DNA gap filling (GO:0006297) | 44616 | 0.846691068 | 0.92427877 |
| positive regulation of phospholipase activity (GO:001051 | 44616 | 0.846691068 | 0.92427877 |
| protein insertion into mitochondrial membrane (GO:005 | 44616 | 0.846691068 | 0.92427877 |
| regulation of cellular response to transforming growth fa | 44616 | 0.846691068 | 0.92427877 |
| negative regulation of mitochondrion organization (GO:0 | 12479 | 0.847240713 | 0.92427877 |
| neural tube closure (GO:0001843) | 12479 | 0.847240713 | 0.92427877 |
| phosphatidylserine metabolic process (GO:0006658) | 12479 | 0.847240713 | 0.92427877 |
| positive regulation of calcium ion transport into cytosol ( | 12479 | 0.847240713 | 0.92427877 |
| positive regulation of telomerase activity (GO:0051973) | 12479 | 0.847240713 | 0.92427877 |
| protein insertion into membrane (GO:0051205) | 12479 | 0.847240713 | 0.92427877 |
| regulation of glucose metabolic process (GO:0010906) | 12479 | 0.847240713 | 0.92427877 |
| sister chromatid segregation (GO:0000819) | 12479 | 0.847240713 | 0.92427877 |
| striated muscle tissue development (GO:0014706) | 12479 | 0.847240713 | 0.92427877 |
| synaptic vesicle recycling (GO:0036465) | 12479 | 0.847240713 | 0.92427877 |
| endomembrane system organization (GO:0010256) | 22/199 | 0.848768455 | 0.925751024 |
| intracellular protein transmembrane transport (GO:0065 | 16163 | 0.852937299 | 0.930102701 |
| cytoskeleton-dependent cytokinesis (GO:0061640) | 26481 | 0.857901329 | 0.93246898 |
| monocarboxylic acid biosynthetic process (GO:0072330) | 23163 | 0.858338968 | 0.93246898 |
| striated muscle contraction (GO:0006941) | 19845 | 0.860372532 | 0.93246898 |
| embryonic skeletal system development (GO:0048706) | 12844 | 0.861022786 | 0.93246898 |
| muscle cell differentiation (GO:0042692) | 12844 | 0.861022786 | 0.93246898 |
| regulation of blood circulation (GO:1903522) | 12844 | 0.861022786 | 0.93246898 |
| regulation of postsynaptic membrane potential (GO:006 | 12844 | 0.861022786 | 0.93246898 |
| regulation of protein polymerization (GO:0032271) | 12844 | 0.861022786 | 0.93246898 |
| smoothened signaling pathway (GO:0007224) | 12844 | 0.861022786 | 0.93246898 |
| cAMP-mediated signaling (GO:0019933) | 44617 | 0.862690762 | 0.93246898 |
| regulation of rhodopsin mediated signaling pathway (GO | 44617 | 0.862690762 | 0.93246898 |
| regulation of ryanodine-sensitive calcium-release channe | 44617 | 0.862690762 | 0.93246898 |
| ribosomal large subunit assembly (GO:0000027) | 44617 | 0.862690762 | 0.93246898 |
| RNA splicing, via transesterification reactions (GO:00003 | 44617 | 0.862690762 | 0.93246898 |
| atrial septum development (GO:0003283) | 44575 | 0.863722951 | 0.93246898 |
| atrioventricular valve development (GO:0003171) | 44575 | 0.863722951 | 0.93246898 |
| cardiac left ventricle morphogenesis (GO:0003214) | 44575 | 0.863722951 | 0.93246898 |
| cell junction maintenance (GO:0034331) | 44575 | 0.863722951 | 0.93246898 |
| COPII-coated vesicle cargo loading (GO:0090110) | 44575 | 0.863722951 | 0.93246898 |
| ether lipid metabolic process (GO:0046485) | 44575 | 0.863722951 | 0.93246898 |
| heart process (GO:0003015) | 44575 | 0.863722951 | 0.93246898 |
| histone H3-K4 trimethylation (GO:0080182) | 44575 | 0.863722951 | 0.93246898 |
| intestinal absorption (GO:0050892) | 44575 | 0.863722951 | 0.93246898 |
| ncRNA transcription (GO:0098781) | 44575 | 0.863722951 | 0.93246898 |
| negative regulation of cell activation (GO:0050866) | 44575 | 0.863722951 | 0.93246898 |
| negative regulation of DNA-templated transcription, elon | 44575 | 0.863722951 | 0.93246898 |
| negative regulation of fibroblast growth factor receptor s | 44575 | 0.863722951 | 0.93246898 |

| | | | |
|---|---|---|---|
| negative regulation of mRNA splicing, via spliceosome (G | 44575 | 0.863722951 | 0.93246898 |
| negative regulation of T cell differentiation (GO:0045581) | 44575 | 0.863722951 | 0.93246898 |
| neurotransmitter biosynthetic process (GO:0042136) | 44575 | 0.863722951 | 0.93246898 |
| ovarian follicle development (GO:0001541) | 44575 | 0.863722951 | 0.93246898 |
| pancreas development (GO:0031016) | 44575 | 0.863722951 | 0.93246898 |
| positive regulation of cellular response to insulin stimulus | 44575 | 0.863722951 | 0.93246898 |
| positive regulation of mitochondrial translation (GO:007( | 44575 | 0.863722951 | 0.93246898 |
| protein localization to kinetochore (GO:0034501) | 44575 | 0.863722951 | 0.93246898 |
| protein maturation by iron-sulfur cluster transfer (GO:00! | 44575 | 0.863722951 | 0.93246898 |
| quinone biosynthetic process (GO:1901663) | 44575 | 0.863722951 | 0.93246898 |
| regulation of cAMP-dependent protein kinase activity (G( | 44575 | 0.863722951 | 0.93246898 |
| regulation of gastrulation (GO:0010470) | 44575 | 0.863722951 | 0.93246898 |
| regulation of histone deacetylation (GO:0031063) | 44575 | 0.863722951 | 0.93246898 |
| regulation of lipase activity (GO:0060191) | 44575 | 0.863722951 | 0.93246898 |
| regulation of sensory perception of pain (GO:0051930) | 44575 | 0.863722951 | 0.93246898 |
| regulation of transcription of nucleolar large rRNA by RN/ | 44575 | 0.863722951 | 0.93246898 |
| respiratory electron transport chain (GO:0022904) | 44575 | 0.863722951 | 0.93246898 |
| response to UV-A (GO:0070141) | 44575 | 0.863722951 | 0.93246898 |
| retinal metabolic process (GO:0042574) | 44575 | 0.863722951 | 0.93246898 |
| sulfate transport (GO:0008272) | 44575 | 0.863722951 | 0.93246898 |
| tRNA wobble uridine modification (GO:0002098) | 44575 | 0.863722951 | 0.93246898 |
| microtubule bundle formation (GO:0001578) | 16528 | 0.86492682 | 0.933574658 |
| protein-DNA complex assembly (GO:0065004) | 15/143 | 0.867495849 | 0.936153082 |
| diterpenoid metabolic process (GO:0016101) | 23529 | 0.868300732 | 0.936438095 |
| negative regulation of cell cycle process (GO:0010948) | 23529 | 0.868300732 | 0.936438095 |
| pre-replicative complex assembly (GO:0036388) | 23529 | 0.868300732 | 0.936438095 |
| regulation of sodium ion transport (GO:0002028) | 13210 | 0.873699938 | 0.942065416 |
| regulation of cellular protein localization (GO:1903827) | 16893 | 0.876067455 | 0.944344708 |
| global genome nucleotide-excision repair (GO:0070911) | 44618 | 0.877145215 | 0.944344708 |
| histone methylation (GO:0016571) | 44618 | 0.877145215 | 0.944344708 |
| epithelial cell differentiation (GO:0030855) | 10/101 | 0.877257949 | 0.944344708 |
| ribonucleoprotein complex assembly (GO:0022618) | 14/136 | 0.877925158 | 0.944344708 |
| aflatoxin metabolic process (GO:0046222) | 44576 | 0.881812547 | 0.944344708 |
| catecholamine metabolic process (GO:0006584) | 44576 | 0.881812547 | 0.944344708 |
| detection of mechanical stimulus involved in sensory per | 44576 | 0.881812547 | 0.944344708 |
| electron transport chain (GO:0022900) | 44576 | 0.881812547 | 0.944344708 |
| eye photoreceptor cell development (GO:0042462) | 44576 | 0.881812547 | 0.944344708 |
| G protein-coupled glutamate receptor signaling pathway | 44576 | 0.881812547 | 0.944344708 |
| glucosamine-containing compound metabolic process (G | 44576 | 0.881812547 | 0.944344708 |
| medium-chain fatty acid metabolic process (GO:0051791 | 44576 | 0.881812547 | 0.944344708 |
| mitotic recombination (GO:0006312) | 44576 | 0.881812547 | 0.944344708 |
| mycotoxin metabolic process (GO:0043385) | 44576 | 0.881812547 | 0.944344708 |
| myoblast fusion (GO:0007520) | 44576 | 0.881812547 | 0.944344708 |
| negative regulation of cellular component movement (G( | 44576 | 0.881812547 | 0.944344708 |
| negative regulation of mRNA processing (GO:0050686) | 44576 | 0.881812547 | 0.944344708 |
| organic heteropentacyclic compound metabolic process ( | 44576 | 0.881812547 | 0.944344708 |
| photoreceptor cell development (GO:0042461) | 44576 | 0.881812547 | 0.944344708 |

| | | | |
|---|---|---|---|
| phototransduction, visible light (GO:0007603) | 44576 | 0.881812547 | 0.944344708 |
| positive regulation of double-strand break repair via hom | 44576 | 0.881812547 | 0.944344708 |
| positive regulation of glycogen metabolic process (GO:00 | 44576 | 0.881812547 | 0.944344708 |
| positive regulation of histone H3-K4 methylation (GO:005 | 44576 | 0.881812547 | 0.944344708 |
| protein kinase A signaling (GO:0010737) | 44576 | 0.881812547 | 0.944344708 |
| protein localization to chromosome, telomeric region (G( | 44576 | 0.881812547 | 0.944344708 |
| regulation of branching involved in ureteric bud morphog | 44576 | 0.881812547 | 0.944344708 |
| regulation of cytoplasmic translation (GO:2000765) | 44576 | 0.881812547 | 0.944344708 |
| regulation of establishment of protein localization (GO:0( | 44576 | 0.881812547 | 0.944344708 |
| regulation of non-canonical Wnt signaling pathway (GO:2 | 44576 | 0.881812547 | 0.944344708 |
| regulation of phospholipase C activity (GO:1900274) | 44576 | 0.881812547 | 0.944344708 |
| ubiquinone biosynthetic process (GO:0006744) | 44576 | 0.881812547 | 0.944344708 |
| ubiquinone metabolic process (GO:0006743) | 44576 | 0.881812547 | 0.944344708 |
| protein O-linked glycosylation (GO:0006493) | 10/102 | 0.884509539 | 0.947037728 |
| acyl-CoA metabolic process (GO:0006637) | 13575 | 0.885341064 | 0.947395415 |
| proton transmembrane transport (GO:1902600) | 13575 | 0.885341064 | 0.947395415 |
| organelle organization (GO:0006996) | 48/420 | 0.88539071 | 0.947395415 |
| bile acid biosynthetic process (GO:0006699) | 44619 | 0.890182673 | 0.950565054 |
| microtubule polymerization or depolymerization (GO:003 | 44619 | 0.890182673 | 0.950565054 |
| monovalent inorganic cation homeostasis (GO:0055067) | 44619 | 0.890182673 | 0.950565054 |
| peptidyl-lysine methylation (GO:0018022) | 44619 | 0.890182673 | 0.950565054 |
| peroxisomal membrane transport (GO:0015919) | 44619 | 0.890182673 | 0.950565054 |
| positive regulation of BMP signaling pathway (GO:00305: | 44619 | 0.890182673 | 0.950565054 |
| positive regulation of calcium ion transmembrane transp | 44619 | 0.890182673 | 0.950565054 |
| protein peptidyl-prolyl isomerization (GO:0000413) | 44619 | 0.890182673 | 0.950565054 |
| regulation of cardiac muscle cell contraction (GO:008600 | 44619 | 0.890182673 | 0.950565054 |
| regulation of transcription regulatory region DNA binding | 44619 | 0.890182673 | 0.950565054 |
| regulation of cell cycle G2/M phase transition (GO:19027 | 31260 | 0.891389363 | 0.951657982 |
| actomyosin structure organization (GO:0031032) | 27942 | 0.892495251 | 0.952447171 |
| establishment of protein localization to organelle (GO:00 | 27942 | 0.892495251 | 0.952447171 |
| establishment of protein localization to mitochondrion (( | 17624 | 0.895977855 | 0.954066113 |
| actin-myosin filament sliding (GO:0033275) | 13940 | 0.896013783 | 0.954066113 |
| muscle filament sliding (GO:0030049) | 13940 | 0.896013783 | 0.954066113 |
| negative regulation of endothelial cell migration (GO:001 | 13940 | 0.896013783 | 0.954066113 |
| regulation of axon extension (GO:0030516) | 13940 | 0.896013783 | 0.954066113 |
| sterol biosynthetic process (GO:0016126) | 13940 | 0.896013783 | 0.954066113 |
| heme catabolic process (GO:0042167) | 44577 | 0.897501654 | 0.954066113 |
| mesenchyme development (GO:0060485) | 44577 | 0.897501654 | 0.954066113 |
| mRNA cis splicing, via spliceosome (GO:0045292) | 44577 | 0.897501654 | 0.954066113 |
| N-acetylglucosamine metabolic process (GO:0006044) | 44577 | 0.897501654 | 0.954066113 |
| negative regulation of histone modification (GO:0031057 | 44577 | 0.897501654 | 0.954066113 |
| negative regulation of release of cytochrome c from mitc | 44577 | 0.897501654 | 0.954066113 |
| neural tube development (GO:0021915) | 44577 | 0.897501654 | 0.954066113 |
| oligodendrocyte development (GO:0014003) | 44577 | 0.897501654 | 0.954066113 |
| porphyrin-containing compound catabolic process (GO:0( | 44577 | 0.897501654 | 0.954066113 |
| positive regulation of collagen biosynthetic process (GO:( | 44577 | 0.897501654 | 0.954066113 |
| regulation of cellular carbohydrate metabolic process (G( | 44577 | 0.897501654 | 0.954066113 |

| | | | |
|---|---|---|---|
| retrograde axonal transport (GO:0008090) | 44577 | 0.897501654 | 0.954066113 |
| transepithelial transport (GO:0070633) | 44577 | 0.897501654 | 0.954066113 |
| chordate embryonic development (GO:0043009) | 21306 | 0.898596813 | 0.954839526 |
| regulation of pathway-restricted SMAD protein phosphor | 21306 | 0.898596813 | 0.954839526 |
| bile acid and bile salt transport (GO:0015721) | 44620 | 0.90192456 | 0.957005328 |
| macromolecule catabolic process (GO:0009057) | 44620 | 0.90192456 | 0.957005328 |
| negative regulation of axonogenesis (GO:0050771) | 44620 | 0.90192456 | 0.957005328 |
| protein depolymerization (GO:0051261) | 44620 | 0.90192456 | 0.957005328 |
| regulation of small molecule metabolic process (GO:0062 | 44620 | 0.90192456 | 0.957005328 |
| regulation of sodium ion transmembrane transport (GO:1 | 44620 | 0.90192456 | 0.957005328 |
| sarcomere organization (GO:0045214) | 44620 | 0.90192456 | 0.957005328 |
| mitochondrion organization (GO:0007005) | 18/175 | 0.903148542 | 0.958108368 |
| regulation of exit from mitosis (GO:0007096) | 14305 | 0.905783887 | 0.960707899 |
| mitotic DNA damage checkpoint signaling (GO:0044773) | 21671 | 0.90660183 | 0.961379161 |
| adenylate cyclase-activating adrenergic receptor signaling | 44578 | 0.911108712 | 0.962230079 |
| cellular biogenic amine biosynthetic process (GO:004240 | 44578 | 0.911108712 | 0.962230079 |
| cellular response to gamma radiation (GO:0071480) | 44578 | 0.911108712 | 0.962230079 |
| detection of visible light (GO:0009584) | 44578 | 0.911108712 | 0.962230079 |
| embryo development ending in birth or egg hatching (GC | 44578 | 0.911108712 | 0.962230079 |
| epithelial cell morphogenesis (GO:0003382) | 44578 | 0.911108712 | 0.962230079 |
| gliogenesis (GO:0042063) | 44578 | 0.911108712 | 0.962230079 |
| maturation of 5.8S rRNA (GO:0000460) | 44578 | 0.911108712 | 0.962230079 |
| mitochondrial ATP synthesis coupled proton transport (G | 44578 | 0.911108712 | 0.962230079 |
| negative regulation of cell aging (GO:0090344) | 44578 | 0.911108712 | 0.962230079 |
| positive regulation of dendrite extension (GO:1903861) | 44578 | 0.911108712 | 0.962230079 |
| positive regulation of heart rate (GO:0010460) | 44578 | 0.911108712 | 0.962230079 |
| preassembly of GPI anchor in ER membrane (GO:001625 | 44578 | 0.911108712 | 0.962230079 |
| protein heterooligomerization (GO:0051291) | 44578 | 0.911108712 | 0.962230079 |
| protein localization to chromosome, centromeric region | 44578 | 0.911108712 | 0.962230079 |
| pulmonary valve morphogenesis (GO:0003184) | 44578 | 0.911108712 | 0.962230079 |
| purine ribonucleotide transport (GO:0015868) | 44578 | 0.911108712 | 0.962230079 |
| regulation of cellular response to growth factor stimulus | 44578 | 0.911108712 | 0.962230079 |
| ribosomal small subunit assembly (GO:0000028) | 44578 | 0.911108712 | 0.962230079 |
| snRNA metabolic process (GO:0016073) | 44578 | 0.911108712 | 0.962230079 |
| determination of bilateral symmetry (GO:0009855) | 47150 | 0.912485033 | 0.962900619 |
| glial cell differentiation (GO:0010001) | 47150 | 0.912485033 | 0.962900619 |
| nucleotide-excision repair, preincision complex assembly | 47150 | 0.912485033 | 0.962900619 |
| telomere maintenance via telomerase (GO:0007004) | 47150 | 0.912485033 | 0.962900619 |
| regulation of microtubule polymerization (GO:0031113) | 14671 | 0.914714921 | 0.96505768 |
| collagen fibril organization (GO:0030199) | 32721 | 0.917619283 | 0.967925314 |
| maturation of SSU-rRNA from tricistronic rRNA transcript | 10990 | 0.921970773 | 0.969372644 |
| protein N-linked glycosylation via asparagine (GO:001827 | 10990 | 0.921970773 | 0.969372644 |
| regulation of catecholamine secretion (GO:0050433) | 10990 | 0.921970773 | 0.969372644 |
| regulation of vasoconstriction (GO:0019229) | 10990 | 0.921970773 | 0.969372644 |
| RNA-dependent DNA biosynthetic process (GO:0006278) | 10990 | 0.921970773 | 0.969372644 |
| snRNA 3'-end processing (GO:0034472) | 10990 | 0.921970773 | 0.969372644 |
| adenine nucleotide transport (GO:0051503) | 44579 | 0.922909932 | 0.969372644 |

| | | | |
|---|---|---|---|
| base conversion or substitution editing (GO:0016553) | 44579 | 0.922909932 | 0.969372644 |
| cellular response to zinc ion (GO:0071294) | 44579 | 0.922909932 | 0.969372644 |
| embryonic heart tube development (GO:0035050) | 44579 | 0.922909932 | 0.969372644 |
| fluid transport (GO:0042044) | 44579 | 0.922909932 | 0.969372644 |
| negative regulation of peptide hormone secretion (GO:0( | 44579 | 0.922909932 | 0.969372644 |
| negative regulation of tumor necrosis factor-mediated si | 44579 | 0.922909932 | 0.969372644 |
| nitric oxide mediated signal transduction (GO:0007263) | 44579 | 0.922909932 | 0.969372644 |
| regulation of cartilage development (GO:0061035) | 44579 | 0.922909932 | 0.969372644 |
| regulation of delayed rectifier potassium channel activity | 44579 | 0.922909932 | 0.969372644 |
| regulation of dendrite extension (GO:1903859) | 44579 | 0.922909932 | 0.969372644 |
| regulation of mRNA polyadenylation (GO:1900363) | 44579 | 0.922909932 | 0.969372644 |
| regulation of ventricular cardiac muscle cell membrane re | 44579 | 0.922909932 | 0.969372644 |
| sodium ion import across plasma membrane (GO:009871 | 44579 | 0.922909932 | 0.969372644 |
| tRNA metabolic process (GO:0006399) | 44579 | 0.922909932 | 0.969372644 |
| organelle assembly (GO:0070925) | 47/425 | 0.926099855 | 0.972526531 |
| antigen processing and presentation of peptide antigen v | 9/100 | 0.926970963 | 0.973244574 |
| determination of left/right symmetry (GO:0007368) | 15401 | 0.930300912 | 0.975782553 |
| positive regulation of ATPase activity (GO:0032781) | 15401 | 0.930300912 | 0.975782553 |
| embryonic skeletal system morphogenesis (GO:0048704) | 11355 | 0.930480957 | 0.975782553 |
| peptidyl-asparagine modification (GO:0018196) | 11355 | 0.930480957 | 0.975782553 |
| regulation of potassium ion transport (GO:0043266) | 11355 | 0.930480957 | 0.975782553 |
| cell-cell junction organization (GO:0045216) | 30133 | 0.930900899 | 0.975782553 |
| central nervous system development (GO:0007417) | 28/268 | 0.931605505 | 0.975782553 |
| atrioventricular valve morphogenesis (GO:0003181) | 44580 | 0.933144894 | 0.975782553 |
| DNA double-strand break processing (GO:0000729) | 44580 | 0.933144894 | 0.975782553 |
| ethanol metabolic process (GO:0006067) | 44580 | 0.933144894 | 0.975782553 |
| heparan sulfate proteoglycan biosynthetic process (GO:0 | 44580 | 0.933144894 | 0.975782553 |
| nuclear membrane organization (GO:0071763) | 44580 | 0.933144894 | 0.975782553 |
| nucleotide-sugar biosynthetic process (GO:0009226) | 44580 | 0.933144894 | 0.975782553 |
| piRNA metabolic process (GO:0034587) | 44580 | 0.933144894 | 0.975782553 |
| positive regulation of G protein-coupled receptor signalir | 44580 | 0.933144894 | 0.975782553 |
| positive regulation of mitochondrial fission (GO:0090141 | 44580 | 0.933144894 | 0.975782553 |
| protein import into mitochondrial matrix (GO:0030150) | 44580 | 0.933144894 | 0.975782553 |
| purine ribonucleoside bisphosphate metabolic process (G | 44580 | 0.933144894 | 0.975782553 |
| synaptic transmission, cholinergic (GO:0007271) | 44580 | 0.933144894 | 0.975782553 |
| telomere capping (GO:0016233) | 44580 | 0.933144894 | 0.975782553 |
| retinoid metabolic process (GO:0001523) | 33817 | 0.933536564 | 0.975995663 |
| cardiac muscle tissue development (GO:0048738) | 19450 | 0.933921409 | 0.976201553 |
| endoplasmic reticulum organization (GO:0007029) | 26816 | 0.934229287 | 0.976326925 |
| epidermis development (GO:0008544) | 30498 | 0.935971864 | 0.977430368 |
| development of primary male sexual characteristics (GO: | 15766 | 0.937069307 | 0.977430368 |
| male gonad development (GO:0008584) | 15766 | 0.937069307 | 0.977430368 |
| myofibril assembly (GO:0030239) | 15766 | 0.937069307 | 0.977430368 |
| positive regulation of neuron differentiation (GO:004566 | 15766 | 0.937069307 | 0.977430368 |
| positive regulation of peptide hormone secretion (GO:00 | 15766 | 0.937069307 | 0.977430368 |
| signal release from synapse (GO:0099643) | 15766 | 0.937069307 | 0.977430368 |
| positive regulation of transferase activity (GO:0051347) | 14/148 | 0.937694707 | 0.977430368 |

| | | | |
|---|---|---|---|
| cardiac muscle tissue morphogenesis (GO:0055008) | 11720 | 0.938107375 | 0.977430368 |
| intra-Golgi vesicle-mediated transport (GO:0006891) | 11720 | 0.938107375 | 0.977430368 |
| long-chain fatty acid transport (GO:0015909) | 11720 | 0.938107375 | 0.977430368 |
| mitochondrial membrane organization (GO:0007006) | 11720 | 0.938107375 | 0.977430368 |
| peptidyl-proline modification (GO:0018208) | 11720 | 0.938107375 | 0.977430368 |
| regulation of dendritic spine morphogenesis (GO:006100 | 11720 | 0.938107375 | 0.977430368 |
| regulation of intracellular estrogen receptor signaling pat | 11720 | 0.938107375 | 0.977430368 |
| inorganic anion transport (GO:0015698) | 19815 | 0.939805294 | 0.978806837 |
| regulation of potassium ion transmembrane transport (G | 19815 | 0.939805294 | 0.978806837 |
| protein targeting to ER (GO:0045047) | 9/103 | 0.940723179 | 0.979543872 |
| cellular macromolecule biosynthetic process (GO:003464 | 33/314 | 0.94128834 | 0.979543872 |
| adrenergic receptor signaling pathway (GO:0071875) | 44581 | 0.942021403 | 0.979543872 |
| early endosome to late endosome transport (GO:004502 | 44581 | 0.942021403 | 0.979543872 |
| fatty acid transport (GO:0015908) | 44581 | 0.942021403 | 0.979543872 |
| neuromuscular synaptic transmission (GO:0007274) | 44581 | 0.942021403 | 0.979543872 |
| oligodendrocyte differentiation (GO:0048709) | 44581 | 0.942021403 | 0.979543872 |
| positive regulation of hormone secretion (GO:0046887) | 44581 | 0.942021403 | 0.979543872 |
| negative regulation of cell death (GO:0060548) | 34547 | 0.942592359 | 0.979941426 |
| detection of chemical stimulus involved in sensory perce | 16132 | 0.94322528 | 0.979967596 |
| neurotransmitter secretion (GO:0007269) | 16132 | 0.94322528 | 0.979967596 |
| positive regulation of cell division (GO:0051781) | 16132 | 0.94322528 | 0.979967596 |
| nuclear-transcribed mRNA catabolic process, nonsense-m | 10/113 | 0.943372079 | 0.979967596 |
| inorganic anion transmembrane transport (GO:0098661) | 23863 | 0.943952369 | 0.980374362 |
| steroid metabolic process (GO:0008202) | 9/104 | 0.944774424 | 0.980610358 |
| positive regulation of telomere maintenance via telomer | 12086 | 0.944934639 | 0.980610358 |
| primary alcohol metabolic process (GO:0034308) | 12086 | 0.944934639 | 0.980610358 |
| regulation of histone acetylation (GO:0035065) | 12086 | 0.944934639 | 0.980610358 |
| protein import into nucleus (GO:0006606) | 27912 | 0.948535755 | 0.982823805 |
| nucleotide-excision repair (GO:0006289) | 9/105 | 0.948580032 | 0.982823805 |
| chromosome condensation (GO:0030261) | 16497 | 0.948817998 | 0.982823805 |
| regulation of DNA repair (GO:0006282) | 16497 | 0.948817998 | 0.982823805 |
| calcium ion transport into cytosol (GO:0060402) | 44582 | 0.949719701 | 0.982823805 |
| cellular response to inorganic substance (GO:0071241) | 44582 | 0.949719701 | 0.982823805 |
| error-prone translesion synthesis (GO:0042276) | 44582 | 0.949719701 | 0.982823805 |
| innate immune response in mucosa (GO:0002227) | 44582 | 0.949719701 | 0.982823805 |
| negative regulation of response to wounding (GO:19030 | 44582 | 0.949719701 | 0.982823805 |
| nuclear envelope organization (GO:0006998) | 44582 | 0.949719701 | 0.982823805 |
| regulation of cholesterol metabolic process (GO:0090181 | 44582 | 0.949719701 | 0.982823805 |
| regulation of transposition (GO:0010528) | 44582 | 0.949719701 | 0.982823805 |
| retinol metabolic process (GO:0042572) | 44582 | 0.949719701 | 0.982823805 |
| sensory organ development (GO:0007423) | 20546 | 0.95016323 | 0.982823805 |
| tight junction assembly (GO:0120192) | 20546 | 0.95016323 | 0.982823805 |
| cellular amide metabolic process (GO:0043603) | 10/115 | 0.950646144 | 0.982823805 |
| cell-cell adhesion via plasma-membrane adhesion molecu | 16/170 | 0.950832033 | 0.982823805 |
| histone lysine methylation (GO:0034968) | 12451 | 0.951040475 | 0.982823805 |
| secondary alcohol biosynthetic process (GO:1902653) | 12451 | 0.951040475 | 0.982823805 |
| termination of RNA polymerase II transcription (GO:0006 | 12451 | 0.951040475 | 0.982823805 |

| | | | |
|---|---|---|---|
| tRNA methylation (GO:0030488) | 12451 | 0.951040475 | 0.982823805 |
| ribosomal large subunit biogenesis (GO:0042273) | 20911 | 0.954703624 | 0.985325605 |
| chaperone-mediated protein complex assembly (GO:005 | 44583 | 0.956396126 | 0.985325605 |
| exogenous drug catabolic process (GO:0042738) | 44583 | 0.956396126 | 0.985325605 |
| female gonad development (GO:0008585) | 44583 | 0.956396126 | 0.985325605 |
| glutathione derivative biosynthetic process (GO:1901687 | 44583 | 0.956396126 | 0.985325605 |
| glutathione derivative metabolic process (GO:1901685) | 44583 | 0.956396126 | 0.985325605 |
| homologous chromosome segregation (GO:0045143) | 44583 | 0.956396126 | 0.985325605 |
| limb morphogenesis (GO:0035108) | 44583 | 0.956396126 | 0.985325605 |
| negative regulation of lipid biosynthetic process (GO:005 | 44583 | 0.956396126 | 0.985325605 |
| organelle fusion (GO:0048284) | 44583 | 0.956396126 | 0.985325605 |
| positive regulation of translational initiation (GO:004594 | 44583 | 0.956396126 | 0.985325605 |
| regulation of cardiac muscle cell membrane repolarizatio | 44583 | 0.956396126 | 0.985325605 |
| acylglycerol catabolic process (GO:0046464) | 12816 | 0.956496059 | 0.985325605 |
| cholesterol biosynthetic process (GO:0006695) | 12816 | 0.956496059 | 0.985325605 |
| maturation of SSU-rRNA (GO:0030490) | 12816 | 0.956496059 | 0.985325605 |
| oxoacid metabolic process (GO:0043436) | 12816 | 0.956496059 | 0.985325605 |
| embryonic heart tube morphogenesis (GO:0003143) | 17227 | 0.958495182 | 0.986557785 |
| positive regulation of pathway-restricted SMAD protein p | 17227 | 0.958495182 | 0.986557785 |
| regulation of synapse assembly (GO:0051963) | 17227 | 0.958495182 | 0.986557785 |
| telomere organization (GO:0032200) | 17227 | 0.958495182 | 0.986557785 |
| DNA replication (GO:0006260) | 9/108 | 0.958641713 | 0.986557785 |
| double-strand break repair (GO:0006302) | 15/164 | 0.95897263 | 0.986702874 |
| neuron differentiation (GO:0030182) | 16/174 | 0.961159508 | 0.988187252 |
| axonal transport (GO:0098930) | 13181 | 0.961366385 | 0.988187252 |
| cyclic-nucleotide-mediated signaling (GO:0019935) | 13181 | 0.961366385 | 0.988187252 |
| positive regulation of telomere maintenance via telomer | 13181 | 0.961366385 | 0.988187252 |
| telomere maintenance via telomere lengthening (GO:001 | 13181 | 0.961366385 | 0.988187252 |
| cilium organization (GO:0044782) | 22/228 | 0.961759153 | 0.988395411 |
| drug catabolic process (GO:0042737) | 44584 | 0.962186276 | 0.988443287 |
| nucleotide-excision repair, DNA damage recognition (GO: | 44584 | 0.962186276 | 0.988443287 |
| fatty acid oxidation (GO:0019395) | 21641 | 0.962660207 | 0.988734633 |
| mitotic spindle organization (GO:0007052) | 14/157 | 0.964138003 | 0.990056716 |
| centromere complex assembly (GO:0034508) | 13547 | 0.965710657 | 0.990692508 |
| nucleotide-excision repair, DNA incision, 5'-to lesion (GO: | 13547 | 0.965710657 | 0.990692508 |
| positive regulation of cytokinesis (GO:0032467) | 13547 | 0.965710657 | 0.990692508 |
| protein insertion into ER membrane (GO:0045048) | 13547 | 0.965710657 | 0.990692508 |
| regulation of glucose import (GO:0046324) | 13547 | 0.965710657 | 0.990692508 |
| skeletal system development (GO:0001501) | 14/158 | 0.966352157 | 0.991054108 |
| protein glycosylation (GO:0006486) | 11/130 | 0.966507526 | 0.991054108 |
| DNA packaging (GO:0006323) | 44585 | 0.967207762 | 0.991054108 |
| ncRNA 3'-end processing (GO:0043628) | 44585 | 0.967207762 | 0.991054108 |
| peptidyl-glutamic acid modification (GO:0018200) | 44585 | 0.967207762 | 0.991054108 |
| sulfur amino acid metabolic process (GO:0000096) | 44585 | 0.967207762 | 0.991054108 |
| spliceosomal snRNP assembly (GO:0000387) | 13912 | 0.969582673 | 0.992713514 |
| chloride transmembrane transport (GO:1902476) | 18323 | 0.969843949 | 0.992713514 |
| epithelium development (GO:0060429) | 10/122 | 0.97002591 | 0.992713514 |

| | | | |
|---|---|---|---|
| cytoplasmic translation (GO:0002181) | 34151 | 0.971180376 | 0.992713514 |
| antigen processing and presentation of exogenous peptic | 8/103 | 0.97119871 | 0.992713514 |
| 3'-phosphoadenosine 5'-phosphosulfate metabolic proce | 44586 | 0.971562594 | 0.992713514 |
| cardiac conduction system development (GO:0003161) | 44586 | 0.971562594 | 0.992713514 |
| outflow tract septum morphogenesis (GO:0003148) | 44586 | 0.971562594 | 0.992713514 |
| peroxisome organization (GO:0007031) | 44586 | 0.971562594 | 0.992713514 |
| regulation of AMPA receptor activity (GO:2000311) | 44586 | 0.971562594 | 0.992713514 |
| regulation of cardiac muscle cell action potential (GO:009 | 44586 | 0.971562594 | 0.992713514 |
| double-strand break repair via nonhomologous end joinir | 22737 | 0.972192342 | 0.992713514 |
| transcription-coupled nucleotide-excision repair (GO:000 | 26785 | 0.972761476 | 0.992713514 |
| DNA duplex unwinding (GO:0032508) | 18688 | 0.972923839 | 0.992713514 |
| gonad development (GO:0008406) | 18688 | 0.972923839 | 0.992713514 |
| mitotic nuclear membrane organization (GO:0101024) | 18688 | 0.972923839 | 0.992713514 |
| mitotic nuclear membrane reassembly (GO:0007084) | 18688 | 0.972923839 | 0.992713514 |
| regulation of smoothened signaling pathway (GO:000858 | 18688 | 0.972923839 | 0.992713514 |
| DNA recombination (GO:0006310) | 14277 | 0.973031225 | 0.992713514 |
| DNA replication-independent nucleosome assembly (GO: | 14277 | 0.973031225 | 0.992713514 |
| nucleotide-excision repair, DNA incision (GO:0033683) | 14277 | 0.973031225 | 0.992713514 |
| regulation of heart rate by cardiac conduction (GO:00860 | 14277 | 0.973031225 | 0.992713514 |
| cotranslational protein targeting to membrane (GO:0006 | 34516 | 0.973480545 | 0.992976916 |
| regulation of cytokinesis (GO:0032465) | 30834 | 0.974015702 | 0.993312564 |
| neuropeptide signaling pathway (GO:0007218) | 23102 | 0.974826245 | 0.993312564 |
| DNA geometric change (GO:0032392) | 44587 | 0.975339247 | 0.993312564 |
| DNA modification (GO:0006304) | 44587 | 0.975339247 | 0.993312564 |
| maturation of LSU-rRNA (GO:0000470) | 44587 | 0.975339247 | 0.993312564 |
| positive regulation of glucose import (GO:0046326) | 44587 | 0.975339247 | 0.993312564 |
| retinoic acid metabolic process (GO:0042573) | 44587 | 0.975339247 | 0.993312564 |
| snRNA processing (GO:0016180) | 44587 | 0.975339247 | 0.993312564 |
| cilium movement (GO:0003341) | 19054 | 0.975703609 | 0.993488877 |
| detection of chemical stimulus involved in sensory percep | 14642 | 0.976100473 | 0.993698208 |
| tRNA processing (GO:0008033) | 23468 | 0.97722438 | 0.9945799 |
| regulation of cell cycle process (GO:0010564) | 8/106 | 0.977349449 | 0.9945799 |
| bicellular tight junction assembly (GO:0070830) | 19419 | 0.978210516 | 0.994698124 |
| calcium-ion regulated exocytosis (GO:0017156) | 44588 | 0.978614459 | 0.994698124 |
| dendritic spine organization (GO:0097061) | 44588 | 0.978614459 | 0.994698124 |
| melanosome organization (GO:0032438) | 44588 | 0.978614459 | 0.994698124 |
| mitotic chromosome condensation (GO:0007076) | 44588 | 0.978614459 | 0.994698124 |
| nucleobase-containing small molecule interconversion (G | 44588 | 0.978614459 | 0.994698124 |
| sensory perception of bitter taste (GO:0050913) | 15008 | 0.97883032 | 0.994722909 |
| double-strand break repair via homologous recombinatic | 35612 | 0.979406268 | 0.994947904 |
| chloride transport (GO:0006821) | 27881 | 0.979434761 | 0.994947904 |
| nuclear membrane reassembly (GO:0031468) | 19784 | 0.980469622 | 0.995637104 |
| antigen processing and presentation of exogenous peptic | 35977 | 0.98109167 | 0.995637104 |
| ATP-dependent chromatin remodeling (GO:0043044) | 15373 | 0.98125676 | 0.995637104 |
| cellular respiration (GO:0045333) | 15373 | 0.98125676 | 0.995637104 |
| cristae formation (GO:0042407) | 44589 | 0.981454785 | 0.995637104 |
| GPI anchor metabolic process (GO:0006505) | 44589 | 0.981454785 | 0.995637104 |

| | | | |
|---|---|---|---|
| response to ketone (GO:1901654) | 44589 | 0.981454785 | 0.995637104 |
| sensory perception of mechanical stimulus (GO:0050954) | 32295 | 0.981800814 | 0.99579368 |
| protein targeting to mitochondrion (GO:0006626) | 20149 | 0.982503928 | 0.996256221 |
| cilium assembly (GO:0060271) | 30/314 | 0.9826404 | 0.996256221 |
| protein N-linked glycosylation (GO:0006487) | 24563 | 0.983191202 | 0.996261011 |
| protein import (GO:0017038) | 32660 | 0.983373923 | 0.996261011 |
| DNA-dependent DNA replication maintenance of fidelity | 15738 | 0.983412216 | 0.996261011 |
| heart looping (GO:0001947) | 15738 | 0.983412216 | 0.996261011 |
| mismatch repair (GO:0006298) | 44590 | 0.983917951 | 0.996384748 |
| regulation of calcium ion transmembrane transporter act | 44590 | 0.983917951 | 0.996384748 |
| nervous system development (GO:0007399) | 45/447 | 0.984212687 | 0.996418118 |
| telomere maintenance (GO:0000723) | 20515 | 0.98433451 | 0.996418118 |
| recombinational repair (GO:0000725) | 28976 | 0.984552234 | 0.996444352 |
| cardiac ventricle morphogenesis (GO:0003208) | 16103 | 0.985325852 | 0.996504099 |
| mitotic spindle assembly (GO:0090307) | 16103 | 0.985325852 | 0.996504099 |
| CENP-A containing chromatin organization (GO:0061641) | 44591 | 0.986054023 | 0.996504099 |
| CENP-A containing nucleosome assembly (GO:0034080) | 44591 | 0.986054023 | 0.996504099 |
| estrogen metabolic process (GO:0008210) | 44591 | 0.986054023 | 0.996504099 |
| histone H3-K4 methylation (GO:0051568) | 44591 | 0.986054023 | 0.996504099 |
| non-motile cilium assembly (GO:1905515) | 44591 | 0.986054023 | 0.996504099 |
| sensory perception of sound (GO:0007605) | 33390 | 0.986145828 | 0.996504099 |
| peptide biosynthetic process (GO:0043043) | 13/162 | 0.986491135 | 0.996659169 |
| fatty acid catabolic process (GO:0009062) | 25659 | 0.987655146 | 0.99764116 |
| chondrocyte differentiation (GO:0002062) | 11689 | 0.989512806 | 0.998631205 |
| chromatin remodeling at centromere (GO:0031055) | 11689 | 0.989512806 | 0.998631205 |
| GPI anchor biosynthetic process (GO:0006506) | 11689 | 0.989512806 | 0.998631205 |
| protein transmembrane import into intracellular organell | 11689 | 0.989512806 | 0.998631205 |
| determination of heart left/right asymmetry (GO:006137 | 17199 | 0.9898646 | 0.998631205 |
| apical junction assembly (GO:0043297) | 21976 | 0.989980887 | 0.998631205 |
| homophilic cell adhesion via plasma membrane adhesion | 21976 | 0.989980887 | 0.998631205 |
| DNA repair (GO:0006281) | 27/298 | 0.990375997 | 0.998835819 |
| exonucleolytic catabolism of deadenylated mRNA (GO:00 | 12055 | 0.990905839 | 0.999124875 |
| positive regulation of DNA repair (GO:0045739) | 17564 | 0.991047253 | 0.999124875 |
| axoneme assembly (GO:0035082) | 12420 | 0.992113846 | 0.99981211 |
| protein localization to chromosome (GO:0034502) | 12420 | 0.992113846 | 0.99981211 |
| brain development (GO:0007420) | 11/150 | 0.992461046 | 0.999968024 |
| base-excision repair, gap-filling (GO:0006287) | 12785 | 0.993161394 | 0.999993541 |
| nuclear-transcribed mRNA catabolic process, exonucleoly | 12785 | 0.993161394 | 0.999993541 |
| protein localization to cilium (GO:0061512) | 12785 | 0.993161394 | 0.999993541 |
| import into nucleus (GO:0051170) | 28216 | 0.994093592 | 0.999993541 |
| translation (GO:0006412) | 17/214 | 0.994709089 | 0.999993541 |
| mitochondrial respiratory chain complex assembly (GO:0 | 32994 | 0.994794772 | 0.999993541 |
| SRP-dependent cotranslational protein targeting to mem | 32994 | 0.994794772 | 0.999993541 |
| anterograde axonal transport (GO:0008089) | 13516 | 0.994857513 | 0.999993541 |
| histone exchange (GO:0043486) | 13881 | 0.995540586 | 0.999993541 |
| odontogenesis (GO:0042476) | 14246 | 0.996132907 | 0.999993541 |
| replication fork processing (GO:0031297) | 14246 | 0.996132907 | 0.999993541 |

| | | | |
|---|---|---|---|
| synapse assembly (GO:0007416) | 25263 | 0.996425339 | 0.999993541 |
| glycolipid biosynthetic process (GO:0009247) | 14611 | 0.99664653 | 0.999993541 |
| intraciliary transport involved in cilium assembly (GO:003 | 14611 | 0.99664653 | 0.999993541 |
| DNA-dependent DNA replication (GO:0006261) | 8/129 | 0.996887486 | 0.999993541 |
| generation of neurons (GO:0048699) | 15/202 | 0.996967891 | 0.999993541 |
| spindle assembly (GO:0051225) | 30773 | 0.997231848 | 0.999993541 |
| regulation of neurotransmitter receptor activity (GO:009 | 21217 | 0.997453905 | 0.999993541 |
| mitochondrial gene expression (GO:0140053) | 15342 | 0.997478102 | 0.999993541 |
| endoplasmic reticulum to Golgi vesicle-mediated transpo | 13/185 | 0.99764184 | 0.999993541 |
| regulation of cation channel activity (GO:2001257) | 32599 | 0.9984062 | 0.999993541 |
| synapse organization (GO:0050808) | 7/126 | 0.998560586 | 0.999993541 |
| base-excision repair (GO:0006284) | 17533 | 0.998926876 | 0.999993541 |
| DNA metabolic process (GO:0006259) | 21/277 | 0.999023591 | 0.999993541 |
| inner mitochondrial membrane organization (GO:000700 | 17899 | 0.999069191 | 0.999993541 |
| fatty acid beta-oxidation (GO:0006635) | 18994 | 0.999392339 | 0.999993541 |
| translational elongation (GO:0006414) | 4/104 | 0.999707547 | 0.999993541 |
| mitochondrial respiratory chain complex I assembly (GO: | 21186 | 0.999740219 | 0.999993541 |
| NADH dehydrogenase complex assembly (GO:0010257) | 21186 | 0.999740219 | 0.999993541 |
| anterograde trans-synaptic signaling (GO:0098916) | 15/244 | 0.999896446 | 0.999993541 |
| retrograde vesicle-mediated transport, Golgi to endoplas | 31079 | 0.999920333 | 0.999993541 |
| aerobic electron transport chain (GO:0019646) | 25569 | 0.9999506 | 0.999993541 |
| mitochondrial translational elongation (GO:0070125) | 32540 | 0.999951628 | 0.999993541 |
| mitochondrial ATP synthesis coupled electron transport ( | 25934 | 0.999956746 | 0.999993541 |
| translational termination (GO:0006415) | 35096 | 0.999978765 | 0.999993541 |
| sensory perception of chemical stimulus (GO:0007606) | 36192 | 0.999984578 | 0.999993541 |
| chemical synaptic transmission (GO:0007268) | 18/306 | 0.999987317 | 0.999993541 |
| mitochondrial translation (GO:0032543) | 2/105 | 0.999991068 | 0.999993541 |
| mitochondrial translational termination (GO:0070126) | 32509 | 0.999993541 | 0.999993541 |

**gnificant highly-correlated RNA-seq-based modules (turquoise; Significant tern**

| Old P-value | Old Adjusted P-value | Odds Ratio | Combined Score | Genes | KEGG |
|---|---|---|---|---|---|
| 0 | 0 | 3.792617643 | 393.7961679 | IFITM3;IL1 | |
| 0 | 0 | 3.994324532 | 359.1120712 | RIPOR2;CS | |
| 0 | 0 | 4.088642659 | 272.8887854 | FCN1;IL23I | |
| 0 | 0 | 4.648627681 | 266.0853869 | IL1RN;CD4 | |
| 0 | 0 | 5.849254743 | 305.555873 | IFITM3;CSI | |
| 0 | 0 | 5.534342476 | 284.7111431 | CXCL8;CD8 | |
| 0 | 0 | 2.793930239 | 143.6253436 | CSF3;NCF1 | |
| 0 | 0 | 2.866140304 | 140.5712103 | FCN1;RAB3 | |
| 0 | 0 | 2.807684292 | 130.1022442 | FCN1;RAB3 | |
| 0 | 0 | 2.774384119 | 126.2438106 | FCN1;RAB3 | |
| 0 | 0 | 9.509974457 | 420.1752391 | CXCL8;IL23 | |
| 0 | 0 | 8.297066718 | 356.1670308 | FCN1;C5AI | |
| 0 | 0 | 4.074884709 | 172.202669 | TNFAIP6;SI | |
| 0 | 0 | 3.788850341 | 153.6903072 | CD40;TRAI | |
| 0 | 0 | 5.440169296 | 210.1388236 | CXCL8;CD8 | |
| 0 | 0 | 4.072529384 | 153.288873 | IL23R;TNF/ | |
| 0 | 0 | 3.341695838 | 118.6269927 | CD40;CRTC | |
| 0 | 0 | 8.906314274 | 312.6007125 | FCN1;SERP | |
| 0 | 0 | 5.332363829 | 185.0102778 | TNFAIP3;P | |
| 0 | 0 | 5.107124915 | 174.4546693 | CXCL8;CD8 | |
| 0 | 0 | 4.087192399 | 133.3583609 | CD40;IL1R/ | |
| 0 | 0 | 3.784292133 | 119.4332716 | CD40;TRAI | |
| 0 | 0 | 2.83819448 | 88.29932046 | IFITM3;FCI | |
| 0 | 0 | 6.616972477 | 205.7457209 | RNF31;RN/ | |
| 0 | 0 | 4.483651919 | 136.9034726 | IFITM3;RTI | |
| 0 | 0 | 4.483651919 | 136.9034726 | TNFAIP3;P | |
| 0 | 0 | 8.614762386 | 256.7272305 | PTPRJ;PLA | |
| 0 | 0 | 7.035557744 | 204.2476701 | RNASEL;TN | |
| 0 | 0 | 4.126820832 | 118.6956021 | IFITM3;RTI | |
| 0 | 0 | 3.966290596 | 111.6518866 | SERPINE1;I | |
| 0 | 0 | 5.16130834 | 140.9168011 | ILRUN;UBA | |
| 0 | 0 | 5.784425134 | 155.6593984 | CD274;CD8 | |
| 0 | 0 | 4.761357836 | 127.4271181 | SERPINE1;I | |
| 0 | 0 | 4.320652174 | 115.6040485 | RARG;IL23 | |
| 0 | 0 | 7.074369588 | 188.4342842 | DYSF;PLA2 | |
| 0 | 0 | 5.319580176 | 141.6071227 | SEMA7A;X | |
| 0 | 0 | 4.962222647 | 131.2369056 | ITGAM;UB | |
| 0 | 0 | 5.643015521 | 149.1381495 | NOD2;TNF | |
| 0 | 0 | 2.03159473 | 53.07117817 | CSF3;CSF1; | |
| 0 | 0 | 4.928189555 | 126.5108449 | CD81;SERP | |
| 0 | 0 | 4.993025651 | 126.3899519 | CD274;IL23 | |
| 0 | 0 | 1.700711343 | 42.46611478 | ZNF296;M/ | |
| 0 | 0 | 2.930926141 | 72.84464771 | RET;CXCL8 | |
| 0 | 0 | 7.421315689 | 184.3224556 | CD40;IL23I | |

| | | | | |
|---|---|---|---|---|
| 0 | 0 | 3.742016484 | 92.10063244 | ACVRL1;RE |
| 0 | 0 | 5.224220144 | 126.7652076 | DDX3X;CG/ |
| 0 | 0 | 1.869476462 | 44.95478939 | CSF3;CSF1; |
| 0 | 0 | 3.045965608 | 73.19832625 | CD40;CXCL |
| 0 | 0 | 11.97113559 | 283.5005197 | CD40;TICA |
| 0 | 0 | 3.414809121 | 80.26408156 | TNF;PIK3C( |
| 0 | 0 | 4.529398856 | 105.5566126 | FMR1;PLA2 |
| 0 | 0 | 3.578038302 | 82.28751979 | NRP1;CSF3 |
| 0 | 0 | 3.782511088 | 86.93312188 | CCL14;CIIT |
| 0 | 0 | 5.657208213 | 129.7983209 | IFITM3;RN/ |
| 0 | 0 | 5.657208213 | 129.7983209 | IFITM3;RN/ |
| 0 | 0 | 4.446788991 | 101.9479932 | NRP1;CSF3 |
| 0 | 0 | 2.379422563 | 52.95891615 | RET;CXCL8 |
| 0 | 0 | 1.748642565 | 38.77636765 | ZNF296;CR |
| 0 | 0 | 4.53392052 | 100.4818787 | CCL14;HD/ |
| 0 | 0 | 3.249693283 | 72.011883 | IL1RN;CD4 |
| 0 | 0 | 11.3682612 | 251.1278018 | CD40;TICA |
| 0 | 0 | 1.698907186 | 37.51728929 | TP53RK;UE |
| 0 | 0 | 6.866359082 | 151.4786705 | DDX3X;REL |
| 0 | 0 | 6.140005778 | 135.3302154 | NLRC4;NOI |
| 0 | 0 | 2.885527926 | 63.49107078 | DGKG;DGK |
| 0 | 0 | 5.934996192 | 127.8026677 | IL23R;CEBF |
| 0 | 0 | 4.636186454 | 99.24587846 | IFITM3;CCI |
| 0 | 0 | 2.23052074 | 47.73515741 | ACVRL1;BC |
| 0 | 0 | 9.404264554 | 200.1513251 | CD40;RIPK |
| 0 | 0 | 3.778251599 | 79.49808349 | CCL14;CD4 |
| 0 | 0 | 4.694255797 | 98.73357182 | LY96;PTPR. |
| 0 | 0 | 5.431529412 | 113.4763211 | NLRC4;NOI |
| 0 | 0 | 23.00132626 | 478.6579551 | TICAM2;M |
| 0 | 0 | 28.46868687 | 579.3552668 | TICAM2;M |
| 0 | 0 | 4.357259542 | 88.31401028 | TNFAIP3;N |
| 0 | 0 | 4.649408848 | 94.21464668 | CD274;CD4 |
| 0 | 0 | 1.786933399 | 36.05824039 | FCMR;STE/ |
| 0 | 0 | 4.904745153 | 97.75185156 | CD40;IL23F |
| 0 | 0 | 4.400101742 | 87.47725706 | NFAT5;DD) |
| 0 | 0 | 5.235551723 | 102.8906235 | FCN1;LY96 |
| 0 | 0 | 4.310054583 | 84.10391307 | LY96;PTPR. |
| 0 | 0 | 5.085670765 | 97.72232857 | RNF31;RO( |
| 0 | 0 | 3.268507509 | 62.49039516 | SEMA7A;C |
| 0 | 0 | 4.397509213 | 82.38959256 | ITGAM;CD8 |
| 0 | 0 | 15.33244916 | 284.1606597 | ASAH1;ML |
| 0 | 0 | 4.6152 | 84.69290215 | CIITA;PTAF |
| 0 | 0 | 12.32396702 | 225.3700004 | IFNGR1;ST/ |
| 0 | 0 | 5.451554938 | 99.14798538 | CD74;EGR1 |
| 0 | 0 | 1.923817017 | 34.98275873 | CCNT1;TP5 |
| 0 | 0 | 7.353387824 | 131.1131964 | DDX3X;DD |
| 0 | 0 | 6.283244681 | 111.5624178 | ILRUN;IL10 |

| | | | | |
|---|---|---|---|---|
| 0 | 0 | 7.89313093 | 139.2935848 | IL15;IL23R;… |
| 0 | 0 | 4.844043782 | 83.47806333 | CD40;IL23R… |
| 0 | 0 | 5.570593223 | 95.05988349 | CD274;IL23… |
| 0 | 0 | 7.984527423 | 134.5140591 | IFNAR2;STA… |
| 0 | 0 | 24.07065355 | 402.6662415 | SOCS3;SOC… |
| 0 | 0 | 1.987337536 | 33.11168883 | RET;DGKG;… |
| 0 | 0 | 9.858036391 | 164.1648506 | IFNAR2;STA… |
| 0 | 0 | 5.981769844 | 98.33083889 | CD74;EGR1… |
| 0 | 0 | 2.243960084 | 36.83958575 | RET;NRP1;… |
| 0 | 0 | 1.924370631 | 31.43472635 | FAM13B;TI… |
| 0 | 0 | 4.137586567 | 66.68083248 | IFITM3;RN… |
| 0 | 0 | 1.872072779 | 30.10236393 | CSF3;CSF1;… |
| 0 | 0 | 2.84145977 | 45.41784302 | HDAC4;GFI… |
| 0 | 0 | 12.19805195 | 194.4594389 | LYN;PRKCE… |
| 0 | 0 | 10.21986443 | 162.2660984 | ZBP1;MLKL… |
| 0 | 0 | 3.401353769 | 53.71318128 | TENM1;CD… |
| 0 | 0 | 2.324206196 | 36.44904116 | CSRNP1;FA… |
| 0 | 0 | 18.05194928 | 280.446109 | STAT1;OAS… |
| 0 | 0 | 3.844222222 | 59.6495006 | CD274;SDC… |
| 0 | 0 | 2.551435103 | 39.10500437 | IL23R;SERP… |
| 0 | 0 | 1.946393003 | 29.46841562 | CXCL8;DDX… |
| 0 | 0 | 21.87413293 | 326.8647358 | STAT5A;IL1… |
| 0 | 0 | 21.87413293 | 326.8647358 | STAT5A;IL1… |
| 0 | 0 | 4.93512538 | 73.73802363 | LGALS3BP;… |
| 0 | 0 | 4.670761761 | 69.62036134 | NFAT5;DDX… |
| 0 | 0 | 2.492121588 | 36.80602039 | CD40;CD81… |
| 0 | 0 | 4.015387757 | 58.73241418 | CD274;CD8… |
| 0 | 0 | 6.208754847 | 90.24681705 | DDX3X;DD… |
| 0 | 0 | 6.208754847 | 90.24681705 | NOD2;CYR… |
| 0 | 0 | 2.969105846 | 43.00715588 | CD40;TNF;… |
| 0 | 0 | 29.52061271 | 426.2249839 | STAT1;CAN… |
| 0 | 0 | 29.52061271 | 426.2249839 | FCER2;IFN… |
| 0 | 0 | 1.919894515 | 27.59832778 | ACVRL1;BT… |
| 0 | 0 | 3.919556788 | 56.02519478 | NRP1;FLT1… |
| 0 | 0 | 1.793855867 | 25.63625228 | FCMR;ZFA… |
| 0 | 0 | 6.572241183 | 93.71646441 | IL10;XBP1;… |
| 0 | 0 | 4.205249261 | 59.58853151 | TRAF2;TNF… |
| 0 | 0 | 121429 | 1717887.119 | CYP27B1;I… |
| 0 | 0 | 52.46427221 | 739.6139063 | STAT5A;ST… |
| 0 | 0 | 52.46427221 | 739.6139063 | STAT5A;ST… |
| 0 | 0 | 2.102178793 | 29.62773488 | RET;BCAR3… |
| 0 | 0 | 3.722430943 | 52.35692962 | DDX3X;BCL… |
| 0 | 0 | 5.881638847 | 82.50899042 | OTUD4;LYN… |
| 0 | 0 | 5.881638847 | 82.50899042 | CCR1;CD74… |
| 0 | 0 | 7.039829958 | 98.63541247 | ERAP1;XIA… |
| 0 | 0 | 1.785299882 | 24.98389128 | CSF1;FMR1… |
| 0 | 0 | 2.028574815 | 28.27163519 | RET;LRRK2… |

| | | | | |
|---|---|---|---|---|
| 0 | 0 | 3.94986687 | 54.85101476 | TCF7L2;SR( |
| 0 | 0 | 16.4046538 | 226.6762593 | FCER2;EDN |
| 0 | 0 | 2.861291555 | 39.48181266 | PRR7;DDX3 |
| 0 | 0 | 8.537159091 | 116.4482374 | IL15;IL23R; |
| 0 | 0 | 9.847974252 | 133.4972357 | IFIH1;DDX3 |
| 0 | 0 | 9.847974252 | 133.4972357 | ITGAM;TIC |
| 0 | 0 | 1.525537536 | 20.44649407 | NAB1;GFI1 |
| 0 | 0 | 2.276740225 | 30.39839287 | CD40;BTG1 |
| 0 | 0 | 1.624597062 | 21.66481417 | GFI1;WWC |
| 0 | 0 | 3.419686273 | 45.48229044 | GFI1;NLRC |
| 0 | 0 | 3.761343259 | 49.80504947 | EGR1;PRR7 |
| 0 | 0 | 5.841317996 | 77.23975642 | MAP2K3;N |
| 0 | 0 | 7.073658437 | 93.36749658 | TASL;DDX3 |
| 0 | 0 | 19.67927383 | 259.3390014 | IL1R1;IL23A |
| 0 | 0 | 19.67927383 | 259.3390014 | CLEC7A;IL2 |
| 0 | 0 | 3.881095837 | 51.03692374 | MAP2K3;R |
| 0 | 0 | 5.32086856 | 69.61588885 | USP15;STA |
| 0 | 0 | 4.186170213 | 54.49173974 | IFITM3;RN |
| 0 | 0 | 3.088603222 | 40.05833405 | IL1RN;PELI |
| 0 | 0 | 6.159140447 | 79.56608782 | IL10;GHSR; |
| 0 | 0 | 6.159140447 | 79.56608782 | NFAT5;CYF |
| 0 | 0 | 3.235555556 | 41.74658565 | ILRUN;TNF |
| 0 | 0 | 1.477438616 | 18.97922884 | JMJD1C;RE |
| 0 | 0 | 1.84768365 | 23.72983118 | FCMR;TNF |
| 0 | 0 | 8.753250032 | 112.3711161 | IL6;RSAD2; |
| 0 | 0 | 2.203589744 | 28.18145515 | TNFAIP6;TI |
| 0 | 0 | 4.73314611 | 59.6203676 | GBP5;NOS |
| 0 | 0 | 4.461723072 | 56.17755644 | EGR1;DAX |
| 0 | 0 | 2.175962733 | 27.17837075 | PTPRO;WV |
| 0 | 0 | 1.798333345 | 22.43518411 | RET;ACVRL |
| 0 | 0 | 5.79650359 | 71.97317188 | CD274;STA |
| 0 | 0 | 7.113478535 | 88.10456194 | IL10;DDX3 |
| 0 | 0 | 1.659124646 | 20.52177413 | RIPOR2;AD |
| 0 | 0 | 2.522447005 | 31.14495575 | AMER1;PFI |
| 0 | 0 | 2.522447005 | 31.14495575 | ARHGAP9; |
| 0 | 0 | 3.509978446 | 43.29780294 | CCR1;NRP1 |
| 0 | 0 | 2.733355684 | 33.71677301 | DDX3X;SR( |
| 0 | 0 | 1.907762883 | 23.46688352 | ACVRL1;BT |
| 0 | 0 | 5.256579446 | 64.58666779 | ZBP1;CD74 |
| 0 | 0 | 2.30117632 | 28.17858821 | ILRUN;RAN |
| 0 | 0 | 4.307621816 | 52.63638125 | LYN;PTPN1 |
| 0 | 0 | 4.307621816 | 52.63638125 | ILRUN;IL10 |
| 0 | 0 | 4.307621816 | 52.63638125 | ANXA1;CD |
| 0 | 0 | 2.876827709 | 35.10111636 | PTGFR;PRK |
| 0 | 0 | 45.88888889 | 559.4253085 | STAT1;STA |
| 0 | 0 | 45.88888889 | 559.4253085 | STAT1;STA |
| 0 | 0 | 45.88888889 | 559.4253085 | TICAM2;IR |

| | | | | |
|---|---|---|---|---|
| 0 | 0 | 14.75860439 | 178.9599366 | CLEC7A;IL2 |
| 0 | 0 | 14.75860439 | 178.9599366 | RIPK2;ERBI |
| 0 | 0 | 104082 | 1262017.68 | CD14;LBP;I |
| 0 | 0 | 104082 | 1262017.68 | CD14;LBP;I |
| 0 | 0 | 9.023892884 | 108.6033112 | PYCARD;SE |
| 0 | 0 | 2.393967936 | 28.39864942 | CDKN1A;D |
| 0 | 0 | 4.163793976 | 49.38648895 | TCF7L2;ED |
| 0 | 0 | 2.93842636 | 34.78005615 | RGS14;PTP |
| 0 | 0 | 6.565909091 | 77.62242997 | ANXA1;CD |
| 0 | 0 | 2.021576296 | 23.89039973 | NRP1;MAN |
| 0 | 0 | 1.946313301 | 22.98934825 | RARG;PRR |
| 0 | 0 | 1.895343833 | 22.15452089 | RARG;PRR |
| 0 | 0 | 5.746352785 | 66.70960511 | LYN;SRC;EF |
| 0 | 0 | 4.225291407 | 48.41598289 | ILRUN;IL10 |
| 0 | 0 | 17.48607435 | 200.0109323 | STAT5A;ST |
| 0 | 0 | 17.48607435 | 200.0109323 | STAT5A;ST |
| 0 | 0 | 17.48607435 | 200.0109323 | CASP8;RIPI |
| 0 | 0 | 4.778156997 | 54.64331046 | IL10;XBP1; |
| 0 | 0 | 6.096563853 | 68.85915997 | OTUD4;GF |
| 0 | 0 | 9.372466353 | 105.2796455 | GBP5;IL6;C |
| 0 | 0 | 11.80620272 | 132.4912678 | GBP6;BATF |
| 0 | 0 | 3.54091653 | 39.24315735 | CCL14;CCR |
| 0 | 0 | 4.926175133 | 54.48753127 | DUSP5;PTF |
| 0 | 0 | 2.391261168 | 25.94570365 | PPP1R15A; |
| 0 | 0 | 22.94312169 | 248.0055853 | IL23A;IL23I |
| 0 | 0 | 22.94312169 | 248.0055853 | NFKBIA;IL6 |
| 0 | 0 | 5.68979798 | 61.4496381 | SELPLG;SR |
| 0 | 0 | 1.925385373 | 20.75894169 | CD40;FMR |
| 0 | 0 | 3.943149905 | 42.46430778 | IL10;SERPI |
| 0 | 0 | 1.985596318 | 21.29747468 | NRP1;FLT4 |
| 0 | 0 | 4.691324597 | 49.97313119 | ITGAM;IFN |
| 0 | 0 | 2.122805465 | 22.44006512 | ARHGAP9; |
| 0 | 0 | 3.151750634 | 33.26729584 | BATF2;CDK |
| 0 | 0 | 8.200435112 | 86.50536721 | IL10;ANXA |
| 0 | 0 | 3.67308723 | 38.73726847 | CD74;CD4 |
| 0 | 0 | 3.367799689 | 35.40302719 | DDX3X;RIP |
| 0 | 0 | 13.11379962 | 137.2244587 | PARP10;PA |
| 0 | 0 | 3.815731162 | 39.84719616 | MAP3K2;C |
| 0 | 0 | 2.937289356 | 30.59088823 | FASLG;NOI |
| 0 | 0 | 3.215034419 | 33.41753421 | IFITM3;RN |
| 0 | 0 | 3.459931637 | 35.89081745 | RNF31;SPP |
| 0 | 0 | 3.989039129 | 41.3164326 | C1R;RIPK2; |
| 0 | 0 | 2.50911139 | 25.9584746 | CD40;RAM |
| 0 | 0 | 5.333877841 | 55.12304381 | MAP3K1;L |
| 0 | 0 | 39.31847374 | 404.9702418 | TICAM2;SA |
| 0 | 0 | 39.31847374 | 404.9702418 | IL23A;IL23I |
| 0 | 0 | 39.31847374 | 404.9702418 | OTUD4;ITC |

| | | | | |
|---|---|---|---|---|
| 0 | 0 | 4.477824109 | 45.95452013 | NLGN3;IL1 |
| 0 | 0 | 2.012569924 | 20.51254832 | CD40;DDX3 |
| 0 | 0 | 6.561695685 | 66.83241063 | DCSTAMP; |
| 0 | 0 | 2.615195874 | 26.45665121 | ERO1A;PPF |
| 0 | 0 | 86735 | 876329.3346 | JAML;PECA |
| 0 | 0 | 2.415101781 | 24.17405648 | ACVRL1;NF |
| 0 | 0 | 2.923460547 | 29.23410548 | HEXIM1;LY |
| 0 | 0 | 2.759249481 | 27.40664608 | RYR1;SLC2 |
| 0 | 0 | 2.632685714 | 26.10186411 | BCAR3;TEN |
| 0 | 0 | 3.07786862 | 30.42323038 | TENM1;CD |
| 0 | 0 | 2.050492956 | 20.25584378 | ACVRL1;PR |
| 0 | 0 | 2.050492956 | 20.25584378 | USP36;RAF |
| 0 | 0 | 2.965839111 | 29.11907693 | LYN;FCER1 |
| 0 | 0 | 8.432029393 | 82.0487652 | CCR1;CXCF |
| 0 | 0 | 15.29453263 | 148.7873055 | TICAM2;NI |
| 0 | 0 | 15.29453263 | 148.7873055 | TBX21;NFK |
| 0 | 0 | 15.29453263 | 148.7873055 | CLEC7A;IL2 |
| 0 | 0 | 2.715294772 | 26.39396577 | DDX3X;SRC |
| 0 | 0 | 2.715294772 | 26.39396577 | CXCL8;DGK |
| 0 | 0 | 2.651782974 | 25.7575117 | FLT1;WNT |
| 0 | 0 | 3.722673242 | 36.14464948 | LAPTM5;IL |
| 0 | 0 | 6.014540752 | 58.11406517 | LYN;TICAM |
| 0 | 0 | 10.49043478 | 101.0525498 | LFNG;DOCI |
| 0 | 0 | 2.908635566 | 27.87819984 | NRP1;SEM |
| 0 | 0 | 3.478446255 | 33.15177873 | KMT2E;LYN |
| 0 | 0 | 3.478446255 | 33.15177873 | URI1;DDX3 |
| 0 | 0 | 5.250132526 | 50.03278464 | IFIH1;CYLD |
| 0 | 0 | 5.250132526 | 50.03278464 | C3;IL1A;IL6 |
| 0 | 0 | 2.159966052 | 20.55566599 | ACVRL1;NF |
| 0 | 0 | 4.376910446 | 41.5300818 | ANXA1;RIP |
| 0 | 0 | 2.437977436 | 22.86569349 | MALT1;PSI |
| 0 | 0 | 6.559591373 | 61.41011804 | LYN;STAT5 |
| 0 | 0 | 6.559591373 | 61.41011804 | ABCA1;NFK |
| 0 | 0 | 1.929397704 | 17.97667965 | RET;FLT1;C |
| 0 | 0 | 1.572695612 | 14.62100224 | NCF1;CD80 |
| 0 | 0 | 3.939802881 | 36.22822846 | EDN1;SMA |
| 0 | 0 | 5.551563501 | 50.94456811 | IL10;SLC7A |
| 0 | 0 | 2.892694793 | 26.52323467 | LYN;FCER1 |
| 0 | 0 | 2.489801802 | 22.76273329 | CD40;RAM |
| 0 | 0 | 2.800584425 | 25.58229203 | CCL14;EDN |
| 0 | 0 | 2.591422408 | 23.60954806 | SDC4;VPS4 |
| 0 | 0 | 3.489603092 | 31.78264231 | ANXA1;RIP |
| 0 | 0 | 7.377600227 | 67.15267245 | CD274;IL23 |
| 0 | 0 | 4.921715259 | 44.7797986 | TRAF3IP2;I |
| 0 | 0 | 4.921715259 | 44.7797986 | IL10;IL6;TH |
| 0 | 0 | 2.345581595 | 21.26856451 | DDX3X;SRC |
| 0 | 0 | 3.056730761 | 27.64795875 | JUN;ANXA1 |

| | | | | |
|---|---|---|---|---|
| 0 | 0 | 19.65810351 | 177.5824918 | SEMA4D;IL |
| 0 | 0 | 19.65810351 | 177.5824918 | C3;C4A;CC |
| 0 | 0 | 19.65810351 | 177.5824918 | PRR7;CASF |
| 0 | 0 | 19.65810351 | 177.5824918 | C3;C4A;CC |
| 0 | 0 | 19.65810351 | 177.5824918 | SPN;IL6;ST |
| 0 | 0 | 2.263370706 | 20.37224462 | BCAR3;CIIT |
| 0 | 0 | 3.623344187 | 32.54115353 | CREBBP;M |
| 0 | 0 | 3.284819734 | 29.47350731 | CPB2;C1S;( |
| 0 | 0 | 2.835811329 | 25.35986811 | CCL14;EDN |
| 0 | 0 | 8.74152489 | 77.88269094 | LYN;STAT5 |
| 0 | 0 | 8.74152489 | 77.88269094 | DDX58;IL2 |
| 0 | 0 | 8.74152489 | 77.88269094 | ANXA1;CD |
| 0 | 0 | 8.74152489 | 77.88269094 | CD74;IL6;R |
| 0 | 0 | 5.962920923 | 52.72220503 | C3;PYCARD |
| 0 | 0 | 11.4702381 | 101.382982 | CXCL8;HSP |
| 0 | 0 | 11.4702381 | 101.382982 | CXCL10;CX |
| 0 | 0 | 3.383662114 | 29.85422234 | LYN;PTPN1 |
| 0 | 0 | 3.383662114 | 29.85422234 | CPB2;C1S;( |
| 0 | 0 | 1.869324878 | 16.49085416 | USP36;LRR |
| 0 | 0 | 2.987086973 | 26.29233088 | CDKN2D;U |
| 0 | 0 | 2.265112635 | 19.90299077 | CFD;LGALS |
| 0 | 0 | 1.907101727 | 16.75370863 | CCL14;BCA |
| 0 | 0 | 4.266117424 | 37.1861526 | PDIA3;SEC |
| 0 | 0 | 3.195856198 | 27.82730996 | CDKN2D;D |
| 0 | 0 | 4.631935319 | 40.26265328 | RET;PYCAR |
| 0 | 0 | 4.631935319 | 40.26265328 | ART1;PARF |
| 0 | 0 | 3.502363797 | 30.41393346 | IL10;SERPI |
| 0 | 0 | 3.044743226 | 26.25764552 | PAQR8;CD |
| 0 | 0 | 3.647544436 | 31.21511654 | DDX3X;SEF |
| 0 | 0 | 6.557488654 | 55.96350071 | CCR1;CXCF |
| 0 | 0 | 6.557488654 | 55.96350071 | SOCS3;IL6; |
| 0 | 0 | 6.557488654 | 55.96350071 | PYCARD;RI |
| 0 | 0 | 6.557488654 | 55.96350071 | CXCL10;CX |
| 0 | 0 | 2.816652193 | 24.02119737 | CCL14;EDN |
| 0 | 0 | 2.133962171 | 18.17068435 | NLGN3;RAI |
| 0 | 0 | 2.098004538 | 17.71247351 | BCAR3;CCL |
| 0 | 0 | 32.75302115 | 276.0651384 | CD300A;IL |
| 0 | 0 | 32.75302115 | 276.0651384 | IFNG;GFI1; |
| 0 | 0 | 32.75302115 | 276.0651384 | IL6;IRAK3;I |
| 0 | 0 | 32.75302115 | 276.0651384 | IL6;STAT3; |
| 0 | 0 | 32.75302115 | 276.0651384 | TAP2;TAP1 |
| 0 | 0 | 2.36157265 | 19.90384976 | TENM1;CD |
| 0 | 0 | 2.399966036 | 20.13355153 | ERO1A;PRI |
| 0 | 0 | 4.062734488 | 33.97833084 | DDX3X;SEF |
| 0 | 0 | 4.062734488 | 33.97833084 | CD274;RIP |
| 0 | 0 | 5.465695548 | 45.70293032 | C3;NFKBIA |
| 0 | 0 | 1.79039097 | 14.93151544 | RET;NRP1; |

| | | | | |
|---|---|---|---|---|
| 0 | 0 | 2.760159574 | 22.93551093 | LYN;SRC;PI… |
| 0 | 0 | 2.298780488 | 19.09363013 | MALT1;PSI… |
| 0 | 0 | 7.492303538 | 61.96965342 | IL6;IL1B;OS… |
| 0 | 0 | 7.492303538 | 61.96965342 | CCR1;CSF1… |
| 0 | 0 | 3.031615823 | 24.86520377 | XBP1;RIPK… |
| 0 | 0 | 2.243137629 | 18.38918815 | ILRUN;TNF… |
| 0 | 0 | 1.93524438 | 15.75370175 | RNF31;PTP… |
| 0 | 0 | 3.675755968 | 29.89692099 | FCN1;IFITN… |
| 0 | 0 | 3.675755968 | 29.89692099 | STAT5A;ST… |
| 0 | 0 | 2.628962372 | 21.27639119 | LYN;CSF3;F… |
| 0 | 0 | 2.628962372 | 21.27639119 | AMER1;HD… |
| 0 | 0 | 13.10464677 | 105.5540031 | RIPK2;NOD… |
| 0 | 0 | 13.10464677 | 105.5540031 | SLC2A10;A… |
| 0 | 0 | 13.10464677 | 105.5540031 | CASP8;PEL… |
| 0 | 0 | 13.10464677 | 105.5540031 | DDX3X;MA… |
| 0 | 0 | 13.10464677 | 105.5540031 | IL23A;IL23… |
| 0 | 0 | 1.943239283 | 15.65108484 | BCAR3;CXC… |
| 0 | 0 | 3.101152841 | 24.8917984 | CAST;CDKN… |
| 0 | 0 | 3.101152841 | 24.8917984 | CPB2;C1S;C… |
| 0 | 0 | 5.901399395 | 47.31401819 | STAT5A;ST… |
| 0 | 0 | 5.901399395 | 47.31401819 | STAT5A;ST… |
| 0 | 0 | 5.901399395 | 47.31401819 | SELP;SPN;S… |
| 0 | 0 | 2.417265422 | 19.25019703 | BCAR3;TEN… |
| 0 | 0 | 4.143884892 | 32.93452325 | SOCS3;SOC… |
| 0 | 0 | 2.108693397 | 16.74711481 | RAMP2;SR… |
| 0 | 0 | 2.519651458 | 19.92736962 | GALNT11;N… |
| 0 | 0 | 4.509864686 | 35.65012314 | EDN1;SMA… |
| 0 | 0 | 2.025807589 | 15.99206936 | MTMR3;PF… |
| 0 | 0 | 2.653692717 | 20.93482814 | GSDMD;NC… |
| 0 | 0 | 1.996932496 | 15.6632293 | SMG1;FLT1… |
| 0 | 0 | 3.534176699 | 27.6788361 | RIOK3;STAT… |
| 0 | 0 | 2.218204942 | 17.35820346 | RYR1;SLC2… |
| 0 | 0 | 2.038947518 | 15.93465628 | RYR1;PTGF… |
| 0 | 0 | 2.088296389 | 16.28909201 | RAMP2;CD… |
| 0 | 0 | 3.017163598 | 23.47033601 | CCR1;CSF1… |
| 0 | 0 | 2.245853659 | 17.44331595 | CDKN1A;C… |
| 0 | 0 | 2.245853659 | 17.44331595 | ERO1A;PPF… |
| 0 | 0 | 2.883398899 | 22.28287584 | IL10;USP36… |
| 0 | 0 | 3.709025033 | 28.58892128 | ZBP1;AKIRI… |
| 0 | 0 | 3.709025033 | 28.58892128 | TRAF3IP2;I… |
| 0 | 0 | 6.555387524 | 50.48603007 | CD81;IL23A… |
| 0 | 0 | 6.555387524 | 50.48603007 | DDX58;IL2… |
| 0 | 0 | 3.28222138 | 25.2574445 | AKIRIN2;GI… |
| 0 | 0 | 1.656767524 | 12.7295351 | TRAF3IP2;I… |
| 0 | 0 | 1.526170634 | 11.68202779 | CIITA;CDKN… |
| 0 | 0 | 2.218666667 | 16.88246938 | DIRAS3;CD… |
| 0 | 0 | 3.936463461 | 29.93825705 | P2RY12;GF… |

| | | | | |
|---|---|---|---|---|
| 0 | 0 | 1.830372359 | 13.91455562 | RET;CSF3;[illegible] |
| 0 | 0 | 3.089607174 | 23.48540211 | LYN;ROCK1[illegible] |
| 0 | 0 | 1.964495783 | 14.90654317 | RET;CSF3;[illegible] |
| 0 | 0 | 2.003255057 | 15.12396891 | URI1;INSIG[illegible] |
| 0 | 0 | 5.364599092 | 40.47247611 | STAT3;IL1[illegible] |
| 0 | 0 | 5.364599092 | 40.47247611 | PYCARD;S[illegible] |
| 0 | 0 | 3.403084782 | 25.66915084 | ROCK1;PLE[illegible] |
| 0 | 0 | 4.244333615 | 31.97145357 | CYLD;ITCH;[illegible] |
| 0 | 0 | 4.244333615 | 31.97145357 | EDN1;SMA[illegible] |
| 0 | 0 | 2.49254905 | 18.71769826 | TCF7L2;DD[illegible] |
| 0 | 0 | 4.684341387 | 35.1520318 | ADGRG3;C[illegible] |
| 0 | 0 | 2.814583898 | 21.11488108 | GALNT11;[illegible] |
| 0 | 0 | 7.645943563 | 56.83820175 | SBNO2;IFN[illegible] |
| 0 | 0 | 7.645943563 | 56.83820175 | PPP1R15A;[illegible] |
| 0 | 0 | 7.645943563 | 56.83820175 | MOV10;P[illegible] |
| 0 | 0 | 7.645943563 | 56.83820175 | IRAK2;TNI[illegible] |
| 0 | 0 | 3.176159847 | 23.5986337 | IL10;EDN1;[illegible] |
| 0 | 0 | 3.176159847 | 23.5986337 | IL10;LYN;C[illegible] |
| 0 | 0 | 3.554277146 | 26.31334555 | NCF1;GAD[illegible] |
| 0 | 0 | 2.399251614 | 17.65457584 | LYN;STAR[illegible] |
| 0 | 0 | 3.001159326 | 22.07270709 | BCL2A1;CI[illegible] |
| 0 | 0 | 3.001159326 | 22.07270709 | PPP1R16B;[illegible] |
| 0 | 0 | 16.37556647 | 119.4176137 | CASP8;RIP[illegible] |
| 0 | 0 | 16.37556647 | 119.4176137 | LFNG;DOC[illegible] |
| 0 | 0 | 16.37556647 | 119.4176137 | CYLD;DUS[illegible] |
| 0 | 0 | 16.37556647 | 119.4176137 | CD74;MBP[illegible] |
| 0 | 0 | 16.37556647 | 119.4176137 | IL23A;IL23[illegible] |
| 0 | 0 | 16.37556647 | 119.4176137 | IL23A;IL23[illegible] |
| 0 | 0 | 16.37556647 | 119.4176137 | CD74;ANX[illegible] |
| 0 | 0 | 16.37556647 | 119.4176137 | IL6;STAT3;[illegible] |
| 0 | 0 | 3.748796452 | 27.29447259 | SLC7A5;CL[illegible] |
| 0 | 0 | 9.827918398 | 71.329274 | KMT2E;PR[illegible] |
| 0 | 0 | 9.827918398 | 71.329274 | ROCK1;IL1[illegible] |
| 0 | 0 | 9.827918398 | 71.329274 | BST1;RAC2[illegible] |
| 0 | 0 | 1.942218909 | 14.08612009 | RNF31;PTP[illegible] |
| 0 | 0 | 2.119126863 | 15.3089374 | FCN1;SRC;[illegible] |
| 0 | 0 | 5.826675068 | 41.88629299 | NOTCH3;K[illegible] |
| 0 | 0 | 5.826675068 | 41.88629299 | BST1;ANX[illegible] |
| 0 | 0 | 3.076727161 | 22.07474533 | DGKD;SRC;[illegible] |
| 0 | 0 | 1.77232238 | 12.69999541 | ACVRL1;O[illegible] |
| 0 | 0 | 2.077011056 | 14.86152779 | NRP1;CDK[illegible] |
| 0 | 0 | 4.371799723 | 31.12286429 | PYCARD;S[illegible] |
| 0 | 0 | 4.371799723 | 31.12286429 | C3;IL1A;IL[illegible] |
| 0 | 0 | 4.371799723 | 31.12286429 | IL10;IL1RN[illegible] |
| 0 | 0 | 2.917625248 | 20.76379802 | STAT5A;ST[illegible] |
| 0 | 0 | 2.116168582 | 14.82961951 | CDKN1B;C[illegible] |
| 0 | 0 | 3.57819008 | 24.95024708 | IL10;FOSL1[illegible] |

| | | | | |
|---|---|---|---|---|
| 0 | 0 | 1.530239359 | 10.65395036 | RET;BCAR3 |
| 0 | 0 | 2.220499609 | 15.45129157 | NCF1;FLT4 |
| 0 | 0 | 2.072818631 | 14.38450129 | RYR1;STEA |
| 0 | 0 | 2.253154377 | 15.54615001 | NRP1;EDN |
| 0 | 0 | 2.478555919 | 17.0956529 | ACVRL1;RI |
| 0 | 0 | 2.478555919 | 17.0956529 | NCF1;GAD |
| 0 | 0 | 2.478555919 | 17.0956529 | ROCK1;PR |
| 0 | 0 | 2.163357153 | 14.85937401 | NCF1;FLT4 |
| 0 | 0 | 2.626489564 | 18.02880375 | NRP1;PTP |
| 0 | 0 | 2.626489564 | 18.02880375 | LYN;GPR3 |
| 0 | 0 | 6.553287982 | 44.96869783 | CASP8;IFI1 |
| 0 | 0 | 6.553287982 | 44.96869783 | RAP1B;SLC |
| 0 | 0 | 6.553287982 | 44.96869783 | PYCARD;B |
| 0 | 0 | 6.553287982 | 44.96869783 | PTPN1;BCL |
| 0 | 0 | 3.797123391 | 26.01848606 | CD74;BST1 |
| 0 | 0 | 3.797123391 | 26.01848606 | IFNAR2;ZB |
| 0 | 0 | 1.443524423 | 9.876216118 | FMR1;NAB |
| 0 | 0 | 3.280492424 | 22.42259919 | RET;NAPS |
| 0 | 0 | 1.903257171 | 12.9417341 | SMG1;ROC |
| 0 | 0 | 5.243705104 | 35.25823158 | SELP;SPN; |
| 0 | 0 | 5.243705104 | 35.25823158 | IFNG;IFNG |
| 0 | 0 | 2.136191473 | 14.3544428 | CSF3;FLT1; |
| 0 | 0 | 4.538752473 | 30.44388468 | IL10;LYN;P |
| 0 | 0 | 4.538752473 | 30.44388468 | PDP1;DUS |
| 0 | 0 | 4.538752473 | 30.44388468 | LYN;IFNG; |
| 0 | 0 | 4.538752473 | 30.44388468 | LYN;SELP; |
| 0 | 0 | 2.895408732 | 19.37811562 | CDKN1A;IT |
| 0 | 0 | 2.895408732 | 19.37811562 | RAMP2;CX |
| 0 | 0 | 2.255490608 | 15.07043592 | PTPN1;CD |
| 0 | 0 | 3.422419044 | 22.86237123 | KMT2E;AD |
| 0 | 0 | 3.422419044 | 22.86237123 | PAK1;EDN |
| 0 | 0 | 2.088051611 | 13.88056988 | PHLDB1;CL |
| 0 | 0 | 7.861881375 | 51.7973519 | STEAP3;ST |
| 0 | 0 | 7.861881375 | 51.7973519 | IFNG;DDIT |
| 0 | 0 | 7.861881375 | 51.7973519 | ANXA1;TB |
| 0 | 0 | 7.861881375 | 51.7973519 | ADAM17;N |
| 0 | 0 | 7.861881375 | 51.7973519 | PRR7;GSN; |
| 0 | 0 | 7.861881375 | 51.7973519 | HAAO;KYN |
| 0 | 0 | 26.19252548 | 172.47096 | SLC11A1;S |
| 0 | 0 | 26.19252548 | 172.47096 | SLC11A1;S |
| 0 | 0 | 26.19252548 | 172.47096 | B2M;TNF; |
| 0 | 0 | 26.19252548 | 172.47096 | STEAP3;ST |
| 0 | 0 | 26.19252548 | 172.47096 | BCAR3;ED |
| 0 | 0 | 26.19252548 | 172.47096 | PPP1R15A |
| 0 | 0 | 26.19252548 | 172.47096 | CYP27B1;G |
| 0 | 0 | 26.19252548 | 172.47096 | MAP2K3;Z |
| 0 | 0 | 26.19252548 | 172.47096 | IL23A;IL23 |

| | | | | |
|---|---|---|---|---|
| 0 | 0 | 26.19252548 | 172.47096 | CD74;ANX |
| 0 | 0 | 26.19252548 | 172.47096 | IL1R1;IL1B |
| 0 | 0 | 26.19252548 | 172.47096 | RBPJ;HIF1 |
| 0 | 0 | 26.19252548 | 172.47096 | PTPN1;ITC |
| 0 | 0 | 26.19252548 | 172.47096 | SLC9A3R1; |
| 0 | 0 | 26.19252548 | 172.47096 | IL1R1;IL1B |
| 0 | 0 | 26.19252548 | 172.47096 | RIPK2;TNIF |
| 0 | 0 | 1.872092518 | 12.28934682 | CDKN1A;P |
| 0 | 0 | 3.607059046 | 23.59084605 | ILRUN;ITC |
| 0 | 0 | 3.607059046 | 23.59084605 | CCR1;CXCL |
| 0 | 0 | 2.963292547 | 19.26760192 | UTP15;DD |
| 0 | 0 | 2.963292547 | 19.26760192 | IL10;EGR1; |
| 0 | 0 | 2.963292547 | 19.26760192 | IL10;IL10R |
| 0 | 0 | 1.904007634 | 12.37706159 | PTGFR;GP |
| 0 | 0 | 2.446320366 | 15.89539826 | RTP4;NCF1 |
| 0 | 0 | 1.941455087 | 12.51287361 | RYR1;CCL1 |
| 0 | 0 | 10.9164149 | 70.16691693 | GSN;CASS |
| 0 | 0 | 10.9164149 | 70.16691693 | ERAP2;ERA |
| 0 | 0 | 10.9164149 | 70.16691693 | P2RY12;CS |
| 0 | 0 | 10.9164149 | 70.16691693 | CIITA;XBP1 |
| 0 | 0 | 10.9164149 | 70.16691693 | CD300A;IL |
| 0 | 0 | 10.9164149 | 70.16691693 | SLAMF8;IL |
| 0 | 0 | 10.9164149 | 70.16691693 | IL10;GBP5; |
| 0 | 0 | 10.9164149 | 70.16691693 | PARP10;D |
| 0 | 0 | 10.9164149 | 70.16691693 | FLOT1;FLO |
| 0 | 0 | 3.279628928 | 20.99525568 | IL23A;IL23 |
| 0 | 0 | 3.279628928 | 20.99525568 | MN1;CDK |
| 0 | 0 | 1.985990695 | 12.71103171 | ERO1A;PR |
| 0 | 0 | 5.733796296 | 36.43525817 | DAXX;DDX |
| 0 | 0 | 5.733796296 | 36.43525817 | CXCL8;HSP |
| 0 | 0 | 5.733796296 | 36.43525817 | CLN3;ANX |
| 0 | 0 | 5.733796296 | 36.43525817 | RAP1B;SLC |
| 0 | 0 | 5.733796296 | 36.43525817 | CD74;EDN |
| 0 | 0 | 5.733796296 | 36.43525817 | PNPT1;STE |
| 0 | 0 | 5.733796296 | 36.43525817 | PNPT1;STE |
| 0 | 0 | 4.21431273 | 26.69857732 | ACVRL1;N |
| 0 | 0 | 4.21431273 | 26.69857732 | PYCARD;N |
| 0 | 0 | 2.399137025 | 15.15455487 | KMT2E;CD |
| 0 | 0 | 3.045819805 | 19.23787955 | RIPK2;SH2 |
| 0 | 0 | 3.045819805 | 19.23787955 | IL10;IL1RN |
| 0 | 0 | 4.766729679 | 30.03087766 | C3;NR4A3; |
| 0 | 0 | 2.058622002 | 12.94900906 | AMER1;PR |
| 0 | 0 | 2.870523873 | 17.9907453 | ARG2;RSA |
| 0 | 0 | 3.435096067 | 21.45245136 | C3;CXCL8; |
| 0 | 0 | 2.625255973 | 16.39009758 | CCL14;CCR |
| 0 | 0 | 2.625255973 | 16.39009758 | LYN;FLT1;S |
| 0 | 0 | 2.016517517 | 12.57089312 | SRC;CLEC1 |

| | | | | |
|---|---|---|---|---|
| 0 | 0 | 2.016517517 | 12.57089312 | SRC;CLEC1[illegible] |
| 0 | 0 | 1.602747418 | 9.946191862 | OTUD4;US[illegible] |
| 0 | 0 | 1.914801476 | 11.69970747 | RNASEH2B[illegible] |
| 0 | 0 | 3.642535839 | 22.23721168 | SELP;ROCK[illegible] |
| 0 | 0 | 3.642535839 | 22.23721168 | CYP27B1;F[illegible] |
| 0 | 0 | 3.642535839 | 22.23721168 | BST1;P2RX[illegible] |
| 0 | 0 | 3.642535839 | 22.23721168 | SPRED2;CD[illegible] |
| 0 | 0 | 3.642535839 | 22.23721168 | IL10;XBP1;[illegible] |
| 0 | 0 | 3.642535839 | 22.23721168 | IFITM3;IFN[illegible] |
| 0 | 0 | 2.41152461 | 14.70751929 | BCAR3;DG[illegible] |
| 0 | 0 | 1.760671101 | 10.71610779 | CFD;LGALS[illegible] |
| 0 | 0 | 2.053248668 | 12.46878539 | CDKN1A;C[illegible] |
| 0 | 0 | 2.561077351 | 15.47623622 | SLC13A3;S[illegible] |
| 0 | 0 | 2.561077351 | 15.47623622 | RHBDD1;N[illegible] |
| 0 | 0 | 2.783377542 | 16.81675329 | TLE3;IL15;[illegible] |
| 0 | 0 | 2.264232487 | 13.63891554 | PDIA3;FCE[illegible] |
| 0 | 0 | 6.551190026 | 39.3985662 | XBP1;DGA[illegible] |
| 0 | 0 | 6.551190026 | 39.3985662 | DDX5;C5A[illegible] |
| 0 | 0 | 6.551190026 | 39.3985662 | LYN;CSF1;[illegible] |
| 0 | 0 | 6.551190026 | 39.3985662 | IL6;IL1B;CC[illegible] |
| 0 | 0 | 6.551190026 | 39.3985662 | PARP10;PA[illegible] |
| 0 | 0 | 6.551190026 | 39.3985662 | IL10;CIITA;[illegible] |
| 0 | 0 | 1.200213258 | 7.204979362 | ZNF296;M[illegible] |
| 0 | 0 | 3.933131619 | 23.55586627 | CYLD;ITCH[illegible] |
| 0 | 0 | 3.933131619 | 23.55586627 | HSPA5;DD[illegible] |
| 0 | 0 | 3.933131619 | 23.55586627 | PTPN1;HH[illegible] |
| 0 | 0 | 3.278766086 | 19.5595986 | IL10;CDKN[illegible] |
| 0 | 0 | 1.823478094 | 10.80282901 | PPP1R15B;[illegible] |
| 0 | 0 | 4.369250158 | 25.82985908 | FMNL3;IKB[illegible] |
| 0 | 0 | 4.369250158 | 25.82985908 | PYCARD;IL[illegible] |
| 0 | 0 | 5.096413874 | 30.06821065 | FGD3;FGD[illegible] |
| 0 | 0 | 5.096413874 | 30.06821065 | SPRED2;SP[illegible] |
| 0 | 0 | 5.096413874 | 30.06821065 | KMT2E;PR[illegible] |
| 0 | 0 | 5.096413874 | 30.06821065 | SELP;NFAT[illegible] |
| 0 | 0 | 5.096413874 | 30.06821065 | CD74;SEM[illegible] |
| 0 | 0 | 5.096413874 | 30.06821065 | EDN1;ANX[illegible] |
| 0 | 0 | 2.499954855 | 14.62241331 | TCF7L2;EG[illegible] |
| 0 | 0 | 2.499954855 | 14.62241331 | RAMP2;CX[illegible] |
| 0 | 0 | 2.842436869 | 16.60158014 | XBP1;IL23[illegible] |
| 0 | 0 | 3.450624291 | 20.03736219 | ERO1A;PP[illegible] |
| 0 | 0 | 2.31668713 | 13.31789257 | LYN;FLT1;I[illegible] |
| 0 | 0 | 8.186839124 | 46.92960497 | XBP1;HSP[illegible] |
| 0 | 0 | 8.186839124 | 46.92960497 | NR4A1;NR[illegible] |
| 0 | 0 | 8.186839124 | 46.92960497 | KYNU;KYA[illegible] |
| 0 | 0 | 8.186839124 | 46.92960497 | CD74;SEM[illegible] |
| 0 | 0 | 8.186839124 | 46.92960497 | GBP5;DDX[illegible] |
| 0 | 0 | 8.186839124 | 46.92960497 | IFNG;IL12[illegible] |

| | | | | |
|---|---|---|---|---|
| 0 | 0 | 8.186839124 | 46.92960497 | IKBKB;ROC |
| 0 | 0 | 8.186839124 | 46.92960497 | BTG1;NOT |
| 0 | 0 | 8.186839124 | 46.92960497 | IFNG;CLEC |
| 0 | 0 | 8.186839124 | 46.92960497 | NCR1;NOS |
| 0 | 0 | 8.186839124 | 46.92960497 | PPP1R15A; |
| 0 | 0 | 8.186839124 | 46.92960497 | CD274;ZFP |
| 0 | 0 | 8.186839124 | 46.92960497 | CDKN2D;C |
| 0 | 0 | 2.188504874 | 12.48530542 | RET;P2RY1 |
| 0 | 0 | 3.136030017 | 17.87663586 | BCAR3;AD |
| 0 | 0 | 3.687098147 | 20.89271337 | RIPOR2;CD |
| 0 | 0 | 2.441675265 | 13.82377373 | PLK3;JUN; |
| 0 | 0 | 2.914720855 | 16.44622378 | PNKD;ANX |
| 0 | 0 | 2.914720855 | 16.44622378 | SPRED2;SP |
| 0 | 0 | 2.523473494 | 14.21306693 | DIRAS3;CD |
| 0 | 0 | 2.121358429 | 11.92992678 | HDAC4;CD |
| 0 | 0 | 2.121358429 | 11.92992678 | MAP2K3;S |
| 0 | 0 | 2.75058651 | 15.45168601 | CCL14;HSD |
| 0 | 0 | 2.75058651 | 15.45168601 | TRAF3IP2; |
| 0 | 0 | 2.75058651 | 15.45168601 | IL10;CD40; |
| 0 | 0 | 13.09550774 | 73.3030207 | CD14;TLR6 |
| 0 | 0 | 13.09550774 | 73.3030207 | HERC5;UB |
| 0 | 0 | 13.09550774 | 73.3030207 | AGO2;ABC |
| 0 | 0 | 13.09550774 | 73.3030207 | LYN;ACOD |
| 0 | 0 | 13.09550774 | 73.3030207 | FCER2;IFN |
| 0 | 0 | 13.09550774 | 73.3030207 | TASL;DDX3 |
| 0 | 0 | 13.09550774 | 73.3030207 | PNP;ADA2; |
| 0 | 0 | 13.09550774 | 73.3030207 | OTUD4;ZB |
| 0 | 0 | 13.09550774 | 73.3030207 | IL23A;IL23 |
| 0 | 0 | 13.09550774 | 73.3030207 | CD74;NLR |
| 0 | 0 | 13.09550774 | 73.3030207 | ROCK1;PR |
| 0 | 0 | 1.713682323 | 9.576399392 | USP36;CD |
| 0 | 0 | 1.877442177 | 10.45302024 | ACVRL1;CD |
| 0 | 0 | 4.032921332 | 22.40027211 | PPP1R15A; |
| 0 | 0 | 4.032921332 | 22.40027211 | XBP1;SESN |
| 0 | 0 | 3.277903897 | 18.1139233 | PYCARD;LY |
| 0 | 0 | 3.277903897 | 18.1139233 | CD74;IL6;T |
| 0 | 0 | 5.61498192 | 30.9566673 | ABCA1;NF |
| 0 | 0 | 5.61498192 | 30.9566673 | CD74;MBP |
| 0 | 0 | 5.61498192 | 30.9566673 | IL6;PTPRC; |
| 0 | 0 | 5.61498192 | 30.9566673 | RGS2;GSN; |
| 0 | 0 | 5.61498192 | 30.9566673 | CDKN1A;H |
| 0 | 0 | 4.586507937 | 25.17853871 | IL10;IL6;SI |
| 0 | 0 | 4.586507937 | 25.17853871 | SPRED2;SP |
| 0 | 0 | 4.586507937 | 25.17853871 | CD300A;D |
| 0 | 0 | 4.586507937 | 25.17853871 | IKBKB;ROC |
| 0 | 0 | 4.586507937 | 25.17853871 | GBP5;DDX |
| 0 | 0 | 2.188053657 | 11.99698127 | NCOA1;ST |

| | | | | |
|---|---|---|---|---|
| 0 | 0 | 3.00518862 | 16.37422296 | IL10;MAD2 |
| 0 | 0 | 2.460244503 | 13.38891843 | ACVRL1;M. |
| 0 | 0 | 2.810461405 | 15.21055592 | ZFYVE28;R |
| 0 | 0 | 2.810461405 | 15.21055592 | SLC9A3R1; |
| 0 | 0 | 2.810461405 | 15.21055592 | XBP1;ANX/ |
| 0 | 0 | 2.664476799 | 14.3980205 | P2RY12;NF |
| 0 | 0 | 1.717315827 | 9.256003188 | SMG1;ROC |
| 0 | 0 | 3.470009789 | 18.61643479 | CCR1;LYN; |
| 0 | 0 | 3.470009789 | 18.61643479 | DUSP10;SC |
| 0 | 0 | 2.276687003 | 12.19930924 | DGKD;SRC; |
| 0 | 0 | 1.471966647 | 7.815243974 | SERPINE1;I |
| 0 | 0 | 1.792222646 | 9.512443821 | RET;FLT1;C |
| 0 | 0 | 2.332886698 | 12.37496687 | IL10;HDAC |
| 0 | 0 | 3.121633065 | 16.42304184 | CDKN2D;C |
| 0 | 0 | 3.121633065 | 16.42304184 | ABCA1;NFI |
| 0 | 0 | 2.400099854 | 12.6207151 | TLE3;IL15;I |
| 0 | 0 | 1.644127774 | 8.597476184 | RAMP2;RC |
| 0 | 0 | 3.744639482 | 19.56304545 | CDKN2D;C |
| 0 | 0 | 3.744639482 | 19.56304545 | CD74;RIPO |
| 0 | 0 | 2.481898344 | 12.96053417 | XBP1;SYAP |
| 0 | 0 | 2.481898344 | 12.96053417 | SRC;PRKCE |
| 0 | 0 | 2.884814534 | 15.02801977 | NLGN3;UG |
| 0 | 0 | 2.884814534 | 15.02801977 | CCR1;CSF1 |
| 0 | 0 | 2.583585859 | 13.43057588 | KMT2E;LYI |
| 0 | 0 | 1.48318639 | 7.709214395 | UBE2D1;HI |
| 0 | 0 | 2.713392263 | 14.08791337 | LFNG;ADAI |
| 0 | 0 | 6.549093656 | 33.75606817 | DCSTAMP; |
| 0 | 0 | 6.549093656 | 33.75606817 | IFIH1;IRF3; |
| 0 | 0 | 6.549093656 | 33.75606817 | CD74;FCER |
| 0 | 0 | 6.549093656 | 33.75606817 | HAAO;KYN |
| 0 | 0 | 6.549093656 | 33.75606817 | ABCA1;NR |
| 0 | 0 | 6.549093656 | 33.75606817 | IL10;IL1RN |
| 0 | 0 | 6.549093656 | 33.75606817 | SELP;SOX1 |
| 0 | 0 | 6.549093656 | 33.75606817 | PRKCB;IL1I |
| 0 | 0 | 6.549093656 | 33.75606817 | PARP10;PA |
| 0 | 0 | 6.549093656 | 33.75606817 | NRP1;MYC |
| 0 | 0 | 6.549093656 | 33.75606817 | IL10;IL1RN |
| 0 | 0 | 6.549093656 | 33.75606817 | ABCA1;KLF |
| 0 | 0 | 1.856266861 | 9.551495557 | HDAC4;CSI |
| 0 | 0 | 4.169312169 | 21.33580885 | CCDC186;S |
| 0 | 0 | 4.169312169 | 21.33580885 | ADAM17;C |
| 0 | 0 | 2.147703985 | 10.96644383 | PDIA3;NCF |
| 0 | 0 | 1.780687258 | 9.085844809 | RAMP2;RC |
| 0 | 0 | 1.67941762 | 8.568174083 | CCL14;NRF |
| 0 | 0 | 1.693076238 | 8.628275345 | BCAR3;RET |
| 0 | 0 | 1.760014433 | 8.968459517 | CDKN1A;G |
| 0 | 0 | 1.741118869 | 8.865752948 | RNF31;TEN |

| | | | | |
|---|---|---|---|---|
| 0 | 0 | 3.27704236 | 16.6560155 | PTPN1;PTF |
| 0 | 0 | 4.912825841 | 24.91557424 | ADAM19;C |
| 0 | 0 | 4.912825841 | 24.91557424 | TRPC5;TRP |
| 0 | 0 | 4.912825841 | 24.91557424 | CCR1;SRGN |
| 0 | 0 | 4.912825841 | 24.91557424 | CD74;MDN |
| 0 | 0 | 4.912825841 | 24.91557424 | IL10;TLR8;I |
| 0 | 0 | 4.912825841 | 24.91557424 | HEXIM1;IFI |
| 0 | 0 | 4.912825841 | 24.91557424 | DYNC1H1;S |
| 0 | 0 | 4.912825841 | 24.91557424 | PYCARD;SF |
| 0 | 0 | 4.912825841 | 24.91557424 | CD74;SEM |
| 0 | 0 | 4.912825841 | 24.91557424 | DGKG;CD4 |
| 0 | 0 | 4.912825841 | 24.91557424 | RGS2;GSN; |
| 0 | 0 | 4.912825841 | 24.91557424 | ADAM17;P |
| 0 | 0 | 1.997918594 | 10.11284861 | PLK3;PRKA |
| 0 | 0 | 2.979568672 | 14.92932245 | PREX1;NRF |
| 0 | 0 | 2.622794396 | 13.06542042 | CCL14;IL6;I |
| 0 | 0 | 2.622794396 | 13.06542042 | PTPN1;AD |
| 0 | 0 | 2.019574045 | 10.05029815 | IL10;HDAC |
| 0 | 0 | 1.516329085 | 7.522127058 | OTUD4;CX |
| 0 | 0 | 1.516329085 | 7.522127058 | OTUD4;US |
| 0 | 0 | 2.109230144 | 10.45461427 | TRAF2;TIR |
| 0 | 0 | 2.109230144 | 10.45461427 | HM13;VPS |
| 0 | 0 | 1.969262918 | 9.707511844 | ROCK1;PRI |
| 0 | 0 | 3.494795211 | 17.18902159 | EDN1;PAK |
| 0 | 0 | 3.494795211 | 17.18902159 | LYN;ELF1;C |
| 0 | 0 | 2.144967608 | 10.47106612 | IL10;KDM4 |
| 0 | 0 | 8.729835158 | 42.43679444 | IL18RAP;IL |
| 0 | 0 | 8.729835158 | 42.43679444 | NAPSA;AB |
| 0 | 0 | 8.729835158 | 42.43679444 | C3;CFD;CFI |
| 0 | 0 | 8.729835158 | 42.43679444 | IL18RAP;IL |
| 0 | 0 | 8.729835158 | 42.43679444 | PYCARD;M |
| 0 | 0 | 8.729835158 | 42.43679444 | NR1H2;NR |
| 0 | 0 | 8.729835158 | 42.43679444 | RAP1B;PN |
| 0 | 0 | 8.729835158 | 42.43679444 | NR1H2;NR |
| 0 | 0 | 8.729835158 | 42.43679444 | TBX21;ZC3 |
| 0 | 0 | 8.729835158 | 42.43679444 | RIPK2;NOD |
| 0 | 0 | 8.729835158 | 42.43679444 | IL23A;IL23 |
| 0 | 0 | 8.729835158 | 42.43679444 | BCL2L11;B |
| 0 | 0 | 8.729835158 | 42.43679444 | IFNG;CLEC |
| 0 | 0 | 8.729835158 | 42.43679444 | PNPT1;ZC3 |
| 0 | 0 | 8.729835158 | 42.43679444 | SFPQ;RIPK |
| 0 | 0 | 8.729835158 | 42.43679444 | PTPN1;ITC |
| 0 | 0 | 8.729835158 | 42.43679444 | CD74;ANX |
| 0 | 0 | 8.729835158 | 42.43679444 | SENP7;USF |
| 0 | 0 | 8.729835158 | 42.43679444 | SLC6A6;AS |
| 0 | 0 | 8.729835158 | 42.43679444 | ANXA1;CD |
| 0 | 0 | 8.729835158 | 42.43679444 | TASL;DDX3 |

| | | | | |
|---|---|---|---|---|
| 0 | 0 | 8.729835158 | 42.43679444 | RYR1;CCL3 . |
| 0 | 0 | 8.729835158 | 42.43679444 | S1PR1;S1P . |
| 0 | 0 | 8.729835158 | 42.43679444 | NAPSA;AB( . |
| 0 | 0 | 8.729835158 | 42.43679444 | MX2;MX1; . |
| 0 | 0 | 3.104387292 | 14.95622196 | PTPN1;PTF . |
| 0 | 0 | 2.435670996 | 11.72048046 | IL10;BTG3; . |
| 0 | 0 | 2.435670996 | 11.72048046 | MAP2K3;S . |
| 0 | 0 | 3.82164903 | 18.25833134 | CD74;RIPO . |
| 0 | 0 | 3.82164903 | 18.25833134 | PDP1;EDN . |
| 0 | 0 | 3.82164903 | 18.25833134 | CD74;EDN . |
| 0 | 0 | 3.82164903 | 18.25833134 | GSDMD;ZB . |
| 0 | 0 | 3.82164903 | 18.25833134 | LYN;ADAM . |
| 0 | 0 | 3.82164903 | 18.25833134 | IL12B;TNFS . |
| 0 | 0 | 3.82164903 | 18.25833134 | IL6;IL1B;IL . |
| 0 | 0 | 3.82164903 | 18.25833134 | TRAF2;TNF . |
| 0 | 0 | 2.849857705 | 13.60391883 | FCN1;XKR8 . |
| 0 | 0 | 2.849857705 | 13.60391883 | PPP1R16B; . |
| 0 | 0 | 2.670816161 | 12.72610484 | XBP1;SPTL . |
| 0 | 0 | 2.670816161 | 12.72610484 | UGCG;PTP . |
| 0 | 0 | 2.011015994 | 9.562236405 | NIBAN1;DI . |
| 0 | 0 | 1.864141201 | 8.840548349 | CCR1;CCL1 . |
| 0 | 0 | 1.468571828 | 6.933020232 | CDKN1A;CI . |
| 0 | 0 | 1.716120083 | 8.049203853 | RAMP2;RC . |
| 0 | 0 | 4.366704445 | 20.42850275 | CASP8;CSF . |
| 0 | 0 | 4.366704445 | 20.42850275 | CD300A;ZC . |
| 0 | 0 | 4.366704445 | 20.42850275 | FCER2;MA . |
| 0 | 0 | 4.366704445 | 20.42850275 | LAMP1;IL2 . |
| 0 | 0 | 4.366704445 | 20.42850275 | CASP8;CSF . |
| 0 | 0 | 4.366704445 | 20.42850275 | C3;NR1H2; . |
| 0 | 0 | 2.036262514 | 9.509152552 | FUS;CLK4;F . |
| 0 | 0 | 5.457263343 | 25.44631774 | IL10;NR4A . |
| 0 | 0 | 5.457263343 | 25.44631774 | SLC9A3R1; . |
| 0 | 0 | 5.457263343 | 25.44631774 | DYNC1H1;S . |
| 0 | 0 | 5.457263343 | 25.44631774 | ATF7IP;SET . |
| 0 | 0 | 5.457263343 | 25.44631774 | P2RY12;RA . |
| 0 | 0 | 5.457263343 | 25.44631774 | CASP8;CSF . |
| 0 | 0 | 5.457263343 | 25.44631774 | NT5C3A;DI . |
| 0 | 0 | 5.457263343 | 25.44631774 | MBD2;BAZ . |
| 0 | 0 | 5.457263343 | 25.44631774 | CD274;CD8 . |
| 0 | 0 | 5.457263343 | 25.44631774 | CD274;RIP . |
| 0 | 0 | 5.457263343 | 25.44631774 | IL6;RSAD2; . |
| 0 | 0 | 5.457263343 | 25.44631774 | LYN;ACOD . |
| 0 | 0 | 5.457263343 | 25.44631774 | INSIG1;PM . |
| 0 | 0 | 5.457263343 | 25.44631774 | RIPK3;TNF . |
| 0 | 0 | 5.457263343 | 25.44631774 | HAAO;KYN . |
| 0 | 0 | 3.276181474 | 15.18292217 | ZNFX1;MB . |
| 0 | 0 | 3.276181474 | 15.18292217 | GGT5;CYP4 . |

| | | | | |
|---|---|---|---|---|
| 0 | 0 | 3.276181474 | 15.18292217 | DGAT2;ELC… |
| 0 | 0 | 3.276181474 | 15.18292217 | CCR1;SRGN… |
| 0 | 0 | 3.276181474 | 15.18292217 | ITGAM;DD… |
| 0 | 0 | 3.276181474 | 15.18292217 | RAP1GAP2… |
| 0 | 0 | 1.978980608 | 9.144139866 | ARRDC4;AI… |
| 0 | 0 | 1.978980608 | 9.144139866 | DIRAS3;CD… |
| 0 | 0 | 2.458585763 | 11.27803036 | MOV10;BT… |
| 0 | 0 | 2.186252211 | 10.0210684 | MYLIP;DEN… |
| 0 | 0 | 2.948997731 | 13.47351972 | CD274;DDI… |
| 0 | 0 | 2.948997731 | 13.47351972 | ADAM17;II… |
| 0 | 0 | 2.575281172 | 11.73878918 | NRP1;NR4… |
| 0 | 0 | 2.575281172 | 11.73878918 | ING5;KAT2… |
| 0 | 0 | 2.730955984 | 12.42311446 | LAT2;PTPR… |
| 0 | 0 | 2.730955984 | 12.42311446 | ROCK1;IFN… |
| 0 | 0 | 2.730955984 | 12.42311446 | RET;LYN;P2… |
| 0 | 0 | 2.239244355 | 10.11298489 | OTUD4;IL1… |
| 0 | 0 | 1.819011407 | 8.206500153 | DIRAS3;JUI… |
| 0 | 0 | 1.790414606 | 8.056140296 | CD74;CREE… |
| 0 | 0 | 3.527472527 | 15.75466694 | CXCL8;TRIN… |
| 0 | 0 | 3.527472527 | 15.75466694 | PNP;IL23A;… |
| 0 | 0 | 3.527472527 | 15.75466694 | CLEC3B;GS… |
| 0 | 0 | 3.527472527 | 15.75466694 | EGR1;DAX… |
| 0 | 0 | 1.902056947 | 8.477048325 | ACVRL1;US… |
| 0 | 0 | 1.426277338 | 6.31537658 | RARG;MAN… |
| 0 | 0 | 1.544185157 | 6.791334674 | PSMB10;A… |
| 0 | 0 | 3.083287001 | 13.47307868 | ACVRL1;IKI… |
| 0 | 0 | 3.083287001 | 13.47307868 | PYCARD;B… |
| 0 | 0 | 1.917866947 | 8.366649217 | IL10;HDAC… |
| 0 | 0 | 1.917866947 | 8.366649217 | WASHC4;E… |
| 0 | 0 | 1.917866947 | 8.366649217 | SLC22A3;S… |
| 0 | 0 | 1.397633008 | 6.081420903 | RNF13;RNI… |
| 0 | 0 | 2.621566402 | 11.36725331 | CD81;CD3… |
| 0 | 0 | 1.874776192 | 8.126865076 | TCF7L2;CSI… |
| 0 | 0 | 2.808407175 | 12.17318472 | DAXX;DDX… |
| 0 | 0 | 3.929807329 | 16.99786475 | CHI3L1;TR… |
| 0 | 0 | 3.929807329 | 16.99786475 | MGAM;PH… |
| 0 | 0 | 3.929807329 | 16.99786475 | PIK3CA;SR… |
| 0 | 0 | 3.929807329 | 16.99786475 | PTPRC;ZC3… |
| 0 | 0 | 3.929807329 | 16.99786475 | CNOT7;ST… |
| 0 | 0 | 3.929807329 | 16.99786475 | NRP1;PTPN… |
| 0 | 0 | 3.929807329 | 16.99786475 | MAD2L2;P… |
| 0 | 0 | 3.929807329 | 16.99786475 | FLCN;SEH1… |
| 0 | 0 | 3.929807329 | 16.99786475 | PPP1R15A;… |
| 0 | 0 | 3.929807329 | 16.99786475 | RAP1B;RIP… |
| 0 | 0 | 3.929807329 | 16.99786475 | IL10;NLRP1… |
| 0 | 0 | 3.929807329 | 16.99786475 | IL10;MAD2… |
| 0 | 0 | 1.991294982 | 8.580441194 | ARHGAP9;… |

| | | | | |
|---|---|---|---|---|
| 0 | 0 | 1.991294982 | 8.580441194 | ABCA1;NRI |
| 0 | 0 | 2.242992424 | 9.615529155 | ACVRL1;RC |
| 0 | 0 | 2.242992424 | 9.615529155 | IVNS1ABP; |
| 0 | 0 | 2.242992424 | 9.615529155 | PXK;NDFIP |
| 0 | 0 | 1.727549467 | 7.401063613 | ILRUN;PRK |
| 0 | 0 | 6.546998867 | 28.00948369 | NRP1;SPRE |
| 0 | 0 | 6.546998867 | 28.00948369 | IL10;NFKBI |
| 0 | 0 | 6.546998867 | 28.00948369 | HYAL2;CXC |
| 0 | 0 | 6.546998867 | 28.00948369 | HYAL2;CXC |
| 0 | 0 | 6.546998867 | 28.00948369 | KMT2E;AD |
| 0 | 0 | 6.546998867 | 28.00948369 | RAP1B;STX |
| 0 | 0 | 6.546998867 | 28.00948369 | TBX21;ZC3 |
| 0 | 0 | 6.546998867 | 28.00948369 | PYCARD;CI |
| 0 | 0 | 6.546998867 | 28.00948369 | IL12A;CALF |
| 0 | 0 | 6.546998867 | 28.00948369 | IL6;PDPN;F |
| 0 | 0 | 6.546998867 | 28.00948369 | IL6;RSAD2; |
| 0 | 0 | 6.546998867 | 28.00948369 | RGCC;RIC1 |
| 0 | 0 | 6.546998867 | 28.00948369 | IFNG;ABCA |
| 0 | 0 | 6.546998867 | 28.00948369 | TLR6;FBXO |
| 0 | 0 | 6.546998867 | 28.00948369 | GFI1;XIAP; |
| 0 | 0 | 6.546998867 | 28.00948369 | ANXA1;IL1 |
| 0 | 0 | 6.546998867 | 28.00948369 | SESN2;ANK |
| 0 | 0 | 6.546998867 | 28.00948369 | XBP1;SERP |
| 0 | 0 | 6.546998867 | 28.00948369 | MX2;MX1; |
| 0 | 0 | 4.677384549 | 19.82280163 | KDM4A;KD |
| 0 | 0 | 4.677384549 | 19.82280163 | NT5C3A;DI |
| 0 | 0 | 4.677384549 | 19.82280163 | PPP1R3B;P |
| 0 | 0 | 4.677384549 | 19.82280163 | CD53;RIPO |
| 0 | 0 | 4.677384549 | 19.82280163 | IL23A;IL12I |
| 0 | 0 | 4.677384549 | 19.82280163 | STX1B;STX |
| 0 | 0 | 1.813164894 | 7.615297555 | CCR1;CCL1 |
| 0 | 0 | 2.134846709 | 8.96634003 | HACE1;DTX |
| 0 | 0 | 3.27532124 | 13.69057561 | VCAN;CHST |
| 0 | 0 | 3.27532124 | 13.69057561 | PARP10;HA |
| 0 | 0 | 3.27532124 | 13.69057561 | ZFP36;TUT |
| 0 | 0 | 3.27532124 | 13.69057561 | PTPRC;CD8 |
| 0 | 0 | 3.27532124 | 13.69057561 | PIK3CA;SR |
| 0 | 0 | 3.27532124 | 13.69057561 | CLN3;PRKC |
| 0 | 0 | 1.956246839 | 8.169362205 | PLK3;BTG2 |
| 0 | 0 | 2.403318193 | 10.0293733 | TGM1;BCR |
| 0 | 0 | 2.403318193 | 10.0293733 | PPP1R16B; |
| 0 | 0 | 1.904450021 | 7.889006005 | RYR1;CLIC4 |
| 0 | 0 | 1.614813472 | 6.684977752 | AVIL;NEDD |
| 0 | 0 | 2.520591403 | 10.41992436 | DCSTAMP; |
| 0 | 0 | 2.520591403 | 10.41992436 | TRPC5;TRP |
| 0 | 0 | 2.911825247 | 12.00465669 | KDM5A;KD |
| 0 | 0 | 2.911825247 | 12.00465669 | CDKN2D;CI |

| | | | | |
|---|---|---|---|---|
| 0 | 0 | 2.911825247 | 12.00465669 | KMT2E;AD… |
| 0 | 0 | 2.911825247 | 12.00465669 | IL23A;IL23… |
| 0 | 0 | 1.860401863 | 7.660366186 | SLC22A3;S… |
| 0 | 0 | 2.680597579 | 11.02722229 | CD74;EDN… |
| 0 | 0 | 2.680597579 | 11.02722229 | GOLGA7B;… |
| 0 | 0 | 1.822488079 | 7.472143189 | CXCL8;EGR… |
| 0 | 0 | 2.247460378 | 9.114791449 | IL10;ADAM… |
| 0 | 0 | 1.460670329 | 5.883493073 | RTP4;VPS4… |
| 0 | 0 | 3.572346052 | 14.313563 | IL10;APOA… |
| 0 | 0 | 3.572346052 | 14.313563 | FCN1;IFITM… |
| 0 | 0 | 3.572346052 | 14.313563 | NT5C3A;DI… |
| 0 | 0 | 3.572346052 | 14.313563 | STX1B;SYN… |
| 0 | 0 | 1.832936933 | 7.331538971 | SEMA6A;N… |
| 0 | 0 | 1.832936933 | 7.331538971 | CREBBP;EG… |
| 0 | 0 | 2.325657493 | 9.287621232 | MEF2A;NR… |
| 0 | 0 | 1.14544213 | 4.560578375 | ZNF296;GF… |
| 0 | 0 | 1.715161597 | 6.821087878 | CCR1;CCL1… |
| 0 | 0 | 1.509777385 | 5.995013765 | GALNT11;F… |
| 0 | 0 | 2.130596591 | 8.456971124 | ZBTB17;XB… |
| 0 | 0 | 9.817924528 | 38.94855912 | ANXA1;RSA… |
| 0 | 0 | 9.817924528 | 38.94855912 | PTPRC;LRP… |
| 0 | 0 | 9.817924528 | 38.94855912 | DAXX;ZC3H… |
| 0 | 0 | 9.817924528 | 38.94855912 | CYP24A1;V… |
| 0 | 0 | 9.817924528 | 38.94855912 | CXCL13;S1… |
| 0 | 0 | 9.817924528 | 38.94855912 | ISG20;TREX… |
| 0 | 0 | 9.817924528 | 38.94855912 | SLC2A10;S… |
| 0 | 0 | 9.817924528 | 38.94855912 | PNP;ADA2;… |
| 0 | 0 | 9.817924528 | 38.94855912 | DDX3X;EIF… |
| 0 | 0 | 9.817924528 | 38.94855912 | ADGRG3;D… |
| 0 | 0 | 9.817924528 | 38.94855912 | MPV17L;H… |
| 0 | 0 | 9.817924528 | 38.94855912 | HSPA5;DNA… |
| 0 | 0 | 9.817924528 | 38.94855912 | NR1H2;NR… |
| 0 | 0 | 9.817924528 | 38.94855912 | IKBKB;ROC… |
| 0 | 0 | 9.817924528 | 38.94855912 | STX1B;NR1… |
| 0 | 0 | 9.817924528 | 38.94855912 | NOS2;S100… |
| 0 | 0 | 9.817924528 | 38.94855912 | CD81;LGAL… |
| 0 | 0 | 9.817924528 | 38.94855912 | IL23A;IL23… |
| 0 | 0 | 9.817924528 | 38.94855912 | IL6;IL1B;TN… |
| 0 | 0 | 9.817924528 | 38.94855912 | IL1B;PTGS2… |
| 0 | 0 | 9.817924528 | 38.94855912 | GBP5;TLR9… |
| 0 | 0 | 9.817924528 | 38.94855912 | NOS2;CLEC… |
| 0 | 0 | 9.817924528 | 38.94855912 | SELP;SELE;… |
| 0 | 0 | 9.817924528 | 38.94855912 | CIITA;NLRP… |
| 0 | 0 | 9.817924528 | 38.94855912 | CSF1;IL12B… |
| 0 | 0 | 9.817924528 | 38.94855912 | TLR6;TLR4;… |
| 0 | 0 | 9.817924528 | 38.94855912 | MX2;MX1;… |
| 0 | 0 | 9.817924528 | 38.94855912 | MX2;MX1;… |

| | | | | | |
|---|---|---|---|---|---|
| 0 | 0 | 9.817924528 | 38.94855912 | IFNG;GTF2 | . |
| 0 | 0 | 9.817924528 | 38.94855912 | CLEC7A;PL | . |
| 0 | 0 | 9.817924528 | 38.94855912 | PTPRC;PTP | . |
| 0 | 0 | 9.817924528 | 38.94855912 | IL1B;PTGS2 | . |
| 0 | 0 | 9.817924528 | 38.94855912 | P2RY12;GP | . |
| 0 | 0 | 9.817924528 | 38.94855912 | PNPT1;ZC3 | . |
| 0 | 0 | 9.817924528 | 38.94855912 | NFKB1;RC3 | . |
| 0 | 0 | 9.817924528 | 38.94855912 | CSF1;IL12B | . |
| 0 | 0 | 9.817924528 | 38.94855912 | IL23A;IL18; | . |
| 0 | 0 | 9.817924528 | 38.94855912 | XIAP;BIRC2 | . |
| 0 | 0 | 9.817924528 | 38.94855912 | HIP1R;PTP | . |
| 0 | 0 | 9.817924528 | 38.94855912 | NRP1;PTPR | . |
| 0 | 0 | 9.817924528 | 38.94855912 | CAST;TCF7 | . |
| 0 | 0 | 9.817924528 | 38.94855912 | DAXX;ZC3H | . |
| 0 | 0 | 9.817924528 | 38.94855912 | PELI2;PELI1 | . |
| 0 | 0 | 9.817924528 | 38.94855912 | KYNU;IDO2 | . |
| 0 | 0 | 2.427096033 | 9.567332934 | CAST;IL10; | . |
| 0 | 0 | 2.427096033 | 9.567332934 | XKR8;ITGA | . |
| 0 | 0 | 2.427096033 | 9.567332934 | STAT5B;MA | . |
| 0 | 0 | 2.427096033 | 9.567332934 | LYN;STAT5 | . |
| 0 | 0 | 2.427096033 | 9.567332934 | SLC4A7;CL | . |
| 0 | 0 | 1.889714771 | 7.410593046 | PHLDB1;SM | . |
| 0 | 0 | 3.056790123 | 11.96941002 | ADAM17;H | . |
| 0 | 0 | 3.056790123 | 11.96941002 | ITGAM;IFN | . |
| 0 | 0 | 3.056790123 | 11.96941002 | ZFP36;PTP | . |
| 0 | 0 | 3.056790123 | 11.96941002 | CD74;RIPO | . |
| 0 | 0 | 3.056790123 | 11.96941002 | IL10;ROCK1 | . |
| 0 | 0 | 3.056790123 | 11.96941002 | TRIM8;CHM | . |
| 0 | 0 | 2.563901861 | 10.01284344 | ATXN3;PTP | . |
| 0 | 0 | 1.844722486 | 7.194036378 | DIRAS3;HD | . |
| 0 | 0 | 2.184904708 | 8.50523272 | SERPINB4; | . |
| 0 | 0 | 2.758412098 | 10.73506774 | CDKN1A;ID | . |
| 0 | 0 | 2.758412098 | 10.73506774 | VCAN;CHST | . |
| 0 | 0 | 2.758412098 | 10.73506774 | STAT5B;MA | . |
| 0 | 0 | 2.758412098 | 10.73506774 | IL1A;EDN1 | . |
| 0 | 0 | 2.758412098 | 10.73506774 | EGR2;DESI | . |
| 0 | 0 | 2.758412098 | 10.73506774 | ERO1A;RYR | . |
| 0 | 0 | 2.758412098 | 10.73506774 | IKBKB;MAP | . |
| 0 | 0 | 1.806268088 | 7.017770879 | ILRUN;CDK | . |
| 0 | 0 | 4.092475453 | 15.82222339 | ISG20;DFFB | . |
| 0 | 0 | 4.092475453 | 15.82222339 | SRC;CXCR4 | . |
| 0 | 0 | 4.092475453 | 15.82222339 | ANXA1;NO | . |
| 0 | 0 | 4.092475453 | 15.82222339 | MAP2K3;W | . |
| 0 | 0 | 4.092475453 | 15.82222339 | TRIM8;TRI | . |
| 0 | 0 | 4.092475453 | 15.82222339 | P2RX5;CLE | . |
| 0 | 0 | 4.092475453 | 15.82222339 | SOCS1;CD8 | . |
| 0 | 0 | 4.092475453 | 15.82222339 | ATF7IP;SET | . |

| | | | | |
|---|---|---|---|---|
| 0 | 0 | 4.092475453 | 15.82222339 | ABCA7;STX |
| 0 | 0 | 4.092475453 | 15.82222339 | PNP;AMPD |
| 0 | 0 | 4.092475453 | 15.82222339 | LYN;ELF1;F |
| 0 | 0 | 4.092475453 | 15.82222339 | LYN;CELA2 |
| 0 | 0 | 4.092475453 | 15.82222339 | SBNO2;SEL |
| 0 | 0 | 1.545077092 | 5.943308048 | NOTCH3;R |
| 0 | 0 | 1.360985146 | 5.225661232 | BTG2;CDKI |
| 0 | 0 | 2.252850587 | 8.610287025 | PRKAA1;XE |
| 0 | 0 | 2.252850587 | 8.610287025 | ITGAM;LAI |
| 0 | 0 | 2.252850587 | 8.610287025 | SRC;RNASE |
| 0 | 0 | 1.563186464 | 5.942980246 | PHLDB1;DI |
| 0 | 0 | 5.237297093 | 19.90199489 | RNF31;CD4 |
| 0 | 0 | 5.237297093 | 19.90199489 | UGCG;NR4 |
| 0 | 0 | 5.237297093 | 19.90199489 | PNP;AMPD |
| 0 | 0 | 5.237297093 | 19.90199489 | CD300A;LG |
| 0 | 0 | 5.237297093 | 19.90199489 | IL10;APOA |
| 0 | 0 | 5.237297093 | 19.90199489 | IL23A;IL23I |
| 0 | 0 | 5.237297093 | 19.90199489 | GALNT11;C |
| 0 | 0 | 5.237297093 | 19.90199489 | CXCL8;IL1E |
| 0 | 0 | 5.237297093 | 19.90199489 | SLC11A1;N |
| 0 | 0 | 5.237297093 | 19.90199489 | KLF7;MAFF |
| 0 | 0 | 5.237297093 | 19.90199489 | BCL2L11;B |
| 0 | 0 | 5.237297093 | 19.90199489 | IL6;IL1B;HE |
| 0 | 0 | 5.237297093 | 19.90199489 | CLN3;NR1H |
| 0 | 0 | 5.237297093 | 19.90199489 | IFNG;IL12E |
| 0 | 0 | 5.237297093 | 19.90199489 | CDKN2D;C |
| 0 | 0 | 5.237297093 | 19.90199489 | IL18;IL12B; |
| 0 | 0 | 2.340278904 | 8.797810875 | BCL2L12;P |
| 0 | 0 | 2.125730426 | 7.942637715 | HDAC4;MA |
| 0 | 0 | 1.928377113 | 7.187275328 | CDKN1B;U |
| 0 | 0 | 3.274461655 | 12.17313963 | RYR1;CASP |
| 0 | 0 | 3.274461655 | 12.17313963 | IFIH1;DDX5 |
| 0 | 0 | 3.274461655 | 12.17313963 | ELOVL5;AC |
| 0 | 0 | 3.274461655 | 12.17313963 | CD74;MDN |
| 0 | 0 | 3.274461655 | 12.17313963 | IL6;SMAD3 |
| 0 | 0 | 3.274461655 | 12.17313963 | NR4A3;ELC |
| 0 | 0 | 3.274461655 | 12.17313963 | SLC7A5;FC |
| 0 | 0 | 3.274461655 | 12.17313963 | ATXN3;XBF |
| 0 | 0 | 3.274461655 | 12.17313963 | FLCN;SOX1 |
| 0 | 0 | 3.274461655 | 12.17313963 | RAP1B;RIP |
| 0 | 0 | 3.274461655 | 12.17313963 | LYN;CELA2 |
| 0 | 0 | 3.274461655 | 12.17313963 | SMAD3;ITC |
| 0 | 0 | 3.274461655 | 12.17313963 | IFITM3;IFN |
| 0 | 0 | 1.624357641 | 6.025004084 | ACVRL1;CD |
| 0 | 0 | 2.456930787 | 9.111332942 | PFKFB4;PR |
| 0 | 0 | 2.456930787 | 9.111332942 | IL10;IL6;IL1 |
| 0 | 0 | 2.456930787 | 9.111332942 | PYCARD;DI |

| | | | | |
|---|---|---|---|---|
| 0 | 0 | 1.658329503 | 6.108244905 | ACVRL1;JU |
| 0 | 0 | 1.658329503 | 6.108244905 | PPP1R15A; |
| 0 | 0 | 1.658329503 | 6.108244905 | MAP2K3;R |
| 0 | 0 | 1.827545461 | 6.727970751 | TCF7L2;UR |
| 0 | 0 | 1.40404835 | 5.166423553 | AMER1;CD |
| 0 | 0 | 2.620340265 | 9.630790886 | P2RY12;FG |
| 0 | 0 | 2.620340265 | 9.630790886 | SMAD3;IFN |
| 0 | 0 | 2.620340265 | 9.630790886 | ANXA1;CLE |
| 0 | 0 | 2.620340265 | 9.630790886 | PTPN1;NDI |
| 0 | 0 | 2.865575397 | 10.52000108 | ADAM19;A |
| 0 | 0 | 2.865575397 | 10.52000108 | CD40;IL6;G |
| 0 | 0 | 2.865575397 | 10.52000108 | SLC7A5;FC |
| 0 | 0 | 2.18445622 | 7.990549593 | ZFYVE28;R |
| 0 | 0 | 2.18445622 | 7.990549593 | NRP1;PAK1 |
| 0 | 0 | 1.504715331 | 5.480795014 | DGKG;SLC4 |
| 0 | 0 | 1.463876147 | 5.292013734 | RET;NRP1; |
| 0 | 0 | 1.891183589 | 6.808400144 | SLC13A3;P |
| 0 | 0 | 2.069670579 | 7.422638305 | PREX1;GHS |
| 0 | 0 | 2.069670579 | 7.422638305 | RANBP2;SE |
| 0 | 0 | 2.259449163 | 8.101431523 | MAP4K1;P |
| 0 | 0 | 2.259449163 | 8.101431523 | RNF126;DT |
| 0 | 0 | 1.840475288 | 6.58762266 | VASP;TENN |
| 0 | 0 | 1.568110589 | 5.602951146 | NOTCH3;R |
| 0 | 0 | 1.703651457 | 6.067907973 | SDC4;CHPF |
| 0 | 0 | 1.7618171 | 6.272112974 | AMER1;PLI |
| 0 | 0 | 1.473244892 | 5.237793307 | NOTCH4;LF |
| 0 | 0 | 1.27185553 | 4.515255514 | RNF13;FBX |
| 0 | 0 | 3.637546156 | 12.86783113 | ERAP2;ERA |
| 0 | 0 | 3.637546156 | 12.86783113 | PGS1;PNPL |
| 0 | 0 | 3.637546156 | 12.86783113 | IL10;NFKBI |
| 0 | 0 | 3.637546156 | 12.86783113 | NOD1;CD1 |
| 0 | 0 | 3.637546156 | 12.86783113 | PARP10;NA |
| 0 | 0 | 3.637546156 | 12.86783113 | CD74;TIFAI |
| 0 | 0 | 3.637546156 | 12.86783113 | FGR;ITGAM |
| 0 | 0 | 3.637546156 | 12.86783113 | DUSP10;SC |
| 0 | 0 | 3.637546156 | 12.86783113 | ATF7IP;SET |
| 0 | 0 | 3.637546156 | 12.86783113 | NR1H2;NR |
| 0 | 0 | 3.637546156 | 12.86783113 | AGO2;ARH |
| 0 | 0 | 3.637546156 | 12.86783113 | RIPOR2;LR |
| 0 | 0 | 3.637546156 | 12.86783113 | P2RY12;NF |
| 0 | 0 | 3.637546156 | 12.86783113 | IL6;GPR18 |
| 0 | 0 | 3.637546156 | 12.86783113 | NRP1;PIK3 |
| 0 | 0 | 1.981606765 | 7.006696023 | NRP1;RAM |
| 0 | 0 | 2.358517398 | 8.307197131 | VMP1;CLN |
| 0 | 0 | 2.358517398 | 8.307197131 | CREBBP;GS |
| 0 | 0 | 2.358517398 | 8.307197131 | KCNJ12;PR |
| 0 | 0 | 2.120085051 | 7.422462532 | SPN;IL10;C |

| | | | | |
|---|---|---|---|---|
| 0 | 0 | 2.120085051 | 7.422462532 | PTPN1;PTF |
| 0 | 0 | 1.855393594 | 6.45139134 | SMAD3;DT |
| 0 | 0 | 1.855393594 | 6.45139134 | KMT2E;LYI |
| 0 | 0 | 2.495418129 | 8.665125958 | PREX1;PST |
| 0 | 0 | 2.495418129 | 8.665125958 | RAP1B;PPF |
| 0 | 0 | 2.495418129 | 8.665125958 | PPP1R16B; |
| 0 | 0 | 2.495418129 | 8.665125958 | PTPRC;RIPI |
| 0 | 0 | 1.659574468 | 5.761151336 | UPF2;RANI |
| 0 | 0 | 1.808638343 | 6.262297468 | CDKN1A;A |
| 0 | 0 | 1.808638343 | 6.262297468 | NRP1;ROCI |
| 0 | 0 | 1.706899242 | 5.898140741 | ACVRL1;PP |
| 0 | 0 | 1.76932341 | 6.112688712 | DDX3X;HSI |
| 0 | 0 | 3.022405626 | 10.43932124 | ATF7IP;KM |
| 0 | 0 | 3.022405626 | 10.43932124 | ATF7IP;KM |
| 0 | 0 | 3.022405626 | 10.43932124 | GGT5;ALO |
| 0 | 0 | 3.022405626 | 10.43932124 | MORC3;HS |
| 0 | 0 | 3.022405626 | 10.43932124 | KAT2B;ZC3 |
| 0 | 0 | 3.022405626 | 10.43932124 | CLEC7A;TN |
| 0 | 0 | 3.022405626 | 10.43932124 | CCR1;CXCL |
| 0 | 0 | 3.022405626 | 10.43932124 | ADAM19;N |
| 0 | 0 | 3.022405626 | 10.43932124 | KLF7;IFNG; |
| 0 | 0 | 3.022405626 | 10.43932124 | GRAMD1C |
| 0 | 0 | 1.434197051 | 4.942635931 | ISYNA1;PFI |
| 0 | 0 | 2.69685652 | 9.288909133 | SLC6A6;SL |
| 0 | 0 | 2.69685652 | 9.288909133 | CLEC7A;CL |
| 0 | 0 | 2.69685652 | 9.288909133 | LYN;BATF2 |
| 0 | 0 | 2.69685652 | 9.288909133 | LYN;FCER1 |
| 0 | 0 | 2.69685652 | 9.288909133 | ATAD1;SLC |
| 0 | 0 | 2.69685652 | 9.288909133 | PFKFB4;PR |
| 0 | 0 | 2.69685652 | 9.288909133 | NRP1;ROCI |
| 0 | 0 | 2.69685652 | 9.288909133 | GADD45B; |
| 0 | 0 | 2.69685652 | 9.288909133 | OTUB2;OT |
| 0 | 0 | 2.69685652 | 9.288909133 | NRP1;SEM |
| 0 | 0 | 2.69685652 | 9.288909133 | IL1A;LYPLA |
| 0 | 0 | 2.016485597 | 6.941357677 | NRP1;PAK |
| 0 | 0 | 1.590465323 | 5.45079457 | PPP1R16B; |
| 0 | 0 | 2.184008072 | 7.469704308 | CCL14;RET |
| 0 | 0 | 2.184008072 | 7.469704308 | CPB2;EDN |
| 0 | 0 | 1.540660287 | 5.254132488 | KDM5A;HD |
| 0 | 0 | 1.936458333 | 6.587597866 | SRGN;CCR |
| 0 | 0 | 4.364162577 | 14.83033461 | ANXA1;PN |
| 0 | 0 | 4.364162577 | 14.83033461 | ANXA1;PN |
| 0 | 0 | 4.364162577 | 14.83033461 | LYN;DOCK |
| 0 | 0 | 4.364162577 | 14.83033461 | TLR9;TLR4; |
| 0 | 0 | 4.364162577 | 14.83033461 | SLC25A37; |
| 0 | 0 | 4.364162577 | 14.83033461 | BATF2;DCS |
| 0 | 0 | 4.364162577 | 14.83033461 | MAD2L2;R |

| | | | | |
|---|---|---|---|---|
| 0 | 0 | 4.364162577 | 14.83033461 | RAB11FIP1 |
| 0 | 0 | 4.364162577 | 14.83033461 | PYCARD;RE |
| 0 | 0 | 4.364162577 | 14.83033461 | MAD2L2;H |
| 0 | 0 | 4.364162577 | 14.83033461 | PRKCB;IL1B |
| 0 | 0 | 4.364162577 | 14.83033461 | SOCS3;SOC |
| 0 | 0 | 4.364162577 | 14.83033461 | SEMA6A;IL |
| 0 | 0 | 4.364162577 | 14.83033461 | GPR35;FM |
| 0 | 0 | 4.364162577 | 14.83033461 | KAT2B;CRE |
| 0 | 0 | 4.364162577 | 14.83033461 | PNPLA8;EN |
| 0 | 0 | 4.364162577 | 14.83033461 | PYCARD;M |
| 0 | 0 | 4.364162577 | 14.83033461 | F12;SERPIN |
| 0 | 0 | 4.364162577 | 14.83033461 | F12;SERPIN |
| 0 | 0 | 4.364162577 | 14.83033461 | CD53;RIPO |
| 0 | 0 | 4.364162577 | 14.83033461 | XBP1;FICD; |
| 0 | 0 | 4.364162577 | 14.83033461 | TESC;RARA |
| 0 | 0 | 4.364162577 | 14.83033461 | LRRK2;STK |
| 0 | 0 | 4.364162577 | 14.83033461 | P2RY12;CS |
| 0 | 0 | 4.364162577 | 14.83033461 | IL23A;IL23 |
| 0 | 0 | 4.364162577 | 14.83033461 | HCK;GSN;F |
| 0 | 0 | 4.364162577 | 14.83033461 | EGR1;IFNG |
| 0 | 0 | 4.364162577 | 14.83033461 | DCSTAMP; |
| 0 | 0 | 6.54490566 | 22.10206724 | NF1;DLL1;S |
| 0 | 0 | 6.54490566 | 22.10206724 | DAXX;ZC3H |
| 0 | 0 | 6.54490566 | 22.10206724 | XBP1;DGAT |
| 0 | 0 | 6.54490566 | 22.10206724 | OVGP1;CH |
| 0 | 0 | 6.54490566 | 22.10206724 | OVGP1;CH |
| 0 | 0 | 6.54490566 | 22.10206724 | PAK1;FAP;I |
| 0 | 0 | 6.54490566 | 22.10206724 | IFIH1;DDX5 |
| 0 | 0 | 6.54490566 | 22.10206724 | NIPBL;ECE1 |
| 0 | 0 | 6.54490566 | 22.10206724 | SLC2A10;S |
| 0 | 0 | 6.54490566 | 22.10206724 | NINJ1;SLAM |
| 0 | 0 | 6.54490566 | 22.10206724 | ANXA2;FLC |
| 0 | 0 | 6.54490566 | 22.10206724 | DCSTAMP; |
| 0 | 0 | 6.54490566 | 22.10206724 | CD274;SOC |
| 0 | 0 | 6.54490566 | 22.10206724 | SMAD3;AT |
| 0 | 0 | 6.54490566 | 22.10206724 | SPRED2;SP |
| 0 | 0 | 6.54490566 | 22.10206724 | MAEA;IRF7 |
| 0 | 0 | 6.54490566 | 22.10206724 | CD300A;LG |
| 0 | 0 | 6.54490566 | 22.10206724 | GRK2;TIFA |
| 0 | 0 | 6.54490566 | 22.10206724 | NDFIP2;PP |
| 0 | 0 | 6.54490566 | 22.10206724 | GFI1;SNAI1 |
| 0 | 0 | 6.54490566 | 22.10206724 | JAML;PECA |
| 0 | 0 | 6.54490566 | 22.10206724 | SLC22A3;S |
| 0 | 0 | 6.54490566 | 22.10206724 | SLC11A1;N |
| 0 | 0 | 6.54490566 | 22.10206724 | MAPK8;PR |
| 0 | 0 | 6.54490566 | 22.10206724 | SLC11A1;N |
| 0 | 0 | 6.54490566 | 22.10206724 | IL1B;PTGS2 |

| | | | | |
|---|---|---|---|---|
| 0 | 0 | 6.54490566 | 22.10206724 | IQSEC1;AR |
| 0 | 0 | 6.54490566 | 22.10206724 | TESC;HCLS |
| 0 | 0 | 6.54490566 | 22.10206724 | SLC25A37; |
| 0 | 0 | 6.54490566 | 22.10206724 | P2RY12;CS |
| 0 | 0 | 6.54490566 | 22.10206724 | IL12B;IL12 |
| 0 | 0 | 6.54490566 | 22.10206724 | IL12B;IL12 |
| 0 | 0 | 6.54490566 | 22.10206724 | DOCK3;SR |
| 0 | 0 | 6.54490566 | 22.10206724 | CLEC3B;F1 |
| 0 | 0 | 6.54490566 | 22.10206724 | NRP1;ANX |
| 0 | 0 | 6.54490566 | 22.10206724 | NR1H2;NR |
| 0 | 0 | 6.54490566 | 22.10206724 | IL18;RARA; |
| 0 | 0 | 6.54490566 | 22.10206724 | FLOT1;PTP |
| 0 | 0 | 6.54490566 | 22.10206724 | VPS4B;CEP |
| 0 | 0 | 6.54490566 | 22.10206724 | IQSEC1;AR |
| 0 | 0 | 6.54490566 | 22.10206724 | EGR1;FFAR |
| 0 | 0 | 6.54490566 | 22.10206724 | IL10;SUPT |
| 0 | 0 | 6.54490566 | 22.10206724 | IRF7;NOD2 |
| 0 | 0 | 6.54490566 | 22.10206724 | ERBIN;PTP |
| 0 | 0 | 6.54490566 | 22.10206724 | FLCN;HES1 |
| 0 | 0 | 6.54490566 | 22.10206724 | NR4A1;NR |
| 0 | 0 | 6.54490566 | 22.10206724 | RYR1;CCL3 |
| 0 | 0 | 6.54490566 | 22.10206724 | INPP5K;AI |
| 0 | 0 | 6.54490566 | 22.10206724 | SYAP1;PRR |
| 0 | 0 | 2.059392235 | 6.900821999 | TCF7L2;SM |
| 0 | 0 | 1.777880617 | 5.954266843 | CREBBP;EG |
| 0 | 0 | 1.777880617 | 5.954266843 | CDKN1A;A |
| 0 | 0 | 1.710551935 | 5.727888762 | RTP4;ATAD |
| 0 | 0 | 1.741576963 | 5.827883618 | PPP1R16B; |
| 0 | 0 | 1.741576963 | 5.827883618 | P2RY12;P2 |
| 0 | 0 | 2.26767427 | 7.587514575 | CA12;SLC4 |
| 0 | 0 | 2.26767427 | 7.587514575 | CASP4;ATP |
| 0 | 0 | 2.26767427 | 7.587514575 | CREB1;ELO |
| 0 | 0 | 1.382905328 | 4.621498207 | AMER1;DR |
| 0 | 0 | 1.527290096 | 5.087823729 | SLC22A3;R |
| 0 | 0 | 1.965959864 | 6.495345129 | MAD2L2;E |
| 0 | 0 | 1.407120997 | 4.629036409 | DDX5;NFIX |
| 0 | 0 | 1.620337922 | 5.322402566 | XBP1;PKLR |
| 0 | 0 | 1.620337922 | 5.322402566 | HDAC4;RA |
| 0 | 0 | 2.381852552 | 7.816576561 | P2RY12;GS |
| 0 | 0 | 2.381852552 | 7.816576561 | VCAN;CHS |
| 0 | 0 | 2.381852552 | 7.816576561 | AEBP2;PHF |
| 0 | 0 | 2.381852552 | 7.816576561 | IL10;GHSR; |
| 0 | 0 | 2.381852552 | 7.816576561 | SNX18;ITG |
| 0 | 0 | 2.381852552 | 7.816576561 | P2RY12;FG |
| 0 | 0 | 2.381852552 | 7.816576561 | SMAD3;IF |
| 0 | 0 | 1.893316498 | 6.196395191 | TCF7L2;CS |
| 0 | 0 | 2.113434147 | 6.895330586 | ISG20;DFF |

| | | | | |
|---|---|---|---|---|
| 0 | 0 | 2.113434147 | 6.895330586 | FLCN;SEH1 . |
| 0 | 0 | 2.113434147 | 6.895330586 | IFITM3;HE) . |
| 0 | 0 | 3.273602719 | 10.62178509 | DDX17;DA) . |
| 0 | 0 | 3.273602719 | 10.62178509 | OCSTAMP; . |
| 0 | 0 | 3.273602719 | 10.62178509 | IFIH1;DDX5 . |
| 0 | 0 | 3.273602719 | 10.62178509 | CPB2;SERP . |
| 0 | 0 | 3.273602719 | 10.62178509 | MGAM;PH . |
| 0 | 0 | 3.273602719 | 10.62178509 | PHKG1;RB1 . |
| 0 | 0 | 3.273602719 | 10.62178509 | ABCA1;NP( . |
| 0 | 0 | 3.273602719 | 10.62178509 | DDX17;TCF . |
| 0 | 0 | 3.273602719 | 10.62178509 | MAD2L2;D . |
| 0 | 0 | 3.273602719 | 10.62178509 | CASP3;DDI . |
| 0 | 0 | 3.273602719 | 10.62178509 | SDCBP;SD( . |
| 0 | 0 | 3.273602719 | 10.62178509 | IL10;IL1B;F . |
| 0 | 0 | 3.273602719 | 10.62178509 | IFNG;IL1B;S . |
| 0 | 0 | 3.273602719 | 10.62178509 | USP15;DH) . |
| 0 | 0 | 3.273602719 | 10.62178509 | ITGA5;KLF4 . |
| 0 | 0 | 3.273602719 | 10.62178509 | NT5C3A;DI . |
| 0 | 0 | 3.273602719 | 10.62178509 | PPP1R16B; . |
| 0 | 0 | 3.273602719 | 10.62178509 | IL6;FAP;PD . |
| 0 | 0 | 3.273602719 | 10.62178509 | RAP1B;SEL . |
| 0 | 0 | 3.273602719 | 10.62178509 | BTG2;ZFP3 . |
| 0 | 0 | 3.273602719 | 10.62178509 | CXCL10;AD . |
| 0 | 0 | 3.273602719 | 10.62178509 | GPR65;GPI . |
| 0 | 0 | 3.273602719 | 10.62178509 | TNFSF14;D . |
| 0 | 0 | 1.787717968 | 5.797188797 | TCF7L2;CR . |
| 0 | 0 | 1.748148148 | 5.662468794 | PNPT1;FM . |
| 0 | 0 | 2.546884186 | 8.23531756 | GSN;TMSB . |
| 0 | 0 | 2.546884186 | 8.23531756 | CD300A;LA . |
| 0 | 0 | 2.546884186 | 8.23531756 | GGT5;NPEI . |
| 0 | 0 | 2.546884186 | 8.23531756 | PGS1;PNPL . |
| 0 | 0 | 2.546884186 | 8.23531756 | KLF7;ZFP36 . |
| 0 | 0 | 2.806357602 | 9.016068808 | PSEN1;BAK . |
| 0 | 0 | 2.806357602 | 9.016068808 | RARG;NIPE . |
| 0 | 0 | 2.806357602 | 9.016068808 | SLC2A9;SL( . |
| 0 | 0 | 2.806357602 | 9.016068808 | CDKN1A;C! . |
| 0 | 0 | 2.806357602 | 9.016068808 | ALAS1;NRF . |
| 0 | 0 | 2.806357602 | 9.016068808 | FCN1;IFITN . |
| 0 | 0 | 2.806357602 | 9.016068808 | IL6;F12;OS . |
| 0 | 0 | 2.806357602 | 9.016068808 | IL15;IL23A; . |
| 0 | 0 | 2.002071242 | 6.421057474 | INPP4A;ISY . |
| 0 | 0 | 2.002071242 | 6.421057474 | ILRUN;NFK . |
| 0 | 0 | 2.183560262 | 6.9414358 | IL1A;EDN1 . |
| 0 | 0 | 2.183560262 | 6.9414358 | SMAD3;IFN . |
| 0 | 0 | 1.9178988 | 6.08152081 | ISG20;DFFI . |
| 0 | 0 | 1.9178988 | 6.08152081 | TGM1;BCR . |
| 0 | 0 | 1.509457607 | 4.754280989 | OGFR;URI1 . |

| | | | | |
|---|---|---|---|---|
| 0 | 0 | 1.852050395 | 5.830896248 | GSN;MID1I |
| 0 | 0 | 1.852050395 | 5.830896248 | ANXA1;RIC |
| 0 | 0 | 1.799136631 | 5.641921774 | PLK3;BTG2 |
| 0 | 0 | 1.799136631 | 5.641921774 | MTMR3;RC |
| 0 | 0 | 1.799136631 | 5.641921774 | USP36;FUS |
| 0 | 0 | 1.719390515 | 5.385919274 | H2AW;CDK |
| 0 | 0 | 1.719390515 | 5.385919274 | SLC44A3;C |
| 0 | 0 | 1.755692895 | 5.497390435 | XBP1;ANXA |
| 0 | 0 | 1.755692895 | 5.497390435 | SERPINB10 |
| 0 | 0 | 1.755692895 | 5.497390435 | HDAC4;RA |
| 0 | 0 | 1.755692895 | 5.497390435 | PRKAA1;SE |
| 0 | 0 | 1.755692895 | 5.497390435 | ZNF143;US |
| 0 | 0 | 1.568299374 | 4.90641303 | RIPK3;SLC1 |
| 0 | 0 | 1.568299374 | 4.90641303 | HDAC4;USI |
| 0 | 0 | 2.047271093 | 6.372006957 | SDCBP;TCF |
| 0 | 0 | 2.278162242 | 7.0676673 | ABCA1;ATF |
| 0 | 0 | 2.278162242 | 7.0676673 | RBM3;SETX |
| 0 | 0 | 2.278162242 | 7.0676673 | PPP1R15A; |
| 0 | 0 | 1.947848681 | 5.979140969 | INPP4A;ISY |
| 0 | 0 | 3.740495066 | 11.42554993 | CD74;FCER |
| 0 | 0 | 3.740495066 | 11.42554993 | ATP6V1G1 |
| 0 | 0 | 3.740495066 | 11.42554993 | PRKAA1;XE |
| 0 | 0 | 3.740495066 | 11.42554993 | CLN3;LAMI |
| 0 | 0 | 3.740495066 | 11.42554993 | NRP1;PAK1 |
| 0 | 0 | 3.740495066 | 11.42554993 | CXCL8;VEG |
| 0 | 0 | 3.740495066 | 11.42554993 | ZFP36;EIF6 |
| 0 | 0 | 3.740495066 | 11.42554993 | ZFP36;EIF6 |
| 0 | 0 | 3.740495066 | 11.42554993 | IFNG;IL10R |
| 0 | 0 | 3.740495066 | 11.42554993 | PRKCD;TNF |
| 0 | 0 | 3.740495066 | 11.42554993 | LYN;ANXA1 |
| 0 | 0 | 3.740495066 | 11.42554993 | C3;ZC3H12 |
| 0 | 0 | 3.740495066 | 11.42554993 | PRKCD;TNF |
| 0 | 0 | 3.740495066 | 11.42554993 | HSPA5;DDI |
| 0 | 0 | 3.740495066 | 11.42554993 | CNEP1R1;N |
| 0 | 0 | 3.740495066 | 11.42554993 | AGO2;ABC |
| 0 | 0 | 3.740495066 | 11.42554993 | SRC;PDPN; |
| 0 | 0 | 3.740495066 | 11.42554993 | SPN;STK10 |
| 0 | 0 | 3.740495066 | 11.42554993 | RIPK3;IL1B |
| 0 | 0 | 3.740495066 | 11.42554993 | RSAD2;TLR |
| 0 | 0 | 3.740495066 | 11.42554993 | IL10;IL1RN |
| 0 | 0 | 3.740495066 | 11.42554993 | SYNJ1;KIF5 |
| 0 | 0 | 2.412698413 | 7.327579254 | RAP1B;PPF |
| 0 | 0 | 2.412698413 | 7.327579254 | NRP1;RIPC |
| 0 | 0 | 2.412698413 | 7.327579254 | TRPC6;SLC |
| 0 | 0 | 1.691560547 | 5.12370542 | SEMA6A;N |
| 0 | 0 | 1.724791509 | 5.214227565 | CDKN1A;PI |
| 0 | 0 | 2.105454398 | 6.359793915 | CDKN2D;PI |

| | | | | |
|---|---|---|---|---|
| 0 | 0 | 2.61911598 | 7.834392797 | BCL2A1;CA |
| 0 | 0 | 2.61911598 | 7.834392797 | PYCARD;IF |
| 0 | 0 | 2.61911598 | 7.834392797 | CASP3;DDI |
| 0 | 0 | 2.61911598 | 7.834392797 | PFKFB4;PR |
| 0 | 0 | 2.61911598 | 7.834392797 | ANXA1;RSA |
| 0 | 0 | 2.61911598 | 7.834392797 | C3;ZC3H12 |
| 0 | 0 | 2.61911598 | 7.834392797 | PFKFB4;PR |
| 0 | 0 | 2.61911598 | 7.834392797 | TENM1;ZN |
| 0 | 0 | 2.975830816 | 8.874193995 | HAAO;KYN |
| 0 | 0 | 2.975830816 | 8.874193995 | GPR183;CD |
| 0 | 0 | 2.975830816 | 8.874193995 | SOCS3;SOC |
| 0 | 0 | 2.975830816 | 8.874193995 | CD40;RGC( |
| 0 | 0 | 2.975830816 | 8.874193995 | CD53;RIPO |
| 0 | 0 | 2.975830816 | 8.874193995 | ROCK1;IFN |
| 0 | 0 | 2.975830816 | 8.874193995 | ABCA1;NR1 |
| 0 | 0 | 2.975830816 | 8.874193995 | ILRUN;HER |
| 0 | 0 | 2.975830816 | 8.874193995 | CDKN2D;N |
| 0 | 0 | 2.975830816 | 8.874193995 | LYN;FGR;C |
| 0 | 0 | 1.896479939 | 5.571511173 | RBM3;NFK |
| 0 | 0 | 2.183112791 | 6.40406593 | PIK3CA;SR( |
| 0 | 0 | 2.183112791 | 6.40406593 | MOV10;ZF |
| 0 | 0 | 2.183112791 | 6.40406593 | FLT1;PTPR( |
| 0 | 0 | 2.183112791 | 6.40406593 | SEMA4A;SI |
| 0 | 0 | 2.183112791 | 6.40406593 | IL10;IL6;IL1 |
| 0 | 0 | 1.66461407 | 4.874979458 | PLK3;LRRK |
| 0 | 0 | 1.828482869 | 5.339696473 | IVNS1ABP; |
| 0 | 0 | 4.908396226 | 14.3304437 | STAT3;DLL |
| 0 | 0 | 4.908396226 | 14.3304437 | VMP1;CAL |
| 0 | 0 | 4.908396226 | 14.3304437 | SLC22A3;S |
| 0 | 0 | 4.908396226 | 14.3304437 | KLF4;ASS1; |
| 0 | 0 | 4.908396226 | 14.3304437 | HYAL1;HAS |
| 0 | 0 | 4.908396226 | 14.3304437 | LY96;LBP;T |
| 0 | 0 | 4.908396226 | 14.3304437 | P2RY12;CC |
| 0 | 0 | 4.908396226 | 14.3304437 | HYAL1;HAS |
| 0 | 0 | 4.908396226 | 14.3304437 | ARHGEF2;I |
| 0 | 0 | 4.908396226 | 14.3304437 | C5AR1;SUF |
| 0 | 0 | 4.908396226 | 14.3304437 | DAAM2;NF |
| 0 | 0 | 4.908396226 | 14.3304437 | ANXA1;TBX |
| 0 | 0 | 4.908396226 | 14.3304437 | CD81;PRK( |
| 0 | 0 | 4.908396226 | 14.3304437 | IL6;IL1B;TN |
| 0 | 0 | 4.908396226 | 14.3304437 | ZNF304;JA |
| 0 | 0 | 4.908396226 | 14.3304437 | IL1B;ACOD |
| 0 | 0 | 4.908396226 | 14.3304437 | PTPN1;BCL |
| 0 | 0 | 4.908396226 | 14.3304437 | CD180;LY8 |
| 0 | 0 | 4.908396226 | 14.3304437 | IL10;FNIP1 |
| 0 | 0 | 4.908396226 | 14.3304437 | TNIP1;ZC3I |
| 0 | 0 | 4.908396226 | 14.3304437 | CAMK1D;C |

| | | | | |
|---|---|---|---|---|
| 0 | 0 | 4.908396226 | 14.3304437 | AGO2;ACV |
| 0 | 0 | 4.908396226 | 14.3304437 | HSPA5;SEC |
| 0 | 0 | 4.908396226 | 14.3304437 | CLN3;LAMI |
| 0 | 0 | 4.908396226 | 14.3304437 | PRKCD;CCL |
| 0 | 0 | 4.908396226 | 14.3304437 | SLC25A37; |
| 0 | 0 | 4.908396226 | 14.3304437 | KDM5A;PR |
| 0 | 0 | 4.908396226 | 14.3304437 | PLCG2;SIRI |
| 0 | 0 | 4.908396226 | 14.3304437 | PLEK;PTK2 |
| 0 | 0 | 4.908396226 | 14.3304437 | SPRED2;SP |
| 0 | 0 | 4.908396226 | 14.3304437 | CIITA;NLRF |
| 0 | 0 | 4.908396226 | 14.3304437 | P2RY12;CS |
| 0 | 0 | 4.908396226 | 14.3304437 | IKBKB;ROC |
| 0 | 0 | 4.908396226 | 14.3304437 | CCL4;CCL3 |
| 0 | 0 | 4.908396226 | 14.3304437 | IL12B;IL12 |
| 0 | 0 | 4.908396226 | 14.3304437 | DOCK3;SR |
| 0 | 0 | 4.908396226 | 14.3304437 | DHX8;TFIP |
| 0 | 0 | 4.908396226 | 14.3304437 | P2RY12;NF |
| 0 | 0 | 4.908396226 | 14.3304437 | FAS;FASLG |
| 0 | 0 | 4.908396226 | 14.3304437 | RAMP2;HE |
| 0 | 0 | 1.731044492 | 5.042117192 | CDKN1B;H |
| 0 | 0 | 1.331300903 | 3.864009429 | NLGN3;CD |
| 0 | 0 | 2.291931217 | 6.540827137 | IL6;HP;LBP |
| 0 | 0 | 2.291931217 | 6.540827137 | ENPP2;AP |
| 0 | 0 | 2.291931217 | 6.540827137 | PTPN1;DA |
| 0 | 0 | 2.291931217 | 6.540827137 | ID2;USP2; |
| 0 | 0 | 2.291931217 | 6.540827137 | SLC7A5;SL |
| 0 | 0 | 2.291931217 | 6.540827137 | IL10;RAMP |
| 0 | 0 | 2.291931217 | 6.540827137 | DDX5;ZFP3 |
| 0 | 0 | 2.291931217 | 6.540827137 | KLHL18;PP |
| 0 | 0 | 2.291931217 | 6.540827137 | ZFYVE28;A |
| 0 | 0 | 2.291931217 | 6.540827137 | IL10;IL1RN |
| 0 | 0 | 1.614908403 | 4.599789298 | TCF7L2;CR |
| 0 | 0 | 1.614908403 | 4.599789298 | EGR1;DAX |
| 0 | 0 | 1.66613125 | 4.693642913 | CDKN1A;R |
| 0 | 0 | 1.847745492 | 5.195009771 | STEAP3;AT |
| 0 | 0 | 1.698889871 | 4.768394953 | NRROS;SM |
| 0 | 0 | 1.502605156 | 4.214230283 | ABCA1;PT |
| 0 | 0 | 1.556914389 | 4.364667786 | CDKN2D;C |
| 0 | 0 | 1.786823173 | 5.007001008 | PLK3;BTG2 |
| 0 | 0 | 1.786823173 | 5.007001008 | AMER1;FZI |
| 0 | 0 | 2.455279562 | 6.843602553 | GCLC;MTH |
| 0 | 0 | 2.455279562 | 6.843602553 | CCR1;SRGN |
| 0 | 0 | 2.455279562 | 6.843602553 | LAPTM5;TI |
| 0 | 0 | 2.455279562 | 6.843602553 | FOSL1;SET |
| 0 | 0 | 2.455279562 | 6.843602553 | HEXIM1;SF |
| 0 | 0 | 2.455279562 | 6.843602553 | IFIH1;ELF1 |
| 0 | 0 | 1.512976385 | 4.199201517 | USP36;ATF |

| | | | | |
|---|---|---|---|---|
| 0 | 0 | 1.478546932 | 4.080017399 | RTP4;NCF1 |
| 0 | 0 | 1.478546932 | 4.080017399 | CELA2A;RS |
| 0 | 0 | 1.591746152 | 4.388498427 | HSPA5;RHI |
| 0 | 0 | 3.272744432 | 9.022160169 | PXN;STAT3 |
| 0 | 0 | 3.272744432 | 9.022160169 | SYNJ1;INPI |
| 0 | 0 | 3.272744432 | 9.022160169 | IL10;LYN;C |
| 0 | 0 | 3.272744432 | 9.022160169 | SEMA6A;IL |
| 0 | 0 | 3.272744432 | 9.022160169 | GRK2;ZC3I |
| 0 | 0 | 3.272744432 | 9.022160169 | DUSP10;PT |
| 0 | 0 | 3.272744432 | 9.022160169 | IL6;STAT3; |
| 0 | 0 | 3.272744432 | 9.022160169 | ABCA1;APC |
| 0 | 0 | 3.272744432 | 9.022160169 | ARG2;CGA |
| 0 | 0 | 3.272744432 | 9.022160169 | PTPN1;XBI |
| 0 | 0 | 3.272744432 | 9.022160169 | ROCK1;FLC |
| 0 | 0 | 3.272744432 | 9.022160169 | IL10;LYN;B |
| 0 | 0 | 3.272744432 | 9.022160169 | CLEC3B;F1 |
| 0 | 0 | 3.272744432 | 9.022160169 | CASP8;TNI |
| 0 | 0 | 1.524567431 | 4.188100251 | SRGN;C1R; |
| 0 | 0 | 2.727687563 | 7.488437037 | PXK;PPIF;T |
| 0 | 0 | 2.727687563 | 7.488437037 | CD74;MDN |
| 0 | 0 | 2.727687563 | 7.488437037 | F12;SERPIN |
| 0 | 0 | 2.727687563 | 7.488437037 | TNIP2;CCL |
| 0 | 0 | 2.727687563 | 7.488437037 | MEF2A;ED |
| 0 | 0 | 2.727687563 | 7.488437037 | CXCL8;VEG |
| 0 | 0 | 2.727687563 | 7.488437037 | IL10;MAPK |
| 0 | 0 | 2.727687563 | 7.488437037 | PNPT1;NO |
| 0 | 0 | 2.727687563 | 7.488437037 | HM13;RHE |
| 0 | 0 | 2.727687563 | 7.488437037 | ACVRL1;PI |
| 0 | 0 | 1.964863843 | 5.359794177 | SPN;IL10;L |
| 0 | 0 | 1.964863843 | 5.359794177 | MIDN;RAB |
| 0 | 0 | 1.964863843 | 5.359794177 | FCER2;IL1A |
| 0 | 0 | 1.964863843 | 5.359794177 | XBP1;IFNG |
| 0 | 0 | 1.398625247 | 3.765923807 | AMER1;DR |
| 0 | 0 | 1.398625247 | 3.765923807 | RET;RETRE |
| 0 | 0 | 1.801447767 | 4.84682774 | TIAM2;PAK |
| 0 | 0 | 1.801447767 | 4.84682774 | NRP1;PDPI |
| 0 | 0 | 1.74701502 | 4.697038825 | GGT5;PNPI |
| 0 | 0 | 1.74701502 | 4.697038825 | BST1;CDKN |
| 0 | 0 | 2.182665659 | 5.855287122 | LRRK2;FYN |
| 0 | 0 | 2.182665659 | 5.855287122 | ID2;USP2;E |
| 0 | 0 | 1.443713555 | 3.823805719 | ACVRL1;DI |
| 0 | 0 | 1.588673998 | 4.202115993 | VASP;NRP1 |
| 0 | 0 | 1.250009949 | 3.287304118 | STEAP3;SY |
| 0 | 0 | 2.014948379 | 5.287369785 | XBP1;SESN |
| 0 | 0 | 2.014948379 | 5.287369785 | PPP1R15A; |
| 0 | 0 | 1.638025767 | 4.267563095 | GBP6;GSDI |
| 0 | 0 | 1.638025767 | 4.267563095 | ZNF143;CC |

| | | | | |
|---|---|---|---|---|
| 0 | 0 | 2.310718016 | 6.005717866 | DGKG;PGS |
| 0 | 0 | 2.310718016 | 6.005717866 | ATF7IP;KM |
| 0 | 0 | 2.310718016 | 6.005717866 | SLC2A9;SL |
| 0 | 0 | 2.310718016 | 6.005717866 | HYAL1;HAS |
| 0 | 0 | 2.310718016 | 6.005717866 | RGS2;CXCL |
| 0 | 0 | 2.310718016 | 6.005717866 | GOLGA7B; |
| 0 | 0 | 2.310718016 | 6.005717866 | GOLGA7B; |
| 0 | 0 | 2.310718016 | 6.005717866 | NFKBIA;AB |
| 0 | 0 | 2.310718016 | 6.005717866 | LYN;STK10 |
| 0 | 0 | 1.251908004 | 3.252721538 | MRPS15;SI |
| 0 | 0 | 1.669994058 | 4.325517791 | OSBPL2;DY |
| 0 | 0 | 1.708937655 | 4.408000478 | VMP1;GAE |
| 0 | 0 | 1.708937655 | 4.408000478 | PYCARD;B |
| 0 | 0 | 1.708937655 | 4.408000478 | KLHL18;KN |
| 0 | 0 | 1.81937613 | 4.68981807 | SDCBP;DYN |
| 0 | 0 | 1.81937613 | 4.68981807 | PARP10;CD |
| 0 | 0 | 1.757408467 | 4.524459868 | GFI1;MPV1 |
| 0 | 0 | 1.757408467 | 4.524459868 | CLN3;IFNG |
| 0 | 0 | 1.757408467 | 4.524459868 | ACVRL1;NF |
| 0 | 0 | 1.500379651 | 3.838716696 | NIBAN1;AN |
| 0 | 0 | 1.500379651 | 3.838716696 | HACE1;CDI |
| 0 | 0 | 1.564871886 | 3.999984429 | P2RY12;NF |
| 0 | 0 | 3.926490566 | 10.0161581 | ANXA1;RSA |
| 0 | 0 | 3.926490566 | 10.0161581 | CLN3;SLC6 |
| 0 | 0 | 3.926490566 | 10.0161581 | KYNU;KYAT |
| 0 | 0 | 3.926490566 | 10.0161581 | PRKCD;NFK |
| 0 | 0 | 3.926490566 | 10.0161581 | CDKN1A;H |
| 0 | 0 | 3.926490566 | 10.0161581 | SLC22A3;S |
| 0 | 0 | 3.926490566 | 10.0161581 | MX2;MX1; |
| 0 | 0 | 3.926490566 | 10.0161581 | MX2;MX1; |
| 0 | 0 | 3.926490566 | 10.0161581 | IFNG;IFNG |
| 0 | 0 | 3.926490566 | 10.0161581 | CD274;LGA |
| 0 | 0 | 3.926490566 | 10.0161581 | PRKCD;CCL |
| 0 | 0 | 3.926490566 | 10.0161581 | CYP27B1;G |
| 0 | 0 | 3.926490566 | 10.0161581 | PTPN1;DUS |
| 0 | 0 | 3.926490566 | 10.0161581 | CLEC7A;DD |
| 0 | 0 | 3.926490566 | 10.0161581 | FAP;DLC1; |
| 0 | 0 | 3.926490566 | 10.0161581 | PRKCE;PTK |
| 0 | 0 | 3.926490566 | 10.0161581 | PDPN;PLAU |
| 0 | 0 | 3.926490566 | 10.0161581 | CLEC7A;IL1 |
| 0 | 0 | 3.926490566 | 10.0161581 | PNP;AMPD |
| 0 | 0 | 3.926490566 | 10.0161581 | IRF1;PTPN |
| 0 | 0 | 3.926490566 | 10.0161581 | IL10;DDX3 |
| 0 | 0 | 3.926490566 | 10.0161581 | LYN;CD274 |
| 0 | 0 | 3.926490566 | 10.0161581 | SIRPA;CD4 |
| 0 | 0 | 3.926490566 | 10.0161581 | KAT2B;IFN |
| 0 | 0 | 3.926490566 | 10.0161581 | IL1B;ACTN |

| | | | | |
|---|---|---|---|---|
| 0 | 0 | 3.926490566 | 10.0161581 | DDX3X;DD |
| 0 | 0 | 3.926490566 | 10.0161581 | CDKN1A;M |
| 0 | 0 | 3.926490566 | 10.0161581 | DDX39B;A |
| 0 | 0 | 3.926490566 | 10.0161581 | IL15;JAK2;J |
| 0 | 0 | 3.926490566 | 10.0161581 | ACTA2;ED |
| 0 | 0 | 3.926490566 | 10.0161581 | NRG1;RBP |
| 0 | 0 | 3.926490566 | 10.0161581 | DDX39B;A |
| 0 | 0 | 3.926490566 | 10.0161581 | ACVRL1;A |
| 0 | 0 | 1.366358495 | 3.479828355 | SLC22A3;K |
| 0 | 0 | 2.517720195 | 6.372154673 | ZFP36;MA |
| 0 | 0 | 2.517720195 | 6.372154673 | ADAM19;C |
| 0 | 0 | 2.517720195 | 6.372154673 | CLN3;SLC2 |
| 0 | 0 | 2.517720195 | 6.372154673 | DDX17;DD |
| 0 | 0 | 2.517720195 | 6.372154673 | PARP10;M |
| 0 | 0 | 2.517720195 | 6.372154673 | PRKAA1;IL |
| 0 | 0 | 2.517720195 | 6.372154673 | RIPOR2;FL |
| 0 | 0 | 2.517720195 | 6.372154673 | MEF2A;ED |
| 0 | 0 | 2.517720195 | 6.372154673 | KLHL18;PL |
| 0 | 0 | 2.517720195 | 6.372154673 | CRTC2;CA |
| 0 | 0 | 2.517720195 | 6.372154673 | EGR2;ID2; |
| 0 | 0 | 2.517720195 | 6.372154673 | PPP1R15A; |
| 0 | 0 | 2.517720195 | 6.372154673 | LYN;RNF13 |
| 0 | 0 | 2.517720195 | 6.372154673 | XBP1;PPP2 |
| 0 | 0 | 2.517720195 | 6.372154673 | DDX5;IL6; |
| 0 | 0 | 2.517720195 | 6.372154673 | CLN3;IRAK |
| 0 | 0 | 2.517720195 | 6.372154673 | CXCL8;SET |
| 0 | 0 | 1.512392351 | 3.823352118 | NRP1;PTP |
| 0 | 0 | 2.083333333 | 5.255065375 | MAD2L2;S |
| 0 | 0 | 2.083333333 | 5.255065375 | ZFP36;CDK |
| 0 | 0 | 2.083333333 | 5.255065375 | MAD2L2;P |
| 0 | 0 | 2.083333333 | 5.255065375 | MAPK8;M |
| 0 | 0 | 1.609195402 | 4.038425642 | ABCA1;XB |
| 0 | 0 | 1.609195402 | 4.038425642 | PRKAA1;D |
| 0 | 0 | 1.526066893 | 3.812709711 | GSDMD;P |
| 0 | 0 | 2.908938383 | 7.260762061 | AMPD1;A |
| 0 | 0 | 2.908938383 | 7.260762061 | CLEC7A;CL |
| 0 | 0 | 2.908938383 | 7.260762061 | SLC22A3;M |
| 0 | 0 | 2.908938383 | 7.260762061 | ATP1A3;A |
| 0 | 0 | 2.908938383 | 7.260762061 | ABCA1;NP |
| 0 | 0 | 2.908938383 | 7.260762061 | JUN;CCL4; |
| 0 | 0 | 2.908938383 | 7.260762061 | CYP27B1;G |
| 0 | 0 | 2.908938383 | 7.260762061 | CD274;TA |
| 0 | 0 | 2.908938383 | 7.260762061 | PHF1;BCO |
| 0 | 0 | 2.908938383 | 7.260762061 | IL10;NR4A |
| 0 | 0 | 2.908938383 | 7.260762061 | CD74;MAR |
| 0 | 0 | 2.908938383 | 7.260762061 | PTPN1;HH |
| 0 | 0 | 2.908938383 | 7.260762061 | ARG2;CGA |

| | | | | |
|---|---|---|---|---|
| 0 | 0 | 2.908938383 | 7.260762061 | ELOVL5;MI… |
| 0 | 0 | 2.908938383 | 7.260762061 | BTG2;ZFP3… |
| 0 | 0 | 2.908938383 | 7.260762061 | SEC16B;CD… |
| 0 | 0 | 2.908938383 | 7.260762061 | PLCG2;RAE… |
| 0 | 0 | 2.908938383 | 7.260762061 | TRPC6;NR1… |
| 0 | 0 | 2.908938383 | 7.260762061 | PNP;GDA;[… |
| 0 | 0 | 2.908938383 | 7.260762061 | FAP;F12;F1… |
| 0 | 0 | 2.908938383 | 7.260762061 | NFAT5;IL6;… |
| 0 | 0 | 2.908938383 | 7.260762061 | TASL;CLN3… |
| 0 | 0 | 2.908938383 | 7.260762061 | HP;SLAMF… |
| 0 | 0 | 2.908938383 | 7.260762061 | SLC9A3R1;… |
| 0 | 0 | 2.908938383 | 7.260762061 | SEC16B;CD… |
| 0 | 0 | 2.908938383 | 7.260762061 | IL10;ADCY… |
| 0 | 0 | 2.908938383 | 7.260762061 | NRP1;PLXN… |
| 0 | 0 | 2.908938383 | 7.260762061 | SYNJ1;KIF5… |
| 0 | 0 | 1.94020864 | 4.816130324 | CDKN2D;C… |
| 0 | 0 | 1.94020864 | 4.816130324 | C3;ASAH1;… |
| 0 | 0 | 1.94020864 | 4.816130324 | FLCN;SEH1… |
| 0 | 0 | 1.94020864 | 4.816130324 | IFIH1;DDX… |
| 0 | 0 | 1.94020864 | 4.816130324 | NRP1;SEM… |
| 0 | 0 | 1.482432115 | 3.677997074 | KDM5A;KD… |
| 0 | 0 | 1.672480605 | 4.138248417 | ABCA1;ATF… |
| 0 | 0 | 1.770101543 | 4.352341639 | HDAC4;ELK… |
| 0 | 0 | 1.770101543 | 4.352341639 | ILRUN;FLC… |
| 0 | 0 | 1.770101543 | 4.352341639 | ANXA1;CLE… |
| 0 | 0 | 2.182218864 | 5.291791954 | RYBP;DTX3… |
| 0 | 0 | 2.182218864 | 5.291791954 | ABCA1;ATF… |
| 0 | 0 | 2.182218864 | 5.291791954 | GPAT4;NO… |
| 0 | 0 | 2.182218864 | 5.291791954 | APOA2;EN… |
| 0 | 0 | 2.182218864 | 5.291791954 | SETX;PLK3;… |
| 0 | 0 | 2.182218864 | 5.291791954 | MEF2A;ED… |
| 0 | 0 | 2.182218864 | 5.291791954 | ACVRL1;AD… |
| 0 | 0 | 1.581359188 | 3.822252998 | ABCA1;XBF… |
| 0 | 0 | 1.505357216 | 3.638482189 | VASP;TENN… |
| 0 | 0 | 1.267361568 | 3.0543108 | NRP1;GAL… |
| 0 | 0 | 1.992638601 | 4.727262304 | NRP1;NF1;… |
| 0 | 0 | 1.992638601 | 4.727262304 | PIK3CA;PR… |
| 0 | 0 | 1.992638601 | 4.727262304 | PNPLA8;NF… |
| 0 | 0 | 1.992638601 | 4.727262304 | NRP1;SDC… |
| 0 | 0 | 1.992638601 | 4.727262304 | NCR1;LAM… |
| 0 | 0 | 1.535520517 | 3.61803017 | SMURF1;LI… |
| 0 | 0 | 1.870807453 | 4.393221679 | PYCARD;DI… |
| 0 | 0 | 1.870807453 | 4.393221679 | GABARAPL… |
| 0 | 0 | 1.870807453 | 4.393221679 | P2RY12;ST… |
| 0 | 0 | 1.870807453 | 4.393221679 | NRP1;TESK… |
| 0 | 0 | 1.870807453 | 4.393221679 | RGS2;ZC3H… |
| 0 | 0 | 1.723420356 | 4.04180892 | GRAMD1C… |

| | | | | |
|---|---|---|---|---|
| 0 | 0 | 1.723420356 | 4.04180892 | USP36;SUN… |
| 0 | 0 | 1.395919589 | 3.2714992 | PLK3;CREB… |
| 0 | 0 | 1.785930408 | 4.181367682 | SELP;TENM… |
| 0 | 0 | 1.785930408 | 4.181367682 | KCNK9;KCN… |
| 0 | 0 | 1.785930408 | 4.181367682 | NUB1;DESI… |
| 0 | 0 | 2.337748166 | 5.46091791 | GSN;PIK3C… |
| 0 | 0 | 2.337748166 | 5.46091791 | USP36;USF… |
| 0 | 0 | 2.337748166 | 5.46091791 | PYCARD;ZC… |
| 0 | 0 | 2.337748166 | 5.46091791 | IL6;IL1B;TN… |
| 0 | 0 | 2.337748166 | 5.46091791 | LAPTM5;PT… |
| 0 | 0 | 2.337748166 | 5.46091791 | CLN3;TIFAI… |
| 0 | 0 | 2.337748166 | 5.46091791 | NRP1;ROCI… |
| 0 | 0 | 2.337748166 | 5.46091791 | NR1H2;CN… |
| 0 | 0 | 2.337748166 | 5.46091791 | HACE1;RHO… |
| 0 | 0 | 2.337748166 | 5.46091791 | IKBKB;ROC… |
| 0 | 0 | 2.337748166 | 5.46091791 | SDCBP;SDC… |
| 0 | 0 | 2.337748166 | 5.46091791 | DAAM2;NF… |
| 0 | 0 | 2.337748166 | 5.46091791 | RAP1B;FM… |
| 0 | 0 | 2.337748166 | 5.46091791 | SEMA6A;IL… |
| 0 | 0 | 1.554466574 | 3.618217023 | NLGN3;SLC… |
| 0 | 0 | 1.554466574 | 3.618217023 | HSPA5;RHE… |
| 0 | 0 | 1.351232246 | 3.12758833 | SLC44A3;A… |
| 0 | 0 | 1.497545913 | 3.451708072 | FMR1;LRRI… |
| 0 | 0 | 1.576943042 | 3.628090918 | GALNT11;F… |
| 0 | 0 | 1.424643736 | 3.273461292 | MTMR3;PL… |
| 0 | 0 | 1.339951338 | 3.077276923 | PTGFR;RAN… |
| 0 | 0 | 1.456124816 | 3.344052775 | UPF2;RANI… |
| 0 | 0 | 1.456124816 | 3.344052775 | CA12;SLC2… |
| 0 | 0 | 1.604030632 | 3.650799473 | FOSL1;SET… |
| 0 | 0 | 1.511809646 | 3.435690819 | IL10;HEXIN… |
| 0 | 0 | 1.511809646 | 3.435690819 | PRKCE;RHO… |
| 0 | 0 | 2.617893545 | 5.931718959 | ABCA1;FCE… |
| 0 | 0 | 2.617893545 | 5.931718959 | PM20D2;M… |
| 0 | 0 | 2.617893545 | 5.931718959 | SETDB1;SN… |
| 0 | 0 | 2.617893545 | 5.931718959 | SOX13;PIM… |
| 0 | 0 | 2.617893545 | 5.931718959 | MAPK8;NC… |
| 0 | 0 | 2.617893545 | 5.931718959 | TESC;CHP1… |
| 0 | 0 | 2.617893545 | 5.931718959 | ADAM17;C… |
| 0 | 0 | 2.617893545 | 5.931718959 | CD81;PRKD… |
| 0 | 0 | 2.617893545 | 5.931718959 | C3;NR4A3;… |
| 0 | 0 | 2.617893545 | 5.931718959 | CD74;MAR… |
| 0 | 0 | 2.617893545 | 5.931718959 | CTBP2;CAL… |
| 0 | 0 | 2.617893545 | 5.931718959 | SLC9A3R1;… |
| 0 | 0 | 2.617893545 | 5.931718959 | ACTA2;RO… |
| 0 | 0 | 2.067245939 | 4.679428466 | VPS4B;TMI… |
| 0 | 0 | 2.067245939 | 4.679428466 | HK3;PKLR;… |
| 0 | 0 | 2.067245939 | 4.679428466 | HDAC4;PLE… |

| | | | | |
|---|---|---|---|---|
| 0 | 0 | 2.067245939 | 4.679428466 | CD300A;NI |
| 0 | 0 | 2.067245939 | 4.679428466 | USP25;XBF |
| 0 | 0 | 2.067245939 | 4.679428466 | ERO1A;RYF |
| 0 | 0 | 2.067245939 | 4.679428466 | PTPN1;NLF |
| 0 | 0 | 2.067245939 | 4.679428466 | SLC25A19; |
| 0 | 0 | 2.067245939 | 4.679428466 | BCAR3;DGI |
| 0 | 0 | 2.067245939 | 4.679428466 | RIPK2;PRK |
| 0 | 0 | 2.067245939 | 4.679428466 | NR1H2;AP |
| 0 | 0 | 2.067245939 | 4.679428466 | HDAC4;XBI |
| 0 | 0 | 1.465572838 | 3.313905425 | ACVRL1;TC |
| 0 | 0 | 3.271886792 | 7.348395267 | GSN;PIK3C |
| 0 | 0 | 3.271886792 | 7.348395267 | SRC;RAB3D |
| 0 | 0 | 3.271886792 | 7.348395267 | OVGP1;CH |
| 0 | 0 | 3.271886792 | 7.348395267 | GGT5;GGT |
| 0 | 0 | 3.271886792 | 7.348395267 | GGT5;GGT |
| 0 | 0 | 3.271886792 | 7.348395267 | CD274;CD |
| 0 | 0 | 3.271886792 | 7.348395267 | IL6;TMEM |
| 0 | 0 | 3.271886792 | 7.348395267 | DAAM2;NF |
| 0 | 0 | 3.271886792 | 7.348395267 | F12;SERPIN |
| 0 | 0 | 3.271886792 | 7.348395267 | PHF23;IRA |
| 0 | 0 | 3.271886792 | 7.348395267 | FMR1;TEN |
| 0 | 0 | 3.271886792 | 7.348395267 | GRIPAP1;S |
| 0 | 0 | 3.271886792 | 7.348395267 | GALNT11; |
| 0 | 0 | 3.271886792 | 7.348395267 | NOS2;S10 |
| 0 | 0 | 3.271886792 | 7.348395267 | PGS1;CRLS |
| 0 | 0 | 3.271886792 | 7.348395267 | IL10;XBP1; |
| 0 | 0 | 3.271886792 | 7.348395267 | PHLDB1;R |
| 0 | 0 | 3.271886792 | 7.348395267 | MICU1;MC |
| 0 | 0 | 3.271886792 | 7.348395267 | C3;RAB31; |
| 0 | 0 | 3.271886792 | 7.348395267 | CD40;FLT4 |
| 0 | 0 | 3.271886792 | 7.348395267 | ADAM17;C |
| 0 | 0 | 3.271886792 | 7.348395267 | NLGN3;LRF |
| 0 | 0 | 3.271886792 | 7.348395267 | SLC22A3;S |
| 0 | 0 | 3.271886792 | 7.348395267 | BST1;OLFN |
| 0 | 0 | 3.271886792 | 7.348395267 | RGS14;DDI |
| 0 | 0 | 3.271886792 | 7.348395267 | PPP1R3B;P |
| 0 | 0 | 3.271886792 | 7.348395267 | NR1H2;NR |
| 0 | 0 | 3.271886792 | 7.348395267 | SPRED2;SP |
| 0 | 0 | 3.271886792 | 7.348395267 | CAMK1D;C |
| 0 | 0 | 3.271886792 | 7.348395267 | DDIT3;TMS |
| 0 | 0 | 3.271886792 | 7.348395267 | DDX17;DD |
| 0 | 0 | 3.271886792 | 7.348395267 | ANXA1;AN |
| 0 | 0 | 3.271886792 | 7.348395267 | NR4A3;STA |
| 0 | 0 | 3.271886792 | 7.348395267 | ATXN3;F12 |
| 0 | 0 | 3.271886792 | 7.348395267 | MX1;ISG15 |
| 0 | 0 | 3.271886792 | 7.348395267 | PLXNA2;PL |
| 0 | 0 | 1.386376926 | 3.106595521 | MEF2A;AC |

| | | | | |
|---|---|---|---|---|
| 0 | 0 | 1.679133075 | 3.755701929 | PYCARD;VA… |
| 0 | 0 | 1.909501764 | 4.26131572 | OVGP1;SD… |
| 0 | 0 | 1.909501764 | 4.26131572 | TGM1;FCG… |
| 0 | 0 | 1.909501764 | 4.26131572 | SMURF1;FI… |
| 0 | 0 | 1.733302928 | 3.856410077 | ATXN3;BST… |
| 0 | 0 | 1.806192556 | 4.012717262 | FLT1;RGCC… |
| 0 | 0 | 1.806192556 | 4.012717262 | CYLD;ITCH;… |
| 0 | 0 | 1.806192556 | 4.012717262 | ATP6V1G1… |
| 0 | 0 | 1.806192556 | 4.012717262 | SDCBP;SD… |
| 0 | 0 | 1.806192556 | 4.012717262 | IL10;CDKN… |
| 0 | 0 | 1.571859144 | 3.430525845 | IL6;IFNG;R… |
| 0 | 0 | 1.571859144 | 3.430525845 | RET;CDKN2… |
| 0 | 0 | 1.287956652 | 2.806435524 | RNF13;RNI… |
| 0 | 0 | 1.190311235 | 2.591684986 | ZNF331;AC… |
| 0 | 0 | 2.181772407 | 4.708576187 | ACVRL1;SN… |
| 0 | 0 | 2.181772407 | 4.708576187 | ATP1A3;AT… |
| 0 | 0 | 2.181772407 | 4.708576187 | PNPT1;IFI1… |
| 0 | 0 | 2.181772407 | 4.708576187 | NIPBL;CXC… |
| 0 | 0 | 2.181772407 | 4.708576187 | SLC9A3R1;… |
| 0 | 0 | 2.181772407 | 4.708576187 | SUN2;MOF… |
| 0 | 0 | 2.181772407 | 4.708576187 | HCK;SECTN… |
| 0 | 0 | 2.181772407 | 4.708576187 | SEMA4A;SI… |
| 0 | 0 | 2.181772407 | 4.708576187 | PPP1R15A;… |
| 0 | 0 | 2.181772407 | 4.708576187 | PNP;AMPD… |
| 0 | 0 | 2.181772407 | 4.708576187 | SLC22A3;S… |
| 0 | 0 | 2.181772407 | 4.708576187 | ADAM17;C… |
| 0 | 0 | 2.181772407 | 4.708576187 | LAMP1;IL1… |
| 0 | 0 | 2.181772407 | 4.708576187 | MAPK8;IFN… |
| 0 | 0 | 2.181772407 | 4.708576187 | MOV10;CN… |
| 0 | 0 | 2.181772407 | 4.708576187 | BST1;CAM… |
| 0 | 0 | 2.181772407 | 4.708576187 | MAP2K3;W… |
| 0 | 0 | 2.181772407 | 4.708576187 | TBX21;SLA… |
| 0 | 0 | 1.600824291 | 3.45164409 | VMP1;GAB… |
| 0 | 0 | 1.520447781 | 3.230110675 | VASP;TENN… |
| 0 | 0 | 1.520447781 | 3.230110675 | TUT7;PNPT… |
| 0 | 0 | 1.683682732 | 3.559560861 | DDX17;DD… |
| 0 | 0 | 1.683682732 | 3.559560861 | C3;ATAD1;… |
| 0 | 0 | 1.683682732 | 3.559560861 | CASP4;ATP… |
| 0 | 0 | 1.963770306 | 4.151066078 | P2RY12;PT… |
| 0 | 0 | 1.963770306 | 4.151066078 | LYN;BATF3… |
| 0 | 0 | 1.963770306 | 4.151066078 | GHSR;SERF… |
| 0 | 0 | 1.963770306 | 4.151066078 | USP36;SM… |
| 0 | 0 | 1.963770306 | 4.151066078 | NDFIP2;RS… |
| 0 | 0 | 1.963770306 | 4.151066078 | SDCBP;ITG… |
| 0 | 0 | 1.963770306 | 4.151066078 | PRPSAP1;P… |
| 0 | 0 | 1.963770306 | 4.151066078 | TRIM25;TR… |
| 0 | 0 | 1.963770306 | 4.151066078 | FLCN;SMG… |

| | | | | |
|---|---|---|---|---|
| 0 | 0 | 1.745885318 | 3.669559986 | ABCA1;CLN |
| 0 | 0 | 1.745885318 | 3.669559986 | FLCN;PRKA |
| 0 | 0 | 1.540949284 | 3.224357543 | TSPAN14;G |
| 0 | 0 | 1.403876389 | 2.92495928 | SEMA7A;EI |
| 0 | 0 | 1.403876389 | 2.92495928 | DESI1;LRRK |
| 0 | 0 | 1.435753581 | 2.980019752 | ACVRL1;NF |
| 0 | 0 | 1.362386771 | 2.827028732 | PNPT1;FM |
| 0 | 0 | 1.260871481 | 2.608372404 | ITGAM;ELN |
| 0 | 0 | 1.565933252 | 3.228986597 | KAT2B;H1- |
| 0 | 0 | 2.379765949 | 4.905324086 | ATP1A3;AT |
| 0 | 0 | 2.379765949 | 4.905324086 | XKR8;RHO |
| 0 | 0 | 2.379765949 | 4.905324086 | SLC9A3R1; |
| 0 | 0 | 2.379765949 | 4.905324086 | B4GALNT1 |
| 0 | 0 | 2.379765949 | 4.905324086 | HDAC4;ELK |
| 0 | 0 | 2.379765949 | 4.905324086 | TUT7;XRN1 |
| 0 | 0 | 2.379765949 | 4.905324086 | P2RY12;CC |
| 0 | 0 | 2.379765949 | 4.905324086 | CCL8;FMR1 |
| 0 | 0 | 2.379765949 | 4.905324086 | EFEMP1;RI |
| 0 | 0 | 2.379765949 | 4.905324086 | SETDB1;IN |
| 0 | 0 | 2.379765949 | 4.905324086 | ART1;PADI |
| 0 | 0 | 2.379765949 | 4.905324086 | DAXX;TAL1 |
| 0 | 0 | 2.379765949 | 4.905324086 | SPRED2;DI |
| 0 | 0 | 2.379765949 | 4.905324086 | TMSB4X;F( |
| 0 | 0 | 2.379765949 | 4.905324086 | RIC1;FAP;S |
| 0 | 0 | 2.379765949 | 4.905324086 | IL6;PTPRC; |
| 0 | 0 | 2.379765949 | 4.905324086 | GSN;PIK3C |
| 0 | 0 | 2.379765949 | 4.905324086 | NCF1;NCF4 |
| 0 | 0 | 1.219586587 | 2.480444635 | RET;CPB2;I |
| 0 | 0 | 1.38727983 | 2.797207723 | P2RY12;EG |
| 0 | 0 | 1.348951815 | 2.716810847 | PHLDB1;SE |
| 0 | 0 | 4.361624544 | 8.775057436 | ADSS2;APF |
| 0 | 0 | 4.361624544 | 8.775057436 | GLS;KCTD7 |
| 0 | 0 | 4.361624544 | 8.775057436 | NRG1;RBP. |
| 0 | 0 | 4.361624544 | 8.775057436 | RSAD2;CDI |
| 0 | 0 | 4.361624544 | 8.775057436 | SIRT1;SUPT |
| 0 | 0 | 4.361624544 | 8.775057436 | IL6ST;IL6R |
| 0 | 0 | 4.361624544 | 8.775057436 | TGFBR1;VE |
| 0 | 0 | 4.361624544 | 8.775057436 | POLB;LIG4 |
| 0 | 0 | 4.361624544 | 8.775057436 | MAOB;MA |
| 0 | 0 | 4.361624544 | 8.775057436 | ABT1;NOP |
| 0 | 0 | 4.361624544 | 8.775057436 | ABT1;NOP |
| 0 | 0 | 4.361624544 | 8.775057436 | CYP24A1;C |
| 0 | 0 | 4.361624544 | 8.775057436 | PFKFB4;PF |
| 0 | 0 | 4.361624544 | 8.775057436 | ROCK1;TES |
| 0 | 0 | 4.361624544 | 8.775057436 | SLC9A3R1; |
| 0 | 0 | 4.361624544 | 8.775057436 | ID2;HOPX |
| 0 | 0 | 4.361624544 | 8.775057436 | PADI3;PAD |

| | | | | | |
|---|---|---|---|---|---|
| 0 | 0 | 4.361624544 | 8.775057436 | KDM6B;KD | . |
| 0 | 0 | 4.361624544 | 8.775057436 | KMT5C;KM | . |
| 0 | 0 | 4.361624544 | 8.775057436 | KMT5C;KM | . |
| 0 | 0 | 4.361624544 | 8.775057436 | IRF2BP2;FN | . |
| 0 | 0 | 4.361624544 | 8.775057436 | FTH1;SRI | . |
| 0 | 0 | 4.361624544 | 8.775057436 | LRRK2;PRK | . |
| 0 | 0 | 4.361624544 | 8.775057436 | SLC1A2;SL | . |
| 0 | 0 | 4.361624544 | 8.775057436 | RIPK3;PDP | . |
| 0 | 0 | 4.361624544 | 8.775057436 | CLN3;LAM | . |
| 0 | 0 | 4.361624544 | 8.775057436 | HORMAD1 | . |
| 0 | 0 | 4.361624544 | 8.775057436 | NIN;CEP19 | . |
| 0 | 0 | 4.361624544 | 8.775057436 | MOV10;AG | . |
| 0 | 0 | 4.361624544 | 8.775057436 | F12;SERPIN | . |
| 0 | 0 | 4.361624544 | 8.775057436 | STXBP3;CB | . |
| 0 | 0 | 4.361624544 | 8.775057436 | PIH1D1;PL | . |
| 0 | 0 | 4.361624544 | 8.775057436 | PHF1;SUPT | . |
| 0 | 0 | 4.361624544 | 8.775057436 | PIH1D1;SIF | . |
| 0 | 0 | 4.361624544 | 8.775057436 | IL10;NR4A | . |
| 0 | 0 | 4.361624544 | 8.775057436 | PARP3;FCG | . |
| 0 | 0 | 4.361624544 | 8.775057436 | CD300A;LG | . |
| 0 | 0 | 4.361624544 | 8.775057436 | WWC2;WW | . |
| 0 | 0 | 4.361624544 | 8.775057436 | DDIT3;TMS | . |
| 0 | 0 | 4.361624544 | 8.775057436 | RIPOR2;LR | . |
| 0 | 0 | 4.361624544 | 8.775057436 | FLT1;CCL2 | . |
| 0 | 0 | 4.361624544 | 8.775057436 | IFNG;ADCY | . |
| 0 | 0 | 4.361624544 | 8.775057436 | SLC25A29; | . |
| 0 | 0 | 4.361624544 | 8.775057436 | ALKBH1;JM | . |
| 0 | 0 | 4.361624544 | 8.775057436 | NRP1;VEGI | . |
| 0 | 0 | 4.361624544 | 8.775057436 | IFNG;LGAL | . |
| 0 | 0 | 4.361624544 | 8.775057436 | PIM1;RBPJ | . |
| 0 | 0 | 4.361624544 | 8.775057436 | OTUD4;TO | . |
| 0 | 0 | 4.361624544 | 8.775057436 | PRKD2;VEG | . |
| 0 | 0 | 4.361624544 | 8.775057436 | PHF1;ZNF3 | . |
| 0 | 0 | 4.361624544 | 8.775057436 | FGR;GAB2 | . |
| 0 | 0 | 4.361624544 | 8.775057436 | VEGFC;VEG | . |
| 0 | 0 | 4.361624544 | 8.775057436 | STAT3;MBI | . |
| 0 | 0 | 4.361624544 | 8.775057436 | CCL4;CCL3 | . |
| 0 | 0 | 4.361624544 | 8.775057436 | SLC6A6;RIF | . |
| 0 | 0 | 4.361624544 | 8.775057436 | SLC46A2;S | . |
| 0 | 0 | 4.361624544 | 8.775057436 | CD81;LDLR | . |
| 0 | 0 | 4.361624544 | 8.775057436 | RSAD2;PTF | . |
| 0 | 0 | 4.361624544 | 8.775057436 | E2F3;SOD2 | . |
| 0 | 0 | 4.361624544 | 8.775057436 | NOS2;SLCC | . |
| 0 | 0 | 4.361624544 | 8.775057436 | PADI3;PAD | . |
| 0 | 0 | 4.361624544 | 8.775057436 | FLOT1;FLO | . |
| 0 | 0 | 4.361624544 | 8.775057436 | PDXK;HAA | . |
| 0 | 0 | 4.361624544 | 8.775057436 | NQO2;CYP | . |

| | | | | | |
|---|---|---|---|---|---|
| 0 | 0 | 4.361624544 | 8.775057436 | CD81;ICAN | . |
| 0 | 0 | 4.361624544 | 8.775057436 | USP15;PLA | . |
| 0 | 0 | 4.361624544 | 8.775057436 | IL6;LDLR | . |
| 0 | 0 | 4.361624544 | 8.775057436 | F12;SERPIN | . |
| 0 | 0 | 4.361624544 | 8.775057436 | CLN3;SRC | . |
| 0 | 0 | 4.361624544 | 8.775057436 | CLEC7A;LR | . |
| 0 | 0 | 4.361624544 | 8.775057436 | CLN3;MICL | . |
| 0 | 0 | 4.361624544 | 8.775057436 | MAPK8;MA | . |
| 0 | 0 | 4.361624544 | 8.775057436 | BCL6;TNFA | . |
| 0 | 0 | 4.361624544 | 8.775057436 | CCPG1;PBX | . |
| 0 | 0 | 4.361624544 | 8.775057436 | PIH1D1;ZN | . |
| 0 | 0 | 4.361624544 | 8.775057436 | PIH1D1;SIF | . |
| 0 | 0 | 4.361624544 | 8.775057436 | TCF7L2;FFA | . |
| 0 | 0 | 4.361624544 | 8.775057436 | MPV17L;FY | . |
| 0 | 0 | 4.361624544 | 8.775057436 | P2RY6;PTK | . |
| 0 | 0 | 4.361624544 | 8.775057436 | FSCN1;RAF | . |
| 0 | 0 | 4.361624544 | 8.775057436 | NCR1;PIK3 | . |
| 0 | 0 | 4.361624544 | 8.775057436 | CD300A;FC | . |
| 0 | 0 | 4.361624544 | 8.775057436 | MTMR3;M | . |
| 0 | 0 | 4.361624544 | 8.775057436 | SLAMF8;LE | . |
| 0 | 0 | 4.361624544 | 8.775057436 | STX1B;STX | . |
| 0 | 0 | 4.361624544 | 8.775057436 | GRIPAP1;R | . |
| 0 | 0 | 4.361624544 | 8.775057436 | EDN1;ECE1 | . |
| 0 | 0 | 4.361624544 | 8.775057436 | DOCK4;ATI | . |
| 0 | 0 | 4.361624544 | 8.775057436 | CYP27B1;N | . |
| 0 | 0 | 4.361624544 | 8.775057436 | XBP1;KHK | . |
| 0 | 0 | 4.361624544 | 8.775057436 | KMO;TNF | . |
| 0 | 0 | 4.361624544 | 8.775057436 | RNASEH2B | . |
| 0 | 0 | 4.361624544 | 8.775057436 | TRIR;XRN1 | . |
| 0 | 0 | 4.361624544 | 8.775057436 | ABT1;NOP! | . |
| 0 | 0 | 4.361624544 | 8.775057436 | EGR2;NF1 | . |
| 0 | 0 | 4.361624544 | 8.775057436 | SLC22A3;S | . |
| 0 | 0 | 4.361624544 | 8.775057436 | SLC22A3;S | . |
| 0 | 0 | 4.361624544 | 8.775057436 | TSTD1;SQC | . |
| 0 | 0 | 4.361624544 | 8.775057436 | TSTD1;SQC | . |
| 0 | 0 | 4.361624544 | 8.775057436 | CCL2;ITGAI | . |
| 0 | 0 | 1.270015732 | 2.542231641 | AMER1;DR | . |
| 0 | 0 | 1.455433455 | 2.908949142 | PPP1R16B; | . |
| 0 | 0 | 2.045293618 | 4.081315177 | AMER1;FZI | . |
| 0 | 0 | 2.045293618 | 4.081315177 | SELP;PTGF | . |
| 0 | 0 | 2.045293618 | 4.081315177 | URI1;SRC;A | . |
| 0 | 0 | 2.045293618 | 4.081315177 | BCR;ACTN2 | . |
| 0 | 0 | 2.045293618 | 4.081315177 | DTX3L;UBE | . |
| 0 | 0 | 2.045293618 | 4.081315177 | PTPN1;NLF | . |
| 0 | 0 | 2.045293618 | 4.081315177 | ASTL;DUSP | . |
| 0 | 0 | 2.045293618 | 4.081315177 | IL10;JUN;P | . |
| 0 | 0 | 2.045293618 | 4.081315177 | PRKAA1;IL1 | . |

| | | | | |
|---|---|---|---|---|
| 0 | 0 | 2.045293618 | 4.081315177 | CD180;LY8 |
| 0 | 0 | 2.045293618 | 4.081315177 | STARD3;NF |
| 0 | 0 | 2.045293618 | 4.081315177 | CLN3;SLC1 |
| 0 | 0 | 2.804312668 | 5.577259685 | RAMP2;AC |
| 0 | 0 | 2.804312668 | 5.577259685 | NOS2;SLC3 |
| 0 | 0 | 2.804312668 | 5.577259685 | KYNU;ATP |
| 0 | 0 | 2.804312668 | 5.577259685 | ADCY4;AD |
| 0 | 0 | 2.804312668 | 5.577259685 | LRRK2;ATF |
| 0 | 0 | 2.804312668 | 5.577259685 | SESN2;KLH |
| 0 | 0 | 2.804312668 | 5.577259685 | TLR6;TLR4; |
| 0 | 0 | 2.804312668 | 5.577259685 | RYR1;P2RY |
| 0 | 0 | 2.804312668 | 5.577259685 | HAAO;KYN |
| 0 | 0 | 2.804312668 | 5.577259685 | ACVRL1;PR |
| 0 | 0 | 2.804312668 | 5.577259685 | VPS4B;CHM |
| 0 | 0 | 2.804312668 | 5.577259685 | VPS4B;CHM |
| 0 | 0 | 2.804312668 | 5.577259685 | ANXA1;CCI |
| 0 | 0 | 2.804312668 | 5.577259685 | DTX3L;WA |
| 0 | 0 | 2.804312668 | 5.577259685 | KDM4A;KD |
| 0 | 0 | 2.804312668 | 5.577259685 | CLN3;GRIN |
| 0 | 0 | 2.804312668 | 5.577259685 | UGCG;STA |
| 0 | 0 | 2.804312668 | 5.577259685 | ENO1;B2M |
| 0 | 0 | 2.804312668 | 5.577259685 | CSF1;HYAL |
| 0 | 0 | 2.804312668 | 5.577259685 | CD274;LGA |
| 0 | 0 | 2.804312668 | 5.577259685 | FAP;TNFRS |
| 0 | 0 | 2.804312668 | 5.577259685 | CD46;FCGF |
| 0 | 0 | 2.804312668 | 5.577259685 | NLRP12;TE |
| 0 | 0 | 2.804312668 | 5.577259685 | INSIG1;UBA |
| 0 | 0 | 2.804312668 | 5.577259685 | IL10;ADAM |
| 0 | 0 | 2.804312668 | 5.577259685 | LYN;ACOD |
| 0 | 0 | 2.804312668 | 5.577259685 | C3;SCARF1 |
| 0 | 0 | 2.804312668 | 5.577259685 | CD74;LGAL |
| 0 | 0 | 2.804312668 | 5.577259685 | IQSEC1;AR |
| 0 | 0 | 2.804312668 | 5.577259685 | CD74;DCST |
| 0 | 0 | 2.804312668 | 5.577259685 | NIBAN2;FL |
| 0 | 0 | 2.804312668 | 5.577259685 | CD274;LGA |
| 0 | 0 | 2.804312668 | 5.577259685 | CD74;LGAL |
| 0 | 0 | 2.804312668 | 5.577259685 | OTUB2;VCI |
| 0 | 0 | 2.804312668 | 5.577259685 | PDPN;PDE |
| 0 | 0 | 2.804312668 | 5.577259685 | IL6;FCER1G |
| 0 | 0 | 2.804312668 | 5.577259685 | ZFP36;NUF |
| 0 | 0 | 2.804312668 | 5.577259685 | PDE4B;SRI; |
| 0 | 0 | 2.804312668 | 5.577259685 | SELP;SELE; |
| 0 | 0 | 2.804312668 | 5.577259685 | LYN;CD300 |
| 0 | 0 | 2.804312668 | 5.577259685 | WWC2;WV |
| 0 | 0 | 2.804312668 | 5.577259685 | FLCN;PRKA |
| 0 | 0 | 2.804312668 | 5.577259685 | C3;RAB31; |
| 0 | 0 | 2.804312668 | 5.577259685 | MTMR3;AE |

| | | | | |
|---|---|---|---|---|
| 0 | 0 | 2.804312668 | 5.577259685 | LRRK2;ATF |
| 0 | 0 | 2.804312668 | 5.577259685 | SESN2;KLH |
| 0 | 0 | 2.804312668 | 5.577259685 | RARG;RAR |
| 0 | 0 | 2.804312668 | 5.577259685 | RYR1;CCL3 |
| 0 | 0 | 2.804312668 | 5.577259685 | ABCA1;SRC |
| 0 | 0 | 2.804312668 | 5.577259685 | SLC22A3;S |
| 0 | 0 | 1.68946887 | 3.35941304 | C3;SERPINI |
| 0 | 0 | 1.762413512 | 3.481587857 | NAIF1;SFN |
| 0 | 0 | 1.762413512 | 3.481587857 | SEMA4A;SI |
| 0 | 0 | 1.762413512 | 3.481587857 | CYLD;ITCH |
| 0 | 0 | 1.762413512 | 3.481587857 | NFKBIA;AB |
| 0 | 0 | 1.762413512 | 3.481587857 | ANXA1;AN |
| 0 | 0 | 1.762413512 | 3.481587857 | STX1B;PNK |
| 0 | 0 | 1.762413512 | 3.481587857 | ACTN2;STX |
| 0 | 0 | 1.870149495 | 3.692769199 | MAPK8;MT |
| 0 | 0 | 1.870149495 | 3.692769199 | SLC6A9;GP |
| 0 | 0 | 1.870149495 | 3.692769199 | PRKCD;HIP |
| 0 | 0 | 1.870149495 | 3.692769199 | LYN;ADAM |
| 0 | 0 | 1.870149495 | 3.692769199 | EDN1;NIBA |
| 0 | 0 | 1.870149495 | 3.692769199 | EDN1;FAP; |
| 0 | 0 | 1.350816951 | 2.664189945 | FZD1;LYN;I |
| 0 | 0 | 1.275633287 | 2.511061603 | BTG3;BTG2 |
| 0 | 0 | 1.423838877 | 2.79571579 | CELA2A;XB |
| 0 | 0 | 1.558924743 | 3.022748186 | DDX3X;HSI |
| 0 | 0 | 1.558924743 | 3.022748186 | IFNG;PRKC |
| 0 | 0 | 1.558924743 | 3.022748186 | RYR1;GBP5 |
| 0 | 0 | 1.322398084 | 2.564034587 | MEF2A;RYI |
| 0 | 0 | 1.482628793 | 2.8503355 | ITCH;RNF1 |
| 0 | 0 | 1.267055386 | 2.432758343 | RNASEL;TL |
| 0 | 0 | 1.336955595 | 2.5563446 | RANBP2;CI |
| 0 | 0 | 1.355231303 | 2.557999866 | RAP1GAP2 |
| 0 | 0 | 1.63657845 | 3.078539888 | GABARAPL |
| 0 | 0 | 1.63657845 | 3.078539888 | FLCN;SLC9 |
| 0 | 0 | 1.63657845 | 3.078539888 | NLGN3;P2I |
| 0 | 0 | 1.63657845 | 3.078539888 | IL6;KYNU;F |
| 0 | 0 | 2.181326287 | 4.097488245 | NIPBL;ID2; |
| 0 | 0 | 2.181326287 | 4.097488245 | CCL8;CCL4 |
| 0 | 0 | 2.181326287 | 4.097488245 | CCL8;CCL4 |
| 0 | 0 | 2.181326287 | 4.097488245 | NOS2;PRO |
| 0 | 0 | 2.181326287 | 4.097488245 | CD300A;SL |
| 0 | 0 | 2.181326287 | 4.097488245 | MAD2L2;D |
| 0 | 0 | 2.181326287 | 4.097488245 | PNP;GDA;X |
| 0 | 0 | 2.181326287 | 4.097488245 | CYP27B1;IS |
| 0 | 0 | 2.181326287 | 4.097488245 | MAD2L2;K |
| 0 | 0 | 2.181326287 | 4.097488245 | MAD2L2;C |
| 0 | 0 | 2.181326287 | 4.097488245 | PPP1R16B; |
| 0 | 0 | 2.181326287 | 4.097488245 | PRKCD;TNI |

| | | | | |
|---|---|---|---|---|
| 0 | 0 | 2.181326287 | 4.097488245 | CD74;DCST |
| 0 | 0 | 2.181326287 | 4.097488245 | PPIF;NUPR |
| 0 | 0 | 2.181326287 | 4.097488245 | UTP15;USF |
| 0 | 0 | 2.181326287 | 4.097488245 | ASAH1;ACE |
| 0 | 0 | 2.181326287 | 4.097488245 | CTSH;CD46 |
| 0 | 0 | 1.37883959 | 2.573332919 | CD74;TNFS |
| 0 | 0 | 1.37883959 | 2.573332919 | AMER1;FZI |
| 0 | 0 | 1.697040956 | 3.154487908 | XKR8;RIPO |
| 0 | 0 | 1.410487656 | 2.609706936 | ZNF473;AL |
| 0 | 0 | 1.924871157 | 3.553869531 | GSN;RHOG |
| 0 | 0 | 1.924871157 | 3.553869531 | PSME1;CTS |
| 0 | 0 | 1.924871157 | 3.553869531 | PSMA2;PSI |
| 0 | 0 | 1.924871157 | 3.553869531 | XBP1;BCL2 |
| 0 | 0 | 1.924871157 | 3.553869531 | MTMR3;AI |
| 0 | 0 | 1.924871157 | 3.553869531 | GPR35;FM |
| 0 | 0 | 1.785039668 | 3.29335993 | XBP1;SESN |
| 0 | 0 | 1.785039668 | 3.29335993 | STAT5B;ZF |
| 0 | 0 | 1.785039668 | 3.29335993 | KAT2B;CRE |
| 0 | 0 | 1.785039668 | 3.29335993 | SLC7A5;PR |
| 0 | 0 | 1.785039668 | 3.29335993 | GPR35;FM |
| 0 | 0 | 1.785039668 | 3.29335993 | SLC6A6;SL( |
| 0 | 0 | 1.785039668 | 3.29335993 | P2RY8;GPF |
| 0 | 0 | 1.785039668 | 3.29335993 | KLF7;LRP5; |
| 0 | 0 | 1.785039668 | 3.29335993 | IGSF10;NIF |
| 0 | 0 | 1.455088561 | 2.6806904 | KLF10;FLCI |
| 0 | 0 | 1.455088561 | 2.6806904 | MAPK8;RA |
| 0 | 0 | 1.310182371 | 2.410429886 | SMG1;MTI |
| 0 | 0 | 1.339753321 | 2.446051677 | UPF2;RANI |
| 0 | 0 | 1.550491679 | 2.810870781 | DDX17;DD |
| 0 | 0 | 1.224378905 | 2.216132707 | ITGAM;ELN |
| 0 | 0 | 1.323959192 | 2.396156614 | KDM6B;DY |
| 0 | 0 | 1.202807417 | 2.171520223 | ACVRL1;RI |
| 0 | 0 | 1.310064565 | 2.353166167 | SEMA6A;SI |
| 0 | 0 | 1.389036251 | 2.472050602 | XBP1;SMA |
| 0 | 0 | 1.58689351 | 2.82370709 | NRP1;ROCI |
| 0 | 0 | 1.58689351 | 2.82370709 | PYCARD;B( |
| 0 | 0 | 2.453632075 | 4.340521773 | PHKG2;PRI |
| 0 | 0 | 2.453632075 | 4.340521773 | SERTAD1;Z |
| 0 | 0 | 2.453632075 | 4.340521773 | C5AR2;C5A |
| 0 | 0 | 2.453632075 | 4.340521773 | GRK2;ADRI |
| 0 | 0 | 2.453632075 | 4.340521773 | SLC9A3R1; |
| 0 | 0 | 2.453632075 | 4.340521773 | GLUL;ASS1 |
| 0 | 0 | 2.453632075 | 4.340521773 | WNT2B;AR |
| 0 | 0 | 2.453632075 | 4.340521773 | C1S;C1R;CI |
| 0 | 0 | 2.453632075 | 4.340521773 | SYNJ1;INPF |
| 0 | 0 | 2.453632075 | 4.340521773 | LIG4;CCR6; |
| 0 | 0 | 2.453632075 | 4.340521773 | UGCG;WDI |

| | | | | |
|---|---|---|---|---|
| 0 | 0 | 2.453632075 | 4.340521773 | CD274;LGA… |
| 0 | 0 | 2.453632075 | 4.340521773 | IL6;TMEM… |
| 0 | 0 | 2.453632075 | 4.340521773 | SOCS3;SOC… |
| 0 | 0 | 2.453632075 | 4.340521773 | PTPRC;IRA… |
| 0 | 0 | 2.453632075 | 4.340521773 | GFI1;SNAI1… |
| 0 | 0 | 2.453632075 | 4.340521773 | GALNT11;S… |
| 0 | 0 | 2.453632075 | 4.340521773 | APOA2;EN… |
| 0 | 0 | 2.453632075 | 4.340521773 | SYNJ1;INPP… |
| 0 | 0 | 2.453632075 | 4.340521773 | PRKAA1;AE… |
| 0 | 0 | 2.453632075 | 4.340521773 | RIPOR2;CC… |
| 0 | 0 | 2.453632075 | 4.340521773 | IL18;RARA;… |
| 0 | 0 | 2.453632075 | 4.340521773 | EGR2;PARI… |
| 0 | 0 | 2.453632075 | 4.340521773 | ZFP36;FMF… |
| 0 | 0 | 2.453632075 | 4.340521773 | DDX5;IL6;F… |
| 0 | 0 | 2.453632075 | 4.340521773 | LGALS9;CD… |
| 0 | 0 | 2.453632075 | 4.340521773 | NLGN3;LRF… |
| 0 | 0 | 2.453632075 | 4.340521773 | SRC;LRRK2… |
| 0 | 0 | 2.453632075 | 4.340521773 | ATXN3;USF… |
| 0 | 0 | 2.453632075 | 4.340521773 | DIRAS3;DN… |
| 0 | 0 | 2.453632075 | 4.340521773 | CLEC7A;IL1… |
| 0 | 0 | 2.453632075 | 4.340521773 | LYN;RNF13… |
| 0 | 0 | 2.453632075 | 4.340521773 | ACTA2;EDN… |
| 0 | 0 | 1.324623158 | 2.338900635 | UPF2;RANI… |
| 0 | 0 | 1.428549792 | 2.52155141 | RANBP2;ZF… |
| 0 | 0 | 1.487892546 | 2.621149782 | ACVRL1;NF… |
| 0 | 0 | 1.487892546 | 2.621149782 | KMT2E;KA… |
| 0 | 0 | 1.487892546 | 2.621149782 | SERPINB4;… |
| 0 | 0 | 1.636337868 | 2.862094558 | ATP6V1G1… |
| 0 | 0 | 1.636337868 | 2.862094558 | IL6;GDI1;IL… |
| 0 | 0 | 1.636337868 | 2.862094558 | SPN;IL10;C… |
| 0 | 0 | 1.636337868 | 2.862094558 | P2RY12;FG… |
| 0 | 0 | 1.636337868 | 2.862094558 | CYP27B1;C… |
| 0 | 0 | 1.395417288 | 2.421842965 | CELA2A;NC… |
| 0 | 0 | 1.149579387 | 1.984034402 | FBXO25;UE… |
| 0 | 0 | 1.707330694 | 2.943784819 | PUS10;TUT… |
| 0 | 0 | 1.707330694 | 2.943784819 | ACVRL1;CD… |
| 0 | 0 | 1.707330694 | 2.943784819 | EIF6;ELOVL… |
| 0 | 0 | 1.707330694 | 2.943784819 | SLC11A1;X… |
| 0 | 0 | 1.440325511 | 2.480336179 | ATP6V1G1… |
| 0 | 0 | 1.440325511 | 2.480336179 | UGCG;ZDH… |
| 0 | 0 | 1.510648202 | 2.601190424 | CDKN1A;N… |
| 0 | 0 | 1.510648202 | 2.601190424 | C3;CLN3;R… |
| 0 | 0 | 1.325380266 | 2.280893812 | ITGAM;CD… |
| 0 | 0 | 2.013415803 | 3.451587782 | DGAT2;ZC… |
| 0 | 0 | 2.013415803 | 3.451587782 | ASAH1;GC… |
| 0 | 0 | 2.013415803 | 3.451587782 | EDEM1;SE… |
| 0 | 0 | 2.013415803 | 3.451587782 | RYBP;DTX3… |

| | | | | |
|---|---|---|---|---|
| 0 | 0 | 2.013415803 | 3.451587782 | VPS4B;CHI |
| 0 | 0 | 2.013415803 | 3.451587782 | SLC9A3R1; |
| 0 | 0 | 2.013415803 | 3.451587782 | HDAC4;FLC |
| 0 | 0 | 2.013415803 | 3.451587782 | IL10;CDKN |
| 0 | 0 | 2.013415803 | 3.451587782 | EDN1;PIM: |
| 0 | 0 | 2.013415803 | 3.451587782 | ADAM17;C |
| 0 | 0 | 2.013415803 | 3.451587782 | BCAR3;BRF |
| 0 | 0 | 2.013415803 | 3.451587782 | NRP1;DOC |
| 0 | 0 | 2.013415803 | 3.451587782 | MN1;TMEI |
| 0 | 0 | 2.013415803 | 3.451587782 | RYR1;P2RY |
| 0 | 0 | 2.013415803 | 3.451587782 | PLEK;INPP! |
| 0 | 0 | 1.817828969 | 3.107035923 | DGAT2;GP/ |
| 0 | 0 | 1.817828969 | 3.107035923 | NRP1;NR4/ |
| 0 | 0 | 1.817828969 | 3.107035923 | RPIA;HK3;T |
| 0 | 0 | 1.817828969 | 3.107035923 | GDI1;INSIG |
| 0 | 0 | 1.817828969 | 3.107035923 | PPP1R15A; |
| 0 | 0 | 1.817828969 | 3.107035923 | RBM3;CELI |
| 0 | 0 | 3.2710298 | 5.545614628 | PTK2B;JAK |
| 0 | 0 | 3.2710298 | 5.545614628 | KIFC3;INA\ |
| 0 | 0 | 3.2710298 | 5.545614628 | SLC25A29; |
| 0 | 0 | 3.2710298 | 5.545614628 | HSD3B7;C> |
| 0 | 0 | 3.2710298 | 5.545614628 | SIRT1;EZH: |
| 0 | 0 | 3.2710298 | 5.545614628 | CBLIF;TCN: |
| 0 | 0 | 3.2710298 | 5.545614628 | SLC2A10;S |
| 0 | 0 | 3.2710298 | 5.545614628 | SLC6A6;SL( |
| 0 | 0 | 3.2710298 | 5.545614628 | JADE3;JAD |
| 0 | 0 | 3.2710298 | 5.545614628 | NRXN2;STF |
| 0 | 0 | 3.2710298 | 5.545614628 | AMPD1;AN |
| 0 | 0 | 3.2710298 | 5.545614628 | CLN3;CFTR |
| 0 | 0 | 3.2710298 | 5.545614628 | SDS;SDSL |
| 0 | 0 | 3.2710298 | 5.545614628 | SLC25A29; |
| 0 | 0 | 3.2710298 | 5.545614628 | SLC25A29; |
| 0 | 0 | 3.2710298 | 5.545614628 | SLC7A5;SL( |
| 0 | 0 | 3.2710298 | 5.545614628 | ALOX5AP;/ |
| 0 | 0 | 3.2710298 | 5.545614628 | FASLG;RIPI |
| 0 | 0 | 3.2710298 | 5.545614628 | MOCOS;M |
| 0 | 0 | 3.2710298 | 5.545614628 | ATXN3;USF |
| 0 | 0 | 3.2710298 | 5.545614628 | ACVRL1;PR |
| 0 | 0 | 3.2710298 | 5.545614628 | GSN;PIK3C |
| 0 | 0 | 3.2710298 | 5.545614628 | CD300A;FC |
| 0 | 0 | 3.2710298 | 5.545614628 | IL12B;IL12/ |
| 0 | 0 | 3.2710298 | 5.545614628 | PPP1R10;N |
| 0 | 0 | 3.2710298 | 5.545614628 | HYOU1;EN |
| 0 | 0 | 3.2710298 | 5.545614628 | HAS1;HYAI |
| 0 | 0 | 3.2710298 | 5.545614628 | CD300A;CI |
| 0 | 0 | 3.2710298 | 5.545614628 | DUSP1;SLA |
| 0 | 0 | 3.2710298 | 5.545614628 | GDI1;INPP! |

| | | | | | |
|---|---|---|---|---|---|
| 0 | 0 | 3.2710298 | 5.545614628 | ARHGDIB;( | . |
| 0 | 0 | 3.2710298 | 5.545614628 | SLC25A19; | . |
| 0 | 0 | 3.2710298 | 5.545614628 | SOD2;HIF1 | . |
| 0 | 0 | 3.2710298 | 5.545614628 | RPIA;TKT | . |
| 0 | 0 | 3.2710298 | 5.545614628 | CALR;TAPE | . |
| 0 | 0 | 3.2710298 | 5.545614628 | VPS4B;CEP | . |
| 0 | 0 | 3.2710298 | 5.545614628 | PRKAA1;AI | . |
| 0 | 0 | 3.2710298 | 5.545614628 | BCL2L11;D | . |
| 0 | 0 | 3.2710298 | 5.545614628 | SIRT1;PTPI | . |
| 0 | 0 | 3.2710298 | 5.545614628 | FMR1;IL1B | . |
| 0 | 0 | 3.2710298 | 5.545614628 | PLCG2;TNF | . |
| 0 | 0 | 3.2710298 | 5.545614628 | CD274;FBX | . |
| 0 | 0 | 3.2710298 | 5.545614628 | TMEM119; | . |
| 0 | 0 | 3.2710298 | 5.545614628 | FBXO38;M | . |
| 0 | 0 | 3.2710298 | 5.545614628 | EGR1;NAB: | . |
| 0 | 0 | 3.2710298 | 5.545614628 | CHM;RABC | . |
| 0 | 0 | 3.2710298 | 5.545614628 | BCL2L11;B | . |
| 0 | 0 | 3.2710298 | 5.545614628 | ITCH;UBE2 | . |
| 0 | 0 | 3.2710298 | 5.545614628 | TCF4;SUPT | . |
| 0 | 0 | 3.2710298 | 5.545614628 | GCH1;MTH | . |
| 0 | 0 | 3.2710298 | 5.545614628 | VDAC3;SLC | . |
| 0 | 0 | 3.2710298 | 5.545614628 | STAT3;HES | . |
| 0 | 0 | 3.2710298 | 5.545614628 | RAB11FIP1 | . |
| 0 | 0 | 3.2710298 | 5.545614628 | KLF7;SIRT1 | . |
| 0 | 0 | 3.2710298 | 5.545614628 | FLT4;TMBI | . |
| 0 | 0 | 3.2710298 | 5.545614628 | OTUD4;TO | . |
| 0 | 0 | 3.2710298 | 5.545614628 | SDCBP;SDC | . |
| 0 | 0 | 3.2710298 | 5.545614628 | EIF2AK2;N | . |
| 0 | 0 | 3.2710298 | 5.545614628 | IL1A;IL1B | . |
| 0 | 0 | 3.2710298 | 5.545614628 | ZFP36;GSN | . |
| 0 | 0 | 3.2710298 | 5.545614628 | TNFRSF1B; | . |
| 0 | 0 | 3.2710298 | 5.545614628 | VEGFC;VEC | . |
| 0 | 0 | 3.2710298 | 5.545614628 | PLEKHM2;I | . |
| 0 | 0 | 3.2710298 | 5.545614628 | RIPOR2;OL | . |
| 0 | 0 | 3.2710298 | 5.545614628 | LRRK2;MA | . |
| 0 | 0 | 3.2710298 | 5.545614628 | GCH1;STK2 | . |
| 0 | 0 | 3.2710298 | 5.545614628 | ATP1A3;KC | . |
| 0 | 0 | 3.2710298 | 5.545614628 | EDN1;MYL | . |
| 0 | 0 | 3.2710298 | 5.545614628 | BST1;GCH1 | . |
| 0 | 0 | 3.2710298 | 5.545614628 | CD81;LGAI | . |
| 0 | 0 | 3.2710298 | 5.545614628 | CYP4F2;PT | . |
| 0 | 0 | 3.2710298 | 5.545614628 | NIPBL;REC | . |
| 0 | 0 | 3.2710298 | 5.545614628 | NRP1;ALCA | . |
| 0 | 0 | 3.2710298 | 5.545614628 | SRC;PXN | . |
| 0 | 0 | 3.2710298 | 5.545614628 | STAT5A;ST | . |
| 0 | 0 | 3.2710298 | 5.545614628 | RPUSD4;PL | . |
| 0 | 0 | 3.2710298 | 5.545614628 | LIMK2;TES | . |

| | | | | |
|---|---|---|---|---|
| 0 | 0 | 3.2710298 | 5.545614628 | NRXN2;STI |
| 0 | 0 | 1.402958836 | 2.372196719 | AMER1;FZI |
| 0 | 0 | 1.540131213 | 2.592105089 | ERO1A;RYI |
| 0 | 0 | 1.540131213 | 2.592105089 | IL10;MAD2 |
| 0 | 0 | 1.540131213 | 2.592105089 | IL10;H1-4;I |
| 0 | 0 | 1.454744188 | 2.442141981 | SPRED2;SP |
| 0 | 0 | 1.454744188 | 2.442141981 | HK3;SDS;SI |
| 0 | 0 | 1.454744188 | 2.442141981 | RANBP2;SE |
| 0 | 0 | 1.454744188 | 2.442141981 | RANBP2;SE |
| 0 | 0 | 1.273566785 | 2.137274045 | BCAR3;ATI |
| 0 | 0 | 1.26403142 | 2.112694842 | ARRDC4;AI |
| 0 | 0 | 1.309711684 | 2.176600413 | PRR7;CDKI |
| 0 | 0 | 1.579821201 | 2.600075348 | P2RY11;AD |
| 0 | 0 | 1.579821201 | 2.600075348 | SYS1;RAB3 |
| 0 | 0 | 1.579821201 | 2.600075348 | CWC22;SA |
| 0 | 0 | 1.579821201 | 2.600075348 | CYP27B1;C |
| 0 | 0 | 1.579821201 | 2.600075348 | ATAD1;SD( |
| 0 | 0 | 1.579821201 | 2.600075348 | STEAP3;AT |
| 0 | 0 | 1.348026213 | 2.218382769 | EDN1;CXCI |
| 0 | 0 | 1.412002197 | 2.323440072 | SPRED1;CD |
| 0 | 0 | 1.282788906 | 2.105350263 | ASAH1;ELC |
| 0 | 0 | 1.472796899 | 2.408370784 | PTPN1;DA) |
| 0 | 0 | 1.472796899 | 2.408370784 | ISG20;ZFP3 |
| 0 | 0 | 1.261809487 | 2.052958019 | AMER1;DD |
| 0 | 0 | 1.378265808 | 2.231962296 | TENM1;IL1 |
| 0 | 0 | 1.245005185 | 2.013702917 | ACVRL1;CD |
| 0 | 0 | 1.636097469 | 2.636646077 | HAAO;KYN |
| 0 | 0 | 1.636097469 | 2.636646077 | ELOVL5;AC |
| 0 | 0 | 1.636097469 | 2.636646077 | SYS1;RAB3 |
| 0 | 0 | 1.636097469 | 2.636646077 | RNASE10;N |
| 0 | 0 | 1.636097469 | 2.636646077 | IL6;GJB6;PI |
| 0 | 0 | 1.636097469 | 2.636646077 | ABCA3;PDI |
| 0 | 0 | 1.636097469 | 2.636646077 | TAF1A;UBT |
| 0 | 0 | 1.201752129 | 1.931200161 | MTMR3;M |
| 0 | 0 | 1.423037063 | 2.276070811 | RNF31;USI |
| 0 | 0 | 1.423037063 | 2.276070811 | RANBP2;SE |
| 0 | 0 | 1.350962001 | 2.159713922 | DDX3X;KLH |
| 0 | 0 | 1.496041048 | 2.38128324 | TIAM2;SEN |
| 0 | 0 | 1.496041048 | 2.38128324 | RANBP2;SE |
| 0 | 0 | 1.496041048 | 2.38128324 | RANBP2;SE |
| 0 | 0 | 1.722054381 | 2.72599695 | HK3;PKLR;I |
| 0 | 0 | 1.722054381 | 2.72599695 | KDM6B;NF |
| 0 | 0 | 1.722054381 | 2.72599695 | NR4A3;LRF |
| 0 | 0 | 1.722054381 | 2.72599695 | ABCA1;NP( |
| 0 | 0 | 1.722054381 | 2.72599695 | HK3;PKLR;I |
| 0 | 0 | 1.722054381 | 2.72599695 | PRKAA1;IL: |
| 0 | 0 | 1.722054381 | 2.72599695 | HES1;CALR |

| | | | | |
|---|---|---|---|---|
| 0 | 0 | 1.722054381 | 2.72599695 | FLCN;SDCB |
| 0 | 0 | 1.722054381 | 2.72599695 | FLCN;SDCB |
| 0 | 0 | 1.722054381 | 2.72599695 | CD74;FMR |
| 0 | 0 | 1.722054381 | 2.72599695 | ATP1A3;AT |
| 0 | 0 | 1.722054381 | 2.72599695 | NUDT7;PN |
| 0 | 0 | 1.722054381 | 2.72599695 | TGM1;ZFP |
| 0 | 0 | 2.180880503 | 3.443752116 | KDM6B;NF |
| 0 | 0 | 2.180880503 | 3.443752116 | KLF4;ASS1; |
| 0 | 0 | 2.180880503 | 3.443752116 | GABARAPL |
| 0 | 0 | 2.180880503 | 3.443752116 | GABARAPL |
| 0 | 0 | 2.180880503 | 3.443752116 | KLF9;LMO |
| 0 | 0 | 2.180880503 | 3.443752116 | CYP27B1;C |
| 0 | 0 | 2.180880503 | 3.443752116 | RIDA;SDS;S |
| 0 | 0 | 2.180880503 | 3.443752116 | PRKCD;PR |
| 0 | 0 | 2.180880503 | 3.443752116 | HSPA5;INS |
| 0 | 0 | 2.180880503 | 3.443752116 | PLK3;SDE2 |
| 0 | 0 | 2.180880503 | 3.443752116 | PRKCB;CN |
| 0 | 0 | 2.180880503 | 3.443752116 | CCL8;ZC3H |
| 0 | 0 | 2.180880503 | 3.443752116 | SERPING1; |
| 0 | 0 | 2.180880503 | 3.443752116 | PRKCB;IL1 |
| 0 | 0 | 2.180880503 | 3.443752116 | IRF7;HCLS1 |
| 0 | 0 | 2.180880503 | 3.443752116 | RAMP2;PT |
| 0 | 0 | 2.180880503 | 3.443752116 | USPL1;PM |
| 0 | 0 | 2.180880503 | 3.443752116 | CYP27B1;C |
| 0 | 0 | 2.180880503 | 3.443752116 | CLN3;PLA2 |
| 0 | 0 | 2.180880503 | 3.443752116 | ITGAM;DD |
| 0 | 0 | 2.180880503 | 3.443752116 | ASTL;RHBD |
| 0 | 0 | 2.180880503 | 3.443752116 | HSPA5;SEC |
| 0 | 0 | 2.180880503 | 3.443752116 | FZD1;NLG |
| 0 | 0 | 2.180880503 | 3.443752116 | NT5C3A;SL |
| 0 | 0 | 2.180880503 | 3.443752116 | IL6;ID2;HE |
| 0 | 0 | 2.180880503 | 3.443752116 | DTX3L;ZNF |
| 0 | 0 | 2.180880503 | 3.443752116 | CREBBP;PF |
| 0 | 0 | 2.180880503 | 3.443752116 | IQSEC1;AR |
| 0 | 0 | 1.384768241 | 2.176877469 | HHEX;TIFA |
| 0 | 0 | 1.384768241 | 2.176877469 | CDKN2D;C |
| 0 | 0 | 1.869492531 | 2.928274767 | SLC6A6;SL |
| 0 | 0 | 1.869492531 | 2.928274767 | DDX17;DD |
| 0 | 0 | 1.869492531 | 2.928274767 | NRP1;NR4 |
| 0 | 0 | 1.869492531 | 2.928274767 | ZFP36;ANX |
| 0 | 0 | 1.869492531 | 2.928274767 | HYAL1;HY |
| 0 | 0 | 1.869492531 | 2.928274767 | TLR9;NOD |
| 0 | 0 | 1.869492531 | 2.928274767 | MTFP1;MX |
| 0 | 0 | 1.869492531 | 2.928274767 | LAPTM5;P |
| 0 | 0 | 1.869492531 | 2.928274767 | C3;SCARF1 |
| 0 | 0 | 1.869492531 | 2.928274767 | NRP1;PTPF |
| 0 | 0 | 1.869492531 | 2.928274767 | P2RY12;RE |

| | | | | |
|---|---|---|---|---|
| 0 | 0 | 1.869492531 | 2.928274767 | CSF1;IL18;I… |
| 0 | 0 | 1.869492531 | 2.928274767 | RBM3;CELI… |
| 0 | 0 | 1.869492531 | 2.928274767 | XKR8;RIPO… |
| 0 | 0 | 1.869492531 | 2.928274767 | FCER2;GCH… |
| 0 | 0 | 1.869492531 | 2.928274767 | NLGN3;CCI… |
| 0 | 0 | 1.869492531 | 2.928274767 | FAP;ODC1;… |
| 0 | 0 | 1.869492531 | 2.928274767 | MPV17L;H… |
| 0 | 0 | 1.869492531 | 2.928274767 | SFPQ;SOD2… |
| 0 | 0 | 1.869492531 | 2.928274767 | HDAC4;EGI… |
| 0 | 0 | 1.869492531 | 2.928274767 | UTP15;UBT… |
| 0 | 0 | 1.869492531 | 2.928274767 | SFN;BAK1;S… |
| 0 | 0 | 1.869492531 | 2.928274767 | DGAT2;GPA… |
| 0 | 0 | 1.354424265 | 2.100236469 | PAK1;SMA… |
| 0 | 0 | 1.52707231 | 2.364739403 | GCLC;SLC3… |
| 0 | 0 | 1.52707231 | 2.364739403 | CCR1;P2RX… |
| 0 | 0 | 1.52707231 | 2.364739403 | NLRP12;DD… |
| 0 | 0 | 1.139896027 | 1.755864335 | AVIL;UBE2… |
| 0 | 0 | 1.392679177 | 2.121886852 | NCOA1;SLC… |
| 0 | 0 | 1.570562901 | 2.365849921 | ASAH1;ARC… |
| 0 | 0 | 1.570562901 | 2.365849921 | VPS4B;CHN… |
| 0 | 0 | 1.570562901 | 2.365849921 | ILRUN;MAI… |
| 0 | 0 | 1.570562901 | 2.365849921 | RNF31;USF… |
| 0 | 0 | 1.570562901 | 2.365849921 | ING5;KAT2… |
| 0 | 0 | 1.570562901 | 2.365849921 | SLC4A7;CLI… |
| 0 | 0 | 1.570562901 | 2.365849921 | ARG2;PNPT… |
| 0 | 0 | 1.570562901 | 2.365849921 | XBP1;DGAT… |
| 0 | 0 | 1.454400336 | 2.189096223 | P2RY12;ST… |
| 0 | 0 | 1.454400336 | 2.189096223 | DDX5;RIDA… |
| 0 | 0 | 1.454400336 | 2.189096223 | ZFP36;FUS… |
| 0 | 0 | 1.454400336 | 2.189096223 | CXCL8;CRE… |
| 0 | 0 | 1.454400336 | 2.189096223 | NRP1;CSF1… |
| 0 | 0 | 1.358561981 | 2.039894917 | CYLD;LIMK… |
| 0 | 0 | 1.358561981 | 2.039894917 | ATF7IP;TCF… |
| 0 | 0 | 1.331390909 | 1.976455081 | ING5;KAT2… |
| 0 | 0 | 1.331390909 | 1.976455081 | ITCH;PSMA… |
| 0 | 0 | 1.402501621 | 2.067301961 | DDX17;DD… |
| 0 | 0 | 1.309242424 | 1.926311951 | ERO1A;USI… |
| 0 | 0 | 1.635857251 | 2.399165426 | MTMR3;SY… |
| 0 | 0 | 1.635857251 | 2.399165426 | CCNT1;ELL… |
| 0 | 0 | 1.635857251 | 2.399165426 | CREB1;IL1E… |
| 0 | 0 | 1.635857251 | 2.399165426 | CLN3;ASTL… |
| 0 | 0 | 1.635857251 | 2.399165426 | NUB1;PSM… |
| 0 | 0 | 1.635857251 | 2.399165426 | DDX58;RIC… |
| 0 | 0 | 1.635857251 | 2.399165426 | CYP4F2;AT… |
| 0 | 0 | 1.290843202 | 1.886021637 | CDK17;CDI… |
| 0 | 0 | 1.477726575 | 2.152926659 | MOV10;RII… |
| 0 | 0 | 1.477726575 | 2.152926659 | SDCBP;RYE… |

| | | | | |
|---|---|---|---|---|
| 0 | 0 | 1.477726575 | 2.152926659 | NIBAN2;RG |
| 0 | 0 | 1.477726575 | 2.152926659 | EDN1;ANX |
| 0 | 0 | 1.477726575 | 2.152926659 | RAP1B;VGI |
| 0 | 0 | 1.477726575 | 2.152926659 | GGT5;STAT |
| 0 | 0 | 1.477726575 | 2.152926659 | NLGN3;SYN |
| 0 | 0 | 1.275316798 | 1.853233309 | CDKN2D;C |
| 0 | 0 | 1.363586203 | 1.978640134 | MEF2A;CX |
| 0 | 0 | 1.363586203 | 1.978640134 | CCR1;TRPC |
| 0 | 0 | 2.616672954 | 3.785374989 | KDM3A;TK |
| 0 | 0 | 2.616672954 | 3.785374989 | ACTN2;SRC |
| 0 | 0 | 2.616672954 | 3.785374989 | ARG2;NOS |
| 0 | 0 | 2.616672954 | 3.785374989 | EMP1;EMP |
| 0 | 0 | 2.616672954 | 3.785374989 | ATP2B1;SL |
| 0 | 0 | 2.616672954 | 3.785374989 | PLA2G4A;P |
| 0 | 0 | 2.616672954 | 3.785374989 | PGS1;CRLS |
| 0 | 0 | 2.616672954 | 3.785374989 | MAOB;MA |
| 0 | 0 | 2.616672954 | 3.785374989 | VPS4B;ETF |
| 0 | 0 | 2.616672954 | 3.785374989 | RYR1;SLC8 |
| 0 | 0 | 2.616672954 | 3.785374989 | NR4A3;TGI |
| 0 | 0 | 2.616672954 | 3.785374989 | SNX18;SNX |
| 0 | 0 | 2.616672954 | 3.785374989 | NRP1;PHA |
| 0 | 0 | 2.616672954 | 3.785374989 | SLC22A3;S |
| 0 | 0 | 2.616672954 | 3.785374989 | ACVRL1;RE |
| 0 | 0 | 2.616672954 | 3.785374989 | ACVRL1;RE |
| 0 | 0 | 2.616672954 | 3.785374989 | VPS4B;LYS |
| 0 | 0 | 2.616672954 | 3.785374989 | PARD3;EZR |
| 0 | 0 | 2.616672954 | 3.785374989 | ELOVL4;EL |
| 0 | 0 | 2.616672954 | 3.785374989 | ELOVL4;EL |
| 0 | 0 | 2.616672954 | 3.785374989 | ELOVL4;EL |
| 0 | 0 | 2.616672954 | 3.785374989 | ELOVL4;EL |
| 0 | 0 | 2.616672954 | 3.785374989 | JAML;SOX1 |
| 0 | 0 | 2.616672954 | 3.785374989 | GJB2;GJB6 |
| 0 | 0 | 2.616672954 | 3.785374989 | SOD2;HIF1 |
| 0 | 0 | 2.616672954 | 3.785374989 | SLC9A3R1; |
| 0 | 0 | 2.616672954 | 3.785374989 | SLC1A5;SL |
| 0 | 0 | 2.616672954 | 3.785374989 | GGT5;GGT |
| 0 | 0 | 2.616672954 | 3.785374989 | TKT;KHK |
| 0 | 0 | 2.616672954 | 3.785374989 | P2RY12;NF |
| 0 | 0 | 2.616672954 | 3.785374989 | ING5;BRPF |
| 0 | 0 | 2.616672954 | 3.785374989 | BCL2L12;C |
| 0 | 0 | 2.616672954 | 3.785374989 | FLVCR1;LC |
| 0 | 0 | 2.616672954 | 3.785374989 | SLC11A2;S |
| 0 | 0 | 2.616672954 | 3.785374989 | ALOX5AP;A |
| 0 | 0 | 2.616672954 | 3.785374989 | LIG4;BATF |
| 0 | 0 | 2.616672954 | 3.785374989 | RB1CC1;AT |
| 0 | 0 | 2.616672954 | 3.785374989 | SMURF1;C |
| 0 | 0 | 2.616672954 | 3.785374989 | AGO2;ADA |

| | | | | | |
|---|---|---|---|---|---|
| 0 | 0 | 2.616672954 | 3.785374989 | VMP1;CAL | . |
| 0 | 0 | 2.616672954 | 3.785374989 | B2M;TLR2 | . |
| 0 | 0 | 2.616672954 | 3.785374989 | DGAT2;PLA | . |
| 0 | 0 | 2.616672954 | 3.785374989 | CCL8;ZC3H | . |
| 0 | 0 | 2.616672954 | 3.785374989 | NLRP3;FCG | . |
| 0 | 0 | 2.616672954 | 3.785374989 | RGCC;SUSD | . |
| 0 | 0 | 2.616672954 | 3.785374989 | TIFAB;NR1 | . |
| 0 | 0 | 2.616672954 | 3.785374989 | ITGAV;PSE | . |
| 0 | 0 | 2.616672954 | 3.785374989 | IL10;ROCK1 | . |
| 0 | 0 | 2.616672954 | 3.785374989 | OTUB2;PAI | . |
| 0 | 0 | 2.616672954 | 3.785374989 | CALR;EZH2 | . |
| 0 | 0 | 2.616672954 | 3.785374989 | PRKCQ;JAK | . |
| 0 | 0 | 2.616672954 | 3.785374989 | ROCK1;PHA | . |
| 0 | 0 | 2.616672954 | 3.785374989 | HAAO;KYN | . |
| 0 | 0 | 2.616672954 | 3.785374989 | SMAD3;AC | . |
| 0 | 0 | 2.616672954 | 3.785374989 | DPYS;DPYD | . |
| 0 | 0 | 2.616672954 | 3.785374989 | FSCN1;AIF1 | . |
| 0 | 0 | 2.616672954 | 3.785374989 | TAP2;TAP1 | . |
| 0 | 0 | 2.616672954 | 3.785374989 | RB1CC1;AT | . |
| 0 | 0 | 2.616672954 | 3.785374989 | ROCK1;ABC | . |
| 0 | 0 | 2.616672954 | 3.785374989 | LYN;PICALI | . |
| 0 | 0 | 2.616672954 | 3.785374989 | RNASE10;C | . |
| 0 | 0 | 2.616672954 | 3.785374989 | RNASE10;C | . |
| 0 | 0 | 2.616672954 | 3.785374989 | HERPUD1;I | . |
| 0 | 0 | 2.616672954 | 3.785374989 | RNASE10;C | . |
| 0 | 0 | 2.616672954 | 3.785374989 | EGR1;HIF1 | . |
| 0 | 0 | 2.616672954 | 3.785374989 | CYP4F2;IL1 | . |
| 0 | 0 | 2.616672954 | 3.785374989 | IL9;RARA | . |
| 0 | 0 | 2.616672954 | 3.785374989 | SRC;ENPP2 | . |
| 0 | 0 | 2.616672954 | 3.785374989 | NR4A3;NEC | . |
| 0 | 0 | 2.616672954 | 3.785374989 | AGO2;STAT | . |
| 0 | 0 | 2.616672954 | 3.785374989 | CLN3;ANKF | . |
| 0 | 0 | 2.616672954 | 3.785374989 | FSCN1;TNF | . |
| 0 | 0 | 2.616672954 | 3.785374989 | ZBP1;NLRC | . |
| 0 | 0 | 2.616672954 | 3.785374989 | HYAL2;INP | . |
| 0 | 0 | 2.616672954 | 3.785374989 | ANXA1;AN | . |
| 0 | 0 | 2.616672954 | 3.785374989 | SEC16B;LR | . |
| 0 | 0 | 2.616672954 | 3.785374989 | SIGLEC1;TF | . |
| 0 | 0 | 2.616672954 | 3.785374989 | STAU1;STA | . |
| 0 | 0 | 2.616672954 | 3.785374989 | PARP10;TA | . |
| 0 | 0 | 2.616672954 | 3.785374989 | FGF18;CXC | . |
| 0 | 0 | 2.616672954 | 3.785374989 | CXCL8;ITGA | . |
| 0 | 0 | 2.616672954 | 3.785374989 | RGCC;SMA | . |
| 0 | 0 | 2.616672954 | 3.785374989 | PHF1;SUPT | . |
| 0 | 0 | 2.616672954 | 3.785374989 | EGR1;PPTC | . |
| 0 | 0 | 2.616672954 | 3.785374989 | RIOK3;DHX | . |
| 0 | 0 | 2.616672954 | 3.785374989 | AGO2;STAT | . |

| | | | | | |
|---|---|---|---|---|---|
| 0 | 0 | 2.616672954 | 3.785374989 | TIFAB;SFM | . |
| 0 | 0 | 2.616672954 | 3.785374989 | RFK;SLAMF | . |
| 0 | 0 | 2.616672954 | 3.785374989 | HIF1A;MCL | . |
| 0 | 0 | 2.616672954 | 3.785374989 | TIFAB;SRI | . |
| 0 | 0 | 2.616672954 | 3.785374989 | SCN4A;KCN | . |
| 0 | 0 | 2.616672954 | 3.785374989 | NRG1;SIK1 | . |
| 0 | 0 | 2.616672954 | 3.785374989 | SMAD3;HII | . |
| 0 | 0 | 2.616672954 | 3.785374989 | E2F3;SOD2 | . |
| 0 | 0 | 2.616672954 | 3.785374989 | RYR1;SLC8 | . |
| 0 | 0 | 2.616672954 | 3.785374989 | B2M;TLR2 | . |
| 0 | 0 | 2.616672954 | 3.785374989 | ATP6V1G1 | . |
| 0 | 0 | 2.616672954 | 3.785374989 | PRPSAP1;T | . |
| 0 | 0 | 2.616672954 | 3.785374989 | LTV1;NOP9 | . |
| 0 | 0 | 2.616672954 | 3.785374989 | DDX58;TRI | . |
| 0 | 0 | 2.616672954 | 3.785374989 | SLC13A3;S | . |
| 0 | 0 | 2.616672954 | 3.785374989 | GRK2;GRK5 | . |
| 0 | 0 | 2.616672954 | 3.785374989 | TP53RK;OS | . |
| 0 | 0 | 2.616672954 | 3.785374989 | CTBP2;SIRT | . |
| 0 | 0 | 1.155993616 | 1.66000731 | RET;NLGN3 | . |
| 0 | 0 | 1.744759029 | 2.499471086 | GCH1;MAC | . |
| 0 | 0 | 1.744759029 | 2.499471086 | UGCG;TME | . |
| 0 | 0 | 1.744759029 | 2.499471086 | CLIC4;PARI | . |
| 0 | 0 | 1.744759029 | 2.499471086 | S1PR1;NRG | . |
| 0 | 0 | 1.744759029 | 2.499471086 | ROCK1;FLC | . |
| 0 | 0 | 1.744759029 | 2.499471086 | SOD2;HIF1 | . |
| 0 | 0 | 1.744759029 | 2.499471086 | NRP1;HCK; | . |
| 0 | 0 | 1.744759029 | 2.499471086 | AMER1;CD | . |
| 0 | 0 | 1.744759029 | 2.499471086 | CEP120;ZN | . |
| 0 | 0 | 1.744759029 | 2.499471086 | P2RY12;CS | . |
| 0 | 0 | 1.744759029 | 2.499471086 | USP15;DH) | . |
| 0 | 0 | 1.744759029 | 2.499471086 | UGCG;TME | . |
| 0 | 0 | 1.744759029 | 2.499471086 | ASAH1;SPT | . |
| 0 | 0 | 1.415010474 | 2.013659601 | HDAC4;MII | . |
| 0 | 0 | 1.309125331 | 1.86009716 | DUSP10;RC | . |
| 0 | 0 | 1.309125331 | 1.86009716 | PIK3CA;AT | . |
| 0 | 0 | 1.309125331 | 1.86009716 | IL1A;STX1E | . |
| 0 | 0 | 1.962679245 | 2.77421427 | MYCBP2;A | . |
| 0 | 0 | 1.962679245 | 2.77421427 | SERTAD1;Z | . |
| 0 | 0 | 1.962679245 | 2.77421427 | ABT1;RCL1 | . |
| 0 | 0 | 1.962679245 | 2.77421427 | NIPBL;PIH1 | . |
| 0 | 0 | 1.962679245 | 2.77421427 | ELOVL4;EL | . |
| 0 | 0 | 1.962679245 | 2.77421427 | SLC2A10;S | . |
| 0 | 0 | 1.962679245 | 2.77421427 | ABCA1;AP( | . |
| 0 | 0 | 1.962679245 | 2.77421427 | CD46;GNL1 | . |
| 0 | 0 | 1.962679245 | 2.77421427 | LRRK2;PHF | . |
| 0 | 0 | 1.962679245 | 2.77421427 | PGP;SIK1;C | . |
| 0 | 0 | 1.962679245 | 2.77421427 | HDAC4;XBI | . |

| | | | | |
|---|---|---|---|---|
| 0 | 0 | 1.962679245 | 2.77421427 | USP15;TGF |
| 0 | 0 | 1.962679245 | 2.77421427 | RPIA;TKT;R |
| 0 | 0 | 1.962679245 | 2.77421427 | ATXN3;HEF |
| 0 | 0 | 1.962679245 | 2.77421427 | NIPBL;PRKI |
| 0 | 0 | 1.962679245 | 2.77421427 | NIPBL;SEM |
| 0 | 0 | 1.962679245 | 2.77421427 | PRKAA1;DI |
| 0 | 0 | 1.962679245 | 2.77421427 | NOS2;TMS |
| 0 | 0 | 1.962679245 | 2.77421427 | P2RY12;DE |
| 0 | 0 | 1.962679245 | 2.77421427 | LRRK2;ISG |
| 0 | 0 | 1.962679245 | 2.77421427 | PNP;GDA;X |
| 0 | 0 | 1.962679245 | 2.77421427 | LRRK2;ENT |
| 0 | 0 | 1.962679245 | 2.77421427 | CLN3;CALC |
| 0 | 0 | 1.962679245 | 2.77421427 | SRI;CALM1 |
| 0 | 0 | 1.962679245 | 2.77421427 | IL9;RARA;L |
| 0 | 0 | 1.962679245 | 2.77421427 | SAFB2;SAF |
| 0 | 0 | 1.962679245 | 2.77421427 | DDX3X;KA |
| 0 | 0 | 1.962679245 | 2.77421427 | EDEM1;UB |
| 0 | 0 | 1.962679245 | 2.77421427 | SLC11A2;H |
| 0 | 0 | 1.962679245 | 2.77421427 | LMO2;KLF |
| 0 | 0 | 1.962679245 | 2.77421427 | LTV1;EIF6; |
| 0 | 0 | 1.962679245 | 2.77421427 | ATP1A3;AT |
| 0 | 0 | 1.962679245 | 2.77421427 | ASAH1;SPT |
| 0 | 0 | 1.962679245 | 2.77421427 | ASAH1;SPT |
| 0 | 0 | 1.962679245 | 2.77421427 | NF1;DLL1; |
| 0 | 0 | 1.962679245 | 2.77421427 | ELOVL4;EL |
| 0 | 0 | 1.510069455 | 2.126339549 | POLR2A;PC |
| 0 | 0 | 1.510069455 | 2.126339549 | POLR2A;PC |
| 0 | 0 | 1.510069455 | 2.126339549 | P2RY12;P2 |
| 0 | 0 | 1.510069455 | 2.126339549 | JUN;CCNT1 |
| 0 | 0 | 1.510069455 | 2.126339549 | ACVRL1;AD |
| 0 | 0 | 1.510069455 | 2.126339549 | ACVRL1;CD |
| 0 | 0 | 1.510069455 | 2.126339549 | PIK3CA;SR |
| 0 | 0 | 1.510069455 | 2.126339549 | OTUB2;OT |
| 0 | 0 | 1.510069455 | 2.126339549 | RBM3;ROC |
| 0 | 0 | 1.510069455 | 2.126339549 | VPS4B;CEP |
| 0 | 0 | 1.510069455 | 2.126339549 | TENM1;PLI |
| 0 | 0 | 1.510069455 | 2.126339549 | IFNG;SLC6 |
| 0 | 0 | 1.369806143 | 1.916441541 | PAK1;LRRK |
| 0 | 0 | 1.369806143 | 1.916441541 | MAD2L2;SI |
| 0 | 0 | 1.246943677 | 1.73865497 | CREBBP;SN |
| 0 | 0 | 1.200411757 | 1.669833973 | PPP1R15A; |
| 0 | 0 | 1.431464947 | 1.961918867 | SDCBP;EDN |
| 0 | 0 | 1.431464947 | 1.961918867 | GK5;IPMK; |
| 0 | 0 | 1.431464947 | 1.961918867 | MTMR3;SY |
| 0 | 0 | 1.431464947 | 1.961918867 | RAP1B;PAF |
| 0 | 0 | 1.431464947 | 1.961918867 | DYNC1H1; |
| 0 | 0 | 1.431464947 | 1.961918867 | RNF31;USF |

| | | | | |
|---|---|---|---|---|
| 0 | 0 | 1.557869371 | 2.11737118 | NRROS;NF |
| 0 | 0 | 1.557869371 | 2.11737118 | CASP3;DVL |
| 0 | 0 | 1.557869371 | 2.11737118 | SCN9A;SCN |
| 0 | 0 | 1.557869371 | 2.11737118 | PNPT1;PAF |
| 0 | 0 | 1.557869371 | 2.11737118 | IL6;IL1B;RF |
| 0 | 0 | 1.557869371 | 2.11737118 | GDI1;TMBI |
| 0 | 0 | 1.557869371 | 2.11737118 | SEC16B;M |
| 0 | 0 | 1.557869371 | 2.11737118 | PNPLA8;M |
| 0 | 0 | 1.557869371 | 2.11737118 | CHST11;CS |
| 0 | 0 | 1.557869371 | 2.11737118 | CYP27B1;R |
| 0 | 0 | 1.557869371 | 2.11737118 | PNPT1;PAF |
| 0 | 0 | 1.255332292 | 1.692480673 | SLC24A1;T |
| 0 | 0 | 1.255332292 | 1.692480673 | SLC24A1;S |
| 0 | 0 | 1.377693762 | 1.853312765 | NLGN3;AT |
| 0 | 0 | 1.101219508 | 1.453304074 | ERO1A;GA |
| 0 | 0 | 1.094886527 | 1.442645133 | ZNF331;AC |
| 0 | 0 | 1.454057004 | 1.913890126 | SLC44A3;C |
| 0 | 0 | 1.454057004 | 1.913890126 | NFKBIA;AB |
| 0 | 0 | 1.308891411 | 1.721590997 | MAPK8;RC |
| 0 | 0 | 1.635617214 | 2.144681054 | ATXN3;RNI |
| 0 | 0 | 1.635617214 | 2.144681054 | DAXX;RIOK |
| 0 | 0 | 1.635617214 | 2.144681054 | ADRB3;PHI |
| 0 | 0 | 1.635617214 | 2.144681054 | NEU1;B4G |
| 0 | 0 | 1.635617214 | 2.144681054 | XBP1;NIPB |
| 0 | 0 | 1.635617214 | 2.144681054 | DDX17;TU |
| 0 | 0 | 1.635617214 | 2.144681054 | VPS4B;CHN |
| 0 | 0 | 1.635617214 | 2.144681054 | XBP1;PPP2 |
| 0 | 0 | 1.635617214 | 2.144681054 | ZFYVE16;N |
| 0 | 0 | 1.635617214 | 2.144681054 | NRP1;PLAU |
| 0 | 0 | 1.635617214 | 2.144681054 | SLC22A3;S |
| 0 | 0 | 1.238296652 | 1.603206118 | RET;FZD1;I |
| 0 | 0 | 1.209569011 | 1.565795731 | ABCA1;NC |
| 0 | 0 | 1.388007055 | 1.789367615 | GGT5;ALO |
| 0 | 0 | 1.388007055 | 1.789367615 | MAPK8;MI |
| 0 | 0 | 1.388007055 | 1.789367615 | MAD2L2;P |
| 0 | 0 | 1.784150943 | 2.262557668 | CXCL10;DD |
| 0 | 0 | 1.784150943 | 2.262557668 | NRP1;NOT |
| 0 | 0 | 1.784150943 | 2.262557668 | ADCY4;AD |
| 0 | 0 | 1.784150943 | 2.262557668 | MBD2;CHD |
| 0 | 0 | 1.784150943 | 2.262557668 | LYN;FGR;C |
| 0 | 0 | 1.784150943 | 2.262557668 | APOA2;PL |
| 0 | 0 | 1.784150943 | 2.262557668 | EFEMP1;TC |
| 0 | 0 | 1.784150943 | 2.262557668 | PTPRC;TNF |
| 0 | 0 | 1.784150943 | 2.262557668 | SERPINE1; |
| 0 | 0 | 1.784150943 | 2.262557668 | ID2;DLL1;T |
| 0 | 0 | 1.784150943 | 2.262557668 | PRKCB;NEK |
| 0 | 0 | 1.784150943 | 2.262557668 | ENTPD1;EN |

| | | | | | |
|---|---|---|---|---|---|
| 0 | 0 | 1.784150943 | 2.262557668 | PPP1R15B; | . |
| 0 | 0 | 1.784150943 | 2.262557668 | SLC9A3R1; | . |
| 0 | 0 | 1.784150943 | 2.262557668 | PIM1;GLI1 | . |
| 0 | 0 | 1.784150943 | 2.262557668 | STX4;ADCY | . |
| 0 | 0 | 1.784150943 | 2.262557668 | ADCY8;ZD | . |
| 0 | 0 | 1.784150943 | 2.262557668 | SETX;CALC | . |
| 0 | 0 | 1.784150943 | 2.262557668 | ITGAM;CLE | . |
| 0 | 0 | 1.784150943 | 2.262557668 | TUT7;AGO | . |
| 0 | 0 | 1.784150943 | 2.262557668 | DESI1;UBA | . |
| 0 | 0 | 1.784150943 | 2.262557668 | MOV10;SE | . |
| 0 | 0 | 1.784150943 | 2.262557668 | PIH1D1;PA | . |
| 0 | 0 | 1.784150943 | 2.262557668 | NIPBL;ANX | . |
| 0 | 0 | 1.784150943 | 2.262557668 | SLCO3A1;S | . |
| 0 | 0 | 1.784150943 | 2.262557668 | HSD11B1; | . |
| 0 | 0 | 1.784150943 | 2.262557668 | RIPK3;HES | . |
| 0 | 0 | 1.486971299 | 1.87327845 | EFEMP1;N | . |
| 0 | 0 | 1.486971299 | 1.87327845 | CLN3;TMB | . |
| 0 | 0 | 1.486971299 | 1.87327845 | GHSR;EDN | . |
| 0 | 0 | 1.486971299 | 1.87327845 | RAP1B;RO | . |
| 0 | 0 | 1.486971299 | 1.87327845 | IL6;NF1;PR | . |
| 0 | 0 | 1.308774584 | 1.648525362 | CCDC88B; | . |
| 0 | 0 | 1.283152686 | 1.606293422 | LYN;PTPN1 | . |
| 0 | 0 | 1.262932122 | 1.574801439 | PPP1R15B; | . |
| 0 | 0 | 2.180435056 | 2.715627452 | GCH1;PSA | . |
| 0 | 0 | 2.180435056 | 2.715627452 | EPHX2;TET | . |
| 0 | 0 | 2.180435056 | 2.715627452 | CLN3;RUB | . |
| 0 | 0 | 2.180435056 | 2.715627452 | NRP1;ALC | . |
| 0 | 0 | 2.180435056 | 2.715627452 | NRP1;BCA | . |
| 0 | 0 | 2.180435056 | 2.715627452 | EZH1;EZH2 | . |
| 0 | 0 | 2.180435056 | 2.715627452 | NRP1;VEG | . |
| 0 | 0 | 2.180435056 | 2.715627452 | GCLC;GGT | . |
| 0 | 0 | 2.180435056 | 2.715627452 | ABT1;NOP | . |
| 0 | 0 | 2.180435056 | 2.715627452 | SRC;GRB2 | . |
| 0 | 0 | 2.180435056 | 2.715627452 | NIPBL;PIH1 | . |
| 0 | 0 | 2.180435056 | 2.715627452 | CD81;COR | . |
| 0 | 0 | 2.180435056 | 2.715627452 | ACVRL1;PD | . |
| 0 | 0 | 2.180435056 | 2.715627452 | NR4A3;PIK | . |
| 0 | 0 | 2.180435056 | 2.715627452 | NR4A3;PIK | . |
| 0 | 0 | 2.180435056 | 2.715627452 | HORMAD1 | . |
| 0 | 0 | 2.180435056 | 2.715627452 | SLC22A3;S | . |
| 0 | 0 | 2.180435056 | 2.715627452 | ANXA1;RO | . |
| 0 | 0 | 2.180435056 | 2.715627452 | PTPRC;TO | . |
| 0 | 0 | 2.180435056 | 2.715627452 | CD46;CD5 | . |
| 0 | 0 | 2.180435056 | 2.715627452 | VDR;KLF9 | . |
| 0 | 0 | 2.180435056 | 2.715627452 | PTPRC;LDL | . |
| 0 | 0 | 2.180435056 | 2.715627452 | ZC3H12A; | . |
| 0 | 0 | 2.180435056 | 2.715627452 | ID2;EREG | . |

| | | | | | |
|---|---|---|---|---|---|
| 0 | 0 | 2.180435056 | 2.715627452 | MN1;EIF2A | . |
| 0 | 0 | 2.180435056 | 2.715627452 | RGS2;ANXA | . |
| 0 | 0 | 2.180435056 | 2.715627452 | LRRK2;NFE | . |
| 0 | 0 | 2.180435056 | 2.715627452 | PPP1R10;N | . |
| 0 | 0 | 2.180435056 | 2.715627452 | PTPRC;FCG | . |
| 0 | 0 | 2.180435056 | 2.715627452 | NRP1;ALCA | . |
| 0 | 0 | 2.180435056 | 2.715627452 | SLC22A3;S | . |
| 0 | 0 | 2.180435056 | 2.715627452 | CLEC5A;LR | . |
| 0 | 0 | 2.180435056 | 2.715627452 | HDAC4;SIR | . |
| 0 | 0 | 2.180435056 | 2.715627452 | RIPK1;MAF | . |
| 0 | 0 | 2.180435056 | 2.715627452 | PIK3CA;PIK | . |
| 0 | 0 | 2.180435056 | 2.715627452 | PLSCR2;PLS | . |
| 0 | 0 | 2.180435056 | 2.715627452 | LPCAT2;PL | . |
| 0 | 0 | 2.180435056 | 2.715627452 | LYN;PICALI | . |
| 0 | 0 | 2.180435056 | 2.715627452 | ID2;HES1 | . |
| 0 | 0 | 2.180435056 | 2.715627452 | PRKD2;VEC | . |
| 0 | 0 | 2.180435056 | 2.715627452 | ACTN2;ZDH | . |
| 0 | 0 | 2.180435056 | 2.715627452 | ITGAM;NO | . |
| 0 | 0 | 2.180435056 | 2.715627452 | NR1H2;NR | . |
| 0 | 0 | 2.180435056 | 2.715627452 | C3;RAB31 | . |
| 0 | 0 | 2.180435056 | 2.715627452 | PROK2;ENC | . |
| 0 | 0 | 2.180435056 | 2.715627452 | ANXA1;SIR | . |
| 0 | 0 | 2.180435056 | 2.715627452 | ATP11A;M | . |
| 0 | 0 | 2.180435056 | 2.715627452 | CXCR4;TNF | . |
| 0 | 0 | 2.180435056 | 2.715627452 | DTX3L;EZR | . |
| 0 | 0 | 2.180435056 | 2.715627452 | DTX3L;EZR | . |
| 0 | 0 | 2.180435056 | 2.715627452 | IFNG;TNF | . |
| 0 | 0 | 2.180435056 | 2.715627452 | DOCK4;NR | . |
| 0 | 0 | 2.180435056 | 2.715627452 | MAPRE1;M | . |
| 0 | 0 | 2.180435056 | 2.715627452 | MAPRE1;M | . |
| 0 | 0 | 2.180435056 | 2.715627452 | HOXC9;DLI | . |
| 0 | 0 | 2.180435056 | 2.715627452 | DPYS;DPYD | . |
| 0 | 0 | 2.180435056 | 2.715627452 | PNP;PRDM | . |
| 0 | 0 | 2.180435056 | 2.715627452 | IRF1;CCR2 | . |
| 0 | 0 | 2.180435056 | 2.715627452 | VMP1;TLR9 | . |
| 0 | 0 | 2.180435056 | 2.715627452 | TARM1;CD | . |
| 0 | 0 | 2.180435056 | 2.715627452 | ABCA1;NRI | . |
| 0 | 0 | 2.180435056 | 2.715627452 | EDN1;HIF1 | . |
| 0 | 0 | 2.180435056 | 2.715627452 | SEMA4D;N | . |
| 0 | 0 | 2.180435056 | 2.715627452 | FYN;FCGR2 | . |
| 0 | 0 | 2.180435056 | 2.715627452 | PRKCD;SIRT | . |
| 0 | 0 | 2.180435056 | 2.715627452 | RPS7;MDM | . |
| 0 | 0 | 2.180435056 | 2.715627452 | HECW2;NE | . |
| 0 | 0 | 2.180435056 | 2.715627452 | FSCN1;CDH | . |
| 0 | 0 | 2.180435056 | 2.715627452 | TSFM;ALKE | . |
| 0 | 0 | 2.180435056 | 2.715627452 | F3;VEGFA | . |
| 0 | 0 | 2.180435056 | 2.715627452 | DTX3L;EZR | . |

| | | | | |
|---|---|---|---|---|
| 0 | 0 | 2.180435056 | 2.715627452 | SLC9A3R1; . |
| 0 | 0 | 2.180435056 | 2.715627452 | IL10;PLAU . |
| 0 | 0 | 2.180435056 | 2.715627452 | ETF1;SHFL . |
| 0 | 0 | 2.180435056 | 2.715627452 | NRP1;BCAI . |
| 0 | 0 | 2.180435056 | 2.715627452 | GPX3;PRK( . |
| 0 | 0 | 2.180435056 | 2.715627452 | LIG4;SIRT1 . |
| 0 | 0 | 2.180435056 | 2.715627452 | ANXA1;CFI . |
| 0 | 0 | 2.180435056 | 2.715627452 | ELL;SNAPC . |
| 0 | 0 | 2.180435056 | 2.715627452 | ADAM17;R . |
| 0 | 0 | 2.180435056 | 2.715627452 | INSIG1;ARI . |
| 0 | 0 | 2.180435056 | 2.715627452 | CRACR2B;( . |
| 0 | 0 | 2.180435056 | 2.715627452 | RAMP2;HE . |
| 0 | 0 | 1.246569662 | 1.55092379 | SETX;SPREI . |
| 0 | 0 | 1.158207078 | 1.426524148 | AMER1;DD . |
| 0 | 0 | 1.402045442 | 1.724949768 | BCR;LAMB . |
| 0 | 0 | 1.402045442 | 1.724949768 | MAPK8;CA . |
| 0 | 0 | 1.402045442 | 1.724949768 | TAF1A;UBT . |
| 0 | 0 | 1.347105509 | 1.633235707 | SLC6A6;SL( . |
| 0 | 0 | 1.347105509 | 1.633235707 | PPP1R15A; . |
| 0 | 0 | 1.308657845 | 1.572183904 | NIPBL;DOP . |
| 0 | 0 | 1.308657845 | 1.572183904 | SYS1;RAB3 . |
| 0 | 0 | 1.539315613 | 1.848647545 | SUN2;ACTI . |
| 0 | 0 | 1.539315613 | 1.848647545 | ACTA2;SLC . |
| 0 | 0 | 1.539315613 | 1.848647545 | ELOVL5;PN . |
| 0 | 0 | 1.539315613 | 1.848647545 | SLC24A1;R . |
| 0 | 0 | 1.539315613 | 1.848647545 | MAD2L2;IK . |
| 0 | 0 | 1.539315613 | 1.848647545 | MAD2L2;IK . |
| 0 | 0 | 1.539315613 | 1.848647545 | RASSF2;DD . |
| 0 | 0 | 1.539315613 | 1.848647545 | TRPC5;LIG4 . |
| 0 | 0 | 1.539315613 | 1.848647545 | NXF1;DDX1 . |
| 0 | 0 | 1.539315613 | 1.848647545 | NIBAN2;IFI . |
| 0 | 0 | 1.539315613 | 1.848647545 | PIM1;GLI1; . |
| 0 | 0 | 1.539315613 | 1.848647545 | GRIN3A;FN . |
| 0 | 0 | 1.539315613 | 1.848647545 | MAD2L2;F1 . |
| 0 | 0 | 1.539315613 | 1.848647545 | CREB1;AD( . |
| 0 | 0 | 1.539315613 | 1.848647545 | SCN9A;CCL . |
| 0 | 0 | 1.539315613 | 1.848647545 | MAD2L2;IK . |
| 0 | 0 | 1.539315613 | 1.848647545 | SLC30A1;S . |
| 0 | 0 | 1.189988623 | 1.427104427 | TCF7L2;MI . |
| 0 | 0 | 1.197206864 | 1.431715524 | KDM6B;DY . |
| 0 | 0 | 1.280248964 | 1.529581205 | IFIH1;RANI . |
| 0 | 0 | 1.205570292 | 1.438311136 | DDX17;DD . |
| 0 | 0 | 1.241085646 | 1.476443601 | RYR1;MEF2 . |
| 0 | 0 | 1.241085646 | 1.476443601 | NPEPPS;EG . |
| 0 | 0 | 1.422238277 | 1.660961639 | PNPLA8;EN . |
| 0 | 0 | 1.422238277 | 1.660961639 | PHKG1;PHI . |
| 0 | 0 | 1.422238277 | 1.660961639 | ZNF473;TU . |

| | | | | |
|---|---|---|---|---|
| 0 | 0 | 1.422238277 | 1.660961639 | B3GNT3;B3 |
| 0 | 0 | 1.422238277 | 1.660961639 | PPP1R15A; |
| 0 | 0 | 1.422238277 | 1.660961639 | ATP6V1G1 |
| 0 | 0 | 1.422238277 | 1.660961639 | EDN1;CD8( |
| 0 | 0 | 1.422238277 | 1.660961639 | CD74;CDKI |
| 0 | 0 | 1.422238277 | 1.660961639 | ZFYVE16;C |
| 0 | 0 | 1.422238277 | 1.660961639 | CLEC7A;PL |
| 0 | 0 | 1.422238277 | 1.660961639 | TSFM;CCN |
| 0 | 0 | 1.422238277 | 1.660961639 | RET;IL1A;IL |
| 0 | 0 | 1.422238277 | 1.660961639 | NIPBL;OAS |
| 0 | 0 | 1.422238277 | 1.660961639 | URI1;RIPK3 |
| 0 | 0 | 1.422238277 | 1.660961639 | MBOAT1;P |
| 0 | 0 | 1.422238277 | 1.660961639 | TRIR;ZC3H |
| 0 | 0 | 1.422238277 | 1.660961639 | CD74;FCER |
| 0 | 0 | 1.353620885 | 1.556515035 | KAT2B;LRR |
| 0 | 0 | 1.353620885 | 1.556515035 | RIPOR2;FL( |
| 0 | 0 | 1.353620885 | 1.556515035 | MTMR3;LR |
| 0 | 0 | 1.353620885 | 1.556515035 | DYNC1H1;I |
| 0 | 0 | 1.182714177 | 1.359391296 | EGLN3;AG/ |
| 0 | 0 | 1.308541194 | 1.491749145 | ING5;KAT2 |
| 0 | 0 | 1.308541194 | 1.491749145 | SOCS3;SM( |
| 0 | 0 | 1.635377358 | 1.864075099 | IL6;LBP;IL6 |
| 0 | 0 | 1.635377358 | 1.864075099 | P2RY12;P2 |
| 0 | 0 | 1.635377358 | 1.864075099 | ATP7A;ARI |
| 0 | 0 | 1.635377358 | 1.864075099 | SRC;ZYX;PI |
| 0 | 0 | 1.635377358 | 1.864075099 | DUSP5;DU |
| 0 | 0 | 1.635377358 | 1.864075099 | TMEM129; |
| 0 | 0 | 1.635377358 | 1.864075099 | DYNC1H1;I |
| 0 | 0 | 1.635377358 | 1.864075099 | LRRK2;ENT |
| 0 | 0 | 1.635377358 | 1.864075099 | GCH1;MO( |
| 0 | 0 | 1.635377358 | 1.864075099 | KAT8;JADE |
| 0 | 0 | 1.635377358 | 1.864075099 | KAT8;JADE |
| 0 | 0 | 1.635377358 | 1.864075099 | ALOX5AP;I |
| 0 | 0 | 1.635377358 | 1.864075099 | ALOX5AP;/ |
| 0 | 0 | 1.635377358 | 1.864075099 | WTAP;TRM |
| 0 | 0 | 1.635377358 | 1.864075099 | XBP1;LRRK |
| 0 | 0 | 1.635377358 | 1.864075099 | PTPRC;LGA |
| 0 | 0 | 1.635377358 | 1.864075099 | ARG2;GCH |
| 0 | 0 | 1.635377358 | 1.864075099 | ENTPD1;EI |
| 0 | 0 | 1.635377358 | 1.864075099 | TEC;PTK2B |
| 0 | 0 | 1.635377358 | 1.864075099 | CHKA;ETNI |
| 0 | 0 | 1.635377358 | 1.864075099 | PPP1R10;L |
| 0 | 0 | 1.635377358 | 1.864075099 | ZFP36;CNC |
| 0 | 0 | 1.635377358 | 1.864075099 | IFIH1;CD74 |
| 0 | 0 | 1.635377358 | 1.864075099 | ACTB;ACT( |
| 0 | 0 | 1.635377358 | 1.864075099 | DGKD;HIP1 |
| 0 | 0 | 1.635377358 | 1.864075099 | MOV10;D\ |

| | | | | |
|---|---|---|---|---|
| 0 | 0 | 1.635377358 | 1.864075099 | ZFP36;CNC |
| 0 | 0 | 1.635377358 | 1.864075099 | OAS2;NRG |
| 0 | 0 | 1.635377358 | 1.864075099 | ITGAM;CLE |
| 0 | 0 | 1.635377358 | 1.864075099 | ZC3H12C;K |
| 0 | 0 | 1.635377358 | 1.864075099 | SRC;ZYX;PI |
| 0 | 0 | 1.635377358 | 1.864075099 | SYCP1;HOF |
| 0 | 0 | 1.635377358 | 1.864075099 | SYCP1;HOF |
| 0 | 0 | 1.635377358 | 1.864075099 | CYP27B1;C |
| 0 | 0 | 1.208073865 | 1.373992943 | SLC24A1;K |
| 0 | 0 | 1.276665591 | 1.448944247 | SDC4;HAS1 |
| 0 | 0 | 1.276665591 | 1.448944247 | MYLIP;STX |
| 0 | 0 | 1.219979728 | 1.384286953 | CREBBP;FN |
| 0 | 0 | 1.219979728 | 1.384286953 | NRP1;CSF1 |
| 0 | 0 | 1.219979728 | 1.384286953 | PPP1R16B; |
| 0 | 0 | 1.234589053 | 1.398898695 | OTUD4;DN |
| 0 | 0 | 1.083959968 | 1.217628828 | CSF1;CHM |
| 0 | 0 | 1.158901587 | 1.294639783 | GGT5;CHPI |
| 0 | 0 | 1.120767394 | 1.235076485 | DDX5;SMG |
| 0 | 0 | 1.45371419 | 1.599816901 | SLC6A6;SL |
| 0 | 0 | 1.45371419 | 1.599816901 | ALAS1;FLV |
| 0 | 0 | 1.45371419 | 1.599816901 | TEX2;SMPI |
| 0 | 0 | 1.45371419 | 1.599816901 | MICU1;SLC |
| 0 | 0 | 1.45371419 | 1.599816901 | CEBPG;PLE |
| 0 | 0 | 1.45371419 | 1.599816901 | HEXIM1;CI |
| 0 | 0 | 1.45371419 | 1.599816901 | DAXX;POLI |
| 0 | 0 | 1.45371419 | 1.599816901 | MAD2L2;IK |
| 0 | 0 | 1.45371419 | 1.599816901 | NRP1;SEM |
| 0 | 0 | 1.45371419 | 1.599816901 | IL1B;PRKCI |
| 0 | 0 | 1.45371419 | 1.599816901 | VPS4B;CHN |
| 0 | 0 | 1.45371419 | 1.599816901 | DAXX;POLI |
| 0 | 0 | 1.45371419 | 1.599816901 | CSF1;SLAM |
| 0 | 0 | 1.45371419 | 1.599816901 | RNF126;VF |
| 0 | 0 | 1.45371419 | 1.599816901 | SLC30A1;S |
| 0 | 0 | 1.362899673 | 1.475591836 | TLE4;TCF7I |
| 0 | 0 | 1.362899673 | 1.475591836 | SLC13A3;P |
| 0 | 0 | 1.362899673 | 1.475591836 | HK3;PKLR;I |
| 0 | 0 | 1.362899673 | 1.475591836 | P2RY12;PT |
| 0 | 0 | 1.362899673 | 1.475591836 | CCL3;RARA |
| 0 | 0 | 1.362899673 | 1.475591836 | PGP;SIK1;S |
| 0 | 0 | 1.362899673 | 1.475591836 | ITGAM;GD |
| 0 | 0 | 1.362899673 | 1.475591836 | ARRDC4;D |
| 0 | 0 | 1.19957734 | 1.297914077 | DUSP11;P( |
| 0 | 0 | 1.868836558 | 2.017189951 | ADA2;XDH |
| 0 | 0 | 1.868836558 | 2.017189951 | SDS;SDSL |
| 0 | 0 | 1.868836558 | 2.017189951 | ABCA7;PSE |
| 0 | 0 | 1.868836558 | 2.017189951 | PIK3CA;BN |
| 0 | 0 | 1.868836558 | 2.017189951 | LIMK2;EZR |

| | | | | | |
|---|---|---|---|---|---|
| 0 | 0 | 1.868836558 | 2.017189951 | RAMP2;LA | . |
| 0 | 0 | 1.868836558 | 2.017189951 | ANXA2;SR | . |
| 0 | 0 | 1.868836558 | 2.017189951 | ADCY8;CFT | . |
| 0 | 0 | 1.868836558 | 2.017189951 | TRAF3IP2;I | . |
| 0 | 0 | 1.868836558 | 2.017189951 | INSIG1;AR | . |
| 0 | 0 | 1.868836558 | 2.017189951 | C1S;C1R | . |
| 0 | 0 | 1.868836558 | 2.017189951 | NIPBL;ID2 | . |
| 0 | 0 | 1.868836558 | 2.017189951 | IL10;GSN | . |
| 0 | 0 | 1.868836558 | 2.017189951 | SNAI1;RBP | . |
| 0 | 0 | 1.868836558 | 2.017189951 | CLN3;CRLS | . |
| 0 | 0 | 1.868836558 | 2.017189951 | ISYNA1;ITP | . |
| 0 | 0 | 1.868836558 | 2.017189951 | TRAF3IP2;I | . |
| 0 | 0 | 1.868836558 | 2.017189951 | SRC;IL6ST | . |
| 0 | 0 | 1.868836558 | 2.017189951 | SLC2A1;SL | . |
| 0 | 0 | 1.868836558 | 2.017189951 | APOA2;OL | . |
| 0 | 0 | 1.868836558 | 2.017189951 | NR4A3;PIK | . |
| 0 | 0 | 1.868836558 | 2.017189951 | HIGD1A;C | . |
| 0 | 0 | 1.868836558 | 2.017189951 | ANXA1;VP | . |
| 0 | 0 | 1.868836558 | 2.017189951 | LYN;TNFAI | . |
| 0 | 0 | 1.868836558 | 2.017189951 | RGS2;APO | . |
| 0 | 0 | 1.868836558 | 2.017189951 | ZC3H12A;S | . |
| 0 | 0 | 1.868836558 | 2.017189951 | ZC3H12A;S | . |
| 0 | 0 | 1.868836558 | 2.017189951 | LDLRAD4;S | . |
| 0 | 0 | 1.868836558 | 2.017189951 | PRKCD;AL | . |
| 0 | 0 | 1.868836558 | 2.017189951 | UBAC2;UB | . |
| 0 | 0 | 1.868836558 | 2.017189951 | ADRB3;SO | . |
| 0 | 0 | 1.868836558 | 2.017189951 | TBX21;SM | . |
| 0 | 0 | 1.868836558 | 2.017189951 | MAD2L2;R | . |
| 0 | 0 | 1.868836558 | 2.017189951 | XRN1;DXO | . |
| 0 | 0 | 1.868836558 | 2.017189951 | ENTPD1;E | . |
| 0 | 0 | 1.868836558 | 2.017189951 | HSD11B1; | . |
| 0 | 0 | 1.868836558 | 2.017189951 | ARG2;ODC | . |
| 0 | 0 | 1.868836558 | 2.017189951 | PARD3;NF | . |
| 0 | 0 | 1.868836558 | 2.017189951 | PLAU;F11 | . |
| 0 | 0 | 1.868836558 | 2.017189951 | TMSB4X;E | . |
| 0 | 0 | 1.868836558 | 2.017189951 | PPP1R16B; | . |
| 0 | 0 | 1.868836558 | 2.017189951 | ADAM19;F | . |
| 0 | 0 | 1.868836558 | 2.017189951 | DTX3L;PAR | . |
| 0 | 0 | 1.868836558 | 2.017189951 | P2RY6;CD2 | . |
| 0 | 0 | 1.868836558 | 2.017189951 | MID1IP1;T | . |
| 0 | 0 | 1.868836558 | 2.017189951 | FGR;GAB2 | . |
| 0 | 0 | 1.868836558 | 2.017189951 | FITM1;OS | . |
| 0 | 0 | 1.868836558 | 2.017189951 | NR1H2;NR | . |
| 0 | 0 | 1.868836558 | 2.017189951 | GDA;DPYD | . |
| 0 | 0 | 1.868836558 | 2.017189951 | PIK3CA;AR | . |
| 0 | 0 | 1.868836558 | 2.017189951 | LYN;PICAL | . |
| 0 | 0 | 1.868836558 | 2.017189951 | CD274;IRF | . |

| | | | | |
|---|---|---|---|---|
| 0 | 0 | 1.868836558 | 2.017189951 | CD46;CD5 . |
| 0 | 0 | 1.868836558 | 2.017189951 | IL10;CD55 . |
| 0 | 0 | 1.868836558 | 2.017189951 | HERPUD1; . |
| 0 | 0 | 1.868836558 | 2.017189951 | RNASE10; . |
| 0 | 0 | 1.868836558 | 2.017189951 | PIM1;ACT . |
| 0 | 0 | 1.868836558 | 2.017189951 | ADCY8;CFT . |
| 0 | 0 | 1.868836558 | 2.017189951 | DCUN1D3; . |
| 0 | 0 | 1.868836558 | 2.017189951 | PARD3;NF . |
| 0 | 0 | 1.868836558 | 2.017189951 | NIPBL;STR . |
| 0 | 0 | 1.868836558 | 2.017189951 | AGO2;ADA . |
| 0 | 0 | 1.868836558 | 2.017189951 | LIG4;BATF . |
| 0 | 0 | 1.868836558 | 2.017189951 | C3;ITGAM . |
| 0 | 0 | 1.868836558 | 2.017189951 | MTHFD2; . |
| 0 | 0 | 1.211656227 | 1.305666431 | ABCA1;PR . |
| 0 | 0 | 1.211656227 | 1.305666431 | CELA2A;RA . |
| 0 | 0 | 1.308424632 | 1.406032598 | DUSP5;DU . |
| 0 | 0 | 1.308424632 | 1.406032598 | ILRUN;RIP . |
| 0 | 0 | 1.308424632 | 1.406032598 | TCF7L2;M . |
| 0 | 0 | 1.226763427 | 1.317673272 | PNPLA8;C . |
| 0 | 0 | 1.226763427 | 1.317673272 | NLGN3;NR . |
| 0 | 0 | 1.226763427 | 1.317673272 | IL1A;EDN1 . |
| 0 | 0 | 1.246196777 | 1.335838321 | SLC7A5;SL . |
| 0 | 0 | 1.246196777 | 1.335838321 | HSD3B7;P . |
| 0 | 0 | 1.246196777 | 1.335838321 | NRP1;TESK . |
| 0 | 0 | 1.272119342 | 1.363357559 | HK3;PRKA . |
| 0 | 0 | 1.272119342 | 1.363357559 | RET;LYN;S . |
| 0 | 0 | 1.272119342 | 1.363357559 | CDKN2D;P . |
| 0 | 0 | 1.272119342 | 1.363357559 | NCR1;CAS . |
| 0 | 0 | 1.272119342 | 1.363357559 | IL10;CLN3; . |
| 0 | 0 | 1.272119342 | 1.363357559 | P2RY12;P2 . |
| 0 | 0 | 1.272119342 | 1.363357559 | CNP;MAO . |
| 0 | 0 | 1.179843884 | 1.216939395 | NRP1;RILP . |
| 0 | 0 | 1.179843884 | 1.216939395 | TIAM2;IFN . |
| 0 | 0 | 1.179843884 | 1.216939395 | IL1A;TCF7 . |
| 0 | 0 | 1.509492017 | 1.548827907 | MOV10;ZF . |
| 0 | 0 | 1.509492017 | 1.548827907 | DFFB;CASP . |
| 0 | 0 | 1.509492017 | 1.548827907 | RB1CC1;W . |
| 0 | 0 | 1.509492017 | 1.548827907 | ZFP36;AN . |
| 0 | 0 | 1.509492017 | 1.548827907 | GRAMD1C . |
| 0 | 0 | 1.509492017 | 1.548827907 | ASAH1;AC . |
| 0 | 0 | 1.509492017 | 1.548827907 | PDPN;ARH . |
| 0 | 0 | 1.509492017 | 1.548827907 | B4GALNT1 . |
| 0 | 0 | 1.509492017 | 1.548827907 | SYS1;DOP1 . |
| 0 | 0 | 1.509492017 | 1.548827907 | DNMT3L;F . |
| 0 | 0 | 1.509492017 | 1.548827907 | ATP1B3;AT . |
| 0 | 0 | 1.509492017 | 1.548827907 | NIPBL;POG . |
| 0 | 0 | 1.509492017 | 1.548827907 | RHOG;RAC . |

| | | | | |
|---|---|---|---|---|
| 0 | 0 | 1.509492017 | 1.548827907 | ACVRL1;SR |
| 0 | 0 | 1.509492017 | 1.548827907 | ACVRL1;SR |
| 0 | 0 | 1.509492017 | 1.548827907 | NLGN3;RET |
| 0 | 0 | 1.509492017 | 1.548827907 | ARG2;GCH |
| 0 | 0 | 1.509492017 | 1.548827907 | ENTPD1;EN |
| 0 | 0 | 1.509492017 | 1.548827907 | JUN;CCNT1 |
| 0 | 0 | 1.509492017 | 1.548827907 | CEP83;SNX |
| 0 | 0 | 1.509492017 | 1.548827907 | AMPD1;AN |
| 0 | 0 | 1.509492017 | 1.548827907 | FLCN;TMSB |
| 0 | 0 | 1.509492017 | 1.548827907 | H1-4;MIER |
| 0 | 0 | 1.509492017 | 1.548827907 | RHBDF2;PT |
| 0 | 0 | 1.509492017 | 1.548827907 | IL18;RARA; |
| 0 | 0 | 1.509492017 | 1.548827907 | NEK6;HECV |
| 0 | 0 | 1.509492017 | 1.548827907 | FMR1;FLOT |
| 0 | 0 | 1.509492017 | 1.548827907 | ARHGAP17 |
| 0 | 0 | 1.189557321 | 1.218733686 | SLC4A7;SLC |
| 0 | 0 | 1.189557321 | 1.218733686 | BST1;CSF1; |
| 0 | 0 | 1.20165797 | 1.223317969 | ATP6V1G1 |
| 0 | 0 | 1.20165797 | 1.223317969 | SPTLC2;ELO |
| 0 | 0 | 1.20165797 | 1.223317969 | MYLIP;STX |
| 0 | 0 | 1.120085917 | 1.138609641 | NCOA1;CR |
| 0 | 0 | 1.217145118 | 1.231945449 | ABCA1;STA |
| 0 | 0 | 1.217145118 | 1.231945449 | PIK3CA;PTA |
| 0 | 0 | 1.145026525 | 1.156623429 | BTG2;EDN1 |
| 0 | 0 | 1.377123443 | 1.389307031 | DFFB;CASP |
| 0 | 0 | 1.377123443 | 1.389307031 | IL1A;IL1B;T |
| 0 | 0 | 1.377123443 | 1.389307031 | IL1A;IL1B;T |
| 0 | 0 | 1.377123443 | 1.389307031 | RNASEH2B |
| 0 | 0 | 1.377123443 | 1.389307031 | LAMP1;IL1 |
| 0 | 0 | 1.377123443 | 1.389307031 | DDX3X;RAS |
| 0 | 0 | 1.377123443 | 1.389307031 | ATAD1;FM |
| 0 | 0 | 1.377123443 | 1.389307031 | UTP15;PIH |
| 0 | 0 | 1.377123443 | 1.389307031 | DYNC1H1;( |
| 0 | 0 | 1.237666238 | 1.246701424 | SLC7A5;SLC |
| 0 | 0 | 1.266144266 | 1.271320939 | FLCN;NR4A |
| 0 | 0 | 1.266144266 | 1.271320939 | NRP1;SEM |
| 0 | 0 | 1.266144266 | 1.271320939 | NRP1;HCK; |
| 0 | 0 | 1.266144266 | 1.271320939 | CYLD;PHLD |
| 0 | 0 | 1.308308157 | 1.313202078 | SETX;CELF3 |
| 0 | 0 | 1.308308157 | 1.313202078 | VPS4B;CHN |
| 0 | 0 | 1.308308157 | 1.313202078 | CLN3;GSN; |
| 0 | 0 | 1.308308157 | 1.313202078 | GRK2;MYB |
| 0 | 0 | 1.308308157 | 1.313202078 | GRIN3A;PN |
| 0 | 0 | 1.308308157 | 1.313202078 | MT2A;TMC |
| 0 | 0 | 1.123587928 | 1.121176958 | SYS1;RIC1; |
| 0 | 0 | 1.16074699 | 1.141006347 | BCAR3;ATP |
| 0 | 0 | 1.16074699 | 1.141006347 | SLC22A3;ST |

| | | | | | |
|---|---|---|---|---|---|
| 0 | 0 | 1.168247662 | 1.137645184 | FLCN;RAM | . |
| 0 | 0 | 1.189413989 | 1.136441255 | GALC;UGC | . |
| 0 | 0 | 1.189413989 | 1.136441255 | ANXA1;RA | . |
| 0 | 0 | 1.205026455 | 1.140584761 | CDKN1A;IF | . |
| 0 | 0 | 1.205026455 | 1.140584761 | SEMA4A;SI | . |
| 0 | 0 | 1.205026455 | 1.140584761 | NLGN3;RG | . |
| 0 | 0 | 1.635137684 | 1.537388251 | NF1;STRA6 | . |
| 0 | 0 | 1.635137684 | 1.537388251 | MICU1;MC | . |
| 0 | 0 | 1.635137684 | 1.537388251 | CHP1;PKD | . |
| 0 | 0 | 1.635137684 | 1.537388251 | ATP6V1G1 | . |
| 0 | 0 | 1.635137684 | 1.537388251 | PTGFR;PTG | . |
| 0 | 0 | 1.635137684 | 1.537388251 | CA12;SLC1 | . |
| 0 | 0 | 1.635137684 | 1.537388251 | CSGALNAC | . |
| 0 | 0 | 1.635137684 | 1.537388251 | FCN1;MAS | . |
| 0 | 0 | 1.635137684 | 1.537388251 | LAPTM5;KI | . |
| 0 | 0 | 1.635137684 | 1.537388251 | ITGAM;SM | . |
| 0 | 0 | 1.635137684 | 1.537388251 | KDM6B;PE | . |
| 0 | 0 | 1.635137684 | 1.537388251 | LPCAT2;PL | . |
| 0 | 0 | 1.635137684 | 1.537388251 | EIF5;DENR | . |
| 0 | 0 | 1.635137684 | 1.537388251 | HK3;HK2 | . |
| 0 | 0 | 1.635137684 | 1.537388251 | DIRAS3;DN | . |
| 0 | 0 | 1.635137684 | 1.537388251 | PRAF2;SLC | . |
| 0 | 0 | 1.635137684 | 1.537388251 | SDS;PSAT1 | . |
| 0 | 0 | 1.635137684 | 1.537388251 | GPAT4;VE | . |
| 0 | 0 | 1.635137684 | 1.537388251 | FITM1;CDS | . |
| 0 | 0 | 1.635137684 | 1.537388251 | DGKG;DGK | . |
| 0 | 0 | 1.635137684 | 1.537388251 | VMP1;PRK | . |
| 0 | 0 | 1.635137684 | 1.537388251 | ENPP2;SM | . |
| 0 | 0 | 1.635137684 | 1.537388251 | NIN;NINL | . |
| 0 | 0 | 1.635137684 | 1.537388251 | TUT7;ISG1 | . |
| 0 | 0 | 1.635137684 | 1.537388251 | CA12;SLC1 | . |
| 0 | 0 | 1.635137684 | 1.537388251 | UBE2B;PD | . |
| 0 | 0 | 1.635137684 | 1.537388251 | PDE3B;SER | . |
| 0 | 0 | 1.635137684 | 1.537388251 | RIC1;ANXA | . |
| 0 | 0 | 1.635137684 | 1.537388251 | SFPQ;CRY2 | . |
| 0 | 0 | 1.635137684 | 1.537388251 | HDAC4;NU | . |
| 0 | 0 | 1.635137684 | 1.537388251 | DNAJB6;H | . |
| 0 | 0 | 1.635137684 | 1.537388251 | CD300A;SL | . |
| 0 | 0 | 1.635137684 | 1.537388251 | BCL6;PRKC | . |
| 0 | 0 | 1.635137684 | 1.537388251 | HDAC4;NU | . |
| 0 | 0 | 1.635137684 | 1.537388251 | HECW2;PR | . |
| 0 | 0 | 1.635137684 | 1.537388251 | HECW2;PR | . |
| 0 | 0 | 1.635137684 | 1.537388251 | PARP3;SRC | . |
| 0 | 0 | 1.635137684 | 1.537388251 | RIOK3;DH | . |
| 0 | 0 | 1.635137684 | 1.537388251 | RAPGEF1; | . |
| 0 | 0 | 1.635137684 | 1.537388251 | ATP1A3;PS | . |
| 0 | 0 | 1.635137684 | 1.537388251 | XRN1;DXO | . |

| | | | | |
|---|---|---|---|---|
| 0 | 0 | 1.635137684 | 1.537388251 | APOA2;ATI |
| 0 | 0 | 1.635137684 | 1.537388251 | CLN3;PLA2 |
| 0 | 0 | 1.635137684 | 1.537388251 | NR4A3;STF |
| 0 | 0 | 1.635137684 | 1.537388251 | TMEM79;S |
| 0 | 0 | 1.635137684 | 1.537388251 | STAT1;LRP |
| 0 | 0 | 1.635137684 | 1.537388251 | HDAC4;PIA |
| 0 | 0 | 1.635137684 | 1.537388251 | ANXA2;ECI |
| 0 | 0 | 1.635137684 | 1.537388251 | USP15;DH) |
| 0 | 0 | 1.635137684 | 1.537388251 | UTP15;RIO |
| 0 | 0 | 1.635137684 | 1.537388251 | ZNF143;EL |
| 0 | 0 | 1.635137684 | 1.537388251 | NLGN3;LRI |
| 0 | 0 | 1.635137684 | 1.537388251 | PUS10;SM, |
| 0 | 0 | 1.635137684 | 1.537388251 | NRP1;ARF6 |
| 0 | 0 | 1.635137684 | 1.537388251 | GALNT3;G, |
| 0 | 0 | 1.635137684 | 1.537388251 | CHM;RABG |
| 0 | 0 | 1.635137684 | 1.537388251 | RAP1B;RAI |
| 0 | 0 | 1.635137684 | 1.537388251 | DNMT3L;N |
| 0 | 0 | 1.635137684 | 1.537388251 | NIPBL;HPS |
| 0 | 0 | 1.635137684 | 1.537388251 | FMR1;IL1B |
| 0 | 0 | 1.635137684 | 1.537388251 | FMR1;ADC |
| 0 | 0 | 1.635137684 | 1.537388251 | DCUN1D3; |
| 0 | 0 | 1.635137684 | 1.537388251 | ADAM15;B |
| 0 | 0 | 1.635137684 | 1.537388251 | EFHB;SLC8 |
| 0 | 0 | 1.635137684 | 1.537388251 | HYAL2;INP |
| 0 | 0 | 1.635137684 | 1.537388251 | ATP7A;SOI |
| 0 | 0 | 1.635137684 | 1.537388251 | RPUSD4;G, |
| 0 | 0 | 1.635137684 | 1.537388251 | GSN;TMSB |
| 0 | 0 | 1.635137684 | 1.537388251 | NF1;STRA6 |
| 0 | 0 | 1.635137684 | 1.537388251 | ARG2;ASS1 |
| 0 | 0 | 1.226506422 | 1.150507542 | IL10;CD40; |
| 0 | 0 | 1.226506422 | 1.150507542 | SDCBP;USI |
| 0 | 0 | 1.226506422 | 1.150507542 | TCF7L2;ST) |
| 0 | 0 | 1.226506422 | 1.150507542 | PPP1R15A; |
| 0 | 0 | 1.257915989 | 1.170509433 | ERO1A;HSI |
| 0 | 0 | 1.257915989 | 1.170509433 | RAP1B;RAI |
| 0 | 0 | 1.257915989 | 1.170509433 | SEMA4A;SI |
| 0 | 0 | 1.257915989 | 1.170509433 | SDCBP;RYE |
| 0 | 0 | 1.257915989 | 1.170509433 | P2RY8;GPI |
| 0 | 0 | 1.257915989 | 1.170509433 | STAT5A;ST |
| 0 | 0 | 1.257915989 | 1.170509433 | CBLIF;SLC2 |
| 0 | 0 | 1.116969658 | 1.036584226 | ATP6V1G1 |
| 0 | 0 | 1.30819177 | 1.210197697 | DFFB;CASP |
| 0 | 0 | 1.30819177 | 1.210197697 | NLGN3;NR |
| 0 | 0 | 1.30819177 | 1.210197697 | TG;NF1;TG |
| 0 | 0 | 1.30819177 | 1.210197697 | RAMP2;LA |
| 0 | 0 | 1.30819177 | 1.210197697 | PIK3CA;AT |
| 0 | 0 | 1.30819177 | 1.210197697 | ZFYVE28;P |

| | | | | |
|---|---|---|---|---|
| 0 | 0 | 1.30819177 | 1.210197697 | ALAS1;FLV… |
| 0 | 0 | 1.30819177 | 1.210197697 | PIK3CA;AV… |
| 0 | 0 | 1.30819177 | 1.210197697 | MAN1A2;E… |
| 0 | 0 | 1.30819177 | 1.210197697 | PRPSAP1;P… |
| 0 | 0 | 1.30819177 | 1.210197697 | PRKAA1;D… |
| 0 | 0 | 1.401590296 | 1.296124775 | ADCY4;AD… |
| 0 | 0 | 1.401590296 | 1.296124775 | ATP1A3;AT… |
| 0 | 0 | 1.401590296 | 1.296124775 | GALNT11;S… |
| 0 | 0 | 1.401590296 | 1.296124775 | GGT5;GGT… |
| 0 | 0 | 1.401590296 | 1.296124775 | MT2A;XIAF… |
| 0 | 0 | 1.401590296 | 1.296124775 | RYBP;PCGF… |
| 0 | 0 | 1.401590296 | 1.296124775 | SCN4B;SLC… |
| 0 | 0 | 1.401590296 | 1.296124775 | SLC2A9;SL… |
| 0 | 0 | 1.401590296 | 1.296124775 | KLF7;MIDN… |
| 0 | 0 | 1.401590296 | 1.296124775 | F12;CASP4… |
| 0 | 0 | 1.401590296 | 1.296124775 | LRRK2;NF1… |
| 0 | 0 | 1.401590296 | 1.296124775 | ANXA2;ECI… |
| 0 | 0 | 1.401590296 | 1.296124775 | EGR1;ASAH… |
| 0 | 0 | 1.401590296 | 1.296124775 | NLRC5;NAI… |
| 0 | 0 | 1.401590296 | 1.296124775 | ABCA1;AP… |
| 0 | 0 | 1.401590296 | 1.296124775 | KCNK9;KCN… |
| 0 | 0 | 1.401590296 | 1.296124775 | PTPRC;NR… |
| 0 | 0 | 1.090454488 | 1.006984123 | MEF2A;RO… |
| 0 | 0 | 1.090420147 | 0.994942236 | HACE1;RO… |
| 0 | 0 | 1.162915354 | 1.048585958 | ACVRL1;NI… |
| 0 | 0 | 1.162915354 | 1.048585958 | SPTLC2;CA… |
| 0 | 0 | 1.124343537 | 1.002968994 | ITCH;PSMA… |
| 0 | 0 | 1.174060507 | 1.043952297 | FOLH1B;KY… |
| 0 | 0 | 1.174060507 | 1.043952297 | SETX;GCFC… |
| 0 | 0 | 1.174060507 | 1.043952297 | WASHC4;S… |
| 0 | 0 | 1.103448276 | 0.981145899 | SRGN;TESC… |
| 0 | 0 | 1.104437229 | 0.968050032 | SLC24A1;S… |
| 0 | 0 | 1.189270873 | 1.041978148 | TENM1;DY… |
| 0 | 0 | 1.189270873 | 1.041978148 | PTPN1;PTF… |
| 0 | 0 | 1.189270873 | 1.041978148 | CLN3;CCD… |
| 0 | 0 | 1.132135168 | 0.979589491 | PTPN1;ZFY… |
| 0 | 0 | 1.067168244 | 0.923178263 | MEF2A;AC… |
| 0 | 0 | 1.105599138 | 0.954450971 | NPC1;CHST… |
| 0 | 0 | 1.105599138 | 0.954450971 | ELOVL5;PN… |
| 0 | 0 | 1.211256574 | 1.044778641 | SLC9A3R1;… |
| 0 | 0 | 1.211256574 | 1.044778641 | XBP1;IL6;A… |
| 0 | 0 | 1.211256574 | 1.044778641 | SLC11A1;A… |
| 0 | 0 | 1.211256574 | 1.044778641 | CDKN1A;CI… |
| 0 | 0 | 1.211256574 | 1.044778641 | CNOT2;CRY… |
| 0 | 0 | 1.211256574 | 1.044778641 | ANXA1;MN… |
| 0 | 0 | 1.211256574 | 1.044778641 | CDK17;GFI… |
| 0 | 0 | 1.211256574 | 1.044778641 | XBP1;SESN… |

| | | | | |
|---|---|---|---|---|
| 0 | 0 | 1.211256574 | 1.044778641 | SETX;BRF2 |
| 0 | 0 | 1.211256574 | 1.044778641 | TAF1A;UB |
| 0 | 0 | 1.090248832 | 0.928552156 | CSF1;LRRK |
| 0 | 0 | 1.137568834 | 0.967714298 | SDC4;HYAL |
| 0 | 0 | 1.054037517 | 0.896005025 | CDKN1A;C |
| 0 | 0 | 1.245825019 | 1.057032387 | HAS1;CSG |
| 0 | 0 | 1.245825019 | 1.057032387 | CYP27B1;T |
| 0 | 0 | 1.245825019 | 1.057032387 | CYP27B1;C |
| 0 | 0 | 1.245825019 | 1.057032387 | FLCN;LIG4; |
| 0 | 0 | 1.245825019 | 1.057032387 | PXK;ACTN |
| 0 | 0 | 1.245825019 | 1.057032387 | CCL3;CCL2 |
| 0 | 0 | 1.245825019 | 1.057032387 | ATXN7;MI |
| 0 | 0 | 1.245825019 | 1.057032387 | LRRK2;STA |
| 0 | 0 | 1.245825019 | 1.057032387 | DYNC1H1; |
| 0 | 0 | 1.144642857 | 0.955870264 | GALC;UGC |
| 0 | 0 | 1.308075472 | 1.091280188 | HYAL1;CH |
| 0 | 0 | 1.308075472 | 1.091280188 | ACVRL1;PR |
| 0 | 0 | 1.308075472 | 1.091280188 | PLA2G4A; |
| 0 | 0 | 1.308075472 | 1.091280188 | PLA2G4A; |
| 0 | 0 | 1.308075472 | 1.091280188 | ANXA1;PT |
| 0 | 0 | 1.308075472 | 1.091280188 | NLGN3;ST |
| 0 | 0 | 1.308075472 | 1.091280188 | EGR2;PAR |
| 0 | 0 | 1.308075472 | 1.091280188 | FZD1;NLG |
| 0 | 0 | 1.308075472 | 1.091280188 | CEP83;SN |
| 0 | 0 | 1.308075472 | 1.091280188 | TNFRSF1B; |
| 0 | 0 | 1.308075472 | 1.091280188 | AMER1;RI |
| 0 | 0 | 1.308075472 | 1.091280188 | SRC;DENN |
| 0 | 0 | 1.308075472 | 1.091280188 | EIF6;PRKA |
| 0 | 0 | 1.308075472 | 1.091280188 | STAT5A;G |
| 0 | 0 | 1.308075472 | 1.091280188 | DYNC1H1; |
| 0 | 0 | 1.308075472 | 1.091280188 | ADAM17;F |
| 0 | 0 | 1.308075472 | 1.091280188 | RIDA;ROC |
| 0 | 0 | 1.308075472 | 1.091280188 | TRIR;XRN1 |
| 0 | 0 | 1.453371893 | 1.195039938 | XYLT1;B3G |
| 0 | 0 | 1.453371893 | 1.195039938 | HPSE;VEG |
| 0 | 0 | 1.453371893 | 1.195039938 | NF1;VEGF |
| 0 | 0 | 1.453371893 | 1.195039938 | ATP1A1;K |
| 0 | 0 | 1.453371893 | 1.195039938 | MID1IP1;P |
| 0 | 0 | 1.453371893 | 1.195039938 | DRAXIN;SC |
| 0 | 0 | 1.453371893 | 1.195039938 | CEP120;N |
| 0 | 0 | 1.453371893 | 1.195039938 | MYADM;I |
| 0 | 0 | 1.453371893 | 1.195039938 | GRAMD1C |
| 0 | 0 | 1.453371893 | 1.195039938 | ATP7A;SO |
| 0 | 0 | 1.453371893 | 1.195039938 | TBXAS1;PT |
| 0 | 0 | 1.453371893 | 1.195039938 | MAD2L2;K |
| 0 | 0 | 1.453371893 | 1.195039938 | RARG;EFE |
| 0 | 0 | 1.453371893 | 1.195039938 | GCLC;GGT |

| | | | | | |
|---|---|---|---|---|---|
| 0 | 0 | 1.453371893 | 1.195039938 | GK5;PGP | . |
| 0 | 0 | 1.453371893 | 1.195039938 | DGKG;PGP | . |
| 0 | 0 | 1.453371893 | 1.195039938 | PLK3;VCPII | . |
| 0 | 0 | 1.453371893 | 1.195039938 | UGCG;LDLI | . |
| 0 | 0 | 1.453371893 | 1.195039938 | VMP1;PRK | . |
| 0 | 0 | 1.453371893 | 1.195039938 | FZD1;NR4/ | . |
| 0 | 0 | 1.453371893 | 1.195039938 | IFI16;VEGF | . |
| 0 | 0 | 1.453371893 | 1.195039938 | APOA2;NFI | . |
| 0 | 0 | 1.453371893 | 1.195039938 | ILRUN;ITCI | . |
| 0 | 0 | 1.453371893 | 1.195039938 | PRKCD;ALC | . |
| 0 | 0 | 1.453371893 | 1.195039938 | RIPOR2;LR | . |
| 0 | 0 | 1.453371893 | 1.195039938 | ACTN2;PIC | . |
| 0 | 0 | 1.453371893 | 1.195039938 | SEMA6A;E | . |
| 0 | 0 | 1.453371893 | 1.195039938 | YTHDC2;IG | . |
| 0 | 0 | 1.453371893 | 1.195039938 | DCSTAMP; | . |
| 0 | 0 | 1.453371893 | 1.195039938 | DAXX;H3-3 | . |
| 0 | 0 | 1.453371893 | 1.195039938 | TGM1;ARE | . |
| 0 | 0 | 1.453371893 | 1.195039938 | ABCA3;AB( | . |
| 0 | 0 | 1.453371893 | 1.195039938 | ELL;SUPT6I | . |
| 0 | 0 | 1.453371893 | 1.195039938 | ANXA2;RAI | . |
| 0 | 0 | 1.453371893 | 1.195039938 | NLGN3;LRI | . |
| 0 | 0 | 1.453371893 | 1.195039938 | PLEK;FLOT | . |
| 0 | 0 | 1.453371893 | 1.195039938 | DGKD;PAR | . |
| 0 | 0 | 1.453371893 | 1.195039938 | APOA2;LO) | . |
| 0 | 0 | 1.453371893 | 1.195039938 | UPP1;DCK | . |
| 0 | 0 | 1.453371893 | 1.195039938 | UPP1;DCK | . |
| 0 | 0 | 1.453371893 | 1.195039938 | PPP1R10;N | . |
| 0 | 0 | 1.453371893 | 1.195039938 | LYN;LGALS | . |
| 0 | 0 | 1.453371893 | 1.195039938 | DAXX;H3-3 | . |
| 0 | 0 | 1.453371893 | 1.195039938 | DNAJB6;HS | . |
| 0 | 0 | 1.453371893 | 1.195039938 | LYN;CD30( | . |
| 0 | 0 | 1.453371893 | 1.195039938 | STAT1;LRP | . |
| 0 | 0 | 1.453371893 | 1.195039938 | LAMP1;CCI | . |
| 0 | 0 | 1.453371893 | 1.195039938 | SRC;LRRK2 | . |
| 0 | 0 | 1.453371893 | 1.195039938 | RGS2;PLA2 | . |
| 0 | 0 | 1.453371893 | 1.195039938 | GCH1;CALI | . |
| 0 | 0 | 1.453371893 | 1.195039938 | MTHFD2;N | . |
| 0 | 0 | 1.453371893 | 1.195039938 | SLC2A9;PN | . |
| 0 | 0 | 1.154225649 | 0.944335498 | PTPRC;SH3 | . |
| 0 | 0 | 1.154225649 | 0.944335498 | CYP4F2;CY | . |
| 0 | 0 | 1.154225649 | 0.944335498 | SLC9A3R1; | . |
| 0 | 0 | 1.035624117 | 0.840981761 | ITGAM;ELN | . |
| 0 | 0 | 1.06794208 | 0.864306355 | ACVRL1;CD | . |
| 0 | 0 | 1.090146354 | 0.881439497 | HCK;CYLD; | . |
| 0 | 0 | 1.06073132 | 0.853223056 | RYR1;ARH( | . |
| 0 | 0 | 1.113300889 | 0.893226836 | TNIP1;MAI | . |
| 0 | 0 | 1.113300889 | 0.893226836 | RYR1;ITPR: | . |

| | | | | |
|---|---|---|---|---|
| 0 | 0 | 1.076352129 | 0.86217199 | NCOA1;CR |
| 0 | 0 | 1.167929974 | 0.933716532 | FSCN1;NED |
| 0 | 0 | 1.167929974 | 0.933716532 | RFLNB;FSC |
| 0 | 0 | 1.167929974 | 0.933716532 | B3GNT3;B3 |
| 0 | 0 | 1.167929974 | 0.933716532 | RAMP2;DY |
| 0 | 0 | 1.167929974 | 0.933716532 | ELOVL4;EL |
| 0 | 0 | 1.090112246 | 0.863859641 | NIPBL;HYA |
| 0 | 0 | 1.116660214 | 0.875510186 | PLLP;PARD |
| 0 | 0 | 1.116660214 | 0.875510186 | SLC25A16; |
| 0 | 0 | 1.116660214 | 0.875510186 | USP36;FLC |
| 0 | 0 | 1.116660214 | 0.875510186 | NRP1;LAM |
| 0 | 0 | 1.064770835 | 0.831321658 | ACTN2;SRC |
| 0 | 0 | 1.189127973 | 0.925462322 | IL1B;PDE4 |
| 0 | 0 | 1.189127973 | 0.925462322 | ERO1A;HSI |
| 0 | 0 | 1.189127973 | 0.925462322 | CANX;SIGL |
| 0 | 0 | 1.189127973 | 0.925462322 | DNMT3L;B |
| 0 | 0 | 1.189127973 | 0.925462322 | RPS7;MAR |
| 0 | 0 | 1.189127973 | 0.925462322 | PHF1;PAF1 |
| 0 | 0 | 1.189127973 | 0.925462322 | MAPK8;CA |
| 0 | 0 | 1.189127973 | 0.925462322 | CCNT1;POI |
| 0 | 0 | 1.189127973 | 0.925462322 | ROCK1;PLE |
| 0 | 0 | 1.189127973 | 0.925462322 | ACVRL1;EF |
| 0 | 0 | 1.189127973 | 0.925462322 | DUSP1;NEI |
| 0 | 0 | 1.189127973 | 0.925462322 | EGR2;PARI |
| 0 | 0 | 1.189127973 | 0.925462322 | MAPK8;CA |
| 0 | 0 | 1.090078164 | 0.845010342 | NPEPPS;RII |
| 0 | 0 | 1.12118301 | 0.856365565 | DGKG;GK5 |
| 0 | 0 | 1.12118301 | 0.856365565 | HK3;PKLR;I |
| 0 | 0 | 1.12118301 | 0.856365565 | SMG1;FXR |
| 0 | 0 | 1.12118301 | 0.856365565 | ACVRL1;ST |
| 0 | 0 | 1.054929199 | 0.804344051 | SMAD3;SM |
| 0 | 0 | 1.090044108 | 0.824582409 | VCAN;SDC |
| 0 | 0 | 1.090044108 | 0.824582409 | SLC25A16; |
| 0 | 0 | 1.090044108 | 0.824582409 | ZFP36;TUT |
| 0 | 0 | 1.22625 | 0.923631838 | SVIL;GSN;A |
| 0 | 0 | 1.22625 | 0.923631838 | SMAD3;AC |
| 0 | 0 | 1.22625 | 0.923631838 | SVIL;GSN;A |
| 0 | 0 | 1.22625 | 0.923631838 | ADCY4;AD |
| 0 | 0 | 1.22625 | 0.923631838 | EIF4A1;EIF |
| 0 | 0 | 1.22625 | 0.923631838 | GCH1;CALI |
| 0 | 0 | 1.22625 | 0.923631838 | CYP4F2;EP |
| 0 | 0 | 1.22625 | 0.923631838 | MID1IP1;S |
| 0 | 0 | 1.22625 | 0.923631838 | PM20D2;N |
| 0 | 0 | 1.22625 | 0.923631838 | KAT8;JADE |
| 0 | 0 | 1.22625 | 0.923631838 | RGS14;SGK |
| 0 | 0 | 1.22625 | 0.923631838 | MEF2A;LRI |
| 0 | 0 | 1.22625 | 0.923631838 | INPP5K;SL |

| | | | | |
|---|---|---|---|---|
| 0 | 0 | 1.22625 | 0.923631838 | ILRUN;FLCI |
| 0 | 0 | 1.22625 | 0.923631838 | PTBP2;PTB |
| 0 | 0 | 1.22625 | 0.923631838 | HDAC4;XBI |
| 0 | 0 | 1.22625 | 0.923631838 | RANBP2;KI |
| 0 | 0 | 1.22625 | 0.923631838 | CEP120;CH |
| 0 | 0 | 1.22625 | 0.923631838 | NLGN3;LRI |
| 0 | 0 | 1.22625 | 0.923631838 | CIITA;IL6;R |
| 0 | 0 | 1.22625 | 0.923631838 | NAIF1;PPIF |
| 0 | 0 | 1.22625 | 0.923631838 | VPS4B;CHN |
| 0 | 0 | 1.22625 | 0.923631838 | P2RY12;P2 |
| 0 | 0 | 1.22625 | 0.923631838 | KDM6B;NF |
| 0 | 0 | 1.072179528 | 0.8063504 | CLN3;AGAI |
| 0 | 0 | 1.072179528 | 0.8063504 | EDN1;PIK3 |
| 0 | 0 | 1.072179528 | 0.8063504 | RYR1;GBP5 |
| 0 | 0 | 1.127591416 | 0.835389507 | GRK2;GSN; |
| 0 | 0 | 1.127591416 | 0.835389507 | ACVRL1;PA |
| 0 | 0 | 1.127591416 | 0.835389507 | ACVRL1;RG |
| 0 | 0 | 1.127591416 | 0.835389507 | EDN1;PIK3 |
| 0 | 0 | 1.127591416 | 0.835389507 | NIBAN2;CE |
| 0 | 0 | 1.127591416 | 0.835389507 | CYP27B1;Z |
| 0 | 0 | 1.127591416 | 0.835389507 | PAQR8;UR |
| 0 | 0 | 1.090010078 | 0.802135147 | RAMP2;CD |
| 0 | 0 | 1.070196672 | 0.784847065 | ABCA1;STE |
| 0 | 0 | 1.070196672 | 0.784847065 | PSMA2;OD |
| 0 | 0 | 1.021093821 | 0.748478347 | SLC24A1;S |
| 0 | 0 | 1.049653947 | 0.767460055 | RET;CD74; |
| 0 | 0 | 1.039099173 | 0.759090274 | FZD1;TCF7 |
| 0 | 0 | 1.307959261 | 0.943498513 | RB1CC1;SC |
| 0 | 0 | 1.307959261 | 0.943498513 | FOLH1B;AT |
| 0 | 0 | 1.307959261 | 0.943498513 | NABP2;NA |
| 0 | 0 | 1.307959261 | 0.943498513 | PCGF5;LRII |
| 0 | 0 | 1.307959261 | 0.943498513 | VPS9D1;AT |
| 0 | 0 | 1.307959261 | 0.943498513 | UBE2S;CHN |
| 0 | 0 | 1.307959261 | 0.943498513 | STAT3;VEG |
| 0 | 0 | 1.307959261 | 0.943498513 | GLUL;GLS |
| 0 | 0 | 1.307959261 | 0.943498513 | CCL3;CCL2 |
| 0 | 0 | 1.307959261 | 0.943498513 | NIN;NINL |
| 0 | 0 | 1.307959261 | 0.943498513 | MEF2A;LRI |
| 0 | 0 | 1.307959261 | 0.943498513 | PTX3;IGF2I |
| 0 | 0 | 1.307959261 | 0.943498513 | ACTB;ACTG |
| 0 | 0 | 1.307959261 | 0.943498513 | ZMYND10; |
| 0 | 0 | 1.307959261 | 0.943498513 | SUSD2;TXN |
| 0 | 0 | 1.307959261 | 0.943498513 | LIMK2;TES |
| 0 | 0 | 1.307959261 | 0.943498513 | ZC3H12A;T |
| 0 | 0 | 1.307959261 | 0.943498513 | EDN1;SERI |
| 0 | 0 | 1.307959261 | 0.943498513 | DUSP1;SLA |
| 0 | 0 | 1.307959261 | 0.943498513 | SDCBP;ANI |

| | | | | |
|---|---|---|---|---|
| 0 | 0 | 1.307959261 | 0.943498513 | SRI;CALM1 |
| 0 | 0 | 1.307959261 | 0.943498513 | SRI;CALM1 |
| 0 | 0 | 1.307959261 | 0.943498513 | SLC22A3;G |
| 0 | 0 | 1.307959261 | 0.943498513 | GCLC;GGT |
| 0 | 0 | 1.307959261 | 0.943498513 | UPP1;DCK |
| 0 | 0 | 1.307959261 | 0.943498513 | EDN1;TIFA |
| 0 | 0 | 1.307959261 | 0.943498513 | SLC37A1;S |
| 0 | 0 | 1.307959261 | 0.943498513 | ABCA1;AB |
| 0 | 0 | 1.307959261 | 0.943498513 | B3GNT3;B |
| 0 | 0 | 1.307959261 | 0.943498513 | ACTN2;PLE |
| 0 | 0 | 1.307959261 | 0.943498513 | ATP1B3;GL |
| 0 | 0 | 1.307959261 | 0.943498513 | FZR1;UBE2 |
| 0 | 0 | 1.307959261 | 0.943498513 | DPYS;DPY |
| 0 | 0 | 1.307959261 | 0.943498513 | PIK3CA;PL |
| 0 | 0 | 1.307959261 | 0.943498513 | GSN;CORO |
| 0 | 0 | 1.307959261 | 0.943498513 | SRI;CALM1 |
| 0 | 0 | 1.307959261 | 0.943498513 | APOA2;AB |
| 0 | 0 | 1.307959261 | 0.943498513 | LGALS9;FC |
| 0 | 0 | 1.307959261 | 0.943498513 | NR4A3;ST |
| 0 | 0 | 1.307959261 | 0.943498513 | RNASE10; |
| 0 | 0 | 1.307959261 | 0.943498513 | JARID2;SIR |
| 0 | 0 | 1.307959261 | 0.943498513 | ASS1;FUT4 |
| 0 | 0 | 1.307959261 | 0.943498513 | STK26;FYN |
| 0 | 0 | 1.307959261 | 0.943498513 | NRDE2;SU |
| 0 | 0 | 1.307959261 | 0.943498513 | DOCK4;NR |
| 0 | 0 | 1.307959261 | 0.943498513 | PSAT1;GG |
| 0 | 0 | 1.307959261 | 0.943498513 | ASS1;IDH3 |
| 0 | 0 | 1.137361104 | 0.811987866 | NLGN3;ST |
| 0 | 0 | 1.137361104 | 0.811987866 | LIMK2;PRK |
| 0 | 0 | 1.137361104 | 0.811987866 | MEF2A;M |
| 0 | 0 | 1.054825284 | 0.752624446 | NLGN3;VA |
| 0 | 0 | 1.067736584 | 0.761291588 | MAD2L2;H |
| 0 | 0 | 1.089976074 | 0.777009778 | ASAH1;SPT |
| 0 | 0 | 1.089976074 | 0.777009778 | IL1B;BHLH |
| 0 | 0 | 1.042567664 | 0.725718736 | PRKAA1;X |
| 0 | 0 | 1.019181655 | 0.708129812 | NIBAN1;TS |
| 0 | 0 | 1.051025232 | 0.728962211 | CCR1;PTGI |
| 0 | 0 | 1.064599483 | 0.735088089 | CCR1;TRP |
| 0 | 0 | 1.089942095 | 0.748158472 | ABCA3;PD |
| 0 | 0 | 1.089942095 | 0.748158472 | RNASE10; |
| 0 | 0 | 1.089942095 | 0.748158472 | MEF2A;M |
| 0 | 0 | 1.089942095 | 0.748158472 | NLGN3;BC |
| 0 | 0 | 1.089942095 | 0.748158472 | ABCA3;PD |
| 0 | 0 | 1.032563423 | 0.702733738 | RNF126;R |
| 0 | 0 | 1.154051054 | 0.785285107 | HES1;MD |
| 0 | 0 | 1.154051054 | 0.785285107 | GCLC;MTH |
| 0 | 0 | 1.154051054 | 0.785285107 | NR4A2;M |

| | | | | | |
|---|---|---|---|---|---|
| 0 | 0 | 1.154051054 | 0.785285107 | NFAT5;ED | . |
| 0 | 0 | 1.154051054 | 0.785285107 | CEBPG;PLE | . |
| 0 | 0 | 1.154051054 | 0.785285107 | HEXIM1;H | . |
| 0 | 0 | 1.154051054 | 0.785285107 | CCL3;TLR4 | . |
| 0 | 0 | 1.154051054 | 0.785285107 | XRN1;SRC; | . |
| 0 | 0 | 1.154051054 | 0.785285107 | CYP27B1;V | . |
| 0 | 0 | 1.154051054 | 0.785285107 | BTG1;VEZ | . |
| 0 | 0 | 1.154051054 | 0.785285107 | AMPD1;A | . |
| 0 | 0 | 1.154051054 | 0.785285107 | CLN3;ZDH | . |
| 0 | 0 | 1.154051054 | 0.785285107 | NMUR2;P | . |
| 0 | 0 | 1.038021969 | 0.702424859 | H1-4;DAX | . |
| 0 | 0 | 1.63489819 | 1.103542165 | PRPSAP1 | . |
| 0 | 0 | 1.63489819 | 1.103542165 | PRPSAP1 | . |
| 0 | 0 | 1.63489819 | 1.103542165 | NUDT3 | . |
| 0 | 0 | 1.63489819 | 1.103542165 | NUDT3 | . |
| 0 | 0 | 1.63489819 | 1.103542165 | KIF5C | . |
| 0 | 0 | 1.63489819 | 1.103542165 | KIF5C | . |
| 0 | 0 | 1.63489819 | 1.103542165 | CD1D | . |
| 0 | 0 | 1.63489819 | 1.103542165 | CD1D | . |
| 0 | 0 | 1.63489819 | 1.103542165 | CD1D | . |
| 0 | 0 | 1.63489819 | 1.103542165 | DFFB | . |
| 0 | 0 | 1.63489819 | 1.103542165 | AMER1 | . |
| 0 | 0 | 1.63489819 | 1.103542165 | SLC9A3R1 | . |
| 0 | 0 | 1.63489819 | 1.103542165 | FOLH1B | . |
| 0 | 0 | 1.63489819 | 1.103542165 | ATG7 | . |
| 0 | 0 | 1.63489819 | 1.103542165 | DAGLB | . |
| 0 | 0 | 1.63489819 | 1.103542165 | NRG1 | . |
| 0 | 0 | 1.63489819 | 1.103542165 | PAF1 | . |
| 0 | 0 | 1.63489819 | 1.103542165 | MDM2 | . |
| 0 | 0 | 1.63489819 | 1.103542165 | MT2A | . |
| 0 | 0 | 1.63489819 | 1.103542165 | EGR1 | . |
| 0 | 0 | 1.63489819 | 1.103542165 | DCSTAMP | . |
| 0 | 0 | 1.63489819 | 1.103542165 | XBP1 | . |
| 0 | 0 | 1.63489819 | 1.103542165 | NCOA4 | . |
| 0 | 0 | 1.63489819 | 1.103542165 | ATP7A | . |
| 0 | 0 | 1.63489819 | 1.103542165 | TOX | . |
| 0 | 0 | 1.63489819 | 1.103542165 | CXCR4 | . |
| 0 | 0 | 1.63489819 | 1.103542165 | LRRC8C | . |
| 0 | 0 | 1.63489819 | 1.103542165 | RPRD1A | . |
| 0 | 0 | 1.63489819 | 1.103542165 | AIDA | . |
| 0 | 0 | 1.63489819 | 1.103542165 | NUDT3 | . |
| 0 | 0 | 1.63489819 | 1.103542165 | NUDT3 | . |
| 0 | 0 | 1.63489819 | 1.103542165 | NUDT3 | . |
| 0 | 0 | 1.63489819 | 1.103542165 | NUDT3 | . |
| 0 | 0 | 1.63489819 | 1.103542165 | STRA6 | . |
| 0 | 0 | 1.63489819 | 1.103542165 | NUDT3 | . |
| 0 | 0 | 1.63489819 | 1.103542165 | SLC2A1 | . |

| | | | | | |
|---|---|---|---|---|---|
| 0 | 0 | 1.63489819 | 1.103542165 | NUDT1 | . |
| 0 | 0 | 1.63489819 | 1.103542165 | GCH1 | . |
| 0 | 0 | 1.63489819 | 1.103542165 | ABCA3 | . |
| 0 | 0 | 1.63489819 | 1.103542165 | NIPBL | . |
| 0 | 0 | 1.63489819 | 1.103542165 | FAM20A | . |
| 0 | 0 | 1.63489819 | 1.103542165 | NRG1 | . |
| 0 | 0 | 1.63489819 | 1.103542165 | IL10 | . |
| 0 | 0 | 1.63489819 | 1.103542165 | FSCN1 | . |
| 0 | 0 | 1.63489819 | 1.103542165 | NABP2 | . |
| 0 | 0 | 1.63489819 | 1.103542165 | TNFAIP2 | . |
| 0 | 0 | 1.63489819 | 1.103542165 | RAB27A | . |
| 0 | 0 | 1.63489819 | 1.103542165 | STRA6 | . |
| 0 | 0 | 1.63489819 | 1.103542165 | WNT2B | . |
| 0 | 0 | 1.63489819 | 1.103542165 | KHK | . |
| 0 | 0 | 1.63489819 | 1.103542165 | KHK | . |
| 0 | 0 | 1.63489819 | 1.103542165 | SLC2A6 | . |
| 0 | 0 | 1.63489819 | 1.103542165 | ADCY8 | . |
| 0 | 0 | 1.63489819 | 1.103542165 | RET | . |
| 0 | 0 | 1.63489819 | 1.103542165 | SLC37A1 | . |
| 0 | 0 | 1.63489819 | 1.103542165 | UGCG | . |
| 0 | 0 | 1.63489819 | 1.103542165 | GLUL | . |
| 0 | 0 | 1.63489819 | 1.103542165 | PGP | . |
| 0 | 0 | 1.63489819 | 1.103542165 | LIPE | . |
| 0 | 0 | 1.63489819 | 1.103542165 | ALAS1 | . |
| 0 | 0 | 1.63489819 | 1.103542165 | SLC37A1 | . |
| 0 | 0 | 1.63489819 | 1.103542165 | PRKCB | . |
| 0 | 0 | 1.63489819 | 1.103542165 | CELA2A | . |
| 0 | 0 | 1.63489819 | 1.103542165 | LRRK2 | . |
| 0 | 0 | 1.63489819 | 1.103542165 | IDH3A | . |
| 0 | 0 | 1.63489819 | 1.103542165 | PMVK | . |
| 0 | 0 | 1.63489819 | 1.103542165 | SLC7A1 | . |
| 0 | 0 | 1.63489819 | 1.103542165 | CYP4F2 | . |
| 0 | 0 | 1.63489819 | 1.103542165 | LRP5 | . |
| 0 | 0 | 1.63489819 | 1.103542165 | EDEM1 | . |
| 0 | 0 | 1.63489819 | 1.103542165 | LGALS9 | . |
| 0 | 0 | 1.63489819 | 1.103542165 | ABHD1 | . |
| 0 | 0 | 1.63489819 | 1.103542165 | PTPRC | . |
| 0 | 0 | 1.63489819 | 1.103542165 | CYP4F2 | . |
| 0 | 0 | 1.63489819 | 1.103542165 | STAT1 | . |
| 0 | 0 | 1.63489819 | 1.103542165 | PNPT1 | . |
| 0 | 0 | 1.63489819 | 1.103542165 | ATG7 | . |
| 0 | 0 | 1.63489819 | 1.103542165 | ZNF473 | . |
| 0 | 0 | 1.63489819 | 1.103542165 | SAMSN1 | . |
| 0 | 0 | 1.63489819 | 1.103542165 | PHF23 | . |
| 0 | 0 | 1.63489819 | 1.103542165 | ID2 | . |
| 0 | 0 | 1.63489819 | 1.103542165 | PPIF | . |
| 0 | 0 | 1.63489819 | 1.103542165 | PRKAA1 | . |

| | | | | | |
|---|---|---|---|---|---|
| 0 | 0 | 1.63489819 | 1.103542165 | FCGR2B | . |
| 0 | 0 | 1.63489819 | 1.103542165 | KDM5A | . |
| 0 | 0 | 1.63489819 | 1.103542165 | IL10 | . |
| 0 | 0 | 1.63489819 | 1.103542165 | KLF4 | . |
| 0 | 0 | 1.63489819 | 1.103542165 | FCGR2B | . |
| 0 | 0 | 1.63489819 | 1.103542165 | SLAMF8 | . |
| 0 | 0 | 1.63489819 | 1.103542165 | IRAK3 | . |
| 0 | 0 | 1.63489819 | 1.103542165 | PTPN2 | . |
| 0 | 0 | 1.63489819 | 1.103542165 | FLCN | . |
| 0 | 0 | 1.63489819 | 1.103542165 | HIF1A | . |
| 0 | 0 | 1.63489819 | 1.103542165 | SERPINE1 | . |
| 0 | 0 | 1.63489819 | 1.103542165 | SIRT1 | . |
| 0 | 0 | 1.63489819 | 1.103542165 | PARP10 | . |
| 0 | 0 | 1.63489819 | 1.103542165 | IRF1 | . |
| 0 | 0 | 1.63489819 | 1.103542165 | DOCK4 | . |
| 0 | 0 | 1.63489819 | 1.103542165 | TLR2 | . |
| 0 | 0 | 1.63489819 | 1.103542165 | PRKAA1 | . |
| 0 | 0 | 1.63489819 | 1.103542165 | SLC28A3 | . |
| 0 | 0 | 1.63489819 | 1.103542165 | SMPDL3A | . |
| 0 | 0 | 1.63489819 | 1.103542165 | YTHDC2 | . |
| 0 | 0 | 1.63489819 | 1.103542165 | SLC13A5 | . |
| 0 | 0 | 1.63489819 | 1.103542165 | ALKBH1 | . |
| 0 | 0 | 1.63489819 | 1.103542165 | LOXL1 | . |
| 0 | 0 | 1.63489819 | 1.103542165 | TIFAB | . |
| 0 | 0 | 1.63489819 | 1.103542165 | ATP7A | . |
| 0 | 0 | 1.63489819 | 1.103542165 | PML | . |
| 0 | 0 | 1.63489819 | 1.103542165 | YTHDC2 | . |
| 0 | 0 | 1.63489819 | 1.103542165 | TENT4B | . |
| 0 | 0 | 1.63489819 | 1.103542165 | GRK2 | . |
| 0 | 0 | 1.63489819 | 1.103542165 | NLGN3 | . |
| 0 | 0 | 1.63489819 | 1.103542165 | SIK1 | . |
| 0 | 0 | 1.63489819 | 1.103542165 | PHLDB1 | . |
| 0 | 0 | 1.63489819 | 1.103542165 | NPHP1 | . |
| 0 | 0 | 1.63489819 | 1.103542165 | SIRT1 | . |
| 0 | 0 | 1.63489819 | 1.103542165 | RGS2 | . |
| 0 | 0 | 1.63489819 | 1.103542165 | LGALS9 | . |
| 0 | 0 | 1.63489819 | 1.103542165 | PRKCD | . |
| 0 | 0 | 1.63489819 | 1.103542165 | PARP3 | . |
| 0 | 0 | 1.63489819 | 1.103542165 | LRRK2 | . |
| 0 | 0 | 1.63489819 | 1.103542165 | RIN2 | . |
| 0 | 0 | 1.63489819 | 1.103542165 | TGFBR1 | . |
| 0 | 0 | 1.63489819 | 1.103542165 | NR4A3 | . |
| 0 | 0 | 1.63489819 | 1.103542165 | CFAP69 | . |
| 0 | 0 | 1.63489819 | 1.103542165 | PRKD2 | . |
| 0 | 0 | 1.63489819 | 1.103542165 | NOD2 | . |
| 0 | 0 | 1.63489819 | 1.103542165 | CLN3 | . |
| 0 | 0 | 1.63489819 | 1.103542165 | PIH1D1 | . |

| | | | | | |
|---|---|---|---|---|---|
| 0 | 0 | 1.63489819 | 1.103542165 | RPS7 | . |
| 0 | 0 | 1.63489819 | 1.103542165 | ADCY8 | . |
| 0 | 0 | 1.63489819 | 1.103542165 | ANXA2 | . |
| 0 | 0 | 1.63489819 | 1.103542165 | PTK2 | . |
| 0 | 0 | 1.63489819 | 1.103542165 | NSMCE2 | . |
| 0 | 0 | 1.63489819 | 1.103542165 | NSMCE2 | . |
| 0 | 0 | 1.63489819 | 1.103542165 | LAPTM5 | . |
| 0 | 0 | 1.63489819 | 1.103542165 | SOX15 | . |
| 0 | 0 | 1.63489819 | 1.103542165 | STX1B | . |
| 0 | 0 | 1.63489819 | 1.103542165 | RIPOR2 | . |
| 0 | 0 | 1.63489819 | 1.103542165 | NRDE2 | . |
| 0 | 0 | 1.63489819 | 1.103542165 | IL1B | . |
| 0 | 0 | 1.63489819 | 1.103542165 | EPB41L2 | . |
| 0 | 0 | 1.63489819 | 1.103542165 | BIRC2 | . |
| 0 | 0 | 1.63489819 | 1.103542165 | DCUN1D3 | . |
| 0 | 0 | 1.63489819 | 1.103542165 | NRDE2 | . |
| 0 | 0 | 1.63489819 | 1.103542165 | SLC8A1 | . |
| 0 | 0 | 1.63489819 | 1.103542165 | DDX3X | . |
| 0 | 0 | 1.63489819 | 1.103542165 | NRXN2 | . |
| 0 | 0 | 1.63489819 | 1.103542165 | EDEM1 | . |
| 0 | 0 | 1.63489819 | 1.103542165 | TNFAIP3 | . |
| 0 | 0 | 1.63489819 | 1.103542165 | MAPRE1 | . |
| 0 | 0 | 1.63489819 | 1.103542165 | MAPRE1 | . |
| 0 | 0 | 1.63489819 | 1.103542165 | PARP3 | . |
| 0 | 0 | 1.63489819 | 1.103542165 | STRA6 | . |
| 0 | 0 | 1.63489819 | 1.103542165 | CMPK2 | . |
| 0 | 0 | 1.63489819 | 1.103542165 | SLC28A3 | . |
| 0 | 0 | 1.63489819 | 1.103542165 | DCK | . |
| 0 | 0 | 1.63489819 | 1.103542165 | ADAM17 | . |
| 0 | 0 | 1.63489819 | 1.103542165 | STAT5A | . |
| 0 | 0 | 1.63489819 | 1.103542165 | VEGFA | . |
| 0 | 0 | 1.63489819 | 1.103542165 | PHLDB1 | . |
| 0 | 0 | 1.63489819 | 1.103542165 | RBPJ | . |
| 0 | 0 | 1.63489819 | 1.103542165 | DLL1 | . |
| 0 | 0 | 1.63489819 | 1.103542165 | COX17 | . |
| 0 | 0 | 1.63489819 | 1.103542165 | CXCL10 | . |
| 0 | 0 | 1.63489819 | 1.103542165 | IL6R | . |
| 0 | 0 | 1.63489819 | 1.103542165 | PTX3 | . |
| 0 | 0 | 1.63489819 | 1.103542165 | JAK2 | . |
| 0 | 0 | 1.63489819 | 1.103542165 | NOS2 | . |
| 0 | 0 | 1.63489819 | 1.103542165 | DUSP6 | . |
| 0 | 0 | 1.63489819 | 1.103542165 | ITGAM | . |
| 0 | 0 | 1.63489819 | 1.103542165 | PTPRG | . |
| 0 | 0 | 1.63489819 | 1.103542165 | S100A9 | . |
| 0 | 0 | 1.63489819 | 1.103542165 | IL16 | . |
| 0 | 0 | 1.63489819 | 1.103542165 | ANXA2 | . |
| 0 | 0 | 1.63489819 | 1.103542165 | NSMCE2 | . |

| | | | | | |
|---|---|---|---|---|---|
| 0 | 0 | 1.63489819 | 1.103542165 | DYNC1H1 | . |
| 0 | 0 | 1.63489819 | 1.103542165 | ITGAM | . |
| 0 | 0 | 1.63489819 | 1.103542165 | LRRK2 | . |
| 0 | 0 | 1.63489819 | 1.103542165 | FMR1 | . |
| 0 | 0 | 1.63489819 | 1.103542165 | SOX15 | . |
| 0 | 0 | 1.63489819 | 1.103542165 | PRDM1 | . |
| 0 | 0 | 1.63489819 | 1.103542165 | ITGAM | . |
| 0 | 0 | 1.63489819 | 1.103542165 | GSN | . |
| 0 | 0 | 1.63489819 | 1.103542165 | BIRC2 | . |
| 0 | 0 | 1.63489819 | 1.103542165 | NLGN3 | . |
| 0 | 0 | 1.63489819 | 1.103542165 | OAS2 | . |
| 0 | 0 | 1.63489819 | 1.103542165 | FLOT1 | . |
| 0 | 0 | 1.63489819 | 1.103542165 | ZNF143 | . |
| 0 | 0 | 1.63489819 | 1.103542165 | TSPO | . |
| 0 | 0 | 1.63489819 | 1.103542165 | C3 | . |
| 0 | 0 | 1.63489819 | 1.103542165 | STAT1 | . |
| 0 | 0 | 1.63489819 | 1.103542165 | TGM2 | . |
| 0 | 0 | 1.63489819 | 1.103542165 | HDAC4 | . |
| 0 | 0 | 1.63489819 | 1.103542165 | DCSTAMP | . |
| 0 | 0 | 1.63489819 | 1.103542165 | SQSTM1 | . |
| 0 | 0 | 1.63489819 | 1.103542165 | ADCY8 | . |
| 0 | 0 | 1.63489819 | 1.103542165 | ACVRL1 | . |
| 0 | 0 | 1.63489819 | 1.103542165 | EIF6 | . |
| 0 | 0 | 1.63489819 | 1.103542165 | PNPT1 | . |
| 0 | 0 | 1.63489819 | 1.103542165 | PNPT1 | . |
| 0 | 0 | 1.63489819 | 1.103542165 | PKD1L3 | . |
| 0 | 0 | 1.63489819 | 1.103542165 | ATP7A | . |
| 0 | 0 | 1.63489819 | 1.103542165 | ZFAND1 | . |
| 0 | 0 | 1.63489819 | 1.103542165 | TIFAB | . |
| 0 | 0 | 1.63489819 | 1.103542165 | ACTG1 | . |
| 0 | 0 | 1.63489819 | 1.103542165 | EDN1 | . |
| 0 | 0 | 1.63489819 | 1.103542165 | SOX15 | . |
| 0 | 0 | 1.63489819 | 1.103542165 | EDEM1 | . |
| 0 | 0 | 1.63489819 | 1.103542165 | SMURF1 | . |
| 0 | 0 | 1.63489819 | 1.103542165 | VPS4B | . |
| 0 | 0 | 1.63489819 | 1.103542165 | SLC28A3 | . |
| 0 | 0 | 1.63489819 | 1.103542165 | VEGFA | . |
| 0 | 0 | 1.63489819 | 1.103542165 | TBC1D23 | . |
| 0 | 0 | 1.63489819 | 1.103542165 | CYP27B1 | . |
| 0 | 0 | 1.63489819 | 1.103542165 | TIFAB | . |
| 0 | 0 | 1.63489819 | 1.103542165 | CAST | . |
| 0 | 0 | 1.046321361 | 0.702910961 | SERPINB4;[illegible] | . |
| 0 | 0 | 1.046321361 | 0.702910961 | METTL1;AI[illegible] | . |
| 0 | 0 | 1.060455998 | 0.705342035 | SLC22A3;S[illegible] | . |
| 0 | 0 | 1.060455998 | 0.705342035 | DGKG;DGK[illegible] | . |
| 0 | 0 | 1.060455998 | 0.705342035 | YTHDC2;RF[illegible] | . |
| 0 | 0 | 1.032526475 | 0.677038202 | SCUBE3;GS[illegible] | . |

| | | | | |
|---|---|---|---|---|
| 0 | 0 | 1.032526475 | 0.677038202 | SCUBE3;GS |
| 0 | 0 | 1.032526475 | 0.677038202 | GPR132;PS |
| 0 | 0 | 1.089908141 | 0.713767904 | PDPN;TAP2 |
| 0 | 0 | 1.089908141 | 0.713767904 | CACNA1A;I |
| 0 | 0 | 1.089908141 | 0.713767904 | GK5;TNIP1 |
| 0 | 0 | 1.089908141 | 0.713767904 | MT2A;SLC3 |
| 0 | 0 | 1.089908141 | 0.713767904 | ATP2B1;M |
| 0 | 0 | 1.089908141 | 0.713767904 | NLGN3;SEN |
| 0 | 0 | 1.089908141 | 0.713767904 | GBP5;ARL5 |
| 0 | 0 | 1.089908141 | 0.713767904 | LRP4;NRXN |
| 0 | 0 | 1.089908141 | 0.713767904 | MYBPH;NN |
| 0 | 0 | 1.089908141 | 0.713767904 | PLCG2;RAB |
| 0 | 0 | 1.089908141 | 0.713767904 | SLC9A3R1; |
| 0 | 0 | 1.089908141 | 0.713767904 | IVNS1ABP; |
| 0 | 0 | 1.019094499 | 0.660725156 | CUL4A;PSN |
| 0 | 0 | 1.040343915 | 0.67378183 | PSMA2;OD |
| 0 | 0 | 1.040343915 | 0.67378183 | GNAL;ADO |
| 0 | 0 | 1.021743757 | 0.655513852 | ASAH1;SPT |
| 0 | 0 | 1.054721762 | 0.670627121 | ROCK1;ELN |
| 0 | 0 | 1.054721762 | 0.670627121 | MEF2A;KLF |
| 0 | 0 | 1.054721762 | 0.670627121 | ACVRL1;H1 |
| 0 | 0 | 1.054721762 | 0.670627121 | FLCN;IL6;S |
| 0 | 0 | 1.054721762 | 0.670627121 | DDX5;SAFE |
| 0 | 0 | 1.188985289 | 0.754321931 | SLC8A1;VE |
| 0 | 0 | 1.188985289 | 0.754321931 | FYN;SUPT6 |
| 0 | 0 | 1.188985289 | 0.754321931 | VCAN;HYA |
| 0 | 0 | 1.188985289 | 0.754321931 | NFKBIA;NF |
| 0 | 0 | 1.188985289 | 0.754321931 | TAL1;ATF4 |
| 0 | 0 | 1.188985289 | 0.754321931 | STX6;OPTN |
| 0 | 0 | 1.188985289 | 0.754321931 | BCL2L11;B |
| 0 | 0 | 1.188985289 | 0.754321931 | NF1;STRA6 |
| 0 | 0 | 1.188985289 | 0.754321931 | PARD3;NF1 |
| 0 | 0 | 1.188985289 | 0.754321931 | LTB4R2;CC |
| 0 | 0 | 1.188985289 | 0.754321931 | H1-4;ATAD |
| 0 | 0 | 1.188985289 | 0.754321931 | H1-4;ATAD |
| 0 | 0 | 1.188985289 | 0.754321931 | H1-4;ATAD |
| 0 | 0 | 1.188985289 | 0.754321931 | ELANE;PTP |
| 0 | 0 | 1.188985289 | 0.754321931 | IL10;EDN1 |
| 0 | 0 | 1.188985289 | 0.754321931 | DUSP1;SLA |
| 0 | 0 | 1.188985289 | 0.754321931 | ANKRD2;D |
| 0 | 0 | 1.188985289 | 0.754321931 | ACTN2;PTK |
| 0 | 0 | 1.188985289 | 0.754321931 | MPV17L;FY |
| 0 | 0 | 1.188985289 | 0.754321931 | HES1;N4BP |
| 0 | 0 | 1.188985289 | 0.754321931 | BIN2;SNX9 |
| 0 | 0 | 1.188985289 | 0.754321931 | B3GNT3;B3 |
| 0 | 0 | 1.188985289 | 0.754321931 | ODC1;SAT1 |
| 0 | 0 | 1.188985289 | 0.754321931 | CCL3;CCL2 |

| | | | | |
|---|---|---|---|---|
| 0 | 0 | 1.188985289 | 0.754321931 | PRKCE;CD2 |
| 0 | 0 | 1.188985289 | 0.754321931 | PUS10;GAI |
| 0 | 0 | 1.188985289 | 0.754321931 | GCH1;CALI |
| 0 | 0 | 1.188985289 | 0.754321931 | ROCK1;ACT |
| 0 | 0 | 1.188985289 | 0.754321931 | PNPT1;NEI |
| 0 | 0 | 1.188985289 | 0.754321931 | CLN3;RAB3 |
| 0 | 0 | 1.188985289 | 0.754321931 | GCLC;ROCI |
| 0 | 0 | 1.188985289 | 0.754321931 | H2AW;SIRT |
| 0 | 0 | 1.188985289 | 0.754321931 | GCLC;ROCI |
| 0 | 0 | 1.013082279 | 0.637137684 | LYN;SDCBF |
| 0 | 0 | 1.032489611 | 0.640549581 | RARG;NIPE |
| 0 | 0 | 1.032489611 | 0.640549581 | ANXA1;BTI |
| 0 | 0 | 1.032489611 | 0.640549581 | IL6;LRP5;TI |
| 0 | 0 | 1.032489611 | 0.640549581 | ACTN2;KCI |
| 0 | 0 | 1.014665606 | 0.628783487 | CCNT1;ELL |
| 0 | 0 | 1.005970932 | 0.621128867 | HAL;SDS;N |
| 0 | 0 | 1.089874214 | 0.670293799 | ADCY4;AD( |
| 0 | 0 | 1.089874214 | 0.670293799 | MID1IP1;P |
| 0 | 0 | 1.089874214 | 0.670293799 | MICU1;SLC |
| 0 | 0 | 1.089874214 | 0.670293799 | ACTN2;PRI |
| 0 | 0 | 1.089874214 | 0.670293799 | ATXN7;MII |
| 0 | 0 | 1.089874214 | 0.670293799 | TMBIM1;N |
| 0 | 0 | 1.089874214 | 0.670293799 | TMBIM1;N |
| 0 | 0 | 1.089874214 | 0.670293799 | SPRY1;CYR |
| 0 | 0 | 1.089874214 | 0.670293799 | ROCK1;PTI |
| 0 | 0 | 1.089874214 | 0.670293799 | RPUSD4;TS |
| 0 | 0 | 1.089874214 | 0.670293799 | SRC;ACOD |
| 0 | 0 | 1.089874214 | 0.670293799 | GAR1;TFB1 |
| 0 | 0 | 1.089874214 | 0.670293799 | DYNC1H1;I |
| 0 | 0 | 1.017166373 | 0.6176905 | SEMA4A;SI |
| 0 | 0 | 1.017166373 | 0.6176905 | GHSR;SEM |
| 0 | 0 | 1.000026738 | 0.606955342 | IVNS1ABP; |
| 0 | 0 | 1.046251416 | 0.628477449 | DYNC1H1;/ |
| 0 | 0 | 1.046251416 | 0.628477449 | FUS;FMR1; |
| 0 | 0 | 1.046251416 | 0.628477449 | DDX3X;CN( |
| 0 | 0 | 1.046251416 | 0.628477449 | MT2A;HAA |
| 0 | 0 | 0.998953865 | 0.596862942 | DDX5;IL6;S |
| 0 | 0 | 0.994983278 | 0.594240674 | TCF7L2;PH |
| 0 | 0 | 1.0217027 | 0.601600525 | CLN3;GRIN |
| 0 | 0 | 1.0217027 | 0.601600525 | NRP1;YRD( |
| 0 | 0 | 1.0217027 | 0.601600525 | RYR1;IGSF8 |
| 0 | 0 | 0.993179607 | 0.583807096 | ITCH;PSMA |
| 0 | 0 | 0.990667171 | 0.570692439 | FLCN;PLK3 |
| 0 | 0 | 0.990667171 | 0.570692439 | PDXK;VNN |
| 0 | 0 | 0.994996549 | 0.566342183 | ACVRL1;BC |
| 0 | 0 | 0.982802829 | 0.557989223 | FCN1;RTP4 |
| 0 | 0 | 0.986924421 | 0.553385991 | GALNT11;( |

| | | | | |
|---|---|---|---|---|
| 0 | 0 | 1.089840312 | 0.609333531 | LRP5;IFITM |
| 0 | 0 | 1.089840312 | 0.609333531 | SLC1A2;SL( |
| 0 | 0 | 1.089840312 | 0.609333531 | SLC24A1;S |
| 0 | 0 | 1.089840312 | 0.609333531 | MT2A;ATP |
| 0 | 0 | 1.089840312 | 0.609333531 | ABCA13;SC |
| 0 | 0 | 1.089840312 | 0.609333531 | SLC25A16; |
| 0 | 0 | 1.089840312 | 0.609333531 | ACVRL1;KD |
| 0 | 0 | 1.089840312 | 0.609333531 | TIFAB;STR/ |
| 0 | 0 | 1.089840312 | 0.609333531 | ATP1A1;KC |
| 0 | 0 | 1.089840312 | 0.609333531 | DGAT2;PL/ |
| 0 | 0 | 1.089840312 | 0.609333531 | MMRN2;KI |
| 0 | 0 | 1.089840312 | 0.609333531 | EDN1;SERF |
| 0 | 0 | 1.089840312 | 0.609333531 | LTB4R2;CC |
| 0 | 0 | 1.089840312 | 0.609333531 | RELA;PPAR |
| 0 | 0 | 1.089840312 | 0.609333531 | PTPRC;SM/ |
| 0 | 0 | 1.089840312 | 0.609333531 | SESN2;DEP |
| 0 | 0 | 1.089840312 | 0.609333531 | FMR1;AGC |
| 0 | 0 | 1.089840312 | 0.609333531 | CLN3;TIFAI |
| 0 | 0 | 1.089840312 | 0.609333531 | SEH1L;NUF |
| 0 | 0 | 1.089840312 | 0.609333531 | PNP;CMPK |
| 0 | 0 | 1.089840312 | 0.609333531 | CMPK2;SM |
| 0 | 0 | 1.089840312 | 0.609333531 | PNP;SOCS1 |
| 0 | 0 | 1.089840312 | 0.609333531 | SIRT1;PTPI |
| 0 | 0 | 1.089840312 | 0.609333531 | SLC2A10;C |
| 0 | 0 | 1.089840312 | 0.609333531 | CSF1;PTK2 |
| 0 | 0 | 1.089840312 | 0.609333531 | ACTN2;ATF |
| 0 | 0 | 1.089840312 | 0.609333531 | NIPBL;IFNC |
| 0 | 0 | 1.089840312 | 0.609333531 | JAK2;TGFB |
| 0 | 0 | 1.089840312 | 0.609333531 | CD81;LAM |
| 0 | 0 | 1.089840312 | 0.609333531 | UPP1;DCK |
| 0 | 0 | 1.089840312 | 0.609333531 | BST1;SLC2/ |
| 0 | 0 | 1.089840312 | 0.609333531 | PDPN;CD4 |
| 0 | 0 | 1.089840312 | 0.609333531 | RIN2;XDH |
| 0 | 0 | 1.089840312 | 0.609333531 | RFK;ADSS2 |
| 0 | 0 | 1.089840312 | 0.609333531 | NRROS;CD |
| 0 | 0 | 1.089840312 | 0.609333531 | ALKBH1;DF |
| 0 | 0 | 1.089840312 | 0.609333531 | ADRB3;SOI |
| 0 | 0 | 0.978636335 | 0.546200369 | LTV1;RPL1( |
| 0 | 0 | 0.978063961 | 0.544956454 | ROCK1;FUS |
| 0 | 0 | 1.03245283 | 0.574111396 | DDX3X;ZF/ |
| 0 | 0 | 1.03245283 | 0.574111396 | DGAT2;ACS |
| 0 | 0 | 1.03245283 | 0.574111396 | FITM1;RAE |
| 0 | 0 | 1.03245283 | 0.574111396 | WTAP;TRM |
| 0 | 0 | 1.03245283 | 0.574111396 | ZFP36;PAF |
| 0 | 0 | 1.03245283 | 0.574111396 | PPP1R15A; |
| 0 | 0 | 1.03245283 | 0.574111396 | PGP;SIK1;C |
| 0 | 0 | 1.03245283 | 0.574111396 | MBOAT1;P |

| | | | | | |
|---|---|---|---|---|---|
| 0 | 0 | 1.03245283 | 0.574111396 | VMP1;P2R | . |
| 0 | 0 | 1.03245283 | 0.574111396 | LTB4R2;NF | . |
| 0 | 0 | 1.03245283 | 0.574111396 | FAP;DLC1;[illegible] | . |
| 0 | 0 | 1.307843137 | 0.725364338 | NRG1 | . |
| 0 | 0 | 1.307843137 | 0.725364338 | GK5 | . |
| 0 | 0 | 1.307843137 | 0.725364338 | SLC25A29 | . |
| 0 | 0 | 1.307843137 | 0.725364338 | ATP11B | . |
| 0 | 0 | 1.307843137 | 0.725364338 | RAMP2 | . |
| 0 | 0 | 1.307843137 | 0.725364338 | ASS1 | . |
| 0 | 0 | 1.307843137 | 0.725364338 | ACVRL1 | . |
| 0 | 0 | 1.307843137 | 0.725364338 | SLC25A29 | . |
| 0 | 0 | 1.307843137 | 0.725364338 | ARHGEF2 | . |
| 0 | 0 | 1.307843137 | 0.725364338 | NFKBIA | . |
| 0 | 0 | 1.307843137 | 0.725364338 | ZFP36 | . |
| 0 | 0 | 1.307843137 | 0.725364338 | B2M | . |
| 0 | 0 | 1.307843137 | 0.725364338 | NIPBL | . |
| 0 | 0 | 1.307843137 | 0.725364338 | CLN3 | . |
| 0 | 0 | 1.307843137 | 0.725364338 | LDLR | . |
| 0 | 0 | 1.307843137 | 0.725364338 | SLC44A2 | . |
| 0 | 0 | 1.307843137 | 0.725364338 | CSGALNAC | . |
| 0 | 0 | 1.307843137 | 0.725364338 | NUDT3 | . |
| 0 | 0 | 1.307843137 | 0.725364338 | LIG4 | . |
| 0 | 0 | 1.307843137 | 0.725364338 | RARG | . |
| 0 | 0 | 1.307843137 | 0.725364338 | SLC2A10 | . |
| 0 | 0 | 1.307843137 | 0.725364338 | RPUSD4 | . |
| 0 | 0 | 1.307843137 | 0.725364338 | CTSH | . |
| 0 | 0 | 1.307843137 | 0.725364338 | ASRGL1 | . |
| 0 | 0 | 1.307843137 | 0.725364338 | NPHP1 | . |
| 0 | 0 | 1.307843137 | 0.725364338 | STAT1 | . |
| 0 | 0 | 1.307843137 | 0.725364338 | UBE2S | . |
| 0 | 0 | 1.307843137 | 0.725364338 | LRP5 | . |
| 0 | 0 | 1.307843137 | 0.725364338 | WDR77 | . |
| 0 | 0 | 1.307843137 | 0.725364338 | PTPRO | . |
| 0 | 0 | 1.307843137 | 0.725364338 | PECAM1 | . |
| 0 | 0 | 1.307843137 | 0.725364338 | GK5 | . |
| 0 | 0 | 1.307843137 | 0.725364338 | CLN3 | . |
| 0 | 0 | 1.307843137 | 0.725364338 | GALC | . |
| 0 | 0 | 1.307843137 | 0.725364338 | ST6GALNA | . |
| 0 | 0 | 1.307843137 | 0.725364338 | GHSR | . |
| 0 | 0 | 1.307843137 | 0.725364338 | LRRC8C | . |
| 0 | 0 | 1.307843137 | 0.725364338 | ANXA1 | . |
| 0 | 0 | 1.307843137 | 0.725364338 | SLC22A3 | . |
| 0 | 0 | 1.307843137 | 0.725364338 | MAOB | . |
| 0 | 0 | 1.307843137 | 0.725364338 | SQOR | . |
| 0 | 0 | 1.307843137 | 0.725364338 | ATP7A | . |
| 0 | 0 | 1.307843137 | 0.725364338 | SLC4A7 | . |
| 0 | 0 | 1.307843137 | 0.725364338 | PSAT1 | . |

| | | | | | |
|---|---|---|---|---|---|
| 0 | 0 | 1.307843137 | 0.725364338 | SLC11A1 | . |
| 0 | 0 | 1.307843137 | 0.725364338 | STAT1 | . |
| 0 | 0 | 1.307843137 | 0.725364338 | TCF7L2 | . |
| 0 | 0 | 1.307843137 | 0.725364338 | ABHD1 | . |
| 0 | 0 | 1.307843137 | 0.725364338 | HORMAD1 | . |
| 0 | 0 | 1.307843137 | 0.725364338 | STAT1 | . |
| 0 | 0 | 1.307843137 | 0.725364338 | KLF4 | . |
| 0 | 0 | 1.307843137 | 0.725364338 | TACC3 | . |
| 0 | 0 | 1.307843137 | 0.725364338 | TACC3 | . |
| 0 | 0 | 1.307843137 | 0.725364338 | SRSF12 | . |
| 0 | 0 | 1.307843137 | 0.725364338 | CYP4F2 | . |
| 0 | 0 | 1.307843137 | 0.725364338 | LDLR | . |
| 0 | 0 | 1.307843137 | 0.725364338 | TGM2 | . |
| 0 | 0 | 1.307843137 | 0.725364338 | USP25 | . |
| 0 | 0 | 1.307843137 | 0.725364338 | ZFP36 | . |
| 0 | 0 | 1.307843137 | 0.725364338 | LRRK2 | . |
| 0 | 0 | 1.307843137 | 0.725364338 | LRRK2 | . |
| 0 | 0 | 1.307843137 | 0.725364338 | E2F3 | . |
| 0 | 0 | 1.307843137 | 0.725364338 | HES1 | . |
| 0 | 0 | 1.307843137 | 0.725364338 | CRY2 | . |
| 0 | 0 | 1.307843137 | 0.725364338 | INPP5K | . |
| 0 | 0 | 1.307843137 | 0.725364338 | N4BP2L2 | . |
| 0 | 0 | 1.307843137 | 0.725364338 | PIH1D1 | . |
| 0 | 0 | 1.307843137 | 0.725364338 | DUSP1 | . |
| 0 | 0 | 1.307843137 | 0.725364338 | TNF | . |
| 0 | 0 | 1.307843137 | 0.725364338 | SRGAP2 | . |
| 0 | 0 | 1.307843137 | 0.725364338 | SLC2A10 | . |
| 0 | 0 | 1.307843137 | 0.725364338 | PTPN2 | . |
| 0 | 0 | 1.307843137 | 0.725364338 | SIK1 | . |
| 0 | 0 | 1.307843137 | 0.725364338 | HNF1B | . |
| 0 | 0 | 1.307843137 | 0.725364338 | GRIPAP1 | . |
| 0 | 0 | 1.307843137 | 0.725364338 | DPH3 | . |
| 0 | 0 | 1.307843137 | 0.725364338 | DPH3 | . |
| 0 | 0 | 1.307843137 | 0.725364338 | CREBBP | . |
| 0 | 0 | 1.307843137 | 0.725364338 | ACTN4 | . |
| 0 | 0 | 1.307843137 | 0.725364338 | MYBL1 | . |
| 0 | 0 | 1.307843137 | 0.725364338 | FLOT1 | . |
| 0 | 0 | 1.307843137 | 0.725364338 | ACVR1B | . |
| 0 | 0 | 1.307843137 | 0.725364338 | CYP4F2 | . |
| 0 | 0 | 1.307843137 | 0.725364338 | CALCOCO2 | . |
| 0 | 0 | 1.307843137 | 0.725364338 | HK2 | . |
| 0 | 0 | 1.307843137 | 0.725364338 | CXCL13 | . |
| 0 | 0 | 1.307843137 | 0.725364338 | S1PR2 | . |
| 0 | 0 | 1.307843137 | 0.725364338 | LIF | . |
| 0 | 0 | 1.307843137 | 0.725364338 | S1PR2 | . |
| 0 | 0 | 1.307843137 | 0.725364338 | ATF3 | . |
| 0 | 0 | 1.307843137 | 0.725364338 | TRPC6 | . |

| | | | | | |
|---|---|---|---|---|---|
| 0 | 0 | 1.307843137 | 0.725364338 | ANXA2 | . |
| 0 | 0 | 1.307843137 | 0.725364338 | CCL3 | . |
| 0 | 0 | 1.307843137 | 0.725364338 | STX1B | . |
| 0 | 0 | 1.307843137 | 0.725364338 | CHP1 | . |
| 0 | 0 | 1.307843137 | 0.725364338 | FMR1 | . |
| 0 | 0 | 1.307843137 | 0.725364338 | NIBAN2 | . |
| 0 | 0 | 1.307843137 | 0.725364338 | TIRAP | . |
| 0 | 0 | 1.307843137 | 0.725364338 | PLEKHA4 | . |
| 0 | 0 | 1.307843137 | 0.725364338 | NRXN2 | . |
| 0 | 0 | 1.307843137 | 0.725364338 | CAST | . |
| 0 | 0 | 1.307843137 | 0.725364338 | HNF1B | . |
| 0 | 0 | 1.307843137 | 0.725364338 | SERPINB4 | . |
| 0 | 0 | 1.307843137 | 0.725364338 | TNFAIP3 | . |
| 0 | 0 | 1.307843137 | 0.725364338 | UBE2S | . |
| 0 | 0 | 1.307843137 | 0.725364338 | ARL13B | . |
| 0 | 0 | 1.307843137 | 0.725364338 | SQSTM1 | . |
| 0 | 0 | 1.307843137 | 0.725364338 | MSRB3 | . |
| 0 | 0 | 1.307843137 | 0.725364338 | VCPIP1 | . |
| 0 | 0 | 1.307843137 | 0.725364338 | TM9SF4 | . |
| 0 | 0 | 1.307843137 | 0.725364338 | PNP | . |
| 0 | 0 | 1.307843137 | 0.725364338 | TENT4B | . |
| 0 | 0 | 1.307843137 | 0.725364338 | SGK1 | . |
| 0 | 0 | 1.307843137 | 0.725364338 | RAB3GAP1 | . |
| 0 | 0 | 1.307843137 | 0.725364338 | RIN2 | . |
| 0 | 0 | 1.307843137 | 0.725364338 | SEMA4A | . |
| 0 | 0 | 1.307843137 | 0.725364338 | PTPRO | . |
| 0 | 0 | 1.307843137 | 0.725364338 | IL6 | . |
| 0 | 0 | 1.307843137 | 0.725364338 | MIDN | . |
| 0 | 0 | 1.307843137 | 0.725364338 | FYN | . |
| 0 | 0 | 1.307843137 | 0.725364338 | XBP1 | . |
| 0 | 0 | 1.307843137 | 0.725364338 | NFKB1 | . |
| 0 | 0 | 1.307843137 | 0.725364338 | RAC2 | . |
| 0 | 0 | 1.307843137 | 0.725364338 | ACSL5 | . |
| 0 | 0 | 1.307843137 | 0.725364338 | PTK2 | . |
| 0 | 0 | 1.307843137 | 0.725364338 | DUSP1 | . |
| 0 | 0 | 1.307843137 | 0.725364338 | DUSP1 | . |
| 0 | 0 | 1.307843137 | 0.725364338 | SUPT6H | . |
| 0 | 0 | 1.307843137 | 0.725364338 | VEGFA | . |
| 0 | 0 | 1.307843137 | 0.725364338 | OAS2 | . |
| 0 | 0 | 1.307843137 | 0.725364338 | MAP1B | . |
| 0 | 0 | 1.307843137 | 0.725364338 | RAB3GAP1 | . |
| 0 | 0 | 1.307843137 | 0.725364338 | PDE4B | . |
| 0 | 0 | 1.307843137 | 0.725364338 | LRRK2 | . |
| 0 | 0 | 1.307843137 | 0.725364338 | SUPT6H | . |
| 0 | 0 | 1.307843137 | 0.725364338 | TIFAB | . |
| 0 | 0 | 1.307843137 | 0.725364338 | STXBP1 | . |
| 0 | 0 | 1.307843137 | 0.725364338 | IL27 | . |

| | | | | | |
|---|---|---|---|---|---|
| 0 | 0 | 1.307843137 | 0.725364338 | JAK3 | . |
| 0 | 0 | 1.307843137 | 0.725364338 | BCOR | . |
| 0 | 0 | 1.307843137 | 0.725364338 | ALKBH1 | . |
| 0 | 0 | 1.307843137 | 0.725364338 | FMR1 | . |
| 0 | 0 | 1.307843137 | 0.725364338 | SMAD7 | . |
| 0 | 0 | 1.307843137 | 0.725364338 | APOA2 | . |
| 0 | 0 | 1.307843137 | 0.725364338 | PTPRO | . |
| 0 | 0 | 1.307843137 | 0.725364338 | NR4A3 | . |
| 0 | 0 | 1.307843137 | 0.725364338 | ZFP36 | . |
| 0 | 0 | 1.307843137 | 0.725364338 | EGR1 | . |
| 0 | 0 | 1.307843137 | 0.725364338 | LRRK2 | . |
| 0 | 0 | 1.307843137 | 0.725364338 | NCOA4 | . |
| 0 | 0 | 1.307843137 | 0.725364338 | PIH1D1 | . |
| 0 | 0 | 1.307843137 | 0.725364338 | LDLR | . |
| 0 | 0 | 1.307843137 | 0.725364338 | SLC13A3 | . |
| 0 | 0 | 1.307843137 | 0.725364338 | DYSF | . |
| 0 | 0 | 1.307843137 | 0.725364338 | LRRK2 | . |
| 0 | 0 | 1.307843137 | 0.725364338 | TIFAB | . |
| 0 | 0 | 1.307843137 | 0.725364338 | SERPINB1 | . |
| 0 | 0 | 1.307843137 | 0.725364338 | VEGFA | . |
| 0 | 0 | 1.307843137 | 0.725364338 | MEF2A | . |
| 0 | 0 | 1.307843137 | 0.725364338 | TGFBR1 | . |
| 0 | 0 | 1.307843137 | 0.725364338 | SLC22A1 | . |
| 0 | 0 | 0.980729955 | 0.542231455 | CCNT1;ELL | . |
| 0 | 0 | 0.976924417 | 0.539187212 | NCOA1;SL( | . |
| 0 | 0 | 1.005952899 | 0.55419997 | ATP1A1;SC | . |
| 0 | 0 | 1.005952899 | 0.55419997 | TPPP3;NDE | . |
| 0 | 0 | 1.005952899 | 0.55419997 | SLC9A3R1; | . |
| 0 | 0 | 1.005952899 | 0.55419997 | TRIR;XRN1 | . |
| 0 | 0 | 1.005952899 | 0.55419997 | LFNG;NOT( | . |
| 0 | 0 | 1.005952899 | 0.55419997 | KMT5C;TE | . |
| 0 | 0 | 1.005952899 | 0.55419997 | MAD2L2;FI | . |
| 0 | 0 | 0.975824623 | 0.53238819 | ACOT9;GP/ | . |
| 0 | 0 | 0.990684794 | 0.540051722 | ACTN2;KCI | . |
| 0 | 0 | 0.990684794 | 0.540051722 | SARS1;PPA | . |
| 0 | 0 | 0.990684794 | 0.540051722 | HES1;MDN | . |
| 0 | 0 | 0.973630602 | 0.529583357 | TRIR;UTP1 | . |
| 0 | 0 | 0.980751794 | 0.528812994 | ABCA1;STE | . |
| 0 | 0 | 0.980751794 | 0.528812994 | SMG1;ALYI | . |
| 0 | 0 | 0.971842584 | 0.523541201 | FZD1;PSM/ | . |
| 0 | 0 | 0.973770123 | 0.519312555 | DDX17;DD | . |
| 0 | 0 | 0.973770123 | 0.519312555 | PSMA2;OD | . |
| 0 | 0 | 0.973770123 | 0.519312555 | DESI1;FMR | . |
| 0 | 0 | 0.973770123 | 0.519312555 | ELOVL5;PN | . |
| 0 | 0 | 0.968544463 | 0.515477167 | RYR1;KCNJ | . |
| 0 | 0 | 0.968544463 | 0.515477167 | SLC24A1;E | . |
| 0 | 0 | 0.968496749 | 0.515088686 | GGT5;XBP | . |

| | | | | |
|---|---|---|---|---|
| 0 | 0 | 0.965767045 | 0.50804538 | DYNC1H1;S |
| 0 | 0 | 0.964596612 | 0.503460438 | SETX;ZNF4 |
| 0 | 0 | 0.964596612 | 0.503460438 | ABCA1;STA |
| 0 | 0 | 0.95882917 | 0.490203763 | BTG2;RIDA |
| 0 | 0 | 0.968639459 | 0.489345062 | CHKA;PNPI |
| 0 | 0 | 0.968639459 | 0.489345062 | P2RY12;ST |
| 0 | 0 | 0.968639459 | 0.489345062 | ARRDC4;FZ |
| 0 | 0 | 0.968639459 | 0.489345062 | CHST11;CS |
| 0 | 0 | 0.968639459 | 0.489345062 | STXBP3;ST |
| 0 | 0 | 0.968639459 | 0.489345062 | CYP4F2;AD |
| 0 | 0 | 0.96149147 | 0.485094606 | TRPC6;PPII |
| 0 | 0 | 0.980773585 | 0.493214993 | DNAH17;C |
| 0 | 0 | 0.980773585 | 0.493214993 | CCDC88B;C |
| 0 | 0 | 0.980773585 | 0.493214993 | CTSH;ITGA |
| 0 | 0 | 0.980773585 | 0.493214993 | PLK3;NABP |
| 0 | 0 | 0.980773585 | 0.493214993 | LRCH1;FICI |
| 0 | 0 | 0.980773585 | 0.493214993 | FUT4;ST3G |
| 0 | 0 | 0.980773585 | 0.493214993 | PIH1D1;IL1 |
| 0 | 0 | 0.980773585 | 0.493214993 | MED13;ME |
| 0 | 0 | 0.980773585 | 0.493214993 | HDAC4;LYF |
| 0 | 0 | 0.980773585 | 0.493214993 | PTK2B;FYN |
| 0 | 0 | 0.980773585 | 0.493214993 | LRRK2;ID2; |
| 0 | 0 | 0.980773585 | 0.493214993 | SERPINB4; |
| 0 | 0 | 0.980773585 | 0.493214993 | SRI;CALM1 |
| 0 | 0 | 0.980773585 | 0.493214993 | SLC2A10;JA |
| 0 | 0 | 0.980773585 | 0.493214993 | ASAH1;EPH |
| 0 | 0 | 0.980773585 | 0.493214993 | IL1A;DAXX |
| 0 | 0 | 0.956775733 | 0.480678685 | PM20D2;E |
| 0 | 0 | 0.953428131 | 0.476219393 | GK5;DGAT |
| 0 | 0 | 0.950926276 | 0.471783557 | SERPINB10 |
| 0 | 0 | 0.950926276 | 0.471783557 | DDX3X;CN |
| 0 | 0 | 1.005948408 | 0.496465301 | NUDT7;PM |
| 0 | 0 | 1.005948408 | 0.496465301 | STARD3;TS |
| 0 | 0 | 1.005948408 | 0.496465301 | SLC24A1;S |
| 0 | 0 | 1.005948408 | 0.496465301 | MEF2A;MY |
| 0 | 0 | 1.005948408 | 0.496465301 | HIP1R;PICA |
| 0 | 0 | 1.005948408 | 0.496465301 | VCAN;CSGA |
| 0 | 0 | 1.005948408 | 0.496465301 | NIPBL;TGFI |
| 0 | 0 | 1.005948408 | 0.496465301 | ID2;JAK2 |
| 0 | 0 | 1.005948408 | 0.496465301 | ATP1A1;KC |
| 0 | 0 | 1.005948408 | 0.496465301 | PNPT1;TFB |
| 0 | 0 | 1.005948408 | 0.496465301 | SIRT1;PIAS |
| 0 | 0 | 1.005948408 | 0.496465301 | NOS2;FFAF |
| 0 | 0 | 1.005948408 | 0.496465301 | LRRK2;SER |
| 0 | 0 | 1.005948408 | 0.496465301 | H1-4;PAF1 |
| 0 | 0 | 1.005948408 | 0.496465301 | ADAM19;P |
| 0 | 0 | 1.005948408 | 0.496465301 | CYP27B1;V |

| | | | | | |
|---|---|---|---|---|---|
| 0 | 0 | 1.005948408 | 0.496465301 | NOS2;NF1 | . |
| 0 | 0 | 1.005948408 | 0.496465301 | TCF7L2;IL1 | . |
| 0 | 0 | 1.005948408 | 0.496465301 | XBP1;CYP4 | . |
| 0 | 0 | 1.005948408 | 0.496465301 | EGLN3;JMJ | . |
| 0 | 0 | 1.005948408 | 0.496465301 | ATP7A;CO) | . |
| 0 | 0 | 1.005948408 | 0.496465301 | PSEN1;LDL | . |
| 0 | 0 | 1.005948408 | 0.496465301 | TMSB4X;PI | . |
| 0 | 0 | 1.005948408 | 0.496465301 | THEMIS2;C | . |
| 0 | 0 | 1.005948408 | 0.496465301 | FITM1;OSE | . |
| 0 | 0 | 1.005948408 | 0.496465301 | CLN3;STXB | . |
| 0 | 0 | 1.005948408 | 0.496465301 | GRIN3A;AD | . |
| 0 | 0 | 0.9489837 | 0.467407614 | ACVRL1;DD | . |
| 0 | 0 | 0.9489837 | 0.467407614 | XBP1;ELOV | . |
| 0 | 0 | 0.9489837 | 0.467407614 | IL1A;MT2A | . |
| 0 | 0 | 0.947430182 | 0.46311108 | ROCK1;LRF | . |
| 0 | 0 | 0.945096554 | 0.454790083 | DYNC1H1;I | . |
| 0 | 0 | 0.945096554 | 0.454790083 | MN1;CDKN | . |
| 0 | 0 | 0.945096554 | 0.454790083 | SLC24A1;E | . |
| 0 | 0 | 0.952252939 | 0.457625588 | UTP15;RPS | . |
| 0 | 0 | 0.943422024 | 0.446844934 | AMER1;LRI | . |
| 0 | 0 | 0.933941389 | 0.437018436 | ING5;MOR | . |
| 0 | 0 | 0.933941389 | 0.437018436 | DGKG;DGK | . |
| 0 | 0 | 0.933916424 | 0.436717801 | ARHGEF11 | . |
| 0 | 0 | 0.933966336 | 0.435961774 | NIPBL;CXCI | . |
| 0 | 0 | 0.933966336 | 0.435961774 | ATXN1;RGS | . |
| 0 | 0 | 0.933966336 | 0.435961774 | LTV1;NOM | . |
| 0 | 0 | 0.933966336 | 0.435961774 | GGT5;NUD | . |
| 0 | 0 | 0.933966336 | 0.435961774 | STX1B;STX4 | . |
| 0 | 0 | 0.933891439 | 0.43557254 | CXCL10;CX | . |
| 0 | 0 | 0.933991264 | 0.432584849 | IL10;CD40; | . |
| 0 | 0 | 0.933991264 | 0.432584849 | LAMB3;PA | . |
| 0 | 0 | 0.933991264 | 0.432584849 | OTUB2;MA | . |
| 0 | 0 | 0.933991264 | 0.432584849 | ATXN7;MII | . |
| 0 | 0 | 0.933991264 | 0.432584849 | C3;NR4A3; | . |
| 0 | 0 | 0.933991264 | 0.432584849 | PPP1R10;N | . |
| 0 | 0 | 0.933991264 | 0.432584849 | MOV10;PD | . |
| 0 | 0 | 0.933991264 | 0.432584849 | IGSF8;SVIL | . |
| 0 | 0 | 0.933841414 | 0.431822245 | SPART;PTP | . |
| 0 | 0 | 1.089806435 | 0.502190397 | DGAT2 | . |
| 0 | 0 | 1.089806435 | 0.502190397 | GNAL | . |
| 0 | 0 | 1.089806435 | 0.502190397 | ALDH3B1 | . |
| 0 | 0 | 1.089806435 | 0.502190397 | DCHS1 | . |
| 0 | 0 | 1.089806435 | 0.502190397 | SCN4B | . |
| 0 | 0 | 1.089806435 | 0.502190397 | B4GALT5 | . |
| 0 | 0 | 1.089806435 | 0.502190397 | RAMP2 | . |
| 0 | 0 | 1.089806435 | 0.502190397 | PTGER2 | . |
| 0 | 0 | 1.089806435 | 0.502190397 | LDLR | . |

| | | | | | |
|---|---|---|---|---|---|
| 0 | 0 | 1.089806435 | 0.502190397 | ASS1 | . |
| 0 | 0 | 1.089806435 | 0.502190397 | DDR2 | . |
| 0 | 0 | 1.089806435 | 0.502190397 | SLC6A8 | . |
| 0 | 0 | 1.089806435 | 0.502190397 | LRRC8C | . |
| 0 | 0 | 1.089806435 | 0.502190397 | PTK2 | . |
| 0 | 0 | 1.089806435 | 0.502190397 | TMEM120/ | . |
| 0 | 0 | 1.089806435 | 0.502190397 | LIG4 | . |
| 0 | 0 | 1.089806435 | 0.502190397 | IGSF3 | . |
| 0 | 0 | 1.089806435 | 0.502190397 | RFK | . |
| 0 | 0 | 1.089806435 | 0.502190397 | SAG | . |
| 0 | 0 | 1.089806435 | 0.502190397 | NEU1 | . |
| 0 | 0 | 1.089806435 | 0.502190397 | DLL1 | . |
| 0 | 0 | 1.089806435 | 0.502190397 | UGCG | . |
| 0 | 0 | 1.089806435 | 0.502190397 | PLK3 | . |
| 0 | 0 | 1.089806435 | 0.502190397 | FLVCR1 | . |
| 0 | 0 | 1.089806435 | 0.502190397 | LRP5 | . |
| 0 | 0 | 1.089806435 | 0.502190397 | RARG | . |
| 0 | 0 | 1.089806435 | 0.502190397 | SETDB1 | . |
| 0 | 0 | 1.089806435 | 0.502190397 | NLGN3 | . |
| 0 | 0 | 1.089806435 | 0.502190397 | EREG | . |
| 0 | 0 | 1.089806435 | 0.502190397 | ATG7 | . |
| 0 | 0 | 1.089806435 | 0.502190397 | SLC24A1 | . |
| 0 | 0 | 1.089806435 | 0.502190397 | NRROS | . |
| 0 | 0 | 1.089806435 | 0.502190397 | ME2 | . |
| 0 | 0 | 1.089806435 | 0.502190397 | TENT4B | . |
| 0 | 0 | 1.089806435 | 0.502190397 | SFPQ | . |
| 0 | 0 | 1.089806435 | 0.502190397 | KAT2B | . |
| 0 | 0 | 1.089806435 | 0.502190397 | FMR1 | . |
| 0 | 0 | 1.089806435 | 0.502190397 | GRIN3A | . |
| 0 | 0 | 1.089806435 | 0.502190397 | OTUB2 | . |
| 0 | 0 | 1.089806435 | 0.502190397 | TFIP11 | . |
| 0 | 0 | 1.089806435 | 0.502190397 | DLL1 | . |
| 0 | 0 | 1.089806435 | 0.502190397 | INPP5K | . |
| 0 | 0 | 1.089806435 | 0.502190397 | SRI | . |
| 0 | 0 | 1.089806435 | 0.502190397 | INSIG1 | . |
| 0 | 0 | 1.089806435 | 0.502190397 | ID2 | . |
| 0 | 0 | 1.089806435 | 0.502190397 | HORMAD1 | . |
| 0 | 0 | 1.089806435 | 0.502190397 | MPV17L | . |
| 0 | 0 | 1.089806435 | 0.502190397 | MEFV | . |
| 0 | 0 | 1.089806435 | 0.502190397 | CHP1 | . |
| 0 | 0 | 1.089806435 | 0.502190397 | CHP1 | . |
| 0 | 0 | 1.089806435 | 0.502190397 | ADAR | . |
| 0 | 0 | 1.089806435 | 0.502190397 | DHX58 | . |
| 0 | 0 | 1.089806435 | 0.502190397 | SOX15 | . |
| 0 | 0 | 1.089806435 | 0.502190397 | GRIPAP1 | . |
| 0 | 0 | 1.089806435 | 0.502190397 | ATP7A | . |
| 0 | 0 | 1.089806435 | 0.502190397 | STRA6 | . |

| | | | | | |
|---|---|---|---|---|---|
| 0 | 0 | 1.089806435 | 0.502190397 | PSEN1 | . |
| 0 | 0 | 1.089806435 | 0.502190397 | ZFP36 | . |
| 0 | 0 | 1.089806435 | 0.502190397 | IPCEF1 | . |
| 0 | 0 | 1.089806435 | 0.502190397 | VNN2 | . |
| 0 | 0 | 1.089806435 | 0.502190397 | RPIA | . |
| 0 | 0 | 1.089806435 | 0.502190397 | SQSTM1 | . |
| 0 | 0 | 1.089806435 | 0.502190397 | HES1 | . |
| 0 | 0 | 1.089806435 | 0.502190397 | STAT3 | . |
| 0 | 0 | 1.089806435 | 0.502190397 | ABCA1 | . |
| 0 | 0 | 1.089806435 | 0.502190397 | SAT1 | . |
| 0 | 0 | 1.089806435 | 0.502190397 | ATG7 | . |
| 0 | 0 | 1.089806435 | 0.502190397 | NF1 | . |
| 0 | 0 | 1.089806435 | 0.502190397 | CGAS | . |
| 0 | 0 | 1.089806435 | 0.502190397 | TGFBR1 | . |
| 0 | 0 | 1.089806435 | 0.502190397 | GRK2 | . |
| 0 | 0 | 1.089806435 | 0.502190397 | PIH1D1 | . |
| 0 | 0 | 1.089806435 | 0.502190397 | PAK1 | . |
| 0 | 0 | 1.089806435 | 0.502190397 | PAK1 | . |
| 0 | 0 | 1.089806435 | 0.502190397 | OPTN | . |
| 0 | 0 | 1.089806435 | 0.502190397 | FMR1 | . |
| 0 | 0 | 1.089806435 | 0.502190397 | GHSR | . |
| 0 | 0 | 1.089806435 | 0.502190397 | CHP1 | . |
| 0 | 0 | 1.089806435 | 0.502190397 | ATP1B3 | . |
| 0 | 0 | 1.089806435 | 0.502190397 | RGS2 | . |
| 0 | 0 | 1.089806435 | 0.502190397 | ADCY8 | . |
| 0 | 0 | 1.089806435 | 0.502190397 | SOD2 | . |
| 0 | 0 | 1.089806435 | 0.502190397 | PRODH | . |
| 0 | 0 | 1.089806435 | 0.502190397 | TFIP11 | . |
| 0 | 0 | 1.089806435 | 0.502190397 | ARL13B | . |
| 0 | 0 | 1.089806435 | 0.502190397 | DDX3X | . |
| 0 | 0 | 1.089806435 | 0.502190397 | ENPP4 | . |
| 0 | 0 | 1.089806435 | 0.502190397 | SIRT1 | . |
| 0 | 0 | 1.089806435 | 0.502190397 | VEGFA | . |
| 0 | 0 | 1.089806435 | 0.502190397 | SLC8B1 | . |
| 0 | 0 | 1.089806435 | 0.502190397 | SOCS1 | . |
| 0 | 0 | 1.089806435 | 0.502190397 | CGAS | . |
| 0 | 0 | 1.089806435 | 0.502190397 | CNOT2 | . |
| 0 | 0 | 1.089806435 | 0.502190397 | ITGAM | . |
| 0 | 0 | 1.089806435 | 0.502190397 | E2F3 | . |
| 0 | 0 | 1.089806435 | 0.502190397 | NF1 | . |
| 0 | 0 | 1.089806435 | 0.502190397 | CRY2 | . |
| 0 | 0 | 1.089806435 | 0.502190397 | INPP5K | . |
| 0 | 0 | 1.089806435 | 0.502190397 | LDLR | . |
| 0 | 0 | 1.089806435 | 0.502190397 | NAIF1 | . |
| 0 | 0 | 1.089806435 | 0.502190397 | CXCL10 | . |
| 0 | 0 | 1.089806435 | 0.502190397 | FFAR3 | . |
| 0 | 0 | 1.089806435 | 0.502190397 | ABCA3 | . |

| | | | | | |
|---|---|---|---|---|---|
| 0 | 0 | 1.089806435 | 0.502190397 | DENND3 | . |
| 0 | 0 | 1.089806435 | 0.502190397 | NLGN3 | . |
| 0 | 0 | 1.089806435 | 0.502190397 | CFLAR | . |
| 0 | 0 | 1.089806435 | 0.502190397 | EREG | . |
| 0 | 0 | 1.089806435 | 0.502190397 | FBXO38 | . |
| 0 | 0 | 1.089806435 | 0.502190397 | IL2RA | . |
| 0 | 0 | 1.089806435 | 0.502190397 | RBPJ | . |
| 0 | 0 | 1.089806435 | 0.502190397 | ITGAV | . |
| 0 | 0 | 1.089806435 | 0.502190397 | PRKAA1 | . |
| 0 | 0 | 1.089806435 | 0.502190397 | PICALM | . |
| 0 | 0 | 1.089806435 | 0.502190397 | RYR1 | . |
| 0 | 0 | 1.089806435 | 0.502190397 | RFK | . |
| 0 | 0 | 1.089806435 | 0.502190397 | EIF6 | . |
| 0 | 0 | 1.089806435 | 0.502190397 | SLC38A2 | . |
| 0 | 0 | 1.089806435 | 0.502190397 | GJB6 | . |
| 0 | 0 | 1.089806435 | 0.502190397 | SMAD3 | . |
| 0 | 0 | 1.089806435 | 0.502190397 | LIG4 | . |
| 0 | 0 | 1.089806435 | 0.502190397 | SLC6A2 | . |
| 0 | 0 | 1.089806435 | 0.502190397 | STX1B | . |
| 0 | 0 | 1.089806435 | 0.502190397 | SLC25A19 | . |
| 0 | 0 | 1.089806435 | 0.502190397 | SLC7A5 | . |
| 0 | 0 | 1.089806435 | 0.502190397 | CASP8 | . |
| 0 | 0 | 1.089806435 | 0.502190397 | DLL1 | . |
| 0 | 0 | 1.089806435 | 0.502190397 | VEGFA | . |
| 0 | 0 | 1.089806435 | 0.502190397 | SLC39A8 | . |
| 0 | 0 | 0.933816373 | 0.429518397 | FZD1;PSM/ | . |
| 0 | 0 | 0.934016173 | 0.424842266 | FUT4;ST3G | . |
| 0 | 0 | 0.934016173 | 0.424842266 | MYLK3;SLC | . |
| 0 | 0 | 0.934016173 | 0.424842266 | CD74;GPR1 | . |
| 0 | 0 | 0.934016173 | 0.424842266 | DLC1;INPP | . |
| 0 | 0 | 0.934016173 | 0.424842266 | ITGAM;CHI | . |
| 0 | 0 | 0.934016173 | 0.424842266 | TMEM129; | . |
| 0 | 0 | 0.934016173 | 0.424842266 | P2RY12;DE | . |
| 0 | 0 | 0.917454316 | 0.4021057 | ACVRL1;DI | . |
| 0 | 0 | 0.915185185 | 0.400496049 | GCLC;MTH | . |
| 0 | 0 | 0.915185185 | 0.400496049 | GPR132;PS | . |
| 0 | 0 | 0.915185185 | 0.400496049 | STAT1;HYA | . |
| 0 | 0 | 0.934041063 | 0.407447847 | RAMP2;LA | . |
| 0 | 0 | 0.934041063 | 0.407447847 | GPAT4;VEC | . |
| 0 | 0 | 0.934041063 | 0.407447847 | MORF4L2;I | . |
| 0 | 0 | 0.934041063 | 0.407447847 | TMEM50B; | . |
| 0 | 0 | 0.934041063 | 0.407447847 | KAT2B;CRE | . |
| 0 | 0 | 0.934041063 | 0.407447847 | SLFN11;GT | . |
| 0 | 0 | 0.934041063 | 0.407447847 | LTB4R2;CC | . |
| 0 | 0 | 0.934041063 | 0.407447847 | SERPINB4;I | . |
| 0 | 0 | 0.934041063 | 0.407447847 | PRKCD;ALC | . |
| 0 | 0 | 0.934041063 | 0.407447847 | ATP1A3;PS | . |

| | | | | |
|---|---|---|---|---|
| 0 | 0 | 0.934041063 | 0.407447847 | RGS14;NUI . |
| 0 | 0 | 0.934041063 | 0.407447847 | STRA8;HOI . |
| 0 | 0 | 0.934041063 | 0.407447847 | PRKCE;PPA . |
| 0 | 0 | 0.934041063 | 0.407447847 | ACVR1B;SN . |
| 0 | 0 | 0.934041063 | 0.407447847 | NR4A3;PRI . |
| 0 | 0 | 0.934041063 | 0.407447847 | LYN;CD74 . |
| 0 | 0 | 0.934041063 | 0.407447847 | IKBKB;PSEI . |
| 0 | 0 | 0.934041063 | 0.407447847 | EDN1;ECE1 . |
| 0 | 0 | 0.934041063 | 0.407447847 | VGF;STAT1 . |
| 0 | 0 | 0.934041063 | 0.407447847 | GCLC;SARS . |
| 0 | 0 | 0.934041063 | 0.407447847 | NRP1;NF1 . |
| 0 | 0 | 0.912169108 | 0.397391512 | SNX18;RO( . |
| 0 | 0 | 0.912169108 | 0.397391512 | LYN;DYNC1 . |
| 0 | 0 | 0.912169108 | 0.397391512 | EIF2AK2;FA . |
| 0 | 0 | 0.912169108 | 0.397391512 | TCF7L2;PR . |
| 0 | 0 | 0.912169108 | 0.397391512 | ABCA1;STA . |
| 0 | 0 | 0.907970376 | 0.39198633 | SLC7A5;SL( . |
| 0 | 0 | 0.907970376 | 0.39198633 | WASHC4;R . |
| 0 | 0 | 0.907970376 | 0.39198633 | ACTN2;KCI . |
| 0 | 0 | 0.907970376 | 0.39198633 | IL1B;BHLHI . |
| 0 | 0 | 0.907970376 | 0.39198633 | RYBP;MAX . |
| 0 | 0 | 0.907970376 | 0.39198633 | SPART;LRR . |
| 0 | 0 | 0.901732599 | 0.382804388 | ABCA1;CLN . |
| 0 | 0 | 0.901732599 | 0.382804388 | NLGN3;NR . |
| 0 | 0 | 0.901732599 | 0.382804388 | NIPBL;FMF . |
| 0 | 0 | 0.901732599 | 0.382804388 | FUS;SLC11 . |
| 0 | 0 | 0.910070047 | 0.376037441 | BTG2;RIDA . |
| 0 | 0 | 0.927462881 | 0.382324892 | PHLDB1;XE . |
| 0 | 0 | 0.901583991 | 0.371170882 | CXCL8;RPL . |
| 0 | 0 | 0.913579342 | 0.376105763 | MORC3;PP . |
| 0 | 0 | 0.891509434 | 0.366804143 | PNPLA8;IL1 . |
| 0 | 0 | 0.891509434 | 0.366804143 | PRKAA1;XE . |
| 0 | 0 | 0.891509434 | 0.366804143 | NLGN3;LRI . |
| 0 | 0 | 0.891509434 | 0.366804143 | NR4A3;ADI . |
| 0 | 0 | 0.891509434 | 0.366804143 | EGR2;NF1; . |
| 0 | 0 | 0.891509434 | 0.366804143 | ATXN3;NG . |
| 0 | 0 | 0.891509434 | 0.366804143 | MYLIP;SMI . |
| 0 | 0 | 0.891509434 | 0.366804143 | UBE2B;PDI . |
| 0 | 0 | 0.891509434 | 0.366804143 | TCF7L2;AP . |
| 0 | 0 | 0.891509434 | 0.366804143 | ID2;HOPX;! . |
| 0 | 0 | 0.891386475 | 0.361405182 | DGKG;DGK . |
| 0 | 0 | 0.928105974 | 0.375878512 | PUS10;TUT . |
| 0 | 0 | 0.883379603 | 0.352586851 | LRP5;RFLN . |
| 0 | 0 | 0.883379603 | 0.352586851 | MAD2L2;P. . |
| 0 | 0 | 0.891263238 | 0.346725583 | CUL4A;ISG . |
| 0 | 0 | 0.871624512 | 0.339062655 | NR4A2;DR/ . |
| 0 | 0 | 0.871624512 | 0.339062655 | STRA8;DIA . |

| | | | | | |
|---|---|---|---|---|---|
| 0 | 0 | 0.871624512 | 0.339062655 | PLK3;RB1C | . |
| 0 | 0 | 0.871624512 | 0.339062655 | GCH1;HSD | . |
| 0 | 0 | 0.871624512 | 0.339062655 | CXCL10;P2 | . |
| 0 | 0 | 0.871624512 | 0.339062655 | PIK3CA;AV | . |
| 0 | 0 | 0.871624512 | 0.339062655 | RANBP2;SE | . |
| 0 | 0 | 0.871624512 | 0.339062655 | RANBP2;SE | . |
| 0 | 0 | 0.871624512 | 0.339062655 | NRG1;RBP. | . |
| 0 | 0 | 0.934065934 | 0.360551289 | ADAR | . |
| 0 | 0 | 0.934065934 | 0.360551289 | KIF5C | . |
| 0 | 0 | 0.934065934 | 0.360551289 | B2M | . |
| 0 | 0 | 0.934065934 | 0.360551289 | KIF5C | . |
| 0 | 0 | 0.934065934 | 0.360551289 | PIH1D1 | . |
| 0 | 0 | 0.934065934 | 0.360551289 | METRNL | . |
| 0 | 0 | 0.934065934 | 0.360551289 | ITPK1 | . |
| 0 | 0 | 0.934065934 | 0.360551289 | MDM4 | . |
| 0 | 0 | 0.934065934 | 0.360551289 | HDAC4 | . |
| 0 | 0 | 0.934065934 | 0.360551289 | DCHS1 | . |
| 0 | 0 | 0.934065934 | 0.360551289 | PDE4B | . |
| 0 | 0 | 0.934065934 | 0.360551289 | IFNAR1 | . |
| 0 | 0 | 0.934065934 | 0.360551289 | FYN | . |
| 0 | 0 | 0.934065934 | 0.360551289 | DGAT2 | . |
| 0 | 0 | 0.934065934 | 0.360551289 | ATP7A | . |
| 0 | 0 | 0.934065934 | 0.360551289 | ANKFY1 | . |
| 0 | 0 | 0.934065934 | 0.360551289 | DDIT3 | . |
| 0 | 0 | 0.934065934 | 0.360551289 | SLC9A3R1 | . |
| 0 | 0 | 0.934065934 | 0.360551289 | ZFAND6 | . |
| 0 | 0 | 0.934065934 | 0.360551289 | PDPN | . |
| 0 | 0 | 0.934065934 | 0.360551289 | ATP7A | . |
| 0 | 0 | 0.934065934 | 0.360551289 | ZSWIM6 | . |
| 0 | 0 | 0.934065934 | 0.360551289 | KHK | . |
| 0 | 0 | 0.934065934 | 0.360551289 | GALC | . |
| 0 | 0 | 0.934065934 | 0.360551289 | HAL | . |
| 0 | 0 | 0.934065934 | 0.360551289 | HAL | . |
| 0 | 0 | 0.934065934 | 0.360551289 | PCGF5 | . |
| 0 | 0 | 0.934065934 | 0.360551289 | KDM5A | . |
| 0 | 0 | 0.934065934 | 0.360551289 | LYN | . |
| 0 | 0 | 0.934065934 | 0.360551289 | KAT2B | . |
| 0 | 0 | 0.934065934 | 0.360551289 | ATF4 | . |
| 0 | 0 | 0.934065934 | 0.360551289 | TDRD7 | . |
| 0 | 0 | 0.934065934 | 0.360551289 | LYPLA1 | . |
| 0 | 0 | 0.934065934 | 0.360551289 | CHD1 | . |
| 0 | 0 | 0.934065934 | 0.360551289 | ID2 | . |
| 0 | 0 | 0.934065934 | 0.360551289 | TTC19 | . |
| 0 | 0 | 0.934065934 | 0.360551289 | PNPT1 | . |
| 0 | 0 | 0.934065934 | 0.360551289 | SQSTM1 | . |
| 0 | 0 | 0.934065934 | 0.360551289 | SFPQ | . |
| 0 | 0 | 0.934065934 | 0.360551289 | SFPQ | . |

| | | | | | |
|---|---|---|---|---|---|
| 0 | 0 | 0.934065934 | 0.360551289 | CORO1A | . |
| 0 | 0 | 0.934065934 | 0.360551289 | SOCS1 | . |
| 0 | 0 | 0.934065934 | 0.360551289 | BCL6 | . |
| 0 | 0 | 0.934065934 | 0.360551289 | SLC30A1 | . |
| 0 | 0 | 0.934065934 | 0.360551289 | SIRT1 | . |
| 0 | 0 | 0.934065934 | 0.360551289 | SLAMF8 | . |
| 0 | 0 | 0.934065934 | 0.360551289 | RNF122 | . |
| 0 | 0 | 0.934065934 | 0.360551289 | CCNT1 | . |
| 0 | 0 | 0.934065934 | 0.360551289 | RGS2 | . |
| 0 | 0 | 0.934065934 | 0.360551289 | NF1 | . |
| 0 | 0 | 0.934065934 | 0.360551289 | LDLR | . |
| 0 | 0 | 0.934065934 | 0.360551289 | ACTN2 | . |
| 0 | 0 | 0.934065934 | 0.360551289 | DLL1 | . |
| 0 | 0 | 0.934065934 | 0.360551289 | JMJD6 | . |
| 0 | 0 | 0.934065934 | 0.360551289 | EGLN3 | . |
| 0 | 0 | 0.934065934 | 0.360551289 | SOCS1 | . |
| 0 | 0 | 0.934065934 | 0.360551289 | EZR | . |
| 0 | 0 | 0.934065934 | 0.360551289 | JAML | . |
| 0 | 0 | 0.934065934 | 0.360551289 | PAK1 | . |
| 0 | 0 | 0.934065934 | 0.360551289 | LGALS9 | . |
| 0 | 0 | 0.934065934 | 0.360551289 | STRA8 | . |
| 0 | 0 | 0.934065934 | 0.360551289 | ATP7A | . |
| 0 | 0 | 0.934065934 | 0.360551289 | DVL2 | . |
| 0 | 0 | 0.934065934 | 0.360551289 | CALM1 | . |
| 0 | 0 | 0.934065934 | 0.360551289 | ATP7A | . |
| 0 | 0 | 0.934065934 | 0.360551289 | DAGLB | . |
| 0 | 0 | 0.934065934 | 0.360551289 | NRXN2 | . |
| 0 | 0 | 0.934065934 | 0.360551289 | SRC | . |
| 0 | 0 | 0.934065934 | 0.360551289 | FOLH1B | . |
| 0 | 0 | 0.934065934 | 0.360551289 | NGLY1 | . |
| 0 | 0 | 0.934065934 | 0.360551289 | LYPLA1 | . |
| 0 | 0 | 0.934065934 | 0.360551289 | KIF5C | . |
| 0 | 0 | 0.934065934 | 0.360551289 | PDP1 | . |
| 0 | 0 | 0.934065934 | 0.360551289 | ARHGAP35 | . |
| 0 | 0 | 0.934065934 | 0.360551289 | PDP1 | . |
| 0 | 0 | 0.934065934 | 0.360551289 | PLK3 | . |
| 0 | 0 | 0.934065934 | 0.360551289 | CALM1 | . |
| 0 | 0 | 0.934065934 | 0.360551289 | DPH3 | . |
| 0 | 0 | 0.934065934 | 0.360551289 | LRRK2 | . |
| 0 | 0 | 0.934065934 | 0.360551289 | TGFBR1 | . |
| 0 | 0 | 0.934065934 | 0.360551289 | DNM2 | . |
| 0 | 0 | 0.934065934 | 0.360551289 | CLN3 | . |
| 0 | 0 | 0.934065934 | 0.360551289 | IRF7 | . |
| 0 | 0 | 0.934065934 | 0.360551289 | ANXA1 | . |
| 0 | 0 | 0.934065934 | 0.360551289 | ATP1B3 | . |
| 0 | 0 | 0.934065934 | 0.360551289 | CHP1 | . |
| 0 | 0 | 0.934065934 | 0.360551289 | EPB41L2 | . |

| | | | | | |
|---|---|---|---|---|---|
| 0 | 0 | 0.934065934 | 0.360551289 | VCPIP1 | . |
| 0 | 0 | 0.934065934 | 0.360551289 | GLUL | . |
| 0 | 0 | 0.934065934 | 0.360551289 | NIBAN2 | . |
| 0 | 0 | 0.934065934 | 0.360551289 | EVI2B | . |
| 0 | 0 | 0.934065934 | 0.360551289 | SOX15 | . |
| 0 | 0 | 0.934065934 | 0.360551289 | TTC19 | . |
| 0 | 0 | 0.934065934 | 0.360551289 | PDE4B | . |
| 0 | 0 | 0.934065934 | 0.360551289 | DENR | . |
| 0 | 0 | 0.934065934 | 0.360551289 | CASP8 | . |
| 0 | 0 | 0.934065934 | 0.360551289 | CFLAR | . |
| 0 | 0 | 0.934065934 | 0.360551289 | SAT1 | . |
| 0 | 0 | 0.934065934 | 0.360551289 | GGT1 | . |
| 0 | 0 | 0.934065934 | 0.360551289 | CTSH | . |
| 0 | 0 | 0.934065934 | 0.360551289 | TG | . |
| 0 | 0 | 0.934065934 | 0.360551289 | SMG1 | . |
| 0 | 0 | 0.934065934 | 0.360551289 | ACVRL1 | . |
| 0 | 0 | 0.934065934 | 0.360551289 | CYP4F2 | . |
| 0 | 0 | 0.934065934 | 0.360551289 | CLN3 | . |
| 0 | 0 | 0.871721363 | 0.336462154 | ABCA7;PSE | . |
| 0 | 0 | 0.871721363 | 0.336462154 | ACVRL1;RE | . |
| 0 | 0 | 0.871721363 | 0.336462154 | CDKN2D;SI | . |
| 0 | 0 | 0.871721363 | 0.336462154 | ACVRL1;M | . |
| 0 | 0 | 0.871721363 | 0.336462154 | ROCK1;AB( | . |
| 0 | 0 | 0.871721363 | 0.336462154 | HES1;TGFB | . |
| 0 | 0 | 0.871721363 | 0.336462154 | SLC37A1;S | . |
| 0 | 0 | 0.871721363 | 0.336462154 | RGCC;VSIR | . |
| 0 | 0 | 0.871721363 | 0.336462154 | RAB3D;RAI | . |
| 0 | 0 | 0.871721363 | 0.336462154 | PTPRC;N4E | . |
| 0 | 0 | 0.871721363 | 0.336462154 | NABP2;PRI | . |
| 0 | 0 | 0.871721363 | 0.336462154 | CALM1;SL( | . |
| 0 | 0 | 0.871721363 | 0.336462154 | PRKAA1;AE | . |
| 0 | 0 | 0.871721363 | 0.336462154 | IL16;SRI | . |
| 0 | 0 | 0.871721363 | 0.336462154 | PTPRC;EIF2 | . |
| 0 | 0 | 0.871721363 | 0.336462154 | AGO2;NRD | . |
| 0 | 0 | 0.914734043 | 0.350145223 | LYN;BTG2; | . |
| 0 | 0 | 0.879884005 | 0.336789703 | DYNC1H1;/ | . |
| 0 | 0 | 0.933030585 | 0.352214358 | STXBP3;PP | . |
| 0 | 0 | 0.896915795 | 0.334118761 | SLC24A1;T | . |
| 0 | 0 | 0.852698934 | 0.317349194 | NIPBL;NF1 | . |
| 0 | 0 | 0.852698934 | 0.317349194 | ZFYVE28;SI | . |
| 0 | 0 | 0.852698934 | 0.317349194 | XRN1;SRC; | . |
| 0 | 0 | 0.852698934 | 0.317349194 | MOV10;TU | . |
| 0 | 0 | 0.852698934 | 0.317349194 | PAK1;CLIP1 | . |
| 0 | 0 | 0.852698934 | 0.317349194 | PPP1R3B;P | . |
| 0 | 0 | 0.852698934 | 0.317349194 | KCNS1;ACT | . |
| 0 | 0 | 0.852698934 | 0.317349194 | CLN3;GRIP | . |
| 0 | 0 | 0.852698934 | 0.317349194 | SMG1;PPP | . |

| | | | | |
|---|---|---|---|---|
| 0 | 0 | 0.906145908 | 0.335085793 | HEXIM1;PL |
| 0 | 0 | 0.860083082 | 0.317254907 | SARS1;PPA |
| 0 | 0 | 0.877909996 | 0.321640393 | MEF2A;KLF |
| 0 | 0 | 0.877909996 | 0.321640393 | ENPP2;APC |
| 0 | 0 | 0.920423341 | 0.336376704 | DDX5;DHX |
| 0 | 0 | 0.910671736 | 0.332514534 | KLHL18;DY |
| 0 | 0 | 0.863232505 | 0.308817064 | P2RY12;GN |
| 0 | 0 | 0.863232505 | 0.308817064 | LTV1;CUL4 |
| 0 | 0 | 0.863232505 | 0.308817064 | WTAP;MET |
| 0 | 0 | 0.843458883 | 0.300561606 | EDN1;GCLC |
| 0 | 0 | 0.843458883 | 0.300561606 | ACVRL1;DU |
| 0 | 0 | 0.843458883 | 0.300561606 | KCNJ12;KC |
| 0 | 0 | 0.843458883 | 0.300561606 | LRRK2;RAB |
| 0 | 0 | 0.852531989 | 0.298988559 | ARG2;ASAI |
| 0 | 0 | 0.852531989 | 0.298988559 | WNT2B;HY |
| 0 | 0 | 0.864949497 | 0.298310889 | KLF7;PARP |
| 0 | 0 | 0.817191626 | 0.279319743 | H2AW;NFE |
| 0 | 0 | 0.817191626 | 0.279319743 | HHEX;TOP |
| 0 | 0 | 0.817191626 | 0.279319743 | CREBBP;EC |
| 0 | 0 | 0.817191626 | 0.279319743 | TLR9;NOD |
| 0 | 0 | 0.817191626 | 0.279319743 | GRK2;RGS |
| 0 | 0 | 0.817191626 | 0.279319743 | EDEM1;NG |
| 0 | 0 | 0.817191626 | 0.279319743 | APOA2;AB |
| 0 | 0 | 0.817191626 | 0.279319743 | BFSP2;DNA |
| 0 | 0 | 0.817191626 | 0.279319743 | MREG;RAB |
| 0 | 0 | 0.817191626 | 0.279319743 | CIITA;IL6 |
| 0 | 0 | 0.817191626 | 0.279319743 | PPIF;CBARI |
| 0 | 0 | 0.817191626 | 0.279319743 | MAD2L2;C |
| 0 | 0 | 0.817191626 | 0.279319743 | H2AW;NFE |
| 0 | 0 | 0.817191626 | 0.279319743 | PRPSAP1;P |
| 0 | 0 | 0.817191626 | 0.279319743 | DNAH17;ZI |
| 0 | 0 | 0.817191626 | 0.279319743 | MREG;RAB |
| 0 | 0 | 0.817191626 | 0.279319743 | ACVRL1;TA |
| 0 | 0 | 0.817191626 | 0.279319743 | IFNG;LIF |
| 0 | 0 | 0.817191626 | 0.279319743 | CYP2S1;GS |
| 0 | 0 | 0.817191626 | 0.279319743 | ZNF207;HC |
| 0 | 0 | 0.817191626 | 0.279319743 | LRRK2;SMA |
| 0 | 0 | 0.817191626 | 0.279319743 | P2RY12;SA |
| 0 | 0 | 0.817191626 | 0.279319743 | PDPN;CFTF |
| 0 | 0 | 0.837981253 | 0.285544647 | NRP1;PAK |
| 0 | 0 | 0.837981253 | 0.285544647 | CRTC2;CHS |
| 0 | 0 | 0.837981253 | 0.285544647 | CYP27B1;C |
| 0 | 0 | 0.817122642 | 0.275063996 | CYP4F2;LPI |
| 0 | 0 | 0.817122642 | 0.275063996 | DLC1;INPP |
| 0 | 0 | 0.817122642 | 0.275063996 | NLGN3;SYN |
| 0 | 0 | 0.817122642 | 0.275063996 | HEXIM1;EL |
| 0 | 0 | 0.866010598 | 0.287436281 | H2AW;NFE |

| | | | | | |
|---|---|---|---|---|---|
| 0 | 0 | 0.817053605 | 0.266621455 | SELP;SELL;( | . |
| 0 | 0 | 0.817053605 | 0.266621455 | ATXN3;IL1/ | . |
| 0 | 0 | 0.817053605 | 0.266621455 | RARG;NIPE | . |
| 0 | 0 | 0.817053605 | 0.266621455 | TMEM119; | . |
| 0 | 0 | 0.817053605 | 0.266621455 | PHLDB1;NI | . |
| 0 | 0 | 0.817053605 | 0.266621455 | CYLD;PHLD | . |
| 0 | 0 | 0.817053605 | 0.266621455 | LYZ;B2M;A | . |
| 0 | 0 | 0.817260558 | 0.265906899 | SLC9A3R1 | . |
| 0 | 0 | 0.817260558 | 0.265906899 | CLN3 | . |
| 0 | 0 | 0.817260558 | 0.265906899 | SCARF1 | . |
| 0 | 0 | 0.817260558 | 0.265906899 | APOA2 | . |
| 0 | 0 | 0.817260558 | 0.265906899 | CFAP54 | . |
| 0 | 0 | 0.817260558 | 0.265906899 | DDR2 | . |
| 0 | 0 | 0.817260558 | 0.265906899 | CDADC1 | . |
| 0 | 0 | 0.817260558 | 0.265906899 | TOP1 | . |
| 0 | 0 | 0.817260558 | 0.265906899 | NIPBL | . |
| 0 | 0 | 0.817260558 | 0.265906899 | TMEM119 | . |
| 0 | 0 | 0.817260558 | 0.265906899 | HSD17B1 | . |
| 0 | 0 | 0.817260558 | 0.265906899 | FUT4 | . |
| 0 | 0 | 0.817260558 | 0.265906899 | ALOX12 | . |
| 0 | 0 | 0.817260558 | 0.265906899 | ALOX12 | . |
| 0 | 0 | 0.817260558 | 0.265906899 | PRKCD | . |
| 0 | 0 | 0.817260558 | 0.265906899 | SLC7A5 | . |
| 0 | 0 | 0.817260558 | 0.265906899 | SLCO3A1 | . |
| 0 | 0 | 0.817260558 | 0.265906899 | SLC4A7 | . |
| 0 | 0 | 0.817260558 | 0.265906899 | LDLR | . |
| 0 | 0 | 0.817260558 | 0.265906899 | HSD17B1 | . |
| 0 | 0 | 0.817260558 | 0.265906899 | POGZ | . |
| 0 | 0 | 0.817260558 | 0.265906899 | FUT4 | . |
| 0 | 0 | 0.817260558 | 0.265906899 | FUT4 | . |
| 0 | 0 | 0.817260558 | 0.265906899 | ASRGL1 | . |
| 0 | 0 | 0.817260558 | 0.265906899 | CLN3 | . |
| 0 | 0 | 0.817260558 | 0.265906899 | SLC9A3R1 | . |
| 0 | 0 | 0.817260558 | 0.265906899 | SLC9A3R1 | . |
| 0 | 0 | 0.817260558 | 0.265906899 | KDM6B | . |
| 0 | 0 | 0.817260558 | 0.265906899 | RPUSD4 | . |
| 0 | 0 | 0.817260558 | 0.265906899 | DCHS1 | . |
| 0 | 0 | 0.817260558 | 0.265906899 | ROCK1 | . |
| 0 | 0 | 0.817260558 | 0.265906899 | SERPINE1 | . |
| 0 | 0 | 0.817260558 | 0.265906899 | RNF122 | . |
| 0 | 0 | 0.817260558 | 0.265906899 | CCNT1 | . |
| 0 | 0 | 0.817260558 | 0.265906899 | EREG | . |
| 0 | 0 | 0.817260558 | 0.265906899 | CMPK2 | . |
| 0 | 0 | 0.817260558 | 0.265906899 | CMPK2 | . |
| 0 | 0 | 0.817260558 | 0.265906899 | CMPK2 | . |
| 0 | 0 | 0.817260558 | 0.265906899 | NEU1 | . |
| 0 | 0 | 0.817260558 | 0.265906899 | ZFAND6 | . |

| | | | | | |
|---|---|---|---|---|---|
| 0 | 0 | 0.817260558 | 0.265906899 | SEC16B | . |
| 0 | 0 | 0.817260558 | 0.265906899 | GTF2H1 | . |
| 0 | 0 | 0.817260558 | 0.265906899 | RAB3GAP1 | . |
| 0 | 0 | 0.817260558 | 0.265906899 | FBXO7 | . |
| 0 | 0 | 0.817260558 | 0.265906899 | ID2 | . |
| 0 | 0 | 0.817260558 | 0.265906899 | PTPN22 | . |
| 0 | 0 | 0.817260558 | 0.265906899 | APOA2 | . |
| 0 | 0 | 0.817260558 | 0.265906899 | TESC | . |
| 0 | 0 | 0.817260558 | 0.265906899 | GLRX | . |
| 0 | 0 | 0.817260558 | 0.265906899 | PROK2 | . |
| 0 | 0 | 0.817260558 | 0.265906899 | LRRC8C | . |
| 0 | 0 | 0.817260558 | 0.265906899 | UBE2S | . |
| 0 | 0 | 0.817260558 | 0.265906899 | GCH1 | . |
| 0 | 0 | 0.817260558 | 0.265906899 | LRRK2 | . |
| 0 | 0 | 0.817260558 | 0.265906899 | IL1B | . |
| 0 | 0 | 0.817260558 | 0.265906899 | SIRT1 | . |
| 0 | 0 | 0.817260558 | 0.265906899 | PIH1D1 | . |
| 0 | 0 | 0.817260558 | 0.265906899 | LYN | . |
| 0 | 0 | 0.817260558 | 0.265906899 | LIF | . |
| 0 | 0 | 0.817260558 | 0.265906899 | NUGGC | . |
| 0 | 0 | 0.817260558 | 0.265906899 | SIRT1 | . |
| 0 | 0 | 0.817260558 | 0.265906899 | PLEKHA4 | . |
| 0 | 0 | 0.817260558 | 0.265906899 | RYR1 | . |
| 0 | 0 | 0.817260558 | 0.265906899 | SCARF1 | . |
| 0 | 0 | 0.817260558 | 0.265906899 | NRP1 | . |
| 0 | 0 | 0.817260558 | 0.265906899 | DLL1 | . |
| 0 | 0 | 0.817260558 | 0.265906899 | ALOX12 | . |
| 0 | 0 | 0.881437122 | 0.285951137 | GGT5;SLC1 | . |
| 0 | 0 | 0.881437122 | 0.285951137 | LYN;TENM | . |
| 0 | 0 | 0.816984517 | 0.257064067 | CLN3;SCN | . |
| 0 | 0 | 0.816984517 | 0.257064067 | SLC6A6;SL | . |
| 0 | 0 | 0.816984517 | 0.257064067 | CDKN1A;F | . |
| 0 | 0 | 0.855651382 | 0.268597175 | ZNF473;ZF | . |
| 0 | 0 | 0.831746032 | 0.259575224 | CRTC2;AD | . |
| 0 | 0 | 0.882715226 | 0.275165786 | SMAD3;SR | . |
| 0 | 0 | 0.867209672 | 0.266515604 | LYN;BTG2; | . |
| 0 | 0 | 0.784392453 | 0.238798964 | NOS2;FFA | . |
| 0 | 0 | 0.784392453 | 0.238798964 | PAK1;CLIP | . |
| 0 | 0 | 0.784392453 | 0.238798964 | FCN1;SMU | . |
| 0 | 0 | 0.784392453 | 0.238798964 | MAP1B;SK | . |
| 0 | 0 | 0.784392453 | 0.238798964 | SIPA1L1;L | . |
| 0 | 0 | 0.784392453 | 0.238798964 | HES1;TGF | . |
| 0 | 0 | 0.769077152 | 0.232946415 | SDS;SDSL | . |
| 0 | 0 | 0.769077152 | 0.232946415 | FOLH1B;A | . |
| 0 | 0 | 0.769077152 | 0.232946415 | VPS9D1;A | . |
| 0 | 0 | 0.769077152 | 0.232946415 | NGLY1;AO | . |
| 0 | 0 | 0.769077152 | 0.232946415 | DAXX;MT2 | . |

| | | | | | |
|---|---|---|---|---|---|
| 0 | 0 | 0.769077152 | 0.232946415 | PKD1L3;PT | . |
| 0 | 0 | 0.769077152 | 0.232946415 | ACVRL1;M | . |
| 0 | 0 | 0.769077152 | 0.232946415 | UGGT1;MA | . |
| 0 | 0 | 0.769077152 | 0.232946415 | MREG;RAB | . |
| 0 | 0 | 0.769077152 | 0.232946415 | LRRK2;RAB | . |
| 0 | 0 | 0.769077152 | 0.232946415 | PLA2G4A;C | . |
| 0 | 0 | 0.769077152 | 0.232946415 | NRG1;TAP | . |
| 0 | 0 | 0.769077152 | 0.232946415 | MAN1A2;G | . |
| 0 | 0 | 0.769077152 | 0.232946415 | LYN;FCGR2 | . |
| 0 | 0 | 0.769077152 | 0.232946415 | NRG1;TAP | . |
| 0 | 0 | 0.769077152 | 0.232946415 | GSDMD;NU | . |
| 0 | 0 | 0.769077152 | 0.232946415 | USP36;FBX | . |
| 0 | 0 | 0.769077152 | 0.232946415 | KAT2B;CEP | . |
| 0 | 0 | 0.769077152 | 0.232946415 | PIH1D1;BC | . |
| 0 | 0 | 0.769077152 | 0.232946415 | IFNG;LIF | . |
| 0 | 0 | 0.769077152 | 0.232946415 | CNOT2;TAI | . |
| 0 | 0 | 0.769077152 | 0.232946415 | FFAR3;FFA | . |
| 0 | 0 | 0.769077152 | 0.232946415 | ACTN4;MY | . |
| 0 | 0 | 0.792248647 | 0.236660773 | DDX17;TU | . |
| 0 | 0 | 0.792248647 | 0.236660773 | CLN3;MBO | . |
| 0 | 0 | 0.792248647 | 0.236660773 | DDX5;SAFE | . |
| 0 | 0 | 0.792248647 | 0.236660773 | RANBP2;SE | . |
| 0 | 0 | 0.816846183 | 0.237938857 | ANKRD28;S | . |
| 0 | 0 | 0.816846183 | 0.237938857 | SERPINB10 | . |
| 0 | 0 | 0.816846183 | 0.237938857 | ANKRD28;S | . |
| 0 | 0 | 0.816846183 | 0.237938857 | ANKRD28;S | . |
| 0 | 0 | 0.797012011 | 0.231467666 | PARP10;UE | . |
| 0 | 0 | 0.797012011 | 0.231467666 | RBM3;RPL | . |
| 0 | 0 | 0.797012011 | 0.231467666 | MAD2L2;P | . |
| 0 | 0 | 0.797012011 | 0.231467666 | PARM1;SR | . |
| 0 | 0 | 0.828258507 | 0.237682916 | CLN3;SYNJ | . |
| 0 | 0 | 0.828258507 | 0.237682916 | CLN3;CLIC4 | . |
| 0 | 0 | 0.894013493 | 0.255714041 | DDX5;DHX | . |
| 0 | 0 | 0.800197374 | 0.225013857 | ASAH1;SPT | . |
| 0 | 0 | 0.852028876 | 0.237378077 | PLK3;DYNC | . |
| 0 | 0 | 0.852028876 | 0.237378077 | PLK3;SEC1 | . |
| 0 | 0 | 0.827023377 | 0.227921694 | SYS1;DOP1 | . |
| 0 | 0 | 0.726411932 | 0.200193267 | SLC6A6 | . |
| 0 | 0 | 0.726411932 | 0.200193267 | ACTB | . |
| 0 | 0 | 0.726411932 | 0.200193267 | STAT1 | . |
| 0 | 0 | 0.726411932 | 0.200193267 | FMR1 | . |
| 0 | 0 | 0.726411932 | 0.200193267 | SESN2 | . |
| 0 | 0 | 0.726411932 | 0.200193267 | APOA2 | . |
| 0 | 0 | 0.726411932 | 0.200193267 | KAT5 | . |
| 0 | 0 | 0.726411932 | 0.200193267 | ALKBH1 | . |
| 0 | 0 | 0.726411932 | 0.200193267 | ALKBH1 | . |
| 0 | 0 | 0.726411932 | 0.200193267 | LRIF1 | . |

| | | | | | |
|---|---|---|---|---|---|
| 0 | 0 | 0.726411932 | 0.200193267 | PAF1 | . |
| 0 | 0 | 0.726411932 | 0.200193267 | NIPBL | . |
| 0 | 0 | 0.726411932 | 0.200193267 | SLC6A9 | . |
| 0 | 0 | 0.726411932 | 0.200193267 | NEU1 | . |
| 0 | 0 | 0.726411932 | 0.200193267 | BATF | . |
| 0 | 0 | 0.726411932 | 0.200193267 | HDAC4 | . |
| 0 | 0 | 0.726411932 | 0.200193267 | SLC2A1 | . |
| 0 | 0 | 0.726411932 | 0.200193267 | MYLIP | . |
| 0 | 0 | 0.726411932 | 0.200193267 | MYLIP | . |
| 0 | 0 | 0.726411932 | 0.200193267 | STRA6 | . |
| 0 | 0 | 0.726411932 | 0.200193267 | ROCK1 | . |
| 0 | 0 | 0.726411932 | 0.200193267 | CXCR4 | . |
| 0 | 0 | 0.726411932 | 0.200193267 | NDFIP2 | . |
| 0 | 0 | 0.726411932 | 0.200193267 | TRIB1 | . |
| 0 | 0 | 0.726411932 | 0.200193267 | SIRT1 | . |
| 0 | 0 | 0.726411932 | 0.200193267 | SLC9A3R1 | . |
| 0 | 0 | 0.726411932 | 0.200193267 | JUN | . |
| 0 | 0 | 0.726411932 | 0.200193267 | ARHGAP35 | . |
| 0 | 0 | 0.726411932 | 0.200193267 | NUP98 | . |
| 0 | 0 | 0.726411932 | 0.200193267 | SIDT2 | . |
| 0 | 0 | 0.726411932 | 0.200193267 | NT5C3A | . |
| 0 | 0 | 0.726411932 | 0.200193267 | CYP4F2 | . |
| 0 | 0 | 0.726411932 | 0.200193267 | PLA2G5 | . |
| 0 | 0 | 0.726411932 | 0.200193267 | PAF1 | . |
| 0 | 0 | 0.726411932 | 0.200193267 | PIH1D1 | . |
| 0 | 0 | 0.726411932 | 0.200193267 | RIN2 | . |
| 0 | 0 | 0.726411932 | 0.200193267 | AGO2 | . |
| 0 | 0 | 0.726411932 | 0.200193267 | LMTK2 | . |
| 0 | 0 | 0.726411932 | 0.200193267 | SIRT1 | . |
| 0 | 0 | 0.726411932 | 0.200193267 | CXCL13 | . |
| 0 | 0 | 0.726411932 | 0.200193267 | CCDC65 | . |
| 0 | 0 | 0.726411932 | 0.200193267 | DDX39B | . |
| 0 | 0 | 0.726411932 | 0.200193267 | ANXA2 | . |
| 0 | 0 | 0.726411932 | 0.200193267 | PAK1 | . |
| 0 | 0 | 0.726411932 | 0.200193267 | ATF7IP | . |
| 0 | 0 | 0.726411932 | 0.200193267 | GTF2B | . |
| 0 | 0 | 0.726411932 | 0.200193267 | SDS | . |
| 0 | 0 | 0.726411932 | 0.200193267 | PIH1D1 | . |
| 0 | 0 | 0.726411932 | 0.200193267 | STXBP1 | . |
| 0 | 0 | 0.726411932 | 0.200193267 | TFIP11 | . |
| 0 | 0 | 0.726411932 | 0.200193267 | FZD1 | . |
| 0 | 0 | 0.754179971 | 0.207612443 | NDE1;NUS. | . |
| 0 | 0 | 0.754179971 | 0.207612443 | SYCP1;HOF | . |
| 0 | 0 | 0.754179971 | 0.207612443 | PRDX5;MA | . |
| 0 | 0 | 0.754179971 | 0.207612443 | KAT2B;KRE | . |
| 0 | 0 | 0.754179971 | 0.207612443 | HSD11B1;L | . |
| 0 | 0 | 0.754179971 | 0.207612443 | EDN1;SERF | . |

| | | | | |
|---|---|---|---|---|
| 0 | 0 | 0.754179971 | 0.207612443 | FZR1;UBE2 |
| 0 | 0 | 0.754179971 | 0.207612443 | ATAD1;ZFA |
| 0 | 0 | 0.754179971 | 0.207612443 | COTL1;HCL |
| 0 | 0 | 0.754179971 | 0.207612443 | CLN3;ZDHH |
| 0 | 0 | 0.768902805 | 0.210180376 | RANBP2;SE |
| 0 | 0 | 0.768902805 | 0.210180376 | RYBP;MAX |
| 0 | 0 | 0.72630873 | 0.195047248 | SLC9A3R1; |
| 0 | 0 | 0.72630873 | 0.195047248 | MTHFD2;M |
| 0 | 0 | 0.72630873 | 0.195047248 | NR4A3;LRF |
| 0 | 0 | 0.72630873 | 0.195047248 | ANXA1;FAI |
| 0 | 0 | 0.72630873 | 0.195047248 | KDM6B;SN |
| 0 | 0 | 0.72630873 | 0.195047248 | NDE1;NDE |
| 0 | 0 | 0.72630873 | 0.195047248 | COX17;CO |
| 0 | 0 | 0.72630873 | 0.195047248 | NIBAN2;B4 |
| 0 | 0 | 0.72630873 | 0.195047248 | VPS4B;STC |
| 0 | 0 | 0.72630873 | 0.195047248 | EDN1;RGS |
| 0 | 0 | 0.72630873 | 0.195047248 | PRKAA1;IL |
| 0 | 0 | 0.72630873 | 0.195047248 | AGO2;SNIF |
| 0 | 0 | 0.72630873 | 0.195047248 | H2AW;NFE |
| 0 | 0 | 0.72630873 | 0.195047248 | PRKD2;SPF |
| 0 | 0 | 0.72630873 | 0.195047248 | LRRK2;AKA |
| 0 | 0 | 0.890141383 | 0.236761702 | DDX5;DHX |
| 0 | 0 | 0.825141992 | 0.210303809 | MEF2A;RYI |
| 0 | 0 | 0.788587849 | 0.199835392 | HSD11B1;H |
| 0 | 0 | 0.788587849 | 0.199835392 | PUS10;ME |
| 0 | 0 | 0.74689101 | 0.186750188 | CAMK1D;S |
| 0 | 0 | 0.726205451 | 0.180728103 | NDE1;LLGL |
| 0 | 0 | 0.726205451 | 0.180728103 | SLC4A7;SL |
| 0 | 0 | 0.726205451 | 0.180728103 | EPHX2;ALC |
| 0 | 0 | 0.726205451 | 0.180728103 | VASP;DLC1 |
| 0 | 0 | 0.726205451 | 0.180728103 | ADCY8;ZDH |
| 0 | 0 | 0.791934468 | 0.194508572 | UBE2S;NDI |
| 0 | 0 | 0.841078655 | 0.206314031 | DYNC1H1; |
| 0 | 0 | 0.688042248 | 0.163883444 | GRK2;RGS |
| 0 | 0 | 0.688042248 | 0.163883444 | DDIT3;WLS |
| 0 | 0 | 0.688042248 | 0.163883444 | NRP1;NF1 |
| 0 | 0 | 0.688042248 | 0.163883444 | KAT5;NRIP |
| 0 | 0 | 0.688042248 | 0.163883444 | CBLIF;TCN |
| 0 | 0 | 0.688042248 | 0.163883444 | ACVRL1;M |
| 0 | 0 | 0.688042248 | 0.163883444 | XYLT1;HPS |
| 0 | 0 | 0.688042248 | 0.163883444 | PRDX5;GP |
| 0 | 0 | 0.688042248 | 0.163883444 | MREG;RAB |
| 0 | 0 | 0.688042248 | 0.163883444 | CORO1A;C |
| 0 | 0 | 0.688042248 | 0.163883444 | CUL4A;GTF |
| 0 | 0 | 0.688042248 | 0.163883444 | CUL4A;GTF |
| 0 | 0 | 0.688042248 | 0.163883444 | PDCL;GLI1 |
| 0 | 0 | 0.688042248 | 0.163883444 | NIPBL;PIH1 |

| | | | | | |
|---|---|---|---|---|---|
| 0 | 0 | 0.688042248 | 0.163883444 | CALM1;SL( | . |
| 0 | 0 | 0.688042248 | 0.163883444 | NR1H2;NR | . |
| 0 | 0 | 0.794542462 | 0.188965315 | NPEPPS;DI | . |
| 0 | 0 | 0.807347175 | 0.190607423 | NIBAN1;RE | . |
| 0 | 0 | 0.653733032 | 0.153197431 | GSN | . |
| 0 | 0 | 0.653733032 | 0.153197431 | ACTN1 | . |
| 0 | 0 | 0.653733032 | 0.153197431 | SCARF1 | . |
| 0 | 0 | 0.653733032 | 0.153197431 | HIF1A | . |
| 0 | 0 | 0.653733032 | 0.153197431 | NFAT5 | . |
| 0 | 0 | 0.653733032 | 0.153197431 | MMP1 | . |
| 0 | 0 | 0.653733032 | 0.153197431 | B4GALT5 | . |
| 0 | 0 | 0.653733032 | 0.153197431 | SLC25A16 | . |
| 0 | 0 | 0.653733032 | 0.153197431 | GNAT2 | . |
| 0 | 0 | 0.653733032 | 0.153197431 | GNAT2 | . |
| 0 | 0 | 0.653733032 | 0.153197431 | UGGT1 | . |
| 0 | 0 | 0.653733032 | 0.153197431 | ALAS1 | . |
| 0 | 0 | 0.653733032 | 0.153197431 | RAPGEF6 | . |
| 0 | 0 | 0.653733032 | 0.153197431 | HSD11B1 | . |
| 0 | 0 | 0.653733032 | 0.153197431 | HSD11B1 | . |
| 0 | 0 | 0.653733032 | 0.153197431 | IPMK | . |
| 0 | 0 | 0.653733032 | 0.153197431 | LYPLA1 | . |
| 0 | 0 | 0.653733032 | 0.153197431 | STAT1 | . |
| 0 | 0 | 0.653733032 | 0.153197431 | TFB1M | . |
| 0 | 0 | 0.653733032 | 0.153197431 | PNPT1 | . |
| 0 | 0 | 0.653733032 | 0.153197431 | RYR1 | . |
| 0 | 0 | 0.653733032 | 0.153197431 | CBARP | . |
| 0 | 0 | 0.653733032 | 0.153197431 | KAT2B | . |
| 0 | 0 | 0.653733032 | 0.153197431 | CNOT2 | . |
| 0 | 0 | 0.653733032 | 0.153197431 | LGALS9 | . |
| 0 | 0 | 0.653733032 | 0.153197431 | ACTN2 | . |
| 0 | 0 | 0.653733032 | 0.153197431 | LRRK2 | . |
| 0 | 0 | 0.653733032 | 0.153197431 | EGLN3 | . |
| 0 | 0 | 0.653733032 | 0.153197431 | GSN | . |
| 0 | 0 | 0.653733032 | 0.153197431 | ACVRL1 | . |
| 0 | 0 | 0.653733032 | 0.153197431 | EZR | . |
| 0 | 0 | 0.653733032 | 0.153197431 | VEGFA | . |
| 0 | 0 | 0.653733032 | 0.153197431 | PLEKHA4 | . |
| 0 | 0 | 0.653733032 | 0.153197431 | CHMP2A | . |
| 0 | 0 | 0.653733032 | 0.153197431 | WDR45 | . |
| 0 | 0 | 0.653733032 | 0.153197431 | TTLL7 | . |
| 0 | 0 | 0.653733032 | 0.153197431 | SLC25A16 | . |
| 0 | 0 | 0.653733032 | 0.153197431 | MID1IP1 | . |
| 0 | 0 | 0.653733032 | 0.153197431 | ACTN2 | . |
| 0 | 0 | 0.653733032 | 0.153197431 | PIH1D1 | . |
| 0 | 0 | 0.653733032 | 0.153197431 | ATP7A | . |
| 0 | 0 | 0.653733032 | 0.153197431 | PAF1 | . |
| 0 | 0 | 0.653733032 | 0.153197431 | LYN | . |

| | | | | | |
|---|---|---|---|---|---|
| 0 | 0 | 0.653733032 | 0.153197431 | FMR1 | . |
| 0 | 0 | 0.653733032 | 0.153197431 | PTGER2 | . |
| 0 | 0 | 0.653733032 | 0.153197431 | SAG | . |
| 0 | 0 | 0.653733032 | 0.153197431 | SLC25A16 | . |
| 0 | 0 | 0.653733032 | 0.153197431 | HM13 | . |
| 0 | 0 | 0.653733032 | 0.153197431 | EGR2 | . |
| 0 | 0 | 0.653733032 | 0.153197431 | SMPDL3A | . |
| 0 | 0 | 0.653733032 | 0.153197431 | NSMCE2 | . |
| 0 | 0 | 0.653733032 | 0.153197431 | UAP1 | . |
| 0 | 0 | 0.653733032 | 0.153197431 | LIG4 | . |
| 0 | 0 | 0.796628 | 0.183369785 | ACVRL1;FL | . |
| 0 | 0 | 0.726102093 | 0.165998467 | ADAM15;P | . |
| 0 | 0 | 0.726102093 | 0.165998467 | NCOA1;SL | . |
| 0 | 0 | 0.726102093 | 0.165998467 | TMEM119; | . |
| 0 | 0 | 0.726102093 | 0.165998467 | DNMT3L;P | . |
| 0 | 0 | 0.80803507 | 0.184238846 | BCR;NLGN | . |
| 0 | 0 | 0.742541541 | 0.169101944 | NEU1;ST6 | . |
| 0 | 0 | 0.700229111 | 0.157502133 | IL18;RICTO | . |
| 0 | 0 | 0.700229111 | 0.157502133 | ATP1A1;SC | . |
| 0 | 0 | 0.700229111 | 0.157502133 | SEMA4D;LI | . |
| 0 | 0 | 0.700229111 | 0.157502133 | CLN3;NDE | . |
| 0 | 0 | 0.700229111 | 0.157502133 | ACOT9;GP | . |
| 0 | 0 | 0.700229111 | 0.157502133 | SMAD3;XI | . |
| 0 | 0 | 0.700229111 | 0.157502133 | ACOT9;GP | . |
| 0 | 0 | 0.700229111 | 0.157502133 | STARD3;HS | . |
| 0 | 0 | 0.700229111 | 0.157502133 | VASP;DLC1 | . |
| 0 | 0 | 0.753901369 | 0.169261043 | NIPBL;EFE | . |
| 0 | 0 | 0.753901369 | 0.169261043 | DAXX;H2B | . |
| 0 | 0 | 0.783903177 | 0.174963659 | FZR1;PSM | . |
| 0 | 0 | 0.768553319 | 0.16514432 | ACVRL1;SP | . |
| 0 | 0 | 0.653602414 | 0.138120215 | RAPGEF1; | . |
| 0 | 0 | 0.653602414 | 0.138120215 | ABT1;NOP | . |
| 0 | 0 | 0.653602414 | 0.138120215 | SCN9A;SC | . |
| 0 | 0 | 0.653602414 | 0.138120215 | CUL4A;GT | . |
| 0 | 0 | 0.653602414 | 0.138120215 | FMR1;PSE | . |
| 0 | 0 | 0.653602414 | 0.138120215 | TOX;VEGF | . |
| 0 | 0 | 0.653602414 | 0.138120215 | ATAD1;RA | . |
| 0 | 0 | 0.653602414 | 0.138120215 | HEG1;JAK1 | . |
| 0 | 0 | 0.653602414 | 0.138120215 | SMG1;LIG | . |
| 0 | 0 | 0.653602414 | 0.138120215 | TFB1M;MF | . |
| 0 | 0 | 0.653602414 | 0.138120215 | CBLIF;TCN | . |
| 0 | 0 | 0.789511775 | 0.165837472 | VASP;NLG | . |
| 0 | 0 | 0.789511775 | 0.165837472 | EML4;NIPE | . |
| 0 | 0 | 0.725998657 | 0.152318422 | NR4A2;NR | . |
| 0 | 0 | 0.725998657 | 0.152318422 | TRAF7;DD | . |
| 0 | 0 | 0.706436901 | 0.147602934 | CRTC2;EN | . |
| 0 | 0 | 0.706436901 | 0.147602934 | GRK2;PIK3 | . |

| | | | | | |
|---|---|---|---|---|---|
| 0 | 0 | 0.706436901 | 0.147602934 | ADPGK;CH | . |
| 0 | 0 | 0.706436901 | 0.147602934 | TMEM119; | . |
| 0 | 0 | 0.706436901 | 0.147602934 | VCPKMT;E | . |
| 0 | 0 | 0.706436901 | 0.147602934 | C3;ITGAM; | . |
| 0 | 0 | 0.676044242 | 0.137397741 | ROCK1;ELN | . |
| 0 | 0 | 0.676044242 | 0.137397741 | DNAH17;C | . |
| 0 | 0 | 0.676044242 | 0.137397741 | PRKAA1;XE | . |
| 0 | 0 | 0.676044242 | 0.137397741 | SCN9A;CA( | . |
| 0 | 0 | 0.676044242 | 0.137397741 | SEMA4D;H | . |
| 0 | 0 | 0.801945181 | 0.161886173 | RANBP2;SU | . |
| 0 | 0 | 0.757371031 | 0.152111021 | ABCA1;STA | . |
| 0 | 0 | 0.594268477 | 0.118790602 | AIF1 | . |
| 0 | 0 | 0.594268477 | 0.118790602 | UAP1 | . |
| 0 | 0 | 0.594268477 | 0.118790602 | CD47 | . |
| 0 | 0 | 0.594268477 | 0.118790602 | SEH1L | . |
| 0 | 0 | 0.594268477 | 0.118790602 | ARHGAP35 | . |
| 0 | 0 | 0.594268477 | 0.118790602 | NFAT5 | . |
| 0 | 0 | 0.594268477 | 0.118790602 | GCH1 | . |
| 0 | 0 | 0.594268477 | 0.118790602 | ATP7A | . |
| 0 | 0 | 0.594268477 | 0.118790602 | TIFAB | . |
| 0 | 0 | 0.594268477 | 0.118790602 | VCAN | . |
| 0 | 0 | 0.594268477 | 0.118790602 | NIPBL | . |
| 0 | 0 | 0.594268477 | 0.118790602 | SNX8 | . |
| 0 | 0 | 0.594268477 | 0.118790602 | VCPIP1 | . |
| 0 | 0 | 0.594268477 | 0.118790602 | KIFC3 | . |
| 0 | 0 | 0.594268477 | 0.118790602 | ALDH2 | . |
| 0 | 0 | 0.594268477 | 0.118790602 | NRP1 | . |
| 0 | 0 | 0.594268477 | 0.118790602 | IPCEF1 | . |
| 0 | 0 | 0.594268477 | 0.118790602 | PTPRO | . |
| 0 | 0 | 0.594268477 | 0.118790602 | SLC9A3R1 | . |
| 0 | 0 | 0.594268477 | 0.118790602 | LAMB3 | . |
| 0 | 0 | 0.594268477 | 0.118790602 | NIPAL4 | . |
| 0 | 0 | 0.594268477 | 0.118790602 | TAL1 | . |
| 0 | 0 | 0.594268477 | 0.118790602 | MREG | . |
| 0 | 0 | 0.594268477 | 0.118790602 | SNAI1 | . |
| 0 | 0 | 0.594268477 | 0.118790602 | MEF2A | . |
| 0 | 0 | 0.594268477 | 0.118790602 | LDLR | . |
| 0 | 0 | 0.594268477 | 0.118790602 | CHP1 | . |
| 0 | 0 | 0.594268477 | 0.118790602 | CHP1 | . |
| 0 | 0 | 0.594268477 | 0.118790602 | RGS2 | . |
| 0 | 0 | 0.594268477 | 0.118790602 | SIRT1 | . |
| 0 | 0 | 0.594268477 | 0.118790602 | SIRT1 | . |
| 0 | 0 | 0.594268477 | 0.118790602 | MAPRE1 | . |
| 0 | 0 | 0.594268477 | 0.118790602 | HP | . |
| 0 | 0 | 0.594268477 | 0.118790602 | HEXIM1 | . |
| 0 | 0 | 0.594268477 | 0.118790602 | DYNC1H1 | . |
| 0 | 0 | 0.594268477 | 0.118790602 | APOA2 | . |

| | | | | | |
|---|---|---|---|---|---|
| 0 | 0 | 0.594268477 | 0.118790602 | VEGFA | . |
| 0 | 0 | 0.594268477 | 0.118790602 | NR4A3 | . |
| 0 | 0 | 0.594268477 | 0.118790602 | NSMCE2 | . |
| 0 | 0 | 0.594268477 | 0.118790602 | NSMCE2 | . |
| 0 | 0 | 0.594268477 | 0.118790602 | NSMCE2 | . |
| 0 | 0 | 0.594268477 | 0.118790602 | PLEK | . |
| 0 | 0 | 0.594268477 | 0.118790602 | VEGFA | . |
| 0 | 0 | 0.594268477 | 0.118790602 | IK | . |
| 0 | 0 | 0.594268477 | 0.118790602 | NRXN2 | . |
| 0 | 0 | 0.594268477 | 0.118790602 | ADSS2 | . |
| 0 | 0 | 0.594268477 | 0.118790602 | PRPSAP1 | . |
| 0 | 0 | 0.594268477 | 0.118790602 | SCARF1 | . |
| 0 | 0 | 0.594268477 | 0.118790602 | SOCS1 | . |
| 0 | 0 | 0.594268477 | 0.118790602 | NUGGC | . |
| 0 | 0 | 0.594268477 | 0.118790602 | SRI | . |
| 0 | 0 | 0.594268477 | 0.118790602 | STOX1 | . |
| 0 | 0 | 0.594268477 | 0.118790602 | CCL3 | . |
| 0 | 0 | 0.594268477 | 0.118790602 | PTK2B | . |
| 0 | 0 | 0.594268477 | 0.118790602 | DAGLB | . |
| 0 | 0 | 0.594268477 | 0.118790602 | SERPINE1 | . |
| 0 | 0 | 0.594268477 | 0.118790602 | PKD1L3 | . |
| 0 | 0 | 0.594268477 | 0.118790602 | TENT4B | . |
| 0 | 0 | 0.594268477 | 0.118790602 | SGPP1 | . |
| 0 | 0 | 0.594268477 | 0.118790602 | TG | . |
| 0 | 0 | 0.594268477 | 0.118790602 | ANXA1 | . |
| 0 | 0 | 0.71017503 | 0.137201366 | EML4;SEH1 | . |
| 0 | 0 | 0.68780672 | 0.13128341 | OTUB2;PPI | . |
| 0 | 0 | 0.622442564 | 0.116720965 | NIPBL;TIFA | . |
| 0 | 0 | 0.622442564 | 0.116720965 | VPS4B;STC | . |
| 0 | 0 | 0.622442564 | 0.116720965 | LIF;VEGFA | . |
| 0 | 0 | 0.622442564 | 0.116720965 | VCPKMT;E | . |
| 0 | 0 | 0.622442564 | 0.116720965 | TCF7L2;IL1 | . |
| 0 | 0 | 0.622442564 | 0.116720965 | PIK3CA;MA | . |
| 0 | 0 | 0.622442564 | 0.116720965 | NABP2;PRI | . |
| 0 | 0 | 0.622442564 | 0.116720965 | COX17;CO | . |
| 0 | 0 | 0.622442564 | 0.116720965 | PRKAA1;AT | . |
| 0 | 0 | 0.653471698 | 0.119965159 | HSD3B7;NI | . |
| 0 | 0 | 0.653471698 | 0.119965159 | CDKN1A;FZ | . |
| 0 | 0 | 0.653471698 | 0.119965159 | RAMP2;SP | . |
| 0 | 0 | 0.712655837 | 0.127184366 | PARM1;RG | . |
| 0 | 0 | 0.712655837 | 0.127184366 | CDKN2D;SI | . |
| 0 | 0 | 0.695024748 | 0.123581712 | EHD4;STX6 | . |
| 0 | 0 | 0.695024748 | 0.123581712 | SPART;SMU | . |
| 0 | 0 | 0.695024748 | 0.123581712 | CXCL10;CX | . |
| 0 | 0 | 0.695024748 | 0.123581712 | XRN1;SRC; | . |
| 0 | 0 | 0.725791551 | 0.128742807 | ANKRD28;S | . |
| 0 | 0 | 0.778753128 | 0.137559468 | SLC24A1;K | . |

| | | | | | |
|---|---|---|---|---|---|
| 0 | 0 | 0.789775367 | 0.139006813 | SLC24A1;K | . |
| 0 | 0 | 0.735951436 | 0.128944578 | KLF7;MAP: | . |
| 0 | 0 | 0.735951436 | 0.128944578 | RANBP2;CI | . |
| 0 | 0 | 0.735951436 | 0.128944578 | PXK;KCNJ1 | . |
| 0 | 0 | 0.670131932 | 0.116795694 | GGT5;GCL( | . |
| 0 | 0 | 0.781246458 | 0.134178873 | PLK3;DYN( | . |
| 0 | 0 | 0.544714681 | 0.093117664 | ELOVL5 | . |
| 0 | 0 | 0.544714681 | 0.093117664 | PNPT1 | . |
| 0 | 0 | 0.544714681 | 0.093117664 | MYCBP2 | . |
| 0 | 0 | 0.544714681 | 0.093117664 | TIFAB | . |
| 0 | 0 | 0.544714681 | 0.093117664 | VCAN | . |
| 0 | 0 | 0.544714681 | 0.093117664 | CALM1 | . |
| 0 | 0 | 0.544714681 | 0.093117664 | CDADC1 | . |
| 0 | 0 | 0.544714681 | 0.093117664 | H3-3A | . |
| 0 | 0 | 0.544714681 | 0.093117664 | TMEM119 | . |
| 0 | 0 | 0.544714681 | 0.093117664 | CABYR | . |
| 0 | 0 | 0.544714681 | 0.093117664 | ZMYND10 | . |
| 0 | 0 | 0.544714681 | 0.093117664 | POGZ | . |
| 0 | 0 | 0.544714681 | 0.093117664 | ALOX12 | . |
| 0 | 0 | 0.544714681 | 0.093117664 | CABYR | . |
| 0 | 0 | 0.544714681 | 0.093117664 | HIF1A | . |
| 0 | 0 | 0.544714681 | 0.093117664 | ACTN4 | . |
| 0 | 0 | 0.544714681 | 0.093117664 | SIRT1 | . |
| 0 | 0 | 0.544714681 | 0.093117664 | SLFN11 | . |
| 0 | 0 | 0.544714681 | 0.093117664 | ZFYVE28 | . |
| 0 | 0 | 0.544714681 | 0.093117664 | CHP1 | . |
| 0 | 0 | 0.544714681 | 0.093117664 | ACTN4 | . |
| 0 | 0 | 0.544714681 | 0.093117664 | DYSF | . |
| 0 | 0 | 0.544714681 | 0.093117664 | TNF | . |
| 0 | 0 | 0.544714681 | 0.093117664 | TNF | . |
| 0 | 0 | 0.544714681 | 0.093117664 | SIRT1 | . |
| 0 | 0 | 0.544714681 | 0.093117664 | ABCA3 | . |
| 0 | 0 | 0.544714681 | 0.093117664 | SCN4B | . |
| 0 | 0 | 0.544714681 | 0.093117664 | APOA2 | . |
| 0 | 0 | 0.544714681 | 0.093117664 | ADAM17 | . |
| 0 | 0 | 0.544714681 | 0.093117664 | DCUN1D3 | . |
| 0 | 0 | 0.544714681 | 0.093117664 | FLCN | . |
| 0 | 0 | 0.544714681 | 0.093117664 | KCNJ2 | . |
| 0 | 0 | 0.544714681 | 0.093117664 | DUSP1 | . |
| 0 | 0 | 0.544714681 | 0.093117664 | MX2 | . |
| 0 | 0 | 0.544714681 | 0.093117664 | TOX | . |
| 0 | 0 | 0.544714681 | 0.093117664 | RB1CC1 | . |
| 0 | 0 | 0.544714681 | 0.093117664 | SRSF12 | . |
| 0 | 0 | 0.544714681 | 0.093117664 | GTF2B | . |
| 0 | 0 | 0.544714681 | 0.093117664 | APOA2 | . |
| 0 | 0 | 0.544714681 | 0.093117664 | UAP1 | . |
| 0 | 0 | 0.594115428 | 0.098872326 | STARD3;TS | . |

| | | | | | |
|---|---|---|---|---|---|
| 0 | 0 | 0.594115428 | 0.098872326 | ACVRL1;AI | . |
| 0 | 0 | 0.594115428 | 0.098872326 | NDE1;NDE | . |
| 0 | 0 | 0.594115428 | 0.098872326 | TIFAB;TTC3 | . |
| 0 | 0 | 0.594115428 | 0.098872326 | LRRK2;LRP | . |
| 0 | 0 | 0.594115428 | 0.098872326 | POLB;LIG4 | . |
| 0 | 0 | 0.594115428 | 0.098872326 | FLT1;PLA2( | . |
| 0 | 0 | 0.594115428 | 0.098872326 | BCL2L11;B | . |
| 0 | 0 | 0.594115428 | 0.098872326 | SMAD3;SN | . |
| 0 | 0 | 0.632355447 | 0.104825834 | FLCN;MPV | . |
| 0 | 0 | 0.632355447 | 0.104825834 | VASP;DLC1 | . |
| 0 | 0 | 0.632355447 | 0.104825834 | MBOAT1;P | . |
| 0 | 0 | 0.632355447 | 0.104825834 | CXCL10;CX | . |
| 0 | 0 | 0.632355447 | 0.104825834 | PARM1;PR | . |
| 0 | 0 | 0.632355447 | 0.104825834 | RTP4;SEC6 | . |
| 0 | 0 | 0.632355447 | 0.104825834 | RUBCNL;SI | . |
| 0 | 0 | 0.632355447 | 0.104825834 | NUSAP1;ZI | . |
| 0 | 0 | 0.632355447 | 0.104825834 | IGSF8;SVIL | . |
| 0 | 0 | 0.632355447 | 0.104825834 | NLGN3;SYI | . |
| 0 | 0 | 0.811145684 | 0.13300263 | WASHC4;S | . |
| 0 | 0 | 0.653340883 | 0.103926438 | HSPA5;SEC | . |
| 0 | 0 | 0.703378103 | 0.10780408 | SNX18;RO( | . |
| 0 | 0 | 0.687570835 | 0.105030701 | HSD3B7;XE | . |
| 0 | 0 | 0.666579321 | 0.100246735 | ARHGEF11 | . |
| 0 | 0 | 0.612558962 | 0.091659838 | SLC2A10;X | . |
| 0 | 0 | 0.612558962 | 0.091659838 | FBXO40;NI | . |
| 0 | 0 | 0.612558962 | 0.091659838 | KCNJ12;EC | . |
| 0 | 0 | 0.612558962 | 0.091659838 | NLGN3;LRI | . |
| 0 | 0 | 0.612558962 | 0.091659838 | TPPP3;COT | . |
| 0 | 0 | 0.612558962 | 0.091659838 | ARL13B;GL | . |
| 0 | 0 | 0.568251521 | 0.083930171 | KSR1;PDE7 | . |
| 0 | 0 | 0.568251521 | 0.083930171 | SAG;CALM | . |
| 0 | 0 | 0.568251521 | 0.083930171 | SRI;CALM1 | . |
| 0 | 0 | 0.568251521 | 0.083930171 | TRAF7;RPL | . |
| 0 | 0 | 0.568251521 | 0.083930171 | SF3B1;SMI | . |
| 0 | 0 | 0.502784546 | 0.073659555 | MDM4 | . |
| 0 | 0 | 0.502784546 | 0.073659555 | MDM4 | . |
| 0 | 0 | 0.502784546 | 0.073659555 | RBPJ | . |
| 0 | 0 | 0.502784546 | 0.073659555 | MYADM | . |
| 0 | 0 | 0.502784546 | 0.073659555 | SEC24B | . |
| 0 | 0 | 0.502784546 | 0.073659555 | LIPE | . |
| 0 | 0 | 0.502784546 | 0.073659555 | FYN | . |
| 0 | 0 | 0.502784546 | 0.073659555 | TET2 | . |
| 0 | 0 | 0.502784546 | 0.073659555 | UGCG | . |
| 0 | 0 | 0.502784546 | 0.073659555 | USPL1 | . |
| 0 | 0 | 0.502784546 | 0.073659555 | LDLR | . |
| 0 | 0 | 0.502784546 | 0.073659555 | HEXIM1 | . |
| 0 | 0 | 0.502784546 | 0.073659555 | SPRY1 | . |

| | | | | | |
|---|---|---|---|---|---|
| 0 | 0 | 0.502784546 | 0.073659555 | SRSF12 | . |
| 0 | 0 | 0.502784546 | 0.073659555 | IRF1 | . |
| 0 | 0 | 0.502784546 | 0.073659555 | DAGLB | . |
| 0 | 0 | 0.502784546 | 0.073659555 | VEGFA | . |
| 0 | 0 | 0.502784546 | 0.073659555 | TCF7L2 | . |
| 0 | 0 | 0.502784546 | 0.073659555 | SIRT1 | . |
| 0 | 0 | 0.502784546 | 0.073659555 | RPUSD4 | . |
| 0 | 0 | 0.502784546 | 0.073659555 | IK | . |
| 0 | 0 | 0.502784546 | 0.073659555 | LTO1 | . |
| 0 | 0 | 0.502784546 | 0.073659555 | COQ10B | . |
| 0 | 0 | 0.502784546 | 0.073659555 | SIRT1 | . |
| 0 | 0 | 0.502784546 | 0.073659555 | PHLDB1 | . |
| 0 | 0 | 0.502784546 | 0.073659555 | NIPBL | . |
| 0 | 0 | 0.502784546 | 0.073659555 | APOA2 | . |
| 0 | 0 | 0.502784546 | 0.073659555 | CCL3 | . |
| 0 | 0 | 0.502784546 | 0.073659555 | PIH1D1 | . |
| 0 | 0 | 0.502784546 | 0.073659555 | GPD2 | . |
| 0 | 0 | 0.502784546 | 0.073659555 | MMP1 | . |
| 0 | 0 | 0.502784546 | 0.073659555 | DHRS13 | . |
| 0 | 0 | 0.502784546 | 0.073659555 | SLC26A11 | . |
| 0 | 0 | 0.502784546 | 0.073659555 | DPH3 | . |
| 0 | 0 | 0.63736891 | 0.092488843 | CLIP1;TPPF | . |
| 0 | 0 | 0.764917196 | 0.108728812 | DAXX;H2B( | . |
| 0 | 0 | 0.675677084 | 0.095417198 | SDC4;CYP4 | . |
| 0 | 0 | 0.675677084 | 0.095417198 | RYBP;PPP1 | . |
| 0 | 0 | 0.675677084 | 0.095417198 | PSMA2;PSI | . |
| 0 | 0 | 0.593962264 | 0.080195765 | SIK1;ATP1/ | . |
| 0 | 0 | 0.622157508 | 0.082319021 | VCPIP1;PTI | . |
| 0 | 0 | 0.54454294 | 0.071380169 | CUL4A;GTF | . |
| 0 | 0 | 0.54454294 | 0.071380169 | CREBBP;EZ | . |
| 0 | 0 | 0.717466416 | 0.093955243 | KDM6B;AN | . |
| 0 | 0 | 0.749010741 | 0.097516652 | ABT1;RPL1 | . |
| 0 | 0 | 0.46684443 | 0.058717721 | GGT1 | . |
| 0 | 0 | 0.46684443 | 0.058717721 | ATP7A | . |
| 0 | 0 | 0.46684443 | 0.058717721 | TMEM120/ | . |
| 0 | 0 | 0.46684443 | 0.058717721 | POR | . |
| 0 | 0 | 0.46684443 | 0.058717721 | VEGFA | . |
| 0 | 0 | 0.46684443 | 0.058717721 | DAGLB | . |
| 0 | 0 | 0.46684443 | 0.058717721 | CHST3 | . |
| 0 | 0 | 0.46684443 | 0.058717721 | ABHD1 | . |
| 0 | 0 | 0.46684443 | 0.058717721 | NSMCE2 | . |
| 0 | 0 | 0.46684443 | 0.058717721 | GGT1 | . |
| 0 | 0 | 0.46684443 | 0.058717721 | CD81 | . |
| 0 | 0 | 0.46684443 | 0.058717721 | ACTN1 | . |
| 0 | 0 | 0.46684443 | 0.058717721 | SRSF12 | . |
| 0 | 0 | 0.46684443 | 0.058717721 | GGT1 | . |
| 0 | 0 | 0.46684443 | 0.058717721 | VEGFA | . |

| | | | | | |
|---|---|---|---|---|---|
| 0 | 0 | 0.46684443 | 0.058717721 | SAG | . |
| 0 | 0 | 0.46684443 | 0.058717721 | FUS | . |
| 0 | 0 | 0.46684443 | 0.058717721 | PHKG2 | . |
| 0 | 0 | 0.46684443 | 0.058717721 | KMT2E | . |
| 0 | 0 | 0.46684443 | 0.058717721 | EZR | . |
| 0 | 0 | 0.46684443 | 0.058717721 | NABP2 | . |
| 0 | 0 | 0.46684443 | 0.058717721 | VEGFA | . |
| 0 | 0 | 0.46684443 | 0.058717721 | FMR1 | . |
| 0 | 0 | 0.46684443 | 0.058717721 | CEP120 | . |
| 0 | 0 | 0.46684443 | 0.058717721 | DAAM2 | . |
| 0 | 0 | 0.46684443 | 0.058717721 | FLT1 | . |
| 0 | 0 | 0.46684443 | 0.058717721 | COQ10B | . |
| 0 | 0 | 0.46684443 | 0.058717721 | COQ10B | . |
| 0 | 0 | 0.709626742 | 0.087086799 | GALNT11;( | . |
| 0 | 0 | 0.576459489 | 0.070202577 | ACOT9;GP/ | . |
| 0 | 0 | 0.576459489 | 0.070202577 | ATP1A3;AT | . |
| 0 | 0 | 0.84081481 | 0.102349235 | ALAS1;LRR | . |
| 0 | 0 | 0.522731045 | 0.060808564 | HSD3B7;OS | . |
| 0 | 0 | 0.522731045 | 0.060808564 | TPPP3;ZNF | . |
| 0 | 0 | 0.522731045 | 0.060808564 | CYP4F2;SL( | . |
| 0 | 0 | 0.522731045 | 0.060808564 | VCPKMT;EI | . |
| 0 | 0 | 0.522731045 | 0.060808564 | ATAD1;RAI | . |
| 0 | 0 | 0.522731045 | 0.060808564 | ACVRL1;RE | . |
| 0 | 0 | 0.522731045 | 0.060808564 | CXCL10;CX | . |
| 0 | 0 | 0.522731045 | 0.060808564 | PPIF;FKBP5 | . |
| 0 | 0 | 0.522731045 | 0.060808564 | PDE4B;SRI | . |
| 0 | 0 | 0.522731045 | 0.060808564 | MAD2L2;N | . |
| 0 | 0 | 0.67836889 | 0.077994752 | DYNC1H1;I | . |
| 0 | 0 | 0.662449199 | 0.075343055 | ROCK1;ACT | . |
| 0 | 0 | 0.662449199 | 0.075343055 | LAMP1;PPI | . |
| 0 | 0 | 0.593808985 | 0.06522373 | TIMM17A; | . |
| 0 | 0 | 0.559956873 | 0.061482976 | MYBPC2;TI | . |
| 0 | 0 | 0.559956873 | 0.061482976 | MYBPC2;TI | . |
| 0 | 0 | 0.559956873 | 0.061482976 | ACVRL1;RG | . |
| 0 | 0 | 0.559956873 | 0.061482976 | SEMA7A;LI | . |
| 0 | 0 | 0.559956873 | 0.061482976 | INSIG1;PM | . |
| 0 | 0 | 0.43569633 | 0.047116339 | GSTA1 | . |
| 0 | 0 | 0.43569633 | 0.047116339 | TGFBR1 | . |
| 0 | 0 | 0.43569633 | 0.047116339 | SRSF12 | . |
| 0 | 0 | 0.43569633 | 0.047116339 | CHST3 | . |
| 0 | 0 | 0.43569633 | 0.047116339 | JARID2 | . |
| 0 | 0 | 0.43569633 | 0.047116339 | PPIF | . |
| 0 | 0 | 0.43569633 | 0.047116339 | ARL13B | . |
| 0 | 0 | 0.43569633 | 0.047116339 | B4GALT5 | . |
| 0 | 0 | 0.43569633 | 0.047116339 | GSTA1 | . |
| 0 | 0 | 0.43569633 | 0.047116339 | RGCC | . |
| 0 | 0 | 0.43569633 | 0.047116339 | SIRT1 | . |

| | | | | | |
|---|---|---|---|---|---|
| 0 | 0 | 0.43569633 | 0.047116339 | DYNC1H1 | . |
| 0 | 0 | 0.43569633 | 0.047116339 | SLC1A2 | . |
| 0 | 0 | 0.61612894 | 0.065877017 | FLCN;XYLT | . |
| 0 | 0 | 0.61612894 | 0.065877017 | ACVRL1;SD | . |
| 0 | 0 | 0.502596988 | 0.051880272 | NCOA1;SL( | . |
| 0 | 0 | 0.502596988 | 0.051880272 | SMURF1;EI | . |
| 0 | 0 | 0.502596988 | 0.051880272 | GDI1;LRP4 | . |
| 0 | 0 | 0.502596988 | 0.051880272 | GSN;VPS4E | . |
| 0 | 0 | 0.502596988 | 0.051880272 | EPHX2;SIR | . |
| 0 | 0 | 0.502596988 | 0.051880272 | FXYD5;SCN | . |
| 0 | 0 | 0.502596988 | 0.051880272 | ACTN2;MY | . |
| 0 | 0 | 0.747942325 | 0.076191569 | MEF2A;PN | . |
| 0 | 0 | 0.544371069 | 0.053867987 | RGCC;UBE: | . |
| 0 | 0 | 0.604684178 | 0.059290446 | CDKN1A;FZ | . |
| 0 | 0 | 0.408441742 | 0.03802309 | ADRB3 | . |
| 0 | 0 | 0.408441742 | 0.03802309 | SAT1 | . |
| 0 | 0 | 0.408441742 | 0.03802309 | MDM2 | . |
| 0 | 0 | 0.408441742 | 0.03802309 | GNAT2 | . |
| 0 | 0 | 0.408441742 | 0.03802309 | RICTOR | . |
| 0 | 0 | 0.408441742 | 0.03802309 | RILPL2 | . |
| 0 | 0 | 0.408441742 | 0.03802309 | ANXA1 | . |
| 0 | 0 | 0.408441742 | 0.03802309 | EIF6 | . |
| 0 | 0 | 0.408441742 | 0.03802309 | ATP5PB | . |
| 0 | 0 | 0.408441742 | 0.03802309 | SIRT1 | . |
| 0 | 0 | 0.408441742 | 0.03802309 | SMURF1 | . |
| 0 | 0 | 0.408441742 | 0.03802309 | EDN1 | . |
| 0 | 0 | 0.408441742 | 0.03802309 | PIGH | . |
| 0 | 0 | 0.408441742 | 0.03802309 | TMEM120/ | . |
| 0 | 0 | 0.408441742 | 0.03802309 | IK | . |
| 0 | 0 | 0.408441742 | 0.03802309 | STRA6 | . |
| 0 | 0 | 0.408441742 | 0.03802309 | LRRC8C | . |
| 0 | 0 | 0.408441742 | 0.03802309 | XIAP | . |
| 0 | 0 | 0.408441742 | 0.03802309 | ABT1 | . |
| 0 | 0 | 0.408441742 | 0.03802309 | INTS6 | . |
| 0 | 0 | 0.483954343 | 0.044322279 | GALNT11;[ | . |
| 0 | 0 | 0.483954343 | 0.044322279 | EGR2;STAT | . |
| 0 | 0 | 0.483954343 | 0.044322279 | CUL4A;GTF | . |
| 0 | 0 | 0.483954343 | 0.044322279 | ANKRD28;( | . |
| 0 | 0 | 0.529627741 | 0.047212512 | PAK1;CLIP: | . |
| 0 | 0 | 0.64471983 | 0.055428304 | ADAMTS2; | . |
| 0 | 0 | 0.466643315 | 0.037910922 | ABT1;NOP! | . |
| 0 | 0 | 0.466643315 | 0.037910922 | UGGT1;UB | . |
| 0 | 0 | 0.466643315 | 0.037910922 | GRK2;FFAF | . |
| 0 | 0 | 0.466643315 | 0.037910922 | ECE1;ATP2 | . |
| 0 | 0 | 0.466643315 | 0.037910922 | ANKRD28;( | . |
| 0 | 0 | 0.466643315 | 0.037910922 | USB1;INTS( | . |
| 0 | 0 | 0.384393576 | 0.030837448 | LRRC8C | . |

| | | | | | |
|---|---|---|---|---|---|
| 0 | 0 | 0.384393576 | 0.030837448 | ADAR | . |
| 0 | 0 | 0.384393576 | 0.030837448 | MT2A | . |
| 0 | 0 | 0.384393576 | 0.030837448 | HES1 | . |
| 0 | 0 | 0.384393576 | 0.030837448 | PDPN | . |
| 0 | 0 | 0.384393576 | 0.030837448 | MIDN | . |
| 0 | 0 | 0.384393576 | 0.030837448 | PTPN2 | . |
| 0 | 0 | 0.384393576 | 0.030837448 | NOS2 | . |
| 0 | 0 | 0.384393576 | 0.030837448 | TAPT1 | . |
| 0 | 0 | 0.384393576 | 0.030837448 | KCNS1 | . |
| 0 | 0 | 0.384393576 | 0.030837448 | SMURF1 | . |
| 0 | 0 | 0.384393576 | 0.030837448 | CCNT1 | . |
| 0 | 0 | 0.384393576 | 0.030837448 | SCN4B | . |
| 0 | 0 | 0.384393576 | 0.030837448 | SLC8A1 | . |
| 0 | 0 | 0.384393576 | 0.030837448 | SARS1 | . |
| 0 | 0 | 0.809632431 | 0.062158085 | GALNT11;[illegible] | . |
| 0 | 0 | 0.645475553 | 0.048948372 | CD74;DYN[illegible] | . |
| 0 | 0 | 0.502409289 | 0.036297656 | GALNT11;[illegible] | . |
| 0 | 0 | 0.502409289 | 0.036297656 | VMP1;DN[illegible] | . |
| 0 | 0 | 0.450526151 | 0.032462062 | HOXC9;TG[illegible] | . |
| 0 | 0 | 0.450526151 | 0.032462062 | UGGT1;UB | . |
| 0 | 0 | 0.450526151 | 0.032462062 | ACTN2;PT[illegible] | . |
| 0 | 0 | 0.609241623 | 0.043623195 | FLCN;SMA[illegible] | . |
| 0 | 0 | 0.760311111 | 0.053864873 | ITGAM;SL[illegible] | . |
| 0 | 0 | 0.363017429 | 0.025118915 | MDM4 | . |
| 0 | 0 | 0.363017429 | 0.025118915 | KAT5 | . |
| 0 | 0 | 0.363017429 | 0.025118915 | ALDH2 | . |
| 0 | 0 | 0.363017429 | 0.025118915 | XYLT1 | . |
| 0 | 0 | 0.363017429 | 0.025118915 | CHMP2A | . |
| 0 | 0 | 0.363017429 | 0.025118915 | UAP1 | . |
| 0 | 0 | 0.363017429 | 0.025118915 | MYBL1 | . |
| 0 | 0 | 0.363017429 | 0.025118915 | C3 | . |
| 0 | 0 | 0.363017429 | 0.025118915 | MCU | . |
| 0 | 0 | 0.363017429 | 0.025118915 | TIMM17A | . |
| 0 | 0 | 0.363017429 | 0.025118915 | BPNT1 | . |
| 0 | 0 | 0.363017429 | 0.025118915 | CHRNE | . |
| 0 | 0 | 0.363017429 | 0.025118915 | TFIP11 | . |
| 0 | 0 | 0.621586101 | 0.042749676 | DHRS13;S[illegible] | . |
| 0 | 0 | 0.533062149 | 0.036441722 | KDM6B;GJ | . |
| 0 | 0 | 0.584610006 | 0.039772996 | VCPIP1;SE[illegible] | . |
| 0 | 0 | 0.601190476 | 0.039780691 | UGCG;LA[illegible] | . |
| 0 | 0 | 0.489820755 | 0.031837385 | WNT2B;SC | . |
| 0 | 0 | 0.489820755 | 0.031837385 | WNT2B;SC | . |
| 0 | 0 | 0.489820755 | 0.031837385 | ACTN2;CFL | . |
| 0 | 0 | 0.489820755 | 0.031837385 | TCF4;ARH[illegible] | . |
| 0 | 0 | 0.489820755 | 0.031837385 | TCF7L2;AC | . |
| 0 | 0 | 0.489820755 | 0.031837385 | STXBP3;ST[illegible] | . |
| 0 | 0 | 0.681460074 | 0.043838909 | ARRDC4;R[illegible] | . |

| | | | | | |
|---|---|---|---|---|---|
| 0 | 0 | 0.435483465 | 0.027823415 | S1PR1;SM | . |
| 0 | 0 | 0.435483465 | 0.027823415 | GOSR1;CO | . |
| 0 | 0 | 0.435483465 | 0.027823415 | MID1IP1;P | . |
| 0 | 0 | 0.435483465 | 0.027823415 | PPIF;BAK1 | . |
| 0 | 0 | 0.435483465 | 0.027823415 | PPIF;FKBP | . |
| 0 | 0 | 0.435483465 | 0.027823415 | SIPA1L1;LR | . |
| 0 | 0 | 0.435483465 | 0.027823415 | PAK1;CNO | . |
| 0 | 0 | 0.522370706 | 0.03243011 | CLIC5;CLIC | . |
| 0 | 0 | 0.522370706 | 0.03243011 | KCNJ12;KC | . |
| 0 | 0 | 0.624766633 | 0.038177215 | RPS9;RPS7 | . |
| 0 | 0 | 0.764958844 | 0.046284422 | MRPS15;A | . |
| 0 | 0 | 0.343891403 | 0.0205397 | ADRB3 | . |
| 0 | 0 | 0.343891403 | 0.0205397 | LMTK2 | . |
| 0 | 0 | 0.343891403 | 0.0205397 | PPARD | . |
| 0 | 0 | 0.343891403 | 0.0205397 | RIMBP3B | . |
| 0 | 0 | 0.343891403 | 0.0205397 | SOX13 | . |
| 0 | 0 | 0.343891403 | 0.0205397 | EDN1 | . |
| 0 | 0 | 0.607060272 | 0.035890236 | CSF1;LRRK | . |
| 0 | 0 | 0.477846295 | 0.027930177 | RTP4;GNA | . |
| 0 | 0 | 0.477846295 | 0.027930177 | STXBP3;ST | . |
| 0 | 0 | 0.477846295 | 0.027930177 | SVIL;TAL1; | . |
| 0 | 0 | 0.63343729 | 0.036925913 | UPF2;CTIF; | . |
| 0 | 0 | 0.544026939 | 0.03137924 | GABRR2;SL | . |
| 0 | 0 | 0.618154312 | 0.035116781 | HSD11B1;S | . |
| 0 | 0 | 0.421411275 | 0.023868532 | FBXO4;PRK | . |
| 0 | 0 | 0.421411275 | 0.023868532 | GPD2;DHR | . |
| 0 | 0 | 0.421411275 | 0.023868532 | FLCN;IL1B | . |
| 0 | 0 | 0.559458147 | 0.029559415 | RANBP2;P | . |
| 0 | 0 | 0.611679747 | 0.032290033 | POLB;CUL4 | . |
| 0 | 0 | 0.466442049 | 0.024506064 | DFFB;H1-4 | . |
| 0 | 0 | 0.466442049 | 0.024506064 | CEBPG;SIR | . |
| 0 | 0 | 0.326677979 | 0.016852791 | SLC8A1 | . |
| 0 | 0 | 0.326677979 | 0.016852791 | FMR1 | . |
| 0 | 0 | 0.326677979 | 0.016852791 | MAD2L2 | . |
| 0 | 0 | 0.326677979 | 0.016852791 | NOS2 | . |
| 0 | 0 | 0.326677979 | 0.016852791 | SERPINE1 | . |
| 0 | 0 | 0.326677979 | 0.016852791 | SUN2 | . |
| 0 | 0 | 0.326677979 | 0.016852791 | EPHX2 | . |
| 0 | 0 | 0.326677979 | 0.016852791 | MYBL1 | . |
| 0 | 0 | 0.326677979 | 0.016852791 | DHRS3 | . |
| 0 | 0 | 0.502221448 | 0.025674308 | SH3PXD2B | . |
| 0 | 0 | 0.502221448 | 0.025674308 | RAMP2;PA | . |
| 0 | 0 | 0.62129975 | 0.031446077 | ACOT9;DN | . |
| 0 | 0 | 0.677393142 | 0.034152708 | CD164;TEN | . |
| 0 | 0 | 0.408218597 | 0.020492025 | SETDB1;EZ | . |
| 0 | 0 | 0.408218597 | 0.020492025 | INSIG1;PM | . |
| 0 | 0 | 0.408218597 | 0.020492025 | SETX;ZNF4 | . |

| | | | | | |
|---|---|---|---|---|---|
| 0 | 0 | 0.408218597 | 0.020492025 | METTL1;M | . |
| 0 | 0 | 0.492717081 | 0.022839569 | TRAF7;RPL | . |
| 0 | 0 | 0.311103929 | 0.013869976 | HOPX | . |
| 0 | 0 | 0.311103929 | 0.013869976 | CYP2S1 | . |
| 0 | 0 | 0.311103929 | 0.013869976 | VEGFA | . |
| 0 | 0 | 0.311103929 | 0.013869976 | GSTA1 | . |
| 0 | 0 | 0.311103929 | 0.013869976 | GSTA1 | . |
| 0 | 0 | 0.311103929 | 0.013869976 | SYCP1 | . |
| 0 | 0 | 0.311103929 | 0.013869976 | NIPBL | . |
| 0 | 0 | 0.311103929 | 0.013869976 | SIK1 | . |
| 0 | 0 | 0.311103929 | 0.013869976 | STX4 | . |
| 0 | 0 | 0.311103929 | 0.013869976 | DDX3X | . |
| 0 | 0 | 0.311103929 | 0.013869976 | SCN4B | . |
| 0 | 0 | 0.395825475 | 0.017605767 | LIPE;APOA | . |
| 0 | 0 | 0.395825475 | 0.017605767 | INSIG1;PM | . |
| 0 | 0 | 0.395825475 | 0.017605767 | RIOK3;RIOI | . |
| 0 | 0 | 0.395825475 | 0.017605767 | PPA1;BPNT | . |
| 0 | 0 | 0.445188679 | 0.018871879 | TBX21;HES | . |
| 0 | 0 | 0.445188679 | 0.018871879 | ACVRL1;SD | . |
| 0 | 0 | 0.445188679 | 0.018871879 | NLGN3;IL1 | . |
| 0 | 0 | 0.445188679 | 0.018871879 | PARP3;LTC | . |
| 0 | 0 | 0.593040847 | 0.025048787 | FAM111B;I | . |
| 0 | 0 | 0.656308674 | 0.027494572 | KDM4A;PA | . |
| 0 | 0 | 0.66009034 | 0.026149414 | FZD1;TENN | . |
| 0 | 0 | 0.38416136 | 0.015135838 | DYNC1H1;I | . |
| 0 | 0 | 0.38416136 | 0.015135838 | KSR1;PDE7 | . |
| 0 | 0 | 0.38416136 | 0.015135838 | FBXO4;PRK | . |
| 0 | 0 | 0.38416136 | 0.015135838 | ANKRD28;( | . |
| 0 | 0 | 0.69577812 | 0.027129238 | GALNT11;C | . |
| 0 | 0 | 0.296945701 | 0.011446429 | CYP2S1 | . |
| 0 | 0 | 0.296945701 | 0.011446429 | CUL4A | . |
| 0 | 0 | 0.474745187 | 0.018066323 | CYP24A1;C | . |
| 0 | 0 | 0.638237094 | 0.023308954 | RANBP2;SU | . |
| 0 | 0 | 0.373163766 | 0.013020063 | POGZ;OIP5 | . |
| 0 | 0 | 0.373163766 | 0.013020063 | CUL4A;GTF | . |
| 0 | 0 | 0.373163766 | 0.013020063 | SVIL;PRKCE | . |
| 0 | 0 | 0.373163766 | 0.013020063 | SEC61G;UE | . |
| 0 | 0 | 0.373163766 | 0.013020063 | PRKAG2;CF | . |
| 0 | 0 | 0.633768052 | 0.021691954 | RFLNB;XYL | . |
| 0 | 0 | 0.602764649 | 0.020533897 | NPC1;GALN | . |
| 0 | 0 | 0.284018624 | 0.009469736 | H1-4 | . |
| 0 | 0 | 0.284018624 | 0.009469736 | INTS6 | . |
| 0 | 0 | 0.284018624 | 0.009469736 | TTLL7 | . |
| 0 | 0 | 0.284018624 | 0.009469736 | GCLC | . |
| 0 | 0 | 0.362777149 | 0.011206017 | SRSF12;WI | . |
| 0 | 0 | 0.41670012 | 0.012759398 | GABRR2;CI | . |
| 0 | 0 | 0.582232042 | 0.017718775 | SLC9A3R1; | . |

| | | | | | |
|---|---|---|---|---|---|
| 0 | 0 | 0.53097699 | 0.015527394 | EIF4A1;RPS | . |
| 0 | 0 | 0.54926276 | 0.016051758 | CD74;DYN( | . |
| 0 | 0 | 0.272168803 | 0.007851956 | BPNT1 | . |
| 0 | 0 | 0.272168803 | 0.007851956 | GJB6 | . |
| 0 | 0 | 0.272168803 | 0.007851956 | NRP1 | . |
| 0 | 0 | 0.272168803 | 0.007851956 | SEC16B | . |
| 0 | 0 | 0.272168803 | 0.007851956 | NLGN3 | . |
| 0 | 0 | 0.272168803 | 0.007851956 | CALM1 | . |
| 0 | 0 | 0.450111298 | 0.012693864 | POLB;KAT5 | . |
| 0 | 0 | 0.479801626 | 0.013250379 | CUL4A;POI | . |
| 0 | 0 | 0.407995283 | 0.011199256 | CUL4A;AN) | . |
| 0 | 0 | 0.407995283 | 0.011199256 | WNT2B;SC | . |
| 0 | 0 | 0.407995283 | 0.011199256 | SEH1L;CHN | . |
| 0 | 0 | 0.407995283 | 0.011199256 | SEH1L;CHN | . |
| 0 | 0 | 0.407995283 | 0.011199256 | CREBBP;PD | . |
| 0 | 0 | 0.352951971 | 0.009649391 | TREX1;LIG4 | . |
| 0 | 0 | 0.352951971 | 0.009649391 | OIP5;H3-3A | . |
| 0 | 0 | 0.352951971 | 0.009649391 | CUL4A;GTF | . |
| 0 | 0 | 0.352951971 | 0.009649391 | SCN4B;KCN | . |
| 0 | 0 | 0.524843398 | 0.014106446 | RPS9;RPS7 | . |
| 0 | 0 | 0.50184534 | 0.013212511 | FLCN;PLK3 | . |
| 0 | 0 | 0.442456699 | 0.011280891 | LTB4R2;NN | . |
| 0 | 0 | 0.261266968 | 0.006523816 | ANXA1 | . |
| 0 | 0 | 0.261266968 | 0.006523816 | PARP3 | . |
| 0 | 0 | 0.261266968 | 0.006523816 | EIF6 | . |
| 0 | 0 | 0.261266968 | 0.006523816 | CREBL2 | . |
| 0 | 0 | 0.261266968 | 0.006523816 | CYP2S1 | . |
| 0 | 0 | 0.261266968 | 0.006523816 | INTS6 | . |
| 0 | 0 | 0.399645745 | 0.009829854 | CABYR;DNA | . |
| 0 | 0 | 0.343643908 | 0.008312662 | RTP4;GNAT | . |
| 0 | 0 | 0.435057254 | 0.01002328 | SARS1;MET | . |
| 0 | 0 | 0.532356005 | 0.012196817 | PLK3;FLCN | . |
| 0 | 0 | 0.391630189 | 0.008627762 | RAMP2;PA | . |
| 0 | 0 | 0.251203736 | 0.005430403 | ARHGAP17 | . |
| 0 | 0 | 0.251203736 | 0.005430403 | FYN | . |
| 0 | 0 | 0.251203736 | 0.005430403 | LYST | . |
| 0 | 0 | 0.251203736 | 0.005430403 | NUSAP1 | . |
| 0 | 0 | 0.251203736 | 0.005430403 | GLRX | . |
| 0 | 0 | 0.334813181 | 0.007163988 | RTP4;GNAT | . |
| 0 | 0 | 0.507260435 | 0.01055545 | NIPBL;SFP( | . |
| 0 | 0 | 0.459448534 | 0.009547179 | CLIC5;GAB | . |
| 0 | 0 | 0.383928968 | 0.007572468 | SEH1L;CHN | . |
| 0 | 0 | 0.501657073 | 0.009576322 | CD74;DYN( | . |
| 0 | 0 | 0.326423991 | 0.006176308 | MBD2;ACT | . |
| 0 | 0 | 0.326423991 | 0.006176308 | NR4A3;CO( | . |
| 0 | 0 | 0.241885928 | 0.004527943 | ATP5PB | . |
| 0 | 0 | 0.241885928 | 0.004527943 | PIGH | . |

| | | | | | |
|---|---|---|---|---|---|
| 0 | 0 | 0.241885928 | 0.004527943 | NR1H3 | . |
| 0 | 0 | 0.477254508 | 0.008765652 | CLIC5;RIPC | . |
| 0 | 0 | 0.376523948 | 0.006646001 | TIMM17A; | . |
| 0 | 0 | 0.687163392 | 0.012033636 | GALNT11;A | . |
| 0 | 0 | 0.414268337 | 0.00702254 | SLC39A8;U | . |
| 0 | 0 | 0.471477144 | 0.007904711 | ATAD1;PPF | . |
| 0 | 0 | 0.318444029 | 0.005326582 | SLFN11;AL | . |
| 0 | 0 | 0.318444029 | 0.005326582 | TBX21;DLL | . |
| 0 | 0 | 0.233233678 | 0.003781364 | TREX1 | . |
| 0 | 0 | 0.233233678 | 0.003781364 | CALM1 | . |
| 0 | 0 | 0.727311487 | 0.011573897 | HDAC4;NL( | . |
| 0 | 0 | 0.369398362 | 0.005832612 | PARP3;NSM | . |
| 0 | 0 | 0.440745693 | 0.006861672 | SFPQ;NSM | . |
| 0 | 0 | 0.310844066 | 0.00459517 | RBPJ;SMAI | . |
| 0 | 0 | 0.310844066 | 0.00459517 | ZNF207;CE | . |
| 0 | 0 | 0.225178135 | 0.003162432 | OIP5 | . |
| 0 | 0 | 0.225178135 | 0.003162432 | OIP5 | . |
| 0 | 0 | 0.225178135 | 0.003162432 | HSD17B1 | . |
| 0 | 0 | 0.225178135 | 0.003162432 | TET2 | . |
| 0 | 0 | 0.225178135 | 0.003162432 | ARL13B | . |
| 0 | 0 | 0.46033023 | 0.006422084 | CLIC5;RIPC | . |
| 0 | 0 | 0.568369941 | 0.007730366 | GGT5;RPS9 | . |
| 0 | 0 | 0.395369322 | 0.004911153 | NUDT7;AB | . |
| 0 | 0 | 0.210626186 | 0.002220542 | WNT2B | . |
| 0 | 0 | 0.210626186 | 0.002220542 | OIP5 | . |
| 0 | 0 | 0.210626186 | 0.002220542 | PIGH | . |
| 0 | 0 | 0.210626186 | 0.002220542 | TIMM17A | . |
| 0 | 0 | 0.290070833 | 0.002954984 | TBX21;DLL | . |
| 0 | 0 | 0.343396226 | 0.003457877 | RAMP2;PA | . |
| 0 | 0 | 0.343396226 | 0.003457877 | PECAM1;C | . |
| 0 | 0 | 0.647867057 | 0.006265272 | H2AW;BTG | . |
| 0 | 0 | 0.204032334 | 0.001863992 | CNOT7 | . |
| 0 | 0 | 0.283748544 | 0.002551769 | CEBPG;SIR | . |
| 0 | 0 | 0.19783811 | 0.001566366 | CCDC65 | . |
| 0 | 0 | 0.19783811 | 0.001566366 | PARP3 | . |
| 0 | 0 | 0.515436856 | 0.003900576 | EGR2;NIPB | . |
| 0 | 0 | 0.192008251 | 0.001317579 | POLB | . |
| 0 | 0 | 0.192008251 | 0.001317579 | CNOT7 | . |
| 0 | 0 | 0.192008251 | 0.001317579 | SNX10 | . |
| 0 | 0 | 0.3573124 | 0.00211669 | PPP1R10;S | . |
| 0 | 0 | 0.561437496 | 0.002978402 | TSFM;MRP | . |
| 0 | 0 | 0.383463657 | 0.002001229 | SDHAF2;C( | . |
| 0 | 0 | 0.383463657 | 0.002001229 | RPS9;RPS7 | . |
| 0 | 0 | 0.181320178 | 9.35E-04 | KIF5C | . |
| 0 | 0 | 0.176409441 | 7.88E-04 | OIP5 | . |
| 0 | 0 | 0.171757164 | 6.65E-04 | BCOR | . |
| 0 | 0 | 0.171757164 | 6.65E-04 | ALYREF | . |

| | | | | | |
|---|---|---|---|---|---|
| 0 | 0 | 0.296415094 | 0.001061482 | NLGN3;FZD | . |
| 0 | 0 | 0.167343466 | 5.62E-04 | PIGH | . |
| 0 | 0 | 0.167343466 | 5.62E-04 | IFT52 | . |
| 0 | 0 | 0.430589448 | 0.001342306 | POLB;PSM. | . |
| 0 | 0 | 0.521785667 | 0.001584515 | FZD1;NRP1 | . |
| 0 | 0 | 0.32591544 | 9.03E-04 | NEK6;ZNF2 | . |
| 0 | 0 | 0.232944441 | 5.94E-04 | ACTN2;PTK | . |
| 0 | 0 | 0.159161976 | 4.02E-04 | MRPS15 | . |
| 0 | 0 | 0.49170851 | 0.001160897 | SEC16B;DY | . |
| 0 | 0 | 0.30665512 | 4.89E-04 | KCNS1;ACT | . |
| 0 | 0 | 0.382997644 | 5.52E-04 | CAST;NLGN | . |
| 0 | 0 | 0.138795289 | 1.49E-04 | POLB | . |
| 0 | 0 | 0.532673288 | 5.20E-04 | DFFB;TCF7 | . |
| 0 | 0 | 0.135895865 | 1.27E-04 | ATP5PB | . |
| 0 | 0 | 0.12787981 | 7.77E-05 | PPARD | . |
| 0 | 0 | 0.260430351 | 7.62E-05 | TSFM;MRP | . |
| 0 | 0 | 0.114379085 | 2.97E-05 | FOXRED1 | . |
| 0 | 0 | 0.114379085 | 2.97E-05 | FOXRED1 | . |
| 0 | 0 | 0.425044115 | 4.40E-05 | NLGN3;CN | . |
| 0 | 0 | 0.156921916 | 1.25E-05 | COPZ1;TAF | . |
| 0 | 0 | 0.094421492 | 4.66E-06 | SDHAF2 | . |
| 0 | 0 | 0.149672429 | 7.24E-06 | TSFM;MRP | . |
| 0 | 0 | 0.093067227 | 4.03E-06 | SDHAF2 | . |
| 0 | 0 | 0.138470429 | 2.94E-06 | MRPS15;ET | . |
| 0 | 0 | 0.134164505 | 2.07E-06 | GNAL;C5AI | . |
| 0 | 0 | 0.404625237 | 5.13E-06 | NLGN3;STX | . |
| 0 | 0 | 0.12630515 | 1.13E-06 | TSFM;MRP | . |
| 0 | 0 | 0.07395362 | 4.78E-07 | MRPS15 | . |

**ns were highlighted)**

| Term | Overlap | P-value | Adjusted P-value | Old P-value | Old Adjusted P-value |
|---|---|---|---|---|---|
| TNF signaling pathway | 57/112 | 1.20E-21 | 2.76E-19 | 0 | 0 |
| Lipid and atherosclerosis | 84/215 | 1.79E-21 | 2.76E-19 | 0 | 0 |
| NOD-like receptor signaling pathw | 73/181 | 8.96E-20 | 9.23E-18 | 0 | 0 |
| NF-kappa B signaling pathway | 52/104 | 1.99E-19 | 1.54E-17 | 0 | 0 |
| Hepatitis B | 67/162 | 5.91E-19 | 3.65E-17 | 0 | 0 |
| C-type lectin receptor signaling pat | 51/104 | 1.31E-18 | 6.74E-17 | 0 | 0 |
| Pertussis | 42/76 | 4.68E-18 | 2.07E-16 | 0 | 0 |
| Influenza A | 68/172 | 5.73E-18 | 2.21E-16 | 0 | 0 |
| Toll-like receptor signaling pathwa | 49/104 | 5.03E-17 | 1.73E-15 | 0 | 0 |
| Measles | 58/139 | 8.19E-17 | 2.53E-15 | 0 | 0 |
| Tuberculosis | 68/180 | 9.41E-17 | 2.64E-15 | 0 | 0 |
| Epstein-Barr virus infection | 73/202 | 1.19E-16 | 3.05E-15 | 0 | 0 |
| Chemokine signaling pathway | 70/192 | 2.95E-16 | 6.99E-15 | 0 | 0 |
| Coronavirus disease | 79/232 | 3.39E-16 | 6.99E-15 | 0 | 0 |
| Osteoclast differentiation | 54/127 | 3.53E-16 | 6.99E-15 | 0 | 0 |
| Necroptosis | 62/159 | 3.62E-16 | 6.99E-15 | 0 | 0 |
| Kaposi sarcoma-associated herpes | 70/193 | 4.04E-16 | 7.34E-15 | 0 | 0 |
| Human cytomegalovirus infection | 75/225 | 6.94E-15 | 1.19E-13 | 0 | 0 |
| Toxoplasmosis | 48/112 | 1.06E-14 | 1.72E-13 | 0 | 0 |
| JAK-STAT signaling pathway | 60/162 | 1.71E-14 | 2.64E-13 | 0 | 0 |
| Pathways in cancer | 135/531 | 1.90E-14 | 2.80E-13 | 0 | 0 |
| Leishmaniasis | 38/77 | 2.43E-14 | 3.42E-13 | 0 | 0 |
| Cytokine-cytokine receptor interac | 87/295 | 1.59E-13 | 2.14E-12 | 0 | 0 |
| Shigellosis | 76/246 | 4.06E-13 | 5.23E-12 | 0 | 0 |
| Th17 cell differentiation | 44/107 | 7.81E-13 | 9.65E-12 | 0 | 0 |
| Viral protein interaction with cytol | 42/100 | 1.10E-12 | 1.31E-11 | 0 | 0 |
| Yersinia infection | 51/137 | 1.24E-12 | 1.41E-11 | 0 | 0 |
| IL-17 signaling pathway | 40/94 | 2.28E-12 | 2.52E-11 | 0 | 0 |
| Salmonella infection | 73/249 | 1.93E-11 | 2.00E-10 | 0 | 0 |
| MAPK signaling pathway | 82/294 | 1.94E-11 | 2.00E-10 | 0 | 0 |
| Apoptosis | 50/142 | 2.23E-11 | 2.23E-10 | 0 | 0 |
| Th1 and Th2 cell differentiation | 38/92 | 2.39E-11 | 2.31E-10 | 0 | 0 |
| Chagas disease | 40/102 | 4.98E-11 | 4.66E-10 | 0 | 0 |
| Inflammatory bowel disease | 30/65 | 1.09E-10 | 9.86E-10 | 0 | 0 |
| PD-L1 expression and PD-1 checkp | 36/89 | 1.62E-10 | 1.43E-09 | 0 | 0 |
| AGE-RAGE signaling pathway in dia | 38/100 | 4.43E-10 | 3.71E-09 | 0 | 0 |
| Legionellosis | 27/57 | 4.45E-10 | 3.71E-09 | 0 | 0 |
| RIG-I-like receptor signaling pathw | 30/70 | 1.02E-09 | 8.26E-09 | 0 | 0 |
| Hepatitis C | 50/157 | 1.27E-09 | 1.01E-08 | 0 | 0 |
| Pathogenic Escherichia coli infectio | 58/197 | 1.85E-09 | 1.43E-08 | 0 | 0 |
| FoxO signaling pathway | 44/131 | 1.93E-09 | 1.45E-08 | 0 | 0 |
| Viral carcinogenesis | 59/203 | 2.31E-09 | 1.70E-08 | 0 | 0 |
| Human T-cell leukemia virus 1 infe | 61/219 | 7.53E-09 | 5.41E-08 | 0 | 0 |
| Fluid shear stress and atherosclerc | 43/139 | 4.62E-08 | 3.25E-07 | 0 | 0 |

| | | | | | |
|---|---|---|---|---|---|
| Malaria | 22/50 | 9.63E-08 | 6.61E-07 | 0 | 0 |
| Human immunodeficiency virus 1 i | 56/212 | 2.17E-07 | 1.46E-06 | 0 | 0 |
| Transcriptional misregulation in ca | 51/192 | 6.17E-07 | 4.06E-06 | 0 | 0 |
| African trypanosomiasis | 17/37 | 1.31E-06 | 8.44E-06 | 0 | 0 |
| Epithelial cell signaling in Helicoba | 25/70 | 1.60E-06 | 1.01E-05 | 0 | 0 |
| Rheumatoid arthritis | 30/93 | 1.81E-06 | 1.12E-05 | 0 | 0 |
| Cytosolic DNA-sensing pathway | 23/63 | 2.68E-06 | 1.62E-05 | 0 | 0 |
| Neurotrophin signaling pathway | 35/119 | 2.93E-06 | 1.74E-05 | 0 | 0 |
| HIF-1 signaling pathway | 32/109 | 8.00E-06 | 4.65E-05 | 0 | 0 |
| Growth hormone synthesis, secret | 34/119 | 8.13E-06 | 4.65E-05 | 0 | 0 |
| Chronic myeloid leukemia | 25/76 | 8.71E-06 | 4.89E-05 | 0 | 0 |
| Non-small cell lung cancer | 24/72 | 1.02E-05 | 5.56E-05 | 0 | 0 |
| Rap1 signaling pathway | 51/210 | 1.03E-05 | 5.56E-05 | 0 | 0 |
| Notch signaling pathway | 21/59 | 1.14E-05 | 6.08E-05 | 0 | 0 |
| Fc gamma R-mediated phagocytos | 29/97 | 1.42E-05 | 7.46E-05 | 0 | 0 |
| Amoebiasis | 30/102 | 1.46E-05 | 7.50E-05 | 0 | 0 |
| Platelet activation | 34/124 | 2.12E-05 | 1.08E-04 | 0 | 0 |
| Complement and coagulation casc | 26/85 | 2.50E-05 | 1.24E-04 | 0 | 0 |
| Neutrophil extracellular trap forma | 46/189 | 2.57E-05 | 1.26E-04 | 0 | 0 |
| Pancreatic cancer | 24/76 | 2.82E-05 | 1.36E-04 | 0 | 0 |
| B cell receptor signaling pathway | 25/81 | 3.00E-05 | 1.43E-04 | 0 | 0 |
| Fc epsilon RI signaling pathway | 22/68 | 3.96E-05 | 1.84E-04 | 0 | 0 |
| Small cell lung cancer | 27/92 | 4.00E-05 | 1.84E-04 | 0 | 0 |
| Human papillomavirus infection | 70/331 | 4.29E-05 | 1.95E-04 | 0 | 0 |
| Proteoglycans in cancer | 48/205 | 5.01E-05 | 2.24E-04 | 0 | 0 |
| Adipocytokine signaling pathway | 22/69 | 5.10E-05 | 2.25E-04 | 0 | 0 |
| Leukocyte transendothelial migrat | 31/114 | 5.72E-05 | 2.49E-04 | 0 | 0 |
| T cell receptor signaling pathway | 29/104 | 5.97E-05 | 2.56E-04 | 0 | 0 |
| Prolactin signaling pathway | 22/70 | 6.51E-05 | 2.76E-04 | 0 | 0 |
| Natural killer cell mediated cytoto: | 34/131 | 7.20E-05 | 3.01E-04 | 0 | 0 |
| Acute myeloid leukemia | 21/67 | 9.89E-05 | 4.08E-04 | 0 | 0 |
| Non-alcoholic fatty liver disease | 38/155 | 1.06E-04 | 4.29E-04 | 0 | 0 |
| Insulin resistance | 29/108 | 1.25E-04 | 5.00E-04 | 0 | 0 |
| Ferroptosis | 15/41 | 1.41E-04 | 5.57E-04 | 0 | 0 |
| Sphingolipid signaling pathway | 30/119 | 3.22E-04 | 0.001259411 | 0 | 0 |
| GnRH signaling pathway | 25/93 | 3.48E-04 | 0.001343723 | 0 | 0 |
| Focal adhesion | 44/201 | 4.91E-04 | 0.001871406 | 0 | 0 |
| Phagosome | 35/152 | 6.81E-04 | 0.002565683 | 0 | 0 |
| Inflammatory mediator regulation | 25/98 | 8.11E-04 | 0.003019282 | 0 | 0 |
| Protein processing in endoplasmic | 38/171 | 8.50E-04 | 0.003126036 | 0 | 0 |
| Apelin signaling pathway | 32/137 | 8.74E-04 | 0.003141814 | 0 | 0 |
| Insulin signaling pathway | 32/137 | 8.74E-04 | 0.003141814 | 0 | 0 |
| VEGF signaling pathway | 17/59 | 0.001285144 | 0.004564476 | 0 | 0 |
| ErbB signaling pathway | 22/85 | 0.00132402 | 0.004649115 | 0 | 0 |
| Colorectal cancer | 22/86 | 0.001560102 | 0.005416533 | 0 | 0 |
| Gastric acid secretion | 20/76 | 0.001730431 | 0.005941146 | 0 | 0 |
| Bladder cancer | 13/41 | 0.001806872 | 0.006135422 | 0 | 0 |

| | | | | | |
|---|---|---|---|---|---|
| Cellular senescence | 34/156 | 0.002176199 | 0.00730919 | 0 | 0 |
| PI3K-Akt signaling pathway | 66/354 | 0.002466201 | 0.00819415 | 0 | 0 |
| p53 signaling pathway | 19/73 | 0.002555804 | 0.008401525 | 0 | 0 |
| Parathyroid hormone synthesis, se | 25/106 | 0.00264379 | 0.008599274 | 0 | 0 |
| Relaxin signaling pathway | 29/129 | 0.00274344 | 0.008830448 | 0 | 0 |
| Renal cell carcinoma | 18/69 | 0.003186985 | 0.010152356 | 0 | 0 |
| Prostate cancer | 23/97 | 0.003566402 | 0.011245085 | 0 | 0 |
| Adherens junction | 18/71 | 0.004435084 | 0.013842838 | 0 | 0 |
| Hematopoietic cell lineage | 23/99 | 0.00467053 | 0.014431938 | 0 | 0 |
| mTOR signaling pathway | 32/154 | 0.006151656 | 0.018820411 | 0 | 0 |
| Aldosterone synthesis and secretic | 22/98 | 0.008498157 | 0.025494471 | 0 | 0 |
| Choline metabolism in cancer | 22/98 | 0.008498157 | 0.025494471 | 0 | 0 |
| Calcium signaling pathway | 45/240 | 0.009883261 | 0.029364689 | 0 | 0 |
| Oxytocin signaling pathway | 31/154 | 0.010982979 | 0.032310195 | 0 | 0 |
| Phospholipase D signaling pathway | 30/148 | 0.011083756 | 0.032310195 | 0 | 0 |
| Staphylococcus aureus infection | 21/95 | 0.011895661 | 0.03435289 | 0 | 0 |
| Ras signaling pathway | 43/232 | 0.013903582 | 0.039779692 | 0 | 0 |
| Type II diabetes mellitus | 17137 | 0.014619736 | 0.041444939 | 0 | 0 |
| Glioma | 17/75 | 0.017475871 | 0.048649046 | 0 | 0 |
| Thyroid hormone synthesis | 17/75 | 0.017475871 | 0.048649046 | 0 | 0 |
| Regulation of actin cytoskeleton | 40/218 | 0.020030366 | 0.055262348 | 0 | 0 |
| MicroRNAs in cancer | 54/310 | 0.021156267 | 0.057852092 | 0 | 0 |
| Endocytosis | 45/252 | 0.02239656 | 0.060706466 | 0 | 0 |
| Antigen processing and presentati | 17/78 | 0.025187069 | 0.067676559 | 0 | 0 |
| Pancreatic secretion | 21/102 | 0.025544643 | 0.068045644 | 0 | 0 |
| Thyroid hormone signaling pathwa | 24/121 | 0.027215693 | 0.071877344 | 0 | 0 |
| Breast cancer | 28/147 | 0.029768873 | 0.077954082 | 0 | 0 |
| Cell adhesion molecules | 28/148 | 0.032221539 | 0.083667693 | 0 | 0 |
| Intestinal immune network for IgA | 17838 | 0.046439139 | 0.119580783 | 0 | 0 |
| Mineral absorption | 13/60 | 0.048392494 | 0.123525522 | 0 | 0 |
| Thyroid cancer | 13759 | 0.048770595 | 0.123525522 | 0 | 0 |
| Phosphatidylinositol signaling syst | 19/97 | 0.050909266 | 0.126919854 | 0 | 0 |
| Systemic lupus erythematosus | 25/135 | 0.051325159 | 0.126919854 | 0 | 0 |
| Type I diabetes mellitus | 15980 | 0.051342983 | 0.126919854 | 0 | 0 |
| Inositol phosphate metabolism | 15/73 | 0.054017501 | 0.132471491 | 0 | 0 |
| Allograft rejection | 14124 | 0.056675255 | 0.136817608 | 0 | 0 |
| Primary immunodeficiency | 14124 | 0.056675255 | 0.136817608 | 0 | 0 |
| Signaling pathways regulating pluri | 26/143 | 0.057525927 | 0.137794664 | 0 | 0 |
| Autophagy | 25/137 | 0.059416554 | 0.141228577 | 0 | 0 |
| Pathways of neurodegeneration | 75/475 | 0.060334663 | 0.141390216 | 0 | 0 |
| Mitophagy | 14/68 | 0.060399704 | 0.141390216 | 0 | 0 |
| SNARE interactions in vesicular tra | 12267 | 0.062395717 | 0.144964486 | 0 | 0 |
| Nitrogen metabolism | 44698 | 0.064226289 | 0.148103906 | 0 | 0 |
| Cholinergic synapse | 21/113 | 0.067373637 | 0.154210769 | 0 | 0 |
| Chemical carcinogenesis | 40/239 | 0.070615443 | 0.159576803 | 0 | 0 |
| Vascular smooth muscle contractic | 24/133 | 0.07075088 | 0.159576803 | 0 | 0 |
| Longevity regulating pathway | 19/102 | 0.077421024 | 0.173355771 | 0 | 0 |

| | | | | | |
|---|---|---|---|---|---|
| Glycosaminoglycan biosynthesis | 19664 | 0.085155566 | 0.18930266 | 0 | 0 |
| cAMP signaling pathway | 36/216 | 0.08654631 | 0.19102007 | 0 | 0 |
| Viral myocarditis | 22251 | 0.09317742 | 0.203097441 | 0 | 0 |
| Glycerophospholipid metabolism | 18/98 | 0.093332805 | 0.203097441 | 0 | 0 |
| Tryptophan metabolism | 15585 | 0.096204173 | 0.207881745 | 0 | 0 |
| Wnt signaling pathway | 28/166 | 0.106396229 | 0.228308575 | 0 | 0 |
| Circadian rhythm | 44773 | 0.107352498 | 0.228771874 | 0 | 0 |
| Aldosterone-regulated sodium rea | 13728 | 0.108430678 | 0.229486845 | 0 | 0 |
| Ubiquitin mediated proteolysis | 24/140 | 0.11123807 | 0.233826963 | 0 | 0 |
| cGMP-PKG signaling pathway | 28/167 | 0.112397595 | 0.234667952 | 0 | 0 |
| Arginine and proline metabolism | 18537 | 0.118486103 | 0.244081373 | 0 | 0 |
| Vibrio cholerae infection | 18537 | 0.118486103 | 0.244081373 | 0 | 0 |
| Herpes simplex virus 1 infection | 75/498 | 0.130178799 | 0.266392377 | 0 | 0 |
| Neomycin, kanamycin and gentam | 44597 | 0.13373725 | 0.27094079 | 0 | 0 |
| ABC transporters | 16681 | 0.134155149 | 0.27094079 | 0 | 0 |
| Bacterial invasion of epithelial cells | 14/77 | 0.135513727 | 0.271023302 | 0 | 0 |
| Pantothenate and CoA biosynthesi | 44702 | 0.1359502 | 0.271023302 | 0 | 0 |
| Estrogen signaling pathway | 23/137 | 0.137930805 | 0.273209095 | 0 | 0 |
| Arginine biosynthesis | 44703 | 0.157821668 | 0.308651237 | 0 | 0 |
| Histidine metabolism | 44703 | 0.157821668 | 0.308651237 | 0 | 0 |
| Salivary secretion | 16/93 | 0.165180845 | 0.32101183 | 0 | 0 |
| Hepatocellular carcinoma | 27/168 | 0.167102137 | 0.322716003 | 0 | 0 |
| Axon guidance | 29/182 | 0.168594139 | 0.323575087 | 0 | 0 |
| Glucagon signaling pathway | 18/107 | 0.171065251 | 0.326291127 | 0 | 0 |
| Tight junction | 27/169 | 0.175101776 | 0.331941403 | 0 | 0 |
| Proximal tubule bicarbonate reclar | 44704 | 0.181010279 | 0.341049856 | 0 | 0 |
| Vitamin B6 metabolism | 44598 | 0.183530814 | 0.343703161 | 0 | 0 |
| Graft-versus-host disease | 15554 | 0.18580794 | 0.345871406 | 0 | 0 |
| Spinocerebellar ataxia | 23/143 | 0.189050741 | 0.349800472 | 0 | 0 |
| Cocaine addiction | 18142 | 0.194906332 | 0.358488431 | 0 | 0 |
| Hypertrophic cardiomyopathy | 15/90 | 0.208343277 | 0.37953648 | 0 | 0 |
| Fatty acid biosynthesis | 44669 | 0.208806477 | 0.37953648 | 0 | 0 |
| Central carbon metabolism in canc | 25903 | 0.212110295 | 0.383287024 | 0 | 0 |
| Asthma | 11475 | 0.221713185 | 0.398310316 | 0 | 0 |
| Endometrial cancer | 21459 | 0.234322679 | 0.418530104 | 0 | 0 |
| Glycosphingolipid biosynthesis | 16650 | 0.240972983 | 0.426426658 | 0 | 0 |
| Melanoma | 26634 | 0.241503771 | 0.426426658 | 0 | 0 |
| RNA degradation | 13/79 | 0.243887078 | 0.428188109 | 0 | 0 |
| Lysosome | 20/128 | 0.249242688 | 0.43511859 | 0 | 0 |
| Long-term depression | 22190 | 0.268392537 | 0.465917382 | 0 | 0 |
| Long-term potentiation | 24777 | 0.270717586 | 0.467328124 | 0 | 0 |
| Carbohydrate digestion and absorp | 17380 | 0.280438842 | 0.481420012 | 0 | 0 |
| Arachidonic acid metabolism | 22555 | 0.285979993 | 0.488219988 | 0 | 0 |
| Amphetamine addiction | 25508 | 0.304331178 | 0.516694142 | 0 | 0 |
| Nicotinate and nicotinamide metal | 12936 | 0.316671621 | 0.53448409 | 0 | 0 |
| Morphine addiction | 14/91 | 0.318268843 | 0.53448409 | 0 | 0 |
| Pyrimidine metabolism | 20699 | 0.322075196 | 0.537952624 | 0 | 0 |

| | | | | | |
|---|---|---|---|---|---|
| Serotonergic synapse | 17/113 | 0.327218677 | 0.542547848 | 0 | 0 |
| Gastric cancer | 22/149 | 0.328338018 | 0.542547848 | 0 | 0 |
| Synaptic vesicle cycle | 28825 | 0.3372244 | 0.553848417 | 0 | 0 |
| Alzheimer disease | 52/369 | 0.340659772 | 0.553848417 | 0 | 0 |
| Mucin type O-glycan biosynthesis | 13302 | 0.341434435 | 0.553848417 | 0 | 0 |
| Cholesterol metabolism | 18476 | 0.342346433 | 0.553848417 | 0 | 0 |
| Progesterone-mediated oocyte ma | 15/100 | 0.346117809 | 0.557033348 | 0 | 0 |
| Purine metabolism | 19/129 | 0.34877101 | 0.558395037 | 0 | 0 |
| TGF-beta signaling pathway | 14/94 | 0.364172339 | 0.577807777 | 0 | 0 |
| Protein export | 44674 | 0.364639119 | 0.577807777 | 0 | 0 |
| beta-Alanine metabolism | 44711 | 0.366505904 | 0.577807777 | 0 | 0 |
| Gap junction | 13/88 | 0.383648262 | 0.601763009 | 0 | 0 |
| Dopaminergic synapse | 19/132 | 0.388247557 | 0.605901491 | 0 | 0 |
| Sulfur metabolism | 44602 | 0.390542122 | 0.606419677 | 0 | 0 |
| RNA polymerase | 44712 | 0.39435065 | 0.609271754 | 0 | 0 |
| Phenylalanine metabolism | 44637 | 0.39662939 | 0.60974369 | 0 | 0 |
| GABAergic synapse | 13/89 | 0.399919616 | 0.611758224 | 0 | 0 |
| Peroxisome | 30286 | 0.404736581 | 0.614795261 | 0 | 0 |
| Endocrine and other factor-regulat | 19572 | 0.40588425 | 0.614795261 | 0 | 0 |
| Hippo signaling pathway | 23/163 | 0.408934683 | 0.616394229 | 0 | 0 |
| Proteasome | 16984 | 0.410992217 | 0.616488326 | 0 | 0 |
| Adrenergic signaling in cardiomyoc | 21/150 | 0.430893645 | 0.64321805 | 0 | 0 |
| Other types of O-glycan biosynthe: | 17349 | 0.43383905 | 0.644501281 | 0 | 0 |
| Taurine and hypotaurine metaboli: | 44603 | 0.439440316 | 0.649260495 | 0 | 0 |
| Glycine, serine and threonine meta | 14763 | 0.441244997 | 0.649260495 | 0 | 0 |
| Glutamatergic synapse | 16/114 | 0.445160893 | 0.651918085 | 0 | 0 |
| Regulation of lipolysis in adipocyte | 20302 | 0.448286414 | 0.652193711 | 0 | 0 |
| Fructose and mannose metabolism | 12175 | 0.449570422 | 0.652193711 | 0 | 0 |
| Folate biosynthesis | 44677 | 0.459199858 | 0.658941586 | 0 | 0 |
| Maturity onset diabetes of the you | 44677 | 0.459199858 | 0.658941586 | 0 | 0 |
| Basal cell carcinoma | 23255 | 0.460619361 | 0.658941586 | 0 | 0 |
| Glycosaminoglycan degradation | 44639 | 0.470849586 | 0.670472452 | 0 | 0 |
| Melanogenesis | 14/101 | 0.473686597 | 0.671418157 | 0 | 0 |
| Sphingolipid metabolism | 18080 | 0.479075535 | 0.675955892 | 0 | 0 |
| Collecting duct acid secretion | 44678 | 0.489719421 | 0.687833187 | 0 | 0 |
| Cortisol synthesis and secretion | 23986 | 0.499787379 | 0.698797738 | 0 | 0 |
| Alcoholism | 25/186 | 0.504506854 | 0.702218999 | 0 | 0 |
| D-Glutamine and D-glutamate met | 44566 | 0.509160816 | 0.705518799 | 0 | 0 |
| Dilated cardiomyopathy | 13/96 | 0.513463217 | 0.70830417 | 0 | 0 |
| Phototransduction | 44679 | 0.519500176 | 0.713446909 | 0 | 0 |
| Ovarian steroidogenesis | 18810 | 0.523272471 | 0.715447759 | 0 | 0 |
| Starch and sucrose metabolism | 13271 | 0.529494126 | 0.71933336 | 0 | 0 |
| Parkinson disease | 33/249 | 0.530770246 | 0.71933336 | 0 | 0 |
| Bile secretion | 33208 | 0.539322173 | 0.727731666 | 0 | 0 |
| Prion disease | 36/273 | 0.542584218 | 0.728950101 | 0 | 0 |
| Alanine, aspartate and glutamate r | 13636 | 0.55498094 | 0.742377102 | 0 | 0 |
| Glycerolipid metabolism | 22494 | 0.570799254 | 0.759674351 | 0 | 0 |

| | | | | | |
|---|---|---|---|---|---|
| Caffeine metabolism | 44567 | 0.574286772 | 0.759674351 | 0 | 0 |
| Renin secretion | 25447 | 0.575287372 | 0.759674351 | 0 | 0 |
| Arrhythmogenic right ventricular c | 28399 | 0.579506883 | 0.761989902 | 0 | 0 |
| Cushing syndrome | 20/155 | 0.58893817 | 0.771109722 | 0 | 0 |
| Insulin secretion | 31717 | 0.599743958 | 0.781944655 | 0 | 0 |
| Galactose metabolism | 11414 | 0.603392107 | 0.783395635 | 0 | 0 |
| ECM-receptor interaction | 32448 | 0.63130875 | 0.816210894 | 0 | 0 |
| Glutathione metabolism | 21002 | 0.645576197 | 0.830334537 | 0 | 0 |
| Circadian entrainment | 35765 | 0.647607195 | 0.830334537 | 0 | 0 |
| Amyotrophic lateral sclerosis | 46/364 | 0.662427986 | 0.84582747 | 0 | 0 |
| Glycolysis / Gluconeogenesis | 24685 | 0.679402154 | 0.857365159 | 0 | 0 |
| Riboflavin metabolism | 44569 | 0.679769188 | 0.857365159 | 0 | 0 |
| Selenocompound metabolism | 44609 | 0.679787909 | 0.857365159 | 0 | 0 |
| Cell cycle | 15/124 | 0.689072515 | 0.865542306 | 0 | 0 |
| Biosynthesis of unsaturated fatty a | 44647 | 0.71417653 | 0.889840919 | 0 | 0 |
| Fatty acid elongation | 44647 | 0.71417653 | 0.889840919 | 0 | 0 |
| Autoimmune thyroid disease | 19511 | 0.721833756 | 0.895769601 | 0 | 0 |
| Protein digestion and absorption | 12/103 | 0.728372401 | 0.900268287 | 0 | 0 |
| Thermogenesis | 28/232 | 0.733516171 | 0.903013931 | 0 | 0 |
| Lysine degradation | 23193 | 0.747299175 | 0.916331131 | 0 | 0 |
| GnRH secretion | 23559 | 0.762041721 | 0.926382381 | 0 | 0 |
| One carbon pool by folate | 44612 | 0.764490315 | 0.926382381 | 0 | 0 |
| Steroid biosynthesis | 44612 | 0.764490315 | 0.926382381 | 0 | 0 |
| Pentose phosphate pathway | 44650 | 0.779684168 | 0.940497054 | 0 | 0 |
| PPAR signaling pathway | 27242 | 0.782225705 | 0.940497054 | 0 | 0 |
| Ether lipid metabolism | 18019 | 0.796336069 | 0.953751339 | 0 | 0 |
| Cysteine and methionine metaboli | 18384 | 0.81074202 | 0.967255923 | 0 | 0 |
| Mannose type O-glycan biosynthes | 44615 | 0.829011825 | 0.985248668 | 0 | 0 |
| Fatty acid degradation | 15797 | 0.840055253 | 0.990393154 | 0 | 0 |
| Non-homologous end-joining | 44574 | 0.842865744 | 0.990393154 | 0 | 0 |
| Retrograde endocannabinoid signa | 16/148 | 0.842955985 | 0.990393154 | 0 | 0 |
| Vasopressin-regulated water reabs | 16163 | 0.852937299 | 0.994942981 | 0 | 0 |
| mRNA surveillance pathway | 36069 | 0.853268252 | 0.994942981 | 0 | 0 |
| alpha-Linolenic acid metabolism | 44617 | 0.862690762 | 0.999671977 | 0 | 0 |
| Ribosome biogenesis in eukaryote: | 11/108 | 0.863794233 | 0.999671977 | 0 | 0 |
| RNA transport | 20/186 | 0.871216099 | 0.999994349 | 0 | 0 |
| Tyrosine metabolism | 13210 | 0.873699938 | 0.999994349 | 0 | 0 |
| Hedgehog signaling pathway | 20576 | 0.880788178 | 0.999994349 | 0 | 0 |
| Thiamine metabolism | 44576 | 0.881812547 | 0.999994349 | 0 | 0 |
| AMPK signaling pathway | 12/120 | 0.886925275 | 0.999994349 | 0 | 0 |
| Primary bile acid biosynthesis | 44578 | 0.911108712 | 0.999994349 | 0 | 0 |
| Linoleic acid metabolism | 47150 | 0.912485033 | 0.999994349 | 0 | 0 |
| Nicotine addiction | 14671 | 0.914714921 | 0.999994349 | 0 | 0 |
| Drug metabolism | 10/108 | 0.920966686 | 0.999994349 | 0 | 0 |
| Glyoxylate and dicarboxylate meta | 10990 | 0.921970773 | 0.999994349 | 0 | 0 |
| Other glycan degradation | 44579 | 0.922909932 | 0.999994349 | 0 | 0 |
| Fat digestion and absorption | 15766 | 0.937069307 | 0.999994349 | 0 | 0 |

| | | | | | |
|---|---|---|---|---|---|
| Diabetic cardiomyopathy | 20/203 | 0.94363422 | 0.999994349 | 0 | 0 |
| Spliceosome | 14/150 | 0.944704931 | 0.999994349 | 0 | 0 |
| Base excision repair | 12086 | 0.944934639 | 0.999994349 | 0 | 0 |
| Basal transcription factors | 16497 | 0.948817998 | 0.999994349 | 0 | 0 |
| Terpenoid backbone biosynthesis | 44583 | 0.956396126 | 0.999994349 | 0 | 0 |
| Pyruvate metabolism | 17227 | 0.958495182 | 0.999994349 | 0 | 0 |
| Amino sugar and nucleotide sugar | 17593 | 0.962662976 | 0.999994349 | 0 | 0 |
| Oocyte meiosis | 11/129 | 0.964092285 | 0.999994349 | 0 | 0 |
| Vitamin digestion and absorption | 44585 | 0.967207762 | 0.999994349 | 0 | 0 |
| Various types of N-glycan biosynth | 14277 | 0.973031225 | 0.999994349 | 0 | 0 |
| Neuroactive ligand-receptor intera | 34/341 | 0.974180439 | 0.999994349 | 0 | 0 |
| Glycosylphosphatidylinositol (GPI)- | 44587 | 0.975339247 | 0.999994349 | 0 | 0 |
| Taste transduction | 31564 | 0.978229476 | 0.999994349 | 0 | 0 |
| Cardiac muscle contraction | 31929 | 0.980089605 | 0.999994349 | 0 | 0 |
| Aminoacyl-tRNA biosynthesis | 24198 | 0.981389503 | 0.999994349 | 0 | 0 |
| Porphyrin and chlorophyll metabo | 15738 | 0.983412216 | 0.999994349 | 0 | 0 |
| Ascorbate and aldarate metabolisr | 44591 | 0.986054023 | 0.999994349 | 0 | 0 |
| Citrate cycle (TCA cycle) | 44591 | 0.986054023 | 0.999994349 | 0 | 0 |
| Nucleotide excision repair | 17199 | 0.9898646 | 0.999994349 | 0 | 0 |
| Valine, leucine and isoleucine degr | 17564 | 0.991047253 | 0.999994349 | 0 | 0 |
| Metabolism of xenobiotics by cyto | 27851 | 0.993428764 | 0.999994349 | 0 | 0 |
| DNA replication | 13150 | 0.994069791 | 0.999994349 | 0 | 0 |
| Huntington disease | 26/306 | 0.996537216 | 0.999994349 | 0 | 0 |
| Oxidative phosphorylation | 8/133 | 0.99784414 | 0.999994349 | 0 | 0 |
| Steroid hormone biosynthesis | 22313 | 0.998262528 | 0.999994349 | 0 | 0 |
| N-Glycan biosynthesis | 18264 | 0.999192586 | 0.999994349 | 0 | 0 |
| Retinol metabolism | 24869 | 0.999292156 | 0.999994349 | 0 | 0 |
| Fanconi anemia pathway | 19725 | 0.9995425 | 0.999994349 | 0 | 0 |
| Ribosome | 7/158 | 0.999941635 | 0.999994349 | 0 | 0 |
| Olfactory transduction | 5/440 | 0.999994349 | 0.999994349 | 0 | 0 |

| Odds Ratio | Combined Score | Genes |
|---|---|---|
| 6.903235747 | 332.5341705 | CSF1;TNFAIP3;TNF;CXCL2;ICAM1;IKBKB;CASP7;CASP8;CASP3;MAP3K8;JUNB;M |
| 4.297106725 | 205.2890892 | ERO1A;CD40;CXCL8;NCF1;NCF4;FASLG;TNF;CXCL2;ICAM1;IKBKB;PYCARD;CASP |
| 4.516390324 | 198.0833205 | CXCL8;TNFAIP3;ANTXR2;TNF;CXCL2;PYCARD;IKBKB;PANX1;PSTPIP1;CASP8;NA |
| 6.649365629 | 286.3163338 | CD40;CXCL8;TNFAIP3;TNF;CXCL2;ICAM1;TNFSF13B;IKBKB;PLAU;TRIM25;LBP;E |
| 4.705027069 | 197.4828464 | CDKN1A;CXCL8;DDX3X;FASLG;TNF;IFIH1;IKBKB;CASP8;CASP3;JAK2;IKBKE;JAK3 |
| 6.395617305 | 263.3543186 | LSP1;TNF;PYCARD;IKBKB;CASP8;IL12B;IL12A;IKBKE;IL10;FCER1G;KSR1;PRKCD; |
| 8.190979341 | 326.841345 | CXCL8;ITGAM;C1S;C1R;LY96;NOD1;TNF;RELA;C2;PYCARD;C3;C4A;CASP7;MAP |
| 4.361419432 | 173.1518025 | CIITA;CXCL8;CALCOCO2;ADAR;FASLG;TNF;ACTB;ACTG1;ICAM1;PYCARD;IFIH1; |
| 5.916129032 | 222.0222883 | CD40;CXCL8;CD80;TNF;IKBKB;CASP8;IL12B;IL12A;MAP3K8;LBP;IKBKE;IFNAR2; |
| 4.764280787 | 176.4712071 | CDKN1B;TNFAIP3;ADAR;FASLG;IFIH1;IKBKB;CCND2;CASP8;CASP3;IL12B;IL12A; |
| 4.0480105 | 149.3806289 | CIITA;ITGAM;LSP1;TCIRG1;TNF;FCGR3A;CASP8;LAMP1;CASP3;LAMP2;IL12B;IL |
| 3.776557899 | 138.4895053 | CD40;CDKN1A;CDKN1B;TNFAIP3;RBPJ;ITGAL;TNF;ICAM1;IKBKB;CCND2;CASP8; |
| 3.826247279 | 136.8244908 | CXCL8;NCF1;CXCL13;CXCL2;PIK3CG;CRKL;CXCL16;IKBKB;PREX1;RAC2;CCR6;CC |
| 3.449086135 | 122.8590688 | NRP1;CSF3;CXCL8;RPLP1;F13A1;CGAS;ADAR;TNF;IFIH1;IKBKB;C4A;C3AR1;IL12 |
| 4.91651689 | 174.9307634 | CSF1;NCF1;NCF4;TNF;SIRPB1;IKBKB;FCGR3A;SIRPA;JUNB;JAK1;IFNAR2;IL1R1;I |
| 4.255412272 | 151.3010884 | H2AW;TNFAIP3;FASLG;PYGM;TNF;PYCARD;CASP8;FTH1;CAPN2;TNFSF10;JAK2; |
| 3.79491925 | 134.5140861 | CDKN1A;CXCL8;CXCL2;PIK3CG;ICAM1;IKBKB;PREX1;ZFP36;CASP8;CASP3;CCR5; |
| 3.335337471 | 108.7352046 | CDKN1A;CXCL8;CGAS;FASLG;TNF;CRKL;IKBKB;CASP8;CASP3;RAC2;ITGAV;CCR5 |
| 4.975911708 | 160.1119006 | CD40;CIITA;LY96;XIAP;TNF;PIK3R6;PIK3CG;RELA;PIK3R5;IKBKB;MAPK8;SOCS1; |
| 3.912116331 | 124.0213913 | CSF3;CDKN1A;IL23R;IL27RA;CCND2;PIM1;IL12B;IL12A;JAK2;IL6R;JAK3;IL13RA1 |
| 2.294976172 | 72.50532224 | IL23R;ITGA2B;CRKL;FRAT1;RASSF1;FRAT2;CCND2;RASSF5;PIM1;IL12B;IL12A;IL |
| 6.449026818 | 202.1610444 | ITGAM;NCF1;NCF4;PTGS2;TNF;RELA;C3;FCGR3A;IL12B;IL12A;JAK2;JAK1;IL10;J |
| 2.793732013 | 82.3263216 | ACVRL1;IL1RN;CD40;CSF3;CXCL8;CSF1;IL23R;IL27;FASLG;IL1RAP;CXCL13;TNF;C |
| 2.979871716 | 85.0218662 | CXCL8;CALCOCO2;UBE2D1;CGAS;TNF;ACTB;HK2;CRKL;ACTG1;IKBKB;PYCARD;H |
| 4.626816818 | 128.9868547 | IL23R;IL1RAP;IL2RG;HIF1A;RELA;IL27RA;IKBKB;MAPK8;TBX21;IL21R;STAT6;JAK |
| 4.79495239 | 132.0219491 | CCL14;CXCL8;CSF1;IL20RA;CXCR4;CXCL13;IL2RG;TNF;CXCL2;IL18RAP;CCL8;CXC |
| 3.933964035 | 107.8667528 | CXCL8;TNF;ACTB;ACTG1;CRKL;PYCARD;IKBKB;RAC2;MAP2K3;IL10;IL18;RHOG;T |
| 4.90226928 | 131.4114428 | CSF3;CXCL8;TRAF3IP2;TNFAIP3;PTGS2;CXCL2;TNF;RELA;HSP90B1;IKBKB;MAPK |
| 2.760489341 | 68.09880583 | CXCL8;TNF;ACTB;PIK3CG;ACTG1;IKBKB;PYCARD;CASP7;CASP8;KIF5C;CASP3;TU |
| 2.57786413 | 63.58813302 | FLT1;CSF1;FLT4;FASLG;IL1RAP;AREG;TNF;DUSP16;CRKL;ELK4;IKBKB;DUSP10;C |
| 3.602657469 | 88.35297598 | FASLG;TNF;ACTB;ACTG1;LMNB1;IKBKB;CASP7;CASP8;CASP6;CASP3;CAPN2;TN |
| 4.653593938 | 113.812853 | RBPJL;NOTCH3;MAML2;RBPJ;IL2RG;DLL1;RELA;IKBKB;MAPK8;TBX21;STAT4;IL1 |
| 4.26774317 | 101.2442283 | CXCL8;SERPINE1;FASLG;TNF;RELA;C3;IKBKB;MAPK8;CASP8;CCL3;IL12B;CCL2;IL |
| 5.657208213 | 129.7983209 | IL23R;NOD2;IL2RG;TNF;RELA;IL18RAP;TBX21;IL21R;STAT4;IL12B;IL12A;STAT6; |
| 4.488677082 | 101.189453 | CD274;HIF1A;RELA;IKBKB;JAK2;JAK1;MAP2K3;BATF2;BATF3;JUN;TICAM2;IFNG |
| 4.051255166 | 87.2498826 | CDKN1B;CXCL8;SERPINE1;TNF;RELA;ICAM1;MAPK8;CASP3;PIM1;PLCG2;CCL2;J |
| 5.934996192 | 127.8026677 | CXCL8;ITGAM;NLRC4;CXCL2;TNF;RELA;PYCARD;C3;CASP7;CASP8;CASP3;IL12B; |
| 4.948627526 | 102.4763668 | CXCL8;DDX3X;TNF;TANK;RELA;IFIH1;IKBKB;MAPK8;CASP8;DHX58;IL12B;TRIM2 |
| 3.094919234 | 63.38833755 | CDKN1A;CD81;FASLG;TNF;IKBKB;CASP8;CASP3;IKBKE;JAK1;IFNAR2;RSAD2;DD |
| 2.766981328 | 55.63967986 | CXCL8;FASLG;TNF;ACTB;ACTG1;IKBKB;PYCARD;CASP7;CASP8;CASP3;TUBB1;CA |
| 3.345801227 | 67.14010984 | CDKN1A;PRKAA1;CDKN1B;FBXO25;AGAP2;PRKAG2;FASLG;NLK;STK4;IKBKB;MA |
| 2.717213334 | 54.03042628 | CDKN1A;CDKN1B;DDX3X;GTF2B;HDAC11;RBPJ;POLB;CCND2;CASP8;CASP3;CC |
| 2.560285298 | 47.8901587 | NRP1;CD40;CDKN1A;CRTC2;SLC2A1;ITGAL;TNF;ETS2;ICAM1;ELK4;IKBKB;POLB; |
| 2.96054039 | 50.0021337 | PRKAA1;NCF1;SDC4;SRC;ITGA2B;TNF;ACTB;RELA;ACTG1;ICAM1;HSP90B1;IKBK |

| | | |
|---|---|---|
| 5.172096433 | 83.56035219 | IL10;CD40;CSF3;CXCL8;GYPC;CD81;IL18;ITGAL;TNF;SELE;ICAM1;SELP;IL6;IFNG; |
| 2.376252678 | 36.45869742 | CGAS;FASLG;TNF;CRKL;IKBKB;CASP8;CASP3;RAC2;CCR5;B2M;MAP2K3;PDIA3;F |
| 2.391793547 | 34.1981952 | CD40;CDKN1A;CDKN1B;CXCL8;FLT1;ITGAM;CCNT1;JMJD1C;ELK4;HHEX;CCND2 |
| 5.587234446 | 75.67836758 | IL10;PRKCB;IL18;FASLG;TNF;SELE;ICAM1;IL6;IFNG;IL1B;TLR9;IL12B;FAS;IL12A;I |
| 3.657618806 | 48.81197434 | CXCL8;SRC;NOD1;TCIRG1;CXCL2;RELA;IKBKB;MAPK8;PAK1;CXCR1;CASP3;CXCR |
| 3.137810214 | 41.48071425 | CXCL8;FLT1;CSF1;CD80;TCIRG1;ITGAL;CXCL2;TNF;ICAM1;TNFSF13B;CCL3;CCL2 |
| 3.78384981 | 48.54392195 | ZBP1;RIPK3;DDX58;TREX1;IL18;CGAS;ADAR;RELA;NFKB1;IKBKB;PYCARD;NFKBI |
| 2.747485358 | 35.00245914 | FASLG;PSEN1;RELA;CRKL;RAP1B;IKBKB;MAPK8;IRAK2;ARHGDIB;PLCG2;SH2B2; |
| 2.738322342 | 32.13789536 | CDKN1A;PFKFB3;CDKN1B;FLT1;SERPINE1;SLC2A1;ENO1;ENO3;HIF1A;HK2;REL/ |
| 2.636426117 | 30.89892338 | ITPR1;ADCY4;ITPR2;ADCY8;ADCY7;CRKL;SOCS3;MAPK8;SOCS1;PLCG2;JAK2;JUI |
| 3.226191542 | 37.58787459 | CDKN1A;CDKN1B;CTBP2;RELA;CRKL;IKBKB;E2F2;E2F3;BAK1;STAT5A;STAT5B;SI |
| 3.290034234 | 37.82862754 | RET;STAT5A;STAT5B;CDKN1A;GADD45B;PRKCB;STAT3;TGFA;BRAF;STK4;GADD |
| 2.118805563 | 24.34101572 | DOCK4;FLT1;ITGAM;CSF1;FLT4;ITGA2B;ITGAL;ACTB;CRKL;ACTG1;SIPA1L1;RASS |
| 3.634308511 | 41.36191225 | TLE4;RBPJL;TLE3;NOTCH3;CREBBP;MAML2;DTX3L;CTBP2;NOTCH4;PSEN1;RBPJ |
| 2.808302322 | 31.33950978 | NCF1;CRKL;FCGR3A;PAK1;INPP5D;PLCG2;RAC2;PIP5K1B;LYN;VASP;MARCKSL1; |
| 2.744154276 | 30.55993772 | SERPINB10;CXCL8;ITGAM;CD1D;TNF;CXCL2;RELA;CASP3;IL12B;IL12A;CD14;IL1 |
| 2.489236774 | 26.78325742 | ROCK1;SRC;ITGA2B;ITPR1;ADCY4;ITPR2;RASGRP2;ADCY8;ADCY7;ACTB;ARHGA |
| 2.900053551 | 30.73357056 | CFD;CPB2;ITGAM;C1S;C1R;SERPINE1;C5AR1;F13A1;C2;C3;C4A;C7;PLAU;C3AR1 |
| 2.122805465 | 22.44006512 | HDAC4;H2AW;ITGAM;SELPLG;NCF1;SRC;HDAC11;ITGA2B;NCF4;C5AR1;ITGAL;/ |
| 3.03625245 | 31.80461572 | CDKN1A;SMAD3;GADD45B;STAT1;STAT3;TGFA;BRAF;TGFBR1;RELA;NFKB1;GAI |
| 2.937289356 | 30.59088823 | CD81;DAPP1;MALT1;RELA;IKBKB;INPP5D;RAC2;PLCG2;LYN;JUN;CD72;PRKCB;F |
| 3.144960587 | 31.87702283 | MAP2K3;LYN;FCER1G;PLA2G4A;GAB2;MAPK14;TNF;VAV1;MAPK13;MAPK11;N |
| 2.733692659 | 27.68623255 | CDKN1A;CDKN1B;MAX;ITGA2B;XIAP;PTGS2;RELA;IKBKB;CASP3;E2F2;ITGAV;E2 |
| 1.774081342 | 17.84272792 | CDKN1A;CDKN1B;WNT2B;MAML2;ITGA2B;FASLG;TCIRG1;RBPJ;TNF;IKBKB;LFN |
| 2.017482304 | 19.97800628 | CDKN1A;DDX5;WNT2B;SDC4;ROCK1;SRC;PXN;ITPR1;ITPR2;FASLG;HIF1A;TNF;A |
| 3.07786862 | 30.42323038 | PRKAA1;STAT3;SLC2A1;ACSL6;PRKAG2;ACSL5;ACSL4;TRAF2;TNFRSF1B;TNF;PR |
| 2.459191457 | 24.02439151 | ITGAM;NCF1;ROCK1;NCF4;PXN;CXCR4;ITGAL;ACTB;ARHGAP35;ACTG1;ICAM1; |
| 2.545162602 | 24.75577399 | TNF;MALT1;RELA;IKBKB;MAPK8;PAK1;MAP3K8;FYN;IL10;JUN;FOS;MAPK14;NF |
| 3.013572153 | 29.04999916 | STAT5A;STAT5B;CISH;SRC;STAT1;STAT3;FOS;MAPK14;RELA;NFKB1;MAPK13;SC |
| 2.308664281 | 22.02257242 | FASLG;ITGAL;TNF;ICAM1;FCGR3A;PAK1;CASP3;SH3BP2;TNFSF10;PLCG2;RAC2; |
| 3.000867253 | 27.67160549 | STAT5A;STAT5B;TCF7L2;ITGAM;BCL2A1;CEBPE;STAT3;BRAF;DUSP6;RELA;NFKE |
| 2.139987907 | 19.595052 | PRKAA1;CXCL8;PRKAG2;FASLG;TNF;COX7A1;RELA;IKBKB;SOCS3;CASP7;MAPK8 |
| 2.415734023 | 21.71679581 | CRTC2;PRKAA1;SLC2A1;PRKAG2;PYGM;TNF;RELA;IKBKB;SOCS3;MAPK8;PPARG |
| 3.788053304 | 33.59956099 | STEAP3;NCOA4;SLC11A2;ACSL6;ACSL5;CYBB;SLC3A2;ACSL4;SAT1;GCLC;MAP1L |
| 2.217805326 | 17.83338273 | ASAH1;ROCK1;TNF;RELA;MAPK8;SPTLC2;PPP2R3C;RAC2;S1PR1;FCER1A;FYN;S |
| 2.417265422 | 19.25019703 | SRC;ADCY4;ITPR1;ITPR2;ADCY8;ADCY7;MAPK8;PTK2B;MAP2K3;MAP3K2;EGR1 |
| 1.846523426 | 14.07042951 | FLT1;ROCK1;SRC;FLT4;ITGA2B;PXN;XIAP;ACTB;ARHGAP35;MYLK3;CRKL;ACTG1 |
| 1.968782851 | 14.35666463 | ITGAM;NCF1;C1R;NCF4;TCIRG1;CORO1A;ACTB;FCAR;ACTG1;C3;FCGR3A;LAMP |
| 2.251047726 | 16.02124012 | SRC;PTGER2;ITPR1;ADCY4;ITPR2;ALOX12;IL1RAP;ADCY8;ADCY7;MAPK8;PLCG2 |
| 1.880797596 | 13.29820555 | ERO1A;PPP1R15A;ERO1B;NGLY1;UBE2D1;UBE2J2;HSP90B1;UBE2J1;HERPUD1; |
| 2.004847296 | 14.11803216 | RYR1;HDAC4;NOTCH3;PRKAA1;SERPINE1;PDE3B;ITPR1;ADCY4;ITPR2;PRKAG2;/ |
| 2.004847296 | 14.11803216 | PRKAA1;PDE3B;PRKAG2;PYGM;HK2;CRKL;IKBKB;LIPE;SOCS3;HK3;MAPK8;SOCS |
| 2.657209697 | 17.68873856 | PRKCB;SRC;PXN;PLA2G4A;PTGS2;MAPK14;PTK2;MAPK13;VEGFA;MAPK11;MA |
| 2.294064059 | 15.20295264 | STAT5A;STAT5B;CDKN1A;JUN;CDKN1B;PRKCB;SRC;TGFA;NRG1;BRAF;AREG;PTI |
| 2.258088655 | 14.59403667 | TCF7L2;CDKN1A;JUN;SMAD3;GADD45B;TGFA;BRAF;FOS;AREG;TGFBR1;EREG;C |
| 2.345369215 | 14.9151056 | PRKCB;ADCY4;ITPR1;KCNJ15;ATP1A3;ITPR2;KCNJ16;ATP1B3;SLC4A2;ATP1A1;A |
| 3.045819805 | 19.23787955 | CDKN1A;CXCL8;SRC;MMP1;BRAF;VEGFA;RASSF1;MDM2;E2F2;E2F3;KRAS;RAF1 |

| | | |
|---|---|---|
| 1.832917081 | 11.23610346 | CDKN1A;CXCL8;TRAF3IP2;SERPINE1;ITPR1;ITPR2;RELA;CCND2;RASSF5;CAPN2; |
| 1.511153524 | 9.07459257 | CDKN1A;CRTC2;CSF3;CDKN1B;FLT1;CSF1;FLT4;ITGA2B;FASLG;AREG;PIK3CG;IKI |
| 2.310012936 | 13.78936475 | CDKN1A;STEAP3;CD82;GADD45B;SERPINE1;SHISA5;PPM1D;GADD45G;COP1;C |
| 2.027782476 | 12.03598791 | CDKN1A;ITPR1;ADCY4;LRP5;ITPR2;ADCY8;ADCY7;CYP27B1;SLC9A3R1;MMP25; |
| 1.906108994 | 11.24326507 | SRC;ADCY4;ADCY8;ADCY7;RELA;MAPK8;GNG4;INSL3;JUN;EDN1;NOS2;MMP1;V |
| 2.31668713 | 13.31789257 | CDKN1A;CREBBP;EGLN3;JUN;SLC2A1;TGFA;BRAF;HIF1A;CRKL;VEGFA;RAP1B;FL |
| 2.041306135 | 11.5052055 | TCF7L2;CREBBP;CDKN1A;CDKN1B;IL1R2;TGFA;BRAF;NFKB1;RELA;HSP90B1;NFI |
| 2.229007196 | 12.07722616 | PTPN1;TCF7L2;CREBBP;SMAD3;SRC;ACTN1;PTPRJ;ACTN4;NLK;ACTB;TGFBR1;A |
| 1.987357414 | 10.66511914 | CSF3;IL4R;ITGAM;CSF1;IL1R1;IL1R2;ITGA2B;CD1D;TNF;FCER2;IL1A;IL6;CD5;IL1 |
| 1.723782063 | 8.775833165 | PRKAA1;SEH1L;WNT2B;LRP5;SLC3A2;TNF;IKBKB;FLCN;SESN2;DVL2;RICTOR;ATI |
| 1.90022805 | 9.060108622 | CAMK1D;KCNK9;PRKCB;PRKCE;ITPR1;ADCY4;ITPR2;ATP1A3;ATP1B3;ATP1A1;A |
| 1.90022805 | 9.060108622 | DGKG;SLC22A3;JUN;SLC44A3;LYPLA1;DGKD;CHKA;PRKCB;SLC44A2;SLC22A1;PI |
| 1.517697027 | 7.007074775 | RET;RYR1;PTGFR;FLT1;FLT4;PTAFR;ADCY4;ITPR1;CXCR4;CACNA1A;ITPR2;MST1 |
| 1.655609508 | 7.469130893 | RYR1;CDKN1A;PRKAA1;ROCK1;SRC;ITPR1;ADCY4;ITPR2;PRKAG2;ADCY8;PTGS2 |
| 1.669940617 | 7.518531311 | DGKG;PTGFR;DGKD;CXCL8;ADCY4;LPAR3;ADCY8;ADCY7;PIK3R6;PIK3CG;PIK3R! |
| 1.862384991 | 8.253311016 | IL10;CFD;ITGAM;SELPLG;C1S;C1R;C5AR1;PTAFR;ITGAL;FCAR;ICAM1;C2;C3;SELI |
| 1.495659754 | 6.394856018 | FLT1;CSF1;FLT4;TGFA;FASLG;PLA2G5;RASGRP2;STK4;RELA;ETS2;RASGRP4;IKBK |
| 2.313695793 | 9.77625063 | IKBKB;HK3;SOCS3;MAPK8;SOCS1;PKLR;PIK3CA;PRKCE;PRKCD;CACNA1A;TNF;H |
| 1.922407252 | 7.779855585 | CDKN1A;CAMK1D;GADD45B;PRKCB;TGFA;BRAF;GADD45G;PIK3CA;PLCG2;MDI |
| 1.922407252 | 7.779855585 | HSPA5;GPX3;PRKCB;ADCY4;ITPR1;ATP1A3;ITPR2;ATP1B3;ATP1A1;TTF1;ADCY8 |
| 1.476541235 | 5.774023176 | ITGAM;ROCK1;SRC;ITGA2B;PXN;CXCR4;ITGAL;ACTB;ARHGAP35;MYLK3;CRKL;A |
| 1.387123052 | 5.348495593 | CDKN1A;CDKN1B;MIR146A;CRKL;GLS;IKBKB;RASSF1;CCND2;PLAU;CASP3;PIM1 |
| 1.428714324 | 5.427468399 | WASHC4;SRC;VPS4B;AGAP2;CXCR4;IL2RG;RAB11FIP1;GRK2;CYTH4;CXCR1;GRK |
| 1.827545461 | 6.727970751 | PDIA3;CD74;CIITA;HSPA5;TAP2;TAP1;HSPA2;TNF;TAPBP;CREB1;IFNG;CANX;RF |
| 1.700748621 | 6.23720243 | CPB2;CELA2A;PRKCB;RAB3D;ITPR1;ADCY4;ITPR2;ATP1A3;ATP1B3;SLC4A2;PLA: |
| 1.623446648 | 5.850839234 | NCOA1;NOTCH3;CREBBP;PRKCB;SRC;STAT1;NOTCH4;SLC2A1;ATP1A3;ATP1B3; |
| 1.544246499 | 5.426933064 | NOTCH3;CDKN1A;WNT2B;NOTCH4;FLT4;LRP5;DLL1;FRAT1;FRAT2;DVL2;E2F2;E |
| 1.531288889 | 5.260161319 | NLGN3;CD274;CD40;ITGAM;SELPLG;SDC4;CD80;SDC3;ICAM3;NRXN2;ITGAL;VS |
| 1.947848681 | 5.979140969 | IL10;IL15RA;CD40;IL6;IL15;CD80;CXCR4;ITGB7;LTBR;ICOSLG;TNFSF13B |
| 1.812540297 | 5.48911619 | VDR;SLC11A2;ATP1A3;ATP1B3;SLC30A1;ATP1A1;ATP2B1;SLC8A1;MT2A;FTH1; |
| 2.105454398 | 6.359793915 | RET;TCF7L2;CDKN1A;GADD45B;NCOA4;BRAF;KRAS;BAK1;GADD45G |
| 1.597020229 | 4.755463636 | DGKG;DGKD;MTMR3;PRKCB;INPP1;IPMK;ITPK1;ITPR1;ITPR2;MTMR7;INPP4A;S |
| 1.490677321 | 4.426676949 | H2AW;CD40;C1S;C1R;CD80;TNF;C2;C3;C4A;FCGR3A;C7;TRIM21;ELANE;H3-3A; |
| 1.985117922 | 5.894265737 | IL1A;IFNG;CD80;IL1B;LTA;IL12B;FAS;FASLG;IL12A;TNF |
| 1.694955687 | 4.94663866 | ISYNA1;MTMR3;INPP1;IPMK;ITPK1;PIK3CG;MTMR7;INPP4A;PIK3CA;SYNJ1;INP |
| 2.032735145 | 5.8347987 | IL10;CD40;IFNG;CD80;IL12B;FAS;FASLG;IL12A;TNF |
| 2.032735145 | 5.8347987 | CD40;CIITA;PTPRC;RFX5;TAP2;TAP1;IL2RG;IL7R;JAK3 |
| 1.457513852 | 4.161959255 | WNT2B;ACVR1B;DVL2;JAK2;JARID2;SKIL;JAK3;JAK1;FZD1;SMAD3;SETDB1;PCGI |
| 1.463888209 | 4.132823445 | PRKAA1;MTMR3;ITPR1;HIF1A;TANK;MAPK8;LAMP1;ATG101;RB1CC1;TP53INP. |
| 1.232568852 | 3.460866604 | RYR1;WNT2B;CSF1;LRRK2;FASLG;UBE2L6;TNF;FRAT1;CASP7;FRAT2;CASP8;KIF! |
| 1.698889871 | 4.768394953 | GABARAPL1;USP15;JUN;CALCOCO2;SRC;HIF1A;RELA;MAPK8;FUNDC1;PGAM5; |
| 2.095667297 | 5.813923109 | STX1B;GOSR1;STX6;STX4;STX3;SNAP29;VAMP5;STX11 |
| 2.727687563 | 7.488437037 | CA12;CA5B;CA4;CA9;GLUL |
| 1.496444265 | 4.036660617 | PRKCB;KCNJ12;ITPR1;ADCY4;CACNA1A;ITPR2;FOS;ADCY8;GNG11;ADCY7;PIK3I |
| 1.319109901 | 3.496309255 | SRC;NR1I3;ADCY4;XIAP;CACNA1A;ADCY8;PTGS2;ADCY7;DLL1;RELA;HSP90B1;H |
| 1.443713555 | 3.823805719 | ARHGEF11;EDN1;PTGIR;RAMP2;ROCK1;PRKCB;PRKCE;PRKCD;ITPR1;ADCY4;ITP |
| 1.500379651 | 3.838716696 | PRKAA1;ADCY4;PRKAG2;HSPA2;ADCY8;SOD2;PRKAB1;SIRT1;ADCY7;NFKB1;REI |

| | | |
|---|---|---|
| 1.715466277 | 4.225666073 | CHST11;CHSY1;CHPF2;CSGALNACT2;CHST15;XYLT1;B3GNT2;B3GALT6;B4GALT |
| 1.31196026 | 3.210465981 | ROCK1;PTGER2;PDE3B;ADCY4;ATP1A3;ATP1A1;GLI1;ADCY8;ADCY7;RELA;RAP1 |
| 1.637542598 | 3.886297748 | CD40;CASP8;CD80;CASP3;RAC2;FYN;ITGAL;BID;ACTB;CD55;ACTG1;ICAM1 |
| 1.474411765 | 3.496690803 | DGKG;LYPLA1;DGKD;CHKA;MBOAT1;PLD4;PLA2G4A;PLA2G5;GPCPD1;CRLS1;P |
| 1.785930408 | 4.181367682 | MAOB;ALDH2;MAOA;HAAO;KYNU;KYAT3;KMO;IDO2;IDO1 |
| 1.330164251 | 2.980346257 | WNT2B;CTBP2;LRP5;PRICKLE3;PSEN1;NLK;FRAT1;MAPK8;CCND2;FRAT2;DVL2; |
| 1.909501764 | 4.26131572 | PRKAA1;CREB1;BHLHE40;CRY2;PRKAG2;FBXL3;PRKAB1 |
| 1.806192556 | 4.012717262 | PIK3CA;PRKCB;ATP1A3;SFN;ATP1B3;KRAS;ATP1A1;SGK1 |
| 1.356042025 | 2.977980299 | MAP3K1;UBA7;UBE2B;SMURF1;UBE2D1;XIAP;UBE2L6;UBE2G1;UBE2J2;PIAS2; |
| 1.320517986 | 2.886272979 | ROCK1;PDE3B;ITPR1;ADCY4;ITPR2;ATP1A3;ATP1A1;ADCY8;ADCY7;MYLK3;SLC |
| 1.637060159 | 3.491783175 | ARG2;ALDH2;MAOB;NOS2;MAOA;ODC1;LAP3;PRODH;SAT1;AGMAT |
| 1.637060159 | 3.491783175 | ERO1A;ATP6V1G1;SEC61G;PLCG2;TCIRG1;ATP6V1E1;ATP6V0D1;ACTB;CFTR;A |
| 1.16396789 | 2.373151737 | ZNF331;CGAS;FASLG;TNF;IFIH1;IKBKB;CASP8;CASP3;IL12B;IL12A;JAK2;IKBKE;B |
| 4.361624544 | 8.775057436 | HK3;HK2 |
| 1.636819213 | 3.287974218 | ABCA1;ABCA6;ABCA3;TAP2;TAP1;ABCA7;ABCA13;ABCG1;CFTR |
| 1.455433455 | 2.908949142 | SRC;PXN;RHOG;ACTB;PTK2;ACTG1;CRKL;DNM2;MAD2L2;PIK3CA;ELMO2;HCLS |
| 2.045293618 | 4.081315177 | ALDH2;VNN2;DPYS;DPYD;UPB1 |
| 1.321989861 | 2.618866054 | NCOA1;JUN;SRC;PRKCD;ITPR1;ADCY4;ITPR2;TGFA;HSPA2;FOS;ADCY8;ADCY7;H |
| 1.924871157 | 3.553869531 | ARG2;NOS2;GLUL;ASS1;GLS |
| 1.924871157 | 3.553869531 | HAL;MAOB;ALDH2;MAOA;ALDH3B1 |
| 1.360853784 | 2.450508975 | PRKCB;ADCY4;ITPR1;ATP1A3;ITPR2;ATP1B3;SLC4A2;ATP1A1;ATP2B1;ADCY8;L |
| 1.254670966 | 2.244794625 | CDKN1A;WNT2B;LRP5;TGFA;ACTB;ACTG1;FRAT1;FRAT2;DVL2;PLCG2;E2F2;E2F |
| 1.24199446 | 2.211074296 | NRP1;SEMA7A;ROCK1;SRC;CXCR4;RND1;PAK1;PLCG2;PLXNA2;RAC2;PLXNA1;F |
| 1.324623158 | 2.338900635 | CRTC2;CREBBP;PRKAA1;PDE3B;ITPR1;SLC2A1;ITPR2;PRKAG2;PYGM;PRKAB1;S |
| 1.245762848 | 2.170602111 | PRKAA1;ROCK1;SRC;PRKAG2;ARHGAP17;CD1D;ACTB;ACTG1;SLC9A3R1;MAPK |
| 1.817828969 | 3.107035923 | CA4;ATP1A3;ATP1B3;ATP1A1;GLS |
| 3.2710298 | 5.545614628 | PDXK;PSAT1 |
| 1.540131213 | 2.592105089 | IL1A;IL6;IFNG;CD80;IL1B;FAS;FASLG;TNF |
| 1.255453105 | 2.091258243 | RYR1;RBPJL;XBP1;PRKCB;GTF2B;ITPR1;CACNA1A;ITPR2;TRAF2;RBPJ;ATXN3;M |
| 1.472796899 | 2.408370784 | JUN;CREB1;GRIN3A;MAOB;MAOA;FOSB;RELA;NFKB1;ATF4 |
| 1.309476876 | 2.054003775 | EDN1;PRKAA1;TPM4;ITGA2B;PRKAG2;TNF;PRKAB1;ACTB;SLC8A1;ACTG1;IL6;IT |
| 1.869492531 | 2.928274767 | ACSL6;ACSL5;ACSL4;ACSF3 |
| 1.354424265 | 2.100236469 | RET;HK3;SLC7A5;PIK3CA;SLC2A1;KRAS;SLC1A5;RAF1;SLC16A3;HIF1A;HK2;GLS |
| 1.570562901 | 2.365849921 | IL10;CD40;FCER1G;IL9;FCER1A;TNF |
| 1.363586203 | 1.978640134 | TCF7L2;CDKN1A;PIK3CA;GADD45B;BRAF;GRB2;KRAS;BAK1;RAF1;GADD45G |
| 1.415010474 | 2.013659601 | B3GNT3;B3GNT2;B4GALNT1;ST6GALNAC4;FUT4;B4GALT4;ST3GAL3;ST6GALN |
| 1.309125331 | 1.86009716 | CDKN1A;PIK3CA;GADD45B;FGF18;MDM2;E2F2;E2F3;BRAF;KRAS;BAK1;RAF1;G |
| 1.289330808 | 1.819310178 | BTG3;BTG2;BTG1;PNPT1;ENO1;TOB2;ENO3;TENT4B;CNOT7;PAN3;XRN1;CNOT |
| 1.212460086 | 1.684505 | CD164;NAPSA;ASAH1;SLC11A1;SLC11A2;LAPTM5;TCIRG1;LITAF;IGF2R;GALC;C |
| 1.308891411 | 1.721590997 | RYR1;LYN;PRKCB;ITPR1;PLA2G4A;CACNA1A;ITPR2;BRAF;KRAS;RAF1 |
| 1.28555883 | 1.679812879 | RAP1B;CREBBP;PRKCB;ITPR1;ITPR2;BRAF;KRAS;RAF1;ADCY8;CALM1;ATF4 |
| 1.342290728 | 1.706587908 | HK3;MGAM;PIK3CA;PRKCB;ATP1A3;ATP1B3;ATP1A1;HK2 |
| 1.283152686 | 1.606293422 | GGT5;GPX3;CYP4F2;EPHX2;TBXAS1;PLA2G4A;ALOX12;PLA2G5;GGT1;PTGS2 |
| 1.241085646 | 1.476443601 | JUN;CREB1;GRIN3A;MAOB;PRKCB;MAOA;FOSB;FOS;CALM1;SIRT1;ATF4 |
| 1.353620885 | 1.556515035 | BST1;PNP;NAPRT;NAMPT;CD38;SIRT1 |
| 1.189844638 | 1.362204145 | PRKCB;PDE3B;ADCY4;CACNA1A;ADCY8;ADCY7;GNG11;GABRR2;GRK2;GRK5;G |
| 1.2529372 | 1.419540553 | NT5C3A;ENTPD1;PNP;DPYS;DPYD;CMPK2;UPP1;DCK;UPB1 |

| | | |
|---|---|---|
| 1.158901587 | 1.294639783 | MAOB;PRKCB;MAOA;DUSP1;ITPR1;CACNA1A;ITPR2;PLA2G4A;ALOX12;BRAF;P |
| 1.133786441 | 1.262711178 | FZD1;TCF7L2;CDKN1A;SMAD3;CDKN1B;WNT2B;GADD45B;LRP5;BRAF;TGFBR1; |
| 1.189700871 | 1.293212813 | STX1B;ATP6V1G1;SLC6A9;STXBP1;SLC1A2;CACNA1A;TCIRG1;STX3;ATP6V1E1;A |
| 1.074034859 | 1.15659703 | WNT2B;CSF1;TNF;IKBKB;FRAT1;CASP7;FRAT2;CASP8;KIF5C;CASP3;RB1CC1;TUB |
| 1.308424632 | 1.406032598 | GALNT11;GALNT3;GALNT1;B3GNT3;ST6GALNAC4;B4GALT5 |
| 1.246196777 | 1.335838321 | ABCA1;MYLIP;STARD3;NPC1;APOA2;VDAC3;TSPO;LDLR |
| 1.154751817 | 1.22516405 | PDE3B;ADCY4;BRAF;ADCY8;MAPK14;ADCY7;MAPK13;MAD2L2;STK10;MAPK11 |
| 1.130334093 | 1.190625778 | ENTPD1;PKLR;GDA;PDE3B;ADCY4;AMPD1;ADA2;AMPD3;ADCY8;DCK;ADCY7;A |
| 1.145026525 | 1.156623429 | TGIF1;CREBBP;SMAD3;ROCK1;SMURF1;ACVR1B;TNF;TGFBR1;SMAD7;ZFYVE16 |
| 1.377123443 | 1.389307031 | HSPA5;SEC61G;SRPRA;SEC62 |
| 1.308308157 | 1.313202078 | ALDH2;DPYS;ALDH3B1;DPYD;UPB1 |
| 1.134020202 | 1.08642439 | MAP3K2;PRKCB;SRC;ADCY4;ITPR1;ITPR2;ADCY8;ADCY7;TUBA4A;TUBB1;GRB2; |
| 1.100133718 | 1.040849832 | MAOB;PRKCB;MAOA;ITPR1;CACNA1A;ITPR2;FOS;MAPK14;GNG11;MAPK13;M |
| 1.635137684 | 1.537388251 | SQOR;BPNT1 |
| 1.257915989 | 1.170509433 | POLR3A;POLR2A;POLR2C;POLR1F;POLR2K |
| 1.401590296 | 1.296124775 | MAOB;MAOA;ALDH3B1 |
| 1.119034091 | 1.025585471 | GABARAPL1;PRKCB;SRC;ADCY4;CACNA1A;ADCY8;ADCY7;GNG11;GLS;GABRR2; |
| 1.121458322 | 1.014380182 | PRDX5;NUDT7;NOS2;EPHX2;MPV17L;PMVK;ACSL6;ACSL5;ACSL4;FAR2;SOD2;X |
| 1.162915354 | 1.048585958 | PRKCB;VDR;ATP1A3;ATP1B3;ATP1A1;ATP2B1;SLC8A1;DNM2 |
| 1.074853341 | 0.961133681 | FZD1;TCF7L2;SMAD3;WNT2B;SERPINE1;AREG;ACTB;TGFBR1;ACTG1;STK3;SMA |
| 1.174060507 | 1.043952297 | IFNG;PSMA2;PSME1;PSME2;PSMB10;PSMB8;PSMB9 |
| 1.064943097 | 0.896569186 | TPM4;CREM;ADCY4;ATP1A3;ATP1B3;ATP1A1;ATP2B1;ADCY8;MAPK14;ADCY7; |
| 1.144642857 | 0.955870264 | LFNG;GALNT11;GALNT3;GALNT1;MFNG;EOGT;ST3GAL3 |
| 1.453371893 | 1.195039938 | GGT5;GGT1 |
| 1.154225649 | 0.944335498 | ALAS1;SDS;MAOB;MAOA;PSAT1;SDSL |
| 1.06794208 | 0.864306355 | PRKCB;ITPR1;SLC1A2;ADCY4;CACNA1A;ITPR2;PLA2G4A;ADCY8;GNG11;ADCY7; |
| 1.113300889 | 0.893226836 | LIPE;ADRB3;PIK3CA;PDE3B;ADCY4;ADCY8;PTGS2;ADCY7 |
| 1.167929974 | 0.933716532 | HK3;PFKFB4;PFKFB3;HK2;KHK |
| 1.189127973 | 0.925462322 | GCH1;MOCOS;ALPL;MOCS1 |
| 1.189127973 | 0.925462322 | HHEX;PKLR;HES1;HNF1B |
| 1.090078164 | 0.845010342 | FZD1;TCF7L2;CDKN1A;WNT2B;GADD45B;DVL2;BAK1;GLI1;GADD45G |
| 1.22625 | 0.923631838 | HYAL1;HYAL2;HPSE |
| 1.052471112 | 0.786416271 | FZD1;TCF7L2;CREBBP;EDN1;WNT2B;PRKCB;ADCY4;ADCY8;ADCY7;CREB1;DVL2 |
| 1.090010078 | 0.802135147 | GALC;UGCG;ASAH1;SPTLC2;NEU1;ACER3;SGPP1 |
| 1.137361104 | 0.811987866 | ATP6V1G1;TCIRG1;ATP6V1E1;ATP6V0D1 |
| 1.051025232 | 0.728962211 | NR4A1;CREB1;ADCY4;ITPR1;ITPR2;ADCY8;ADCY7;LDLR;ATF4 |
| 1.015461773 | 0.694752395 | HDAC4;H2AW;MAOB;MAOA;HDAC11;GNG4;H3-3A;H3-3B;H2AC6;H2BC5;BRAF |
| 1.63489819 | 1.103542165 | GLS |
| 1.024242424 | 0.682736324 | TPM4;ITGA2B;ADCY4;ADCY8;TNF;ADCY7;ACTB;SLC8A1;ACTG1;ITGAV;ITGB7;IT |
| 1.089908141 | 0.713767904 | SLC24A1;GNAT2;SAG;CALM1 |
| 1.040343915 | 0.67378183 | HSD17B1;ADCY4;PLA2G4A;ADCY8;PTGS2;ADCY7;LDLR |
| 1.054721762 | 0.670627121 | HK3;MGAM;PYGM;HK2;GANC |
| 0.998945081 | 0.63275782 | MAOB;UBA7;MAOA;LRRK2;ITPR1;ITPR2;UBE2L6;UBE2J2;COX7A1;UBE2J1;MAP |
| 1.005970932 | 0.621128867 | SLC10A1;SLC22A1;SLC2A1;ADCY4;ATP1A3;ATP1B3;SLC4A2;ATP1A1;ADCY8;AD |
| 0.993117058 | 0.607203652 | RYR1;NCF1;NCF4;ITPR1;ITPR2;TNF;COX7A1;MAPK8;C7;KIF5C;CASP3;TUBB1;RA |
| 1.0217027 | 0.601600525 | ADSS2;GLUL;ASRGL1;ASS1;GLS |
| 0.986924421 | 0.553385991 | DGKG;DGKD;DGAT2;ALDH2;GPAT4;MBOAT1;GPAT3;LPIN2 |

| | | |
|---|---|---|
| 1.307843137 | 0.725364338 | XDH |
| 0.980729955 | 0.542231455 | EDN1;CREB1;ADRB3;PTGER2;PDE3B;ITPR1;ITPR2;CALM1;KCNJ2 |
| 0.975824623 | 0.53238819 | TCF7L2;ACTN2;ITGA2B;ITGAV;ITGB7;ITGA5;ACTB;SLC8A1;ACTG1;ITGA9 |
| 0.968448889 | 0.512729843 | FZD1;TCF7L2;CDKN1A;CDKN1B;WNT2B;ITPR1;ADCY4;ITPR2;BRAF;ADCY8;ADCY |
| 0.95882917 | 0.490203763 | CCKAR;CREB1;PRKCB;SLC2A1;ADCY4;ATP1A3;ATP1B3;ATP1A1;ADCY8;ADCY7;A |
| 0.968639459 | 0.489345062 | HK3;MGAM;HK2;GANC |
| 0.933816373 | 0.429518397 | SDC4;LAMB3;ITGA2B;COL6A3;ITGAV;ITGB7;CD47;ITGA5;FREM1;CD44;ITGA9 |
| 0.915185185 | 0.400496049 | GGT5;GCLC;GPX3;ODC1;GSTA1;LAP3;GGT1 |
| 0.922752077 | 0.400908968 | RYR1;CREB1;RASD1;PRKCB;GNG4;ITPR1;ADCY4;FOS;CALM1;ADCY8;GNG11;AD |
| 0.94488472 | 0.389144562 | SEH1L;SLC1A2;TANK;TNF;COX7A1;ACTB;ACTG1;NXF1;KIF5C;ATG101;CASP3;RB |
| 0.886251642 | 0.342573529 | HK3;ALDH2;PKLR;ADPGK;ALDH3B1;ENO1;ENO3;HK2 |
| 0.934065934 | 0.360551289 | RFK |
| 0.871721363 | 0.336462154 | KYAT3;SCLY |
| 0.899242545 | 0.334885807 | CDKN2D;ZBTB17;CREBBP;CDKN1A;SMAD3;CDKN1B;GADD45B;GADD45G;MAD |
| 0.817122642 | 0.275063996 | ELOVL4;ELOVL5;HACD2 |
| 0.817122642 | 0.275063996 | ELOVL4;ELOVL5;HACD2 |
| 0.834344782 | 0.271963377 | IL10;CD40;TG;CD80;FAS;FASLG |
| 0.861611694 | 0.273081642 | CPB2;CELA2A;ELN;COL6A3;ATP1A3;ATP1B3;SLC3A2;SLC1A5;ATP1A1;SLC38A2; |
| 0.896366013 | 0.277788879 | KDM3A;PRKAA1;KDM3B;COX17;ADCY4;PRKAG2;ADCY8;ADCY7;COX7A1;ACTB; |
| 0.816846183 | 0.237938857 | KMT2E;ALDH2;SETDB1;KMT5C;KMT5B;EZH1;EZH2 |
| 0.802469136 | 0.218074175 | TRPC5;PIK3CA;PRKCB;ITPR1;ITPR2;KRAS;RAF1 |
| 0.72630873 | 0.195047248 | MTHFD2;MTHFS |
| 0.72630873 | 0.195047248 | CYP27B1;CYP24A1 |
| 0.726205451 | 0.180728103 | RPIA;TKT;RBKS |
| 0.791934468 | 0.194508572 | MMP1;ACSL6;APOA2;OLR1;ACSL5;NR1H3;ACSL4;PPARD |
| 0.742541541 | 0.169101944 | LPCAT2;ENPP2;PLA2G4A;PLD4;PLA2G5 |
| 0.725998657 | 0.152318422 | GCLC;SDS;PSAT1;KYAT3;SDSL |
| 0.622442564 | 0.116720965 | FUT4;ST3GAL3 |
| 0.670131932 | 0.116795694 | ALDH2;ACSL6;ACSL5;ACSL4 |
| 0.544714681 | 0.093117664 | LIG4 |
| 0.791303249 | 0.13518667 | PRKCB;ITPR1;ADCY4;CACNA1A;ITPR2;ADCY8;PTGS2;MAPK14;GNG11;ADCY7;M |
| 0.653340883 | 0.103926438 | DYNC1H1;CREB1;ARHGDIB;STX4 |
| 0.742054484 | 0.11775017 | UPF2;NXF1;SMG1;DDX19B;DDX39B;FUS;PPP2R3C;PAPOLG;ALYREF;ETF1 |
| 0.568251521 | 0.083930171 | PLA2G4A;PLA2G5 |
| 0.740418458 | 0.108412585 | RCL1;UTP15;NXF1;XRN1;EIF6;XRN2;SBDS;GAR1;RIOK1;UTP14A;GTPBP4 |
| 0.786175465 | 0.10838626 | UPF2;RANBP2;EIF4A1;SEH1L;NUP210;FUS;FMR1;ALYREF;THOC2;SENP2;EIF1;N |
| 0.593962264 | 0.080195765 | MAOB;MAOA;ALDH3B1 |
| 0.640364907 | 0.081286715 | SCUBE2;CCND2;GRK2;SMURF1;GLI1 |
| 0.46684443 | 0.058717721 | ALPL |
| 0.725272414 | 0.087028733 | LIPE;CRTC2;PFKFB4;PRKAA1;PFKFB3;CREB1;PIK3CA;PPP2R3C;PRKAG2;PRKAB1 |
| 0.408441742 | 0.03802309 | HSD3B7 |
| 0.483954343 | 0.044322279 | PLA2G4A;PLA2G5 |
| 0.529627741 | 0.047212512 | GABRR2;GRIN3A;CACNA1A |
| 0.665948559 | 0.054828487 | MAOB;MAOA;ALDH3B1;DPYS;DPYD;GSTA1;FMO2;UPP1;UPB1;XDH |
| 0.466643315 | 0.037910922 | PGP;GLUL |
| 0.384393576 | 0.030837448 | NEU1 |
| 0.489820755 | 0.031837385 | ABCA1;DGAT2;PLA2G5 |

| | | |
|---|---|---|
| 0.712437142 | 0.041333228 | SMAD3;NCF1;PRKCB;ATP5PB;PRKCD;NCF4;SLC2A1;CYBB;MAPK14;COX7A1;REI |
| 0.671360587 | 0.038188764 | DDX5;DHX8;FUS;ALYREF;THOC2;HSPA2;PQBP1;U2AF1L4;DDX39B;TRA2A;SRSF |
| 0.421411275 | 0.023868532 | POLB;PARP3 |
| 0.466442049 | 0.024506064 | GTF2B;GTF2H1;TAF5L |
| 0.311103929 | 0.013869976 | PMVK |
| 0.445188679 | 0.018871879 | ALDH2;PKLR;ME2 |
| 0.43527044 | 0.016562868 | HK3;UAP1;HK2 |
| 0.607908108 | 0.02223014 | MAD2L2;MAPK11;ITPR1;ADCY4;ITPR2;CALM1;ADCY8;MAPK14;ADCY7;REC8;M |
| 0.284018624 | 0.009469736 | CBLIF |
| 0.352951971 | 0.009649391 | MAN1A2;ST3GAL3 |
| 0.720567439 | 0.018849134 | PTGFR;PTGER2;PTAFR;C5AR1;LPAR3;LTB4R;C3;P2RY8;GABRR2;P2RY6;HTR7;C( |
| 0.261266968 | 0.006523816 | PIGH |
| 0.489242539 | 0.010768717 | SCN9A;ADCY4;CACNA1A;PKD1L3;ADCY8;TRPM5 |
| 0.483174523 | 0.009717257 | TPM4;ATP1A3;ATP1B3;ATP1A1;COX7A1;SLC8A1 |
| 0.42097444 | 0.007908363 | SARS1;CARS1;TARS1;WARS1 |
| 0.318444029 | 0.005326582 | ALAS1;CPOX |
| 0.225178135 | 0.003162432 | ALDH2 |
| 0.225178135 | 0.003162432 | IDH3A |
| 0.290070833 | 0.002954984 | CUL4A;GTF2H1 |
| 0.283748544 | 0.002551769 | ALDH2;ACSF3 |
| 0.362296045 | 0.002388589 | HSD11B1;ALDH3B1;CYP2S1;GSTA1 |
| 0.186511528 | 0.001109345 | RNASEH2B |
| 0.603270977 | 0.002092623 | ITPR1;SLC1A2;COX7A1;MAPK8;CASP8;POLR2A;KIF5C;ATG101;CASP3;RB1CC1;I |
| 0.4167138 | 8.99E-04 | ATP6V1G1;PPA1;ATP5PB;COX17;TCIRG1;ATP6V1E1;ATP6V0D1;COX7A1 |
| 0.221061448 | 3.84E-04 | HSD11B1;HSD17B1 |
| 0.133114784 | 1.08E-04 | MAN1A2 |
| 0.197535521 | 1.40E-04 | CYP2S1;DHRS3 |
| 0.123039927 | 5.63E-05 | HES1 |
| 0.301271944 | 1.76E-05 | RPS9;MRPS15;RPS7;RPL10;RPLP1;FAU;RPL18 |
| 0.073410425 | 4.15E-07 | RGS2;GRK2;GNAL;CALM1;SLC8A1 |

IAP3K5;MAP2K3;EDN1;RIPK3;IL15;TRAF2;FOS;TRAF1;TNFRSF1B;TNFRSF1A;CREB1;PIK3CA;TRAF3;IL1B;IRF1;LTA;P
?7;CASP8;CASP6;CASP3;TNFSF10;IL12B;OLR1;IL12A;LBP;JAK2;IKBKE;MAP3K5;MAP2K3;TICAM2;MMP1;IL18;CYBB
MPT;CASP4;IKBKE;JAK1;IFNAR2;GBP5;GABARAPL1;RIPK3;RIPK2;PRKCD;IL18;CYBB;TRAF2;IRAK4;TYK2;IRF3;OAS2;
DARADD;TICAM2;IL1R1;PRKCB;DDX58;TRAF2;IRAK4;TRAF1;TIRAP;TNFRSF1A;TRAF3;IL1B;LTA;PRKCQ;LTB;TLR4;B
;JAK1;MAP2K3;TICAM2;PRKCB;DDX58;FOS;IRAK4;TYK2;TGFBR1;TIRAP;CREB1;IRF3;PIK3CA;TRAF3;VDAC3;IRF7;RA
PIK3CA;IL23A;IL1B;IRF1;MAPKAPK2;CLEC4E;RAF1;IRF9;SRC;ITPR1;ITPR2;PTGS2;MALT1;RELA;RELB;MAPK8;PAK1;
K8;CASP3;IL12B;NLRP3;IL12A;CD14;IL10;JUN;TICAM2;NOS2;FOS;IRAK4;MAPK14;TIRAP;NFKB1;MAPK13;IL1A;MA
KBKB;CASP8;CASP3;TNFSF10;IL12B;TRIM25;IL12A;JAK2;IKBKE;KPNA1;JAK1;IFNAR2;RSAD2;PRKCB;DDX58;IFNGR1
MAP2K3;TICAM2;FOS;IRAK4;TIRAP;IRF3;PIK3CA;TRAF3;IL1B;IRF7;TLR9;TLR8;IRF5;TLR6;TLR4;TLR3;IFNAR1;TLR2;L
IKBKE;JAK3;JAK1;IFNAR2;DDX58;FOS;IRAK4;TYK2;IL1A;IRF3;PIK3CA;OAS2;TRAF3;IL1B;IRF7;TLR9;CD46;TLR4;IRF9;
12A;LBP;JAK2;JAK1;IL10;FCER1G;KSR1;IFNGR1;RIPK2;IFNGR2;IL18;IRAK4;TIRAP;TNFRSF1A;IL1A;CREB1;IFNG;IL23
CASP3;B2M;IKBKE;JAK3;JAK1;IFNAR2;MAP2K3;PDIA3;ENTPD1;DDX58;TAP2;TAP1;TRAF2;TYK2;IRAK4;TAPBP;FCE
R5;JAK2;JAK3;CCR3;CCR2;PRKCB;PRKCD;VAV1;FGR;HCK;PIK3CA;PARD3;RAF1;DOCK2;CCL14;ROCK1;SRC;PXN;ADC
B;IL12A;IKBKE;IL6R;JAK1;IFNAR2;RPS9;RPS7;PRKCB;DDX58;MMP1;CYBB;FOS;TYK2;IRAK4;TNFRSF1A;ADAM17;IRF
NGR1;IFNGR2;TRAF2;FOS;GAB2;TYK2;TGFBR1;TNFRSF1A;IL1A;CREB1;IFNG;PIK3CA;IL1B;LCP2;SQSTM1;IRF9;IFNA
;GLUL;JAK3;JAK1;IFNAR2;ZBP1;TICAM2;H2AC6;RIPK3;IFNGR1;IFNGR2;CYBB;PLA2G4A;TRAF2;TYK2;TNFRSF1A;IL1
;JAK2;IKBKE;CCR3;JAK1;IFNAR2;GABARAPL1;IFNGR1;TRAF2;FOS;TYK2;TNFRSF1A;HCK;CREB1;IRF3;PIK3CA;TRAF3;
;B2M;IL6R;CCR3;JAK1;PDIA3;ARHGEF11;IL1R1;PRKCB;TAP2;TAP1;TRAF2;TAPBP;TNFRSF1A;CREB1;IRF3;PIK3CA;IL
CASP8;CASP3;IL12B;IL12A;CCR5;JAK2;LDLR;JAK1;MAP2K3;IL10;LAMB3;NOS2;IFNGR1;IL10RB;STAT1;IFNGR2;IL10
;JAK1;IFNAR2;IL10;IL15RA;IL4R;CISH;IFNGR1;IL15;IFNGR2;TYK2;PIAS2;PIAS1;IFNG;PIK3CA;IL23A;IL6ST;RAF1;STA
6R;IL13RA1;IFNAR2;IL15RA;IL4R;PRKCB;BCR;EML4;IL23A;IL6ST;RAF1;IFNAR1;NOTCH3;CTBP2;MAX;NOTCH4;TGF
JN;MARCKSL1;NOS2;IFNGR1;PRKCB;STAT1;IFNGR2;CYBB;FOS;IRAK4;MAPK14;NFKB1;MAPK13;NFKBIA;IL1A;MAP
XCL2;IL27RA;CXCL16;TNFSF13B;IL18RAP;TNFSF10;IL12B;IL12A;CCR6;CCR5;IL6R;TNFRSF4;CCR3;CCR2;IL13RA1;IL
IK3;CASP4;CAPN2;H3-3A;H3-3B;CAST;GABARAPL1;FNBP1;IL1R1;RIPK2;ACTN1;PRKCE;PRKCD;IL18;TRAF2;ACTN4;
2;IL6R;JAK3;JAK1;STAT5A;STAT5B;JUN;IL4R;SMAD3;IL1R1;IFNGR1;STAT1;IFNGR2;STAT3;FOS;TYK2;MAPK14;NFK
R1;CCL4;CXCR2;TNFSF10;CCL3;CCL2;CCR6;CCR5;IL6R;CCR3;CCR2;CCR1;IL10;XCR1;TNFSF14;CCL20;IL10RB;IL10R
TRAF2;FOS;IRAK4;VAV1;IRF3;PIK3CA;IL1B;ELMO2;ARHGEF1;LCP2;ITGA5;TLR4;SKAP2;ARF6;ROCK1;SRC;PXN;NLRC
8;CASP8;CASP3;CCL2;MAPK6;IKBKE;USP25;JUN;MMP1;CCL20;TRAF2;FOS;MAPK14;IL17RC;NFKB1;MAPK13;FOSL
BB1;CASP4;TNFSF10;KPNA3;KPNA1;MAP2K3;ANXA2;RIPK3;RIPK2;IL18;RHOG;RHOH;TRAF2;FOS;IRAK4;TUBA4A;
ASP3;RAC2;MAP3K8;MAP3K5;MAP4K1;MAP2K3;DUSP5;DAXX;DUSP2;IL1R1;PRKCB;DUSP1;PLA2G4A;TRAF2;FOS;
FSF10;CTSH;CTSC;MAP3K5;DFFB;DAXX;PARP3;TRAF2;FOS;TRAF1;NGF;TUBA4A;TNFRSF1A;PIK3CA;DDIT3;RAF1;B
2B;IL12A;STAT6;JAK2;JAK3;JAK1;STAT5A;STAT5B;JUN;IL4R;IFNGR1;STAT1;IFNGR2;FOS;TYK2;MAPK14;NFKB1;M
12A;IL10;JUN;NOS2;IFNGR1;IFNGR2;FOS;IRAK4;CFLAR;MAPK14;NFKB1;TGFBR1;TNFRSF1A;MAPK13;NFKBIA;MA
IL10;JUN;IL4R;SMAD3;IFNGR1;STAT1;IFNGR2;STAT3;IL18;NFKB1;IL1A;IL6;IFNG;IL23A;IL1B;TLR4;IL18R1;TLR2
R1;STAT1;IFNGR2;STAT3;FOS;MAPK14;TIRAP;NFKB1;BATF;MAPK13;EML4;NFKBIA;MAPK11;IFNG;PIK3CA;TLR9;P
AK2;STAT5A;EGR1;STAT5B;JUN;EDN1;SMAD3;PRKCB;STAT1;PRKCE;PRKCD;STAT3;CYBB;VEGFC;MAPK14;SELE;F3

25;IL12A;RIPK1;IKBKE;MAP3K1;DDX58;TRAF2;ISG15;MAPK14;NFKB1;MAPK13;NFKBIA;CXCL10;CYLD;MAPK11;IRF
X58;TRAF2;TYK2;PIAS1;TNFRSF1A;IFNG;IRF3;PIK3CA;OAS2;TRAF3;IRF7;RAF1;IRF9;TLR3;IFNAR1;RNASEL;RELA;SO
SP4;TNFSF10;ARHGEF11;IL1R1;IL18;TRAF2;FOS;IRAK4;TUBA4A;TIRAP;TNFRSF1A;IL1B;HCLS1;ARHGEF1;ARHGEF2
PK8;CCND2;BCL2L11;TNFSF10;S1PR1;S1PR4;IL10;CDKN2D;PLK3;GABARAPL1;CREBBP;SMAD3;GADD45B;STAT3;
R5;JAK3;CCR3;JAK1;ACTN1;TRAF2;ACTN4;TRAF1;KAT2B;CREB1;H2BC15;IRF3;PIK3CA;TRAF3;MAPKAPK2;RASA2;V
ZFP36;CCND2;KAT5;TSPO;B2M;JAK3;JAK1;IL15RA;IL1R1;IL15;IL1R2;FOS;TGFBR1;TNFRSF1A;KAT2B;CREB1;PIK3C
B;MAPK8;RAC2;CCL2;ITGAV;MAP3K5;MEF2A;JUN;EDN1;IL1R1;DUSP1;IL1R2;FOS;MAPK14;SELE;NFKB1;PTK2;ASS

RKCB;TAP2;TAP1;TRAF2;FOS;IRAK4;TNFRSF1B;TAPBP;TNFRSF1A;IRF3;PIK3CA;RAF1;TLR4;TLR2;PXN;ITPR1;CXCR4
;PLAU;NFKBIZ;ITGB7;H3-3A;H3-3B;IL1R2;LMO2;TRAF1;DUSP6;ETV6;DDIT3;BMP2K;RARA;ERG;NUPR1;BIRC2;BIR

2;PLCG2;ATP6V1E1;LYN;ATP6V1G1;JUN;MAPK14;NFKB1;MAPK13;NFKBIA;MAPK11;ADAM17;ATP6V0D1;HBEGF
;ATP6V1E1;ATP6V1G1;JUN;IL15;MMP1;CCL20;IL18;FOS;VEGFA;IL1A;IL6;IFNG;IL23A;IL1B;LTB;ATP6V0D1;TLR4;TL

MAP3K5;JUN;MAP3K1;RIPK2;PRKCD;BRAF;IRAK3;IRAK4;NGF;MAPK14;NFKB1;MAPK13;NFKBIA;MAPK11;PIK3CA;
;HK3;MKNK1;PLCG2;IL6R;EIF4E;CREBBP;EDN1;EGLN3;NOS2;PRKCB;IFNGR1;IFNGR2;STAT3;CYBB;NFKB1;VEGFA;
NB;MAP2K3;STAT5A;GHSR;STAT5B;CREBBP;MAP3K1;PRKCB;STAT1;STAT3;FOS;MAPK14;PTK2;MAPK13;MAPK11;
AD3;GADD45B;BRAF;GAB2;TGFBR1;NFKB1;GADD45G;BCR;NFKBIA;PIK3CA;MDM2;GRB2;KRAS;RAF1

F5;RAC2;MAP2K3;PRKCB;ARAP3;NGF;VAV1;APBB1IP;ADORA2A;PIK3CA;ADORA2B;PARD3;RAPGEF1;PRKD2;LCP

;GSN;PRKCB;PRKCE;LIMK2;PRKCD;LIMK1;PLA2G4A;GAB2;VAV1;DNM2;HCK;MARCKS;PTPRC;FCGR2A;PIK3CA;RAF
0;SERPINB4;ARG2;LAMB3;NOS2;IL1R1;PRKCB;ACTN1;IL1R2;ACTN4;NFKB1;PTK2;IL6;GNAL;IFNG;PIK3CA;IL1B;TLR
P35;MYLK3;PIK3R6;PIK3CG;ACTG1;PIK3R5;RAP1B;PLCG2;FYN;LYN;VASP;P2RY12;PTGIR;FCER1G;PLA2G4A;MAPK

CTB;RELA;ACTG1;C3;FCGR3A;CLEC7A;CASP4;PLCG2;RAC2;ATG7;ELANE;H3-3A;H3-3B;GSDMD;H2AC6;H2BC5;PR

F3;BAK1;ZBTB17;GADD45B;LAMB3;NOS2;TRAF2;TRAF1;NFKB1;PTK2;GADD45G;NFKBIA;PIK3CA;TRAF3;BIRC2;BIR
G;CCND2;CASP8;CASP3;ITGAV;ITGB7;ATP6V1E1;IKBKE;JAK1;IFNAR2;ATP6V1G1;TYK2;TNFRSF1A;CREB1;IRF3;PIK
CTB;ACTG1;PAK1;PLAU;CASP3;PLCG2;IL12B;ITGAV;FZD1;PRKCB;STAT3;PLAUR;BRAF;MIR21;MAPK14;PTK2;VAV1

RAP1B;RASSF5;PLCG2;RAC2;PTK2B;VASP;PRKCB;ACTN1;CYBB;RHOH;ACTN4;MAPK14;PTK2;VAV1;MAPK13;MAP
KB1;VAV1;MAPK13;NFKBIA;MAPK11;PTPRC;TEC;IFNG;PIK3CA;PRKCQ;GRB2;LCP2;KRAS;PTPN6;RAF1;NFKBIE;NF

PTK2B;FYN;BID;IFNAR2;FCER1G;PRKCB;IFNGR1;IFNGR2;BRAF;VAV1;NCR1;IFNG;PIK3CA;FAS;GRB2;CD48;LCP2;KR

;BCL2L11;CASP8;CASP3;BID;IL6R;MAP3K5;MLXIP;JUN;XBP1;PKLR;NR1H3;TRAF2;FOS;MAPK14;PRKAB1;NFKB1;T
C1B;MLXIP;PTPN1;PRKCB;PRKCE;NR1H2;PRKCD;STAT3;NR1H3;PRKAB1;NFKB1;TNFRSF1A;NFKBIA;IL6;CREB1;PIK

1PR2;BID;S1PR4;MAP3K5;FCER1G;PRKCB;PRKCE;TRAF2;GAB2;MAPK14;NFKB1;SGPP1;TNFRSF1A;MAPK13;MAP
;JUN;MAP3K1;PRKCB;PRKCD;PLA2G4A;MAPK14;MAPK13;MAPK11;GRB2;KRAS;RAF1;CALM1;ATF4;HBEGF
;RAP1B;PAK1;MAPK8;CCND2;RAC2;CAPN2;PIP5K1B;ITGAV;ITGB7;FYN;VASP;JUN;LAMB3;PRKCB;ACTN1;VEGFC;
1;CLEC7A;SEC61G;LAMP2;TUBB1;OLR1;ITGAV;CD14;ATP6V1E1;DYNC1H1;ATP6V1G1;TAP2;CYBB;TAP1;TUBA4A;
;MAP2K3;IL1R1;PRKCB;PRKCE;PRKCD;PLA2G4A;NGF;MAPK14;MAPK13;MAPK11;PIK3CA;IL1B;PRKCQ;CALM1
;ATXN3;MAPK8;MAN1A2;SEC61G;CAPN2;BAK1;SEC62;UGGT1;MARCHF6;MAP3K5;PDIA3;XBP1;SEC24B;HSPA5;E
ADCY8;ADCY7;MYLK3;SLC8A1;PIK3R6;PIK3CG;PIK3R5;LIPE;GNG4;MEF2A;EGR1;GABARAPL1;SMAD3;NOS2;PRKC
1;PHKG1;MKNK1;PHKG2;FLOT1;FLOT2;EIF4E;SH2B2;PTPN1;PKLR;BRAF;PRKAB1;PIK3CA;PPP1R3B;PPP1R3D;RAP

E2F2;E2F3;MAP2K3;SMAD3;GADD45B;MAPK14;SIRT1;NFKB1;TGFBR1;HIPK3;GADD45G;MAPK13;IL1A;MAPK11;I
BKB;CCND2;ITGAV;ITGB7;JAK2;JAK3;IL6R;JAK1;IFNAR2;IL4R;NGF;EREG;CREB1;PIK3CA;DDIT4;COL6A3;SGK3;ITGA5

;PDE4B;MEF2A;ARHGEF11;EGR1;PRKCB;VDR;BRAF;FOS;NR4A2;CREB1;CYP24A1;ARHGEF1;RAF1;ATF4;HBEGF
VEGFC;FOS;MAPK14;GNG11;NFKB1;TGFBR1;MAPK13;VEGFA;NFKBIA;ACTA2;MAPK11;CREB1;PIK3CA;GRB2;KRAS

P6V1E1;FNIP1;EIF4E;FNIP2;FZD1;ATP6V1G1;PRKCB;BRAF;TNFRSF1A;SLC7A5;CLIP1;PIK3CA;DDIT4;GRB2;KRAS;RA

LR;ADCY8;ADCY7;MYLK3;SLC8A1;HTR7;CCKAR;PHKG1;HRH2;PHKG2;PLCG2;CD38;PTK2B;CAMK1D;NOS2;PRKCB;V
;ADCY7;ACTB;MYLK3;PIK3R6;PIK3CG;ACTG1;PIK3R5;RGS2;CD38;KCNJ2;JUN;CAMK1D;PRKCB;KCNJ12;PLA2G4A;F
5;CYTH4;CXCR1;CXCR2;PLCG2;FCER1A;PTK2B;PIP5K1B;FYN;RALGDS;CYTH1;FCER1G;PLA2G4A;GAB2;DNM2;PIK3C

KB;RAP1B;RASSF1;PAK1;MAPK8;HTR7;GNG4;RASSF5;PLCG2;RAC2;SHOC2;RALGDS;KSR1;PRKCB;VEGFC;PLA2G4A;

ACTG1;FGD3;PAK1;TMSB4X;RAC2;PIP5K1B;ITGAV;ITGB7;GSN;ACTN1;LIMK2;LIMK1;BRAF;ACTN4;SSH2;PTK2;VAV1
1;PRKCB;MIR23A;PRKCE;MIR27A;MIRLET7I;MIR21;SIRT1;PIK3CA;DDIT4;MIR101-1;FSCN1;ITGA5;EZR;RAF1;CD44;I
K5;KIF5C;CXCR2;GRK6;PIP5K1B;CCR5;LDLR;RAB11FIP4;CYTH1;SMAD3;IQSEC1;SMURF1;IQSEC3;ARAP3;VPS37A;HS

ATP1A1;HIF1A;ACTB;MED12L;ACTG1;KAT2B;MED13;MED30;PIK3CA;PLCG2;MDM2;ITGAV;KRAS;RAF1
E2F3;HES1;BAK1;FZD1;NCOA1;TCF7L2;JUN;GADD45B;BRAF;FOS;GADD45G;NFKB2;PIK3CA;FGF18;GRB2;KRAS;RAF
SIR;ICAM1;SPN;ALCAM;ITGAV;ITGB7;ICOSLG;PDCD1LG2;SELE;SELP;VCAN;PTPRC;SELL;PECAM1;SIGLEC1;NECTIN2

2;LAMP2;ATG7;SNAP29;GABARAPL1;PRKCD;CFLAR;VMP1;ATG16L2;PIK3CA;ATG16L1;DDIT4;PRKCQ;KRAS;RAF1;S
5C;CASP3;RB1CC1;TUBB1;CAPN2;MAP3K5;MAP2K3;DAXX;GABARAPL1;PRKCB;GPX3;CYBB;TRAF2;TNFRSF1B;TUBA

SD11B1;JAK2;IKBKE;KPNA3;KPNA1;PAQR8;STAT5A;STAT5B;JUN;PRKCB;VDR;EPHX2;STAT3;MIRLET7I;FOS;KLF4;N
PR2;PLA2G4A;BRAF;PLA2G5;ADCY8;ADCY7;MYLK3;ACTA2;ADORA2A;ADORA2B;PRKCQ;ARHGEF1;RAF1;CALM1

1B;LIPE;PAK1;MAPK8;RAC2;PDE4B;FFAR2;GHSR;CREBBP;JUN;EDN1;ARAP3;BRAF;ATP1B3;FOS;ATP2B1;VAV1;NFK

RAC2;TBL1X;TLE4;FZD1;TLE3;TCF7L2;CREBBP;JUN;SMAD3;PRKCB;SENP2;DKK4;FOSL1;DAAM2;ROR2;LGR6;PPARI

3A1;PIK3R6;PIK3CG;PIK3R5;RGS2;MEF2A;VASP;TRPC6;PRKCE;ATP1B3;ATP2B1;CREB1;ADRB3;PPIF;VDAC3;RAF1;C

2M;JAK1;IFNAR2;PDIA3;DAXX;IFNGR1;DDX58;IFNGR2;TAP2;TAP1;TRAF2;TYK2;IRAK4;TNFRSF1A;TAPBP;PIK3CA;II

F3;BAK1;FZD1;TCF7L2;SMAD3;GADD45B;PRKCB;BRAF;TGFBR1;GADD45G;PIK3CA;GSTA1;GRB2;KRAS;RAF1;NFE2L
YN;SRGAP2;PLXNA4;SEMA4A;TRPC5;SEMA6A;TRPC6;UNC5A;SEMA4D;LIMK2;SEMA4B;LIMK1;SSH2;PTK2;PIK3CA

3;MAP3K5;RAB8B;VASP;JUN;MAP3K1;ACTN1;PRKCE;ACTN4;PRKAB1;TUBA4A;PARD3;HCLS1;ARHGEF2;EZR;RAPG

:B1;CAPN2;MAP3K5;CYBB;TRAF2;TUBA4A;TNFRSF1A;DKK4;IL1A;ADAM17;PIK3CA;PSMA2;DDIT3;IL1B;VDAC3;PP

:;GNG11;H2AJ;H3-5;GRIN3A;CREB1;H2BC15;ADORA2A;ADORA2B;FOSB;GRB2;KRAS;RAF1;CALM1;ATF4

'K8;KIF5C;CASP3;TUBB1;MAP3K5;DAXX;XBP1;HSPA5;DUSP1;ATP5PB;SEPTIN5;UBE2G1;TUBA4A;GNAL;ADORA2A;

AC2;FYN;EGR1;HSPA5;ATP5PB;PRKCD;CYBB;HSPA2;MAPK14;TUBA4A;MAPK13;IL1A;MAPK11;IL6;GRIN3A;CREB1;I

1CC1;TUBB1;BID;MAP3K5;RANBP2;MAP2K3;DAXX;GABARAPL1;XBP1;NUP210;DNAH17;HSPA5;NOS2;ATP5PB;FU

ACTG1;LIPE;SLC25A29;MAP3K5;MAP2K3;ATP5PB;ACSL6;ACSL5;ACSL4;MAPK14;PRKAB1;MAPK13;MAPK11;CREB

:KAR;HRH2;CHRNE;C3AR1;S1PR1;TSPO;S1PR2;INSL3;S1PR4;GHSR;EDN1;PTGIR;P2RY11;GPR35;P2RY14;P2RX5;GF

'OLR2C;TUBB1;POLR2K;MAP3K5;TGM2;CREBBP;DNAH17;ATP5PB;GPX3;TRAF2;SOD2;TUBA4A;CREB1;PSMA2;VD

GAM5;BIRC2;ATF4;BIRC3;NOD2;PTGS2;RELA;SOCS3;MAPK8;CCL2;RIPK1;JUN;MLKL;CCL20;LIF;VEGFC;CFLAR;MAI
;TRAF2;FOS;IRAK4;VAV1;TIRAP;TNFRSF1A;IRF3;PIK3CA;TRAF3;DDIT3;IL1B;IRF7;ARHGEF1;TLR6;TLR4;ABCG1;ATF
TRAF3;IL1B;VDAC3;IRF7;TXNIP;RBCK1;TLR4;NAIP;BIRC2;IRF9;IFNAR1;BIRC3;RNF31;RNASEL;ITPR1;ITPR2;XIAP;NC
IRC2;BIRC3;BCL2A1;LY96;XIAP;PTGS2;MALT1;RELA;RELB;CCL4;PLCG2;RIPK1;CD14;LYN;GADD45B;TNFSF14;CFLAI
AF1;TLR4;TLR3;ATF4;IFNAR1;TLR2;SRC;RELA;MAPK8;STAT4;E2F2;PTK2B;E2F3;STAT6;BID;STAT5A;STAT5B;CREBBI
CLEC7A;PLCG2;NLRP3;PLK3;EGR2;JUN;EGR3;STAT1;STAT2;MAPK14;NFKB1;NFKB2;MAPK13;NFKBIA;CYLD;MAPK1

L;IFNGR2;IL18;IRAK4;TYK2;TNFRSF1A;IL1A;IFNG;IRF3;PIK3CA;OAS2;TRAF3;IL1B;IRF7;NUP98;RAF1;TLR4;IRF9;TLR
Y96;RELA;MAPK8;CCL4;CCL3;RIPK1;CD14;JUN;STAT1;MAPK14;NFKB1;MAPK13;NFKBIA;CXCL10;MAPK11;CXCL11
;IFNAR1;TLR2;IL2RG;RELA;MAPK8;BAK1;BID;SLAMF1;STAT5A;STAT5B;JUN;STAT1;MX2;STAT2;MX1;STAT3;EIF2AK
A;IL1B;RFX5;TLR9;TLR6;ATP6V0D1;CLEC4E;RAF1;TLR4;TLR2;SRC;CEBPG;NOD2;CORO1A;MALT1;RELA;C3;CYP27B
R2;IRF3;PIK3CA;OAS2;TRAF3;IRF7;CD44;IRF9;TLR2;IFNAR1;RBPJL;RELA;RELB;MAPK8;BCL2L11;PLCG2;E2F2;RIPK1
Y4;CXCR4;RASGRP2;ADCY8;ADCY7;PIK3R6;RELA;PIK3R5;RAP1B;PAK1;GRK2;CCL8;GRK5;CXCR1;GNG4;GRK6;CCL
F3;PIK3CA;OAS2;TRAF3;IL1B;TLR8;MASP2;IL6ST;TLR4;CFB;TLR3;IRF9;HBEGF;TLR2;IFNAR1;CFD;RPL10;C1S;C1R;C5
AR1;RELA;RELB;SOCS3;MAPK8;SOCS1;PLCG2;FYN;JUN;STAT1;STAT2;MAPK14;NFKB1;FOSL2;NFKB2;MAPK13;FOSL
A;H2AJ;IFNG;IL1B;VDAC3;PGAM5;RBCK1;SQSTM1;TLR4;BIRC2;IRF9;TLR3;IFNAR1;BIRC3;CHMP5;RNF31;VPS4B;XI
MAPKAPK2;IRF7;IL6ST;RAF1;TLR3;IRF9;IFNAR1;SRC;ITPR1;ITPR2;PTGS2;HIF1A;PIK3R6;RELA;PIK3R5;C3;MAPK8;G
1B;ARHGEF1;RAF1;ATF4;ROCK1;SRC;PTGER2;PXN;ADCY4;ITPR1;CXCR4;ITPR2;ADCY8;PTGS2;ADCY7;RELA;GNG4;
RA;STAT3;HSPA2;IRAK4;TYK2;MAPK14;NFKB1;TNFRSF1A;MAPK13;NFKBIA;MAPK11;IFNG;PPIF;TAB2;TLR4;MYD8
M2;IRF9;IFNAR1;IL20RA;CSF2RB;IL2RG;IL22RA2;SOCS3;SOCS1;IL21R;STAT4;STAT6;MCL1;STAT5A;STAT5B;CREBBI
A;LPAR3;CSF2RB;RASGRP2;STK4;HIF1A;RASGRP4;HSP90B1;BCL2L11;PLCG2;DVL2;RALGDS;FZD1;STAT5A;ZBTB17;

10;IFNAR2;IL15RA;IL4R;IL1R1;IFNGR1;IL15;IL1R2;IFNGR2;TNFRSF18;IL18;IL16;NGF;TNFRSF1B;IL17RC;TGFBR1;TN
TNFRSF1A;IRF3;PIK3CA;IL1B;HCLS1;ELMO2;PRKCQ;RBCK1;ARHGEF2;ITGA5;TLR4;SQSTM1;NAIP;CD44;ARF6;RNF3
B1;TGFBR1;MAPK13;NFKBIA;MAPK11;IL6;IFNG;IL23A;IL1B;IL2RA;IL2RB;RARA;PRKCQ;IL6ST;NFKBIE;NFKBIB

4;MEFV;RELA;MAPK8;NLRP3;CCL2;PTK2B;PIP5K1B;JUN;FYB1;LIMK1;MAPK14;NFKB1;PTK2;MAPK13;NFKBIA;MAI

TIRAP;DNM2;TNFRSF1A;PIK3CA;IL1B;TLR9;ELMO2;TLR6;RAF1;TLR4;NAIP;BIRC2;ARF6;TLR2;BIRC3;LY96;NOD1;NL
IRAK4;NGF;DUSP6;TGFBR1;EREG;TNFRSF1A;IL1A;MAPKAPK3;DDIT3;IL1B;MAPKAPK2;RASA2;RAF1;ATF4;MAX;TG
RC2;ATF4;BIRC3;BCL2A1;ITPR1;ITPR2;XIAP;CSF2RB;RELA;MAPK8;BCL2L11;RIPK1;BAK1;BID;MCL1;JUN;GADD45B;

CS3;E2F2;RIPK1;E2F3;BAK1;BID;LDLR;STAT1;MX2;STAT2;MX1;STAT3;EIF2AK2;NR1H3;BRAF;CFLAR;NFKB1;NFKBIA
;EZR;TLR4;NAIP;ARF6;ROCK1;SRC;RELA;SLC9A3R1;PAK1;MAPK8;CYTH4;NLRP3;RIPK1;FYN;BAK1;CYTH1;JUN;SEC2
BRAF;MAPK14;SOD2;PRKAB1;SIRT1;TGFBR1;GADD45G;MAPK13;MAPK11;IL6;BCL6;PIK3CA;CCNG2;MDM2;GRB2;
DAC3;IRF7;ATP6V0D1;IL6ST;IRF9;ATF4;HDAC4;RBPJL;SRC;PXN;RELA;C3;BAK1;LYN;STAT5A;STAT5B;CREBBP;EGR2
A;CANX;VDAC3;LTA;ATF4;ADCY4;XIAP;ADCY8;IL2RG;ADCY7;RELA;RELB;MAPK8;E2F2;E2F3;STAT5A;EGR1;STAT5B;
1;TNFRSF1A;MAPK13;VEGFA;IL1A;MAPK11;IFNG;PIK3CA;IL1B;GSTA1;PECAM1;ARHGEF2;CALM1;SQSTM1;NFE2L

;ITPR2;RELA;PAK1;MAPK8;GNG4;PLCG2;PTK2B;RIPK1;BAK1;BID;JUN;LIMK2;LIMK1;MAPK14;GNG11;PTK2;NFKB1

3;DDX5;BCL2A1;MAX;CEBPE;RELA;CD14;BAK1;ELANE;ZBTB17;GADD45B;FUS;NFKB1;PTK2;PML;GADD45G;IL6;H

;RAF1;RAPGEF6;RGS14;SRC;ADCY4;LPAR3;ADCY8;RASGRP2;ADCY7;RAP1GAP;RAP1B;RALGDS;VASP;FYB1;VEGFC

CB;CYBB;MAPK14;NFKB1;MAPK13;SELP;MAPK11;H2AJ;H3-5;H2BC15;FCGR2A;PIK3CA;PPIF;VDAC3;TLR8;PADI4;

CA;TRAF3;PARD3;MFNG;IRF1;COL6A3;MAML3;ITGA5;ATP6V0D1;RAF1;LLGL1;TLR3;IRF9;IFNAR1;ITGA9;RBPJL;N

;VEGFA;MAPK13;MAPK11;PIK3CA;MDM2;HCLS1;FAS;GRB2;ARHGEF1;KRAS;PTPN6;HPSE;ITGA5;EZR;RAF1;TLR4;

RAF;ACTN4;PTK2;VAV1;VEGFA;PIK3CA;RAPGEF1;ZYX;COL6A3;GRB2;ITGA5;RAF1;BIRC2;BCAR1;BIRC3;ITGA9

EM1;DNAJB12;EIF2AK2;TRAF2;UBE2G1;HSPA2;PDIA6;DNAJC1;DDIT3;DNAJB11;CANX;HYOU1;CALR;ATF4;NFE2L

;SGK1;RAF1;TLR4;ATF4;TLR2;IFNAR1;ITGA9;PRKAA1;TGFA;LPAR3;IL2RG;PIK3R6;RELA;PIK3R5;HSP90B1;BCL2L11

EGFC;ATP2B1;NGF;MCOLN2;TPCN2;VEGFA;P2RX5;GNAL;ADRB3;LTB4R2;ADORA2A;ADORA2B;FGF18;ORAI2;VDA

GAB2;NGF;GNG11;NFKB1;VEGFA;PIK3CA;RASA3;FGF18;RASA2;REL;NF1;GRB2;KRAS;CALM1;RAF1;ARF6

HDAC4;NOTCH3;ROCK1;NOTCH4;PTGS2;SLC7A1;BCL2L11;SOCS1;PLCG2;E2F2;BMF;E2F3;BAK1;MCL1;CREBBP;STA
SPA2;ARAP1;IGF2R;TGFBR1;PML;RNF41;DNM2;SPART;ITCH;ZFYVE16;EHD4;RAB31;PARD3;IL2RA;IL2RB;CHMP2A;

A4A;TNFRSF1A;DKK4;IL1A;PSMA2;DDIT3;IL1B;VDAC3;PPIF;RAF1;SQSTM1;OPTN;ATF4;UBA7;ITPR1;LRP5;ITPR2;PS

FNG;IRF3;TRAF3;OAS2;IL1B;TLR9;IRF7;LTA;SRSF3;SRSF5;ITGA5;BIRC2;TLR3;IRF9;IFNAR1;BIRC3;TLR2;ZNF473;RNA

IF;RAF1;ATF4;ITPR1;LRP5;ITPR2;PSEN1;PTGS2;COX7A1;RELA;MAPK8;ATG101;DVL2;BID;FZD1;XBP1;NOS2;ATP5PI

JS;GPX3;ALYREF;NRG1;TRAF2;TNFRSF1B;MAPK14;TUBA4A;TNFRSF1A;MAPK13;SETX;MAPK11;NUP210L;PSMA2;I

4;TLR2;SRC;ITPR1;LY96;TANK;RELA;HSP90B1;RAP1B;MAPK8;CCL3;NLRP3;CCL2;CD14;BID;LDLR;ABCA1;LYN;JUN;X
D1;NLRC4;NOD2;MEFV;TANK;RELA;MAPK8;IFI16;NLRP3;CCL2;RIPK1;GBP4;GSDMD;JUN;STAT1;STAT2;ERBIN;MA

;EGR2;JUN;EGR3;SMAD3;MAP3K1;SLC10A1;STAT1;STAT2;STAT3;BRAF;MAPK14;NFKB1;MAPK13;NFKBIA;MAPK1

;IFNAR1;RNASEL;RELA;SOCS3;NXF1;NLRP3;CCL2;BAK1;BID;CREBBP;STAT1;MX2;STAT2;MX1;EIF2AK2;NFKB1;PML

L;MAPK8;CLEC7A;IRAK2;CD14;BID;PLK3;CD74;CREBBP;NOS2;VDR;IL10RB;STAT1;IL10RA;MAPK14;NFKB1;MAPK13
;E2F3;HES1;BAK1;BID;LYN;JUN;GADD45B;STAT1;STAT2;STAT3;EIF2AK2;ISG15;MAPK14;NFKB1;GADD45G;NFKB2;
4;CXCR2;PLCG2;CCL3;CCL2;PTK2B;CCR1;LYN;STAT5B;XCR1;CCL20;STAT1;STAT2;STAT3;BRAF;GNG11;NFKB1;PTK2;
AR1;RELA;C2;C3;MAPK8;C7;PLCG2;NLRP3;CCL2;RPL18;JUN;STAT1;STAT2;MX2;STAT3;MX1;EIF2AK2;ISG15;MAPK

AP;MAPK8;STAT4;NLRP3;RIPK1;STAT6;BID;STAT5A;STAT5B;MLKL;STAT1;STAT2;STAT3;EIF2AK2;CFLAR;SPATA2L;C
NG4;PLCG2;E2F2;E2F3;BAK1;BID;CCR1;LYN;TCF7L2;CREBBP;JUN;STAT1;STAT2;STAT3;EIF2AK2;MAPK14;GNG11;
CXCR2;CCL4;CCL3;CCL2;E2F2;PTK2B;RIPK1;E2F3;BAK1;BID;CCR1;IL10RB;IL10RA;STAT3;MAPK14;GNG11;PTK2;NF

STAT5B;CREBBP;JUN;SMAD3;GADD45B;BRAF;GNG11;NFKB1;PTK2;NFKB2;GADD45G;NFKBIA;IL6;FGF18;MDM2;G

FRSF1A;IL1A;IFNG;IL23A;IL1B;LTA;LTB;IL6ST;IFNAR1;CCL14;IL20RA;CXCR4;CSF2RB;ACVR1B;IL2RG;CCL8;CXCR1;CX
1;ROCK1;SRC;PXN;ITPR1;ITPR2;NOD1;NLRC4;RELA;MALT1;U2AF1L4;C3;MAPK8;CYTH4;PLCG2;NLRP3;RIPK1;CD1

RC4;RELA;HSP90B1;PAK1;MAPK8;CYTH4;NLRP3;SNX9;RIPK1;CD14;BAK1;CYTH1;GSDMD;DYNC1H1;TCF7L2;JUN;M
FA;CACNA1A;NLK;RASGRP2;STK4;RELA;RASGRP4;RELB;STK3;RAP1B;PAK1;MAPK8;MKNK1;CD14;MAP4K4;MAP3K

4B;MAPK14;NFKB1;MAPK13;NFKBIA;MAPK11;IL6;FCGR2A;ABI1;FAS;TAB2;PTPN6;MYD88;MYO1F;NFKBIB

OTCH3;NOTCH4;PXN;PSEN1;PTGS2;RELA;SLC9A3R1;PPP2R3C;DVL2;HES1;BAK1;HES4;FZD1;TCF7L2;CREBBP;LAMB

;GNG4;PPP2R3C;EIF4E;MCL1;LAMB3;OSM;VEGFC;GNG11;PTK2;NFKB1;VEGFA;NR4A1;IL6;IL2RA;FGF18;IL2RB;MD

SEN1;PTGS2;UBE2J2;TANK;COX7A1;RELA;UBE2J1;ATXN3;MAPK8;ATXN1;ATG101;DVL2;BAK1;BID;FZD1;XBP1;DNA

SEL;SRC;CFP;RELA;C3;SOCS3;NXF1;CCL2;ZNF304;BAK1;BID;ZNF300;CD74;TNFSF14;STAT1;STAT2;ALYREF;EIF2AK2

BP1;HSPA5;STAT3;HSPA2;POU2F2;MAPK14;SOD2;SELE;PTK2;NFKB1;MAPK13;NFKBIA;SELP;MAPK11;IL6;FAS;TAB

RB2;CALM1;IL7R;NFE2L2;RET;CDKN1A;CDKN1B;CXCL8;WNT2B;FLT4;SLC2A1;FASLG;GLI1;IKBKB;CASP7;CASP8;CA

CR2;CCL4;CCL3;IL21R;CCL2;RELT;CCR1;XCR1;TNFSF14;CCL20;IL10RB;IL10RA;LIF;OSM;CXCL10;CXCL11;IL6;IL2RA;I

4;CYTH1;JUN;MAPK14;PTK2;NFKB1;MAPK13;NFKBIA;MAPK11;H3-5;TNIP1;ATG16L1;MDM2;TAB2;MYD88;BCAR1

K2;JUN;MAP3K1;GADD45B;VEGFC;BRAF;HSPA2;MAPK14;NFKB1;GADD45G;VEGFA;NFKB2;MAPK13;NR4A1;MAPK

AH17;HSPA5;NOS2;FUS;ATP5PB;SEPTIN5;BRAF;UBE2G1;MAPK14;NFKB1;MAPK13;MAPK11;IL6;FAS;KRAS;CALM1;

SP3;RAC2;ITGAV;JAK2;JAK3;JAK1;NCOA1;ARHGEF11;EDN1;IFNGR1;IL15;MMP1;IFNGR2;NCOA4;TRAF2;FOS;TRAF

1;TGFBR1;PIK3CA;IFNG;TRAF3;RARA;ARHGEF1;BIRC2;PPARD;BIRC3;ROCK1;PTGER2;ADCY4;LRP5;CXCR4;XIAP;AD

CY8;PTGS2;IL2RG;ADCY7;DLL1;RELA;MAPK8;GNG4;STAT4;E2F2;STAT6;E2F3;HES1;BAK1;BID;TCF7L2;EGLN3;LAM[illegible]

B3;NOS2;STAT1;STAT2;STAT3;VEGFC;PML;VEGFA;IL2RA;IL2RB;GSTA1;FAS;KRAS

**Supplementary File S6: Comprehensive details of the results of functional enrichment an**

| BP | Term | Overlap | P-value | Adjusted P-value |
|---|---|---|---|---|
| | fatty acid catabolic process (GO:0009062) | 37/70 | 2.76E-17 | 1.25E-13 |
| | cilium assembly (GO:0060271) | 87/314 | 1.90E-15 | 2.96E-12 |
| | DNA repair (GO:0006281) | 84/298 | 1.96E-15 | 2.96E-12 |
| | fatty acid beta-oxidation (GO:0006635) | 29/52 | 1.16E-14 | 1.30E-11 |
| | interstrand cross-link repair (GO:0036297) | 27/55 | 5.47E-12 | 4.94E-09 |
| | DNA metabolic process (GO:0006259) | 72/277 | 1.43E-11 | 1.07E-08 |
| | cilium organization (GO:0044782) | 62/228 | 4.90E-11 | 3.16E-08 |
| | recombinational repair (GO:0000725) | 31/79 | 2.02E-10 | 1.10E-07 |
| | double-strand break repair via homologous r | 35/97 | 2.18E-10 | 1.10E-07 |
| | fatty acid oxidation (GO:0019395) | 26/59 | 2.81E-10 | 1.27E-07 |
| | double-strand break repair (GO:0006302) | 47/164 | 1.62E-09 | 6.67E-07 |
| | intraciliary transport involved in cilium assem | 20/40 | 2.06E-09 | 7.77E-07 |
| | plasma membrane bounded cell projection a | 67/278 | 2.28E-09 | 7.92E-07 |
| | cellular response to DNA damage stimulus (G | 78/350 | 5.28E-09 | 1.70E-06 |
| | base-excision repair (GO:0006284) | 21/48 | 1.70E-08 | 5.12E-06 |
| | cellular amide metabolic process (GO:00436( | 35/115 | 3.62E-08 | 1.02E-05 |
| | base-excision repair, gap-filling (GO:0006287 | 17/35 | 5.86E-08 | 1.56E-05 |
| | ciliary basal body-plasma membrane docking | 30/95 | 1.35E-07 | 3.40E-05 |
| | short-chain fatty acid metabolic process (GO: | 44782 | 2.42E-07 | 5.76E-05 |
| | DNA replication (GO:0006260) | 31/108 | 8.96E-07 | 2.02E-04 |
| | regulation of cell cycle G2/M phase transitior | 26/85 | 1.79E-06 | 3.86E-04 |
| | protein localization to ciliary transition zone ( | 44718 | 2.28E-06 | 4.61E-04 |
| | nucleotide-excision repair, DNA incision (GO: | 16/39 | 2.45E-06 | 4.61E-04 |
| | replication fork processing (GO:0031297) | 16/39 | 2.45E-06 | 4.61E-04 |
| | branched-chain amino acid catabolic process | 44885 | 2.79E-06 | 4.80E-04 |
| | branched-chain amino acid metabolic proces | 44885 | 2.79E-06 | 4.80E-04 |
| | organelle assembly (GO:0070925) | 81/425 | 2.87E-06 | 4.80E-04 |
| | DNA-dependent DNA replication (GO:00062€ | 33/129 | 6.57E-06 | 0.00106058 |
| | fatty acid metabolic process (GO:0006631) | 32/124 | 7.40E-06 | 0.001153151 |
| | fatty acid beta-oxidation using acyl-CoA oxida | 44819 | 8.96E-06 | 0.001348763 |
| | mitotic cell cycle phase transition (GO:00447 | 46/209 | 9.78E-06 | 0.001424826 |
| | nucleotide-excision repair (GO:0006289) | 28/105 | 1.39E-05 | 0.001965622 |
| | nucleobase-containing small molecule interc | 44922 | 1.69E-05 | 0.002313987 |
| | cellular amino acid catabolic process (GO:00( | 25/90 | 1.85E-05 | 0.002430527 |
| | glucuronate catabolic process (GO:0006064) | 44686 | 1.99E-05 | 0.002430527 |
| | glucuronate catabolic process to xylulose 5-p | 44686 | 1.99E-05 | 0.002430527 |
| | xylulose 5-phosphate biosynthetic process (G | 44686 | 1.99E-05 | 0.002430527 |
| | pyrimidine deoxyribonucleotide catabolic prc | 44786 | 2.26E-05 | 0.002519851 |
| | fatty acid beta-oxidation using acyl-CoA dehy | 44752 | 2.29E-05 | 0.002519851 |
| | myelin assembly (GO:0032288) | 44752 | 2.29E-05 | 0.002519851 |
| | telomeric loop disassembly (GO:0090657) | 44752 | 2.29E-05 | 0.002519851 |
| | nucleotide-excision repair, DNA incision, 5'-tc | 14/37 | 3.11E-05 | 0.003294719 |
| | DNA duplex unwinding (GO:0032508) | 17/51 | 3.14E-05 | 0.003294719 |
| | DNA-dependent DNA replication maintenanc | 15/43 | 4.98E-05 | 0.005103993 |

| | | | |
|---|---|---|---|
| depyrimidination (GO:0045008) | 44720 | 5.20E-05 | 0.005103993 |
| short-chain fatty acid catabolic process (GO:( | 44720 | 5.20E-05 | 0.005103993 |
| regulation of G2/M transition of mitotic cell ( | 34/149 | 6.30E-05 | 0.005969413 |
| monocarboxylic acid metabolic process (GO:( | 33/143 | 6.34E-05 | 0.005969413 |
| protein localization to cilium (GO:0061512) | 13/35 | 7.47E-05 | 0.006889515 |
| regulation of sequestering of triglyceride (GC | 44788 | 9.12E-05 | 0.008104908 |
| water-soluble vitamin metabolic process (GO | 21/76 | 9.15E-05 | 0.008104908 |
| nucleotide-sugar biosynthetic process (GO:0( | 44823 | 1.07E-04 | 0.009335882 |
| hexose metabolic process (GO:0019318) | 14/41 | 1.15E-04 | 0.009824897 |
| telomere maintenance (GO:0000723) | 17/56 | 1.19E-04 | 0.00993236 |
| protein N-linked glycosylation (GO:0006487) | 19/67 | 1.34E-04 | 0.01077101 |
| G2/M transition of mitotic cell cycle (GO:000 | 30/130 | 1.34E-04 | 0.01077101 |
| biotin metabolic process (GO:0006768) | 44721 | 1.41E-04 | 0.01077101 |
| positive regulation of DNA-directed DNA poly | 44721 | 1.41E-04 | 0.01077101 |
| regulation of DNA-directed DNA polymerase | 44721 | 1.41E-04 | 0.01077101 |
| cell cycle G2/M phase transition (GO:004483 | 30/131 | 1.55E-04 | 0.011641678 |
| regulation of mitotic cell cycle phase transitic | 39/188 | 1.72E-04 | 0.012720089 |
| glutathione metabolic process (GO:0006749) | 14/43 | 2.05E-04 | 0.014967367 |
| mismatch repair (GO:0006298) | 44894 | 2.15E-04 | 0.015406286 |
| nucleic acid phosphodiester bond hydrolysis | 20/75 | 2.26E-04 | 0.015948073 |
| intraciliary retrograde transport (GO:003572 | 44755 | 2.39E-04 | 0.016596916 |
| acyl-CoA biosynthetic process (GO:0071616) | 44790 | 2.79E-04 | 0.019078137 |
| cellular response to leucine starvation (GO:1! | 44722 | 3.17E-04 | 0.021399395 |
| mitotic spindle organization (GO:0007052) | 33/157 | 4.08E-04 | 0.027104032 |
| DNA strand elongation (GO:0022616) | 44791 | 4.51E-04 | 0.029549131 |
| organonitrogen compound biosynthetic proc | 33/158 | 4.60E-04 | 0.029677461 |
| monocarboxylic acid biosynthetic process (G( | 17/63 | 5.61E-04 | 0.035701239 |
| long-chain fatty acid transport (GO:0015909) | 11994 | 5.78E-04 | 0.036193618 |
| sphingolipid metabolic process (GO:0006665 | 26/116 | 5.85E-04 | 0.036193618 |
| protein glycosylation (GO:0006486) | 28/130 | 7.22E-04 | 0.044070059 |
| lipid transport across blood-brain barrier (GC | 44656 | 7.88E-04 | 0.046624772 |
| folic acid transport (GO:0015884) | 44689 | 8.26E-04 | 0.046624772 |
| glycoside catabolic process (GO:0016139) | 44689 | 8.26E-04 | 0.046624772 |
| regulation of acetyl-CoA biosynthetic process | 44689 | 8.26E-04 | 0.046624772 |
| regulation of acyl-CoA biosynthetic process (( | 44689 | 8.26E-04 | 0.046624772 |
| telomeric D-loop disassembly (GO:0061820) | 44689 | 8.26E-04 | 0.046624772 |
| nucleotide-excision repair, DNA gap filling (G | 44828 | 8.91E-04 | 0.049680693 |
| nucleotide-excision repair, preincision compl | 44863 | 9.95E-04 | 0.054843374 |
| DNA recombination (GO:0006310) | 14580 | 0.001019996 | 0.054861192 |
| DNA replication-independent nucleosome as | 14580 | 0.001019996 | 0.054861192 |
| fatty acid transport (GO:0015908) | 44793 | 0.001051912 | 0.055912234 |
| carboxylic acid catabolic process (GO:004639 | 44724 | 0.001138084 | 0.059101858 |
| carnitine metabolic process (GO:0009437) | 44724 | 0.001138084 | 0.059101858 |
| non-motile cilium assembly (GO:1905515) | 44864 | 0.001340974 | 0.068846816 |
| mitotic nuclear division (GO:0140014) | 18/74 | 0.001469567 | 0.074302376 |
| error-prone translesion synthesis (GO:00422 | 44794 | 0.001529465 | 0.074302376 |
| intracellular lipid transport (GO:0032365) | 44794 | 0.001529465 | 0.074302376 |

| | | | |
|---|---|---|---|
| nucleotide-excision repair, DNA incision, 3'-t( | 44794 | 0.001529465 | 0.074302376 |
| nucleotide-excision repair, preincision compl | 44794 | 0.001529465 | 0.074302376 |
| L-phenylalanine metabolic process (GO:0006 | 44690 | 0.001681744 | 0.0799802 |
| phosphatidylglycerol biosynthetic process (G | 44690 | 0.001681744 | 0.0799802 |
| DNA modification (GO:0006304) | 44830 | 0.001720341 | 0.080963563 |
| establishment of protein localization to plasn | 13455 | 0.001745659 | 0.081308125 |
| histone H2A monoubiquitination (GO:003551 | 44759 | 0.001784707 | 0.082278631 |
| carboxylic acid biosynthetic process (GO:004 | 44725 | 0.00190892 | 0.086245023 |
| UV-damage excision repair (GO:0070914) | 44725 | 0.00190892 | 0.086245023 |
| regulation of DNA recombination (GO:00000 | 15676 | 0.002080826 | 0.088870052 |
| attachment of GPI anchor to protein (GO:001 | 44657 | 0.002148854 | 0.088870052 |
| N-acetylneuraminate catabolic process (GO:( | 44657 | 0.002148854 | 0.088870052 |
| negative regulation of sequestering of triglyc | 44657 | 0.002148854 | 0.088870052 |
| regulation of protein localization to cilium (G | 44657 | 0.002148854 | 0.088870052 |
| regulation of t-circle formation (GO:1904429 | 44657 | 0.002148854 | 0.088870052 |
| glutathione derivative biosynthetic process (( | 44795 | 0.002163724 | 0.088870052 |
| glutathione derivative metabolic process (GC | 44795 | 0.002163724 | 0.088870052 |
| nucleotide-excision repair, DNA duplex unwir | 44795 | 0.002163724 | 0.088870052 |
| protein mannosylation (GO:0035268) | 44795 | 0.002163724 | 0.088870052 |
| positive regulation of cytokinesis (GO:00324( | 13820 | 0.002229948 | 0.090764908 |
| DNA damage checkpoint signaling (GO:00000 | 11963 | 0.002323579 | 0.093214386 |
| Golgi to plasma membrane transport (GO:00 | 13/48 | 0.002341162 | 0.093214386 |
| peptide metabolic process (GO:0006518) | 19/83 | 0.002352023 | 0.093214386 |
| DNA strand elongation involved in DNA replic | 44760 | 0.002633253 | 0.101765675 |
| protein O-linked mannosylation (GO:003526 | 44760 | 0.002633253 | 0.101765675 |
| microtubule cytoskeleton organization involv | 26/128 | 0.002635366 | 0.101765675 |
| DNA replication initiation (GO:0006270) | 14185 | 0.002817607 | 0.106974367 |
| histone exchange (GO:0043486) | 14185 | 0.002817607 | 0.106974367 |
| very long-chain fatty acid metabolic process ( | 12328 | 0.002995546 | 0.107519584 |
| cardiolipin metabolic process (GO:0032048) | 44726 | 0.003017953 | 0.107519584 |
| ribonucleoside monophosphate biosynthetic | 44726 | 0.003017953 | 0.107519584 |
| cellular response to acidic pH (GO:0071468) | 44691 | 0.00304615 | 0.107519584 |
| cellular response to oxidised low-density lipo | 44691 | 0.00304615 | 0.107519584 |
| mitotic DNA replication (GO:1902969) | 44691 | 0.00304615 | 0.107519584 |
| N-acetylneuraminate metabolic process (GO: | 44691 | 0.00304615 | 0.107519584 |
| regulation of glycoprotein biosynthetic proce | 44691 | 0.00304615 | 0.107519584 |
| serine family amino acid catabolic process (G | 44691 | 0.00304615 | 0.107519584 |
| positive regulation of cell division (GO:00517 | 16407 | 0.003194405 | 0.111878451 |
| phosphatidic acid metabolic process (GO:004 | 13/50 | 0.003462302 | 0.120328327 |
| fatty acid transmembrane transport (GO:190 | 44761 | 0.003760206 | 0.128701598 |
| regulation of centriole replication (GO:00465 | 44761 | 0.003760206 | 0.128701598 |
| sister chromatid segregation (GO:0000819) | 12693 | 0.003813673 | 0.12955017 |
| cellular response to oxidative stress (GO:003 | 25/125 | 0.003898866 | 0.131455795 |
| dicarboxylic acid transport (GO:0006835) | 44833 | 0.004011886 | 0.133091094 |
| hydrogen peroxide metabolic process (GO:0C | 44833 | 0.004011886 | 0.133091094 |
| peptidyl-glutamic acid modification (GO:0018 | 44797 | 0.004035741 | 0.133091094 |
| small GTPase mediated signal transduction (( | 22/106 | 0.004111353 | 0.134602112 |

| | | | |
|---|---|---|---|
| dicarboxylic acid metabolic process (GO:0043 | 14/57 | 0.004320163 | 0.140085944 |
| organonitrogen compound catabolic process | 15/63 | 0.004387231 | 0.140085944 |
| mitotic recombination (GO:0006312) | 44727 | 0.004545362 | 0.140085944 |
| response to acidic pH (GO:0010447) | 44727 | 0.004545362 | 0.140085944 |
| ubiquinone metabolic process (GO:0006743) | 44727 | 0.004545362 | 0.140085944 |
| amino sugar catabolic process (GO:0046348) | 44658 | 0.004557909 | 0.140085944 |
| cardiolipin biosynthetic process (GO:003204‹ | 44658 | 0.004557909 | 0.140085944 |
| regulation of vascular associated smooth mu | 44658 | 0.004557909 | 0.140085944 |
| xenobiotic catabolic process (GO:0042178) | 44658 | 0.004557909 | 0.140085944 |
| positive regulation of cell cycle process (GO:( | 21/101 | 0.004872614 | 0.148284802 |
| glycolipid metabolic process (GO:0006664) | 13/52 | 0.004983824 | 0.148284802 |
| formation of extrachromosomal circular DNA | 44692 | 0.005059434 | 0.148284802 |
| ketone body metabolic process (GO:1902224 | 44692 | 0.005059434 | 0.148284802 |
| t-circle formation (GO:0090656) | 44692 | 0.005059434 | 0.148284802 |
| telomere maintenance via telomere trimming | 44692 | 0.005059434 | 0.148284802 |
| UDP-N-acetylglucosamine biosynthetic proce | 44692 | 0.005059434 | 0.148284802 |
| membrane lipid biosynthetic process (GO:00‹ | 14/58 | 0.005105213 | 0.148284802 |
| CENP-A containing chromatin organization (G | 44834 | 0.005156566 | 0.148284802 |
| CENP-A containing nucleosome assembly (GC | 44834 | 0.005156566 | 0.148284802 |
| base-excision repair, AP site formation (GO:0 | 44762 | 0.005218522 | 0.148284802 |
| cellular modified amino acid biosynthetic prc | 44762 | 0.005218522 | 0.148284802 |
| telomere organization (GO:0032200) | 17502 | 0.005720406 | 0.16152997 |
| organic substance transport (GO:0071702) | 26/136 | 0.006136363 | 0.172199309 |
| phosphate-containing compound metabolic p | 37/212 | 0.006202729 | 0.172987207 |
| translesion synthesis (GO:0019985) | 15646 | 0.006520379 | 0.175743114 |
| phosphatidylethanolamine metabolic proces: | 11567 | 0.006538655 | 0.175743114 |
| purine nucleoside bisphosphate metabolic pr | 11567 | 0.006538655 | 0.175743114 |
| ribonucleoside bisphosphate metabolic proce | 11567 | 0.006538655 | 0.175743114 |
| DNA unwinding involved in DNA replication (( | 44728 | 0.006573835 | 0.175743114 |
| nuclear transport (GO:0051169) | 44728 | 0.006573835 | 0.175743114 |
| nucleoside diphosphate metabolic process (G | 44728 | 0.006573835 | 0.175743114 |
| cellular response to ionizing radiation (GO:00 | 17868 | 0.006846823 | 0.180900273 |
| phosphatidic acid biosynthetic process (GO:0 | 17868 | 0.006846823 | 0.180900273 |
| DNA geometric change (GO:0032392) | 44799 | 0.006957664 | 0.181703629 |
| global genome nucleotide-excision repair (GC | 44799 | 0.006957664 | 0.181703629 |
| proton transmembrane transport (GO:19026 | 13789 | 0.007359585 | 0.191095435 |
| amino sugar biosynthetic process (GO:00463 | 44693 | 0.007860407 | 0.199799129 |
| glycine metabolic process (GO:0006544) | 44693 | 0.007860407 | 0.199799129 |
| purine nucleoside monophosphate biosynthe | 44693 | 0.007860407 | 0.199799129 |
| DNA synthesis involved in DNA repair (GO:00 | 16011 | 0.007871679 | 0.199799129 |
| protein transport (GO:0015031) | 58/369 | 0.008177276 | 0.202461342 |
| chromatin remodeling at centromere (GO:00 | 11933 | 0.008188036 | 0.202461342 |
| diacylglycerol biosynthetic process (GO:0006 | 44659 | 0.00829025 | 0.202461342 |
| negative regulation of macrophage migratior | 44659 | 0.00829025 | 0.202461342 |
| negative regulation of sodium ion transport ( | 44659 | 0.00829025 | 0.202461342 |
| negative regulation of vascular associated sm | 44659 | 0.00829025 | 0.202461342 |
| postsynapse organization (GO:0099173) | 44659 | 0.00829025 | 0.202461342 |

| | | | |
|---|---|---|---|
| sphingolipid biosynthetic process (GO:0030148 | 16/74 | 0.008897801 | 0.215272937 |
| bile acid biosynthetic process (GO:0006699) | 44800 | 0.008910146 | 0.215272937 |
| negative regulation of G0 to G1 transition (G | 14154 | 0.008981723 | 0.215848007 |
| dolichol-linked oligosaccharide biosynthetic p | 44729 | 0.009186093 | 0.219591373 |
| error-free translesion synthesis (GO:0070987 | 44764 | 0.009349416 | 0.22231927 |
| cellular protein modification process (GO:000 | 142/1025 | 0.009598322 | 0.227043019 |
| bile acid metabolic process (GO:0008206) | 12298 | 0.010135224 | 0.238494493 |
| cellular response to chemical stress (GO:0062 | 20/101 | 0.010245178 | 0.23983272 |
| DNA integrity checkpoint signaling (GO:00315 | 14519 | 0.010863764 | 0.25300251 |
| amide transport (GO:0042886) | 44801 | 0.011243917 | 0.259183763 |
| carbohydrate derivative biosynthetic process | 44801 | 0.011243917 | 0.259183763 |
| oligosaccharide-lipid intermediate biosynthet | 44694 | 0.011580095 | 0.260915345 |
| UDP-N-acetylglucosamine metabolic process | 44694 | 0.011580095 | 0.260915345 |
| nucleotide metabolic process (GO:0009117) | 44765 | 0.012132809 | 0.260915345 |
| nucleotide-excision repair, DNA damage reco | 44765 | 0.012132809 | 0.260915345 |
| cellular response to pH (GO:0071467) | 44730 | 0.012462521 | 0.260915345 |
| regulation of establishment or maintenance ( | 44730 | 0.012462521 | 0.260915345 |
| response to axon injury (GO:0048678) | 44730 | 0.012462521 | 0.260915345 |
| amino-acid betaine biosynthetic process (GO | 44625 | 0.012647291 | 0.260915345 |
| carnitine biosynthetic process (GO:0045329) | 44625 | 0.012647291 | 0.260915345 |
| DNA replication, synthesis of RNA primer (GC | 44625 | 0.012647291 | 0.260915345 |
| double-strand break repair via synthesis-dep | 44625 | 0.012647291 | 0.260915345 |
| erythrose 4-phosphate/phosphoenolpyruvate | 44625 | 0.012647291 | 0.260915345 |
| ethanolamine-containing compound metabo | 44625 | 0.012647291 | 0.260915345 |
| fatty acid derivative catabolic process (GO:19 | 44625 | 0.012647291 | 0.260915345 |
| folate transmembrane transport (GO:009883 | 44625 | 0.012647291 | 0.260915345 |
| L-phenylalanine catabolic process (GO:00065 | 44625 | 0.012647291 | 0.260915345 |
| leucine catabolic process (GO:0006552) | 44625 | 0.012647291 | 0.260915345 |
| N-acylethanolamine metabolic process (GO:0 | 44625 | 0.012647291 | 0.260915345 |
| negative regulation of macrophage chemotax | 44625 | 0.012647291 | 0.260915345 |
| positive regulation of dendritic cell differenti | 44625 | 0.012647291 | 0.260915345 |
| positive regulation of DNA ligation (GO:0051 | 44625 | 0.012647291 | 0.260915345 |
| positive regulation of protein localization to ( | 44625 | 0.012647291 | 0.260915345 |
| regulation of inflammatory response to wour | 44625 | 0.012647291 | 0.260915345 |
| phosphorylation (GO:0016310) | 61/400 | 0.012766164 | 0.262170588 |
| regulation of protein kinase activity (GO:0045 | 21/110 | 0.013058547 | 0.263266958 |
| signal transduction in response to DNA dama | 19329 | 0.013188814 | 0.263266958 |
| exonucleolytic trimming involved in rRNA pro | 44660 | 0.01357707 | 0.263266958 |
| exonucleolytic trimming to generate mature | 44660 | 0.01357707 | 0.263266958 |
| fatty acid alpha-oxidation (GO:0001561) | 44660 | 0.01357707 | 0.263266958 |
| galactose metabolic process (GO:0006012) | 44660 | 0.01357707 | 0.263266958 |
| ketone body biosynthetic process (GO:00469 | 44660 | 0.01357707 | 0.263266958 |
| L-ascorbic acid metabolic process (GO:00198 | 44660 | 0.01357707 | 0.263266958 |
| N-acylphosphatidylethanolamine metabolic p | 44660 | 0.01357707 | 0.263266958 |
| nucleoside diphosphate phosphorylation (GC | 44660 | 0.01357707 | 0.263266958 |
| nucleoside triphosphate biosynthetic process | 44660 | 0.01357707 | 0.263266958 |
| nucleotide phosphorylation (GO:0046939) | 44660 | 0.01357707 | 0.263266958 |

| | | | |
|---|---|---|---|
| positive regulation of sequestering of triglyce | 44660 | 0.01357707 | 0.263266958 |
| regulation of cytokinesis (GO:0032465) | 17/84 | 0.013837236 | 0.267165091 |
| transition metal ion homeostasis (GO:005507 | 44802 | 0.013998982 | 0.269137866 |
| platelet degranulation (GO:0002576) | 23/125 | 0.01491913 | 0.285612841 |
| negative regulation of organelle assembly (G | 13028 | 0.015045952 | 0.286825362 |
| regulation of G0 to G1 transition (GO:007031 | 15250 | 0.015505089 | 0.293994873 |
| glycosphingolipid metabolic process (GO:000 | 17472 | 0.015552186 | 0.293994873 |
| ether lipid metabolic process (GO:0046485) | 44695 | 0.016336852 | 0.300040228 |
| nucleoside phosphate biosynthetic process (( | 44695 | 0.016336852 | 0.300040228 |
| nucleoside triphosphate metabolic process (( | 44695 | 0.016336852 | 0.300040228 |
| pre-miRNA processing (GO:0031054) | 44695 | 0.016336852 | 0.300040228 |
| protein localization to vacuole (GO:0072665) | 44695 | 0.016336852 | 0.300040228 |
| quinone biosynthetic process (GO:1901663) | 44695 | 0.016336852 | 0.300040228 |
| respiratory electron transport chain (GO:002 | 44695 | 0.016336852 | 0.300040228 |
| sphingosine metabolic process (GO:0006670) | 44731 | 0.016478981 | 0.300209829 |
| telomere capping (GO:0016233) | 44731 | 0.016478981 | 0.300209829 |
| Golgi to plasma membrane protein transport | 44803 | 0.017214411 | 0.312348223 |
| transcription-coupled nucleotide-excision rep | 15/73 | 0.017515908 | 0.316547498 |
| telomere maintenance via telomere lengther | 13394 | 0.018070209 | 0.325263759 |
| glycerophospholipid metabolic process (GO:0 | 16/80 | 0.018495147 | 0.330652233 |
| anion transmembrane transport (GO:009865 | 14/67 | 0.01851594 | 0.330652233 |
| glycerophospholipid biosynthetic process (G( | 30/177 | 0.018728048 | 0.333123313 |
| peroxisome organization (GO:0007031) | 44767 | 0.019402744 | 0.342289081 |
| iron ion homeostasis (GO:0055072) | 13/61 | 0.019470627 | 0.342289081 |
| positive regulation of DNA biosynthetic proce | 13/61 | 0.019470627 | 0.342289081 |
| regulation of mitotic cell cycle (GO:0007346) | 30/178 | 0.020126271 | 0.351171338 |
| acetyl-CoA biosynthetic process (GO:000608! | 44661 | 0.020597699 | 0.351171338 |
| glycosphingolipid catabolic process (GO:0046 | 44661 | 0.020597699 | 0.351171338 |
| long-chain fatty acid import into cell (GO:004 | 44661 | 0.020597699 | 0.351171338 |
| membrane lipid catabolic process (GO:00464 | 44661 | 0.020597699 | 0.351171338 |
| removal of superoxide radicals (GO:0019430) | 44661 | 0.020597699 | 0.351171338 |
| rRNA 3'-end processing (GO:0031125) | 44661 | 0.020597699 | 0.351171338 |
| UV protection (GO:0009650) | 44661 | 0.020597699 | 0.351171338 |
| cellular nitrogen compound biosynthetic pro( | 44804 | 0.020927722 | 0.352803916 |
| regulation of cellular senescence (GO:200077 | 44804 | 0.020927722 | 0.352803916 |
| steroid hormone biosynthetic process (GO:0: | 44804 | 0.020927722 | 0.352803916 |
| folic acid-containing compound metabolic pr( | 44732 | 0.021304901 | 0.356026906 |
| microtubule nucleation (GO:0007020) | 44732 | 0.021304901 | 0.356026906 |
| acyl-CoA metabolic process (GO:0006637) | 13759 | 0.021512914 | 0.356026906 |
| centromere complex assembly (GO:0034508) | 13759 | 0.021512914 | 0.356026906 |
| regulation of vascular associated smooth mu | 13759 | 0.021512914 | 0.356026906 |
| protein phosphorylation (GO:0006468) | 72/496 | 0.021597291 | 0.35611884 |
| medium-chain fatty acid metabolic process (( | 44696 | 0.022232608 | 0.356902842 |
| regulation of establishment of protein localiz | 44696 | 0.022232608 | 0.356902842 |
| ubiquinone biosynthetic process (GO:000674 | 44696 | 0.022232608 | 0.356902842 |
| protein O-linked glycosylation (GO:0006493) | 19/102 | 0.022436798 | 0.356902842 |
| lipid transport (GO:0006869) | 20/109 | 0.022737234 | 0.356902842 |

| | | | |
|---|---|---|---|
| cellular carbohydrate catabolic process (GO:( | 44626 | 0.023145758 | 0.356902842 |
| cellular response to X-ray (GO:0071481) | 44626 | 0.023145758 | 0.356902842 |
| erythrose 4-phosphate/phosphoenolpyruvat( | 44626 | 0.023145758 | 0.356902842 |
| galactose catabolic process (GO:0019388) | 44626 | 0.023145758 | 0.356902842 |
| GDP-mannose metabolic process (GO:00196 | 44626 | 0.023145758 | 0.356902842 |
| glycoside metabolic process (GO:0016137) | 44626 | 0.023145758 | 0.356902842 |
| glycosyl compound catabolic process (GO:19( | 44626 | 0.023145758 | 0.356902842 |
| histone-serine phosphorylation (GO:0035404 | 44626 | 0.023145758 | 0.356902842 |
| isoleucine metabolic process (GO:0006549) | 44626 | 0.023145758 | 0.356902842 |
| negative regulation of lamellipodium organiz | 44626 | 0.023145758 | 0.356902842 |
| purine nucleobase biosynthetic process (GO:( | 44626 | 0.023145758 | 0.356902842 |
| regulation of cell cycle checkpoint (GO:19019 | 44626 | 0.023145758 | 0.356902842 |
| regulation of hydrogen peroxide metabolic p | 44626 | 0.023145758 | 0.356902842 |
| regulation of peptidyl-cysteine S-nitrosylatioı | 44626 | 0.023145758 | 0.356902842 |
| negative regulation of receptor-mediated en( | 44768 | 0.023987781 | 0.368628548 |
| iron ion transport (GO:0006826) | 18568 | 0.024254961 | 0.371470902 |
| phospholipid metabolic process (GO:000664 | 15/76 | 0.024704813 | 0.377082254 |
| GPI anchor biosynthetic process (GO:000650 | 11902 | 0.02517429 | 0.382954351 |
| hydrogen peroxide catabolic process (GO:004 | 44733 | 0.027001647 | 0.406091332 |
| sterol transport (GO:0015918) | 44733 | 0.027001647 | 0.406091332 |
| vacuolar acidification (GO:0007035) | 44733 | 0.027001647 | 0.406091332 |
| cellular response to UV (GO:0034644) | 15/77 | 0.02753235 | 0.406091332 |
| purine ribonucleotide metabolic process (GO | 15/77 | 0.02753235 | 0.406091332 |
| cholesterol transport (GO:0030301) | 18933 | 0.027818858 | 0.406091332 |
| intrinsic apoptotic signaling pathway in respc | 18933 | 0.027818858 | 0.406091332 |
| lipid homeostasis (GO:0055088) | 13/64 | 0.028228691 | 0.406091332 |
| microtubule bundle formation (GO:0001578) | 16711 | 0.028963314 | 0.406091332 |
| monocarboxylic acid catabolic process (GO:0 | 44769 | 0.02926496 | 0.406091332 |
| negative regulation of plasma membrane bou | 44769 | 0.02926496 | 0.406091332 |
| peroxisomal membrane transport (GO:00159 | 44769 | 0.02926496 | 0.406091332 |
| ceramide catabolic process (GO:0046514) | 44697 | 0.029350243 | 0.406091332 |
| heme catabolic process (GO:0042167) | 44697 | 0.029350243 | 0.406091332 |
| negative regulation of telomere maintenance | 44697 | 0.029350243 | 0.406091332 |
| porphyrin-containing compound catabolic pr( | 44697 | 0.029350243 | 0.406091332 |
| purine ribonucleoside monophosphate biosy | 44697 | 0.029350243 | 0.406091332 |
| regulation of cellular carbohydrate metabolic | 44697 | 0.029350243 | 0.406091332 |
| regulation of ceramide biosynthetic process ( | 44697 | 0.029350243 | 0.406091332 |
| regulation of establishment of cell polarity (G | 44697 | 0.029350243 | 0.406091332 |
| regulation of fatty acid oxidation (GO:004632 | 44697 | 0.029350243 | 0.406091332 |
| resolution of meiotic recombination interme( | 44697 | 0.029350243 | 0.406091332 |
| cellular glucan metabolic process (GO:00060 | 44662 | 0.029476725 | 0.406091332 |
| cellular response to superoxide (GO:0071451 | 44662 | 0.029476725 | 0.406091332 |
| central nervous system myelination (GO:002 | 44662 | 0.029476725 | 0.406091332 |
| negative regulation of centrosome duplicatio | 44662 | 0.029476725 | 0.406091332 |
| nuclear DNA replication (GO:0033260) | 44662 | 0.029476725 | 0.406091332 |
| positive regulation of ceramide biosynthetic ( | 44662 | 0.029476725 | 0.406091332 |
| positive regulation of sphingolipid biosynthet | 44662 | 0.029476725 | 0.406091332 |

| | | | |
|---|---|---|---|
| telomere maintenance via recombination (G( | 44662 | 0.029476725 | 0.406091332 |
| peptidyl-serine modification (GO:0018209) | 28/169 | 0.02948162 | 0.406091332 |
| cellular iron ion homeostasis (GO:0006879) | 21520 | 0.030013856 | 0.412165966 |
| chemical homeostasis (GO:0048878) | 13/65 | 0.03170316 | 0.431986054 |
| positive regulation of protein localization to ( | 19299 | 0.031743995 | 0.431986054 |
| protein homotetramerization (GO:0051289) | 19299 | 0.031743995 | 0.431986054 |
| postreplication repair (GO:0006301) | 17076 | 0.033327814 | 0.452177369 |
| fatty-acyl-CoA metabolic process (GO:003533 | 44734 | 0.033621243 | 0.454792738 |
| fatty acid derivative biosynthetic process (GC | 14855 | 0.034621406 | 0.46415285 |
| glycolipid biosynthetic process (GO:0009247) | 14855 | 0.034621406 | 0.46415285 |
| sulfur compound catabolic process (GO:0044 | 14855 | 0.034621406 | 0.46415285 |
| positive regulation of fibroblast proliferation | 44770 | 0.035271981 | 0.464750579 |
| protein localization to lysosome (GO:006146: | 44770 | 0.035271981 | 0.464750579 |
| regulation of small molecule metabolic proce | 44770 | 0.035271981 | 0.464750579 |
| positive regulation of DNA binding (GO:0043: | 12632 | 0.035394744 | 0.464750579 |
| cardiolipin acyl-chain remodeling (GO:00359( | 44627 | 0.037086672 | 0.464750579 |
| ganglioside catabolic process (GO:0006689) | 44627 | 0.037086672 | 0.464750579 |
| heme transport (GO:0015886) | 44627 | 0.037086672 | 0.464750579 |
| hemostasis (GO:0007599) | 44627 | 0.037086672 | 0.464750579 |
| left/right pattern formation (GO:0060972) | 44627 | 0.037086672 | 0.464750579 |
| leucine metabolic process (GO:0006551) | 44627 | 0.037086672 | 0.464750579 |
| miRNA loading onto RISC involved in gene sil( | 44627 | 0.037086672 | 0.464750579 |
| negative regulation of centriole replication (( | 44627 | 0.037086672 | 0.464750579 |
| negative regulation of protein import (GO:19 | 44627 | 0.037086672 | 0.464750579 |
| negative regulation of protein import into nu | 44627 | 0.037086672 | 0.464750579 |
| negative regulation of ruffle assembly (GO:19 | 44627 | 0.037086672 | 0.464750579 |
| nucleobase biosynthetic process (GO:004611 | 44627 | 0.037086672 | 0.464750579 |
| positive regulation of integrin-mediated signa | 44627 | 0.037086672 | 0.464750579 |
| positive regulation of potassium ion import a | 44627 | 0.037086672 | 0.464750579 |
| protein localization to ciliary membrane (GO: | 44627 | 0.037086672 | 0.464750579 |
| regulation of defense response to bacterium | 44627 | 0.037086672 | 0.464750579 |
| regulation of Rab protein signal transduction | 44627 | 0.037086672 | 0.464750579 |
| regulation of vascular associated smooth mu | 44627 | 0.037086672 | 0.464750579 |
| very long-chain fatty acid catabolic process (( | 44627 | 0.037086672 | 0.464750579 |
| regulation of protein localization to plasma n | 15/80 | 0.037448347 | 0.464750579 |
| diol biosynthetic process (GO:0034312) | 44698 | 0.037751984 | 0.464750579 |
| DNA biosynthetic process (GO:0071897) | 44698 | 0.037751984 | 0.464750579 |
| histone H2A ubiquitination (GO:0033522) | 44698 | 0.037751984 | 0.464750579 |
| modified amino acid transport (GO:0072337) | 44698 | 0.037751984 | 0.464750579 |
| negative regulation of cell aging (GO:009034 | 44698 | 0.037751984 | 0.464750579 |
| positive regulation of muscle hypertrophy (G | 44698 | 0.037751984 | 0.464750579 |
| peptidyl-threonine phosphorylation (GO:001: | 22251 | 0.038079803 | 0.467512368 |
| keratan sulfate catabolic process (GO:004234 | 44663 | 0.0402849 | 0.481500472 |
| lysine catabolic process (GO:0006554) | 44663 | 0.0402849 | 0.481500472 |
| lysine metabolic process (GO:0006553) | 44663 | 0.0402849 | 0.481500472 |
| mitochondrial genome maintenance (GO:000 | 44663 | 0.0402849 | 0.481500472 |
| negative regulation of cilium assembly (GO:1 | 44663 | 0.0402849 | 0.481500472 |

| | | | |
|---|---|---|---|
| negative regulation of leukocyte chemotaxis | 44663 | 0.0402849 | 0.481500472 |
| negative regulation of microtubule polymeriz | 44663 | 0.0402849 | 0.481500472 |
| nonribosomal peptide biosynthetic process ( | 44663 | 0.0402849 | 0.481500472 |
| regulation of actin nucleation (GO:0051125) | 44663 | 0.0402849 | 0.481500472 |
| sphingoid metabolic process (GO:0046519) | 44663 | 0.0402849 | 0.481500472 |
| cellular response to amino acid starvation (G | 14/74 | 0.040591451 | 0.482354979 |
| glycosaminoglycan metabolic process (GO:00 | 20029 | 0.04074242 | 0.482354979 |
| cytoskeleton-dependent intracellular transpc | 44735 | 0.041205405 | 0.482354979 |
| hyaluronan metabolic process (GO:0030212) | 44735 | 0.041205405 | 0.482354979 |
| positive regulation of cell cycle G2/M phase t | 44735 | 0.041205405 | 0.482354979 |
| protein deacylation (GO:0035601) | 44735 | 0.041205405 | 0.482354979 |
| proteolysis (GO:0006508) | 43/287 | 0.041361752 | 0.482354979 |
| oxoacid metabolic process (GO:0043436) | 12997 | 0.041424022 | 0.482354979 |
| regulation of TORC1 signaling (GO:1903432) | 12997 | 0.041424022 | 0.482354979 |
| smoothened signaling pathway (GO:0007224 | 12997 | 0.041424022 | 0.482354979 |
| protein targeting to peroxisome (GO:000662 | 44771 | 0.04204067 | 0.488276984 |
| response to amino acid starvation (GO:19909 | 14/75 | 0.044835447 | 0.519401405 |
| cellular respiration (GO:0045333) | 15585 | 0.045911987 | 0.529159079 |
| regulation of double-strand break repair (GO | 15585 | 0.045911987 | 0.529159079 |
| amyloid precursor protein metabolic process | 44699 | 0.047478712 | 0.537189726 |
| dendritic cell migration (GO:0036336) | 44699 | 0.047478712 | 0.537189726 |
| hyaluronan catabolic process (GO:0030214) | 44699 | 0.047478712 | 0.537189726 |
| outer dynein arm assembly (GO:0036158) | 44699 | 0.047478712 | 0.537189726 |
| positive regulation of cardiac muscle hypertr | 44699 | 0.047478712 | 0.537189726 |
| prostanoid metabolic process (GO:0006692) | 44699 | 0.047478712 | 0.537189726 |
| regulation of telomere maintenance via telor | 44699 | 0.047478712 | 0.537189726 |
| glucose metabolic process (GO:0006006) | 22981 | 0.047559958 | 0.537189726 |
| regulation of sodium ion transport (GO:0002 | 13363 | 0.048096546 | 0.541895749 |
| secondary alcohol metabolic process (GO:19 | 18172 | 0.049125909 | 0.55211656 |
| alpha-amino acid catabolic process (GO:1901 | 44772 | 0.049596495 | 0.55264925 |
| microtubule polymerization (GO:0046785) | 44772 | 0.049596495 | 0.55264925 |
| regulation of cation transmembrane transpo | 44772 | 0.049596495 | 0.55264925 |
| regulation of cardiac muscle hypertrophy (GC | 44736 | 0.049784915 | 0.55264925 |
| regulation of ruffle assembly (GO:1900027) | 44736 | 0.049784915 | 0.55264925 |
| glycosaminoglycan catabolic process (GO:000 | 20760 | 0.051366231 | 0.554444792 |
| alpha-linolenic acid metabolic process (GO:0 | 44664 | 0.053042607 | 0.554444792 |
| DNA replication-independent nucleosome or | 44664 | 0.053042607 | 0.554444792 |
| myelination in peripheral nervous system (G | 44664 | 0.053042607 | 0.554444792 |
| negative regulation of cellular senescence (G | 44664 | 0.053042607 | 0.554444792 |
| negative regulation of DNA-dependent DNA r | 44664 | 0.053042607 | 0.554444792 |
| negative regulation of nucleocytoplasmic tra | 44664 | 0.053042607 | 0.554444792 |
| positive regulation of calcineurin-mediated si | 44664 | 0.053042607 | 0.554444792 |
| positive regulation of calcineurin-NFAT signal | 44664 | 0.053042607 | 0.554444792 |
| positive regulation of calcium ion import (GO | 44664 | 0.053042607 | 0.554444792 |
| regulation of cell aging (GO:0090342) | 44664 | 0.053042607 | 0.554444792 |
| spindle assembly (GO:0051225) | 15/84 | 0.054347524 | 0.554444792 |
| aromatic compound catabolic process (GO:0 | 44628 | 0.054364551 | 0.554444792 |

| | | | |
|---|---|---|---|
| cellular response to epinephrine stimulus (GO | 44628 | 0.054364551 | 0.554444792 |
| cellular response to hepatocyte growth facto | 44628 | 0.054364551 | 0.554444792 |
| DNA damage induced protein phosphorylatic | 44628 | 0.054364551 | 0.554444792 |
| elastic fiber assembly (GO:0048251) | 44628 | 0.054364551 | 0.554444792 |
| establishment of protein localization to pero: | 44628 | 0.054364551 | 0.554444792 |
| fructose metabolic process (GO:0006000) | 44628 | 0.054364551 | 0.554444792 |
| histone H2A-K119 monoubiquitination (GO:0 | 44628 | 0.054364551 | 0.554444792 |
| lipid import into cell (GO:0140354) | 44628 | 0.054364551 | 0.554444792 |
| methionine metabolic process (GO:0006555) | 44628 | 0.054364551 | 0.554444792 |
| mitochondrial DNA replication (GO:0006264) | 44628 | 0.054364551 | 0.554444792 |
| negative regulation of glial cell apoptotic pro | 44628 | 0.054364551 | 0.554444792 |
| peptidyl-lysine deacetylation (GO:0034983) | 44628 | 0.054364551 | 0.554444792 |
| platelet activating factor metabolic process ( | 44628 | 0.054364551 | 0.554444792 |
| positive regulation of chromosome segregati | 44628 | 0.054364551 | 0.554444792 |
| positive regulation of leukocyte differentiatic | 44628 | 0.054364551 | 0.554444792 |
| positive regulation of triglyceride catabolic pı | 44628 | 0.054364551 | 0.554444792 |
| pre-replicative complex assembly involved in | 44628 | 0.054364551 | 0.554444792 |
| regulation of mitochondrial gene expression | 44628 | 0.054364551 | 0.554444792 |
| regulation of potassium ion import (GO:1903 | 44628 | 0.054364551 | 0.554444792 |
| replication fork protection (GO:0048478) | 44628 | 0.054364551 | 0.554444792 |
| response to epinephrine (GO:0071871) | 44628 | 0.054364551 | 0.554444792 |
| response to hepatocyte growth factor (GO:0 | 44628 | 0.054364551 | 0.554444792 |
| somatic hypermutation of immunoglobulin g | 44628 | 0.054364551 | 0.554444792 |
| positive regulation of calcium ion transport ( | 13728 | 0.055429968 | 0.561508059 |
| regulation of actin cytoskeleton reorganizatic | 13728 | 0.055429968 | 0.561508059 |
| regulation of protein autophosphorylation (G | 13728 | 0.055429968 | 0.561508059 |
| sulfur compound biosynthetic process (GO:0 | 19/113 | 0.056649554 | 0.572578718 |
| prostaglandin metabolic process (GO:000669 | 44773 | 0.057958227 | 0.576051414 |
| vitamin transport (GO:0051180) | 44773 | 0.057958227 | 0.576051414 |
| negative regulation of cell cycle process (GO: | 23712 | 0.058542524 | 0.576051414 |
| aspartate family amino acid catabolic proces: | 44700 | 0.058550055 | 0.576051414 |
| aspartate family amino acid metabolic proce: | 44700 | 0.058550055 | 0.576051414 |
| diol metabolic process (GO:0034311) | 44700 | 0.058550055 | 0.576051414 |
| DNA double-strand break processing (GO:00 | 44700 | 0.058550055 | 0.576051414 |
| folic acid metabolic process (GO:0046655) | 44700 | 0.058550055 | 0.576051414 |
| heparan sulfate proteoglycan biosynthetic pr | 44700 | 0.058550055 | 0.576051414 |
| positive regulation of actin cytoskeleton reor | 44700 | 0.058550055 | 0.576051414 |
| prostaglandin biosynthetic process (GO:0001 | 44700 | 0.058550055 | 0.576051414 |
| regulated exocytosis (GO:0045055) | 28/180 | 0.058880491 | 0.576051414 |
| protein tetramerization (GO:0051262) | 13/71 | 0.059195565 | 0.576051414 |
| retrograde vesicle-mediated transport, Golgi | 15/85 | 0.059280714 | 0.576051414 |
| regulation of calcium ion import (GO:009027 | 44737 | 0.059379293 | 0.576051414 |
| cation transmembrane transport (GO:009865 | 16316 | 0.059415662 | 0.576051414 |
| hexose biosynthetic process (GO:0019319) | 16316 | 0.059415662 | 0.576051414 |
| mitotic spindle assembly (GO:0090307) | 16316 | 0.059415662 | 0.576051414 |
| positive regulation of receptor-mediated end | 16316 | 0.059415662 | 0.576051414 |
| protein-DNA complex assembly (GO:006500 | 23/143 | 0.059755921 | 0.578109744 |

| | | | |
|---|---|---|---|
| regulation of protein modification process (G | 18902 | 0.062057019 | 0.599088912 |
| negative regulation of TOR signaling (GO:003 | 14093 | 0.063437456 | 0.611109649 |
| epithelium development (GO:0060429) | 20/122 | 0.064124123 | 0.616410189 |
| regulation of cellular catabolic process (GO:0 | 24077 | 0.064619364 | 0.619851992 |
| axonemal dynein complex assembly (GO:007 | 11871 | 0.067137737 | 0.629590959 |
| positive regulation of telomere maintenance | 11871 | 0.067137737 | 0.629590959 |
| regulation of centrosome duplication (GO:00 | 11871 | 0.067137737 | 0.629590959 |
| regulation of intracellular protein transport ( | 11871 | 0.067137737 | 0.629590959 |
| regulation of transcription involved in G1/S t | 11871 | 0.067137737 | 0.629590959 |
| calcium ion import across plasma membrane | 44665 | 0.067724924 | 0.629590959 |
| dicarboxylic acid catabolic process (GO:0043 | 44665 | 0.067724924 | 0.629590959 |
| negative regulation of cell activation (GO:005 | 44665 | 0.067724924 | 0.629590959 |
| negative regulation of TORC1 signaling (GO:1 | 44665 | 0.067724924 | 0.629590959 |
| nuclear pore organization (GO:0006999) | 44665 | 0.067724924 | 0.629590959 |
| positive regulation of sodium ion transmemb | 44665 | 0.067724924 | 0.629590959 |
| protein maturation by iron-sulfur cluster tran | 44665 | 0.067724924 | 0.629590959 |
| regulation of cAMP-dependent protein kinase | 44665 | 0.067724924 | 0.629590959 |
| regulation of gastrulation (GO:0010470) | 44665 | 0.067724924 | 0.629590959 |
| response to X-ray (GO:0010165) | 44665 | 0.067724924 | 0.629590959 |
| mitochondrion organization (GO:0007005) | 27/175 | 0.067963679 | 0.630513143 |
| post-translational protein modification (GO:0 | 49/345 | 0.068356195 | 0.632855101 |
| cilium movement (GO:0003341) | 19268 | 0.06927441 | 0.640044546 |
| mononuclear cell differentiation (GO:190313 | 44738 | 0.069996755 | 0.641138017 |
| negative regulation of telomere maintenance | 44738 | 0.069996755 | 0.641138017 |
| calcium ion transmembrane transport (GO:00 | 15/87 | 0.070040139 | 0.641138017 |
| lipid biosynthetic process (GO:0008610) | 14/80 | 0.070727646 | 0.641138017 |
| ganglioside metabolic process (GO:0001573) | 44701 | 0.070965117 | 0.641138017 |
| negative regulation of lipid storage (GO:0010 | 44701 | 0.070965117 | 0.641138017 |
| positive regulation of G2/M transition of mit | 44701 | 0.070965117 | 0.641138017 |
| production of small RNA involved in gene sile | 44701 | 0.070965117 | 0.641138017 |
| purine ribonucleoside monophosphate meta | 44701 | 0.070965117 | 0.641138017 |
| regulation of nuclear division (GO:0051783) | 44701 | 0.070965117 | 0.641138017 |
| regulation of protein localization to cell perip | 44701 | 0.070965117 | 0.641138017 |
| positive regulation of cell cycle (GO:0045787 | 24442 | 0.07109565 | 0.641138017 |
| neutrophil degranulation (GO:0043312) | 66/481 | 0.071362521 | 0.642262686 |
| positive regulation of intracellular transport ( | 14458 | 0.072127551 | 0.6433487 |
| positive regulation of protein kinase activity ( | 24/154 | 0.074398979 | 0.6433487 |
| amyloid-beta formation (GO:0034205) | 44629 | 0.074758315 | 0.6433487 |
| axon regeneration (GO:0031103) | 44629 | 0.074758315 | 0.6433487 |
| estrogen biosynthetic process (GO:0006703) | 44629 | 0.074758315 | 0.6433487 |
| fructose 1,6-bisphosphate metabolic process | 44629 | 0.074758315 | 0.6433487 |
| hepoxilin biosynthetic process (GO:0051122) | 44629 | 0.074758315 | 0.6433487 |
| hepoxilin metabolic process (GO:0051121) | 44629 | 0.074758315 | 0.6433487 |
| negative regulation of bone resorption (GO:0 | 44629 | 0.074758315 | 0.6433487 |
| negative regulation of platelet aggregation (G | 44629 | 0.074758315 | 0.6433487 |
| negative regulation of smooth muscle cell dif | 44629 | 0.074758315 | 0.6433487 |
| peroxisomal transport (GO:0043574) | 44629 | 0.074758315 | 0.6433487 |

| | | | |
|---|---|---|---|
| positive regulation of chromatin binding (GO: | 44629 | 0.074758315 | 0.6433487 |
| positive regulation of extracellular matrix ass | 44629 | 0.074758315 | 0.6433487 |
| positive regulation of mitochondrial calcium i | 44629 | 0.074758315 | 0.6433487 |
| protein K6-linked ubiquitination (GO:008502( | 44629 | 0.074758315 | 0.6433487 |
| purine nucleobase metabolic process (GO:00 | 44629 | 0.074758315 | 0.6433487 |
| regulation of ER to Golgi vesicle-mediated tra | 44629 | 0.074758315 | 0.6433487 |
| regulation of fibroblast apoptotic process (G( | 44629 | 0.074758315 | 0.6433487 |
| regulation of mammary gland epithelial cell p | 44629 | 0.074758315 | 0.6433487 |
| regulation of skeletal muscle cell differentiati | 44629 | 0.074758315 | 0.6433487 |
| response to misfolded protein (GO:0051788) | 44629 | 0.074758315 | 0.6433487 |
| small RNA loading onto RISC (GO:0070922) | 44629 | 0.074758315 | 0.6433487 |
| alpha-amino acid metabolic process (GO:190 | 17046 | 0.075226284 | 0.644919072 |
| regulation of fibroblast proliferation (GO:004 | 17046 | 0.075226284 | 0.644919072 |
| regulation of DNA replication (GO:0006275) | 19633 | 0.07700177 | 0.657581423 |
| keratan sulfate metabolic process (GO:00423 | 12236 | 0.077139919 | 0.657581423 |
| positive regulation of organelle organization | 12236 | 0.077139919 | 0.657581423 |
| positive regulation of protein-containing com | 19/118 | 0.080489786 | 0.684845295 |
| neutrophil activation involved in immune res | 66/485 | 0.081220796 | 0.688105037 |
| nucleocytoplasmic transport (GO:0006913) | 14824 | 0.081504093 | 0.688105037 |
| positive regulation of catalytic activity (GO:0( | 14824 | 0.081504093 | 0.688105037 |
| regulation of microtubule polymerization (G( | 14824 | 0.081504093 | 0.688105037 |
| cellular lipid catabolic process (GO:0044242) | 44739 | 0.081634418 | 0.688105037 |
| phosphatidylinositol biosynthetic process (G( | 20/126 | 0.08358516 | 0.692027786 |
| regulation of DNA binding (GO:0051101) | 17411 | 0.084011767 | 0.692027786 |
| acetyl-CoA metabolic process (GO:0006084) | 44666 | 0.084267598 | 0.692027786 |
| C21-steroid hormone biosynthetic process (G | 44666 | 0.084267598 | 0.692027786 |
| calcium ion import into cytosol (GO:1902656 | 44666 | 0.084267598 | 0.692027786 |
| cellular response to ATP (GO:0071318) | 44666 | 0.084267598 | 0.692027786 |
| electron transport chain (GO:0022900) | 44666 | 0.084267598 | 0.692027786 |
| icosanoid biosynthetic process (GO:0046456) | 44666 | 0.084267598 | 0.692027786 |
| nitric oxide biosynthetic process (GO:000680 | 44666 | 0.084267598 | 0.692027786 |
| protein localization to chromosome, telomer | 44666 | 0.084267598 | 0.692027786 |
| regulation of clathrin-dependent endocytosis | 44666 | 0.084267598 | 0.692027786 |
| T cell proliferation (GO:0042098) | 44666 | 0.084267598 | 0.692027786 |
| androgen metabolic process (GO:0008209) | 44702 | 0.084703711 | 0.692027786 |
| heparan sulfate proteoglycan metabolic proc | 44702 | 0.084703711 | 0.692027786 |
| positive regulation of lipid storage (GO:0010 | 44702 | 0.084703711 | 0.692027786 |
| positive regulation of vascular associated sm | 44702 | 0.084703711 | 0.692027786 |
| regulation of cholesterol metabolic process ( | 44702 | 0.084703711 | 0.692027786 |
| protein maturation (GO:0051604) | 16/97 | 0.086594321 | 0.699984745 |
| axoneme assembly (GO:0035082) | 12601 | 0.087962748 | 0.699984745 |
| negative regulation of mitochondrion organiz | 12601 | 0.087962748 | 0.699984745 |
| neural tube closure (GO:0001843) | 12601 | 0.087962748 | 0.699984745 |
| protein localization to chromosome (GO:003 | 12601 | 0.087962748 | 0.699984745 |
| positive regulation of cellular biosynthetic pr | 27/180 | 0.089001589 | 0.699984745 |
| neutrophil mediated immunity (GO:0002446 | 66/488 | 0.089221768 | 0.699984745 |
| mitochondrial translation (GO:0032543) | 17/105 | 0.090674654 | 0.699984745 |

| | | | |
|---|---|---|---|
| cellular response to organic cyclic compound | 23/150 | 0.09124038 | 0.699984745 |
| carbohydrate catabolic process (GO:0016052 | 15189 | 0.091566212 | 0.699984745 |
| carboxylic acid transmembrane transport (GO | 15189 | 0.091566212 | 0.699984745 |
| import into cell (GO:0098657) | 15189 | 0.091566212 | 0.699984745 |
| positive regulation of DNA repair (GO:00457 | 17777 | 0.093385324 | 0.699984745 |
| positive regulation of endocytosis (GO:00458 | 17777 | 0.093385324 | 0.699984745 |
| double-strand break repair via nonhomologo | 22951 | 0.093774297 | 0.699984745 |
| negative regulation of kinase activity (GO:00 | 22951 | 0.093774297 | 0.699984745 |
| ceramide metabolic process (GO:0006672) | 20363 | 0.093997595 | 0.699984745 |
| glycogen metabolic process (GO:0005977) | 44740 | 0.094278726 | 0.699984745 |
| GPI anchor metabolic process (GO:0006505) | 44740 | 0.094278726 | 0.699984745 |
| macromolecule catabolic process (GO:00090 | 44740 | 0.094278726 | 0.699984745 |
| regulation of calcineurin-NFAT signaling casc | 44740 | 0.094278726 | 0.699984745 |
| regulation of neuron migration (GO:2001222 | 44740 | 0.094278726 | 0.699984745 |
| regulation of plasma membrane bounded cel | 44740 | 0.094278726 | 0.699984745 |
| transcription by RNA polymerase III (GO:000 | 44740 | 0.094278726 | 0.699984745 |
| cellular macromolecule biosynthetic process | 44/314 | 0.094724208 | 0.699984745 |
| peptidyl-lysine modification (GO:0018205) | 17/106 | 0.097005332 | 0.699984745 |
| aromatic amino acid family metabolic proces | 44630 | 0.097970027 | 0.699984745 |
| calcium import into the mitochondrion (GO:0 | 44630 | 0.097970027 | 0.699984745 |
| cellular response to leucine (GO:0071233) | 44630 | 0.097970027 | 0.699984745 |
| DNA replication-dependent nucleosome asse | 44630 | 0.097970027 | 0.699984745 |
| DNA replication-dependent nucleosome orga | 44630 | 0.097970027 | 0.699984745 |
| ether lipid biosynthetic process (GO:0008611 | 44630 | 0.097970027 | 0.699984745 |
| L-glutamate transmembrane transport (GO:0 | 44630 | 0.097970027 | 0.699984745 |
| lipoprotein transport (GO:0042953) | 44630 | 0.097970027 | 0.699984745 |
| negative regulation of transcription from RN | 44630 | 0.097970027 | 0.699984745 |
| neuron projection regeneration (GO:003110 | 44630 | 0.097970027 | 0.699984745 |
| nuclear pore complex assembly (GO:005129 | 44630 | 0.097970027 | 0.699984745 |
| nucleic acid transport (GO:0050657) | 44630 | 0.097970027 | 0.699984745 |
| phosphatidylcholine catabolic process (GO:0 | 44630 | 0.097970027 | 0.699984745 |
| pinocytosis (GO:0006907) | 44630 | 0.097970027 | 0.699984745 |
| production of siRNA involved in RNA interfer | 44630 | 0.097970027 | 0.699984745 |
| regulation of attachment of spindle microtub | 44630 | 0.097970027 | 0.699984745 |
| regulation of DNA damage checkpoint (GO:2 | 44630 | 0.097970027 | 0.699984745 |
| regulation of hair cycle (GO:0042634) | 44630 | 0.097970027 | 0.699984745 |
| regulation of low-density lipoprotein particle | 44630 | 0.097970027 | 0.699984745 |
| regulation of macrophage migration (GO:190 | 44630 | 0.097970027 | 0.699984745 |
| regulation of microtubule nucleation (GO:00 | 44630 | 0.097970027 | 0.699984745 |
| regulation of RNA polymerase II transcription | 44630 | 0.097970027 | 0.699984745 |
| response to leucine (GO:0043201) | 44630 | 0.097970027 | 0.699984745 |
| telomere maintenance in response to DNA d | 44630 | 0.097970027 | 0.699984745 |
| urea cycle (GO:0000050) | 44630 | 0.097970027 | 0.699984745 |
| phosphatidylcholine metabolic process (GO:0 | 13/77 | 0.099352045 | 0.699984745 |
| response to UV (GO:0009411) | 16/99 | 0.099683529 | 0.699984745 |
| heme biosynthetic process (GO:0006783) | 44703 | 0.099727975 | 0.699984745 |
| positive regulation of axon extension (GO:00 | 44703 | 0.099727975 | 0.699984745 |

| | | | |
|---|---|---|---|
| regulation of macrophage chemotaxis (GO:0( | 44703 | 0.099727975 | 0.699984745 |
| regulation of vacuole organization (GO:0044( | 44703 | 0.099727975 | 0.699984745 |
| sterol metabolic process (GO:0016125) | 25903 | 0.101071666 | 0.699984745 |
| calcium ion transmembrane import into cyto | 15554 | 0.102308387 | 0.699984745 |
| determination of left/right symmetry (GO:00 | 15554 | 0.102308387 | 0.699984745 |
| positive regulation of ATPase activity (GO:00: | 15554 | 0.102308387 | 0.699984745 |
| apoptotic DNA fragmentation (GO:0006309) | 44667 | 0.102573395 | 0.699984745 |
| aromatic amino acid family catabolic process | 44667 | 0.102573395 | 0.699984745 |
| N-acetylglucosamine metabolic process (GO: | 44667 | 0.102573395 | 0.699984745 |
| negative regulation of platelet activation (GC | 44667 | 0.102573395 | 0.699984745 |
| negative regulation of release of cytochrome | 44667 | 0.102573395 | 0.699984745 |
| neural tube development (GO:0021915) | 44667 | 0.102573395 | 0.699984745 |
| nitric oxide metabolic process (GO:0046209) | 44667 | 0.102573395 | 0.699984745 |
| positive regulation of TORC1 signaling (GO:19 | 44667 | 0.102573395 | 0.699984745 |
| sphingolipid catabolic process (GO:0030149) | 44667 | 0.102573395 | 0.699984745 |
| aggresome assembly (GO:0070842) | 44597 | 0.104114597 | 0.699984745 |
| alditol phosphate metabolic process (GO:005 | 44597 | 0.104114597 | 0.699984745 |
| CD4-positive, alpha-beta T cell activation (GC | 44597 | 0.104114597 | 0.699984745 |
| cellular response to salt stress (GO:0071472) | 44597 | 0.104114597 | 0.699984745 |
| cellular response to vitamin (GO:0071295) | 44597 | 0.104114597 | 0.699984745 |
| de novo centriole assembly (GO:0097742) | 44597 | 0.104114597 | 0.699984745 |
| de novo centriole assembly involved in multi- | 44597 | 0.104114597 | 0.699984745 |
| deoxyribonucleotide metabolic process (GO:( | 44597 | 0.104114597 | 0.699984745 |
| DNA double-strand break processing involved | 44597 | 0.104114597 | 0.699984745 |
| DNA protection (GO:0042262) | 44597 | 0.104114597 | 0.699984745 |
| double-strand break repair via single-strand a | 44597 | 0.104114597 | 0.699984745 |
| drug transport (GO:0015893) | 44597 | 0.104114597 | 0.699984745 |
| establishment of protein localization to telon | 44597 | 0.104114597 | 0.699984745 |
| establishment of T cell polarity (GO:0001768 | 44597 | 0.104114597 | 0.699984745 |
| glucose import in response to insulin stimulu | 44597 | 0.104114597 | 0.699984745 |
| histone-threonine phosphorylation (GO:0035 | 44597 | 0.104114597 | 0.699984745 |
| L-methionine salvage from methylthioadenos | 44597 | 0.104114597 | 0.699984745 |
| left/right axis specification (GO:0070986) | 44597 | 0.104114597 | 0.699984745 |
| meiotic mismatch repair (GO:0000710) | 44597 | 0.104114597 | 0.699984745 |
| mitochondrial DNA repair (GO:0043504) | 44597 | 0.104114597 | 0.699984745 |
| negative regulation of cardiac muscle cell prc | 44597 | 0.104114597 | 0.699984745 |
| negative regulation of cholesterol biosynthet | 44597 | 0.104114597 | 0.699984745 |
| negative regulation of cholesterol metabolic | 44597 | 0.104114597 | 0.699984745 |
| negative regulation of cysteine-type endopep | 44597 | 0.104114597 | 0.699984745 |
| negative regulation of hydrogen peroxide me | 44597 | 0.104114597 | 0.699984745 |
| negative regulation of regulatory T cell differ | 44597 | 0.104114597 | 0.699984745 |
| negative regulation of smooth muscle cell ap | 44597 | 0.104114597 | 0.699984745 |
| negative regulation of sphingolipid biosynthe | 44597 | 0.104114597 | 0.699984745 |
| negative regulation of sterol biosynthetic prc | 44597 | 0.104114597 | 0.699984745 |
| plus-end-directed vesicle transport along mic | 44597 | 0.104114597 | 0.699984745 |
| positive regulation of basement membrane a | 44597 | 0.104114597 | 0.699984745 |
| positive regulation of glycoprotein metabolic | 44597 | 0.104114597 | 0.699984745 |

| | | | |
|---|---|---|---|
| positive regulation of low-density lipoprotein | 44597 | 0.104114597 | 0.699984745 |
| positive regulation of toll-like receptor 9 sign | 44597 | 0.104114597 | 0.699984745 |
| positive regulation of translation in response | 44597 | 0.104114597 | 0.699984745 |
| positive regulation of vascular associated sm | 44597 | 0.104114597 | 0.699984745 |
| protein lipoylation (GO:0009249) | 44597 | 0.104114597 | 0.699984745 |
| protein processing involved in protein targeti | 44597 | 0.104114597 | 0.699984745 |
| pyrimidine ribonucleotide metabolic process | 44597 | 0.104114597 | 0.699984745 |
| regulation of adenylate cyclase-activating G p | 44597 | 0.104114597 | 0.699984745 |
| regulation of barbed-end actin filament capp | 44597 | 0.104114597 | 0.699984745 |
| regulation of basement membrane assembly | 44597 | 0.104114597 | 0.699984745 |
| regulation of cellular response to vascular en | 44597 | 0.104114597 | 0.699984745 |
| regulation of intestinal lipid absorption (GO:1 | 44597 | 0.104114597 | 0.699984745 |
| regulation of intracellular cholesterol transpo | 44597 | 0.104114597 | 0.699984745 |
| RNA import into mitochondrion (GO:003592 | 44597 | 0.104114597 | 0.699984745 |
| rRNA transport (GO:0051029) | 44597 | 0.104114597 | 0.699984745 |
| succinyl-CoA metabolic process (GO:000610 | 44597 | 0.104114597 | 0.699984745 |
| transcription initiation from RNA polymerase | 44597 | 0.104114597 | 0.699984745 |
| G1/S transition of mitotic cell cycle (GO:0000 | 14/85 | 0.104895879 | 0.704189569 |
| Golgi organization (GO:0007030) | 20/130 | 0.106581096 | 0.714441232 |
| calcium ion transport (GO:0006816) | 18/115 | 0.107080165 | 0.716723239 |
| determination of bilateral symmetry (GO:000 | 44741 | 0.107906051 | 0.717996378 |
| production of miRNAs involved in gene silenc | 44741 | 0.107906051 | 0.717996378 |
| protein ADP-ribosylation (GO:0006471) | 44741 | 0.107906051 | 0.717996378 |
| regulation of neuronal synaptic plasticity (GC | 44741 | 0.107906051 | 0.717996378 |
| regulation of TOR signaling (GO:0032006) | 26268 | 0.109588842 | 0.728121161 |
| negative regulation of DNA metabolic proces | 13332 | 0.11202869 | 0.738898716 |
| negative regulation of fat cell differentiation | 13332 | 0.11202869 | 0.738898716 |
| regulation of receptor internalization (GO:00 | 13332 | 0.11202869 | 0.738898716 |
| transferrin transport (GO:0033572) | 13332 | 0.11202869 | 0.738898716 |
| transition metal ion transport (GO:0000041) | 13332 | 0.11202869 | 0.738898716 |
| transport across blood-brain barrier (GO:015 | 14/86 | 0.112748709 | 0.742563657 |
| regulation of mitotic nuclear division (GO:00 | 21094 | 0.113041405 | 0.743407671 |
| organic hydroxy compound biosynthetic proc | 18507 | 0.113880295 | 0.747836008 |
| acylglycerol biosynthetic process (GO:00464 | 44704 | 0.115984253 | 0.75181758 |
| diacylglycerol metabolic process (GO:004633 | 44704 | 0.115984253 | 0.75181758 |
| hexose transmembrane transport (GO:00086 | 44704 | 0.115984253 | 0.75181758 |
| mitotic cell cycle checkpoint signaling (GO:00 | 44704 | 0.115984253 | 0.75181758 |
| negative regulation of reactive oxygen specie | 44704 | 0.115984253 | 0.75181758 |
| positive regulation of protein autophosphory | 44704 | 0.115984253 | 0.75181758 |
| positive regulation of receptor internalizatior | 44704 | 0.115984253 | 0.75181758 |
| regulation of steroid metabolic process (GO:( | 44704 | 0.115984253 | 0.75181758 |
| regulation of telomere capping (GO:1904353 | 44704 | 0.115984253 | 0.75181758 |
| regulation of programmed cell death (GO:004 | 28/194 | 0.120576483 | 0.7559857 |
| protein autoubiquitination (GO:0051865) | 24047 | 0.121095688 | 0.7559857 |
| intrinsic apoptotic signaling pathway (GO:009 | 16/102 | 0.121496337 | 0.7559857 |
| cellular protein localization (GO:0034613) | 45/329 | 0.122208935 | 0.7559857 |
| protein N-linked glycosylation via asparagine | 44742 | 0.122483446 | 0.7559857 |

| | | | |
|---|---|---|---|
| amyloid-beta metabolic process (GO:005043 | 44668 | 0.122518455 | 0.7559857 |
| monosaccharide transmembrane transport ( | 44668 | 0.122518455 | 0.7559857 |
| negative regulation of vascular associated sm | 44668 | 0.122518455 | 0.7559857 |
| positive regulation of cyclin-dependent prote | 44668 | 0.122518455 | 0.7559857 |
| positive regulation of telomere capping (GO: | 44668 | 0.122518455 | 0.7559857 |
| protein heterooligomerization (GO:0051291) | 44668 | 0.122518455 | 0.7559857 |
| regulation of cellular response to growth fact | 44668 | 0.122518455 | 0.7559857 |
| RNA interference (GO:0016246) | 44668 | 0.122518455 | 0.7559857 |
| mitochondrial respiratory chain complex I as | 21459 | 0.123319248 | 0.7559857 |
| NADH dehydrogenase complex assembly (GC | 21459 | 0.123319248 | 0.7559857 |
| positive regulation of mitochondrion organiz | 21459 | 0.123319248 | 0.7559857 |
| calcineurin-NFAT signaling cascade (GO:0033 | 44631 | 0.123655031 | 0.7559857 |
| cellular response to UV-A (GO:0071492) | 44631 | 0.123655031 | 0.7559857 |
| cyclooxygenase pathway (GO:0019371) | 44631 | 0.123655031 | 0.7559857 |
| glutathione biosynthetic process (GO:00067 | 44631 | 0.123655031 | 0.7559857 |
| glycerolipid metabolic process (GO:0046486) | 44631 | 0.123655031 | 0.7559857 |
| hepatocyte growth factor receptor signaling | 44631 | 0.123655031 | 0.7559857 |
| lipoprotein catabolic process (GO:0042159) | 44631 | 0.123655031 | 0.7559857 |
| lipoprotein localization (GO:0044872) | 44631 | 0.123655031 | 0.7559857 |
| mitochondrial protein processing (GO:00349 | 44631 | 0.123655031 | 0.7559857 |
| mitochondrion morphogenesis (GO:0070584 | 44631 | 0.123655031 | 0.7559857 |
| monocyte differentiation (GO:0030224) | 44631 | 0.123655031 | 0.7559857 |
| negative regulation of bone remodeling (GO: | 44631 | 0.123655031 | 0.7559857 |
| negative regulation of homotypic cell-cell adl | 44631 | 0.123655031 | 0.7559857 |
| peptidyl-proline hydroxylation (GO:0019511) | 44631 | 0.123655031 | 0.7559857 |
| positive regulation of bone resorption (GO:0 | 44631 | 0.123655031 | 0.7559857 |
| protein kinase C-activating G protein-couplec | 44631 | 0.123655031 | 0.7559857 |
| protein polyglutamylation (GO:0018095) | 44631 | 0.123655031 | 0.7559857 |
| regulation of biosynthetic process (GO:0009 | 44631 | 0.123655031 | 0.7559857 |
| regulation of histone H3-K9 acetylation (GO: | 44631 | 0.123655031 | 0.7559857 |
| regulation of lamellipodium morphogenesis ( | 44631 | 0.123655031 | 0.7559857 |
| regulation of lipid kinase activity (GO:004355 | 44631 | 0.123655031 | 0.7559857 |
| regulation of long-term neuronal synaptic pl | 44631 | 0.123655031 | 0.7559857 |
| regulation of melanin biosynthetic process (G | 44631 | 0.123655031 | 0.7559857 |
| regulation of mitochondrial fusion (GO:0010 | 44631 | 0.123655031 | 0.7559857 |
| regulation of pH (GO:0006885) | 44631 | 0.123655031 | 0.7559857 |
| response to prostaglandin E (GO:0034695) | 44631 | 0.123655031 | 0.7559857 |
| negative regulation of cellular component or | 13/80 | 0.124425464 | 0.759667898 |
| regulation of smoothened signaling pathway | 18872 | 0.124985286 | 0.76050002 |
| phospholipid biosynthetic process (GO:0008 | 13697 | 0.125235063 | 0.76050002 |
| regulation of intracellular steroid hormone re | 13697 | 0.125235063 | 0.76050002 |
| tRNA transport (GO:0051031) | 13697 | 0.125235063 | 0.76050002 |
| peptidyl-serine phosphorylation (GO:001810 | 23/156 | 0.125589354 | 0.761627787 |
| apoptotic nuclear changes (GO:0030262) | 44705 | 0.133405138 | 0.79670546 |
| C21-steroid hormone metabolic process (GO | 44705 | 0.133405138 | 0.79670546 |
| cellular response to catecholamine stimulus ( | 44705 | 0.133405138 | 0.79670546 |
| extracellular matrix assembly (GO:0085029) | 44705 | 0.133405138 | 0.79670546 |

| | | | |
|---|---|---|---|
| histone monoubiquitination (GO:0010390) | 44705 | 0.133405138 | 0.79670546 |
| porphyrin-containing compound biosynthetic | 44705 | 0.133405138 | 0.79670546 |
| positive regulation of carbohydrate metaboli | 44705 | 0.133405138 | 0.79670546 |
| sulfur amino acid metabolic process (GO:000 | 44705 | 0.133405138 | 0.79670546 |
| positive regulation of cellular amide metabol | 13/81 | 0.133514168 | 0.79670546 |
| cellular transition metal ion homeostasis (GO | 15/96 | 0.133776799 | 0.79670546 |
| mitotic DNA damage checkpoint signaling (G | 21824 | 0.134089676 | 0.79670546 |
| positive regulation of protein localization to | 19238 | 0.136645706 | 0.79670546 |
| activation of protein kinase B activity (GO:00 | 11475 | 0.137969504 | 0.79670546 |
| aminoglycan catabolic process (GO:0006026) | 11475 | 0.137969504 | 0.79670546 |
| negative regulation of response to DNA dam | 11475 | 0.137969504 | 0.79670546 |
| peptidyl-asparagine modification (GO:00181 | 11475 | 0.137969504 | 0.79670546 |
| proteoglycan biosynthetic process (GO:0030 | 11475 | 0.137969504 | 0.79670546 |
| regulation of transmembrane receptor prote | 11475 | 0.137969504 | 0.79670546 |
| tube closure (GO:0060606) | 11475 | 0.137969504 | 0.79670546 |
| SCF-dependent proteasomal ubiquitin-depen | 14/89 | 0.138321379 | 0.79670546 |
| regulation of axon extension (GO:0030516) | 14062 | 0.139189366 | 0.79670546 |
| regulation of macroautophagy (GO:0016241) | 17/112 | 0.140659677 | 0.79670546 |
| peptidyl-threonine modification (GO:001821 | 24777 | 0.141529857 | 0.79670546 |
| regulation of cyclin-dependent protein serine | 13/82 | 0.142960123 | 0.79670546 |
| acidic amino acid transport (GO:0015800) | 44669 | 0.14395839 | 0.79670546 |
| cellular response to alkaloid (GO:0071312) | 44669 | 0.14395839 | 0.79670546 |
| melanosome transport (GO:0032402) | 44669 | 0.14395839 | 0.79670546 |
| microtubule depolymerization (GO:0007019) | 44669 | 0.14395839 | 0.79670546 |
| negative regulation of tumor necrosis factor- | 44669 | 0.14395839 | 0.79670546 |
| nucleotide biosynthetic process (GO:000916 | 44669 | 0.14395839 | 0.79670546 |
| pigment granule transport (GO:0051904) | 44669 | 0.14395839 | 0.79670546 |
| positive regulation of fatty acid metabolic pr | 44669 | 0.14395839 | 0.79670546 |
| protein localization to centrosome (GO:0071 | 44669 | 0.14395839 | 0.79670546 |
| regulation of platelet aggregation (GO:00903 | 44669 | 0.14395839 | 0.79670546 |
| regulation of vascular endothelial cell prolife | 44669 | 0.14395839 | 0.79670546 |
| sodium ion import across plasma membrane | 44669 | 0.14395839 | 0.79670546 |
| 3'-phosphoadenosine 5'-phosphosulfate bios | 44598 | 0.144599043 | 0.79670546 |
| activation of cysteine-type endopeptidase ac | 44598 | 0.144599043 | 0.79670546 |
| carnitine transport (GO:0015879) | 44598 | 0.144599043 | 0.79670546 |
| cellular hyperosmotic response (GO:0071474 | 44598 | 0.144599043 | 0.79670546 |
| cellular lipid biosynthetic process (GO:00973 | 44598 | 0.144599043 | 0.79670546 |
| cholesterol import (GO:0070508) | 44598 | 0.144599043 | 0.79670546 |
| establishment of lymphocyte polarity (GO:00 | 44598 | 0.144599043 | 0.79670546 |
| gastrulation with mouth forming second (GO | 44598 | 0.144599043 | 0.79670546 |
| glycerol ether biosynthetic process (GO:0046 | 44598 | 0.144599043 | 0.79670546 |
| hydrogen peroxide biosynthetic process (GO: | 44598 | 0.144599043 | 0.79670546 |
| inclusion body assembly (GO:0070841) | 44598 | 0.144599043 | 0.79670546 |
| melanosome assembly (GO:1903232) | 44598 | 0.144599043 | 0.79670546 |
| mitochondrial proton-transporting ATP synth | 44598 | 0.144599043 | 0.79670546 |
| mitotic DNA replication initiation (GO:19029 | 44598 | 0.144599043 | 0.79670546 |
| negative regulation of cardiac muscle tissue | 44598 | 0.144599043 | 0.79670546 |

| | | | |
|---|---|---|---|
| negative regulation of fat cell proliferation (G | 44598 | 0.144599043 | 0.79670546 |
| negative regulation of glucocorticoid recepto | 44598 | 0.144599043 | 0.79670546 |
| negative regulation of histone H3-K9 methyla | 44598 | 0.144599043 | 0.79670546 |
| neuroblast proliferation (GO:0007405) | 44598 | 0.144599043 | 0.79670546 |
| nuclear cell cycle DNA replication initiation (C | 44598 | 0.144599043 | 0.79670546 |
| plus-end-directed organelle transport along r | 44598 | 0.144599043 | 0.79670546 |
| positive regulation of cholesterol metabolic p | 44598 | 0.144599043 | 0.79670546 |
| positive regulation of ion transmembrane tra | 44598 | 0.144599043 | 0.79670546 |
| positive regulation of lipoprotein particle clea | 44598 | 0.144599043 | 0.79670546 |
| positive regulation of microglial cell activatio | 44598 | 0.144599043 | 0.79670546 |
| positive regulation of nuclear cell cycle DNA r | 44598 | 0.144599043 | 0.79670546 |
| positive regulation of T-helper 2 cell different | 44598 | 0.144599043 | 0.79670546 |
| protein K27-linked ubiquitination (GO:00443 | 44598 | 0.144599043 | 0.79670546 |
| protein K29-linked ubiquitination (GO:00355 | 44598 | 0.144599043 | 0.79670546 |
| protein localization to non-motile cilium (GO | 44598 | 0.144599043 | 0.79670546 |
| protein repair (GO:0030091) | 44598 | 0.144599043 | 0.79670546 |
| pteridine-containing compound biosynthetic | 44598 | 0.144599043 | 0.79670546 |
| purine ribonucleoside bisphosphate biosynth | 44598 | 0.144599043 | 0.79670546 |
| regulation of endoplasmic reticulum tubular | 44598 | 0.144599043 | 0.79670546 |
| regulation of glomerular filtration (GO:00030 | 44598 | 0.144599043 | 0.79670546 |
| regulation of T cell activation via T cell recept | 44598 | 0.144599043 | 0.79670546 |
| sterol import (GO:0035376) | 44598 | 0.144599043 | 0.79670546 |
| tyrosine catabolic process (GO:0006572) | 44598 | 0.144599043 | 0.79670546 |
| valine metabolic process (GO:0006573) | 44598 | 0.144599043 | 0.79670546 |
| positive regulation of apoptotic process (GO: | 42/310 | 0.145286377 | 0.799517479 |
| activation of GTPase activity (GO:0090630) | 16/105 | 0.145891195 | 0.801869125 |
| mitochondrial respiratory chain complex asse | 14/90 | 0.147503526 | 0.805559425 |
| IRE1-mediated unfolded protein response (G | 19603 | 0.148845604 | 0.805559425 |
| protein homooligomerization (GO:0051260) | 18/121 | 0.151407477 | 0.805559425 |
| calcineurin-mediated signaling (GO:0097720) | 44632 | 0.151445088 | 0.805559425 |
| double-strand break repair via break-induced | 44632 | 0.151445088 | 0.805559425 |
| exit from mitosis (GO:0010458) | 44632 | 0.151445088 | 0.805559425 |
| inorganic ion transmembrane transport (GO: | 44632 | 0.151445088 | 0.805559425 |
| intrinsic apoptotic signaling pathway in respo | 44632 | 0.151445088 | 0.805559425 |
| microtubule anchoring (GO:0034453) | 44632 | 0.151445088 | 0.805559425 |
| negative regulation of JUN kinase activity (GC | 44632 | 0.151445088 | 0.805559425 |
| negative regulation of leukocyte apoptotic pr | 44632 | 0.151445088 | 0.805559425 |
| negative regulation of receptor internalizatio | 44632 | 0.151445088 | 0.805559425 |
| negative regulation of release of sequesterec | 44632 | 0.151445088 | 0.805559425 |
| negative regulation of ryanodine-sensitive ca | 44632 | 0.151445088 | 0.805559425 |
| nucleotide-sugar metabolic process (GO:000 | 44632 | 0.151445088 | 0.805559425 |
| positive regulation of actin filament depolym | 44632 | 0.151445088 | 0.805559425 |
| positive regulation of sodium ion transmemb | 44632 | 0.151445088 | 0.805559425 |
| protein import into peroxisome matrix (GO:0 | 44632 | 0.151445088 | 0.805559425 |
| protein localization to condensed chromosor | 44632 | 0.151445088 | 0.805559425 |
| pyrimidine-containing compound metabolic p | 44632 | 0.151445088 | 0.805559425 |
| regulation of chromatin binding (GO:003556 | 44632 | 0.151445088 | 0.805559425 |

| | | | |
|---|---|---|---|
| regulation of dendritic cell differentiation (G( | 44632 | 0.151445088 | 0.805559425 |
| regulation of DNA-dependent DNA replicatio | 44632 | 0.151445088 | 0.805559425 |
| regulation of oxidative stress-induced cell de | 44632 | 0.151445088 | 0.805559425 |
| regulation of triglyceride catabolic process (G | 44632 | 0.151445088 | 0.805559425 |
| replicative senescence (GO:0090399) | 44632 | 0.151445088 | 0.805559425 |
| regulation of double-strand break repair via h | 17015 | 0.151812432 | 0.805559425 |
| 3'-phosphoadenosine 5'-phosphosulfate met | 44706 | 0.151911605 | 0.805559425 |
| peripheral nervous system development (GO | 44706 | 0.151911605 | 0.805559425 |
| regulation of cholesterol transport (GO:0032 | 44706 | 0.151911605 | 0.805559425 |
| long-chain fatty acid metabolic process (GO:( | 13/83 | 0.152756531 | 0.80909028 |
| muscle contraction (GO:0006936) | 19/129 | 0.153578061 | 0.812074298 |
| negative regulation of cellular metabolic proc | 14427 | 0.153859141 | 0.812074298 |
| organophosphate biosynthetic process (GO:C | 14427 | 0.153859141 | 0.812074298 |
| negative regulation of microtubule polymeriz | 11841 | 0.154315301 | 0.812094828 |
| superoxide metabolic process (GO:0006801) | 11841 | 0.154315301 | 0.812094828 |
| protein processing (GO:0016485) | 16/106 | 0.154582017 | 0.812094828 |
| regulation of cell cycle process (GO:0010564) | 16/106 | 0.154582017 | 0.812094828 |
| negative regulation of response to stimulus (( | 22555 | 0.157060065 | 0.823198812 |
| response to light stimulus (GO:0009416) | 22555 | 0.157060065 | 0.823198812 |
| cytoplasmic microtubule organization (GO:0( | 19968 | 0.161566221 | 0.844856695 |
| regulation of proteasomal protein catabolic p | 19968 | 0.161566221 | 0.844856695 |
| endoplasmic reticulum to Golgi vesicle-media | 26/185 | 0.161761054 | 0.84489762 |
| positive regulation of protein transport (GO:( | 25508 | 0.163658213 | 0.853819637 |
| regulation of apoptotic process (GO:0042981 | 94/742 | 0.165342052 | 0.858955526 |
| polyol metabolic process (GO:0019751) | 17380 | 0.165720263 | 0.858955526 |
| establishment of melanosome localization (G | 44670 | 0.166733952 | 0.858955526 |
| mitochondrion localization (GO:0051646) | 44670 | 0.166733952 | 0.858955526 |
| N-glycan processing (GO:0006491) | 44670 | 0.166733952 | 0.858955526 |
| negative regulation of protein localization to | 44670 | 0.166733952 | 0.858955526 |
| NLS-bearing protein import into nucleus (GO: | 44670 | 0.166733952 | 0.858955526 |
| pore complex assembly (GO:0046931) | 44670 | 0.166733952 | 0.858955526 |
| positive regulation of triglyceride metabolic p | 44670 | 0.166733952 | 0.858955526 |
| regulation of neuroinflammatory response (G | 44670 | 0.166733952 | 0.858955526 |
| response to ATP (GO:0033198) | 44670 | 0.166733952 | 0.858955526 |
| regulation of anatomical structure morphoge | 18/123 | 0.168141153 | 0.865218372 |
| carboxylic acid metabolic process (GO:00197 | 14793 | 0.169207185 | 0.869328112 |
| organic acid transport (GO:0015849) | 15/100 | 0.170073371 | 0.869328112 |
| cellular response to drug (GO:0035690) | 44707 | 0.171415145 | 0.869328112 |
| hippo signaling (GO:0035329) | 44707 | 0.171415145 | 0.869328112 |
| purine-containing compound biosynthetic pr | 44707 | 0.171415145 | 0.869328112 |
| apoptotic cell clearance (GO:0043277) | 12206 | 0.17146539 | 0.869328112 |
| apoptotic mitochondrial changes (GO:00086: | 12206 | 0.17146539 | 0.869328112 |
| lytic vacuole organization (GO:0080171) | 12206 | 0.17146539 | 0.869328112 |
| mitotic G2 DNA damage checkpoint signaling | 12206 | 0.17146539 | 0.869328112 |
| phosphatidylcholine biosynthetic process (G( | 12206 | 0.17146539 | 0.869328112 |
| positive regulation of ATP metabolic process | 12206 | 0.17146539 | 0.869328112 |
| primary alcohol metabolic process (GO:0034: | 12206 | 0.17146539 | 0.869328112 |

| | | | |
|---|---|---|---|
| regulation of cholesterol efflux (GO:0010874 | 12206 | 0.17146539 | 0.869328112 |
| glycoprotein metabolic process (GO:000910C | 20333 | 0.174786171 | 0.869328112 |
| cellular response to alcohol (GO:0097306) | 17746 | 0.180187254 | 0.869328112 |
| inositol phosphate metabolic process (GO:00 | 17746 | 0.180187254 | 0.869328112 |
| substrate adhesion-dependent cell spreading | 17746 | 0.180187254 | 0.869328112 |
| negative regulation of apoptotic signaling pat | 28825 | 0.180217884 | 0.869328112 |
| cell part morphogenesis (GO:0032990) | 44633 | 0.18096584 | 0.869328112 |
| cellular component maintenance (GO:00439! | 44633 | 0.18096584 | 0.869328112 |
| cytoplasmic sequestering of protein (GO:005 | 44633 | 0.18096584 | 0.869328112 |
| fatty acid elongation (GO:0030497) | 44633 | 0.18096584 | 0.869328112 |
| glucan biosynthetic process (GO:0009250) | 44633 | 0.18096584 | 0.869328112 |
| glucose import (GO:0046323) | 44633 | 0.18096584 | 0.869328112 |
| glycogen biosynthetic process (GO:0005978) | 44633 | 0.18096584 | 0.869328112 |
| intracellular sterol transport (GO:0032366) | 44633 | 0.18096584 | 0.869328112 |
| macrophage activation involved in immune r | 44633 | 0.18096584 | 0.869328112 |
| negative regulation of hydrogen peroxide-inc | 44633 | 0.18096584 | 0.869328112 |
| negative regulation of macrophage derived f | 44633 | 0.18096584 | 0.869328112 |
| positive regulation of neuron migration (GO:: | 44633 | 0.18096584 | 0.869328112 |
| positive regulation of protein acetylation (GC | 44633 | 0.18096584 | 0.869328112 |
| positive regulation of ruffle assembly (GO:19 | 44633 | 0.18096584 | 0.869328112 |
| positive regulation of vascular endothelial ce | 44633 | 0.18096584 | 0.869328112 |
| protein heterotetramerization (GO:0051290) | 44633 | 0.18096584 | 0.869328112 |
| regulation of actomyosin structure organizati | 44633 | 0.18096584 | 0.869328112 |
| regulation of mitotic cell cycle spindle assem | 44633 | 0.18096584 | 0.869328112 |
| regulation of protein acetylation (GO:190198 | 44633 | 0.18096584 | 0.869328112 |
| response to nitric oxide (GO:0071731) | 44633 | 0.18096584 | 0.869328112 |
| sphingoid biosynthetic process (GO:0046520 | 44633 | 0.18096584 | 0.869328112 |
| sphingosine biosynthetic process (GO:00465: | 44633 | 0.18096584 | 0.869328112 |
| very long-chain fatty acid biosynthetic proces | 44633 | 0.18096584 | 0.869328112 |
| regulation of signal transduction by p53 class | 22/156 | 0.18137285 | 0.869328112 |
| cell cycle G1/S phase transition (GO:0044843 | 23285 | 0.181834349 | 0.869328112 |
| positive regulation of DNA metabolic process | 23285 | 0.181834349 | 0.869328112 |
| positive regulation of endothelial cell migrati | 13/86 | 0.184162653 | 0.869328112 |
| gluconeogenesis (GO:0006094) | 15158 | 0.185192093 | 0.869328112 |
| positive regulation of TOR signaling (GO:0032 | 15158 | 0.185192093 | 0.869328112 |
| regulation of release of cytochrome c from m | 15158 | 0.185192093 | 0.869328112 |
| fatty acid biosynthetic process (GO:0006633) | 26238 | 0.187378869 | 0.869328112 |
| myeloid leukocyte differentiation (GO:00025 | 26238 | 0.187378869 | 0.869328112 |
| aldehyde catabolic process (GO:0046185) | 44599 | 0.187604008 | 0.869328112 |
| amino-acid betaine metabolic process (GO:0( | 44599 | 0.187604008 | 0.869328112 |
| autophagosome membrane docking (GO:001 | 44599 | 0.187604008 | 0.869328112 |
| bleb assembly (GO:0032060) | 44599 | 0.187604008 | 0.869328112 |
| cellular hypotonic response (GO:0071476) | 44599 | 0.187604008 | 0.869328112 |
| cellular nitrogen compound catabolic proces | 44599 | 0.187604008 | 0.869328112 |
| cellular response to interleukin-18 (GO:0071: | 44599 | 0.187604008 | 0.869328112 |
| cellular response to prostaglandin E stimulus | 44599 | 0.187604008 | 0.869328112 |
| cyclic nucleotide transport (GO:0070729) | 44599 | 0.187604008 | 0.869328112 |

| | | | |
|---|---|---|---|
| flavin-containing compound metabolic proce | 44599 | 0.187604008 | 0.869328112 |
| G-quadruplex DNA unwinding (GO:0044806) | 44599 | 0.187604008 | 0.869328112 |
| glomerular epithelial cell development (GO:0 | 44599 | 0.187604008 | 0.869328112 |
| hexose catabolic process (GO:0019320) | 44599 | 0.187604008 | 0.869328112 |
| interleukin-18-mediated signaling pathway (( | 44599 | 0.187604008 | 0.869328112 |
| L-methionine salvage (GO:0071267) | 44599 | 0.187604008 | 0.869328112 |
| membrane fission (GO:0090148) | 44599 | 0.187604008 | 0.869328112 |
| mitochondrial DNA metabolic process (GO:0( | 44599 | 0.187604008 | 0.869328112 |
| negative regulation of dendritic cell apoptoti | 44599 | 0.187604008 | 0.869328112 |
| negative regulation of low-density lipoprotei | 44599 | 0.187604008 | 0.869328112 |
| nicotinamide nucleotide biosynthetic process | 44599 | 0.187604008 | 0.869328112 |
| Notch receptor processing, ligand-dependent | 44599 | 0.187604008 | 0.869328112 |
| pentose metabolic process (GO:0019321) | 44599 | 0.187604008 | 0.869328112 |
| peptide transport (GO:0015833) | 44599 | 0.187604008 | 0.869328112 |
| photoreceptor cell differentiation (GO:00465 | 44599 | 0.187604008 | 0.869328112 |
| positive regulation of helicase activity (GO:0( | 44599 | 0.187604008 | 0.869328112 |
| positive regulation of lipopolysaccharide-me( | 44599 | 0.187604008 | 0.869328112 |
| positive regulation of mitophagy in response | 44599 | 0.187604008 | 0.869328112 |
| positive regulation of mitotic cell cycle spindl | 44599 | 0.187604008 | 0.869328112 |
| positive regulation of oxidative stress-induce | 44599 | 0.187604008 | 0.869328112 |
| positive regulation of spindle checkpoint (GO | 44599 | 0.187604008 | 0.869328112 |
| positive regulation of striated muscle contrac | 44599 | 0.187604008 | 0.869328112 |
| protection from non-homologous end joining | 44599 | 0.187604008 | 0.869328112 |
| proton-transporting ATP synthase complex a: | 44599 | 0.187604008 | 0.869328112 |
| pyrimidine ribonucleotide biosynthetic proce | 44599 | 0.187604008 | 0.869328112 |
| pyrimidine-containing compound biosyntheti | 44599 | 0.187604008 | 0.869328112 |
| regulation by virus of viral protein levels in h( | 44599 | 0.187604008 | 0.869328112 |
| regulation of fat cell proliferation (GO:0070З4 | 44599 | 0.187604008 | 0.869328112 |
| regulation of glial cell apoptotic process (GO: | 44599 | 0.187604008 | 0.869328112 |
| regulation of glucocorticoid receptor signalin | 44599 | 0.187604008 | 0.869328112 |
| regulation of metallopeptidase activity (GO:1 | 44599 | 0.187604008 | 0.869328112 |
| regulation of microtubule binding (GO:19045 | 44599 | 0.187604008 | 0.869328112 |
| regulation of mitochondrial membrane perm | 44599 | 0.187604008 | 0.869328112 |
| regulation of oxidative stress-induced neuror | 44599 | 0.187604008 | 0.869328112 |
| regulation of T-helper 1 cell differentiation (( | 44599 | 0.187604008 | 0.869328112 |
| riboflavin metabolic process (GO:0006771) | 44599 | 0.187604008 | 0.869328112 |
| serine transport (GO:0032329) | 44599 | 0.187604008 | 0.869328112 |
| thiamine-containing compound metabolic pr | 44599 | 0.187604008 | 0.869328112 |
| phosphatidylserine metabolic process (GO:0( | 12571 | 0.189358815 | 0.873874492 |
| positive regulation of mitochondrial outer m( | 12571 | 0.189358815 | 0.873874492 |
| tRNA export from nucleus (GO:0006409) | 12571 | 0.189358815 | 0.873874492 |
| tRNA-containing ribonucleoprotein complex ( | 12571 | 0.189358815 | 0.873874492 |
| energy reserve metabolic process (GO:00061 | 44671 | 0.190676166 | 0.874796407 |
| eye morphogenesis (GO:0048592) | 44671 | 0.190676166 | 0.874796407 |
| miRNA metabolic process (GO:0010586) | 44671 | 0.190676166 | 0.874796407 |
| positive regulation of cyclin-dependent prote | 44671 | 0.190676166 | 0.874796407 |
| skeletal muscle contraction (GO:0003009) | 44671 | 0.190676166 | 0.874796407 |

| | | | |
|---|---|---|---|
| positive regulation of programmed cell death | 38/286 | 0.190720332 | 0.874796407 |
| dendritic spine organization (GO:0097061) | 44708 | 0.191819859 | 0.875396085 |
| negative regulation of cation channel activity | 44708 | 0.191819859 | 0.875396085 |
| negative regulation of protein polymerization | 44708 | 0.191819859 | 0.875396085 |
| peptidyl-lysine methylation (GO:0018022) | 44708 | 0.191819859 | 0.875396085 |
| positive regulation of mitotic cell cycle (GO:0 | 44708 | 0.191819859 | 0.875396085 |
| pre-replicative complex assembly (GO:00363 | 23651 | 0.194852164 | 0.88536978 |
| protein catabolic process (GO:0030163) | 23651 | 0.194852164 | 0.88536978 |
| regulation of cilium assembly (GO:1902017) | 23651 | 0.194852164 | 0.88536978 |
| aminoglycan biosynthetic process (GO:00060 | 18111 | 0.19518112 | 0.88536978 |
| oligosaccharide metabolic process (GO:0009: | 18111 | 0.19518112 | 0.88536978 |
| response to hydrogen peroxide (GO:0042542 | 18111 | 0.19518112 | 0.88536978 |
| negative regulation of supramolecular fiber c | 26604 | 0.199797502 | 0.904494102 |
| response to ionizing radiation (GO:0010212) | 26604 | 0.199797502 | 0.904494102 |
| monocarboxylic acid transport (GO:0015718) | 21064 | 0.20262671 | 0.91638386 |
| organelle organization (GO:0006996) | 54/420 | 0.205930171 | 0.923651142 |
| intracellular pH reduction (GO:0051452) | 12936 | 0.207930137 | 0.923651142 |
| negative regulation of cell cycle G1/S phase t | 12936 | 0.207930137 | 0.923651142 |
| positive regulation of immunoglobulin produ | 12936 | 0.207930137 | 0.923651142 |
| regulation of phosphatidylinositol 3-kinase ac | 12936 | 0.207930137 | 0.923651142 |
| steroid biosynthetic process (GO:0006694) | 24016 | 0.208262433 | 0.923651142 |
| translational termination (GO:0006415) | 14/96 | 0.20902445 | 0.923651142 |
| translational elongation (GO:0006414) | 15/104 | 0.210835728 | 0.923651142 |
| 2-oxoglutarate metabolic process (GO:00061 | 44634 | 0.211849732 | 0.923651142 |
| amyloid precursor protein catabolic process ( | 44634 | 0.211849732 | 0.923651142 |
| cellular response to low-density lipoprotein p | 44634 | 0.211849732 | 0.923651142 |
| coenzyme A metabolic process (GO:0015936 | 44634 | 0.211849732 | 0.923651142 |
| COPII-coated vesicle cargo loading (GO:0090: | 44634 | 0.211849732 | 0.923651142 |
| covalent chromatin modification (GO:001656 | 44634 | 0.211849732 | 0.923651142 |
| cytoplasmic sequestering of transcription fac | 44634 | 0.211849732 | 0.923651142 |
| negative regulation of DNA damage response | 44634 | 0.211849732 | 0.923651142 |
| negative regulation of neuroinflammatory re | 44634 | 0.211849732 | 0.923651142 |
| negative regulation of pri-miRNA transcriptio | 44634 | 0.211849732 | 0.923651142 |
| negative regulation of T cell differentiation (( | 44634 | 0.211849732 | 0.923651142 |
| nucleoside phosphate catabolic process (GO: | 44634 | 0.211849732 | 0.923651142 |
| peptidyl-serine dephosphorylation (GO:0070 | 44634 | 0.211849732 | 0.923651142 |
| positive regulation of cellular response to ins | 44634 | 0.211849732 | 0.923651142 |
| positive regulation of glucose metabolic proc | 44634 | 0.211849732 | 0.923651142 |
| positive regulation of glycoprotein biosynthe | 44634 | 0.211849732 | 0.923651142 |
| positive regulation of SMAD protein signal tra | 44634 | 0.211849732 | 0.923651142 |
| positive regulation of T-helper cell differentia | 44634 | 0.211849732 | 0.923651142 |
| positive regulation of type 2 immune respons | 44634 | 0.211849732 | 0.923651142 |
| protein localization to kinetochore (GO:0034 | 44634 | 0.211849732 | 0.923651142 |
| pyrimidine nucleoside biosynthetic process (( | 44634 | 0.211849732 | 0.923651142 |
| regulation of cell projection organization (GC | 44634 | 0.211849732 | 0.923651142 |
| regulation of integrin-mediated signaling patl | 44634 | 0.211849732 | 0.923651142 |
| regulation of response to endoplasmic reticu | 44634 | 0.211849732 | 0.923651142 |

| | | | |
|---|---|---|---|
| response to UV-A (GO:0070141) | 44634 | 0.211849732 | 0.923651142 |
| steroid catabolic process (GO:0006706) | 44634 | 0.211849732 | 0.923651142 |
| sulfate transport (GO:0008272) | 44634 | 0.211849732 | 0.923651142 |
| phosphatidylinositol metabolic process (GO:( | 16/112 | 0.212208859 | 0.923651142 |
| endoplasmic reticulum organization (GO:000 | 26969 | 0.212564792 | 0.923651142 |
| calcium ion import (GO:0070509) | 44709 | 0.213024456 | 0.923651142 |
| L-amino acid transport (GO:0015807) | 44709 | 0.213024456 | 0.923651142 |
| phagosome acidification (GO:0090383) | 44709 | 0.213024456 | 0.923651142 |
| positive regulation of phosphatidylinositol 3- | 44709 | 0.213024456 | 0.923651142 |
| regulation of sodium ion transmembrane tra | 44709 | 0.213024456 | 0.923651142 |
| sulfur compound transport (GO:0072348) | 44709 | 0.213024456 | 0.923651142 |
| calcium ion transport into cytosol (GO:00604 | 44672 | 0.215610864 | 0.92615706 |
| cellular response to estradiol stimulus (GO:0( | 44672 | 0.215610864 | 0.92615706 |
| extrinsic apoptotic signaling pathway in abse | 44672 | 0.215610864 | 0.92615706 |
| intrinsic apoptotic signaling pathway in respo | 44672 | 0.215610864 | 0.92615706 |
| melanosome localization (GO:0032400) | 44672 | 0.215610864 | 0.92615706 |
| positive regulation of glycolytic process (GO:( | 44672 | 0.215610864 | 0.92615706 |
| positive regulation of purine nucleotide meta | 44672 | 0.215610864 | 0.92615706 |
| positive regulation of smoothened signaling p | 44672 | 0.215610864 | 0.92615706 |
| activation of cysteine-type endopeptidase ac | 29921 | 0.215857323 | 0.92615706 |
| negative regulation of protein modification p | 29921 | 0.215857323 | 0.92615706 |
| positive regulation of tumor necrosis factor s | 29921 | 0.215857323 | 0.92615706 |
| mitochondrial translational termination (GO: | 13/89 | 0.218367714 | 0.936039213 |
| cellular response to glucose starvation (GO:0 | 15888 | 0.218889334 | 0.936498118 |
| negative regulation of proteolysis (GO:00458 | 15888 | 0.218889334 | 0.936498118 |
| dephosphorylation (GO:0016311) | 21/153 | 0.223672235 | 0.94267407 |
| regulation of peptide hormone secretion (GC | 27334 | 0.225661505 | 0.94267407 |
| mitotic nuclear membrane organization (GO: | 18841 | 0.226607776 | 0.94267407 |
| mitotic nuclear membrane reassembly (GO:0 | 18841 | 0.226607776 | 0.94267407 |
| intrinsic apoptotic signaling pathway by p53 ( | 13302 | 0.227110428 | 0.94267407 |
| positive regulation of telomere maintenance | 13302 | 0.227110428 | 0.94267407 |
| regulation of protein import into nucleus (GC | 13302 | 0.227110428 | 0.94267407 |
| Wnt signaling pathway, calcium modulating p | 13302 | 0.227110428 | 0.94267407 |
| antigen processing and presentation of exoge | 14/98 | 0.231748171 | 0.94267407 |
| aspartate family amino acid biosynthetic pro | 44600 | 0.232017168 | 0.94267407 |
| benzene-containing compound metabolic pro | 44600 | 0.232017168 | 0.94267407 |
| chromatin silencing at telomere (GO:000634 | 44600 | 0.232017168 | 0.94267407 |
| dendritic spine maintenance (GO:0097062) | 44600 | 0.232017168 | 0.94267407 |
| ER overload response (GO:0006983) | 44600 | 0.232017168 | 0.94267407 |
| glyoxylate metabolic process (GO:0046487) | 44600 | 0.232017168 | 0.94267407 |
| heparan sulfate proteoglycan biosynthetic pr | 44600 | 0.232017168 | 0.94267407 |
| isoprenoid metabolic process (GO:0006720) | 44600 | 0.232017168 | 0.94267407 |
| macromolecule depalmitoylation (GO:00987 | 44600 | 0.232017168 | 0.94267407 |
| macromolecule modification (GO:0043412) | 44600 | 0.232017168 | 0.94267407 |
| mitotic spindle elongation (GO:0000022) | 44600 | 0.232017168 | 0.94267407 |
| mitotic spindle midzone assembly (GO:00512 | 44600 | 0.232017168 | 0.94267407 |
| multi-ciliated epithelial cell differentiation (G | 44600 | 0.232017168 | 0.94267407 |

| | | | |
|---|---|---|---|
| negative regulation of actin nucleation (GO:0 | 44600 | 0.232017168 | 0.94267407 |
| negative regulation of appetite (GO:0032099 | 44600 | 0.232017168 | 0.94267407 |
| negative regulation of cAMP-dependent prot | 44600 | 0.232017168 | 0.94267407 |
| negative regulation of mitochondrial fusion ( | 44600 | 0.232017168 | 0.94267407 |
| negative regulation of mitotic nuclear divisio | 44600 | 0.232017168 | 0.94267407 |
| negative regulation of monooxygenase activi | 44600 | 0.232017168 | 0.94267407 |
| negative regulation of neural precursor cell p | 44600 | 0.232017168 | 0.94267407 |
| negative regulation of response to reactive o | 44600 | 0.232017168 | 0.94267407 |
| peptidyl-lysine hydroxylation (GO:0017185) | 44600 | 0.232017168 | 0.94267407 |
| peptidyl-proline hydroxylation to 4-hydroxy-l | 44600 | 0.232017168 | 0.94267407 |
| positive regulation of adaptive immune respo | 44600 | 0.232017168 | 0.94267407 |
| positive regulation of execution phase of apo | 44600 | 0.232017168 | 0.94267407 |
| positive regulation of insulin-like growth fact | 44600 | 0.232017168 | 0.94267407 |
| positive regulation of melanin biosynthetic p | 44600 | 0.232017168 | 0.94267407 |
| positive regulation of membrane invaginatio | 44600 | 0.232017168 | 0.94267407 |
| positive regulation of neuron projection arbo | 44600 | 0.232017168 | 0.94267407 |
| positive regulation of secondary metabolite b | 44600 | 0.232017168 | 0.94267407 |
| protein deglutamylation (GO:0035608) | 44600 | 0.232017168 | 0.94267407 |
| protein depalmitoylation (GO:0002084) | 44600 | 0.232017168 | 0.94267407 |
| pyrimidine nucleobase catabolic process (GO | 44600 | 0.232017168 | 0.94267407 |
| regulation of extracellular matrix constituent | 44600 | 0.232017168 | 0.94267407 |
| regulation of oxidative stress-induced neuror | 44600 | 0.232017168 | 0.94267407 |
| regulation of protein ADP-ribosylation (GO:0 | 44600 | 0.232017168 | 0.94267407 |
| regulation of protein glycosylation (GO:0060 | 44600 | 0.232017168 | 0.94267407 |
| regulation of T-helper 2 cell differentiation (G | 44600 | 0.232017168 | 0.94267407 |
| response to hydroperoxide (GO:0033194) | 44600 | 0.232017168 | 0.94267407 |
| ribosome disassembly (GO:0032790) | 44600 | 0.232017168 | 0.94267407 |
| single strand break repair (GO:0000012) | 44600 | 0.232017168 | 0.94267407 |
| skeletal muscle tissue regeneration (GO:0043 | 44600 | 0.232017168 | 0.94267407 |
| spermidine metabolic process (GO:0008216) | 44600 | 0.232017168 | 0.94267407 |
| stress-induced premature senescence (GO:0 | 44600 | 0.232017168 | 0.94267407 |
| T cell mediated cytotoxicity (GO:0001913) | 44600 | 0.232017168 | 0.94267407 |
| tubulin complex assembly (GO:0007021) | 44600 | 0.232017168 | 0.94267407 |
| vitamin K metabolic process (GO:0042373) | 44600 | 0.232017168 | 0.94267407 |
| chromosome organization (GO:0051276) | 15/106 | 0.232716597 | 0.944666292 |
| glycolytic process (GO:0006096) | 44710 | 0.234924124 | 0.950212348 |
| negative regulation of myeloid leukocyte diff | 44710 | 0.234924124 | 0.950212348 |
| protein K11-linked ubiquitination (GO:00709 | 44710 | 0.234924124 | 0.950212348 |
| telomere maintenance via telomerase (GO:0 | 44710 | 0.234924124 | 0.950212348 |
| supramolecular fiber organization (GO:00974 | 45/351 | 0.236447296 | 0.954036368 |
| positive regulation of potassium ion transme | 16254 | 0.236503039 | 0.954036368 |
| response to amyloid-beta (GO:1904645) | 16254 | 0.236503039 | 0.954036368 |
| cellular response to osmotic stress (GO:0071 | 44673 | 0.241362618 | 0.955938473 |
| maturation of 5.8S rRNA from tricistronic rRN | 44673 | 0.241362618 | 0.955938473 |
| mitochondrial calcium ion homeostasis (GO:( | 44673 | 0.241362618 | 0.955938473 |
| positive regulation of calcium ion transmemb | 44673 | 0.241362618 | 0.955938473 |
| positive regulation of lipid catabolic process ( | 44673 | 0.241362618 | 0.955938473 |

| | | | |
|---|---|---|---|
| protein import into peroxisome membrane (( | 44673 | 0.241362618 | 0.955938473 |
| regulation of execution phase of apoptosis (G | 44673 | 0.241362618 | 0.955938473 |
| ameboidal-type cell migration (GO:0001667) | 19207 | 0.242964516 | 0.955938473 |
| negative regulation of protein metabolic proc | 19207 | 0.242964516 | 0.955938473 |
| negative regulation of protein-containing cor | 19207 | 0.242964516 | 0.955938473 |
| glucan catabolic process (GO:0009251) | 44635 | 0.243745268 | 0.955938473 |
| glucosamine-containing compound metaboli | 44635 | 0.243745268 | 0.955938473 |
| glycogen catabolic process (GO:0005980) | 44635 | 0.243745268 | 0.955938473 |
| intracellular cholesterol transport (GO:00323 | 44635 | 0.243745268 | 0.955938473 |
| mitochondrial RNA metabolic process (GO:00 | 44635 | 0.243745268 | 0.955938473 |
| modulation by host of viral genome replicatio | 44635 | 0.243745268 | 0.955938473 |
| negative regulation of leukocyte activation (( | 44635 | 0.243745268 | 0.955938473 |
| phosphatidylethanolamine biosynthetic proc | 44635 | 0.243745268 | 0.955938473 |
| photoreceptor cell development (GO:004246 | 44635 | 0.243745268 | 0.955938473 |
| placenta development (GO:0001890) | 44635 | 0.243745268 | 0.955938473 |
| positive regulation of chromosome organizat | 44635 | 0.243745268 | 0.955938473 |
| positive regulation of histone H3-K4 methyla | 44635 | 0.243745268 | 0.955938473 |
| positive regulation of protein export from nu | 44635 | 0.243745268 | 0.955938473 |
| prostanoid biosynthetic process (GO:004645 | 44635 | 0.243745268 | 0.955938473 |
| protein hydroxylation (GO:0018126) | 44635 | 0.243745268 | 0.955938473 |
| regulation of lymphocyte activation (GO:005 | 44635 | 0.243745268 | 0.955938473 |
| regulation of microglial cell activation (GO:19 | 44635 | 0.243745268 | 0.955938473 |
| regulation of neuron projection arborization | 44635 | 0.243745268 | 0.955938473 |
| RNA phosphodiester bond hydrolysis, endonu | 44635 | 0.243745268 | 0.955938473 |
| synaptonemal complex assembly (GO:00071 | 44635 | 0.243745268 | 0.955938473 |
| synaptonemal complex organization (GO:007 | 44635 | 0.243745268 | 0.955938473 |
| tRNA splicing, via endonucleolytic cleavage a | 44635 | 0.243745268 | 0.955938473 |
| glutamine family amino acid metabolic proce | 13667 | 0.24682824 | 0.965515142 |
| negative regulation of G1/S transition of mito | 13667 | 0.24682824 | 0.965515142 |
| phagosome maturation (GO:0090382) | 13667 | 0.24682824 | 0.965515142 |
| positive regulation of intracellular protein tra | 20/148 | 0.252540837 | 0.98700649 |
| regulation of intracellular signal transduction | 55/437 | 0.253168118 | 0.988602902 |
| vascular transport (GO:0010232) | 31017 | 0.254170292 | 0.990603611 |
| amino acid transmembrane transport (GO:00 | 16619 | 0.254557502 | 0.990603611 |
| cellular response to radiation (GO:0071478) | 16619 | 0.254557502 | 0.990603611 |
| mitotic G2/M transition checkpoint (GO:004 | 16619 | 0.254557502 | 0.990603611 |
| positive regulation of cellular component bio | 13/92 | 0.255015349 | 0.991404918 |
| ion transport (GO:0006811) | 16/116 | 0.255265561 | 0.991404918 |
| antigen processing and presentation of pepti | 14/100 | 0.255421719 | 0.991404918 |
| fatty-acyl-CoA biosynthetic process (GO:0046 | 44711 | 0.257412259 | 0.993158487 |
| long-chain fatty acid biosynthetic process (G( | 44711 | 0.257412259 | 0.993158487 |
| negative regulation of gene expression, epige | 44711 | 0.257412259 | 0.993158487 |
| positive regulation of lipid kinase activity (GC | 44711 | 0.257412259 | 0.993158487 |
| primary neural tube formation (GO:0014020) | 44711 | 0.257412259 | 0.993158487 |
| regulation of protein localization to membrai | 44711 | 0.257412259 | 0.993158487 |
| RNA-dependent DNA biosynthetic process (G | 44711 | 0.257412259 | 0.993158487 |
| regulation of protein secretion (GO:0050708) | 17/125 | 0.26569081 | 0.999994883 |

| | | | |
|---|---|---|---|
| positive regulation of tumor necrosis factor p | 28430 | 0.266719991 | 0.999994883 |
| regulation of organelle assembly (GO:190211 | 28430 | 0.266719991 | 0.999994883 |
| lysosome organization (GO:0007040) | 14032 | 0.267010528 | 0.999994883 |
| ncRNA export from nucleus (GO:0097064) | 14032 | 0.267010528 | 0.999994883 |
| positive regulation of protein localization to r | 14/101 | 0.267580297 | 0.999994883 |
| DNA catabolic process, endonucleolytic (GO:( | 44674 | 0.267758063 | 0.999994883 |
| DNA methylation (GO:0006306) | 44674 | 0.267758063 | 0.999994883 |
| membrane protein ectodomain proteolysis (( | 44674 | 0.267758063 | 0.999994883 |
| negative regulation of intracellular protein tr | 44674 | 0.267758063 | 0.999994883 |
| peptidyl-L-cysteine S-palmitoylation (GO:001 | 44674 | 0.267758063 | 0.999994883 |
| peptidyl-S-diacylglycerol-L-cysteine biosynthe | 44674 | 0.267758063 | 0.999994883 |
| protein alkylation (GO:0008213) | 44674 | 0.267758063 | 0.999994883 |
| regulation of interleukin-4 production (GO:0( | 44674 | 0.267758063 | 0.999994883 |
| regulation of protein export from nucleus (G( | 44674 | 0.267758063 | 0.999994883 |
| regulation of SMAD protein signal transductio | 44674 | 0.267758063 | 0.999994883 |
| cellular response to starvation (GO:0009267) | 21/158 | 0.27087078 | 0.999994883 |
| protein localization to organelle (GO:003336! | 19/142 | 0.273942893 | 0.999994883 |
| inorganic cation transmembrane transport (( | 35/274 | 0.27623423 | 0.999994883 |
| cellular polysaccharide catabolic process (GO | 44636 | 0.276323392 | 0.999994883 |
| dendritic cell chemotaxis (GO:0002407) | 44636 | 0.276323392 | 0.999994883 |
| negative regulation of immune effector proc | 44636 | 0.276323392 | 0.999994883 |
| polyol biosynthetic process (GO:0046173) | 44636 | 0.276323392 | 0.999994883 |
| positive regulation of immunoglobulin media | 44636 | 0.276323392 | 0.999994883 |
| positive regulation of small molecule metabo | 44636 | 0.276323392 | 0.999994883 |
| protein localization to microtubule organizing | 44636 | 0.276323392 | 0.999994883 |
| regulation of ATP biosynthetic process (GO:2 | 44636 | 0.276323392 | 0.999994883 |
| regulation of cholesterol storage (GO:001088 | 44636 | 0.276323392 | 0.999994883 |
| regulation of ERBB signaling pathway (GO:19 | 44636 | 0.276323392 | 0.999994883 |
| response to organophosphorus (GO:0046683 | 44636 | 0.276323392 | 0.999994883 |
| RNA splicing, via endonucleolytic cleavage an | 44636 | 0.276323392 | 0.999994883 |
| serine family amino acid metabolic process (( | 44636 | 0.276323392 | 0.999994883 |
| T cell mediated immunity (GO:0002456) | 44636 | 0.276323392 | 0.999994883 |
| nuclear membrane reassembly (GO:0031468 | 19937 | 0.276762947 | 0.999994883 |
| unsaturated fatty acid metabolic process (GC | 19937 | 0.276762947 | 0.999994883 |
| alpha-amino acid biosynthetic process (GO:1! | 44601 | 0.27694827 | 0.999994883 |
| bone resorption (GO:0045453) | 44601 | 0.27694827 | 0.999994883 |
| cilium movement involved in cell motility (G( | 44601 | 0.27694827 | 0.999994883 |
| endosome to melanosome transport (GO:00: | 44601 | 0.27694827 | 0.999994883 |
| endosome to pigment granule transport (GO | 44601 | 0.27694827 | 0.999994883 |
| glomerular filtration (GO:0003094) | 44601 | 0.27694827 | 0.999994883 |
| glucosamine-containing compound catabolic | 44601 | 0.27694827 | 0.999994883 |
| inositol metabolic process (GO:0006020) | 44601 | 0.27694827 | 0.999994883 |
| kynurenine metabolic process (GO:0070189) | 44601 | 0.27694827 | 0.999994883 |
| lipoprotein metabolic process (GO:0042157) | 44601 | 0.27694827 | 0.999994883 |
| lysosomal lumen acidification (GO:0007042) | 44601 | 0.27694827 | 0.999994883 |
| negative regulation of nitric oxide biosynthet | 44601 | 0.27694827 | 0.999994883 |
| negative regulation of nitric oxide metabolic | 44601 | 0.27694827 | 0.999994883 |

| | | | |
|---|---|---|---|
| neuronal ion channel clustering (GO:0045161 | 44601 | 0.27694827 | 0.999994883 |
| Notch receptor processing (GO:0007220) | 44601 | 0.27694827 | 0.999994883 |
| nucleoside diphosphate catabolic process (G( | 44601 | 0.27694827 | 0.999994883 |
| oligosaccharide catabolic process (GO:00093 | 44601 | 0.27694827 | 0.999994883 |
| organic cyclic compound catabolic process (G | 44601 | 0.27694827 | 0.999994883 |
| peptidyl-cysteine modification (GO:0018198) | 44601 | 0.27694827 | 0.999994883 |
| peripheral nervous system axon ensheathme | 44601 | 0.27694827 | 0.999994883 |
| peroxisome fission (GO:0016559) | 44601 | 0.27694827 | 0.999994883 |
| positive regulation of autophagy of mitochor | 44601 | 0.27694827 | 0.999994883 |
| positive regulation of ligase activity (GO:0051 | 44601 | 0.27694827 | 0.999994883 |
| positive regulation of megakaryocyte differer | 44601 | 0.27694827 | 0.999994883 |
| positive regulation of phagocytosis, engulfme | 44601 | 0.27694827 | 0.999994883 |
| positive regulation of smooth muscle cell apc | 44601 | 0.27694827 | 0.999994883 |
| protein hexamerization (GO:0034214) | 44601 | 0.27694827 | 0.999994883 |
| regulation of cellular response to oxidative st | 44601 | 0.27694827 | 0.999994883 |
| regulation of dendritic cell antigen processing | 44601 | 0.27694827 | 0.999994883 |
| regulation of ER-associated ubiquitin-depend | 44601 | 0.27694827 | 0.999994883 |
| regulation of fertilization (GO:0080154) | 44601 | 0.27694827 | 0.999994883 |
| regulation of helicase activity (GO:0051095) | 44601 | 0.27694827 | 0.999994883 |
| regulation of mitotic centrosome separation | 44601 | 0.27694827 | 0.999994883 |
| regulation of nuclear cell cycle DNA replicatic | 44601 | 0.27694827 | 0.999994883 |
| response to UV-C (GO:0010225) | 44601 | 0.27694827 | 0.999994883 |
| response to vitamin (GO:0033273) | 44601 | 0.27694827 | 0.999994883 |
| Schwann cell development (GO:0014044) | 44601 | 0.27694827 | 0.999994883 |
| synapse pruning (GO:0098883) | 44601 | 0.27694827 | 0.999994883 |
| tetrahydrofolate interconversion (GO:003599 | 44601 | 0.27694827 | 0.999994883 |
| unsaturated fatty acid biosynthetic process (( | 44601 | 0.27694827 | 0.999994883 |
| regulation of protein phosphorylation (GO:0( | 34/266 | 0.278501736 | 0.999994883 |
| mitotic sister chromatid segregation (GO:000 | 14/102 | 0.279934011 | 0.999994883 |
| regulation of proteasomal ubiquitin-depende | 14/102 | 0.279934011 | 0.999994883 |
| embryonic skeletal system morphogenesis (G | 44712 | 0.280382019 | 0.999994883 |
| positive regulation of locomotion (GO:00400 | 44712 | 0.280382019 | 0.999994883 |
| positive regulation of mononuclear cell migra | 44712 | 0.280382019 | 0.999994883 |
| positive regulation of proteolysis involved in | 44712 | 0.280382019 | 0.999994883 |
| regulation of macrophage derived foam cell ( | 44712 | 0.280382019 | 0.999994883 |
| regulation of exit from mitosis (GO:0007096) | 14397 | 0.287583506 | 0.999994883 |
| positive regulation of cysteine-type endopep | 16/119 | 0.289541528 | 0.999994883 |
| determination of heart left/right asymmetry | 17349 | 0.291772687 | 0.999994883 |
| embryonic heart tube morphogenesis (GO:0( | 17349 | 0.291772687 | 0.999994883 |
| antigen processing and presentation of exoge | 14/103 | 0.292467759 | 0.999994883 |
| negative regulation of protein phosphorylatic | 20/152 | 0.292878915 | 0.999994883 |
| regulation of cell cycle phase transition (GO:1 | 13/95 | 0.293685661 | 0.999994883 |
| negative regulation of cytokine-mediated sig | 20302 | 0.294120694 | 0.999994883 |
| pyruvate metabolic process (GO:0006090) | 20302 | 0.294120694 | 0.999994883 |
| triglyceride metabolic process (GO:0006641) | 20302 | 0.294120694 | 0.999994883 |
| canonical glycolysis (GO:0061621) | 44675 | 0.294628646 | 0.999994883 |
| cholesterol efflux (GO:0033344) | 44675 | 0.294628646 | 0.999994883 |

| | | | |
|---|---|---|---|
| dendritic cell differentiation (GO:0097028) | 44675 | 0.294628646 | 0.999994883 |
| glucose catabolic process to pyruvate (GO:00 | 44675 | 0.294628646 | 0.999994883 |
| leukotriene metabolic process (GO:0006691) | 44675 | 0.294628646 | 0.999994883 |
| positive regulation of viral life cycle (GO:190: | 44675 | 0.294628646 | 0.999994883 |
| protein kinase B signaling (GO:0043491) | 44675 | 0.294628646 | 0.999994883 |
| purine ribonucleotide catabolic process (GO:( | 44675 | 0.294628646 | 0.999994883 |
| cellular response to reactive oxygen species ( | 23255 | 0.295268062 | 0.999994883 |
| positive regulation of cellular protein localiza | 26207 | 0.295595341 | 0.999994883 |
| regulation of glycolytic process (GO:0006110 | 26207 | 0.295595341 | 0.999994883 |
| negative regulation of macromolecule metab | 25/194 | 0.300390018 | 0.999994883 |
| protein-containing complex subunit organiza | 15/112 | 0.303267863 | 0.999994883 |
| negative regulation of double-strand break re | 11810 | 0.303727704 | 0.999994883 |
| protein transmembrane import into intracell | 11810 | 0.303727704 | 0.999994883 |
| regulation of cell morphogenesis (GO:00226( | 11810 | 0.303727704 | 0.999994883 |
| regulation of dendrite morphogenesis (GO:0( | 11810 | 0.303727704 | 0.999994883 |
| regulation of dendritic spine morphogenesis | 11810 | 0.303727704 | 0.999994883 |
| regulation of intracellular estrogen receptor : | 11810 | 0.303727704 | 0.999994883 |
| RNA biosynthetic process (GO:0032774) | 11810 | 0.303727704 | 0.999994883 |
| organic hydroxy compound transport (GO:00 | 14763 | 0.308473443 | 0.999994883 |
| regulation of nitric oxide biosynthetic proces: | 14763 | 0.308473443 | 0.999994883 |
| cellular response to gamma radiation (GO:00 | 44637 | 0.30928159 | 0.999994883 |
| copper ion homeostasis (GO:0055070) | 44637 | 0.30928159 | 0.999994883 |
| negative regulation of amyloid-beta formatio | 44637 | 0.30928159 | 0.999994883 |
| preassembly of GPI anchor in ER membrane ( | 44637 | 0.30928159 | 0.999994883 |
| protein localization to chromosome, centrom | 44637 | 0.30928159 | 0.999994883 |
| purine ribonucleotide transport (GO:0015868 | 44637 | 0.30928159 | 0.999994883 |
| regulation of cholesterol biosynthetic proces: | 44637 | 0.30928159 | 0.999994883 |
| response to purine-containing compound (G( | 44637 | 0.30928159 | 0.999994883 |
| reverse cholesterol transport (GO:0043691) | 44637 | 0.30928159 | 0.999994883 |
| tail-anchored membrane protein insertion in | 44637 | 0.30928159 | 0.999994883 |
| purine ribonucleotide biosynthetic process (( | 17715 | 0.310823856 | 0.999994883 |
| cell morphogenesis involved in differentiatio | 26573 | 0.311046603 | 0.999994883 |
| regulation of extrinsic apoptotic signaling pat | 23621 | 0.311699752 | 0.999994883 |
| cellular biogenic amine catabolic process (GC | 44602 | 0.321692826 | 0.999994883 |
| cellular response to hydroxyurea (GO:00727 | 44602 | 0.321692826 | 0.999994883 |
| cellular response to oxygen levels (GO:00714 | 44602 | 0.321692826 | 0.999994883 |
| cellular response to prostaglandin stimulus (( | 44602 | 0.321692826 | 0.999994883 |
| defense response to tumor cell (GO:0002357 | 44602 | 0.321692826 | 0.999994883 |
| DNA damage response, signal transduction b | 44602 | 0.321692826 | 0.999994883 |
| fructose 6-phosphate metabolic process (GO | 44602 | 0.321692826 | 0.999994883 |
| histone H4 deacetylation (GO:0070933) | 44602 | 0.321692826 | 0.999994883 |
| iron ion transmembrane transport (GO:0034 | 44602 | 0.321692826 | 0.999994883 |
| L-serine metabolic process (GO:0006563) | 44602 | 0.321692826 | 0.999994883 |
| lipid droplet formation (GO:0140042) | 44602 | 0.321692826 | 0.999994883 |
| microtubule anchoring at centrosome (GO:0( | 44602 | 0.321692826 | 0.999994883 |
| myelin maintenance (GO:0043217) | 44602 | 0.321692826 | 0.999994883 |
| negative regulation of cell adhesion mediate( | 44602 | 0.321692826 | 0.999994883 |

| | | | |
|---|---|---|---|
| negative regulation of cholesterol storage (G( | 44602 | 0.321692826 | 0.999994883 |
| negative regulation of extracellular matrix or | 44602 | 0.321692826 | 0.999994883 |
| negative regulation of heterotypic cell-cell ac | 44602 | 0.321692826 | 0.999994883 |
| negative regulation of leukocyte migration (G | 44602 | 0.321692826 | 0.999994883 |
| negative regulation of phosphatidylinositol 3- | 44602 | 0.321692826 | 0.999994883 |
| negative regulation of telomerase activity (G( | 44602 | 0.321692826 | 0.999994883 |
| peptidyl-amino acid modification (GO:00181! | 44602 | 0.321692826 | 0.999994883 |
| positive regulation of epidermis developmen | 44602 | 0.321692826 | 0.999994883 |
| protein O-linked glycosylation via serine (GO: | 44602 | 0.321692826 | 0.999994883 |
| protein poly-ADP-ribosylation (GO:0070212) | 44602 | 0.321692826 | 0.999994883 |
| Rap protein signal transduction (GO:0032486 | 44602 | 0.321692826 | 0.999994883 |
| regulation of cell activation (GO:0050865) | 44602 | 0.321692826 | 0.999994883 |
| regulation of cell-cell adhesion involved in ga | 44602 | 0.321692826 | 0.999994883 |
| regulation of cilium movement (GO:0003352 | 44602 | 0.321692826 | 0.999994883 |
| regulation of epithelial cell apoptotic process | 44602 | 0.321692826 | 0.999994883 |
| regulation of histone phosphorylation (GO:0( | 44602 | 0.321692826 | 0.999994883 |
| regulation of hydrogen peroxide-induced cell | 44602 | 0.321692826 | 0.999994883 |
| regulation of organ growth (GO:0046620) | 44602 | 0.321692826 | 0.999994883 |
| regulation of peptidyl-lysine acetylation (GO: | 44602 | 0.321692826 | 0.999994883 |
| regulation of phagocytosis, engulfment (GO:( | 44602 | 0.321692826 | 0.999994883 |
| regulation of type B pancreatic cell developm | 44602 | 0.321692826 | 0.999994883 |
| renal filtration (GO:0097205) | 44602 | 0.321692826 | 0.999994883 |
| SNARE complex assembly (GO:0035493) | 44602 | 0.321692826 | 0.999994883 |
| cellular polysaccharide biosynthetic process ( | 44676 | 0.321812841 | 0.999994883 |
| energy derivation by oxidation of organic cor | 44676 | 0.321812841 | 0.999994883 |
| fat-soluble vitamin metabolic process (GO:00 | 44676 | 0.321812841 | 0.999994883 |
| glycolytic process through glucose-6-phospha | 44676 | 0.321812841 | 0.999994883 |
| in utero embryonic development (GO:00170 | 44676 | 0.321812841 | 0.999994883 |
| negative regulation of endocytosis (GO:0045 | 44676 | 0.321812841 | 0.999994883 |
| negative regulation of response to endoplasr | 44676 | 0.321812841 | 0.999994883 |
| negative regulation of sequestering of calciur | 44676 | 0.321812841 | 0.999994883 |
| phosphatidylglycerol metabolic process (GO: | 44676 | 0.321812841 | 0.999994883 |
| positive regulation of ERBB signaling pathway | 44676 | 0.321812841 | 0.999994883 |
| mitochondrial translational elongation (GO:0 | 32843 | 0.322620228 | 0.999994883 |
| post-Golgi vesicle-mediated transport (GO:0( | 32843 | 0.322620228 | 0.999994883 |
| regulation of hydrolase activity (GO:0051336 | 26938 | 0.326678635 | 0.999994883 |
| neuron projection extension (GO:1990138) | 12175 | 0.327345971 | 0.999994883 |
| positive regulation of telomere maintenance | 12175 | 0.327345971 | 0.999994883 |
| regulation of protein localization to cell surfa | 12175 | 0.327345971 | 0.999994883 |
| positive regulation of kinase activity (GO:003 | 15/114 | 0.328073392 | 0.999994883 |
| mitotic G1 DNA damage checkpoint signaling | 23986 | 0.328323605 | 0.999994883 |
| negative regulation of DNA recombination (G | 21033 | 0.329539519 | 0.999994883 |
| regulation of reactive oxygen species metabc | 21033 | 0.329539519 | 0.999994883 |
| regulation of response to DNA damage stimu | 15128 | 0.329607385 | 0.999994883 |
| mitotic cytokinesis (GO:0000281) | 18080 | 0.330097643 | 0.999994883 |
| regulation of stress-activated MAPK cascade | 18080 | 0.330097643 | 0.999994883 |
| regulation of autophagy (GO:0010506) | 29/231 | 0.333474195 | 0.999994883 |

| | | | |
|---|---|---|---|
| negative regulation of neuron death (GO:190 | 13/98 | 0.333913963 | 0.999994883 |
| regulation of neuron apoptotic process (GO:( | 13/98 | 0.333913963 | 0.999994883 |
| negative regulation of protein kinase activity | 33208 | 0.336818446 | 0.999994883 |
| positive regulation of transferase activity (GC | 19/148 | 0.338597248 | 0.999994883 |
| positive regulation of cytoskeleton organizati | 30256 | 0.339680802 | 0.999994883 |
| regulation of neuron projection development | 21/165 | 0.342276015 | 0.999994883 |
| adenine nucleotide transport (GO:0051503) | 44638 | 0.342346224 | 0.999994883 |
| embryonic heart tube development (GO:003! | 44638 | 0.342346224 | 0.999994883 |
| intracellular estrogen receptor signaling path | 44638 | 0.342346224 | 0.999994883 |
| negative regulation of biosynthetic process (( | 44638 | 0.342346224 | 0.999994883 |
| negative regulation of cation transmembrane | 44638 | 0.342346224 | 0.999994883 |
| negative regulation of leukocyte cell-cell adh | 44638 | 0.342346224 | 0.999994883 |
| negative regulation of signal transduction by | 44638 | 0.342346224 | 0.999994883 |
| positive regulation of CREB transcription fact | 44638 | 0.342346224 | 0.999994883 |
| positive regulation of extracellular matrix org | 44638 | 0.342346224 | 0.999994883 |
| positive regulation of interleukin-4 productio | 44638 | 0.342346224 | 0.999994883 |
| regulation of adaptive immune response (GO | 44638 | 0.342346224 | 0.999994883 |
| regulation of fatty acid metabolic process (G( | 44638 | 0.342346224 | 0.999994883 |
| regulation of mitochondrial outer membrane | 44638 | 0.342346224 | 0.999994883 |
| regulation of multicellular organism growth ( | 44638 | 0.342346224 | 0.999994883 |
| release of cytochrome c from mitochondria ( | 44638 | 0.342346224 | 0.999994883 |
| triglyceride biosynthetic process (GO:001943 | 44638 | 0.342346224 | 0.999994883 |
| DNA damage response, signal transduction b | 27303 | 0.342462558 | 0.999994883 |
| proteolysis involved in cellular protein catabo | 27303 | 0.342462558 | 0.999994883 |
| cellular response to nutrient levels (GO:0031 | 24351 | 0.345104875 | 0.999994883 |
| negative regulation of neuron projection dev | 21398 | 0.347514897 | 0.999994883 |
| receptor metabolic process (GO:0043112) | 21398 | 0.347514897 | 0.999994883 |
| endomembrane system organization (GO:00: | 25/199 | 0.347647062 | 0.999994883 |
| lamellipodium assembly (GO:0030032) | 44677 | 0.349157871 | 0.999994883 |
| negative regulation of intrinsic apoptotic sign | 44677 | 0.349157871 | 0.999994883 |
| organelle fission (GO:0048285) | 44677 | 0.349157871 | 0.999994883 |
| positive regulation of protein insertion into n | 44677 | 0.349157871 | 0.999994883 |
| proteoglycan metabolic process (GO:000602! | 44677 | 0.349157871 | 0.999994883 |
| regulation of monocyte chemotaxis (GO:009( | 44677 | 0.349157871 | 0.999994883 |
| regulation of myelination (GO:0031641) | 44677 | 0.349157871 | 0.999994883 |
| regulation of protein insertion into mitochon | 44677 | 0.349157871 | 0.999994883 |
| regulation of tumor necrosis factor productic | 16/124 | 0.349499471 | 0.999994883 |
| neuron migration (GO:0001764) | 18445 | 0.349539838 | 0.999994883 |
| phosphatidylinositol phosphate biosynthetic | 15493 | 0.350913806 | 0.999994883 |
| positive regulation of protein tyrosine kinase | 15493 | 0.350913806 | 0.999994883 |
| regulation of ubiquitin-dependent protein ca | 15493 | 0.350913806 | 0.999994883 |
| skeletal system morphogenesis (GO:0048705 | 15493 | 0.350913806 | 0.999994883 |
| cellular response to amino acid stimulus (GO | 12540 | 0.351136856 | 0.999994883 |
| negative regulation of blood vessel endotheli | 12540 | 0.351136856 | 0.999994883 |
| positive regulation of telomerase activity (GC | 12540 | 0.351136856 | 0.999994883 |
| regulation of glucose metabolic process (GO: | 12540 | 0.351136856 | 0.999994883 |
| positive regulation of transport (GO:0051050 | 33573 | 0.351137263 | 0.999994883 |

| | | | |
|---|---|---|---|
| Ras protein signal transduction (GO:0007265 | 18/142 | 0.36519424 | 0.999994883 |
| response to reactive oxygen species (GO:000 | 21763 | 0.365611025 | 0.999994883 |
| actin filament network formation (GO:00516 | 44603 | 0.365701215 | 0.999994883 |
| axonal transport of mitochondrion (GO:0019 | 44603 | 0.365701215 | 0.999994883 |
| camera-type eye morphogenesis (GO:004859 | 44603 | 0.365701215 | 0.999994883 |
| carnitine shuttle (GO:0006853) | 44603 | 0.365701215 | 0.999994883 |
| cellular response to cholesterol (GO:0071397 | 44603 | 0.365701215 | 0.999994883 |
| coenzyme A biosynthetic process (GO:00159: | 44603 | 0.365701215 | 0.999994883 |
| copper ion transport (GO:0006825) | 44603 | 0.365701215 | 0.999994883 |
| detection of light stimulus involved in sensor | 44603 | 0.365701215 | 0.999994883 |
| detection of light stimulus involved in visual p | 44603 | 0.365701215 | 0.999994883 |
| DNA damage response, signal transduction re | 44603 | 0.365701215 | 0.999994883 |
| embryonic eye morphogenesis (GO:0048048 | 44603 | 0.365701215 | 0.999994883 |
| glycerol metabolic process (GO:0006071) | 44603 | 0.365701215 | 0.999994883 |
| inositol phosphate biosynthetic process (GO: | 44603 | 0.365701215 | 0.999994883 |
| mitochondrial transcription (GO:0006390) | 44603 | 0.365701215 | 0.999994883 |
| negative regulation of nuclear division (GO:0 | 44603 | 0.365701215 | 0.999994883 |
| negative regulation of protein maturation (G | 44603 | 0.365701215 | 0.999994883 |
| negative regulation of response to cytokine s | 44603 | 0.365701215 | 0.999994883 |
| peptidyl-lysine monomethylation (GO:00180: | 44603 | 0.365701215 | 0.999994883 |
| positive regulation by host of viral process (G | 44603 | 0.365701215 | 0.999994883 |
| positive regulation of DNA-dependent DNA re | 44603 | 0.365701215 | 0.999994883 |
| positive regulation of interleukin-13 producti | 44603 | 0.365701215 | 0.999994883 |
| positive regulation of myelination (GO:00316 | 44603 | 0.365701215 | 0.999994883 |
| positive regulation of vacuole organization (G | 44603 | 0.365701215 | 0.999994883 |
| positive regulation of viral release from host | 44603 | 0.365701215 | 0.999994883 |
| positive regulation of voltage-gated potassiu | 44603 | 0.365701215 | 0.999994883 |
| proteasome assembly (GO:0043248) | 44603 | 0.365701215 | 0.999994883 |
| protein oxidation (GO:0018158) | 44603 | 0.365701215 | 0.999994883 |
| purine nucleoside bisphosphate biosynthetic | 44603 | 0.365701215 | 0.999994883 |
| pyrimidine nucleoside salvage (GO:0043097) | 44603 | 0.365701215 | 0.999994883 |
| pyrimidine-containing compound salvage (GO | 44603 | 0.365701215 | 0.999994883 |
| rDNA heterochromatin assembly (GO:000018 | 44603 | 0.365701215 | 0.999994883 |
| regulation of amyloid precursor protein biosy | 44603 | 0.365701215 | 0.999994883 |
| regulation of cysteine-type endopeptidase ac | 44603 | 0.365701215 | 0.999994883 |
| regulation of dendritic cell apoptotic process | 44603 | 0.365701215 | 0.999994883 |
| regulation of microtubule-based movement ( | 44603 | 0.365701215 | 0.999994883 |
| regulation of monocyte chemotactic protein- | 44603 | 0.365701215 | 0.999994883 |
| regulation of toll-like receptor 9 signaling pat | 44603 | 0.365701215 | 0.999994883 |
| response to hydroxyurea (GO:0072710) | 44603 | 0.365701215 | 0.999994883 |
| ribonucleoside bisphosphate biosynthetic pro | 44603 | 0.365701215 | 0.999994883 |
| somite development (GO:0061053) | 44603 | 0.365701215 | 0.999994883 |
| sphingomyelin metabolic process (GO:00066: | 44603 | 0.365701215 | 0.999994883 |
| tetrahydrobiopterin metabolic process (GO:0 | 44603 | 0.365701215 | 0.999994883 |
| tetrahydrofolate metabolic process (GO:004€ | 44603 | 0.365701215 | 0.999994883 |
| tryptophan catabolic process (GO:0006569) | 44603 | 0.365701215 | 0.999994883 |
| urate metabolic process (GO:0046415) | 44603 | 0.365701215 | 0.999994883 |

| | | | |
|---|---|---|---|
| V(D)J recombination (GO:0033151) | 44603 | 0.365701215 | 0.999994883 |
| positive regulation of protein phosphorylatio | 45/371 | 0.371072844 | 0.999994883 |
| heart looping (GO:0001947) | 15858 | 0.372323175 | 0.999994883 |
| positive regulation of neuron differentiation | 15858 | 0.372323175 | 0.999994883 |
| regulation of carbohydrate catabolic process | 15858 | 0.372323175 | 0.999994883 |
| protein import into nucleus (GO:0006606) | 28034 | 0.374370827 | 0.999994883 |
| regulation of T cell proliferation (GO:0042129 | 28034 | 0.374370827 | 0.999994883 |
| cellular component assembly (GO:0022607) | 32/261 | 0.374488602 | 0.999994883 |
| cellular response to amyloid-beta (GO:19046 | 12905 | 0.375004636 | 0.999994883 |
| cellular response to hydrogen peroxide (GO:( | 12905 | 0.375004636 | 0.999994883 |
| negative regulation of T cell proliferation (GC | 12905 | 0.375004636 | 0.999994883 |
| positive regulation of reproductive process (( | 12905 | 0.375004636 | 0.999994883 |
| regulation of autophagosome assembly (GO:1 | 12905 | 0.375004636 | 0.999994883 |
| actin filament capping (GO:0051693) | 44639 | 0.375273517 | 0.999994883 |
| barbed-end actin filament capping (GO:0051( | 44639 | 0.375273517 | 0.999994883 |
| detection of mechanical stimulus (GO:00509& | 44639 | 0.375273517 | 0.999994883 |
| leukotriene biosynthetic process (GO:001937 | 44639 | 0.375273517 | 0.999994883 |
| negative regulation of amyloid precursor pro | 44639 | 0.375273517 | 0.999994883 |
| neuromuscular process (GO:0050905) | 44639 | 0.375273517 | 0.999994883 |
| positive regulation of cellular protein catabol | 44639 | 0.375273517 | 0.999994883 |
| positive regulation of monocyte chemotaxis ( | 44639 | 0.375273517 | 0.999994883 |
| purine ribonucleoside bisphosphate metabol | 44639 | 0.375273517 | 0.999994883 |
| Rac protein signal transduction (GO:0016601 | 44639 | 0.375273517 | 0.999994883 |
| regulation of autophagy of mitochondrion (G | 44639 | 0.375273517 | 0.999994883 |
| regulation of histone H3-K4 methylation (GO | 44639 | 0.375273517 | 0.999994883 |
| regulation of insulin-like growth factor recep | 44639 | 0.375273517 | 0.999994883 |
| regulation of lymphocyte proliferation (GO:0 | 44639 | 0.375273517 | 0.999994883 |
| regulation of mitochondrial membrane perm | 44639 | 0.375273517 | 0.999994883 |
| response to cholesterol (GO:0070723) | 44639 | 0.375273517 | 0.999994883 |
| vesicle transport along actin filament (GO:00 | 44639 | 0.375273517 | 0.999994883 |
| L-alpha-amino acid transmembrane transpor | 44678 | 0.376520997 | 0.999994883 |
| mitotic chromosome condensation (GO:0007 | 44678 | 0.376520997 | 0.999994883 |
| monovalent inorganic cation homeostasis (G | 44678 | 0.376520997 | 0.999994883 |
| positive regulation of nitric oxide biosyntheti | 44678 | 0.376520997 | 0.999994883 |
| release of sequestered calcium ion into cytos | 44678 | 0.376520997 | 0.999994883 |
| nitrogen compound transport (GO:0071705) | 18/143 | 0.3768264 | 0.999994883 |
| receptor-mediated endocytosis (GO:0006898 | 18/143 | 0.3768264 | 0.999994883 |
| regulation of cellular response to stress (GO:( | 15/118 | 0.378941549 | 0.999994883 |
| response to organic cyclic compound (GO:00 | 22129 | 0.383786313 | 0.999994883 |
| amide biosynthetic process (GO:0043604) | 19176 | 0.388717843 | 0.999994883 |
| plasma membrane bounded cell projection n | 19176 | 0.388717843 | 0.999994883 |
| positive regulation of developmental growth | 19176 | 0.388717843 | 0.999994883 |
| regulation of telomere maintenance via telor | 19176 | 0.388717843 | 0.999994883 |
| positive regulation of cell growth (GO:00303( | 13/102 | 0.389126932 | 0.999994883 |
| cholesterol metabolic process (GO:0008203) | 28399 | 0.390438113 | 0.999994883 |
| negative regulation of developmental proces | 28399 | 0.390438113 | 0.999994883 |
| positive regulation of osteoblast differentiati | 16224 | 0.393768454 | 0.999994883 |

| | | | |
|---|---|---|---|
| negative regulation of cell death (GO:006054 | 34669 | 0.394606387 | 0.999994883 |
| positive regulation of epithelial cell migratior | 34669 | 0.394606387 | 0.999994883 |
| regulation of kinase activity (GO:0043549) | 34669 | 0.394606387 | 0.999994883 |
| regulation of protein serine/threonine kinase | 14/111 | 0.397316193 | 0.999994883 |
| regulation of Wnt signaling pathway (GO:003 | 14/111 | 0.397316193 | 0.999994883 |
| regulation of embryonic development (GO:0( | 13271 | 0.398858534 | 0.999994883 |
| retina homeostasis (GO:0001895) | 13271 | 0.398858534 | 0.999994883 |
| negative regulation of phosphorylation (GO:( | 31717 | 0.400394769 | 0.999994883 |
| positive regulation of proteasomal protein ca | 31717 | 0.400394769 | 0.999994883 |
| protein stabilization (GO:0050821) | 22/179 | 0.401320679 | 0.999994883 |
| protein lipidation (GO:0006497) | 22494 | 0.402000126 | 0.999994883 |
| regulation of DNA-templated transcription, ir | 22494 | 0.402000126 | 0.999994883 |
| regulation of endocytosis (GO:0030100) | 22494 | 0.402000126 | 0.999994883 |
| cellular response to organonitrogen compour | 13/103 | 0.403088809 | 0.999994883 |
| bile acid and bile salt transport (GO:0015721 | 44679 | 0.403770412 | 0.999994883 |
| cristae formation (GO:0042407) | 44679 | 0.403770412 | 0.999994883 |
| cytosolic calcium ion transport (GO:0060401) | 44679 | 0.403770412 | 0.999994883 |
| glycerophospholipid catabolic process (GO:0( | 44679 | 0.403770412 | 0.999994883 |
| negative regulation of calcium ion transmem | 44679 | 0.403770412 | 0.999994883 |
| photoperiodism (GO:0009648) | 44679 | 0.403770412 | 0.999994883 |
| positive regulation of microtubule polymeriz | 44679 | 0.403770412 | 0.999994883 |
| positive regulation of nitric oxide metabolic p | 44679 | 0.403770412 | 0.999994883 |
| protein localization to Golgi apparatus (GO:0 | 44679 | 0.403770412 | 0.999994883 |
| regulation of DNA damage response, signal tı | 44679 | 0.403770412 | 0.999994883 |
| regulation of immunoglobulin production (G( | 44679 | 0.403770412 | 0.999994883 |
| regulation of postsynapse organization (GO:( | 44679 | 0.403770412 | 0.999994883 |
| regulation of reactive oxygen species biosynt | 44679 | 0.403770412 | 0.999994883 |
| RNA phosphodiester bond hydrolysis (GO:00! | 44679 | 0.403770412 | 0.999994883 |
| cellular response to nitrogen compound (GO: | 44640 | 0.40784951 | 0.999994883 |
| inositol phosphate-mediated signaling (GO:0( | 44640 | 0.40784951 | 0.999994883 |
| insulin secretion (GO:0030073) | 44640 | 0.40784951 | 0.999994883 |
| negative regulation of osteoclast differentiat | 44640 | 0.40784951 | 0.999994883 |
| negative regulation of telomere maintenance | 44640 | 0.40784951 | 0.999994883 |
| nucleobase-containing compound biosynthet | 44640 | 0.40784951 | 0.999994883 |
| positive regulation of dendrite morphogenes | 44640 | 0.40784951 | 0.999994883 |
| positive regulation of epithelial cell differenti | 44640 | 0.40784951 | 0.999994883 |
| positive regulation of natural killer cell media | 44640 | 0.40784951 | 0.999994883 |
| positive regulation of nitrogen compound me | 44640 | 0.40784951 | 0.999994883 |
| protein targeting to lysosome (GO:0006622) | 44640 | 0.40784951 | 0.999994883 |
| regulation of fibroblast growth factor recept | 44640 | 0.40784951 | 0.999994883 |
| positive regulation of B cell activation (GO:00 | 19541 | 0.408351479 | 0.999994883 |
| actin crosslink formation (GO:0051764) | 44604 | 0.408552428 | 0.999994883 |
| axonal fasciculation (GO:0007413) | 44604 | 0.408552428 | 0.999994883 |
| C-terminal protein amino acid modification (( | 44604 | 0.408552428 | 0.999994883 |
| calcium ion-regulated exocytosis of neurotra | 44604 | 0.408552428 | 0.999994883 |
| cell cycle checkpoint signaling (GO:0000075) | 44604 | 0.408552428 | 0.999994883 |
| cellular response to fluid shear stress (GO:00 | 44604 | 0.408552428 | 0.999994883 |

| | | | |
|---|---|---|---|
| cellular response to thyroid hormone stimulu | 44604 | 0.408552428 | 0.999994883 |
| cGMP metabolic process (GO:0046068) | 44604 | 0.408552428 | 0.999994883 |
| depurination (GO:0045007) | 44604 | 0.408552428 | 0.999994883 |
| eye photoreceptor cell differentiation (GO:00 | 44604 | 0.408552428 | 0.999994883 |
| ganglion development (GO:0061548) | 44604 | 0.408552428 | 0.999994883 |
| Golgi localization (GO:0051645) | 44604 | 0.408552428 | 0.999994883 |
| hemidesmosome assembly (GO:0031581) | 44604 | 0.408552428 | 0.999994883 |
| homocysteine metabolic process (GO:005066 | 44604 | 0.408552428 | 0.999994883 |
| macrophage chemotaxis (GO:0048246) | 44604 | 0.408552428 | 0.999994883 |
| magnesium ion transport (GO:0015693) | 44604 | 0.408552428 | 0.999994883 |
| megakaryocyte differentiation (GO:0030219) | 44604 | 0.408552428 | 0.999994883 |
| microtubule anchoring at microtubule organi | 44604 | 0.408552428 | 0.999994883 |
| mitotic G1/S transition checkpoint signaling ( | 44604 | 0.408552428 | 0.999994883 |
| mitotic nuclear membrane disassembly (GO:( | 44604 | 0.408552428 | 0.999994883 |
| morphogenesis of a polarized epithelium (GC | 44604 | 0.408552428 | 0.999994883 |
| negative regulation of amyloid fibril formatio | 44604 | 0.408552428 | 0.999994883 |
| negative regulation of calcineurin-mediated s | 44604 | 0.408552428 | 0.999994883 |
| negative regulation of calcineurin-NFAT signa | 44604 | 0.408552428 | 0.999994883 |
| negative regulation of cell division (GO:0051 | 44604 | 0.408552428 | 0.999994883 |
| negative regulation of gene silencing by miRN | 44604 | 0.408552428 | 0.999994883 |
| negative regulation of oxidoreductase activit | 44604 | 0.408552428 | 0.999994883 |
| neurotransmitter uptake (GO:0001504) | 44604 | 0.408552428 | 0.999994883 |
| nucleoside salvage (GO:0043174) | 44604 | 0.408552428 | 0.999994883 |
| P-body assembly (GO:0033962) | 44604 | 0.408552428 | 0.999994883 |
| phosphate ion transmembrane transport (GC | 44604 | 0.408552428 | 0.999994883 |
| poly-N-acetyllactosamine biosynthetic proces | 44604 | 0.408552428 | 0.999994883 |
| positive regulation of fatty acid oxidation (GC | 44604 | 0.408552428 | 0.999994883 |
| positive regulation of myeloid leukocyte med | 44604 | 0.408552428 | 0.999994883 |
| positive regulation of neuroinflammatory res | 44604 | 0.408552428 | 0.999994883 |
| positive regulation of protein maturation (GC | 44604 | 0.408552428 | 0.999994883 |
| positive regulation of ubiquitin protein ligase | 44604 | 0.408552428 | 0.999994883 |
| protein auto-ADP-ribosylation (GO:0070213) | 44604 | 0.408552428 | 0.999994883 |
| protein localization to synapse (GO:0035418) | 44604 | 0.408552428 | 0.999994883 |
| pyrimidine nucleobase metabolic process (GC | 44604 | 0.408552428 | 0.999994883 |
| regulation of CD4-positive, alpha-beta T cell c | 44604 | 0.408552428 | 0.999994883 |
| regulation of cholesterol esterification (GO:0 | 44604 | 0.408552428 | 0.999994883 |
| regulation of flagellated sperm motility (GO:1 | 44604 | 0.408552428 | 0.999994883 |
| regulation of histone H3-K9 methylation (GO | 44604 | 0.408552428 | 0.999994883 |
| regulation of leukocyte cell-cell adhesion (GC | 44604 | 0.408552428 | 0.999994883 |
| regulation of nucleobase-containing compou | 44604 | 0.408552428 | 0.999994883 |
| regulation of response to oxidative stress (GC | 44604 | 0.408552428 | 0.999994883 |
| regulation of sensory perception (GO:005193 | 44604 | 0.408552428 | 0.999994883 |
| regulation of vascular associated smooth mu | 44604 | 0.408552428 | 0.999994883 |
| sensory perception of taste (GO:0050909) | 44604 | 0.408552428 | 0.999994883 |
| serine family amino acid biosynthetic process | 44604 | 0.408552428 | 0.999994883 |
| smooth muscle cell differentiation (GO:0051 | 44604 | 0.408552428 | 0.999994883 |
| thyroid hormone generation (GO:0006590) | 44604 | 0.408552428 | 0.999994883 |

| | | | |
|---|---|---|---|
| tricarboxylic acid metabolic process (GO:007: | 44604 | 0.408552428 | 0.999994883 |
| tyrosine metabolic process (GO:0006570) | 44604 | 0.408552428 | 0.999994883 |
| apoptotic process (GO:0006915) | 28/231 | 0.4105335 | 0.999994883 |
| positive regulation of GTPase activity (GO:00 | 26/214 | 0.411102326 | 0.999994883 |
| cellular response to insulin stimulus (GO:003: | 16/129 | 0.411611081 | 0.999994883 |
| COPII-coated vesicle budding (GO:0090114) | 25812 | 0.413119466 | 0.999994883 |
| chromosome condensation (GO:0030261) | 16589 | 0.415185517 | 0.999994883 |
| negative regulation of T cell activation (GO:0 | 16589 | 0.415185517 | 0.999994883 |
| protein deacetylation (GO:0006476) | 16589 | 0.415185517 | 0.999994883 |
| steroid metabolic process (GO:0008202) | 13/104 | 0.417077767 | 0.999994883 |
| anterograde axonal transport (GO:0008089) | 13636 | 0.42261326 | 0.999994883 |
| neural crest cell migration (GO:0001755) | 13636 | 0.42261326 | 0.999994883 |
| protein insertion into ER membrane (GO:004 | 13636 | 0.42261326 | 0.999994883 |
| regulation of transport (GO:0051049) | 13636 | 0.42261326 | 0.999994883 |
| regulation of cyclin-dependent protein kinase | 19906 | 0.427949765 | 0.999994883 |
| regulation of megakaryocyte differentiation ( | 19906 | 0.427949765 | 0.999994883 |
| striated muscle contraction (GO:0006941) | 19906 | 0.427949765 | 0.999994883 |
| negative regulation of neuron apoptotic proc | 26177 | 0.430179414 | 0.999994883 |
| regulation of G1/S transition of mitotic cell cy | 26177 | 0.430179414 | 0.999994883 |
| establishment of mitotic spindle localization | 44680 | 0.430785809 | 0.999994883 |
| homologous chromosome pairing at meiosis | 44680 | 0.430785809 | 0.999994883 |
| lamellipodium organization (GO:0097581) | 44680 | 0.430785809 | 0.999994883 |
| limb development (GO:0060173) | 44680 | 0.430785809 | 0.999994883 |
| lipid catabolic process (GO:0016042) | 44680 | 0.430785809 | 0.999994883 |
| negative regulation of interferon-gamma pro | 44680 | 0.430785809 | 0.999994883 |
| negative regulation of transferase activity (G | 44680 | 0.430785809 | 0.999994883 |
| positive regulation of viral genome replicatio | 44680 | 0.430785809 | 0.999994883 |
| regulation of double-strand break repair via r | 44680 | 0.430785809 | 0.999994883 |
| regulation of fatty acid biosynthetic process ( | 44680 | 0.430785809 | 0.999994883 |
| regulation of response to biotic stimulus (GO | 44680 | 0.430785809 | 0.999994883 |
| metal ion transport (GO:0030001) | 32448 | 0.431001811 | 0.999994883 |
| negative regulation of tumor necrosis factor | 16954 | 0.436513498 | 0.999994883 |
| positive regulation of nucleocytoplasmic tran | 16954 | 0.436513498 | 0.999994883 |
| regulation of cellular protein localization (GO | 16954 | 0.436513498 | 0.999994883 |
| regulation of telomerase activity (GO:005197 | 16954 | 0.436513498 | 0.999994883 |
| positive regulation of cellular component org | 14/114 | 0.437665214 | 0.999994883 |
| actin cytoskeleton reorganization (GO:00315 | 23224 | 0.438386794 | 0.999994883 |
| glycosaminoglycan biosynthetic process (GO: | 35765 | 0.43840844 | 0.999994883 |
| regulation of fat cell differentiation (GO:004! | 29495 | 0.438759483 | 0.999994883 |
| actin filament-based transport (GO:0099515) | 44641 | 0.439889272 | 0.999994883 |
| aerobic respiration (GO:0009060) | 44641 | 0.439889272 | 0.999994883 |
| cellular response to inorganic substance (GO: | 44641 | 0.439889272 | 0.999994883 |
| cobalamin metabolic process (GO:0009235) | 44641 | 0.439889272 | 0.999994883 |
| linoleic acid metabolic process (GO:0043651) | 44641 | 0.439889272 | 0.999994883 |
| mitochondrial calcium ion transmembrane tr | 44641 | 0.439889272 | 0.999994883 |
| NAD biosynthetic process (GO:0009435) | 44641 | 0.439889272 | 0.999994883 |
| negative regulation of protein localization to | 44641 | 0.439889272 | 0.999994883 |

| | | | |
|---|---|---|---|
| positive regulation of T cell cytokine producti | 44641 | 0.439889272 | 0.999994883 |
| positive regulation of transcription regulatory | 44641 | 0.439889272 | 0.999994883 |
| protein localization to chromatin (GO:007116 | 44641 | 0.439889272 | 0.999994883 |
| purinergic nucleotide receptor signaling path | 44641 | 0.439889272 | 0.999994883 |
| regulation of cardiac muscle contraction by c | 44641 | 0.439889272 | 0.999994883 |
| regulation of cell-substrate junction assembly | 44641 | 0.439889272 | 0.999994883 |
| regulation of lipid catabolic process (GO:005( | 44641 | 0.439889272 | 0.999994883 |
| regulation of lipopolysaccharide-mediated sig | 44641 | 0.439889272 | 0.999994883 |
| regulation of lipoprotein lipase activity (GO:0 | 44641 | 0.439889272 | 0.999994883 |
| regulation of mitochondrial translation (GO:C | 44641 | 0.439889272 | 0.999994883 |
| regulation of transcription by RNA polymeras | 44641 | 0.439889272 | 0.999994883 |
| zinc ion transport (GO:0006829) | 44641 | 0.439889272 | 0.999994883 |
| positive regulation of protein serine/threonir | 13/106 | 0.4450661 | 0.999994883 |
| actin-myosin filament sliding (GO:0033275) | 14001 | 0.446189423 | 0.999994883 |
| muscle filament sliding (GO:0030049) | 14001 | 0.446189423 | 0.999994883 |
| negative regulation of endothelial cell migrat | 14001 | 0.446189423 | 0.999994883 |
| positive regulation of production of molecula | 14001 | 0.446189423 | 0.999994883 |
| regulation of apoptotic signaling pathway (G( | 14001 | 0.446189423 | 0.999994883 |
| protein import (GO:0017038) | 32813 | 0.446286388 | 0.999994883 |
| cytoskeleton-dependent cytokinesis (GO:006 | 26543 | 0.447198382 | 0.999994883 |
| extrinsic apoptotic signaling pathway (GO:00 | 26543 | 0.447198382 | 0.999994883 |
| regulation of blood vessel endothelial cell mi | 20271 | 0.447466498 | 0.999994883 |
| chondroitin sulfate catabolic process (GO:003 | 44605 | 0.449931791 | 0.999994883 |
| dermatan sulfate metabolic process (GO:003 | 44605 | 0.449931791 | 0.999994883 |
| detection of calcium ion (GO:0005513) | 44605 | 0.449931791 | 0.999994883 |
| embryonic appendage morphogenesis (GO:0 | 44605 | 0.449931791 | 0.999994883 |
| establishment of protein localization to chror | 44605 | 0.449931791 | 0.999994883 |
| Golgi ribbon formation (GO:0090161) | 44605 | 0.449931791 | 0.999994883 |
| lipid oxidation (GO:0034440) | 44605 | 0.449931791 | 0.999994883 |
| mitochondrion transport along microtubule ( | 44605 | 0.449931791 | 0.999994883 |
| negative regulation of adenylate cyclase activ | 44605 | 0.449931791 | 0.999994883 |
| negative regulation of alcohol biosynthetic pr | 44605 | 0.449931791 | 0.999994883 |
| negative regulation of CD4-positive, alpha-be | 44605 | 0.449931791 | 0.999994883 |
| negative regulation of cell morphogenesis inv | 44605 | 0.449931791 | 0.999994883 |
| negative regulation of chromatin organizatior | 44605 | 0.449931791 | 0.999994883 |
| negative regulation of chromatin silencing (G | 44605 | 0.449931791 | 0.999994883 |
| negative regulation of gene silencing (GO:00( | 44605 | 0.449931791 | 0.999994883 |
| negative regulation of histone methylation (( | 44605 | 0.449931791 | 0.999994883 |
| negative regulation of intrinsic apoptotic sigr | 44605 | 0.449931791 | 0.999994883 |
| negative regulation of mononuclear cell migr | 44605 | 0.449931791 | 0.999994883 |
| negative regulation of substrate adhesion-de | 44605 | 0.449931791 | 0.999994883 |
| pentose-phosphate shunt (GO:0006098) | 44605 | 0.449931791 | 0.999994883 |
| poly-N-acetyllactosamine metabolic process | 44605 | 0.449931791 | 0.999994883 |
| polyamine metabolic process (GO:0006595) | 44605 | 0.449931791 | 0.999994883 |
| positive regulation of calcium ion-dependent | 44605 | 0.449931791 | 0.999994883 |
| positive regulation of calcium-mediated signa | 44605 | 0.449931791 | 0.999994883 |
| positive regulation of fatty acid biosynthetic | 44605 | 0.449931791 | 0.999994883 |

| | | | |
|---|---|---|---|
| positive regulation of insulin receptor signali | 44605 | 0.449931791 | 0.999994883 |
| positive regulation of lipid transport (GO:003 | 44605 | 0.449931791 | 0.999994883 |
| positive regulation of protein localization to | 44605 | 0.449931791 | 0.999994883 |
| positive regulation of reactive oxygen specie | 44605 | 0.449931791 | 0.999994883 |
| positive regulation of sodium ion transport ( | 44605 | 0.449931791 | 0.999994883 |
| positive regulation of steroid metabolic proc | 44605 | 0.449931791 | 0.999994883 |
| pteridine-containing compound metabolic pr | 44605 | 0.449931791 | 0.999994883 |
| regulation of chemokine (C-X-C motif) ligand | 44605 | 0.449931791 | 0.999994883 |
| regulation of histone modification (GO:0031 | 44605 | 0.449931791 | 0.999994883 |
| regulation of lysosomal lumen pH (GO:00357 | 44605 | 0.449931791 | 0.999994883 |
| regulation of short-term neuronal synaptic pl | 44605 | 0.449931791 | 0.999994883 |
| regulation of T cell differentiation in thymus | 44605 | 0.449931791 | 0.999994883 |
| regulation of tube diameter (GO:0035296) | 44605 | 0.449931791 | 0.999994883 |
| response to iron ion (GO:0010039) | 44605 | 0.449931791 | 0.999994883 |
| response to thyroid hormone (GO:0097066) | 44605 | 0.449931791 | 0.999994883 |
| semaphorin-plexin signaling pathway involve | 44605 | 0.449931791 | 0.999994883 |
| spinal cord development (GO:0021510) | 44605 | 0.449931791 | 0.999994883 |
| transport along microtubule (GO:0010970) | 44605 | 0.449931791 | 0.999994883 |
| tRNA 5'-leader removal (GO:0001682) | 44605 | 0.449931791 | 0.999994883 |
| vascular process in circulatory system (GO:00 | 44605 | 0.449931791 | 0.999994883 |
| cellular response to molecule of bacterial ori | 14/115 | 0.451112169 | 0.999994883 |
| regulation of small GTPase mediated signal tr | 17/141 | 0.454377405 | 0.999994883 |
| positive regulation of phosphorylation (GO:0 | 30/253 | 0.455184828 | 0.999994883 |
| regulation of cellular ketone metabolic proce | 23590 | 0.456484936 | 0.999994883 |
| tRNA processing (GO:0008033) | 23590 | 0.456484936 | 0.999994883 |
| non-canonical Wnt signaling pathway (GO:00 | 15/124 | 0.456650371 | 0.999994883 |
| activation of protein kinase C activity (GO:19 | 44566 | 0.456678815 | 0.999994883 |
| ADP transport (GO:0015866) | 44566 | 0.456678815 | 0.999994883 |
| AMP biosynthetic process (GO:0006167) | 44566 | 0.456678815 | 0.999994883 |
| antigen processing and presentation of lipid | 44566 | 0.456678815 | 0.999994883 |
| antigen processing and presentation, endoge | 44566 | 0.456678815 | 0.999994883 |
| antigen processing and presentation, exogen | 44566 | 0.456678815 | 0.999994883 |
| auditory receptor cell morphogenesis (GO:00 | 44566 | 0.456678815 | 0.999994883 |
| auditory receptor cell stereocilium organizati | 44566 | 0.456678815 | 0.999994883 |
| C-terminal protein deglutamylation (GO:003 | 44566 | 0.456678815 | 0.999994883 |
| cell migration involved in gastrulation (GO:00 | 44566 | 0.456678815 | 0.999994883 |
| cell-cell adhesion mediated by integrin (GO:0 | 44566 | 0.456678815 | 0.999994883 |
| cellular response to cGMP (GO:0071321) | 44566 | 0.456678815 | 0.999994883 |
| cellular response to erythropoietin (GO:0036 | 44566 | 0.456678815 | 0.999994883 |
| cellular response to macrophage colony-stim | 44566 | 0.456678815 | 0.999994883 |
| cellular response to nutrient (GO:0031670) | 44566 | 0.456678815 | 0.999994883 |
| cellular response to testosterone stimulus (G | 44566 | 0.456678815 | 0.999994883 |
| cerebellar Purkinje cell differentiation (GO:0 | 44566 | 0.456678815 | 0.999994883 |
| cerebral cortex neuron differentiation (GO:0 | 44566 | 0.456678815 | 0.999994883 |
| chemokine (C-X-C motif) ligand 12 signaling | 44566 | 0.456678815 | 0.999994883 |
| ciliary neurotrophic factor-mediated signalin | 44566 | 0.456678815 | 0.999994883 |
| CRD-mediated mRNA stabilization (GO:0070 | 44566 | 0.456678815 | 0.999994883 |

| | | | |
|---|---|---|---|
| cyclic-GMP-AMP transmembrane import acro | 44566 | 0.456678815 | 0.999994883 |
| deadenylation-independent decapping of nu | 44566 | 0.456678815 | 0.999994883 |
| determination of dorsal/ventral asymmetry ( | 44566 | 0.456678815 | 0.999994883 |
| determination of left/right asymmetry in late | 44566 | 0.456678815 | 0.999994883 |
| DNA ligation involved in DNA repair (GO:005 | 44566 | 0.456678815 | 0.999994883 |
| embryonic viscerocranium morphogenesis (G | 44566 | 0.456678815 | 0.999994883 |
| enamel mineralization (GO:0070166) | 44566 | 0.456678815 | 0.999994883 |
| endothelin receptor signaling pathway (GO:0 | 44566 | 0.456678815 | 0.999994883 |
| establishment of monopolar cell polarity (GO | 44566 | 0.456678815 | 0.999994883 |
| fructose transmembrane transport (GO:0015 | 44566 | 0.456678815 | 0.999994883 |
| gamma-aminobutyric acid metabolic process | 44566 | 0.456678815 | 0.999994883 |
| glomerular visceral epithelial cell migration ( | 44566 | 0.456678815 | 0.999994883 |
| glucose import across plasma membrane (GC | 44566 | 0.456678815 | 0.999994883 |
| glucose-6-phosphate transport (GO:0015760 | 44566 | 0.456678815 | 0.999994883 |
| glutathione transport (GO:0034635) | 44566 | 0.456678815 | 0.999994883 |
| glycerol biosynthetic process (GO:0006114) | 44566 | 0.456678815 | 0.999994883 |
| glycerol ether metabolic process (GO:000666 | 44566 | 0.456678815 | 0.999994883 |
| hematopoietic stem cell migration (GO:0035 | 44566 | 0.456678815 | 0.999994883 |
| heparin metabolic process (GO:0030202) | 44566 | 0.456678815 | 0.999994883 |
| hexose phosphate transport (GO:0015712) | 44566 | 0.456678815 | 0.999994883 |
| histone citrullination (GO:0036414) | 44566 | 0.456678815 | 0.999994883 |
| histone H4-K20 methylation (GO:0034770) | 44566 | 0.456678815 | 0.999994883 |
| histone H4-K20 trimethylation (GO:0034773) | 44566 | 0.456678815 | 0.999994883 |
| homoserine metabolic process (GO:0009092 | 44566 | 0.456678815 | 0.999994883 |
| intracellular sequestering of iron ion (GO:000 | 44566 | 0.456678815 | 0.999994883 |
| isocitrate metabolic process (GO:0006102) | 44566 | 0.456678815 | 0.999994883 |
| L-aspartate transmembrane transport (GO:0 | 44566 | 0.456678815 | 0.999994883 |
| late endosome to Golgi transport (GO:00344 | 44566 | 0.456678815 | 0.999994883 |
| late endosome to lysosome transport (GO:19 | 44566 | 0.456678815 | 0.999994883 |
| long-chain fatty acid catabolic process (GO:0 | 44566 | 0.456678815 | 0.999994883 |
| mannose trimming involved in glycoprotein E | 44566 | 0.456678815 | 0.999994883 |
| mature conventional dendritic cell differentia | 44566 | 0.456678815 | 0.999994883 |
| medium-chain fatty acid catabolic process (G | 44566 | 0.456678815 | 0.999994883 |
| meiotic sister chromatid segregation (GO:004 | 44566 | 0.456678815 | 0.999994883 |
| membrane raft distribution (GO:0031580) | 44566 | 0.456678815 | 0.999994883 |
| mesonephric duct development (GO:007217 | 44566 | 0.456678815 | 0.999994883 |
| metanephric distal tubule development (GO: | 44566 | 0.456678815 | 0.999994883 |
| mRNA cleavage involved in gene silencing by | 44566 | 0.456678815 | 0.999994883 |
| negative regulation of anion transmembrane | 44566 | 0.456678815 | 0.999994883 |
| negative regulation of CD4-positive, alpha-be | 44566 | 0.456678815 | 0.999994883 |
| negative regulation of cell volume (GO:00457 | 44566 | 0.456678815 | 0.999994883 |
| negative regulation of cellular respiration (G | 44566 | 0.456678815 | 0.999994883 |
| negative regulation of ceramide biosynthetic | 44566 | 0.456678815 | 0.999994883 |
| negative regulation of CREB transcription fac | 44566 | 0.456678815 | 0.999994883 |
| negative regulation of gene silencing by RNA | 44566 | 0.456678815 | 0.999994883 |
| negative regulation of histone deacetylation | 44566 | 0.456678815 | 0.999994883 |
| negative regulation of hydrogen peroxide-inc | 44566 | 0.456678815 | 0.999994883 |

| | | | |
|---|---|---|---|
| negative regulation of interferon-alpha produ | 44566 | 0.456678815 | 0.999994883 |
| negative regulation of lipid transport (GO:003 | 44566 | 0.456678815 | 0.999994883 |
| negative regulation of macrophage differenti | 44566 | 0.456678815 | 0.999994883 |
| negative regulation of mast cell activation inv | 44566 | 0.456678815 | 0.999994883 |
| negative regulation of metallopeptidase activ | 44566 | 0.456678815 | 0.999994883 |
| negative regulation of oxidative stress-induce | 44566 | 0.456678815 | 0.999994883 |
| negative regulation of posttranscriptional gen | 44566 | 0.456678815 | 0.999994883 |
| negative regulation of protein acetylation (GO | 44566 | 0.456678815 | 0.999994883 |
| negative regulation of response to food (GO: | 44566 | 0.456678815 | 0.999994883 |
| negative regulation of smooth muscle contra | 44566 | 0.456678815 | 0.999994883 |
| negative regulation of vascular endothelial ce | 44566 | 0.456678815 | 0.999994883 |
| nephric duct development (GO:0072176) | 44566 | 0.456678815 | 0.999994883 |
| nucleoside transport (GO:0015858) | 44566 | 0.456678815 | 0.999994883 |
| nucleoside triphosphate catabolic process (G | 44566 | 0.456678815 | 0.999994883 |
| oxaloacetate metabolic process (GO:000610 | 44566 | 0.456678815 | 0.999994883 |
| oxaloacetate transport (GO:0015729) | 44566 | 0.456678815 | 0.999994883 |
| pentose catabolic process (GO:0019323) | 44566 | 0.456678815 | 0.999994883 |
| peptidyl-lysine oxidation (GO:0018057) | 44566 | 0.456678815 | 0.999994883 |
| PML body organization (GO:0030578) | 44566 | 0.456678815 | 0.999994883 |
| positive regulation by host of viral genome re | 44566 | 0.456678815 | 0.999994883 |
| positive regulation of anoikis (GO:2000210) | 44566 | 0.456678815 | 0.999994883 |
| positive regulation of attachment of spindle r | 44566 | 0.456678815 | 0.999994883 |
| positive regulation of axon extension involve | 44566 | 0.456678815 | 0.999994883 |
| positive regulation of bicellular tight junction | 44566 | 0.456678815 | 0.999994883 |
| positive regulation of cAMP-dependent prote | 44566 | 0.456678815 | 0.999994883 |
| positive regulation of fertilization (GO:19055 | 44566 | 0.456678815 | 0.999994883 |
| positive regulation of fibroblast growth facto | 44566 | 0.456678815 | 0.999994883 |
| positive regulation of heat generation (GO:00 | 44566 | 0.456678815 | 0.999994883 |
| positive regulation of histone H3-K9 acetylati | 44566 | 0.456678815 | 0.999994883 |
| positive regulation of leukocyte tethering or r | 44566 | 0.456678815 | 0.999994883 |
| positive regulation of maintenance of mitotic | 44566 | 0.456678815 | 0.999994883 |
| positive regulation of maintenance of sister c | 44566 | 0.456678815 | 0.999994883 |
| positive regulation of mast cell activation inv | 44566 | 0.456678815 | 0.999994883 |
| positive regulation of mast cell chemotaxis (G | 44566 | 0.456678815 | 0.999994883 |
| positive regulation of mitochondrial depolari | 44566 | 0.456678815 | 0.999994883 |
| positive regulation of non-motile cilium asser | 44566 | 0.456678815 | 0.999994883 |
| positive regulation of peroxisome proliferato | 44566 | 0.456678815 | 0.999994883 |
| positive regulation of protein catabolic proce | 44566 | 0.456678815 | 0.999994883 |
| positive regulation of protein localization to c | 44566 | 0.456678815 | 0.999994883 |
| positive regulation of protein monoubiquitina | 44566 | 0.456678815 | 0.999994883 |
| positive regulation of the force of heart contr | 44566 | 0.456678815 | 0.999994883 |
| postsynaptic endocytosis (GO:0140239) | 44566 | 0.456678815 | 0.999994883 |
| postsynaptic neurotransmitter receptor inter | 44566 | 0.456678815 | 0.999994883 |
| protein C-linked glycosylation via 2'-alpha-ma | 44566 | 0.456678815 | 0.999994883 |
| protein C-linked glycosylation via tryptophan | 44566 | 0.456678815 | 0.999994883 |
| protein citrullination (GO:0018101) | 44566 | 0.456678815 | 0.999994883 |
| protein deglycosylation involved in glycoprot | 44566 | 0.456678815 | 0.999994883 |

| | | | |
|---|---|---|---|
| protein K69-linked ufmylation (GO:1990592) | 44566 | 0.456678815 | 0.999994883 |
| protein localization to axon (GO:0099612) | 44566 | 0.456678815 | 0.999994883 |
| protein localization to microtubule end (GO:1 | 44566 | 0.456678815 | 0.999994883 |
| protein localization to microtubule plus-end ( | 44566 | 0.456678815 | 0.999994883 |
| protein localization to site of double-strand b | 44566 | 0.456678815 | 0.999994883 |
| protein polyufmylation (GO:1990564) | 44566 | 0.456678815 | 0.999994883 |
| protein sialylation (GO:1990743) | 44566 | 0.456678815 | 0.999994883 |
| protein side chain deglutamylation (GO:0035 | 44566 | 0.456678815 | 0.999994883 |
| pyrimidine deoxyribonucleotide biosynthetic | 44566 | 0.456678815 | 0.999994883 |
| pyrimidine nucleobase biosynthetic process ( | 44566 | 0.456678815 | 0.999994883 |
| pyrimidine nucleoside transport (GO:001586 | 44566 | 0.456678815 | 0.999994883 |
| pyrimidine nucleotide metabolic process (GO | 44566 | 0.456678815 | 0.999994883 |
| receptor-mediated virion attachment to host | 44566 | 0.456678815 | 0.999994883 |
| regulation of astrocyte activation (GO:00618 | 44566 | 0.456678815 | 0.999994883 |
| regulation of caveolin-mediated endocytosis | 44566 | 0.456678815 | 0.999994883 |
| regulation of cellular response to osmotic str | 44566 | 0.456678815 | 0.999994883 |
| regulation of chaperone-mediated protein fo | 44566 | 0.456678815 | 0.999994883 |
| regulation of cytochrome-c oxidase activity ( | 44566 | 0.456678815 | 0.999994883 |
| regulation of germinal center formation (GO: | 44566 | 0.456678815 | 0.999994883 |
| regulation of glomerular mesangial cell prolif | 44566 | 0.456678815 | 0.999994883 |
| regulation of granulocyte colony-stimulating | 44566 | 0.456678815 | 0.999994883 |
| regulation of hippocampal neuron apoptotic | 44566 | 0.456678815 | 0.999994883 |
| regulation of histone H4-K16 acetylation (GO | 44566 | 0.456678815 | 0.999994883 |
| regulation of hydrogen peroxide biosynthetic | 44566 | 0.456678815 | 0.999994883 |
| regulation of inward rectifier potassium chan | 44566 | 0.456678815 | 0.999994883 |
| regulation of low-density lipoprotein particle | 44566 | 0.456678815 | 0.999994883 |
| regulation of maintenance of mitotic sister ch | 44566 | 0.456678815 | 0.999994883 |
| regulation of metaphase plate congression (G | 44566 | 0.456678815 | 0.999994883 |
| regulation of microglial cell mediated cytotox | 44566 | 0.456678815 | 0.999994883 |
| regulation of microvillus assembly (GO:00325 | 44566 | 0.456678815 | 0.999994883 |
| regulation of mitophagy (GO:1901524) | 44566 | 0.456678815 | 0.999994883 |
| regulation of morphogenesis of a branching s | 44566 | 0.456678815 | 0.999994883 |
| regulation of myoblast proliferation (GO:200 | 44566 | 0.456678815 | 0.999994883 |
| regulation of natural killer cell differentiation | 44566 | 0.456678815 | 0.999994883 |
| regulation of neutrophil activation (GO:19025 | 44566 | 0.456678815 | 0.999994883 |
| regulation of phosphatidylinositol biosynthet | 44566 | 0.456678815 | 0.999994883 |
| regulation of phosphatidylinositol dephospho | 44566 | 0.456678815 | 0.999994883 |
| regulation of postsynaptic density organizatio | 44566 | 0.456678815 | 0.999994883 |
| regulation of protein monoubiquitination (GO | 44566 | 0.456678815 | 0.999994883 |
| regulation of respiratory burst involved in inf | 44566 | 0.456678815 | 0.999994883 |
| regulation of snRNA transcription by RNA pol | 44566 | 0.456678815 | 0.999994883 |
| regulation of sterol transport (GO:0032371) | 44566 | 0.456678815 | 0.999994883 |
| regulation of synaptic vesicle recycling (GO:1 | 44566 | 0.456678815 | 0.999994883 |
| regulation of triglyceride metabolic process ( | 44566 | 0.456678815 | 0.999994883 |
| regulation of type B pancreatic cell apoptotic | 44566 | 0.456678815 | 0.999994883 |
| renal tubule development (GO:0061326) | 44566 | 0.456678815 | 0.999994883 |
| rescue of stalled ribosome (GO:0072344) | 44566 | 0.456678815 | 0.999994883 |

| | | | |
|---|---|---|---|
| response to acetylcholine (GO:1905144) | 44566 | 0.456678815 | 0.999994883 |
| response to cGMP (GO:0070305) | 44566 | 0.456678815 | 0.999994883 |
| response to denervation involved in regulatic | 44566 | 0.456678815 | 0.999994883 |
| response to macrophage colony-stimulating f | 44566 | 0.456678815 | 0.999994883 |
| response to mitochondrial depolarisation (GC | 44566 | 0.456678815 | 0.999994883 |
| response to morphine (GO:0043278) | 44566 | 0.456678815 | 0.999994883 |
| response to prostaglandin (GO:0034694) | 44566 | 0.456678815 | 0.999994883 |
| ribose phosphate metabolic process (GO:001 | 44566 | 0.456678815 | 0.999994883 |
| ribosome localization (GO:0033750) | 44566 | 0.456678815 | 0.999994883 |
| Schwann cell differentiation (GO:0014037) | 44566 | 0.456678815 | 0.999994883 |
| sensory perception of sour taste (GO:005091 | 44566 | 0.456678815 | 0.999994883 |
| short-term memory (GO:0007614) | 44566 | 0.456678815 | 0.999994883 |
| sialic acid transport (GO:0015739) | 44566 | 0.456678815 | 0.999994883 |
| sulfide oxidation (GO:0019418) | 44566 | 0.456678815 | 0.999994883 |
| sulfide oxidation, using sulfide:quinone oxidc | 44566 | 0.456678815 | 0.999994883 |
| tight junction organization (GO:0120193) | 44566 | 0.456678815 | 0.999994883 |
| Toll signaling pathway (GO:0008063) | 44566 | 0.456678815 | 0.999994883 |
| transsulfuration (GO:0019346) | 44566 | 0.456678815 | 0.999994883 |
| tricuspid valve morphogenesis (GO:0003186) | 44566 | 0.456678815 | 0.999994883 |
| trivalent inorganic anion homeostasis (GO:0C | 44566 | 0.456678815 | 0.999994883 |
| trophoblast giant cell differentiation (GO:006 | 44566 | 0.456678815 | 0.999994883 |
| tryptophan catabolic process to kynurenine ( | 44566 | 0.456678815 | 0.999994883 |
| ubiquitin-dependent glycoprotein ERAD path | 44566 | 0.456678815 | 0.999994883 |
| uridine transport (GO:0015862) | 44566 | 0.456678815 | 0.999994883 |
| vascular endothelial growth factor receptor-2 | 44566 | 0.456678815 | 0.999994883 |
| vesicle fusion with Golgi apparatus (GO:0048 | 44566 | 0.456678815 | 0.999994883 |
| vesicle tethering to Golgi (GO:0099041) | 44566 | 0.456678815 | 0.999994883 |
| vitamin D3 metabolic process (GO:0070640) | 44566 | 0.456678815 | 0.999994883 |
| chondroitin sulfate metabolic process (GO:0C | 44681 | 0.457458652 | 0.999994883 |
| establishment of spindle orientation (GO:005 | 44681 | 0.457458652 | 0.999994883 |
| estrogen metabolic process (GO:0008210) | 44681 | 0.457458652 | 0.999994883 |
| ion homeostasis (GO:0050801) | 44681 | 0.457458652 | 0.999994883 |
| morphogenesis of an epithelium (GO:000200 | 44681 | 0.457458652 | 0.999994883 |
| ncRNA catabolic process (GO:0034661) | 44681 | 0.457458652 | 0.999994883 |
| positive regulation of B cell proliferation (GO | 44681 | 0.457458652 | 0.999994883 |
| positive regulation of DNA recombination (GC | 44681 | 0.457458652 | 0.999994883 |
| regulation of T cell activation (GO:0050863) | 17319 | 0.457695073 | 0.999994883 |
| regulation of cytokine production (GO:00018 | 18/150 | 0.459169711 | 0.999994883 |
| positive regulation of autophagy (GO:001050 | 33178 | 0.461528755 | 0.999994883 |
| positive regulation of binding (GO:0051099) | 33178 | 0.461528755 | 0.999994883 |
| protein complex oligomerization (GO:005125 | 33178 | 0.461528755 | 0.999994883 |
| cellular response to oxygen-containing comp | 38/323 | 0.463046977 | 0.999994883 |
| ubiquitin-dependent ERAD pathway (GO:003 | 26908 | 0.464146465 | 0.999994883 |
| regulation of protein-containing complex ass | 14/116 | 0.464532339 | 0.999994883 |
| DNA damage response, signal transduction b | 20637 | 0.466857819 | 0.999994883 |
| positive regulation of establishment of prote | 20637 | 0.466857819 | 0.999994883 |
| regulation of ATP metabolic process (GO:190 | 20637 | 0.466857819 | 0.999994883 |

| | | | |
|---|---|---|---|
| membrane protein proteolysis (GO:0033619) | 14366 | 0.469513813 | 0.999994883 |
| phospholipid dephosphorylation (GO:004683 | 14366 | 0.469513813 | 0.999994883 |
| regulation of dendrite development (GO:005 | 14366 | 0.469513813 | 0.999994883 |
| regulation of intracellular transport (GO:0032 | 14366 | 0.469513813 | 0.999994883 |
| regulation of protein tyrosine kinase activity | 14366 | 0.469513813 | 0.999994883 |
| regulation of receptor-mediated endocytosis | 14366 | 0.469513813 | 0.999994883 |
| ephrin receptor signaling pathway (GO:0048( | 30225 | 0.470808832 | 0.999994883 |
| regulation of gene expression, epigenetic (GC | 30225 | 0.470808832 | 0.999994883 |
| amino acid import (GO:0043090) | 44642 | 0.471235573 | 0.999994883 |
| cell projection assembly (GO:0030031) | 44642 | 0.471235573 | 0.999994883 |
| chaperone-mediated protein complex assem | 44642 | 0.471235573 | 0.999994883 |
| glutamate metabolic process (GO:0006536) | 44642 | 0.471235573 | 0.999994883 |
| limb morphogenesis (GO:0035108) | 44642 | 0.471235573 | 0.999994883 |
| lipid droplet organization (GO:0034389) | 44642 | 0.471235573 | 0.999994883 |
| membrane lipid metabolic process (GO:0006 | 44642 | 0.471235573 | 0.999994883 |
| membrane protein intracellular domain prote | 44642 | 0.471235573 | 0.999994883 |
| metallo-sulfur cluster assembly (GO:0031163 | 44642 | 0.471235573 | 0.999994883 |
| microglial cell activation (GO:0001774) | 44642 | 0.471235573 | 0.999994883 |
| negative regulation of lipid biosynthetic proc | 44642 | 0.471235573 | 0.999994883 |
| negative regulation of lymphocyte activation | 44642 | 0.471235573 | 0.999994883 |
| neuronal action potential (GO:0019228) | 44642 | 0.471235573 | 0.999994883 |
| osteoclast differentiation (GO:0030316) | 44642 | 0.471235573 | 0.999994883 |
| regulation of centrosome cycle (GO:0046605 | 44642 | 0.471235573 | 0.999994883 |
| regulation of platelet activation (GO:0010543 | 44642 | 0.471235573 | 0.999994883 |
| tetrapyrrole metabolic process (GO:0033013 | 44642 | 0.471235573 | 0.999994883 |
| zinc ion transmembrane transport (GO:0071! | 44642 | 0.471235573 | 0.999994883 |
| cognition (GO:0050890) | 17685 | 0.478676666 | 0.999994883 |
| histone H4 acetylation (GO:0043967) | 17685 | 0.478676666 | 0.999994883 |
| negative regulation of mitotic cell cycle (GO:( | 17685 | 0.478676666 | 0.999994883 |
| negative regulation of tumor necrosis factor : | 17685 | 0.478676666 | 0.999994883 |
| negative regulation of axon extension (GO:0C | 11414 | 0.483692216 | 0.999994883 |
| negative regulation of proteasomal ubiquitin | 11414 | 0.483692216 | 0.999994883 |
| protein acetylation (GO:0006473) | 11414 | 0.483692216 | 0.999994883 |
| protein palmitoylation (GO:0018345) | 11414 | 0.483692216 | 0.999994883 |
| regulation of protein ubiquitination (GO:003: | 13/109 | 0.486818653 | 0.999994883 |
| activation of cysteine-type endopeptidase ac | 44606 | 0.489612115 | 0.999994883 |
| C4-dicarboxylate transport (GO:0015740) | 44606 | 0.489612115 | 0.999994883 |
| cellular copper ion homeostasis (GO:000687{ | 44606 | 0.489612115 | 0.999994883 |
| ceramide transport (GO:0035627) | 44606 | 0.489612115 | 0.999994883 |
| cGMP-mediated signaling (GO:0019934) | 44606 | 0.489612115 | 0.999994883 |
| intestinal absorption (GO:0050892) | 44606 | 0.489612115 | 0.999994883 |
| low-density lipoprotein particle remodeling (( | 44606 | 0.489612115 | 0.999994883 |
| negative regulation of coagulation (GO:0050{ | 44606 | 0.489612115 | 0.999994883 |
| negative regulation of cyclase activity (GO:0C | 44606 | 0.489612115 | 0.999994883 |
| negative regulation of fibroblast growth facto | 44606 | 0.489612115 | 0.999994883 |
| negative regulation of translational initiation | 44606 | 0.489612115 | 0.999994883 |
| neurotransmitter biosynthetic process (GO:0 | 44606 | 0.489612115 | 0.999994883 |

| | | | |
|---|---|---|---|
| nuclear membrane disassembly (GO:005108: | 44606 | 0.489612115 | 0.999994883 |
| positive regulation of alpha-beta T cell differe | 44606 | 0.489612115 | 0.999994883 |
| positive regulation of long-term synaptic pot | 44606 | 0.489612115 | 0.999994883 |
| positive regulation of mitochondrial translati | 44606 | 0.489612115 | 0.999994883 |
| positive regulation of superoxide anion gene | 44606 | 0.489612115 | 0.999994883 |
| positive regulation of transmembrane transp | 44606 | 0.489612115 | 0.999994883 |
| protein modification by small protein conjug | 44606 | 0.489612115 | 0.999994883 |
| regulation of chromatin organization (GO:19( | 44606 | 0.489612115 | 0.999994883 |
| regulation of fatty acid beta-oxidation (GO:0( | 44606 | 0.489612115 | 0.999994883 |
| regulation of intrinsic apoptotic signaling pat | 44606 | 0.489612115 | 0.999994883 |
| regulation of protein kinase C signaling (GO:C | 44606 | 0.489612115 | 0.999994883 |
| regulation of sensory perception of pain (GO: | 44606 | 0.489612115 | 0.999994883 |
| regulation of trophoblast cell migration (GO:: | 44606 | 0.489612115 | 0.999994883 |
| renal absorption (GO:0070293) | 44606 | 0.489612115 | 0.999994883 |
| T-helper cell differentiation (GO:0042093) | 44606 | 0.489612115 | 0.999994883 |
| tRNA wobble uridine modification (GO:0002( | 44606 | 0.489612115 | 0.999994883 |
| negative regulation of mitotic cell cycle phas | 33909 | 0.491802294 | 0.999994883 |
| cellular component disassembly (GO:002241 | 24320 | 0.492316285 | 0.999994883 |
| extracellular matrix disassembly (GO:002261 | 24320 | 0.492316285 | 0.999994883 |
| negative regulation of G2/M transition of mit | 24320 | 0.492316285 | 0.999994883 |
| regulation of protein localization (GO:003288 | 24320 | 0.492316285 | 0.999994883 |
| negative regulation of I-kappaB kinase/NF-ka | 14732 | 0.49251957 | 0.999994883 |
| positive regulation of double-strand break re | 14732 | 0.49251957 | 0.999994883 |
| regulation of sodium ion transmembrane tra | 14732 | 0.49251957 | 0.999994883 |
| epithelial cell differentiation (GO:0030855) | 12/101 | 0.49640466 | 0.999994883 |
| intracellular protein transport (GO:0006886) | 39/336 | 0.497592756 | 0.999994883 |
| cellular response to mechanical stimulus (GO | 18050 | 0.499408614 | 0.999994883 |
| negative regulation of catabolic process (GO: | 18050 | 0.499408614 | 0.999994883 |
| negative regulation of cell projection organiz | 18050 | 0.499408614 | 0.999994883 |
| positive regulation of reactive oxygen specie | 18050 | 0.499408614 | 0.999994883 |
| receptor internalization (GO:0031623) | 18050 | 0.499408614 | 0.999994883 |
| RNA catabolic process (GO:0006401) | 18050 | 0.499408614 | 0.999994883 |
| cilium-dependent cell motility (GO:0060285) | 44643 | 0.501757184 | 0.999994883 |
| glucose 6-phosphate metabolic process (GO:( | 44643 | 0.501757184 | 0.999994883 |
| negative regulation of phosphoprotein phosp | 44643 | 0.501757184 | 0.999994883 |
| negative regulation of smoothened signaling | 44643 | 0.501757184 | 0.999994883 |
| oligosaccharide biosynthetic process (GO:00( | 44643 | 0.501757184 | 0.999994883 |
| positive regulation of cholesterol efflux (GO:( | 44643 | 0.501757184 | 0.999994883 |
| positive regulation of epithelial cell apoptotic | 44643 | 0.501757184 | 0.999994883 |
| positive regulation of natural killer cell media | 44643 | 0.501757184 | 0.999994883 |
| regulation of B cell receptor signaling pathwa | 44643 | 0.501757184 | 0.999994883 |
| regulation of peptidase activity (GO:0052547 | 44643 | 0.501757184 | 0.999994883 |
| triglyceride catabolic process (GO:0019433) | 44643 | 0.501757184 | 0.999994883 |
| chordate embryonic development (GO:0043( | 21367 | 0.50510143 | 0.999994883 |
| muscle organ development (GO:0007517) | 21367 | 0.50510143 | 0.999994883 |
| positive regulation of mitotic cell cycle phase | 21367 | 0.50510143 | 0.999994883 |
| regulation of calcium ion transport (GO:0051 | 21367 | 0.50510143 | 0.999994883 |

| | | | |
|---|---|---|---|
| regulation of tumor necrosis factor-mediatec | 21367 | 0.50510143 | 0.999994883 |
| endodermal cell differentiation (GO:0035987 | 11780 | 0.50940142 | 0.999994883 |
| gene silencing by miRNA (GO:0035195) | 11780 | 0.50940142 | 0.999994883 |
| metal ion export (GO:0070839) | 11780 | 0.50940142 | 0.999994883 |
| negative regulation of DNA biosynthetic proc | 11780 | 0.50940142 | 0.999994883 |
| regulation of cellular biosynthetic process (G | 11780 | 0.50940142 | 0.999994883 |
| regulation of chemotaxis (GO:0050920) | 11780 | 0.50940142 | 0.999994883 |
| establishment of protein localization to orgar | 28004 | 0.514285484 | 0.999994883 |
| regulation of cell division (GO:0051302) | 28004 | 0.514285484 | 0.999994883 |
| endosome organization (GO:0007032) | 15097 | 0.515146251 | 0.999994883 |
| epithelial cell migration (GO:0010631) | 15097 | 0.515146251 | 0.999994883 |
| response to growth factor (GO:0070848) | 15097 | 0.515146251 | 0.999994883 |
| proteasome-mediated ubiquitin-dependent p | 37/321 | 0.517056767 | 0.999994883 |
| cytoskeleton organization (GO:0007010) | 14/120 | 0.517656761 | 0.999994883 |
| acetyl-CoA biosynthetic process from pyruva | 44567 | 0.519094735 | 0.999994883 |
| adiponectin-activated signaling pathway (GO | 44567 | 0.519094735 | 0.999994883 |
| alditol metabolic process (GO:0019400) | 44567 | 0.519094735 | 0.999994883 |
| amino-acid betaine transport (GO:0015838) | 44567 | 0.519094735 | 0.999994883 |
| aminophospholipid transport (GO:0015917) | 44567 | 0.519094735 | 0.999994883 |
| anterior/posterior axis specification, embryo | 44567 | 0.519094735 | 0.999994883 |
| aromatic amino acid transport (GO:0015801) | 44567 | 0.519094735 | 0.999994883 |
| aspartate metabolic process (GO:0006531) | 44567 | 0.519094735 | 0.999994883 |
| B cell chemotaxis (GO:0035754) | 44567 | 0.519094735 | 0.999994883 |
| cell junction disassembly (GO:0150146) | 44567 | 0.519094735 | 0.999994883 |
| cellular heat acclimation (GO:0070370) | 44567 | 0.519094735 | 0.999994883 |
| cellular response to bacterial lipopeptide (GC | 44567 | 0.519094735 | 0.999994883 |
| cellular response to cold (GO:0070417) | 44567 | 0.519094735 | 0.999994883 |
| cellular response to granulocyte macrophage | 44567 | 0.519094735 | 0.999994883 |
| cellular response to iron ion (GO:0071281) | 44567 | 0.519094735 | 0.999994883 |
| cellular response to magnesium ion (GO:007: | 44567 | 0.519094735 | 0.999994883 |
| cellular triglyceride homeostasis (GO:003535 | 44567 | 0.519094735 | 0.999994883 |
| choline catabolic process (GO:0042426) | 44567 | 0.519094735 | 0.999994883 |
| dehydroascorbic acid transport (GO:0070837 | 44567 | 0.519094735 | 0.999994883 |
| dendrite extension (GO:0097484) | 44567 | 0.519094735 | 0.999994883 |
| dermatan sulfate proteoglycan metabolic prc | 44567 | 0.519094735 | 0.999994883 |
| detection of virus (GO:0009597) | 44567 | 0.519094735 | 0.999994883 |
| determination of liver left/right asymmetry (( | 44567 | 0.519094735 | 0.999994883 |
| double-strand break repair via classical nonh | 44567 | 0.519094735 | 0.999994883 |
| embryonic camera-type eye development (G | 44567 | 0.519094735 | 0.999994883 |
| embryonic camera-type eye morphogenesis ( | 44567 | 0.519094735 | 0.999994883 |
| epithelial tube formation (GO:0072175) | 44567 | 0.519094735 | 0.999994883 |
| foam cell differentiation (GO:0090077) | 44567 | 0.519094735 | 0.999994883 |
| galactolipid biosynthetic process (GO:001937 | 44567 | 0.519094735 | 0.999994883 |
| galactosylceramide biosynthetic process (GO | 44567 | 0.519094735 | 0.999994883 |
| gamma-aminobutyric acid transport (GO:001 | 44567 | 0.519094735 | 0.999994883 |
| glomerulus vasculature development (GO:00 | 44567 | 0.519094735 | 0.999994883 |
| glycerol-3-phosphate metabolic process (GO: | 44567 | 0.519094735 | 0.999994883 |

| | | | |
|---|---|---|---|
| glycolipid transport (GO:0046836) | 44567 | 0.519094735 | 0.999994883 |
| glycosylceramide catabolic process (GO:0046 | 44567 | 0.519094735 | 0.999994883 |
| glycosylceramide metabolic process (GO:000 | 44567 | 0.519094735 | 0.999994883 |
| growth hormone secretion (GO:0030252) | 44567 | 0.519094735 | 0.999994883 |
| guanine nucleotide transmembrane transpor | 44567 | 0.519094735 | 0.999994883 |
| heat acclimation (GO:0010286) | 44567 | 0.519094735 | 0.999994883 |
| heparan sulfate proteoglycan biosynthetic pr | 44567 | 0.519094735 | 0.999994883 |
| hexose import across plasma membrane (GO | 44567 | 0.519094735 | 0.999994883 |
| hydrogen sulfide metabolic process (GO:007( | 44567 | 0.519094735 | 0.999994883 |
| IMP biosynthetic process (GO:0006188) | 44567 | 0.519094735 | 0.999994883 |
| L-ornithine transmembrane transport (GO:19 | 44567 | 0.519094735 | 0.999994883 |
| L-serine biosynthetic process (GO:0006564) | 44567 | 0.519094735 | 0.999994883 |
| leukocyte chemotaxis involved in inflammatc | 44567 | 0.519094735 | 0.999994883 |
| lipoxin metabolic process (GO:2001300) | 44567 | 0.519094735 | 0.999994883 |
| macromolecule biosynthetic process (GO:00( | 44567 | 0.519094735 | 0.999994883 |
| macrophage derived foam cell differentiatior | 44567 | 0.519094735 | 0.999994883 |
| magnesium ion homeostasis (GO:0010960) | 44567 | 0.519094735 | 0.999994883 |
| maintenance of DNA methylation (GO:00102 | 44567 | 0.519094735 | 0.999994883 |
| maintenance of DNA repeat elements (GO:0( | 44567 | 0.519094735 | 0.999994883 |
| medium-chain fatty acid biosynthetic process | 44567 | 0.519094735 | 0.999994883 |
| meiotic DNA double-strand break formation ( | 44567 | 0.519094735 | 0.999994883 |
| metaphase/anaphase transition of cell cycle ( | 44567 | 0.519094735 | 0.999994883 |
| metaphase/anaphase transition of mitotic ce | 44567 | 0.519094735 | 0.999994883 |
| mitochondrial ribosome assembly (GO:0061( | 44567 | 0.519094735 | 0.999994883 |
| mRNA 5'-splice site recognition (GO:0000395 | 44567 | 0.519094735 | 0.999994883 |
| multinuclear osteoclast differentiation (GO:0 | 44567 | 0.519094735 | 0.999994883 |
| N-terminal peptidyl-methionine acetylation ( | 44567 | 0.519094735 | 0.999994883 |
| negative regulation of actin filament depolyn | 44567 | 0.519094735 | 0.999994883 |
| negative regulation of amino acid transport ( | 44567 | 0.519094735 | 0.999994883 |
| negative regulation of anion transport (GO:1! | 44567 | 0.519094735 | 0.999994883 |
| negative regulation of Arp2/3 complex-medi | 44567 | 0.519094735 | 0.999994883 |
| negative regulation of astrocyte differentiatio | 44567 | 0.519094735 | 0.999994883 |
| negative regulation of B cell receptor signalin | 44567 | 0.519094735 | 0.999994883 |
| negative regulation of chemokine-mediated s | 44567 | 0.519094735 | 0.999994883 |
| negative regulation of cytokinesis (GO:00324 | 44567 | 0.519094735 | 0.999994883 |
| negative regulation of endoplasmic reticulum | 44567 | 0.519094735 | 0.999994883 |
| negative regulation of ERAD pathway (GO:19 | 44567 | 0.519094735 | 0.999994883 |
| negative regulation of glial cell proliferation ( | 44567 | 0.519094735 | 0.999994883 |
| negative regulation of glycogen biosynthetic | 44567 | 0.519094735 | 0.999994883 |
| negative regulation of lipoprotein lipase activ | 44567 | 0.519094735 | 0.999994883 |
| negative regulation of mast cell activation (G | 44567 | 0.519094735 | 0.999994883 |
| negative regulation of monocyte chemotaxis | 44567 | 0.519094735 | 0.999994883 |
| negative regulation of myeloid leukocyte me | 44567 | 0.519094735 | 0.999994883 |
| negative regulation of T cell differentiation in | 44567 | 0.519094735 | 0.999994883 |
| negative regulation of toll-like receptor 2 sigr | 44567 | 0.519094735 | 0.999994883 |
| negative regulation of trophoblast cell migrat | 44567 | 0.519094735 | 0.999994883 |
| nephric duct morphogenesis (GO:0072178) | 44567 | 0.519094735 | 0.999994883 |

| | | | |
|---|---|---|---|
| nuclear polyadenylation-dependent ncRNA ca | 44567 | 0.519094735 | 0.999994883 |
| nuclear polyadenylation-dependent rRNA cat | 44567 | 0.519094735 | 0.999994883 |
| nuclear polyadenylation-dependent tRNA cat | 44567 | 0.519094735 | 0.999994883 |
| nucleotide transport (GO:0006862) | 44567 | 0.519094735 | 0.999994883 |
| pentose-phosphate shunt, non-oxidative bra | 44567 | 0.519094735 | 0.999994883 |
| peptidyl-diphthamide biosynthetic process fr | 44567 | 0.519094735 | 0.999994883 |
| peptidyl-diphthamide metabolic process (GO | 44567 | 0.519094735 | 0.999994883 |
| peroxisome proliferator activated receptor si | 44567 | 0.519094735 | 0.999994883 |
| photoreceptor cell outer segment organizatio | 44567 | 0.519094735 | 0.999994883 |
| polyphosphate metabolic process (GO:00067 | 44567 | 0.519094735 | 0.999994883 |
| positive regulation of antigen processing and | 44567 | 0.519094735 | 0.999994883 |
| positive regulation of autophagy of mitochor | 44567 | 0.519094735 | 0.999994883 |
| positive regulation of centriole replication (G | 44567 | 0.519094735 | 0.999994883 |
| positive regulation of cholesterol storage (GC | 44567 | 0.519094735 | 0.999994883 |
| positive regulation of dendritic cell antigen p | 44567 | 0.519094735 | 0.999994883 |
| positive regulation of endoplasmic reticulum | 44567 | 0.519094735 | 0.999994883 |
| positive regulation of gluconeogenesis (GO:0 | 44567 | 0.519094735 | 0.999994883 |
| positive regulation of gonad development (G | 44567 | 0.519094735 | 0.999994883 |
| positive regulation of histone phosphorylatio | 44567 | 0.519094735 | 0.999994883 |
| positive regulation of male gonad developme | 44567 | 0.519094735 | 0.999994883 |
| positive regulation of microglial cell migratio | 44567 | 0.519094735 | 0.999994883 |
| positive regulation of natural killer cell media | 44567 | 0.519094735 | 0.999994883 |
| positive regulation of natural killer cell media | 44567 | 0.519094735 | 0.999994883 |
| positive regulation of non-membrane spanni | 44567 | 0.519094735 | 0.999994883 |
| positive regulation of plasminogen activation | 44567 | 0.519094735 | 0.999994883 |
| positive regulation of receptor binding (GO:1 | 44567 | 0.519094735 | 0.999994883 |
| positive regulation of tau-protein kinase activ | 44567 | 0.519094735 | 0.999994883 |
| positive regulation of Wnt signaling pathway, | 44567 | 0.519094735 | 0.999994883 |
| proline transport (GO:0015824) | 44567 | 0.519094735 | 0.999994883 |
| pronephros development (GO:0048793) | 44567 | 0.519094735 | 0.999994883 |
| protein localization to nuclear envelope (GO: | 44567 | 0.519094735 | 0.999994883 |
| protein localization to nucleolus (GO:190257 | 44567 | 0.519094735 | 0.999994883 |
| protein sulfation (GO:0006477) | 44567 | 0.519094735 | 0.999994883 |
| protein ufmylation (GO:0071569) | 44567 | 0.519094735 | 0.999994883 |
| protein-DNA covalent cross-linking repair (GC | 44567 | 0.519094735 | 0.999994883 |
| proton-transporting V-type ATPase complex a | 44567 | 0.519094735 | 0.999994883 |
| pyrimidine nucleotide-sugar transmembrane | 44567 | 0.519094735 | 0.999994883 |
| regulation of adiponectin secretion (GO:007C | 44567 | 0.519094735 | 0.999994883 |
| regulation of cardiac muscle hypertrophy in r | 44567 | 0.519094735 | 0.999994883 |
| regulation of collagen fibril organization (GO: | 44567 | 0.519094735 | 0.999994883 |
| regulation of digestive system process (GO:0 | 44567 | 0.519094735 | 0.999994883 |
| regulation of excitatory synapse assembly (G | 44567 | 0.519094735 | 0.999994883 |
| regulation of glucagon secretion (GO:007009 | 44567 | 0.519094735 | 0.999994883 |
| regulation of hyaluronan biosynthetic proces | 44567 | 0.519094735 | 0.999994883 |
| regulation of immunoglobulin mediated imm | 44567 | 0.519094735 | 0.999994883 |
| regulation of interleukin-1-mediated signalin | 44567 | 0.519094735 | 0.999994883 |
| regulation of keratinocyte apoptotic process | 44567 | 0.519094735 | 0.999994883 |

| | | | |
|---|---|---|---|
| regulation of mast cell chemotaxis (GO:0060 | 44567 | 0.519094735 | 0.999994883 |
| regulation of mitotic sister chromatid segreg | 44567 | 0.519094735 | 0.999994883 |
| regulation of mitotic spindle checkpoint (GO: | 44567 | 0.519094735 | 0.999994883 |
| regulation of nitric oxide mediated signal tra | 44567 | 0.519094735 | 0.999994883 |
| regulation of non-motile cilium assembly (GC | 44567 | 0.519094735 | 0.999994883 |
| regulation of nuclease activity (GO:0032069) | 44567 | 0.519094735 | 0.999994883 |
| regulation of protein autoubiquitination (GO: | 44567 | 0.519094735 | 0.999994883 |
| regulation of resting membrane potential (G | 44567 | 0.519094735 | 0.999994883 |
| regulation of retrograde transport, endosom | 44567 | 0.519094735 | 0.999994883 |
| regulation of T-helper 17 cell lineage commit | 44567 | 0.519094735 | 0.999994883 |
| regulation of type 2 immune response (GO:0 | 44567 | 0.519094735 | 0.999994883 |
| regulation of ventricular cardiac muscle cell r | 44567 | 0.519094735 | 0.999994883 |
| replication-born double-strand break repair v | 44567 | 0.519094735 | 0.999994883 |
| response to catecholamine (GO:0071869) | 44567 | 0.519094735 | 0.999994883 |
| response to DNA damage checkpoint signalin | 44567 | 0.519094735 | 0.999994883 |
| response to granulocyte macrophage colony- | 44567 | 0.519094735 | 0.999994883 |
| response to testosterone (GO:0033574) | 44567 | 0.519094735 | 0.999994883 |
| sialylation (GO:0097503) | 44567 | 0.519094735 | 0.999994883 |
| signal complex assembly (GO:0007172) | 44567 | 0.519094735 | 0.999994883 |
| snRNA modification (GO:0040031) | 44567 | 0.519094735 | 0.999994883 |
| spinal cord motor neuron differentiation (GO | 44567 | 0.519094735 | 0.999994883 |
| succinate transmembrane transport (GO:007 | 44567 | 0.519094735 | 0.999994883 |
| T-tubule organization (GO:0033292) | 44567 | 0.519094735 | 0.999994883 |
| tripartite regional subdivision (GO:0007351) | 44567 | 0.519094735 | 0.999994883 |
| tRNA surveillance (GO:0106354) | 44567 | 0.519094735 | 0.999994883 |
| xenobiotic transport across blood-brain barri | 44567 | 0.519094735 | 0.999994883 |
| amino acid transport (GO:0006865) | 18415 | 0.519845256 | 0.999994883 |
| positive regulation of cell morphogenesis inv | 18415 | 0.519845256 | 0.999994883 |
| cardiac myofibril assembly (GO:0055003) | 44607 | 0.527437763 | 0.999994883 |
| cellular response to estrogen stimulus (GO:0 | 44607 | 0.527437763 | 0.999994883 |
| contractile actin filament bundle assembly (G | 44607 | 0.527437763 | 0.999994883 |
| embryonic cranial skeleton morphogenesis ( | 44607 | 0.527437763 | 0.999994883 |
| endoplasmic reticulum tubular network orga | 44607 | 0.527437763 | 0.999994883 |
| establishment of apical/basal cell polarity (G | 44607 | 0.527437763 | 0.999994883 |
| eye photoreceptor cell development (GO:00 | 44607 | 0.527437763 | 0.999994883 |
| glucuronate metabolic process (GO:0019585 | 44607 | 0.527437763 | 0.999994883 |
| histone H3 deacetylation (GO:0070932) | 44607 | 0.527437763 | 0.999994883 |
| histone mRNA catabolic process (GO:007104 | 44607 | 0.527437763 | 0.999994883 |
| integrated stress response signaling (GO:014 | 44607 | 0.527437763 | 0.999994883 |
| lipoxygenase pathway (GO:0019372) | 44607 | 0.527437763 | 0.999994883 |
| macrophage differentiation (GO:0030225) | 44607 | 0.527437763 | 0.999994883 |
| macrophage migration (GO:1905517) | 44607 | 0.527437763 | 0.999994883 |
| membrane raft organization (GO:0031579) | 44607 | 0.527437763 | 0.999994883 |
| mitotic intra-S DNA damage checkpoint signa | 44607 | 0.527437763 | 0.999994883 |
| myoblast differentiation (GO:0045445) | 44607 | 0.527437763 | 0.999994883 |
| negative regulation of blood pressure (GO:0C | 44607 | 0.527437763 | 0.999994883 |
| negative regulation of cellular component m | 44607 | 0.527437763 | 0.999994883 |

| | | | |
|---|---|---|---|
| negative regulation of protein processing (GC | 44607 | 0.527437763 | 0.999994883 |
| nucleobase-containing small molecule catabo | 44607 | 0.527437763 | 0.999994883 |
| positive regulation of acute inflammatory res | 44607 | 0.527437763 | 0.999994883 |
| positive regulation of double-strand break re | 44607 | 0.527437763 | 0.999994883 |
| positive regulation of natural killer cell activa | 44607 | 0.527437763 | 0.999994883 |
| protein homotrimerization (GO:0070207) | 44607 | 0.527437763 | 0.999994883 |
| protein trimerization (GO:0070206) | 44607 | 0.527437763 | 0.999994883 |
| pyrimidine nucleoside metabolic process (GC | 44607 | 0.527437763 | 0.999994883 |
| regulation of aerobic respiration (GO:190371 | 44607 | 0.527437763 | 0.999994883 |
| regulation of amyloid fibril formation (GO:19 | 44607 | 0.527437763 | 0.999994883 |
| regulation of dephosphorylation (GO:003530 | 44607 | 0.527437763 | 0.999994883 |
| regulation of integrin activation (GO:003362 | 44607 | 0.527437763 | 0.999994883 |
| regulation of macrophage differentiation (GC | 44607 | 0.527437763 | 0.999994883 |
| regulation of phospholipase C activity (GO:19 | 44607 | 0.527437763 | 0.999994883 |
| regulation of superoxide anion generation (G | 44607 | 0.527437763 | 0.999994883 |
| regulation of triglyceride biosynthetic proces | 44607 | 0.527437763 | 0.999994883 |
| steroid hormone mediated signaling pathway | 44607 | 0.527437763 | 0.999994883 |
| stress fiber assembly (GO:0043149) | 44607 | 0.527437763 | 0.999994883 |
| superoxide anion generation (GO:0042554) | 44607 | 0.527437763 | 0.999994883 |
| tRNA 5'-end processing (GO:0099116) | 44607 | 0.527437763 | 0.999994883 |
| import into nucleus (GO:0051170) | 28369 | 0.53067704 | 0.999994883 |
| inorganic cation import across plasma memb | 28369 | 0.53067704 | 0.999994883 |
| positive regulation of endothelial cell prolifer | 28369 | 0.53067704 | 0.999994883 |
| axon extension (GO:0048675) | 44644 | 0.531346959 | 0.999994883 |
| carbohydrate biosynthetic process (GO:0016 | 44644 | 0.531346959 | 0.999994883 |
| DNA packaging (GO:0006323) | 44644 | 0.531346959 | 0.999994883 |
| establishment of mitotic spindle orientation ( | 44644 | 0.531346959 | 0.999994883 |
| iron-sulfur cluster assembly (GO:0016226) | 44644 | 0.531346959 | 0.999994883 |
| ncRNA 3'-end processing (GO:0043628) | 44644 | 0.531346959 | 0.999994883 |
| positive regulation of cellular response to tra | 44644 | 0.531346959 | 0.999994883 |
| positive regulation of neuron apoptotic proce | 44644 | 0.531346959 | 0.999994883 |
| positive regulation of transforming growth fa | 44644 | 0.531346959 | 0.999994883 |
| regulation of axon extension involved in axor | 44644 | 0.531346959 | 0.999994883 |
| regulation of cellular response to transformir | 44644 | 0.531346959 | 0.999994883 |
| regulation of vascular endothelial growth fac | 44644 | 0.531346959 | 0.999994883 |
| response to gamma radiation (GO:0010332) | 44644 | 0.531346959 | 0.999994883 |
| regulation of Ras protein signal transduction | 31686 | 0.533639108 | 0.999994883 |
| actin filament bundle assembly (GO:0051017 | 12145 | 0.534512503 | 0.999994883 |
| exonucleolytic catabolism of deadenylated m | 12145 | 0.534512503 | 0.999994883 |
| negative regulation of JNK cascade (GO:0046 | 12145 | 0.534512503 | 0.999994883 |
| negative regulation of smooth muscle cell pro | 12145 | 0.534512503 | 0.999994883 |
| positive regulation of cholesterol transport (( | 12145 | 0.534512503 | 0.999994883 |
| regulation of histone acetylation (GO:003506 | 12145 | 0.534512503 | 0.999994883 |
| heterophilic cell-cell adhesion via plasma me | 15462 | 0.537339813 | 0.999994883 |
| mitochondrial gene expression (GO:0140053 | 15462 | 0.537339813 | 0.999994883 |
| negative regulation of protein catabolic proce | 15462 | 0.537339813 | 0.999994883 |
| inositol lipid-mediated signaling (GO:004801 | 18780 | 0.539944985 | 0.999994883 |

| | | | |
|---|---|---|---|
| mitotic metaphase plate congression (GO:00( | 18780 | 0.539944985 | 0.999994883 |
| negative regulation of transport (GO:005105 | 18780 | 0.539944985 | 0.999994883 |
| negative regulation of blood vessel morphog | 28734 | 0.546868371 | 0.999994883 |
| neuron projection morphogenesis (GO:00488 | 16/140 | 0.547992272 | 0.999994883 |
| protein targeting to membrane (GO:0006612 | 32051 | 0.54895625 | 0.999994883 |
| regulation of JNK cascade (GO:0046328) | 12/105 | 0.552884671 | 0.999994883 |
| organelle disassembly (GO:1903008) | 12510 | 0.558962574 | 0.999994883 |
| phosphatidylinositol 3-kinase signaling (GO:0 | 12510 | 0.558962574 | 0.999994883 |
| regulation of amyloid-beta formation (GO:19 | 12510 | 0.558962574 | 0.999994883 |
| response to steroid hormone (GO:0048545) | 12510 | 0.558962574 | 0.999994883 |
| anion transport (GO:0006820) | 15827 | 0.559052517 | 0.999994883 |
| lipid modification (GO:0030258) | 15827 | 0.559052517 | 0.999994883 |
| positive regulation of axonogenesis (GO:005( | 15827 | 0.559052517 | 0.999994883 |
| positive regulation of leukocyte mediated cyt | 15827 | 0.559052517 | 0.999994883 |
| regulation of cell-substrate adhesion (GO:001 | 15827 | 0.559052517 | 0.999994883 |
| regulation of cytokine production involved in | 15827 | 0.559052517 | 0.999994883 |
| regulation of purine nucleotide metabolic pro | 15827 | 0.559052517 | 0.999994883 |
| substantia nigra development (GO:0021762) | 15827 | 0.559052517 | 0.999994883 |
| negative regulation of cellular biosynthetic pı | 19146 | 0.559670254 | 0.999994883 |
| regulation of intrinsic apoptotic signaling path | 19146 | 0.559670254 | 0.999994883 |
| negative regulation of ion transport (GO:004: | 44645 | 0.559919776 | 0.999994883 |
| organophosphate ester transport (GO:00157 | 44645 | 0.559919776 | 0.999994883 |
| phosphatidylinositol dephosphorylation (GO: | 44645 | 0.559919776 | 0.999994883 |
| positive regulation of release of cytochrome | 44645 | 0.559919776 | 0.999994883 |
| receptor catabolic process (GO:0032801) | 44645 | 0.559919776 | 0.999994883 |
| regulation of protein processing (GO:007061 | 44645 | 0.559919776 | 0.999994883 |
| regulation of proteolysis involved in cellular p | 44645 | 0.559919776 | 0.999994883 |
| regulation of ryanodine-sensitive calcium-rel( | 44645 | 0.559919776 | 0.999994883 |
| O-glycan processing (GO:0016266) | 22463 | 0.560579382 | 0.999994883 |
| pattern recognition receptor signaling pathw | 22463 | 0.560579382 | 0.999994883 |
| axon development (GO:0061564) | 29099 | 0.562838079 | 0.999994883 |
| positive regulation of proteasomal ubiquitin- | 29099 | 0.562838079 | 0.999994883 |
| positive regulation of ubiquitin-dependent pr | 29099 | 0.562838079 | 0.999994883 |
| regulation of cellular response to heat (GO:1! | 29099 | 0.562838079 | 0.999994883 |
| regulation of neuron differentiation (GO:004: | 29099 | 0.562838079 | 0.999994883 |
| B cell activation involved in immune respons( | 44608 | 0.563311222 | 0.999994883 |
| basement membrane organization (GO:0071 | 44608 | 0.563311222 | 0.999994883 |
| cellular response to sterol (GO:0036315) | 44608 | 0.563311222 | 0.999994883 |
| dendritic spine morphogenesis (GO:0060997 | 44608 | 0.563311222 | 0.999994883 |
| glycosphingolipid biosynthetic process (GO:0 | 44608 | 0.563311222 | 0.999994883 |
| histone H2A acetylation (GO:0043968) | 44608 | 0.563311222 | 0.999994883 |
| mesenchyme development (GO:0060485) | 44608 | 0.563311222 | 0.999994883 |
| motor neuron axon guidance (GO:0008045) | 44608 | 0.563311222 | 0.999994883 |
| negative regulation of cell-substrate junction | 44608 | 0.563311222 | 0.999994883 |
| negative regulation of DNA replication (GO:0 | 44608 | 0.563311222 | 0.999994883 |
| negative regulation of focal adhesion assemb | 44608 | 0.563311222 | 0.999994883 |
| negative regulation of histone modification (( | 44608 | 0.563311222 | 0.999994883 |

| | | | |
|---|---|---|---|
| negative regulation of lyase activity (GO:005: | 44608 | 0.563311222 | 0.999994883 |
| neuron projection organization (GO:0106027 | 44608 | 0.563311222 | 0.999994883 |
| oligodendrocyte development (GO:0014003) | 44608 | 0.563311222 | 0.999994883 |
| platelet-derived growth factor receptor signa | 44608 | 0.563311222 | 0.999994883 |
| positive regulation by host of viral transcripti | 44608 | 0.563311222 | 0.999994883 |
| positive regulation of isotype switching (GO:( | 44608 | 0.563311222 | 0.999994883 |
| positive regulation of leukocyte adhesion to v | 44608 | 0.563311222 | 0.999994883 |
| positive regulation of syncytium formation by | 44608 | 0.563311222 | 0.999994883 |
| regulation of amyloid-beta clearance (GO:19( | 44608 | 0.563311222 | 0.999994883 |
| regulation of chromatin silencing (GO:003193 | 44608 | 0.563311222 | 0.999994883 |
| regulation of defense response to virus (GO:( | 44608 | 0.563311222 | 0.999994883 |
| regulation of granulocyte macrophage colony | 44608 | 0.563311222 | 0.999994883 |
| regulation of interleukin-1 production (GO:0( | 44608 | 0.563311222 | 0.999994883 |
| regulation of interleukin-13 production (GO:( | 44608 | 0.563311222 | 0.999994883 |
| regulation of intrinsic apoptotic signaling patl | 44608 | 0.563311222 | 0.999994883 |
| regulation of isotype switching (GO:0045191 | 44608 | 0.563311222 | 0.999994883 |
| regulation of leukocyte activation (GO:00026 | 44608 | 0.563311222 | 0.999994883 |
| regulation of mast cell degranulation (GO:004 | 44608 | 0.563311222 | 0.999994883 |
| regulation of neurotransmitter transport (GC | 44608 | 0.563311222 | 0.999994883 |
| regulation of oxidative phosphorylation (GO: | 44608 | 0.563311222 | 0.999994883 |
| regulation of Rac protein signal transduction | 44608 | 0.563311222 | 0.999994883 |
| retrograde axonal transport (GO:0008090) | 44608 | 0.563311222 | 0.999994883 |
| transepithelial transport (GO:0070633) | 44608 | 0.563311222 | 0.999994883 |
| positive regulation of cold-induced thermoge | 35735 | 0.565354285 | 0.999994883 |
| negative regulation of programmed cell deat | 43/381 | 0.573257518 | 0.999994883 |
| acylglycerol acyl-chain remodeling (GO:0036: | 44568 | 0.574343152 | 0.999994883 |
| adenylate cyclase-activating dopamine recep | 44568 | 0.574343152 | 0.999994883 |
| angiotensin-activated signaling pathway (GO: | 44568 | 0.574343152 | 0.999994883 |
| arginine catabolic process (GO:0006527) | 44568 | 0.574343152 | 0.999994883 |
| aspartate transmembrane transport (GO:001 | 44568 | 0.574343152 | 0.999994883 |
| atrioventricular valve formation (GO:00031900 | 44568 | 0.574343152 | 0.999994883 |
| axon ensheathment in central nervous syster | 44568 | 0.574343152 | 0.999994883 |
| calcitonin family receptor signaling pathway ( | 44568 | 0.574343152 | 0.999994883 |
| calcium ion export (GO:1901660) | 44568 | 0.574343152 | 0.999994883 |
| catecholamine catabolic process (GO:004242 | 44568 | 0.574343152 | 0.999994883 |
| catecholamine uptake (GO:0090493) | 44568 | 0.574343152 | 0.999994883 |
| Cdc42 protein signal transduction (GO:00324 | 44568 | 0.574343152 | 0.999994883 |
| cellular amino acid biosynthetic process (GO: | 44568 | 0.574343152 | 0.999994883 |
| cellular protein complex disassembly (GO:004 | 44568 | 0.574343152 | 0.999994883 |
| cellular response to laminar fluid shear stress | 44568 | 0.574343152 | 0.999994883 |
| cellular response to monoamine stimulus (G( | 44568 | 0.574343152 | 0.999994883 |
| cellular response to platelet-derived growth f | 44568 | 0.574343152 | 0.999994883 |
| citrulline metabolic process (GO:0000052) | 44568 | 0.574343152 | 0.999994883 |
| collagen-activated tyrosine kinase receptor si | 44568 | 0.574343152 | 0.999994883 |
| coronary vasculature development (GO:0060 | 44568 | 0.574343152 | 0.999994883 |
| creatine metabolic process (GO:0006600) | 44568 | 0.574343152 | 0.999994883 |
| cytochrome complex assembly (GO:0017004 | 44568 | 0.574343152 | 0.999994883 |

| | | | |
|---|---|---|---|
| definitive hemopoiesis (GO:0060216) | 44568 | 0.574343152 | 0.999994883 |
| dendrite arborization (GO:0140059) | 44568 | 0.574343152 | 0.999994883 |
| dense core granule cytoskeletal transport (G( | 44568 | 0.574343152 | 0.999994883 |
| desmosome organization (GO:0002934) | 44568 | 0.574343152 | 0.999994883 |
| determination of digestive tract left/right asy | 44568 | 0.574343152 | 0.999994883 |
| DNA ligation (GO:0006266) | 44568 | 0.574343152 | 0.999994883 |
| dolichyl diphosphate biosynthetic process (G | 44568 | 0.574343152 | 0.999994883 |
| dolichyl diphosphate metabolic process (GO: | 44568 | 0.574343152 | 0.999994883 |
| dopamine transport (GO:0015872) | 44568 | 0.574343152 | 0.999994883 |
| endosome to lysosome transport via multive | 44568 | 0.574343152 | 0.999994883 |
| ethanol catabolic process (GO:0006068) | 44568 | 0.574343152 | 0.999994883 |
| excitatory chemical synaptic transmission (G( | 44568 | 0.574343152 | 0.999994883 |
| glial cell migration (GO:0008347) | 44568 | 0.574343152 | 0.999994883 |
| glomerular visceral epithelial cell developme | 44568 | 0.574343152 | 0.999994883 |
| glutamine transport (GO:0006868) | 44568 | 0.574343152 | 0.999994883 |
| glycosylceramide biosynthetic process (GO:0 | 44568 | 0.574343152 | 0.999994883 |
| Golgi disassembly (GO:0090166) | 44568 | 0.574343152 | 0.999994883 |
| heart trabecula formation (GO:0060347) | 44568 | 0.574343152 | 0.999994883 |
| histone H3-K4 dimethylation (GO:0044648) | 44568 | 0.574343152 | 0.999994883 |
| histone H3-K9 methylation (GO:0051567) | 44568 | 0.574343152 | 0.999994883 |
| hyaluronan biosynthetic process (GO:003021 | 44568 | 0.574343152 | 0.999994883 |
| hyperosmotic response (GO:0006972) | 44568 | 0.574343152 | 0.999994883 |
| hypotonic response (GO:0006971) | 44568 | 0.574343152 | 0.999994883 |
| insulin secretion involved in cellular response | 44568 | 0.574343152 | 0.999994883 |
| intermembrane lipid transfer (GO:0120009) | 44568 | 0.574343152 | 0.999994883 |
| iron coordination entity transport (GO:19016 | 44568 | 0.574343152 | 0.999994883 |
| iron import into cell (GO:0033212) | 44568 | 0.574343152 | 0.999994883 |
| L-alanine transport (GO:0015808) | 44568 | 0.574343152 | 0.999994883 |
| lipoxin biosynthetic process (GO:2001301) | 44568 | 0.574343152 | 0.999994883 |
| long-term synaptic depression (GO:0060292) | 44568 | 0.574343152 | 0.999994883 |
| macropinocytosis (GO:0044351) | 44568 | 0.574343152 | 0.999994883 |
| maintenance of protein location in extracellu | 44568 | 0.574343152 | 0.999994883 |
| malate metabolic process (GO:0006108) | 44568 | 0.574343152 | 0.999994883 |
| membrane biogenesis (GO:0044091) | 44568 | 0.574343152 | 0.999994883 |
| metanephric nephron tubule development (( | 44568 | 0.574343152 | 0.999994883 |
| miRNA catabolic process (GO:0010587) | 44568 | 0.574343152 | 0.999994883 |
| mitochondrion-endoplasmic reticulum memb | 44568 | 0.574343152 | 0.999994883 |
| modulation by symbiont of host defense resp | 44568 | 0.574343152 | 0.999994883 |
| negative regulation by host of viral genome r | 44568 | 0.574343152 | 0.999994883 |
| negative regulation of acute inflammatory re | 44568 | 0.574343152 | 0.999994883 |
| negative regulation of DNA repair (GO:00457 | 44568 | 0.574343152 | 0.999994883 |
| negative regulation of double-strand break re | 44568 | 0.574343152 | 0.999994883 |
| negative regulation of glycogen metabolic pr | 44568 | 0.574343152 | 0.999994883 |
| negative regulation of interferon-gamma-me | 44568 | 0.574343152 | 0.999994883 |
| negative regulation of meiotic nuclear divisio | 44568 | 0.574343152 | 0.999994883 |
| negative regulation of membrane protein ect | 44568 | 0.574343152 | 0.999994883 |
| negative regulation of mitochondrial outer m | 44568 | 0.574343152 | 0.999994883 |

| | | | |
|---|---|---|---|
| negative regulation of MyD88-independent t | 44568 | 0.574343152 | 0.999994883 |
| negative regulation of NLRP3 inflammasome | 44568 | 0.574343152 | 0.999994883 |
| negative regulation of p38MAPK cascade (GC | 44568 | 0.574343152 | 0.999994883 |
| negative regulation of protein kinase activity | 44568 | 0.574343152 | 0.999994883 |
| negative regulation of protein sumoylation (( | 44568 | 0.574343152 | 0.999994883 |
| negative regulation of response to interferon | 44568 | 0.574343152 | 0.999994883 |
| negative regulation of steroid metabolic proc | 44568 | 0.574343152 | 0.999994883 |
| negative regulation of stress-activated protei | 44568 | 0.574343152 | 0.999994883 |
| negative regulation of striated muscle tissue | 44568 | 0.574343152 | 0.999994883 |
| negative regulation of telomere capping (GO: | 44568 | 0.574343152 | 0.999994883 |
| nodal signaling pathway (GO:0038092) | 44568 | 0.574343152 | 0.999994883 |
| nuclear-transcribed mRNA catabolic process, | 44568 | 0.574343152 | 0.999994883 |
| nucleobase catabolic process (GO:0046113) | 44568 | 0.574343152 | 0.999994883 |
| pharyngeal arch artery morphogenesis (GO:0 | 44568 | 0.574343152 | 0.999994883 |
| polyamine biosynthetic process (GO:000659( | 44568 | 0.574343152 | 0.999994883 |
| positive regulation by symbiont of host autop | 44568 | 0.574343152 | 0.999994883 |
| positive regulation of amyloid-beta clearance | 44568 | 0.574343152 | 0.999994883 |
| positive regulation of B cell receptor signaling | 44568 | 0.574343152 | 0.999994883 |
| positive regulation of blood circulation (GO:1 | 44568 | 0.574343152 | 0.999994883 |
| positive regulation of chemokine (C-X-C moti | 44568 | 0.574343152 | 0.999994883 |
| positive regulation of cilium movement (GO:( | 44568 | 0.574343152 | 0.999994883 |
| positive regulation of cilium-dependent cell r | 44568 | 0.574343152 | 0.999994883 |
| positive regulation of ER-associated ubiquitin | 44568 | 0.574343152 | 0.999994883 |
| positive regulation of fibroblast apoptotic pr( | 44568 | 0.574343152 | 0.999994883 |
| positive regulation of flagellated sperm motil | 44568 | 0.574343152 | 0.999994883 |
| positive regulation of gastrulation (GO:20005 | 44568 | 0.574343152 | 0.999994883 |
| positive regulation of histone H4 acetylation | 44568 | 0.574343152 | 0.999994883 |
| positive regulation of icosanoid secretion (G( | 44568 | 0.574343152 | 0.999994883 |
| positive regulation of lamellipodium morpho, | 44568 | 0.574343152 | 0.999994883 |
| positive regulation of mast cell activation (G( | 44568 | 0.574343152 | 0.999994883 |
| positive regulation of membrane depolarizati | 44568 | 0.574343152 | 0.999994883 |
| positive regulation of microtubule nucleation | 44568 | 0.574343152 | 0.999994883 |
| positive regulation of phospholipid biosynthe | 44568 | 0.574343152 | 0.999994883 |
| positive regulation of pinocytosis (GO:00485 | 44568 | 0.574343152 | 0.999994883 |
| positive regulation of protein localization to ( | 44568 | 0.574343152 | 0.999994883 |
| positive regulation of receptor catabolic proc | 44568 | 0.574343152 | 0.999994883 |
| positive regulation of RNA polymerase II tran | 44568 | 0.574343152 | 0.999994883 |
| positive regulation of trophoblast cell migrati | 44568 | 0.574343152 | 0.999994883 |
| positive regulation of vesicle fusion (GO:0031 | 44568 | 0.574343152 | 0.999994883 |
| positive regulation of voltage-gated calcium ( | 44568 | 0.574343152 | 0.999994883 |
| protein localization to cytoplasmic stress gra | 44568 | 0.574343152 | 0.999994883 |
| regulation of cardiac muscle cell membrane p | 44568 | 0.574343152 | 0.999994883 |
| regulation of cilium beat frequency (GO:0003 | 44568 | 0.574343152 | 0.999994883 |
| regulation of entry of bacterium into host cel | 44568 | 0.574343152 | 0.999994883 |
| regulation of extracellular matrix assembly (( | 44568 | 0.574343152 | 0.999994883 |
| regulation of glial cell differentiation (GO:004 | 44568 | 0.574343152 | 0.999994883 |
| regulation of histone deacetylase activity (GC | 44568 | 0.574343152 | 0.999994883 |

| | | | |
|---|---|---|---|
| regulation of ketone biosynthetic process (GO | 44568 | 0.574343152 | 0.999994883 |
| regulation of male gonad development (GO:2 | 44568 | 0.574343152 | 0.999994883 |
| regulation of microglial cell migration (GO:19 | 44568 | 0.574343152 | 0.999994883 |
| regulation of NAD(P)H oxidase activity (GO:0 | 44568 | 0.574343152 | 0.999994883 |
| regulation of natural killer cell mediated cyto | 44568 | 0.574343152 | 0.999994883 |
| regulation of non-membrane spanning prote | 44568 | 0.574343152 | 0.999994883 |
| regulation of phospholipid transport (GO:200 | 44568 | 0.574343152 | 0.999994883 |
| regulation of potassium ion export across pla | 44568 | 0.574343152 | 0.999994883 |
| regulation of T cell mediated immune respon | 44568 | 0.574343152 | 0.999994883 |
| regulation of transforming growth factor bet | 44568 | 0.574343152 | 0.999994883 |
| regulation of transforming growth factor bet | 44568 | 0.574343152 | 0.999994883 |
| regulation of vesicle size (GO:0097494) | 44568 | 0.574343152 | 0.999994883 |
| response to alkaloid (GO:0043279) | 44568 | 0.574343152 | 0.999994883 |
| response to host defenses (GO:0052200) | 44568 | 0.574343152 | 0.999994883 |
| response to increased oxygen levels (GO:003 | 44568 | 0.574343152 | 0.999994883 |
| response to lead ion (GO:0010288) | 44568 | 0.574343152 | 0.999994883 |
| retinal rod cell development (GO:0046548) | 44568 | 0.574343152 | 0.999994883 |
| ribosomal large subunit export from nucleus | 44568 | 0.574343152 | 0.999994883 |
| ribosomal small subunit export from nucleus | 44568 | 0.574343152 | 0.999994883 |
| rRNA-containing ribonucleoprotein complex | 44568 | 0.574343152 | 0.999994883 |
| selenocysteine incorporation (GO:0001514) | 44568 | 0.574343152 | 0.999994883 |
| smooth muscle cell migration (GO:0014909) | 44568 | 0.574343152 | 0.999994883 |
| somatic recombination of immunoglobulin ge | 44568 | 0.574343152 | 0.999994883 |
| substrate-dependent cell migration, cell exte | 44568 | 0.574343152 | 0.999994883 |
| succinate transport (GO:0015744) | 44568 | 0.574343152 | 0.999994883 |
| synaptic vesicle budding from presynaptic en | 44568 | 0.574343152 | 0.999994883 |
| tachykinin receptor signaling pathway (GO:0 | 44568 | 0.574343152 | 0.999994883 |
| translational readthrough (GO:0006451) | 44568 | 0.574343152 | 0.999994883 |
| vitamin A metabolic process (GO:0006776) | 44568 | 0.574343152 | 0.999994883 |
| white fat cell differentiation (GO:0050872) | 44568 | 0.574343152 | 0.999994883 |
| phosphatidylinositol-mediated signaling (GO: | 26146 | 0.578353792 | 0.999994883 |
| negative regulation of cell cycle (GO:0045786 | 29465 | 0.578566343 | 0.999994883 |
| positive regulation of cell adhesion (GO:0045 | 29465 | 0.578566343 | 0.999994883 |
| positive regulation of macroautophagy (GO:0 | 19511 | 0.578987533 | 0.999994883 |
| regulation of endothelial cell migration (GO:0 | 32782 | 0.578989006 | 0.999994883 |
| response to calcium ion (GO:0051592) | 12/107 | 0.580242559 | 0.999994883 |
| embryonic organ morphogenesis (GO:00485 | 16193 | 0.580242768 | 0.999994883 |
| intracellular protein transmembrane transpo | 16193 | 0.580242768 | 0.999994883 |
| mRNA catabolic process (GO:0006402) | 16193 | 0.580242768 | 0.999994883 |
| positive regulation of protein modification pr | 24/214 | 0.581731531 | 0.999994883 |
| regulation of phosphorylation (GO:0042325) | 14/125 | 0.581825163 | 0.999994883 |
| blood vessel diameter maintenance (GO:009 | 12875 | 0.582699068 | 0.999994883 |
| embryonic skeletal system development (GO | 12875 | 0.582699068 | 0.999994883 |
| muscle cell differentiation (GO:0042692) | 12875 | 0.582699068 | 0.999994883 |
| nuclear-transcribed mRNA catabolic process, | 12875 | 0.582699068 | 0.999994883 |
| positive regulation of lipid biosynthetic proce | 12875 | 0.582699068 | 0.999994883 |
| regulation of JUN kinase activity (GO:004350 | 12875 | 0.582699068 | 0.999994883 |

| | | | |
|---|---|---|---|
| regulation of lipid biosynthetic process (GO:0 | 12875 | 0.582699068 | 0.999994883 |
| response to estrogen (GO:0043627) | 12875 | 0.582699068 | 0.999994883 |
| semaphorin-plexin signaling pathway (GO:00 | 12875 | 0.582699068 | 0.999994883 |
| negative regulation of protein localization to | 44646 | 0.587410441 | 0.999994883 |
| negative regulation of signaling receptor acti | 44646 | 0.587410441 | 0.999994883 |
| phosphatidylethanolamine acyl-chain remod | 44646 | 0.587410441 | 0.999994883 |
| positive regulation of histone methylation (G | 44646 | 0.587410441 | 0.999994883 |
| positive regulation of ion transmembrane tra | 44646 | 0.587410441 | 0.999994883 |
| positive regulation of microtubule polymeriz | 44646 | 0.587410441 | 0.999994883 |
| regulation of actin filament bundle assembly | 44646 | 0.587410441 | 0.999994883 |
| regulation of biomineral tissue development | 44646 | 0.587410441 | 0.999994883 |
| regulation of glycogen biosynthetic process ( | 44646 | 0.587410441 | 0.999994883 |
| regulation of telomere maintenance (GO:003 | 44646 | 0.587410441 | 0.999994883 |
| snRNA processing (GO:0016180) | 44646 | 0.587410441 | 0.999994883 |
| TOR signaling (GO:0031929) | 44646 | 0.587410441 | 0.999994883 |
| positive regulation of stress-activated MAPK | 36465 | 0.593565601 | 0.999994883 |
| negative regulation of gene expression (GO:0 | 36/322 | 0.594998027 | 0.999994883 |
| COPII vesicle coating (GO:0048208) | 23193 | 0.595951349 | 0.999994883 |
| positive regulation of viral process (GO:0048 | 23193 | 0.595951349 | 0.999994883 |
| vesicle coating (GO:0006901) | 23193 | 0.595951349 | 0.999994883 |
| vesicle targeting, rough ER to cis-Golgi (GO:0 | 23193 | 0.595951349 | 0.999994883 |
| cellular modified amino acid catabolic proces | 44609 | 0.597181815 | 0.999994883 |
| cellular response to fatty acid (GO:0071398) | 44609 | 0.597181815 | 0.999994883 |
| detection of visible light (GO:0009584) | 44609 | 0.597181815 | 0.999994883 |
| epithelial cell morphogenesis (GO:0003382) | 44609 | 0.597181815 | 0.999994883 |
| mitochondrial ATP synthesis coupled proton | 44609 | 0.597181815 | 0.999994883 |
| negative regulation of ATP metabolic process | 44609 | 0.597181815 | 0.999994883 |
| negative regulation of chemokine production | 44609 | 0.597181815 | 0.999994883 |
| negative regulation of execution phase of ap | 44609 | 0.597181815 | 0.999994883 |
| negative regulation of intrinsic apoptotic sign | 44609 | 0.597181815 | 0.999994883 |
| negative regulation of phagocytosis (GO:005 | 44609 | 0.597181815 | 0.999994883 |
| phosphate ion transport (GO:0006817) | 44609 | 0.597181815 | 0.999994883 |
| positive regulation of dendrite extension (GC | 44609 | 0.597181815 | 0.999994883 |
| positive regulation of DNA replication (GO:00 | 44609 | 0.597181815 | 0.999994883 |
| positive regulation of macrophage activation | 44609 | 0.597181815 | 0.999994883 |
| positive regulation of regulated secretory pat | 44609 | 0.597181815 | 0.999994883 |
| positive regulation of smooth muscle cell mig | 44609 | 0.597181815 | 0.999994883 |
| pyrimidine-containing compound catabolic p | 44609 | 0.597181815 | 0.999994883 |
| regulation of Arp2/3 complex-mediated actir | 44609 | 0.597181815 | 0.999994883 |
| regulation of cardiac muscle contraction by r | 44609 | 0.597181815 | 0.999994883 |
| regulation of cellular respiration (GO:004345 | 44609 | 0.597181815 | 0.999994883 |
| regulation of leukocyte migration (GO:00026 | 44609 | 0.597181815 | 0.999994883 |
| regulation of metal ion transport (GO:001095 | 44609 | 0.597181815 | 0.999994883 |
| regulation of NLRP3 inflammasome complex | 44609 | 0.597181815 | 0.999994883 |
| ribosomal small subunit assembly (GO:00000 | 44609 | 0.597181815 | 0.999994883 |
| snRNA metabolic process (GO:0016073) | 44609 | 0.597181815 | 0.999994883 |
| synaptic vesicle membrane organization (GO: | 44609 | 0.597181815 | 0.999994883 |

| | | | |
|---|---|---|---|
| inorganic anion transport (GO:0015698) | 19876 | 0.597867242 | 0.999994883 |
| regulation of focal adhesion assembly (GO:0( | 19876 | 0.597867242 | 0.999994883 |
| regulation of potassium ion transmembrane | 19876 | 0.597867242 | 0.999994883 |
| regulation of proteolysis (GO:0030162) | 19876 | 0.597867242 | 0.999994883 |
| regulation of GTPase activity (GO:0043087) | 21/189 | 0.598483373 | 0.999994883 |
| extracellular structure organization (GO:0043 | 24/216 | 0.600754674 | 0.999994883 |
| positive regulation of chemotaxis (GO:00509 | 16558 | 0.600874911 | 0.999994883 |
| regulation of insulin receptor signaling pathw | 16558 | 0.600874911 | 0.999994883 |
| zymogen activation (GO:0031638) | 16558 | 0.600874911 | 0.999994883 |
| axonal transport (GO:0098930) | 13241 | 0.605679118 | 0.999994883 |
| cyclic-nucleotide-mediated signaling (GO:001 | 13241 | 0.605679118 | 0.999994883 |
| dendrite morphogenesis (GO:0048813) | 13241 | 0.605679118 | 0.999994883 |
| embryonic limb morphogenesis (GO:003032( | 13241 | 0.605679118 | 0.999994883 |
| endoderm formation (GO:0001706) | 13241 | 0.605679118 | 0.999994883 |
| regulation of microtubule-based process (GO | 13241 | 0.605679118 | 0.999994883 |
| regulation of osteoclast differentiation (GO:0 | 13241 | 0.605679118 | 0.999994883 |
| regulation of actin cytoskeleton organization | 33512 | 0.608111515 | 0.999994883 |
| positive regulation of protein ubiquitination ( | 30195 | 0.609227174 | 0.999994883 |
| external encapsulating structure organizatior | 24/217 | 0.610134822 | 0.999994883 |
| antigen processing and presentation of exoge | 26877 | 0.61082226 | 0.999994883 |
| insulin receptor signaling pathway (GO:0008 | 26877 | 0.61082226 | 0.999994883 |
| positive regulation of JNK cascade (GO:0046 | 26877 | 0.61082226 | 0.999994883 |
| protein secretion (GO:0009306) | 23559 | 0.613078792 | 0.999994883 |
| calcium-ion regulated exocytosis (GO:00171! | 44647 | 0.613771602 | 0.999994883 |
| chondroitin sulfate proteoglycan biosyntheti | 44647 | 0.613771602 | 0.999994883 |
| entrainment of circadian clock by photoperio | 44647 | 0.613771602 | 0.999994883 |
| melanosome organization (GO:0032438) | 44647 | 0.613771602 | 0.999994883 |
| microtubule polymerization or depolymeriza | 44647 | 0.613771602 | 0.999994883 |
| peptide hormone secretion (GO:0030072) | 44647 | 0.613771602 | 0.999994883 |
| positive regulation of cellular component mo | 44647 | 0.613771602 | 0.999994883 |
| positive regulation of nuclear division (GO:00 | 44647 | 0.613771602 | 0.999994883 |
| positive regulation of toll-like receptor signal | 44647 | 0.613771602 | 0.999994883 |
| protein export from nucleus (GO:0006611) | 44647 | 0.613771602 | 0.999994883 |
| regulation of toll-like receptor signaling path | 44647 | 0.613771602 | 0.999994883 |
| regulation of transcription regulatory region | 44647 | 0.613771602 | 0.999994883 |
| regulation of vasculature development (GO:1 | 44647 | 0.613771602 | 0.999994883 |
| negative regulation of cysteine-type endope | 20241 | 0.616283647 | 0.999994883 |
| protein targeting to mitochondrion (GO:000€ | 20241 | 0.616283647 | 0.999994883 |
| regulation of cardiac conduction (GO:190377 | 20241 | 0.616283647 | 0.999994883 |
| negative regulation of Wnt signaling pathway | 21/191 | 0.618431328 | 0.999994883 |
| negative regulation of apoptotic process (GO | 54/485 | 0.618478677 | 0.999994883 |
| establishment of protein localization to extra | 16923 | 0.620918971 | 0.999994883 |
| positive regulation of smooth muscle cell pro | 16923 | 0.620918971 | 0.999994883 |
| regulation of DNA metabolic process (GO:00! | 16923 | 0.620918971 | 0.999994883 |
| regulation of endothelial cell proliferation (G | 33878 | 0.622295557 | 0.999994883 |
| ammonium transmembrane transport (GO:0( | 44569 | 0.623246824 | 0.999994883 |
| anterograde axonal protein transport (GO:00 | 44569 | 0.623246824 | 0.999994883 |

| | | | |
|---|---|---|---|
| antigen processing and presentation of endo; | 44569 | 0.623246824 | 0.999994883 |
| aromatic compound biosynthetic process (G( | 44569 | 0.623246824 | 0.999994883 |
| autophagosome-lysosome fusion (GO:00619( | 44569 | 0.623246824 | 0.999994883 |
| axo-dendritic protein transport (GO:0099640 | 44569 | 0.623246824 | 0.999994883 |
| axon extension involved in axon guidance (G( | 44569 | 0.623246824 | 0.999994883 |
| basic amino acid transport (GO:0015802) | 44569 | 0.623246824 | 0.999994883 |
| cell migration involved in heart development | 44569 | 0.623246824 | 0.999994883 |
| cellular response to angiotensin (GO:190438! | 44569 | 0.623246824 | 0.999994883 |
| cellular response to interferon-alpha (GO:003 | 44569 | 0.623246824 | 0.999994883 |
| cellular response to potassium ion (GO:0035( | 44569 | 0.623246824 | 0.999994883 |
| cellular response to UV-B (GO:0071493) | 44569 | 0.623246824 | 0.999994883 |
| cysteine metabolic process (GO:0006534) | 44569 | 0.623246824 | 0.999994883 |
| cytoplasmic sequestering of NF-kappaB (GO:( | 44569 | 0.623246824 | 0.999994883 |
| dopamine uptake (GO:0090494) | 44569 | 0.623246824 | 0.999994883 |
| dynamin family protein polymerization involv | 44569 | 0.623246824 | 0.999994883 |
| dynamin family protein polymerization involv | 44569 | 0.623246824 | 0.999994883 |
| establishment of Golgi localization (GO:0051( | 44569 | 0.623246824 | 0.999994883 |
| excitatory synapse assembly (GO:1904861) | 44569 | 0.623246824 | 0.999994883 |
| fusion of virus membrane with host plasma n | 44569 | 0.623246824 | 0.999994883 |
| galactosylceramide metabolic process (GO:0( | 44569 | 0.623246824 | 0.999994883 |
| histidine catabolic process (GO:0006548) | 44569 | 0.623246824 | 0.999994883 |
| histidine metabolic process (GO:0006547) | 44569 | 0.623246824 | 0.999994883 |
| histone H3-K4 demethylation (GO:0034720) | 44569 | 0.623246824 | 0.999994883 |
| histone H3-K4 monomethylation (GO:00976! | 44569 | 0.623246824 | 0.999994883 |
| immunological synapse formation (GO:00017 | 44569 | 0.623246824 | 0.999994883 |
| internal protein amino acid acetylation (GO:( | 44569 | 0.623246824 | 0.999994883 |
| kidney mesenchyme development (GO:0072( | 44569 | 0.623246824 | 0.999994883 |
| mammary gland epithelial cell differentiation | 44569 | 0.623246824 | 0.999994883 |
| meiotic sister chromatid cohesion (GO:00511 | 44569 | 0.623246824 | 0.999994883 |
| membrane fusion involved in viral entry into | 44569 | 0.623246824 | 0.999994883 |
| mitochondrial respiratory chain complex III a | 44569 | 0.623246824 | 0.999994883 |
| mitophagy (GO:0000423) | 44569 | 0.623246824 | 0.999994883 |
| monoamine transport (GO:0015844) | 44569 | 0.623246824 | 0.999994883 |
| negative regulation of alpha-beta T cell differ | 44569 | 0.623246824 | 0.999994883 |
| negative regulation of CD4-positive, alpha-be | 44569 | 0.623246824 | 0.999994883 |
| negative regulation of fatty acid oxidation (G | 44569 | 0.623246824 | 0.999994883 |
| negative regulation of microglial cell activatic | 44569 | 0.623246824 | 0.999994883 |
| negative regulation of mitochondrial membra | 44569 | 0.623246824 | 0.999994883 |
| negative regulation of muscle cell apoptotic p | 44569 | 0.623246824 | 0.999994883 |
| negative regulation of nitrogen compound m | 44569 | 0.623246824 | 0.999994883 |
| negative regulation of osteoblast proliferatio | 44569 | 0.623246824 | 0.999994883 |
| negative regulation of phospholipase activity | 44569 | 0.623246824 | 0.999994883 |
| negative regulation of potassium ion transme | 44569 | 0.623246824 | 0.999994883 |
| negative regulation of T-helper 17 cell differe | 44569 | 0.623246824 | 0.999994883 |
| neuron projection extension involved in neur | 44569 | 0.623246824 | 0.999994883 |
| neurotransmitter receptor diffusion trapping | 44569 | 0.623246824 | 0.999994883 |
| neurotransmitter reuptake (GO:0098810) | 44569 | 0.623246824 | 0.999994883 |

| | | | |
|---|---|---|---|
| osteoblast development (GO:0002076) | 44569 | 0.623246824 | 0.999994883 |
| peptidyl-tyrosine dephosphorylation involved | 44569 | 0.623246824 | 0.999994883 |
| plasma membrane phospholipid scrambling ( | 44569 | 0.623246824 | 0.999994883 |
| positive regulation of apoptotic cell clearance | 44569 | 0.623246824 | 0.999994883 |
| positive regulation of aspartic-type peptidase | 44569 | 0.623246824 | 0.999994883 |
| positive regulation of CD4-positive, alpha-bet | 44569 | 0.623246824 | 0.999994883 |
| positive regulation of defense response to ba | 44569 | 0.623246824 | 0.999994883 |
| positive regulation of dendritic cell chemotax | 44569 | 0.623246824 | 0.999994883 |
| positive regulation of dendritic cell cytokine p | 44569 | 0.623246824 | 0.999994883 |
| positive regulation of early endosome to late | 44569 | 0.623246824 | 0.999994883 |
| positive regulation of extracellular matrix dis | 44569 | 0.623246824 | 0.999994883 |
| positive regulation of glial cell migration (GO: | 44569 | 0.623246824 | 0.999994883 |
| positive regulation of lipoprotein lipase activi | 44569 | 0.623246824 | 0.999994883 |
| positive regulation of meiotic nuclear divisior | 44569 | 0.623246824 | 0.999994883 |
| positive regulation of monocyte chemotactic | 44569 | 0.623246824 | 0.999994883 |
| positive regulation of myotube differentiatio | 44569 | 0.623246824 | 0.999994883 |
| positive regulation of oligodendrocyte differe | 44569 | 0.623246824 | 0.999994883 |
| positive regulation of peptide secretion (GO:( | 44569 | 0.623246824 | 0.999994883 |
| positive regulation of ryanodine-sensitive cal | 44569 | 0.623246824 | 0.999994883 |
| positive regulation of steroid biosynthetic pr | 44569 | 0.623246824 | 0.999994883 |
| positive regulation of T-helper 2 cell cytokine | 44569 | 0.623246824 | 0.999994883 |
| positive regulation of vascular associated sm | 44569 | 0.623246824 | 0.999994883 |
| postsynaptic neurotransmitter receptor diffu | 44569 | 0.623246824 | 0.999994883 |
| protein deglycosylation (GO:0006517) | 44569 | 0.623246824 | 0.999994883 |
| protein localization to microtubule (GO:0035 | 44569 | 0.623246824 | 0.999994883 |
| protein localization to microtubule cytoskele | 44569 | 0.623246824 | 0.999994883 |
| protein localization to presynapse (GO:19053 | 44569 | 0.623246824 | 0.999994883 |
| protein retention in ER lumen (GO:0006621) | 44569 | 0.623246824 | 0.999994883 |
| regulation of actin filament length (GO:0030 | 44569 | 0.623246824 | 0.999994883 |
| regulation of alpha-beta T cell differentiation | 44569 | 0.623246824 | 0.999994883 |
| regulation of alpha-beta T cell proliferation (( | 44569 | 0.623246824 | 0.999994883 |
| regulation of apoptotic cell clearance (GO:20 | 44569 | 0.623246824 | 0.999994883 |
| regulation of ATPase-coupled calcium transm | 44569 | 0.623246824 | 0.999994883 |
| regulation of cardiac muscle cell action poter | 44569 | 0.623246824 | 0.999994883 |
| regulation of CD4-positive, alpha-beta T cell a | 44569 | 0.623246824 | 0.999994883 |
| regulation of Cdc42 protein signal transducti | 44569 | 0.623246824 | 0.999994883 |
| regulation of chemokine-mediated signaling | 44569 | 0.623246824 | 0.999994883 |
| regulation of cytoplasmic translational elong | 44569 | 0.623246824 | 0.999994883 |
| regulation of dendritic cell chemotaxis (GO:2 | 44569 | 0.623246824 | 0.999994883 |
| regulation of dopamine receptor signaling pa | 44569 | 0.623246824 | 0.999994883 |
| regulation of endodeoxyribonuclease activity | 44569 | 0.623246824 | 0.999994883 |
| regulation of interleukin-18 production (GO:( | 44569 | 0.623246824 | 0.999994883 |
| regulation of intestinal cholesterol absorptio | 44569 | 0.623246824 | 0.999994883 |
| regulation of lymphocyte apoptotic process ( | 44569 | 0.623246824 | 0.999994883 |
| regulation of meiotic nuclear division (GO:00 | 44569 | 0.623246824 | 0.999994883 |
| regulation of metaphase/anaphase transitior | 44569 | 0.623246824 | 0.999994883 |
| regulation of microvillus organization (GO:00 | 44569 | 0.623246824 | 0.999994883 |

| | | | |
|---|---|---|---|
| regulation of MyD88-independent toll-like re | 44569 | 0.623246824 | 0.999994883 |
| regulation of myeloid leukocyte differentiatic | 44569 | 0.623246824 | 0.999994883 |
| regulation of pattern recognition receptor sig | 44569 | 0.623246824 | 0.999994883 |
| regulation of phospholipase A2 activity (GO:C | 44569 | 0.623246824 | 0.999994883 |
| regulation of protein K63-linked ubiquitinatic | 44569 | 0.623246824 | 0.999994883 |
| regulation of protein localization to cell corte | 44569 | 0.623246824 | 0.999994883 |
| regulation of proton transport (GO:0010155) | 44569 | 0.623246824 | 0.999994883 |
| regulation of response to wounding (GO:190 | 44569 | 0.623246824 | 0.999994883 |
| regulation of striated muscle tissue developn | 44569 | 0.623246824 | 0.999994883 |
| regulation of translational termination (GO:0 | 44569 | 0.623246824 | 0.999994883 |
| regulation of vascular wound healing (GO:00 | 44569 | 0.623246824 | 0.999994883 |
| regulation of wound healing, spreading of ep | 44569 | 0.623246824 | 0.999994883 |
| respiratory chain complex III assembly (GO:0 | 44569 | 0.623246824 | 0.999994883 |
| response to potassium ion (GO:0035864) | 44569 | 0.623246824 | 0.999994883 |
| RNA secondary structure unwinding (GO:001 | 44569 | 0.623246824 | 0.999994883 |
| rRNA base methylation (GO:0070475) | 44569 | 0.623246824 | 0.999994883 |
| self proteolysis (GO:0097264) | 44569 | 0.623246824 | 0.999994883 |
| sequestering of BMP from receptor via BMP | 44569 | 0.623246824 | 0.999994883 |
| snRNA transcription by RNA polymerase III (G | 44569 | 0.623246824 | 0.999994883 |
| spleen development (GO:0048536) | 44569 | 0.623246824 | 0.999994883 |
| SREBP signaling pathway (GO:0032933) | 44569 | 0.623246824 | 0.999994883 |
| sulfur amino acid biosynthetic process (GO:0 | 44569 | 0.623246824 | 0.999994883 |
| synaptic vesicle budding (GO:0070142) | 44569 | 0.623246824 | 0.999994883 |
| T-helper cell lineage commitment (GO:00022 | 44569 | 0.623246824 | 0.999994883 |
| TORC1 signaling (GO:0038202) | 44569 | 0.623246824 | 0.999994883 |
| U4 snRNA 3'-end processing (GO:0034475) | 44569 | 0.623246824 | 0.999994883 |
| vascular associated smooth muscle contracti | 44569 | 0.623246824 | 0.999994883 |
| wound healing, spreading of epidermal cells ( | 44569 | 0.623246824 | 0.999994883 |
| positive regulation of plasma membrane bou | 30560 | 0.624128015 | 0.999994883 |
| regulation of defense response (GO:0031347 | 30560 | 0.624128015 | 0.999994883 |
| regulation of osteoblast differentiation (GO:C | 30560 | 0.624128015 | 0.999994883 |
| regulation of stress fiber assembly (GO:0051 | 27242 | 0.626558887 | 0.999994883 |
| lysosome localization (GO:0032418) | 13606 | 0.627868903 | 0.999994883 |
| ncRNA metabolic process (GO:0034660) | 13606 | 0.627868903 | 0.999994883 |
| negative regulation of endothelial cell prolife | 13606 | 0.627868903 | 0.999994883 |
| negative regulation of stress-activated MAPK | 13606 | 0.627868903 | 0.999994883 |
| phagocytosis, engulfment (GO:0006911) | 13606 | 0.627868903 | 0.999994883 |
| positive regulation of myeloid cell differentia | 13606 | 0.627868903 | 0.999994883 |
| carbohydrate derivative transport (GO:19012 | 44610 | 0.629036238 | 0.999994883 |
| DNA conformation change (GO:0071103) | 44610 | 0.629036238 | 0.999994883 |
| embryonic axis specification (GO:0000578) | 44610 | 0.629036238 | 0.999994883 |
| glycoprotein catabolic process (GO:0006516) | 44610 | 0.629036238 | 0.999994883 |
| high-density lipoprotein particle remodeling ( | 44610 | 0.629036238 | 0.999994883 |
| intermediate filament organization (GO:0045 | 44610 | 0.629036238 | 0.999994883 |
| mitochondrial fusion (GO:0008053) | 44610 | 0.629036238 | 0.999994883 |
| negative regulation of double-strand break re | 44610 | 0.629036238 | 0.999994883 |
| negative regulation of fibroblast proliferatior | 44610 | 0.629036238 | 0.999994883 |

| | | | |
|---|---|---|---|
| negative regulation of interleukin-8 productic | 44610 | 0.629036238 | 0.999994883 |
| negative regulation of lipid catabolic process | 44610 | 0.629036238 | 0.999994883 |
| negative regulation of Rho protein signal trar | 44610 | 0.629036238 | 0.999994883 |
| neuron maturation (GO:0042551) | 44610 | 0.629036238 | 0.999994883 |
| nitric oxide mediated signal transduction (GC | 44610 | 0.629036238 | 0.999994883 |
| positive chemotaxis (GO:0050918) | 44610 | 0.629036238 | 0.999994883 |
| positive regulation of amyloid-beta formatioı | 44610 | 0.629036238 | 0.999994883 |
| positive regulation of cell migration involved | 44610 | 0.629036238 | 0.999994883 |
| positive regulation of excitatory postsynaptic | 44610 | 0.629036238 | 0.999994883 |
| positive regulation of glial cell differentiation | 44610 | 0.629036238 | 0.999994883 |
| positive regulation of macrophage derived fo | 44610 | 0.629036238 | 0.999994883 |
| positive regulation of nervous system proces | 44610 | 0.629036238 | 0.999994883 |
| positive regulation of stress-activated proteir | 44610 | 0.629036238 | 0.999994883 |
| programmed necrotic cell death (GO:009730 | 44610 | 0.629036238 | 0.999994883 |
| regulation of cellular protein catabolic proces | 44610 | 0.629036238 | 0.999994883 |
| regulation of chromosome segregation (GO:( | 44610 | 0.629036238 | 0.999994883 |
| regulation of dendrite extension (GO:190385 | 44610 | 0.629036238 | 0.999994883 |
| regulation of membrane depolarization (GO:( | 44610 | 0.629036238 | 0.999994883 |
| regulation of oxidative stress-induced intrinsi | 44610 | 0.629036238 | 0.999994883 |
| regulation of protein-containing complex disa | 44610 | 0.629036238 | 0.999994883 |
| rRNA catabolic process (GO:0016075) | 44610 | 0.629036238 | 0.999994883 |
| ventricular cardiac muscle cell action potenti | 44610 | 0.629036238 | 0.999994883 |
| regulation of RNA metabolic process (GO:005 | 23924 | 0.629807128 | 0.999994883 |
| tRNA modification (GO:0006400) | 23924 | 0.629807128 | 0.999994883 |
| extracellular matrix organization (GO:003019 | 33/300 | 0.631767688 | 0.999994883 |
| negative regulation of metabolic process (GC | 20607 | 0.634214733 | 0.999994883 |
| calcium-mediated signaling (GO:0019722) | 11/102 | 0.634236003 | 0.999994883 |
| visual perception (GO:0007601) | 34243 | 0.636211501 | 0.999994883 |
| cellular response to acid chemical (GO:00712 | 44648 | 0.638971724 | 0.999994883 |
| histone mRNA metabolic process (GO:00083 | 44648 | 0.638971724 | 0.999994883 |
| keratan sulfate biosynthetic process (GO:001 | 44648 | 0.638971724 | 0.999994883 |
| negative regulation of axonogenesis (GO:005 | 44648 | 0.638971724 | 0.999994883 |
| negative regulation of phosphatase activity (( | 44648 | 0.638971724 | 0.999994883 |
| neutral amino acid transport (GO:0015804) | 44648 | 0.638971724 | 0.999994883 |
| positive regulation of protein import into nuc | 44648 | 0.638971724 | 0.999994883 |
| positive regulation of response to wounding | 44648 | 0.638971724 | 0.999994883 |
| protein depolymerization (GO:0051261) | 44648 | 0.638971724 | 0.999994883 |
| protein localization to cell surface (GO:00343 | 44648 | 0.638971724 | 0.999994883 |
| regulation of B cell activation (GO:0050864) | 44648 | 0.638971724 | 0.999994883 |
| regulation of calcium-mediated signaling (GO | 44648 | 0.638971724 | 0.999994883 |
| regulation of microtubule polymerization or ( | 44648 | 0.638971724 | 0.999994883 |
| histone deacetylation (GO:0016575) | 17288 | 0.640350373 | 0.999994883 |
| negative regulation of cold-induced thermog | 17288 | 0.640350373 | 0.999994883 |
| regulation of cellular metabolic process (GO:( | 17288 | 0.640350373 | 0.999994883 |
| regulation of protein localization to nucleus ( | 17288 | 0.640350373 | 0.999994883 |
| positive regulation of response to external st | 15/139 | 0.641103139 | 0.999994883 |
| integrin-mediated signaling pathway (GO:00( | 27607 | 0.641943024 | 0.999994883 |

| | | | |
|---|---|---|---|
| positive regulation of lymphocyte proliferatio | 27607 | 0.641943024 | 0.999994883 |
| regulation of transcription from RNA polyme | 27607 | 0.641943024 | 0.999994883 |
| cell-cell junction assembly (GO:0007043) | 24289 | 0.646119589 | 0.999994883 |
| positive regulation of T cell proliferation (GO: | 24289 | 0.646119589 | 0.999994883 |
| negative regulation of proteasomal protein c | 13971 | 0.649242942 | 0.999994883 |
| response to cAMP (GO:0051591) | 13971 | 0.649242942 | 0.999994883 |
| response to estradiol (GO:0032355) | 13971 | 0.649242942 | 0.999994883 |
| synaptic vesicle endocytosis (GO:0048488) | 13971 | 0.649242942 | 0.999994883 |
| icosanoid metabolic process (GO:0006690) | 20972 | 0.651642057 | 0.999994883 |
| actomyosin structure organization (GO:0031( | 27973 | 0.656961547 | 0.999994883 |
| ATP synthesis coupled proton transport (GO: | 44611 | 0.658890649 | 0.999994883 |
| carbohydrate derivative catabolic process (G | 44611 | 0.658890649 | 0.999994883 |
| cellular glucuronidation (GO:0052695) | 44611 | 0.658890649 | 0.999994883 |
| DNA alkylation (GO:0006305) | 44611 | 0.658890649 | 0.999994883 |
| DNA methylation or demethylation (GO:004 | 44611 | 0.658890649 | 0.999994883 |
| ethanol metabolic process (GO:0006067) | 44611 | 0.658890649 | 0.999994883 |
| excitatory postsynaptic potential (GO:00600 | 44611 | 0.658890649 | 0.999994883 |
| histone H4-K16 acetylation (GO:0043984) | 44611 | 0.658890649 | 0.999994883 |
| negative regulation of cytokine production in | 44611 | 0.658890649 | 0.999994883 |
| peptidyl-lysine dimethylation (GO:0018027) | 44611 | 0.658890649 | 0.999994883 |
| positive regulation of cilium assembly (GO:0( | 44611 | 0.658890649 | 0.999994883 |
| positive regulation of focal adhesion assembl | 44611 | 0.658890649 | 0.999994883 |
| positive regulation of oxidoreductase activity | 44611 | 0.658890649 | 0.999994883 |
| postsynaptic membrane organization (GO:00 | 44611 | 0.658890649 | 0.999994883 |
| pyroptosis (GO:0070269) | 44611 | 0.658890649 | 0.999994883 |
| regulation of leukocyte chemotaxis (GO:0002 | 44611 | 0.658890649 | 0.999994883 |
| regulation of mitotic spindle assembly (GO:1 | 44611 | 0.658890649 | 0.999994883 |
| regulation of oligodendrocyte differentiation | 44611 | 0.658890649 | 0.999994883 |
| regulation of T cell cytokine production (GO:( | 44611 | 0.658890649 | 0.999994883 |
| response to fatty acid (GO:0070542) | 44611 | 0.658890649 | 0.999994883 |
| synaptic transmission, cholinergic (GO:00072 | 44611 | 0.658890649 | 0.999994883 |
| ureteric bud development (GO:0001657) | 44611 | 0.658890649 | 0.999994883 |
| myelination (GO:0042552) | 17654 | 0.659149626 | 0.999994883 |
| positive regulation of blood vessel endothelia | 17654 | 0.659149626 | 0.999994883 |
| regulation of mitochondrion organization (G( | 17654 | 0.659149626 | 0.999994883 |
| regulation of insulin secretion (GO:0050796) | 11/104 | 0.660123788 | 0.999994883 |
| vascular endothelial growth factor receptor s | 24654 | 0.662001938 | 0.999994883 |
| glial cell differentiation (GO:0010001) | 44649 | 0.662993155 | 0.999994883 |
| intrinsic apoptotic signaling pathway in respo | 44649 | 0.662993155 | 0.999994883 |
| MyD88-dependent toll-like receptor signaling | 44649 | 0.662993155 | 0.999994883 |
| negative regulation of mRNA catabolic proce: | 44649 | 0.662993155 | 0.999994883 |
| negative regulation of wound healing (GO:00 | 44649 | 0.662993155 | 0.999994883 |
| regulation of DNA biosynthetic process (GO:2 | 44649 | 0.662993155 | 0.999994883 |
| regulation of gluconeogenesis (GO:0006111) | 44649 | 0.662993155 | 0.999994883 |
| positive regulation of cellular catabolic proce | 15/141 | 0.663264699 | 0.999994883 |
| actin-mediated cell contraction (GO:0070252 | 44570 | 0.666534103 | 0.999994883 |
| adult heart development (GO:0007512) | 44570 | 0.666534103 | 0.999994883 |

| | | | |
|---|---|---|---|
| androgen biosynthetic process (GO:0006702) | 44570 | 0.666534103 | 0.999994883 |
| associative learning (GO:0008306) | 44570 | 0.666534103 | 0.999994883 |
| astral microtubule organization (GO:0030953 | 44570 | 0.666534103 | 0.999994883 |
| basement membrane assembly (GO:0070831 | 44570 | 0.666534103 | 0.999994883 |
| CD40 signaling pathway (GO:0023035) | 44570 | 0.666534103 | 0.999994883 |
| cellular response to nitric oxide (GO:0071732 | 44570 | 0.666534103 | 0.999994883 |
| cellular response to reactive nitrogen species | 44570 | 0.666534103 | 0.999994883 |
| cellular response to sterol depletion (GO:007 | 44570 | 0.666534103 | 0.999994883 |
| cholesterol catabolic process (GO:0006707) | 44570 | 0.666534103 | 0.999994883 |
| chylomicron remodeling (GO:0034371) | 44570 | 0.666534103 | 0.999994883 |
| collagen-activated signaling pathway (GO:003 | 44570 | 0.666534103 | 0.999994883 |
| complement activation, classical pathway (G | 44570 | 0.666534103 | 0.999994883 |
| development of primary female sexual chara | 44570 | 0.666534103 | 0.999994883 |
| DNA topological change (GO:0006265) | 44570 | 0.666534103 | 0.999994883 |
| embryonic forelimb morphogenesis (GO:003 | 44570 | 0.666534103 | 0.999994883 |
| endochondral ossification (GO:0001958) | 44570 | 0.666534103 | 0.999994883 |
| epithelial cilium movement involved in deter | 44570 | 0.666534103 | 0.999994883 |
| flavonoid glucuronidation (GO:0052696) | 44570 | 0.666534103 | 0.999994883 |
| fucose catabolic process (GO:0019317) | 44570 | 0.666534103 | 0.999994883 |
| glycerolipid biosynthetic process (GO:004501 | 44570 | 0.666534103 | 0.999994883 |
| histone phosphorylation (GO:0016572) | 44570 | 0.666534103 | 0.999994883 |
| IMP metabolic process (GO:0046040) | 44570 | 0.666534103 | 0.999994883 |
| intestinal cholesterol absorption (GO:003029 | 44570 | 0.666534103 | 0.999994883 |
| isoprenoid biosynthetic process (GO:0008299 | 44570 | 0.666534103 | 0.999994883 |
| L-fucose catabolic process (GO:0042355) | 44570 | 0.666534103 | 0.999994883 |
| L-fucose metabolic process (GO:0042354) | 44570 | 0.666534103 | 0.999994883 |
| mesodermal cell differentiation (GO:0048333 | 44570 | 0.666534103 | 0.999994883 |
| mitral valve morphogenesis (GO:0003183) | 44570 | 0.666534103 | 0.999994883 |
| negative regulation of alpha-beta T cell activa | 44570 | 0.666534103 | 0.999994883 |
| negative regulation of biomineralization (GO: | 44570 | 0.666534103 | 0.999994883 |
| negative regulation of exocytosis (GO:00459 | 44570 | 0.666534103 | 0.999994883 |
| negative regulation of glycoprotein biosynthe | 44570 | 0.666534103 | 0.999994883 |
| negative regulation of leukocyte degranulatic | 44570 | 0.666534103 | 0.999994883 |
| negative regulation of leukocyte proliferatior | 44570 | 0.666534103 | 0.999994883 |
| negative regulation of lipase activity (GO:006 | 44570 | 0.666534103 | 0.999994883 |
| negative regulation of lipid localization (GO:1 | 44570 | 0.666534103 | 0.999994883 |
| negative regulation of membrane potential (( | 44570 | 0.666534103 | 0.999994883 |
| negative regulation of necroptotic process (G | 44570 | 0.666534103 | 0.999994883 |
| negative regulation of pathway-restricted SM | 44570 | 0.666534103 | 0.999994883 |
| negative regulation of protein-containing cor | 44570 | 0.666534103 | 0.999994883 |
| nuclear RNA surveillance (GO:0071027) | 44570 | 0.666534103 | 0.999994883 |
| organic acid catabolic process (GO:0016054) | 44570 | 0.666534103 | 0.999994883 |
| ornithine metabolic process (GO:0006591) | 44570 | 0.666534103 | 0.999994883 |
| phosphorylation of RNA polymerase II C-term | 44570 | 0.666534103 | 0.999994883 |
| positive regulation of ATP biosynthetic proce | 44570 | 0.666534103 | 0.999994883 |
| positive regulation of autophagosome assem | 44570 | 0.666534103 | 0.999994883 |
| positive regulation of B cell differentiation (G | 44570 | 0.666534103 | 0.999994883 |

| | | | |
|---|---|---|---|
| positive regulation of blood pressure (GO:004 | 44570 | 0.666534103 | 0.999994883 |
| positive regulation of cholesterol esterificatic | 44570 | 0.666534103 | 0.999994883 |
| positive regulation of fatty acid beta-oxidatio | 44570 | 0.666534103 | 0.999994883 |
| positive regulation of mast cell degranulatior | 44570 | 0.666534103 | 0.999994883 |
| positive regulation of memory T cell different | 44570 | 0.666534103 | 0.999994883 |
| positive regulation of mitochondrial membra | 44570 | 0.666534103 | 0.999994883 |
| positive regulation of NLRP3 inflammasome c | 44570 | 0.666534103 | 0.999994883 |
| positive regulation of protein kinase A signali | 44570 | 0.666534103 | 0.999994883 |
| positive regulation of protein kinase C signali | 44570 | 0.666534103 | 0.999994883 |
| positive regulation of triglyceride lipase activ | 44570 | 0.666534103 | 0.999994883 |
| progesterone metabolic process (GO:004244 | 44570 | 0.666534103 | 0.999994883 |
| quaternary ammonium group transport (GO: | 44570 | 0.666534103 | 0.999994883 |
| regulation of animal organ morphogenesis (G | 44570 | 0.666534103 | 0.999994883 |
| regulation of CD8-positive, alpha-beta T cell a | 44570 | 0.666534103 | 0.999994883 |
| regulation of complement activation, classica | 44570 | 0.666534103 | 0.999994883 |
| regulation of epidermis development (GO:00 | 44570 | 0.666534103 | 0.999994883 |
| regulation of glucan biosynthetic process (GC | 44570 | 0.666534103 | 0.999994883 |
| regulation of lipid transport (GO:0032368) | 44570 | 0.666534103 | 0.999994883 |
| regulation of PERK-mediated unfolded protei | 44570 | 0.666534103 | 0.999994883 |
| regulation of peroxisome proliferator activat | 44570 | 0.666534103 | 0.999994883 |
| regulation of pinocytosis (GO:0048548) | 44570 | 0.666534103 | 0.999994883 |
| regulation of respiratory burst (GO:0060263) | 44570 | 0.666534103 | 0.999994883 |
| regulation of smooth muscle cell apoptotic p | 44570 | 0.666534103 | 0.999994883 |
| regulation of sterol biosynthetic process (GO | 44570 | 0.666534103 | 0.999994883 |
| regulation of tumor necrosis factor superfam | 44570 | 0.666534103 | 0.999994883 |
| regulation of vesicle fusion (GO:0031338) | 44570 | 0.666534103 | 0.999994883 |
| regulation of Wnt signaling pathway, planar c | 44570 | 0.666534103 | 0.999994883 |
| response to magnesium ion (GO:0032026) | 44570 | 0.666534103 | 0.999994883 |
| response to type I interferon (GO:0034340) | 44570 | 0.666534103 | 0.999994883 |
| response to vitamin D (GO:0033280) | 44570 | 0.666534103 | 0.999994883 |
| retinal rod cell differentiation (GO:0060221) | 44570 | 0.666534103 | 0.999994883 |
| semaphorin-plexin signaling pathway involve | 44570 | 0.666534103 | 0.999994883 |
| sensory organ morphogenesis (GO:0090596) | 44570 | 0.666534103 | 0.999994883 |
| somatic recombination of immunoglobulin ge | 44570 | 0.666534103 | 0.999994883 |
| sterol catabolic process (GO:0016127) | 44570 | 0.666534103 | 0.999994883 |
| sympathetic ganglion development (GO:0061 | 44570 | 0.666534103 | 0.999994883 |
| T-helper 1 type immune response (GO:0042C | 44570 | 0.666534103 | 0.999994883 |
| very-low-density lipoprotein particle remode | 44570 | 0.666534103 | 0.999994883 |
| glucose homeostasis (GO:0042593) | 31656 | 0.66695454 | 0.999994883 |
| regulation of neuron death (GO:1901214) | 31656 | 0.66695454 | 0.999994883 |
| positive regulation of epithelial cell proliferat | 13/123 | 0.667181378 | 0.999994883 |
| fat cell differentiation (GO:0045444) | 21337 | 0.668550577 | 0.999994883 |
| negative regulation of hydrolase activity (GO | 21337 | 0.668550577 | 0.999994883 |
| cellular response to ketone (GO:1901655) | 14336 | 0.669783396 | 0.999994883 |
| mitochondrial electron transport, NADH to ul | 14336 | 0.669783396 | 0.999994883 |
| negative regulation of lymphocyte proliferati | 14336 | 0.669783396 | 0.999994883 |
| protein monoubiquitination (GO:0006513) | 14336 | 0.669783396 | 0.999994883 |

| | | | |
|---|---|---|---|
| regulation of cellular component biogenesis ( | 14336 | 0.669783396 | 0.999994883 |
| regulation of ion transmembrane transporter | 14336 | 0.669783396 | 0.999994883 |
| regulation of nitric-oxide synthase activity (G | 14336 | 0.669783396 | 0.999994883 |
| vesicle cytoskeletal trafficking (GO:0099518) | 14336 | 0.669783396 | 0.999994883 |
| regulation of cell cycle (GO:0051726) | 32/296 | 0.670008816 | 0.999994883 |
| adherens junction organization (GO:0034332 | 18019 | 0.677301999 | 0.999994883 |
| inner mitochondrial membrane organization | 18019 | 0.677301999 | 0.999994883 |
| regulation of cellular localization (GO:006034 | 18019 | 0.677301999 | 0.999994883 |
| positive regulation of protein localization to r | 25020 | 0.67744237 | 0.999994883 |
| regulation of translational initiation (GO:000( | 25020 | 0.67744237 | 0.999994883 |
| negative regulation of angiogenesis (GO:001( | 32021 | 0.680569717 | 0.999994883 |
| actin filament organization (GO:0007015) | 16/152 | 0.682559629 | 0.999994883 |
| negative regulation of intracellular signal tran | 21/198 | 0.68446785 | 0.999994883 |
| negative regulation of DNA-binding transcrip | 15/143 | 0.68466458 | 0.999994883 |
| cell projection organization (GO:0030030) | 44650 | 0.6858303 | 0.999994883 |
| maintenance of blood-brain barrier (GO:003 | 44650 | 0.6858303 | 0.999994883 |
| negative regulation of cytoskeleton organizat | 44650 | 0.6858303 | 0.999994883 |
| negative regulation of interleukin-1 productio | 44650 | 0.6858303 | 0.999994883 |
| peptidyl-lysine trimethylation (GO:0018023) | 44650 | 0.6858303 | 0.999994883 |
| plasma membrane invagination (GO:009902 | 44650 | 0.6858303 | 0.999994883 |
| positive regulation of protein import (GO:190 | 44650 | 0.6858303 | 0.999994883 |
| positive regulation of small GTPase mediated | 44650 | 0.6858303 | 0.999994883 |
| regulation of natural killer cell mediated cyto | 44650 | 0.6858303 | 0.999994883 |
| response to alcohol (GO:0097305) | 44650 | 0.6858303 | 0.999994883 |
| snRNA 3'-end processing (GO:0034472) | 44650 | 0.6858303 | 0.999994883 |
| antigen processing and presentation of exoge | 28703 | 0.685858618 | 0.999994883 |
| protein modification process (GO:0036211) | 28703 | 0.685858618 | 0.999994883 |
| early endosome to late endosome transport | 44612 | 0.686784081 | 0.999994883 |
| endoplasmic reticulum calcium ion homeosta | 44612 | 0.686784081 | 0.999994883 |
| epithelial cilium movement involved in extra | 44612 | 0.686784081 | 0.999994883 |
| establishment of epithelial cell polarity (GO:0 | 44612 | 0.686784081 | 0.999994883 |
| gastrulation (GO:0007369) | 44612 | 0.686784081 | 0.999994883 |
| glucose transmembrane transport (GO:1904( | 44612 | 0.686784081 | 0.999994883 |
| granulocyte differentiation (GO:0030851) | 44612 | 0.686784081 | 0.999994883 |
| hepaticobiliary system development (GO:006 | 44612 | 0.686784081 | 0.999994883 |
| negative regulation of axon extension involve | 44612 | 0.686784081 | 0.999994883 |
| negative regulation of cyclin-dependent prot | 44612 | 0.686784081 | 0.999994883 |
| oligodendrocyte differentiation (GO:004870 | 44612 | 0.686784081 | 0.999994883 |
| organic cation transport (GO:0015695) | 44612 | 0.686784081 | 0.999994883 |
| positive regulation of activated T cell prolifer | 44612 | 0.686784081 | 0.999994883 |
| positive regulation of alpha-beta T cell activa | 44612 | 0.686784081 | 0.999994883 |
| positive regulation of hormone secretion (GC | 44612 | 0.686784081 | 0.999994883 |
| positive regulation of lymphocyte differentia | 44612 | 0.686784081 | 0.999994883 |
| positive regulation of peptidyl-lysine acetylat | 44612 | 0.686784081 | 0.999994883 |
| positive regulation of protein processing (GO | 44612 | 0.686784081 | 0.999994883 |
| regulation of acute inflammatory response (( | 44612 | 0.686784081 | 0.999994883 |
| regulation of smooth muscle cell migration (( | 44612 | 0.686784081 | 0.999994883 |

| | | | |
|---|---|---|---|
| xenobiotic transport (GO:0042908) | 44612 | 0.686784081 | 0.999994883 |
| negative regulation of cell-substrate adhesion | 14702 | 0.689479358 | 0.999994883 |
| positive regulation of intrinsic apoptotic signa | 14702 | 0.689479358 | 0.999994883 |
| positive regulation of wound healing (GO:009 | 14702 | 0.689479358 | 0.999994883 |
| protein targeting (GO:0006605) | 13/125 | 0.689888945 | 0.999994883 |
| cellular response to calcium ion (GO:0071277 | 25385 | 0.692431402 | 0.999994883 |
| cellular response to decreased oxygen levels | 25385 | 0.692431402 | 0.999994883 |
| negative regulation of binding (GO:0051100) | 32387 | 0.693840845 | 0.999994883 |
| sensory perception of mechanical stimulus (G | 32387 | 0.693840845 | 0.999994883 |
| positive regulation of ion transport (GO:0043 | 18384 | 0.694797176 | 0.999994883 |
| regulation of generation of precursor metabo | 18384 | 0.694797176 | 0.999994883 |
| cellular protein catabolic process (GO:00442 | 22068 | 0.700766989 | 0.999994883 |
| positive regulation of defense response (GO: | 36069 | 0.701429418 | 0.999994883 |
| regulation of peptidyl-serine phosphorylation | 36069 | 0.701429418 | 0.999994883 |
| adherens junction assembly (GO:0034333) | 44571 | 0.704849758 | 0.999994883 |
| alanine transport (GO:0032328) | 44571 | 0.704849758 | 0.999994883 |
| amino sugar metabolic process (GO:0006040 | 44571 | 0.704849758 | 0.999994883 |
| arginine metabolic process (GO:0006525) | 44571 | 0.704849758 | 0.999994883 |
| cellular response to acetylcholine (GO:19051 | 44571 | 0.704849758 | 0.999994883 |
| cellular response to interleukin-4 (GO:00713 | 44571 | 0.704849758 | 0.999994883 |
| cellular response to purine-containing compo | 44571 | 0.704849758 | 0.999994883 |
| cGMP biosynthetic process (GO:0006182) | 44571 | 0.704849758 | 0.999994883 |
| chloride ion homeostasis (GO:0055064) | 44571 | 0.704849758 | 0.999994883 |
| chondroitin sulfate proteoglycan metabolic p | 44571 | 0.704849758 | 0.999994883 |
| chylomicron assembly (GO:0034378) | 44571 | 0.704849758 | 0.999994883 |
| complement activation, lectin pathway (GO:( | 44571 | 0.704849758 | 0.999994883 |
| dicarboxylic acid biosynthetic process (GO:00 | 44571 | 0.704849758 | 0.999994883 |
| forelimb morphogenesis (GO:0035136) | 44571 | 0.704849758 | 0.999994883 |
| genetic imprinting (GO:0071514) | 44571 | 0.704849758 | 0.999994883 |
| hematopoietic stem cell differentiation (GO:( | 44571 | 0.704849758 | 0.999994883 |
| histone H3-K36 demethylation (GO:0070544) | 44571 | 0.704849758 | 0.999994883 |
| I-kappaB phosphorylation (GO:0007252) | 44571 | 0.704849758 | 0.999994883 |
| immunoglobulin mediated immune response | 44571 | 0.704849758 | 0.999994883 |
| indolalkylamine catabolic process (GO:00462 | 44571 | 0.704849758 | 0.999994883 |
| inner ear receptor cell stereocilium organizat | 44571 | 0.704849758 | 0.999994883 |
| lactation (GO:0007595) | 44571 | 0.704849758 | 0.999994883 |
| leptin-mediated signaling pathway (GO:0033 | 44571 | 0.704849758 | 0.999994883 |
| lipid phosphorylation (GO:0046834) | 44571 | 0.704849758 | 0.999994883 |
| lung epithelium development (GO:0060428) | 44571 | 0.704849758 | 0.999994883 |
| methylguanosine-cap decapping (GO:011015 | 44571 | 0.704849758 | 0.999994883 |
| modification-dependent macromolecule cata | 44571 | 0.704849758 | 0.999994883 |
| modulation by symbiont of host process (GO | 44571 | 0.704849758 | 0.999994883 |
| monocyte activation (GO:0042117) | 44571 | 0.704849758 | 0.999994883 |
| monovalent inorganic anion homeostasis (GC | 44571 | 0.704849758 | 0.999994883 |
| muscle cell migration (GO:0014812) | 44571 | 0.704849758 | 0.999994883 |
| negative regulation of alpha-beta T cell prolif | 44571 | 0.704849758 | 0.999994883 |
| negative regulation of cellular protein catabo | 44571 | 0.704849758 | 0.999994883 |

| | | | |
|---|---|---|---|
| negative regulation of circadian rhythm (GO:( | 44571 | 0.704849758 | 0.999994883 |
| negative regulation of glycolytic process (GO: | 44571 | 0.704849758 | 0.999994883 |
| negative regulation of hormone secretion (G( | 44571 | 0.704849758 | 0.999994883 |
| negative regulation of humoral immune resp | 44571 | 0.704849758 | 0.999994883 |
| negative regulation of inclusion body assemb | 44571 | 0.704849758 | 0.999994883 |
| negative regulation of protein autophosphor | 44571 | 0.704849758 | 0.999994883 |
| negative regulation of purine nucleotide met | 44571 | 0.704849758 | 0.999994883 |
| negative regulation of sodium ion transmeml | 44571 | 0.704849758 | 0.999994883 |
| negative regulation of toll-like receptor 4 sigr | 44571 | 0.704849758 | 0.999994883 |
| negative regulation of transforming growth fa | 44571 | 0.704849758 | 0.999994883 |
| negative regulation of voltage-gated calcium | 44571 | 0.704849758 | 0.999994883 |
| neuron projection fasciculation (GO:0106030 | 44571 | 0.704849758 | 0.999994883 |
| neuron remodeling (GO:0016322) | 44571 | 0.704849758 | 0.999994883 |
| nuclear mRNA surveillance (GO:0071028) | 44571 | 0.704849758 | 0.999994883 |
| nuclear-transcribed mRNA catabolic process, | 44571 | 0.704849758 | 0.999994883 |
| nucleoside metabolic process (GO:0009116) | 44571 | 0.704849758 | 0.999994883 |
| omega-hydroxylase P450 pathway (GO:0097; | 44571 | 0.704849758 | 0.999994883 |
| phosphate ion homeostasis (GO:0055062) | 44571 | 0.704849758 | 0.999994883 |
| positive regulation by symbiont of entry into | 44571 | 0.704849758 | 0.999994883 |
| positive regulation of circadian rhythm (GO:0 | 44571 | 0.704849758 | 0.999994883 |
| positive regulation of coagulation (GO:00508 | 44571 | 0.704849758 | 0.999994883 |
| positive regulation of dendritic spine morphc | 44571 | 0.704849758 | 0.999994883 |
| positive regulation of hemostasis (GO:19000 | 44571 | 0.704849758 | 0.999994883 |
| positive regulation of keratinocyte migration | 44571 | 0.704849758 | 0.999994883 |
| positive regulation of skeletal muscle tissue c | 44571 | 0.704849758 | 0.999994883 |
| positive regulation of transcription by RNA pc | 44571 | 0.704849758 | 0.999994883 |
| positive regulation of transcription of nucleol | 44571 | 0.704849758 | 0.999994883 |
| positive regulation of vascular endothelial gr( | 44571 | 0.704849758 | 0.999994883 |
| positive regulation of viral entry into host cel | 44571 | 0.704849758 | 0.999994883 |
| primary alcohol catabolic process (GO:00343 | 44571 | 0.704849758 | 0.999994883 |
| primary miRNA processing (GO:0031053) | 44571 | 0.704849758 | 0.999994883 |
| protein insertion into ER membrane by stop-t | 44571 | 0.704849758 | 0.999994883 |
| protein prenylation (GO:0018342) | 44571 | 0.704849758 | 0.999994883 |
| receptor guanylyl cyclase signaling pathway ( | 44571 | 0.704849758 | 0.999994883 |
| regulation of actin filament-based movemen | 44571 | 0.704849758 | 0.999994883 |
| regulation of bile acid biosynthetic process (( | 44571 | 0.704849758 | 0.999994883 |
| regulation of carbohydrate biosynthetic proc | 44571 | 0.704849758 | 0.999994883 |
| regulation of cell-cell adhesion mediated by i | 44571 | 0.704849758 | 0.999994883 |
| regulation of DNA methylation (GO:0044030) | 44571 | 0.704849758 | 0.999994883 |
| regulation of granulocyte differentiation (GO | 44571 | 0.704849758 | 0.999994883 |
| regulation of high voltage-gated calcium char | 44571 | 0.704849758 | 0.999994883 |
| regulation of leukocyte tethering or rolling (G | 44571 | 0.704849758 | 0.999994883 |
| regulation of mast cell activation (GO:00330( | 44571 | 0.704849758 | 0.999994883 |
| regulation of memory T cell differentiation (G | 44571 | 0.704849758 | 0.999994883 |
| regulation of phospholipid metabolic process | 44571 | 0.704849758 | 0.999994883 |
| regulation of protein localization to centroso | 44571 | 0.704849758 | 0.999994883 |
| regulation of receptor binding (GO:1900120) | 44571 | 0.704849758 | 0.999994883 |

| | | | |
|---|---|---|---|
| regulation of store-operated calcium entry (G | 44571 | 0.704849758 | 0.999994883 |
| regulation of tau-protein kinase activity (GO: | 44571 | 0.704849758 | 0.999994883 |
| response to interleukin-4 (GO:0070670) | 44571 | 0.704849758 | 0.999994883 |
| response to laminar fluid shear stress (GO:00 | 44571 | 0.704849758 | 0.999994883 |
| retinoic acid receptor signaling pathway (GO: | 44571 | 0.704849758 | 0.999994883 |
| sarcoplasmic reticulum calcium ion transport | 44571 | 0.704849758 | 0.999994883 |
| sequestering of actin monomers (GO:004298 | 44571 | 0.704849758 | 0.999994883 |
| sulfur amino acid catabolic process (GO:0000 | 44571 | 0.704849758 | 0.999994883 |
| collagen fibril organization (GO:0030199) | 32752 | 0.706761666 | 0.999994883 |
| positive regulation of inflammatory response | 32752 | 0.706761666 | 0.999994883 |
| carbohydrate homeostasis (GO:0033500) | 25750 | 0.706961756 | 0.999994883 |
| regulation of plasma membrane bounded cel | 25750 | 0.706961756 | 0.999994883 |
| positive regulation of gene expression (GO:0( | 52/482 | 0.707302803 | 0.999994883 |
| 'de novo' posttranslational protein folding (G | 44651 | 0.70748793 | 0.999994883 |
| cellular response to cAMP (GO:0071320) | 44651 | 0.70748793 | 0.999994883 |
| negative regulation of DNA binding (GO:0043 | 44651 | 0.70748793 | 0.999994883 |
| negative regulation of osteoblast differentiat | 44651 | 0.70748793 | 0.999994883 |
| positive regulation of protein targeting to mi | 44651 | 0.70748793 | 0.999994883 |
| positive regulation of ubiquitin-protein transf | 44651 | 0.70748793 | 0.999994883 |
| regulation of calcium ion-dependent exocyto | 44651 | 0.70748793 | 0.999994883 |
| regulation of cellular component size (GO:00 | 44651 | 0.70748793 | 0.999994883 |
| regulation of developmental growth (GO:004 | 44651 | 0.70748793 | 0.999994883 |
| triglyceride homeostasis (GO:0070328) | 44651 | 0.70748793 | 0.999994883 |
| JNK cascade (GO:0007254) | 15067 | 0.708326162 | 0.999994883 |
| nucleobase-containing compound catabolic p | 15067 | 0.708326162 | 0.999994883 |
| protein methylation (GO:0006479) | 15067 | 0.708326162 | 0.999994883 |
| regulation of mitochondrial membrane poter | 15067 | 0.708326162 | 0.999994883 |
| regulation of cellular amine metabolic proces | 18749 | 0.71162892 | 0.999994883 |
| regulation of epithelial cell migration (GO:00 | 18749 | 0.71162892 | 0.999994883 |
| regulation of substrate adhesion-dependent | 18749 | 0.71162892 | 0.999994883 |
| autonomic nervous system development (GC | 44613 | 0.71277298 | 0.999994883 |
| cellular response to steroid hormone stimulu | 44613 | 0.71277298 | 0.999994883 |
| chondroitin sulfate biosynthetic process (GO: | 44613 | 0.71277298 | 0.999994883 |
| myotube differentiation (GO:0014902) | 44613 | 0.71277298 | 0.999994883 |
| negative regulation of ion transmembrane tr | 44613 | 0.71277298 | 0.999994883 |
| negative regulation of microtubule depolyme | 44613 | 0.71277298 | 0.999994883 |
| negative regulation of protein localization to | 44613 | 0.71277298 | 0.999994883 |
| negative regulation of reproductive process ( | 44613 | 0.71277298 | 0.999994883 |
| negative regulation of response to wounding | 44613 | 0.71277298 | 0.999994883 |
| neuron apoptotic process (GO:0051402) | 44613 | 0.71277298 | 0.999994883 |
| positive regulation of antigen receptor-media | 44613 | 0.71277298 | 0.999994883 |
| regulation of anoikis (GO:2000209) | 44613 | 0.71277298 | 0.999994883 |
| regulation of cardiac muscle cell proliferatior | 44613 | 0.71277298 | 0.999994883 |
| regulation of ubiquitin protein ligase activity | 44613 | 0.71277298 | 0.999994883 |
| rRNA modification (GO:0000154) | 44613 | 0.71277298 | 0.999994883 |
| sensory perception of pain (GO:0019233) | 44613 | 0.71277298 | 0.999994883 |
| regulation of mRNA stability (GO:0043488) | 15/146 | 0.715255493 | 0.999994883 |

| | | | |
|---|---|---|---|
| cholesterol homeostasis (GO:0042632) | 26115 | 0.721028238 | 0.999994883 |
| Fc-gamma receptor signaling pathway involv | 26115 | 0.721028238 | 0.999994883 |
| histone acetylation (GO:0016573) | 26115 | 0.721028238 | 0.999994883 |
| cell-matrix adhesion (GO:0007160) | 10/100 | 0.725348372 | 0.999994883 |
| positive regulation of translation (GO:004572 | 10/100 | 0.725348372 | 0.999994883 |
| regulation of transforming growth factor beta | 10/100 | 0.725348372 | 0.999994883 |
| ATP-dependent chromatin remodeling (GO:0 | 15432 | 0.726324705 | 0.999994883 |
| ceramide biosynthetic process (GO:0046513) | 15432 | 0.726324705 | 0.999994883 |
| monocyte chemotaxis (GO:0002548) | 15432 | 0.726324705 | 0.999994883 |
| nucleic acid-templated transcription (GO:009 | 15432 | 0.726324705 | 0.999994883 |
| regulation of nervous system development (( | 15432 | 0.726324705 | 0.999994883 |
| regulation of angiogenesis (GO:0045765) | 21/203 | 0.727518884 | 0.999994883 |
| activation of phospholipase C activity (GO:00 | 11749 | 0.727979614 | 0.999994883 |
| cardiac muscle tissue morphogenesis (GO:00 | 11749 | 0.727979614 | 0.999994883 |
| cellular response to topologically incorrect pr | 11749 | 0.727979614 | 0.999994883 |
| intra-Golgi vesicle-mediated transport (GO:0( | 11749 | 0.727979614 | 0.999994883 |
| mitochondrial membrane organization (GO:0 | 11749 | 0.727979614 | 0.999994883 |
| modulation by host of symbiont process (GO | 11749 | 0.727979614 | 0.999994883 |
| negative regulation of cell-matrix adhesion (( | 11749 | 0.727979614 | 0.999994883 |
| negative regulation of developmental growth | 11749 | 0.727979614 | 0.999994883 |
| negative regulation of intracellular steroid hc | 11749 | 0.727979614 | 0.999994883 |
| peptidyl-proline modification (GO:0018208) | 11749 | 0.727979614 | 0.999994883 |
| positive regulation of lamellipodium organiza | 11749 | 0.727979614 | 0.999994883 |
| regulation of cell migration involved in sprou | 11749 | 0.727979614 | 0.999994883 |
| regulation of NMDA receptor activity (GO:20 | 11749 | 0.727979614 | 0.999994883 |
| response to amino acid (GO:0043200) | 11749 | 0.727979614 | 0.999994883 |
| positive regulation of cysteine-type endopep | 22798 | 0.730804722 | 0.999994883 |
| response to endoplasmic reticulum stress (G( | 11/110 | 0.731220166 | 0.999994883 |
| sensory perception of sound (GO:0007605) | 33482 | 0.731533817 | 0.999994883 |
| positive regulation of macromolecule biosynt | 13/129 | 0.732452486 | 0.999994883 |
| Fc-gamma receptor signaling pathway (GO:0( | 26481 | 0.734627613 | 0.999994883 |
| positive regulation of protein secretion (GO:( | 26481 | 0.734627613 | 0.999994883 |
| sterol homeostasis (GO:0055092) | 26481 | 0.734627613 | 0.999994883 |
| homologous chromosome segregation (GO:0 | 44614 | 0.736926691 | 0.999994883 |
| natural killer cell mediated cytotoxicity (GO:( | 44614 | 0.736926691 | 0.999994883 |
| negative regulation of cyclin-dependent prot | 44614 | 0.736926691 | 0.999994883 |
| negative regulation of interleukin-2 productic | 44614 | 0.736926691 | 0.999994883 |
| negative regulation of myeloid cell differentia | 44614 | 0.736926691 | 0.999994883 |
| negative regulation of protein dephosphoryla | 44614 | 0.736926691 | 0.999994883 |
| organelle fusion (GO:0048284) | 44614 | 0.736926691 | 0.999994883 |
| phosphatidylserine acyl-chain remodeling (G( | 44614 | 0.736926691 | 0.999994883 |
| positive regulation of amyloid precursor prot | 44614 | 0.736926691 | 0.999994883 |
| positive regulation of DNA-templated transcr | 44614 | 0.736926691 | 0.999994883 |
| positive regulation of peptidase activity (GO:( | 44614 | 0.736926691 | 0.999994883 |
| positive regulation of signal transduction by p | 44614 | 0.736926691 | 0.999994883 |
| proteasomal ubiquitin-independent protein c | 44614 | 0.736926691 | 0.999994883 |
| protein localization to cell junction (GO:1902 | 44614 | 0.736926691 | 0.999994883 |

| | | | |
|---|---|---|---|
| regulation of adenylate cyclase activity (GO:0 | 44614 | 0.736926691 | 0.999994883 |
| regulation of endoplasmic reticulum stress-ir | 44614 | 0.736926691 | 0.999994883 |
| regulation of lipid storage (GO:0010883) | 44614 | 0.736926691 | 0.999994883 |
| regulation of response to cytokine stimulus ( | 44614 | 0.736926691 | 0.999994883 |
| rRNA methylation (GO:0031167) | 44614 | 0.736926691 | 0.999994883 |
| ubiquitin-dependent protein catabolic proces | 44614 | 0.736926691 | 0.999994883 |
| cellular response to lipopolysaccharide (GO:0 | 12/120 | 0.736931566 | 0.999994883 |
| protein dephosphorylation (GO:0006470) | 14/139 | 0.73830548 | 0.999994883 |
| alcohol catabolic process (GO:0046164) | 44572 | 0.738764569 | 0.999994883 |
| aminoglycan metabolic process (GO:0006022 | 44572 | 0.738764569 | 0.999994883 |
| angiogenesis involved in wound healing (GO: | 44572 | 0.738764569 | 0.999994883 |
| atrial septum morphogenesis (GO:0060413) | 44572 | 0.738764569 | 0.999994883 |
| B cell mediated immunity (GO:0019724) | 44572 | 0.738764569 | 0.999994883 |
| cardiac muscle cell membrane repolarization | 44572 | 0.738764569 | 0.999994883 |
| cell differentiation in spinal cord (GO:002151 | 44572 | 0.738764569 | 0.999994883 |
| cell proliferation in forebrain (GO:0021846) | 44572 | 0.738764569 | 0.999994883 |
| cellular response to increased oxygen levels ( | 44572 | 0.738764569 | 0.999994883 |
| chromatin-mediated maintenance of transcri | 44572 | 0.738764569 | 0.999994883 |
| enteric nervous system development (GO:00 | 44572 | 0.738764569 | 0.999994883 |
| establishment of epithelial cell apical/basal p | 44572 | 0.738764569 | 0.999994883 |
| Golgi inheritance (GO:0048313) | 44572 | 0.738764569 | 0.999994883 |
| humoral immune response mediated by circu | 44572 | 0.738764569 | 0.999994883 |
| induction of positive chemotaxis (GO:005093 | 44572 | 0.738764569 | 0.999994883 |
| inositol phosphate dephosphorylation (GO:0( | 44572 | 0.738764569 | 0.999994883 |
| insulin-like growth factor receptor signaling p | 44572 | 0.738764569 | 0.999994883 |
| intestinal lipid absorption (GO:0098856) | 44572 | 0.738764569 | 0.999994883 |
| isotype switching (GO:0045190) | 44572 | 0.738764569 | 0.999994883 |
| lipoprotein biosynthetic process (GO:004215 | 44572 | 0.738764569 | 0.999994883 |
| metanephric mesenchyme development (GO | 44572 | 0.738764569 | 0.999994883 |
| miRNA mediated inhibition of translation (GC | 44572 | 0.738764569 | 0.999994883 |
| myotube cell development (GO:0014904) | 44572 | 0.738764569 | 0.999994883 |
| negative regulation of activated T cell prolifer | 44572 | 0.738764569 | 0.999994883 |
| negative regulation of activin receptor signali | 44572 | 0.738764569 | 0.999994883 |
| negative regulation of cholesterol transport ( | 44572 | 0.738764569 | 0.999994883 |
| negative regulation of defense response to vi | 44572 | 0.738764569 | 0.999994883 |
| negative regulation of interleukin-17 product | 44572 | 0.738764569 | 0.999994883 |
| negative regulation of lymphocyte migration | 44572 | 0.738764569 | 0.999994883 |
| negative regulation of oxidative stress-induce | 44572 | 0.738764569 | 0.999994883 |
| negative regulation of potassium ion transme | 44572 | 0.738764569 | 0.999994883 |
| negative regulation of programmed necrotic | 44572 | 0.738764569 | 0.999994883 |
| negative regulation of protein localization to | 44572 | 0.738764569 | 0.999994883 |
| negative regulation of sprouting angiogenesis | 44572 | 0.738764569 | 0.999994883 |
| negative regulation of T-helper cell differenti | 44572 | 0.738764569 | 0.999994883 |
| negative regulation of translation, ncRNA-me | 44572 | 0.738764569 | 0.999994883 |
| organophosphate catabolic process (GO:004( | 44572 | 0.738764569 | 0.999994883 |
| phosphorylated carbohydrate dephosphoryla | 44572 | 0.738764569 | 0.999994883 |
| positive regulation of actin nucleation (GO:0( | 44572 | 0.738764569 | 0.999994883 |

| | | | |
|---|---|---|---|
| positive regulation of alcohol biosynthetic pr | 44572 | 0.738764569 | 0.999994883 |
| positive regulation of cellular respiration (GO | 44572 | 0.738764569 | 0.999994883 |
| positive regulation of cytoplasmic transport ( | 44572 | 0.738764569 | 0.999994883 |
| positive regulation of dendrite development | 44572 | 0.738764569 | 0.999994883 |
| positive regulation of DNA methylation-depe | 44572 | 0.738764569 | 0.999994883 |
| positive regulation of histone exchange (GO:1 | 44572 | 0.738764569 | 0.999994883 |
| positive regulation of keratinocyte proliferati | 44572 | 0.738764569 | 0.999994883 |
| positive regulation of macrophage differentia | 44572 | 0.738764569 | 0.999994883 |
| positive regulation of non-canonical Wnt sigr | 44572 | 0.738764569 | 0.999994883 |
| positive regulation of phospholipid transport | 44572 | 0.738764569 | 0.999994883 |
| positive regulation of transcription from RNA | 44572 | 0.738764569 | 0.999994883 |
| positive regulation of triglyceride biosyntheti | 44572 | 0.738764569 | 0.999994883 |
| protein kinase C signaling (GO:0070528) | 44572 | 0.738764569 | 0.999994883 |
| protein localization to phagophore assembly | 44572 | 0.738764569 | 0.999994883 |
| pyrimidine nucleoside catabolic process (GO: | 44572 | 0.738764569 | 0.999994883 |
| regulation of cardiac muscle cell apoptotic pr | 44572 | 0.738764569 | 0.999994883 |
| regulation of CD4-positive, alpha-beta T cell p | 44572 | 0.738764569 | 0.999994883 |
| regulation of coagulation (GO:0050818) | 44572 | 0.738764569 | 0.999994883 |
| regulation of gene expression by genetic imp | 44572 | 0.738764569 | 0.999994883 |
| regulation of histone exchange (GO:1900049 | 44572 | 0.738764569 | 0.999994883 |
| regulation of inclusion body assembly (GO:00 | 44572 | 0.738764569 | 0.999994883 |
| regulation of lymphocyte migration (GO:2000 | 44572 | 0.738764569 | 0.999994883 |
| regulation of mast cell activation involved in | 44572 | 0.738764569 | 0.999994883 |
| regulation of mitochondrial depolarization (G | 44572 | 0.738764569 | 0.999994883 |
| regulation of phospholipase activity (GO:001 | 44572 | 0.738764569 | 0.999994883 |
| regulation of stress-activated protein kinase s | 44572 | 0.738764569 | 0.999994883 |
| regulation of T cell apoptotic process (GO:007 | 44572 | 0.738764569 | 0.999994883 |
| regulation of T cell mediated immunity (GO:0 | 44572 | 0.738764569 | 0.999994883 |
| regulation of T-helper 2 cell cytokine product | 44572 | 0.738764569 | 0.999994883 |
| regulation of toll-like receptor 2 signaling pat | 44572 | 0.738764569 | 0.999994883 |
| response to sterol (GO:0036314) | 44572 | 0.738764569 | 0.999994883 |
| signal peptide processing (GO:0006465) | 44572 | 0.738764569 | 0.999994883 |
| skeletal muscle cell differentiation (GO:0035 | 44572 | 0.738764569 | 0.999994883 |
| synaptic vesicle transport (GO:0048489) | 44572 | 0.738764569 | 0.999994883 |
| tryptophan metabolic process (GO:0006568) | 44572 | 0.738764569 | 0.999994883 |
| vasoconstriction (GO:0042310) | 44572 | 0.738764569 | 0.999994883 |
| xenobiotic glucuronidation (GO:0052697) | 44572 | 0.738764569 | 0.999994883 |
| Fc-epsilon receptor signaling pathway (GO:00 | 11/111 | 0.742059775 | 0.999994883 |
| regulation of response to external stimulus (( | 13/130 | 0.742479246 | 0.999994883 |
| positive regulation of NIK/NF-kappaB signalin | 19480 | 0.74329545 | 0.999994883 |
| positive regulation of phospholipase C activit | 15797 | 0.743480808 | 0.999994883 |
| spermatid development (GO:0007286) | 15797 | 0.743480808 | 0.999994883 |
| tRNA aminoacylation for protein translation ( | 15797 | 0.743480808 | 0.999994883 |
| proteasomal protein catabolic process (GO:0 | 21/205 | 0.743692008 | 0.999994883 |
| positive regulation of hydrolase activity (GO:( | 15/149 | 0.743954795 | 0.999994883 |
| amyloid fibril formation (GO:1990000) | 23163 | 0.744999799 | 0.999994883 |
| cellular protein-containing complex assembly | 17/168 | 0.74609982 | 0.999994883 |

| | | | |
|---|---|---|---|
| actin filament bundle organization (GO:0061! | 12114 | 0.747326302 | 0.999994883 |
| muscle cell development (GO:0055001) | 12114 | 0.747326302 | 0.999994883 |
| negative chemotaxis (GO:0050919) | 12114 | 0.747326302 | 0.999994883 |
| positive regulation of cytokine production inv | 12114 | 0.747326302 | 0.999994883 |
| regulation of neurotransmitter secretion (GO | 12114 | 0.747326302 | 0.999994883 |
| RNA stabilization (GO:0043489) | 12114 | 0.747326302 | 0.999994883 |
| signal transduction by p53 class mediator (GC | 12114 | 0.747326302 | 0.999994883 |
| vasculogenesis (GO:0001570) | 12114 | 0.747326302 | 0.999994883 |
| positive regulation of cellular protein metabc | 10/102 | 0.747975547 | 0.999994883 |
| positive regulation of vasculature developme | 10/102 | 0.747975547 | 0.999994883 |
| positive regulation of signal transduction (GC | 26/252 | 0.748887151 | 0.999994883 |
| cellular protein metabolic process (GO:0044Z | 44/417 | 0.749032073 | 0.999994883 |
| protein autophosphorylation (GO:0046777) | 16/159 | 0.749412109 | 0.999994883 |
| glycoprotein biosynthetic process (GO:00091 | 30529 | 0.751129752 | 0.999994883 |
| regulation of translation (GO:0006417) | 18/178 | 0.751540981 | 0.999994883 |
| Fc receptor signaling pathway (GO:0038093) | 11/112 | 0.752603375 | 0.999994883 |
| positive regulation of peptidyl-serine phosph | 34213 | 0.754861129 | 0.999994883 |
| regulation of heart contraction (GO:0008016 | 34213 | 0.754861129 | 0.999994883 |
| positive regulation of leukocyte chemotaxis ( | 19845 | 0.758135045 | 0.999994883 |
| regulation of cellular amino acid metabolic pi | 19845 | 0.758135045 | 0.999994883 |
| diterpenoid metabolic process (GO:0016101) | 23529 | 0.758646784 | 0.999994883 |
| regulation of transcription initiation from RN. | 23529 | 0.758646784 | 0.999994883 |
| negative regulation of G protein-coupled rec | 44615 | 0.759323757 | 0.999994883 |
| negative regulation of GTPase activity (GO:0( | 44615 | 0.759323757 | 0.999994883 |
| positive regulation of histone acetylation (GC | 44615 | 0.759323757 | 0.999994883 |
| positive regulation of JUN kinase activity (GO | 44615 | 0.759323757 | 0.999994883 |
| positive regulation of transcription initiation | 44615 | 0.759323757 | 0.999994883 |
| regulation of calcium ion transmembrane tra | 44615 | 0.759323757 | 0.999994883 |
| regulation of release of sequestered calcium | 44615 | 0.759323757 | 0.999994883 |
| regulation of supramolecular fiber organizati | 44615 | 0.759323757 | 0.999994883 |
| vesicle transport along microtubule (GO:004 | 44615 | 0.759323757 | 0.999994883 |
| homotypic cell-cell adhesion (GO:0034109) | 16163 | 0.759804592 | 0.999994883 |
| intracellular steroid hormone receptor signal | 16163 | 0.759804592 | 0.999994883 |
| positive regulation of cell-matrix adhesion (G | 16163 | 0.759804592 | 0.999994883 |
| positive regulation of stress fiber assembly (C | 16163 | 0.759804592 | 0.999994883 |
| Fc receptor mediated stimulatory signaling p | 27211 | 0.760421098 | 0.999994883 |
| regulation of signal transduction (GO:000996 | 20/198 | 0.761874857 | 0.999994883 |
| positive regulation of metabolic process (GO: | 11/113 | 0.762849925 | 0.999994883 |
| response to insulin (GO:0032868) | 30895 | 0.762961931 | 0.999994883 |
| regulation of cell growth (GO:0001558) | 22/217 | 0.764265195 | 0.999994883 |
| cell-substrate junction assembly (GO:000704 | 12479 | 0.765555026 | 0.999994883 |
| central nervous system neuron differentiatio | 12479 | 0.765555026 | 0.999994883 |
| histone lysine methylation (GO:0034968) | 12479 | 0.765555026 | 0.999994883 |
| organic cyclic compound biosynthetic proces | 12479 | 0.765555026 | 0.999994883 |
| positive regulation of calcium ion transport ir | 12479 | 0.765555026 | 0.999994883 |
| positive regulation of epidermal growth facto | 12479 | 0.765555026 | 0.999994883 |
| positive regulation of interleukin-12 producti | 12479 | 0.765555026 | 0.999994883 |

| | | | |
|---|---|---|---|
| positive regulation of pri-miRNA transcriptior | 12479 | 0.765555026 | 0.999994883 |
| positive regulation of release of sequestered | 12479 | 0.765555026 | 0.999994883 |
| protein insertion into membrane (GO:00512( | 12479 | 0.765555026 | 0.999994883 |
| regulation of activated T cell proliferation (G( | 12479 | 0.765555026 | 0.999994883 |
| tRNA methylation (GO:0030488) | 12479 | 0.765555026 | 0.999994883 |
| hemopoiesis (GO:0030097) | 34578 | 0.765980109 | 0.999994883 |
| negative regulation of cellular protein metab | 34578 | 0.765980109 | 0.999994883 |
| adipose tissue development (GO:0060612) | 44573 | 0.768783811 | 0.999994883 |
| angiotensin maturation (GO:0002003) | 44573 | 0.768783811 | 0.999994883 |
| ATP transport (GO:0015867) | 44573 | 0.768783811 | 0.999994883 |
| attachment of mitotic spindle microtubules t | 44573 | 0.768783811 | 0.999994883 |
| brain morphogenesis (GO:0048854) | 44573 | 0.768783811 | 0.999994883 |
| cell differentiation in hindbrain (GO:0021533 | 44573 | 0.768783811 | 0.999994883 |
| cellular response to nitrogen levels (GO:0043 | 44573 | 0.768783811 | 0.999994883 |
| cellular response to nitrogen starvation (GO:( | 44573 | 0.768783811 | 0.999994883 |
| dermatan sulfate biosynthetic process (GO:0( | 44573 | 0.768783811 | 0.999994883 |
| early endosome to Golgi transport (GO:0034 | 44573 | 0.768783811 | 0.999994883 |
| endoplasmic reticulum membrane organizati | 44573 | 0.768783811 | 0.999994883 |
| energy coupled proton transport, down elect | 44573 | 0.768783811 | 0.999994883 |
| epithelial cell-cell adhesion (GO:0090136) | 44573 | 0.768783811 | 0.999994883 |
| ethanol oxidation (GO:0006069) | 44573 | 0.768783811 | 0.999994883 |
| glomerular visceral epithelial cell differentiat | 44573 | 0.768783811 | 0.999994883 |
| inositol phosphate catabolic process (GO:007 | 44573 | 0.768783811 | 0.999994883 |
| maintenance of protein localization in endop | 44573 | 0.768783811 | 0.999994883 |
| melanocyte differentiation (GO:0030318) | 44573 | 0.768783811 | 0.999994883 |
| mitochondrial electron transport, ubiquinol t | 44573 | 0.768783811 | 0.999994883 |
| mitochondrion distribution (GO:0048311) | 44573 | 0.768783811 | 0.999994883 |
| modulation by host of viral process (GO:0044 | 44573 | 0.768783811 | 0.999994883 |
| motile cilium assembly (GO:0044458) | 44573 | 0.768783811 | 0.999994883 |
| negative regulation by host of viral process (( | 44573 | 0.768783811 | 0.999994883 |
| negative regulation of cardiac muscle hypertı | 44573 | 0.768783811 | 0.999994883 |
| negative regulation of cellular response to va | 44573 | 0.768783811 | 0.999994883 |
| negative regulation of complement activatioı | 44573 | 0.768783811 | 0.999994883 |
| negative regulation of fatty acid biosynthetic | 44573 | 0.768783811 | 0.999994883 |
| negative regulation of hemostasis (GO:19000 | 44573 | 0.768783811 | 0.999994883 |
| negative regulation of production of miRNAs | 44573 | 0.768783811 | 0.999994883 |
| negative regulation of transcription elongatic | 44573 | 0.768783811 | 0.999994883 |
| nuclear body organization (GO:0030575) | 44573 | 0.768783811 | 0.999994883 |
| nucleoside catabolic process (GO:0009164) | 44573 | 0.768783811 | 0.999994883 |
| organic hydroxy compound catabolic process | 44573 | 0.768783811 | 0.999994883 |
| peptidyl-methionine modification (GO:00182 | 44573 | 0.768783811 | 0.999994883 |
| phasic smooth muscle contraction (GO:0014 | 44573 | 0.768783811 | 0.999994883 |
| phospholipid efflux (GO:0033700) | 44573 | 0.768783811 | 0.999994883 |
| phospholipid homeostasis (GO:0055091) | 44573 | 0.768783811 | 0.999994883 |
| positive regulation of cellular senescence (GC | 44573 | 0.768783811 | 0.999994883 |
| positive regulation of endoplasmic reticulum | 44573 | 0.768783811 | 0.999994883 |
| positive regulation of metaphase/anaphase t | 44573 | 0.768783811 | 0.999994883 |

| | | | |
|---|---|---|---|
| positive regulation of mitotic metaphase/ana | 44573 | 0.768783811 | 0.999994883 |
| positive regulation of mitotic sister chromati | 44573 | 0.768783811 | 0.999994883 |
| positive regulation of protein depolymerizati | 44573 | 0.768783811 | 0.999994883 |
| purine nucleotide biosynthetic process (GO:0 | 44573 | 0.768783811 | 0.999994883 |
| regulation of actin filament depolymerizatior | 44573 | 0.768783811 | 0.999994883 |
| regulation of alcohol biosynthetic process (G | 44573 | 0.768783811 | 0.999994883 |
| regulation of amyloid precursor protein catal | 44573 | 0.768783811 | 0.999994883 |
| regulation of angiotensin levels in blood (GO: | 44573 | 0.768783811 | 0.999994883 |
| regulation of axon regeneration (GO:004867 | 44573 | 0.768783811 | 0.999994883 |
| regulation of feeding behavior (GO:0060259) | 44573 | 0.768783811 | 0.999994883 |
| regulation of glycogen metabolic process (GC | 44573 | 0.768783811 | 0.999994883 |
| regulation of hemopoiesis (GO:1903706) | 44573 | 0.768783811 | 0.999994883 |
| regulation of keratinocyte migration (GO:005 | 44573 | 0.768783811 | 0.999994883 |
| regulation of muscle system process (GO:009 | 44573 | 0.768783811 | 0.999994883 |
| regulation of natural killer cell activation (GO | 44573 | 0.768783811 | 0.999994883 |
| regulation of plasminogen activation (GO:001 | 44573 | 0.768783811 | 0.999994883 |
| regulation of sequestering of calcium ion (GC | 44573 | 0.768783811 | 0.999994883 |
| regulation of sister chromatid cohesion (GO:( | 44573 | 0.768783811 | 0.999994883 |
| toll-like receptor 3 signaling pathway (GO:00 | 44573 | 0.768783811 | 0.999994883 |
| regulation of cell-matrix adhesion (GO:00019 | 23894 | 0.771749106 | 0.999994883 |
| protein sumoylation (GO:0016925) | 20210 | 0.772320168 | 0.999994883 |
| regulation of gene silencing by RNA (GO:006( | 20210 | 0.772320168 | 0.999994883 |
| regulation of posttranscriptional gene silenci | 20210 | 0.772320168 | 0.999994883 |
| activation of protein kinase activity (GO:0032 | 11/114 | 0.772799096 | 0.999994883 |
| histone modification (GO:0016570) | 11/114 | 0.772799096 | 0.999994883 |
| negative regulation of cell motility (GO:2000 | 11/114 | 0.772799096 | 0.999994883 |
| Wnt signaling pathway, planar cell polarity pa | 31260 | 0.774386229 | 0.999994883 |
| neural crest cell development (GO:0014032) | 16528 | 0.775309906 | 0.999994883 |
| positive regulation of response to biotic stim | 16528 | 0.775309906 | 0.999994883 |
| regulation of pri-miRNA transcription by RNA | 16528 | 0.775309906 | 0.999994883 |
| sensory perception of light stimulus (GO:005 | 34943 | 0.776736509 | 0.999994883 |
| regulation of macromolecule metabolic proc | 20/200 | 0.777018313 | 0.999994883 |
| cellular response to lipid (GO:0071396) | 22/219 | 0.778685245 | 0.999994883 |
| cardiac muscle cell development (GO:005501 | 44616 | 0.78004889 | 0.999994883 |
| heterochromatin assembly (GO:0031507) | 44616 | 0.78004889 | 0.999994883 |
| immune response-activating cell surface rece | 44616 | 0.78004889 | 0.999994883 |
| negative regulation of lipid metabolic proces | 44616 | 0.78004889 | 0.999994883 |
| negative regulation of protein depolymerizat | 44616 | 0.78004889 | 0.999994883 |
| negative regulation of synaptic transmission | 44616 | 0.78004889 | 0.999994883 |
| nucleotide-binding domain, leucine rich repe | 44616 | 0.78004889 | 0.999994883 |
| positive regulation of cell-substrate junction | 44616 | 0.78004889 | 0.999994883 |
| protein alpha-1,2-demannosylation (GO:003 | 44616 | 0.78004889 | 0.999994883 |
| regulation of monooxygenase activity (GO:00 | 44616 | 0.78004889 | 0.999994883 |
| positive regulation of protein metabolic proc | 11/115 | 0.782451235 | 0.999994883 |
| acylglycerol catabolic process (GO:0046464) | 12844 | 0.782697748 | 0.999994883 |
| negative regulation of epithelial cell migratio | 12844 | 0.782697748 | 0.999994883 |
| negative regulation of neurogenesis (GO:005 | 12844 | 0.782697748 | 0.999994883 |

| | | | |
|---|---|---|---|
| regulation of blood circulation (GO:1903522) | 12844 | 0.782697748 | 0.999994883 |
| regulation of ion transmembrane transport (( | 12844 | 0.782697748 | 0.999994883 |
| regulation of protein polymerization (GO:003 | 12844 | 0.782697748 | 0.999994883 |
| negative regulation of cell migration (GO:003 | 14/144 | 0.783763263 | 0.999994883 |
| cell morphogenesis involved in neuron differ | 27942 | 0.78435054 | 0.999994883 |
| cellular response to hormone stimulus (GO:0 | 27942 | 0.78435054 | 0.999994883 |
| positive regulation of growth (GO:0045927) | 27942 | 0.78435054 | 0.999994883 |
| regulation of interferon-gamma production ( | 31625 | 0.785404851 | 0.999994883 |
| chemokine-mediated signaling pathway (GO: | 20576 | 0.785859898 | 0.999994883 |
| nuclear-transcribed mRNA catabolic process, | 20576 | 0.785859898 | 0.999994883 |
| peptidyl-tyrosine modification (GO:0018212) | 20576 | 0.785859898 | 0.999994883 |
| tight junction assembly (GO:0120192) | 20576 | 0.785859898 | 0.999994883 |
| interleukin-12-mediated signaling pathway (( | 16893 | 0.790013784 | 0.999994883 |
| regulation of B cell proliferation (GO:003088 | 16893 | 0.790013784 | 0.999994883 |
| tumor necrosis factor-mediated signaling pat | 11/116 | 0.791807327 | 0.999994883 |
| positive regulation of cell motility (GO:20001 | 22/221 | 0.792501974 | 0.999994883 |
| negative regulation of cell growth (GO:00303 | 12/126 | 0.794513459 | 0.999994883 |
| negative regulation of growth (GO:0045926) | 12/126 | 0.794513459 | 0.999994883 |
| AMP metabolic process (GO:0046033) | 44574 | 0.795354769 | 0.999994883 |
| cellular biogenic amine metabolic process (G | 44574 | 0.795354769 | 0.999994883 |
| chromatin organization involved in regulatior | 44574 | 0.795354769 | 0.999994883 |
| embryonic hemopoiesis (GO:0035162) | 44574 | 0.795354769 | 0.999994883 |
| endochondral bone morphogenesis (GO:006( | 44574 | 0.795354769 | 0.999994883 |
| extracellular transport (GO:0006858) | 44574 | 0.795354769 | 0.999994883 |
| high-density lipoprotein particle assembly (G | 44574 | 0.795354769 | 0.999994883 |
| homeostasis of number of cells (GO:0048872 | 44574 | 0.795354769 | 0.999994883 |
| inner dynein arm assembly (GO:0036159) | 44574 | 0.795354769 | 0.999994883 |
| kinetochore organization (GO:0051383) | 44574 | 0.795354769 | 0.999994883 |
| lymphocyte mediated immunity (GO:000244 | 44574 | 0.795354769 | 0.999994883 |
| microtubule-based transport (GO:0099111) | 44574 | 0.795354769 | 0.999994883 |
| mitotic DNA integrity checkpoint signaling (G | 44574 | 0.795354769 | 0.999994883 |
| muscle tissue development (GO:0060537) | 44574 | 0.795354769 | 0.999994883 |
| negative regulation by host of viral transcript | 44574 | 0.795354769 | 0.999994883 |
| negative regulation of autophagosome assen | 44574 | 0.795354769 | 0.999994883 |
| negative regulation of endoplasmic reticulum | 44574 | 0.795354769 | 0.999994883 |
| negative regulation of epidermal growth fact | 44574 | 0.795354769 | 0.999994883 |
| negative regulation of gluconeogenesis (GO:( | 44574 | 0.795354769 | 0.999994883 |
| negative regulation of hippo signaling (GO:0C | 44574 | 0.795354769 | 0.999994883 |
| negative regulation of locomotion (GO:0040( | 44574 | 0.795354769 | 0.999994883 |
| negative regulation of macromolecule biosyn | 44574 | 0.795354769 | 0.999994883 |
| negative regulation of potassium ion transpo | 44574 | 0.795354769 | 0.999994883 |
| negative regulation of reactive oxygen specie | 44574 | 0.795354769 | 0.999994883 |
| negative regulation of vascular endothelial gr | 44574 | 0.795354769 | 0.999994883 |
| plasma membrane repair (GO:0001778) | 44574 | 0.795354769 | 0.999994883 |
| positive regulation of CD4-positive, alpha-bet | 44574 | 0.795354769 | 0.999994883 |
| positive regulation of cell aging (GO:0090343 | 44574 | 0.795354769 | 0.999994883 |
| positive regulation of cellular carbohydrate n | 44574 | 0.795354769 | 0.999994883 |

| | | | |
|---|---|---|---|
| positive regulation of ERAD pathway (GO:190 | 44574 | 0.795354769 | 0.999994883 |
| positive regulation of heterochromatin assen | 44574 | 0.795354769 | 0.999994883 |
| positive regulation of protein exit from endo | 44574 | 0.795354769 | 0.999994883 |
| positive regulation of purine nucleotide biosy | 44574 | 0.795354769 | 0.999994883 |
| positive regulation of transporter activity (GC | 44574 | 0.795354769 | 0.999994883 |
| protein neddylation (GO:0045116) | 44574 | 0.795354769 | 0.999994883 |
| purine ribonucleoside triphosphate metaboli | 44574 | 0.795354769 | 0.999994883 |
| purine-containing compound salvage (GO:00 | 44574 | 0.795354769 | 0.999994883 |
| regulation of antigen receptor-mediated sign | 44574 | 0.795354769 | 0.999994883 |
| regulation of homotypic cell-cell adhesion (G | 44574 | 0.795354769 | 0.999994883 |
| regulation of nucleocytoplasmic transport (G | 44574 | 0.795354769 | 0.999994883 |
| regulation of oxidoreductase activity (GO:005 | 44574 | 0.795354769 | 0.999994883 |
| regulation of platelet-derived growth factor r | 44574 | 0.795354769 | 0.999994883 |
| regulation of protein exit from endoplasmic r | 44574 | 0.795354769 | 0.999994883 |
| regulation of receptor signaling pathway via S | 44574 | 0.795354769 | 0.999994883 |
| regulation of retrograde protein transport, EI | 44574 | 0.795354769 | 0.999994883 |
| response to UV-B (GO:0010224) | 44574 | 0.795354769 | 0.999994883 |
| reticulophagy (GO:0061709) | 44574 | 0.795354769 | 0.999994883 |
| ribosomal subunit export from nucleus (GO:0 | 44574 | 0.795354769 | 0.999994883 |
| sodium ion export across plasma membrane | 44574 | 0.795354769 | 0.999994883 |
| synaptic vesicle localization (GO:0097479) | 44574 | 0.795354769 | 0.999994883 |
| transcription preinitiation complex assembly | 44574 | 0.795354769 | 0.999994883 |
| triglyceride-rich lipoprotein particle remodeli | 44574 | 0.795354769 | 0.999994883 |
| positive regulation of NF-kappaB transcriptio | 15/155 | 0.795487786 | 0.999994883 |
| positive regulation of protein modification by | 28307 | 0.795625258 | 0.999994883 |
| ERAD pathway (GO:0036503) | 31990 | 0.796021054 | 0.999994883 |
| regulation of transcription from RNA polymer | 31990 | 0.796021054 | 0.999994883 |
| sodium ion transmembrane transport (GO:00 | 31990 | 0.796021054 | 0.999994883 |
| regulation of gene silencing by miRNA (GO:00 | 24624 | 0.796342528 | 0.999994883 |
| calcium ion homeostasis (GO:0055074) | 35674 | 0.797168732 | 0.999994883 |
| arachidonic acid metabolic process (GO:0019 | 20941 | 0.798765442 | 0.999994883 |
| cellular response to peptide (GO:1901653) | 20941 | 0.798765442 | 0.999994883 |
| negative regulation of cell cycle G2/M phase | 20941 | 0.798765442 | 0.999994883 |
| positive regulation of cell junction assembly ( | 20941 | 0.798765442 | 0.999994883 |
| positive regulation of gene expression, epige | 20941 | 0.798765442 | 0.999994883 |
| positive regulation of interferon-gamma proc | 20941 | 0.798765442 | 0.999994883 |
| regulation of bone mineralization (GO:00305 | 20941 | 0.798765442 | 0.999994883 |
| renal system development (GO:0072001) | 20941 | 0.798765442 | 0.999994883 |
| macrophage activation (GO:0042116) | 13210 | 0.798790326 | 0.999994883 |
| negative regulation of cellular protein localiz | 13210 | 0.798790326 | 0.999994883 |
| platelet aggregation (GO:0070527) | 13210 | 0.798790326 | 0.999994883 |
| positive regulation of bone mineralization (G | 13210 | 0.798790326 | 0.999994883 |
| positive regulation of mitotic nuclear division | 13210 | 0.798790326 | 0.999994883 |
| positive regulation of substrate adhesion-dep | 13210 | 0.798790326 | 0.999994883 |
| positive regulation of T cell mediated immun | 13210 | 0.798790326 | 0.999994883 |
| regulation of androgen receptor signaling pat | 13210 | 0.798790326 | 0.999994883 |
| regulation of cell cycle G1/S phase transition | 13210 | 0.798790326 | 0.999994883 |

| | | | |
|---|---|---|---|
| negative regulation of canonical Wnt signalin | 16/165 | 0.798828989 | 0.999994883 |
| acylglycerol homeostasis (GO:0055090) | 44617 | 0.799190521 | 0.999994883 |
| cAMP-mediated signaling (GO:0019933) | 44617 | 0.799190521 | 0.999994883 |
| chemical synaptic transmission, postsynaptic | 44617 | 0.799190521 | 0.999994883 |
| divalent inorganic cation homeostasis (GO:0( | 44617 | 0.799190521 | 0.999994883 |
| endosome transport via multivesicular body : | 44617 | 0.799190521 | 0.999994883 |
| negative regulation of actin filament polymer | 44617 | 0.799190521 | 0.999994883 |
| negative regulation of antigen receptor-medi | 44617 | 0.799190521 | 0.999994883 |
| negative regulation of cell junction assembly | 44617 | 0.799190521 | 0.999994883 |
| negative regulation of cellular carbohydrate r | 44617 | 0.799190521 | 0.999994883 |
| peptide catabolic process (GO:0043171) | 44617 | 0.799190521 | 0.999994883 |
| positive regulation of lipid metabolic process | 44617 | 0.799190521 | 0.999994883 |
| positive regulation of peptidyl-threonine pho | 44617 | 0.799190521 | 0.999994883 |
| protein quality control for misfolded or incor | 44617 | 0.799190521 | 0.999994883 |
| receptor signaling pathway via STAT (GO:009 | 44617 | 0.799190521 | 0.999994883 |
| regulation of epidermal cell differentiation (( | 44617 | 0.799190521 | 0.999994883 |
| regulation of heterotypic cell-cell adhesion (( | 44617 | 0.799190521 | 0.999994883 |
| regulation of microtubule depolymerization ( | 44617 | 0.799190521 | 0.999994883 |
| regulation of mitochondrial fission (GO:0090: | 44617 | 0.799190521 | 0.999994883 |
| regulation of rhodopsin mediated signaling p | 44617 | 0.799190521 | 0.999994883 |
| regulation of spindle assembly (GO:0090169) | 44617 | 0.799190521 | 0.999994883 |
| response to hexose (GO:0009746) | 44617 | 0.799190521 | 0.999994883 |
| ventricular cardiac muscle tissue developmer | 44617 | 0.799190521 | 0.999994883 |
| epithelial to mesenchymal transition (GO:00( | 17258 | 0.803935941 | 0.999994883 |
| positive regulation of neuron death (GO:190: | 17258 | 0.803935941 | 0.999994883 |
| positive regulation of pathway-restricted SM. | 17258 | 0.803935941 | 0.999994883 |
| regulation of establishment of planar polarity | 32356 | 0.806239061 | 0.999994883 |
| positive regulation of multicellular organisma | 35/345 | 0.807262083 | 0.999994883 |
| regulation of myeloid cell differentiation (GO | 24990 | 0.807848979 | 0.999994883 |
| autophagosome assembly (GO:0000045) | 21306 | 0.811049845 | 0.999994883 |
| nucleosome assembly (GO:0006334) | 21306 | 0.811049845 | 0.999994883 |
| regulation of pathway-restricted SMAD prote | 21306 | 0.811049845 | 0.999994883 |
| negative regulation of epithelial cell apoptoti | 13575 | 0.813871603 | 0.999994883 |
| regulation of erythrocyte differentiation (GO | 13575 | 0.813871603 | 0.999994883 |
| regulation of microtubule cytoskeleton orgar | 13575 | 0.813871603 | 0.999994883 |
| regulation of p38MAPK cascade (GO:190074 | 13575 | 0.813871603 | 0.999994883 |
| regulation of cytosolic calcium ion concentra | 14/148 | 0.815924078 | 0.999994883 |
| regulation of cysteine-type endopeptidase ac | 32721 | 0.816063975 | 0.999994883 |
| second-messenger-mediated signaling (GO:0 | 32721 | 0.816063975 | 0.999994883 |
| mRNA-containing ribonucleoprotein complex | 36404 | 0.816180339 | 0.999994883 |
| negative regulation of NF-kappaB transcriptic | 29037 | 0.81682355 | 0.999994883 |
| chaperone cofactor-dependent protein refol | 44618 | 0.81683883 | 0.999994883 |
| clathrin-dependent endocytosis (GO:007258: | 44618 | 0.81683883 | 0.999994883 |
| glial cell development (GO:0021782) | 44618 | 0.81683883 | 0.999994883 |
| histone lysine demethylation (GO:0070076) | 44618 | 0.81683883 | 0.999994883 |
| histone methylation (GO:0016571) | 44618 | 0.81683883 | 0.999994883 |
| meiosis I (GO:0007127) | 44618 | 0.81683883 | 0.999994883 |

| | | | |
|---|---|---|---|
| membrane depolarization during action pote | 44618 | 0.81683883 | 0.999994883 |
| negative regulation of interleukin-1 beta proc | 44618 | 0.81683883 | 0.999994883 |
| negative regulation of protein transport (GO: | 44618 | 0.81683883 | 0.999994883 |
| positive regulation of filopodium assembly (G | 44618 | 0.81683883 | 0.999994883 |
| positive regulation of G1/S transition of mito | 44618 | 0.81683883 | 0.999994883 |
| positive regulation of interleukin-2 productio | 44618 | 0.81683883 | 0.999994883 |
| regulation of mitotic metaphase/anaphase tr | 44618 | 0.81683883 | 0.999994883 |
| regulation of regulatory T cell differentiation | 44618 | 0.81683883 | 0.999994883 |
| regulation of viral life cycle (GO:1903900) | 44618 | 0.81683883 | 0.999994883 |
| retinoic acid metabolic process (GO:0042573 | 44618 | 0.81683883 | 0.999994883 |
| cellular response to interleukin-12 (GO:0071: | 17624 | 0.817098312 | 0.999994883 |
| establishment of protein localization to mito | 17624 | 0.817098312 | 0.999994883 |
| negative regulation of MAP kinase activity (G | 17624 | 0.817098312 | 0.999994883 |
| regulation of interleukin-2 production (GO:0( | 17624 | 0.817098312 | 0.999994883 |
| regulation of protein metabolic process (GO: | 17624 | 0.817098312 | 0.999994883 |
| positive regulation of MAP kinase activity (G( | 25355 | 0.818841112 | 0.999994883 |
| positive regulation of protein polymerization | 25355 | 0.818841112 | 0.999994883 |
| antigen processing and presentation of endo | 44575 | 0.818873391 | 0.999994883 |
| aorta development (GO:0035904) | 44575 | 0.818873391 | 0.999994883 |
| atrial septum development (GO:0003283) | 44575 | 0.818873391 | 0.999994883 |
| atrioventricular valve development (GO:0003 | 44575 | 0.818873391 | 0.999994883 |
| bone morphogenesis (GO:0060349) | 44575 | 0.818873391 | 0.999994883 |
| carbohydrate transport (GO:0008643) | 44575 | 0.818873391 | 0.999994883 |
| heart process (GO:0003015) | 44575 | 0.818873391 | 0.999994883 |
| membrane repolarization during action poter | 44575 | 0.818873391 | 0.999994883 |
| microvillus organization (GO:0032528) | 44575 | 0.818873391 | 0.999994883 |
| myeloid dendritic cell activation (GO:000177 | 44575 | 0.818873391 | 0.999994883 |
| N-terminal protein amino acid modification ( | 44575 | 0.818873391 | 0.999994883 |
| NAD biosynthesis via nicotinamide riboside s | 44575 | 0.818873391 | 0.999994883 |
| negative regulation of cell migration involvec | 44575 | 0.818873391 | 0.999994883 |
| negative regulation of DNA-templated transc | 44575 | 0.818873391 | 0.999994883 |
| negative regulation of smooth muscle cell mi | 44575 | 0.818873391 | 0.999994883 |
| neuromuscular process controlling balance (( | 44575 | 0.818873391 | 0.999994883 |
| nuclear migration (GO:0007097) | 44575 | 0.818873391 | 0.999994883 |
| ovarian follicle development (GO:0001541) | 44575 | 0.818873391 | 0.999994883 |
| polarized epithelial cell differentiation (GO:0( | 44575 | 0.818873391 | 0.999994883 |
| positive regulation of brown fat cell different | 44575 | 0.818873391 | 0.999994883 |
| positive regulation of glycogen biosynthetic p | 44575 | 0.818873391 | 0.999994883 |
| positive regulation of granulocyte macrophag | 44575 | 0.818873391 | 0.999994883 |
| positive regulation of insulin secretion involv | 44575 | 0.818873391 | 0.999994883 |
| positive regulation of leukocyte degranulatio | 44575 | 0.818873391 | 0.999994883 |
| positive regulation of potassium ion transme | 44575 | 0.818873391 | 0.999994883 |
| positive regulation of T cell chemotaxis (GO:( | 44575 | 0.818873391 | 0.999994883 |
| positive regulation of T cell receptor signaling | 44575 | 0.818873391 | 0.999994883 |
| potassium ion export across plasma membra | 44575 | 0.818873391 | 0.999994883 |
| regulation of DNA methylation-dependent he | 44575 | 0.818873391 | 0.999994883 |
| regulation of histone deacetylation (GO:0031 | 44575 | 0.818873391 | 0.999994883 |

| | | | |
|---|---|---|---|
| regulation of lamellipodium organization (GC | 44575 | 0.818873391 | 0.999994883 |
| regulation of lipase activity (GO:0060191) | 44575 | 0.818873391 | 0.999994883 |
| regulation of nitric-oxide synthase biosynthe | 44575 | 0.818873391 | 0.999994883 |
| regulation of transcription of nucleolar large | 44575 | 0.818873391 | 0.999994883 |
| relaxation of muscle (GO:0090075) | 44575 | 0.818873391 | 0.999994883 |
| response to chemokine (GO:1990868) | 44575 | 0.818873391 | 0.999994883 |
| secretion (GO:0046903) | 44575 | 0.818873391 | 0.999994883 |
| sequestering of extracellular ligand from rece | 44575 | 0.818873391 | 0.999994883 |
| smooth muscle contraction (GO:0006939) | 44575 | 0.818873391 | 0.999994883 |
| sodium-independent organic anion transport | 44575 | 0.818873391 | 0.999994883 |
| striated muscle cell development (GO:00550 | 44575 | 0.818873391 | 0.999994883 |
| substrate-dependent cell migration (GO:0006 | 44575 | 0.818873391 | 0.999994883 |
| thymus development (GO:0048538) | 44575 | 0.818873391 | 0.999994883 |
| tRNA wobble base modification (GO:0002097 | 44575 | 0.818873391 | 0.999994883 |
| vascular endothelial growth factor signaling p | 44575 | 0.818873391 | 0.999994883 |
| vasodilation (GO:0042311) | 44575 | 0.818873391 | 0.999994883 |
| phospholipid transport (GO:0015914) | 21671 | 0.82272773 | 0.999994883 |
| positive regulation of apoptotic signaling path | 21671 | 0.82272773 | 0.999994883 |
| negative regulation of cellular response to gr | 29403 | 0.826761805 | 0.999994883 |
| positive regulation of canonical Wnt signaling | 12/130 | 0.827495709 | 0.999994883 |
| connective tissue development (GO:0061448 | 13940 | 0.827982606 | 0.999994883 |
| negative regulation of ubiquitin-dependent p | 13940 | 0.827982606 | 0.999994883 |
| positive regulation of insulin secretion (GO:0 | 13940 | 0.827982606 | 0.999994883 |
| positive regulation of potassium ion transpor | 13940 | 0.827982606 | 0.999994883 |
| spliceosomal snRNP assembly (GO:0000387) | 13940 | 0.827982606 | 0.999994883 |
| tRNA aminoacylation (GO:0043039) | 13940 | 0.827982606 | 0.999994883 |
| aerobic electron transport chain (GO:001964 | 25720 | 0.829329785 | 0.999994883 |
| establishment of protein localization to mem | 25720 | 0.829329785 | 0.999994883 |
| positive regulation of cell-substrate adhesion | 25720 | 0.829329785 | 0.999994883 |
| positive regulation of actin filament polymeri | 17989 | 0.829524629 | 0.999994883 |
| regulation of smooth muscle cell proliferatio | 17989 | 0.829524629 | 0.999994883 |
| response to unfolded protein (GO:0006986) | 17989 | 0.829524629 | 0.999994883 |
| camera-type eye development (GO:0043010 | 44619 | 0.833084176 | 0.999994883 |
| cellular response to cadmium ion (GO:00712 | 44619 | 0.833084176 | 0.999994883 |
| cytoplasmic pattern recognition receptor sig | 44619 | 0.833084176 | 0.999994883 |
| modulation of excitatory postsynaptic potent | 44619 | 0.833084176 | 0.999994883 |
| negative regulation of catalytic activity (GO:0 | 44619 | 0.833084176 | 0.999994883 |
| negative regulation of endothelial cell apopto | 44619 | 0.833084176 | 0.999994883 |
| negative regulation of insulin receptor signali | 44619 | 0.833084176 | 0.999994883 |
| positive regulation of erythrocyte differentia | 44619 | 0.833084176 | 0.999994883 |
| positive regulation of striated muscle cell diff | 44619 | 0.833084176 | 0.999994883 |
| presynaptic endocytosis (GO:0140238) | 44619 | 0.833084176 | 0.999994883 |
| protein peptidyl-prolyl isomerization (GO:000 | 44619 | 0.833084176 | 0.999994883 |
| regulation of blood coagulation (GO:003019 | 44619 | 0.833084176 | 0.999994883 |
| regulation of cardiac muscle cell contraction | 44619 | 0.833084176 | 0.999994883 |
| homophilic cell adhesion via plasma membra | 22037 | 0.833815052 | 0.999994883 |
| Notch signaling pathway (GO:0007219) | 22037 | 0.833815052 | 0.999994883 |

| | | | |
|---|---|---|---|
| RNA methylation (GO:0001510) | 22037 | 0.833815052 | 0.999994883 |
| positive regulation of supramolecular fiber or | 33451 | 0.834558824 | 0.999994883 |
| toll-like receptor signaling pathway (GO:0002 | 33451 | 0.834558824 | 0.999994883 |
| cellular response to hypoxia (GO:0071456) | 12/131 | 0.835082904 | 0.999994883 |
| cellular response to metal ion (GO:0071248) | 12/131 | 0.835082904 | 0.999994883 |
| mitochondrial ATP synthesis coupled electron | 26085 | 0.839326835 | 0.999994883 |
| nucleic acid metabolic process (GO:0090304) | 26085 | 0.839326835 | 0.999994883 |
| regulation of hematopoietic stem cell differe | 26085 | 0.839326835 | 0.999994883 |
| aflatoxin metabolic process (GO:0046222) | 44576 | 0.839690177 | 0.999994883 |
| androgen receptor signaling pathway (GO:00 | 44576 | 0.839690177 | 0.999994883 |
| catecholamine metabolic process (GO:00065 | 44576 | 0.839690177 | 0.999994883 |
| cellular response to arsenic-containing substa | 44576 | 0.839690177 | 0.999994883 |
| cellular sodium ion homeostasis (GO:000688 | 44576 | 0.839690177 | 0.999994883 |
| clathrin coat assembly (GO:0048268) | 44576 | 0.839690177 | 0.999994883 |
| dermatan sulfate proteoglycan biosynthetic p | 44576 | 0.839690177 | 0.999994883 |
| endoderm development (GO:0007492) | 44576 | 0.839690177 | 0.999994883 |
| engulfment of apoptotic cell (GO:0043652) | 44576 | 0.839690177 | 0.999994883 |
| establishment of spindle localization (GO:005 | 44576 | 0.839690177 | 0.999994883 |
| ganglioside biosynthetic process (GO:000157 | 44576 | 0.839690177 | 0.999994883 |
| GTP metabolic process (GO:0046039) | 44576 | 0.839690177 | 0.999994883 |
| heterocycle biosynthetic process (GO:001813 | 44576 | 0.839690177 | 0.999994883 |
| histone H4-K5 acetylation (GO:0043981) | 44576 | 0.839690177 | 0.999994883 |
| histone H4-K8 acetylation (GO:0043982) | 44576 | 0.839690177 | 0.999994883 |
| mammary gland epithelium development (GO | 44576 | 0.839690177 | 0.999994883 |
| membrane repolarization during cardiac mus | 44576 | 0.839690177 | 0.999994883 |
| microvillus assembly (GO:0030033) | 44576 | 0.839690177 | 0.999994883 |
| mRNA methylation (GO:0080009) | 44576 | 0.839690177 | 0.999994883 |
| mycotoxin metabolic process (GO:0043385) | 44576 | 0.839690177 | 0.999994883 |
| myoblast fusion (GO:0007520) | 44576 | 0.839690177 | 0.999994883 |
| negative regulation of androgen receptor sign | 44576 | 0.839690177 | 0.999994883 |
| negative regulation of endoplasmic reticulum | 44576 | 0.839690177 | 0.999994883 |
| negative regulation of ubiquitin-protein trans | 44576 | 0.839690177 | 0.999994883 |
| nucleosome positioning (GO:0016584) | 44576 | 0.839690177 | 0.999994883 |
| organic heteropentacyclic compound metabo | 44576 | 0.839690177 | 0.999994883 |
| peptidyl-arginine modification (GO:0018195) | 44576 | 0.839690177 | 0.999994883 |
| peptidyl-tyrosine autophosphorylation (GO:0 | 44576 | 0.839690177 | 0.999994883 |
| positive regulation of cell killing (GO:003134 | 44576 | 0.839690177 | 0.999994883 |
| positive regulation of chromatin organization | 44576 | 0.839690177 | 0.999994883 |
| positive regulation of DNA damage response, | 44576 | 0.839690177 | 0.999994883 |
| positive regulation of endothelial cell chemot | 44576 | 0.839690177 | 0.999994883 |
| positive regulation of exosomal secretion (GO | 44576 | 0.839690177 | 0.999994883 |
| positive regulation of glycogen metabolic pro | 44576 | 0.839690177 | 0.999994883 |
| positive regulation of lyase activity (GO:0051 | 44576 | 0.839690177 | 0.999994883 |
| positive regulation of pattern recognition rec | 44576 | 0.839690177 | 0.999994883 |
| positive regulation of response to cytokine st | 44576 | 0.839690177 | 0.999994883 |
| positive regulation of secretion (GO:0051047 | 44576 | 0.839690177 | 0.999994883 |
| protein localization to cell-cell junction (GO:0 | 44576 | 0.839690177 | 0.999994883 |

| | | | |
|---|---|---|---|
| regulation of endothelial cell chemotaxis (GO | 44576 | 0.839690177 | 0.999994883 |
| regulation of extracellular matrix disassembly | 44576 | 0.839690177 | 0.999994883 |
| regulation of non-canonical Wnt signaling pa | 44576 | 0.839690177 | 0.999994883 |
| regulation of secretion (GO:0051046) | 44576 | 0.839690177 | 0.999994883 |
| regulation of synaptic transmission, GABAerg | 44576 | 0.839690177 | 0.999994883 |
| regulation of T cell chemotaxis (GO:0010819) | 44576 | 0.839690177 | 0.999994883 |
| response to ethanol (GO:0045471) | 44576 | 0.839690177 | 0.999994883 |
| response to muscle stretch (GO:0035994) | 44576 | 0.839690177 | 0.999994883 |
| vitamin D metabolic process (GO:0042359) | 44576 | 0.839690177 | 0.999994883 |
| neuron projection development (GO:003117 | 16/171 | 0.840909207 | 0.999994883 |
| protein-containing complex assembly (GO:00 | 26/267 | 0.841053416 | 0.999994883 |
| endothelial cell migration (GO:0043542) | 14305 | 0.841165848 | 0.999994883 |
| negative regulation of protein secretion (GO: | 14305 | 0.841165848 | 0.999994883 |
| negative regulation of secretion by cell (GO:1 | 14305 | 0.841165848 | 0.999994883 |
| protein acylation (GO:0043543) | 14305 | 0.841165848 | 0.999994883 |
| regulation of peptidyl-threonine phosphoryla | 14305 | 0.841165848 | 0.999994883 |
| regulation of protein targeting to mitochondı | 14305 | 0.841165848 | 0.999994883 |
| response to peptide (GO:1901652) | 14305 | 0.841165848 | 0.999994883 |
| regulation of complement activation (GO:003 | 18354 | 0.841240039 | 0.999994883 |
| regulation of cytoskeleton organization (GO:( | 10/112 | 0.841708529 | 0.999994883 |
| potassium ion transport (GO:0006813) | 11/122 | 0.841852347 | 0.999994883 |
| regulation of mRNA catabolic process (GO:00 | 11/122 | 0.841852347 | 0.999994883 |
| regulation of cell death (GO:0010941) | 9/102 | 0.84211168 | 0.999994883 |
| retinoid metabolic process (GO:0001523) | 33817 | 0.843242611 | 0.999994883 |
| positive regulation of ERK1 and ERK2 cascade | 16/172 | 0.847228507 | 0.999994883 |
| internal peptidyl-lysine acetylation (GO:0018 | 44620 | 0.848015872 | 0.999994883 |
| leukocyte cell-cell adhesion (GO:0007159) | 44620 | 0.848015872 | 0.999994883 |
| negative regulation of cellular response to in: | 44620 | 0.848015872 | 0.999994883 |
| negative regulation of oxidative stress-induce | 44620 | 0.848015872 | 0.999994883 |
| nucleotide-binding oligomerization domain c | 44620 | 0.848015872 | 0.999994883 |
| positive regulation of cell communication (G( | 44620 | 0.848015872 | 0.999994883 |
| positive regulation of monooxygenase activit | 44620 | 0.848015872 | 0.999994883 |
| positive regulation of synapse assembly (GO: | 44620 | 0.848015872 | 0.999994883 |
| receptor clustering (GO:0043113) | 44620 | 0.848015872 | 0.999994883 |
| regulation of DNA-templated transcription, e | 44620 | 0.848015872 | 0.999994883 |
| regulation of multicellular organismal develo | 44620 | 0.848015872 | 0.999994883 |
| regulation of muscle contraction (GO:000693 | 44620 | 0.848015872 | 0.999994883 |
| regulation of phosphatase activity (GO:0010S | 44620 | 0.848015872 | 0.999994883 |
| response to ketone (GO:1901654) | 44620 | 0.848015872 | 0.999994883 |
| T cell activation involved in immune response | 44620 | 0.848015872 | 0.999994883 |
| ventricular septum morphogenesis (GO:0060 | 44620 | 0.848015872 | 0.999994883 |
| negative regulation of cytokine production (G | 17/182 | 0.848824265 | 0.999994883 |
| positive regulation of secretion by cell (GO:1S | 26451 | 0.848844929 | 0.999994883 |
| positive regulation of cell migration (GO:003( | 26/269 | 0.851188714 | 0.999994883 |
| blood circulation (GO:0008015) | 18719 | 0.852270751 | 0.999994883 |
| mesenchymal cell differentiation (GO:00487( | 18719 | 0.852270751 | 0.999994883 |
| negative regulation of protein ubiquitination | 18719 | 0.852270751 | 0.999994883 |

| | | | |
|---|---|---|---|
| positive regulation of actin filament bundle a | 18719 | 0.852270751 | 0.999994883 |
| regulation of axonogenesis (GO:0050770) | 18719 | 0.852270751 | 0.999994883 |
| sensory perception (GO:0007600) | 18719 | 0.852270751 | 0.999994883 |
| digestive tract development (GO:0048565) | 14671 | 0.853464727 | 0.999994883 |
| lymphocyte migration (GO:0072676) | 14671 | 0.853464727 | 0.999994883 |
| negative regulation of blood coagulation (GO | 14671 | 0.853464727 | 0.999994883 |
| positive regulation of cation channel activity | 14671 | 0.853464727 | 0.999994883 |
| positive regulation of ossification (GO:00457 | 14671 | 0.853464727 | 0.999994883 |
| response to organonitrogen compound (GO:( | 14671 | 0.853464727 | 0.999994883 |
| synaptic vesicle exocytosis (GO:0016079) | 14671 | 0.853464727 | 0.999994883 |
| positive regulation of interleukin-1 productio | 22767 | 0.854287101 | 0.999994883 |
| cellular metal ion homeostasis (GO:0006875) | 9/104 | 0.857742192 | 0.999994883 |
| mRNA transport (GO:0051028) | 9/104 | 0.857742192 | 0.999994883 |
| positive regulation of macromolecule metab( | 38/384 | 0.857825322 | 0.999994883 |
| regulation of actin filament polymerization (( | 26816 | 0.857897431 | 0.999994883 |
| regulation of actin filament-based process (G | 26816 | 0.857897431 | 0.999994883 |
| body fluid secretion (GO:0007589) | 44577 | 0.858115396 | 0.999994883 |
| cellular response to corticosteroid stimulus (( | 44577 | 0.858115396 | 0.999994883 |
| digestive tract morphogenesis (GO:0048546) | 44577 | 0.858115396 | 0.999994883 |
| eosinophil chemotaxis (GO:0048245) | 44577 | 0.858115396 | 0.999994883 |
| eosinophil migration (GO:0072677) | 44577 | 0.858115396 | 0.999994883 |
| filopodium assembly (GO:0046847) | 44577 | 0.858115396 | 0.999994883 |
| male meiosis I (GO:0007141) | 44577 | 0.858115396 | 0.999994883 |
| membrane repolarization (GO:0086009) | 44577 | 0.858115396 | 0.999994883 |
| mitotic sister chromatid cohesion (GO:00070 | 44577 | 0.858115396 | 0.999994883 |
| mRNA cis splicing, via spliceosome (GO:0045: | 44577 | 0.858115396 | 0.999994883 |
| N-terminal protein amino acid acetylation (G | 44577 | 0.858115396 | 0.999994883 |
| necroptotic process (GO:0070266) | 44577 | 0.858115396 | 0.999994883 |
| negative regulation of anoikis (GO:2000811) | 44577 | 0.858115396 | 0.999994883 |
| negative regulation of epithelial cell different | 44577 | 0.858115396 | 0.999994883 |
| negative regulation of macrophage activation | 44577 | 0.858115396 | 0.999994883 |
| odontogenesis of dentin-containing tooth (G( | 44577 | 0.858115396 | 0.999994883 |
| positive regulation of alpha-beta T cell prolife | 44577 | 0.858115396 | 0.999994883 |
| positive regulation of double-strand break re | 44577 | 0.858115396 | 0.999994883 |
| positive regulation of endothelial cell apopto | 44577 | 0.858115396 | 0.999994883 |
| positive regulation of myeloid leukocyte cyto | 44577 | 0.858115396 | 0.999994883 |
| positive regulation of vasoconstriction (GO:0 | 44577 | 0.858115396 | 0.999994883 |
| regulation of activin receptor signaling pathw | 44577 | 0.858115396 | 0.999994883 |
| regulation of mitotic sister chromatid separa | 44577 | 0.858115396 | 0.999994883 |
| regulation of phosphate metabolic process (( | 44577 | 0.858115396 | 0.999994883 |
| regulation of rRNA processing (GO:2000232) | 44577 | 0.858115396 | 0.999994883 |
| sympathetic nervous system development (G | 44577 | 0.858115396 | 0.999994883 |
| interleukin-1-mediated signaling pathway (G( | 34547 | 0.859521839 | 0.999994883 |
| negative regulation of MAPK cascade (GO:00 | 34547 | 0.859521839 | 0.999994883 |
| protein ubiquitination (GO:0016567) | 53/525 | 0.86102452 | 0.999994883 |
| actin filament polymerization (GO:0030041) | 47150 | 0.861721227 | 0.999994883 |
| aggrephagy (GO:0035973) | 47150 | 0.861721227 | 0.999994883 |

| | | | |
|---|---|---|---|
| positive regulation of extrinsic apoptotic sign | 47150 | 0.861721227 | 0.999994883 |
| positive regulation of leukocyte migration (G | 47150 | 0.861721227 | 0.999994883 |
| regulation of actin polymerization or depolyr | 47150 | 0.861721227 | 0.999994883 |
| regulation of ubiquitin-protein transferase ac | 47150 | 0.861721227 | 0.999994883 |
| response to cadmium ion (GO:0046686) | 47150 | 0.861721227 | 0.999994883 |
| anaphase-promoting complex-dependent cat | 30864 | 0.862318465 | 0.999994883 |
| regulation of release of sequestered calcium | 19085 | 0.862643732 | 0.999994883 |
| sprouting angiogenesis (GO:0002040) | 19085 | 0.862643732 | 0.999994883 |
| positive regulation of Ras protein signal trans | 23132 | 0.863708401 | 0.999994883 |
| acylglycerol metabolic process (GO:0006639) | 15036 | 0.864923009 | 0.999994883 |
| cortical cytoskeleton organization (GO:00308 | 15036 | 0.864923009 | 0.999994883 |
| heart contraction (GO:0060047) | 15036 | 0.864923009 | 0.999994883 |
| negative regulation of cell-cell adhesion (GO: | 15036 | 0.864923009 | 0.999994883 |
| positive regulation of biomineral tissue devel | 15036 | 0.864923009 | 0.999994883 |
| positive regulation of cation transmembrane | 15036 | 0.864923009 | 0.999994883 |
| regulation of cell development (GO:0060284) | 15036 | 0.864923009 | 0.999994883 |
| regulation of filopodium assembly (GO:00514 | 15036 | 0.864923009 | 0.999994883 |
| positive regulation of cellular metabolic proc | 9/105 | 0.865080353 | 0.999994883 |
| negative regulation of protein binding (GO:00 | 27181 | 0.866498267 | 0.999994883 |
| NIK/NF-kappaB signaling (GO:0038061) | 27181 | 0.866498267 | 0.999994883 |
| regulation of MAPK cascade (GO:0043408) | 15/166 | 0.869917329 | 0.999994883 |
| positive regulation of angiogenesis (GO:0045 | 10/116 | 0.87059241 | 0.999994883 |
| protein localization to nucleus (GO:0034504) | 9/106 | 0.872110204 | 0.999994883 |
| regulation of phosphatidylinositol 3-kinase si | 9/106 | 0.872110204 | 0.999994883 |
| RNA export from nucleus (GO:0006405) | 9/106 | 0.872110204 | 0.999994883 |
| bicellular tight junction assembly (GO:007083 | 19450 | 0.872386427 | 0.999994883 |
| cellular macromolecule catabolic process (GC | 19450 | 0.872386427 | 0.999994883 |
| negative regulation of cysteine-type endopep | 19450 | 0.872386427 | 0.999994883 |
| positive regulation of phagocytosis (GO:0050 | 19450 | 0.872386427 | 0.999994883 |
| regulation of immune effector process (GO:0 | 19450 | 0.872386427 | 0.999994883 |
| negative regulation of intrinsic apoptotic sign | 23498 | 0.872611909 | 0.999994883 |
| artery morphogenesis (GO:0048844) | 10990 | 0.874284832 | 0.999994883 |
| cardiac muscle cell action potential (GO:0086 | 10990 | 0.874284832 | 0.999994883 |
| histone H3-K4 methylation (GO:0051568) | 10990 | 0.874284832 | 0.999994883 |
| multivesicular body assembly (GO:0036258) | 10990 | 0.874284832 | 0.999994883 |
| phosphatidylcholine acyl-chain remodeling (C | 10990 | 0.874284832 | 0.999994883 |
| positive regulation of phosphate metabolic p | 10990 | 0.874284832 | 0.999994883 |
| regulation of long-term synaptic potentiation | 10990 | 0.874284832 | 0.999994883 |
| regulation of macrophage activation (GO:004 | 10990 | 0.874284832 | 0.999994883 |
| regulation of vasoconstriction (GO:0019229) | 10990 | 0.874284832 | 0.999994883 |
| termination of RNA polymerase I transcriptio | 10990 | 0.874284832 | 0.999994883 |
| cellular biogenic amine biosynthetic process | 44578 | 0.874423701 | 0.999994883 |
| establishment of protein localization to endo | 44578 | 0.874423701 | 0.999994883 |
| gliogenesis (GO:0042063) | 44578 | 0.874423701 | 0.999994883 |
| maturation of 5.8S rRNA (GO:0000460) | 44578 | 0.874423701 | 0.999994883 |
| membrane depolarization during cardiac mus | 44578 | 0.874423701 | 0.999994883 |
| mesonephric tubule development (GO:00721 | 44578 | 0.874423701 | 0.999994883 |

| | | | |
|---|---|---|---|
| mitochondrial electron transport, cytochrom | 44578 | 0.874423701 | 0.999994883 |
| muscle tissue morphogenesis (GO:0060415) | 44578 | 0.874423701 | 0.999994883 |
| negative regulation of fatty acid metabolic pr | 44578 | 0.874423701 | 0.999994883 |
| negative regulation of peptidyl-threonine pho | 44578 | 0.874423701 | 0.999994883 |
| pharyngeal system development (GO:006003 | 44578 | 0.874423701 | 0.999994883 |
| positive regulation of blood coagulation (GO: | 44578 | 0.874423701 | 0.999994883 |
| positive regulation of cardiac muscle tissue g | 44578 | 0.874423701 | 0.999994883 |
| positive regulation of cytokine production inv | 44578 | 0.874423701 | 0.999994883 |
| positive regulation of positive chemotaxis (G( | 44578 | 0.874423701 | 0.999994883 |
| positive regulation of stem cell differentiatio | 44578 | 0.874423701 | 0.999994883 |
| positive regulation of stem cell proliferation ( | 44578 | 0.874423701 | 0.999994883 |
| protein autoprocessing (GO:0016540) | 44578 | 0.874423701 | 0.999994883 |
| regulation of cell killing (GO:0031341) | 44578 | 0.874423701 | 0.999994883 |
| regulation of Golgi organization (GO:190335 | 44578 | 0.874423701 | 0.999994883 |
| regulation of osteoblast proliferation (GO:00 | 44578 | 0.874423701 | 0.999994883 |
| regulation of steroid biosynthetic process (G( | 44578 | 0.874423701 | 0.999994883 |
| ruffle organization (GO:0031529) | 44578 | 0.874423701 | 0.999994883 |
| stabilization of membrane potential (GO:003 | 44578 | 0.874423701 | 0.999994883 |
| sulfation (GO:0051923) | 44578 | 0.874423701 | 0.999994883 |
| positive regulation of immune response (GO: | 27546 | 0.874661807 | 0.999994883 |
| regulation of hematopoietic progenitor cell d | 27546 | 0.874661807 | 0.999994883 |
| histone H3 acetylation (GO:0043966) | 15401 | 0.875584392 | 0.999994883 |
| positive regulation of epithelial to mesenchy | 15401 | 0.875584392 | 0.999994883 |
| negative regulation of nucleic acid-templatec | 46/464 | 0.875822906 | 0.999994883 |
| positive regulation of developmental process | 16/177 | 0.875988534 | 0.999994883 |
| positive regulation of cell projection organiza | 10/117 | 0.877108608 | 0.999994883 |
| negative regulation of cellular amide metabo | 31594 | 0.877724152 | 0.999994883 |
| mRNA export from nucleus (GO:0006406) | 9/107 | 0.87883942 | 0.999994883 |
| protein polyubiquitination (GO:0000209) | 30/314 | 0.881129321 | 0.999994883 |
| regulation of humoral immune response (GO | 19815 | 0.881526516 | 0.999994883 |
| nervous system development (GO:0007399) | 44/447 | 0.881809712 | 0.999994883 |
| chloride transport (GO:0006821) | 27912 | 0.882402756 | 0.999994883 |
| regulation of epithelial to mesenchymal trans | 27912 | 0.882402756 | 0.999994883 |
| RNA transport (GO:0050658) | 27912 | 0.882402756 | 0.999994883 |
| cellular glucose homeostasis (GO:0001678) | 15766 | 0.885492135 | 0.999994883 |
| myofibril assembly (GO:0030239) | 15766 | 0.885492135 | 0.999994883 |
| positive regulation of peptide hormone secre | 15766 | 0.885492135 | 0.999994883 |
| cardiac muscle cell action potential involved | 11355 | 0.885788035 | 0.999994883 |
| multivesicular body organization (GO:003625 | 11355 | 0.885788035 | 0.999994883 |
| negative regulation of interleukin-6 productio | 11355 | 0.885788035 | 0.999994883 |
| negative regulation of response to biotic stim | 11355 | 0.885788035 | 0.999994883 |
| organelle transport along microtubule (GO:0( | 11355 | 0.885788035 | 0.999994883 |
| positive regulation of RNA splicing (GO:0033 | 11355 | 0.885788035 | 0.999994883 |
| receptor signaling pathway via JAK-STAT (GO | 11355 | 0.885788035 | 0.999994883 |
| regulation of cellular pH (GO:0030641) | 11355 | 0.885788035 | 0.999994883 |
| regulation of potassium ion transport (GO:00 | 11355 | 0.885788035 | 0.999994883 |
| alternative mRNA splicing, via spliceosome (( | 44579 | 0.888858213 | 0.999994883 |

| | | | |
|---|---|---|---|
| cell migration involved in sprouting angiogen | 44579 | 0.888858213 | 0.999994883 |
| cellular response to glucocorticoid stimulus ( | 44579 | 0.888858213 | 0.999994883 |
| cellular response to zinc ion (GO:0071294) | 44579 | 0.888858213 | 0.999994883 |
| embryonic digit morphogenesis (GO:004273 | 44579 | 0.888858213 | 0.999994883 |
| endothelial cell proliferation (GO:0001935) | 44579 | 0.888858213 | 0.999994883 |
| epithelial structure maintenance (GO:001066 | 44579 | 0.888858213 | 0.999994883 |
| establishment of vesicle localization (GO:005 | 44579 | 0.888858213 | 0.999994883 |
| leukocyte tethering or rolling (GO:0050901) | 44579 | 0.888858213 | 0.999994883 |
| long-chain fatty-acyl-CoA biosynthetic proces | 44579 | 0.888858213 | 0.999994883 |
| maintenance of gastrointestinal epithelium ( | 44579 | 0.888858213 | 0.999994883 |
| mitochondrial fission (GO:0000266) | 44579 | 0.888858213 | 0.999994883 |
| phosphatidylglycerol acyl-chain remodeling ( | 44579 | 0.888858213 | 0.999994883 |
| phosphatidylinositol acyl-chain remodeling ( | 44579 | 0.888858213 | 0.999994883 |
| positive regulation of cell adhesion mediated | 44579 | 0.888858213 | 0.999994883 |
| positive regulation of cell cycle phase transiti | 44579 | 0.888858213 | 0.999994883 |
| positive regulation of lymphocyte chemotaxi | 44579 | 0.888858213 | 0.999994883 |
| positive regulation of mRNA splicing, via splic | 44579 | 0.888858213 | 0.999994883 |
| positive regulation of response to endoplasm | 44579 | 0.888858213 | 0.999994883 |
| regulation of B cell differentiation (GO:00455 | 44579 | 0.888858213 | 0.999994883 |
| regulation of brown fat cell differentiation (G | 44579 | 0.888858213 | 0.999994883 |
| regulation of cartilage development (GO:006 | 44579 | 0.888858213 | 0.999994883 |
| regulation of catabolic process (GO:0009894) | 44579 | 0.888858213 | 0.999994883 |
| regulation of early endosome to late endoso | 44579 | 0.888858213 | 0.999994883 |
| regulation of production of miRNAs involved | 44579 | 0.888858213 | 0.999994883 |
| regulation of protein sumoylation (GO:00332 | 44579 | 0.888858213 | 0.999994883 |
| regulation of transforming growth factor bet | 44579 | 0.888858213 | 0.999994883 |
| regulation of ventricular cardiac muscle cell r | 44579 | 0.888858213 | 0.999994883 |
| response to interferon-alpha (GO:0035455) | 44579 | 0.888858213 | 0.999994883 |
| rRNA transcription (GO:0009303) | 44579 | 0.888858213 | 0.999994883 |
| T cell migration (GO:0072678) | 44579 | 0.888858213 | 0.999994883 |
| tRNA metabolic process (GO:0006399) | 44579 | 0.888858213 | 0.999994883 |
| positive regulation of cell death (GO:001094 | 24228 | 0.888943751 | 0.999994883 |
| positive regulation of phosphatidylinositol 3- | 28277 | 0.889736049 | 0.999994883 |
| negative regulation of protein modification b | 20180 | 0.890091698 | 0.999994883 |
| positive regulation of response to DNA dama | 20180 | 0.890091698 | 0.999994883 |
| brain development (GO:0007420) | 13/150 | 0.891563782 | 0.999994883 |
| metal ion homeostasis (GO:0055065) | 16132 | 0.894688759 | 0.999994883 |
| mRNA stabilization (GO:0048255) | 16132 | 0.894688759 | 0.999994883 |
| protein K63-linked ubiquitination (GO:00705 | 16132 | 0.894688759 | 0.999994883 |
| regulation of endothelial cell apoptotic proce | 16132 | 0.894688759 | 0.999994883 |
| negative regulation of cell differentiation (GC | 17/191 | 0.895912279 | 0.999994883 |
| aortic valve morphogenesis (GO:0003180) | 11720 | 0.896308565 | 0.999994883 |
| B cell proliferation (GO:0042100) | 11720 | 0.896308565 | 0.999994883 |
| cellular response to glucose stimulus (GO:00 | 11720 | 0.896308565 | 0.999994883 |
| hematopoietic progenitor cell differentiation | 11720 | 0.896308565 | 0.999994883 |
| liver development (GO:0001889) | 11720 | 0.896308565 | 0.999994883 |
| membrane depolarization (GO:0051899) | 11720 | 0.896308565 | 0.999994883 |

| | | | |
|---|---|---|---|
| negative regulation of chemotaxis (GO:0050! | 11720 | 0.896308565 | 0.999994883 |
| positive regulation of glucose transmembran | 11720 | 0.896308565 | 0.999994883 |
| positive regulation of interleukin-10 producti | 11720 | 0.896308565 | 0.999994883 |
| protein K63-linked deubiquitination (GO:007( | 11720 | 0.896308565 | 0.999994883 |
| skeletal muscle organ development (GO:006( | 11720 | 0.896308565 | 0.999994883 |
| transcription initiation from RNA polymerase | 11720 | 0.896308565 | 0.999994883 |
| positive regulation of catabolic process (GO:( | 24593 | 0.896411573 | 0.999994883 |
| regulation of epidermal growth factor recept | 24593 | 0.896411573 | 0.999994883 |
| negative regulation of protein serine/threoni | 28642 | 0.896676761 | 0.999994883 |
| autophagosome organization (GO:1905037) | 20546 | 0.898109507 | 0.999994883 |
| mitochondrial transport (GO:0006839) | 20546 | 0.898109507 | 0.999994883 |
| positive regulation of interleukin-1 beta prod | 20546 | 0.898109507 | 0.999994883 |
| regulation of type I interferon production (G( | 32690 | 0.898122727 | 0.999994883 |
| activin receptor signaling pathway (GO:0032! | 44580 | 0.901634146 | 0.999994883 |
| atrioventricular valve morphogenesis (GO:00 | 44580 | 0.901634146 | 0.999994883 |
| branching involved in ureteric bud morphoge | 44580 | 0.901634146 | 0.999994883 |
| cardiac atrium morphogenesis (GO:0003209) | 44580 | 0.901634146 | 0.999994883 |
| cellular response to copper ion (GO:0071280 | 44580 | 0.901634146 | 0.999994883 |
| dopamine metabolic process (GO:0042417) | 44580 | 0.901634146 | 0.999994883 |
| endoplasmic reticulum mannose trimming (G | 44580 | 0.901634146 | 0.999994883 |
| establishment or maintenance of apical/basa | 44580 | 0.901634146 | 0.999994883 |
| glycerolipid catabolic process (GO:0046503) | 44580 | 0.901634146 | 0.999994883 |
| heart trabecula morphogenesis (GO:0061384 | 44580 | 0.901634146 | 0.999994883 |
| leukocyte adhesion to vascular endothelial ce | 44580 | 0.901634146 | 0.999994883 |
| long-term memory (GO:0007616) | 44580 | 0.901634146 | 0.999994883 |
| myeloid cell activation involved in immune re | 44580 | 0.901634146 | 0.999994883 |
| negative regulation of immune system proce | 44580 | 0.901634146 | 0.999994883 |
| negative regulation of oxidative stress-induce | 44580 | 0.901634146 | 0.999994883 |
| negative regulation of RNA metabolic proces: | 44580 | 0.901634146 | 0.999994883 |
| negative regulation of T cell receptor signalin | 44580 | 0.901634146 | 0.999994883 |
| nuclear membrane organization (GO:007176 | 44580 | 0.901634146 | 0.999994883 |
| positive regulation of establishment of prote | 44580 | 0.901634146 | 0.999994883 |
| positive regulation of G protein-coupled rece | 44580 | 0.901634146 | 0.999994883 |
| positive regulation of macrophage migration | 44580 | 0.901634146 | 0.999994883 |
| positive regulation of mitochondrial fission (( | 44580 | 0.901634146 | 0.999994883 |
| positive regulation of neutrophil chemotaxis | 44580 | 0.901634146 | 0.999994883 |
| protein import into mitochondrial matrix (GC | 44580 | 0.901634146 | 0.999994883 |
| regulation of bicellular tight junction assembl | 44580 | 0.901634146 | 0.999994883 |
| regulation of collagen biosynthetic process (( | 44580 | 0.901634146 | 0.999994883 |
| regulation of exosomal secretion (GO:190354 | 44580 | 0.901634146 | 0.999994883 |
| regulation of glucose transmembrane transp( | 44580 | 0.901634146 | 0.999994883 |
| regulation of synaptic vesicle exocytosis (GO: | 44580 | 0.901634146 | 0.999994883 |
| regulation of vascular endothelial growth fac | 44580 | 0.901634146 | 0.999994883 |
| striated muscle cell differentiation (GO:0051: | 44580 | 0.901634146 | 0.999994883 |
| ureteric bud morphogenesis (GO:0060675) | 44580 | 0.901634146 | 0.999994883 |
| membrane organization (GO:0061024) | 22/242 | 0.902467795 | 0.999994883 |
| cellular divalent inorganic cation homeostasi: | 9/111 | 0.902910568 | 0.999994883 |

| | | | |
|---|---|---|---|
| action potential (GO:0001508) | 16497 | 0.903215795 | 0.999994883 |
| sodium ion transport (GO:0006814) | 33055 | 0.904247377 | 0.999994883 |
| carboxylic acid transport (GO:0046942) | 20911 | 0.905607151 | 0.999994883 |
| protein K48-linked ubiquitination (GO:00709: | 20911 | 0.905607151 | 0.999994883 |
| antigen processing and presentation of pepti | 12086 | 0.905920293 | 0.999994883 |
| regulation of sprouting angiogenesis (GO:190 | 12086 | 0.905920293 | 0.999994883 |
| regulation of canonical Wnt signaling pathwa | 23/253 | 0.906848156 | 0.999994883 |
| neuron development (GO:0048666) | 12/143 | 0.907326826 | 0.999994883 |
| negative regulation of multicellular organism | 19/214 | 0.908810691 | 0.999994883 |
| negative regulation of extrinsic apoptotic sigr | 29373 | 0.909440941 | 0.999994883 |
| heart development (GO:0007507) | 14/164 | 0.909845886 | 0.999994883 |
| negative regulation of cellular catabolic proce | 25324 | 0.910049626 | 0.999994883 |
| synapse assembly (GO:0007416) | 25324 | 0.910049626 | 0.999994883 |
| regulation of stem cell differentiation (GO:20 | 33420 | 0.910054741 | 0.999994883 |
| cellular response to organic substance (GO:0( | 10/123 | 0.910801539 | 0.999994883 |
| erythrocyte differentiation (GO:0030218) | 16862 | 0.911113584 | 0.999994883 |
| positive regulation of organelle assembly (GC | 16862 | 0.911113584 | 0.999994883 |
| positive regulation of response to stimulus (G | 16862 | 0.911113584 | 0.999994883 |
| posttranscriptional regulation of gene expres | 16862 | 0.911113584 | 0.999994883 |
| vesicle organization (GO:0016050) | 16862 | 0.911113584 | 0.999994883 |
| eye development (GO:0001654) | 21276 | 0.912611375 | 0.999994883 |
| regulation of phagocytosis (GO:0050764) | 21276 | 0.912611375 | 0.999994883 |
| RNA modification (GO:0009451) | 21276 | 0.912611375 | 0.999994883 |
| adaptive immune response based on somatic | 44581 | 0.912942 | 0.999994883 |
| calcium-independent cell-cell adhesion via pl | 44581 | 0.912942 | 0.999994883 |
| cardiac epithelial to mesenchymal transition | 44581 | 0.912942 | 0.999994883 |
| cardiac ventricle development (GO:0003231) | 44581 | 0.912942 | 0.999994883 |
| cellular monovalent inorganic cation homeos | 44581 | 0.912942 | 0.999994883 |
| cellular response to misfolded protein (GO:0( | 44581 | 0.912942 | 0.999994883 |
| cortical actin cytoskeleton organization (GO:( | 44581 | 0.912942 | 0.999994883 |
| lipopolysaccharide-mediated signaling pathw | 44581 | 0.912942 | 0.999994883 |
| maintenance of protein location in nucleus (( | 44581 | 0.912942 | 0.999994883 |
| mesoderm formation (GO:0001707) | 44581 | 0.912942 | 0.999994883 |
| mitotic cytokinetic process (GO:1902410) | 44581 | 0.912942 | 0.999994883 |
| modulation by symbiont of entry into host (G | 44581 | 0.912942 | 0.999994883 |
| natural killer cell mediated immunity (GO:00( | 44581 | 0.912942 | 0.999994883 |
| negative regulation of dephosphorylation (G( | 44581 | 0.912942 | 0.999994883 |
| neuromuscular synaptic transmission (GO:00 | 44581 | 0.912942 | 0.999994883 |
| positive regulation of embryonic developmer | 44581 | 0.912942 | 0.999994883 |
| positive regulation of epidermal cell different | 44581 | 0.912942 | 0.999994883 |
| positive regulation of heart contraction (GO:( | 44581 | 0.912942 | 0.999994883 |
| post-transcriptional gene silencing by RNA (G | 44581 | 0.912942 | 0.999994883 |
| regulation of hippo signaling (GO:0035330) | 44581 | 0.912942 | 0.999994883 |
| regulation of lymphocyte differentiation (GO | 44581 | 0.912942 | 0.999994883 |
| regulation of protein kinase A signaling (GO:( | 44581 | 0.912942 | 0.999994883 |
| regulation of protein modification by small pi | 44581 | 0.912942 | 0.999994883 |
| regulation of tissue remodeling (GO:0034103 | 44581 | 0.912942 | 0.999994883 |

| | | | |
|---|---|---|---|
| negative regulation of transcription by RNA p | 68/684 | 0.913478994 | 0.999994883 |
| positive regulation of peptidyl-tyrosine phosp | 11/134 | 0.913522682 | 0.999994883 |
| autophagosome maturation (GO:0097352) | 12451 | 0.914693091 | 0.999994883 |
| B cell receptor signaling pathway (GO:005085 | 12451 | 0.914693091 | 0.999994883 |
| cardiac muscle contraction (GO:0060048) | 12451 | 0.914693091 | 0.999994883 |
| cellular response to vascular endothelial grov | 12451 | 0.914693091 | 0.999994883 |
| epithelial tube morphogenesis (GO:0060562) | 12451 | 0.914693091 | 0.999994883 |
| protein destabilization (GO:0031648) | 12451 | 0.914693091 | 0.999994883 |
| regulation of cell adhesion mediated by integ | 12451 | 0.914693091 | 0.999994883 |
| regulation of lamellipodium assembly (GO:00 | 12451 | 0.914693091 | 0.999994883 |
| regulation of myoblast differentiation (GO:00 | 12451 | 0.914693091 | 0.999994883 |
| secondary alcohol biosynthetic process (GO:1 | 12451 | 0.914693091 | 0.999994883 |
| synaptic vesicle recycling (GO:0036465) | 12451 | 0.914693091 | 0.999994883 |
| termination of RNA polymerase II transcriptic | 12451 | 0.914693091 | 0.999994883 |
| transcription by RNA polymerase I (GO:00063 | 12451 | 0.914693091 | 0.999994883 |
| ventricular cardiac muscle tissue morphogen | 12451 | 0.914693091 | 0.999994883 |
| positive regulation of transmembrane recept | 33786 | 0.915556901 | 0.999994883 |
| T cell activation (GO:0042110) | 33786 | 0.915556901 | 0.999994883 |
| DNA-templated transcription, termination (G | 25689 | 0.916258826 | 0.999994883 |
| kidney development (GO:0001822) | 25689 | 0.916258826 | 0.999994883 |
| snRNA transcription by RNA polymerase II (G | 25689 | 0.916258826 | 0.999994883 |
| positive regulation of DNA-binding transcript | 22/246 | 0.916827754 | 0.999994883 |
| positive regulation of cell-cell adhesion (GO:0 | 17227 | 0.918421126 | 0.999994883 |
| regulation of wound healing (GO:0061041) | 17227 | 0.918421126 | 0.999994883 |
| regulation of secretion by cell (GO:1903530) | 21641 | 0.919148346 | 0.999994883 |
| selective autophagy (GO:0061912) | 21641 | 0.919148346 | 0.999994883 |
| cell-cell junction organization (GO:0045216) | 30103 | 0.920815695 | 0.999994883 |
| regulation of G protein-coupled receptor sigr | 30103 | 0.920815695 | 0.999994883 |
| response to metal ion (GO:0010038) | 26054 | 0.922086988 | 0.999994883 |
| snRNA transcription (GO:0009301) | 26054 | 0.922086988 | 0.999994883 |
| protein localization to plasma membrane (GO | 11/136 | 0.922305213 | 0.999994883 |
| cholesterol biosynthetic process (GO:000669 | 12816 | 0.922692778 | 0.999994883 |
| lung development (GO:0030324) | 12816 | 0.922692778 | 0.999994883 |
| negative regulation of Ras protein signal tran | 12816 | 0.922692778 | 0.999994883 |
| peptidyl-tyrosine dephosphorylation (GO:003 | 12816 | 0.922692778 | 0.999994883 |
| positive regulation of lymphocyte activation ( | 12816 | 0.922692778 | 0.999994883 |
| regulation of mitotic spindle organization (GO | 12816 | 0.922692778 | 0.999994883 |
| regulation of postsynaptic membrane potent | 12816 | 0.922692778 | 0.999994883 |
| regulation of regulated secretory pathway (G | 12816 | 0.922692778 | 0.999994883 |
| regulation of T cell receptor signaling pathwa | 12816 | 0.922692778 | 0.999994883 |
| regulation of trans-synaptic signaling (GO:009 | 12816 | 0.922692778 | 0.999994883 |
| actin nucleation (GO:0045010) | 44582 | 0.922950408 | 0.999994883 |
| anterior/posterior axis specification (GO:000 | 44582 | 0.922950408 | 0.999994883 |
| beta-catenin destruction complex disassemb | 44582 | 0.922950408 | 0.999994883 |
| calcium-mediated signaling using intracellula | 44582 | 0.922950408 | 0.999994883 |
| cyclic purine nucleotide metabolic process (G | 44582 | 0.922950408 | 0.999994883 |
| endocardial cushion development (GO:00031 | 44582 | 0.922950408 | 0.999994883 |

| | | | |
|---|---|---|---|
| epithelial cell development (GO:0002064) | 44582 | 0.922950408 | 0.999994883 |
| focal adhesion assembly (GO:0048041) | 44582 | 0.922950408 | 0.999994883 |
| histone ubiquitination (GO:0016574) | 44582 | 0.922950408 | 0.999994883 |
| innate immune response in mucosa (GO:000: | 44582 | 0.922950408 | 0.999994883 |
| long-term synaptic potentiation (GO:006029: | 44582 | 0.922950408 | 0.999994883 |
| mitotic spindle assembly checkpoint signaling | 44582 | 0.922950408 | 0.999994883 |
| mitotic spindle checkpoint signaling (GO:007: | 44582 | 0.922950408 | 0.999994883 |
| natural killer cell activation involved in immu | 44582 | 0.922950408 | 0.999994883 |
| negative regulation of cell communication (G | 44582 | 0.922950408 | 0.999994883 |
| negative regulation of peptidyl-serine phospl | 44582 | 0.922950408 | 0.999994883 |
| nuclear-transcribed mRNA poly(A) tail shorte | 44582 | 0.922950408 | 0.999994883 |
| positive regulation of immune system proces | 44582 | 0.922950408 | 0.999994883 |
| positive regulation of transcription from RNA | 44582 | 0.922950408 | 0.999994883 |
| regulation of dendritic spine development (G | 44582 | 0.922950408 | 0.999994883 |
| regulation of fibroblast migration (GO:00107 | 44582 | 0.922950408 | 0.999994883 |
| response to progesterone (GO:0032570) | 44582 | 0.922950408 | 0.999994883 |
| retinol metabolic process (GO:0042572) | 44582 | 0.922950408 | 0.999994883 |
| spindle assembly checkpoint signaling (GO:00 | 44582 | 0.922950408 | 0.999994883 |
| stress granule assembly (GO:0034063) | 44582 | 0.922950408 | 0.999994883 |
| central nervous system development (GO:00 | 24/268 | 0.923876124 | 0.999994883 |
| positive regulation of cytosolic calcium ion cc | 12/147 | 0.924604243 | 0.999994883 |
| apical junction assembly (GO:0043297) | 22007 | 0.92524356 | 0.999994883 |
| cellular response to chemokine (GO:1990869 | 22007 | 0.92524356 | 0.999994883 |
| ribonucleoprotein complex biogenesis (GO:0( | 22007 | 0.92524356 | 0.999994883 |
| regulation of interleukin-1 beta production (( | 30468 | 0.926019075 | 0.999994883 |
| cellular response to growth factor stimulus (( | 13/158 | 0.926981833 | 0.999994883 |
| negative regulation of epithelial cell prolifera | 26420 | 0.927552795 | 0.999994883 |
| activation of adenylate cyclase activity (GO:0 | 13181 | 0.929981126 | 0.999994883 |
| aortic valve development (GO:0003176) | 13181 | 0.929981126 | 0.999994883 |
| cellular response to heat (GO:0034605) | 13181 | 0.929981126 | 0.999994883 |
| membrane fusion (GO:0061025) | 13181 | 0.929981126 | 0.999994883 |
| negative regulation of toll-like receptor signa | 13181 | 0.929981126 | 0.999994883 |
| regulation of defense response to virus by hc | 13181 | 0.929981126 | 0.999994883 |
| regulation of ossification (GO:0030278) | 13181 | 0.929981126 | 0.999994883 |
| mRNA metabolic process (GO:0016071) | 34881 | 0.930351639 | 0.999994883 |
| cellular response to peptide hormone stimulu | 8/106 | 0.930594758 | 0.999994883 |
| cellular defense response (GO:0006968) | 17958 | 0.93141406 | 0.999994883 |
| response to glucose (GO:0009749) | 17958 | 0.93141406 | 0.999994883 |
| cellular response to nerve growth factor stim | 44583 | 0.931808638 | 0.999994883 |
| exogenous drug catabolic process (GO:00427 | 44583 | 0.931808638 | 0.999994883 |
| female gonad development (GO:0008585) | 44583 | 0.931808638 | 0.999994883 |
| mRNA modification (GO:0016556) | 44583 | 0.931808638 | 0.999994883 |
| negative regulation of B cell activation (GO:0 | 44583 | 0.931808638 | 0.999994883 |
| negative regulation of biomineral tissue deve | 44583 | 0.931808638 | 0.999994883 |
| negative regulation of DNA recombination at | 44583 | 0.931808638 | 0.999994883 |
| negative regulation of mitotic metaphase/an | 44583 | 0.931808638 | 0.999994883 |
| negative regulation of phosphate metabolic | | 44583 | 0.931808638 | 0.999994883 |

| | | | |
|---|---|---|---|
| negative regulation of small molecule metab | 44583 | 0.931808638 | 0.999994883 |
| peptidyl-threonine dephosphorylation (GO:0 | 44583 | 0.931808638 | 0.999994883 |
| positive regulation of dendritic spine develop | 44583 | 0.931808638 | 0.999994883 |
| positive regulation of granulocyte chemotaxi | 44583 | 0.931808638 | 0.999994883 |
| positive regulation of neutrophil migration (G | 44583 | 0.931808638 | 0.999994883 |
| positive regulation of translational initiation ( | 44583 | 0.931808638 | 0.999994883 |
| regulation of cardiac muscle cell membrane r | 44583 | 0.931808638 | 0.999994883 |
| regulation of chromosome organization (GO: | 44583 | 0.931808638 | 0.999994883 |
| regulation of DNA recombination at telomere | 44583 | 0.931808638 | 0.999994883 |
| regulation of membrane protein ectodomain | 44583 | 0.931808638 | 0.999994883 |
| regulation of viral release from host cell (GO: | 44583 | 0.931808638 | 0.999994883 |
| regulation of voltage-gated calcium channel a | 44583 | 0.931808638 | 0.999994883 |
| regulation of cell migration (GO:0030334) | 38/408 | 0.932360072 | 0.999994883 |
| positive regulation of synaptic transmission ( | 26785 | 0.932674494 | 0.999994883 |
| potassium ion transmembrane transport (GO | 11/139 | 0.934051073 | 0.999994883 |
| regulation of protein binding (GO:0043393) | 9/118 | 0.935364734 | 0.999994883 |
| I-kappaB kinase/NF-kappaB signaling (GO:00 | 22737 | 0.936206891 | 0.999994883 |
| stress-activated MAPK cascade (GO:0051403 | 22737 | 0.936206891 | 0.999994883 |
| positive regulation of exocytosis (GO:004592 | 13547 | 0.936615926 | 0.999994883 |
| positive regulation of signaling receptor activ | 13547 | 0.936615926 | 0.999994883 |
| regulation of intracellular pH (GO:0051453) | 13547 | 0.936615926 | 0.999994883 |
| skeletal muscle tissue development (GO:000 | 13547 | 0.936615926 | 0.999994883 |
| actin polymerization or depolymerization (GO | 18323 | 0.937169824 | 0.999994883 |
| regulation of cellular component movement | 18323 | 0.937169824 | 0.999994883 |
| regulation of cytokine-mediated signaling pat | 27150 | 0.937469855 | 0.999994883 |
| regulation of MAP kinase activity (GO:00434 | 35612 | 0.938905206 | 0.999994883 |
| artery development (GO:0060840) | 44584 | 0.939648819 | 0.999994883 |
| blood vessel endothelial cell migration (GO:0 | 44584 | 0.939648819 | 0.999994883 |
| drug catabolic process (GO:0042737) | 44584 | 0.939648819 | 0.999994883 |
| ear morphogenesis (GO:0042471) | 44584 | 0.939648819 | 0.999994883 |
| ERK1 and ERK2 cascade (GO:0070371) | 44584 | 0.939648819 | 0.999994883 |
| negative regulation of extrinsic apoptotic sig | 44584 | 0.939648819 | 0.999994883 |
| negative regulation of signal transduction in a | 44584 | 0.939648819 | 0.999994883 |
| positive regulation of mRNA processing (GO: | 44584 | 0.939648819 | 0.999994883 |
| positive regulation of transcription by RNA po | 44584 | 0.939648819 | 0.999994883 |
| regulation of cysteine-type endopeptidase ac | 44584 | 0.939648819 | 0.999994883 |
| regulation of interferon-gamma-mediated sig | 44584 | 0.939648819 | 0.999994883 |
| regulation of mononuclear cell migration (GO | 44584 | 0.939648819 | 0.999994883 |
| regulation of necroptotic process (GO:00605 | 44584 | 0.939648819 | 0.999994883 |
| regulation of neural precursor cell proliferati | 44584 | 0.939648819 | 0.999994883 |
| regulation of toll-like receptor 4 signaling pat | 44584 | 0.939648819 | 0.999994883 |
| respiratory chain complex IV assembly (GO:0 | 44584 | 0.939648819 | 0.999994883 |
| response to copper ion (GO:0046688) | 44584 | 0.939648819 | 0.999994883 |
| response to nutrient levels (GO:0031667) | 44584 | 0.939648819 | 0.999994883 |
| negative regulation of peptidase activity (GO | 23102 | 0.941122022 | 0.999994883 |
| positive regulation of T cell activation (GO:00 | 27515 | 0.941956125 | 0.999994883 |
| ubiquitin-dependent protein catabolic proces | 32/354 | 0.942137449 | 0.999994883 |

| | | | |
|---|---|---|---|
| positive regulation of fat cell differentiation ( | 18688 | 0.942475992 | 0.999994883 |
| regulation of interleukin-12 production (GO:( | 18688 | 0.942475992 | 0.999994883 |
| modulation of chemical synaptic transmissio | 8/109 | 0.942484327 | 0.999994883 |
| negative regulation of innate immune respor | 13912 | 0.94265108 | 0.999994883 |
| positive regulation of signaling (GO:0023056) | 13912 | 0.94265108 | 0.999994883 |
| regulation of cell communication (GO:00106 | 13912 | 0.94265108 | 0.999994883 |
| regulation of phosphoprotein phosphatase a | 13912 | 0.94265108 | 0.999994883 |
| sterol biosynthetic process (GO:0016126) | 13912 | 0.94265108 | 0.999994883 |
| ventricular septum development (GO:00032 | 13912 | 0.94265108 | 0.999994883 |
| cation transport (GO:0006812) | 14/174 | 0.945224587 | 0.999994883 |
| positive regulation of Wnt signaling pathway | 12/153 | 0.945353853 | 0.999994883 |
| plasma membrane organization (GO:000700 | 23468 | 0.945689342 | 0.999994883 |
| regulation of interleukin-6 production (GO:0 | 8/110 | 0.946027932 | 0.999994883 |
| positive regulation of interleukin-6 productio | 27881 | 0.946150006 | 0.999994883 |
| regulation of BMP signaling pathway (GO:00 | 27881 | 0.946150006 | 0.999994883 |
| regulation of RNA splicing (GO:0043484) | 27881 | 0.946150006 | 0.999994883 |
| organic anion transport (GO:0015711) | 36342 | 0.946516335 | 0.999994883 |
| amino acid import across plasma membrane | 44585 | 0.946587905 | 0.999994883 |
| dorsal/ventral pattern formation (GO:00099 | 44585 | 0.946587905 | 0.999994883 |
| long-chain fatty-acyl-CoA metabolic process ( | 44585 | 0.946587905 | 0.999994883 |
| lymphocyte activation involved in immune re | 44585 | 0.946587905 | 0.999994883 |
| mammary gland development (GO:0030879) | 44585 | 0.946587905 | 0.999994883 |
| negative regulation of neuron differentiation | 44585 | 0.946587905 | 0.999994883 |
| negative regulation of protein tyrosine kinas | 44585 | 0.946587905 | 0.999994883 |
| negative regulation of stress fiber assembly ( | 44585 | 0.946587905 | 0.999994883 |
| neuromuscular junction development (GO:0( | 44585 | 0.946587905 | 0.999994883 |
| phospholipid catabolic process (GO:0009395 | 44585 | 0.946587905 | 0.999994883 |
| positive regulation of lamellipodium assembl | 44585 | 0.946587905 | 0.999994883 |
| positive regulation of p38MAPK cascade (GO | 44585 | 0.946587905 | 0.999994883 |
| positive regulation of phospholipase activity | 44585 | 0.946587905 | 0.999994883 |
| positive regulation of protein targeting to me | 44585 | 0.946587905 | 0.999994883 |
| positive regulation of transcription from RNA | 44585 | 0.946587905 | 0.999994883 |
| potassium ion homeostasis (GO:0055075) | 44585 | 0.946587905 | 0.999994883 |
| protein exit from endoplasmic reticulum (GO | 44585 | 0.946587905 | 0.999994883 |
| purine nucleotide metabolic process (GO:00( | 44585 | 0.946587905 | 0.999994883 |
| regulation of cell size (GO:0008361) | 44585 | 0.946587905 | 0.999994883 |
| regulation of cytokine production involved in | 44585 | 0.946587905 | 0.999994883 |
| regulation of keratinocyte proliferation (GO:( | 44585 | 0.946587905 | 0.999994883 |
| wound healing, spreading of cells (GO:00443 | 44585 | 0.946587905 | 0.999994883 |
| cartilage development (GO:0051216) | 19054 | 0.947363657 | 0.999994883 |
| myeloid cell differentiation (GO:0030099) | 19054 | 0.947363657 | 0.999994883 |
| negative regulation of BMP signaling pathwa | 19054 | 0.947363657 | 0.999994883 |
| regulation of exocytosis (GO:0017157) | 19054 | 0.947363657 | 0.999994883 |
| positive regulation of neuron projection deve | 32295 | 0.947763444 | 0.999994883 |
| odontogenesis (GO:0042476) | 14277 | 0.948136739 | 0.999994883 |
| regulation of T cell differentiation (GO:00455 | 14277 | 0.948136739 | 0.999994883 |
| regulation of cell motility (GO:2000145) | 10/133 | 0.949620051 | 0.999994883 |

| | | | |
|---|---|---|---|
| RNA metabolic process (GO:0016070) | 10/133 | 0.949620051 | 0.999994883 |
| BMP signaling pathway (GO:0030509) | 23833 | 0.949930123 | 0.999994883 |
| inorganic anion transmembrane transport (G | 23833 | 0.949930123 | 0.999994883 |
| positive regulation of type I interferon produ | 28246 | 0.950067624 | 0.999994883 |
| heart morphogenesis (GO:0003007) | 19419 | 0.951862267 | 0.999994883 |
| positive regulation of chemokine production | 19419 | 0.951862267 | 0.999994883 |
| cellular response to hexose stimulus (GO:007 | 44586 | 0.952729422 | 0.999994883 |
| detection of chemical stimulus involved in se | 44586 | 0.952729422 | 0.999994883 |
| negative regulation of signaling (GO:0023057 | 44586 | 0.952729422 | 0.999994883 |
| positive regulation of DNA-templated transcr | 44586 | 0.952729422 | 0.999994883 |
| positive regulation of T cell migration (GO:20 | 44586 | 0.952729422 | 0.999994883 |
| regulation of AMPA receptor activity (GO:20( | 44586 | 0.952729422 | 0.999994883 |
| regulation of cardiac muscle cell action poter | 44586 | 0.952729422 | 0.999994883 |
| regulation of dopamine secretion (GO:00140 | 44586 | 0.952729422 | 0.999994883 |
| regulation of interferon-alpha production (G( | 44586 | 0.952729422 | 0.999994883 |
| regulation of myotube differentiation (GO:00 | 44586 | 0.952729422 | 0.999994883 |
| regulation of neutrophil chemotaxis (GO:009 | 44586 | 0.952729422 | 0.999994883 |
| response to dsRNA (GO:0043331) | 44586 | 0.952729422 | 0.999994883 |
| ribosomal large subunit assembly (GO:00000 | 44586 | 0.952729422 | 0.999994883 |
| RNA splicing, via transesterification reactions | 44586 | 0.952729422 | 0.999994883 |
| sodium ion homeostasis (GO:0055078) | 44586 | 0.952729422 | 0.999994883 |
| autophagy of mitochondrion (GO:0000422) | 14642 | 0.953119447 | 0.999994883 |
| negative regulation of ERBB signaling pathwa | 14642 | 0.953119447 | 0.999994883 |
| negative regulation of protein kinase B signal | 14642 | 0.953119447 | 0.999994883 |
| ribosomal small subunit biogenesis (GO:0042 | 14642 | 0.953119447 | 0.999994883 |
| stem cell differentiation (GO:0048863) | 14642 | 0.953119447 | 0.999994883 |
| protein modification by small protein conjuga | 37/409 | 0.95336988 | 0.999994883 |
| regulation of cell differentiation (GO:004559! | 12/156 | 0.953724811 | 0.999994883 |
| embryonic organ development (GO:0048568 | 24198 | 0.953864707 | 0.999994883 |
| negative regulation of cellular macromolecul | 51/547 | 0.956275053 | 0.999994883 |
| cell junction assembly (GO:0034329) | 7/102 | 0.956352056 | 0.999994883 |
| regulation of protein catabolic process (GO:0 | 7/102 | 0.956352056 | 0.999994883 |
| regulation of viral genome replication (GO:0( | 24563 | 0.957512505 | 0.999994883 |
| regulation of protein dephosphorylation (GO | 15008 | 0.957642312 | 0.999994883 |
| regulation of viral transcription (GO:0046782 | 15008 | 0.957642312 | 0.999994883 |
| visual system development (GO:0150063) | 15008 | 0.957642312 | 0.999994883 |
| cellular calcium ion homeostasis (GO:000687 | 10/136 | 0.957910554 | 0.999994883 |
| endothelial cell development (GO:0001885) | 44587 | 0.958165006 | 0.999994883 |
| metanephros development (GO:0001656) | 44587 | 0.958165006 | 0.999994883 |
| mRNA polyadenylation (GO:0006378) | 44587 | 0.958165006 | 0.999994883 |
| negative regulation of cell development (GO: | 44587 | 0.958165006 | 0.999994883 |
| negative regulation of macroautophagy (GO: | 44587 | 0.958165006 | 0.999994883 |
| negative regulation of proteolysis involved in | 44587 | 0.958165006 | 0.999994883 |
| negative regulation of transposition (GO:001 | 44587 | 0.958165006 | 0.999994883 |
| positive regulation of glucose import (GO:004 | 44587 | 0.958165006 | 0.999994883 |
| positive regulation of T cell mediated cytoto> | 44587 | 0.958165006 | 0.999994883 |
| positive regulation of viral transcription (GO: | 44587 | 0.958165006 | 0.999994883 |

| | | | |
|---|---|---|---|
| regulation of chondrocyte differentiation (GC | 44587 | 0.958165006 | 0.999994883 |
| regulation of potassium ion transmembrane | 44587 | 0.958165006 | 0.999994883 |
| RNA polyadenylation (GO:0043631) | 44587 | 0.958165006 | 0.999994883 |
| transcription, DNA-templated (GO:0006351) | 18/221 | 0.958174272 | 0.999994883 |
| response to lipid (GO:0033993) | 8/114 | 0.95834163 | 0.999994883 |
| negative regulation of transcription, DNA-ten | 93/948 | 0.95859877 | 0.999994883 |
| regulation of NIK/NF-kappaB signaling (GO:19 | 29342 | 0.960315239 | 0.999994883 |
| regulation of organelle organization (GO:003 | 29342 | 0.960315239 | 0.999994883 |
| circulatory system development (GO:007235 | 9/126 | 0.960508097 | 0.999994883 |
| regulation of peptidyl-tyrosine phosphorylati | 33756 | 0.960821083 | 0.999994883 |
| canonical Wnt signaling pathway (GO:006007 | 24929 | 0.960891995 | 0.999994883 |
| negative regulation of transforming growth f | 24929 | 0.960891995 | 0.999994883 |
| response to lipopolysaccharide (GO:0032496 | 12/159 | 0.960947233 | 0.999994883 |
| cellular response to lectin (GO:1990858) | 8/115 | 0.960996825 | 0.999994883 |
| stimulatory C-type lectin receptor signaling p | 8/115 | 0.960996825 | 0.999994883 |
| potassium ion import across plasma membra | 15373 | 0.961745179 | 0.999994883 |
| regulation of chemokine production (GO:003 | 15373 | 0.961745179 | 0.999994883 |
| regulation of extrinsic apoptotic signaling pat | 15373 | 0.961745179 | 0.999994883 |
| acute inflammatory response (GO:0002526) | 44588 | 0.962975769 | 0.999994883 |
| cellular response to unfolded protein (GO:00 | 44588 | 0.962975769 | 0.999994883 |
| cellular senescence (GO:0090398) | 44588 | 0.962975769 | 0.999994883 |
| negative regulation of actin filament bundle a | 44588 | 0.962975769 | 0.999994883 |
| positive regulation of calcium ion transmemb | 44588 | 0.962975769 | 0.999994883 |
| regulation of epidermal growth factor-activat | 44588 | 0.962975769 | 0.999994883 |
| regulation of transcription elongation from R | 44588 | 0.962975769 | 0.999994883 |
| response to glucocorticoid (GO:0051384) | 44588 | 0.962975769 | 0.999994883 |
| stress-activated protein kinase signaling casc | 44588 | 0.962975769 | 0.999994883 |
| regulation of interleukin-8 production (GO:00 | 29707 | 0.963277109 | 0.999994883 |
| blood vessel morphogenesis (GO:0048514) | 20515 | 0.963294003 | 0.999994883 |
| Rho protein signal transduction (GO:0007266 | 20515 | 0.963294003 | 0.999994883 |
| sensory organ development (GO:0007423) | 20515 | 0.963294003 | 0.999994883 |
| cell chemotaxis (GO:0060326) | 25294 | 0.964020737 | 0.999994883 |
| peptidyl-tyrosine phosphorylation (GO:00181 | 25294 | 0.964020737 | 0.999994883 |
| regulation of cellular component organizatioi | 10/139 | 0.964964818 | 0.999994883 |
| positive regulation of protein kinase B signali | 12/161 | 0.96518894 | 0.999994883 |
| plasma membrane bounded cell projection o | 9/128 | 0.965233856 | 0.999994883 |
| natural killer cell activation (GO:0030101) | 15738 | 0.965464812 | 0.999994883 |
| negative regulation of type I interferon produ | 15738 | 0.965464812 | 0.999994883 |
| positive regulation of T cell differentiation (G | 15738 | 0.965464812 | 0.999994883 |
| regulation of cell-cell adhesion (GO:0022407) | 15738 | 0.965464812 | 0.999994883 |
| signal release from synapse (GO:0099643) | 15738 | 0.965464812 | 0.999994883 |
| nucleosome organization (GO:0034728) | 34486 | 0.966166888 | 0.999994883 |
| negative regulation of signal transduction (G( | 22/267 | 0.966559059 | 0.999994883 |
| cellular response to light stimulus (GO:00714 | 25659 | 0.966915379 | 0.999994883 |
| neutrophil chemotaxis (GO:0030593) | 25659 | 0.966915379 | 0.999994883 |
| wound healing (GO:0042060) | 25659 | 0.966915379 | 0.999994883 |
| protein localization to cell periphery (GO:199 | 10/140 | 0.967068962 | 0.999994883 |

| | | | |
|---|---|---|---|
| cellular zinc ion homeostasis (GO:0006882) | 44589 | 0.967233504 | 0.999994883 |
| mucosal immune response (GO:0002385) | 44589 | 0.967233504 | 0.999994883 |
| positive regulation of defense response to vi | 44589 | 0.967233504 | 0.999994883 |
| regulation of extrinsic apoptotic signaling pat | 44589 | 0.967233504 | 0.999994883 |
| regulation of insulin secretion involved in cell | 44589 | 0.967233504 | 0.999994883 |
| regulation of protein transport (GO:0051223) | 44589 | 0.967233504 | 0.999994883 |
| sarcomere organization (GO:0045214) | 44589 | 0.967233504 | 0.999994883 |
| negative regulation of cell population prolifer | 33/379 | 0.967499887 | 0.999994883 |
| negative regulation of cellular process (GO:0( | 52/566 | 0.967646602 | 0.999994883 |
| protein localization to membrane (GO:00726 | 15/195 | 0.968443004 | 0.999994883 |
| positive regulation of cell differentiation (GO | 21/258 | 0.968554753 | 0.999994883 |
| antibacterial humoral response (GO:0019731 | 16103 | 0.968835072 | 0.999994883 |
| cardiac ventricle morphogenesis (GO:000320 | 16103 | 0.968835072 | 0.999994883 |
| lymphocyte chemotaxis (GO:0048247) | 16103 | 0.968835072 | 0.999994883 |
| neurogenesis (GO:0022008) | 16103 | 0.968835072 | 0.999994883 |
| neurotransmitter secretion (GO:0007269) | 16103 | 0.968835072 | 0.999994883 |
| positive regulation of biosynthetic process (G | 16103 | 0.968835072 | 0.999994883 |
| regulation of inflammatory response (GO:00! | 16/206 | 0.969060041 | 0.999994883 |
| positive regulation of protein binding (GO:00 | 21245 | 0.969438097 | 0.999994883 |
| regulation of neurotransmitter receptor activ | 21245 | 0.969438097 | 0.999994883 |
| regulation of primary metabolic process (GO: | 9/130 | 0.969443926 | 0.999994883 |
| cellular response to BMP stimulus (GO:00717 | 26024 | 0.969591681 | 0.999994883 |
| regulation of endopeptidase activity (GO:005 | 26024 | 0.969591681 | 0.999994883 |
| innate immune response activating cell surfa | 8/119 | 0.97015798 | 0.999994883 |
| nuclear export (GO:0051168) | 30803 | 0.970984201 | 0.999994883 |
| establishment of endothelial barrier (GO:006 | 44590 | 0.971001763 | 0.999994883 |
| negative regulation of epithelial to mesenchy | 44590 | 0.971001763 | 0.999994883 |
| regulation of protein targeting to membrane | 44590 | 0.971001763 | 0.999994883 |
| regulation of T cell mediated cytotoxicity (GC | 44590 | 0.971001763 | 0.999994883 |
| response to zinc ion (GO:0010043) | 44590 | 0.971001763 | 0.999994883 |
| negative regulation of transmembrane recep | 7/108 | 0.971287561 | 0.999994883 |
| positive regulation of nervous system develo | 16469 | 0.971887105 | 0.999994883 |
| positive regulation of RNA metabolic process | 16469 | 0.971887105 | 0.999994883 |
| regulation of DNA repair (GO:0006282) | 16469 | 0.971887105 | 0.999994883 |
| positive regulation of neurogenesis (GO:005( | 26390 | 0.972064538 | 0.999994883 |
| macroautophagy (GO:0016236) | 8/120 | 0.972119061 | 0.999994883 |
| negative regulation of autophagy (GO:00105( | 21610 | 0.972132168 | 0.999994883 |
| protein polymerization (GO:0051258) | 21610 | 0.972132168 | 0.999994883 |
| negative regulation of defense response (GO | 31168 | 0.973200076 | 0.999994883 |
| positive regulation of protein catabolic proce | 31168 | 0.973200076 | 0.999994883 |
| transmembrane receptor protein tyrosine kir | 35/404 | 0.973339511 | 0.999994883 |
| MAPK cascade (GO:0000165) | 25/303 | 0.973616668 | 0.999994883 |
| extrinsic apoptotic signaling pathway via dea | 44591 | 0.974336793 | 0.999994883 |
| heterotypic cell-cell adhesion (GO:0034113) | 44591 | 0.974336793 | 0.999994883 |
| mRNA splice site selection (GO:0006376) | 44591 | 0.974336793 | 0.999994883 |
| negative regulation of Notch signaling pathw | 44591 | 0.974336793 | 0.999994883 |
| positive regulation of endopeptidase activity | 44591 | 0.974336793 | 0.999994883 |

| | | | |
|---|---|---|---|
| regulation of catecholamine secretion (GO:0( | 44591 | 0.974336793 | 0.999994883 |
| regulation of striated muscle contraction (GC | 44591 | 0.974336793 | 0.999994883 |
| respiratory tube development (GO:0030323) | 44591 | 0.974336793 | 0.999994883 |
| synaptic vesicle cycle (GO:0099504) | 44591 | 0.974336793 | 0.999994883 |
| TRIF-dependent toll-like receptor signaling pa | 44591 | 0.974336793 | 0.999994883 |
| zinc ion homeostasis (GO:0055069) | 44591 | 0.974336793 | 0.999994883 |
| granulocyte chemotaxis (GO:0071621) | 26755 | 0.974347999 | 0.999994883 |
| negative regulation of cell adhesion (GO:000 | 26755 | 0.974347999 | 0.999994883 |
| regulation of Rho protein signal transduction | 26755 | 0.974347999 | 0.999994883 |
| regulation of synaptic transmission, glutamat | 16834 | 0.974649511 | 0.999994883 |
| transmembrane receptor protein serine/thre | 9/133 | 0.974897276 | 0.999994883 |
| positive regulation of interleukin-8 productio | 22341 | 0.976859164 | 0.999994883 |
| epidermal growth factor receptor signaling p | 17199 | 0.977148524 | 0.999994883 |
| regulation of synapse assembly (GO:0051963 | 17199 | 0.977148524 | 0.999994883 |
| MyD88-independent toll-like receptor signali | 44592 | 0.977288382 | 0.999994883 |
| peptide cross-linking (GO:0018149) | 44592 | 0.977288382 | 0.999994883 |
| phospholipid translocation (GO:0045332) | 44592 | 0.977288382 | 0.999994883 |
| renal water homeostasis (GO:0003091) | 44592 | 0.977288382 | 0.999994883 |
| response to retinoic acid (GO:0032526) | 22706 | 0.978926207 | 0.999994883 |
| retrograde transport, endosome to Golgi (GC | 32264 | 0.978938743 | 0.999994883 |
| regulation of I-kappaB kinase/NF-kappaB sigr | 17/224 | 0.97906799 | 0.999994883 |
| nucleus organization (GO:0006997) | 17564 | 0.979408174 | 0.999994883 |
| regulation of interleukin-10 production (GO:( | 17564 | 0.979408174 | 0.999994883 |
| cell-cell adhesion via plasma-membrane adhe | 12/170 | 0.979610356 | 0.999994883 |
| axon guidance (GO:0007411) | 15/203 | 0.979695521 | 0.999994883 |
| 7-methylguanosine mRNA capping (GO:0006: | 11689 | 0.9799006 | 0.999994883 |
| 7-methylguanosine RNA capping (GO:000945 | 11689 | 0.9799006 | 0.999994883 |
| chondrocyte differentiation (GO:0002062) | 11689 | 0.9799006 | 0.999994883 |
| ERBB2 signaling pathway (GO:0038128) | 11689 | 0.9799006 | 0.999994883 |
| establishment or maintenance of epithelial c | 11689 | 0.9799006 | 0.999994883 |
| positive regulation of immune effector proce | 11689 | 0.9799006 | 0.999994883 |
| regulation of cellular amide metabolic proces | 11689 | 0.9799006 | 0.999994883 |
| regulation of cellular protein metabolic proce | 17930 | 0.981450452 | 0.999994883 |
| neutrophil migration (GO:1990266) | 28216 | 0.981841037 | 0.999994883 |
| positive regulation of histone modification (G | 12055 | 0.98221245 | 0.999994883 |
| regulation of interleukin-17 production (GO:( | 12055 | 0.98221245 | 0.999994883 |
| regulation of ERK1 and ERK2 cascade (GO:00 | 18/238 | 0.982450477 | 0.999994883 |
| chloride transmembrane transport (GO:1902 | 18295 | 0.983295453 | 0.999994883 |
| negative regulation of ERK1 and ERK2 cascad | 18295 | 0.983295453 | 0.999994883 |
| ribosome assembly (GO:0042255) | 18295 | 0.983295453 | 0.999994883 |
| antigen receptor-mediated signaling pathway | 13/185 | 0.983892587 | 0.999994883 |
| cytosolic transport (GO:0016482) | 7/116 | 0.983955815 | 0.999994883 |
| axonogenesis (GO:0007409) | 18/240 | 0.984238888 | 0.999994883 |
| female gamete generation (GO:0007292) | 12420 | 0.984258458 | 0.999994883 |
| negative regulation of extrinsic apoptotic sigr | 12420 | 0.984258458 | 0.999994883 |
| regulation of keratinocyte differentiation (GC | 12420 | 0.984258458 | 0.999994883 |
| striated muscle tissue development (GO:001 | 12420 | 0.984258458 | 0.999994883 |

| | | | |
|---|---|---|---|
| positive regulation of cytokine production (G | 27/335 | 0.984341319 | 0.999994883 |
| regulation of cellular macromolecule biosynt | 40/468 | 0.984627408 | 0.999994883 |
| regulation of ion transport (GO:0043269) | 28946 | 0.984759493 | 0.999994883 |
| regulation of lipid metabolic process (GO:001 | 33725 | 0.984815109 | 0.999994883 |
| Wnt signaling pathway (GO:0016055) | 33725 | 0.984815109 | 0.999994883 |
| ERBB signaling pathway (GO:0038127) | 24167 | 0.985562805 | 0.999994883 |
| positive regulation of MAPK cascade (GO:004 | 21/274 | 0.985761293 | 0.999994883 |
| regulation of epithelial cell proliferation (GO: | 34090 | 0.986021435 | 0.999994883 |
| cellular response to virus (GO:0098586) | 12785 | 0.98606918 | 0.999994883 |
| positive regulation of cell cycle G1/S phase tr | 12785 | 0.98606918 | 0.999994883 |
| respiratory system development (GO:006054 | 12785 | 0.98606918 | 0.999994883 |
| response to exogenous dsRNA (GO:0043330) | 12785 | 0.98606918 | 0.999994883 |
| endocytic recycling (GO:0032456) | 19025 | 0.986465419 | 0.999994883 |
| response to peptide hormone (GO:0043434) | 19025 | 0.986465419 | 0.999994883 |
| calcium-dependent cell-cell adhesion via plas | 13150 | 0.987671664 | 0.999994883 |
| membrane assembly (GO:0071709) | 13150 | 0.987671664 | 0.999994883 |
| mitochondrion disassembly (GO:0061726) | 13150 | 0.987671664 | 0.999994883 |
| positive regulation of cytokine-mediated sign | 13150 | 0.987671664 | 0.999994883 |
| positive regulation of innate immune respons | 13150 | 0.987671664 | 0.999994883 |
| positive regulation of interferon-beta produc | 13150 | 0.987671664 | 0.999994883 |
| positive regulation of myeloid leukocyte diffe | 13150 | 0.987671664 | 0.999994883 |
| regulation of cardiac muscle contraction (GO | 13150 | 0.987671664 | 0.999994883 |
| interferon-gamma-mediated signaling pathw | 24898 | 0.988076961 | 0.999994883 |
| regulation of immune response (GO:0050776 | 12/179 | 0.98837376 | 0.999994883 |
| DNA-templated transcription, initiation (GO:( | 11/168 | 0.988699026 | 0.999994883 |
| generation of neurons (GO:0048699) | 14/202 | 0.988700136 | 0.999994883 |
| regulation of cell adhesion (GO:0030155) | 8/133 | 0.988874125 | 0.999994883 |
| regulation of alternative mRNA splicing, via s | 19756 | 0.98904631 | 0.999994883 |
| glutamate receptor signaling pathway (GO:00 | 13516 | 0.989089848 | 0.999994883 |
| regulation of glucose import (GO:0046324) | 13516 | 0.989089848 | 0.999994883 |
| regulation of vesicle-mediated transport (GO | 35186 | 0.989119927 | 0.999994883 |
| DNA-templated transcription, elongation (GC | 25263 | 0.989170407 | 0.999994883 |
| RNA 3'-end processing (GO:0031123) | 25263 | 0.989170407 | 0.999994883 |
| translation (GO:0006412) | 15/214 | 0.989266409 | 0.999994883 |
| epidermis development (GO:0008544) | 30407 | 0.989313954 | 0.999994883 |
| skeletal system development (GO:0001501) | 10/158 | 0.989850588 | 0.999994883 |
| T cell receptor signaling pathway (GO:005085 | 10/158 | 0.989850588 | 0.999994883 |
| lymphocyte proliferation (GO:0046651) | 13881 | 0.990344919 | 0.999994883 |
| mRNA destabilization (GO:0061157) | 13881 | 0.990344919 | 0.999994883 |
| cellular response to tumor necrosis factor (G | 13/194 | 0.990770104 | 0.999994883 |
| RNA splicing (GO:0008380) | 35916 | 0.990810574 | 0.999994883 |
| B cell activation (GO:0042113) | 31138 | 0.991071414 | 0.999994883 |
| gland development (GO:0048732) | 25993 | 0.991074592 | 0.999994883 |
| protein-containing complex disassembly (GO | 20486 | 0.991144484 | 0.999994883 |
| regulation of viral entry into host cell (GO:00 | 14246 | 0.991455631 | 0.999994883 |
| regulation of protein kinase B signaling (GO:0 | 14/207 | 0.991687302 | 0.999994883 |
| positive regulation of proteolysis (GO:00458 | 20852 | 0.992040669 | 0.999994883 |

| | | | |
|---|---|---|---|
| bone development (GO:0060348) | 14611 | 0.992438581 | 0.999994883 |
| synapse organization (GO:0050808) | 7/126 | 0.992504685 | 0.999994883 |
| chromatin assembly (GO:0031497) | 26724 | 0.992653356 | 0.999994883 |
| neurotransmitter transport (GO:0006836) | 26724 | 0.992653356 | 0.999994883 |
| cellular response to transforming growth fact | 6/114 | 0.992877136 | 0.999994883 |
| keratinocyte differentiation (GO:0030216) | 14977 | 0.99330846 | 0.999994883 |
| transcription initiation from RNA polymerase | 8/140 | 0.993380157 | 0.999994883 |
| protein deubiquitination (GO:0016579) | 19/267 | 0.993559163 | 0.999994883 |
| positive regulation of tyrosine phosphorylatio | 21582 | 0.993574727 | 0.999994883 |
| regulation of cation channel activity (GO:200 | 32599 | 0.993791909 | 0.999994883 |
| transforming growth factor beta receptor sig | 32599 | 0.993791909 | 0.999994883 |
| modification-dependent protein catabolic pro | 13/201 | 0.994109749 | 0.999994883 |
| negative regulation of translation (GO:00171 | 32964 | 0.994335601 | 0.999994883 |
| positive regulation of cellular process (GO:00 | 53/625 | 0.994557638 | 0.999994883 |
| cellular response to interleukin-1 (GO:00713 | 9/155 | 0.994614247 | 0.999994883 |
| RNA processing (GO:0006396) | 11/179 | 0.994621092 | 0.999994883 |
| cellular response to retinoic acid (GO:007130 | 15707 | 0.994759513 | 0.999994883 |
| development of primary male sexual characte | 15707 | 0.994759513 | 0.999994883 |
| adenylate cyclase-activating G protein-couple | 6/118 | 0.9948733 | 0.999994883 |
| branching morphogenesis of an epithelial tub | 16072 | 0.995362381 | 0.999994883 |
| detection of chemical stimulus involved in se | 16072 | 0.995362381 | 0.999994883 |
| negative regulation of epidermal growth fact | 16072 | 0.995362381 | 0.999994883 |
| anterior/posterior pattern specification (GO:( | 23043 | 0.995823905 | 0.999994883 |
| negative regulation of endopeptidase activity | 23043 | 0.995823905 | 0.999994883 |
| neuropeptide signaling pathway (GO:000721 | 23043 | 0.995823905 | 0.999994883 |
| positive regulation of Notch signaling pathwa | 16438 | 0.995895883 | 0.999994883 |
| regulation of receptor signaling pathway via J | 16438 | 0.995895883 | 0.999994883 |
| mRNA 3'-end processing (GO:0031124) | 28915 | 0.995931706 | 0.999994883 |
| positive regulation of I-kappaB kinase/NF-kap | 10/171 | 0.995935294 | 0.999994883 |
| antimicrobial humoral immune response med | 23408 | 0.996252271 | 0.999994883 |
| regulation of innate immune response (GO:0 | 23408 | 0.996252271 | 0.999994883 |
| ribonucleoprotein complex assembly (GO:002 | 7/136 | 0.996610577 | 0.999994883 |
| regulation of nucleic acid-templated transcri | 33/430 | 0.996628829 | 0.999994883 |
| G protein-coupled receptor signaling pathwa | 23774 | 0.99663734 | 0.999994883 |
| neuron differentiation (GO:0030182) | 10/174 | 0.996731365 | 0.999994883 |
| peptide biosynthetic process (GO:0043043) | 9/162 | 0.996802492 | 0.999994883 |
| response to cytokine (GO:0034097) | 8/150 | 0.99692811 | 0.999994883 |
| negative regulation of inflammatory respons | 13/212 | 0.997166851 | 0.999994883 |
| regulation of gene expression (GO:0010468) | 97/1079 | 0.99719636 | 0.999994883 |
| regulation of Notch signaling pathway (GO:00 | 30376 | 0.99727106 | 0.999994883 |
| transcription elongation from RNA polymeras | 24504 | 0.997294327 | 0.999994883 |
| ncRNA processing (GO:0034470) | 12/201 | 0.997333807 | 0.999994883 |
| regulation of interferon-beta production (GO | 17899 | 0.997482666 | 0.999994883 |
| enzyme linked receptor protein signaling path | 7/140 | 0.997552346 | 0.999994883 |
| negative regulation of immune response (GO | 10/178 | 0.997564656 | 0.999994883 |
| regulation of tyrosine phosphorylation of STA | 24869 | 0.997573623 | 0.999994883 |
| positive regulation of nucleic acid-templated | 40/511 | 0.997629106 | 0.999994883 |

| | | | |
|---|---|---|---|
| chromatin organization (GO:0006325) | 7/142 | 0.997923311 | 0.999994883 |
| response to interleukin-1 (GO:0070555) | 31472 | 0.997982303 | 0.999994883 |
| protein modification by small protein remova | 18/276 | 0.998075677 | 0.999994883 |
| gene expression (GO:0010467) | 25/356 | 0.998233756 | 0.999994883 |
| chromatin remodeling (GO:0006338) | 4/103 | 0.998322905 | 0.999994883 |
| negative regulation of response to external s | 5/118 | 0.998415162 | 0.999994883 |
| cardiac muscle tissue development (GO:0048 | 19360 | 0.998455675 | 0.999994883 |
| epidermal cell differentiation (GO:0009913) | 19360 | 0.998455675 | 0.999994883 |
| defense response to Gram-negative bacteriu | 26696 | 0.998596037 | 0.999994883 |
| response to molecule of bacterial origin (GO: | 26696 | 0.998596037 | 0.999994883 |
| regulation of mRNA splicing, via spliceosome | 32933 | 0.998655005 | 0.999994883 |
| fibroblast growth factor receptor signaling pa | 27426 | 0.998873077 | 0.999994883 |
| negative regulation of inflammatory respons | 6/136 | 0.998896502 | 0.999994883 |
| cellular response to fibroblast growth factor : | 33664 | 0.998903128 | 0.999994883 |
| regulation of inflammatory response to antig | 6/137 | 0.998989115 | 0.999994883 |
| ribosomal large subunit biogenesis (GO:0042 | 20821 | 0.999052248 | 0.999994883 |
| inflammatory response (GO:0006954) | 13/230 | 0.999195023 | 0.999994883 |
| anterograde trans-synaptic signaling (GO:009 | 14/244 | 0.999292744 | 0.999994883 |
| skin development (GO:0043588) | 29252 | 0.999350742 | 0.999994883 |
| B cell differentiation (GO:0030183) | 22282 | 0.999417973 | 0.999994883 |
| regulation of neurogenesis (GO:0050767) | 22647 | 0.999484683 | 0.999994883 |
| transcription by RNA polymerase II (GO:0006 | 20/320 | 0.99948746 | 0.999994883 |
| positive regulation of intracellular signal tran | 40/546 | 0.999573969 | 0.999994883 |
| positive regulation of transcription, DNA-tem | 102/1183 | 0.999581772 | 0.999994883 |
| positive regulation of cell population prolifer | 33/474 | 0.999661461 | 0.999994883 |
| ribosome biogenesis (GO:0042254) | 9/192 | 0.999699699 | 0.999994883 |
| cellular response to interferon-gamma (GO:0 | 4/121 | 0.999706942 | 0.999994883 |
| defense response to symbiont (GO:0140546) | 4/124 | 0.99978167 | 0.999994883 |
| defense response to Gram-positive bacteriun | 25569 | 0.999804582 | 0.999994883 |
| response to tumor necrosis factor (GO:00346 | 3/110 | 0.999828654 | 0.999994883 |
| cell junction organization (GO:0034330) | 26299 | 0.999846357 | 0.999994883 |
| cotranslational protein targeting to membrar | 34366 | 0.99986186 | 0.999994883 |
| nuclear-transcribed mRNA catabolic process, | 3/113 | 0.999874282 | 0.999994883 |
| defense response to virus (GO:0051607) | 4/133 | 0.999909474 | 0.999994883 |
| positive regulation of transcription by RNA p | 71/908 | 0.999913318 | 0.999994883 |
| RNA splicing, via transesterification reactions | 12/251 | 0.999934141 | 0.999994883 |
| response to interferon-gamma (GO:0034341 | 29221 | 0.99994034 | 0.999994883 |
| phospholipase C-activating G protein-couplec | 29587 | 0.999946846 | 0.999994883 |
| chemical synaptic transmission (GO:0007268 | 16/306 | 0.999948915 | 0.999994883 |
| nuclear-transcribed mRNA catabolic process | 6/171 | 0.999951317 | 0.999994883 |
| rRNA processing (GO:0006364) | 6/173 | 0.999959017 | 0.999994883 |
| lymphocyte differentiation (GO:0030098) | 30682 | 0.999962191 | 0.999994883 |
| mRNA splicing, via spliceosome (GO:0000398 | 13/274 | 0.999967501 | 0.999994883 |
| defense response to bacterium (GO:0042742 | 6/176 | 0.999968172 | 0.999994883 |
| regulation of cell population proliferation (G( | 55/764 | 0.999970926 | 0.999994883 |
| rRNA metabolic process (GO:0016072) | 5/162 | 0.999972237 | 0.999994883 |
| adenylate cyclase-modulating G protein-coup | 5/165 | 0.99997834 | 0.999994883 |

| | | | |
|---|---|---|---|
| SRP-dependent cotranslational protein targe | 32874 | 0.999980142 | 0.999994883 |
| mRNA processing (GO:0006397) | 14/300 | 0.999985225 | 0.999994883 |
| regulation of transcription, DNA-templated (( | 197/2244 | 0.999987354 | 0.999994883 |
| cytokine-mediated signaling pathway (GO:00 | 40/621 | 0.999987896 | 0.999994883 |
| sensory perception of chemical stimulus (GO | 36161 | 0.99999112 | 0.999994883 |
| regulation of transcription by RNA polymeras | 188/2206 | 0.999991194 | 0.999994883 |
| protein targeting to ER (GO:0045047) | 1/103 | 0.999993207 | 0.999994883 |
| cellular response to cytokine stimulus (GO:0( | 22/482 | 0.999994384 | 0.999994883 |
| innate immune response (GO:0045087) | 11/302 | 0.999994883 | 0.999994883 |

**ıalysis of significant highly-correlated RNA-seq-based modules (blue; Significan**

| Old P-value | Old Adjusted P-value | Odds Ratio | Combined Score | Genes | KEGG |
|---|---|---|---|---|---|
| 0 | 0 | 8.766291231 | 334.2498805 | ABCD4;AC/ | |
| 0 | 0 | 3.030701457 | 102.7281621 | DYNC2I2;D | |
| 0 | 0 | 3.102052021 | 105.0470961 | DCLRE1B;N | |
| 0 | 0 | 9.829000843 | 315.4306841 | ABCD4;GCI | |
| 0 | 0 | 7.508259912 | 194.7044649 | DCLRE1B;D | |
| 0 | 0 | 2.762084955 | 68.97822484 | DCLRE1B;F | |
| 0 | 0 | 2.930632598 | 69.56983626 | DYNC2I2;D | |
| 0 | 0 | 5.031856796 | 112.3160904 | FEN1;BLM; | |
| 0 | 0 | 4.402576937 | 97.93436231 | BLM;FEN1; | |
| 0 | 0 | 6.130258997 | 134.8251673 | ABCD4;AC/ | |
| 0 | 0 | 3.139760684 | 63.54303684 | DCLRE1B;D | |
| 0 | 0 | 7.76592007 | 155.3099935 | DYNC2I2;D | |
| 0 | 0 | 2.490731728 | 49.56501048 | DYNC2I2;D | |
| 0 | 0 | 2.252634334 | 42.93274461 | FEN1;MCM | |
| 0 | 0 | 6.040421793 | 108.0650671 | RFC5;MBD | |
| 0 | 0 | 3.408515694 | 58.40615456 | CPM;SLC46 | |
| 0 | 0 | 7.325657895 | 121.9945554 | RFC5;MBD | |
| 0 | 0 | 3.590919887 | 56.79325056 | CETN2;CEP | |
| 0 | 0 | 61.86806466 | 942.5012295 | MCEE;MM | |
| 0 | 0 | 3.131589505 | 43.60912426 | BLM;FEN1; | |
| 0 | 0 | 3.42374374 | 45.30117331 | CETN2;CEP | |
| 0 | 0 | 106218 | 1379839.372 | TCTN2;TCT | |
| 0 | 0 | 5.391990546 | 69.66830602 | RFC5;RFC3 | |
| 0 | 0 | 5.391990546 | 69.66830602 | GEN1;ZRAI | |
| 0 | 0 | 9.460192476 | 120.9822326 | ACAD8;MC | |
| 0 | 0 | 9.460192476 | 120.9822326 | ACAD8;MC | |
| 0 | 0 | 1.844512163 | 23.54100156 | DYNC2I2;D | |
| 0 | 0 | 2.673324315 | 31.89960031 | PSMD10;P | |
| 0 | 0 | 2.704443805 | 31.94972107 | ABCD3;AAS | |
| 0 | 0 | 11.6020542 | 134.852937 | ACOT8;AM | |
| 0 | 0 | 2.198995402 | 25.36660553 | CCNJ;CETN | |
| 0 | 0 | 2.82479266 | 31.58693124 | CETN2;OG( | |
| 0 | 0 | 6.19273523 | 68.04639586 | DCTD;RRM | |
| 0 | 0 | 2.985847779 | 32.53659264 | GCDH;ECH | |
| 0 | 0 | 88515 | 958135.3914 | DCXR;AKR1 | |
| 0 | 0 | 88515 | 958135.3914 | DCXR;AKR1 | |
| 0 | 0 | 88515 | 958135.3914 | DCXR;AKR1 | |
| 0 | 0 | 12.37081695 | 132.354171 | MBD4;NEII | |
| 0 | 0 | 18.0349345 | 192.7178479 | GCDH;ACA | |
| 0 | 0 | 18.0349345 | 192.7178479 | ABCA2;CN7 | |
| 0 | 0 | 18.0349345 | 192.7178479 | BLM;RTEL1 | |
| 0 | 0 | 4.713858577 | 48.9154733 | RFC5;RFC3 | |
| 0 | 0 | 3.874780702 | 40.18170499 | BLM;RTEL1 | |
| 0 | 0 | 4.149320771 | 41.10721676 | GEN1;ZRAI | |

| | | | | |
|---|---|---|---|---|
| 0 | 0 | 23.17896115 | 228.6584902 | MBD4;NEI[illegible] |
| 0 | 0 | 23.17896115 | 228.6584902 | MCEE;MM[illegible] |
| 0 | 0 | 2.297803992 | 22.22597777 | PSMD10;C[illegible] |
| 0 | 0 | 2.331227915 | 22.53302032 | AASDH;GL[illegible] |
| 0 | 0 | 4.574371119 | 43.46457186 | INVS;TTC2[illegible] |
| 0 | 0 | 8.835299257 | 82.18892731 | CIDEA;PLI[illegible] |
| 0 | 0 | 2.960600735 | 27.53146857 | ABCD4;MC[illegible] |
| 0 | 0 | 6.959659091 | 63.60064248 | UGDH;GN[illegible] |
| 0 | 0 | 4.014600672 | 36.40587783 | GALT;PMM[illegible] |
| 0 | 0 | 3.37705803 | 30.52454462 | DCLRE1B;[illegible] |
| 0 | 0 | 3.067795215 | 27.36668567 | MCFD2;AL[illegible] |
| 0 | 0 | 2.329466255 | 20.7793054 | CETN2;CE[illegible] |
| 0 | 0 | 15.45176779 | 137.0445756 | MCCC2;BT[illegible] |
| 0 | 0 | 15.45176779 | 137.0445756 | RFC5;RFC3[illegible] |
| 0 | 0 | 15.45176779 | 137.0445756 | RFC5;RFC3[illegible] |
| 0 | 0 | 2.306271209 | 20.23670839 | CETN2;CE[illegible] |
| 0 | 0 | 2.034841072 | 17.64110258 | PSMD10;C[illegible] |
| 0 | 0 | 3.737308744 | 31.73189383 | GSTM1;GS[illegible] |
| 0 | 0 | 4.727690289 | 39.9285104 | MSH6;RN[illegible] |
| 0 | 0 | 2.818381443 | 23.66132371 | RFC5;FEN1[illegible] |
| 0 | 0 | 9.015938865 | 75.19271222 | DYNC2I2;D[illegible] |
| 0 | 0 | 6.871122761 | 56.24276599 | SLC25A1;G[illegible] |
| 0 | 0 | 11.5881711 | 93.34879843 | IMPACT;SE[illegible] |
| 0 | 0 | 2.066379089 | 16.1268278 | AHCTF1;IN[illegible] |
| 0 | 0 | 6.183660987 | 47.63533242 | DCLRE1B;[illegible] |
| 0 | 0 | 2.049731449 | 15.75156483 | ACHE;SLC2[illegible] |
| 0 | 0 | 2.862023265 | 21.42428965 | ACOT8;OS[illegible] |
| 0 | 0 | 4.051618548 | 30.20755397 | ABCD4;SLC[illegible] |
| 0 | 0 | 2.240510788 | 16.67889493 | HACD1;PP[illegible] |
| 0 | 0 | 2.12941695 | 15.40363634 | POGLUT1;[illegible] |
| 0 | 0 | 30.88006978 | 220.6577689 | SLC27A1;F[illegible] |
| 0 | 0 | 12.87085515 | 91.37562389 | SLC46A1;A[illegible] |
| 0 | 0 | 12.87085515 | 91.37562389 | ABHD10;F[illegible] |
| 0 | 0 | 12.87085515 | 91.37562389 | GSTZ1;PD[illegible] |
| 0 | 0 | 12.87085515 | 91.37562389 | GSTZ1;PD[illegible] |
| 0 | 0 | 12.87085515 | 91.37562389 | BLM;WRN;[illegible] |
| 0 | 0 | 4.638461538 | 32.57830159 | RFC5;RFC3[illegible] |
| 0 | 0 | 4.069684487 | 28.13120301 | DDB1;PAR[illegible] |
| 0 | 0 | 3.438074398 | 23.68130847 | GEN1;RAD[illegible] |
| 0 | 0 | 3.438074398 | 23.68130847 | CENPX;CE[illegible] |
| 0 | 0 | 5.152468327 | 35.33122501 | SLC27A1;F[illegible] |
| 0 | 0 | 7.724574422 | 52.36032864 | ACOT8;GN[illegible] |
| 0 | 0 | 7.724574422 | 52.36032864 | TMLHE;AC[illegible] |
| 0 | 0 | 3.865981635 | 25.57099115 | BBS1;TTC8[illegible] |
| 0 | 0 | 2.488920579 | 16.23470003 | NCAPG2;S[illegible] |
| 0 | 0 | 4.755855765 | 30.83144038 | RFC5;RFC3[illegible] |
| 0 | 0 | 4.755855765 | 30.83144038 | ABCD4;AR[illegible] |

| | | | | |
|---|---|---|---|---|
| 0 | 0 | 4.755855765 | 30.83144038 | DDB1;PARI… |
| 0 | 0 | 4.755855765 | 30.83144038 | DDB1;PARI… |
| 0 | 0 | 9.652595986 | 61.66004937 | GSTZ1;QDI… |
| 0 | 0 | 9.652595986 | 61.66004937 | SLC27A1;G… |
| 0 | 0 | 4.092297408 | 26.04842477 | MBD4;NEII… |
| 0 | 0 | 3.402589676 | 21.6085644 | GGA2;BBS… |
| 0 | 0 | 5.408340611 | 34.22668937 | DDB1;PCGI… |
| 0 | 0 | 6.620689655 | 41.45357753 | UGDH;SLC… |
| 0 | 0 | 6.620689655 | 41.45357753 | DDB1;POL… |
| 0 | 0 | 3.093741794 | 19.10382545 | ZRANB3;M… |
| 0 | 0 | 15.43916267 | 94.8400082 | PIGS;PIGU;… |
| 0 | 0 | 15.43916267 | 94.8400082 | GNPDA1;N… |
| 0 | 0 | 15.43916267 | 94.8400082 | PPARG;TRE… |
| 0 | 0 | 15.43916267 | 94.8400082 | CCDC88A;… |
| 0 | 0 | 15.43916267 | 94.8400082 | RTEL1;SLX… |
| 0 | 0 | 4.415902141 | 27.09564167 | GSTZ1;GST… |
| 0 | 0 | 4.415902141 | 27.09564167 | GSTZ1;GST… |
| 0 | 0 | 4.415902141 | 27.09564167 | DDB1;PARI… |
| 0 | 0 | 4.415902141 | 27.09564167 | DPY19L1;B… |
| 0 | 0 | 3.27153577 | 19.97526797 | MAP10;SP… |
| 0 | 0 | 3.514131256 | 21.31196412 | BRIP1;CHE… |
| 0 | 0 | 2.873204904 | 17.40331214 | BBS2;BBS1… |
| 0 | 0 | 2.298761249 | 13.91320524 | CPM;GSTM… |
| 0 | 0 | 4.916395395 | 29.20110325 | GINS1;POL… |
| 0 | 0 | 4.916395395 | 29.20110325 | B4GAT1;P… |
| 0 | 0 | 1.975574378 | 11.73240909 | AHCTF1;IN… |
| 0 | 0 | 3.15018956 | 18.49749486 | PRIM2;POI… |
| 0 | 0 | 3.15018956 | 18.49749486 | ZNHIT1;CE… |
| 0 | 0 | 3.36115283 | 19.53041137 | ABCD4;HA… |
| 0 | 0 | 5.79277608 | 33.61650193 | HADHB;HA… |
| 0 | 0 | 5.79277608 | 33.61650193 | ADK;GMPS… |
| 0 | 0 | 7.721640489 | 44.73823294 | IMPACT;PK… |
| 0 | 0 | 7.721640489 | 44.73823294 | TREM2;AD… |
| 0 | 0 | 7.721640489 | 44.73823294 | GINS1;POL… |
| 0 | 0 | 7.721640489 | 44.73823294 | GNPDA1;N… |
| 0 | 0 | 7.721640489 | 44.73823294 | ABCA2;NE… |
| 0 | 0 | 7.721640489 | 44.73823294 | GCSH;SHM… |
| 0 | 0 | 2.900054705 | 16.66474255 | MAP10;SP… |
| 0 | 0 | 2.717588867 | 15.39737332 | SLC27A1;P… |
| 0 | 0 | 4.506441048 | 25.16072905 | ABCD4;SLC… |
| 0 | 0 | 4.506441048 | 25.16072905 | PLK4;STIL;… |
| 0 | 0 | 3.220922606 | 17.93784181 | LATS1;SGC… |
| 0 | 0 | 1.936949824 | 10.74439538 | VKORC1L1… |
| 0 | 0 | 3.477862762 | 19.19256413 | SLC46A1;S… |
| 0 | 0 | 3.477862762 | 19.19256413 | PRDX2;PRI… |
| 0 | 0 | 3.863477501 | 21.29767177 | AGBL5;VK… |
| 0 | 0 | 2.028351648 | 11.14377041 | RALBP1;DN… |

| | | | | |
|---|---|---|---|---|
| 0 | 0 | 2.518513991 | 13.71195407 | SLC46A1;A |
| 0 | 0 | 2.417698291 | 13.12582177 | LRSAM1;N |
| 0 | 0 | 5.1488433 | 27.77104776 | GEN1;RAD |
| 0 | 0 | 5.1488433 | 27.77104776 | IMPACT;PK |
| 0 | 0 | 5.1488433 | 27.77104776 | COQ3;AIFM |
| 0 | 0 | 10.29219363 | 55.48409681 | GNPDA1;N |
| 0 | 0 | 10.29219363 | 55.48409681 | SLC27A1;T |
| 0 | 0 | 10.29219363 | 55.48409681 | DNMT1;PD |
| 0 | 0 | 10.29219363 | 55.48409681 | GSTM1;GS |
| 0 | 0 | 2.032529657 | 10.82144122 | MAP10;GE |
| 0 | 0 | 2.57793345 | 13.66706323 | ARSA;ABCA |
| 0 | 0 | 6.434336824 | 34.01512639 | BLM;WRN; |
| 0 | 0 | 6.434336824 | 34.01512639 | ACSS3;BDH |
| 0 | 0 | 6.434336824 | 34.01512639 | BLM;WRN; |
| 0 | 0 | 6.434336824 | 34.01512639 | BLM;WRN; |
| 0 | 0 | 6.434336824 | 34.01512639 | GNPDA1;N |
| 0 | 0 | 2.461135667 | 12.98862647 | HACD1;PPI |
| 0 | 0 | 3.312062937 | 17.44624021 | CENPX;CEN |
| 0 | 0 | 3.312062937 | 17.44624021 | CENPX;CEN |
| 0 | 0 | 4.159556601 | 21.86072029 | MBD4;NEIL |
| 0 | 0 | 4.159556601 | 21.86072029 | GGCT;SLC2 |
| 0 | 0 | 2.651028446 | 13.68915659 | DCLRE1B;B |
| 0 | 0 | 1.831063608 | 9.32656466 | SLC48A1;C |
| 0 | 0 | 1.639787611 | 8.33465671 | GPI;GDE1;I |
| 0 | 0 | 2.743092597 | 13.8054988 | RFC5;RFC3 |
| 0 | 0 | 3.161335823 | 15.90159424 | PLA2G15;S |
| 0 | 0 | 3.161335823 | 15.90159424 | ACBD6;ACO |
| 0 | 0 | 3.161335823 | 15.90159424 | ACBD6;ACO |
| 0 | 0 | 4.633697076 | 23.28274267 | BLM;WRN; |
| 0 | 0 | 4.633697076 | 23.28274267 | NXT1;XPO1 |
| 0 | 0 | 4.633697076 | 23.28274267 | ENTPD5;AK |
| 0 | 0 | 2.577242888 | 12.84490258 | BLM;WRN; |
| 0 | 0 | 2.577242888 | 12.84490258 | SLC27A1;P |
| 0 | 0 | 3.433813893 | 17.05888329 | ZRANB3;BL |
| 0 | 0 | 3.433813893 | 17.05888329 | DDB1;PARP |
| 0 | 0 | 2.862556479 | 14.06016668 | ATP6V1A;D |
| 0 | 0 | 5.514834206 | 26.72442848 | GNPDA1;N |
| 0 | 0 | 5.514834206 | 26.72442848 | GCSH;SHM |
| 0 | 0 | 5.514834206 | 26.72442848 | GMPS;ADS |
| 0 | 0 | 2.657220582 | 12.87286238 | RFC5;POLA |
| 0 | 0 | 1.448648555 | 6.962778846 | LRSAM1;TF |
| 0 | 0 | 3.023715415 | 14.52919797 | CENPX;CEN |
| 0 | 0 | 7.718709115 | 36.99326563 | PLA2G15;G |
| 0 | 0 | 7.718709115 | 36.99326563 | DDT;STAP1 |
| 0 | 0 | 7.718709115 | 36.99326563 | WNK1;WN |
| 0 | 0 | 7.718709115 | 36.99326563 | DNMT1;NF |
| 0 | 0 | 7.718709115 | 36.99326563 | CHRNB1;PI |

| | | | | |
|---|---|---|---|---|
| 0 | 0 | 2.133970279 | 10.07650335 | HACD1;PPI… |
| 0 | 0 | 3.252902899 | 15.35553848 | CYP27A1;A… |
| 0 | 0 | 2.760166157 | 13.00745834 | PCGF6;CBX… |
| 0 | 0 | 4.212213801 | 19.75555457 | ALG8;ALG9… |
| 0 | 0 | 3.604541485 | 16.84200888 | RFC5;RFC3… |
| 0 | 0 | 1.255181003 | 5.831780606 | PGAP1;LRF… |
| 0 | 0 | 2.897563374 | 13.30485296 | CYP27A1;A… |
| 0 | 0 | 1.910896404 | 8.753717265 | VKORC1L1… |
| 0 | 0 | 2.664837236 | 12.05125314 | BRIP1;CHE… |
| 0 | 0 | 3.090083006 | 13.86807003 | SLC27A1;S… |
| 0 | 0 | 3.090083006 | 13.86807003 | ALG8;RRM… |
| 0 | 0 | 4.825207243 | 21.51303002 | ALG8;ALG9… |
| 0 | 0 | 4.825207243 | 21.51303002 | GNPDA1;N… |
| 0 | 0 | 3.379066594 | 14.90790808 | DCTD;AK1;… |
| 0 | 0 | 3.379066594 | 14.90790808 | DDB1;PARI… |
| 0 | 0 | 3.860977739 | 16.93050125 | IMPACT;PK… |
| 0 | 0 | 3.860977739 | 16.93050125 | BCAS3;KAN… |
| 0 | 0 | 3.860977739 | 16.93050125 | MORN4;RT… |
| 0 | 0 | 11.57432432 | 50.58341126 | TMLHE;AC… |
| 0 | 0 | 11.57432432 | 50.58341126 | TMLHE;AC… |
| 0 | 0 | 11.57432432 | 50.58341126 | POLA1;PRI… |
| 0 | 0 | 11.57432432 | 50.58341126 | RAD51;FAN… |
| 0 | 0 | 11.57432432 | 50.58341126 | GSTZ1;QDF… |
| 0 | 0 | 11.57432432 | 50.58341126 | NAAA;GDE… |
| 0 | 0 | 11.57432432 | 50.58341126 | OXCT1;FITI… |
| 0 | 0 | 11.57432432 | 50.58341126 | SLC46A1;A… |
| 0 | 0 | 11.57432432 | 50.58341126 | GSTZ1;QDF… |
| 0 | 0 | 11.57432432 | 50.58341126 | MCCC2;IVD… |
| 0 | 0 | 11.57432432 | 50.58341126 | NAAA;GDE… |
| 0 | 0 | 11.57432432 | 50.58341126 | DDT;STAP1… |
| 0 | 0 | 11.57432432 | 50.58341126 | LGALS3;LG… |
| 0 | 0 | 11.57432432 | 50.58341126 | RAD51;XRC… |
| 0 | 0 | 11.57432432 | 50.58341126 | CCDC88A;E… |
| 0 | 0 | 11.57432432 | 50.58341126 | GRN;ALOX… |
| 0 | 0 | 1.397359381 | 6.093824225 | DGKA;TSSK… |
| 0 | 0 | 1.826059912 | 7.922018374 | TSPYL2;WV… |
| 0 | 0 | 2.318993435 | 10.03749921 | CASP9;ACE… |
| 0 | 0 | 6.174618404 | 26.54698737 | ERI1;ERI2;E… |
| 0 | 0 | 6.174618404 | 26.54698737 | ERI1;ERI2;E… |
| 0 | 0 | 6.174618404 | 26.54698737 | ALDH3A2;F… |
| 0 | 0 | 6.174618404 | 26.54698737 | GALE;GALT… |
| 0 | 0 | 6.174618404 | 26.54698737 | ACSS3;BDH… |
| 0 | 0 | 6.174618404 | 26.54698737 | CYB5A;CYB… |
| 0 | 0 | 6.174618404 | 26.54698737 | ABHD4;NA… |
| 0 | 0 | 6.174618404 | 26.54698737 | AK1;CMPK… |
| 0 | 0 | 6.174618404 | 26.54698737 | AK1;CMPK… |
| 0 | 0 | 6.174618404 | 26.54698737 | AK1;CMPK… |

| | | | | |
|---|---|---|---|---|
| 0 | 0 | 6.174618404 | 26.54698737 | CIDEA;PLIN |
| 0 | 0 | 1.962634197 | 8.400843867 | MAP10;PLI |
| 0 | 0 | 2.942769768 | 12.56200937 | BDH2;HEPI |
| 0 | 0 | 1.745317916 | 7.339255534 | CD63;SPAF |
| 0 | 0 | 2.674371302 | 11.22339043 | TCHP;CDK1 |
| 0 | 0 | 2.492630154 | 10.38576042 | PCGF6;CBX |
| 0 | 0 | 2.361439195 | 9.831979829 | SMPD3;NE |
| 0 | 0 | 4.288830716 | 17.64567295 | NCEH1;DH |
| 0 | 0 | 4.288830716 | 17.64567295 | AK1;CMPK |
| 0 | 0 | 4.288830716 | 17.64567295 | AK1;CMPK |
| 0 | 0 | 4.288830716 | 17.64567295 | AGO4;TUT |
| 0 | 0 | 4.288830716 | 17.64567295 | RNF128;GL |
| 0 | 0 | 4.288830716 | 17.64567295 | COQ3;PDS |
| 0 | 0 | 4.288830716 | 17.64567295 | NDUFA5;E |
| 0 | 0 | 3.563777994 | 14.63169486 | ACER2;ABC |
| 0 | 0 | 3.563777994 | 14.63169486 | DCLRE1B;D |
| 0 | 0 | 2.808848644 | 11.40956683 | GGA2;BBS |
| 0 | 0 | 1.999720451 | 8.088160835 | RFC5;RFC3 |
| 0 | 0 | 2.575174825 | 10.33544 | DCLRE1B;R |
| 0 | 0 | 1.933252959 | 7.714156656 | PIGQ;GDE1 |
| 0 | 0 | 2.042165638 | 8.146450568 | SLC25A1;S |
| 0 | 0 | 1.580439851 | 6.286567717 | PI4K2B;MT |
| 0 | 0 | 3.003275109 | 11.83993386 | ABCD4;AC |
| 0 | 0 | 2.093503722 | 8.245993463 | ATP6V1A;S |
| 0 | 0 | 2.093503722 | 8.245993463 | RFC5;RFC3 |
| 0 | 0 | 1.569671789 | 6.130713085 | PSMD10;C |
| 0 | 0 | 5.145224597 | 19.97672504 | DIP2A;MLY |
| 0 | 0 | 5.145224597 | 19.97672504 | NEU3;GM2 |
| 0 | 0 | 5.145224597 | 19.97672504 | SLC27A1;C |
| 0 | 0 | 5.145224597 | 19.97672504 | SGPL1;FUC |
| 0 | 0 | 5.145224597 | 19.97672504 | PRDX2;PRD |
| 0 | 0 | 5.145224597 | 19.97672504 | ERI1;ERI2;E |
| 0 | 0 | 5.145224597 | 19.97672504 | MFAP4;FEI |
| 0 | 0 | 2.686572834 | 10.38811898 | ACER2;PM |
| 0 | 0 | 2.686572834 | 10.38811898 | RBL1;NEK4 |
| 0 | 0 | 2.686572834 | 10.38811898 | STAR;SRD5 |
| 0 | 0 | 3.309035356 | 12.73587535 | SLC46A1;N |
| 0 | 0 | 3.309035356 | 12.73587535 | CCDC57;TU |
| 0 | 0 | 2.483063811 | 9.532734934 | ACBD6;MC |
| 0 | 0 | 2.483063811 | 9.532734934 | CENPX;CEN |
| 0 | 0 | 2.483063811 | 9.532734934 | DNMT1;PD |
| 0 | 0 | 1.318728005 | 5.057568996 | TSSK1B;AA |
| 0 | 0 | 3.859729494 | 14.69088408 | OXSM;ACA |
| 0 | 0 | 3.859729494 | 14.69088408 | KIAA0586; |
| 0 | 0 | 3.859729494 | 14.69088408 | COQ3;PDS |
| 0 | 0 | 1.770629489 | 6.723173808 | GALNT12;C |
| 0 | 0 | 1.738340908 | 6.577449925 | ABCA2;SLC |

| | | | | |
|---|---|---|---|---|
| 0 | 0 | 7.715780296 | 29.05719477 | ABHD10;EI |
| 0 | 0 | 7.715780296 | 29.05719477 | XRCC5;NU( |
| 0 | 0 | 7.715780296 | 29.05719477 | QDPR;KYA |
| 0 | 0 | 7.715780296 | 29.05719477 | GALE;GALT |
| 0 | 0 | 7.715780296 | 29.05719477 | GMDS;PMI |
| 0 | 0 | 7.715780296 | 29.05719477 | FUCA1;FU( |
| 0 | 0 | 7.715780296 | 29.05719477 | FUCA1;FU( |
| 0 | 0 | 7.715780296 | 29.05719477 | RPS6KA5;N |
| 0 | 0 | 7.715780296 | 29.05719477 | ILVBL;ACAI |
| 0 | 0 | 7.715780296 | 29.05719477 | KANK1;ABI |
| 0 | 0 | 7.715780296 | 29.05719477 | SHMT2;GN |
| 0 | 0 | 7.715780296 | 29.05719477 | ETAA1;RFV |
| 0 | 0 | 7.715780296 | 29.05719477 | MMP3;NO |
| 0 | 0 | 7.715780296 | 29.05719477 | ATP2B4;TX |
| 0 | 0 | 2.845047116 | 10.61262524 | NEU3;ABC |
| 0 | 0 | 2.17941988 | 8.105554763 | ATP6V1A;T |
| 0 | 0 | 1.901050272 | 7.035325442 | ABCA2;GD |
| 0 | 0 | 2.574486675 | 9.479084988 | PIGS;PIGU; |
| 0 | 0 | 3.088258402 | 11.15434898 | PRDX2;PRI |
| 0 | 0 | 3.088258402 | 11.15434898 | MSR1;ARV |
| 0 | 0 | 3.088258402 | 11.15434898 | GRN;DMXL |
| 0 | 0 | 1.870282152 | 6.718789642 | TMEM161 |
| 0 | 0 | 1.870282152 | 6.718789642 | ACBD6;AC( |
| 0 | 0 | 2.124814086 | 7.611171503 | STX12;MSF |
| 0 | 0 | 2.124814086 | 7.611171503 | CASP9;MSI |
| 0 | 0 | 1.970021634 | 7.027887488 | ABCA2;ABI |
| 0 | 0 | 2.207258417 | 7.817502965 | TTC26;SPA |
| 0 | 0 | 2.702641921 | 9.544013436 | PLA2G15;A |
| 0 | 0 | 2.702641921 | 9.544013436 | TCHP;KANI |
| 0 | 0 | 2.702641921 | 9.544013436 | ABCD4;ALI |
| 0 | 0 | 3.508646676 | 12.38009998 | ACER2;NEU |
| 0 | 0 | 3.508646676 | 12.38009998 | SLCO2B1;B |
| 0 | 0 | 3.508646676 | 12.38009998 | RTEL1;RAD |
| 0 | 0 | 3.508646676 | 12.38009998 | SLCO2B1;B |
| 0 | 0 | 3.508646676 | 12.38009998 | GMPS;ADS |
| 0 | 0 | 3.508646676 | 12.38009998 | SCARB2;PD |
| 0 | 0 | 3.508646676 | 12.38009998 | SMPD3;SPI |
| 0 | 0 | 3.508646676 | 12.38009998 | BCAS3;KAN |
| 0 | 0 | 3.508646676 | 12.38009998 | ACADVL;FA |
| 0 | 0 | 3.508646676 | 12.38009998 | SLX4;CENP |
| 0 | 0 | 4.409943306 | 15.54132076 | PPP1R3C;G |
| 0 | 0 | 4.409943306 | 15.54132076 | PRDX2;PRI |
| 0 | 0 | 4.409943306 | 15.54132076 | ABCA2;CN |
| 0 | 0 | 4.409943306 | 15.54132076 | CCNF;TRIM |
| 0 | 0 | 4.409943306 | 15.54132076 | ORC3;DON |
| 0 | 0 | 4.409943306 | 15.54132076 | SMPD3;SPI |
| 0 | 0 | 4.409943306 | 15.54132076 | SMPD3;SPI |

| | | | | |
|---|---|---|---|---|
| 0 | 0 | 4.409943306 | 15.54132076 | RAD50;RAI |
| 0 | 0 | 1.537016025 | 5.416426455 | POGLUT1;( |
| 0 | 0 | 2.015831034 | 7.067697383 | ATP6V1A;S |
| 0 | 0 | 1.932027145 | 6.668080453 | ABHD4;AB |
| 0 | 0 | 2.072871989 | 7.151515532 | CLIP3;LGAI |
| 0 | 0 | 2.072871989 | 7.151515532 | TRPM2;SH |
| 0 | 0 | 2.145824224 | 7.298727063 | RFC5;RFC3 |
| 0 | 0 | 2.895078568 | 9.82183541 | SLC25A1;G |
| 0 | 0 | 2.242386646 | 7.541781176 | SLC25A1;G |
| 0 | 0 | 2.242386646 | 7.541781176 | PIGU;SCCP |
| 0 | 0 | 2.242386646 | 7.541781176 | GALNS;TST |
| 0 | 0 | 2.573799127 | 8.60849937 | CDK4;PDGI |
| 0 | 0 | 2.573799127 | 8.60849937 | SCARB2;RN |
| 0 | 0 | 2.573799127 | 8.60849937 | ACADVL;AI |
| 0 | 0 | 2.376180395 | 7.939274771 | TWIST1;PP |
| 0 | 0 | 5.786508282 | 19.06363776 | HADHB;HA |
| 0 | 0 | 5.786508282 | 19.06363776 | NEU3;GM2 |
| 0 | 0 | 5.786508282 | 19.06363776 | SLC48A1;A |
| 0 | 0 | 5.786508282 | 19.06363776 | GPI;VWF;H |
| 0 | 0 | 5.786508282 | 19.06363776 | AHI1;CITED |
| 0 | 0 | 5.786508282 | 19.06363776 | MCCC2;IVI |
| 0 | 0 | 5.786508282 | 19.06363776 | AGO4;DICE |
| 0 | 0 | 5.786508282 | 19.06363776 | TRIM37;CD |
| 0 | 0 | 5.786508282 | 19.06363776 | MDFIC;RAI |
| 0 | 0 | 5.786508282 | 19.06363776 | MDFIC;RAI |
| 0 | 0 | 5.786508282 | 19.06363776 | KANK1;ABI |
| 0 | 0 | 5.786508282 | 19.06363776 | SHMT2;GN |
| 0 | 0 | 5.786508282 | 19.06363776 | CD63;LOXL |
| 0 | 0 | 5.786508282 | 19.06363776 | WNK1;WN |
| 0 | 0 | 5.786508282 | 19.06363776 | ARL3;WDR |
| 0 | 0 | 5.786508282 | 19.06363776 | MAPKBP1; |
| 0 | 0 | 5.786508282 | 19.06363776 | DENND1A; |
| 0 | 0 | 5.786508282 | 19.06363776 | NFATC3;PD |
| 0 | 0 | 5.786508282 | 19.06363776 | ABCD4;AB( |
| 0 | 0 | 1.78365806 | 5.858946981 | STX8;ATP2 |
| 0 | 0 | 3.216077661 | 10.53817717 | ACER2;QDI |
| 0 | 0 | 3.216077661 | 10.53817717 | POLA1;RFC |
| 0 | 0 | 3.216077661 | 10.53817717 | DDB1;KDM |
| 0 | 0 | 3.216077661 | 10.53817717 | SLC46A1;C |
| 0 | 0 | 3.216077661 | 10.53817717 | RBL1;AKT3 |
| 0 | 0 | 3.216077661 | 10.53817717 | PARP1;PRK |
| 0 | 0 | 1.931619256 | 6.312669327 | HIPK4;SBK |
| 0 | 0 | 3.858482338 | 12.39259088 | GALNS;GLE |
| 0 | 0 | 3.858482338 | 12.39259088 | GCDH;HYK |
| 0 | 0 | 3.858482338 | 12.39259088 | GCDH;HYK |
| 0 | 0 | 3.858482338 | 12.39259088 | MGME1;PA |
| 0 | 0 | 3.858482338 | 12.39259088 | TCHP;CDK1 |

| | | | | |
|---|---|---|---|---|
| 0 | 0 | 3.858482338 | 12.39259088 | DDT;STAP1 |
| 0 | 0 | 3.858482338 | 12.39259088 | CLIP3;TBCD |
| 0 | 0 | 3.858482338 | 12.39259088 | GGCT;BDH |
| 0 | 0 | 3.858482338 | 12.39259088 | SCIN;PICK1 |
| 0 | 0 | 3.858482338 | 12.39259088 | SPHK2;NAA |
| 0 | 0 | 1.803197547 | 5.777801599 | ATP6V1A;N |
| 0 | 0 | 1.976235529 | 6.324913072 | NDST1;UG |
| 0 | 0 | 2.724625773 | 8.689337957 | CCDC88A;T |
| 0 | 0 | 2.724625773 | 8.689337957 | CEMIP2;AE |
| 0 | 0 | 2.724625773 | 8.689337957 | RAD51B;AT |
| 0 | 0 | 2.724625773 | 8.689337957 | ABHD10;PI |
| 0 | 0 | 1.365035929 | 4.348183673 | CPM;CUL7 |
| 0 | 0 | 2.28804427 | 7.28489116 | LIPT1;MINI |
| 0 | 0 | 2.28804427 | 7.28489116 | SESN3;RRA |
| 0 | 0 | 2.28804427 | 7.28489116 | TTC26;STIL |
| 0 | 0 | 2.456669313 | 7.785474442 | ALDH3A2;A |
| 0 | 0 | 1.773536402 | 5.506398198 | ATP6V1A;N |
| 0 | 0 | 2.106246027 | 6.489405161 | NDUFA5;C |
| 0 | 0 | 2.106246027 | 6.489405161 | RTEL1;SLF1 |
| 0 | 0 | 2.968519264 | 9.046484812 | BACE1;ACH |
| 0 | 0 | 2.968519264 | 9.046484812 | EPS8;TRPM |
| 0 | 0 | 2.968519264 | 9.046484812 | CEMIP2;HE |
| 0 | 0 | 2.968519264 | 9.046484812 | ODAD1;OD |
| 0 | 0 | 2.968519264 | 9.046484812 | PARP1;PRK |
| 0 | 0 | 2.968519264 | 9.046484812 | GSTM1;GS |
| 0 | 0 | 2.968519264 | 9.046484812 | RTEL1;SLX4 |
| 0 | 0 | 1.85414442 | 5.647286493 | SLC25A1;G |
| 0 | 0 | 2.206203582 | 6.694823854 | P2RX7;WN |
| 0 | 0 | 1.980424473 | 5.967749127 | CYP27A1;A |
| 0 | 0 | 2.349724701 | 7.058185561 | GSTZ1;KYA |
| 0 | 0 | 2.349724701 | 7.058185561 | CCDC57;TU |
| 0 | 0 | 2.349724701 | 7.058185561 | CD63;KCNI |
| 0 | 0 | 2.573112178 | 7.719447843 | PARP1;PRK |
| 0 | 0 | 2.573112178 | 7.719447843 | EPS8;KANK |
| 0 | 0 | 1.888188976 | 5.605606908 | GALNS;CEN |
| 0 | 0 | 3.429568251 | 10.07147516 | ACOT8;HSI |
| 0 | 0 | 3.429568251 | 10.07147516 | NASP;HAT1 |
| 0 | 0 | 3.429568251 | 10.07147516 | CNTNAP1;I |
| 0 | 0 | 3.429568251 | 10.07147516 | RBL1;AKT3 |
| 0 | 0 | 3.429568251 | 10.07147516 | ZRANB3;BL |
| 0 | 0 | 3.429568251 | 10.07147516 | MDFIC;RAE |
| 0 | 0 | 3.429568251 | 10.07147516 | CIB1;IGF1;I |
| 0 | 0 | 3.429568251 | 10.07147516 | CIB1;IGF1;I |
| 0 | 0 | 3.429568251 | 10.07147516 | LGALS3;SP |
| 0 | 0 | 3.429568251 | 10.07147516 | NEK4;MIF;Y |
| 0 | 0 | 1.679876539 | 4.892398903 | NEK7;HAUS |
| 0 | 0 | 4.628945074 | 13.47968695 | PON2;PDX |

| | | | | |
|---|---|---|---|---|
| 0 | 0 | 4.628945074 | 13.47968695 | ATP2B4;SI… |
| 0 | 0 | 4.628945074 | 13.47968695 | HGF;SIRT2… |
| 0 | 0 | 4.628945074 | 13.47968695 | CHEK2;CH… |
| 0 | 0 | 4.628945074 | 13.47968695 | MFAP4;TN… |
| 0 | 0 | 4.628945074 | 13.47968695 | PEX6;HAC… |
| 0 | 0 | 4.628945074 | 13.47968695 | SORD;AKR… |
| 0 | 0 | 4.628945074 | 13.47968695 | PCGF6;TRI… |
| 0 | 0 | 4.628945074 | 13.47968695 | SLC27A1;C… |
| 0 | 0 | 4.628945074 | 13.47968695 | MSRA;MT… |
| 0 | 0 | 4.628945074 | 13.47968695 | MGME1;P… |
| 0 | 0 | 4.628945074 | 13.47968695 | PRKCA;TR… |
| 0 | 0 | 4.628945074 | 13.47968695 | HDAC6;SIR… |
| 0 | 0 | 4.628945074 | 13.47968695 | CHPT1;PLA… |
| 0 | 0 | 4.628945074 | 13.47968695 | SMC6;RAD… |
| 0 | 0 | 4.628945074 | 13.47968695 | LGALS3;LG… |
| 0 | 0 | 4.628945074 | 13.47968695 | APOA4;AB… |
| 0 | 0 | 4.628945074 | 13.47968695 | ORC3;MC… |
| 0 | 0 | 4.628945074 | 13.47968695 | MTRES1;S… |
| 0 | 0 | 4.628945074 | 13.47968695 | WNK1;WN… |
| 0 | 0 | 4.628945074 | 13.47968695 | ZRANB3;B… |
| 0 | 0 | 4.628945074 | 13.47968695 | ATP2B4;SI… |
| 0 | 0 | 4.628945074 | 13.47968695 | HGF;SIRT2… |
| 0 | 0 | 4.628945074 | 13.47968695 | MSH6;PM… |
| 0 | 0 | 2.13000708 | 6.161332813 | P2RX7;LG… |
| 0 | 0 | 2.13000708 | 6.161332813 | TRPM2;BC… |
| 0 | 0 | 2.13000708 | 6.161332813 | MOB1B;IM… |
| 0 | 0 | 1.562452132 | 4.48559876 | B4GALT2;S… |
| 0 | 0 | 2.25169214 | 6.412892949 | GSTM1;GS… |
| 0 | 0 | 2.25169214 | 6.412892949 | SLC27A1;S… |
| 0 | 0 | 1.782629187 | 5.059104994 | TOM1L2;P… |
| 0 | 0 | 2.756326353 | 7.822104824 | GCDH;HYK… |
| 0 | 0 | 2.756326353 | 7.822104824 | MSRA;MT… |
| 0 | 0 | 2.756326353 | 7.822104824 | SPR;SPHK2… |
| 0 | 0 | 2.756326353 | 7.822104824 | BLM;RAD5… |
| 0 | 0 | 2.756326353 | 7.822104824 | SLC46A1;… |
| 0 | 0 | 2.756326353 | 7.822104824 | NDST1;UG… |
| 0 | 0 | 2.756326353 | 7.822104824 | BCAS3;CD… |
| 0 | 0 | 2.756326353 | 7.822104824 | CBR1;PTG… |
| 0 | 0 | 1.424891559 | 4.035642654 | CD63;SPA… |
| 0 | 0 | 1.731573465 | 4.895000022 | SHMT2;PK… |
| 0 | 0 | 1.6557844 | 4.678371218 | GOLPH3L;… |
| 0 | 0 | 2.437547382 | 6.883169992 | LGALS3;SP… |
| 0 | 0 | 1.985664336 | 5.605922437 | TRPM2;SL… |
| 0 | 0 | 1.985664336 | 5.605922437 | SLC25A1;G… |
| 0 | 0 | 1.985664336 | 5.605922437 | MAP10;RA… |
| 0 | 0 | 1.985664336 | 5.605922437 | CD63;AHI1… |
| 0 | 0 | 1.482003078 | 4.175524405 | PSMD10;P… |

| | | | | |
|---|---|---|---|---|
| 0 | 0 | 1.883605106 | 5.235860238 | ABCA2;CC |
| 0 | 0 | 2.058890345 | 5.677803566 | EPM2A;SE |
| 0 | 0 | 1.515668191 | 4.163441468 | CBR1;STIL; |
| 0 | 0 | 1.748895586 | 4.790646768 | SCARB2;A |
| 0 | 0 | 2.161502183 | 5.838236853 | ODAD1;D |
| 0 | 0 | 2.161502183 | 5.838236853 | PKIB;RTEL1 |
| 0 | 0 | 2.161502183 | 5.838236853 | GEN1;STIL; |
| 0 | 0 | 2.161502183 | 5.838236853 | BCAS3;RIP |
| 0 | 0 | 2.161502183 | 5.838236853 | NPAT;KLF1 |
| 0 | 0 | 3.086436982 | 8.309617421 | TRPM2;AT |
| 0 | 0 | 3.086436982 | 8.309617421 | ACOT8;QP |
| 0 | 0 | 3.086436982 | 8.309617421 | C1QTNF1; |
| 0 | 0 | 3.086436982 | 8.309617421 | SESN3;BM |
| 0 | 0 | 3.086436982 | 8.309617421 | NDC1;AHC |
| 0 | 0 | 3.086436982 | 8.309617421 | WNK1;WN |
| 0 | 0 | 3.086436982 | 8.309617421 | NFU1;MA |
| 0 | 0 | 3.086436982 | 8.309617421 | PKIB;MST1 |
| 0 | 0 | 3.086436982 | 8.309617421 | POGLUT1; |
| 0 | 0 | 3.086436982 | 8.309617421 | BLM;XRCC |
| 0 | 0 | 1.4108376 | 3.793434535 | ATP23;AT |
| 0 | 0 | 1.281834604 | 3.43919183 | LRR1;DSC |
| 0 | 0 | 1.838653258 | 4.908615295 | ODAD1;D |
| 0 | 0 | 2.315539066 | 6.157727863 | TRPM2;PD |
| 0 | 0 | 2.315539066 | 6.157727863 | STN1;SLX4 |
| 0 | 0 | 1.6096078 | 4.27944298 | MCUR1;M |
| 0 | 0 | 1.638713017 | 4.340817645 | ABCD3;SR |
| 0 | 0 | 2.572425829 | 6.805524457 | ABCA2;NE |
| 0 | 0 | 2.572425829 | 6.805524457 | ITGB3;TRE |
| 0 | 0 | 2.572425829 | 6.805524457 | RAD51B;C |
| 0 | 0 | 2.572425829 | 6.805524457 | AGO4;ZC3 |
| 0 | 0 | 2.572425829 | 6.805524457 | GMPS;ADS |
| 0 | 0 | 2.572425829 | 6.805524457 | CUL7;PDX |
| 0 | 0 | 2.572425829 | 6.805524457 | ABCA2;M |
| 0 | 0 | 1.716411379 | 4.537726753 | PSMD10;C |
| 0 | 0 | 1.23236973 | 3.25343448 | GPI;GHDC; |
| 0 | 0 | 1.992361786 | 5.238555063 | BCAS3;RIP |
| 0 | 0 | 1.427297032 | 3.708564515 | SLC27A1;C |
| 0 | 0 | 3.857236269 | 10.00372234 | BACE1;AP |
| 0 | 0 | 3.857236269 | 10.00372234 | RTN4RL2; |
| 0 | 0 | 3.857236269 | 10.00372234 | HSD17B14 |
| 0 | 0 | 3.857236269 | 10.00372234 | PFKL;ALDC |
| 0 | 0 | 3.857236269 | 10.00372234 | GSTM1;AL |
| 0 | 0 | 3.857236269 | 10.00372234 | GSTM1;AL |
| 0 | 0 | 3.857236269 | 10.00372234 | P2RX7;UB |
| 0 | 0 | 3.857236269 | 10.00372234 | C1QTNF1; |
| 0 | 0 | 3.857236269 | 10.00372234 | NFATC3;P |
| 0 | 0 | 3.857236269 | 10.00372234 | PEX6;HAC |

| | | | | |
|---|---|---|---|---|
| 0 | 0 | 3.857236269 | 10.00372234 | MED25;ZN . |
| 0 | 0 | 3.857236269 | 10.00372234 | DAG1;EMII . |
| 0 | 0 | 3.857236269 | 10.00372234 | MCUR1;AT . |
| 0 | 0 | 3.857236269 | 10.00372234 | ANKRD39;[illegible] . |
| 0 | 0 | 3.857236269 | 10.00372234 | SHMT2;GM . |
| 0 | 0 | 3.857236269 | 10.00372234 | RINT1;PGA . |
| 0 | 0 | 3.857236269 | 10.00372234 | GAS6;IER3I . |
| 0 | 0 | 3.857236269 | 10.00372234 | MST1;BRCA . |
| 0 | 0 | 3.857236269 | 10.00372234 | GPC1;KLHL . |
| 0 | 0 | 3.857236269 | 10.00372234 | CLU;HDAC . |
| 0 | 0 | 3.857236269 | 10.00372234 | AGO4;DICE . |
| 0 | 0 | 1.878118503 | 4.859170713 | OAT;MTHF . |
| 0 | 0 | 1.878118503 | 4.859170713 | MED25;CD . |
| 0 | 0 | 1.795792192 | 4.604279865 | TSPYL2;CC . |
| 0 | 0 | 2.078249916 | 5.324755552 | B4GALT2;G . |
| 0 | 0 | 2.078249916 | 5.324755552 | P2RX7;ATL . |
| 0 | 0 | 1.483119164 | 3.736904105 | SLF1;TFRC; . |
| 0 | 0 | 1.220322447 | 3.063721958 | GPI;GHDC; . |
| 0 | 0 | 1.929991263 | 4.83868503 | NXT1;XPO . |
| 0 | 0 | 1.929991263 | 4.83868503 | MSH6;BCA . |
| 0 | 0 | 1.929991263 | 4.83868503 | CLIP3;TBC . |
| 0 | 0 | 2.205150589 | 5.525014325 | PLA2G15;A . |
| 0 | 0 | 1.458141712 | 3.618946297 | PI4K2B;MT . |
| 0 | 0 | 1.828590817 | 4.529050825 | TFAP4;E2F . |
| 0 | 0 | 2.805693217 | 6.940605635 | DIP2A;MLY . |
| 0 | 0 | 2.805693217 | 6.940605635 | STAR;FDX1 . |
| 0 | 0 | 2.805693217 | 6.940605635 | TRPM2;AT . |
| 0 | 0 | 2.805693217 | 6.940605635 | P2RX7;P2R . |
| 0 | 0 | 2.805693217 | 6.940605635 | NDUFA5;E . |
| 0 | 0 | 2.805693217 | 6.940605635 | ALOX5;MG . |
| 0 | 0 | 2.805693217 | 6.940605635 | CYB5R3;M . |
| 0 | 0 | 2.805693217 | 6.940605635 | XRCC5;POT . |
| 0 | 0 | 2.805693217 | 6.940605635 | NEU3;DAB . |
| 0 | 0 | 2.805693217 | 6.940605635 | TNFSF18;C . |
| 0 | 0 | 2.411512871 | 5.953050704 | SRD5A1;SP . |
| 0 | 0 | 2.411512871 | 5.953050704 | UGDH;GPC . |
| 0 | 0 | 2.411512871 | 5.953050704 | MSR1;CIDE . |
| 0 | 0 | 2.411512871 | 5.953050704 | DNMT1;PD . |
| 0 | 0 | 2.411512871 | 5.953050704 | ABCA2;ACA . |
| 0 | 0 | 1.526036339 | 3.733480009 | LIPT1;CPM . |
| 0 | 0 | 2.001164483 | 4.864514415 | DNAI4;TTC . |
| 0 | 0 | 2.001164483 | 4.864514415 | PSMD10;V . |
| 0 | 0 | 2.001164483 | 4.864514415 | STIL;KDM2 . |
| 0 | 0 | 2.001164483 | 4.864514415 | SLF1;MRNI . |
| 0 | 0 | 1.364343094 | 3.300483821 | SLC27A1;S . |
| 0 | 0 | 1.211436862 | 2.92759495 | GPI;GHDC; . |
| 0 | 0 | 1.492499003 | 3.582710139 | RARS2;FAS . |

| | | | | |
|---|---|---|---|---|
| 0 | 0 | 1.399760386 | 3.351387104 | H2AZ1;BLN… |
| 0 | 0 | 1.87140077 | 4.473944619 | GPI;NEU3;… |
| 0 | 0 | 1.87140077 | 4.473944619 | SLC27A1;A… |
| 0 | 0 | 1.87140077 | 4.473944619 | LRSAM1;SL… |
| 0 | 0 | 1.781603012 | 4.2242183 | PRKCG;SLF… |
| 0 | 0 | 1.781603012 | 4.2242183 | CLIP3;DAB… |
| 0 | 0 | 1.665482991 | 3.941972535 | DCLRE1B;D… |
| 0 | 0 | 1.665482991 | 3.941972535 | WWTR1;UI… |
| 0 | 0 | 1.715784871 | 4.056949439 | PLA2G15;A… |
| 0 | 0 | 2.104797429 | 4.970478527 | PPP1R3C;G… |
| 0 | 0 | 2.104797429 | 4.970478527 | PIGU;PGAP… |
| 0 | 0 | 2.104797429 | 4.970478527 | LRSAM1;M… |
| 0 | 0 | 2.104797429 | 4.970478527 | ACTN3;ATF… |
| 0 | 0 | 2.104797429 | 4.970478527 | NTNG2;SH… |
| 0 | 0 | 2.104797429 | 4.970478527 | NTNG2;CC… |
| 0 | 0 | 2.104797429 | 4.970478527 | CRCP;ICE2;… |
| 0 | 0 | 1.260955763 | 2.97180249 | FEN1;BLM;… |
| 0 | 0 | 1.475645575 | 3.442665381 | NDC1;LIPT… |
| 0 | 0 | 3.306015693 | 7.680184205 | QDPR;KYA… |
| 0 | 0 | 3.306015693 | 7.680184205 | MCUR1;M… |
| 0 | 0 | 3.306015693 | 7.680184205 | SESN3;SES… |
| 0 | 0 | 3.306015693 | 7.680184205 | NASP;HAT… |
| 0 | 0 | 3.306015693 | 7.680184205 | NASP;HAT… |
| 0 | 0 | 3.306015693 | 7.680184205 | DHRS7B;CH… |
| 0 | 0 | 3.306015693 | 7.680184205 | ARL6IP5;SL… |
| 0 | 0 | 3.306015693 | 7.680184205 | UNC119;PF… |
| 0 | 0 | 3.306015693 | 7.680184205 | IMPACT;CI… |
| 0 | 0 | 3.306015693 | 7.680184205 | RTN4RL2;F… |
| 0 | 0 | 3.306015693 | 7.680184205 | NDC1;AHC… |
| 0 | 0 | 3.306015693 | 7.680184205 | YBX1;CKAP… |
| 0 | 0 | 3.306015693 | 7.680184205 | PLA2G15;P… |
| 0 | 0 | 3.306015693 | 7.680184205 | CARMIL1;P… |
| 0 | 0 | 3.306015693 | 7.680184205 | DROSHA;D… |
| 0 | 0 | 3.306015693 | 7.680184205 | RACGAP1;I… |
| 0 | 0 | 3.306015693 | 7.680184205 | ETAA1;RFV… |
| 0 | 0 | 3.306015693 | 7.680184205 | CDH3;CLO… |
| 0 | 0 | 3.306015693 | 7.680184205 | CNPY2;TRE… |
| 0 | 0 | 3.306015693 | 7.680184205 | MST1;MTU… |
| 0 | 0 | 3.306015693 | 7.680184205 | RANGRF;CI… |
| 0 | 0 | 3.306015693 | 7.680184205 | THRA;HMG… |
| 0 | 0 | 3.306015693 | 7.680184205 | SESN3;SES… |
| 0 | 0 | 3.306015693 | 7.680184205 | DCLRE1B;D… |
| 0 | 0 | 3.306015693 | 7.680184205 | SLC25A15;… |
| 0 | 0 | 1.568704849 | 3.622273983 | ACHE;SLC2… |
| 0 | 0 | 1.489095356 | 3.433488801 | ZRANB3;FE… |
| 0 | 0 | 2.269530849 | 5.231970002 | FECH;COX1… |
| 0 | 0 | 2.269530849 | 5.231970002 | SHTN1;GO… |

| | | | | |
|---|---|---|---|---|
| 0 | 0 | 2.269530849 | 5.231970002 | DDT;MST1 … |
| 0 | 0 | 2.269530849 | 5.231970002 | SCARB2;NF… |
| 0 | 0 | 1.597675998 | 3.661754274 | CYP27A1;A… |
| 0 | 0 | 1.816256778 | 4.140636142 | TRPM2;AT … |
| 0 | 0 | 1.816256778 | 4.140636142 | ODAD3;STI… |
| 0 | 0 | 1.816256778 | 4.140636142 | DNAJB2;M … |
| 0 | 0 | 2.571740078 | 5.856306561 | DFFA;DNAS… |
| 0 | 0 | 2.571740078 | 5.856306561 | GSTZ1;QDF… |
| 0 | 0 | 2.571740078 | 5.856306561 | GNPDA1;N … |
| 0 | 0 | 2.571740078 | 5.856306561 | C1QTNF1;F… |
| 0 | 0 | 2.571740078 | 5.856306561 | PSMD10;H … |
| 0 | 0 | 2.571740078 | 5.856306561 | STIL;ALDH1… |
| 0 | 0 | 2.571740078 | 5.856306561 | CYB5R3;MT… |
| 0 | 0 | 2.571740078 | 5.856306561 | RRAGB;RRA… |
| 0 | 0 | 2.571740078 | 5.856306561 | ACER2;SGF… |
| 0 | 0 | 5.1416122 | 11.63167952 | TRIM37;HD… |
| 0 | 0 | 5.1416122 | 11.63167952 | GPAM;ACP… |
| 0 | 0 | 5.1416122 | 11.63167952 | TNFSF4;HM… |
| 0 | 0 | 5.1416122 | 11.63167952 | AKR1B1;SL … |
| 0 | 0 | 5.1416122 | 11.63167952 | GAS6;FOLF… |
| 0 | 0 | 5.1416122 | 11.63167952 | PLK4;CCDC… |
| 0 | 0 | 5.1416122 | 11.63167952 | PLK4;CCDC… |
| 0 | 0 | 5.1416122 | 11.63167952 | RRM1;RRM… |
| 0 | 0 | 5.1416122 | 11.63167952 | SLX4;SLX1A… |
| 0 | 0 | 5.1416122 | 11.63167952 | GLRX2;DCT… |
| 0 | 0 | 5.1416122 | 11.63167952 | SLX4;SLX1A… |
| 0 | 0 | 5.1416122 | 11.63167952 | SLC46A1;S … |
| 0 | 0 | 5.1416122 | 11.63167952 | POT1;BRCA… |
| 0 | 0 | 5.1416122 | 11.63167952 | CRTAM;CC … |
| 0 | 0 | 5.1416122 | 11.63167952 | SLC27A1;S … |
| 0 | 0 | 5.1416122 | 11.63167952 | PRKCA;VRK… |
| 0 | 0 | 5.1416122 | 11.63167952 | ADI1;MRI1 … |
| 0 | 0 | 5.1416122 | 11.63167952 | AHI1;CITED… |
| 0 | 0 | 5.1416122 | 11.63167952 | MSH6;MSH… |
| 0 | 0 | 5.1416122 | 11.63167952 | MGME1;PA… |
| 0 | 0 | 5.1416122 | 11.63167952 | VGLL4;TP7 … |
| 0 | 0 | 5.1416122 | 11.63167952 | SCAP;SOD1… |
| 0 | 0 | 5.1416122 | 11.63167952 | SCAP;SOD1… |
| 0 | 0 | 5.1416122 | 11.63167952 | PARK7;MM… |
| 0 | 0 | 5.1416122 | 11.63167952 | MMP3;HDA… |
| 0 | 0 | 5.1416122 | 11.63167952 | LAG3;TNFS… |
| 0 | 0 | 5.1416122 | 11.63167952 | DNMT1;IG … |
| 0 | 0 | 5.1416122 | 11.63167952 | ABCA2;ORI… |
| 0 | 0 | 5.1416122 | 11.63167952 | SCAP;SOD1… |
| 0 | 0 | 5.1416122 | 11.63167952 | KIF3A;KIFA … |
| 0 | 0 | 5.1416122 | 11.63167952 | DAG1;CLAS… |
| 0 | 0 | 5.1416122 | 11.63167952 | CCL19;IGF1… |

| | | | | |
|---|---|---|---|---|
| 0 | 0 | 5.1416122 | 11.63167952 | CNPY2;PPA |
| 0 | 0 | 5.1416122 | 11.63167952 | HMGB1;RT |
| 0 | 0 | 5.1416122 | 11.63167952 | IMPACT;EI |
| 0 | 0 | 5.1416122 | 11.63167952 | PDCD4;PPA |
| 0 | 0 | 5.1416122 | 11.63167952 | GCSH;LIPT |
| 0 | 0 | 5.1416122 | 11.63167952 | IMMP1L;IN |
| 0 | 0 | 5.1416122 | 11.63167952 | CMPK1;DH |
| 0 | 0 | 5.1416122 | 11.63167952 | APLP1;PRK |
| 0 | 0 | 5.1416122 | 11.63167952 | CARMIL1;C |
| 0 | 0 | 5.1416122 | 11.63167952 | DAG1;CLAS |
| 0 | 0 | 5.1416122 | 11.63167952 | CD63;ATP2 |
| 0 | 0 | 5.1416122 | 11.63167952 | APOA4;PRA |
| 0 | 0 | 5.1416122 | 11.63167952 | ABCA2;ARV |
| 0 | 0 | 5.1416122 | 11.63167952 | TST;MRPL1 |
| 0 | 0 | 5.1416122 | 11.63167952 | TST;MRPL1 |
| 0 | 0 | 5.1416122 | 11.63167952 | SUCLG2;AC |
| 0 | 0 | 5.1416122 | 11.63167952 | CRCP;POLF |
| 0 | 0 | 1.522878841 | 3.433767493 | KLF11;CAM |
| 0 | 0 | 1.404798978 | 3.145132959 | PI4K2B;BCA |
| 0 | 0 | 1.433564188 | 3.202836877 | MCUR1;MC |
| 0 | 0 | 2.01317063 | 4.482312981 | ODAD3;STI |
| 0 | 0 | 2.01317063 | 4.482312981 | ZC3H7B;AC |
| 0 | 0 | 2.01317063 | 4.482312981 | HPF1;PARF |
| 0 | 0 | 2.01317063 | 4.482312981 | SYNGR1;DI |
| 0 | 0 | 1.570507733 | 3.472423557 | EPM2A;TB |
| 0 | 0 | 1.862942328 | 4.077981281 | ZRANB3;M |
| 0 | 0 | 1.862942328 | 4.077981281 | WWTR1;AI |
| 0 | 0 | 1.862942328 | 4.077981281 | CD63;AHI1 |
| 0 | 0 | 1.862942328 | 4.077981281 | ATP6V1A;T |
| 0 | 0 | 1.862942328 | 4.077981281 | TFRC;HEPH |
| 0 | 0 | 1.501642576 | 3.277475697 | ABCA2;SLC |
| 0 | 0 | 1.642586683 | 3.580840789 | TOM1L2;C |
| 0 | 0 | 1.694503667 | 3.681491249 | CYP27A1;A |
| 0 | 0 | 2.143324607 | 4.617366025 | PLA2G15;G |
| 0 | 0 | 2.143324607 | 4.617366025 | PLA2G15;G |
| 0 | 0 | 2.143324607 | 4.617366025 | SLC45A3;P |
| 0 | 0 | 2.143324607 | 4.617366025 | ZWILCH;IN |
| 0 | 0 | 2.143324607 | 4.617366025 | MMP3;SIR |
| 0 | 0 | 2.143324607 | 4.617366025 | RAD50;RAI |
| 0 | 0 | 2.143324607 | 4.617366025 | CD63;AHI1 |
| 0 | 0 | 2.143324607 | 4.617366025 | ABCA2;ACA |
| 0 | 0 | 2.143324607 | 4.617366025 | PKIB;RTEL1 |
| 0 | 0 | 1.303679239 | 2.757895637 | ANP32A;M |
| 0 | 0 | 1.572688831 | 3.320220143 | SH3RF3;LR |
| 0 | 0 | 1.436905478 | 3.028811626 | GSKIP;ATP |
| 0 | 0 | 1.225600718 | 2.576241043 | CD63;STX1 |
| 0 | 0 | 1.929179398 | 4.050851137 | MCFD2;FU |

| | | | | |
|---|---|---|---|---|
| 0 | 0 | 2.373779731 | 4.983735379 | BACE1;APH |
| 0 | 0 | 2.373779731 | 4.983735379 | SLC45A3;S |
| 0 | 0 | 2.373779731 | 4.983735379 | PDCD4;PPA |
| 0 | 0 | 2.373779731 | 4.983735379 | PSMD10;P |
| 0 | 0 | 2.373779731 | 4.983735379 | PKIB;RTEL1 |
| 0 | 0 | 2.373779731 | 4.983735379 | TMEM120I |
| 0 | 0 | 2.373779731 | 4.983735379 | SFRP4;TWS |
| 0 | 0 | 2.373779731 | 4.983735379 | AGO4;DRC |
| 0 | 0 | 1.608275033 | 3.366085502 | NDUFA11;I |
| 0 | 0 | 1.608275033 | 3.366085502 | NDUFA11;I |
| 0 | 0 | 1.608275033 | 3.366085502 | MLLT11;RA |
| 0 | 0 | 2.892600262 | 6.04628545 | PPP3CA;PP |
| 0 | 0 | 2.892600262 | 6.04628545 | MMP3;MN |
| 0 | 0 | 2.892600262 | 6.04628545 | CBR1;PTGE |
| 0 | 0 | 2.892600262 | 6.04628545 | GGCT;MGS |
| 0 | 0 | 2.892600262 | 6.04628545 | PLA2G15;D |
| 0 | 0 | 2.892600262 | 6.04628545 | HGF;MST1 |
| 0 | 0 | 2.892600262 | 6.04628545 | ABHD10;PI |
| 0 | 0 | 2.892600262 | 6.04628545 | UNC119;PI |
| 0 | 0 | 2.892600262 | 6.04628545 | IMMP1L;IN |
| 0 | 0 | 2.892600262 | 6.04628545 | NUBPL;MY |
| 0 | 0 | 2.892600262 | 6.04628545 | PDE1B;PIR |
| 0 | 0 | 2.892600262 | 6.04628545 | P2RX7;UBA |
| 0 | 0 | 2.892600262 | 6.04628545 | C1QTNF1;C |
| 0 | 0 | 2.892600262 | 6.04628545 | CRTAP;P4H |
| 0 | 0 | 2.892600262 | 6.04628545 | PLEKHM1;S |
| 0 | 0 | 2.892600262 | 6.04628545 | PRKD3;PIC |
| 0 | 0 | 2.892600262 | 6.04628545 | TTLL4;CEP4 |
| 0 | 0 | 2.892600262 | 6.04628545 | SPHK2;ALC |
| 0 | 0 | 2.892600262 | 6.04628545 | SPHK2;CHE |
| 0 | 0 | 2.892600262 | 6.04628545 | KANK1;ARI |
| 0 | 0 | 2.892600262 | 6.04628545 | RBL2;RBL1 |
| 0 | 0 | 2.892600262 | 6.04628545 | SYNGR1;DI |
| 0 | 0 | 2.892600262 | 6.04628545 | CDH3;GIPC |
| 0 | 0 | 2.892600262 | 6.04628545 | VAT1;TFRC |
| 0 | 0 | 2.892600262 | 6.04628545 | PDK4;PDK2 |
| 0 | 0 | 2.892600262 | 6.04628545 | GNG2;TNF |
| 0 | 0 | 1.498209478 | 3.122341108 | CARMIL1;L |
| 0 | 0 | 1.654064685 | 3.439725529 | STK36;SUF |
| 0 | 0 | 1.800742358 | 3.741155342 | GPAM;SPH |
| 0 | 0 | 1.800742358 | 3.741155342 | LATS1;GPA |
| 0 | 0 | 1.800742358 | 3.741155342 | NDC1;XPO |
| 0 | 0 | 1.336157015 | 2.772175453 | PRKCG;HIP |
| 0 | 0 | 2.030403233 | 4.089972462 | DFFA;ACVF |
| 0 | 0 | 2.030403233 | 4.089972462 | STAR;FDX1 |
| 0 | 0 | 2.030403233 | 4.089972462 | GNG2;APLI |
| 0 | 0 | 2.030403233 | 4.089972462 | NTNG2;MF |

| | | | | |
|---|---|---|---|---|
| 0 | 0 | 2.030403233 | 4.089972462 | DDB1;KDM |
| 0 | 0 | 2.030403233 | 4.089972462 | FECH;COX1 |
| 0 | 0 | 2.030403233 | 4.089972462 | P2RX7;ENT |
| 0 | 0 | 2.030403233 | 4.089972462 | MSRA;MTH |
| 0 | 0 | 1.476093283 | 2.972184204 | ABCA2;SPH |
| 0 | 0 | 1.430032136 | 2.876627689 | ATP6V1A;S |
| 0 | 0 | 1.575363858 | 3.165294283 | MDC1;RIN |
| 0 | 0 | 1.615506586 | 3.215445818 | CLIP3;LGAI |
| 0 | 0 | 1.851907464 | 3.668114971 | CCDC88A;F |
| 0 | 0 | 1.851907464 | 3.668114971 | IDUA;GLB1 |
| 0 | 0 | 1.851907464 | 3.668114971 | PSMD10;R |
| 0 | 0 | 1.851907464 | 3.668114971 | MCFD2;FU |
| 0 | 0 | 1.851907464 | 3.668114971 | UGDH;CHS |
| 0 | 0 | 1.851907464 | 3.668114971 | LATS1;SFRI |
| 0 | 0 | 1.851907464 | 3.668114971 | STIL;MTHF |
| 0 | 0 | 1.441331581 | 2.851206773 | FBXW4;PSI |
| 0 | 0 | 1.742555289 | 3.436179506 | SHTN1;GO |
| 0 | 0 | 1.381975993 | 2.710624215 | EPM2A;AT |
| 0 | 0 | 1.516349519 | 2.964834181 | HIPK4;SBK |
| 0 | 0 | 1.454618138 | 2.829507992 | PSMD10;B |
| 0 | 0 | 2.204099433 | 4.272053809 | GIPC1;ARL |
| 0 | 0 | 2.204099433 | 4.272053809 | BLM;RAD5 |
| 0 | 0 | 2.204099433 | 4.272053809 | BBS2;MYO |
| 0 | 0 | 2.204099433 | 4.272053809 | KIF18A;KIF |
| 0 | 0 | 2.204099433 | 4.272053809 | GSTP1;PEL |
| 0 | 0 | 2.204099433 | 4.272053809 | RRM1;MTH |
| 0 | 0 | 2.204099433 | 4.272053809 | BBS2;MYO |
| 0 | 0 | 2.204099433 | 4.272053809 | SLC45A3;A |
| 0 | 0 | 2.204099433 | 4.272053809 | NEDD1;STI |
| 0 | 0 | 2.204099433 | 4.272053809 | C1QTNF1;C |
| 0 | 0 | 2.204099433 | 4.272053809 | AKT3;PPAF |
| 0 | 0 | 2.204099433 | 4.272053809 | SLC9A9;SL |
| 0 | 0 | 3.855991285 | 7.456679646 | SLC26A1;P |
| 0 | 0 | 3.855991285 | 7.456679646 | CASP9;BOK |
| 0 | 0 | 3.855991285 | 7.456679646 | SLC22A5;S |
| 0 | 0 | 3.855991285 | 7.456679646 | AKR1B1;RC |
| 0 | 0 | 3.855991285 | 7.456679646 | DHRS7B;PE |
| 0 | 0 | 3.855991285 | 7.456679646 | STARD5;CD |
| 0 | 0 | 3.855991285 | 7.456679646 | CRTAM;CC |
| 0 | 0 | 3.855991285 | 7.456679646 | UGDH;ME( |
| 0 | 0 | 3.855991285 | 7.456679646 | DHRS7B;PE |
| 0 | 0 | 3.855991285 | 7.456679646 | DUOX2;SO |
| 0 | 0 | 3.855991285 | 7.456679646 | TRIM37;HD |
| 0 | 0 | 3.855991285 | 7.456679646 | HPS4;RAB3 |
| 0 | 0 | 3.855991285 | 7.456679646 | ATP23;TMI |
| 0 | 0 | 3.855991285 | 7.456679646 | POLA1;GIN |
| 0 | 0 | 3.855991285 | 7.456679646 | VGLL4;TP7 |

| | | | | |
|---|---|---|---|---|
| 0 | 0 | 3.855991285 | 7.456679646 | E2F1;TREM… |
| 0 | 0 | 3.855991285 | 7.456679646 | CLOCK;ARN… |
| 0 | 0 | 3.855991285 | 7.456679646 | DNMT1;DN… |
| 0 | 0 | 3.855991285 | 7.456679646 | RACGAP1;I… |
| 0 | 0 | 3.855991285 | 7.456679646 | POLA1;GIN… |
| 0 | 0 | 3.855991285 | 7.456679646 | KIF3A;KIFA… |
| 0 | 0 | 3.855991285 | 7.456679646 | SCAP;LDLR… |
| 0 | 0 | 3.855991285 | 7.456679646 | P2RX7;WN… |
| 0 | 0 | 3.855991285 | 7.456679646 | TREM2;LDI… |
| 0 | 0 | 3.855991285 | 7.456679646 | STAP1;TRE… |
| 0 | 0 | 3.855991285 | 7.456679646 | CDC7;DBF4… |
| 0 | 0 | 3.855991285 | 7.456679646 | CD86;TNFS… |
| 0 | 0 | 3.855991285 | 7.456679646 | UBE2D4;UI… |
| 0 | 0 | 3.855991285 | 7.456679646 | UBE2D4;UI… |
| 0 | 0 | 3.855991285 | 7.456679646 | BBIP1;SPAT… |
| 0 | 0 | 3.855991285 | 7.456679646 | MSRA;MSF… |
| 0 | 0 | 3.855991285 | 7.456679646 | QDPR;SPR… |
| 0 | 0 | 3.855991285 | 7.456679646 | SLC26A1;P… |
| 0 | 0 | 3.855991285 | 7.456679646 | ATL3;LNPK… |
| 0 | 0 | 3.855991285 | 7.456679646 | PDGFB;GA:… |
| 0 | 0 | 3.855991285 | 7.456679646 | LGALS3;HA… |
| 0 | 0 | 3.855991285 | 7.456679646 | STARD5;CD… |
| 0 | 0 | 3.855991285 | 7.456679646 | GSTZ1;FAH… |
| 0 | 0 | 3.855991285 | 7.456679646 | ALDH6A1;I… |
| 0 | 0 | 1.211685475 | 2.337400014 | GRN;ITSN1… |
| 0 | 0 | 1.388234019 | 2.672203573 | BCAS3;RAL… |
| 0 | 0 | 1.422286004 | 2.722117737 | NDUFA11;I… |
| 0 | 0 | 1.578701128 | 3.007182092 | TSPYL2;AC… |
| 0 | 0 | 1.349595505 | 2.547740147 | BLM;ATL3;… |
| 0 | 0 | 2.571054926 | 4.852948904 | PPP3CA;PF… |
| 0 | 0 | 2.571054926 | 4.852948904 | CDC7;MCN… |
| 0 | 0 | 2.571054926 | 4.852948904 | EPS8;SPAS… |
| 0 | 0 | 2.571054926 | 4.852948904 | P2RX3;PKD… |
| 0 | 0 | 2.571054926 | 4.852948904 | ARL6IP5;G… |
| 0 | 0 | 2.571054926 | 4.852948904 | DAG1;GCC… |
| 0 | 0 | 2.571054926 | 4.852948904 | GSTP1;DUS… |
| 0 | 0 | 2.571054926 | 4.852948904 | CCL19;GAS… |
| 0 | 0 | 2.571054926 | 4.852948904 | DLG4;WDF… |
| 0 | 0 | 2.571054926 | 4.852948904 | GSTO1;CLI… |
| 0 | 0 | 2.571054926 | 4.852948904 | GSTO1;CLI… |
| 0 | 0 | 2.571054926 | 4.852948904 | FUT8;GALT… |
| 0 | 0 | 2.571054926 | 4.852948904 | CARMIL1;C… |
| 0 | 0 | 2.571054926 | 4.852948904 | WNK1;WN… |
| 0 | 0 | 2.571054926 | 4.852948904 | PEX6;PEX1… |
| 0 | 0 | 2.571054926 | 4.852948904 | ZWILCH;ZV… |
| 0 | 0 | 2.571054926 | 4.852948904 | DCTD;THTF… |
| 0 | 0 | 2.571054926 | 4.852948904 | MED25;ZN… |

| | | | | |
|---|---|---|---|---|
| 0 | 0 | 2.571054926 | 4.852948904 | LGALS3;LG |
| 0 | 0 | 2.571054926 | 4.852948904 | BLM;GMN |
| 0 | 0 | 2.571054926 | 4.852948904 | LANCL1;M |
| 0 | 0 | 2.571054926 | 4.852948904 | APOA4;AB |
| 0 | 0 | 2.571054926 | 4.852948904 | CHEK2;CH |
| 0 | 0 | 1.624703962 | 3.062744905 | RTEL1;MR |
| 0 | 0 | 1.928773997 | 3.634690646 | TPST1;SLC |
| 0 | 0 | 1.928773997 | 3.634690646 | ALDH3A2; |
| 0 | 0 | 1.928773997 | 3.634690646 | ABCA2;AR |
| 0 | 0 | 1.433756567 | 2.693899442 | ACOT8;GS |
| 0 | 0 | 1.333973182 | 2.499260521 | MYBPC3;C |
| 0 | 0 | 1.688004913 | 3.15946878 | ABCA2;ED |
| 0 | 0 | 1.688004913 | 3.15946878 | PPIP5K1;G |
| 0 | 0 | 1.780579525 | 3.327471093 | CLIP3;TBC |
| 0 | 0 | 1.780579525 | 3.327471093 | PRDX2;PR |
| 0 | 0 | 1.372731258 | 2.562931086 | CPM;ATP2 |
| 0 | 0 | 1.372731258 | 2.562931086 | PLK4;CUL7 |
| 0 | 0 | 1.513413411 | 2.801520373 | RBL1;UBA |
| 0 | 0 | 1.513413411 | 2.801520373 | MSH6;ZRA |
| 0 | 0 | 1.543531469 | 2.813611189 | MAP10;CL |
| 0 | 0 | 1.543531469 | 2.813611189 | PRKCG;US |
| 0 | 0 | 1.263245349 | 2.301171953 | DCTN5;MC |
| 0 | 0 | 1.463895677 | 2.649614716 | ACHE;BCA |
| 0 | 0 | 1.123026681 | 2.02115481 | TFRC;CITE |
| 0 | 0 | 1.582955271 | 2.845289397 | MINPP1;P |
| 0 | 0 | 2.057043175 | 3.684896299 | BBS2;MYO |
| 0 | 0 | 2.057043175 | 3.684896299 | BRAT1;AT |
| 0 | 0 | 2.057043175 | 3.684896299 | FUT8;MGA |
| 0 | 0 | 2.057043175 | 3.684896299 | LATS1;MD |
| 0 | 0 | 2.057043175 | 3.684896299 | NUP35;CB |
| 0 | 0 | 2.057043175 | 3.684896299 | NDC1;P2R |
| 0 | 0 | 2.057043175 | 3.684896299 | SLC27A1;A |
| 0 | 0 | 2.057043175 | 3.684896299 | MMP3;TR |
| 0 | 0 | 2.057043175 | 3.684896299 | P2RX7;P2R |
| 0 | 0 | 1.323738482 | 2.360161451 | POGLUT1; |
| 0 | 0 | 1.636760619 | 2.907920263 | LIPT1;CYB |
| 0 | 0 | 1.362427179 | 2.413574276 | SLC27A1;S |
| 0 | 0 | 1.836823735 | 3.23954525 | ACER2;RA |
| 0 | 0 | 1.836823735 | 3.23954525 | LATS1;MO |
| 0 | 0 | 1.836823735 | 3.23954525 | MTHFD1;S |
| 0 | 0 | 1.714535137 | 3.023366414 | ITGB3;TRE |
| 0 | 0 | 1.714535137 | 3.023366414 | GGCT;AIF |
| 0 | 0 | 1.714535137 | 3.023366414 | GRN;ACP4 |
| 0 | 0 | 1.714535137 | 3.023366414 | RINT1;BL |
| 0 | 0 | 1.714535137 | 3.023366414 | ACHE;SLC2 |
| 0 | 0 | 1.714535137 | 3.023366414 | P2RX7;AC |
| 0 | 0 | 1.714535137 | 3.023366414 | ALDH3A2; |

| | | | | |
|---|---|---|---|---|
| 0 | 0 | 1.714535137 | 3.023366414 | ABCA2;EEF |
| 0 | 0 | 1.509890924 | 2.633539569 | FUT8;MGA |
| 0 | 0 | 1.543294015 | 2.6448335 | GRAMD1B |
| 0 | 0 | 1.543294015 | 2.6448335 | MINPP1;PI |
| 0 | 0 | 1.543294015 | 2.6448335 | NTNG2;PA |
| 0 | 0 | 1.403381739 | 2.404819077 | PSMD10;L( |
| 0 | 0 | 2.313818657 | 3.955350344 | NUBPL;MY |
| 0 | 0 | 2.313818657 | 3.955350344 | PICK1;TREI |
| 0 | 0 | 2.313818657 | 3.955350344 | LATS1;SUF |
| 0 | 0 | 2.313818657 | 3.955350344 | HACD1;HA |
| 0 | 0 | 2.313818657 | 3.955350344 | PPP1R3C;G |
| 0 | 0 | 2.313818657 | 3.955350344 | SLC27A1;S |
| 0 | 0 | 2.313818657 | 3.955350344 | PPP1R3C;G |
| 0 | 0 | 2.313818657 | 3.955350344 | ARV1;STAF |
| 0 | 0 | 2.313818657 | 3.955350344 | FER1L5;GR |
| 0 | 0 | 2.313818657 | 3.955350344 | HGF;PARK7 |
| 0 | 0 | 2.313818657 | 3.955350344 | ABCA5;ITG |
| 0 | 0 | 2.313818657 | 3.955350344 | SHTN1;SEN |
| 0 | 0 | 2.313818657 | 3.955350344 | DIP2A;RAP |
| 0 | 0 | 2.313818657 | 3.955350344 | EPS8;PLEKI |
| 0 | 0 | 2.313818657 | 3.955350344 | AKT3;HMG |
| 0 | 0 | 2.313818657 | 3.955350344 | KRT10;PKD |
| 0 | 0 | 2.313818657 | 3.955350344 | ALMS1;ASA |
| 0 | 0 | 2.313818657 | 3.955350344 | GEN1;XRC( |
| 0 | 0 | 2.313818657 | 3.955350344 | PARK7;RAF |
| 0 | 0 | 2.313818657 | 3.955350344 | MMP3;TXN |
| 0 | 0 | 2.313818657 | 3.955350344 | ACER2;ABC |
| 0 | 0 | 2.313818657 | 3.955350344 | ACER2;ABC |
| 0 | 0 | 2.313818657 | 3.955350344 | HACD1;HA |
| 0 | 0 | 1.267895686 | 2.164552047 | RFC5;BLM; |
| 0 | 0 | 1.456138469 | 2.482219808 | LATS1;NAS |
| 0 | 0 | 1.456138469 | 2.482219808 | RTEL1;WRI |
| 0 | 0 | 1.374601156 | 2.32573708 | GPI;BCAS3 |
| 0 | 0 | 1.588530696 | 2.678837249 | SLC25A1;G |
| 0 | 0 | 1.588530696 | 2.678837249 | RRAGB;RRA |
| 0 | 0 | 1.588530696 | 2.678837249 | PSMD10;M |
| 0 | 0 | 1.414938758 | 2.369488523 | HACD1;AB( |
| 0 | 0 | 1.414938758 | 2.369488523 | FARP2;PAF |
| 0 | 0 | 3.084618736 | 5.161868477 | PDXP;HOG |
| 0 | 0 | 3.084618736 | 5.161868477 | CROT;CPT1 |
| 0 | 0 | 3.084618736 | 5.161868477 | STX17;ATG |
| 0 | 0 | 3.084618736 | 5.161868477 | P2RX7;PM |
| 0 | 0 | 3.084618736 | 5.161868477 | CAB39;SLC |
| 0 | 0 | 3.084618736 | 5.161868477 | PM20D1;T. |
| 0 | 0 | 3.084618736 | 5.161868477 | ALOX5;PD( |
| 0 | 0 | 3.084618736 | 5.161868477 | GNG2;TNF |
| 0 | 0 | 3.084618736 | 5.161868477 | ABCC5;SLC |

| | | | | |
|---|---|---|---|---|
| 0 | 0 | 3.084618736 | 5.161868477 | FLAD1;ACF |
| 0 | 0 | 3.084618736 | 5.161868477 | BLM;WRN |
| 0 | 0 | 3.084618736 | 5.161868477 | MYO1E;FO |
| 0 | 0 | 3.084618736 | 5.161868477 | GALE;GALT |
| 0 | 0 | 3.084618736 | 5.161868477 | ALOX5;PD( |
| 0 | 0 | 3.084618736 | 5.161868477 | ADI1;MRI1 |
| 0 | 0 | 3.084618736 | 5.161868477 | SPAST;COF |
| 0 | 0 | 3.084618736 | 5.161868477 | MGME1;P/ |
| 0 | 0 | 3.084618736 | 5.161868477 | CCL19;GAS |
| 0 | 0 | 3.084618736 | 5.161868477 | ABCA2;ITG |
| 0 | 0 | 3.084618736 | 5.161868477 | QPRT;NMN |
| 0 | 0 | 3.084618736 | 5.161868477 | APH1B;PSE |
| 0 | 0 | 3.084618736 | 5.161868477 | DCXR;XYLB |
| 0 | 0 | 3.084618736 | 5.161868477 | CD209;AB( |
| 0 | 0 | 3.084618736 | 5.161868477 | AGTPBP1;/ |
| 0 | 0 | 3.084618736 | 5.161868477 | MSH6;POT |
| 0 | 0 | 3.084618736 | 5.161868477 | PRKCA;SAS |
| 0 | 0 | 3.084618736 | 5.161868477 | TOMM7;H |
| 0 | 0 | 3.084618736 | 5.161868477 | GEN1;XRC( |
| 0 | 0 | 3.084618736 | 5.161868477 | MMP3;SOI |
| 0 | 0 | 3.084618736 | 5.161868477 | GEN1;XRC( |
| 0 | 0 | 3.084618736 | 5.161868477 | GSTO1;ATF |
| 0 | 0 | 3.084618736 | 5.161868477 | DCLRE1B;D |
| 0 | 0 | 3.084618736 | 5.161868477 | ATP23;TMI |
| 0 | 0 | 3.084618736 | 5.161868477 | CMPK1;DH |
| 0 | 0 | 3.084618736 | 5.161868477 | TPK1;DHOI |
| 0 | 0 | 3.084618736 | 5.161868477 | DDB1;HAC |
| 0 | 0 | 3.084618736 | 5.161868477 | E2F1;TREN |
| 0 | 0 | 3.084618736 | 5.161868477 | PRKCA;TRE |
| 0 | 0 | 3.084618736 | 5.161868477 | CLOCK;ARI |
| 0 | 0 | 3.084618736 | 5.161868477 | SVBP;TIMP |
| 0 | 0 | 3.084618736 | 5.161868477 | PPP2CB;G/ |
| 0 | 0 | 3.084618736 | 5.161868477 | STPG1;CAN |
| 0 | 0 | 3.084618736 | 5.161868477 | TREM2;PA |
| 0 | 0 | 3.084618736 | 5.161868477 | TNFSF4;CC |
| 0 | 0 | 3.084618736 | 5.161868477 | FLAD1;ACF |
| 0 | 0 | 3.084618736 | 5.161868477 | SFXN3;SFX |
| 0 | 0 | 3.084618736 | 5.161868477 | THTPA;TPK |
| 0 | 0 | 1.653208206 | 2.751122903 | PLA2G15;S |
| 0 | 0 | 1.653208206 | 2.751122903 | PPP3CC;YV |
| 0 | 0 | 1.653208206 | 2.751122903 | NDC1;XPO |
| 0 | 0 | 1.653208206 | 2.751122903 | NDC1;XPO |
| 0 | 0 | 1.928368949 | 3.195652057 | PPP1R3C;G |
| 0 | 0 | 1.928368949 | 3.195652057 | AGTPBP1;( |
| 0 | 0 | 1.928368949 | 3.195652057 | TUT4;DRO: |
| 0 | 0 | 1.928368949 | 3.195652057 | PSMD10;P |
| 0 | 0 | 1.928368949 | 3.195652057 | CCDC78;M |

| | | | | |
|---|---|---|---|---|
| 0 | 0 | 1.183955933 | 1.961752412 | ITSN1;PSEI… |
| 0 | 0 | 1.753232588 | 2.89493516 | DLG4;PICK… |
| 0 | 0 | 1.753232588 | 2.89493516 | GSTO1;GN… |
| 0 | 0 | 1.753232588 | 2.89493516 | CLIP3;KANI… |
| 0 | 0 | 1.753232588 | 2.89493516 | SETD4;SUV… |
| 0 | 0 | 1.753232588 | 2.89493516 | CCDC57;PF… |
| 0 | 0 | 1.429091969 | 2.337300128 | PSMD10;P… |
| 0 | 0 | 1.429091969 | 2.337300128 | DDB1;LRSA… |
| 0 | 0 | 1.429091969 | 2.337300128 | TCHP;CCD… |
| 0 | 0 | 1.505567454 | 2.459837255 | NDST1;UG… |
| 0 | 0 | 1.505567454 | 2.459837255 | NEU3;FUT… |
| 0 | 0 | 1.505567454 | 2.459837255 | TRPM2;IM… |
| 0 | 0 | 1.391664157 | 2.241206816 | CLIP3;KANI… |
| 0 | 0 | 1.391664157 | 2.241206816 | BLM;MRNI… |
| 0 | 0 | 1.446814904 | 2.309680641 | SLC27A1;S… |
| 0 | 0 | 1.140400371 | 1.802081357 | ABCD4;PI4… |
| 0 | 0 | 1.596110718 | 2.506776691 | ATP6V1A;… |
| 0 | 0 | 1.596110718 | 2.506776691 | RBL2;RBL1… |
| 0 | 0 | 1.596110718 | 2.506776691 | CD86;SASH… |
| 0 | 0 | 1.596110718 | 2.506776691 | CD19;PDGI… |
| 0 | 0 | 1.403028978 | 2.201291152 | CYP27A1;A… |
| 0 | 0 | 1.317767593 | 2.062706948 | MRPS22;M… |
| 0 | 0 | 1.300899073 | 2.025078354 | EEFSEC;MF… |
| 0 | 0 | 2.103352619 | 3.264146795 | PHYH;IDH1… |
| 0 | 0 | 2.103352619 | 3.264146795 | BACE1;APH… |
| 0 | 0 | 2.103352619 | 3.264146795 | CD9;PPAR… |
| 0 | 0 | 2.103352619 | 3.264146795 | CROT;PPCI… |
| 0 | 0 | 2.103352619 | 3.264146795 | SCAP;SEC2… |
| 0 | 0 | 2.103352619 | 3.264146795 | PARP2;HLC… |
| 0 | 0 | 2.103352619 | 3.264146795 | PSMD10;S… |
| 0 | 0 | 2.103352619 | 3.264146795 | PSMD10;T… |
| 0 | 0 | 2.103352619 | 3.264146795 | GRN;TREM… |
| 0 | 0 | 2.103352619 | 3.264146795 | NFATC3;PI… |
| 0 | 0 | 2.103352619 | 3.264146795 | LAG3;TNFS… |
| 0 | 0 | 2.103352619 | 3.264146795 | ENTPD5;EI… |
| 0 | 0 | 2.103352619 | 3.264146795 | PPP2CB;PP… |
| 0 | 0 | 2.103352619 | 3.264146795 | GKAP1;ECH… |
| 0 | 0 | 2.103352619 | 3.264146795 | DDB1;SLC4… |
| 0 | 0 | 2.103352619 | 3.264146795 | ABCA2;IGF… |
| 0 | 0 | 2.103352619 | 3.264146795 | DAB2;RBPI… |
| 0 | 0 | 2.103352619 | 3.264146795 | CD86;TNFS… |
| 0 | 0 | 2.103352619 | 3.264146795 | CD86;DENI… |
| 0 | 0 | 2.103352619 | 3.264146795 | ZWILCH;ZV… |
| 0 | 0 | 2.103352619 | 3.264146795 | PUDP;TK1;… |
| 0 | 0 | 2.103352619 | 3.264146795 | NTNG2;TRI… |
| 0 | 0 | 2.103352619 | 3.264146795 | CD63;LOXL… |
| 0 | 0 | 2.103352619 | 3.264146795 | WFS1;ALO… |

| | | | | |
|---|---|---|---|---|
| 0 | 0 | 2.103352619 | 3.264146795 | MMP3;MN |
| 0 | 0 | 2.103352619 | 3.264146795 | SPP1;HSD1 |
| 0 | 0 | 2.103352619 | 3.264146795 | SLC26A1;R |
| 0 | 0 | 1.28649715 | 1.994307691 | PI4K2B;MT |
| 0 | 0 | 1.369140349 | 2.12012538 | ALDH3A2;S |
| 0 | 0 | 1.676910236 | 2.593087301 | MCUR1;M( |
| 0 | 0 | 1.676910236 | 2.593087301 | ARL6IP5;SL |
| 0 | 0 | 1.676910236 | 2.593087301 | ATP6V1A;A |
| 0 | 0 | 1.676910236 | 2.593087301 | CD19;PDGI |
| 0 | 0 | 1.676910236 | 2.593087301 | ATP2B4;RA |
| 0 | 0 | 1.676910236 | 2.593087301 | SLC27A1;S |
| 0 | 0 | 1.814832867 | 2.784461865 | TRPM2;AT |
| 0 | 0 | 1.814832867 | 2.784461865 | H2AZ1;FAN |
| 0 | 0 | 1.814832867 | 2.784461865 | CASP9;CAS |
| 0 | 0 | 1.814832867 | 2.784461865 | RPS27L;CD |
| 0 | 0 | 1.814832867 | 2.784461865 | BBS2;MYO |
| 0 | 0 | 1.814832867 | 2.784461865 | P2RX7;ENT |
| 0 | 0 | 1.814832867 | 2.784461865 | P2RX7;ENT |
| 0 | 0 | 1.814832867 | 2.784461865 | STK36;ARN |
| 0 | 0 | 1.342136809 | 2.05768045 | CASP9;ACE |
| 0 | 0 | 1.342136809 | 2.05768045 | WWTR1;IN |
| 0 | 0 | 1.342136809 | 2.05768045 | CD86;SETD |
| 0 | 0 | 1.320115909 | 2.008655202 | MRPS22;M |
| 0 | 0 | 1.50010917 | 2.278949349 | IMPACT;TE |
| 0 | 0 | 1.50010917 | 2.278949349 | CSTB;CDK5 |
| 0 | 0 | 1.22820139 | 1.839321896 | PPM1L;PD |
| 0 | 0 | 1.347331584 | 2.005798351 | GLP1R;TFR |
| 0 | 0 | 1.435378504 | 2.130869077 | NDC1;AHC |
| 0 | 0 | 1.435378504 | 2.130869077 | NDC1;AHC |
| 0 | 0 | 1.542819729 | 2.286950866 | RPS27L;TM |
| 0 | 0 | 1.542819729 | 2.286950866 | PKIB;RTEL1 |
| 0 | 0 | 1.542819729 | 2.286950866 | MDFIC;RAI |
| 0 | 0 | 1.542819729 | 2.286950866 | PPP3CA;GN |
| 0 | 0 | 1.286246167 | 1.880625622 | DCTN5;LA( |
| 0 | 0 | 2.57037037 | 3.755166937 | MTHFD1;P |
| 0 | 0 | 2.57037037 | 3.755166937 | KYAT1;ACA |
| 0 | 0 | 2.57037037 | 3.755166937 | DPY30;SIR |
| 0 | 0 | 2.57037037 | 3.755166937 | PICK1;TREI |
| 0 | 0 | 2.57037037 | 3.755166937 | WFS1;ATG |
| 0 | 0 | 2.57037037 | 3.755166937 | HYI;HOGA |
| 0 | 0 | 2.57037037 | 3.755166937 | NDST1;HS2 |
| 0 | 0 | 2.57037037 | 3.755166937 | PHYH;PDSS |
| 0 | 0 | 2.57037037 | 3.755166937 | ABHD10;PI |
| 0 | 0 | 2.57037037 | 3.755166937 | PARP1;ST3 |
| 0 | 0 | 2.57037037 | 3.755166937 | MAP10;RA |
| 0 | 0 | 2.57037037 | 3.755166937 | MAP10;RA |
| 0 | 0 | 2.57037037 | 3.755166937 | PLK4;CCDC |

| | | | | |
|---|---|---|---|---|
| 0 | 0 | 2.57037037 | 3.755166937 | PICK1;ARP[illegible] |
| 0 | 0 | 2.57037037 | 3.755166937 | BBS2;NENI[illegible] |
| 0 | 0 | 2.57037037 | 3.755166937 | PKIB;PKIG |
| 0 | 0 | 2.57037037 | 3.755166937 | VAT1;TFRC |
| 0 | 0 | 2.57037037 | 3.755166937 | TOM1L2;C |
| 0 | 0 | 2.57037037 | 3.755166937 | ATP2B4;GL |
| 0 | 0 | 2.57037037 | 3.755166937 | KDM2B;SIF |
| 0 | 0 | 2.57037037 | 3.755166937 | PARK7;TXN |
| 0 | 0 | 2.57037037 | 3.755166937 | PLOD3;PLC |
| 0 | 0 | 2.57037037 | 3.755166937 | P4HA2;P4H |
| 0 | 0 | 2.57037037 | 3.755166937 | SASH3;EIF2 |
| 0 | 0 | 2.57037037 | 3.755166937 | SIRT2;BOK |
| 0 | 0 | 2.57037037 | 3.755166937 | CDH3;IGF1 |
| 0 | 0 | 2.57037037 | 3.755166937 | CDH3;GIPC |
| 0 | 0 | 2.57037037 | 3.755166937 | STAP1;TRE |
| 0 | 0 | 2.57037037 | 3.755166937 | DLG4;MAP |
| 0 | 0 | 2.57037037 | 3.755166937 | CDH3;GIPC |
| 0 | 0 | 2.57037037 | 3.755166937 | AGBL5;AGT |
| 0 | 0 | 2.57037037 | 3.755166937 | ABHD10;PI |
| 0 | 0 | 2.57037037 | 3.755166937 | ALDH6A1;[illegible] |
| 0 | 0 | 2.57037037 | 3.755166937 | ADTRP;IER |
| 0 | 0 | 2.57037037 | 3.755166937 | LANCL1;TR |
| 0 | 0 | 2.57037037 | 3.755166937 | HPF1;XRCC |
| 0 | 0 | 2.57037037 | 3.755166937 | ABCA2;FKT |
| 0 | 0 | 2.57037037 | 3.755166937 | CD86;TNFS |
| 0 | 0 | 2.57037037 | 3.755166937 | XRCC1;APC |
| 0 | 0 | 2.57037037 | 3.755166937 | MTIF2;MRI |
| 0 | 0 | 2.57037037 | 3.755166937 | TDP1;ERCC |
| 0 | 0 | 2.57037037 | 3.755166937 | PPP3CA;LA |
| 0 | 0 | 2.57037037 | 3.755166937 | HDAC6;SRI |
| 0 | 0 | 2.57037037 | 3.755166937 | PLA2R1;AR |
| 0 | 0 | 2.57037037 | 3.755166937 | CADM1;CR |
| 0 | 0 | 2.57037037 | 3.755166937 | TBCEL;TBC |
| 0 | 0 | 2.57037037 | 3.755166937 | CBR1;VKOI |
| 0 | 0 | 1.272163419 | 1.854730162 | PARP1;CET |
| 0 | 0 | 1.60694808 | 2.327652553 | GPI;PFKL;A |
| 0 | 0 | 1.60694808 | 2.327652553 | GPR137;TC |
| 0 | 0 | 1.60694808 | 2.327652553 | ANAPC13;[illegible] |
| 0 | 0 | 1.60694808 | 2.327652553 | RTEL1;RAD |
| 0 | 0 | 1.136049002 | 1.638216679 | MAP10;TN |
| 0 | 0 | 1.459483064 | 2.104274245 | GNG2;KCN |
| 0 | 0 | 1.459483064 | 2.104274245 | CDK5;MMI |
| 0 | 0 | 1.713911906 | 2.436248367 | CAB39;WN |
| 0 | 0 | 1.713911906 | 2.436248367 | ERI1;ERI2;[illegible] |
| 0 | 0 | 1.713911906 | 2.436248367 | MCUR1;M[illegible] |
| 0 | 0 | 1.713911906 | 2.436248367 | GSTO1;ATF |
| 0 | 0 | 1.713911906 | 2.436248367 | TWIST1;AP |

| | | | | |
|---|---|---|---|---|
| 0 | 0 | 1.713911906 | 2.436248367 | ALDH3A2;A |
| 0 | 0 | 1.713911906 | 2.436248367 | DFFA;CIDE |
| 0 | 0 | 1.402676834 | 1.984563113 | SGPL1;PKN |
| 0 | 0 | 1.402676834 | 1.984563113 | PRKCG;CD8 |
| 0 | 0 | 1.402676834 | 1.984563113 | IMPACT;TH |
| 0 | 0 | 1.927964255 | 2.721575231 | AGL;PHKB; |
| 0 | 0 | 1.927964255 | 2.721575231 | NAGK;PGM |
| 0 | 0 | 1.927964255 | 2.721575231 | AGL;PHKB; |
| 0 | 0 | 1.927964255 | 2.721575231 | STAR;NPC2 |
| 0 | 0 | 1.927964255 | 2.721575231 | FASTKD1;N |
| 0 | 0 | 1.927964255 | 2.721575231 | NUCKS1;EI |
| 0 | 0 | 1.927964255 | 2.721575231 | CD84;GRN |
| 0 | 0 | 1.927964255 | 2.721575231 | SLC27A1;P |
| 0 | 0 | 1.927964255 | 2.721575231 | AHI1;ARL3 |
| 0 | 0 | 1.927964255 | 2.721575231 | CUL7;PPAR |
| 0 | 0 | 1.927964255 | 2.721575231 | FEN1;RTEL |
| 0 | 0 | 1.927964255 | 2.721575231 | DNMT1;AU |
| 0 | 0 | 1.927964255 | 2.721575231 | GAS6;GTSE |
| 0 | 0 | 1.927964255 | 2.721575231 | PRXL2B;MI |
| 0 | 0 | 1.927964255 | 2.721575231 | ASPH;PLOD |
| 0 | 0 | 1.927964255 | 2.721575231 | CD19;CRTA |
| 0 | 0 | 1.927964255 | 2.721575231 | GRN;STAP1 |
| 0 | 0 | 1.927964255 | 2.721575231 | NTNG2;DL |
| 0 | 0 | 1.927964255 | 2.721575231 | POP7;LACT |
| 0 | 0 | 1.927964255 | 2.721575231 | SYCE2;STA |
| 0 | 0 | 1.927964255 | 2.721575231 | SYCE2;STA |
| 0 | 0 | 1.927964255 | 2.721575231 | TSEN15;FA |
| 0 | 0 | 1.492966869 | 2.088754061 | OAT;ALDH |
| 0 | 0 | 1.492966869 | 2.088754061 | RBL2;RBL1 |
| 0 | 0 | 1.492966869 | 2.088754061 | ATP6V1A;A |
| 0 | 0 | 1.206013944 | 1.659695057 | BCAS3;RIP |
| 0 | 0 | 1.112337759 | 1.528020063 | PHF20;ITSN |
| 0 | 0 | 1.285995624 | 1.76149353 | ABCA2;SLC |
| 0 | 0 | 1.420995174 | 1.944246136 | SLC38A7;S |
| 0 | 0 | 1.420995174 | 1.944246136 | BLM;RAD5 |
| 0 | 0 | 1.420995174 | 1.944246136 | RINT1;BLM |
| 0 | 0 | 1.269768782 | 1.735052116 | SLF1;TFRC; |
| 0 | 0 | 1.234756686 | 1.685999578 | CHRNB1;SI |
| 0 | 0 | 1.256190855 | 1.714498648 | DCTN5;LAC |
| 0 | 0 | 1.542582897 | 2.093402779 | SLC25A1;G |
| 0 | 0 | 1.542582897 | 2.093402779 | GSTM1;GS |
| 0 | 0 | 1.542582897 | 2.093402779 | DNMT1;EE |
| 0 | 0 | 1.542582897 | 2.093402779 | CD19;PDGI |
| 0 | 0 | 1.542582897 | 2.093402779 | STIL;MTHF |
| 0 | 0 | 1.542582897 | 2.093402779 | MMP14;CD |
| 0 | 0 | 1.542582897 | 2.093402779 | RTEL1;RAD |
| 0 | 0 | 1.214729532 | 1.610029264 | GLP1R;ACH |

| | | | | |
|---|---|---|---|---|
| 0 | 0 | 1.285870516 | 1.69934976 | SETD4;FZD |
| 0 | 0 | 1.285870516 | 1.69934976 | ODAD3;TB |
| 0 | 0 | 1.446229812 | 1.909699015 | GRN;ACP4; |
| 0 | 0 | 1.446229812 | 1.909699015 | NDC1;XPO |
| 0 | 0 | 1.241681393 | 1.636952763 | ATP2B4;CII |
| 0 | 0 | 1.623614203 | 2.13939009 | DFFA;DNA |
| 0 | 0 | 1.623614203 | 2.13939009 | DNMT1;DN |
| 0 | 0 | 1.623614203 | 2.13939009 | BACE1;SPP |
| 0 | 0 | 1.623614203 | 2.13939009 | MDFIC;RAI |
| 0 | 0 | 1.623614203 | 2.13939009 | CLIP3;ZDHI |
| 0 | 0 | 1.623614203 | 2.13939009 | CLIP3;ZDHI |
| 0 | 0 | 1.623614203 | 2.13939009 | METTL22;E |
| 0 | 0 | 1.623614203 | 2.13939009 | CD86;TNFS |
| 0 | 0 | 1.623614203 | 2.13939009 | PARK7;GAS |
| 0 | 0 | 1.623614203 | 2.13939009 | WWTR1;D/ |
| 0 | 0 | 1.18303978 | 1.545184109 | ATP6V1A;F |
| 0 | 0 | 1.192102615 | 1.543576922 | BBS2;BBS1 |
| 0 | 0 | 1.13063198 | 1.454564957 | ATP6V1A;S |
| 0 | 0 | 1.779558715 | 2.288838859 | AGL;PHKB; |
| 0 | 0 | 1.779558715 | 2.288838859 | TRPM2;HN |
| 0 | 0 | 1.779558715 | 2.288838859 | LGALS3;GR |
| 0 | 0 | 1.779558715 | 2.288838859 | CYP27A1;P |
| 0 | 0 | 1.779558715 | 2.288838859 | TFRC;SHLD |
| 0 | 0 | 1.779558715 | 2.288838859 | SLC45A3;P |
| 0 | 0 | 1.779558715 | 2.288838859 | NEDD1;STI |
| 0 | 0 | 1.779558715 | 2.288838859 | PARP1;SPH |
| 0 | 0 | 1.779558715 | 2.288838859 | MSR1;TREI |
| 0 | 0 | 1.779558715 | 2.288838859 | MVB12A;V |
| 0 | 0 | 1.779558715 | 2.288838859 | P2RX7;RFC |
| 0 | 0 | 1.779558715 | 2.288838859 | TSEN15;FA |
| 0 | 0 | 1.779558715 | 2.288838859 | MTHFD1;S |
| 0 | 0 | 1.779558715 | 2.288838859 | CADM1;CR |
| 0 | 0 | 1.341538929 | 1.723332762 | NDC1;AHC |
| 0 | 0 | 1.341538929 | 1.723332762 | ACOT8;GST |
| 0 | 0 | 2.203050109 | 2.8285501 | MTHFD1;IL |
| 0 | 0 | 2.203050109 | 2.8285501 | ACP5;TPP1 |
| 0 | 0 | 2.203050109 | 2.8285501 | TEKT3;GAS |
| 0 | 0 | 2.203050109 | 2.8285501 | CD63;RAB3 |
| 0 | 0 | 2.203050109 | 2.8285501 | CD63;RAB3 |
| 0 | 0 | 2.203050109 | 2.8285501 | MYO1E;XP |
| 0 | 0 | 2.203050109 | 2.8285501 | GNPDA1;G |
| 0 | 0 | 2.203050109 | 2.8285501 | PPIP5K1;IN |
| 0 | 0 | 2.203050109 | 2.8285501 | AFMID;KY/ |
| 0 | 0 | 2.203050109 | 2.8285501 | APOA4;CTS |
| 0 | 0 | 2.203050109 | 2.8285501 | GRN;PPT1 |
| 0 | 0 | 2.203050109 | 2.8285501 | ATP2B4;GL |
| 0 | 0 | 2.203050109 | 2.8285501 | ATP2B4;GL |

| | | | | |
|---|---|---|---|---|
| 0 | 0 | 2.203050109 | 2.8285501 | KCNIP2;PI( |
| 0 | 0 | 2.203050109 | 2.8285501 | APH1B;PSE |
| 0 | 0 | 2.203050109 | 2.8285501 | ENTPD5;EN |
| 0 | 0 | 2.203050109 | 2.8285501 | NEU3;MAN |
| 0 | 0 | 2.203050109 | 2.8285501 | HSD17B14 |
| 0 | 0 | 2.203050109 | 2.8285501 | TMX3;PAR |
| 0 | 0 | 2.203050109 | 2.8285501 | CNTNAP1;S |
| 0 | 0 | 2.203050109 | 2.8285501 | ACOT8;PEX |
| 0 | 0 | 2.203050109 | 2.8285501 | PARK7;SLC |
| 0 | 0 | 2.203050109 | 2.8285501 | XRCC1;GCL |
| 0 | 0 | 2.203050109 | 2.8285501 | SCIN;PITHD |
| 0 | 0 | 2.203050109 | 2.8285501 | STAP1;TRE |
| 0 | 0 | 2.203050109 | 2.8285501 | PDCD4;PPA |
| 0 | 0 | 2.203050109 | 2.8285501 | UGDH;SPA |
| 0 | 0 | 2.203050109 | 2.8285501 | AIFM2;ALC |
| 0 | 0 | 2.203050109 | 2.8285501 | CCL19;CD6 |
| 0 | 0 | 2.203050109 | 2.8285501 | USP14;UB( |
| 0 | 0 | 2.203050109 | 2.8285501 | INTS13;LHI |
| 0 | 0 | 2.203050109 | 2.8285501 | MSH6;POT |
| 0 | 0 | 2.203050109 | 2.8285501 | CHEK1;CEF |
| 0 | 0 | 2.203050109 | 2.8285501 | CDC7;DBF4 |
| 0 | 0 | 2.203050109 | 2.8285501 | IMPACT;W |
| 0 | 0 | 2.203050109 | 2.8285501 | HLCS;SPP1 |
| 0 | 0 | 2.203050109 | 2.8285501 | CNTNAP1;S |
| 0 | 0 | 2.203050109 | 2.8285501 | TREM2;C1( |
| 0 | 0 | 2.203050109 | 2.8285501 | MTHFD1;S |
| 0 | 0 | 2.203050109 | 2.8285501 | DECR2;FAD |
| 0 | 0 | 1.131420757 | 1.446330212 | TFRC;ITGB: |
| 0 | 0 | 1.227501692 | 1.562856847 | NCAPG2;KI |
| 0 | 0 | 1.227501692 | 1.562856847 | PSMD10;ZI |
| 0 | 0 | 1.483168882 | 1.886000893 | DYNC2I1;H |
| 0 | 0 | 1.483168882 | 1.886000893 | PDGFB;TW |
| 0 | 0 | 1.483168882 | 1.886000893 | LGALS3;HN |
| 0 | 0 | 1.483168882 | 1.886000893 | OSBPL7;M. |
| 0 | 0 | 1.483168882 | 1.886000893 | MSR1;ABC |
| 0 | 0 | 1.402325305 | 1.747636698 | CDC23;ZW |
| 0 | 0 | 1.198588594 | 1.485598479 | BEX3;CASP |
| 0 | 0 | 1.349792576 | 1.662647836 | AHI1;STIL;I |
| 0 | 0 | 1.349792576 | 1.662647836 | AHI1;STIL;I |
| 0 | 0 | 1.213640636 | 1.492050822 | DCTN5;LA( |
| 0 | 0 | 1.1692019 | 1.435775274 | WWTR1;G: |
| 0 | 0 | 1.223105591 | 1.498604331 | PSMD10;A |
| 0 | 0 | 1.312921186 | 1.606707091 | RNF113A;( |
| 0 | 0 | 1.312921186 | 1.606707091 | GPI;PFKL;G |
| 0 | 0 | 1.312921186 | 1.606707091 | FABP3;FAB |
| 0 | 0 | 1.542346271 | 1.884808131 | GPI;PFKL;A |
| 0 | 0 | 1.542346271 | 1.884808131 | STX12;ABC |

| | | | | |
|---|---|---|---|---|
| 0 | 0 | 1.542346271 | 1.884808131 | TRPM2;TRI. |
| 0 | 0 | 1.542346271 | 1.884808131 | GPI;PFKL;A. |
| 0 | 0 | 1.542346271 | 1.884808131 | ALOX5;DPI. |
| 0 | 0 | 1.542346271 | 1.884808131 | DDB1;LGAI. |
| 0 | 0 | 1.542346271 | 1.884808131 | IGF1;ADTR. |
| 0 | 0 | 1.542346271 | 1.884808131 | FITM2;MLY. |
| 0 | 0 | 1.285620629 | 1.568292159 | TRPM2;IM. |
| 0 | 0 | 1.264596042 | 1.541243942 | WWTR1;C(. |
| 0 | 0 | 1.264596042 | 1.541243942 | NDC1;P2R). |
| 0 | 0 | 1.141631594 | 1.373010165 | DNMT1;CI. |
| 0 | 0 | 1.19306631 | 1.423493739 | NDC1;WDI. |
| 0 | 0 | 1.428155905 | 1.701824406 | OGG1;SHLI. |
| 0 | 0 | 1.428155905 | 1.701824406 | AIFM1;PEX. |
| 0 | 0 | 1.428155905 | 1.701824406 | NTNG2;SP/. |
| 0 | 0 | 1.428155905 | 1.701824406 | CUL7;RAP2. |
| 0 | 0 | 1.428155905 | 1.701824406 | CUX2;CDK!. |
| 0 | 0 | 1.428155905 | 1.701824406 | LATS1;GPA. |
| 0 | 0 | 1.428155905 | 1.701824406 | PRIM2;POI. |
| 0 | 0 | 1.361003415 | 1.600702688 | RALBP1;ST. |
| 0 | 0 | 1.361003415 | 1.600702688 | DDAH2;AT. |
| 0 | 0 | 1.652353967 | 1.939042542 | WRN;XRCC. |
| 0 | 0 | 1.652353967 | 1.939042542 | SLC31A1;C. |
| 0 | 0 | 1.652353967 | 1.939042542 | IGF1;CLU;F. |
| 0 | 0 | 1.652353967 | 1.939042542 | PIGQ;PIGF;. |
| 0 | 0 | 1.652353967 | 1.939042542 | ZWILCH;ZV. |
| 0 | 0 | 1.652353967 | 1.939042542 | ABCC5;SLC. |
| 0 | 0 | 1.652353967 | 1.939042542 | ABCA2;SC/. |
| 0 | 0 | 1.652353967 | 1.939042542 | P2RX7;TRP. |
| 0 | 0 | 1.652353967 | 1.939042542 | ABCA5;AP(. |
| 0 | 0 | 1.652353967 | 1.939042542 | UBL4A;GET. |
| 0 | 0 | 1.316796251 | 1.538714482 | GUCY2C;SL. |
| 0 | 0 | 1.244128807 | 1.452909207 | NTNG2;PA. |
| 0 | 0 | 1.262174189 | 1.471335242 | LGALS3;W'. |
| 0 | 0 | 1.927559913 | 2.186157776 | COMT;ALD. |
| 0 | 0 | 1.927559913 | 2.186157776 | BLM;SPIDR. |
| 0 | 0 | 1.927559913 | 2.186157776 | ATP6V1A;F. |
| 0 | 0 | 1.927559913 | 2.186157776 | GNG2;TNF. |
| 0 | 0 | 1.927559913 | 2.186157776 | PRDX2;ABI. |
| 0 | 0 | 1.927559913 | 2.186157776 | TFAP4;RPS. |
| 0 | 0 | 1.927559913 | 2.186157776 | PFKL;PFKN. |
| 0 | 0 | 1.927559913 | 2.186157776 | SMARCAD. |
| 0 | 0 | 1.927559913 | 2.186157776 | ABCC5;SLC. |
| 0 | 0 | 1.927559913 | 2.186157776 | SHMT2;SEI. |
| 0 | 0 | 1.927559913 | 2.186157776 | BSCL2;FITN. |
| 0 | 0 | 1.927559913 | 2.186157776 | KIF3A;CEP. |
| 0 | 0 | 1.927559913 | 2.186157776 | CLU;SOD1. |
| 0 | 0 | 1.927559913 | 2.186157776 | ACER2;WN. |

| | | | | | |
|---|---|---|---|---|---|
| 0 | 0 | 1.927559913 | 2.186157776 | PPARG;TRE | . |
| 0 | 0 | 1.927559913 | 2.186157776 | EMILIN1;A | . |
| 0 | 0 | 1.927559913 | 2.186157776 | WNK1;MA | . |
| 0 | 0 | 1.927559913 | 2.186157776 | EMILIN1;A | . |
| 0 | 0 | 1.927559913 | 2.186157776 | TWIST1;TR | . |
| 0 | 0 | 1.927559913 | 2.186157776 | POT1;CERS | . |
| 0 | 0 | 1.927559913 | 2.186157776 | TTLL4;FKBI | . |
| 0 | 0 | 1.927559913 | 2.186157776 | SFRP4;KRT | . |
| 0 | 0 | 1.927559913 | 2.186157776 | POGLUT1;I | . |
| 0 | 0 | 1.927559913 | 2.186157776 | PARP1;PAF | . |
| 0 | 0 | 1.927559913 | 2.186157776 | RAP2A;RAI | . |
| 0 | 0 | 1.927559913 | 2.186157776 | EMILIN1;TI | . |
| 0 | 0 | 1.927559913 | 2.186157776 | WNK1;MA | . |
| 0 | 0 | 1.927559913 | 2.186157776 | CFAP206;D | . |
| 0 | 0 | 1.927559913 | 2.186157776 | TEK;BOK | . |
| 0 | 0 | 1.927559913 | 2.186157776 | RPS6KA5;T | . |
| 0 | 0 | 1.927559913 | 2.186157776 | PARK7;TXN | . |
| 0 | 0 | 1.927559913 | 2.186157776 | LATS1;SOD | . |
| 0 | 0 | 1.927559913 | 2.186157776 | DIP2A;PAR | . |
| 0 | 0 | 1.927559913 | 2.186157776 | STAP1;TRE | . |
| 0 | 0 | 1.927559913 | 2.186157776 | CLOCK;ARI | . |
| 0 | 0 | 1.927559913 | 2.186157776 | MYO1E;XP | . |
| 0 | 0 | 1.927559913 | 2.186157776 | STXBP6;VA | . |
| 0 | 0 | 1.468818142 | 1.665324187 | NDST1;B4( | . |
| 0 | 0 | 1.468818142 | 1.665324187 | ACADVL;C( | . |
| 0 | 0 | 1.468818142 | 1.665324187 | VKORC1L1; | . |
| 0 | 0 | 1.468818142 | 1.665324187 | GPI;PFKL;A | . |
| 0 | 0 | 1.468818142 | 1.665324187 | FUT8;STIL; | . |
| 0 | 0 | 1.468818142 | 1.665324187 | SCAMP5;L( | . |
| 0 | 0 | 1.468818142 | 1.665324187 | WFS1;ALO | . |
| 0 | 0 | 1.468818142 | 1.665324187 | TRPM2;ITP | . |
| 0 | 0 | 1.468818142 | 1.665324187 | PLA2G15;S | . |
| 0 | 0 | 1.468818142 | 1.665324187 | NEU3;CCD | . |
| 0 | 0 | 1.202148399 | 1.359965733 | MRPL30;M | . |
| 0 | 0 | 1.202148399 | 1.359965733 | AP1G2;OSI | . |
| 0 | 0 | 1.224311325 | 1.369733016 | SCARB2;VA | . |
| 0 | 0 | 1.377072426 | 1.537828626 | IMPACT;AU | . |
| 0 | 0 | 1.377072426 | 1.537828626 | PKIB;XRCC | . |
| 0 | 0 | 1.377072426 | 1.537828626 | ABCA2;RAI | . |
| 0 | 0 | 1.168831169 | 1.302683306 | EPHA10;XF | . |
| 0 | 0 | 1.239565122 | 1.380572543 | RBL2;PIDD | . |
| 0 | 0 | 1.259189914 | 1.397775093 | MSH6;ZRA | . |
| 0 | 0 | 1.259189914 | 1.397775093 | CBR1;SPHK | . |
| 0 | 0 | 1.322042776 | 1.46727324 | CASP9;ETA | . |
| 0 | 0 | 1.285371179 | 1.424662717 | CUL7;SPAS | . |
| 0 | 0 | 1.285371179 | 1.424662717 | RAP2A;GST | . |
| 0 | 0 | 1.107812964 | 1.216588891 | ATP6V1A;N | . |

| | | | | |
|---|---|---|---|---|
| 0 | 0 | 1.179736273 | 1.294019585 | PRKCG;GRI… |
| 0 | 0 | 1.179736273 | 1.294019585 | PRKCG;GRI… |
| 0 | 0 | 1.186668911 | 1.291346434 | WWTR1;UI… |
| 0 | 0 | 1.136268044 | 1.23051459 | RFC5;RFC3… |
| 0 | 0 | 1.194977388 | 1.290275544 | PLK4;P2RX… |
| 0 | 0 | 1.125082381 | 1.206243357 | SCARB2;NT… |
| 0 | 0 | 1.542109852 | 1.653037983 | ABCC5;SLC… |
| 0 | 0 | 1.542109852 | 1.653037983 | CITED2;IFT… |
| 0 | 0 | 1.542109852 | 1.653037983 | TADA3;TRI… |
| 0 | 0 | 1.542109852 | 1.653037983 | GSTP1;EMI… |
| 0 | 0 | 1.542109852 | 1.653037983 | CAB39;COI… |
| 0 | 0 | 1.542109852 | 1.653037983 | GPNMB;W… |
| 0 | 0 | 1.542109852 | 1.653037983 | PSMD10;T… |
| 0 | 0 | 1.542109852 | 1.653037983 | RPS6KA5;L… |
| 0 | 0 | 1.542109852 | 1.653037983 | EMILIN1;IE… |
| 0 | 0 | 1.542109852 | 1.653037983 | CD86;TNFS… |
| 0 | 0 | 1.542109852 | 1.653037983 | SASH3;TNF… |
| 0 | 0 | 1.542109852 | 1.653037983 | FABP3;PDK… |
| 0 | 0 | 1.542109852 | 1.653037983 | ZNF205;TM… |
| 0 | 0 | 1.542109852 | 1.653037983 | BBS2;IGF1;… |
| 0 | 0 | 1.542109852 | 1.653037983 | GGCT;CLU;… |
| 0 | 0 | 1.542109852 | 1.653037983 | GPAM;MO… |
| 0 | 0 | 1.205113139 | 1.291390739 | RBL2;ACER… |
| 0 | 0 | 1.205113139 | 1.291390739 | DDB1;PSM… |
| 0 | 0 | 1.217749356 | 1.295571969 | RIPOR1;WI… |
| 0 | 0 | 1.233936217 | 1.304206104 | KANK1;RUI… |
| 0 | 0 | 1.233936217 | 1.304206104 | CALCRL;TFI… |
| 0 | 0 | 1.108509896 | 1.171215534 | PI4K2B;CU… |
| 0 | 0 | 1.401974388 | 1.475201062 | CARMIL1;C… |
| 0 | 0 | 1.401974388 | 1.475201062 | TMEM161… |
| 0 | 0 | 1.401974388 | 1.475201062 | ACOT8;PE… |
| 0 | 0 | 1.401974388 | 1.475201062 | PPP3CC;YV… |
| 0 | 0 | 1.401974388 | 1.475201062 | CHST10;CH… |
| 0 | 0 | 1.401974388 | 1.475201062 | HMGB1;PL… |
| 0 | 0 | 1.401974388 | 1.475201062 | TENM4;KIF… |
| 0 | 0 | 1.401974388 | 1.475201062 | PPP3CC;YV… |
| 0 | 0 | 1.142773637 | 1.20134448 | SETD4;FZD… |
| 0 | 0 | 1.255407738 | 1.319606452 | AUTS2;CDK… |
| 0 | 0 | 1.285246617 | 1.34592909 | PI4K2B;TT… |
| 0 | 0 | 1.285246617 | 1.34592909 | DLG4;PDGI… |
| 0 | 0 | 1.285246617 | 1.34592909 | LATS1;XPO… |
| 0 | 0 | 1.285246617 | 1.34592909 | DYNC2I1;W… |
| 0 | 0 | 1.329511946 | 1.391439588 | SESN3;RRA… |
| 0 | 0 | 1.329511946 | 1.391439588 | ATP2B4;PP… |
| 0 | 0 | 1.329511946 | 1.391439588 | PKIB;XRCC… |
| 0 | 0 | 1.329511946 | 1.391439588 | SLC45A3;P… |
| 0 | 0 | 1.171581309 | 1.226151305 | CLIP3;DAB… |

| | | | | | |
|---|---|---|---|---|---|
| 0 | 0 | 1.119697377 | 1.127900172 | FARP2;RIP | . |
| 0 | 0 | 1.20967286 | 1.217155032 | ERCC6L2;G | . |
| 0 | 0 | 1.71328976 | 1.723464355 | FHOD3;CA | . |
| 0 | 0 | 1.71328976 | 1.723464355 | SPAST;MG | . |
| 0 | 0 | 1.71328976 | 1.723464355 | KDM2B;IFT | . |
| 0 | 0 | 1.71328976 | 1.723464355 | SLC25A20; | . |
| 0 | 0 | 1.71328976 | 1.723464355 | GRAMD1B | . |
| 0 | 0 | 1.71328976 | 1.723464355 | PPCDC;AC | . |
| 0 | 0 | 1.71328976 | 1.723464355 | SLC31A1;A | . |
| 0 | 0 | 1.71328976 | 1.723464355 | CEP250;CA | . |
| 0 | 0 | 1.71328976 | 1.723464355 | CEP250;CA | . |
| 0 | 0 | 1.71328976 | 1.723464355 | TFAP4;RPS | . |
| 0 | 0 | 1.71328976 | 1.723464355 | KDM2B;MI | . |
| 0 | 0 | 1.71328976 | 1.723464355 | COQ3;PDX | . |
| 0 | 0 | 1.71328976 | 1.723464355 | PPIP5K1;IP | . |
| 0 | 0 | 1.71328976 | 1.723464355 | MTERF2;TE | . |
| 0 | 0 | 1.71328976 | 1.723464355 | TOM1L2;C | . |
| 0 | 0 | 1.71328976 | 1.723464355 | ACP4;GAS1 | . |
| 0 | 0 | 1.71328976 | 1.723464355 | MAP2K5;T | . |
| 0 | 0 | 1.71328976 | 1.723464355 | SETD4;ME | . |
| 0 | 0 | 1.71328976 | 1.723464355 | NUCKS1;Z | . |
| 0 | 0 | 1.71328976 | 1.723464355 | CDC7;DBF | . |
| 0 | 0 | 1.71328976 | 1.723464355 | SPHK2;TNF | . |
| 0 | 0 | 1.71328976 | 1.723464355 | TENM4;DI | . |
| 0 | 0 | 1.71328976 | 1.723464355 | GRN;LRSA | . |
| 0 | 0 | 1.71328976 | 1.723464355 | DDB1;ZNF | . |
| 0 | 0 | 1.71328976 | 1.723464355 | KCNIP2;TR | . |
| 0 | 0 | 1.71328976 | 1.723464355 | PSMD10;P | . |
| 0 | 0 | 1.71328976 | 1.723464355 | TMX3;LOX | . |
| 0 | 0 | 1.71328976 | 1.723464355 | PPCDC;AC | . |
| 0 | 0 | 1.71328976 | 1.723464355 | PUDP;TK1 | . |
| 0 | 0 | 1.71328976 | 1.723464355 | PUDP;TK1 | . |
| 0 | 0 | 1.71328976 | 1.723464355 | SUV39H1; | . |
| 0 | 0 | 1.71328976 | 1.723464355 | ABCA2;NE | . |
| 0 | 0 | 1.71328976 | 1.723464355 | PARK7;MN | . |
| 0 | 0 | 1.71328976 | 1.723464355 | CCL19;GAS | . |
| 0 | 0 | 1.71328976 | 1.723464355 | DRC1;HDA | . |
| 0 | 0 | 1.71328976 | 1.723464355 | GSTP1;TW | . |
| 0 | 0 | 1.71328976 | 1.723464355 | HMGB1;RT | . |
| 0 | 0 | 1.71328976 | 1.723464355 | BLM;SPIDR | . |
| 0 | 0 | 1.71328976 | 1.723464355 | PPCDC;AC | . |
| 0 | 0 | 1.71328976 | 1.723464355 | MTHFD1;L | . |
| 0 | 0 | 1.71328976 | 1.723464355 | SMPD3;AB | . |
| 0 | 0 | 1.71328976 | 1.723464355 | QDPR;SPR | . |
| 0 | 0 | 1.71328976 | 1.723464355 | MTHFD1;S | . |
| 0 | 0 | 1.71328976 | 1.723464355 | AFMID;KY | . |
| 0 | 0 | 1.71328976 | 1.723464355 | CARMIL1;A | . |

| | | | | |
|---|---|---|---|---|
| 0 | 0 | 1.71328976 | 1.723464355 | LIG1;HMGI |
| 0 | 0 | 1.06512684 | 1.055920833 | TFRC;CAB3 |
| 0 | 0 | 1.25043944 | 1.235425479 | AHI1;STIL;I |
| 0 | 0 | 1.25043944 | 1.235425479 | IMPACT;BF |
| 0 | 0 | 1.25043944 | 1.235425479 | SCARB2;NI |
| 0 | 0 | 1.168462058 | 1.148023852 | IPO11;NXT |
| 0 | 0 | 1.168462058 | 1.148023852 | CD86;TNFS |
| 0 | 0 | 1.078049298 | 1.058853457 | TTC26;LAN |
| 0 | 0 | 1.285122164 | 1.260469524 | CDK5;TREN |
| 0 | 0 | 1.285122164 | 1.260469524 | TRPM2;IM |
| 0 | 0 | 1.285122164 | 1.260469524 | CD86;GPNI |
| 0 | 0 | 1.285122164 | 1.260469524 | SYDE1;ANK |
| 0 | 0 | 1.285122164 | 1.260469524 | LRSAM1;N |
| 0 | 0 | 1.445646251 | 1.416878094 | EPS8;SCIN; |
| 0 | 0 | 1.445646251 | 1.416878094 | EPS8;SCIN; |
| 0 | 0 | 1.445646251 | 1.416878094 | PKD1L2;PK |
| 0 | 0 | 1.445646251 | 1.416878094 | ALOX5;MG |
| 0 | 0 | 1.445646251 | 1.416878094 | IGF1;CLU;F |
| 0 | 0 | 1.445646251 | 1.416878094 | AGTPBP1;U |
| 0 | 0 | 1.445646251 | 1.416878094 | ABCA2;RNI |
| 0 | 0 | 1.445646251 | 1.416878094 | HMGB1;PL |
| 0 | 0 | 1.445646251 | 1.416878094 | TPST1;ABH |
| 0 | 0 | 1.445646251 | 1.416878094 | FARP2;EPS |
| 0 | 0 | 1.445646251 | 1.416878094 | FZD5;PARK |
| 0 | 0 | 1.445646251 | 1.416878094 | DNMT1;AU |
| 0 | 0 | 1.445646251 | 1.416878094 | CDH3;IGFB |
| 0 | 0 | 1.445646251 | 1.416878094 | TNFSF18;L |
| 0 | 0 | 1.445646251 | 1.416878094 | CAMK2A;A |
| 0 | 0 | 1.445646251 | 1.416878094 | ABCA2;GRA |
| 0 | 0 | 1.445646251 | 1.416878094 | MYO1E;MY |
| 0 | 0 | 1.340943135 | 1.3098084 | ARL6IP5;SL |
| 0 | 0 | 1.340943135 | 1.3098084 | NCAPG2;N |
| 0 | 0 | 1.340943135 | 1.3098084 | PDK4;SLC1 |
| 0 | 0 | 1.340943135 | 1.3098084 | DDAH2;CD |
| 0 | 0 | 1.340943135 | 1.3098084 | TRPM2;ITP |
| 0 | 0 | 1.110676613 | 1.083987804 | SCARB2;SL |
| 0 | 0 | 1.110676613 | 1.083987804 | SCARB2;M |
| 0 | 0 | 1.123184398 | 1.089908161 | NDC1;NEK |
| 0 | 0 | 1.186342709 | 1.136124059 | TRPM2;AB |
| 0 | 0 | 1.199475983 | 1.133386702 | PM20D1;S |
| 0 | 0 | 1.199475983 | 1.133386702 | SHTN1;CN |
| 0 | 0 | 1.199475983 | 1.133386702 | CPNE9;SHT |
| 0 | 0 | 1.199475983 | 1.133386702 | PKIB;STN1; |
| 0 | 0 | 1.126458608 | 1.063207603 | CPNE9;PSN |
| 0 | 0 | 1.150957064 | 1.082458781 | CYP27A1;A |
| 0 | 0 | 1.150957064 | 1.082458781 | VAT1;KANI |
| 0 | 0 | 1.217464219 | 1.134667185 | WWTR1;ZH |

| | | | | |
|---|---|---|---|---|
| 0 | 0 | 1.128526445 | 1.049378936 | DUS2;PM2… |
| 0 | 0 | 1.128526445 | 1.049378936 | GPI;BCAS3… |
| 0 | 0 | 1.128526445 | 1.049378936 | TSPYL2;RBI… |
| 0 | 0 | 1.113040808 | 1.027362109 | SLC27A1;B… |
| 0 | 0 | 1.113040808 | 1.027362109 | RNF43;KAI… |
| 0 | 0 | 1.243596239 | 1.143049588 | POGLUT1;I… |
| 0 | 0 | 1.243596239 | 1.143049588 | CDH3;PRD… |
| 0 | 0 | 1.130988626 | 1.035198748 | WWTR1;IN… |
| 0 | 0 | 1.130988626 | 1.035198748 | PSMD10;D… |
| 0 | 0 | 1.080736334 | 0.986706298 | STX12;CRT… |
| 0 | 0 | 1.163892942 | 1.060658986 | PIGU;PIGQ… |
| 0 | 0 | 1.163892942 | 1.060658986 | CDK8;MED… |
| 0 | 0 | 1.163892942 | 1.060658986 | SCAMP5;C… |
| 0 | 0 | 1.113879159 | 1.012068791 | PDXP;TRPV… |
| 0 | 0 | 1.284997819 | 1.165375894 | STARD5;SL… |
| 0 | 0 | 1.284997819 | 1.165375894 | DMAC2L;A… |
| 0 | 0 | 1.284997819 | 1.165375894 | MCUR1;M… |
| 0 | 0 | 1.284997819 | 1.165375894 | PLA2G15;G… |
| 0 | 0 | 1.284997819 | 1.165375894 | GSTO1;GN… |
| 0 | 0 | 1.284997819 | 1.165375894 | FBXL8;CRT… |
| 0 | 0 | 1.284997819 | 1.165375894 | TOGARAM… |
| 0 | 0 | 1.284997819 | 1.165375894 | DDAH2;CD… |
| 0 | 0 | 1.284997819 | 1.165375894 | RIPOR1;GC… |
| 0 | 0 | 1.284997819 | 1.165375894 | PSMD10;T… |
| 0 | 0 | 1.284997819 | 1.165375894 | CD86;SASH… |
| 0 | 0 | 1.284997819 | 1.165375894 | CDK5;ABI3… |
| 0 | 0 | 1.284997819 | 1.165375894 | SPHK2;ALC… |
| 0 | 0 | 1.284997819 | 1.165375894 | POP7;LACT… |
| 0 | 0 | 1.360531309 | 1.220202057 | BLM;ATP2I… |
| 0 | 0 | 1.360531309 | 1.220202057 | PPP3CA;PF… |
| 0 | 0 | 1.360531309 | 1.220202057 | PARK7;CAI… |
| 0 | 0 | 1.360531309 | 1.220202057 | GPR137;TC… |
| 0 | 0 | 1.360531309 | 1.220202057 | STN1;TEN1… |
| 0 | 0 | 1.360531309 | 1.220202057 | POLD2;ADI… |
| 0 | 0 | 1.360531309 | 1.220202057 | CUL7;CUX2… |
| 0 | 0 | 1.360531309 | 1.220202057 | AHI1;SFRP4… |
| 0 | 0 | 1.360531309 | 1.220202057 | CADM1;CR… |
| 0 | 0 | 1.360531309 | 1.220202057 | WFS1;PDG… |
| 0 | 0 | 1.360531309 | 1.220202057 | SCARB2;GC… |
| 0 | 0 | 1.360531309 | 1.220202057 | DSTYK;GPC… |
| 0 | 0 | 1.173333966 | 1.050869588 | VAV3;SASH… |
| 0 | 0 | 1.541873638 | 1.380185107 | EPS8;BAIAI… |
| 0 | 0 | 1.541873638 | 1.380185107 | MEGF8;RTI… |
| 0 | 0 | 1.541873638 | 1.380185107 | AGTPBP1;T… |
| 0 | 0 | 1.541873638 | 1.380185107 | SYT2;DOC2… |
| 0 | 0 | 1.541873638 | 1.380185107 | ZWILCH;ZV… |
| 0 | 0 | 1.541873638 | 1.380185107 | PKD2;MAP… |

| | | | | |
|---|---|---|---|---|
| 0 | 0 | 1.541873638 | 1.380185107 | THRB;CTSE |
| 0 | 0 | 1.541873638 | 1.380185107 | GUCY2C;PI |
| 0 | 0 | 1.541873638 | 1.380185107 | OGG1;POT |
| 0 | 0 | 1.541873638 | 1.380185107 | AGTPBP1;( |
| 0 | 0 | 1.541873638 | 1.380185107 | TUBB3;SEN |
| 0 | 0 | 1.541873638 | 1.380185107 | RIPOR1;TB |
| 0 | 0 | 1.541873638 | 1.380185107 | LAMA3;LAI |
| 0 | 0 | 1.541873638 | 1.380185107 | TST;MMUT |
| 0 | 0 | 1.541873638 | 1.380185107 | LGALS3;ED |
| 0 | 0 | 1.541873638 | 1.380185107 | NIPA1;SLC |
| 0 | 0 | 1.541873638 | 1.380185107 | UBA5;ZFPN |
| 0 | 0 | 1.541873638 | 1.380185107 | KIF3A;CEP |
| 0 | 0 | 1.541873638 | 1.380185107 | RFWD3;RP |
| 0 | 0 | 1.541873638 | 1.380185107 | PRKCA;VRI |
| 0 | 0 | 1.541873638 | 1.380185107 | AHI1;LAM/ |
| 0 | 0 | 1.541873638 | 1.380185107 | TREM2;CLI |
| 0 | 0 | 1.541873638 | 1.380185107 | ACTN3;ATF |
| 0 | 0 | 1.541873638 | 1.380185107 | ACTN3;ATF |
| 0 | 0 | 1.541873638 | 1.380185107 | BLM;ZFYVE |
| 0 | 0 | 1.541873638 | 1.380185107 | PPARG;ESF |
| 0 | 0 | 1.541873638 | 1.380185107 | ETFBKMT;I |
| 0 | 0 | 1.541873638 | 1.380185107 | SLC1A3;SL( |
| 0 | 0 | 1.541873638 | 1.380185107 | PUDP;TK1 |
| 0 | 0 | 1.541873638 | 1.380185107 | LSM4;LSM |
| 0 | 0 | 1.541873638 | 1.380185107 | SLC37A4;S |
| 0 | 0 | 1.541873638 | 1.380185107 | B4GAT1;B3 |
| 0 | 0 | 1.541873638 | 1.380185107 | TWIST1;MI |
| 0 | 0 | 1.541873638 | 1.380185107 | STAP1;TIC/ |
| 0 | 0 | 1.541873638 | 1.380185107 | STAP1;TRE |
| 0 | 0 | 1.541873638 | 1.380185107 | TFR2;NKD2 |
| 0 | 0 | 1.541873638 | 1.380185107 | GORASP1;( |
| 0 | 0 | 1.541873638 | 1.380185107 | PARP1;PAF |
| 0 | 0 | 1.541873638 | 1.380185107 | DLG4;ZDHI |
| 0 | 0 | 1.541873638 | 1.380185107 | ALDH6A1;I |
| 0 | 0 | 1.541873638 | 1.380185107 | TNFSF4;HN |
| 0 | 0 | 1.541873638 | 1.380185107 | ABCA2;AP( |
| 0 | 0 | 1.541873638 | 1.380185107 | TACR2;CFA |
| 0 | 0 | 1.541873638 | 1.380185107 | DNMT1;DN |
| 0 | 0 | 1.541873638 | 1.380185107 | WNK1;ADT |
| 0 | 0 | 1.541873638 | 1.380185107 | PCBP3;IGF |
| 0 | 0 | 1.541873638 | 1.380185107 | AIFM2;ALC |
| 0 | 0 | 1.541873638 | 1.380185107 | ZFHX2;SCN |
| 0 | 0 | 1.541873638 | 1.380185107 | PDGFB;PRI |
| 0 | 0 | 1.541873638 | 1.380185107 | PKD2L1;CD |
| 0 | 0 | 1.541873638 | 1.380185107 | MTHFD1;S |
| 0 | 0 | 1.541873638 | 1.380185107 | EDNRB;TM |
| 0 | 0 | 1.541873638 | 1.380185107 | CTSK;DUO |

| | | | | |
|---|---|---|---|---|
| 0 | 0 | 1.541873638 | 1.380185107 | IDH1;ACO1 |
| 0 | 0 | 1.541873638 | 1.380185107 | GSTZ1;FAH |
| 0 | 0 | 1.06381362 | 0.947110867 | MAGEH1;P |
| 0 | 0 | 1.066617012 | 0.948129863 | CCDC125;A |
| 0 | 0 | 1.091898057 | 0.969252087 | ATP6V1A;S |
| 0 | 0 | 1.137639 | 1.005693882 | MCFD2;TR |
| 0 | 0 | 1.186180036 | 1.042687636 | AIFM1;NCA |
| 0 | 0 | 1.186180036 | 1.042687636 | CD86;LGAL |
| 0 | 0 | 1.186180036 | 1.042687636 | BRMS1L;M |
| 0 | 0 | 1.101576182 | 0.963309184 | SRD5A1;HS |
| 0 | 0 | 1.204665685 | 1.037575901 | SPAST;KIF3 |
| 0 | 0 | 1.204665685 | 1.037575901 | SEMA3A;SI |
| 0 | 0 | 1.204665685 | 1.037575901 | UBL4A;ALD |
| 0 | 0 | 1.204665685 | 1.037575901 | CDK5;REEP |
| 0 | 0 | 1.148304376 | 0.974622721 | CCNB3;BLM |
| 0 | 0 | 1.148304376 | 0.974622721 | SCIN;AGO4 |
| 0 | 0 | 1.148304376 | 0.974622721 | MYBPC3;C |
| 0 | 0 | 1.11922654 | 0.94412681 | PRKCG;GRI |
| 0 | 0 | 1.11922654 | 0.94412681 | LSM10;RBI |
| 0 | 0 | 1.233528129 | 1.038808652 | ZW10;SPD |
| 0 | 0 | 1.233528129 | 1.038808652 | SYCE2;STA |
| 0 | 0 | 1.233528129 | 1.038808652 | CARMIL1;C |
| 0 | 0 | 1.233528129 | 1.038808652 | IQCE;IFT12 |
| 0 | 0 | 1.233528129 | 1.038808652 | PPT1;HSD1 |
| 0 | 0 | 1.233528129 | 1.038808652 | TNFSF4;HN |
| 0 | 0 | 1.233528129 | 1.038808652 | NPRL2;POT |
| 0 | 0 | 1.233528129 | 1.038808652 | DDB1;PPIH |
| 0 | 0 | 1.233528129 | 1.038808652 | MRNIP;SHI |
| 0 | 0 | 1.233528129 | 1.038808652 | ACADVL;SL |
| 0 | 0 | 1.233528129 | 1.038808652 | ELMOD2;T |
| 0 | 0 | 1.101487314 | 0.927059073 | TRPM2;CA |
| 0 | 0 | 1.156460061 | 0.958631354 | GSTP1;CID |
| 0 | 0 | 1.156460061 | 0.958631354 | PIK3R2;EF( |
| 0 | 0 | 1.156460061 | 0.958631354 | ABCA2;PAF |
| 0 | 0 | 1.156460061 | 0.958631354 | PKIB;XRCC |
| 0 | 0 | 1.079465615 | 0.891963532 | SLF1;TFRC; |
| 0 | 0 | 1.121442472 | 0.924801648 | FARP2;SYD |
| 0 | 0 | 1.088510748 | 0.897590633 | B4GALT2;N |
| 0 | 0 | 1.101442938 | 0.907372977 | WWTR1;AI |
| 0 | 0 | 1.284873583 | 1.055179608 | MYO1E;MY |
| 0 | 0 | 1.284873583 | 1.055179608 | SDHB;SLC2 |
| 0 | 0 | 1.284873583 | 1.055179608 | BLM;MMP |
| 0 | 0 | 1.284873583 | 1.055179608 | ABCD4;MN |
| 0 | 0 | 1.284873583 | 1.055179608 | GSTP1;ALC |
| 0 | 0 | 1.284873583 | 1.055179608 | MCUR1;M( |
| 0 | 0 | 1.284873583 | 1.055179608 | NAXE;QPR |
| 0 | 0 | 1.284873583 | 1.055179608 | DAB2;ABI3 |

| | | | | |
|---|---|---|---|---|
| 0 | 0 | 1.284873583 | 1.055179608 | DENND1B; . |
| 0 | 0 | 1.284873583 | 1.055179608 | TWIST1;IGI . |
| 0 | 0 | 1.284873583 | 1.055179608 | MRNIP;MC . |
| 0 | 0 | 1.284873583 | 1.055179608 | P2RX7;ENT . |
| 0 | 0 | 1.284873583 | 1.055179608 | GSTO1;CLI . |
| 0 | 0 | 1.284873583 | 1.055179608 | CLASP1;CC . |
| 0 | 0 | 1.284873583 | 1.055179608 | BSCL2;CIDI . |
| 0 | 0 | 1.284873583 | 1.055179608 | PRKCA;CD3 . |
| 0 | 0 | 1.284873583 | 1.055179608 | APOA4;AN . |
| 0 | 0 | 1.284873583 | 1.055179608 | FASTKD2;S . |
| 0 | 0 | 1.284873583 | 1.055179608 | ICE2;POLR3 . |
| 0 | 0 | 1.284873583 | 1.055179608 | TRPM2;SLC . |
| 0 | 0 | 1.077763968 | 0.872484926 | PSMD10;SI . |
| 0 | 0 | 1.168094558 | 0.942665978 | MYBPC3;M . |
| 0 | 0 | 1.168094558 | 0.942665978 | MYBPC3;M . |
| 0 | 0 | 1.168094558 | 0.942665978 | SVBP;ATP2 . |
| 0 | 0 | 1.168094558 | 0.942665978 | CD86;SASH . |
| 0 | 0 | 1.168094558 | 0.942665978 | PTPMT1;M . |
| 0 | 0 | 1.087303991 | 0.877230778 | ALDH3A2;I . |
| 0 | 0 | 1.101398601 | 0.886353802 | CUL7;SPAS . |
| 0 | 0 | 1.101398601 | 0.886353802 | CASP9;MLI . |
| 0 | 0 | 1.124317686 | 0.904124125 | AKT3;PDGI . |
| 0 | 0 | 1.401624084 | 1.119420086 | IDUA;HEXA . |
| 0 | 0 | 1.401624084 | 1.119420086 | IDUA;CHST . |
| 0 | 0 | 1.401624084 | 1.119420086 | KCNIP2;KC . |
| 0 | 0 | 1.401624084 | 1.119420086 | FBXW4;ME . |
| 0 | 0 | 1.401624084 | 1.119420086 | POT1;BRCA . |
| 0 | 0 | 1.401624084 | 1.119420086 | GCC2;VAM . |
| 0 | 0 | 1.401624084 | 1.119420086 | ALOX5;PLA . |
| 0 | 0 | 1.401624084 | 1.119420086 | SPAST;MG . |
| 0 | 0 | 1.401624084 | 1.119420086 | EDNRB;PAI . |
| 0 | 0 | 1.401624084 | 1.119420086 | SCAP;SOD1 . |
| 0 | 0 | 1.401624084 | 1.119420086 | TWSG1;HN . |
| 0 | 0 | 1.401624084 | 1.119420086 | KANK1;COI . |
| 0 | 0 | 1.401624084 | 1.119420086 | H1-10;ASF . |
| 0 | 0 | 1.401624084 | 1.119420086 | H1-10;ASF . |
| 0 | 0 | 1.401624084 | 1.119420086 | H1-10;ASF . |
| 0 | 0 | 1.401624084 | 1.119420086 | DNMT1;DN . |
| 0 | 0 | 1.401624084 | 1.119420086 | MIF;TAF9B . |
| 0 | 0 | 1.401624084 | 1.119420086 | ADTRP;NBI . |
| 0 | 0 | 1.401624084 | 1.119420086 | KANK1;COI . |
| 0 | 0 | 1.401624084 | 1.119420086 | SHPK;PGD . |
| 0 | 0 | 1.401624084 | 1.119420086 | B4GAT1;B3 . |
| 0 | 0 | 1.401624084 | 1.119420086 | HDAC6;SRI . |
| 0 | 0 | 1.401624084 | 1.119420086 | SCAMP5;D . |
| 0 | 0 | 1.401624084 | 1.119420086 | P2RX3;TRE . |
| 0 | 0 | 1.401624084 | 1.119420086 | SLC45A3;A . |

| | | | | | |
|---|---|---|---|---|---|
| 0 | 0 | 1.401624084 | 1.119420086 | GKAP1;SN) | . |
| 0 | 0 | 1.401624084 | 1.119420086 | SPP1;PRAP | . |
| 0 | 0 | 1.401624084 | 1.119420086 | RANGRF;G | . |
| 0 | 0 | 1.401624084 | 1.119420086 | ZNF205;CD | . |
| 0 | 0 | 1.401624084 | 1.119420086 | WNK3;GPD | . |
| 0 | 0 | 1.401624084 | 1.119420086 | APOA4;LDI | . |
| 0 | 0 | 1.401624084 | 1.119420086 | QDPR;SPR | . |
| 0 | 0 | 1.401624084 | 1.119420086 | HMGB1;M | . |
| 0 | 0 | 1.401624084 | 1.119420086 | HPF1;PARK | . |
| 0 | 0 | 1.401624084 | 1.119420086 | GRN;PPT1 | . |
| 0 | 0 | 1.401624084 | 1.119420086 | SYNGR1;SY | . |
| 0 | 0 | 1.401624084 | 1.119420086 | FOXJ1;SOD | . |
| 0 | 0 | 1.401624084 | 1.119420086 | SCPEP1;GC | . |
| 0 | 0 | 1.401624084 | 1.119420086 | TFR2;ACO1 | . |
| 0 | 0 | 1.401624084 | 1.119420086 | THRB;CTSE | . |
| 0 | 0 | 1.401624084 | 1.119420086 | SEMA3A;P | . |
| 0 | 0 | 1.401624084 | 1.119420086 | LOXL3;PKD | . |
| 0 | 0 | 1.401624084 | 1.119420086 | CLIP3;COP( | . |
| 0 | 0 | 1.401624084 | 1.119420086 | POP7;POP4 | . |
| 0 | 0 | 1.401624084 | 1.119420086 | SCPEP1;GC | . |
| 0 | 0 | 1.068717121 | 0.850740786 | CD86;FZD5 | . |
| 0 | 0 | 1.057028155 | 0.833812494 | VAV3;RALE | . |
| 0 | 0 | 1.037304591 | 0.81641237 | TFRC;EPHA | . |
| 0 | 0 | 1.101354303 | 0.86368158 | PSMD10;P | . |
| 0 | 0 | 1.101354303 | 0.86368158 | DTWD1;PC | . |
| 0 | 0 | 1.060995907 | 0.831648096 | PSMD10;F | . |
| 0 | 0 | 1.927155923 | 1.510456531 | CD86 | . |
| 0 | 0 | 1.927155923 | 1.510456531 | SLC25A4 | . |
| 0 | 0 | 1.927155923 | 1.510456531 | ADSS1 | . |
| 0 | 0 | 1.927155923 | 1.510456531 | CD1B | . |
| 0 | 0 | 1.927155923 | 1.510456531 | CD1B | . |
| 0 | 0 | 1.927155923 | 1.510456531 | CD1B | . |
| 0 | 0 | 1.927155923 | 1.510456531 | SOD1 | . |
| 0 | 0 | 1.927155923 | 1.510456531 | SOD1 | . |
| 0 | 0 | 1.927155923 | 1.510456531 | AGTPBP1 | . |
| 0 | 0 | 1.927155923 | 1.510456531 | MEGF8 | . |
| 0 | 0 | 1.927155923 | 1.510456531 | PLPP3 | . |
| 0 | 0 | 1.927155923 | 1.510456531 | HCN2 | . |
| 0 | 0 | 1.927155923 | 1.510456531 | RHEX | . |
| 0 | 0 | 1.927155923 | 1.510456531 | PDE1B | . |
| 0 | 0 | 1.927155923 | 1.510456531 | PDK2 | . |
| 0 | 0 | 1.927155923 | 1.510456531 | SPP1 | . |
| 0 | 0 | 1.927155923 | 1.510456531 | AGTPBP1 | . |
| 0 | 0 | 1.927155923 | 1.510456531 | CHD5 | . |
| 0 | 0 | 1.927155923 | 1.510456531 | TREM2 | . |
| 0 | 0 | 1.927155923 | 1.510456531 | CNTFR | . |
| 0 | 0 | 1.927155923 | 1.510456531 | YBX1 | . |

| | | | | | |
|---|---|---|---|---|---|
| 0 | 0 | 1.927155923 | 1.510456531 | SLC19A1 | . |
| 0 | 0 | 1.927155923 | 1.510456531 | PNRC2 | . |
| 0 | 0 | 1.927155923 | 1.510456531 | NBL1 | . |
| 0 | 0 | 1.927155923 | 1.510456531 | CITED2 | . |
| 0 | 0 | 1.927155923 | 1.510456531 | XRCC1 | . |
| 0 | 0 | 1.927155923 | 1.510456531 | MTHFD1 | . |
| 0 | 0 | 1.927155923 | 1.510456531 | FAM20C | . |
| 0 | 0 | 1.927155923 | 1.510456531 | EDNRB | . |
| 0 | 0 | 1.927155923 | 1.510456531 | FOXJ1 | . |
| 0 | 0 | 1.927155923 | 1.510456531 | SLC2A8 | . |
| 0 | 0 | 1.927155923 | 1.510456531 | ALDH5A1 | . |
| 0 | 0 | 1.927155923 | 1.510456531 | KANK1 | . |
| 0 | 0 | 1.927155923 | 1.510456531 | SLC5A2 | . |
| 0 | 0 | 1.927155923 | 1.510456531 | SLC37A4 | . |
| 0 | 0 | 1.927155923 | 1.510456531 | ABCC5 | . |
| 0 | 0 | 1.927155923 | 1.510456531 | PDXP | . |
| 0 | 0 | 1.927155923 | 1.510456531 | NCEH1 | . |
| 0 | 0 | 1.927155923 | 1.510456531 | GAS6 | . |
| 0 | 0 | 1.927155923 | 1.510456531 | IDUA | . |
| 0 | 0 | 1.927155923 | 1.510456531 | SLC37A4 | . |
| 0 | 0 | 1.927155923 | 1.510456531 | PADI6 | . |
| 0 | 0 | 1.927155923 | 1.510456531 | SETD4 | . |
| 0 | 0 | 1.927155923 | 1.510456531 | SETD4 | . |
| 0 | 0 | 1.927155923 | 1.510456531 | TST | . |
| 0 | 0 | 1.927155923 | 1.510456531 | FTL | . |
| 0 | 0 | 1.927155923 | 1.510456531 | IDH1 | . |
| 0 | 0 | 1.927155923 | 1.510456531 | SLC1A3 | . |
| 0 | 0 | 1.927155923 | 1.510456531 | GCC2 | . |
| 0 | 0 | 1.927155923 | 1.510456531 | PLEKHM1 | . |
| 0 | 0 | 1.927155923 | 1.510456531 | ADTRP | . |
| 0 | 0 | 1.927155923 | 1.510456531 | EDEM2 | . |
| 0 | 0 | 1.927155923 | 1.510456531 | CCL19 | . |
| 0 | 0 | 1.927155923 | 1.510456531 | ACADM | . |
| 0 | 0 | 1.927155923 | 1.510456531 | SGO2 | . |
| 0 | 0 | 1.927155923 | 1.510456531 | NAXE | . |
| 0 | 0 | 1.927155923 | 1.510456531 | PKD2 | . |
| 0 | 0 | 1.927155923 | 1.510456531 | PKD2 | . |
| 0 | 0 | 1.927155923 | 1.510456531 | AGO4 | . |
| 0 | 0 | 1.927155923 | 1.510456531 | ARL6IP5 | . |
| 0 | 0 | 1.927155923 | 1.510456531 | HMGB1 | . |
| 0 | 0 | 1.927155923 | 1.510456531 | P2RX7 | . |
| 0 | 0 | 1.927155923 | 1.510456531 | ACTN3 | . |
| 0 | 0 | 1.927155923 | 1.510456531 | ORMDL1 | . |
| 0 | 0 | 1.927155923 | 1.510456531 | EIF2AK4 | . |
| 0 | 0 | 1.927155923 | 1.510456531 | PPARG | . |
| 0 | 0 | 1.927155923 | 1.510456531 | SPHK2 | . |
| 0 | 0 | 1.927155923 | 1.510456531 | PARK7 | . |

| | | | | | |
|---|---|---|---|---|---|
| 0 | 0 | 1.927155923 | 1.510456531 | HAVCR2 | . |
| 0 | 0 | 1.927155923 | 1.510456531 | ITGB3 | . |
| 0 | 0 | 1.927155923 | 1.510456531 | C1QC | . |
| 0 | 0 | 1.927155923 | 1.510456531 | CD84 | . |
| 0 | 0 | 1.927155923 | 1.510456531 | TIMP2 | . |
| 0 | 0 | 1.927155923 | 1.510456531 | PARK7 | . |
| 0 | 0 | 1.927155923 | 1.510456531 | PPARG | . |
| 0 | 0 | 1.927155923 | 1.510456531 | PARK7 | . |
| 0 | 0 | 1.927155923 | 1.510456531 | NENF | . |
| 0 | 0 | 1.927155923 | 1.510456531 | SOD1 | . |
| 0 | 0 | 1.927155923 | 1.510456531 | PPARG | . |
| 0 | 0 | 1.927155923 | 1.510456531 | PKD2 | . |
| 0 | 0 | 1.927155923 | 1.510456531 | SLC29A1 | . |
| 0 | 0 | 1.927155923 | 1.510456531 | DCTPP1 | . |
| 0 | 0 | 1.927155923 | 1.510456531 | MDH1 | . |
| 0 | 0 | 1.927155923 | 1.510456531 | SLC25A14 | . |
| 0 | 0 | 1.927155923 | 1.510456531 | XYLB | . |
| 0 | 0 | 1.927155923 | 1.510456531 | LOXL3 | . |
| 0 | 0 | 1.927155923 | 1.510456531 | HABP4 | . |
| 0 | 0 | 1.927155923 | 1.510456531 | NUCKS1 | . |
| 0 | 0 | 1.927155923 | 1.510456531 | TLE5 | . |
| 0 | 0 | 1.927155923 | 1.510456531 | SIRT2 | . |
| 0 | 0 | 1.927155923 | 1.510456531 | MEGF8 | . |
| 0 | 0 | 1.927155923 | 1.510456531 | NPHP4 | . |
| 0 | 0 | 1.927155923 | 1.510456531 | ATP2B4 | . |
| 0 | 0 | 1.927155923 | 1.510456531 | LHFPL2 | . |
| 0 | 0 | 1.927155923 | 1.510456531 | DSTYK | . |
| 0 | 0 | 1.927155923 | 1.510456531 | SLC27A1 | . |
| 0 | 0 | 1.927155923 | 1.510456531 | SPHK2 | . |
| 0 | 0 | 1.927155923 | 1.510456531 | ST3GAL4 | . |
| 0 | 0 | 1.927155923 | 1.510456531 | SLF1 | . |
| 0 | 0 | 1.927155923 | 1.510456531 | SLF1 | . |
| 0 | 0 | 1.927155923 | 1.510456531 | SPHK2 | . |
| 0 | 0 | 1.927155923 | 1.510456531 | VEGFB | . |
| 0 | 0 | 1.927155923 | 1.510456531 | MLLT11 | . |
| 0 | 0 | 1.927155923 | 1.510456531 | SEPTIN9 | . |
| 0 | 0 | 1.927155923 | 1.510456531 | CITED2 | . |
| 0 | 0 | 1.927155923 | 1.510456531 | RNF128 | . |
| 0 | 0 | 1.927155923 | 1.510456531 | EPB41 | . |
| 0 | 0 | 1.927155923 | 1.510456531 | FANCM | . |
| 0 | 0 | 1.927155923 | 1.510456531 | SLC9A1 | . |
| 0 | 0 | 1.927155923 | 1.510456531 | DNM1 | . |
| 0 | 0 | 1.927155923 | 1.510456531 | DNM1 | . |
| 0 | 0 | 1.927155923 | 1.510456531 | DPY19L1 | . |
| 0 | 0 | 1.927155923 | 1.510456531 | DPY19L1 | . |
| 0 | 0 | 1.927155923 | 1.510456531 | PADI6 | . |
| 0 | 0 | 1.927155923 | 1.510456531 | EDEM2 | . |

| | | | | | |
|---|---|---|---|---|---|
| 0 | 0 | 1.927155923 | 1.510456531 | UBA5 | . |
| 0 | 0 | 1.927155923 | 1.510456531 | CNTNAP1 | . |
| 0 | 0 | 1.927155923 | 1.510456531 | MAPRE3 | . |
| 0 | 0 | 1.927155923 | 1.510456531 | MAPRE3 | . |
| 0 | 0 | 1.927155923 | 1.510456531 | SLF1 | . |
| 0 | 0 | 1.927155923 | 1.510456531 | UBA5 | . |
| 0 | 0 | 1.927155923 | 1.510456531 | ST3GAL4 | . |
| 0 | 0 | 1.927155923 | 1.510456531 | AGTPBP1 | . |
| 0 | 0 | 1.927155923 | 1.510456531 | DCTD | . |
| 0 | 0 | 1.927155923 | 1.510456531 | DHODH | . |
| 0 | 0 | 1.927155923 | 1.510456531 | SLC29A1 | . |
| 0 | 0 | 1.927155923 | 1.510456531 | DCTD | . |
| 0 | 0 | 1.927155923 | 1.510456531 | GAS6 | . |
| 0 | 0 | 1.927155923 | 1.510456531 | TREM2 | . |
| 0 | 0 | 1.927155923 | 1.510456531 | UNC119 | . |
| 0 | 0 | 1.927155923 | 1.510456531 | SLC25A23 | . |
| 0 | 0 | 1.927155923 | 1.510456531 | DNAJB2 | . |
| 0 | 0 | 1.927155923 | 1.510456531 | SPHK2 | . |
| 0 | 0 | 1.927155923 | 1.510456531 | FOXJ1 | . |
| 0 | 0 | 1.927155923 | 1.510456531 | PDGFB | . |
| 0 | 0 | 1.927155923 | 1.510456531 | HAVCR2 | . |
| 0 | 0 | 1.927155923 | 1.510456531 | TREM2 | . |
| 0 | 0 | 1.927155923 | 1.510456531 | AUTS2 | . |
| 0 | 0 | 1.927155923 | 1.510456531 | ZNF205 | . |
| 0 | 0 | 1.927155923 | 1.510456531 | TREM2 | . |
| 0 | 0 | 1.927155923 | 1.510456531 | ABCA2 | . |
| 0 | 0 | 1.927155923 | 1.510456531 | SLF1 | . |
| 0 | 0 | 1.927155923 | 1.510456531 | TTL | . |
| 0 | 0 | 1.927155923 | 1.510456531 | STAP1 | . |
| 0 | 0 | 1.927155923 | 1.510456531 | PLD1 | . |
| 0 | 0 | 1.927155923 | 1.510456531 | SLC25A4 | . |
| 0 | 0 | 1.927155923 | 1.510456531 | RTN4 | . |
| 0 | 0 | 1.927155923 | 1.510456531 | KLHL41 | . |
| 0 | 0 | 1.927155923 | 1.510456531 | GAS6 | . |
| 0 | 0 | 1.927155923 | 1.510456531 | GRN | . |
| 0 | 0 | 1.927155923 | 1.510456531 | PDGFB | . |
| 0 | 0 | 1.927155923 | 1.510456531 | MTMR1 | . |
| 0 | 0 | 1.927155923 | 1.510456531 | ABI3 | . |
| 0 | 0 | 1.927155923 | 1.510456531 | FANCM | . |
| 0 | 0 | 1.927155923 | 1.510456531 | GRN | . |
| 0 | 0 | 1.927155923 | 1.510456531 | CC2D1A | . |
| 0 | 0 | 1.927155923 | 1.510456531 | APOA4 | . |
| 0 | 0 | 1.927155923 | 1.510456531 | CDK5 | . |
| 0 | 0 | 1.927155923 | 1.510456531 | FITM2 | . |
| 0 | 0 | 1.927155923 | 1.510456531 | WFS1 | . |
| 0 | 0 | 1.927155923 | 1.510456531 | PKD2 | . |
| 0 | 0 | 1.927155923 | 1.510456531 | MTRES1 | . |

| | | | | | |
|---|---|---|---|---|---|
| 0 | 0 | 1.927155923 | 1.510456531 | ATP2B4 | . |
| 0 | 0 | 1.927155923 | 1.510456531 | HCN2 | . |
| 0 | 0 | 1.927155923 | 1.510456531 | ACTN3 | . |
| 0 | 0 | 1.927155923 | 1.510456531 | PDE1B | . |
| 0 | 0 | 1.927155923 | 1.510456531 | ATG14 | . |
| 0 | 0 | 1.927155923 | 1.510456531 | PRKCG | . |
| 0 | 0 | 1.927155923 | 1.510456531 | CCL19 | . |
| 0 | 0 | 1.927155923 | 1.510456531 | NUDT14 | . |
| 0 | 0 | 1.927155923 | 1.510456531 | XPO1 | . |
| 0 | 0 | 1.927155923 | 1.510456531 | GPC1 | . |
| 0 | 0 | 1.927155923 | 1.510456531 | PKD2L1 | . |
| 0 | 0 | 1.927155923 | 1.510456531 | CUX2 | . |
| 0 | 0 | 1.927155923 | 1.510456531 | SLC17A5 | . |
| 0 | 0 | 1.927155923 | 1.510456531 | ETHE1 | . |
| 0 | 0 | 1.927155923 | 1.510456531 | ETHE1 | . |
| 0 | 0 | 1.927155923 | 1.510456531 | CLDN12 | . |
| 0 | 0 | 1.927155923 | 1.510456531 | PELI3 | . |
| 0 | 0 | 1.927155923 | 1.510456531 | TST | . |
| 0 | 0 | 1.927155923 | 1.510456531 | ZFPM1 | . |
| 0 | 0 | 1.927155923 | 1.510456531 | SFRP4 | . |
| 0 | 0 | 1.927155923 | 1.510456531 | PLK4 | . |
| 0 | 0 | 1.927155923 | 1.510456531 | AFMID | . |
| 0 | 0 | 1.927155923 | 1.510456531 | EDEM2 | . |
| 0 | 0 | 1.927155923 | 1.510456531 | SLC29A1 | . |
| 0 | 0 | 1.927155923 | 1.510456531 | CLEC14A | . |
| 0 | 0 | 1.927155923 | 1.510456531 | BET1 | . |
| 0 | 0 | 1.927155923 | 1.510456531 | WDR11 | . |
| 0 | 0 | 1.927155923 | 1.510456531 | CYP27A1 | . |
| 0 | 0 | 1.186017646 | 0.927547368 | UGDH;CHP | . |
| 0 | 0 | 1.186017646 | 0.927547368 | ZW10;SPD | . |
| 0 | 0 | 1.186017646 | 0.927547368 | HSD17B14 | . |
| 0 | 0 | 1.186017646 | 0.927547368 | WNK1;WN | . |
| 0 | 0 | 1.186017646 | 0.927547368 | AHI1;KDM | . |
| 0 | 0 | 1.186017646 | 0.927547368 | TUT4;EXOS | . |
| 0 | 0 | 1.186017646 | 0.927547368 | SASH3;VA | . |
| 0 | 0 | 1.186017646 | 0.927547368 | MRNIP;TFF | . |
| 0 | 0 | 1.128189841 | 0.881739136 | TNFSF18;L | . |
| 0 | 0 | 1.051358251 | 0.818309341 | CADM1;CR | . |
| 0 | 0 | 1.073479739 | 0.830026256 | EPM2A;SC | . |
| 0 | 0 | 1.073479739 | 0.830026256 | MED25;ZN | . |
| 0 | 0 | 1.073479739 | 0.830026256 | UGDH;TM | . |
| 0 | 0 | 1.028065516 | 0.791535159 | CD86;GST | . |
| 0 | 0 | 1.084127786 | 0.832127832 | DNAJB2;FC | . |
| 0 | 0 | 1.058179382 | 0.811331636 | FARP2;SLF | . |
| 0 | 0 | 1.101310044 | 0.838901476 | RBL2;PIDD | . |
| 0 | 0 | 1.101310044 | 0.838901476 | PPP3CC;YV | . |
| 0 | 0 | 1.101310044 | 0.838901476 | NDC1;SPHI | . |

| | | | | |
|---|---|---|---|---|
| 0 | 0 | 1.133674674 | 0.857123308 | BACE1;APH |
| 0 | 0 | 1.133674674 | 0.857123308 | MTMR1;M |
| 0 | 0 | 1.133674674 | 0.857123308 | RAP2A;RTN |
| 0 | 0 | 1.133674674 | 0.857123308 | MYBPC3;R |
| 0 | 0 | 1.133674674 | 0.857123308 | DLG4;PDGI |
| 0 | 0 | 1.133674674 | 0.857123308 | PPT1;CCL1 |
| 0 | 0 | 1.070725842 | 0.806581144 | VAV3;APH1 |
| 0 | 0 | 1.070725842 | 0.806581144 | DNMT1;TA |
| 0 | 0 | 1.217179828 | 0.915802641 | SFXN3;SLC |
| 0 | 0 | 1.217179828 | 0.915802641 | SCIN;PARV |
| 0 | 0 | 1.217179828 | 0.915802641 | BBS12;NDU |
| 0 | 0 | 1.217179828 | 0.915802641 | OAT;ALDH5 |
| 0 | 0 | 1.217179828 | 0.915802641 | FBXW4;IQ( |
| 0 | 0 | 1.217179828 | 0.915802641 | BSCL2;CIDI |
| 0 | 0 | 1.217179828 | 0.915802641 | ARV1;SPHK |
| 0 | 0 | 1.217179828 | 0.915802641 | APH1B;SPF |
| 0 | 0 | 1.217179828 | 0.915802641 | NFU1;NUB |
| 0 | 0 | 1.217179828 | 0.915802641 | GRN;TREM |
| 0 | 0 | 1.217179828 | 0.915802641 | ABCA2;AC/ |
| 0 | 0 | 1.217179828 | 0.915802641 | LGALS3;GP |
| 0 | 0 | 1.217179828 | 0.915802641 | SCN11A;K( |
| 0 | 0 | 1.217179828 | 0.915802641 | FARP2;GLC |
| 0 | 0 | 1.217179828 | 0.915802641 | GEN1;CEP1 |
| 0 | 0 | 1.217179828 | 0.915802641 | C1QTNF1;F |
| 0 | 0 | 1.217179828 | 0.915802641 | ABCD4;MN |
| 0 | 0 | 1.217179828 | 0.915802641 | TRPM2;SL( |
| 0 | 0 | 1.101265823 | 0.81133549 | TTC8;CUX2 |
| 0 | 0 | 1.101265823 | 0.81133549 | PHF20;HAT |
| 0 | 0 | 1.101265823 | 0.81133549 | TOM1L2;B |
| 0 | 0 | 1.101265823 | 0.81133549 | GSTP1;CID |
| 0 | 0 | 1.142026457 | 0.829461229 | SEMA3A;SI |
| 0 | 0 | 1.142026457 | 0.829461229 | USP14;GIP |
| 0 | 0 | 1.142026457 | 0.829461229 | DSCC1;HAT |
| 0 | 0 | 1.142026457 | 0.829461229 | CLIP3;ZDHI |
| 0 | 0 | 1.043905976 | 0.751469915 | PRKCG;PSN |
| 0 | 0 | 1.284749455 | 0.917493292 | ASPH;LGM |
| 0 | 0 | 1.284749455 | 0.917493292 | SLC25A12; |
| 0 | 0 | 1.284749455 | 0.917493292 | SLC31A1;A |
| 0 | 0 | 1.284749455 | 0.917493292 | ABCA2;PLE |
| 0 | 0 | 1.284749455 | 0.917493292 | CD36;PDE9 |
| 0 | 0 | 1.284749455 | 0.917493292 | SLC46A1;C |
| 0 | 0 | 1.284749455 | 0.917493292 | MPO;PLA2 |
| 0 | 0 | 1.284749455 | 0.917493292 | PROCR;AD |
| 0 | 0 | 1.284749455 | 0.917493292 | EDNRB;PAI |
| 0 | 0 | 1.284749455 | 0.917493292 | GPC1;APLN |
| 0 | 0 | 1.284749455 | 0.917493292 | EIF2AK4;P/ |
| 0 | 0 | 1.284749455 | 0.917493292 | ACHE;ALDH |

| | | | | |
|---|---|---|---|---|
| 0 | 0 | 1.284749455 | 0.917493292 | PRKCA;VRK |
| 0 | 0 | 1.284749455 | 0.917493292 | TNFSF4;NK |
| 0 | 0 | 1.284749455 | 0.917493292 | EIF2AK4;LC |
| 0 | 0 | 1.284749455 | 0.917493292 | FASTKD2;R |
| 0 | 0 | 1.284749455 | 0.917493292 | GSTP1;SOD |
| 0 | 0 | 1.284749455 | 0.917493292 | CLIP3;P2RX |
| 0 | 0 | 1.284749455 | 0.917493292 | UBA5;ATG |
| 0 | 0 | 1.284749455 | 0.917493292 | HPF1;MET |
| 0 | 0 | 1.284749455 | 0.917493292 | TWIST1;TY |
| 0 | 0 | 1.284749455 | 0.917493292 | MIF;TAF9B |
| 0 | 0 | 1.284749455 | 0.917493292 | SPHK2;GPD |
| 0 | 0 | 1.284749455 | 0.917493292 | ZFHX2;SCN |
| 0 | 0 | 1.284749455 | 0.917493292 | SYDE1;ACV |
| 0 | 0 | 1.284749455 | 0.917493292 | WNK4;MA |
| 0 | 0 | 1.284749455 | 0.917493292 | HMGB1;ZF |
| 0 | 0 | 1.284749455 | 0.917493292 | ELP1;ELP4 |
| 0 | 0 | 1.046855254 | 0.74293065 | RBL2;PSMI |
| 0 | 0 | 1.063256052 | 0.753459297 | MMP14;CT |
| 0 | 0 | 1.063256052 | 0.753459297 | MMP14;CT |
| 0 | 0 | 1.063256052 | 0.753459297 | PSMD10;P |
| 0 | 0 | 1.063256052 | 0.753459297 | PARP1;MM |
| 0 | 0 | 1.10122164 | 0.779908383 | MAPKBP1; |
| 0 | 0 | 1.10122164 | 0.779908383 | SLF1;MRNI |
| 0 | 0 | 1.10122164 | 0.779908383 | WNK1;WN |
| 0 | 0 | 1.039352888 | 0.727925178 | LATS1;LGA |
| 0 | 0 | 1.012239311 | 0.706516007 | STX12;IPO |
| 0 | 0 | 1.075594084 | 0.746817942 | CASP8AP2; |
| 0 | 0 | 1.075594084 | 0.746817942 | PRKCG;BSC |
| 0 | 0 | 1.075594084 | 0.746817942 | KANK1;GO |
| 0 | 0 | 1.075594084 | 0.746817942 | CBR1;DCXF |
| 0 | 0 | 1.075594084 | 0.746817942 | CALCRL;TF |
| 0 | 0 | 1.075594084 | 0.746817942 | RNASEH2C |
| 0 | 0 | 1.156255449 | 0.79739882 | TEKT3;DRC |
| 0 | 0 | 1.156255449 | 0.79739882 | GPI;SHPK;F |
| 0 | 0 | 1.156255449 | 0.79739882 | LGALS3;TIF |
| 0 | 0 | 1.156255449 | 0.79739882 | SUFU;IFT1 |
| 0 | 0 | 1.156255449 | 0.79739882 | FUT8;B3G |
| 0 | 0 | 1.156255449 | 0.79739882 | EEPD1;TRE |
| 0 | 0 | 1.156255449 | 0.79739882 | SFRP4;PDC |
| 0 | 0 | 1.156255449 | 0.79739882 | CADM1;CR |
| 0 | 0 | 1.156255449 | 0.79739882 | CD19;LPXN |
| 0 | 0 | 1.156255449 | 0.79739882 | CSTB;SVBP |
| 0 | 0 | 1.156255449 | 0.79739882 | FABP3;FAB |
| 0 | 0 | 1.058001541 | 0.722610839 | FUT8;STIL; |
| 0 | 0 | 1.058001541 | 0.722610839 | SMTN;MEF |
| 0 | 0 | 1.058001541 | 0.722610839 | LSM10;RAI |
| 0 | 0 | 1.058001541 | 0.722610839 | CD84;EPB4 |

| | | | | | |
|---|---|---|---|---|---|
| 0 | 0 | 1.058001541 | 0.722610839 | CLIP3;SPPL | . |
| 0 | 0 | 1.101177497 | 0.742765066 | MMP14;IT | . |
| 0 | 0 | 1.101177497 | 0.742765066 | ZC3H7B;AC | . |
| 0 | 0 | 1.101177497 | 0.742765066 | KCNIP2;SL | . |
| 0 | 0 | 1.101177497 | 0.742765066 | STN1;TEN1 | . |
| 0 | 0 | 1.101177497 | 0.742765066 | PDK4;CTNS | . |
| 0 | 0 | 1.101177497 | 0.742765066 | USP14;PD | . |
| 0 | 0 | 1.035408621 | 0.688522662 | IPO11;LRS | . |
| 0 | 0 | 1.035408621 | 0.688522662 | PLK4;KLHL | . |
| 0 | 0 | 1.070571553 | 0.71011486 | PI4K2B;AL | . |
| 0 | 0 | 1.070571553 | 0.71011486 | KANK1;PK | . |
| 0 | 0 | 1.070571553 | 0.71011486 | ACVR1C;P | . |
| 0 | 0 | 1.004149009 | 0.662339308 | PSMD10;U | . |
| 0 | 0 | 1.018016678 | 0.670305833 | CNTNAP1; | . |
| 0 | 0 | 1.541637631 | 1.010803816 | DLD | . |
| 0 | 0 | 1.541637631 | 1.010803816 | SLC27A1 | . |
| 0 | 0 | 1.541637631 | 1.010803816 | COQ3 | . |
| 0 | 0 | 1.541637631 | 1.010803816 | SLC22A5 | . |
| 0 | 0 | 1.541637631 | 1.010803816 | SCARB2 | . |
| 0 | 0 | 1.541637631 | 1.010803816 | FZD5 | . |
| 0 | 0 | 1.541637631 | 1.010803816 | SLC36A4 | . |
| 0 | 0 | 1.541637631 | 1.010803816 | ASPA | . |
| 0 | 0 | 1.541637631 | 1.010803816 | GAS6 | . |
| 0 | 0 | 1.541637631 | 1.010803816 | C1QC | . |
| 0 | 0 | 1.541637631 | 1.010803816 | RBBP7 | . |
| 0 | 0 | 1.541637631 | 1.010803816 | CD36 | . |
| 0 | 0 | 1.541637631 | 1.010803816 | EIF2AK4 | . |
| 0 | 0 | 1.541637631 | 1.010803816 | PDE1B | . |
| 0 | 0 | 1.541637631 | 1.010803816 | TFR2 | . |
| 0 | 0 | 1.541637631 | 1.010803816 | SLC41A1 | . |
| 0 | 0 | 1.541637631 | 1.010803816 | FITM2 | . |
| 0 | 0 | 1.541637631 | 1.010803816 | ALDH7A1 | . |
| 0 | 0 | 1.541637631 | 1.010803816 | SLC2A8 | . |
| 0 | 0 | 1.541637631 | 1.010803816 | AUTS2 | . |
| 0 | 0 | 1.541637631 | 1.010803816 | CHST14 | . |
| 0 | 0 | 1.541637631 | 1.010803816 | SERINC5 | . |
| 0 | 0 | 1.541637631 | 1.010803816 | PKD2 | . |
| 0 | 0 | 1.541637631 | 1.010803816 | ERCC8 | . |
| 0 | 0 | 1.541637631 | 1.010803816 | KDM2B | . |
| 0 | 0 | 1.541637631 | 1.010803816 | KDM2B | . |
| 0 | 0 | 1.541637631 | 1.010803816 | CEP290 | . |
| 0 | 0 | 1.541637631 | 1.010803816 | PPARG | . |
| 0 | 0 | 1.541637631 | 1.010803816 | B3GALT2 | . |
| 0 | 0 | 1.541637631 | 1.010803816 | B3GALT2 | . |
| 0 | 0 | 1.541637631 | 1.010803816 | SLC6A12 | . |
| 0 | 0 | 1.541637631 | 1.010803816 | TEK | . |
| 0 | 0 | 1.541637631 | 1.010803816 | GPAM | . |

| | | | | | |
|---|---|---|---|---|---|
| 0 | 0 | 1.541637631 | 1.010803816 | NPC2 | . |
| 0 | 0 | 1.541637631 | 1.010803816 | GLA | . |
| 0 | 0 | 1.541637631 | 1.010803816 | GLA | . |
| 0 | 0 | 1.541637631 | 1.010803816 | LTBP4 | . |
| 0 | 0 | 1.541637631 | 1.010803816 | SLC19A1 | . |
| 0 | 0 | 1.541637631 | 1.010803816 | RBBP7 | . |
| 0 | 0 | 1.541637631 | 1.010803816 | HS2ST1 | . |
| 0 | 0 | 1.541637631 | 1.010803816 | SLC5A2 | . |
| 0 | 0 | 1.541637631 | 1.010803816 | ETHE1 | . |
| 0 | 0 | 1.541637631 | 1.010803816 | PFAS | . |
| 0 | 0 | 1.541637631 | 1.010803816 | SLC25A15 | . |
| 0 | 0 | 1.541637631 | 1.010803816 | SERINC5 | . |
| 0 | 0 | 1.541637631 | 1.010803816 | ALOX5 | . |
| 0 | 0 | 1.541637631 | 1.010803816 | ALOX5 | . |
| 0 | 0 | 1.541637631 | 1.010803816 | COL5A1 | . |
| 0 | 0 | 1.541637631 | 1.010803816 | PPARG | . |
| 0 | 0 | 1.541637631 | 1.010803816 | SLC41A1 | . |
| 0 | 0 | 1.541637631 | 1.010803816 | DNMT1 | . |
| 0 | 0 | 1.541637631 | 1.010803816 | MSH6 | . |
| 0 | 0 | 1.541637631 | 1.010803816 | OXSM | . |
| 0 | 0 | 1.541637631 | 1.010803816 | ANKRD31 | . |
| 0 | 0 | 1.541637631 | 1.010803816 | CDC23 | . |
| 0 | 0 | 1.541637631 | 1.010803816 | CDC23 | . |
| 0 | 0 | 1.541637631 | 1.010803816 | FASTKD2 | . |
| 0 | 0 | 1.541637631 | 1.010803816 | PSIP1 | . |
| 0 | 0 | 1.541637631 | 1.010803816 | TCTA | . |
| 0 | 0 | 1.541637631 | 1.010803816 | NAA16 | . |
| 0 | 0 | 1.541637631 | 1.010803816 | SCIN | . |
| 0 | 0 | 1.541637631 | 1.010803816 | ARL6IP5 | . |
| 0 | 0 | 1.541637631 | 1.010803816 | SFRP4 | . |
| 0 | 0 | 1.541637631 | 1.010803816 | PICK1 | . |
| 0 | 0 | 1.541637631 | 1.010803816 | TREM2 | . |
| 0 | 0 | 1.541637631 | 1.010803816 | LPXN | . |
| 0 | 0 | 1.541637631 | 1.010803816 | RNF113A | . |
| 0 | 0 | 1.541637631 | 1.010803816 | ZFYVE19 | . |
| 0 | 0 | 1.541637631 | 1.010803816 | ATP2A1 | . |
| 0 | 0 | 1.541637631 | 1.010803816 | USP14 | . |
| 0 | 0 | 1.541637631 | 1.010803816 | DICER1 | . |
| 0 | 0 | 1.541637631 | 1.010803816 | PASK | . |
| 0 | 0 | 1.541637631 | 1.010803816 | ANGPTL4 | . |
| 0 | 0 | 1.541637631 | 1.010803816 | CD84 | . |
| 0 | 0 | 1.541637631 | 1.010803816 | NBL1 | . |
| 0 | 0 | 1.541637631 | 1.010803816 | CD84 | . |
| 0 | 0 | 1.541637631 | 1.010803816 | FOXJ1 | . |
| 0 | 0 | 1.541637631 | 1.010803816 | TREM2 | . |
| 0 | 0 | 1.541637631 | 1.010803816 | ACVR1C | . |
| 0 | 0 | 1.541637631 | 1.010803816 | AHI1 | . |

| | | | | | |
|---|---|---|---|---|---|
| 0 | 0 | 1.541637631 | 1.010803816 | EXOSC9 | . |
| 0 | 0 | 1.541637631 | 1.010803816 | EXOSC9 | . |
| 0 | 0 | 1.541637631 | 1.010803816 | EXOSC9 | . |
| 0 | 0 | 1.541637631 | 1.010803816 | ABCC5 | . |
| 0 | 0 | 1.541637631 | 1.010803816 | SHPK | . |
| 0 | 0 | 1.541637631 | 1.010803816 | DPH6 | . |
| 0 | 0 | 1.541637631 | 1.010803816 | DPH6 | . |
| 0 | 0 | 1.541637631 | 1.010803816 | PPARG | . |
| 0 | 0 | 1.541637631 | 1.010803816 | AHI1 | . |
| 0 | 0 | 1.541637631 | 1.010803816 | MINPP1 | . |
| 0 | 0 | 1.541637631 | 1.010803816 | CCL19 | . |
| 0 | 0 | 1.541637631 | 1.010803816 | TOMM7 | . |
| 0 | 0 | 1.541637631 | 1.010803816 | PLK4 | . |
| 0 | 0 | 1.541637631 | 1.010803816 | MSR1 | . |
| 0 | 0 | 1.541637631 | 1.010803816 | CCL19 | . |
| 0 | 0 | 1.541637631 | 1.010803816 | BOK | . |
| 0 | 0 | 1.541637631 | 1.010803816 | DDB1 | . |
| 0 | 0 | 1.541637631 | 1.010803816 | CITED2 | . |
| 0 | 0 | 1.541637631 | 1.010803816 | RPS6KA5 | . |
| 0 | 0 | 1.541637631 | 1.010803816 | CITED2 | . |
| 0 | 0 | 1.541637631 | 1.010803816 | TREM2 | . |
| 0 | 0 | 1.541637631 | 1.010803816 | CRTAM | . |
| 0 | 0 | 1.541637631 | 1.010803816 | CRTAM | . |
| 0 | 0 | 1.541637631 | 1.010803816 | STAP1 | . |
| 0 | 0 | 1.541637631 | 1.010803816 | PLGRKT | . |
| 0 | 0 | 1.541637631 | 1.010803816 | MMP9 | . |
| 0 | 0 | 1.541637631 | 1.010803816 | CLU | . |
| 0 | 0 | 1.541637631 | 1.010803816 | DAB2 | . |
| 0 | 0 | 1.541637631 | 1.010803816 | SLC36A4 | . |
| 0 | 0 | 1.541637631 | 1.010803816 | CEP290 | . |
| 0 | 0 | 1.541637631 | 1.010803816 | INTS13 | . |
| 0 | 0 | 1.541637631 | 1.010803816 | WRN | . |
| 0 | 0 | 1.541637631 | 1.010803816 | TPST1 | . |
| 0 | 0 | 1.541637631 | 1.010803816 | UBA5 | . |
| 0 | 0 | 1.541637631 | 1.010803816 | TRAIP | . |
| 0 | 0 | 1.541637631 | 1.010803816 | VMA21 | . |
| 0 | 0 | 1.541637631 | 1.010803816 | SLC35A1 | . |
| 0 | 0 | 1.541637631 | 1.010803816 | RAB11FIP5 | . |
| 0 | 0 | 1.541637631 | 1.010803816 | ATP2B4 | . |
| 0 | 0 | 1.541637631 | 1.010803816 | EMILIN1 | . |
| 0 | 0 | 1.541637631 | 1.010803816 | EPB41 | . |
| 0 | 0 | 1.541637631 | 1.010803816 | ABI3 | . |
| 0 | 0 | 1.541637631 | 1.010803816 | PASK | . |
| 0 | 0 | 1.541637631 | 1.010803816 | PDGFB | . |
| 0 | 0 | 1.541637631 | 1.010803816 | TNFSF4 | . |
| 0 | 0 | 1.541637631 | 1.010803816 | VRK2 | . |
| 0 | 0 | 1.541637631 | 1.010803816 | SFRP4 | . |

| | | | | | |
|---|---|---|---|---|---|
| 0 | 0 | 1.541637631 | 1.010803816 | VEGFB | . |
| 0 | 0 | 1.541637631 | 1.010803816 | CDK5RAP2 | . |
| 0 | 0 | 1.541637631 | 1.010803816 | CDK5RAP2 | . |
| 0 | 0 | 1.541637631 | 1.010803816 | ATP2B4 | . |
| 0 | 0 | 1.541637631 | 1.010803816 | SEPTIN9 | . |
| 0 | 0 | 1.541637631 | 1.010803816 | NEIL1 | . |
| 0 | 0 | 1.541637631 | 1.010803816 | SASH1 | . |
| 0 | 0 | 1.541637631 | 1.010803816 | TREM2 | . |
| 0 | 0 | 1.541637631 | 1.010803816 | ARFIP1 | . |
| 0 | 0 | 1.541637631 | 1.010803816 | LOXL3 | . |
| 0 | 0 | 1.541637631 | 1.010803816 | TNFSF4 | . |
| 0 | 0 | 1.541637631 | 1.010803816 | GPD1L | . |
| 0 | 0 | 1.541637631 | 1.010803816 | XRCC1 | . |
| 0 | 0 | 1.541637631 | 1.010803816 | GNG2 | . |
| 0 | 0 | 1.541637631 | 1.010803816 | SLX4 | . |
| 0 | 0 | 1.541637631 | 1.010803816 | PDE1B | . |
| 0 | 0 | 1.541637631 | 1.010803816 | SPP1 | . |
| 0 | 0 | 1.541637631 | 1.010803816 | ST3GAL4 | . |
| 0 | 0 | 1.541637631 | 1.010803816 | MAPK8IP2 | . |
| 0 | 0 | 1.541637631 | 1.010803816 | METTL4 | . |
| 0 | 0 | 1.541637631 | 1.010803816 | ZC4H2 | . |
| 0 | 0 | 1.541637631 | 1.010803816 | SLC25A14 | . |
| 0 | 0 | 1.541637631 | 1.010803816 | FER1L5 | . |
| 0 | 0 | 1.541637631 | 1.010803816 | FZD5 | . |
| 0 | 0 | 1.541637631 | 1.010803816 | EXOSC9 | . |
| 0 | 0 | 1.541637631 | 1.010803816 | ABCG2 | . |
| 0 | 0 | 1.051089242 | 0.68764791 | SLC38A7;S | . |
| 0 | 0 | 1.051089242 | 0.68764791 | CARMIL1;C | . |
| 0 | 0 | 1.185855539 | 0.758620729 | FHOD3;OB | . |
| 0 | 0 | 1.185855539 | 0.758620729 | BCAS3;SFR | . |
| 0 | 0 | 1.185855539 | 0.758620729 | FAM171A1 | . |
| 0 | 0 | 1.185855539 | 0.758620729 | MTHFD1;T | . |
| 0 | 0 | 1.185855539 | 0.758620729 | RTN4;LNPK | . |
| 0 | 0 | 1.185855539 | 0.758620729 | RAP2A;FOX | . |
| 0 | 0 | 1.185855539 | 0.758620729 | RPGRIP1L;( | . |
| 0 | 0 | 1.185855539 | 0.758620729 | ABHD10;U | . |
| 0 | 0 | 1.185855539 | 0.758620729 | SMARCAD1 | . |
| 0 | 0 | 1.185855539 | 0.758620729 | ERI1;TUT4 | . |
| 0 | 0 | 1.185855539 | 0.758620729 | IMPACT;EII | . |
| 0 | 0 | 1.185855539 | 0.758620729 | PON2;ALO) | . |
| 0 | 0 | 1.185855539 | 0.758620729 | PARP1;MN | . |
| 0 | 0 | 1.185855539 | 0.758620729 | LGALS3;ED | . |
| 0 | 0 | 1.185855539 | 0.758620729 | NAXE;PPT1 | . |
| 0 | 0 | 1.185855539 | 0.758620729 | MDC1;CHE | . |
| 0 | 0 | 1.185855539 | 0.758620729 | IGF1;HINFF | . |
| 0 | 0 | 1.185855539 | 0.758620729 | SCPEP1;AP | . |
| 0 | 0 | 1.185855539 | 0.758620729 | CCDC125;C | . |

| | | | | |
|---|---|---|---|---|
| 0 | 0 | 1.185855539 | 0.758620729 | ACP4;GAS1… |
| 0 | 0 | 1.185855539 | 0.758620729 | ENTPD5;EN… |
| 0 | 0 | 1.185855539 | 0.758620729 | CREB3L3;P… |
| 0 | 0 | 1.185855539 | 0.758620729 | MRNIP;PAI… |
| 0 | 0 | 1.185855539 | 0.758620729 | TICAM1;GA… |
| 0 | 0 | 1.185855539 | 0.758620729 | P2RX3;MIF… |
| 0 | 0 | 1.185855539 | 0.758620729 | P2RX3;MIF… |
| 0 | 0 | 1.185855539 | 0.758620729 | NT5M;DHC… |
| 0 | 0 | 1.185855539 | 0.758620729 | ACTN3;SHN… |
| 0 | 0 | 1.185855539 | 0.758620729 | TREM2;CLU… |
| 0 | 0 | 1.185855539 | 0.758620729 | MTMR1;PF… |
| 0 | 0 | 1.185855539 | 0.758620729 | FARP2;KIF1… |
| 0 | 0 | 1.185855539 | 0.758620729 | PRKCA;C1C… |
| 0 | 0 | 1.185855539 | 0.758620729 | ESR1;RASG… |
| 0 | 0 | 1.185855539 | 0.758620729 | GSTP1;SOD… |
| 0 | 0 | 1.185855539 | 0.758620729 | SLC27A1;F… |
| 0 | 0 | 1.185855539 | 0.758620729 | ESR1;PLPP… |
| 0 | 0 | 1.185855539 | 0.758620729 | FAM171A1… |
| 0 | 0 | 1.185855539 | 0.758620729 | NOXA1;DU… |
| 0 | 0 | 1.185855539 | 0.758620729 | POP7;POP4… |
| 0 | 0 | 1.020124177 | 0.646352366 | IPO11;NXT… |
| 0 | 0 | 1.020124177 | 0.646352366 | TRPM2;SLC… |
| 0 | 0 | 1.020124177 | 0.646352366 | ITGB3;TNF… |
| 0 | 0 | 1.101133391 | 0.696290759 | AUTS2;CDK… |
| 0 | 0 | 1.101133391 | 0.696290759 | FUT8;B3GA… |
| 0 | 0 | 1.101133391 | 0.696290759 | AIFM1;HAT… |
| 0 | 0 | 1.101133391 | 0.696290759 | ZW10;SPDL… |
| 0 | 0 | 1.101133391 | 0.696290759 | NFU1;NUB… |
| 0 | 0 | 1.101133391 | 0.696290759 | INTS14;ERI… |
| 0 | 0 | 1.101133391 | 0.696290759 | DAB2;CITE… |
| 0 | 0 | 1.101133391 | 0.696290759 | BACE1;GRN… |
| 0 | 0 | 1.101133391 | 0.696290759 | DAB2;CITE… |
| 0 | 0 | 1.101133391 | 0.696290759 | SEMA3A;SI… |
| 0 | 0 | 1.101133391 | 0.696290759 | LATS1;LTBI… |
| 0 | 0 | 1.101133391 | 0.696290759 | TMEM204;… |
| 0 | 0 | 1.101133391 | 0.696290759 | WRN;XRCC… |
| 0 | 0 | 1.014141716 | 0.636916996 | EPS8;KANK… |
| 0 | 0 | 1.063145706 | 0.665954638 | EPS8;CALD… |
| 0 | 0 | 1.063145706 | 0.665954638 | EXOSC9;LS… |
| 0 | 0 | 1.063145706 | 0.665954638 | GSTP1;DUS… |
| 0 | 0 | 1.063145706 | 0.665954638 | PDCD4;PPA… |
| 0 | 0 | 1.063145706 | 0.665954638 | EEPD1;ABC… |
| 0 | 0 | 1.063145706 | 0.665954638 | RPS6KA5;C… |
| 0 | 0 | 1.041578227 | 0.646949844 | TENM4;CA… |
| 0 | 0 | 1.041578227 | 0.646949844 | RARS2;LAR… |
| 0 | 0 | 1.041578227 | 0.646949844 | PRKCG;TIM… |
| 0 | 0 | 1.027673505 | 0.633342874 | PLCL1;PIK3… |

| | | | | |
|---|---|---|---|---|
| 0 | 0 | 1.027673505 | 0.633342874 | KIF18A;CD[illegible] |
| 0 | 0 | 1.027673505 | 0.633342874 | SCAMP5;L[illegible] |
| 0 | 0 | 1.005282761 | 0.60673554 | SPARC;NA[illegible] |
| 0 | 0 | 0.994413882 | 0.598134077 | NTNG2;CN[illegible] |
| 0 | 0 | 1.000914258 | 0.600284844 | ALDH3A2;[illegible] |
| 0 | 0 | 0.994423661 | 0.58930128 | SH3RF3;M[illegible] |
| 0 | 0 | 1.027649368 | 0.597755643 | UBA5;MTI[illegible] |
| 0 | 0 | 1.027649368 | 0.597755643 | PIK3R2;IGF[illegible] |
| 0 | 0 | 1.027649368 | 0.597755643 | ABCA2;IGF[illegible] |
| 0 | 0 | 1.027649368 | 0.597755643 | ABCA2;CA[illegible] |
| 0 | 0 | 1.014110866 | 0.589717499 | SLCO2B1;S[illegible] |
| 0 | 0 | 1.014110866 | 0.589717499 | DGKA;ALO[illegible] |
| 0 | 0 | 1.014110866 | 0.589717499 | SHTN1;GO[illegible] |
| 0 | 0 | 1.014110866 | 0.589717499 | CADM1;CR[illegible] |
| 0 | 0 | 1.014110866 | 0.589717499 | PDGFB;TB[illegible] |
| 0 | 0 | 1.014110866 | 0.589717499 | ALOX5;PD[illegible] |
| 0 | 0 | 1.014110866 | 0.589717499 | NDC1;NUP[illegible] |
| 0 | 0 | 1.014110866 | 0.589717499 | YWHAQ;G[illegible] |
| 0 | 0 | 1.005275843 | 0.58346964 | ABCA2;AC[illegible] |
| 0 | 0 | 1.005275843 | 0.58346964 | PTPMT1;S[illegible] |
| 0 | 0 | 1.05102243 | 0.609552821 | WNK1;WN[illegible] |
| 0 | 0 | 1.05102243 | 0.609552821 | OSBPL5;N[illegible] |
| 0 | 0 | 1.05102243 | 0.609552821 | MTMR1;M[illegible] |
| 0 | 0 | 1.05102243 | 0.609552821 | MLLT11;F[illegible] |
| 0 | 0 | 1.05102243 | 0.609552821 | RNF43;MV[illegible] |
| 0 | 0 | 1.05102243 | 0.609552821 | ACP4;GAS[illegible] |
| 0 | 0 | 1.05102243 | 0.609552821 | LATS1;USP[illegible] |
| 0 | 0 | 1.05102243 | 0.609552821 | GSTO1;PK[illegible] |
| 0 | 0 | 0.999053857 | 0.578236806 | GALNT12;[illegible] |
| 0 | 0 | 0.999053857 | 0.578236806 | CTSK;HMG[illegible] |
| 0 | 0 | 0.990865385 | 0.569513054 | SHTN1;NT[illegible] |
| 0 | 0 | 0.990865385 | 0.569513054 | PSMD10;D[illegible] |
| 0 | 0 | 0.990865385 | 0.569513054 | PSMD10;D[illegible] |
| 0 | 0 | 0.990865385 | 0.569513054 | NDC1;RPA[illegible] |
| 0 | 0 | 0.990865385 | 0.569513054 | IMPACT;B[illegible] |
| 0 | 0 | 1.101089325 | 0.631940501 | MSH6;CD1[illegible] |
| 0 | 0 | 1.101089325 | 0.631940501 | NTNG2;NI[illegible] |
| 0 | 0 | 1.101089325 | 0.631940501 | GRAMD1B[illegible] |
| 0 | 0 | 1.101089325 | 0.631940501 | DLG4;DIP2[illegible] |
| 0 | 0 | 1.101089325 | 0.631940501 | ST3GAL5;L[illegible] |
| 0 | 0 | 1.101089325 | 0.631940501 | SPHK2;MS[illegible] |
| 0 | 0 | 1.101089325 | 0.631940501 | CRELD1;PK[illegible] |
| 0 | 0 | 1.101089325 | 0.631940501 | SEMA3A;C[illegible] |
| 0 | 0 | 1.101089325 | 0.631940501 | BCAS3;CO[illegible] |
| 0 | 0 | 1.101089325 | 0.631940501 | TSPYL2;G[illegible] |
| 0 | 0 | 1.101089325 | 0.631940501 | BCAS3;CO[illegible] |
| 0 | 0 | 1.101089325 | 0.631940501 | SPHK2;TW[illegible] |

| | | | | | |
|---|---|---|---|---|---|
| 0 | 0 | 1.101089325 | 0.631940501 | EDNRB;PAI | . |
| 0 | 0 | 1.101089325 | 0.631940501 | PICK1;LGM | . |
| 0 | 0 | 1.101089325 | 0.631940501 | CNTNAP1;I | . |
| 0 | 0 | 1.101089325 | 0.631940501 | PDGFD;PD | . |
| 0 | 0 | 1.101089325 | 0.631940501 | TFAP4;NU | . |
| 0 | 0 | 1.101089325 | 0.631940501 | TFRC;SHLD | . |
| 0 | 0 | 1.101089325 | 0.631940501 | ALOX5;ST3 | . |
| 0 | 0 | 1.101089325 | 0.631940501 | TREM2;RA | . |
| 0 | 0 | 1.101089325 | 0.631940501 | TREM2;CL | . |
| 0 | 0 | 1.101089325 | 0.631940501 | H1-10;ASF | . |
| 0 | 0 | 1.101089325 | 0.631940501 | ELMOD2;Z | . |
| 0 | 0 | 1.101089325 | 0.631940501 | CD84;RAS | . |
| 0 | 0 | 1.101089325 | 0.631940501 | HMGB1;G | . |
| 0 | 0 | 1.101089325 | 0.631940501 | SPHK2;TNF | . |
| 0 | 0 | 1.101089325 | 0.631940501 | TMEM161 | . |
| 0 | 0 | 1.101089325 | 0.631940501 | TFRC;SHLD | . |
| 0 | 0 | 1.101089325 | 0.631940501 | SHPK;MIF | . |
| 0 | 0 | 1.101089325 | 0.631940501 | CD84;SPH | . |
| 0 | 0 | 1.101089325 | 0.631940501 | CAMK2A;K | . |
| 0 | 0 | 1.101089325 | 0.631940501 | ACTN3;SH | . |
| 0 | 0 | 1.101089325 | 0.631940501 | AUTS2;ALS | . |
| 0 | 0 | 1.101089325 | 0.631940501 | MGARP;SC | . |
| 0 | 0 | 1.101089325 | 0.631940501 | SLC1A3;AB | . |
| 0 | 0 | 0.985711815 | 0.562154101 | BSCL2;ACH | . |
| 0 | 0 | 0.98010506 | 0.545350294 | PSMD10;T | . |
| 0 | 0 | 1.284625436 | 0.712361076 | PNPLA2 | . |
| 0 | 0 | 1.284625436 | 0.712361076 | GNG2 | . |
| 0 | 0 | 1.284625436 | 0.712361076 | CAMK2A | . |
| 0 | 0 | 1.284625436 | 0.712361076 | OAT | . |
| 0 | 0 | 1.284625436 | 0.712361076 | SLC25A12 | . |
| 0 | 0 | 1.284625436 | 0.712361076 | ZFPM1 | . |
| 0 | 0 | 1.284625436 | 0.712361076 | CNTNAP1 | . |
| 0 | 0 | 1.284625436 | 0.712361076 | CALCRL | . |
| 0 | 0 | 1.284625436 | 0.712361076 | ATP2B4 | . |
| 0 | 0 | 1.284625436 | 0.712361076 | COMT | . |
| 0 | 0 | 1.284625436 | 0.712361076 | MAPK15 | . |
| 0 | 0 | 1.284625436 | 0.712361076 | SHTN1 | . |
| 0 | 0 | 1.284625436 | 0.712361076 | ILVBL | . |
| 0 | 0 | 1.284625436 | 0.712361076 | APEH | . |
| 0 | 0 | 1.284625436 | 0.712361076 | MAP2K5 | . |
| 0 | 0 | 1.284625436 | 0.712361076 | GNG2 | . |
| 0 | 0 | 1.284625436 | 0.712361076 | MTSS2 | . |
| 0 | 0 | 1.284625436 | 0.712361076 | DDAH2 | . |
| 0 | 0 | 1.284625436 | 0.712361076 | UBASH3B | . |
| 0 | 0 | 1.284625436 | 0.712361076 | APLN | . |
| 0 | 0 | 1.284625436 | 0.712361076 | GATM | . |
| 0 | 0 | 1.284625436 | 0.712361076 | COX15 | . |

| | | | | | |
|---|---|---|---|---|---|
| 0 | 0 | 1.284625436 | 0.712361076 | TEK | . |
| 0 | 0 | 1.284625436 | 0.712361076 | SEMA3A | . |
| 0 | 0 | 1.284625436 | 0.712361076 | TANC2 | . |
| 0 | 0 | 1.284625436 | 0.712361076 | PRKCA | . |
| 0 | 0 | 1.284625436 | 0.712361076 | MEGF8 | . |
| 0 | 0 | 1.284625436 | 0.712361076 | LIG1 | . |
| 0 | 0 | 1.284625436 | 0.712361076 | DOLPP1 | . |
| 0 | 0 | 1.284625436 | 0.712361076 | DOLPP1 | . |
| 0 | 0 | 1.284625436 | 0.712361076 | MAPK15 | . |
| 0 | 0 | 1.284625436 | 0.712361076 | MVB12A | . |
| 0 | 0 | 1.284625436 | 0.712361076 | SULT1C4 | . |
| 0 | 0 | 1.284625436 | 0.712361076 | GRIN2C | . |
| 0 | 0 | 1.284625436 | 0.712361076 | CD9 | . |
| 0 | 0 | 1.284625436 | 0.712361076 | MYO1E | . |
| 0 | 0 | 1.284625436 | 0.712361076 | SLC38A6 | . |
| 0 | 0 | 1.284625436 | 0.712361076 | B3GALT2 | . |
| 0 | 0 | 1.284625436 | 0.712361076 | VRK1 | . |
| 0 | 0 | 1.284625436 | 0.712361076 | TEK | . |
| 0 | 0 | 1.284625436 | 0.712361076 | SETD4 | . |
| 0 | 0 | 1.284625436 | 0.712361076 | SUV39H1 | . |
| 0 | 0 | 1.284625436 | 0.712361076 | ABCC5 | . |
| 0 | 0 | 1.284625436 | 0.712361076 | RCSD1 | . |
| 0 | 0 | 1.284625436 | 0.712361076 | CAB39 | . |
| 0 | 0 | 1.284625436 | 0.712361076 | RAB11FIP5 | . |
| 0 | 0 | 1.284625436 | 0.712361076 | PLEKHA8 | . |
| 0 | 0 | 1.284625436 | 0.712361076 | SLC48A1 | . |
| 0 | 0 | 1.284625436 | 0.712361076 | TFR2 | . |
| 0 | 0 | 1.284625436 | 0.712361076 | SLC36A4 | . |
| 0 | 0 | 1.284625436 | 0.712361076 | ALOX5 | . |
| 0 | 0 | 1.284625436 | 0.712361076 | PICK1 | . |
| 0 | 0 | 1.284625436 | 0.712361076 | CARMIL1 | . |
| 0 | 0 | 1.284625436 | 0.712361076 | NBL1 | . |
| 0 | 0 | 1.284625436 | 0.712361076 | MDH1 | . |
| 0 | 0 | 1.284625436 | 0.712361076 | CLIP3 | . |
| 0 | 0 | 1.284625436 | 0.712361076 | PKD2 | . |
| 0 | 0 | 1.284625436 | 0.712361076 | TUT4 | . |
| 0 | 0 | 1.284625436 | 0.712361076 | PSEN2 | . |
| 0 | 0 | 1.284625436 | 0.712361076 | MRC1 | . |
| 0 | 0 | 1.284625436 | 0.712361076 | EIF2AK4 | . |
| 0 | 0 | 1.284625436 | 0.712361076 | GSTP1 | . |
| 0 | 0 | 1.284625436 | 0.712361076 | TWIST1 | . |
| 0 | 0 | 1.284625436 | 0.712361076 | NUDT16L1 | . |
| 0 | 0 | 1.284625436 | 0.712361076 | PASK | . |
| 0 | 0 | 1.284625436 | 0.712361076 | PPARG | . |
| 0 | 0 | 1.284625436 | 0.712361076 | RAD1 | . |
| 0 | 0 | 1.284625436 | 0.712361076 | TIMP2 | . |
| 0 | 0 | 1.284625436 | 0.712361076 | TMEM14A | . |

| | | | | | |
|---|---|---|---|---|---|
| 0 | 0 | 1.284625436 | 0.712361076 | TICAM1 | . |
| 0 | 0 | 1.284625436 | 0.712361076 | TREM2 | . |
| 0 | 0 | 1.284625436 | 0.712361076 | TREM2 | . |
| 0 | 0 | 1.284625436 | 0.712361076 | CORO1C | . |
| 0 | 0 | 1.284625436 | 0.712361076 | PARK7 | . |
| 0 | 0 | 1.284625436 | 0.712361076 | PPARG | . |
| 0 | 0 | 1.284625436 | 0.712361076 | ABCA2 | . |
| 0 | 0 | 1.284625436 | 0.712361076 | GSTP1 | . |
| 0 | 0 | 1.284625436 | 0.712361076 | SIRT2 | . |
| 0 | 0 | 1.284625436 | 0.712361076 | RAD50 | . |
| 0 | 0 | 1.284625436 | 0.712361076 | ACVR1C | . |
| 0 | 0 | 1.284625436 | 0.712361076 | PNRC2 | . |
| 0 | 0 | 1.284625436 | 0.712361076 | DPYSL2 | . |
| 0 | 0 | 1.284625436 | 0.712361076 | FOLR1 | . |
| 0 | 0 | 1.284625436 | 0.712361076 | SRM | . |
| 0 | 0 | 1.284625436 | 0.712361076 | EIF2AK4 | . |
| 0 | 0 | 1.284625436 | 0.712361076 | TREM2 | . |
| 0 | 0 | 1.284625436 | 0.712361076 | STAP1 | . |
| 0 | 0 | 1.284625436 | 0.712361076 | APLN | . |
| 0 | 0 | 1.284625436 | 0.712361076 | HMGB1 | . |
| 0 | 0 | 1.284625436 | 0.712361076 | TACR2 | . |
| 0 | 0 | 1.284625436 | 0.712361076 | TACR2 | . |
| 0 | 0 | 1.284625436 | 0.712361076 | UBQLN2 | . |
| 0 | 0 | 1.284625436 | 0.712361076 | PRDM11 | . |
| 0 | 0 | 1.284625436 | 0.712361076 | TACR2 | . |
| 0 | 0 | 1.284625436 | 0.712361076 | TENM4 | . |
| 0 | 0 | 1.284625436 | 0.712361076 | AUTS2 | . |
| 0 | 0 | 1.284625436 | 0.712361076 | PLA2R1 | . |
| 0 | 0 | 1.284625436 | 0.712361076 | CORO1C | . |
| 0 | 0 | 1.284625436 | 0.712361076 | SPHK2 | . |
| 0 | 0 | 1.284625436 | 0.712361076 | MLLT11 | . |
| 0 | 0 | 1.284625436 | 0.712361076 | CKAP5 | . |
| 0 | 0 | 1.284625436 | 0.712361076 | FABP3 | . |
| 0 | 0 | 1.284625436 | 0.712361076 | PPT1 | . |
| 0 | 0 | 1.284625436 | 0.712361076 | CEP250 | . |
| 0 | 0 | 1.284625436 | 0.712361076 | ABCA2 | . |
| 0 | 0 | 1.284625436 | 0.712361076 | ESR1 | . |
| 0 | 0 | 1.284625436 | 0.712361076 | SYDE1 | . |
| 0 | 0 | 1.284625436 | 0.712361076 | DOC2B | . |
| 0 | 0 | 1.284625436 | 0.712361076 | NIPSNAP2 | . |
| 0 | 0 | 1.284625436 | 0.712361076 | YBX1 | . |
| 0 | 0 | 1.284625436 | 0.712361076 | SLC9A1 | . |
| 0 | 0 | 1.284625436 | 0.712361076 | CFAP206 | . |
| 0 | 0 | 1.284625436 | 0.712361076 | FUCA2 | . |
| 0 | 0 | 1.284625436 | 0.712361076 | EMILIN1 | . |
| 0 | 0 | 1.284625436 | 0.712361076 | SLC45A3 | . |
| 0 | 0 | 1.284625436 | 0.712361076 | SPHK2 | . |

| | | | | | |
|---|---|---|---|---|---|
| 0 | 0 | 1.284625436 | 0.712361076 | SIRT5 | . |
| 0 | 0 | 1.284625436 | 0.712361076 | CITED2 | . |
| 0 | 0 | 1.284625436 | 0.712361076 | TREM2 | . |
| 0 | 0 | 1.284625436 | 0.712361076 | PARK7 | . |
| 0 | 0 | 1.284625436 | 0.712361076 | CRTAM | . |
| 0 | 0 | 1.284625436 | 0.712361076 | STAP1 | . |
| 0 | 0 | 1.284625436 | 0.712361076 | PRAP1 | . |
| 0 | 0 | 1.284625436 | 0.712361076 | KCNIP2 | . |
| 0 | 0 | 1.284625436 | 0.712361076 | HMGB1 | . |
| 0 | 0 | 1.284625436 | 0.712361076 | ITGB8 | . |
| 0 | 0 | 1.284625436 | 0.712361076 | CDH3 | . |
| 0 | 0 | 1.284625436 | 0.712361076 | ALS2 | . |
| 0 | 0 | 1.284625436 | 0.712361076 | TRPV1 | . |
| 0 | 0 | 1.284625436 | 0.712361076 | MRC1 | . |
| 0 | 0 | 1.284625436 | 0.712361076 | ATP6V1A | . |
| 0 | 0 | 1.284625436 | 0.712361076 | PPP2CB | . |
| 0 | 0 | 1.284625436 | 0.712361076 | RPGRIP1L | . |
| 0 | 0 | 1.284625436 | 0.712361076 | XPO1 | . |
| 0 | 0 | 1.284625436 | 0.712361076 | XPO1 | . |
| 0 | 0 | 1.284625436 | 0.712361076 | XPO1 | . |
| 0 | 0 | 1.284625436 | 0.712361076 | EEFSEC | . |
| 0 | 0 | 1.284625436 | 0.712361076 | ITGB3 | . |
| 0 | 0 | 1.284625436 | 0.712361076 | MSH6 | . |
| 0 | 0 | 1.284625436 | 0.712361076 | SHTN1 | . |
| 0 | 0 | 1.284625436 | 0.712361076 | SLC25A14 | . |
| 0 | 0 | 1.284625436 | 0.712361076 | DNM1 | . |
| 0 | 0 | 1.284625436 | 0.712361076 | TACR2 | . |
| 0 | 0 | 1.284625436 | 0.712361076 | EEFSEC | . |
| 0 | 0 | 1.284625436 | 0.712361076 | ALDH1A2 | . |
| 0 | 0 | 1.284625436 | 0.712361076 | PPARG | . |
| 0 | 0 | 0.978593272 | 0.535847829 | PLCL1;PIK3 | . |
| 0 | 0 | 0.976854132 | 0.534536591 | TSPYL2;LA | . |
| 0 | 0 | 0.976854132 | 0.534536591 | TNFSF18;P | . |
| 0 | 0 | 0.983831273 | 0.53763854 | EPM2A;LR | . |
| 0 | 0 | 0.97546396 | 0.533063535 | GPI;BCAS3 | . |
| 0 | 0 | 0.973377865 | 0.529818389 | CPNE9;TRF | . |
| 0 | 0 | 0.988052088 | 0.537805345 | DYNC2I1;N | . |
| 0 | 0 | 0.988052088 | 0.537805345 | AIFM1;PEX | . |
| 0 | 0 | 0.988052088 | 0.537805345 | AGO4;TUT | . |
| 0 | 0 | 0.973237317 | 0.527247642 | RALBP1;TF | . |
| 0 | 0 | 0.971883842 | 0.526357985 | NDC1;GLM | . |
| 0 | 0 | 0.994443116 | 0.537083219 | SCPEP1;ED | . |
| 0 | 0 | 0.994443116 | 0.537083219 | DYNC2I1;H | . |
| 0 | 0 | 0.994443116 | 0.537083219 | CHRNB1;TI | . |
| 0 | 0 | 0.994443116 | 0.537083219 | EXOSC9;LS | . |
| 0 | 0 | 0.994443116 | 0.537083219 | SLC27A1;F | . |
| 0 | 0 | 0.994443116 | 0.537083219 | GSTP1;DUS | . |

| | | | | |
|---|---|---|---|---|
| 0 | 0 | 0.994443116 | 0.537083219 | STAR;PDK4 |
| 0 | 0 | 0.994443116 | 0.537083219 | BCAS3;SFR |
| 0 | 0 | 0.994443116 | 0.537083219 | FARP2;SEM |
| 0 | 0 | 1.005268944 | 0.534834729 | DAB2;ABI3 |
| 0 | 0 | 1.005268944 | 0.534834729 | CNRIP1;CB |
| 0 | 0 | 1.005268944 | 0.534834729 | PLA2G12A |
| 0 | 0 | 1.005268944 | 0.534834729 | DNMT1;AU |
| 0 | 0 | 1.005268944 | 0.534834729 | WNK1;WN |
| 0 | 0 | 1.005268944 | 0.534834729 | TOGARAM |
| 0 | 0 | 1.005268944 | 0.534834729 | ALMS1;AS |
| 0 | 0 | 1.005268944 | 0.534834729 | ANKH;TWI |
| 0 | 0 | 1.005268944 | 0.534834729 | PPP1R3C;I |
| 0 | 0 | 1.005268944 | 0.534834729 | RTEL1;RAD |
| 0 | 0 | 1.005268944 | 0.534834729 | INTS14;INT |
| 0 | 0 | 1.005268944 | 0.534834729 | CCDC88A; |
| 0 | 0 | 0.963199913 | 0.502412336 | SH3RF3;M |
| 0 | 0 | 0.969637174 | 0.503432895 | DNMT1;CI |
| 0 | 0 | 0.963264192 | 0.498581929 | MCFD2;TR |
| 0 | 0 | 0.963264192 | 0.498581929 | DDB1;MDF |
| 0 | 0 | 0.963264192 | 0.498581929 | MCFD2;TR |
| 0 | 0 | 0.963264192 | 0.498581929 | MCFD2;TR |
| 0 | 0 | 1.027625272 | 0.529775422 | HOGA1;GC |
| 0 | 0 | 1.027625272 | 0.529775422 | PDK4;AKR1 |
| 0 | 0 | 1.027625272 | 0.529775422 | CEP250;CA |
| 0 | 0 | 1.027625272 | 0.529775422 | RILPL1;CCD |
| 0 | 0 | 1.027625272 | 0.529775422 | DMAC2L;A |
| 0 | 0 | 1.027625272 | 0.529775422 | ACTN3;PAI |
| 0 | 0 | 1.027625272 | 0.529775422 | GSTP1;MA |
| 0 | 0 | 1.027625272 | 0.529775422 | DFFA;CIDE |
| 0 | 0 | 1.027625272 | 0.529775422 | MIF;TAF9B |
| 0 | 0 | 1.027625272 | 0.529775422 | FER1L5;HM |
| 0 | 0 | 1.027625272 | 0.529775422 | SLC37A4;S |
| 0 | 0 | 1.027625272 | 0.529775422 | CPNE9;SYT |
| 0 | 0 | 1.027625272 | 0.529775422 | STN1;ATAD |
| 0 | 0 | 1.027625272 | 0.529775422 | STAP1;TRE |
| 0 | 0 | 1.027625272 | 0.529775422 | SCAMP5;D |
| 0 | 0 | 1.027625272 | 0.529775422 | PDGFB;IGF |
| 0 | 0 | 1.027625272 | 0.529775422 | DPYSL2;NT |
| 0 | 0 | 1.027625272 | 0.529775422 | PICK1;ARFI |
| 0 | 0 | 1.027625272 | 0.529775422 | GSTO1;CLI |
| 0 | 0 | 1.027625272 | 0.529775422 | ACTN3;SHI |
| 0 | 0 | 1.027625272 | 0.529775422 | TNFSF18;A |
| 0 | 0 | 1.027625272 | 0.529775422 | EPB41;CAM |
| 0 | 0 | 1.027625272 | 0.529775422 | TREM2;CD |
| 0 | 0 | 1.027625272 | 0.529775422 | XRCC5;RPS |
| 0 | 0 | 1.027625272 | 0.529775422 | INTS3;INTS |
| 0 | 0 | 1.027625272 | 0.529775422 | SYNGR1;SY |

| | | | | |
|---|---|---|---|---|
| 0 | 0 | 0.963280227 | 0.495498396 | SLC26A1;R |
| 0 | 0 | 0.963280227 | 0.495498396 | BCAS3;TEK |
| 0 | 0 | 0.963280227 | 0.495498396 | CD63;GNG |
| 0 | 0 | 0.963280227 | 0.495498396 | CSTB;ASPH |
| 0 | 0 | 0.963038225 | 0.494381967 | VAV3;BCAS |
| 0 | 0 | 0.962989441 | 0.490709205 | SPARC;ITG |
| 0 | 0 | 0.963296248 | 0.490672766 | PDGFB;TRE |
| 0 | 0 | 0.963296248 | 0.490672766 | KANK1;GKA |
| 0 | 0 | 0.963296248 | 0.490672766 | MMP14;AS |
| 0 | 0 | 0.963312255 | 0.483009524 | SPAST;KIF3 |
| 0 | 0 | 0.963312255 | 0.483009524 | GLP1R;CD3 |
| 0 | 0 | 0.963312255 | 0.483009524 | DLG4;TME |
| 0 | 0 | 0.963312255 | 0.483009524 | FBXW4;TW |
| 0 | 0 | 0.963312255 | 0.483009524 | MMP14;IT |
| 0 | 0 | 0.963312255 | 0.483009524 | MAP10;HD |
| 0 | 0 | 0.963312255 | 0.483009524 | UBASH3B; |
| 0 | 0 | 0.951270466 | 0.473159077 | EPS8;TRPM |
| 0 | 0 | 0.949983236 | 0.470777542 | PSMD10;FA |
| 0 | 0 | 0.95794515 | 0.473297063 | SPARC;ITG |
| 0 | 0 | 0.948375172 | 0.467500842 | PSMD10;PS |
| 0 | 0 | 0.948375172 | 0.467500842 | ATP6V1A;F |
| 0 | 0 | 0.948375172 | 0.467500842 | SH3RF3;MA |
| 0 | 0 | 0.946311193 | 0.462993934 | ARL4D;NE |
| 0 | 0 | 0.963328248 | 0.470231733 | SCIN;SYT2; |
| 0 | 0 | 0.963328248 | 0.470231733 | UGDH;CHP |
| 0 | 0 | 0.963328248 | 0.470231733 | FBXL8;CRT |
| 0 | 0 | 0.963328248 | 0.470231733 | PMEL;HPS4 |
| 0 | 0 | 0.963328248 | 0.470231733 | KIF18A;KIF |
| 0 | 0 | 0.963328248 | 0.470231733 | LTBP4;PAR |
| 0 | 0 | 0.963328248 | 0.470231733 | TWIST1;CC |
| 0 | 0 | 0.963328248 | 0.470231733 | PDGFB;IGF |
| 0 | 0 | 0.963328248 | 0.470231733 | HMGB1;TL |
| 0 | 0 | 0.963328248 | 0.470231733 | XPO1;STRA |
| 0 | 0 | 0.963328248 | 0.470231733 | TREM2;CD |
| 0 | 0 | 0.963328248 | 0.470231733 | TWIST1;PA |
| 0 | 0 | 0.963328248 | 0.470231733 | GPNMB;VA |
| 0 | 0 | 0.943567999 | 0.456732161 | BEX3;HGF; |
| 0 | 0 | 0.943567999 | 0.456732161 | IMMP1L;IN |
| 0 | 0 | 0.943567999 | 0.456732161 | ASPH;TME |
| 0 | 0 | 0.951599814 | 0.457309488 | PSMD10;IN |
| 0 | 0 | 0.964783451 | 0.463571273 | TFRC;CITED |
| 0 | 0 | 0.93974801 | 0.447841318 | ARL4D;NE |
| 0 | 0 | 0.93974801 | 0.447841318 | DNMT1;PD |
| 0 | 0 | 0.93974801 | 0.447841318 | KDM1B;XR |
| 0 | 0 | 0.939616283 | 0.445697707 | SPARC;ITG |
| 0 | 0 | 1.101045296 | 0.520588146 | SLC12A6 |
| 0 | 0 | 1.101045296 | 0.520588146 | MAPK8IP3 |

| | | | | | |
|---|---|---|---|---|---|
| 0 | 0 | 1.101045296 | 0.520588146 | ABCB9 | . |
| 0 | 0 | 1.101045296 | 0.520588146 | TPK1 | . |
| 0 | 0 | 1.101045296 | 0.520588146 | PLEKHM1 | . |
| 0 | 0 | 1.101045296 | 0.520588146 | MAPK8IP3 | . |
| 0 | 0 | 1.101045296 | 0.520588146 | SEMA3A | . |
| 0 | 0 | 1.101045296 | 0.520588146 | SLC3A1 | . |
| 0 | 0 | 1.101045296 | 0.520588146 | FOLR1 | . |
| 0 | 0 | 1.101045296 | 0.520588146 | CAMK2A | . |
| 0 | 0 | 1.101045296 | 0.520588146 | GAS6 | . |
| 0 | 0 | 1.101045296 | 0.520588146 | DLG4 | . |
| 0 | 0 | 1.101045296 | 0.520588146 | MFAP4 | . |
| 0 | 0 | 1.101045296 | 0.520588146 | TST | . |
| 0 | 0 | 1.101045296 | 0.520588146 | PSMD10 | . |
| 0 | 0 | 1.101045296 | 0.520588146 | MAPK15 | . |
| 0 | 0 | 1.101045296 | 0.520588146 | DNM1 | . |
| 0 | 0 | 1.101045296 | 0.520588146 | DNM1 | . |
| 0 | 0 | 1.101045296 | 0.520588146 | RIPOR1 | . |
| 0 | 0 | 1.101045296 | 0.520588146 | PLXNB2 | . |
| 0 | 0 | 1.101045296 | 0.520588146 | GAS6 | . |
| 0 | 0 | 1.101045296 | 0.520588146 | B3GALT2 | . |
| 0 | 0 | 1.101045296 | 0.520588146 | CARNS1 | . |
| 0 | 0 | 1.101045296 | 0.520588146 | CARNS1 | . |
| 0 | 0 | 1.101045296 | 0.520588146 | KDM1B | . |
| 0 | 0 | 1.101045296 | 0.520588146 | SETD4 | . |
| 0 | 0 | 1.101045296 | 0.520588146 | CCL19 | . |
| 0 | 0 | 1.101045296 | 0.520588146 | HAT1 | . |
| 0 | 0 | 1.101045296 | 0.520588146 | PKD2 | . |
| 0 | 0 | 1.101045296 | 0.520588146 | LATS1 | . |
| 0 | 0 | 1.101045296 | 0.520588146 | SGO2 | . |
| 0 | 0 | 1.101045296 | 0.520588146 | GAS6 | . |
| 0 | 0 | 1.101045296 | 0.520588146 | UQCC3 | . |
| 0 | 0 | 1.101045296 | 0.520588146 | ATG14 | . |
| 0 | 0 | 1.101045296 | 0.520588146 | PICK1 | . |
| 0 | 0 | 1.101045296 | 0.520588146 | HMGB1 | . |
| 0 | 0 | 1.101045296 | 0.520588146 | TWSG1 | . |
| 0 | 0 | 1.101045296 | 0.520588146 | ACADVL | . |
| 0 | 0 | 1.101045296 | 0.520588146 | GRN | . |
| 0 | 0 | 1.101045296 | 0.520588146 | ARL6IP5 | . |
| 0 | 0 | 1.101045296 | 0.520588146 | IGF1 | . |
| 0 | 0 | 1.101045296 | 0.520588146 | ABCA2 | . |
| 0 | 0 | 1.101045296 | 0.520588146 | ATRAID | . |
| 0 | 0 | 1.101045296 | 0.520588146 | PLA2R1 | . |
| 0 | 0 | 1.101045296 | 0.520588146 | CAB39 | . |
| 0 | 0 | 1.101045296 | 0.520588146 | LOXL3 | . |
| 0 | 0 | 1.101045296 | 0.520588146 | SEMA3A | . |
| 0 | 0 | 1.101045296 | 0.520588146 | DLG4 | . |
| 0 | 0 | 1.101045296 | 0.520588146 | SLC29A1 | . |

| | | | | | |
|---|---|---|---|---|---|
| 0 | 0 | 1.101045296 | 0.520588146 | ACHE | . |
| 0 | 0 | 1.101045296 | 0.520588146 | ACP4 | . |
| 0 | 0 | 1.101045296 | 0.520588146 | P2RX7 | . |
| 0 | 0 | 1.101045296 | 0.520588146 | TREM2 | . |
| 0 | 0 | 1.101045296 | 0.520588146 | GRN | . |
| 0 | 0 | 1.101045296 | 0.520588146 | TNFSF4 | . |
| 0 | 0 | 1.101045296 | 0.520588146 | GRN | . |
| 0 | 0 | 1.101045296 | 0.520588146 | GAS6 | . |
| 0 | 0 | 1.101045296 | 0.520588146 | TICAM1 | . |
| 0 | 0 | 1.101045296 | 0.520588146 | DAB2 | . |
| 0 | 0 | 1.101045296 | 0.520588146 | CLASP1 | . |
| 0 | 0 | 1.101045296 | 0.520588146 | TREM2 | . |
| 0 | 0 | 1.101045296 | 0.520588146 | APOA4 | . |
| 0 | 0 | 1.101045296 | 0.520588146 | SIRT2 | . |
| 0 | 0 | 1.101045296 | 0.520588146 | TWIST1 | . |
| 0 | 0 | 1.101045296 | 0.520588146 | MMP14 | . |
| 0 | 0 | 1.101045296 | 0.520588146 | TENM4 | . |
| 0 | 0 | 1.101045296 | 0.520588146 | TFR2 | . |
| 0 | 0 | 1.101045296 | 0.520588146 | GSTO1 | . |
| 0 | 0 | 1.101045296 | 0.520588146 | STAR | . |
| 0 | 0 | 1.101045296 | 0.520588146 | DENND1B | . |
| 0 | 0 | 1.101045296 | 0.520588146 | PDGFB | . |
| 0 | 0 | 1.101045296 | 0.520588146 | DLG4 | . |
| 0 | 0 | 1.101045296 | 0.520588146 | PARK7 | . |
| 0 | 0 | 1.101045296 | 0.520588146 | KIF20B | . |
| 0 | 0 | 1.101045296 | 0.520588146 | KIF20B | . |
| 0 | 0 | 1.101045296 | 0.520588146 | MAPK8IP3 | . |
| 0 | 0 | 1.101045296 | 0.520588146 | KDELR3 | . |
| 0 | 0 | 1.101045296 | 0.520588146 | EPS8 | . |
| 0 | 0 | 1.101045296 | 0.520588146 | NKAP | . |
| 0 | 0 | 1.101045296 | 0.520588146 | TNFSF4 | . |
| 0 | 0 | 1.101045296 | 0.520588146 | HMGB1 | . |
| 0 | 0 | 1.101045296 | 0.520588146 | ATP2A1 | . |
| 0 | 0 | 1.101045296 | 0.520588146 | RANGRF | . |
| 0 | 0 | 1.101045296 | 0.520588146 | TWSG1 | . |
| 0 | 0 | 1.101045296 | 0.520588146 | RALBP1 | . |
| 0 | 0 | 1.101045296 | 0.520588146 | RNF113A | . |
| 0 | 0 | 1.101045296 | 0.520588146 | DPH6 | . |
| 0 | 0 | 1.101045296 | 0.520588146 | GAS6 | . |
| 0 | 0 | 1.101045296 | 0.520588146 | PALM | . |
| 0 | 0 | 1.101045296 | 0.520588146 | HMGB1 | . |
| 0 | 0 | 1.101045296 | 0.520588146 | CD84 | . |
| 0 | 0 | 1.101045296 | 0.520588146 | APOA4 | . |
| 0 | 0 | 1.101045296 | 0.520588146 | LGALS3 | . |
| 0 | 0 | 1.101045296 | 0.520588146 | SIRT2 | . |
| 0 | 0 | 1.101045296 | 0.520588146 | CDC23 | . |
| 0 | 0 | 1.101045296 | 0.520588146 | PLD1 | . |

| | | | | | |
|---|---|---|---|---|---|
| 0 | 0 | 1.101045296 | 0.520588146 | TICAM1 | . |
| 0 | 0 | 1.101045296 | 0.520588146 | UBASH3B | . |
| 0 | 0 | 1.101045296 | 0.520588146 | CD36 | . |
| 0 | 0 | 1.101045296 | 0.520588146 | PLA2R1 | . |
| 0 | 0 | 1.101045296 | 0.520588146 | SASH1 | . |
| 0 | 0 | 1.101045296 | 0.520588146 | EPB41 | . |
| 0 | 0 | 1.101045296 | 0.520588146 | SPHK2 | . |
| 0 | 0 | 1.101045296 | 0.520588146 | ALOX5 | . |
| 0 | 0 | 1.101045296 | 0.520588146 | SIRT2 | . |
| 0 | 0 | 1.101045296 | 0.520588146 | GLE1 | . |
| 0 | 0 | 1.101045296 | 0.520588146 | ALOX5 | . |
| 0 | 0 | 1.101045296 | 0.520588146 | CLASP1 | . |
| 0 | 0 | 1.101045296 | 0.520588146 | UQCC3 | . |
| 0 | 0 | 1.101045296 | 0.520588146 | DLG4 | . |
| 0 | 0 | 1.101045296 | 0.520588146 | AGO4 | . |
| 0 | 0 | 1.101045296 | 0.520588146 | METTL15 | . |
| 0 | 0 | 1.101045296 | 0.520588146 | LGMN | . |
| 0 | 0 | 1.101045296 | 0.520588146 | NBL1 | . |
| 0 | 0 | 1.101045296 | 0.520588146 | ICE2 | . |
| 0 | 0 | 1.101045296 | 0.520588146 | CITED2 | . |
| 0 | 0 | 1.101045296 | 0.520588146 | SCAP | . |
| 0 | 0 | 1.101045296 | 0.520588146 | MTHFD1 | . |
| 0 | 0 | 1.101045296 | 0.520588146 | DNM1 | . |
| 0 | 0 | 1.101045296 | 0.520588146 | ZFPM1 | . |
| 0 | 0 | 1.101045296 | 0.520588146 | NPRL3 | . |
| 0 | 0 | 1.101045296 | 0.520588146 | EXOSC9 | . |
| 0 | 0 | 1.101045296 | 0.520588146 | EDNRB | . |
| 0 | 0 | 1.101045296 | 0.520588146 | COL5A1 | . |
| 0 | 0 | 0.937092468 | 0.441745183 | EPS8;P2RX | . |
| 0 | 0 | 0.937092468 | 0.441745183 | PSMA6;GR | . |
| 0 | 0 | 0.937092468 | 0.441745183 | WWTR1;ZH | . |
| 0 | 0 | 0.933952897 | 0.436634668 | CARMIL1;C | . |
| 0 | 0 | 0.934068112 | 0.434737613 | PLEKHM1;T | . |
| 0 | 0 | 0.934068112 | 0.434737613 | TUT4;DROS | . |
| 0 | 0 | 0.934068112 | 0.434737613 | SPARC;ALC | . |
| 0 | 0 | 0.934068112 | 0.434737613 | GSTP1;DUS | . |
| 0 | 0 | 0.934068112 | 0.434737613 | MSR1;CD3 | . |
| 0 | 0 | 0.934068112 | 0.434737613 | FAM210B;S | . |
| 0 | 0 | 0.963344227 | 0.446574026 | NPC2;SLC2 | . |
| 0 | 0 | 0.963344227 | 0.446574026 | HAT1;HMG | . |
| 0 | 0 | 0.963344227 | 0.446574026 | STIL;FZD5 | . |
| 0 | 0 | 0.963344227 | 0.446574026 | EDEM2;ST | . |
| 0 | 0 | 0.963344227 | 0.446574026 | ABCA5;APC | . |
| 0 | 0 | 0.963344227 | 0.446574026 | KLHL24;MT | . |
| 0 | 0 | 0.963344227 | 0.446574026 | MIGA2;MIC | . |
| 0 | 0 | 0.963344227 | 0.446574026 | SHLD2;MA | . |
| 0 | 0 | 0.963344227 | 0.446574026 | MED25;GS | . |

| | | | | |
|---|---|---|---|---|
| 0 | 0 | 0.963344227 | 0.446574026 | MAPKBP1; |
| 0 | 0 | 0.963344227 | 0.446574026 | BSCL2;CIDI |
| 0 | 0 | 0.963344227 | 0.446574026 | KANK1;CCI |
| 0 | 0 | 0.963344227 | 0.446574026 | FARP2;PICI |
| 0 | 0 | 0.963344227 | 0.446574026 | DDAH2;CD |
| 0 | 0 | 0.963344227 | 0.446574026 | LGALS3;GP |
| 0 | 0 | 0.963344227 | 0.446574026 | ABCA2;CLU |
| 0 | 0 | 0.963344227 | 0.446574026 | AKT3;CIB1 |
| 0 | 0 | 0.963344227 | 0.446574026 | CUX2;DLG4 |
| 0 | 0 | 0.963344227 | 0.446574026 | TENM4;DI( |
| 0 | 0 | 0.963344227 | 0.446574026 | MSR1;CD3 |
| 0 | 0 | 0.963344227 | 0.446574026 | TENM4;DI( |
| 0 | 0 | 0.963344227 | 0.446574026 | SEMA4C;A |
| 0 | 0 | 0.963344227 | 0.446574026 | ALKBH7;TI( |
| 0 | 0 | 0.963344227 | 0.446574026 | ATRAID;VG |
| 0 | 0 | 0.963344227 | 0.446574026 | SMC6;RAD |
| 0 | 0 | 0.963344227 | 0.446574026 | CPNE9;SYT |
| 0 | 0 | 0.963344227 | 0.446574026 | RANGRF;G |
| 0 | 0 | 0.963344227 | 0.446574026 | TREM2;SO |
| 0 | 0 | 0.963344227 | 0.446574026 | GLE1;HDA( |
| 0 | 0 | 0.963344227 | 0.446574026 | EXOSC9;DI |
| 0 | 0 | 0.963344227 | 0.446574026 | GPD1L;SNT |
| 0 | 0 | 0.92994278 | 0.429951282 | MBNL2;PC |
| 0 | 0 | 0.92994278 | 0.429951282 | DUS2;DTW |
| 0 | 0 | 0.951860087 | 0.437126072 | SPARC;ITG |
| 0 | 0 | 0.92464426 | 0.421053117 | ACTN3;GST |
| 0 | 0 | 0.931287435 | 0.424046972 | P2RX7;TRP |
| 0 | 0 | 0.928242924 | 0.419773934 | BBS2;OAT; |
| 0 | 0 | 0.924742807 | 0.414187749 | RRAGB;RR/ |
| 0 | 0 | 0.924742807 | 0.414187749 | LSM10;ERI |
| 0 | 0 | 0.924742807 | 0.414187749 | B4GALT2;B |
| 0 | 0 | 0.924742807 | 0.414187749 | RUFY3;KIA |
| 0 | 0 | 0.924742807 | 0.414187749 | PPP1R26;T |
| 0 | 0 | 0.924742807 | 0.414187749 | SLC38A6;C |
| 0 | 0 | 0.924742807 | 0.414187749 | PIK3R2;EF( |
| 0 | 0 | 0.924742807 | 0.414187749 | KANK1;GRI |
| 0 | 0 | 0.924742807 | 0.414187749 | KIF18A;KIF |
| 0 | 0 | 0.924742807 | 0.414187749 | GGA2;PIGU |
| 0 | 0 | 0.924742807 | 0.414187749 | CD19;TNFS |
| 0 | 0 | 0.924742807 | 0.414187749 | P2RX3;RGN |
| 0 | 0 | 0.924742807 | 0.414187749 | MAPRE3;C |
| 0 | 0 | 0.917321117 | 0.408886526 | BRMS1L;SN |
| 0 | 0 | 0.917321117 | 0.408886526 | ACTN3;PLC |
| 0 | 0 | 0.917321117 | 0.408886526 | ABCA2;NP( |
| 0 | 0 | 0.917321117 | 0.408886526 | LATS1;WW |
| 0 | 0 | 0.931854485 | 0.414269825 | SETD4;LRS |
| 0 | 0 | 0.919961138 | 0.407778042 | DAB2;ITGB |

| | | | | |
|---|---|---|---|---|
| 0 | 0 | 0.919961138 | 0.407778042 | CD86;VAV3 |
| 0 | 0 | 0.919961138 | 0.407778042 | PSMD10;P |
| 0 | 0 | 0.914129228 | 0.399264836 | CNTNAP1;( |
| 0 | 0 | 0.914129228 | 0.399264836 | CD86;TFRC |
| 0 | 0 | 0.906544214 | 0.391580232 | PRKCG;GIP |
| 0 | 0 | 0.906544214 | 0.391580232 | PKD2;DUO |
| 0 | 0 | 0.906544214 | 0.391580232 | H2AZ1;FAN |
| 0 | 0 | 0.906544214 | 0.391580232 | SYT2;CDK5 |
| 0 | 0 | 0.906462629 | 0.388201559 | PON2;ALO |
| 0 | 0 | 0.906380901 | 0.380797617 | FHOD3;FAI |
| 0 | 0 | 0.906625657 | 0.378242132 | DMAC2L;A |
| 0 | 0 | 0.906625657 | 0.378242132 | FUCA1;STT |
| 0 | 0 | 0.906625657 | 0.378242132 | ABHD10;U |
| 0 | 0 | 0.906625657 | 0.378242132 | DNMT1;DN |
| 0 | 0 | 0.906625657 | 0.378242132 | DNMT1;DN |
| 0 | 0 | 0.906625657 | 0.378242132 | ACSS1;SUL |
| 0 | 0 | 0.906625657 | 0.378242132 | GRIN2C;M |
| 0 | 0 | 0.906625657 | 0.378242132 | PHF20;MSI |
| 0 | 0 | 0.906625657 | 0.378242132 | PDCD4;TRI |
| 0 | 0 | 0.906625657 | 0.378242132 | SETD4;SUV |
| 0 | 0 | 0.906625657 | 0.378242132 | CCDC88A;S |
| 0 | 0 | 0.906625657 | 0.378242132 | TEK;LIMS1 |
| 0 | 0 | 0.906625657 | 0.378242132 | PARK7;SIR |
| 0 | 0 | 0.906625657 | 0.378242132 | CHRNB1;D |
| 0 | 0 | 0.906625657 | 0.378242132 | GSDMB;TR |
| 0 | 0 | 0.906625657 | 0.378242132 | MST1;MTU |
| 0 | 0 | 0.906625657 | 0.378242132 | CEP97;CCS |
| 0 | 0 | 0.906625657 | 0.378242132 | SLC45A3;T |
| 0 | 0 | 0.906625657 | 0.378242132 | FZD5;TNFS |
| 0 | 0 | 0.906625657 | 0.378242132 | PDK4;CD36 |
| 0 | 0 | 0.906625657 | 0.378242132 | ACHE;CHRI |
| 0 | 0 | 0.906625657 | 0.378242132 | FOXJ1;PKD |
| 0 | 0 | 0.895937335 | 0.37343091 | CNTNAP1;( |
| 0 | 0 | 0.895937335 | 0.37343091 | AKT3;PDGI |
| 0 | 0 | 0.895937335 | 0.37343091 | INF2;FZD5; |
| 0 | 0 | 0.911156267 | 0.378428622 | GLP1R;ALC |
| 0 | 0 | 0.898842795 | 0.370760784 | VAV3;ITGB |
| 0 | 0 | 0.889125478 | 0.365422225 | CDK5;GPC |
| 0 | 0 | 0.889125478 | 0.365422225 | AIFM1;DN/ |
| 0 | 0 | 0.889125478 | 0.365422225 | CD36;TAB1 |
| 0 | 0 | 0.889125478 | 0.365422225 | E2F1;CIRBI |
| 0 | 0 | 0.889125478 | 0.365422225 | ALOX5;AD |
| 0 | 0 | 0.889125478 | 0.365422225 | HGF;PDGFI |
| 0 | 0 | 0.889125478 | 0.365422225 | DDB1;MST |
| 0 | 0 | 0.916958808 | 0.376485978 | ENTPD5;NI |
| 0 | 0 | 0.963360192 | 0.390800524 | CCDC88C |
| 0 | 0 | 0.963360192 | 0.390800524 | MYH7 |

| | | | | | |
|---|---|---|---|---|---|
| 0 | 0 | 0.963360192 | 0.390800524 | SRD5A1 | . |
| 0 | 0 | 0.963360192 | 0.390800524 | LGMN | . |
| 0 | 0 | 0.963360192 | 0.390800524 | CLASP1 | . |
| 0 | 0 | 0.963360192 | 0.390800524 | NTNG2 | . |
| 0 | 0 | 0.963360192 | 0.390800524 | CD86 | . |
| 0 | 0 | 0.963360192 | 0.390800524 | MMP3 | . |
| 0 | 0 | 0.963360192 | 0.390800524 | MMP3 | . |
| 0 | 0 | 0.963360192 | 0.390800524 | SCAP | . |
| 0 | 0 | 0.963360192 | 0.390800524 | CYP27A1 | . |
| 0 | 0 | 0.963360192 | 0.390800524 | APOA4 | . |
| 0 | 0 | 0.963360192 | 0.390800524 | UBASH3B | . |
| 0 | 0 | 0.963360192 | 0.390800524 | C1QC | . |
| 0 | 0 | 0.963360192 | 0.390800524 | LHFPL2 | . |
| 0 | 0 | 0.963360192 | 0.390800524 | HMGB1 | . |
| 0 | 0 | 0.963360192 | 0.390800524 | ZNF358 | . |
| 0 | 0 | 0.963360192 | 0.390800524 | INPPL1 | . |
| 0 | 0 | 0.963360192 | 0.390800524 | ODAD4 | . |
| 0 | 0 | 0.963360192 | 0.390800524 | UGT1A6 | . |
| 0 | 0 | 0.963360192 | 0.390800524 | FUT8 | . |
| 0 | 0 | 0.963360192 | 0.390800524 | HDHD5 | . |
| 0 | 0 | 0.963360192 | 0.390800524 | RPS6KA5 | . |
| 0 | 0 | 0.963360192 | 0.390800524 | ADSS1 | . |
| 0 | 0 | 0.963360192 | 0.390800524 | CD36 | . |
| 0 | 0 | 0.963360192 | 0.390800524 | PDSS2 | . |
| 0 | 0 | 0.963360192 | 0.390800524 | FUT8 | . |
| 0 | 0 | 0.963360192 | 0.390800524 | FUT8 | . |
| 0 | 0 | 0.963360192 | 0.390800524 | ITGB3 | . |
| 0 | 0 | 0.963360192 | 0.390800524 | ZFPM1 | . |
| 0 | 0 | 0.963360192 | 0.390800524 | TWSG1 | . |
| 0 | 0 | 0.963360192 | 0.390800524 | GAS6 | . |
| 0 | 0 | 0.963360192 | 0.390800524 | STXBP6 | . |
| 0 | 0 | 0.963360192 | 0.390800524 | ACOT8 | . |
| 0 | 0 | 0.963360192 | 0.390800524 | CD84 | . |
| 0 | 0 | 0.963360192 | 0.390800524 | GSTP1 | . |
| 0 | 0 | 0.963360192 | 0.390800524 | ANGPTL4 | . |
| 0 | 0 | 0.963360192 | 0.390800524 | ITGB3 | . |
| 0 | 0 | 0.963360192 | 0.390800524 | ARL6IP5 | . |
| 0 | 0 | 0.963360192 | 0.390800524 | SLC25A4 | . |
| 0 | 0 | 0.963360192 | 0.390800524 | EMILIN1 | . |
| 0 | 0 | 0.963360192 | 0.390800524 | HDAC6 | . |
| 0 | 0 | 0.963360192 | 0.390800524 | EXOSC9 | . |
| 0 | 0 | 0.963360192 | 0.390800524 | TST | . |
| 0 | 0 | 0.963360192 | 0.390800524 | ASL | . |
| 0 | 0 | 0.963360192 | 0.390800524 | GTF2H5 | . |
| 0 | 0 | 0.963360192 | 0.390800524 | TREM2 | . |
| 0 | 0 | 0.963360192 | 0.390800524 | LRSAM1 | . |
| 0 | 0 | 0.963360192 | 0.390800524 | MMP14 | . |

| | | | | | |
|---|---|---|---|---|---|
| 0 | 0 | 0.963360192 | 0.390800524 | GLP1R | . |
| 0 | 0 | 0.963360192 | 0.390800524 | APOA4 | . |
| 0 | 0 | 0.963360192 | 0.390800524 | TWIST1 | . |
| 0 | 0 | 0.963360192 | 0.390800524 | SPHK2 | . |
| 0 | 0 | 0.963360192 | 0.390800524 | TNFSF4 | . |
| 0 | 0 | 0.963360192 | 0.390800524 | STPG1 | . |
| 0 | 0 | 0.963360192 | 0.390800524 | CD36 | . |
| 0 | 0 | 0.963360192 | 0.390800524 | MIF | . |
| 0 | 0 | 0.963360192 | 0.390800524 | SPHK2 | . |
| 0 | 0 | 0.963360192 | 0.390800524 | APOA4 | . |
| 0 | 0 | 0.963360192 | 0.390800524 | AKR1C4 | . |
| 0 | 0 | 0.963360192 | 0.390800524 | SLC22A5 | . |
| 0 | 0 | 0.963360192 | 0.390800524 | CITED2 | . |
| 0 | 0 | 0.963360192 | 0.390800524 | CRTAM | . |
| 0 | 0 | 0.963360192 | 0.390800524 | TREM2 | . |
| 0 | 0 | 0.963360192 | 0.390800524 | KRT10 | . |
| 0 | 0 | 0.963360192 | 0.390800524 | PPP1R3C | . |
| 0 | 0 | 0.963360192 | 0.390800524 | ITGB3 | . |
| 0 | 0 | 0.963360192 | 0.390800524 | BOK | . |
| 0 | 0 | 0.963360192 | 0.390800524 | CITED2 | . |
| 0 | 0 | 0.963360192 | 0.390800524 | PPT1 | . |
| 0 | 0 | 0.963360192 | 0.390800524 | NOXA1 | . |
| 0 | 0 | 0.963360192 | 0.390800524 | IGF1 | . |
| 0 | 0 | 0.963360192 | 0.390800524 | ABCA2 | . |
| 0 | 0 | 0.963360192 | 0.390800524 | PARK7 | . |
| 0 | 0 | 0.963360192 | 0.390800524 | DOC2B | . |
| 0 | 0 | 0.963360192 | 0.390800524 | DAB2 | . |
| 0 | 0 | 0.963360192 | 0.390800524 | SLC41A1 | . |
| 0 | 0 | 0.963360192 | 0.390800524 | SHMT2 | . |
| 0 | 0 | 0.963360192 | 0.390800524 | SPP1 | . |
| 0 | 0 | 0.963360192 | 0.390800524 | RPGRIP1L | . |
| 0 | 0 | 0.963360192 | 0.390800524 | PLXNB2 | . |
| 0 | 0 | 0.963360192 | 0.390800524 | COL5A1 | . |
| 0 | 0 | 0.963360192 | 0.390800524 | MSH6 | . |
| 0 | 0 | 0.963360192 | 0.390800524 | CYP27A1 | . |
| 0 | 0 | 0.963360192 | 0.390800524 | SEMA3A | . |
| 0 | 0 | 0.963360192 | 0.390800524 | HMGB1 | . |
| 0 | 0 | 0.963360192 | 0.390800524 | APOA4 | . |
| 0 | 0 | 0.900429116 | 0.364703858 | WFS1;ALO | . |
| 0 | 0 | 0.900429116 | 0.364703858 | PM20D1;G | . |
| 0 | 0 | 0.910320809 | 0.368400768 | GRN;TNFSI | . |
| 0 | 0 | 0.888980291 | 0.357941894 | BSCL2;CLIP | . |
| 0 | 0 | 0.888980291 | 0.357941894 | PPP1R26;B | . |
| 0 | 0 | 0.880593109 | 0.352942518 | GNG2;SPH | . |
| 0 | 0 | 0.880593109 | 0.352942518 | NDUFA11;I | . |
| 0 | 0 | 0.880593109 | 0.352942518 | CD86;GPNI | . |
| 0 | 0 | 0.880593109 | 0.352942518 | UBE3D;FAI | . |

| | | | | |
|---|---|---|---|---|
| 0 | 0 | 0.880593109 | 0.352942518 | LATS1;CCL[illegible] |
| 0 | 0 | 0.880593109 | 0.352942518 | SPHK2;WN[illegible] |
| 0 | 0 | 0.880593109 | 0.352942518 | SPR;DDAH[illegible] |
| 0 | 0 | 0.880593109 | 0.352942518 | MYO1E;M[illegible] |
| 0 | 0 | 0.93325306 | 0.373734635 | INTS13;TP[illegible] |
| 0 | 0 | 0.875525543 | 0.341138041 | CDH3;CAD[illegible] |
| 0 | 0 | 0.875525543 | 0.341138041 | DMAC2L;A[illegible] |
| 0 | 0 | 0.875525543 | 0.341138041 | KIAA1614;[illegible] |
| 0 | 0 | 0.884057556 | 0.344279235 | WWTR1;T[illegible] |
| 0 | 0 | 0.884057556 | 0.344279235 | GLE1;IMP[illegible] |
| 0 | 0 | 0.888834723 | 0.342045833 | SPARC;NA[illegible] |
| 0 | 0 | 0.906052557 | 0.346026353 | FHOD3;CA[illegible] |
| 0 | 0 | 0.913601025 | 0.346358577 | EPM2A;PS[illegible] |
| 0 | 0 | 0.902528621 | 0.34190151 | PSMD10;W[illegible] |
| 0 | 0 | 0.856146469 | 0.322874287 | SCIN;PARV[illegible] |
| 0 | 0 | 0.856146469 | 0.322874287 | CLDN12;W[illegible] |
| 0 | 0 | 0.856146469 | 0.322874287 | KANK1;AR[illegible] |
| 0 | 0 | 0.856146469 | 0.322874287 | GSTP1;IGF[illegible] |
| 0 | 0 | 0.856146469 | 0.322874287 | SETD4;SU[illegible] |
| 0 | 0 | 0.856146469 | 0.322874287 | MSR1;CD3[illegible] |
| 0 | 0 | 0.856146469 | 0.322874287 | PIK3R2;EF[illegible] |
| 0 | 0 | 0.856146469 | 0.322874287 | LRRC59;IG[illegible] |
| 0 | 0 | 0.856146469 | 0.322874287 | CADM1;CR[illegible] |
| 0 | 0 | 0.856146469 | 0.322874287 | ABCA2;CC[illegible] |
| 0 | 0 | 0.856146469 | 0.322874287 | INTS14;EX[illegible] |
| 0 | 0 | 0.880384447 | 0.331978685 | PSMD10;P[illegible] |
| 0 | 0 | 0.880384447 | 0.331978685 | UBL4A;LIP[illegible] |
| 0 | 0 | 0.85620915 | 0.321708026 | STX8;RILP[illegible] |
| 0 | 0 | 0.85620915 | 0.321708026 | WFS1;ATP[illegible] |
| 0 | 0 | 0.85620915 | 0.321708026 | ODAD4;SP[illegible] |
| 0 | 0 | 0.85620915 | 0.321708026 | RAP2A;CL[illegible] |
| 0 | 0 | 0.85620915 | 0.321708026 | UGDH;ME[illegible] |
| 0 | 0 | 0.85620915 | 0.321708026 | PPBP;SLC2[illegible] |
| 0 | 0 | 0.85620915 | 0.321708026 | CDK4;E2F1[illegible] |
| 0 | 0 | 0.85620915 | 0.321708026 | CITED2;PK[illegible] |
| 0 | 0 | 0.85620915 | 0.321708026 | SEMA3A;S[illegible] |
| 0 | 0 | 0.85620915 | 0.321708026 | LATS1;TFA[illegible] |
| 0 | 0 | 0.85620915 | 0.321708026 | CDK5;EIF2[illegible] |
| 0 | 0 | 0.85620915 | 0.321708026 | RALBP1;SL[illegible] |
| 0 | 0 | 0.85620915 | 0.321708026 | HMGB1;IG[illegible] |
| 0 | 0 | 0.85620915 | 0.321708026 | TNFSF4;NK[illegible] |
| 0 | 0 | 0.85620915 | 0.321708026 | TFR2;SPP1[illegible] |
| 0 | 0 | 0.85620915 | 0.321708026 | MMP14;G[illegible] |
| 0 | 0 | 0.85620915 | 0.321708026 | RPS6KA5;[illegible] |
| 0 | 0 | 0.85620915 | 0.321708026 | PLGRKT;N[illegible] |
| 0 | 0 | 0.85620915 | 0.321708026 | CREB3L3;G[illegible] |
| 0 | 0 | 0.85620915 | 0.321708026 | PDGFB;IGF[illegible] |

| | | | | | |
|---|---|---|---|---|---|
| 0 | 0 | 0.85620915 | 0.321708026 | ABCC5;AB( | . |
| 0 | 0 | 0.856083733 | 0.318307786 | ACER2;KAN | . |
| 0 | 0 | 0.856083733 | 0.318307786 | SEPTIN4;ST | . |
| 0 | 0 | 0.856083733 | 0.318307786 | KANK1;ST3 | . |
| 0 | 0 | 0.893963441 | 0.33186126 | HPS4;ZDHH | . |
| 0 | 0 | 0.86974926 | 0.319672953 | CPNE9;TRF | . |
| 0 | 0 | 0.86974926 | 0.319672953 | TBL2;FAM1 | . |
| 0 | 0 | 0.877533859 | 0.320749747 | DNAJB2;DA | . |
| 0 | 0 | 0.877533859 | 0.320749747 | WFS1;COL1 | . |
| 0 | 0 | 0.856020942 | 0.31170745 | P2RX7;WFS | . |
| 0 | 0 | 0.856020942 | 0.31170745 | NDC1;NUP | . |
| 0 | 0 | 0.855958097 | 0.304361448 | PSMA6;CTS | . |
| 0 | 0 | 0.875253409 | 0.310395492 | SETD4;LRS | . |
| 0 | 0 | 0.875253409 | 0.310395492 | LATS1;TFR( | . |
| 0 | 0 | 0.856271777 | 0.2994987 | TBCD | . |
| 0 | 0 | 0.856271777 | 0.2994987 | SLC36A4 | . |
| 0 | 0 | 0.856271777 | 0.2994987 | GNE | . |
| 0 | 0 | 0.856271777 | 0.2994987 | DDAH2 | . |
| 0 | 0 | 0.856271777 | 0.2994987 | ATP2B4 | . |
| 0 | 0 | 0.856271777 | 0.2994987 | SHPK | . |
| 0 | 0 | 0.856271777 | 0.2994987 | TRPM2 | . |
| 0 | 0 | 0.856271777 | 0.2994987 | GUCY2C | . |
| 0 | 0 | 0.856271777 | 0.2994987 | SLC12A6 | . |
| 0 | 0 | 0.856271777 | 0.2994987 | XYLT2 | . |
| 0 | 0 | 0.856271777 | 0.2994987 | APOA4 | . |
| 0 | 0 | 0.856271777 | 0.2994987 | MFAP4 | . |
| 0 | 0 | 0.856271777 | 0.2994987 | MTHFD1 | . |
| 0 | 0 | 0.856271777 | 0.2994987 | ZNF358 | . |
| 0 | 0 | 0.856271777 | 0.2994987 | KDM1B | . |
| 0 | 0 | 0.856271777 | 0.2994987 | HOXB4 | . |
| 0 | 0 | 0.856271777 | 0.2994987 | KDM2B | . |
| 0 | 0 | 0.856271777 | 0.2994987 | PRDX4 | . |
| 0 | 0 | 0.856271777 | 0.2994987 | CD19 | . |
| 0 | 0 | 0.856271777 | 0.2994987 | KYAT1 | . |
| 0 | 0 | 0.856271777 | 0.2994987 | SOD1 | . |
| 0 | 0 | 0.856271777 | 0.2994987 | APLN | . |
| 0 | 0 | 0.856271777 | 0.2994987 | BBS2 | . |
| 0 | 0 | 0.856271777 | 0.2994987 | DGKA | . |
| 0 | 0 | 0.856271777 | 0.2994987 | FOXJ1 | . |
| 0 | 0 | 0.856271777 | 0.2994987 | PNRC2 | . |
| 0 | 0 | 0.856271777 | 0.2994987 | TUT4 | . |
| 0 | 0 | 0.856271777 | 0.2994987 | MRC1 | . |
| 0 | 0 | 0.856271777 | 0.2994987 | FER1L5 | . |
| 0 | 0 | 0.856271777 | 0.2994987 | SLC12A6 | . |
| 0 | 0 | 0.856271777 | 0.2994987 | ITGB3 | . |
| 0 | 0 | 0.856271777 | 0.2994987 | TWSG1 | . |
| 0 | 0 | 0.856271777 | 0.2994987 | ATRAID | . |

| | | | | | |
|---|---|---|---|---|---|
| 0 | 0 | 0.856271777 | 0.2994987 | SUV39H1 | . |
| 0 | 0 | 0.856271777 | 0.2994987 | ACTN3 | . |
| 0 | 0 | 0.856271777 | 0.2994987 | RAB11FIP5 | . |
| 0 | 0 | 0.856271777 | 0.2994987 | FOXJ1 | . |
| 0 | 0 | 0.856271777 | 0.2994987 | DNAJB2 | . |
| 0 | 0 | 0.856271777 | 0.2994987 | IMPACT | . |
| 0 | 0 | 0.856271777 | 0.2994987 | ACTN3 | . |
| 0 | 0 | 0.856271777 | 0.2994987 | COMMD1 | . |
| 0 | 0 | 0.856271777 | 0.2994987 | TREM2 | . |
| 0 | 0 | 0.856271777 | 0.2994987 | CDH3 | . |
| 0 | 0 | 0.856271777 | 0.2994987 | GNB5 | . |
| 0 | 0 | 0.856271777 | 0.2994987 | RTN4 | . |
| 0 | 0 | 0.856271777 | 0.2994987 | FARP2 | . |
| 0 | 0 | 0.856271777 | 0.2994987 | EXOSC9 | . |
| 0 | 0 | 0.856271777 | 0.2994987 | EXOSC9 | . |
| 0 | 0 | 0.856271777 | 0.2994987 | NME4 | . |
| 0 | 0 | 0.856271777 | 0.2994987 | CYP2U1 | . |
| 0 | 0 | 0.856271777 | 0.2994987 | SFRP4 | . |
| 0 | 0 | 0.856271777 | 0.2994987 | LGALS1 | . |
| 0 | 0 | 0.856271777 | 0.2994987 | ARNTL | . |
| 0 | 0 | 0.856271777 | 0.2994987 | ST3GAL4 | . |
| 0 | 0 | 0.856271777 | 0.2994987 | CUX2 | . |
| 0 | 0 | 0.856271777 | 0.2994987 | ST3GAL4 | . |
| 0 | 0 | 0.856271777 | 0.2994987 | MMP9 | . |
| 0 | 0 | 0.856271777 | 0.2994987 | ACTN3 | . |
| 0 | 0 | 0.856271777 | 0.2994987 | ICE2 | . |
| 0 | 0 | 0.856271777 | 0.2994987 | PWP1 | . |
| 0 | 0 | 0.856271777 | 0.2994987 | ITGB3 | . |
| 0 | 0 | 0.856271777 | 0.2994987 | LGALS1 | . |
| 0 | 0 | 0.856271777 | 0.2994987 | SULT1C4 | . |
| 0 | 0 | 0.856271777 | 0.2994987 | DROSHA | . |
| 0 | 0 | 0.856271777 | 0.2994987 | EMC9 | . |
| 0 | 0 | 0.856271777 | 0.2994987 | FNTB | . |
| 0 | 0 | 0.856271777 | 0.2994987 | GUCY2C | . |
| 0 | 0 | 0.856271777 | 0.2994987 | MYBPC3 | . |
| 0 | 0 | 0.856271777 | 0.2994987 | STAR | . |
| 0 | 0 | 0.856271777 | 0.2994987 | PDK2 | . |
| 0 | 0 | 0.856271777 | 0.2994987 | WNK1 | . |
| 0 | 0 | 0.856271777 | 0.2994987 | KDM1B | . |
| 0 | 0 | 0.856271777 | 0.2994987 | C1QC | . |
| 0 | 0 | 0.856271777 | 0.2994987 | NIPSNAP2 | . |
| 0 | 0 | 0.856271777 | 0.2994987 | ST3GAL4 | . |
| 0 | 0 | 0.856271777 | 0.2994987 | CD84 | . |
| 0 | 0 | 0.856271777 | 0.2994987 | TNFSF4 | . |
| 0 | 0 | 0.856271777 | 0.2994987 | MTMR1 | . |
| 0 | 0 | 0.856271777 | 0.2994987 | CEP250 | . |
| 0 | 0 | 0.856271777 | 0.2994987 | MMP9 | . |

| | | | | | |
|---|---|---|---|---|---|
| 0 | 0 | 0.856271777 | 0.2994987 | CD84 | . |
| 0 | 0 | 0.856271777 | 0.2994987 | CLU | . |
| 0 | 0 | 0.856271777 | 0.2994987 | SHPK | . |
| 0 | 0 | 0.856271777 | 0.2994987 | MAP2K5 | . |
| 0 | 0 | 0.856271777 | 0.2994987 | PPARG | . |
| 0 | 0 | 0.856271777 | 0.2994987 | ATP2A1 | . |
| 0 | 0 | 0.856271777 | 0.2994987 | SCIN | . |
| 0 | 0 | 0.856271777 | 0.2994987 | TST | . |
| 0 | 0 | 0.866515516 | 0.300734414 | CRTAP;COI | . |
| 0 | 0 | 0.866515516 | 0.300734414 | SETD4;GRN | . |
| 0 | 0 | 0.855895197 | 0.296806231 | WFS1;ALO: | . |
| 0 | 0 | 0.855895197 | 0.296806231 | TRPM2;OD | . |
| 0 | 0 | 0.930435593 | 0.322206508 | TFRC;CITEI | . |
| 0 | 0 | 0.825523104 | 0.285659648 | DNAJB4;EN | . |
| 0 | 0 | 0.825523104 | 0.285659648 | PKD2;HCN: | . |
| 0 | 0 | 0.825523104 | 0.285659648 | TFAP4;E2F | . |
| 0 | 0 | 0.825523104 | 0.285659648 | SUFU;TWIS | . |
| 0 | 0 | 0.825523104 | 0.285659648 | FZD5;HPS4 | . |
| 0 | 0 | 0.825523104 | 0.285659648 | GORASP1;I | . |
| 0 | 0 | 0.825523104 | 0.285659648 | SCAMP5;SY | . |
| 0 | 0 | 0.825523104 | 0.285659648 | EPS8;VAV3 | . |
| 0 | 0 | 0.825523104 | 0.285659648 | LATS1;IGF1 | . |
| 0 | 0 | 0.825523104 | 0.285659648 | FITM2;APC | . |
| 0 | 0 | 0.832899188 | 0.287225794 | MAP4K2;T. | . |
| 0 | 0 | 0.832899188 | 0.287225794 | RNASEH2C | . |
| 0 | 0 | 0.832899188 | 0.287225794 | METTL22;E | . |
| 0 | 0 | 0.832899188 | 0.287225794 | PPA2;ARL6 | . |
| 0 | 0 | 0.837364368 | 0.284870256 | PSMD10;P: | . |
| 0 | 0 | 0.837364368 | 0.284870256 | HDAC6;RTI | . |
| 0 | 0 | 0.837364368 | 0.284870256 | KANK1;CAI | . |
| 0 | 0 | 0.811099645 | 0.274632103 | EDNRB;SEN | . |
| 0 | 0 | 0.811099645 | 0.274632103 | ADTRP;MG | . |
| 0 | 0 | 0.811099645 | 0.274632103 | UGDH;CHP | . |
| 0 | 0 | 0.811099645 | 0.274632103 | IGF1;PLD3 | . |
| 0 | 0 | 0.811099645 | 0.274632103 | CAB39;MN | . |
| 0 | 0 | 0.811099645 | 0.274632103 | CIB1;CLASI | . |
| 0 | 0 | 0.811099645 | 0.274632103 | DAB2;ABI3 | . |
| 0 | 0 | 0.811099645 | 0.274632103 | ACVR1C;IG | . |
| 0 | 0 | 0.811099645 | 0.274632103 | ALOX5;SIG | . |
| 0 | 0 | 0.811099645 | 0.274632103 | CDK5;FAM | . |
| 0 | 0 | 0.811099645 | 0.274632103 | STAP1;KCN | . |
| 0 | 0 | 0.811099645 | 0.274632103 | PDK4;TLE5 | . |
| 0 | 0 | 0.811099645 | 0.274632103 | VGLL4;TP7 | . |
| 0 | 0 | 0.811099645 | 0.274632103 | GORASP1;( | . |
| 0 | 0 | 0.811099645 | 0.274632103 | METTL5;TH | . |
| 0 | 0 | 0.811099645 | 0.274632103 | P2RX7;NIP. | . |
| 0 | 0 | 0.881709495 | 0.295474489 | PSMD10;A | . |

| | | | | |
|---|---|---|---|---|
| 0 | 0 | 0.842474072 | 0.275553873 | GRAMD1B |
| 0 | 0 | 0.842474072 | 0.275553873 | VAV3;NCKI |
| 0 | 0 | 0.842474072 | 0.275553873 | PHF20;HAT |
| 0 | 0 | 0.855706165 | 0.274770011 | CD63;ACTN |
| 0 | 0 | 0.855706165 | 0.274770011 | FASTKD2;R |
| 0 | 0 | 0.855706165 | 0.274770011 | LATS1;DAB |
| 0 | 0 | 0.810934882 | 0.259303006 | ZNHIT1;NA |
| 0 | 0 | 0.810934882 | 0.259303006 | ST3GAL5;C |
| 0 | 0 | 0.810934882 | 0.259303006 | LGALS3;PD |
| 0 | 0 | 0.810934882 | 0.259303006 | THRB;TAF1 |
| 0 | 0 | 0.810934882 | 0.259303006 | TENM4;KIF |
| 0 | 0 | 0.888248614 | 0.282565495 | GRN;SPAR( |
| 0 | 0 | 0.797011695 | 0.253037053 | CD86;LPAR |
| 0 | 0 | 0.797011695 | 0.253037053 | MYBPC3;ZI |
| 0 | 0 | 0.797011695 | 0.253037053 | TM7SF3;HI |
| 0 | 0 | 0.797011695 | 0.253037053 | COG2;COP |
| 0 | 0 | 0.797011695 | 0.253037053 | APOOL;MT |
| 0 | 0 | 0.797011695 | 0.253037053 | TFAP4;NAF |
| 0 | 0 | 0.797011695 | 0.253037053 | ACER2;BCA |
| 0 | 0 | 0.797011695 | 0.253037053 | BBS2;KIAA( |
| 0 | 0 | 0.797011695 | 0.253037053 | DAB2;CLO( |
| 0 | 0 | 0.797011695 | 0.253037053 | FKBP7;PPIH |
| 0 | 0 | 0.797011695 | 0.253037053 | CARMIL1;A |
| 0 | 0 | 0.797011695 | 0.253037053 | AKT3;CIB1; |
| 0 | 0 | 0.797011695 | 0.253037053 | DLG4;CAM |
| 0 | 0 | 0.797011695 | 0.253037053 | RRAGB;RRA |
| 0 | 0 | 0.825294631 | 0.258819818 | ACVR1C;AS |
| 0 | 0 | 0.855643045 | 0.26785108 | SCAMP5;C |
| 0 | 0 | 0.84528505 | 0.264246107 | WFS1;COL1 |
| 0 | 0 | 0.862940848 | 0.268682506 | STN1;ATAD |
| 0 | 0 | 0.829465905 | 0.255800283 | VAV3;NCKI |
| 0 | 0 | 0.829465905 | 0.255800283 | ACHE;GOLI |
| 0 | 0 | 0.829465905 | 0.255800283 | GRAMD1B |
| 0 | 0 | 0.770501089 | 0.235208449 | SGO2;ANK |
| 0 | 0 | 0.770501089 | 0.235208449 | TUBB;KLRF |
| 0 | 0 | 0.770501089 | 0.235208449 | LATS1;TFAI |
| 0 | 0 | 0.770501089 | 0.235208449 | VSIG4;HAV |
| 0 | 0 | 0.770501089 | 0.235208449 | MEIS1;CIB1 |
| 0 | 0 | 0.770501089 | 0.235208449 | TIPRL;STYX |
| 0 | 0 | 0.770501089 | 0.235208449 | MIGA2;MI( |
| 0 | 0 | 0.770501089 | 0.235208449 | PLA2G12A |
| 0 | 0 | 0.770501089 | 0.235208449 | ABCA2;CLU |
| 0 | 0 | 0.770501089 | 0.235208449 | MED24;TW |
| 0 | 0 | 0.770501089 | 0.235208449 | GRN;APH1 |
| 0 | 0 | 0.770501089 | 0.235208449 | CHD5;PLA2 |
| 0 | 0 | 0.770501089 | 0.235208449 | PSMA6;PSI |
| 0 | 0 | 0.770501089 | 0.235208449 | DLG4;KIF3A |

| | | | | | |
|---|---|---|---|---|---|
| 0 | 0 | 0.770501089 | 0.235208449 | EDNRB;PAI | . |
| 0 | 0 | 0.770501089 | 0.235208449 | PARK7;BOI | . |
| 0 | 0 | 0.770501089 | 0.235208449 | ITGB3;ALKI | . |
| 0 | 0 | 0.770501089 | 0.235208449 | MAP2K5;T | . |
| 0 | 0 | 0.770501089 | 0.235208449 | METTL5;M | . |
| 0 | 0 | 0.770501089 | 0.235208449 | MVB12A;V | . |
| 0 | 0 | 0.855579869 | 0.261174521 | CD86;IL36/ | . |
| 0 | 0 | 0.862346036 | 0.261633727 | PPM1L;PD | . |
| 0 | 0 | 0.770601045 | 0.233319494 | CYP27A1 | . |
| 0 | 0 | 0.770601045 | 0.233319494 | XYLT2 | . |
| 0 | 0 | 0.770601045 | 0.233319494 | ITGB3 | . |
| 0 | 0 | 0.770601045 | 0.233319494 | ZFPM1 | . |
| 0 | 0 | 0.770601045 | 0.233319494 | CD19 | . |
| 0 | 0 | 0.770601045 | 0.233319494 | KCNIP2 | . |
| 0 | 0 | 0.770601045 | 0.233319494 | ZC4H2 | . |
| 0 | 0 | 0.770601045 | 0.233319494 | KIF14 | . |
| 0 | 0 | 0.770601045 | 0.233319494 | ATP6V1A | . |
| 0 | 0 | 0.770601045 | 0.233319494 | CHEK1 | . |
| 0 | 0 | 0.770601045 | 0.233319494 | EDNRB | . |
| 0 | 0 | 0.770601045 | 0.233319494 | RAP2A | . |
| 0 | 0 | 0.770601045 | 0.233319494 | VRK1 | . |
| 0 | 0 | 0.770601045 | 0.233319494 | C1QC | . |
| 0 | 0 | 0.770601045 | 0.233319494 | VEGFB | . |
| 0 | 0 | 0.770601045 | 0.233319494 | IMPA2 | . |
| 0 | 0 | 0.770601045 | 0.233319494 | IGF1 | . |
| 0 | 0 | 0.770601045 | 0.233319494 | CD36 | . |
| 0 | 0 | 0.770601045 | 0.233319494 | MSH6 | . |
| 0 | 0 | 0.770601045 | 0.233319494 | ATG10 | . |
| 0 | 0 | 0.770601045 | 0.233319494 | PKD2 | . |
| 0 | 0 | 0.770601045 | 0.233319494 | AGO4 | . |
| 0 | 0 | 0.770601045 | 0.233319494 | IGF1 | . |
| 0 | 0 | 0.770601045 | 0.233319494 | CRTAM | . |
| 0 | 0 | 0.770601045 | 0.233319494 | FSTL3 | . |
| 0 | 0 | 0.770601045 | 0.233319494 | ABCA2 | . |
| 0 | 0 | 0.770601045 | 0.233319494 | TARBP2 | . |
| 0 | 0 | 0.770601045 | 0.233319494 | TNFSF4 | . |
| 0 | 0 | 0.770601045 | 0.233319494 | ADTRP | . |
| 0 | 0 | 0.770601045 | 0.233319494 | PARK7 | . |
| 0 | 0 | 0.770601045 | 0.233319494 | CAB39 | . |
| 0 | 0 | 0.770601045 | 0.233319494 | SLC25A4 | . |
| 0 | 0 | 0.770601045 | 0.233319494 | COMMD1 | . |
| 0 | 0 | 0.770601045 | 0.233319494 | ALOX5 | . |
| 0 | 0 | 0.770601045 | 0.233319494 | TNFSF4 | . |
| 0 | 0 | 0.770601045 | 0.233319494 | AGO4 | . |
| 0 | 0 | 0.770601045 | 0.233319494 | PDXP | . |
| 0 | 0 | 0.770601045 | 0.233319494 | IMPA2 | . |
| 0 | 0 | 0.770601045 | 0.233319494 | SCIN | . |

| | | | | | |
|---|---|---|---|---|---|
| 0 | 0 | 0.770601045 | 0.233319494 | SCAP | . |
| 0 | 0 | 0.770601045 | 0.233319494 | PARK7 | . |
| 0 | 0 | 0.770601045 | 0.233319494 | DAB2 | . |
| 0 | 0 | 0.770601045 | 0.233319494 | ZDHHC15 | . |
| 0 | 0 | 0.770601045 | 0.233319494 | DNMT1 | . |
| 0 | 0 | 0.770601045 | 0.233319494 | POT1 | . |
| 0 | 0 | 0.770601045 | 0.233319494 | CDH3 | . |
| 0 | 0 | 0.770601045 | 0.233319494 | PRKCA | . |
| 0 | 0 | 0.770601045 | 0.233319494 | DAB2 | . |
| 0 | 0 | 0.770601045 | 0.233319494 | PRAP1 | . |
| 0 | 0 | 0.770601045 | 0.233319494 | CREB3L3 | . |
| 0 | 0 | 0.770601045 | 0.233319494 | SLC27A1 | . |
| 0 | 0 | 0.770601045 | 0.233319494 | PDGFB | . |
| 0 | 0 | 0.770601045 | 0.233319494 | STX17 | . |
| 0 | 0 | 0.770601045 | 0.233319494 | NT5M | . |
| 0 | 0 | 0.770601045 | 0.233319494 | CAMK2A | . |
| 0 | 0 | 0.770601045 | 0.233319494 | TWSG1 | . |
| 0 | 0 | 0.770601045 | 0.233319494 | PROCR | . |
| 0 | 0 | 0.770601045 | 0.233319494 | KDM1B | . |
| 0 | 0 | 0.770601045 | 0.233319494 | POT1 | . |
| 0 | 0 | 0.770601045 | 0.233319494 | DNAJB2 | . |
| 0 | 0 | 0.770601045 | 0.233319494 | ADTRP | . |
| 0 | 0 | 0.770601045 | 0.233319494 | SPHK2 | . |
| 0 | 0 | 0.770601045 | 0.233319494 | MLLT11 | . |
| 0 | 0 | 0.770601045 | 0.233319494 | RASGRP1 | . |
| 0 | 0 | 0.770601045 | 0.233319494 | GSTP1 | . |
| 0 | 0 | 0.770601045 | 0.233319494 | LGALS3 | . |
| 0 | 0 | 0.770601045 | 0.233319494 | HMGB1 | . |
| 0 | 0 | 0.770601045 | 0.233319494 | DENND1B | . |
| 0 | 0 | 0.770601045 | 0.233319494 | TREM2 | . |
| 0 | 0 | 0.770601045 | 0.233319494 | ABCA2 | . |
| 0 | 0 | 0.770601045 | 0.233319494 | SEC11C | . |
| 0 | 0 | 0.770601045 | 0.233319494 | KLHL41 | . |
| 0 | 0 | 0.770601045 | 0.233319494 | CDK5 | . |
| 0 | 0 | 0.770601045 | 0.233319494 | KYAT1 | . |
| 0 | 0 | 0.770601045 | 0.233319494 | EDNRB | . |
| 0 | 0 | 0.770601045 | 0.233319494 | UGT1A6 | . |
| 0 | 0 | 0.847038495 | 0.252693165 | PSMD10;V | . |
| 0 | 0 | 0.855516637 | 0.254738942 | USP14;GRI | . |
| 0 | 0 | 0.80238329 | 0.238036366 | CD86;PDCI | . |
| 0 | 0 | 0.790096951 | 0.234194476 | CD86;LPAF | . |
| 0 | 0 | 0.790096951 | 0.234194476 | ZMYND15; | . |
| 0 | 0 | 0.790096951 | 0.234194476 | RARS2;LAR | . |
| 0 | 0 | 0.878493448 | 0.260146769 | PSMD10;U | . |
| 0 | 0 | 0.86182257 | 0.254905576 | BCAS3;RAL | . |
| 0 | 0 | 0.810769832 | 0.238667394 | BACE1;GG | . |
| 0 | 0 | 0.866689904 | 0.253849903 | DNAI4;ND | . |

| | | | | |
|---|---|---|---|---|
| 0 | 0 | 0.770401046 | 0.224381903 | EPS8;CALD… |
| 0 | 0 | 0.770401046 | 0.224381903 | CHRNB1;FI… |
| 0 | 0 | 0.770401046 | 0.224381903 | ITGB3;SEM… |
| 0 | 0 | 0.770401046 | 0.224381903 | FZD5;SPHK… |
| 0 | 0 | 0.770401046 | 0.224381903 | PREPL;CAN… |
| 0 | 0 | 0.770401046 | 0.224381903 | E2F1;CIRBI… |
| 0 | 0 | 0.770401046 | 0.224381903 | ACER2;PDK… |
| 0 | 0 | 0.770401046 | 0.224381903 | CUL7;GLM… |
| 0 | 0 | 0.837008802 | 0.243054795 | ASPH;ATG1… |
| 0 | 0 | 0.837008802 | 0.243054795 | GRN;TNFSI… |
| 0 | 0 | 0.885349326 | 0.256013784 | CD63;KANI… |
| 0 | 0 | 0.907363313 | 0.26220393 | LIPT1;ATP2… |
| 0 | 0 | 0.861356968 | 0.248472402 | CAMK2A;A… |
| 0 | 0 | 0.821459152 | 0.235082609 | B3GALT2;N… |
| 0 | 0 | 0.865988372 | 0.24735186 | KLHL25;SH… |
| 0 | 0 | 0.838604333 | 0.238345537 | PSMD10;V… |
| 0 | 0 | 0.825065559 | 0.232026159 | LATS1;TFR… |
| 0 | 0 | 0.825065559 | 0.232026159 | ASPH;TME… |
| 0 | 0 | 0.7859636 | 0.217628408 | VEGFB;HM… |
| 0 | 0 | 0.7859636 | 0.217628408 | PSMD10;P… |
| 0 | 0 | 0.796745887 | 0.220076336 | ALDH3A2;… |
| 0 | 0 | 0.796745887 | 0.220076336 | CDK8;MED… |
| 0 | 0 | 0.733769063 | 0.202026461 | APLP1;RPG… |
| 0 | 0 | 0.733769063 | 0.202026461 | BCAS3;WN… |
| 0 | 0 | 0.733769063 | 0.202026461 | RPS6KA5;S… |
| 0 | 0 | 0.733769063 | 0.202026461 | DUSP19;SA… |
| 0 | 0 | 0.733769063 | 0.202026461 | MED24;ESI… |
| 0 | 0 | 0.733769063 | 0.202026461 | UBASH3B;I… |
| 0 | 0 | 0.733769063 | 0.202026461 | GSTO1;CLI… |
| 0 | 0 | 0.733769063 | 0.202026461 | MAPRE3;P… |
| 0 | 0 | 0.733769063 | 0.202026461 | KIF3A;KIFA… |
| 0 | 0 | 0.770300916 | 0.211597036 | UBASH3B;I… |
| 0 | 0 | 0.770300916 | 0.211597036 | TADA3;TRI… |
| 0 | 0 | 0.770300916 | 0.211597036 | CIB1;CD36… |
| 0 | 0 | 0.770300916 | 0.211597036 | CARMIL1;C… |
| 0 | 0 | 0.804614482 | 0.220370166 | VAV3;NCKI… |
| 0 | 0 | 0.86477871 | 0.235196431 | TSPYL2;W… |
| 0 | 0 | 0.830335546 | 0.224766816 | BSCL2;ACH… |
| 0 | 0 | 0.810604493 | 0.219306729 | SLC27A1;P… |
| 0 | 0 | 0.86824683 | 0.233419857 | TSPYL2;PSI… |
| 0 | 0 | 0.745507214 | 0.199165372 | ACTN3;LAN… |
| 0 | 0 | 0.745507214 | 0.199165372 | AGTPBP1;Z… |
| 0 | 0 | 0.745507214 | 0.199165372 | SUV39H1;I… |
| 0 | 0 | 0.745507214 | 0.199165372 | STAR;SRD5… |
| 0 | 0 | 0.745507214 | 0.199165372 | P2RX7;P2R… |
| 0 | 0 | 0.745507214 | 0.199165372 | NEU3;CCD… |
| 0 | 0 | 0.745507214 | 0.199165372 | TNFSF4;HN… |

| | | | | | |
|---|---|---|---|---|---|
| 0 | 0 | 0.745507214 | 0.199165372 | PDGFB;PPA | . |
| 0 | 0 | 0.745507214 | 0.199165372 | GSTO1;CD1 | . |
| 0 | 0 | 0.745507214 | 0.199165372 | ALDH3A2;C | . |
| 0 | 0 | 0.745507214 | 0.199165372 | CRTAM;HN | . |
| 0 | 0 | 0.745507214 | 0.199165372 | THUMPD2, | . |
| 0 | 0 | 0.815312629 | 0.217361594 | MEIS1;MEI | . |
| 0 | 0 | 0.815312629 | 0.217361594 | CSTB;EDNF | . |
| 0 | 0 | 0.70050681 | 0.184195098 | DYRK1B | . |
| 0 | 0 | 0.70050681 | 0.184195098 | CPA3 | . |
| 0 | 0 | 0.70050681 | 0.184195098 | SLC25A4 | . |
| 0 | 0 | 0.70050681 | 0.184195098 | RMDN1 | . |
| 0 | 0 | 0.70050681 | 0.184195098 | BBS2 | . |
| 0 | 0 | 0.70050681 | 0.184195098 | AGTPBP1 | . |
| 0 | 0 | 0.70050681 | 0.184195098 | NPRL2 | . |
| 0 | 0 | 0.70050681 | 0.184195098 | NPRL2 | . |
| 0 | 0 | 0.70050681 | 0.184195098 | CHST14 | . |
| 0 | 0 | 0.70050681 | 0.184195098 | STX10 | . |
| 0 | 0 | 0.70050681 | 0.184195098 | RTN4 | . |
| 0 | 0 | 0.70050681 | 0.184195098 | ATP5PO | . |
| 0 | 0 | 0.70050681 | 0.184195098 | ITGB5 | . |
| 0 | 0 | 0.70050681 | 0.184195098 | ACSS1 | . |
| 0 | 0 | 0.70050681 | 0.184195098 | MYO1E | . |
| 0 | 0 | 0.70050681 | 0.184195098 | IMPA2 | . |
| 0 | 0 | 0.70050681 | 0.184195098 | KDELR3 | . |
| 0 | 0 | 0.70050681 | 0.184195098 | SLC24A5 | . |
| 0 | 0 | 0.70050681 | 0.184195098 | UQCC3 | . |
| 0 | 0 | 0.70050681 | 0.184195098 | MTM1 | . |
| 0 | 0 | 0.70050681 | 0.184195098 | ZNF502 | . |
| 0 | 0 | 0.70050681 | 0.184195098 | BBS5 | . |
| 0 | 0 | 0.70050681 | 0.184195098 | EIF2AK4 | . |
| 0 | 0 | 0.70050681 | 0.184195098 | ATP2B4 | . |
| 0 | 0 | 0.70050681 | 0.184195098 | ATP2B4 | . |
| 0 | 0 | 0.70050681 | 0.184195098 | VSIG4 | . |
| 0 | 0 | 0.70050681 | 0.184195098 | ACADVL | . |
| 0 | 0 | 0.70050681 | 0.184195098 | ADTRP | . |
| 0 | 0 | 0.70050681 | 0.184195098 | ESR1 | . |
| 0 | 0 | 0.70050681 | 0.184195098 | RECQL5 | . |
| 0 | 0 | 0.70050681 | 0.184195098 | HABP4 | . |
| 0 | 0 | 0.70050681 | 0.184195098 | NT5M | . |
| 0 | 0 | 0.70050681 | 0.184195098 | PDXP | . |
| 0 | 0 | 0.70050681 | 0.184195098 | NAA16 | . |
| 0 | 0 | 0.70050681 | 0.184195098 | EDNRB | . |
| 0 | 0 | 0.70050681 | 0.184195098 | APOA4 | . |
| 0 | 0 | 0.70050681 | 0.184195098 | FABP3 | . |
| 0 | 0 | 0.70050681 | 0.184195098 | ABI3 | . |
| 0 | 0 | 0.70050681 | 0.184195098 | BOK | . |
| 0 | 0 | 0.70050681 | 0.184195098 | CDC23 | . |

| | | | | | |
|---|---|---|---|---|---|
| 0 | 0 | 0.70050681 | 0.184195098 | CDC23 | . |
| 0 | 0 | 0.70050681 | 0.184195098 | CDC23 | . |
| 0 | 0 | 0.70050681 | 0.184195098 | PDXP | . |
| 0 | 0 | 0.70050681 | 0.184195098 | MTHFD1 | . |
| 0 | 0 | 0.70050681 | 0.184195098 | PDXP | . |
| 0 | 0 | 0.70050681 | 0.184195098 | ABCA2 | . |
| 0 | 0 | 0.70050681 | 0.184195098 | ABCA2 | . |
| 0 | 0 | 0.70050681 | 0.184195098 | CPA3 | . |
| 0 | 0 | 0.70050681 | 0.184195098 | GRN | . |
| 0 | 0 | 0.70050681 | 0.184195098 | EIF2AK4 | . |
| 0 | 0 | 0.70050681 | 0.184195098 | PPP1R3C | . |
| 0 | 0 | 0.70050681 | 0.184195098 | LGALS3 | . |
| 0 | 0 | 0.70050681 | 0.184195098 | MMP9 | . |
| 0 | 0 | 0.70050681 | 0.184195098 | MYL9 | . |
| 0 | 0 | 0.70050681 | 0.184195098 | TICAM1 | . |
| 0 | 0 | 0.70050681 | 0.184195098 | PLGRKT | . |
| 0 | 0 | 0.70050681 | 0.184195098 | UBASH3B | . |
| 0 | 0 | 0.70050681 | 0.184195098 | FEN1 | . |
| 0 | 0 | 0.70050681 | 0.184195098 | TICAM1 | . |
| 0 | 0 | 0.783197331 | 0.202923118 | ACER2;CIB | . |
| 0 | 0 | 0.770200698 | 0.198986041 | NDC1;NUP | . |
| 0 | 0 | 0.770200698 | 0.198986041 | NDC1;NUP | . |
| 0 | 0 | 0.770200698 | 0.198986041 | NDC1;NUP | . |
| 0 | 0 | 0.822227319 | 0.211917717 | CD86;CCD( | . |
| 0 | 0 | 0.822227319 | 0.211917717 | RPS6KA5;P | . |
| 0 | 0 | 0.822227319 | 0.211917717 | KANK1;RAI | . |
| 0 | 0 | 0.800031773 | 0.204555744 | PSMD10;V | . |
| 0 | 0 | 0.751470541 | 0.19124358 | SEMA3A;SI | . |
| 0 | 0 | 0.751470541 | 0.19124358 | GRN;POLR: | . |
| 0 | 0 | 0.751470541 | 0.19124358 | PDGFB;NF/ | . |
| 0 | 0 | 0.80578651 | 0.203585265 | BBS2;OAT; | . |
| 0 | 0 | 0.855072464 | 0.215727394 | SCARB2;M | . |
| 0 | 0 | 0.859333966 | 0.214960986 | CD86;H2A; | . |
| 0 | 0 | 0.700376312 | 0.173972553 | FHOD3;OB | . |
| 0 | 0 | 0.700376312 | 0.173972553 | SUV39H1;S | . |
| 0 | 0 | 0.700376312 | 0.173972553 | CD19;SPG2 | . |
| 0 | 0 | 0.700376312 | 0.173972553 | BSCL2;CIDI | . |
| 0 | 0 | 0.700376312 | 0.173972553 | CIB1;CLASI | . |
| 0 | 0 | 0.700376312 | 0.173972553 | ACHE;PICK | . |
| 0 | 0 | 0.700376312 | 0.173972553 | TAB1;MAP | . |
| 0 | 0 | 0.700376312 | 0.173972553 | TEK;LIMS1 | . |
| 0 | 0 | 0.700376312 | 0.173972553 | EDEM2;M/ | . |
| 0 | 0 | 0.700376312 | 0.173972553 | SPR;DDAH: | . |
| 0 | 0 | 0.814275019 | 0.199760942 | CD86;DDB: | . |
| 0 | 0 | 0.722169246 | 0.176937731 | FABP3;FAB | . |
| 0 | 0 | 0.722169246 | 0.176937731 | SVBP;VASH | . |
| 0 | 0 | 0.722169246 | 0.176937731 | GORASP1;I | . |

| | | | | | |
|---|---|---|---|---|---|
| 0 | 0 | 0.722169246 | 0.176937731 | KCNIP2;AD | . |
| 0 | 0 | 0.722169246 | 0.176937731 | P2RX7;WN | . |
| 0 | 0 | 0.722169246 | 0.176937731 | LATS1;ARH | . |
| 0 | 0 | 0.828943024 | 0.201970529 | KANK1;CIT | . |
| 0 | 0 | 0.781203721 | 0.189753791 | SHTN1;NTI | . |
| 0 | 0 | 0.781203721 | 0.189753791 | BCAS3;THF | . |
| 0 | 0 | 0.781203721 | 0.189753791 | PSMD10;M | . |
| 0 | 0 | 0.789730148 | 0.190764024 | FZD5;TNFS | . |
| 0 | 0 | 0.755055949 | 0.181950928 | WNK1;CIB | . |
| 0 | 0 | 0.755055949 | 0.181950928 | TUT4;EXOS | . |
| 0 | 0 | 0.755055949 | 0.181950928 | TPST1;ABI | . |
| 0 | 0 | 0.755055949 | 0.181950928 | CLDN12;TE | . |
| 0 | 0 | 0.733536851 | 0.17289822 | GSTO1;PD | . |
| 0 | 0 | 0.733536851 | 0.17289822 | SASH3;VA\ | . |
| 0 | 0 | 0.806474191 | 0.188261069 | PSMD10;T | . |
| 0 | 0 | 0.850600254 | 0.197815836 | CARMIL1;C | . |
| 0 | 0 | 0.810272947 | 0.186383321 | TSPYL2;TC | . |
| 0 | 0 | 0.810272947 | 0.186383321 | TSPYL2;TC | . |
| 0 | 0 | 0.642094948 | 0.147018562 | ADSS1 | . |
| 0 | 0 | 0.642094948 | 0.147018562 | SRM | . |
| 0 | 0 | 0.642094948 | 0.147018562 | CHEK1 | . |
| 0 | 0 | 0.642094948 | 0.147018562 | ZFPM1 | . |
| 0 | 0 | 0.642094948 | 0.147018562 | INPPL1 | . |
| 0 | 0 | 0.642094948 | 0.147018562 | SPA17 | . |
| 0 | 0 | 0.642094948 | 0.147018562 | APOA4 | . |
| 0 | 0 | 0.642094948 | 0.147018562 | NAPEPLD | . |
| 0 | 0 | 0.642094948 | 0.147018562 | DNAAF5 | . |
| 0 | 0 | 0.642094948 | 0.147018562 | CENPH | . |
| 0 | 0 | 0.642094948 | 0.147018562 | MYO1G | . |
| 0 | 0 | 0.642094948 | 0.147018562 | SPA17 | . |
| 0 | 0 | 0.642094948 | 0.147018562 | CHEK2 | . |
| 0 | 0 | 0.642094948 | 0.147018562 | POGLUT1 | . |
| 0 | 0 | 0.642094948 | 0.147018562 | TFAP4 | . |
| 0 | 0 | 0.642094948 | 0.147018562 | MTM1 | . |
| 0 | 0 | 0.642094948 | 0.147018562 | WFS1 | . |
| 0 | 0 | 0.642094948 | 0.147018562 | CBLB | . |
| 0 | 0 | 0.642094948 | 0.147018562 | MST1 | . |
| 0 | 0 | 0.642094948 | 0.147018562 | VGLL4 | . |
| 0 | 0 | 0.642094948 | 0.147018562 | CCDC125 | . |
| 0 | 0 | 0.642094948 | 0.147018562 | GSTP1 | . |
| 0 | 0 | 0.642094948 | 0.147018562 | CAB39 | . |
| 0 | 0 | 0.642094948 | 0.147018562 | PARK7 | . |
| 0 | 0 | 0.642094948 | 0.147018562 | EMILIN1 | . |
| 0 | 0 | 0.642094948 | 0.147018562 | FER1L5 | . |
| 0 | 0 | 0.642094948 | 0.147018562 | TNFSF4 | . |
| 0 | 0 | 0.642094948 | 0.147018562 | ABI3 | . |
| 0 | 0 | 0.642094948 | 0.147018562 | SLC45A3 | . |

| | | | | | |
|---|---|---|---|---|---|
| 0 | 0 | 0.642094948 | 0.147018562 | UBQLN2 | . |
| 0 | 0 | 0.642094948 | 0.147018562 | DNMT1 | . |
| 0 | 0 | 0.642094948 | 0.147018562 | EDEM2 | . |
| 0 | 0 | 0.642094948 | 0.147018562 | TREM2 | . |
| 0 | 0 | 0.642094948 | 0.147018562 | WNK3 | . |
| 0 | 0 | 0.642094948 | 0.147018562 | UBE2F | . |
| 0 | 0 | 0.642094948 | 0.147018562 | RAB23 | . |
| 0 | 0 | 0.642094948 | 0.147018562 | ADK | . |
| 0 | 0 | 0.642094948 | 0.147018562 | CD19 | . |
| 0 | 0 | 0.642094948 | 0.147018562 | PRKCA | . |
| 0 | 0 | 0.642094948 | 0.147018562 | AAAS | . |
| 0 | 0 | 0.642094948 | 0.147018562 | HDAC6 | . |
| 0 | 0 | 0.642094948 | 0.147018562 | CBLB | . |
| 0 | 0 | 0.642094948 | 0.147018562 | GCC2 | . |
| 0 | 0 | 0.642094948 | 0.147018562 | PIGU | . |
| 0 | 0 | 0.642094948 | 0.147018562 | EDEM2 | . |
| 0 | 0 | 0.642094948 | 0.147018562 | MFAP4 | . |
| 0 | 0 | 0.642094948 | 0.147018562 | UBA5 | . |
| 0 | 0 | 0.642094948 | 0.147018562 | XPO1 | . |
| 0 | 0 | 0.642094948 | 0.147018562 | SLC9A1 | . |
| 0 | 0 | 0.642094948 | 0.147018562 | CDK5 | . |
| 0 | 0 | 0.642094948 | 0.147018562 | TAF1B | . |
| 0 | 0 | 0.642094948 | 0.147018562 | APOA4 | . |
| 0 | 0 | 0.824605609 | 0.188669586 | TNFSF18;E | . |
| 0 | 0 | 0.77 | 0.176042779 | PSMD10;F/ | . |
| 0 | 0 | 0.779689323 | 0.177870248 | DNAJB2;FC | . |
| 0 | 0 | 0.779689323 | 0.177870248 | PSMD10;P: | . |
| 0 | 0 | 0.779689323 | 0.177870248 | SCN11A;SL | . |
| 0 | 0 | 0.757432863 | 0.172487061 | NDC1;NUP | . |
| 0 | 0 | 0.787383781 | 0.178491174 | CALCB;WF! | . |
| 0 | 0 | 0.74049369 | 0.166380003 | CBR1;CYP2 | . |
| 0 | 0 | 0.74049369 | 0.166380003 | CDK5;TREN | . |
| 0 | 0 | 0.74049369 | 0.166380003 | PSMD10;P: | . |
| 0 | 0 | 0.74049369 | 0.166380003 | CUX2;CLST | . |
| 0 | 0 | 0.74049369 | 0.166380003 | TAF1B;SPH | . |
| 0 | 0 | 0.74049369 | 0.166380003 | FZD5;TNFS | . |
| 0 | 0 | 0.74049369 | 0.166380003 | P2RX7;ANI | . |
| 0 | 0 | 0.74049369 | 0.166380003 | WFS1;ARL: | . |
| 0 | 0 | 0.7002457 | 0.15731495 | FER1L5;TRI | . |
| 0 | 0 | 0.7002457 | 0.15731495 | LATS1;CON | . |
| 0 | 0 | 0.7002457 | 0.15731495 | UBASH3B;I | . |
| 0 | 0 | 0.7002457 | 0.15731495 | P2RX7;ALC | . |
| 0 | 0 | 0.7002457 | 0.15731495 | CDC23;PD( | . |
| 0 | 0 | 0.7002457 | 0.15731495 | CARMIL1;C | . |
| 0 | 0 | 0.7002457 | 0.15731495 | FZD5;TNFS | . |
| 0 | 0 | 0.7002457 | 0.15731495 | DAB2;PARI | . |
| 0 | 0 | 0.7002457 | 0.15731495 | KIF14;E2F1 | . |

| | | | | |
|---|---|---|---|---|
| 0 | 0 | 0.826388991 | 0.185613899 | PSMD10;IN |
| 0 | 0 | 0.669887279 | 0.150159194 | APOA4;AN |
| 0 | 0 | 0.669887279 | 0.150159194 | GLP1R;RAF |
| 0 | 0 | 0.669887279 | 0.150159194 | GRIN2C;M/ |
| 0 | 0 | 0.669887279 | 0.150159194 | WFS1;TMT |
| 0 | 0 | 0.669887279 | 0.150159194 | MVB12A;R |
| 0 | 0 | 0.669887279 | 0.150159194 | KANK1;SCI |
| 0 | 0 | 0.669887279 | 0.150159194 | LGALS3;LP) |
| 0 | 0 | 0.669887279 | 0.150159194 | BCAS3;COF |
| 0 | 0 | 0.669887279 | 0.150159194 | ACTN3;MS |
| 0 | 0 | 0.669887279 | 0.150159194 | TPP1;LTA4 |
| 0 | 0 | 0.669887279 | 0.150159194 | APOA4;PP/ |
| 0 | 0 | 0.669887279 | 0.150159194 | CAB39;WN |
| 0 | 0 | 0.669887279 | 0.150159194 | ZER1;HDA( |
| 0 | 0 | 0.669887279 | 0.150159194 | PKD2;SOCS |
| 0 | 0 | 0.669887279 | 0.150159194 | SFRP4;CTSI |
| 0 | 0 | 0.669887279 | 0.150159194 | WNK1;MA |
| 0 | 0 | 0.669887279 | 0.150159194 | CIB1;CLASF |
| 0 | 0 | 0.669887279 | 0.150159194 | RALBP1;IN |
| 0 | 0 | 0.669887279 | 0.150159194 | FNTB;PPEF |
| 0 | 0 | 0.669887279 | 0.150159194 | CEP97;CCS |
| 0 | 0 | 0.669887279 | 0.150159194 | PFKL;EIF2B |
| 0 | 0 | 0.669887279 | 0.150159194 | MYBPC3;M |
| 0 | 0 | 0.716437286 | 0.156352185 | CUL7;HGF; |
| 0 | 0 | 0.716437286 | 0.156352185 | BACE1;GRI |
| 0 | 0 | 0.716437286 | 0.156352185 | DAB2;TWS |
| 0 | 0 | 0.769899519 | 0.165817093 | PSMD10;V |
| 0 | 0 | 0.868136961 | 0.185874114 | ACHE;SLC2 |
| 0 | 0 | 0.745173963 | 0.159005329 | LGALS3;MI |
| 0 | 0 | 0.726480951 | 0.15214383 | STX12;STX: |
| 0 | 0 | 0.726480951 | 0.15214383 | NASP;HAT: |
| 0 | 0 | 0.726480951 | 0.15214383 | TWSG1;DA |
| 0 | 0 | 0.679611775 | 0.139967854 | WFS1;TEK; |
| 0 | 0 | 0.679611775 | 0.139967854 | FAM210B;: |
| 0 | 0 | 0.679611775 | 0.139967854 | MAPRE3;C |
| 0 | 0 | 0.679611775 | 0.139967854 | TREM2;RA |
| 0 | 0 | 0.804015403 | 0.163564045 | GLP1R;CAL |
| 0 | 0 | 0.760351439 | 0.154550954 | BEX3;ACVF |
| 0 | 0 | 0.760351439 | 0.154550954 | EDNRB;DD |
| 0 | 0 | 0.769798951 | 0.15636152 | GLE1;NDC: |
| 0 | 0 | 0.748526201 | 0.151450939 | PSMD10;A |
| 0 | 0 | 0.641938998 | 0.129872909 | DNAJB4;DI |
| 0 | 0 | 0.641938998 | 0.129872909 | FCHO1;ITS |
| 0 | 0 | 0.641938998 | 0.129872909 | PICK1;EIF2 |
| 0 | 0 | 0.641938998 | 0.129872909 | KDM2B;KD |
| 0 | 0 | 0.641938998 | 0.129872909 | SUV39H1;S |
| 0 | 0 | 0.641938998 | 0.129872909 | SGO2;SYCF |

| | | | | | |
|---|---|---|---|---|---|
| 0 | 0 | 0.641938998 | 0.129872909 | SCN11A;H( | . |
| 0 | 0 | 0.641938998 | 0.129872909 | GSTP1;IGF: | . |
| 0 | 0 | 0.641938998 | 0.129872909 | ADTRP;SEF | . |
| 0 | 0 | 0.641938998 | 0.129872909 | BCAS3;PAL | . |
| 0 | 0 | 0.641938998 | 0.129872909 | LSM10;CDI | . |
| 0 | 0 | 0.641938998 | 0.129872909 | CD86;GLM | . |
| 0 | 0 | 0.641938998 | 0.129872909 | CDC23;CDI | . |
| 0 | 0 | 0.641938998 | 0.129872909 | LAG3;TNFS | . |
| 0 | 0 | 0.641938998 | 0.129872909 | CD209;KPN | . |
| 0 | 0 | 0.641938998 | 0.129872909 | SCPEP1;AL | . |
| 0 | 0 | 0.700114974 | 0.141420326 | GSTO1;PD( | . |
| 0 | 0 | 0.700114974 | 0.141420326 | AIFM1;TOI | . |
| 0 | 0 | 0.700114974 | 0.141420326 | UCHL1;GST | . |
| 0 | 0 | 0.700114974 | 0.141420326 | CD86;GLM | . |
| 0 | 0 | 0.700114974 | 0.141420326 | WFS1;CHEI | . |
| 0 | 0 | 0.733304234 | 0.146562009 | DIRAS1;PD | . |
| 0 | 0 | 0.733304234 | 0.146562009 | CARMIL1;C | . |
| 0 | 0 | 0.592669526 | 0.11843066 | ABCB9 | . |
| 0 | 0 | 0.592669526 | 0.11843066 | PKD2 | . |
| 0 | 0 | 0.592669526 | 0.11843066 | ZFPM1 | . |
| 0 | 0 | 0.592669526 | 0.11843066 | ZFPM1 | . |
| 0 | 0 | 0.592669526 | 0.11843066 | SFRP4 | . |
| 0 | 0 | 0.592669526 | 0.11843066 | SLC50A1 | . |
| 0 | 0 | 0.592669526 | 0.11843066 | SOD1 | . |
| 0 | 0 | 0.592669526 | 0.11843066 | KCNIP2 | . |
| 0 | 0 | 0.592669526 | 0.11843066 | RAP2A | . |
| 0 | 0 | 0.592669526 | 0.11843066 | HMGB1 | . |
| 0 | 0 | 0.592669526 | 0.11843066 | NAA16 | . |
| 0 | 0 | 0.592669526 | 0.11843066 | NAXE | . |
| 0 | 0 | 0.592669526 | 0.11843066 | MAP2K5 | . |
| 0 | 0 | 0.592669526 | 0.11843066 | RECQL5 | . |
| 0 | 0 | 0.592669526 | 0.11843066 | PRKG1 | . |
| 0 | 0 | 0.592669526 | 0.11843066 | TPP1 | . |
| 0 | 0 | 0.592669526 | 0.11843066 | TMEM201 | . |
| 0 | 0 | 0.592669526 | 0.11843066 | SOD1 | . |
| 0 | 0 | 0.592669526 | 0.11843066 | RAP2A | . |
| 0 | 0 | 0.592669526 | 0.11843066 | NAPEPLD | . |
| 0 | 0 | 0.592669526 | 0.11843066 | IGF1 | . |
| 0 | 0 | 0.592669526 | 0.11843066 | RASGRP1 | . |
| 0 | 0 | 0.592669526 | 0.11843066 | PLA2G6 | . |
| 0 | 0 | 0.592669526 | 0.11843066 | SPHK2 | . |
| 0 | 0 | 0.592669526 | 0.11843066 | KCNIP2 | . |
| 0 | 0 | 0.592669526 | 0.11843066 | WNK1 | . |
| 0 | 0 | 0.592669526 | 0.11843066 | KCNN4 | . |
| 0 | 0 | 0.592669526 | 0.11843066 | KCNIP2 | . |
| 0 | 0 | 0.592669526 | 0.11843066 | DNMT1 | . |
| 0 | 0 | 0.592669526 | 0.11843066 | SPHK2 | . |

| | | | | | |
|---|---|---|---|---|---|
| 0 | 0 | 0.592669526 | 0.11843066 | CARMIL1 | . |
| 0 | 0 | 0.592669526 | 0.11843066 | LMF1 | . |
| 0 | 0 | 0.592669526 | 0.11843066 | GSTP1 | . |
| 0 | 0 | 0.592669526 | 0.11843066 | PWP1 | . |
| 0 | 0 | 0.592669526 | 0.11843066 | ATP2A1 | . |
| 0 | 0 | 0.592669526 | 0.11843066 | WNK1 | . |
| 0 | 0 | 0.592669526 | 0.11843066 | TPD52 | . |
| 0 | 0 | 0.592669526 | 0.11843066 | NBL1 | . |
| 0 | 0 | 0.592669526 | 0.11843066 | SMTN | . |
| 0 | 0 | 0.592669526 | 0.11843066 | SLCO2B1 | . |
| 0 | 0 | 0.592669526 | 0.11843066 | KLHL41 | . |
| 0 | 0 | 0.592669526 | 0.11843066 | SHTN1 | . |
| 0 | 0 | 0.592669526 | 0.11843066 | SOD1 | . |
| 0 | 0 | 0.592669526 | 0.11843066 | ELP4 | . |
| 0 | 0 | 0.592669526 | 0.11843066 | VEGFB | . |
| 0 | 0 | 0.592669526 | 0.11843066 | SOD1 | . |
| 0 | 0 | 0.712987202 | 0.139125163 | SCARB2;OS | . |
| 0 | 0 | 0.712987202 | 0.139125163 | WWOX;TN | . |
| 0 | 0 | 0.738230544 | 0.140439981 | GPC1;ATP2 | . |
| 0 | 0 | 0.782628046 | 0.148191683 | PSMD10;SI | . |
| 0 | 0 | 0.660156931 | 0.12461329 | COL11A2;I | . |
| 0 | 0 | 0.660156931 | 0.12461329 | GIPC1;PAR | . |
| 0 | 0 | 0.660156931 | 0.12461329 | DOC2B;PLA | . |
| 0 | 0 | 0.660156931 | 0.12461329 | GNG2;KCN | . |
| 0 | 0 | 0.660156931 | 0.12461329 | GEMIN2;G | . |
| 0 | 0 | 0.660156931 | 0.12461329 | RARS2;LAR | . |
| 0 | 0 | 0.721805434 | 0.135076786 | NDUFA11;I | . |
| 0 | 0 | 0.721805434 | 0.135076786 | GORASP1;Z | . |
| 0 | 0 | 0.721805434 | 0.135076786 | CARMIL1;P | . |
| 0 | 0 | 0.684518099 | 0.127938129 | CARMIL1;S | . |
| 0 | 0 | 0.684518099 | 0.127938129 | XRCC5;PDC | . |
| 0 | 0 | 0.684518099 | 0.127938129 | DNAJB2;DN | . |
| 0 | 0 | 0.61622658 | 0.112535661 | IFT122;CEP | . |
| 0 | 0 | 0.61622658 | 0.112535661 | MMP9;MT | . |
| 0 | 0 | 0.61622658 | 0.112535661 | TAB1;MAP | . |
| 0 | 0 | 0.61622658 | 0.112535661 | CUX2;DLG4 | . |
| 0 | 0 | 0.61622658 | 0.112535661 | CAMK2A;H | . |
| 0 | 0 | 0.61622658 | 0.112535661 | TEK;GAS6 | . |
| 0 | 0 | 0.61622658 | 0.112535661 | KANK1;SLC | . |
| 0 | 0 | 0.61622658 | 0.112535661 | FAM210B;I | . |
| 0 | 0 | 0.61622658 | 0.112535661 | MMP14;IG | . |
| 0 | 0 | 0.61622658 | 0.112535661 | SYT2;CDK5 | . |
| 0 | 0 | 0.61622658 | 0.112535661 | PPIH;PPIL3 | . |
| 0 | 0 | 0.61622658 | 0.112535661 | ST3GAL4;A | . |
| 0 | 0 | 0.61622658 | 0.112535661 | RANGRF;AT | . |
| 0 | 0 | 0.699984135 | 0.12721768 | CD84;CADI | . |
| 0 | 0 | 0.699984135 | 0.12721768 | APH1B;GM | . |

| | | | | | |
|---|---|---|---|---|---|
| 0 | 0 | 0.699984135 | 0.12721768 | THUMPD2_ | . |
| 0 | 0 | 0.741945502 | 0.134182364 | CARMIL1;C | . |
| 0 | 0 | 0.741945502 | 0.134182364 | CTSK;HMG | . |
| 0 | 0 | 0.776007208 | 0.139855335 | PSMD10;P | . |
| 0 | 0 | 0.776007208 | 0.139855335 | CPNE9;TRF | . |
| 0 | 0 | 0.710660444 | 0.124475798 | NDUFA11;I | . |
| 0 | 0 | 0.710660444 | 0.124475798 | FEN1;WRN | . |
| 0 | 0 | 0.710660444 | 0.124475798 | PSMD10;P | . |
| 0 | 0 | 0.550304878 | 0.09615053 | MGST2 | . |
| 0 | 0 | 0.550304878 | 0.09615053 | PLPP1 | . |
| 0 | 0 | 0.550304878 | 0.09615053 | COMT | . |
| 0 | 0 | 0.550304878 | 0.09615053 | GSTO1 | . |
| 0 | 0 | 0.550304878 | 0.09615053 | SLC9A1 | . |
| 0 | 0 | 0.550304878 | 0.09615053 | FCHO1 | . |
| 0 | 0 | 0.550304878 | 0.09615053 | CHST14 | . |
| 0 | 0 | 0.550304878 | 0.09615053 | LAMC1 | . |
| 0 | 0 | 0.550304878 | 0.09615053 | RHOBTB2 | . |
| 0 | 0 | 0.550304878 | 0.09615053 | CLASP1 | . |
| 0 | 0 | 0.550304878 | 0.09615053 | ST3GAL5 | . |
| 0 | 0 | 0.550304878 | 0.09615053 | RAB23 | . |
| 0 | 0 | 0.550304878 | 0.09615053 | ALOX5 | . |
| 0 | 0 | 0.550304878 | 0.09615053 | PHF20 | . |
| 0 | 0 | 0.550304878 | 0.09615053 | PHF20 | . |
| 0 | 0 | 0.550304878 | 0.09615053 | LATS1 | . |
| 0 | 0 | 0.550304878 | 0.09615053 | KCNIP2 | . |
| 0 | 0 | 0.550304878 | 0.09615053 | RAP2A | . |
| 0 | 0 | 0.550304878 | 0.09615053 | METTL8 | . |
| 0 | 0 | 0.550304878 | 0.09615053 | MGST2 | . |
| 0 | 0 | 0.550304878 | 0.09615053 | CD9 | . |
| 0 | 0 | 0.550304878 | 0.09615053 | DAB2 | . |
| 0 | 0 | 0.550304878 | 0.09615053 | PARK7 | . |
| 0 | 0 | 0.550304878 | 0.09615053 | PARK7 | . |
| 0 | 0 | 0.550304878 | 0.09615053 | H1-10 | . |
| 0 | 0 | 0.550304878 | 0.09615053 | MGST2 | . |
| 0 | 0 | 0.550304878 | 0.09615053 | PARK7 | . |
| 0 | 0 | 0.550304878 | 0.09615053 | AATK | . |
| 0 | 0 | 0.550304878 | 0.09615053 | NOXA1 | . |
| 0 | 0 | 0.550304878 | 0.09615053 | POT1 | . |
| 0 | 0 | 0.550304878 | 0.09615053 | PLA2R1 | . |
| 0 | 0 | 0.550304878 | 0.09615053 | LGMN | . |
| 0 | 0 | 0.550304878 | 0.09615053 | SMPD3 | . |
| 0 | 0 | 0.550304878 | 0.09615053 | IGF1 | . |
| 0 | 0 | 0.550304878 | 0.09615053 | PARK7 | . |
| 0 | 0 | 0.550304878 | 0.09615053 | TLR5 | . |
| 0 | 0 | 0.550304878 | 0.09615053 | GAS6 | . |
| 0 | 0 | 0.550304878 | 0.09615053 | SCIN | . |
| 0 | 0 | 0.550304878 | 0.09615053 | MPP7 | . |

| | | | | | |
|---|---|---|---|---|---|
| 0 | 0 | 0.550304878 | 0.09615053 | LGMN | . |
| 0 | 0 | 0.550304878 | 0.09615053 | CLASP1 | . |
| 0 | 0 | 0.550304878 | 0.09615053 | SFRP4 | . |
| 0 | 0 | 0.550304878 | 0.09615053 | SCIN | . |
| 0 | 0 | 0.550304878 | 0.09615053 | USP46 | . |
| 0 | 0 | 0.550304878 | 0.09615053 | WNK1 | . |
| 0 | 0 | 0.550304878 | 0.09615053 | SOD1 | . |
| 0 | 0 | 0.550304878 | 0.09615053 | SLC9A1 | . |
| 0 | 0 | 0.550304878 | 0.09615053 | LGMN | . |
| 0 | 0 | 0.794128212 | 0.137599853 | CNTNAP1;I | . |
| 0 | 0 | 0.829532021 | 0.143592081 | BLM;MCFD | . |
| 0 | 0 | 0.641782912 | 0.111006902 | TNFSF12;G | . |
| 0 | 0 | 0.641782912 | 0.111006902 | ADTRP;SEF | . |
| 0 | 0 | 0.641782912 | 0.111006902 | STXBP6;AD | . |
| 0 | 0 | 0.641782912 | 0.111006902 | ZDHHC6;CI | . |
| 0 | 0 | 0.641782912 | 0.111006902 | CAB39;WN | . |
| 0 | 0 | 0.641782912 | 0.111006902 | FZD5;HPS4 | . |
| 0 | 0 | 0.641782912 | 0.111006902 | MMP3;EIF: | . |
| 0 | 0 | 0.669599348 | 0.115759156 | PROS1;CD: | . |
| 0 | 0 | 0.754520435 | 0.130020086 | P2RX7;SYD | . |
| 0 | 0 | 0.762620889 | 0.131285674 | KCNIP2;SL( | . |
| 0 | 0 | 0.762620889 | 0.131285674 | PSMD10;P: | . |
| 0 | 0 | 0.744839838 | 0.127995242 | ACER2;DU: | . |
| 0 | 0 | 0.73307121 | 0.124989058 | SCPEP1;RE | . |
| 0 | 0 | 0.788992682 | 0.130803023 | PDGFB;CIB | . |
| 0 | 0 | 0.59249204 | 0.097675824 | PHF20;MSI | . |
| 0 | 0 | 0.59249204 | 0.097675824 | CD209;AP( | . |
| 0 | 0 | 0.59249204 | 0.097675824 | KANK1;SLC | . |
| 0 | 0 | 0.59249204 | 0.097675824 | PARK7;TXN | . |
| 0 | 0 | 0.59249204 | 0.097675824 | TAB1;MAP | . |
| 0 | 0 | 0.59249204 | 0.097675824 | DLG4;CLST | . |
| 0 | 0 | 0.59249204 | 0.097675824 | CDH3;PARI | . |
| 0 | 0 | 0.59249204 | 0.097675824 | CUX2;CLST | . |
| 0 | 0 | 0.59249204 | 0.097675824 | DLG4;PICK | . |
| 0 | 0 | 0.59249204 | 0.097675824 | TCEA2;HM | . |
| 0 | 0 | 0.59249204 | 0.097675824 | RACGAP1;( | . |
| 0 | 0 | 0.59249204 | 0.097675824 | MYBPC3;N | . |
| 0 | 0 | 0.59249204 | 0.097675824 | PPP1R26;T | . |
| 0 | 0 | 0.59249204 | 0.097675824 | CATSPERD; | . |
| 0 | 0 | 0.59249204 | 0.097675824 | TNFSF18;E | . |
| 0 | 0 | 0.59249204 | 0.097675824 | CITED2;ZFF | . |
| 0 | 0 | 0.792519936 | 0.129896478 | MAPKBP1; | . |
| 0 | 0 | 0.69985318 | 0.114691072 | ACHE;GOLI | . |
| 0 | 0 | 0.822610369 | 0.13254015 | GRN;RIPOF | . |
| 0 | 0 | 0.655315437 | 0.104752841 | SCPEP1;CY | . |
| 0 | 0 | 0.655315437 | 0.104752841 | CUL7;HGF; | . |
| 0 | 0 | 0.655315437 | 0.104752841 | PRKCG;NXI | . |

| | | | | | |
|---|---|---|---|---|---|
| 0 | 0 | 0.655315437 | 0.104752841 | CARMIL1;C | . |
| 0 | 0 | 0.655315437 | 0.104752841 | RUFY3;CHN | . |
| 0 | 0 | 0.655315437 | 0.104752841 | P2RX7;TTC | . |
| 0 | 0 | 0.624402083 | 0.098937175 | EDNRB;BBS | . |
| 0 | 0 | 0.624402083 | 0.098937175 | CRTAM;CC | . |
| 0 | 0 | 0.624402083 | 0.098937175 | C1QTNF1;F | . |
| 0 | 0 | 0.624402083 | 0.098937175 | GSTO1;KCN | . |
| 0 | 0 | 0.624402083 | 0.098937175 | P2RX7;ALC | . |
| 0 | 0 | 0.624402083 | 0.098937175 | TRPM2;HL( | . |
| 0 | 0 | 0.624402083 | 0.098937175 | SYT2;CDK5 | . |
| 0 | 0 | 0.675346744 | 0.106358979 | P2RX7;FZD | . |
| 0 | 0 | 0.729076187 | 0.11187798 | CALCB;RGN | . |
| 0 | 0 | 0.729076187 | 0.11187798 | GLE1;NDC1 | . |
| 0 | 0 | 0.843851313 | 0.129408639 | CD63;DNM | . |
| 0 | 0 | 0.689368522 | 0.105660017 | LATS1;KAN | . |
| 0 | 0 | 0.689368522 | 0.105660017 | CCDC88A;A | . |
| 0 | 0 | 0.51358885 | 0.078587668 | APLN | . |
| 0 | 0 | 0.51358885 | 0.078587668 | SSTR2 | . |
| 0 | 0 | 0.51358885 | 0.078587668 | BBS7 | . |
| 0 | 0 | 0.51358885 | 0.078587668 | LGALS3 | . |
| 0 | 0 | 0.51358885 | 0.078587668 | LGALS3 | . |
| 0 | 0 | 0.51358885 | 0.078587668 | FGD1 | . |
| 0 | 0 | 0.51358885 | 0.078587668 | SYCP3 | . |
| 0 | 0 | 0.51358885 | 0.078587668 | KCNIP2 | . |
| 0 | 0 | 0.51358885 | 0.078587668 | DSCC1 | . |
| 0 | 0 | 0.51358885 | 0.078587668 | PSIP1 | . |
| 0 | 0 | 0.51358885 | 0.078587668 | NAA16 | . |
| 0 | 0 | 0.51358885 | 0.078587668 | TICAM1 | . |
| 0 | 0 | 0.51358885 | 0.078587668 | PDK4 | . |
| 0 | 0 | 0.51358885 | 0.078587668 | MMP9 | . |
| 0 | 0 | 0.51358885 | 0.078587668 | GRN | . |
| 0 | 0 | 0.51358885 | 0.078587668 | EDA | . |
| 0 | 0 | 0.51358885 | 0.078587668 | TNFSF4 | . |
| 0 | 0 | 0.51358885 | 0.078587668 | SHLD2 | . |
| 0 | 0 | 0.51358885 | 0.078587668 | PDCD4 | . |
| 0 | 0 | 0.51358885 | 0.078587668 | TICAM1 | . |
| 0 | 0 | 0.51358885 | 0.078587668 | SMTNL2 | . |
| 0 | 0 | 0.51358885 | 0.078587668 | FSTL3 | . |
| 0 | 0 | 0.51358885 | 0.078587668 | CDC23 | . |
| 0 | 0 | 0.51358885 | 0.078587668 | SIRT2 | . |
| 0 | 0 | 0.51358885 | 0.078587668 | METTL18 | . |
| 0 | 0 | 0.51358885 | 0.078587668 | SEMA3A | . |
| 0 | 0 | 0.715941764 | 0.108378581 | PSMD10;P | . |
| 0 | 0 | 0.715941764 | 0.108378581 | PSMD10;P | . |
| 0 | 0 | 0.862226767 | 0.129016971 | LRSAM1;U | . |
| 0 | 0 | 0.570515614 | 0.08490611 | MSRB2;WA | . |
| 0 | 0 | 0.570515614 | 0.08490611 | PARK7;HDA | . |

| | | | | | |
|---|---|---|---|---|---|
| 0 | 0 | 0.570515614 | 0.08490611 | WWOX;TN | . |
| 0 | 0 | 0.570515614 | 0.08490611 | TNFSF18;L( | . |
| 0 | 0 | 0.570515614 | 0.08490611 | LATS1;ARH | . |
| 0 | 0 | 0.570515614 | 0.08490611 | PARK7;BM | . |
| 0 | 0 | 0.570515614 | 0.08490611 | MMP9;MT | . |
| 0 | 0 | 0.699722112 | 0.103650275 | PSMD10;P: | . |
| 0 | 0 | 0.64162669 | 0.094802588 | UBASH3B;( | . |
| 0 | 0 | 0.64162669 | 0.094802588 | NAXE;VEGI | . |
| 0 | 0 | 0.663665222 | 0.097240272 | LRRC59;AL | . |
| 0 | 0 | 0.607936035 | 0.088220505 | DGKA;LMF | . |
| 0 | 0 | 0.607936035 | 0.088220505 | FHOD3;RN | . |
| 0 | 0 | 0.607936035 | 0.088220505 | MYBPC3;S( | . |
| 0 | 0 | 0.607936035 | 0.088220505 | WNK1;ADT | . |
| 0 | 0 | 0.607936035 | 0.088220505 | P2RX7;ALC | . |
| 0 | 0 | 0.607936035 | 0.088220505 | GNG2;KCN | . |
| 0 | 0 | 0.607936035 | 0.088220505 | GRN;CLOCI | . |
| 0 | 0 | 0.607936035 | 0.088220505 | TRPM2;BC. | . |
| 0 | 0 | 0.721440669 | 0.104560476 | SFRP4;AHI: | . |
| 0 | 0 | 0.679192236 | 0.097324967 | DNAJB2;D/ | . |
| 0 | 0 | 0.679192236 | 0.097324967 | PSMD10;P: | . |
| 0 | 0 | 0.764056161 | 0.106476648 | PSMD10;G | . |
| 0 | 0 | 0.725882964 | 0.100593855 | GRN;TNFSI | . |
| 0 | 0 | 0.713962584 | 0.09769827 | IPO11;INT! | . |
| 0 | 0 | 0.713962584 | 0.09769827 | ENTPD5;H( | . |
| 0 | 0 | 0.713962584 | 0.09769827 | GLE1;NDC: | . |
| 0 | 0 | 0.628496667 | 0.085804127 | TBCD;PARI | . |
| 0 | 0 | 0.628496667 | 0.085804127 | RNASEH2C | . |
| 0 | 0 | 0.628496667 | 0.085804127 | BEX3;HGF; | . |
| 0 | 0 | 0.628496667 | 0.085804127 | STAP1;TRE | . |
| 0 | 0 | 0.628496667 | 0.085804127 | PROS1;CD: | . |
| 0 | 0 | 0.652379685 | 0.088896107 | TMEM161/ | . |
| 0 | 0 | 0.550108932 | 0.073906619 | PKD2;FOLF | . |
| 0 | 0 | 0.550108932 | 0.073906619 | KCNIP2;HC | . |
| 0 | 0 | 0.550108932 | 0.073906619 | SETD4;DPY | . |
| 0 | 0 | 0.550108932 | 0.073906619 | MVB12A;V | . |
| 0 | 0 | 0.550108932 | 0.073906619 | PLA2G12A | . |
| 0 | 0 | 0.550108932 | 0.073906619 | GLMN;MIF | . |
| 0 | 0 | 0.550108932 | 0.073906619 | EIF2AK4;L( | . |
| 0 | 0 | 0.550108932 | 0.073906619 | SHPK;MIF | . |
| 0 | 0 | 0.550108932 | 0.073906619 | KCNMB4;S | . |
| 0 | 0 | 0.550108932 | 0.073906619 | TAF1B;GTF | . |
| 0 | 0 | 0.481462326 | 0.064607544 | SRM | . |
| 0 | 0 | 0.481462326 | 0.064607544 | SRP14 | . |
| 0 | 0 | 0.481462326 | 0.064607544 | CD9 | . |
| 0 | 0 | 0.481462326 | 0.064607544 | MPHOSPH( | . |
| 0 | 0 | 0.481462326 | 0.064607544 | HCN2 | . |
| 0 | 0 | 0.481462326 | 0.064607544 | PKD2 | . |

| | | | | | |
|---|---|---|---|---|---|
| 0 | 0 | 0.481462326 | 0.064607544 | COX15 | . |
| 0 | 0 | 0.481462326 | 0.064607544 | ZFPM1 | . |
| 0 | 0 | 0.481462326 | 0.064607544 | ACADVL | . |
| 0 | 0 | 0.481462326 | 0.064607544 | SIRT2 | . |
| 0 | 0 | 0.481462326 | 0.064607544 | FOLR1 | . |
| 0 | 0 | 0.481462326 | 0.064607544 | ST3GAL4 | . |
| 0 | 0 | 0.481462326 | 0.064607544 | IGF1 | . |
| 0 | 0 | 0.481462326 | 0.064607544 | TICAM1 | . |
| 0 | 0 | 0.481462326 | 0.064607544 | VEGFB | . |
| 0 | 0 | 0.481462326 | 0.064607544 | HOXB4 | . |
| 0 | 0 | 0.481462326 | 0.064607544 | CCNE1 | . |
| 0 | 0 | 0.481462326 | 0.064607544 | PIDD1 | . |
| 0 | 0 | 0.481462326 | 0.064607544 | NOXA1 | . |
| 0 | 0 | 0.481462326 | 0.064607544 | MYO5A | . |
| 0 | 0 | 0.481462326 | 0.064607544 | ATRAID | . |
| 0 | 0 | 0.481462326 | 0.064607544 | STAR | . |
| 0 | 0 | 0.481462326 | 0.064607544 | CARMIL1 | . |
| 0 | 0 | 0.481462326 | 0.064607544 | KCNN4 | . |
| 0 | 0 | 0.481462326 | 0.064607544 | SULT1C4 | . |
| 0 | 0 | 0.669310914 | 0.089632761 | SASH3;GRI | . |
| 0 | 0 | 0.669310914 | 0.089632761 | PSMD10;P | . |
| 0 | 0 | 0.592314399 | 0.078697106 | TADA3;MB | . |
| 0 | 0 | 0.592314399 | 0.078697106 | WWTR1;D/ | . |
| 0 | 0 | 0.845036443 | 0.11204454 | ZMYND15; | . |
| 0 | 0 | 0.764271963 | 0.101191348 | RALBP1;TF | . |
| 0 | 0 | 0.719058147 | 0.094286107 | SCARB2;CA | . |
| 0 | 0 | 0.681930242 | 0.088939328 | ORMDL1;I( | . |
| 0 | 0 | 0.706637113 | 0.091264362 | GLE1;NDC | . |
| 0 | 0 | 0.811660878 | 0.102716394 | ANKRD39;I | . |
| 0 | 0 | 0.615891845 | 0.077664083 | PROS1;CD | . |
| 0 | 0 | 0.838363847 | 0.10544856 | CNTFR;ACI | . |
| 0 | 0 | 0.659711916 | 0.082534373 | SLC26A1;P | . |
| 0 | 0 | 0.659711916 | 0.082534373 | WWTR1;D/ | . |
| 0 | 0 | 0.659711916 | 0.082534373 | TST;UPF3B | . |
| 0 | 0 | 0.577473845 | 0.070227578 | NUCKS1;PI | . |
| 0 | 0 | 0.577473845 | 0.070227578 | FHOD3;OB | . |
| 0 | 0 | 0.577473845 | 0.070227578 | TFR2;DOC2 | . |
| 0 | 0 | 0.531109609 | 0.064411696 | GPD1L;SNT | . |
| 0 | 0 | 0.531109609 | 0.064411696 | MVB12A;V | . |
| 0 | 0 | 0.531109609 | 0.064411696 | FOXJ1;GAS | . |
| 0 | 0 | 0.531109609 | 0.064411696 | MAPKBP1; | . |
| 0 | 0 | 0.531109609 | 0.064411696 | TMEM201; | . |
| 0 | 0 | 0.531109609 | 0.064411696 | HABP4;CIR | . |
| 0 | 0 | 0.531109609 | 0.064411696 | PKD2;SOCS | . |
| 0 | 0 | 0.531109609 | 0.064411696 | SLC9A9;SL( | . |
| 0 | 0 | 0.531109609 | 0.064411696 | CD63;KCNI | . |
| 0 | 0 | 0.453115392 | 0.053384944 | RSRC1 | . |

| | | | | | |
|---|---|---|---|---|---|
| 0 | 0 | 0.453115392 | 0.053384944 | ADTRP | . |
| 0 | 0 | 0.453115392 | 0.053384944 | SSTR2 | . |
| 0 | 0 | 0.453115392 | 0.053384944 | MT1E | . |
| 0 | 0 | 0.453115392 | 0.053384944 | TWIST1 | . |
| 0 | 0 | 0.453115392 | 0.053384944 | TEK | . |
| 0 | 0 | 0.453115392 | 0.053384944 | CRACD | . |
| 0 | 0 | 0.453115392 | 0.053384944 | CDK5 | . |
| 0 | 0 | 0.453115392 | 0.053384944 | PODXL2 | . |
| 0 | 0 | 0.453115392 | 0.053384944 | HACD1 | . |
| 0 | 0 | 0.453115392 | 0.053384944 | CRACD | . |
| 0 | 0 | 0.453115392 | 0.053384944 | DNM1 | . |
| 0 | 0 | 0.453115392 | 0.053384944 | PLA2G12A | . |
| 0 | 0 | 0.453115392 | 0.053384944 | PLA2G12A | . |
| 0 | 0 | 0.453115392 | 0.053384944 | CIB1 | . |
| 0 | 0 | 0.453115392 | 0.053384944 | ATAD5 | . |
| 0 | 0 | 0.453115392 | 0.053384944 | WNK1 | . |
| 0 | 0 | 0.453115392 | 0.053384944 | CIRBP | . |
| 0 | 0 | 0.453115392 | 0.053384944 | BOK | . |
| 0 | 0 | 0.453115392 | 0.053384944 | MMP14 | . |
| 0 | 0 | 0.453115392 | 0.053384944 | NAPEPLD | . |
| 0 | 0 | 0.453115392 | 0.053384944 | SCIN | . |
| 0 | 0 | 0.453115392 | 0.053384944 | CHEK2 | . |
| 0 | 0 | 0.453115392 | 0.053384944 | DAB2 | . |
| 0 | 0 | 0.453115392 | 0.053384944 | ESR1 | . |
| 0 | 0 | 0.453115392 | 0.053384944 | PARK7 | . |
| 0 | 0 | 0.453115392 | 0.053384944 | ITGB8 | . |
| 0 | 0 | 0.453115392 | 0.053384944 | SNTA1 | . |
| 0 | 0 | 0.453115392 | 0.053384944 | GAS6 | . |
| 0 | 0 | 0.453115392 | 0.053384944 | TAF1B | . |
| 0 | 0 | 0.453115392 | 0.053384944 | MYO1G | . |
| 0 | 0 | 0.453115392 | 0.053384944 | POP7 | . |
| 0 | 0 | 0.630918662 | 0.074272576 | ACER2;MM | . |
| 0 | 0 | 0.650383313 | 0.075984565 | HGF;PDGF | . |
| 0 | 0 | 0.603781329 | 0.070298737 | PRKCG;NX | . |
| 0 | 0 | 0.603781329 | 0.070298737 | NSMCE4A; | . |
| 0 | 0 | 0.729792783 | 0.083764375 | BBS2;ZFHX | . |
| 0 | 0 | 0.56335722 | 0.06269004 | WFS1;TMT | . |
| 0 | 0 | 0.56335722 | 0.06269004 | E2F1;CIRB | . |
| 0 | 0 | 0.56335722 | 0.06269004 | UBE2D4;PE | . |
| 0 | 0 | 0.56335722 | 0.06269004 | PDCD4;TEK | . |
| 0 | 0 | 0.751141863 | 0.082560086 | WWTR1;AI | . |
| 0 | 0 | 0.513376906 | 0.056199649 | TWIST1;EM | . |
| 0 | 0 | 0.513376906 | 0.056199649 | CD19;RAS | . |
| 0 | 0 | 0.513376906 | 0.056199649 | GAS6;RAB1 | . |
| 0 | 0 | 0.513376906 | 0.056199649 | HOXB4;FST | . |
| 0 | 0 | 0.513376906 | 0.056199649 | CITED2;PK | . |
| 0 | 0 | 0.513376906 | 0.056199649 | P2RX7;SCN | . |

| | | | | | |
|---|---|---|---|---|---|
| 0 | 0 | 0.513376906 | 0.056199649 | SEMA3A;SI | . |
| 0 | 0 | 0.513376906 | 0.056199649 | CLIP3;IGF1 | . |
| 0 | 0 | 0.513376906 | 0.056199649 | TNFSF4;HN | . |
| 0 | 0 | 0.513376906 | 0.056199649 | SHMT2;OT | . |
| 0 | 0 | 0.513376906 | 0.056199649 | MYH14;LA | . |
| 0 | 0 | 0.513376906 | 0.056199649 | TAF1B;GTF | . |
| 0 | 0 | 0.620707369 | 0.067877843 | DDB1;ACTI | . |
| 0 | 0 | 0.620707369 | 0.067877843 | NEU3;CCD | . |
| 0 | 0 | 0.641313837 | 0.069941583 | LATS1;PKIE | . |
| 0 | 0 | 0.592136603 | 0.063632937 | STX12;RAB | . |
| 0 | 0 | 0.592136603 | 0.063632937 | TOMM7;M | . |
| 0 | 0 | 0.592136603 | 0.063632937 | P2RX7;FZD | . |
| 0 | 0 | 0.656869741 | 0.070579703 | POLA1;CR( | . |
| 0 | 0 | 0.427918118 | 0.0443094 | ACVR1C | . |
| 0 | 0 | 0.427918118 | 0.0443094 | ZFPM1 | . |
| 0 | 0 | 0.427918118 | 0.0443094 | PKD2 | . |
| 0 | 0 | 0.427918118 | 0.0443094 | ZFPM1 | . |
| 0 | 0 | 0.427918118 | 0.0443094 | MT1E | . |
| 0 | 0 | 0.427918118 | 0.0443094 | COMT | . |
| 0 | 0 | 0.427918118 | 0.0443094 | EDEM2 | . |
| 0 | 0 | 0.427918118 | 0.0443094 | FOXJ1 | . |
| 0 | 0 | 0.427918118 | 0.0443094 | GDE1 | . |
| 0 | 0 | 0.427918118 | 0.0443094 | TEK | . |
| 0 | 0 | 0.427918118 | 0.0443094 | PODXL2 | . |
| 0 | 0 | 0.427918118 | 0.0443094 | EIF2AK4 | . |
| 0 | 0 | 0.427918118 | 0.0443094 | FER1L5 | . |
| 0 | 0 | 0.427918118 | 0.0443094 | ADTRP | . |
| 0 | 0 | 0.427918118 | 0.0443094 | PARK7 | . |
| 0 | 0 | 0.427918118 | 0.0443094 | ESR1 | . |
| 0 | 0 | 0.427918118 | 0.0443094 | LGALS3 | . |
| 0 | 0 | 0.427918118 | 0.0443094 | SPAST | . |
| 0 | 0 | 0.427918118 | 0.0443094 | TREM2 | . |
| 0 | 0 | 0.427918118 | 0.0443094 | PRKCA | . |
| 0 | 0 | 0.427918118 | 0.0443094 | TREM2 | . |
| 0 | 0 | 0.427918118 | 0.0443094 | RALBP1 | . |
| 0 | 0 | 0.427918118 | 0.0443094 | CCL19 | . |
| 0 | 0 | 0.427918118 | 0.0443094 | DNAJC15 | . |
| 0 | 0 | 0.427918118 | 0.0443094 | NPHP4 | . |
| 0 | 0 | 0.427918118 | 0.0443094 | EMILIN1 | . |
| 0 | 0 | 0.427918118 | 0.0443094 | SMPD3 | . |
| 0 | 0 | 0.427918118 | 0.0443094 | CLIP3 | . |
| 0 | 0 | 0.427918118 | 0.0443094 | PREPL | . |
| 0 | 0 | 0.427918118 | 0.0443094 | CD63 | . |
| 0 | 0 | 0.427918118 | 0.0443094 | PLD3 | . |
| 0 | 0 | 0.427918118 | 0.0443094 | PKD2 | . |
| 0 | 0 | 0.768483516 | 0.078863526 | SCARB2;CH | . |
| 0 | 0 | 0.678771596 | 0.069324144 | CALCB;RGN | . |

| | | | | | |
|---|---|---|---|---|---|
| 0 | 0 | 0.549912816 | 0.055977703 | SCN11A;KC | . |
| 0 | 0 | 0.648918819 | 0.065315177 | SCN11A;SL | . |
| 0 | 0 | 0.5809313 | 0.05759915 | SLC17A5;S | . |
| 0 | 0 | 0.5809313 | 0.05759915 | UBE2D4;UI | . |
| 0 | 0 | 0.496788249 | 0.049084643 | ABCB9;SEC | . |
| 0 | 0 | 0.496788249 | 0.049084643 | CEMIP2;AL | . |
| 0 | 0 | 0.768381706 | 0.07513256 | INVS;PSMD | . |
| 0 | 0 | 0.704441512 | 0.068508738 | UGDH;CDK | . |
| 0 | 0 | 0.748862025 | 0.071605039 | BBS2;GRN; | . |
| 0 | 0 | 0.623910248 | 0.059224817 | LGALS3;GS | . |
| 0 | 0 | 0.71759965 | 0.067798851 | MYBPC3;C | . |
| 0 | 0 | 0.601242092 | 0.056670763 | HGF;TIMP2 | . |
| 0 | 0 | 0.601242092 | 0.056670763 | ACHE;FZD5 | . |
| 0 | 0 | 0.641157205 | 0.060429404 | PSMD10;P: | . |
| 0 | 0 | 0.680645898 | 0.06359292 | ACVR1C;AI | . |
| 0 | 0 | 0.537093733 | 0.049996825 | UBA5;CDIN | . |
| 0 | 0 | 0.537093733 | 0.049996825 | PLK4;CCDC | . |
| 0 | 0 | 0.537093733 | 0.049996825 | NSMCE4A; | . |
| 0 | 0 | 0.537093733 | 0.049996825 | ZC3H7B;AT | . |
| 0 | 0 | 0.537093733 | 0.049996825 | PI4K2B;ALS | . |
| 0 | 0 | 0.570141009 | 0.052136628 | MEIS1;COL | . |
| 0 | 0 | 0.570141009 | 0.052136628 | FER1L5;TRI | . |
| 0 | 0 | 0.570141009 | 0.052136628 | DTWD1;TY | . |
| 0 | 0 | 0.405373189 | 0.036922577 | MYO1G | . |
| 0 | 0 | 0.405373189 | 0.036922577 | CLDN12 | . |
| 0 | 0 | 0.405373189 | 0.036922577 | PDCD4 | . |
| 0 | 0 | 0.405373189 | 0.036922577 | TMEM65 | . |
| 0 | 0 | 0.405373189 | 0.036922577 | SLC9A1 | . |
| 0 | 0 | 0.405373189 | 0.036922577 | DNAJC18 | . |
| 0 | 0 | 0.405373189 | 0.036922577 | FHOD3 | . |
| 0 | 0 | 0.405373189 | 0.036922577 | TICAM1 | . |
| 0 | 0 | 0.405373189 | 0.036922577 | PARK7 | . |
| 0 | 0 | 0.405373189 | 0.036922577 | ITGB3 | . |
| 0 | 0 | 0.405373189 | 0.036922577 | CNTROB | . |
| 0 | 0 | 0.405373189 | 0.036922577 | FUCA2 | . |
| 0 | 0 | 0.405373189 | 0.036922577 | TUBB | . |
| 0 | 0 | 0.405373189 | 0.036922577 | PPP1R26 | . |
| 0 | 0 | 0.405373189 | 0.036922577 | CHRNB1 | . |
| 0 | 0 | 0.405373189 | 0.036922577 | TENM4 | . |
| 0 | 0 | 0.405373189 | 0.036922577 | SFRP4 | . |
| 0 | 0 | 0.405373189 | 0.036922577 | APLN | . |
| 0 | 0 | 0.405373189 | 0.036922577 | AGO4 | . |
| 0 | 0 | 0.405373189 | 0.036922577 | VGLL4 | . |
| 0 | 0 | 0.405373189 | 0.036922577 | CRTAM | . |
| 0 | 0 | 0.405373189 | 0.036922577 | MIF | . |
| 0 | 0 | 0.405373189 | 0.036922577 | DNAJB2 | . |
| 0 | 0 | 0.405373189 | 0.036922577 | GPNMB | . |

| | | | | | |
|---|---|---|---|---|---|
| 0 | 0 | 0.846221298 | 0.07657871 | ZNF174;TH | . |
| 0 | 0 | 0.687749397 | 0.06220492 | TNFSF18;D | . |
| 0 | 0 | 0.481236383 | 0.042910255 | STX17;ATG | . |
| 0 | 0 | 0.481236383 | 0.042910255 | VAV3;CD19 | . |
| 0 | 0 | 0.481236383 | 0.042910255 | MYBPC3;M | . |
| 0 | 0 | 0.481236383 | 0.042910255 | VEGFB;GAS | . |
| 0 | 0 | 0.481236383 | 0.042910255 | PKD2;MEG | . |
| 0 | 0 | 0.481236383 | 0.042910255 | CCDC88C;I | . |
| 0 | 0 | 0.481236383 | 0.042910255 | ACER2;CIB | . |
| 0 | 0 | 0.481236383 | 0.042910255 | AUTS2;ABI | . |
| 0 | 0 | 0.481236383 | 0.042910255 | TRIP4;KLHI | . |
| 0 | 0 | 0.481236383 | 0.042910255 | ARV1;APO/ | . |
| 0 | 0 | 0.481236383 | 0.042910255 | SYT2;CDK5 | . |
| 0 | 0 | 0.481236383 | 0.042910255 | LSM10;TTF | . |
| 0 | 0 | 0.481236383 | 0.042910255 | TAF1B;GTF | . |
| 0 | 0 | 0.481236383 | 0.042910255 | MYBPC3;M | . |
| 0 | 0 | 0.633578217 | 0.055896021 | TWSG1;DA | . |
| 0 | 0 | 0.633578217 | 0.055896021 | PPP3CA;TN | . |
| 0 | 0 | 0.591958652 | 0.051770569 | LSM10;TAF | . |
| 0 | 0 | 0.591958652 | 0.051770569 | WFS1;ARL3 | . |
| 0 | 0 | 0.591958652 | 0.051770569 | INTS13;INT | . |
| 0 | 0 | 0.754587912 | 0.06552514 | TNFSF18;E | . |
| 0 | 0 | 0.524857335 | 0.044664966 | CITED2;PLF | . |
| 0 | 0 | 0.524857335 | 0.044664966 | KANK1;ALC | . |
| 0 | 0 | 0.559743092 | 0.04719068 | CAMK2A;K | . |
| 0 | 0 | 0.559743092 | 0.04719068 | UBA5;PARI | . |
| 0 | 0 | 0.607422638 | 0.050109559 | CDH3;CAD | . |
| 0 | 0 | 0.607422638 | 0.050109559 | FNTB;CAM | . |
| 0 | 0 | 0.582956529 | 0.047286934 | PPP3CA;PF | . |
| 0 | 0 | 0.582956529 | 0.047286934 | INTS13;INT | . |
| 0 | 0 | 0.676668416 | 0.054728317 | CCDC88A;F | . |
| 0 | 0 | 0.466627055 | 0.037544324 | ARV1;APO/ | . |
| 0 | 0 | 0.466627055 | 0.037544324 | LOXL3;FOX | . |
| 0 | 0 | 0.466627055 | 0.037544324 | KANK1;CCI | . |
| 0 | 0 | 0.466627055 | 0.037544324 | UBASH3B;/ | . |
| 0 | 0 | 0.466627055 | 0.037544324 | TNFSF4;TI( | . |
| 0 | 0 | 0.466627055 | 0.037544324 | CEP97;CCS | . |
| 0 | 0 | 0.466627055 | 0.037544324 | GRIN2C;M/ | . |
| 0 | 0 | 0.466627055 | 0.037544324 | PREPL;SYT: | . |
| 0 | 0 | 0.466627055 | 0.037544324 | LGALS3;KC | . |
| 0 | 0 | 0.466627055 | 0.037544324 | IQSEC2;FAI | . |
| 0 | 0 | 0.385082753 | 0.030875848 | SCIN | . |
| 0 | 0 | 0.385082753 | 0.030875848 | FZD5 | . |
| 0 | 0 | 0.385082753 | 0.030875848 | FZD5 | . |
| 0 | 0 | 0.385082753 | 0.030875848 | TRPM2 | . |
| 0 | 0 | 0.385082753 | 0.030875848 | GUCY2C | . |
| 0 | 0 | 0.385082753 | 0.030875848 | CRELD1 | . |

| | | | | | |
|---|---|---|---|---|---|
| 0 | 0 | 0.385082753 | 0.030875848 | RILPL1 | . |
| 0 | 0 | 0.385082753 | 0.030875848 | ACTN3 | . |
| 0 | 0 | 0.385082753 | 0.030875848 | PHC1 | . |
| 0 | 0 | 0.385082753 | 0.030875848 | APOA4 | . |
| 0 | 0 | 0.385082753 | 0.030875848 | GRIN2C | . |
| 0 | 0 | 0.385082753 | 0.030875848 | ZW10 | . |
| 0 | 0 | 0.385082753 | 0.030875848 | ZW10 | . |
| 0 | 0 | 0.385082753 | 0.030875848 | KLRF2 | . |
| 0 | 0 | 0.385082753 | 0.030875848 | UBASH3B | . |
| 0 | 0 | 0.385082753 | 0.030875848 | GPD1L | . |
| 0 | 0 | 0.385082753 | 0.030875848 | TUT4 | . |
| 0 | 0 | 0.385082753 | 0.030875848 | TNFSF18 | . |
| 0 | 0 | 0.385082753 | 0.030875848 | RUNX2 | . |
| 0 | 0 | 0.385082753 | 0.030875848 | TANC2 | . |
| 0 | 0 | 0.385082753 | 0.030875848 | CORO1C | . |
| 0 | 0 | 0.385082753 | 0.030875848 | CATSPERD | . |
| 0 | 0 | 0.385082753 | 0.030875848 | RETSAT | . |
| 0 | 0 | 0.385082753 | 0.030875848 | ZW10 | . |
| 0 | 0 | 0.385082753 | 0.030875848 | CIRBP | . |
| 0 | 0 | 0.755511962 | 0.059819383 | ZFHX2;GST | . |
| 0 | 0 | 0.683413567 | 0.053572433 | GLP1R;P2R | . |
| 0 | 0 | 0.549716529 | 0.042712022 | TBCD;PARI | . |
| 0 | 0 | 0.549716529 | 0.042712022 | WNK1;CCL | . |
| 0 | 0 | 0.549716529 | 0.042712022 | XPO1;HEAT | . |
| 0 | 0 | 0.59950002 | 0.046077838 | P2RX7;FZD | . |
| 0 | 0 | 0.689214325 | 0.052257134 | WWOX;HG | . |
| 0 | 0 | 0.574223125 | 0.043184774 | SPARC;MA | . |
| 0 | 0 | 0.452877099 | 0.032874796 | GLP1R;AD( | . |
| 0 | 0 | 0.452877099 | 0.032874796 | TWIST1;EN | . |
| 0 | 0 | 0.452877099 | 0.032874796 | RBBP7;TRF | . |
| 0 | 0 | 0.452877099 | 0.032874796 | FER1L5;DN | . |
| 0 | 0 | 0.452877099 | 0.032874796 | TREM2;TIC | . |
| 0 | 0 | 0.452877099 | 0.032874796 | EIF2AK4;TA | . |
| 0 | 0 | 0.452877099 | 0.032874796 | ANKH;TWI | . |
| 0 | 0 | 0.611874752 | 0.044172864 | LSM10;PCE | . |
| 0 | 0 | 0.627847469 | 0.04516193 | PARP1;TBC | . |
| 0 | 0 | 0.501980592 | 0.0356664 | RAB23;CD5 | . |
| 0 | 0 | 0.501980592 | 0.0356664 | PFKL;EIF2B | . |
| 0 | 0 | 0.366724739 | 0.025900965 | CIB1 | . |
| 0 | 0 | 0.366724739 | 0.025900965 | CYP2U1 | . |
| 0 | 0 | 0.366724739 | 0.025900965 | SOD1 | . |
| 0 | 0 | 0.366724739 | 0.025900965 | METTL8 | . |
| 0 | 0 | 0.366724739 | 0.025900965 | FOXJ1 | . |
| 0 | 0 | 0.366724739 | 0.025900965 | GAS6 | . |
| 0 | 0 | 0.366724739 | 0.025900965 | POT1 | . |
| 0 | 0 | 0.366724739 | 0.025900965 | ZW10 | . |
| 0 | 0 | 0.366724739 | 0.025900965 | STAP1 | . |

| | | | | | |
|---|---|---|---|---|---|
| 0 | 0 | 0.366724739 | 0.025900965 | MST1 | . |
| 0 | 0 | 0.366724739 | 0.025900965 | PPP2CB | . |
| 0 | 0 | 0.366724739 | 0.025900965 | CUX2 | . |
| 0 | 0 | 0.366724739 | 0.025900965 | CCL19 | . |
| 0 | 0 | 0.366724739 | 0.025900965 | CCL19 | . |
| 0 | 0 | 0.366724739 | 0.025900965 | HABP4 | . |
| 0 | 0 | 0.366724739 | 0.025900965 | SNTA1 | . |
| 0 | 0 | 0.366724739 | 0.025900965 | XRCC5 | . |
| 0 | 0 | 0.366724739 | 0.025900965 | POT1 | . |
| 0 | 0 | 0.366724739 | 0.025900965 | TIMP2 | . |
| 0 | 0 | 0.366724739 | 0.025900965 | DDB1 | . |
| 0 | 0 | 0.366724739 | 0.025900965 | GNB5 | . |
| 0 | 0 | 0.78802388 | 0.055190195 | KANK1;GRI | . |
| 0 | 0 | 0.565746587 | 0.039431982 | DLG4;CLST | . |
| 0 | 0 | 0.660696222 | 0.045075445 | KCNIP2;SL( | . |
| 0 | 0 | 0.634928145 | 0.042425096 | DNAJB2;D/ | . |
| 0 | 0 | 0.530700633 | 0.034983143 | IRAK1BP1;I | . |
| 0 | 0 | 0.530700633 | 0.034983143 | MAP4K2;T/ | . |
| 0 | 0 | 0.439912854 | 0.028806364 | SMPD3;CL/ | . |
| 0 | 0 | 0.439912854 | 0.028806364 | PARK7;HD/ | . |
| 0 | 0 | 0.439912854 | 0.028806364 | SLC9A9;SL( | . |
| 0 | 0 | 0.439912854 | 0.028806364 | MYH14;KLI | . |
| 0 | 0 | 0.491272329 | 0.03187904 | SCIN;MSRE | . |
| 0 | 0 | 0.491272329 | 0.03187904 | ARHGAP18 | . |
| 0 | 0 | 0.557515745 | 0.035999169 | CLIP3;SPPL | . |
| 0 | 0 | 0.598209607 | 0.037711586 | UCHL1;PD( | . |
| 0 | 0 | 0.350035635 | 0.021789393 | PKD2 | . |
| 0 | 0 | 0.350035635 | 0.021789393 | ADTRP | . |
| 0 | 0 | 0.350035635 | 0.021789393 | CYP2U1 | . |
| 0 | 0 | 0.350035635 | 0.021789393 | TWIST1 | . |
| 0 | 0 | 0.350035635 | 0.021789393 | IGF1 | . |
| 0 | 0 | 0.350035635 | 0.021789393 | MAP2K5 | . |
| 0 | 0 | 0.350035635 | 0.021789393 | MAP2K5 | . |
| 0 | 0 | 0.350035635 | 0.021789393 | CIRBP | . |
| 0 | 0 | 0.350035635 | 0.021789393 | PWP1 | . |
| 0 | 0 | 0.350035635 | 0.021789393 | BEX3 | . |
| 0 | 0 | 0.350035635 | 0.021789393 | PPARG | . |
| 0 | 0 | 0.350035635 | 0.021789393 | LGALS3 | . |
| 0 | 0 | 0.350035635 | 0.021789393 | SLC25A4 | . |
| 0 | 0 | 0.350035635 | 0.021789393 | KDM2B | . |
| 0 | 0 | 0.350035635 | 0.021789393 | TREM2 | . |
| 0 | 0 | 0.350035635 | 0.021789393 | COX15 | . |
| 0 | 0 | 0.350035635 | 0.021789393 | MT1E | . |
| 0 | 0 | 0.350035635 | 0.021789393 | CD68 | . |
| 0 | 0 | 0.52167614 | 0.0316566 | CSTB;TIMP | . |
| 0 | 0 | 0.54952007 | 0.032859422 | CD86;TFRC | . |
| 0 | 0 | 0.762606776 | 0.045454493 | PSMD10;U | . |

| | | | | | |
|---|---|---|---|---|---|
| 0 | 0 | 0.481010244 | 0.028497372 | NAPEPLD;F | . |
| 0 | 0 | 0.481010244 | 0.028497372 | TNFSF4;HN | . |
| 0 | 0 | 0.609094723 | 0.036080329 | USP46;IQS | . |
| 0 | 0 | 0.427668845 | 0.025257727 | GRN;VSIG4 | . |
| 0 | 0 | 0.427668845 | 0.025257727 | DLG4;CLST | . |
| 0 | 0 | 0.427668845 | 0.025257727 | TSPYL2;GLI | . |
| 0 | 0 | 0.427668845 | 0.025257727 | TIPRL;STYX | . |
| 0 | 0 | 0.427668845 | 0.025257727 | ARV1;APO/ | . |
| 0 | 0 | 0.427668845 | 0.025257727 | CITED2;ZFF | . |
| 0 | 0 | 0.672366404 | 0.03787623 | CHRNB1;K( | . |
| 0 | 0 | 0.654108664 | 0.036758273 | PSMD10;SI | . |
| 0 | 0 | 0.512952464 | 0.028643858 | P2RX7;ARV | . |
| 0 | 0 | 0.603088942 | 0.033461295 | SETD4;SPH | . |
| 0 | 0 | 0.541749625 | 0.029988092 | SETD4;SPH | . |
| 0 | 0 | 0.541749625 | 0.029988092 | SFRP4;TWS | . |
| 0 | 0 | 0.541749625 | 0.029988092 | MBNL2;FA | . |
| 0 | 0 | 0.585138599 | 0.032163343 | SLC35A1;S | . |
| 0 | 0 | 0.334797758 | 0.018377531 | SLC1A3 | . |
| 0 | 0 | 0.334797758 | 0.018377531 | NBL1 | . |
| 0 | 0 | 0.334797758 | 0.018377531 | HACD1 | . |
| 0 | 0 | 0.334797758 | 0.018377531 | EIF2AK4 | . |
| 0 | 0 | 0.334797758 | 0.018377531 | APLN | . |
| 0 | 0 | 0.334797758 | 0.018377531 | EIF2AK4 | . |
| 0 | 0 | 0.334797758 | 0.018377531 | CBLB | . |
| 0 | 0 | 0.334797758 | 0.018377531 | CLASP1 | . |
| 0 | 0 | 0.334797758 | 0.018377531 | ZC4H2 | . |
| 0 | 0 | 0.334797758 | 0.018377531 | GDE1 | . |
| 0 | 0 | 0.334797758 | 0.018377531 | AUTS2 | . |
| 0 | 0 | 0.334797758 | 0.018377531 | SASH1 | . |
| 0 | 0 | 0.334797758 | 0.018377531 | ESR1 | . |
| 0 | 0 | 0.334797758 | 0.018377531 | CIB1 | . |
| 0 | 0 | 0.334797758 | 0.018377531 | CREB3L3 | . |
| 0 | 0 | 0.334797758 | 0.018377531 | SLC12A6 | . |
| 0 | 0 | 0.334797758 | 0.018377531 | RANGRF | . |
| 0 | 0 | 0.334797758 | 0.018377531 | MTHFD1 | . |
| 0 | 0 | 0.334797758 | 0.018377531 | VAV3 | . |
| 0 | 0 | 0.334797758 | 0.018377531 | SPHK2 | . |
| 0 | 0 | 0.334797758 | 0.018377531 | CDH3 | . |
| 0 | 0 | 0.334797758 | 0.018377531 | COL5A1 | . |
| 0 | 0 | 0.47116702 | 0.025477061 | COL11A2;I | . |
| 0 | 0 | 0.47116702 | 0.025477061 | UBA5;CDIN | . |
| 0 | 0 | 0.47116702 | 0.025477061 | PPARG;NBI | . |
| 0 | 0 | 0.47116702 | 0.025477061 | STXBP6;SE | . |
| 0 | 0 | 0.562785449 | 0.03019363 | SCARB2;CL | . |
| 0 | 0 | 0.416086675 | 0.02215934 | EDA;ACP4 | . |
| 0 | 0 | 0.416086675 | 0.02215934 | CRTAM;SO | . |
| 0 | 0 | 0.624953342 | 0.032305913 | TBCCD1;AI | . |

| | | | | | |
|---|---|---|---|---|---|
| 0 | 0 | 0.624953342 | 0.032305913 | RNASEH2C | . |
| 0 | 0 | 0.50451481 | 0.025915338 | PDCD4;GD | . |
| 0 | 0 | 0.50451481 | 0.025915338 | SLC1A3;SL( | . |
| 0 | 0 | 0.534195026 | 0.027362598 | CRCP;POLF | . |
| 0 | 0 | 0.461717524 | 0.022778803 | MYBPC3;M | . |
| 0 | 0 | 0.461717524 | 0.022778803 | TNFSF4;TW | . |
| 0 | 0 | 0.320829704 | 0.015535966 | GAS6 | . |
| 0 | 0 | 0.320829704 | 0.015535966 | PKD2L1 | . |
| 0 | 0 | 0.320829704 | 0.015535966 | UBASH3B | . |
| 0 | 0 | 0.320829704 | 0.015535966 | HMGN1 | . |
| 0 | 0 | 0.320829704 | 0.015535966 | WNK1 | . |
| 0 | 0 | 0.320829704 | 0.015535966 | MAPK8IP2 | . |
| 0 | 0 | 0.320829704 | 0.015535966 | RANGRF | . |
| 0 | 0 | 0.320829704 | 0.015535966 | SYT2 | . |
| 0 | 0 | 0.320829704 | 0.015535966 | HAVCR2 | . |
| 0 | 0 | 0.320829704 | 0.015535966 | MMP14 | . |
| 0 | 0 | 0.320829704 | 0.015535966 | CCL19 | . |
| 0 | 0 | 0.320829704 | 0.015535966 | TICAM1 | . |
| 0 | 0 | 0.320829704 | 0.015535966 | FASTKD2 | . |
| 0 | 0 | 0.320829704 | 0.015535966 | GEMIN2 | . |
| 0 | 0 | 0.320829704 | 0.015535966 | SLC9A1 | . |
| 0 | 0 | 0.405114092 | 0.019451572 | CTSK;ATG1 | . |
| 0 | 0 | 0.405114092 | 0.019451572 | ACP4;LGM | . |
| 0 | 0 | 0.405114092 | 0.019451572 | DAG1;CIB1 | . |
| 0 | 0 | 0.405114092 | 0.019451572 | RPS27L;ME | . |
| 0 | 0 | 0.405114092 | 0.019451572 | HOXB4;ME | . |
| 0 | 0 | 0.762735512 | 0.036422397 | CUL7;MAR | . |
| 0 | 0 | 0.640371991 | 0.030340894 | LGALS3;SC | . |
| 0 | 0 | 0.496349338 | 0.023444284 | STIL;IFT12 | . |
| 0 | 0 | 0.787741828 | 0.035219697 | ZMYND15; | . |
| 0 | 0 | 0.566564008 | 0.025285284 | ACHE;FZD5 | . |
| 0 | 0 | 0.566564008 | 0.025285284 | PRKCG;DD | . |
| 0 | 0 | 0.488443088 | 0.021206489 | DDB1;PPIH | . |
| 0 | 0 | 0.394704206 | 0.017083169 | PPP1R16A; | . |
| 0 | 0 | 0.394704206 | 0.017083169 | MDFIC;TAF | . |
| 0 | 0 | 0.394704206 | 0.017083169 | MEIS1;PBX | . |
| 0 | 0 | 0.609969392 | 0.026229216 | CALCB;WF! | . |
| 0 | 0 | 0.307979094 | 0.013161571 | RAPGEF3 | . |
| 0 | 0 | 0.307979094 | 0.013161571 | PKD2 | . |
| 0 | 0 | 0.307979094 | 0.013161571 | GRSF1 | . |
| 0 | 0 | 0.307979094 | 0.013161571 | IGF1 | . |
| 0 | 0 | 0.307979094 | 0.013161571 | MTM1 | . |
| 0 | 0 | 0.307979094 | 0.013161571 | PRKCG | . |
| 0 | 0 | 0.307979094 | 0.013161571 | TUT4 | . |
| 0 | 0 | 0.307979094 | 0.013161571 | IGF1 | . |
| 0 | 0 | 0.307979094 | 0.013161571 | CD1B | . |
| 0 | 0 | 0.307979094 | 0.013161571 | MDFIC | . |

| | | | | | |
|---|---|---|---|---|---|
| 0 | 0 | 0.307979094 | 0.013161571 | SCIN | . |
| 0 | 0 | 0.307979094 | 0.013161571 | CAB39 | . |
| 0 | 0 | 0.307979094 | 0.013161571 | GRSF1 | . |
| 0 | 0 | 0.680879394 | 0.029090985 | CRCP;THRE | . |
| 0 | 0 | 0.580198983 | 0.024688022 | ACER2;ABC | . |
| 0 | 0 | 0.831483427 | 0.035157343 | ZMYND15; | . |
| 0 | 0 | 0.512739965 | 0.020762725 | CD86;PDCI | . |
| 0 | 0 | 0.512739965 | 0.020762725 | SCARB2;SY | . |
| 0 | 0 | 0.591245293 | 0.023822968 | MTHFD1;C | . |
| 0 | 0 | 0.53648757 | 0.021441834 | ITGB3;HGF | . |
| 0 | 0 | 0.480783908 | 0.019180039 | SFRP4;CDH | . |
| 0 | 0 | 0.480783908 | 0.019180039 | WFIKKN1;C | . |
| 0 | 0 | 0.627195999 | 0.024984842 | CD86;IL36A | . |
| 0 | 0 | 0.574743899 | 0.022865711 | PSMD10;P | . |
| 0 | 0 | 0.574743899 | 0.022865711 | PSMD10;P | . |
| 0 | 0 | 0.384814815 | 0.01500999 | SLC12A6;H | . |
| 0 | 0 | 0.384814815 | 0.01500999 | TNFSF4;TR | . |
| 0 | 0 | 0.384814815 | 0.01500999 | LGALS3;MA | . |
| 0 | 0 | 0.296116993 | 0.011171615 | TNFSF4 | . |
| 0 | 0 | 0.296116993 | 0.011171615 | TM7SF3 | . |
| 0 | 0 | 0.296116993 | 0.011171615 | SMC6 | . |
| 0 | 0 | 0.296116993 | 0.011171615 | CLASP1 | . |
| 0 | 0 | 0.296116993 | 0.011171615 | CD19 | . |
| 0 | 0 | 0.296116993 | 0.011171615 | CBLB | . |
| 0 | 0 | 0.296116993 | 0.011171615 | RECQL5 | . |
| 0 | 0 | 0.296116993 | 0.011171615 | SSTR2 | . |
| 0 | 0 | 0.296116993 | 0.011171615 | CCDC88C | . |
| 0 | 0 | 0.505964683 | 0.01893024 | MAPKBP1; | . |
| 0 | 0 | 0.435508546 | 0.016286545 | CUL7;GLM | . |
| 0 | 0 | 0.435508546 | 0.016286545 | EPS8;SHTN | . |
| 0 | 0 | 0.435508546 | 0.016286545 | AHI1;MEIS | . |
| 0 | 0 | 0.473360395 | 0.017345096 | HGF;CCR9; | . |
| 0 | 0 | 0.473360395 | 0.017345096 | ABI3;PDGF | . |
| 0 | 0 | 0.595682371 | 0.021244199 | LATS1;TFR | . |
| 0 | 0 | 0.618706769 | 0.02192165 | CD86;HGF; | . |
| 0 | 0 | 0.581242287 | 0.020567182 | ARMC2;CA | . |
| 0 | 0 | 0.375407833 | 0.013193942 | PRDX1;RAS | . |
| 0 | 0 | 0.375407833 | 0.013193942 | NLRX1;HAV | . |
| 0 | 0 | 0.375407833 | 0.013193942 | TNFSF4;NK | . |
| 0 | 0 | 0.375407833 | 0.013193942 | CITED2;FST | . |
| 0 | 0 | 0.375407833 | 0.013193942 | CDK5;NAA | . |
| 0 | 0 | 0.524235149 | 0.018043491 | ZNHIT1;NA | . |
| 0 | 0 | 0.689080063 | 0.023437595 | KANK1;WV | . |
| 0 | 0 | 0.466161836 | 0.015683687 | CASP9;TMI | . |
| 0 | 0 | 0.466161836 | 0.015683687 | LGALS3;CC | . |
| 0 | 0 | 0.466161836 | 0.015683687 | PPP3CA;FE | . |
| 0 | 0 | 0.591066564 | 0.019792142 | RAP2A;ARI | . |

| | | | | | |
|---|---|---|---|---|---|
| 0 | 0 | 0.285133566 | 0.009499322 | MT1E | . |
| 0 | 0 | 0.285133566 | 0.009499322 | APOA4 | . |
| 0 | 0 | 0.285133566 | 0.009499322 | EIF2AK4 | . |
| 0 | 0 | 0.285133566 | 0.009499322 | MAP2K5 | . |
| 0 | 0 | 0.285133566 | 0.009499322 | PLA2G6 | . |
| 0 | 0 | 0.285133566 | 0.009499322 | ARFIP1 | . |
| 0 | 0 | 0.285133566 | 0.009499322 | FHOD3 | . |
| 0 | 0 | 0.73119983 | 0.024158821 | MAGED1;G | . |
| 0 | 0 | 0.774594646 | 0.025475131 | GMNN;CHI | . |
| 0 | 0 | 0.639899211 | 0.020518783 | NDC1;ARL3 | . |
| 0 | 0 | 0.679973749 | 0.021725341 | MSR1;WW | . |
| 0 | 0 | 0.366448802 | 0.011602094 | PLA2G6;W | . |
| 0 | 0 | 0.366448802 | 0.011602094 | MYBPC3;M | . |
| 0 | 0 | 0.366448802 | 0.011602094 | CCL19;GAS | . |
| 0 | 0 | 0.366448802 | 0.011602094 | BTBD2;CDI | . |
| 0 | 0 | 0.366448802 | 0.011602094 | CDK5;NAA | . |
| 0 | 0 | 0.366448802 | 0.011602094 | APOA4;CD | . |
| 0 | 0 | 0.646549297 | 0.020320209 | SETD4;GRN | . |
| 0 | 0 | 0.419624316 | 0.013024575 | PPP2CB;EP | . |
| 0 | 0 | 0.419624316 | 0.013024575 | DLG4;CAM | . |
| 0 | 0 | 0.571569959 | 0.017737327 | PSMD10;P | . |
| 0 | 0 | 0.459178161 | 0.014179533 | PDCD4;GD | . |
| 0 | 0 | 0.459178161 | 0.014179533 | APH1B;TIN | . |
| 0 | 0 | 0.553906462 | 0.016781347 | PSMD10;P | . |
| 0 | 0 | 0.486667992 | 0.014329979 | XPO1;STRA | . |
| 0 | 0 | 0.274934669 | 0.008090501 | RAPGEF3 | . |
| 0 | 0 | 0.274934669 | 0.008090501 | VASN | . |
| 0 | 0 | 0.274934669 | 0.008090501 | CIB1 | . |
| 0 | 0 | 0.274934669 | 0.008090501 | CD1B | . |
| 0 | 0 | 0.274934669 | 0.008090501 | MT1E | . |
| 0 | 0 | 0.53272515 | 0.015519725 | WFIKKN1;C | . |
| 0 | 0 | 0.35790647 | 0.010205928 | CUX2;CLST | . |
| 0 | 0 | 0.35790647 | 0.010205928 | HABP4;GRS | . |
| 0 | 0 | 0.35790647 | 0.010205928 | TMEM161A | . |
| 0 | 0 | 0.452399887 | 0.012817882 | CUL7;CUX2 | . |
| 0 | 0 | 0.548929664 | 0.015522079 | STX17;MTI | . |
| 0 | 0 | 0.412107672 | 0.011647609 | HGF;TREM | . |
| 0 | 0 | 0.412107672 | 0.011647609 | MSRB2;CKA | . |
| 0 | 0 | 0.480557373 | 0.013054624 | MAPKBP1; | . |
| 0 | 0 | 0.480557373 | 0.013054624 | DDB1;TNFS | . |
| 0 | 0 | 0.726855147 | 0.019641317 | ATP6V1A;E | . |
| 0 | 0 | 0.689699121 | 0.018440911 | PSMD10;C | . |
| 0 | 0 | 0.265439145 | 0.006900954 | CASP8AP2 | . |
| 0 | 0 | 0.265439145 | 0.006900954 | ITGB3 | . |
| 0 | 0 | 0.265439145 | 0.006900954 | PSIP1 | . |
| 0 | 0 | 0.265439145 | 0.006900954 | MMP14 | . |
| 0 | 0 | 0.265439145 | 0.006900954 | APH1B | . |

| | | | | | |
|---|---|---|---|---|---|
| 0 | 0 | 0.265439145 | 0.006900954 | SYT2 | . |
| 0 | 0 | 0.265439145 | 0.006900954 | MYBPC3 | . |
| 0 | 0 | 0.265439145 | 0.006900954 | LOXL3 | . |
| 0 | 0 | 0.265439145 | 0.006900954 | CDK5 | . |
| 0 | 0 | 0.265439145 | 0.006900954 | TICAM1 | . |
| 0 | 0 | 0.265439145 | 0.006900954 | MT1E | . |
| 0 | 0 | 0.445818085 | 0.011585363 | LGALS3;CC | . |
| 0 | 0 | 0.445818085 | 0.011585363 | ACER2;TBC | . |
| 0 | 0 | 0.445818085 | 0.011585363 | EPS8;KANK | . |
| 0 | 0 | 0.349752426 | 0.008980715 | CDK5;MAP | . |
| 0 | 0 | 0.557646487 | 0.014177142 | TWSG1;AC | . |
| 0 | 0 | 0.397851968 | 0.009314824 | HMGB1;PA | . |
| 0 | 0 | 0.341960784 | 0.007904977 | RPS6KA5;R | . |
| 0 | 0 | 0.341960784 | 0.007904977 | CUX2;CLST | . |
| 0 | 0 | 0.256576655 | 0.005894464 | TICAM1 | . |
| 0 | 0 | 0.256576655 | 0.005894464 | KRT10 | . |
| 0 | 0 | 0.256576655 | 0.005894464 | P2RX7 | . |
| 0 | 0 | 0.256576655 | 0.005894464 | WFS1 | . |
| 0 | 0 | 0.391086549 | 0.008329758 | ACER2;TM | . |
| 0 | 0 | 0.46310898 | 0.009857835 | PREPL;HEA | . |
| 0 | 0 | 0.63020595 | 0.013331497 | MAPKBP1; | . |
| 0 | 0 | 0.334507909 | 0.006960037 | NDC1;NUP | . |
| 0 | 0 | 0.334507909 | 0.006960037 | TNFSF4;HN | . |
| 0 | 0 | 0.583164834 | 0.012013419 | CD84;CDH: | . |
| 0 | 0 | 0.612389748 | 0.012562225 | EPHA10;SE | . |
| 0 | 0 | 0.248285939 | 0.005041233 | GTF2H5 | . |
| 0 | 0 | 0.248285939 | 0.005041233 | GTF2H5 | . |
| 0 | 0 | 0.248285939 | 0.005041233 | RUNX2 | . |
| 0 | 0 | 0.248285939 | 0.005041233 | PRKCA | . |
| 0 | 0 | 0.248285939 | 0.005041233 | RAP2A | . |
| 0 | 0 | 0.248285939 | 0.005041233 | TNFSF4 | . |
| 0 | 0 | 0.248285939 | 0.005041233 | METTL18 | . |
| 0 | 0 | 0.327372178 | 0.006129634 | EDNRB;ME | . |
| 0 | 0 | 0.421294111 | 0.007720577 | LGALS3;CC | . |
| 0 | 0 | 0.240513393 | 0.00431665 | RPS6KA5 | . |
| 0 | 0 | 0.240513393 | 0.00431665 | TNFSF4 | . |
| 0 | 0 | 0.62765567 | 0.011112858 | GSTP1;PDC | . |
| 0 | 0 | 0.320533769 | 0.005399597 | SLC1A3;SL( | . |
| 0 | 0 | 0.320533769 | 0.005399597 | GSTP1;EMI | . |
| 0 | 0 | 0.320533769 | 0.005399597 | RPS27L;MF | . |
| 0 | 0 | 0.580130228 | 0.009420472 | PSMD10;V | . |
| 0 | 0 | 0.493401707 | 0.007980421 | PREPL;HEA | . |
| 0 | 0 | 0.62192996 | 0.009880377 | NTNG2;EPI | . |
| 0 | 0 | 0.23321191 | 0.003700317 | MCM8 | . |
| 0 | 0 | 0.23321191 | 0.003700317 | PARK7 | . |
| 0 | 0 | 0.23321191 | 0.003700317 | CTSK | . |
| 0 | 0 | 0.23321191 | 0.003700317 | MYH14 | . |

| | | | | | |
|---|---|---|---|---|---|
| 0 | 0 | 0.671756107 | 0.01060204 | SETD4;CD8 | . |
| 0 | 0 | 0.715323873 | 0.01108178 | ZNF395;M. | . |
| 0 | 0 | 0.410013083 | 0.006296914 | P2RX7;SYT | . |
| 0 | 0 | 0.441716315 | 0.006758861 | ZFP69;GPA | . |
| 0 | 0 | 0.441716315 | 0.006758861 | SFRP4;FZD | . |
| 0 | 0 | 0.366172624 | 0.005325038 | RPS6KA5;R | . |
| 0 | 0 | 0.636387949 | 0.009126472 | DIRAS1;SEI | . |
| 0 | 0 | 0.436672021 | 0.006147113 | GRN;MAGI | . |
| 0 | 0 | 0.226339926 | 0.003175269 | CCL19 | . |
| 0 | 0 | 0.226339926 | 0.003175269 | LSM10 | . |
| 0 | 0 | 0.226339926 | 0.003175269 | LOXL3 | . |
| 0 | 0 | 0.226339926 | 0.003175269 | TICAM1 | . |
| 0 | 0 | 0.30767756 | 0.004192725 | DENND1B; | . |
| 0 | 0 | 0.30767756 | 0.004192725 | SLC27A1;E | . |
| 0 | 0 | 0.219860627 | 0.002727362 | CDH24 | . |
| 0 | 0 | 0.219860627 | 0.002727362 | SPAST | . |
| 0 | 0 | 0.219860627 | 0.002727362 | CTSK | . |
| 0 | 0 | 0.219860627 | 0.002727362 | GAS6 | . |
| 0 | 0 | 0.219860627 | 0.002727362 | POLR3G | . |
| 0 | 0 | 0.219860627 | 0.002727362 | POLR3G | . |
| 0 | 0 | 0.219860627 | 0.002727362 | PRKCA | . |
| 0 | 0 | 0.219860627 | 0.002727362 | SLC9A1 | . |
| 0 | 0 | 0.354865536 | 0.004256501 | CAMK2A;F | . |
| 0 | 0 | 0.551453766 | 0.006448895 | TREML1;DI | . |
| 0 | 0 | 0.53776797 | 0.006111902 | WWTR1;CI | . |
| 0 | 0 | 0.571313408 | 0.006492516 | DTNB;FZD5 | . |
| 0 | 0 | 0.491477501 | 0.005498763 | TNFSF18;P | . |
| 0 | 0 | 0.29581029 | 0.003258091 | MBNL2;RB | . |
| 0 | 0 | 0.213741289 | 0.002344764 | GRIN2C | . |
| 0 | 0 | 0.213741289 | 0.002344764 | IGF1 | . |
| 0 | 0 | 0.422204323 | 0.004618786 | RINT1;RUF | . |
| 0 | 0 | 0.34946897 | 0.003805249 | TCEA2;ELP | . |
| 0 | 0 | 0.34946897 | 0.003805249 | TUT4;UPF3 | . |
| 0 | 0 | 0.578175716 | 0.006239448 | RARS2;EEF | . |
| 0 | 0 | 0.389164601 | 0.00418101 | ALDH3A2;F | . |
| 0 | 0 | 0.518648294 | 0.00529087 | WWOX;MI | . |
| 0 | 0 | 0.518648294 | 0.00529087 | PSMD10;D | . |
| 0 | 0 | 0.207952726 | 0.002017556 | RASGRP1 | . |
| 0 | 0 | 0.207952726 | 0.002017556 | TUT4 | . |
| 0 | 0 | 0.551000958 | 0.005109297 | TNFSF18;P | . |
| 0 | 0 | 0.413077746 | 0.003813496 | RNF113A;F | . |
| 0 | 0 | 0.379512526 | 0.003403728 | CD86;FCRL | . |
| 0 | 0 | 0.339152008 | 0.00304066 | CITED2;PKI | . |
| 0 | 0 | 0.284822077 | 0.002533481 | STX17;FZD | . |
| 0 | 0 | 0.202468825 | 0.001737401 | LGALS1 | . |
| 0 | 0 | 0.556353675 | 0.004644129 | CD86;HGF; | . |
| 0 | 0 | 0.279627649 | 0.002234554 | ASPH;NKD1 | . |

| | | | | | |
|---|---|---|---|---|---|
| 0 | 0 | 0.197266148 | 0.00149728 | SFRP4 | . |
| 0 | 0 | 0.451682507 | 0.003398254 | ACHE;FZD5 | . |
| 0 | 0 | 0.329424586 | 0.002429099 | H1-10;H2B | . |
| 0 | 0 | 0.329424586 | 0.002429099 | NAAA;SLC1 | . |
| 0 | 0 | 0.426669577 | 0.003049985 | WWOX;TW | . |
| 0 | 0 | 0.192323606 | 0.001291266 | KRT10 | . |
| 0 | 0 | 0.465228961 | 0.003089982 | WWTR1;TH | . |
| 0 | 0 | 0.587040485 | 0.003793261 | PSMD10;U | . |
| 0 | 0 | 0.269785575 | 0.001739039 | TNFSF18;IC | . |
| 0 | 0 | 0.361571022 | 0.002251662 | DLG4;CAM | . |
| 0 | 0 | 0.361571022 | 0.002251662 | TWSG1;ITG | . |
| 0 | 0 | 0.530273037 | 0.003132676 | FBXW4;RN | . |
| 0 | 0 | 0.357346423 | 0.002029907 | IGF2BP3;YI | . |
| 0 | 0 | 0.707358706 | 0.003860216 | CD86;TENI | . |
| 0 | 0 | 0.473024835 | 0.00255448 | PSMD10;P | . |
| 0 | 0 | 0.502241907 | 0.002708805 | RBPMS;FAS | . |
| 0 | 0 | 0.183144599 | 9.62E-04 | BRINP1 | . |
| 0 | 0 | 0.183144599 | 9.62E-04 | LHFPL2 | . |
| 0 | 0 | 0.411337844 | 0.00211423 | GLP1R;CAL | . |
| 0 | 0 | 0.178875294 | 8.31E-04 | PKD2 | . |
| 0 | 0 | 0.178875294 | 8.31E-04 | PKD2L1 | . |
| 0 | 0 | 0.178875294 | 8.31E-04 | CBLB | . |
| 0 | 0 | 0.252037573 | 0.001054737 | HOXB4;HO | . |
| 0 | 0 | 0.252037573 | 0.001054737 | TIMP2;SER | . |
| 0 | 0 | 0.252037573 | 0.001054737 | SSTR2;HCR | . |
| 0 | 0 | 0.174800048 | 7.19E-04 | POGLUT1 | . |
| 0 | 0 | 0.174800048 | 7.19E-04 | PIGU | . |
| 0 | 0 | 0.303314138 | 0.001236488 | UPF3B;MA | . |
| 0 | 0 | 0.476416798 | 0.00194044 | LGALS1;GC | . |
| 0 | 0 | 0.247958395 | 9.31E-04 | PPBP;CXCL | . |
| 0 | 0 | 0.247958395 | 9.31E-04 | POLR3G;TF | . |
| 0 | 0 | 0.4164314 | 0.00141386 | XRCC5;GEN | . |
| 0 | 0 | 0.635394879 | 0.002145644 | ZNF395;MA | . |
| 0 | 0 | 0.244008715 | 8.22E-04 | CALCRL;SST | . |
| 0 | 0 | 0.467621871 | 0.001530989 | UGDH;DTN | . |
| 0 | 0 | 0.451203209 | 0.001445037 | MRPL42;M | . |
| 0 | 0 | 0.432220233 | 0.001329776 | SHMT2;ALI | . |
| 0 | 0 | 0.500646842 | 0.00142042 | GLP1R;GRI | . |
| 0 | 0 | 0.75075773 | 0.002107811 | TFRC;PARK | . |
| 0 | 0 | 0.288083043 | 7.87E-04 | MMP14;PC | . |
| 0 | 0 | 0.23647394 | 6.41E-04 | ELP4;GTF2 | . |
| 0 | 0 | 0.486652079 | 0.001299241 | POP7;HELC | . |
| 0 | 0 | 0.160197082 | 4.04E-04 | POLR3G | . |
| 0 | 0 | 0.403815215 | 9.90E-04 | GUCY2C;AI | . |
| 0 | 0 | 0.456383909 | 0.001112807 | GLP1R;MA | . |
| 0 | 0 | 0.232877798 | 5.66E-04 | TNFSF18;IC | . |
| 0 | 0 | 0.648400306 | 0.001539114 | CD86;BLM | . |

| | | | | | |
|---|---|---|---|---|---|
| 0 | 0 | 0.397787482 | 8.27E-04 | SUV39H1;( | . |
| 0 | 0 | 0.277623134 | 5.61E-04 | PRKCA;CCL | . |
| 0 | 0 | 0.534046961 | 0.001028669 | PSMD10;U | . |
| 0 | 0 | 0.577502021 | 0.001020912 | NXT1;THRE | . |
| 0 | 0 | 0.310193078 | 5.21E-04 | GATAD1;SI | . |
| 0 | 0 | 0.339580534 | 5.39E-04 | MAPKBP1; | . |
| 0 | 0 | 0.147840726 | 2.28E-04 | ZFPM1 | . |
| 0 | 0 | 0.147840726 | 2.28E-04 | KRT10 | . |
| 0 | 0 | 0.216416582 | 3.04E-04 | DROSHA;TI | . |
| 0 | 0 | 0.216416582 | 3.04E-04 | FZD5;SIRT2 | . |
| 0 | 0 | 0.264798725 | 3.56E-04 | MBNL2;CIF | . |
| 0 | 0 | 0.210463485 | 2.37E-04 | FRS3;FGFR | . |
| 0 | 0 | 0.35402075 | 3.91E-04 | GLP1R;CAL | . |
| 0 | 0 | 0.258818804 | 2.84E-04 | DSTYK;FRS | . |
| 0 | 0 | 0.35129831 | 3.55E-04 | GLP1R;CAL | . |
| 0 | 0 | 0.137249564 | 1.30E-04 | HEATR3 | . |
| 0 | 0 | 0.458646404 | 3.69E-04 | NFATC3;LC | . |
| 0 | 0 | 0.465866804 | 3.30E-04 | CHRNB1;K( | . |
| 0 | 0 | 0.196916373 | 1.28E-04 | COL5A1;KF | . |
| 0 | 0 | 0.128070557 | 7.46E-05 | TPD52 | . |
| 0 | 0 | 0.1259639 | 6.49E-05 | ARNTL | . |
| 0 | 0 | 0.509530083 | 2.61E-04 | WWTR1;IN | . |
| 0 | 0 | 0.602324608 | 2.57E-04 | CD86;TFRC | . |
| 0 | 0 | 0.714534547 | 2.99E-04 | CD86;CRT( | . |
| 0 | 0 | 0.570545179 | 1.93E-04 | CD86;GRN | . |
| 0 | 0 | 0.376590622 | 1.13E-04 | HELQ;XPO: | . |
| 0 | 0 | 0.26220269 | 7.69E-05 | CAMK2A;C | . |
| 0 | 0 | 0.255604012 | 5.58E-05 | AGBL5;NDI | . |
| 0 | 0 | 0.111308893 | 2.18E-05 | DROSHA | . |
| 0 | 0 | 0.215059195 | 3.69E-05 | CIB1;CCL19 | . |
| 0 | 0 | 0.108161162 | 1.66E-05 | LIMS1 | . |
| 0 | 0 | 0.166818225 | 2.30E-05 | SRP14;SIL1 | . |
| 0 | 0 | 0.209158279 | 2.63E-05 | UPF3B;MA | . |
| 0 | 0 | 0.237649469 | 2.15E-05 | AGBL5;NDI | . |
| 0 | 0 | 0.642717058 | 5.57E-05 | THRB;THR/ | . |
| 0 | 0 | 0.383743351 | 2.53E-05 | RNF113A;F | . |
| 0 | 0 | 0.097164028 | 5.80E-06 | CCL19 | . |
| 0 | 0 | 0.095944033 | 5.10E-06 | ESR1 | . |
| 0 | 0 | 0.421182482 | 2.15E-05 | CHRNB1;K( | . |
| 0 | 0 | 0.278369906 | 1.36E-05 | ERI1;TUT4; | . |
| 0 | 0 | 0.27500477 | 1.13E-05 | HELQ;ERI1 | . |
| 0 | 0 | 0.092460434 | 3.50E-06 | TPD52 | . |
| 0 | 0 | 0.380367172 | 1.24E-05 | GEMIN2;U | . |
| 0 | 0 | 0.270105528 | 8.60E-06 | GSDMB;DF | . |
| 0 | 0 | 0.587998827 | 1.71E-05 | SETD4;CD8 | . |
| 0 | 0 | 0.243800091 | 6.77E-06 | HELQ;POP4 | . |
| 0 | 0 | 0.239187936 | 5.18E-06 | GLP1R;CAL | . |

| | | | | | |
|---|---|---|---|---|---|
| 0 | 0 | 0.086197784 | 1.71E-06 | SRP14 | . |
| 0 | 0 | 0.373447402 | 5.52E-06 | GEMIN2;U | . |
| 0 | 0 | 0.717480168 | 9.07E-06 | ZNF174;SP | . |
| 0 | 0 | 0.522284085 | 6.32E-06 | CD86;PSM | . |
| 0 | 0 | 0.078241663 | 6.95E-07 | CD36 | . |
| 0 | 0 | 0.69285863 | 6.10E-06 | ZNF572;ZN | . |
| 0 | 0 | 0.075156282 | 5.11E-07 | SRP14 | . |
| 0 | 0 | 0.362490205 | 2.04E-06 | CD86;CNTI | . |
| 0 | 0 | 0.287920196 | 1.47E-06 | TREML1;M | . |

**t terms were highlighted)**

| Term | Overlap | P-value | Adjusted P-value | Old P-value |
|---|---|---|---|---|
| Valine, leucine and isoleucine degradation | 26/48 | 6.92E-13 | 2.16E-10 | 0 |
| Fanconi anemia pathway | 26/54 | 2.37E-11 | 3.70E-09 | 0 |
| Lysosome | 40/128 | 1.66E-09 | 1.73E-07 | 0 |
| Glyoxylate and dicarboxylate metabolism | 17/30 | 2.72E-09 | 2.13E-07 | 0 |
| Propanoate metabolism | 18/34 | 4.05E-09 | 2.53E-07 | 0 |
| Fatty acid degradation | 19/43 | 6.70E-08 | 3.49E-06 | 0 |
| Amino sugar and nucleotide sugar metabolis | 20/48 | 1.01E-07 | 4.48E-06 | 0 |
| DNA replication | 16/36 | 6.65E-07 | 2.59E-05 | 0 |
| Base excision repair | 15/33 | 1.04E-06 | 3.62E-05 | 0 |
| Peroxisome | 25/82 | 3.01E-06 | 9.39E-05 | 0 |
| Nucleotide excision repair | 16/47 | 3.97E-05 | 0.001127243 | 0 |
| Fatty acid elongation | 44892 | 1.01E-04 | 0.00260755 | 0 |
| Mismatch repair | 44857 | 1.09E-04 | 0.00260755 | 0 |
| Fructose and mannose metabolism | 12389 | 1.78E-04 | 0.003974324 | 0 |
| Homologous recombination | 13/41 | 4.60E-04 | 0.009575222 | 0 |
| Lysine degradation | 17/63 | 5.61E-04 | 0.01094032 | 0 |
| Tryptophan metabolism | 13/42 | 5.97E-04 | 0.010962981 | 0 |
| Butanoate metabolism | 44862 | 7.26E-04 | 0.012587952 | 0 |
| Riboflavin metabolism | 44689 | 8.26E-04 | 0.013556934 | 0 |
| N-Glycan biosynthesis | 14/50 | 0.001134877 | 0.017704074 | 0 |
| beta-Alanine metabolism | 44864 | 0.001340974 | 0.01992304 | 0 |
| Galactose metabolism | 44865 | 0.001778132 | 0.025217139 | 0 |
| Other glycan degradation | 44760 | 0.002633253 | 0.035720655 | 0 |
| p53 signaling pathway | 17/73 | 0.003220685 | 0.0418689 | 0 |
| Various types of N-glycan biosynthesis | 14550 | 0.003523814 | 0.043977194 | 0 |
| Glutathione metabolism | 14/57 | 0.004320163 | 0.051841956 | 0 |
| Thiamine metabolism | 44727 | 0.004545362 | 0.052524184 | 0 |
| Glycosylphosphatidylinositol (GPI)-anchor bio | 44799 | 0.006957664 | 0.077528261 | 0 |
| Porphyrin and chlorophyll metabolism | 16011 | 0.007871679 | 0.084688407 | 0 |
| Glycerolipid metabolism | 14/61 | 0.008169261 | 0.084960313 | 0 |
| Biosynthesis of unsaturated fatty acids | 44800 | 0.008910146 | 0.08956441 | 0 |
| Primary bile acid biosynthesis | 44729 | 0.009186093 | 0.08956441 | 0 |
| Mannose type O-glycan biosynthesis | 44765 | 0.012132809 | 0.113889055 | 0 |
| Pentose and glucuronate interconversions | 12663 | 0.012410987 | 0.113889055 | 0 |
| Nicotinate and nicotinamide metabolism | 13028 | 0.015045952 | 0.134123914 | 0 |
| Pyruvate metabolism | 17472 | 0.015552186 | 0.134785609 | 0 |
| Glycosaminoglycan degradation | 44731 | 0.016478981 | 0.138957897 | 0 |
| Ascorbate and aldarate metabolism | 44803 | 0.017214411 | 0.141339372 | 0 |
| Sphingolipid metabolism | 18203 | 0.021034866 | 0.165212598 | 0 |
| Purine metabolism | 23/129 | 0.021181102 | 0.165212598 | 0 |
| Pyrimidine metabolism | 20790 | 0.023257035 | 0.176980366 | 0 |
| Ubiquinone and other terpenoid-quinone bio | 44662 | 0.029476725 | 0.218969954 | 0 |
| Glycolysis / Gluconeogenesis | 13/67 | 0.039559098 | 0.287033452 | 0 |
| PPAR signaling pathway | 14/74 | 0.040591451 | 0.287830292 | 0 |

| | | | | |
|---|---|---|---|---|
| Phosphatidylinositol signaling system | 17/97 | 0.049552492 | 0.329236307 | 0 |
| Citrate cycle (TCA cycle) | 44772 | 0.049596495 | 0.329236307 | 0 |
| Pentose phosphate pathway | 44772 | 0.049596495 | 0.329236307 | 0 |
| Non-homologous end-joining | 44664 | 0.053042607 | 0.33857292 | 0 |
| Glycerophospholipid metabolism | 17/98 | 0.053797698 | 0.33857292 | 0 |
| Arginine and proline metabolism | 18537 | 0.05534365 | 0.33857292 | 0 |
| Cholesterol metabolism | 18537 | 0.05534365 | 0.33857292 | 0 |
| Drug metabolism | 18/108 | 0.066817989 | 0.400907933 | 0 |
| Folate biosynthesis | 44738 | 0.069996755 | 0.410871417 | 0 |
| Inositol phosphate metabolism | 13/73 | 0.071112361 | 0.410871417 | 0 |
| Cell cycle | 20/124 | 0.073420554 | 0.416494781 | 0 |
| Gap junction | 15/88 | 0.075875669 | 0.417619971 | 0 |
| Dopaminergic synapse | 21/132 | 0.076295956 | 0.417619971 | 0 |
| Long-term potentiation | 24807 | 0.077976356 | 0.419459017 | 0 |
| Other types of O-glycan biosynthesis | 17411 | 0.084011767 | 0.435764972 | 0 |
| Glioma | 13/75 | 0.084490336 | 0.435764972 | 0 |
| Arachidonic acid metabolism | 22586 | 0.085574976 | 0.435764972 | 0 |
| Fc gamma R-mediated phagocytosis | 16/97 | 0.086594321 | 0.435764972 | 0 |
| Sulfur metabolism | 44630 | 0.097970027 | 0.471441337 | 0 |
| Synthesis and degradation of ketone bodies | 44630 | 0.097970027 | 0.471441337 | 0 |
| Histidine metabolism | 44703 | 0.099727975 | 0.471441337 | 0 |
| Terpenoid backbone biosynthesis | 44703 | 0.099727975 | 0.471441337 | 0 |
| Phagosome | 23/152 | 0.101922979 | 0.474165193 | 0 |
| Ether lipid metabolism | 18142 | 0.103343696 | 0.474165193 | 0 |
| Fat digestion and absorption | 15919 | 0.113720564 | 0.507580744 | 0 |
| Cysteine and methionine metabolism | 18507 | 0.113880295 | 0.507580744 | 0 |
| Selenocompound metabolism | 44668 | 0.122518455 | 0.538390955 | 0 |
| ECM-receptor interaction | 14/88 | 0.129464968 | 0.561014861 | 0 |
| Vitamin B6 metabolism | 44598 | 0.144599043 | 0.612789909 | 0 |
| Mineral absorption | 22190 | 0.145341196 | 0.612789909 | 0 |
| Glycosaminoglycan biosynthesis | 19603 | 0.148845604 | 0.619197714 | 0 |
| Thyroid hormone signaling pathway | 18/121 | 0.151407477 | 0.621567538 | 0 |
| Calcium signaling pathway | 33/240 | 0.157191084 | 0.636930106 | 0 |
| Glycine, serine and threonine metabolism | 14793 | 0.169207185 | 0.676828738 | 0 |
| Cellular senescence | 22/156 | 0.18137285 | 0.716307967 | 0 |
| One carbon pool by folate | 44671 | 0.190676166 | 0.743637047 | 0 |
| GnRH secretion | 23651 | 0.194852164 | 0.750541667 | 0 |
| AMPK signaling pathway | 17/120 | 0.213220912 | 0.811279567 | 0 |
| Circadian entrainment | 14/97 | 0.220261004 | 0.827969074 | 0 |
| Oocyte meiosis | 18/129 | 0.223719734 | 0.830959014 | 0 |
| Starch and sucrose metabolism | 13302 | 0.227110428 | 0.83109689 | 0 |
| Hypertrophic cardiomyopathy | 13/90 | 0.230331798 | 0.83109689 | 0 |
| Aldosterone synthesis and secretion | 14/98 | 0.231748171 | 0.83109689 | 0 |
| Alanine, aspartate and glutamate metabolism | 13667 | 0.24682824 | 0.875118306 | 0 |
| ABC transporters | 16619 | 0.254557502 | 0.882466006 | 0 |
| Glycosphingolipid biosynthesis | 16619 | 0.254557502 | 0.882466006 | 0 |
| Protein export | 44674 | 0.267758063 | 0.918027645 | 0 |

| | | | | |
|---|---|---|---|---|
| Apoptosis | 19/142 | 0.273942893 | 0.929023723 | 0 |
| Sphingolipid signaling pathway | 16/119 | 0.289541528 | 0.971365125 | 0 |
| Vitamin digestion and absorption | 44675 | 0.294628646 | 0.977916358 | 0 |
| Dilated cardiomyopathy | 13/96 | 0.30694585 | 0.999994944 | 0 |
| mTOR signaling pathway | 20/154 | 0.313805307 | 0.999994944 | 0 |
| Cholinergic synapse | 15/113 | 0.315606229 | 0.999994944 | 0 |
| Inflammatory mediator regulation of TRP cha | 13/98 | 0.333913963 | 0.999994944 | 0 |
| Fatty acid biosynthesis | 44638 | 0.342346224 | 0.999994944 | 0 |
| Glucagon signaling pathway | 14/107 | 0.344089645 | 0.999994944 | 0 |
| Thyroid hormone synthesis | 27668 | 0.358369518 | 0.999994944 | 0 |
| Adrenergic signaling in cardiomyocytes | 19/150 | 0.360942311 | 0.999994944 | 0 |
| VEGF signaling pathway | 21763 | 0.365611025 | 0.999994944 | 0 |
| Rap1 signaling pathway | 26/210 | 0.37247049 | 0.999994944 | 0 |
| Pancreatic cancer | 28034 | 0.374370827 | 0.999994944 | 0 |
| Diabetic cardiomyopathy | 25/203 | 0.386640679 | 0.999994944 | 0 |
| Arrhythmogenic right ventricular cardiomyop | 28399 | 0.390438113 | 0.999994944 | 0 |
| Amphetamine addiction | 25447 | 0.396049571 | 0.999994944 | 0 |
| Insulin secretion | 31717 | 0.400394769 | 0.999994944 | 0 |
| Autophagy | 17/137 | 0.405791629 | 0.999994944 | 0 |
| Proteoglycans in cancer | 25/205 | 0.406394417 | 0.999994944 | 0 |
| Endocrine and other factor-regulated calcium | 19541 | 0.408351479 | 0.999994944 | 0 |
| Glutamatergic synapse | 14/114 | 0.437665214 | 0.999994944 | 0 |
| Prostate cancer | 35765 | 0.43840844 | 0.999994944 | 0 |
| Ubiquitin mediated proteolysis | 17/140 | 0.442230217 | 0.999994944 | 0 |
| Melanoma | 26543 | 0.447198382 | 0.999994944 | 0 |
| Non-small cell lung cancer | 26543 | 0.447198382 | 0.999994944 | 0 |
| Focal adhesion | 24/201 | 0.452206231 | 0.999994944 | 0 |
| Choline metabolism in cancer | 36130 | 0.452989061 | 0.999994944 | 0 |
| cGMP-PKG signaling pathway | 20/167 | 0.456728158 | 0.999994944 | 0 |
| HIF-1 signaling pathway | 13/109 | 0.486818653 | 0.999994944 | 0 |
| Salivary secretion | 34274 | 0.506793151 | 0.999994944 | 0 |
| Amyotrophic lateral sclerosis | 42/364 | 0.512002435 | 0.999994944 | 0 |
| Gastric acid secretion | 28004 | 0.514285484 | 0.999994944 | 0 |
| Ferroptosis | 15097 | 0.515146251 | 0.999994944 | 0 |
| Phosphonate and phosphinate metabolism | 44567 | 0.519094735 | 0.999994944 | 0 |
| Vibrio cholerae infection | 18415 | 0.519845256 | 0.999994944 | 0 |
| SNARE interactions in vesicular transport | 12145 | 0.534512503 | 0.999994944 | 0 |
| Phospholipase D signaling pathway | 17/148 | 0.538300278 | 0.999994944 | 0 |
| Long-term depression | 22098 | 0.542381771 | 0.999994944 | 0 |
| Renin secretion | 25416 | 0.544682261 | 0.999994944 | 0 |
| alpha-Linolenic acid metabolism | 44645 | 0.559919776 | 0.999994944 | 0 |
| Vascular smooth muscle contraction | 15/133 | 0.570552944 | 0.999994944 | 0 |
| Ras signaling pathway | 26/232 | 0.584067526 | 0.999994944 | 0 |
| Insulin resistance | 12/108 | 0.593653642 | 0.999994944 | 0 |
| B cell receptor signaling pathway | 29830 | 0.594034926 | 0.999994944 | 0 |
| Tyrosine metabolism | 13241 | 0.605679118 | 0.999994944 | 0 |
| Collecting duct acid secretion | 44647 | 0.613771602 | 0.999994944 | 0 |

| | | | | |
|---|---|---|---|---|
| Regulation of lipolysis in adipocytes | 20241 | 0.616283647 | 0.999994944 | 0 |
| Small cell lung cancer | 33878 | 0.622295557 | 0.999994944 | 0 |
| Sulfur relay system | 44569 | 0.623246824 | 0.999994944 | 0 |
| African trypanosomiasis | 13606 | 0.627868903 | 0.999994944 | 0 |
| Aldosterone-regulated sodium reabsorption | 13606 | 0.627868903 | 0.999994944 | 0 |
| Hedgehog signaling pathway | 20607 | 0.634214733 | 0.999994944 | 0 |
| Pancreatic secretion | 11/102 | 0.634236003 | 0.999994944 | 0 |
| GnRH signaling pathway | 34243 | 0.636211501 | 0.999994944 | 0 |
| Rheumatoid arthritis | 34243 | 0.636211501 | 0.999994944 | 0 |
| Carbohydrate digestion and absorption | 17288 | 0.640350373 | 0.999994944 | 0 |
| Fluid shear stress and atherosclerosis | 15/139 | 0.641103139 | 0.999994944 | 0 |
| Human immunodeficiency virus 1 infection | 23/212 | 0.646873411 | 0.999994944 | 0 |
| Metabolism of xenobiotics by cytochrome P4 | 27973 | 0.656961547 | 0.999994944 | 0 |
| Serotonergic synapse | 12/113 | 0.657589821 | 0.999994944 | 0 |
| Fc epsilon RI signaling pathway | 25020 | 0.67744237 | 0.999994944 | 0 |
| RNA degradation | 29068 | 0.699720075 | 0.999994944 | 0 |
| Viral myocarditis | 22068 | 0.700766989 | 0.999994944 | 0 |
| Oxytocin signaling pathway | 16/154 | 0.702638919 | 0.999994944 | 0 |
| GABAergic synapse | 32752 | 0.706761666 | 0.999994944 | 0 |
| PD-L1 expression and PD-1 checkpoint pathw | 32752 | 0.706761666 | 0.999994944 | 0 |
| Central carbon metabolism in cancer | 25750 | 0.706961756 | 0.999994944 | 0 |
| RNA polymerase | 44651 | 0.70748793 | 0.999994944 | 0 |
| Bladder cancer | 15067 | 0.708326162 | 0.999994944 | 0 |
| Ovarian steroidogenesis | 18749 | 0.71162892 | 0.999994944 | 0 |
| Pantothenate and CoA biosynthesis | 44613 | 0.71277298 | 0.999994944 | 0 |
| Apelin signaling pathway | 14/137 | 0.718495747 | 0.999994944 | 0 |
| Kaposi sarcoma-associated herpesvirus infec | 20/193 | 0.721154727 | 0.999994944 | 0 |
| Progesterone-mediated oocyte maturation | 10/100 | 0.725348372 | 0.999994944 | 0 |
| Neurotrophin signaling pathway | 12/119 | 0.72638358 | 0.999994944 | 0 |
| Arginine biosynthesis | 44614 | 0.736926691 | 0.999994944 | 0 |
| Huntington disease | 32/306 | 0.740697886 | 0.999994944 | 0 |
| Hepatocellular carcinoma | 17/168 | 0.74609982 | 0.999994944 | 0 |
| PI3K-Akt signaling pathway | 37/354 | 0.754399343 | 0.999994944 | 0 |
| Renin-angiotensin system | 44615 | 0.759323757 | 0.999994944 | 0 |
| Prion disease | 28/273 | 0.765968774 | 0.999994944 | 0 |
| C-type lectin receptor signaling pathway | 10/104 | 0.769297369 | 0.999994944 | 0 |
| T cell receptor signaling pathway | 10/104 | 0.769297369 | 0.999994944 | 0 |
| Oxidative phosphorylation | 13/133 | 0.771054668 | 0.999994944 | 0 |
| Cortisol synthesis and secretion | 23894 | 0.771749106 | 0.999994944 | 0 |
| Hepatitis B | 16/162 | 0.775045454 | 0.999994944 | 0 |
| Spinocerebellar ataxia | 14/143 | 0.775139996 | 0.999994944 | 0 |
| Axon guidance | 18/182 | 0.783455481 | 0.999994944 | 0 |
| Parkinson disease | 25/249 | 0.791455961 | 0.999994944 | 0 |
| Salmonella infection | 25/249 | 0.791455961 | 0.999994944 | 0 |
| Osteoclast differentiation | 12/127 | 0.803159403 | 0.999994944 | 0 |
| Synaptic vesicle cycle | 28672 | 0.806447306 | 0.999994944 | 0 |
| Pathways of neurodegeneration | 49/475 | 0.810007311 | 0.999994944 | 0 |

| | | | | |
|---|---|---|---|---|
| Thyroid cancer | 13575 | 0.813871603 | 0.999994944 | 0 |
| Lipid and atherosclerosis | 21/215 | 0.815177369 | 0.999994944 | 0 |
| Retrograde endocannabinoid signaling | 14/148 | 0.815924078 | 0.999994944 | 0 |
| Growth hormone synthesis, secretion and ac | 11/119 | 0.818117885 | 0.999994944 | 0 |
| Relaxin signaling pathway | 12/129 | 0.819648045 | 0.999994944 | 0 |
| Pathogenic Escherichia coli infection | 19/197 | 0.822026746 | 0.999994944 | 0 |
| Primary immunodeficiency | 13940 | 0.827982606 | 0.999994944 | 0 |
| Cocaine addiction | 17989 | 0.829524629 | 0.999994944 | 0 |
| Melanogenesis | 9/101 | 0.833805563 | 0.999994944 | 0 |
| MAPK signaling pathway | 29/294 | 0.833894805 | 0.999994944 | 0 |
| Protein processing in endoplasmic reticulum | 16/171 | 0.840909207 | 0.999994944 | 0 |
| TNF signaling pathway | 10/112 | 0.841708529 | 0.999994944 | 0 |
| Longevity regulating pathway | 9/102 | 0.84211168 | 0.999994944 | 0 |
| Steroid hormone biosynthesis | 22402 | 0.844328862 | 0.999994944 | 0 |
| Toll-like receptor signaling pathway | 9/104 | 0.857742192 | 0.999994944 | 0 |
| Endocytosis | 24/252 | 0.861636406 | 0.999994944 | 0 |
| Linoleic acid metabolism | 47150 | 0.861721227 | 0.999994944 | 0 |
| Wnt signaling pathway | 15/166 | 0.869917329 | 0.999994944 | 0 |
| ErbB signaling pathway | 31229 | 0.870209805 | 0.999994944 | 0 |
| RNA transport | 17/186 | 0.871455367 | 0.999994944 | 0 |
| Phenylalanine metabolism | 44578 | 0.874423701 | 0.999994944 | 0 |
| Circadian rhythm | 11355 | 0.885788035 | 0.999994944 | 0 |
| Tuberculosis | 16/180 | 0.891080991 | 0.999994944 | 0 |
| Hematopoietic cell lineage | 36373 | 0.894283507 | 0.999994944 | 0 |
| Acute myeloid leukemia | 24593 | 0.896411573 | 0.999994944 | 0 |
| Alzheimer disease | 35/369 | 0.905685091 | 0.999994944 | 0 |
| Human papillomavirus infection | 31/331 | 0.907264923 | 0.999994944 | 0 |
| Morphine addiction | 33420 | 0.910054741 | 0.999994944 | 0 |
| Proteasome | 16862 | 0.911113584 | 0.999994944 | 0 |
| Amoebiasis | 8/102 | 0.911451747 | 0.999994944 | 0 |
| Endometrial cancer | 21276 | 0.912611375 | 0.999994944 | 0 |
| Human cytomegalovirus infection | 20/225 | 0.912764167 | 0.999994944 | 0 |
| Steroid biosynthesis | 44581 | 0.912942 | 0.999994944 | 0 |
| cAMP signaling pathway | 19/216 | 0.916248934 | 0.999994944 | 0 |
| Prolactin signaling pathway | 25689 | 0.916258826 | 0.999994944 | 0 |
| Estrogen signaling pathway | 11/137 | 0.926404553 | 0.999994944 | 0 |
| Yersinia infection | 11/137 | 0.926404553 | 0.999994944 | 0 |
| Neutrophil extracellular trap formation | 16/189 | 0.927664715 | 0.999994944 | 0 |
| Mucin type O-glycan biosynthesis | 13181 | 0.929981126 | 0.999994944 | 0 |
| Parathyroid hormone synthesis, secretion an | 8/106 | 0.930594758 | 0.999994944 | 0 |
| Complement and coagulation cascades | 31199 | 0.935529922 | 0.999994944 | 0 |
| Proximal tubule bicarbonate reclamation | 44584 | 0.939648819 | 0.999994944 | 0 |
| mRNA surveillance pathway | 35977 | 0.942823118 | 0.999994944 | 0 |
| Cardiac muscle contraction | 31929 | 0.943938816 | 0.999994944 | 0 |
| Chronic myeloid leukemia | 27881 | 0.946150006 | 0.999994944 | 0 |
| AGE-RAGE signaling pathway in diabetic com | 7/100 | 0.949995427 | 0.999994944 | 0 |
| Bacterial invasion of epithelial cells | 28246 | 0.950067624 | 0.999994944 | 0 |

| | | | | |
|---|---|---|---|---|
| Cushing syndrome | 12/155 | 0.951068804 | 0.999994944 | 0 |
| Regulation of actin cytoskeleton | 18/218 | 0.951674125 | 0.999994944 | 0 |
| Aminoacyl-tRNA biosynthesis | 24198 | 0.953864707 | 0.999994944 | 0 |
| Platelet activation | 9/124 | 0.955214844 | 0.999994944 | 0 |
| Chagas disease | 7/102 | 0.956352056 | 0.999994944 | 0 |
| Protein digestion and absorption | 7/103 | 0.95924923 | 0.999994944 | 0 |
| Thermogenesis | 19/232 | 0.959477141 | 0.999994944 | 0 |
| Pathways in cancer | 49/531 | 0.960747426 | 0.999994944 | 0 |
| Mitophagy | 24929 | 0.960891995 | 0.999994944 | 0 |
| NF-kappa B signaling pathway | 7/104 | 0.961971541 | 0.999994944 | 0 |
| Renal cell carcinoma | 25294 | 0.964020737 | 0.999994944 | 0 |
| Type I diabetes mellitus | 15738 | 0.965464812 | 0.999994944 | 0 |
| Epithelial cell signaling in Helicobacter pylori | 25659 | 0.966915379 | 0.999994944 | 0 |
| Phototransduction | 44589 | 0.967233504 | 0.999994944 | 0 |
| Vasopressin-regulated water reabsorption | 16103 | 0.968835072 | 0.999994944 | 0 |
| Hippo signaling pathway | 12/163 | 0.969014796 | 0.999994944 | 0 |
| Adherens junction | 26024 | 0.969591681 | 0.999994944 | 0 |
| Chemical carcinogenesis | 19/239 | 0.971203954 | 0.999994944 | 0 |
| Alcoholism | 14/186 | 0.971274325 | 0.999994944 | 0 |
| Ribosome biogenesis in eukaryotes | 7/108 | 0.971287561 | 0.999994944 | 0 |
| Basal transcription factors | 16469 | 0.971887105 | 0.999994944 | 0 |
| Notch signaling pathway | 21610 | 0.972132168 | 0.999994944 | 0 |
| Colorectal cancer | 31533 | 0.97525776 | 0.999994944 | 0 |
| Taste transduction | 31533 | 0.97525776 | 0.999994944 | 0 |
| Tight junction | 12/169 | 0.978332131 | 0.999994944 | 0 |
| Toxoplasmosis | 7/112 | 0.978468124 | 0.999994944 | 0 |
| Intestinal immune network for IgA productio | 17564 | 0.979408174 | 0.999994944 | 0 |
| Transcriptional misregulation in cancer | 14/192 | 0.979567595 | 0.999994944 | 0 |
| Necroptosis | 11/159 | 0.979825447 | 0.999994944 | 0 |
| Pertussis | 27851 | 0.980190488 | 0.999994944 | 0 |
| Basal cell carcinoma | 23071 | 0.980816395 | 0.999994944 | 0 |
| Cytosolic DNA-sensing pathway | 23071 | 0.980816395 | 0.999994944 | 0 |
| Leukocyte transendothelial migration | 7/114 | 0.981398994 | 0.999994944 | 0 |
| Influenza A | 12/172 | 0.98196292 | 0.999994944 | 0 |
| Bile secretion | 32994 | 0.982102214 | 0.999994944 | 0 |
| Measles | 9/139 | 0.983229918 | 0.999994944 | 0 |
| Malaria | 18295 | 0.983295453 | 0.999994944 | 0 |
| Human T-cell leukemia virus 1 infection | 16/219 | 0.984794393 | 0.999994944 | 0 |
| Signaling pathways regulating pluripotency o | 9/143 | 0.987275765 | 0.999994944 | 0 |
| Retinol metabolism | 24898 | 0.988076961 | 0.999994944 | 0 |
| Hepatitis C | 10/157 | 0.989133435 | 0.999994944 | 0 |
| Adipocytokine signaling pathway | 25263 | 0.989170407 | 0.999994944 | 0 |
| Chemokine signaling pathway | 13/192 | 0.989532758 | 0.999994944 | 0 |
| Allograft rejection | 13881 | 0.990344919 | 0.999994944 | 0 |
| Breast cancer | 9/147 | 0.990397692 | 0.999994944 | 0 |
| Insulin signaling pathway | 8/137 | 0.991714272 | 0.999994944 | 0 |
| Legionellosis | 20852 | 0.992040669 | 0.999994944 | 0 |

| | | | | |
|---|---|---|---|---|
| Viral protein interaction with cytokine and cy | 5/100 | 0.992249317 | 0.999994944 | 0 |
| Nicotine addiction | 14611 | 0.992438581 | 0.999994944 | 0 |
| Herpes simplex virus 1 infection | 41/498 | 0.993249376 | 0.999994944 | 0 |
| Graft-versus-host disease | 15342 | 0.994078268 | 0.999994944 | 0 |
| Epstein-Barr virus infection | 13/202 | 0.994481831 | 0.999994944 | 0 |
| Viral carcinogenesis | 13/203 | 0.994831776 | 0.999994944 | 0 |
| FoxO signaling pathway | 7/131 | 0.99494062 | 0.999994944 | 0 |
| Natural killer cell mediated cytotoxicity | 7/131 | 0.99494062 | 0.999994944 | 0 |
| Th1 and Th2 cell differentiation | 33695 | 0.99528862 | 0.999994944 | 0 |
| NOD-like receptor signaling pathway | 11/181 | 0.995317772 | 0.999994944 | 0 |
| Antigen processing and presentation | 28550 | 0.99550746 | 0.999994944 | 0 |
| IL-17 signaling pathway | 34425 | 0.996085905 | 0.999994944 | 0 |
| TGF-beta signaling pathway | 34425 | 0.996085905 | 0.999994944 | 0 |
| Type II diabetes mellitus | 16803 | 0.996368 | 0.999994944 | 0 |
| Shigellosis | 16/246 | 0.996995576 | 0.999994944 | 0 |
| Non-alcoholic fatty liver disease | 8/155 | 0.997929032 | 0.999994944 | 0 |
| Autoimmune thyroid disease | 19360 | 0.998455675 | 0.999994944 | 0 |
| Cell adhesion molecules | 7/148 | 0.998739156 | 0.999994944 | 0 |
| Systemic lupus erythematosus | 6/135 | 0.998795662 | 0.999994944 | 0 |
| Gastric cancer | 7/149 | 0.998840718 | 0.999994944 | 0 |
| Th17 cell differentiation | 4/107 | 0.998856341 | 0.999994944 | 0 |
| MicroRNAs in cancer | 20/310 | 0.999036019 | 0.999994944 | 0 |
| Leishmaniasis | 28157 | 0.999095872 | 0.999994944 | 0 |
| Staphylococcus aureus infection | 34759 | 0.999193212 | 0.999994944 | 0 |
| Ribosome | 7/158 | 0.99946055 | 0.999994944 | 0 |
| Inflammatory bowel disease | 23743 | 0.999642145 | 0.999994944 | 0 |
| Spliceosome | 6/150 | 0.999681833 | 0.999994944 | 0 |
| RIG-I-like receptor signaling pathway | 25569 | 0.999804582 | 0.999994944 | 0 |
| JAK-STAT signaling pathway | 6/162 | 0.999892002 | 0.999994944 | 0 |
| Neuroactive ligand-receptor interaction | 18/341 | 0.999972834 | 0.999994944 | 0 |
| Cytokine-cytokine receptor interaction | 14/295 | 0.999980359 | 0.999994944 | 0 |
| Olfactory transduction | 5/440 | 0.999994547 | 0.999994944 | 0 |
| Coronavirus disease | 7/232 | 0.999994944 | 0.999994944 | 0 |

| Old Adjusted P-value | Odds Ratio | Combined Score | Genes |
|---|---|---|---|
| 0 | 9.201112846 | 257.6291864 | ECHS1;ACAT1;MCEE;OXCT1;DBT;ACADM;MMUT;ACAA1 |
| 0 | 7.226992514 | 176.8105473 | BLM;FAAP24;BRCA2;BRIP1;SLX4;REV3L;POLI;PMS2;FAAI |
| 0 | 3.547549039 | 71.71247101 | SCARB2;CD63;IDUA;HEXA;ABCB9;LIPA;GNS;MFSD8;GGA |
| 0 | 10.14608637 | 200.0902188 | MDH1;SHMT2;AMT;HOGA1;ACAT1;GCSH;CS;MCEE;PCC/ |
| 0 | 8.73096753 | 168.7272644 | ACSS3;ECHS1;BCKDHB;ACAT1;HADHA;MCEE;ALDH6A1;F |
| 0 | 6.14393108 | 101.4851526 | GCDH;ACADVL;ECHS1;ECI1;ECI2;ACADSB;CPT1B;ACAT1; |
| 0 | 5.544576197 | 89.33843535 | GPI;GALT;PMM1;HEXA;MPI;NPL;UGDH;GALE;CYB5R3;G |
| 0 | 6.201841298 | 88.21511081 | RFC5;PRIM2;RNASEH2C;FEN1;RFC3;RNASEH2A;RFC4;LIG |
| 0 | 6.458150745 | 88.94471681 | MBD4;FEN1;LIG1;PARP1;PARP2;OGG1;APEX2;XRCC1;HN |
| 0 | 3.406458488 | 43.30805616 | ABCD4;ABCD3;ECI2;HSD17B4;CROT;PEX26;PRDX1;NUD |
| 0 | 3.998698929 | 40.5191681 | RFC5;RFC3;RFC4;LIG1;RFC2;CETN2;XPA;RPA1;XPC;GTF2 |
| 0 | 5.319253062 | 48.95573546 | HADHB;HADHA;HACD1;ECHS1;MECR;ACOT2;PPT1;HACD |
| 0 | 5.950018499 | 54.30818806 | RFC5;MSH6;RFC3;RFC4;LIG1;RFC2;RPA3;POLD2;RPA1;PI |
| 0 | 4.421881838 | 38.16900508 | PFKL;GMDS;PMM1;FCSK;ENOSF1;FPGT;MPI;GMPPA;SO |
| 0 | 3.592929072 | 27.60637658 | BLM;XRCC3;RPA1;BRCA2;PALB2;RAD54B;RAD51B;BRIP1 |
| 0 | 2.862023265 | 21.42428965 | GCDH;ECHS1;SUV39H1;PLOD3;PLOD2;ACAT1;ALDH3A2; |
| 0 | 3.468838698 | 25.74926197 | GCDH;ECHS1;ACAT1;ALDH3A2;HADHA;EHHADH;AFMID; |
| 0 | 4.296020988 | 31.05012112 | HADHA;ECHS1;ALDH5A1;BDH2;OXCT1;EHHADH;L2HGDI |
| 0 | 12.87085515 | 91.37562389 | FLAD1;ACP5;BLVRB;ACP2;ACP1 |
| 0 | 3.009417433 | 20.40755607 | B4GALT2;ALG8;ALG9;ALG6;ALG14;ALG13;MOGS;ALG10 |
| 0 | 3.865981635 | 25.57099115 | ALDH3A2;HADHA;ALDH6A1;ECHS1;EHHADH;CARNS1;M |
| 0 | 3.681679056 | 23.31309911 | B4GALT2;GALE;PFKL;GALT;GLB1;AKR1B1;GALM;GLA;PFI |
| 0 | 4.916395395 | 29.20110325 | NEU3;GLB1;FUCA1;FUCA2;HEXA;HEXD;MAN2C1 |
| 0 | 2.349616228 | 13.482477 | IGF1;DDB2;CASP9;PIDD1;TP53I3;SESN3;RRM2B;AIFM2;( |
| 0 | 3.037510936 | 17.15650406 | B4GALT2;FUT8;ALG9;MGAT4A;HEXA;MGAT4B;HEXD;M/ |
| 0 | 2.518513991 | 13.71195407 | RRM1;GSTM1;GSTO1;GSTP1;IDH1;GSR;GPX7;MGST2;PG |
| 0 | 5.1488433 | 27.77104776 | THTPA;TPK1;AK1;AK7;ACP1;AK8 |
| 0 | 3.433813893 | 17.05888329 | PIGS;PIGU;PGAP1;PIGQ;PIGK;MPPE1;PIGF;PIGY |
| 0 | 2.657220582 | 12.87286238 | FECH;HEPH;COX15;UROS;HMBS;BLVRB;GUSB;CP;HMOX |
| 0 | 2.303650479 | 11.07451596 | DGKA;MOGAT1;AKR1A1;AKR1B1;AGPAT4;ALDH3A2;GP/ |
| 0 | 3.252902899 | 15.35553848 | HACD1;ACOT2;HSD17B4;HACD3;ACAA1;FADS1;ACOT4;H |
| 0 | 4.212213801 | 19.75555457 | CYP27A1;ACOT8;AMACR;HSD17B4;AKR1C4;SLC27A5 |
| 0 | 3.379066594 | 14.90790808 | B4GALT2;B4GAT1;POMT1;CHST10;LARGE1;POMGNT1;F |
| 0 | 2.781503497 | 12.20850053 | UGDH;DCXR;AKR1A1;SORD;AKR1B1;CRYL1;GUSB;XYLB;U |
| 0 | 2.674371302 | 11.22339043 | NADK2;NNT;NMRK1;QPRT;SIRT5;NT5M;NMNAT2;SIRT2; |
| 0 | 2.361439195 | 9.831979829 | ALDH3A2;MDH1;GLO1;AKR1A1;ACYP2;ACYP1;ACSS1;AL |
| 0 | 3.563777994 | 14.63169486 | GALNS;IDUA;GLB1;HEXA;GUSB;GNS |
| 0 | 2.808848644 | 11.40956683 | ALDH3A2;UGDH;AKR1A1;RGN;GUSB;ALDH7A1;UGT1A6; |
| 0 | 2.236899664 | 8.637953384 | SMPD3;ACER2;ARSA;NEU3;SGPL1;SPHK2;GLB1;PLPP3;G |
| 0 | 1.67907519 | 6.472240288 | GUCY2C;RRM1;PDE1B;ENTPD5;AK1;GMPS;ADK;ENTPD8 |
| 0 | 2.107698428 | 7.927364889 | DCTD;RRM1;RRM2B;NME7;ENTPD5;CMPK1;ENTPD8;NN |
| 0 | 4.409943306 | 15.54132076 | COQ3;VKORC1L1;GGCX;COQ6 |
| 0 | 1.860259778 | 6.008563899 | GPI;AKR1A1;ALDH3A2;MINPP1;PFKL;GALM;ALDOA;ACS! |
| 0 | 1.803197547 | 5.777801599 | SLC27A1;CPT1B;CYP27A1;FABP3;FABP5;EHHADH;PLIN2; |

| | | | |
|---|---|---|---|
| 0 | 1.642494518 | 4.935240594 | PRKCG;PI4K2B;MTMR1;DGKA;MTMR14;INPPL1;PRKCA;I |
| 0 | 2.349724701 | 7.058185561 | CS;MDH1;IDH1;SUCLG2;ACO1;SDHB;DLD |
| 0 | 2.349724701 | 7.058185561 | GPI;PFKL;RGN;PGD;ALDOA;PFKM;PGM1 |
| 0 | 3.429568251 | 10.07147516 | FEN1;RAD50;XRCC5;POLL |
| 0 | 1.622124756 | 4.740699496 | ACHE;PLA2G12A;PNPLA7;DGKA;PLA2G6;PLD1;PLD3;AGI |
| 0 | 1.930804547 | 5.58812167 | ALDH3A2;GATM;OAT;P4HA2;CARNS1;HOGA1;L3HYPDH; |
| 0 | 1.930804547 | 5.58812167 | CYP27A1;NCEH1;OSBPL5;STAR;NPC2;APOA4;CD36;ANG |
| 0 | 1.545677929 | 4.182268971 | RRM1;GSTM1;GSTO1;GSTP1;GMPS;MGST2;NME4;FMO |
| 0 | 2.315539066 | 6.157727863 | QDPR;CBR1;SPR;AKR1B1;GGH;PCBD1 |
| 0 | 1.673664623 | 4.424322572 | PI4K2B;MTMR1;MTMR14;INPPL1;MTM1;FIG4;MINPP1;. |
| 0 | 1.486351812 | 3.881684083 | ANAPC13;CDC7;SMC3;PKMYT1;ANAPC10;CDC14B;CDC2 |
| 0 | 1.587468335 | 4.093539839 | PRKCG;TUBB;PDGFB;PRKCA;ITPR3;TUBB4A;TUBA1C;TUE |
| 0 | 1.46230941 | 3.762720019 | PRKCG;CAMK2A;PRKCA;ITPR3;PPP2R5D;CALML4;COMT |
| 0 | 1.685108415 | 4.299300731 | PRKCG;PPP3CA;PPP3CC;RPS6KA2;CAMK2A;PRKCA;ITPR3 |
| 0 | 1.828590817 | 4.529050825 | B4GALT2;GALNT12;GALNT6;POGLUT1;POFUT1;POMT1; |
| 0 | 1.619491837 | 4.001955611 | PRKCG;CAMK2A;PDGFB;PRKCA;PIK3R2;CALML4;IGF1;DI |
| 0 | 1.698888889 | 4.176484526 | CBR1;PLA2G12A;CYP2U1;PTGES2;ALOX5;PRXL2B;GPX7; |
| 0 | 1.526036339 | 3.733480009 | PRKCG;VAV3;SPHK2;INPPL1;ASAP3;PRKCA;PIK3R2;PLA2 |
| 0 | 3.306015693 | 7.680184205 | TST;ETHE1;PAPSS2 |
| 0 | 3.306015693 | 7.680184205 | BDH2;OXCT1;ACAT1 |
| 0 | 2.269530849 | 5.231970002 | ALDH3A2;CARNS1;ALDH7A1;ASPA;ALDH9A1 |
| 0 | 2.269530849 | 5.231970002 | FNTB;PDSS2;ACAT1;ZMPSTE24;PCYOX1 |
| 0 | 1.377901863 | 3.146491067 | ATP6V1A;MSR1;STX12;TFRC;ITGB5;ITGB3;TUBB;MPO;TU |
| 0 | 1.736964598 | 3.942379849 | PLA2G12A;PEDS1;CHPT1;PLPP3;PLA2G6;PLD1;PLA2G7;F |
| 0 | 1.764263871 | 3.835529122 | SLC27A1;PLA2G12A;APOA4;CD36;PLPP3;PLPP1;ACAT1;S |
| 0 | 1.694503667 | 3.681491249 | DNMT1;TST;MDH1;ADI1;DNMT3B;MRI1;KYAT1;GCLM;S |
| 0 | 2.373779731 | 4.983735379 | TXNRD3;TXNRD1;KYAT1;PAPSS2 |
| 0 | 1.460891904 | 2.986566988 | TNXB;ITGB5;VWF;ITGB3;LAMA3;LAMC1;HMMR;SV2C;IT |
| 0 | 3.855991285 | 7.456679646 | PHOSPHO2;PDXP |
| 0 | 1.54376913 | 2.977423094 | SLC46A1;HEPH;SLC31A1;SLC40A1;ATP2B4;ATP2B2;HMC |
| 0 | 1.578701128 | 3.007182092 | B4GALT2;NDST1;FUT8;EXTL1;CHPF;XYLT2;CHST14;EXTL3 |
| 0 | 1.349595505 | 2.547740147 | PRKCG;THRB;THRA;ITGB3;PRKCA;PIK3R2;ATP2A1;ESR1;S |
| 0 | 1.23198648 | 2.279536107 | PDE1B;CAMK2A;PDGFB;ITPR3;ATP2A1;CALML4;CACNA1 |
| 0 | 1.636760619 | 2.907920263 | GCSH;GATM;SHMT2;AMT;ALDH7A1;DLD;GCAT |
| 0 | 1.267895686 | 2.164552047 | HIPK4;NFATC3;ITPR3;PIK3R2;CALML4;LIN9;CCNB3;RBL2 |
| 0 | 1.928368949 | 3.195652057 | MTFMT;MTHFD1;SHMT2;AMT |
| 0 | 1.429091969 | 2.337300128 | PRKCG;AKT3;SPP1;PRKCA;ITPR3;PIK3R2;CACNA1F;KCNN |
| 0 | 1.274058934 | 1.968964446 | CAB39;CAB39L;STRADA;PIK3R2;PPP2R5D;IGF1;CPT1B;PI |
| 0 | 1.301816992 | 1.969573676 | PRKCG;CAMK2A;PRKCA;ITPR3;CALML4;GRIN2C;GNG3;R |
| 0 | 1.251758121 | 1.874334037 | ANAPC13;CAMK2A;ITPR3;PPP2R5D;CALML4;IGF1;SMC3 |
| 0 | 1.542819729 | 2.286950866 | GPI;AMY2B;GBE1;AGL;PYGL;PGM1 |
| 0 | 1.302897628 | 1.912959131 | MYBPC3;ITGB5;ITGB3;TPM2;ATP2A1;IGF1;CACNA1F;SG |
| 0 | 1.286246167 | 1.880625622 | PRKCG;CAMK2A;ATP2B4;PRKCA;ITPR3;ATP2B2;CALML4 |
| 0 | 1.492966869 | 2.088754061 | ALDH5A1;GPT;ASL;ADSS1;RIMKLA;ASPA |
| 0 | 1.420995174 | 1.944246136 | ABCD4;ABCA2;ABCD3;ABCA5;ABCC5;ABCB9;ABCG2 |
| 0 | 1.420995174 | 1.944246136 | B4GALT2;GLB1;B3GALT2;HEXA;ST3GAL4;ST3GAL5;GLA |
| 0 | 1.623614203 | 2.13939009 | IMMP1L;IMMP2L;SRP14;SEC11C |

| | | | |
|---|---|---|---|
| 0 | 1.192102615 | 1.543576922 | DFFA;PARP1;PARP2;ITPR3;PIK3R2;SEPTIN4;CASP9;PIDD: |
| 0 | 1.198588594 | 1.485598479 | PRKCG;SPHK2;PRKCA;PIK3R2;PPP2R5D;PLD1;ACER2;PPP |
| 0 | 1.542346271 | 1.884808131 | SLC46A1;BTD;APOA4;SLC19A1 |
| 0 | 1.208300804 | 1.427104665 | MYBPC3;ITGB5;ITGB3;TPM2;ATP2A1;IGF1;CACNA1F;SG |
| 0 | 1.151620029 | 1.334707492 | PRKCG;ATP6V1A;FZD5;CAB39;CAB39L;STRADA;NPRL2;N |
| 0 | 1.180825091 | 1.361798291 | PRKCG;ACHE;CAMK2A;PRKCA;ITPR3;PIK3R2;CACNA1F;G |
| 0 | 1.179736273 | 1.294019585 | PRKCG;CAMK2A;TRPV2;PRKCA;ITPR3;PIK3R2;CALML4;IG |
| 0 | 1.542109852 | 1.653037983 | MECR;OXSM;HSD17B8 |
| 0 | 1.161177285 | 1.238805539 | CAMK2A;ITPR3;PHKB;CALML4;PYGL;CPT1B;PPP3CA;PFK |
| 0 | 1.186505667 | 1.217581024 | PRKCG;CREB3L3;GSR;GPX7;PRKCA;ITPR3;TTF2;PLCB2;DU |
| 0 | 1.118793102 | 1.14009172 | TPM2;CAMK2A;ATP2B4;PRKCA;ATP2A1;PPP2R5D;ATP2I |
| 0 | 1.20967286 | 1.217155032 | CASP9;PRKCG;PPP3CA;PPP3CC;SPHK2;AKT3;PRKCA;PIK3 |
| 0 | 1.090053223 | 1.076533801 | ITGB3;PDGFB;LPAR2;PARD6G;PIK3R2;CALML4;SIPA1L2;I |
| 0 | 1.168462058 | 1.148023852 | CASP9;RALBP1;RAD51;CDK4;AKT3;E2F1;PIK3R2;BRCA2;I |
| 0 | 1.083352291 | 1.0294658 | NDUFA11;CAMK2A;NDUFA4L2;PIK3R2;ATP2A1;MPC1;A |
| 0 | 1.150957064 | 1.082458781 | ITGB5;SGCA;ACTN3;SGCB;ITGB3;ITGA10;DAG1;ITGB8;AT |
| 0 | 1.15666521 | 1.071321704 | PRKCG;PPP3CA;PPP3CC;CREB3L3;CAMK2A;PRKCA;CALM |
| 0 | 1.130988626 | 1.035198748 | GLP1R;PRKCG;CREB3L3;CAMK2A;KCNMB4;PRKCA;ITPR3 |
| 0 | 1.092510965 | 0.985352551 | STX17;MTMR14;ATG10;PIK3R2;EIF2AK4;HMGB1;ATG14 |
| 0 | 1.071192782 | 0.964535317 | CD63;ITGB5;ITGB3;CAMK2A;TWIST1;PIK3R2;ITPR3;SLC9 |
| 0 | 1.173333966 | 1.050869588 | PRKCG;ATP2B4;PRKCA;ATP2B2;PLCB2;ESR1;DNM1 |
| 0 | 1.079465615 | 0.891963532 | PRKCG;SLC1A3;PRKCA;ITPR3;PLD1;GRIN2C;PPP3CA;GN( |
| 0 | 1.088510748 | 0.897590633 | CASP9;CREB3L3;CCNE1;PDGFD;GSTP1;AKT3;MMP3;PDG |
| 0 | 1.065682499 | 0.869516653 | UBE2F;ANAPC13;CUL7;UBE2D4;FANCL;WWP1;CBLB;AN/ |
| 0 | 1.101398601 | 0.886353802 | CDK4;HGF;PDGFD;AKT3;PDGFB;E2F1;PIK3R2;IGF1;DDB2 |
| 0 | 1.101398601 | 0.886353802 | CASP9;PRKCG;CDK4;HGF;AKT3;E2F1;PRKCA;PIK3R2;DDE |
| 0 | 1.045493524 | 0.82972137 | PRKCG;VAV3;TNXB;ITGB5;VWF;HGF;ITGB3;LAMA3;PDG |
| 0 | 1.075792581 | 0.851906485 | PRKCG;SLC22A5;DGKA;PDGFD;AKT3;PDGFB;PRKCA;PIK3 |
| 0 | 1.04899931 | 0.822066043 | MEF2B;NFATC3;ATP2B4;ITPR3;ATP2A1;ATP2B2;CALML4 |
| 0 | 1.043905976 | 0.751469915 | PRKCG;TFRC;CAMK2A;PRKCA;PIK3R2;IGF1;PFKL;AKT3;TI |
| 0 | 1.034030045 | 0.702780945 | PRKCG;AMY2B;ATP2B4;PRKCA;ITPR3;ATP2B2;CALML4;K |
| 0 | 1.005360069 | 0.673014067 | DCTN5;NXT1;NDUFA11;NDUFA4L2;ITPR3;DNAH9;HDAC |
| 0 | 1.035408621 | 0.688522662 | PRKCG;CAMK2A;PRKCA;ITPR3;CALML4;SSTR2;PLCB2;CA |
| 0 | 1.070571553 | 0.71011486 | TFRC;SLC40A1;GCLM;CP;FTL |
| 0 | 1.541637631 | 1.010803816 | CHPT1 |
| 0 | 1.051089242 | 0.68764791 | ATP6V1A;ATP6V1H;PRKCA;KDELR3;ATP6V0E2;ATP6V0A |
| 0 | 1.063145706 | 0.665954638 | STX17;STX8;VAMP4;BET1 |
| 0 | 1.000147315 | 0.619429975 | SPHK2;DGKA;PDGFB;LPAR2;PRKCA;PIK3R2;PLD1;DNM1; |
| 0 | 1.017961605 | 0.622773794 | PRKCG;PPP2CB;PRKCA;ITPR3;IGF1;PLCB2;PRKG1 |
| 0 | 1.010792887 | 0.614109909 | PPP3CA;PPP3CC;PDE1B;ITPR3;CALML4;CACNA1F;PLCB2 |
| 0 | 1.05102243 | 0.609552821 | PLA2G12A;PLA2G6;ACAA1 |
| 0 | 0.979571146 | 0.549685675 | PRKCG;PLA2G12A;CALCB;CALCRL;PRKCA;ITPR3;CALML4 |
| 0 | 0.972417095 | 0.522906281 | PDGFB;PIK3R2;CALML4;PLA2G6;PLD1;EFNA4;RASGRP1; |
| 0 | 0.963183807 | 0.502261081 | SLC27A1;CREB3L3;PPP1R3C;TBC1D4;RPS6KA2;AKT3;PIK |
| 0 | 0.96323208 | 0.5016678 | VAV3;PPP3CA;PPP3CC;CD19;AKT3;INPPL1;NFATC3;PIK3 |
| 0 | 0.963312255 | 0.483009524 | GSTZ1;MIF;COMT;FAH |
| 0 | 0.963328248 | 0.470231733 | ATP6V1A;ATP6V0E2;ATP6V0A1 |

| | | | |
|---|---|---|---|
| 0 | 0.943567999 | 0.456732161 | AKT3;PIK3R2;ABHD5;PRKG1;PTGS1;PNPLA2 |
| 0 | 0.939616283 | 0.445697707 | CASP9;CCNE1;CDK4;AKT3;LAMA3;CKS2;E2F1;PIK3R2;LA |
| 0 | 1.101045296 | 0.520588146 | TST |
| 0 | 0.934068112 | 0.434737613 | PRKCG;PRKCA;PLCB2;THOP1 |
| 0 | 0.934068112 | 0.434737613 | PRKCG;PIK3R2;PRKCA;IGF1 |
| 0 | 0.92464426 | 0.421053117 | KIF3A;SUFU;IQCE;GAS1;EFCAB7;MEGF8 |
| 0 | 0.931287435 | 0.424046972 | CPA3;PRKCG;PLA2G12A;AMY2B;ATP2B4;PRKCA;ITPR3;A |
| 0 | 0.928242924 | 0.419773934 | MMP14;CAMK2A;PRKCA;ITPR3;CALML4;CACNA1F;PLD1 |
| 0 | 0.928242924 | 0.419773934 | CD86;ATP6V1A;CTSK;MMP3;ACP5;ATP6V1H;TEK;ATP6V |
| 0 | 0.917321117 | 0.408886526 | AMY2B;AKT3;PIK3R2;SLC37A4;PLCB2 |
| 0 | 0.931854485 | 0.414269825 | GSTM1;GSTO1;ITGB3;GSTP1;PDGFB;MGST2;PIK3R2;CAL |
| 0 | 0.9372618 | 0.408275607 | PRKCG;AP1B1;NFATC3;PRKCA;PIK3R2;ITPR3;CALML4;DD |
| 0 | 0.906380901 | 0.380797617 | CBR1;GSTM1;GSTA4;GSTO1;GSTP1;MGST2;CBR3;UGT1A |
| 0 | 0.915241459 | 0.383645345 | PRKCG;GNG3;GNG2;ALOX5;GNB4;PRKCA;ITPR3;GNB5;C |
| 0 | 0.884057556 | 0.344279235 | VAV3;ALOX5;AKT3;PRKCA;PIK3R2;LAT;MAP2K6 |
| 0 | 0.867935441 | 0.309917975 | PFKL;EXOSC9;DIS3L;LSM5;LSM4;PFKM;LSM3;MPHOSPH |
| 0 | 0.855958097 | 0.304361448 | CD86;CASP9;SGCA;SGCB;DAG1;MYH7 |
| 0 | 0.892819702 | 0.315086918 | PRKCG;CAMK2A;NFATC3;PRKCA;ITPR3;CALML4;CACNA1 |
| 0 | 0.866515516 | 0.300734414 | PRKCG;GNG3;GNG2;PLCL1;GNB4;SLC6A12;PRKCA;GNB5 |
| 0 | 0.866515516 | 0.300734414 | PPP3CA;PPP3CC;AKT3;NFATC3;PIK3R2;TICAM1;RASGRP |
| 0 | 0.855895197 | 0.296806231 | PFKL;IDH1;AKT3;PIK3R2;PFKM;PDK1;SIRT3 |
| 0 | 0.825523104 | 0.285659648 | POLR3GL;POLR3G;POLR3H |
| 0 | 0.832899188 | 0.287225794 | RPS6KA5;CDK4;E2F1;MMP9 |
| 0 | 0.837364368 | 0.284870256 | STAR;ALOX5;ACOT2;IGF1;ACOT4 |
| 0 | 0.811099645 | 0.274632103 | ALDH3A2;PPCDC |
| 0 | 0.876467635 | 0.28975625 | MEF2B;SPHK2;ITPR3;CALML4;SLC9A1;APLN;GNG3;MRA |
| 0 | 0.890026173 | 0.290950948 | CD86;PDGFB;NFATC3;PIK3R2;ITPR3;CALML4;ATG14;TIC |
| 0 | 0.855706165 | 0.274770011 | CCNB3;ANAPC13;CDC23;RPS6KA2;AKT3;PIK3R2;IGF1;PK |
| 0 | 0.863625023 | 0.276081106 | BEX3;RPS6KA5;MAGED1;RPS6KA2;AKT3;CAMK2A;PSEN |
| 0 | 0.770501089 | 0.235208449 | GPT;ASL |
| 0 | 0.898677108 | 0.269749121 | DCTN5;NDUFA11;NDUFA4L2;SLC1A3;DNAH9;CASP9;TU |
| 0 | 0.866689904 | 0.253849903 | PRKCG;GSTM1;FZD5;TXNRD3;GSTO1;HGF;GSTP1;TXNRD |
| 0 | 0.897911839 | 0.253061563 | TNXB;ITGB5;PKN3;ITGB3;LAMA3;PDGFB;LPAR2;PIK3R2; |
| 0 | 0.733769063 | 0.202026461 | CPA3;THOP1 |
| 0 | 0.879330101 | 0.234441605 | NDUFA11;NDUFA4L2;PIK3R2;ITPR3;LAMC1;CACNA1F;C |
| 0 | 0.819107071 | 0.21483351 | PPP3CA;PPP3CC;MRAS;CD209;AKT3;NFATC3;CBLB;ITPR |
| 0 | 0.819107071 | 0.21483351 | VAV3;PPP3CA;PPP3CC;CDK4;AKT3;NFATC3;CBLB;PIK3R |
| 0 | 0.833986427 | 0.216833137 | ATP6V1A;NDUFA11;NDUFA5;COX15;NDUFA4L2;NDUFC |
| 0 | 0.783197331 | 0.202923118 | NCEH1;CREB3L3;STAR;ITPR3;CACNA1F;PLCB2 |
| 0 | 0.84351372 | 0.214955639 | PRKCG;NFATC3;PRKCA;PIK3R2;TICAM1;MMP9;DDB2;CA |
| 0 | 0.835416476 | 0.212790289 | PRKCG;BEAN1;PRKCA;ITPR3;PIK3R2;ATP2A1;ATG14;GRI |
| 0 | 0.844674065 | 0.206135137 | NTNG2;SEMA3A;CAMK2A;SEMA4C;NFATC3;PARD6G;PR |
| 0 | 0.858618508 | 0.200814591 | NDUFA11;CAMK2A;NDUFA4L2;ITPR3;CALML4;TXN;PAR |
| 0 | 0.858618508 | 0.200814591 | DCTN5;CSE1L;GCC2;TXN;ACTR3B;STX10;TUBA1C;TUBA1 |
| 0 | 0.803181429 | 0.176059036 | PPP3CA;PPP3CC;ITGB3;CTSK;AKT3;ACP5;PIK3R2;TREM2 |
| 0 | 0.759111876 | 0.163297657 | ATP6V1A;SLC6A12;SLC1A3;ATP6V1H;ATP6V0E2;DNM1; |
| 0 | 0.884012706 | 0.18627209 | NDUFA11;ATP2A1;PARK7;CALML4;CASP9;TUBA1C;PPP3 |

| | | | |
|---|---|---|---|
| 0 | 0.679611775 | 0.139967854 | CCDC6;PPARG;DDB2 |
| 0 | 0.832734676 | 0.170168964 | VAV3;MMP3;CAMK2A;NFATC3;MIB2;PRKCA;PIK3R2;AP( |
| 0 | 0.804015403 | 0.163564045 | PRKCG;NDUFA11;NDUFA5;NDUFA4L2;PRKCA;ITPR3;NDU |
| 0 | 0.783938466 | 0.157374737 | PRKCG;CREB3L3;AKT3;PRKCA;ITPR3;PIK3R2;IGF1;CACN/ |
| 0 | 0.78936206 | 0.156988519 | GNG3;EDNRB;GNG2;CREB3L3;AKT3;GNB4;VEGFB;PRKC/ |
| 0 | 0.821179134 | 0.160936613 | TUBB;LPAR2;MYO5A;BAIAP2;ACTR3B;TUBB4A;CASP9;TU |
| 0 | 0.660156931 | 0.12461329 | CD19;RFXAP;UNG |
| 0 | 0.684518099 | 0.127938129 | CREB3L3;CDK5;DLG4;GRIN2C |
| 0 | 0.752978679 | 0.136857672 | PRKCG;EDNRB;CREB3L3;FZD5;CAMK2A;PRKCA;CALML4; |
| 0 | 0.841406276 | 0.152839782 | PDGFB;CACNA1F;EFNA4;RASGRP1;RASGRP3;PPP3CA;RP |
| 0 | 0.794128212 | 0.137599853 | UBE2D4;WFS1;EDEM2;SSR3;MOGS;EIF2AK4;DNAJB2;DN |
| 0 | 0.754520435 | 0.130020086 | MMP14;RPS6KA5;CREB3L3;AKT3;MMP3;PIK3R2;TAB1;N |
| 0 | 0.744839838 | 0.127995242 | CREB3L3;SESN3;SESN1;AKT3;CAT;PIK3R2;PPARG;IGF1;S( |
| 0 | 0.687445462 | 0.116324856 | SRD5A1;AKR1C4;COMT;HSD17B8;UGT1A6 |
| 0 | 0.729076187 | 0.11187798 | CD86;CTSK;AKT3;SPP1;PIK3R2;TAB1;TICAM1;TLR5;MAP |
| 0 | 0.809271308 | 0.120518221 | TFRC;IQSEC2;ARAP2;ASAP3;AGAP1;WWP1;VPS37D;PAR |
| 0 | 0.570515614 | 0.08490611 | PLA2G12A;PLA2G6 |
| 0 | 0.764056161 | 0.106476648 | PRKCG;INVS;RNF43;FZD5;CAMK2A;NFATC3;PRKCA;NKD: |
| 0 | 0.690712126 | 0.09602345 | PRKCG;AKT3;CAMK2A;CBLB;PRKCA;PIK3R2;CAMK2G |
| 0 | 0.773585591 | 0.106438128 | NDC1;POP7;NXT1;GEMIN2;POP4;ELAC1;UPF3B;AAAS;M |
| 0 | 0.481462326 | 0.064607544 | MIF |
| 0 | 0.531109609 | 0.064411696 | CLOCK;ARNTL |
| 0 | 0.750163065 | 0.086508772 | SPHK2;CAMK2A;CALML4;CASP9;PPP3CA;PPP3CC;CD20S |
| 0 | 0.676412273 | 0.075577189 | TFRC;IL11RA;CD19;ITGB3;CD9;CD36;FCGR1A;CD1B |
| 0 | 0.620707369 | 0.067877843 | AKT3;PIK3R2;PIM2;FCGR1A;MPO |
| 0 | 0.804644028 | 0.079710946 | NDUFA11;NDUFA4L2;PSEN2;PIK3R2;ITPR3;ATP2A1;CALI |
| 0 | 0.793605472 | 0.077234307 | ATP6V1A;TNXB;ITGB5;ITGB3;LAMA3;PARD6G;PIK3R2;L/ |
| 0 | 0.641157205 | 0.060429404 | PRKCG;GNG3;GNG2;PDE1B;GNB4;PRKCA;GNB5 |
| 0 | 0.537093733 | 0.049996825 | PSMA6;PSMB5;PSMD1 |
| 0 | 0.654713105 | 0.060702789 | PRKCG;PRDX1;LAMA3;PRKCA;PIK3R2;LAMC1;CD1B;PLCI |
| 0 | 0.570141009 | 0.052136628 | CASP9;AKT3;PIK3R2;DDB2 |
| 0 | 0.749724177 | 0.068433126 | PRKCG;ITGB3;NFATC3;PRKCA;PIK3R2;ITPR3;CALML4;CA |
| 0 | 0.405373189 | 0.036922577 | LIPA |
| 0 | 0.741174688 | 0.064828467 | VAV3;GLP1R;CAMK2A;ATP2B4;PIK3R2;ATP2A1;ATP2B2; |
| 0 | 0.591958652 | 0.051770569 | GALT;AKT3;PIK3R2;SOCS6;ESR1 |
| 0 | 0.671259843 | 0.05131396 | CREB3L3;AKT3;ITPR3;PIK3R2;CALML4;KRT10;ESR1;CTSD |
| 0 | 0.671259843 | 0.05131396 | VAV3;RPS6KA2;AKT3;NFATC3;PIK3R2;TAB1;TICAM1;BAI |
| 0 | 0.710772326 | 0.053368276 | PRKCG;H2AZ1;H2AZ2;VWF;ITGB3;PRKCA;PIK3R2;HMGB |
| 0 | 0.452877099 | 0.032874796 | GALNT12;GALNT6 |
| 0 | 0.627847469 | 0.04516193 | PRKCG;MMP14;CREB3L3;PRKCA;ITPR3;PLD1;PLCB2;RUN |
| 0 | 0.584256502 | 0.038936109 | PROCR;VWF;PROS1;VSIG4;CLU;C1QC |
| 0 | 0.350035635 | 0.021789393 | MDH1 |
| 0 | 0.591602284 | 0.034831524 | GLE1;PPP2CB;NXT1;PPP2R2C;UPF3B;PPP2R5D;MAGOHI |
| 0 | 0.56976575 | 0.032872024 | ASPH;TPM2;ATP2A1;CACNA1F;MYH7;SLC9A1 |
| 0 | 0.541749625 | 0.029988092 | CDK4;AKT3;E2F1;PIK3R2;DDB2 |
| 0 | 0.578813917 | 0.029692059 | CDK4;AKT3;VEGFB;PRKCA;PIK3R2;PLCB2;PLCD1 |
| 0 | 0.534195026 | 0.027362598 | ARHGAP10;PIK3R2;SEPTIN9;ACTR3B;DNM1 |

| | | | |
|---|---|---|---|
| 0 | 0.644886842 | 0.032353244 | NCEH1;CREB3L3;FZD5;STAR;CCNE1;CDK4;CAMK2A;E2F1 |
| 0 | 0.691211057 | 0.034237487 | VAV3;ITGB5;ITGB3;PDGFB;LPAR2;PIK3R2;BAIAP2;ACTR3 |
| 0 | 0.496349338 | 0.023444284 | RARS2;LARS2;MTFMT;CARS2 |
| 0 | 0.60159623 | 0.027564536 | VWF;ITGB3;AKT3;ITPR3;PIK3R2;RASGRP1;PLCB2;PRKG1 |
| 0 | 0.566564008 | 0.025285284 | PPP2CB;PPP2R2C;AKT3;PIK3R2;TICAM1;PLCB2;C1QC |
| 0 | 0.560630459 | 0.023324667 | CPA3;COL5A1;COL14A1;COL11A2;SLC3A1;KCNN4;SLC36 |
| 0 | 0.684873066 | 0.028330999 | NDUFA11;NDUFA5;COX15;NDUFA4L2;NDUFC1;CPT1B;S |
| 0 | 0.778773386 | 0.03118499 | CALML4;LAMC1;BRCA2;CASP9;EDNRB;RPS6KA5;SUFU;A |
| 0 | 0.480783908 | 0.019180039 | MRAS;CITED2;E2F1;TOMM7 |
| 0 | 0.55481925 | 0.021510571 | PIDD1;EDA;PARP1;TAB1;CCL19;TICAM1;LAT |
| 0 | 0.473360395 | 0.017345096 | HGF;AKT3;PDGFB;PIK3R2 |
| 0 | 0.375407833 | 0.013193942 | CD86;ICA1 |
| 0 | 0.466161836 | 0.015683687 | ATP6V1A;ATP6V1H;ATP6V0E2;ATP6V0A1 |
| 0 | 0.285133566 | 0.009499322 | CALML4 |
| 0 | 0.366448802 | 0.011602094 | DCTN5;CREB3L3 |
| 0 | 0.610442419 | 0.019213918 | MOB1B;LATS1;WWTR1;PPP2CB;FZD5;YWHAQ;PPP2R2C |
| 0 | 0.459178161 | 0.014179533 | FARP2;BAIAP2;ACP1;NECTIN3 |
| 0 | 0.662816266 | 0.019366687 | PRKCG;CBR1;GSTM1;GSTO1;GSTP1;MGST2;EPHX3;PRKC |
| 0 | 0.625029286 | 0.018217311 | H2AZ1;H2AZ2;CALML4;GRIN2C;H2BC18;HDAC6;GNG3;G |
| 0 | 0.53272515 | 0.015519725 | POP7;NXT1;XPO1;POP4;DROSHA;AK6;REXO5 |
| 0 | 0.35790647 | 0.010205928 | TAF9B;GTF2H5 |
| 0 | 0.412107672 | 0.011647609 | APH1B;PSEN2;CIR1 |
| 0 | 0.474597634 | 0.01189032 | CASP9;MSH6;AKT3;PIK3R2;DDB2 |
| 0 | 0.474597634 | 0.01189032 | P2RX3;PDE1B;PKD2L1;ITPR3;PLCB2 |
| 0 | 0.58691271 | 0.012856948 | TUBA1C;PPP2CB;TUBA1B;TUBA1A;PPP2R2C;CDK4;MYH |
| 0 | 0.51231441 | 0.011151583 | CASP9;ALOX5;AKT3;LAMA3;TAB1;LAMC1;MAP2K6 |
| 0 | 0.334507909 | 0.006960037 | CD86;CCR9 |
| 0 | 0.603754177 | 0.012463922 | CD86;SLC45A3;MMP3;IGF1;BMI1;MPO;MMP9;RUNX2;D |
| 0 | 0.570762692 | 0.011632622 | H2AZ1;H2AZ2;AIFM1;PARP1;CAMK2A;HMGB1;PYGL;TIC |
| 0 | 0.427169647 | 0.00854696 | CALML4;TICAM1;CXCL5;C1QC |
| 0 | 0.384546643 | 0.007448668 | FZD5;SUFU;DDB2 |
| 0 | 0.384546643 | 0.007448668 | POLR3GL;POLR3G;POLR3H |
| 0 | 0.502681304 | 0.009438435 | PRKCG;VAV3;PRKCA;PIK3R2;MYL9;MMP9;RAPGEF3 |
| 0 | 0.575809628 | 0.010480732 | CASP9;NLRX1;NXT1;XPO1;CDK4;AKT3;PRKCA;PIK3R2;KP |
| 0 | 0.452160969 | 0.008165977 | NCEH1;SLC27A5;ABCG2;SLC9A1;UGT1A6 |
| 0 | 0.531727407 | 0.008992729 | CASP9;CCNE1;CD209;CDK4;AKT3;CBLB;PIK3R2;EIF2AK4; |
| 0 | 0.320533769 | 0.005399597 | HGF;CD36 |
| 0 | 0.60469546 | 0.009265384 | CRTC1;NFATC3;PIK3R2;ANAPC10;PPP3CA;CDC23;PPP3C |
| 0 | 0.515737527 | 0.006604474 | PCGF6;MEIS1;ACVR1C;FZD5;AKT3;SMARCAD1;PIK3R2;I( |
| 0 | 0.354865536 | 0.004256501 | RETSAT;ALDH1A2;UGT1A6 |
| 0 | 0.522206259 | 0.005705645 | CASP9;PPP2CB;YWHAQ;PPP2R2C;CDK4;AKT3;E2F1;PIK3 |
| 0 | 0.34946897 | 0.003805249 | AKT3;CD36;CPT1B |
| 0 | 0.557220988 | 0.005863307 | VAV3;PIK3R2;PPBP;CXCL5;GNG3;GNG2;AKT3;GNB4;CCR |
| 0 | 0.207952726 | 0.002017556 | CD86 |
| 0 | 0.500674597 | 0.004830862 | FZD5;CDK4;AKT3;E2F1;PIK3R2;IGF1;BRCA2;ESR1;DDB2 |
| 0 | 0.476129517 | 0.003961514 | PPP1R3C;AKT3;INPPL1;CBLB;PIK3R2;PHKB;CALML4;PYG |
| 0 | 0.279627649 | 0.002234554 | CASP9;TLR5 |

| | | | |
|---|---|---|---|
| 0 | 0.404335446 | 0.003146084 | CCR9;PPBP;CCL19;CCR4;CXCL5 |
| 0 | 0.197266148 | 0.00149728 | GRIN2C |
| 0 | 0.685830734 | 0.004645483 | ZNF275;ZNF570;ITGB3;PIK3R2;ZNF3;ZNF25;CASP9;AKT3 |
| 0 | 0.187622164 | 0.001114351 | CD86 |
| 0 | 0.527437245 | 0.002918548 | ENTPD8;PIK3R2;DDB2;CASP9;CCNE1;CDK4;CD19;AKT3;[illegible] |
| 0 | 0.524631302 | 0.002718443 | PIK3R2;H2BC18;HDAC6;DDB1;RBL2;SCIN;RBL1;CREB3L3 |
| 0 | 0.433346246 | 0.002198028 | CCNB3;PLK4;RBL2;AKT3;CAT;PIK3R2;IGF1 |
| 0 | 0.433346246 | 0.002198028 | PRKCG;VAV3;PPP3CA;PPP3CC;PRKCA;PIK3R2;LAT |
| 0 | 0.349185267 | 0.001649032 | PPP3CA;PPP3CC;NFATC3;LAT |
| 0 | 0.496276568 | 0.002329137 | P2RX7;TRPM2;NLRX1;NEK7;TRPV2;ITPR3;TXN;TAB1;TIC[illegible] |
| 0 | 0.307375763 | 0.001384009 | RFXAP;CTSB;LGMN |
| 0 | 0.341386829 | 0.001338842 | MMP3;MMP9;MAPK15;CXCL5 |
| 0 | 0.341386829 | 0.001338842 | PPP2CB;RBL1;ACVR1C;NBL1 |
| 0 | 0.170905923 | 6.22E-04 | PIK3R2 |
| 0 | 0.532886034 | 0.001603425 | UBE2D4;GLMN;PIK3R2;ITPR3;ATG14;SEPTIN9;ACTR3B;[illegible] |
| 0 | 0.417399988 | 8.65E-04 | NDUFA11;NDUFA5;AKT3;NDUFA4L2;PIK3R2;NDUFC1;P[illegible] |
| 0 | 0.147840726 | 2.28E-04 | CD86 |
| 0 | 0.38073028 | 4.80E-04 | CD86;NTNG2;CNTNAP1;CDH3;CADM1;ITGB8;NECTIN3 |
| 0 | 0.356785399 | 4.30E-04 | CD86;H2AZ1;H2AZ2;FCGR1A;H2BC18;C1QC |
| 0 | 0.378027554 | 4.38E-04 | FZD5;CCNE1;HGF;AKT3;E2F1;PIK3R2;DDB2 |
| 0 | 0.298078999 | 3.41E-04 | PPP3CA;PPP3CC;NFATC3;LAT |
| 0 | 0.527402965 | 5.09E-04 | PRKCG;DNMT1;SLC45A3;TNXB;ITGB3;PDGFB;PRKCA;PIK |
| 0 | 0.204827887 | 1.85E-04 | TAB1;FCGR1A |
| 0 | 0.250336416 | 2.02E-04 | KRT10;FCGR1A;C1QC |
| 0 | 0.355313919 | 1.92E-04 | MRPL30;MRPL18;MRPL19;MRPS21;RPS27L;MRPL10;M[illegible] |
| 0 | 0.120038926 | 4.30E-05 | TLR5 |
| 0 | 0.319347447 | 1.02E-04 | PRPF40B;PPIH;LSM5;MAGOHB;LSM4;LSM3 |
| 0 | 0.111308893 | 2.18E-05 | NLRX1 |
| 0 | 0.294580801 | 3.18E-05 | CNTFR;IL11RA;AKT3;PDGFB;PIK3R2;SOCS6 |
| 0 | 0.424986789 | 1.15E-05 | GLP1R;CHRNB1;CALCB;CALCRL;THRB;THRA;TACR2;LPAR |
| 0 | 0.380201489 | 7.47E-06 | TNFSF18;CNTFR;EDA;IL11RA;TNFSF12;GDF1;PPBP;CXCL[illegible] |
| 0 | 0.086598062 | 4.72E-07 | PDE1B;CAMK2A;CALML4;CAMK2G;PRKG1 |
| 0 | 0.237449782 | 1.20E-06 | PRKCG;VWF;MMP3;RPS27L;PRKCA;PIK3R2;C1QC |

;HADH;ACADS;ACAD8;MCCC2;BCKDHB;MCCC1;ACADSB;ALDH3A2;HADHB;HADHA;ALDH6A1;PCCA;EHHADH;IVD;

P100;FANCI;CENPX;FANCM;POLN;RMI1;FANCL;RPA1;FANCC;FANCE;PALB2;RAD51;RPA3;UBE2T;SLX1A;FAN1;TEL

2;LAPTM4A;CTSO;AP1G2;GM2A;CTSK;SLC17A5;AP1S1;ATP6V1H;ACP5;ACP2;GUSB;CTSD;ATP6V0A1;CTSB;ARSA;

T19;HACL1;MLYCD;ACAA1;DECR2;ACOT8;PHYH;IDH1;ECH1;PEX1;SOD1;AMACR;EHHADH;PEX6;CAT;PXMP2;PEX1

;ARNTL;PPP3CA;PPP2CB;GNG3;PPP3CC;GNG2;CREB3L3;PPP2R2C;AKT3;GNB4;GNB5;CLOCK;PLCB2;CAMK2G

JBB4A;TUBA1C;TUBB6;TUBA1B;TUBA1A;TUBB3;CD209;MRC1;ATP6V1H;CD36;PLA2R1;ATP6V0E2;FCGR1A;RILP;A

LF;PPP3CA;EDNRB;PPP3CC;PDGFD;CAMK2G;PRKCG;MCOLN3;SPHK2;HGF;MST1;TACR2;VEGFB;ATP2B4;PRKCA;PI

;PPP3CA;RAD50;PPP3CC;MRAS;RBL1;CCNE1;CDK4;CHEK2;AKT3;CHEK1;E2F1;RAD1;SLC25A4;MAP2K6

B2;CALML4;CACNA1F;SLC9A1;PPP2CB;RPS6KA5;CREB3L3;PPP2R2C;AKT3;PLCB2;CAMK2G;RAPGEF3;MYH7

EFNA4;RASGRP3;PDGFD;AKT3;MAP2K6;PRKCG;VAV3;FARP2;HGF;VEGFB;PRKCA;IGF1;MRAS;LPAR5;PRKD3;TEK;P

KT3;PDK4;CD36;CTSD;CAMK2G;PDK2;PRKCG;PARP1;NDUFA5;GSR;PRKCA;NDUFC1;CPT1B;SDHB;MMP9;TBC1D4;

A1;GPC1;AKT3;DROSHA;FLNB;CAMK2G;PRKCG;VAV3;FZD5;HGF;PRKCA;IGF1;NUDT16L1;ESR1;MMP9;MRAS;TFAI

FB;VEGFB;PRKCA;PIK3R2;IGF1;PARVB;LAMC1;PARVG;PDGFD;ITGA10;AKT3;CHAD;SPP1;ITGB8;FLNB;MYL9

4;CACNA1F;PPP3CA;EDNRB;PPP3CC;CREB3L3;ADORA3;AKT3;KCNMB4;MYL9;PLCB2;SLC25A4;PRKG1;MYH7

6;GLE1;CASP9;PPP3CA;TUBA1C;TUBA1B;TUBB6;ACTR1B;PPP3CC;TUBA1A;PSMB5;TUBB3;PSMD1;ANXA7;NUP43;

RASGRP3;GNG3;GNG2;PDGFD;AKT3;PRKCG;RALBP1;PLA2G12A;HGF;VEGFB;PRKCA;IGF1;MRAS;GNB4;GNB5;TEK;

DB1;CCNB3;CASP9;PPP3CA;GNG3;PPP3CC;GNG2;AP1G2;AKT3;CHEK1;GNB4;AP1S1;GNB5;TAB1;MAP2K6;CUL4B

BA1C;TUBA1B;TUBB6;ACTR1B;TUBA1A;PSMB5;CREB3L3;TUBB3;PSMD1;IFT57;DNAH12;NDUFA5;TUBB;GPX7;ND

LAMC1;EFNA4;CASP9;PPP2CB;GNG3;GNG2;CREB3L3;YWHAQ;CD19;PDGFD;AKT3;CHAD;SPP1;ITGB8;VWF;HGF;V

ASP9;PPP3CA;TUBA1C;TUBA1B;TUBB6;PPP3CC;TUBA1A;PSMB5;CREB3L3;TUBB3;PSMD1;NDUFA5;TUBB;NDUFC1

K7;CASP9;TUBA1C;TUBA1B;UCHL1;TUBB6;TUBA1A;PSMB5;TUBB3;PSMD1;CAMK2G;NDUFA5;TUBB;NDUFC1;TUE
.B;TUBB6;ACTR1B;TUBA1A;TUBB3;AKT3;FLNB;RILP;MAP2K6;TUBB;DYNLT3;TUBB4A;EXOC4;PLEKHM1;DYNLRB2;T

CA;TUBA1B;TUBB6;UCHL1;TUBA1A;ACTR1B;PPP3CC;TUBB3;PSMD1;IFT57;PRKCG;TUBB;GPX7;PRKCA;NDUFC1;A

OA4;CALML4;TICAM1;MMP9;CASP9;PPP3CA;PPP3CC;AKT3;PPARG;CD36;TAB1;PLCB2;CAMK2G;MAP2K6

S6KA5;PPP3CC;RPS6KA2;PDGFD;AKT3;FLNB;MAP4K3;MAP2K5;MAP2K6;PRKCG;MAP4K2;HGF;NFATC3;VEGFB;PF

D6G;CBLB;PLD1;ACTR3B;DNM1;DAB2;RUFY2;MVB12A;VPS45;LDLRAP1;FOLR2;FOLR1;SNX5;ARF5;SPG21;RAB11F

ML4;CACNA1F;RTN4;CASP9;PPP3CA;TUBA1C;TUBA1B;TUBB6;APH1B;PPP3CC;TUBA1A;PSMB5;TUBB3;AKT3;PSM
AMC1;PPP2CB;CREB3L3;AKT3;CHAD;SPP1;E2F1;ATP6V1H;ITGB8;ATP6V0E2;ATP6V0A1;FZD5;VWF;PPP2R5D;TUB

DHB;CREB3L3;NDUFAF7;RPS6KA2;NDUFAF4;NDUFAF2;ATP5PO;DPF3;PPARG;NDUFAF1;SLC25A20;PRKG1;PNPLA
KT3;PIM2;PRKCG;RALBP1;GSTO1;HGF;PRKCA;MMP9;DDB2;MSH6;CCNE1;CKS2;PPARG;PLCB2;GSTP1;CAMK2A;L

[illegible];ZNF527;ZNF404;ZNF624;ZNF205;ZNF568;ZNF689;ZNF667;ZNF248;ZNF786;EIF2B1;ZNF684;ZNF420;ZNF19;ZNF3

ABCA2;FUCA1;FUCA2;AP1B1;CTNS;MCOLN1;PLA2G15;GALNS;NPC2;GLB1;PPT1;TPP1;DNASE2;CD68;GLA;LGMN

;MAP2K6;UBQLN2;NDC1;DNAH12;NDUFA5;TUBB;GPX7;NDUFC1;ATG14;GRIN2C;TUBB4A;SDHB;SOD1;FIG4;PSM/

TG14;SDHB;PSMA6;ALS2;CAT;PLCB2;SLC25A4;DCTN5;CAMK2A;NDUFA4L2;PSEN2;ITPR3;DNAH9;CACNA1F;PSMB

D1;FZD5;NDUFA5;TUBB;NDUFC1;ATG14;GRIN2C;TUBB4A;SDHB;BACE1;PSMA6;CDK5;ATP5PO;PLCB2;SLC25A4

AMA3;PDGFB;LPAR2;MGST2;PIK3R2;PLD1;RASGRP1;RASGRP3;GNG3;GNG2;E2F1;CAMK2G;GSTM1;FZD5;TXNRD3

83;ZNF283;ZNF140;ZNF382;ZFP1;ZNF181;ZNF180;ZNF10;EIF2AK4;TICAM1;ZFP69;ZNF713;TAB1;ZNF599;ZNF555;

;5;CAMK2G;MAP2K6;DNAH12;FZD5;NDUFA5;GRIN2C;TUBB4A;SOD1;FIG4;CDK5;DLG4;ATP5PO;DNAL1

**Supplementary File S6: Comprehensive details of the results of functional enrichment analysis**

| BP | Term | Overlap | P-value | Adjusted P-value |
|---|---|---|---|---|
| | response to unfolded protein (GO:0006986) | 13/49 | 9.86E-25 | 5.19E-22 |
| | regulation of cellular response to heat (GO:1900034) | 29160 | 1.22E-17 | 3.21E-15 |
| | regulation of cellular response to stress (GO:008013 | 11/118 | 1.22E-15 | 2.14E-13 |
| | chaperone-mediated protein complex assembly (GO | 44764 | 2.51E-14 | 3.30E-12 |
| | 'de novo' posttranslational protein folding (GO:0051 | 44773 | 3.82E-13 | 4.02E-11 |
| | chaperone cofactor-dependent protein refolding (GO | 44738 | 1.77E-11 | 1.55E-09 |
| | protein stabilization (GO:0050821) | 9/179 | 1.63E-10 | 1.22E-08 |
| | cellular protein-containing complex assembly (GO:0 | 8/168 | 2.87E-09 | 1.85E-07 |
| | cellular response to unfolded protein (GO:0034620) | 44708 | 3.17E-09 | 1.85E-07 |
| | positive regulation of DNA biosynthetic process (GO: | 22433 | 4.03E-09 | 2.12E-07 |
| | cellular response to heat (GO:0034605) | 13271 | 1.46E-08 | 6.98E-07 |
| | cellular response to topologically incorrect protein ( | 11780 | 7.00E-07 | 3.07E-05 |
| | positive regulation of telomerase activity (GO:00519 | 12510 | 9.00E-07 | 3.64E-05 |
| | positive regulation of establishment of protein locali | 44630 | 1.18E-06 | 3.88E-05 |
| | negative regulation of inclusion body assembly (GO:( | 44630 | 1.18E-06 | 3.88E-05 |
| | negative regulation of transcription from RNA polym | 44630 | 1.18E-06 | 3.88E-05 |
| | regulation of establishment of protein localization to | 44631 | 1.62E-06 | 4.26E-05 |
| | regulation of inclusion body assembly (GO:0090083) | 44631 | 1.62E-06 | 4.26E-05 |
| | positive regulation of protein localization to Cajal bo | 44631 | 1.62E-06 | 4.26E-05 |
| | regulation of protein localization to Cajal body (GO:1 | 44631 | 1.62E-06 | 4.26E-05 |
| | positive regulation of protein localization to chromo: | 44632 | 2.16E-06 | 5.40E-05 |
| | regulation of telomerase activity (GO:0051972) | 16893 | 3.11E-06 | 7.43E-05 |
| | positive regulation of telomerase RNA localization to | 44635 | 4.44E-06 | 1.01E-04 |
| | regulation of telomerase RNA localization to Cajal bo | 44638 | 7.92E-06 | 1.74E-04 |
| | positive regulation of establishment of protein locali | 44639 | 9.39E-06 | 1.98E-04 |
| | regulation of protein modification by small protein c | 44640 | 1.10E-05 | 2.23E-04 |
| | positive regulation of protein localization to nucleus | 24929 | 1.50E-05 | 2.92E-04 |
| | negative regulation of cellular component organizati | 29312 | 2.85E-05 | 5.36E-04 |
| | telomere maintenance via telomerase (GO:0007004) | 44649 | 3.49E-05 | 6.33E-04 |
| | RNA-dependent DNA biosynthetic process (GO:0006 | 44650 | 3.87E-05 | 6.74E-04 |
| | regulation of transcription from RNA polymerase II p | 31868 | 3.97E-05 | 6.74E-04 |
| | positive regulation of telomere maintenance via telo | 12114 | 5.18E-05 | 8.51E-04 |
| | telomere maintenance via telomere lengthening (GC | 13210 | 6.74E-05 | 0.001043033 |
| | positive regulation of telomere maintenance via telo | 13210 | 6.74E-05 | 0.001043033 |
| | positive regulation of tau-protein kinase activity (GO | 44598 | 7.06E-05 | 0.001060326 |
| | regulation of protein ubiquitination (GO:0031396) | 4/109 | 9.58E-05 | 0.001399663 |
| | regulation of telomere maintenance via telomerase | 19054 | 2.04E-04 | 0.002894727 |
| | regulation of tau-protein kinase activity (GO:190294 | 44602 | 2.10E-04 | 0.002913512 |
| | protein targeting to mitochondrion (GO:0006626) | 20149 | 2.41E-04 | 0.003245155 |
| | positive regulation of transferase activity (GO:00513 | 4/148 | 3.11E-04 | 0.004083144 |
| | central nervous system neuron axonogenesis (GO:00 | 44605 | 3.63E-04 | 0.004660981 |
| | central nervous system neuron development (GO:00 | 44607 | 4.88E-04 | 0.006107907 |
| | neutrophil degranulation (GO:0043312) | 6/481 | 6.10E-04 | 0.007460259 |
| | neutrophil activation involved in immune response ( | 6/485 | 6.37E-04 | 0.007522086 |

| | | | |
|---|---|---|---|
| positive regulation of type I interferon production (G | 28185 | 6.49E-04 | 0.007522086 |
| neutrophil mediated immunity (GO:0002446) | 6/488 | 6.58E-04 | 0.007522086 |
| negative regulation of extrinsic apoptotic signaling p | 29281 | 7.25E-04 | 0.008116063 |
| positive regulation of interferon-alpha production (G | 44612 | 8.76E-04 | 0.009603518 |
| axon extension (GO:0048675) | 44616 | 0.001265945 | 0.013317744 |
| protein insertion into mitochondrial membrane (GO: | 44616 | 0.001265945 | 0.013317744 |
| regulation of peptidyl-serine phosphorylation (GO:0( | 35855 | 0.00130603 | 0.013470036 |
| regulation of interferon-alpha production (GO:0032( | 44617 | 0.001374107 | 0.013899624 |
| negative regulation of programmed cell death (GO:0 | 5/381 | 0.001441319 | 0.014304414 |
| protein peptidyl-prolyl isomerization (GO:0000413) | 44619 | 0.001603223 | 0.01533264 |
| positive regulation of nitric oxide biosynthetic proce: | 44619 | 0.001603223 | 0.01533264 |
| regulation of protein transport (GO:0051223) | 44620 | 0.001724139 | 0.015910479 |
| positive regulation of nitric oxide metabolic process | 44620 | 0.001724139 | 0.015910479 |
| peptidyl-proline modification (GO:0018208) | 11720 | 0.002249771 | 0.020403091 |
| neuron projection extension (GO:1990138) | 12086 | 0.002391579 | 0.021321531 |
| negative regulation of extrinsic apoptotic signaling p | 12451 | 0.002537511 | 0.022245509 |
| positive regulation of interferon-beta production (G( | 13181 | 0.002841674 | 0.024503619 |
| ribonucleoprotein complex assembly (GO:0022618) | 3/136 | 0.003322434 | 0.027789512 |
| regulation of cellular component biogenesis (GO:004 | 14277 | 0.003328402 | 0.027789512 |
| regulation of nitric oxide biosynthetic process (GO:0( | 14642 | 0.003498701 | 0.028754952 |
| positive regulation of ATPase activity (GO:0032781) | 15373 | 0.003851281 | 0.030235427 |
| regulation of chemokine production (GO:0032642) | 15373 | 0.003851281 | 0.030235427 |
| regulation of extrinsic apoptotic signaling pathway v | 15373 | 0.003851281 | 0.030235427 |
| regulation of mRNA stability (GO:0043488) | 3/146 | 0.004054528 | 0.031362967 |
| positive regulation of intracellular protein transport | 3/148 | 0.004211774 | 0.032107144 |
| regulation of cellular protein localization (GO:19038: | 16834 | 0.004603868 | 0.034594778 |
| negative regulation of cell projection organization (G | 17930 | 0.00520926 | 0.037535217 |
| regulation of cellular localization (GO:0060341) | 17930 | 0.00520926 | 0.037535217 |
| regulation of interferon-beta production (GO:00326 | 17930 | 0.00520926 | 0.037535217 |
| regulation of apoptotic process (GO:0042981) | 6/742 | 0.005423111 | 0.038548062 |
| negative regulation of protein ubiquitination (GO:00 | 18660 | 0.005632097 | 0.039499775 |
| positive regulation of chemokine production (GO:00: | 19391 | 0.006070169 | 0.042011956 |
| positive regulation of cytokine production (GO:0001: | 4/335 | 0.006194253 | 0.04231399 |
| negative regulation of protein modification by small | 20121 | 0.006523338 | 0.043990717 |
| positive regulation of interferon-gamma production | 20852 | 0.006991471 | 0.046550809 |
| negative regulation of neuron projection developme | 21217 | 0.007231107 | 0.047544529 |
| positive regulation of interleukin-8 production (GO:0 | 22313 | 0.007972092 | 0.051769387 |
| negative regulation of intrinsic apoptotic signaling pa | 23408 | 0.008745845 | 0.055425474 |
| protein catabolic process (GO:0030163) | 23408 | 0.008745845 | 0.055425474 |
| ERBB signaling pathway (GO:0038127) | 24139 | 0.009279665 | 0.057424752 |
| regulation of protein localization (GO:0032880) | 24139 | 0.009279665 | 0.057424752 |
| vascular endothelial growth factor receptor signaling | 24504 | 0.009551922 | 0.058422222 |
| cellular response to decreased oxygen levels (GO:00: | 25235 | 0.010107048 | 0.058858465 |
| positive regulation of protein polymerization (GO:00 | 25235 | 0.010107048 | 0.058858465 |
| Fc-gamma receptor signaling pathway involved in ph | 25965 | 0.010676216 | 0.058858465 |
| positive regulation of cellular protein localization (G( | 25965 | 0.010676216 | 0.058858465 |
| aggresome assembly (GO:0070842) | 44566 | 0.010952688 | 0.058858465 |

| | | | |
|---|---|---|---|
| fructose 2,6-bisphosphate metabolic process (GO:00 | 44566 | 0.010952688 | 0.058858465 |
| RNA localization to Cajal body (GO:0090670) | 44566 | 0.010952688 | 0.058858465 |
| positive regulation of MHC class I biosynthetic proce | 44566 | 0.010952688 | 0.058858465 |
| scaRNA localization to Cajal body (GO:0090666) | 44566 | 0.010952688 | 0.058858465 |
| positive regulation of nucleotide-binding oligomeriza | 44566 | 0.010952688 | 0.058858465 |
| regulation of protein import (GO:1904589) | 44566 | 0.010952688 | 0.058858465 |
| Fc-gamma receptor signaling pathway (GO:0038094) | 26330 | 0.010966026 | 0.058858465 |
| Fc receptor mediated stimulatory signaling pathway | 27061 | 0.011556016 | 0.059533789 |
| positive regulation of T cell activation (GO:0050870) | 27426 | 0.011856166 | 0.059533789 |
| regulation of RNA splicing (GO:0043484) | 27791 | 0.01215973 | 0.059533789 |
| positive regulation of interleukin-6 production (GO:0 | 27791 | 0.01215973 | 0.059533789 |
| negative regulation of establishment of protein local | 44567 | 0.013129125 | 0.059533789 |
| axo-dendritic transport (GO:0008088) | 44567 | 0.013129125 | 0.059533789 |
| heat acclimation (GO:0010286) | 44567 | 0.013129125 | 0.059533789 |
| positive regulation of immune response to tumor ce | 44567 | 0.013129125 | 0.059533789 |
| inclusion body assembly (GO:0070841) | 44567 | 0.013129125 | 0.059533789 |
| cellular heat acclimation (GO:0070370) | 44567 | 0.013129125 | 0.059533789 |
| signal complex assembly (GO:0007172) | 44567 | 0.013129125 | 0.059533789 |
| regulation of glutamate receptor signaling pathway ( | 44567 | 0.013129125 | 0.059533789 |
| centromeric sister chromatid cohesion (GO:0070601 | 44567 | 0.013129125 | 0.059533789 |
| medium-chain fatty acid biosynthetic process (GO:0( | 44567 | 0.013129125 | 0.059533789 |
| positive regulation of T cell mediated immune respo | 44567 | 0.013129125 | 0.059533789 |
| regulation of microtubule motor activity (GO:200057 | 44567 | 0.013129125 | 0.059533789 |
| regulation of NK T cell activation (GO:0051133) | 44567 | 0.013129125 | 0.059533789 |
| regulation of nucleotide-binding oligomerization dor | 44567 | 0.013129125 | 0.059533789 |
| activation of cysteine-type endopeptidase activity in | 29618 | 0.013728223 | 0.061195301 |
| regulation of interleukin-8 production (GO:0032677) | 29618 | 0.013728223 | 0.061195301 |
| negative regulation of axon regeneration (GO:00486 | 44568 | 0.015300881 | 0.064204334 |
| regulation of T cell mediated immune response to tu | 44568 | 0.015300881 | 0.064204334 |
| regulation of transforming growth factor beta activa | 44568 | 0.015300881 | 0.064204334 |
| negative regulation of mitochondrial outer membrar | 44568 | 0.015300881 | 0.064204334 |
| positive regulation of microtubule nucleation (GO:0( | 44568 | 0.015300881 | 0.064204334 |
| positive regulation of NK T cell activation (GO:00511 | 44568 | 0.015300881 | 0.064204334 |
| regulation of MHC class I biosynthetic process (GO:0 | 44568 | 0.015300881 | 0.064204334 |
| regulation of interferon-gamma production (GO:003 | 31444 | 0.015379745 | 0.064204334 |
| protein import (GO:0017038) | 32540 | 0.016409712 | 0.067068711 |
| toll-like receptor signaling pathway (GO:0002224) | 33270 | 0.017112375 | 0.067068711 |
| regulation of transcription from RNA polymerase II p | 44569 | 0.017467967 | 0.067068711 |
| negative regulation of glucose import (GO:0046325) | 44569 | 0.017467967 | 0.067068711 |
| negative regulation of muscle cell apoptotic process | 44569 | 0.017467967 | 0.067068711 |
| negative regulation of neuron projection regeneratic | 44569 | 0.017467967 | 0.067068711 |
| negative regulation of nitrogen compound metabolic | 44569 | 0.017467967 | 0.067068711 |
| regulation of establishment of protein localization tc | 44569 | 0.017467967 | 0.067068711 |
| negative regulation of striated muscle cell apoptotic | 44569 | 0.017467967 | 0.067068711 |
| peptidyl-proline hydroxylation to 4-hydroxy-L-prolin | 44569 | 0.017467967 | 0.067068711 |
| T cell activation (GO:0042110) | 33635 | 0.017468467 | 0.067068711 |
| positive regulation of peptidyl-serine phosphorylatio | 34001 | 0.017827712 | 0.067952004 |

| | | | |
|---|---|---|---|
| negative regulation of cell death (GO:0060548) | 34366 | 0.018190096 | 0.068834465 |
| protein maturation (GO:0051604) | 35462 | 0.019295932 | 0.072206901 |
| positive regulation of blood vessel endothelial cell pı | 44570 | 0.019630393 | 0.072206901 |
| fructose 1,6-bisphosphate metabolic process (GO:00 | 44570 | 0.019630393 | 0.072206901 |
| positive regulation of tumor necrosis factor-mediate | 44570 | 0.019630393 | 0.072206901 |
| regulation of transforming growth factor beta recept | 2/100 | 0.020429484 | 0.073939204 |
| positive regulation of protein localization to membra | 2/101 | 0.020813425 | 0.073939204 |
| regulation of proteasomal ubiquitin-dependent prot | 2/102 | 0.021200387 | 0.073939204 |
| negative regulation of apoptotic process (GO:00430 | 4/485 | 0.021589286 | 0.073939204 |
| negative regulation of establishment of protein local | 44571 | 0.021788169 | 0.073939204 |
| negative regulation of glycolytic process (GO:004582 | 44571 | 0.021788169 | 0.073939204 |
| negative regulation of leukocyte migration (GO:0002 | 44571 | 0.021788169 | 0.073939204 |
| I-kappaB phosphorylation (GO:0007252) | 44571 | 0.021788169 | 0.073939204 |
| negative regulation of purine nucleotide metabolic p | 44571 | 0.021788169 | 0.073939204 |
| regulation of glycoprotein biosynthetic process (GO: | 44571 | 0.021788169 | 0.073939204 |
| negative regulation of transforming growth factor be | 44571 | 0.021788169 | 0.073939204 |
| regulation of microtubule nucleation (GO:0010968) | 44571 | 0.021788169 | 0.073939204 |
| purine nucleoside bisphosphate biosynthetic process | 44572 | 0.023941303 | 0.076787349 |
| regulation of amyloid precursor protein biosynthetic | 44572 | 0.023941303 | 0.076787349 |
| ribonucleoside bisphosphate biosynthetic process (G | 44572 | 0.023941303 | 0.076787349 |
| isotype switching (GO:0045190) | 44572 | 0.023941303 | 0.076787349 |
| cellular response to UV-A (GO:0071492) | 44572 | 0.023941303 | 0.076787349 |
| coenzyme A biosynthetic process (GO:0015937) | 44572 | 0.023941303 | 0.076787349 |
| tetrahydrobiopterin metabolic process (GO:0046146 | 44572 | 0.023941303 | 0.076787349 |
| cyclooxygenase pathway (GO:0019371) | 44572 | 0.023941303 | 0.076787349 |
| peptidyl-proline hydroxylation (GO:0019511) | 44572 | 0.023941303 | 0.076787349 |
| regulation of interleukin-6 production (GO:0032675) | 2/110 | 0.024403106 | 0.077794143 |
| positive regulation of programmed cell death (GO:00 | 3/286 | 0.024899212 | 0.078897504 |
| regulation of axon regeneration (GO:0048679) | 44573 | 0.026089806 | 0.080724928 |
| negative regulation of glucose transmembrane trans | 44573 | 0.026089806 | 0.080724928 |
| negative regulation of JUN kinase activity (GO:00435 | 44573 | 0.026089806 | 0.080724928 |
| negative regulation of receptor internalization (GO:0 | 44573 | 0.026089806 | 0.080724928 |
| regulation of protein-containing complex assembly ( | 2/116 | 0.026927016 | 0.08282813 |
| positive regulation of cysteine-type endopeptidase a | 2/119 | 0.02822707 | 0.083903499 |
| pteridine-containing compound metabolic process ( | 44574 | 0.028233687 | 0.083903499 |
| positive regulation of protein localization to cell surf | 44574 | 0.028233687 | 0.083903499 |
| toll-like receptor 9 signaling pathway (GO:0034162) | 44574 | 0.028233687 | 0.083903499 |
| transport along microtubule (GO:0010970) | 44574 | 0.028233687 | 0.083903499 |
| protein localization to mitochondrion (GO:0070585) | 44574 | 0.028233687 | 0.083903499 |
| regulation of mRNA catabolic process (GO:0061013) | 2/122 | 0.029552027 | 0.087327899 |
| regulation of response to interferon-gamma (GO:00 | 44575 | 0.030372956 | 0.087781181 |
| response to UV-A (GO:0070141) | 44575 | 0.030372956 | 0.087781181 |
| coenzyme A metabolic process (GO:0015936) | 44575 | 0.030372956 | 0.087781181 |
| N-terminal protein amino acid modification (GO:003 | 44575 | 0.030372956 | 0.087781181 |
| positive regulation of apoptotic process (GO:004306 | 3/310 | 0.030607019 | 0.087974273 |
| cellular response to oxidative stress (GO:0034599) | 2/125 | 0.030901518 | 0.088338034 |
| negative regulation of cell growth (GO:0030308) | 2/126 | 0.031356737 | 0.089154831 |

| | | | |
|---|---|---|---|
| positive regulation of intracellular signal transductio | 4/546 | 0.031547321 | 0.089214466 |
| negative regulation of endoplasmic reticulum stress- | 44576 | 0.032507623 | 0.089523611 |
| regulation of establishment of protein localization (G | 44576 | 0.032507623 | 0.089523611 |
| positive regulation of protein export from nucleus (G | 44576 | 0.032507623 | 0.089523611 |
| medium-chain fatty acid metabolic process (GO:005: | 44576 | 0.032507623 | 0.089523611 |
| prostanoid biosynthetic process (GO:0046457) | 44576 | 0.032507623 | 0.089523611 |
| cellular response to hypoxia (GO:0071456) | 2/131 | 0.033672647 | 0.092017317 |
| regulation of blood vessel endothelial cell proliferati | 44577 | 0.034637697 | 0.092017317 |
| heme catabolic process (GO:0042167) | 44577 | 0.034637697 | 0.092017317 |
| mitotic sister chromatid cohesion (GO:0007064) | 44577 | 0.034637697 | 0.092017317 |
| regulation of phosphate metabolic process (GO:0019 | 44577 | 0.034637697 | 0.092017317 |
| N-terminal protein amino acid acetylation (GO:0006 | 44577 | 0.034637697 | 0.092017317 |
| porphyrin-containing compound catabolic process (G | 44577 | 0.034637697 | 0.092017317 |
| negative regulation of ATP metabolic process (GO:19 | 44578 | 0.036763188 | 0.096206155 |
| positive regulation of macrophage activation (GO:00 | 44578 | 0.036763188 | 0.096206155 |
| regulation of leukocyte migration (GO:0002685) | 44578 | 0.036763188 | 0.096206155 |
| regulation of cellular component organization (GO:0 | 2/139 | 0.037512918 | 0.097682153 |
| positive regulation of cell migration involved in sprou | 44579 | 0.038884106 | 0.098611473 |
| regulation of cellular protein catabolic process (GO:1 | 44579 | 0.038884106 | 0.098611473 |
| positive regulation of phosphoprotein phosphatase a | 44579 | 0.038884106 | 0.098611473 |
| regulation of mitochondrial outer membrane perme | 44579 | 0.038884106 | 0.098611473 |
| endothelial cell proliferation (GO:0001935) | 44579 | 0.038884106 | 0.098611473 |
| protein localization to organelle (GO:0033365) | 2/142 | 0.038994651 | 0.098611473 |
| receptor-mediated endocytosis (GO:0006898) | 2/143 | 0.039493505 | 0.099383603 |
| establishment or maintenance of apical/basal cell pc | 44580 | 0.04100046 | 0.099383603 |
| positive regulation of cellular protein catabolic proce | 44580 | 0.04100046 | 0.099383603 |
| negative regulation of immune system process (GO:( | 44580 | 0.04100046 | 0.099383603 |
| positive regulation of G protein-coupled receptor sig | 44580 | 0.04100046 | 0.099383603 |
| positive regulation of leukocyte activation (GO:0002 | 44580 | 0.04100046 | 0.099383603 |
| regulation of mitotic spindle assembly (GO:1901673) | 44580 | 0.04100046 | 0.099383603 |
| diol metabolic process (GO:0034311) | 44580 | 0.04100046 | 0.099383603 |
| prostaglandin biosynthetic process (GO:0001516) | 44580 | 0.04100046 | 0.099383603 |
| positive regulation of hydrolase activity (GO:005134 | 2/149 | 0.04253765 | 0.102148867 |
| positive regulation of alpha-beta T cell activation (G( | 44581 | 0.043112259 | 0.102148867 |
| protein targeting to lysosome (GO:0006622) | 44581 | 0.043112259 | 0.102148867 |
| positive regulation of cyclin-dependent protein kinas | 44581 | 0.043112259 | 0.102148867 |
| regulation of programmed necrotic cell death (GO:0( | 44581 | 0.043112259 | 0.102148867 |
| positive regulation of protein kinase activity (GO:004 | 2/154 | 0.045139873 | 0.104781782 |
| purinergic nucleotide receptor signaling pathway (G( | 44582 | 0.045219514 | 0.104781782 |
| negative regulation of peptidyl-serine phosphorylati | 44582 | 0.045219514 | 0.104781782 |
| negative regulation of response to wounding (GO:19 | 44582 | 0.045219514 | 0.104781782 |
| positive regulation of phosphatase activity (GO:0010 | 44582 | 0.045219514 | 0.104781782 |
| regulation of endoplasmic reticulum stress-induced i | 44583 | 0.047322234 | 0.108696485 |
| lipid droplet organization (GO:0034389) | 44583 | 0.047322234 | 0.108696485 |
| positive regulation of protein kinase B signaling (GO: | 2/161 | 0.048879819 | 0.110148919 |
| regulation of supramolecular fiber organization (GO: | 44584 | 0.049420428 | 0.110148919 |
| negative regulation of extrinsic apoptotic signaling p | 44584 | 0.049420428 | 0.110148919 |

| | | | |
|---|---|---|---|
| negative regulation of signal transduction in absence | 44584 | 0.049420428 | 0.110148919 |
| regulation of interferon-gamma-mediated signaling | 44584 | 0.049420428 | 0.110148919 |
| regulation of necroptotic process (GO:0060544) | 44584 | 0.049420428 | 0.110148919 |
| regulation of protein export from nucleus (GO:0046 | 44584 | 0.049420428 | 0.110148919 |
| negative regulation of cell population proliferation ( | 3/379 | 0.050517174 | 0.111507899 |
| regulation of neuron projection development (GO:0 | 2/165 | 0.051066235 | 0.111507899 |
| positive regulation of cellular response to transformi | 44585 | 0.051514105 | 0.111507899 |
| long-chain fatty-acyl-CoA metabolic process (GO:003 | 44585 | 0.051514105 | 0.111507899 |
| positive regulation of protein targeting to membrane | 44585 | 0.051514105 | 0.111507899 |
| positive regulation of transforming growth factor be | 44585 | 0.051514105 | 0.111507899 |
| regulation of monooxygenase activity (GO:0032768) | 44585 | 0.051514105 | 0.111507899 |
| negative regulation of cellular carbohydrate metabo | 44586 | 0.053603276 | 0.114615136 |
| regulation of spindle assembly (GO:0090169) | 44586 | 0.053603276 | 0.114615136 |
| negative regulation of response to endoplasmic retic | 44586 | 0.053603276 | 0.114615136 |
| negative regulation of proteolysis involved in cellula | 44587 | 0.05568795 | 0.118590534 |
| regulation of vasculature development (GO:1901342 | 44588 | 0.057768136 | 0.121544159 |
| positive regulation of erythrocyte differentiation (G( | 44588 | 0.057768136 | 0.121544159 |
| regulation of epidermal growth factor-activated rece | 44588 | 0.057768136 | 0.121544159 |
| positive regulation of cellular biosynthetic process ( | 2/180 | 0.059570098 | 0.122007217 |
| negative regulation of axonogenesis (GO:0050771) | 44589 | 0.059843844 | 0.122007217 |
| positive regulation of defense response to virus by h | 44589 | 0.059843844 | 0.122007217 |
| positive regulation of microtubule polymerization (G | 44589 | 0.059843844 | 0.122007217 |
| internal peptidyl-lysine acetylation (GO:0018393) | 44589 | 0.059843844 | 0.122007217 |
| regulation of extrinsic apoptotic signaling pathway ir | 44589 | 0.059843844 | 0.122007217 |
| positive regulation of protein import into nucleus (G | 44589 | 0.059843844 | 0.122007217 |
| regulation of microtubule polymerization or depolyn | 44589 | 0.059843844 | 0.122007217 |
| regulation of protein targeting to membrane (GO:00 | 44590 | 0.061915083 | 0.122677034 |
| negative regulation of mRNA catabolic process (GO:1 | 44590 | 0.061915083 | 0.122677034 |
| transition metal ion homeostasis (GO:0055076) | 44590 | 0.061915083 | 0.122677034 |
| positive regulation of viral genome replication (GO:0 | 44590 | 0.061915083 | 0.122677034 |
| MyD88-dependent toll-like receptor signaling pathw | 44590 | 0.061915083 | 0.122677034 |
| negative regulation of transcription by RNA polymer | 4/684 | 0.062738066 | 0.122677034 |
| extrinsic apoptotic signaling pathway via death doma | 44591 | 0.063981862 | 0.122677034 |
| negative regulation of cytoskeleton organization (GC | 44591 | 0.063981862 | 0.122677034 |
| regulation of type I interferon-mediated signaling pa | 44591 | 0.063981862 | 0.122677034 |
| positive regulation of phosphate metabolic process ( | 44591 | 0.063981862 | 0.122677034 |
| long-chain fatty acid biosynthetic process (GO:0042 | 44591 | 0.063981862 | 0.122677034 |
| positive regulation of protein import (GO:1904591) | 44591 | 0.063981862 | 0.122677034 |
| regulation of macrophage activation (GO:0043030) | 44591 | 0.063981862 | 0.122677034 |
| regulation of protein localization to membrane (GO: | 44591 | 0.063981862 | 0.122677034 |
| negative regulation of axon extension (GO:0030517) | 44592 | 0.066044191 | 0.122677034 |
| purine nucleoside bisphosphate metabolic process ( | 44592 | 0.066044191 | 0.122677034 |
| positive regulation of leukocyte cell-cell adhesion (G | 44592 | 0.066044191 | 0.122677034 |
| ribonucleoside bisphosphate metabolic process (GO: | 44592 | 0.066044191 | 0.122677034 |
| negative regulation of proteasomal ubiquitin-depend | 44592 | 0.066044191 | 0.122677034 |
| positive regulation of protein targeting to mitochon | 44592 | 0.066044191 | 0.122677034 |
| positive regulation of Rho protein signal transductio | 44592 | 0.066044191 | 0.122677034 |

| | | | |
|---|---|---|---|
| positive regulation of RNA splicing (GO:0033120) | 44592 | 0.066044191 | 0.122677034 |
| prostaglandin metabolic process (GO:0006693) | 44592 | 0.066044191 | 0.122677034 |
| protein acetylation (GO:0006473) | 44592 | 0.066044191 | 0.122677034 |
| regulation of programmed cell death (GO:0043067) | 2/194 | 0.067915793 | 0.122677034 |
| ERBB2 signaling pathway (GO:0038128) | 11689 | 0.068102079 | 0.122677034 |
| protein transmembrane import into intracellular org | 11689 | 0.068102079 | 0.122677034 |
| establishment or maintenance of epithelial cell apica | 11689 | 0.068102079 | 0.122677034 |
| negative regulation of developmental growth (GO:0( | 11689 | 0.068102079 | 0.122677034 |
| B cell proliferation (GO:0042100) | 11689 | 0.068102079 | 0.122677034 |
| regulation of cell migration involved in sprouting ang | 11689 | 0.068102079 | 0.122677034 |
| regulation of cellular amide metabolic process (GO:0 | 11689 | 0.068102079 | 0.122677034 |
| negative regulation of microtubule polymerization o | 11689 | 0.068102079 | 0.122677034 |
| positive regulation of interleukin-10 production (GO: | 11689 | 0.068102079 | 0.122677034 |
| apoptotic mitochondrial changes (GO:0008637) | 12055 | 0.070155536 | 0.124248526 |
| negative regulation of JNK cascade (GO:0046329) | 12055 | 0.070155536 | 0.124248526 |
| RNA stabilization (GO:0043489) | 12055 | 0.070155536 | 0.124248526 |
| negative regulation of smooth muscle cell proliferati | 12055 | 0.070155536 | 0.124248526 |
| regulation of protein localization to cell surface (GO: | 12055 | 0.070155536 | 0.124248526 |
| negative regulation of mitochondrion organization (( | 12420 | 0.072204571 | 0.126178087 |
| positive regulation of interleukin-12 production (GO: | 12420 | 0.072204571 | 0.126178087 |
| positive regulation of protein dephosphorylation (G( | 12420 | 0.072204571 | 0.126178087 |
| protein insertion into membrane (GO:0051205) | 12420 | 0.072204571 | 0.126178087 |
| negative regulation of epithelial cell migration (GO:0 | 12785 | 0.074249193 | 0.126802193 |
| negative regulation of neurogenesis (GO:0050768) | 12785 | 0.074249193 | 0.126802193 |
| positive regulation of lymphocyte activation (GO:005 | 12785 | 0.074249193 | 0.126802193 |
| cellular response to virus (GO:0098586) | 12785 | 0.074249193 | 0.126802193 |
| regulation of JUN kinase activity (GO:0043506) | 12785 | 0.074249193 | 0.126802193 |
| regulation of mitotic spindle organization (GO:00602 | 12785 | 0.074249193 | 0.126802193 |
| regulation of protein polymerization (GO:0032271) | 12785 | 0.074249193 | 0.126802193 |
| regulation of protein kinase B signaling (GO:005189( | 2/207 | 0.075991802 | 0.127796911 |
| negative regulation of cellular protein localization (G | 13150 | 0.076289411 | 0.127796911 |
| positive regulation of cytokine-mediated signaling pa | 13150 | 0.076289411 | 0.127796911 |
| regulation of defense response to virus by host (GO: | 13150 | 0.076289411 | 0.127796911 |
| positive regulation of T cell mediated immunity (GO: | 13150 | 0.076289411 | 0.127796911 |
| regulation of protein import into nucleus (GO:00423 | 13150 | 0.076289411 | 0.127796911 |
| acyl-CoA metabolic process (GO:0006637) | 13516 | 0.078325235 | 0.128747105 |
| regulation of transport (GO:0051049) | 13516 | 0.078325235 | 0.128747105 |
| regulation of erythrocyte differentiation (GO:004564 | 13516 | 0.078325235 | 0.128747105 |
| positive regulation of myeloid cell differentiation (G( | 13516 | 0.078325235 | 0.128747105 |
| regulation of glucose import (GO:0046324) | 13516 | 0.078325235 | 0.128747105 |
| protein insertion into ER membrane (GO:0045048) | 13516 | 0.078325235 | 0.128747105 |
| negative regulation of endothelial cell migration (GO | 13881 | 0.080356674 | 0.129655247 |
| regulation of axon extension (GO:0030516) | 13881 | 0.080356674 | 0.129655247 |
| negative regulation of proteasomal protein catabolic | 13881 | 0.080356674 | 0.129655247 |
| negative regulation of ubiquitin-dependent protein c | 13881 | 0.080356674 | 0.129655247 |
| lymphocyte proliferation (GO:0046651) | 13881 | 0.080356674 | 0.129655247 |
| regulation of phosphoprotein phosphatase activity (( | 13881 | 0.080356674 | 0.129655247 |

| | | | |
|---|---|---|---|
| regulation of protein targeting to mitochondrion (GC | 14246 | 0.082383737 | 0.131713816 |
| regulation of protein tyrosine kinase activity (GO:00( | 14246 | 0.082383737 | 0.131713816 |
| regulation of nitric-oxide synthase activity (GO:0050 | 14246 | 0.082383737 | 0.131713816 |
| cellular response to cytokine stimulus (GO:0071345) | 3/482 | 0.08927868 | 0.142304805 |
| signal release from synapse (GO:0099643) | 15707 | 0.090448414 | 0.143733733 |
| intracellular protein transmembrane transport (GO:( | 16072 | 0.092453735 | 0.145166164 |
| neurotransmitter secretion (GO:0007269) | 16072 | 0.092453735 | 0.145166164 |
| mRNA catabolic process (GO:0006402) | 16072 | 0.092453735 | 0.145166164 |
| mRNA stabilization (GO:0048255) | 16072 | 0.092453735 | 0.145166164 |
| regulation of DNA repair (GO:0006282) | 16438 | 0.094454736 | 0.14699169 |
| positive regulation of RNA metabolic process (GO:00 | 16438 | 0.094454736 | 0.14699169 |
| microtubule bundle formation (GO:0001578) | 16438 | 0.094454736 | 0.14699169 |
| establishment of protein localization to extracellular | 16803 | 0.096451425 | 0.148778444 |
| positive regulation of nucleocytoplasmic transport (( | 16803 | 0.096451425 | 0.148778444 |
| positive regulation of smooth muscle cell proliferatic | 16803 | 0.096451425 | 0.148778444 |
| axonogenesis (GO:0007409) | 2/240 | 0.097747909 | 0.150077218 |
| epidermal growth factor receptor signaling pathway | 17168 | 0.098443811 | 0.150077218 |
| regulation of T cell activation (GO:0050863) | 17168 | 0.098443811 | 0.150077218 |
| telomere organization (GO:0032200) | 17168 | 0.098443811 | 0.150077218 |
| establishment of protein localization to mitochondri | 17533 | 0.100431903 | 0.150077218 |
| positive regulation of blood vessel endothelial cell m | 17533 | 0.100431903 | 0.150077218 |
| purine ribonucleotide biosynthetic process (GO:0009 | 17533 | 0.100431903 | 0.150077218 |
| positive regulation of DNA repair (GO:0045739) | 17533 | 0.100431903 | 0.150077218 |
| negative regulation of MAP kinase activity (GO:0043 | 17533 | 0.100431903 | 0.150077218 |
| regulation of interleukin-10 production (GO:003265 | 17533 | 0.100431903 | 0.150077218 |
| regulation of protein metabolic process (GO:005124 | 17533 | 0.100431903 | 0.150077218 |
| negative regulation of catabolic process (GO:000989 | 17899 | 0.10241571 | 0.150476714 |
| regulation of smooth muscle cell proliferation (GO:0 | 17899 | 0.10241571 | 0.150476714 |
| response to hydrogen peroxide (GO:0042542) | 17899 | 0.10241571 | 0.150476714 |
| RNA catabolic process (GO:0006401) | 17899 | 0.10241571 | 0.150476714 |
| cellular response to mechanical stimulus (GO:00712( | 17899 | 0.10241571 | 0.150476714 |
| positive regulation of actin filament polymerization ( | 17899 | 0.10241571 | 0.150476714 |
| regulation of generation of precursor metabolites ar | 18264 | 0.104395242 | 0.152957932 |
| regulation of axonogenesis (GO:0050770) | 18629 | 0.106370508 | 0.154988607 |
| regulation of interleukin-12 production (GO:003265 | 18629 | 0.106370508 | 0.154988607 |
| positive regulation of phosphorylation (GO:0042327 | 2/253 | 0.106756192 | 0.155120875 |
| negative regulation of protein metabolic process (GC | 18994 | 0.108341516 | 0.156990736 |
| positive regulation of macroautophagy (GO:001623 | 19360 | 0.110308275 | 0.158964801 |
| positive regulation of NIK/NF-kappaB signaling (GO:1 | 19360 | 0.110308275 | 0.158964801 |
| regulation of cyclin-dependent protein kinase activit | 19725 | 0.112270794 | 0.160911274 |
| regulation of proteasomal protein catabolic process | 19725 | 0.112270794 | 0.160911274 |
| positive regulation of response to DNA damage stim | 20090 | 0.114229082 | 0.163273091 |
| protein-containing complex disassembly (GO:00329 | 20455 | 0.116183149 | 0.164280474 |
| positive regulation of establishment of protein locali | 20455 | 0.116183149 | 0.164280474 |
| telomere maintenance (GO:0000723) | 20455 | 0.116183149 | 0.164280474 |
| mitochondrial transport (GO:0006839) | 20455 | 0.116183149 | 0.164280474 |
| arachidonic acid metabolic process (GO:0019369) | 20821 | 0.118133003 | 0.166589703 |

| | | | |
|---|---|---|---|
| regulation of tumor necrosis factor-mediated signali | 21186 | 0.120078652 | 0.168430323 |
| cellular iron ion homeostasis (GO:0006879) | 21186 | 0.120078652 | 0.168430323 |
| response to reactive oxygen species (GO:0000302) | 21551 | 0.122020106 | 0.17069834 |
| iron ion homeostasis (GO:0055072) | 22282 | 0.125890464 | 0.17518091 |
| pattern recognition receptor signaling pathway (GO: | 22282 | 0.125890464 | 0.17518091 |
| I-kappaB kinase/NF-kappaB signaling (GO:0007249) | 22647 | 0.127819385 | 0.177395769 |
| positive regulation of DNA metabolic process (GO:00 | 23012 | 0.129744146 | 0.178652935 |
| positive regulation of Ras protein signal transductior | 23012 | 0.129744146 | 0.178652935 |
| positive regulation of viral process (GO:0048524) | 23012 | 0.129744146 | 0.178652935 |
| protein secretion (GO:0009306) | 23377 | 0.131664756 | 0.180353286 |
| regulation of innate immune response (GO:0045088 | 23377 | 0.131664756 | 0.180353286 |
| regulation of RNA metabolic process (GO:0051252) | 23743 | 0.133581224 | 0.182503178 |
| cellular response to nutrient levels (GO:0031669) | 24108 | 0.135493557 | 0.184159202 |
| positive regulation of T cell proliferation (GO:00421( | 24108 | 0.135493557 | 0.184159202 |
| regulation of viral genome replication (GO:0045069) | 24473 | 0.137401765 | 0.186271466 |
| regulation of translational initiation (GO:0006446) | 24838 | 0.139305857 | 0.18788431 |
| negative regulation of transforming growth factor be | 24838 | 0.139305857 | 0.18788431 |
| positive regulation of protein transport (GO:005122 | 25204 | 0.141205842 | 0.189959777 |
| wound healing (GO:0042060) | 25569 | 0.143101727 | 0.192019154 |
| fatty acid biosynthetic process (GO:0006633) | 25934 | 0.144993521 | 0.193079981 |
| histone acetylation (GO:0016573) | 25934 | 0.144993521 | 0.193079981 |
| regulation of glycolytic process (GO:0006110) | 25934 | 0.144993521 | 0.193079981 |
| extrinsic apoptotic signaling pathway (GO:0097191) | 26299 | 0.146881234 | 0.193632905 |
| negative regulation of supramolecular fiber organiza | 26299 | 0.146881234 | 0.193632905 |
| positive regulation of protein secretion (GO:005071 | 26299 | 0.146881234 | 0.193632905 |
| positive regulation of secretion by cell (GO:1903532) | 26299 | 0.146881234 | 0.193632905 |
| regulation of Rho protein signal transduction (GO:00 | 26665 | 0.148764874 | 0.194169538 |
| regulation of actin filament polymerization (GO:003( | 26665 | 0.148764874 | 0.194169538 |
| neurotransmitter transport (GO:0006836) | 26665 | 0.148764874 | 0.194169538 |
| endoplasmic reticulum organization (GO:0007029) | 26665 | 0.148764874 | 0.194169538 |
| regulation of cytokine-mediated signaling pathway (( | 27030 | 0.150644449 | 0.196136089 |
| positive regulation of lymphocyte proliferation (GO:( | 27395 | 0.152519968 | 0.198087662 |
| regulation of T cell proliferation (GO:0042129) | 27760 | 0.154391439 | 0.200024377 |
| cytokine-mediated signaling pathway (GO:0019221) | 3/621 | 0.155794096 | 0.200468699 |
| purine ribonucleotide metabolic process (GO:00091! | 28126 | 0.156258871 | 0.200468699 |
| positive regulation of endothelial cell proliferation (( | 28126 | 0.156258871 | 0.200468699 |
| cellular response to UV (GO:0034644) | 28126 | 0.156258871 | 0.200468699 |
| negative regulation of apoptotic signaling pathway (( | 28491 | 0.158122273 | 0.202365732 |
| positive regulation of proteasomal ubiquitin-depend | 28856 | 0.159981653 | 0.203753873 |
| positive regulation of ubiquitin-dependent protein c | 28856 | 0.159981653 | 0.203753873 |
| regulation of NIK/NF-kappaB signaling (GO:1901222) | 29221 | 0.16183702 | 0.205619015 |
| positive regulation of cytoskeleton organization (GO | 29952 | 0.165535746 | 0.209307217 |
| ephrin receptor signaling pathway (GO:0048013) | 29952 | 0.165535746 | 0.209307217 |
| B cell activation (GO:0042113) | 31048 | 0.171053944 | 0.214735978 |
| regulation of cell cycle G2/M phase transition (GO:1! | 31048 | 0.171053944 | 0.214735978 |
| positive regulation of protein catabolic process (GO:( | 31048 | 0.171053944 | 0.214735978 |
| negative regulation of phosphorylation (GO:004232( | 31413 | 0.172885407 | 0.216004095 |

| | | | |
|---|---|---|---|
| positive regulation of proteasomal protein catabolic | 31413 | 0.172885407 | 0.216004095 |
| positive regulation of inflammatory response (GO:00 | 32509 | 0.1783561 | 0.220741902 |
| regulation of endothelial cell migration (GO:001059 | 32509 | 0.1783561 | 0.220741902 |
| collagen fibril organization (GO:0030199) | 32509 | 0.1783561 | 0.220741902 |
| post-Golgi vesicle-mediated transport (GO:0006892) | 32509 | 0.1783561 | 0.220741902 |
| supramolecular fiber organization (GO:0097435) | 2/351 | 0.18041891 | 0.222770767 |
| positive regulation of supramolecular fiber organizat | 33239 | 0.181983566 | 0.224176477 |
| positive regulation of transmembrane receptor prot | 33604 | 0.183791425 | 0.225874508 |
| ciliary basal body-plasma membrane docking (GO:00 | 34700 | 0.189191607 | 0.231429733 |
| mRNA metabolic process (GO:0016071) | 34700 | 0.189191607 | 0.231429733 |
| cellular transition metal ion homeostasis (GO:00469 | 35065 | 0.190983896 | 0.233080115 |
| positive regulation of defense response (GO:003134 | 35796 | 0.194556867 | 0.236891 |
| positive regulation of protein phosphorylation (GO:0 | 2/371 | 0.196323949 | 0.238490525 |
| cellular response to chemical stress (GO:0062197) | 1/101 | 0.199887426 | 0.242172088 |
| positive regulation of cellular protein metabolic proc | 1/102 | 0.201656605 | 0.242172088 |
| regulation of cell death (GO:0010941) | 1/102 | 0.201656605 | 0.242172088 |
| positive regulation of vasculature development (GO: | 1/102 | 0.201656605 | 0.242172088 |
| mitotic sister chromatid segregation (GO:0000070) | 1/102 | 0.201656605 | 0.242172088 |
| positive regulation of macromolecule metabolic proc | 2/384 | 0.206760787 | 0.247736159 |
| negative regulation of transmembrane receptor prot | 1/108 | 0.212191676 | 0.253665504 |
| response to endoplasmic reticulum stress (GO:0034 | 1/110 | 0.215673081 | 0.257242722 |
| protein-containing complex subunit organization (G | 1/112 | 0.219139449 | 0.26078586 |
| histone modification (GO:0016570) | 1/114 | 0.222590844 | 0.26351637 |
| positive regulation of kinase activity (GO:0033674) | 1/114 | 0.222590844 | 0.26351637 |
| transmembrane receptor protein tyrosine kinase sig | 2/404 | 0.222936853 | 0.26351637 |
| cellular amide metabolic process (GO:0043603) | 1/115 | 0.224310946 | 0.264546093 |
| positive regulation of angiogenesis (GO:0045766) | 1/116 | 0.226027329 | 0.265973993 |
| negative regulation of response to external stimulus | 1/118 | 0.229448966 | 0.268797676 |
| positive regulation of protein-containing complex as | 1/118 | 0.229448966 | 0.268797676 |
| regulation of anatomical structure morphogenesis ( | 1/123 | 0.237938516 | 0.278123688 |
| fatty acid metabolic process (GO:0006631) | 1/124 | 0.239625424 | 0.278856134 |
| defense response to symbiont (GO:0140546) | 1/124 | 0.239625424 | 0.278856134 |
| regulation of phosphorylation (GO:0042325) | 1/125 | 0.241308681 | 0.2795779 |
| regulation of protein secretion (GO:0050708) | 1/125 | 0.241308681 | 0.2795779 |
| negative regulation of growth (GO:0045926) | 1/126 | 0.242988297 | 0.280289133 |
| circulatory system development (GO:0072359) | 1/126 | 0.242988297 | 0.280289133 |
| positive regulation of macromolecule biosynthetic p | 1/129 | 0.248005372 | 0.285450384 |
| G2/M transition of mitotic cell cycle (GO:0000086) | 1/130 | 0.249670498 | 0.28673948 |
| cell cycle G2/M phase transition (GO:0044839) | 1/131 | 0.25133202 | 0.287392701 |
| cellular response to metal ion (GO:0071248) | 1/131 | 0.25133202 | 0.287392701 |
| defense response to virus (GO:0051607) | 1/133 | 0.254644285 | 0.290548577 |
| positive regulation of response to external stimulus ( | 1/139 | 0.264495386 | 0.30113544 |
| positive regulation of cellular catabolic process (GO: | 1/141 | 0.267750723 | 0.304183326 |
| monocarboxylic acid metabolic process (GO:003278 | 1/143 | 0.270991977 | 0.307202112 |
| negative regulation of cell migration (GO:0030336) | 1/144 | 0.272607342 | 0.308368736 |
| regulation of G2/M transition of mitotic cell cycle (G | 1/149 | 0.280631842 | 0.316764698 |
| negative regulation of protein phosphorylation (GO: | 1/152 | 0.285404947 | 0.321462531 |

| | | | |
|---|---|---|---|
| positive regulation of gene expression (GO:0010628) | 2/482 | 0.286716992 | 0.322250294 |
| positive regulation of NF-kappaB transcription factor | 1/155 | 0.290147096 | 0.325410176 |
| regulation of cell differentiation (GO:0045595) | 1/156 | 0.291720968 | 0.326479211 |
| cellular response to starvation (GO:0009267) | 1/158 | 0.294858487 | 0.329289945 |
| heart development (GO:0007507) | 1/164 | 0.304189769 | 0.338991141 |
| positive regulation of I-kappaB kinase/NF-kappaB sig | 1/171 | 0.314923805 | 0.35021125 |
| positive regulation of ERK1 and ERK2 cascade (GO:0( | 1/172 | 0.316443967 | 0.351159339 |
| mitochondrion organization (GO:0007005) | 1/175 | 0.320984703 | 0.355448324 |
| positive regulation of developmental process (GO:0( | 1/177 | 0.32399547 | 0.358028607 |
| regulation of mitotic cell cycle phase transition (GO:: | 1/188 | 0.340322933 | 0.375282731 |
| regulation of GTPase activity (GO:0043087) | 1/189 | 0.341787991 | 0.376109798 |
| negative regulation of transcription, DNA-templated | 3/948 | 0.347007258 | 0.381055987 |
| negative regulation of macromolecule metabolic prc | 1/194 | 0.349065721 | 0.382517852 |
| regulation of signal transduction (GO:0009966) | 1/198 | 0.354831229 | 0.387385459 |
| negative regulation of cellular process (GO:0048523) | 2/566 | 0.354980592 | 0.387385459 |
| regulation of angiogenesis (GO:0045765) | 1/203 | 0.361967969 | 0.394192861 |
| regulation of inflammatory response (GO:0050727) | 1/206 | 0.366212919 | 0.397991727 |
| mitotic cell cycle phase transition (GO:0044772) | 1/209 | 0.370430265 | 0.401744988 |
| phosphate-containing compound metabolic process | 1/212 | 0.37462018 | 0.405453117 |
| positive regulation of GTPase activity (GO:0043547) | 1/214 | 0.377398305 | 0.406785878 |
| positive regulation of protein modification process (( | 1/214 | 0.377398305 | 0.406785878 |
| regulation of cell growth (GO:0001558) | 1/217 | 0.381542891 | 0.41041219 |
| regulation of I-kappaB kinase/NF-kappaB signaling (( | 1/224 | 0.391109017 | 0.419843558 |
| apoptotic process (GO:0006915) | 1/231 | 0.400530489 | 0.429081542 |
| regulation of ERK1 and ERK2 cascade (GO:0070372) | 1/238 | 0.409809443 | 0.438129608 |
| membrane organization (GO:0061024) | 1/242 | 0.415048527 | 0.44283068 |
| positive regulation of DNA-binding transcription fact | 1/246 | 0.420242152 | 0.447464316 |
| regulation of gene expression (GO:0010468) | 3/1079 | 0.426222628 | 0.452477547 |
| RNA splicing, via transesterification reactions with bu | 1/251 | 0.426670843 | 0.452477547 |
| positive regulation of signal transduction (GO:00099 | 1/252 | 0.427948195 | 0.452919015 |
| positive regulation of cell differentiation (GO:00455! | 1/258 | 0.435554108 | 0.460043094 |
| cellular component assembly (GO:0022607) | 1/261 | 0.439319914 | 0.46309073 |
| regulation of protein phosphorylation (GO:0001932) | 1/266 | 0.445541762 | 0.468709934 |
| protein-containing complex assembly (GO:0065003) | 1/267 | 0.44677801 | 0.469072322 |
| positive regulation of MAPK cascade (GO:0043410) | 1/274 | 0.455356635 | 0.476178112 |
| mRNA splicing, via spliceosome (GO:0000398) | 1/274 | 0.455356635 | 0.476178112 |
| DNA metabolic process (GO:0006259) | 1/277 | 0.458993265 | 0.479028685 |
| regulation of cell cycle (GO:0051726) | 1/296 | 0.481480306 | 0.501502259 |
| extracellular matrix organization (GO:0030198) | 1/300 | 0.486096675 | 0.504313316 |
| mRNA processing (GO:0006397) | 1/300 | 0.486096675 | 0.504313316 |
| MAPK cascade (GO:0000165) | 1/303 | 0.489532569 | 0.506878212 |
| chemical synaptic transmission (GO:0007268) | 1/306 | 0.49294601 | 0.509409826 |
| cilium assembly (GO:0060271) | 1/314 | 0.501939835 | 0.517686967 |
| regulation of cell population proliferation (GO:00421 | 2/764 | 0.505144013 | 0.519972116 |
| negative regulation of gene expression (GO:001062! | 1/322 | 0.510777694 | 0.524744272 |
| intracellular protein transport (GO:0006886) | 1/336 | 0.525876582 | 0.539202889 |
| cellular response to DNA damage stimulus (GO:0006 | 1/350 | 0.540519741 | 0.553138879 |

| | | | |
|---|---|---|---|
| gene expression (GO:0010467) | 1/356 | 0.546659218 | 0.558335434 |
| protein transport (GO:0015031) | 1/369 | 0.55968767 | 0.57053433 |
| organelle organization (GO:0006996) | 1/420 | 0.60735684 | 0.617929783 |
| negative regulation of nucleic acid-templated transc | 1/464 | 0.644400569 | 0.654352702 |
| positive regulation of cell population proliferation (G | 1/474 | 0.652330658 | 0.661128952 |
| protein phosphorylation (GO:0006468) | 1/496 | 0.669173374 | 0.676894605 |
| negative regulation of cellular macromolecule biosyn | 1/547 | 0.705209896 | 0.711977745 |
| regulation of transcription by RNA polymerase II (GC | 111856 | 0.730120497 | 0.73571529 |
| positive regulation of cellular process (GO:0048522) | 1/625 | 0.75302464 | 0.757344093 |
| positive regulation of transcription by RNA polymera | 1/908 | 0.870827861 | 0.874151631 |
| positive regulation of transcription, DNA-templated ( | 1/1183 | 0.931826799 | 0.933601707 |
| regulation of transcription, DNA-templated (GO:000( | 125676 | 0.965235434 | 0.965235434 |

**of significant highly-correlated RNA-seq-based modules (cyan; Significant terms**

| Old P-value | Old Adjusted P-value | Odds Ratio | Combined Score | Genes | KEGG |
|---|---|---|---|---|---|
| 0 | 0 | 232.0430108 | 12826.38247 | HSPA8;HSF | . |
| 0 | 0 | 97.49019608 | 3796.68677 | DNAJB1;HS | . |
| 0 | 0 | 61.83489097 | 2123.476966 | DNAJB1;HS | . |
| 0 | 0 | 251.5081081 | 7875.910109 | CCT2;HSP9 | . |
| 0 | 0 | 157.1216216 | 4492.683105 | DNAJB1;CC | . |
| 0 | 0 | 157.3894737 | 3896.518354 | DNAJB1;HS | . |
| 0 | 0 | 29.92840336 | 674.5029385 | CCT6A;CCT | . |
| 0 | 0 | 27.49444444 | 540.761169 | CCT2;HSP9 | . |
| 0 | 0 | 116.1655012 | 2273.188255 | HSPA8;HSF | . |
| 0 | 0 | 57.13205742 | 1104.284182 | CCT6A;CCT | . |
| 0 | 0 | 82.40281224 | 1486.713974 | HSP90AA1 | . |
| 0 | 0 | 71.17142857 | 1008.626002 | HSPA8;HSF | . |
| 0 | 0 | 66.42 | 924.6065911 | CCT2;HSP9 | . |
| 0 | 0 | 208.5261324 | 2846.511229 | CCT6A;CCT | . |
| 0 | 0 | 208.5261324 | 2846.511229 | DNAJB1;DN | . |
| 0 | 0 | 208.5261324 | 2846.511229 | DNAJB1;BA | . |
| 0 | 0 | 182.4512195 | 2432.750116 | CCT6A;CCT | . |
| 0 | 0 | 182.4512195 | 2432.750116 | DNAJB1;DN | . |
| 0 | 0 | 182.4512195 | 2432.750116 | CCT6A;CCT | . |
| 0 | 0 | 182.4512195 | 2432.750116 | CCT6A;CCT | . |
| 0 | 0 | 162.1707317 | 2115.931394 | CCT6A;CCT | . |
| 0 | 0 | 47.41428571 | 601.2932166 | CCT2;HSP9 | . |
| 0 | 0 | 121.6097561 | 1498.89976 | CCT6A;CCT | . |
| 0 | 0 | 97.27317073 | 1142.568606 | CCT6A;CCT | . |
| 0 | 0 | 91.18902439 | 1055.573524 | CCT6A;CCT | . |
| 0 | 0 | 85.82065997 | 979.6154458 | HSP90AA1 | . |
| 0 | 0 | 31.08125 | 345.29123 | CCT6A;CCT | . |
| 0 | 0 | 26.15789474 | 273.7355882 | DNAJB1;HS | . |
| 0 | 0 | 56.08818011 | 575.6726347 | HSP90AA1 | . |
| 0 | 0 | 54.00813008 | 548.7162099 | HSP90AA1 | . |
| 0 | 0 | 23.94337349 | 242.6467116 | DNAJB1;BA | . |
| 0 | 0 | 48.6 | 479.6311896 | CCT6A;CCT | . |
| 0 | 0 | 44.1751663 | 424.2831635 | HSP90AA1 | . |
| 0 | 0 | 44.1751663 | 424.2831635 | CCT6A;CCT | . |
| 0 | 0 | 237.5238095 | 2270.521513 | HSP90AA1 | . |
| 0 | 0 | 18.90571429 | 174.9403644 | DNAJA1;HS | . |
| 0 | 0 | 29.72672972 | 252.6548409 | CCT6A;CCT | . |
| 0 | 0 | 118.7380952 | 1005.249888 | HSP90AA1 | . |
| 0 | 0 | 28.00750469 | 233.3678171 | HSP90AA1 | . |
| 0 | 0 | 13.75833333 | 111.1303205 | CCT2;HSP9 | . |
| 0 | 0 | 86.34199134 | 683.850896 | HSP90AA1 | . |
| 0 | 0 | 73.05128205 | 557.0746629 | HSP90AA1 | . |
| 0 | 0 | 6.47567867 | 47.93469728 | HSPA8;CCT | . |
| 0 | 0 | 6.420283485 | 47.24594968 | HSPA8;CCT | . |

| | | | | | |
|---|---|---|---|---|---|
| 0 | 0 | 19.65919578 | 144.3108152 | HSP90AA1 | . |
| 0 | 0 | 6.379340467 | 46.7386946 | HSPA8;CCT | . |
| 0 | 0 | 18.89040228 | 136.5599222 | PEA15;HM | . |
| 0 | 0 | 52.74603175 | 371.3176123 | TLR7;HSPD | . |
| 0 | 0 | 43.14718615 | 287.8752745 | HSP90AA1 | . |
| 0 | 0 | 43.14718615 | 287.8752745 | HSP90AA1 | . |
| 0 | 0 | 15.29730424 | 101.5857752 | HSP90AA1 | . |
| 0 | 0 | 41.26915114 | 271.9616824 | TLR7;HSPD | . |
| 0 | 0 | 6.676213857 | 43.67710248 | DNAJA1;TI | . |
| 0 | 0 | 37.96380952 | 244.3251845 | FKBP4;PPI | . |
| 0 | 0 | 37.96380952 | 244.3251845 | HSP90AA1 | . |
| 0 | 0 | 36.5018315 | 232.2621485 | DNAJA1;HS | . |
| 0 | 0 | 36.5018315 | 232.2621485 | HSP90AA1 | . |
| 0 | 0 | 31.62857143 | 192.8370933 | FKBP4;PPI | . |
| 0 | 0 | 30.60675883 | 184.7363254 | HSP90AA1 | . |
| 0 | 0 | 29.64880952 | 177.1982383 | PEA15;HM | . |
| 0 | 0 | 27.90196078 | 163.5992917 | HSP90AA1 | . |
| 0 | 0 | 10.90573996 | 62.23968484 | HSP90AA1 | . |
| 0 | 0 | 25.63577864 | 146.258861 | HSPA8;HSF | . |
| 0 | 0 | 24.95989975 | 141.1573039 | HSP90AA1 | . |
| 0 | 0 | 23.70952381 | 131.8095305 | DNAJB1;AH | . |
| 0 | 0 | 23.70952381 | 131.8095305 | HMOX1;TL | . |
| 0 | 0 | 23.70952381 | 131.8095305 | PEA15;HM | . |
| 0 | 0 | 10.13798397 | 55.83921465 | HSPA8;TEN | . |
| 0 | 0 | 9.997140454 | 54.6830729 | HSPA1L;M | . |
| 0 | 0 | 21.54978355 | 115.9563361 | HSP90AA1 | . |
| 0 | 0 | 20.17122594 | 106.046538 | RTN4R;FKE | . |
| 0 | 0 | 20.17122594 | 106.046538 | HSP90AA1 | . |
| 0 | 0 | 20.17122594 | 106.046538 | HSP90AA1 | . |
| 0 | 0 | 4.123283753 | 21.51152412 | DNAJA1;HS | . |
| 0 | 0 | 19.34596696 | 100.1980519 | DNAJA1;HS | . |
| 0 | 0 | 18.58543417 | 94.86691228 | HMOX1;TL | . |
| 0 | 0 | 5.929003021 | 30.14384166 | HMOX1;TL | . |
| 0 | 0 | 17.88230009 | 89.99033326 | DNAJA1;HS | . |
| 0 | 0 | 17.23030303 | 85.51510117 | TLR7;HSPD | . |
| 0 | 0 | 16.92176871 | 83.41354267 | RTN4R;FKE | . |
| 0 | 0 | 16.05891848 | 77.59361647 | TLR7;HSPA | . |
| 0 | 0 | 15.27956989 | 72.41257982 | DNAJA1;HS | . |
| 0 | 0 | 15.27956989 | 72.41257982 | HSPA8;HSF | . |
| 0 | 0 | 14.80059524 | 69.26574678 | HSP90AA1 | . |
| 0 | 0 | 14.80059524 | 69.26574678 | HSP90AA1 | . |
| 0 | 0 | 14.57216117 | 67.77530918 | HSP90AA1 | . |
| 0 | 0 | 14.13574982 | 64.94701728 | TIGAR;HM | . |
| 0 | 0 | 14.13574982 | 64.94701728 | HSP90AA1 | . |
| 0 | 0 | 13.72463768 | 62.3062428 | HSP90AA1 | . |
| 0 | 0 | 13.72463768 | 62.3062428 | NECAB2;HS | . |
| 0 | 0 | 116 | 523.6437583 | BAG3 | . |

| | | | | | |
|---|---|---|---|---|---|
| 0 | 0 | 116 | 523.6437583 | TIGAR | . |
| 0 | 0 | 116 | 523.6437583 | CCT2 | . |
| 0 | 0 | 116 | 523.6437583 | HSPH1 | . |
| 0 | 0 | 116 | 523.6437583 | CCT2 | . |
| 0 | 0 | 116 | 523.6437583 | HSPA1A | . |
| 0 | 0 | 116 | 523.6437583 | HSPA8 | . |
| 0 | 0 | 13.52789116 | 61.05074177 | HSP90AA1 | . |
| 0 | 0 | 13.15079365 | 58.65976046 | HSP90AA1 | . |
| 0 | 0 | 12.96999348 | 57.52071791 | NCK2;HSPI | . |
| 0 | 0 | 12.79407979 | 56.41710205 | RBM38;HS | . |
| 0 | 0 | 12.79407979 | 56.41710205 | TLR7;HSPD | . |
| 0 | 0 | 92.79534884 | 402.0750334 | DNAJA1 | . |
| 0 | 0 | 92.79534884 | 402.0750334 | HSPA8 | . |
| 0 | 0 | 92.79534884 | 402.0750334 | HSPA1A | . |
| 0 | 0 | 92.79534884 | 402.0750334 | HSPD1 | . |
| 0 | 0 | 92.79534884 | 402.0750334 | BAG3 | . |
| 0 | 0 | 92.79534884 | 402.0750334 | HSPA1A | . |
| 0 | 0 | 92.79534884 | 402.0750334 | NCK2 | . |
| 0 | 0 | 92.79534884 | 402.0750334 | NECAB2 | . |
| 0 | 0 | 92.79534884 | 402.0750334 | NAA50 | . |
| 0 | 0 | 92.79534884 | 402.0750334 | ACOT7 | . |
| 0 | 0 | 92.79534884 | 402.0750334 | HSPD1 | . |
| 0 | 0 | 92.79534884 | 402.0750334 | MTCL1 | . |
| 0 | 0 | 92.79534884 | 402.0750334 | HSPH1 | . |
| 0 | 0 | 92.79534884 | 402.0750334 | HSPA1A | . |
| 0 | 0 | 11.98131404 | 51.3794865 | HSPE1;HSP | . |
| 0 | 0 | 11.98131404 | 51.3794865 | TLR7;HSPA | . |
| 0 | 0 | 77.3255814 | 323.208935 | RTN4R | . |
| 0 | 0 | 77.3255814 | 323.208935 | HSPD1 | . |
| 0 | 0 | 77.3255814 | 323.208935 | HSP90AB1 | . |
| 0 | 0 | 77.3255814 | 323.208935 | HSPA1A | . |
| 0 | 0 | 77.3255814 | 323.208935 | HSPA1A | . |
| 0 | 0 | 77.3255814 | 323.208935 | HSPH1 | . |
| 0 | 0 | 77.3255814 | 323.208935 | HSPH1 | . |
| 0 | 0 | 11.26530612 | 47.02931709 | TLR7;HSPD | . |
| 0 | 0 | 10.87520525 | 44.69580966 | HSP90AA1 | . |
| 0 | 0 | 10.62974853 | 43.24132174 | TLR7;HSPD | . |
| 0 | 0 | 66.27574751 | 268.2435667 | HMOX1 | . |
| 0 | 0 | 66.27574751 | 268.2435667 | PEA15 | . |
| 0 | 0 | 66.27574751 | 268.2435667 | BAG3 | . |
| 0 | 0 | 66.27574751 | 268.2435667 | RTN4R | . |
| 0 | 0 | 66.27574751 | 268.2435667 | HSP90AB1 | . |
| 0 | 0 | 66.27574751 | 268.2435667 | DNAJA1 | . |
| 0 | 0 | 66.27574751 | 268.2435667 | BAG3 | . |
| 0 | 0 | 66.27574751 | 268.2435667 | P4HA1 | . |
| 0 | 0 | 10.51111111 | 42.54222889 | NCK2;HSPI | . |
| 0 | 0 | 10.39508111 | 41.86100377 | HSP90AA1 | . |

| | | | | | |
|---|---|---|---|---|---|
| 0 | 0 | 10.2815735 | 41.19701054 | TIGAR;HSP | . |
| 0 | 0 | 9.955388471 | 39.30248946 | NAA50;HSI | . |
| 0 | 0 | 57.98837209 | 227.9335161 | HMOX1 | . |
| 0 | 0 | 57.98837209 | 227.9335161 | TIGAR | . |
| 0 | 0 | 57.98837209 | 227.9335161 | HSPA1A | . |
| 0 | 0 | 9.649173955 | 37.54277574 | HSP90AB1, | . |
| 0 | 0 | 9.551226551 | 36.98384948 | NECAB2;M | . |
| 0 | 0 | 9.455238095 | 36.43799007 | HSP90AB1, | . |
| 0 | 0 | 4.048856549 | 15.52962456 | DNAJA1;B/ | . |
| 0 | 0 | 51.54263566 | 197.222132 | DNAJA1 | . |
| 0 | 0 | 51.54263566 | 197.222132 | TIGAR | . |
| 0 | 0 | 51.54263566 | 197.222132 | HMOX1 | . |
| 0 | 0 | 51.54263566 | 197.222132 | TLR7 | . |
| 0 | 0 | 51.54263566 | 197.222132 | TIGAR | . |
| 0 | 0 | 51.54263566 | 197.222132 | NECAB2 | . |
| 0 | 0 | 51.54263566 | 197.222132 | HSP90AB1 | . |
| 0 | 0 | 51.54263566 | 197.222132 | HSPA1A | . |
| 0 | 0 | 46.38604651 | 173.119691 | ACOT7 | . |
| 0 | 0 | 46.38604651 | 173.119691 | NECAB2 | . |
| 0 | 0 | 46.38604651 | 173.119691 | ACOT7 | . |
| 0 | 0 | 46.38604651 | 173.119691 | HSPD1 | . |
| 0 | 0 | 46.38604651 | 173.119691 | PPID | . |
| 0 | 0 | 46.38604651 | 173.119691 | ACOT7 | . |
| 0 | 0 | 46.38604651 | 173.119691 | HSP90AA1 | . |
| 0 | 0 | 46.38604651 | 173.119691 | PTGES3 | . |
| 0 | 0 | 46.38604651 | 173.119691 | P4HA1 | . |
| 0 | 0 | 8.751322751 | 32.49405409 | TLR7;HSPD | . |
| 0 | 0 | 5.086529346 | 18.78414146 | DNAJA1;PF | . |
| 0 | 0 | 42.16701903 | 153.7498329 | RTN4R | . |
| 0 | 0 | 42.16701903 | 153.7498329 | PEA15 | . |
| 0 | 0 | 42.16701903 | 153.7498329 | DNAJA1 | . |
| 0 | 0 | 42.16701903 | 153.7498329 | NECAB2 | . |
| 0 | 0 | 8.288220551 | 29.95881083 | HSPA8;HSF | . |
| 0 | 0 | 8.074481074 | 28.80549994 | HSPE1;HSP | . |
| 0 | 0 | 38.65116279 | 137.8779524 | HSP90AA1 | . |
| 0 | 0 | 38.65116279 | 137.8779524 | HSP90AB1 | . |
| 0 | 0 | 38.65116279 | 137.8779524 | TLR7 | . |
| 0 | 0 | 38.65116279 | 137.8779524 | BAG3 | . |
| 0 | 0 | 38.65116279 | 137.8779524 | DNAJA1 | . |
| 0 | 0 | 7.871428571 | 27.72004607 | HSPA8;HSF | . |
| 0 | 0 | 35.67620751 | 124.6598994 | HSP90AB1 | . |
| 0 | 0 | 35.67620751 | 124.6598994 | PPID | . |
| 0 | 0 | 35.67620751 | 124.6598994 | ACOT7 | . |
| 0 | 0 | 35.67620751 | 124.6598994 | NAA50 | . |
| 0 | 0 | 4.68316517 | 16.32797674 | DNAJA1;PF | . |
| 0 | 0 | 7.678281069 | 26.69699919 | HMOX1;HS | . |
| 0 | 0 | 7.615975422 | 26.36899078 | RTN4R;HSF | . |

| | | | | | |
|---|---|---|---|---|---|
| 0 | 0 | 3.581918819 | 12.38006646 | HSP90AA1 | . |
| 0 | 0 | 33.12624585 | 113.4998155 | HSPA1A | . |
| 0 | 0 | 33.12624585 | 113.4998155 | DNAJA1 | . |
| 0 | 0 | 33.12624585 | 113.4998155 | BAG3 | . |
| 0 | 0 | 33.12624585 | 113.4998155 | ACOT7 | . |
| 0 | 0 | 33.12624585 | 113.4998155 | PTGES3 | . |
| 0 | 0 | 7.318936877 | 24.81902311 | TIGAR;HM | . |
| 0 | 0 | 30.91627907 | 103.965655 | HMOX1 | . |
| 0 | 0 | 30.91627907 | 103.965655 | HMOX1 | . |
| 0 | 0 | 30.91627907 | 103.965655 | NAA50 | . |
| 0 | 0 | 30.91627907 | 103.965655 | TIGAR | . |
| 0 | 0 | 30.91627907 | 103.965655 | NAA50 | . |
| 0 | 0 | 30.91627907 | 103.965655 | HMOX1 | . |
| 0 | 0 | 28.98255814 | 95.73687424 | TIGAR | . |
| 0 | 0 | 28.98255814 | 95.73687424 | HSPD1 | . |
| 0 | 0 | 28.98255814 | 95.73687424 | HMOX1 | . |
| 0 | 0 | 6.888773027 | 22.61632351 | HSPA8;HSF | . |
| 0 | 0 | 27.27633379 | 88.57088447 | HMOX1 | . |
| 0 | 0 | 27.27633379 | 88.57088447 | HSP90AA1 | . |
| 0 | 0 | 27.27633379 | 88.57088447 | HSP90AB1 | . |
| 0 | 0 | 27.27633379 | 88.57088447 | HSPA1A | . |
| 0 | 0 | 27.27633379 | 88.57088447 | HMOX1 | . |
| 0 | 0 | 6.740136054 | 21.86723097 | DNAJA1;HI | . |
| 0 | 0 | 6.691995947 | 21.62598154 | HSP90AA1 | . |
| 0 | 0 | 25.75968992 | 82.28088014 | MTCL1 | . |
| 0 | 0 | 25.75968992 | 82.28088014 | HSP90AA1 | . |
| 0 | 0 | 25.75968992 | 82.28088014 | HMOX1 | . |
| 0 | 0 | 25.75968992 | 82.28088014 | NECAB2 | . |
| 0 | 0 | 25.75968992 | 82.28088014 | HSPD1 | . |
| 0 | 0 | 25.75968992 | 82.28088014 | HSPA1A | . |
| 0 | 0 | 25.75968992 | 82.28088014 | HSP90AA1 | . |
| 0 | 0 | 25.75968992 | 82.28088014 | PTGES3 | . |
| 0 | 0 | 6.416909621 | 20.26053037 | RTN4R;MT | . |
| 0 | 0 | 24.40269278 | 76.72079425 | HSPH1 | . |
| 0 | 0 | 24.40269278 | 76.72079425 | HSPA8 | . |
| 0 | 0 | 24.40269278 | 76.72079425 | HSP90AB1 | . |
| 0 | 0 | 24.40269278 | 76.72079425 | HSP90AA1 | . |
| 0 | 0 | 6.204260652 | 19.22073326 | HSP90AA1 | . |
| 0 | 0 | 23.18139535 | 71.77485187 | HSP90AB1 | . |
| 0 | 0 | 23.18139535 | 71.77485187 | NCK2 | . |
| 0 | 0 | 23.18139535 | 71.77485187 | RTN4R | . |
| 0 | 0 | 23.18139535 | 71.77485187 | HSP90AB1 | . |
| 0 | 0 | 22.07641196 | 67.35016648 | HSPA1A | . |
| 0 | 0 | 22.07641196 | 67.35016648 | PPID | . |
| 0 | 0 | 5.929020665 | 17.89610065 | HSP90AA1 | . |
| 0 | 0 | 21.07188161 | 63.37139604 | HSPA8 | . |
| 0 | 0 | 21.07188161 | 63.37139604 | HSPA1A | . |

| | | | | | |
|---|---|---|---|---|---|
| 0 | 0 | 21.07188161 | 63.37139604 | HSPA1A | . |
| 0 | 0 | 21.07188161 | 63.37139604 | HSP90AB1 | . |
| 0 | 0 | 21.07188161 | 63.37139604 | HSP90AA1 | . |
| 0 | 0 | 21.07188161 | 63.37139604 | BAG3 | . |
| 0 | 0 | 3.810326933 | 11.37550978 | NCK2;HMC | . |
| 0 | 0 | 5.78235466 | 17.20037584 | RTN4R;FKE | . |
| 0 | 0 | 20.15470172 | 59.77682212 | HSP90AB1 | . |
| 0 | 0 | 20.15470172 | 59.77682212 | ACOT7 | . |
| 0 | 0 | 20.15470172 | 59.77682212 | MTCL1 | . |
| 0 | 0 | 20.15470172 | 59.77682212 | HSP90AB1 | . |
| 0 | 0 | 20.15470172 | 59.77682212 | HSP90AA1 | . |
| 0 | 0 | 19.31395349 | 56.51543007 | TIGAR | . |
| 0 | 0 | 19.31395349 | 56.51543007 | HSPA1A | . |
| 0 | 0 | 19.31395349 | 56.51543007 | HSPA1A | . |
| 0 | 0 | 18.54046512 | 53.54470544 | HSP90AB1 | . |
| 0 | 0 | 17.82647585 | 50.82895022 | HMOX1 | . |
| 0 | 0 | 17.82647585 | 50.82895022 | HSPA1A | . |
| 0 | 0 | 17.82647585 | 50.82895022 | NCK2 | . |
| 0 | 0 | 5.291064741 | 14.9239854 | HSP90AA1 | . |
| 0 | 0 | 17.16537468 | 48.3379819 | RTN4R | . |
| 0 | 0 | 17.16537468 | 48.3379819 | HSP90AA1 | . |
| 0 | 0 | 17.16537468 | 48.3379819 | HSPA1A | . |
| 0 | 0 | 17.16537468 | 48.3379819 | NAA50 | . |
| 0 | 0 | 17.16537468 | 48.3379819 | HSPA1A | . |
| 0 | 0 | 17.16537468 | 48.3379819 | BAG3 | . |
| 0 | 0 | 17.16537468 | 48.3379819 | FKBP4 | . |
| 0 | 0 | 16.55149502 | 46.04611786 | MTCL1 | . |
| 0 | 0 | 16.55149502 | 46.04611786 | TENT5C | . |
| 0 | 0 | 16.55149502 | 46.04611786 | HMOX1 | . |
| 0 | 0 | 16.55149502 | 46.04611786 | PPID | . |
| 0 | 0 | 16.55149502 | 46.04611786 | HSPD1 | . |
| 0 | 0 | 2.834705882 | 7.848696527 | DNAJB1;BA | . |
| 0 | 0 | 15.97995188 | 43.93137486 | BAG3 | . |
| 0 | 0 | 15.97995188 | 43.93137486 | FKBP4 | . |
| 0 | 0 | 15.97995188 | 43.93137486 | HSP90AB1 | . |
| 0 | 0 | 15.97995188 | 43.93137486 | PTGES3 | . |
| 0 | 0 | 15.97995188 | 43.93137486 | ACOT7 | . |
| 0 | 0 | 15.97995188 | 43.93137486 | BAG3 | . |
| 0 | 0 | 15.97995188 | 43.93137486 | HSPD1 | . |
| 0 | 0 | 15.97995188 | 43.93137486 | NECAB2 | . |
| 0 | 0 | 15.44651163 | 41.97483259 | RTN4R | . |
| 0 | 0 | 15.44651163 | 41.97483259 | ACOT7 | . |
| 0 | 0 | 15.44651163 | 41.97483259 | HSPD1 | . |
| 0 | 0 | 15.44651163 | 41.97483259 | ACOT7 | . |
| 0 | 0 | 15.44651163 | 41.97483259 | HSP90AB1 | . |
| 0 | 0 | 15.44651163 | 41.97483259 | HSPA1L | . |
| 0 | 0 | 15.44651163 | 41.97483259 | RTN4R | . |

| | | | | | |
|---|---|---|---|---|---|
| 0 | 0 | 15.44651163 | 41.97483259 | HSPA1A | . |
| 0 | 0 | 15.44651163 | 41.97483259 | PTGES3 | . |
| 0 | 0 | 15.44651163 | 41.97483259 | NAA50 | . |
| 0 | 0 | 4.901785714 | 13.18328741 | HSP90AA1 | . |
| 0 | 0 | 14.94748687 | 40.16012344 | HSP90AA1 | . |
| 0 | 0 | 14.94748687 | 40.16012344 | HSPD1 | . |
| 0 | 0 | 14.94748687 | 40.16012344 | MTCL1 | . |
| 0 | 0 | 14.94748687 | 40.16012344 | RTN4R | . |
| 0 | 0 | 14.94748687 | 40.16012344 | HSPD1 | . |
| 0 | 0 | 14.94748687 | 40.16012344 | HMOX1 | . |
| 0 | 0 | 14.94748687 | 40.16012344 | TIGAR | . |
| 0 | 0 | 14.94748687 | 40.16012344 | FKBP4 | . |
| 0 | 0 | 14.94748687 | 40.16012344 | HSPD1 | . |
| 0 | 0 | 14.47965116 | 38.47302036 | HSPD1 | . |
| 0 | 0 | 14.47965116 | 38.47302036 | DNAJA1 | . |
| 0 | 0 | 14.47965116 | 38.47302036 | TENT5C | . |
| 0 | 0 | 14.47965116 | 38.47302036 | HMOX1 | . |
| 0 | 0 | 14.47965116 | 38.47302036 | HSP90AB1 | . |
| 0 | 0 | 14.04016913 | 36.90110156 | HSPA1A | . |
| 0 | 0 | 14.04016913 | 36.90110156 | HSPD1 | . |
| 0 | 0 | 14.04016913 | 36.90110156 | HSP90AB1 | . |
| 0 | 0 | 14.04016913 | 36.90110156 | HMOX1 | . |
| 0 | 0 | 13.62653899 | 35.43347596 | DNAJA4 | . |
| 0 | 0 | 13.62653899 | 35.43347596 | RTN4R | . |
| 0 | 0 | 13.62653899 | 35.43347596 | HSPD1 | . |
| 0 | 0 | 13.62653899 | 35.43347596 | HSP90AA1 | . |
| 0 | 0 | 13.62653899 | 35.43347596 | DNAJA1 | . |
| 0 | 0 | 13.62653899 | 35.43347596 | HSPA1A | . |
| 0 | 0 | 13.62653899 | 35.43347596 | HSP90AA1 | . |
| 0 | 0 | 4.587921022 | 11.82366806 | HSP90AA1 | . |
| 0 | 0 | 13.23654485 | 34.06055692 | DNAJA1 | . |
| 0 | 0 | 13.23654485 | 34.06055692 | HSPA1A | . |
| 0 | 0 | 13.23654485 | 34.06055692 | HSP90AA1 | . |
| 0 | 0 | 13.23654485 | 34.06055692 | HSPD1 | . |
| 0 | 0 | 13.23654485 | 34.06055692 | BAG3 | . |
| 0 | 0 | 12.86821705 | 32.7738747 | ACOT7 | . |
| 0 | 0 | 12.86821705 | 32.7738747 | DNAJA1 | . |
| 0 | 0 | 12.86821705 | 32.7738747 | HSPA1A | . |
| 0 | 0 | 12.86821705 | 32.7738747 | HSPA1A | . |
| 0 | 0 | 12.86821705 | 32.7738747 | PEA15 | . |
| 0 | 0 | 12.86821705 | 32.7738747 | HMOX1 | . |
| 0 | 0 | 12.51979887 | 31.56592015 | DNAJA4 | . |
| 0 | 0 | 12.51979887 | 31.56592015 | RTN4R | . |
| 0 | 0 | 12.51979887 | 31.56592015 | HSP90AB1 | . |
| 0 | 0 | 12.51979887 | 31.56592015 | HSP90AB1 | . |
| 0 | 0 | 12.51979887 | 31.56592015 | HSPD1 | . |
| 0 | 0 | 12.51979887 | 31.56592015 | HSP90AB1 | . |

| | | | | | |
|---|---|---|---|---|---|
| 0 | 0 | 12.18971848 | 30.43001378 | HSPA1L | . |
| 0 | 0 | 12.18971848 | 30.43001378 | NCK2 | . |
| 0 | 0 | 12.18971848 | 30.43001378 | HSP90AA1 | . |
| 0 | 0 | 2.975253322 | 7.1881899 | HSPA8;HSF | . |
| 0 | 0 | 11.02657807 | 26.49659809 | HSPA8 | . |
| 0 | 0 | 10.76960519 | 25.64293525 | HSPD1 | . |
| 0 | 0 | 10.76960519 | 25.64293525 | HSPA8 | . |
| 0 | 0 | 10.76960519 | 25.64293525 | HSPA1A | . |
| 0 | 0 | 10.76960519 | 25.64293525 | TENT5C | . |
| 0 | 0 | 10.5243129 | 24.83353227 | TIGAR | . |
| 0 | 0 | 10.5243129 | 24.83353227 | HSPA1A | . |
| 0 | 0 | 10.5243129 | 24.83353227 | MTCL1 | . |
| 0 | 0 | 10.28992248 | 24.06520397 | PDIA4 | . |
| 0 | 0 | 10.28992248 | 24.06520397 | BAG3 | . |
| 0 | 0 | 10.28992248 | 24.06520397 | HMOX1 | . |
| 0 | 0 | 3.945178071 | 9.173972981 | HSP90AA1 | . |
| 0 | 0 | 10.06572295 | 23.33505696 | NCK2 | . |
| 0 | 0 | 10.06572295 | 23.33505696 | HSPD1 | . |
| 0 | 0 | 10.06572295 | 23.33505696 | PTGES3 | . |
| 0 | 0 | 9.85106383 | 22.64045732 | HSPD1 | . |
| 0 | 0 | 9.85106383 | 22.64045732 | HMOX1 | . |
| 0 | 0 | 9.85106383 | 22.64045732 | ACOT7 | . |
| 0 | 0 | 9.85106383 | 22.64045732 | TIGAR | . |
| 0 | 0 | 9.85106383 | 22.64045732 | DNAJA1 | . |
| 0 | 0 | 9.85106383 | 22.64045732 | HSPD1 | . |
| 0 | 0 | 9.85106383 | 22.64045732 | HSP90AB1 | . |
| 0 | 0 | 9.645348837 | 21.97900258 | TIGAR | . |
| 0 | 0 | 9.645348837 | 21.97900258 | HMOX1 | . |
| 0 | 0 | 9.645348837 | 21.97900258 | HMOX1 | . |
| 0 | 0 | 9.645348837 | 21.97900258 | HSPA1A | . |
| 0 | 0 | 9.645348837 | 21.97900258 | TLR7 | . |
| 0 | 0 | 9.645348837 | 21.97900258 | NCK2 | . |
| 0 | 0 | 9.448030375 | 21.34849709 | TIGAR | . |
| 0 | 0 | 9.258604651 | 20.74693057 | RTN4R | . |
| 0 | 0 | 9.258604651 | 20.74693057 | HSPD1 | . |
| 0 | 0 | 3.738379814 | 8.363531826 | HSP90AA1 | . |
| 0 | 0 | 9.076607387 | 20.17245912 | HSP90AB1 | . |
| 0 | 0 | 8.901610018 | 19.62338864 | BAG3 | . |
| 0 | 0 | 8.901610018 | 19.62338864 | TLR7 | . |
| 0 | 0 | 8.733216323 | 19.09816008 | HSP90AB1 | . |
| 0 | 0 | 8.733216323 | 19.09816008 | HSP90AB1 | . |
| 0 | 0 | 8.571059432 | 18.59533643 | TIGAR | . |
| 0 | 0 | 8.414799154 | 18.11359115 | HSPA8 | . |
| 0 | 0 | 8.414799154 | 18.11359115 | HSPA1L | . |
| 0 | 0 | 8.414799154 | 18.11359115 | PTGES3 | . |
| 0 | 0 | 8.414799154 | 18.11359115 | HSP90AA1 | . |
| 0 | 0 | 8.264119601 | 17.6516979 | PTGES3 | . |

| | | | | | |
|---|---|---|---|---|---|
| 0 | 0 | 8.11872705 | 17.20852138 | HSPA1A | . |
| 0 | 0 | 8.11872705 | 17.20852138 | HMOX1 | . |
| 0 | 0 | 7.978348035 | 16.78300913 | HMOX1 | . |
| 0 | 0 | 7.711627907 | 15.98113875 | HMOX1 | . |
| 0 | 0 | 7.711627907 | 15.98113875 | TLR7 | . |
| 0 | 0 | 7.584826535 | 15.60302779 | TLR7 | . |
| 0 | 0 | 7.462115529 | 15.23906422 | TIGAR | . |
| 0 | 0 | 7.462115529 | 15.23906422 | RTN4R | . |
| 0 | 0 | 7.462115529 | 15.23906422 | PPID | . |
| 0 | 0 | 7.343300111 | 14.88851389 | PDIA4 | . |
| 0 | 0 | 7.343300111 | 14.88851389 | HSP90AB1 | . |
| 0 | 0 | 7.228197674 | 14.55069131 | RBM38 | . |
| 0 | 0 | 7.116636852 | 14.2249557 | HSPA8 | . |
| 0 | 0 | 7.116636852 | 14.2249557 | NCK2 | . |
| 0 | 0 | 7.00845666 | 13.91070752 | PPID | . |
| 0 | 0 | 6.903505727 | 13.60738519 | NCK2 | . |
| 0 | 0 | 6.903505727 | 13.60738519 | HSPA1A | . |
| 0 | 0 | 6.801641587 | 13.31446223 | PPID | . |
| 0 | 0 | 6.70273003 | 13.03144454 | HMOX1 | . |
| 0 | 0 | 6.606644518 | 12.75786804 | ACOT7 | . |
| 0 | 0 | 6.606644518 | 12.75786804 | NAA50 | . |
| 0 | 0 | 6.606644518 | 12.75786804 | TIGAR | . |
| 0 | 0 | 6.51326564 | 12.4932964 | BAG3 | . |
| 0 | 0 | 6.51326564 | 12.4932964 | HSPA8 | . |
| 0 | 0 | 6.51326564 | 12.4932964 | PPID | . |
| 0 | 0 | 6.51326564 | 12.4932964 | PPID | . |
| 0 | 0 | 6.42248062 | 12.23731909 | RTN4R | . |
| 0 | 0 | 6.42248062 | 12.23731909 | NCK2 | . |
| 0 | 0 | 6.42248062 | 12.23731909 | HSPA8 | . |
| 0 | 0 | 6.42248062 | 12.23731909 | HMOX1 | . |
| 0 | 0 | 6.334182861 | 11.98954947 | HSP90AB1 | . |
| 0 | 0 | 6.248271527 | 11.74962315 | NCK2 | . |
| 0 | 0 | 6.164651163 | 11.51719639 | NCK2 | . |
| 0 | 0 | 2.289604547 | 4.256878653 | HSPA8;HSF | . |
| 0 | 0 | 6.083231334 | 11.29194472 | ACOT7 | . |
| 0 | 0 | 6.083231334 | 11.29194472 | HMOX1 | . |
| 0 | 0 | 6.083231334 | 11.29194472 | PPID | . |
| 0 | 0 | 6.003926306 | 11.07356161 | HSPA1A | . |
| 0 | 0 | 5.926654741 | 10.86175725 | HSPA1A | . |
| 0 | 0 | 5.926654741 | 10.86175725 | HSPA1A | . |
| 0 | 0 | 5.851339417 | 10.65625749 | TLR7 | . |
| 0 | 0 | 5.706287683 | 10.26314712 | NCK2 | . |
| 0 | 0 | 5.706287683 | 10.26314712 | NCK2 | . |
| 0 | 0 | 5.50166113 | 9.714702878 | HSPD1 | . |
| 0 | 0 | 5.50166113 | 9.714702878 | HSP90AA1 | . |
| 0 | 0 | 5.50166113 | 9.714702878 | HSP90AA1 | . |
| 0 | 0 | 5.436662107 | 9.542028607 | TIGAR | . |

| | | | | | |
|---|---|---|---|---|---|
| 0 | 0 | 5.436662107 | 9.542028607 | HSPA1A | . |
| 0 | 0 | 5.250528541 | 9.051770323 | TLR7 | . |
| 0 | 0 | 5.250528541 | 9.051770323 | DNAJA4 | . |
| 0 | 0 | 5.250528541 | 9.051770323 | P4HA1 | . |
| 0 | 0 | 5.250528541 | 9.051770323 | HSPA8 | . |
| 0 | 0 | 2.675262655 | 4.581317344 | HSP90AB1, | . |
| 0 | 0 | 5.133333333 | 8.746372989 | NCK2 | . |
| 0 | 0 | 5.076667519 | 8.599639846 | HSP90AB1 | . |
| 0 | 0 | 4.913904008 | 8.181625526 | HSP90AA1 | . |
| 0 | 0 | 4.913904008 | 8.181625526 | HSPA1A | . |
| 0 | 0 | 4.861933905 | 8.049253281 | HMOX1 | . |
| 0 | 0 | 4.761208343 | 7.794244625 | TLR7 | . |
| 0 | 0 | 2.527680991 | 4.115037311 | HSP90AA1 | . |
| 0 | 0 | 4.617674419 | 7.434460164 | HSPA1A | . |
| 0 | 0 | 4.571724614 | 7.320195173 | HSP90AA1 | . |
| 0 | 0 | 4.571724614 | 7.320195173 | HSPA1A | . |
| 0 | 0 | 4.571724614 | 7.320195173 | HMOX1 | . |
| 0 | 0 | 4.571724614 | 7.320195173 | NAA50 | . |
| 0 | 0 | 2.44003989 | 3.845973242 | DNAJA4;HS | . |
| 0 | 0 | 4.31406216 | 6.687940779 | HSPA1A | . |
| 0 | 0 | 4.234478344 | 6.495653924 | PDIA4 | . |
| 0 | 0 | 4.157762414 | 6.311678756 | PPID | . |
| 0 | 0 | 4.083762091 | 6.135525737 | NAA50 | . |
| 0 | 0 | 4.083762091 | 6.135525737 | TIGAR | . |
| 0 | 0 | 2.316275764 | 3.476421207 | HSP90AA1 | . |
| 0 | 0 | 4.047735618 | 6.050239634 | ACOT7 | . |
| 0 | 0 | 4.012335693 | 5.966741857 | HMOX1 | . |
| 0 | 0 | 3.943351222 | 5.804907341 | RTN4R | . |
| 0 | 0 | 3.943351222 | 5.804907341 | HSP90AA1 | . |
| 0 | 0 | 3.78078536 | 5.428236017 | HMOX1 | . |
| 0 | 0 | 3.749858196 | 5.357341072 | ACOT7 | . |
| 0 | 0 | 3.749858196 | 5.357341072 | TLR7 | . |
| 0 | 0 | 3.719429857 | 5.287832827 | PTGES3 | . |
| 0 | 0 | 3.719429857 | 5.287832827 | PPID | . |
| 0 | 0 | 3.689488372 | 5.219674142 | HSPA1A | . |
| 0 | 0 | 3.689488372 | 5.219674142 | CACYBP | . |
| 0 | 0 | 3.60247093 | 5.022942769 | HSPH1 | . |
| 0 | 0 | 3.574364521 | 4.959835532 | HSP90AA1 | . |
| 0 | 0 | 3.546690519 | 4.897910178 | HSP90AA1 | . |
| 0 | 0 | 3.546690519 | 4.897910178 | TIGAR | . |
| 0 | 0 | 3.492600423 | 4.777485049 | TLR7 | . |
| 0 | 0 | 3.339737108 | 4.441621488 | TLR7 | . |
| 0 | 0 | 3.291694352 | 4.33746193 | HSP90AA1 | . |
| 0 | 0 | 3.245004913 | 4.236892789 | ACOT7 | . |
| 0 | 0 | 3.222149943 | 4.187901829 | HMOX1 | . |
| 0 | 0 | 3.112507857 | 3.955099958 | HSP90AA1 | . |
| 0 | 0 | 3.050207916 | 3.824491729 | NCK2 | . |

| | | | | | |
|---|---|---|---|---|---|
| 0 | 0 | 1.932142857 | 2.413748091 | DNAJA4;HS | . |
| 0 | 0 | 2.990335246 | 3.700142917 | HSPA1A | . |
| 0 | 0 | 2.970892723 | 3.66001364 | HSP90AB1 | . |
| 0 | 0 | 2.932750704 | 3.581650372 | HSPA8 | . |
| 0 | 0 | 2.823940648 | 3.360781739 | CACYBP | . |
| 0 | 0 | 2.706703146 | 3.127391289 | HMOX1 | . |
| 0 | 0 | 2.690738474 | 3.095988151 | NECAB2 | . |
| 0 | 0 | 2.643945469 | 3.004478662 | HSPD1 | . |
| 0 | 0 | 2.613636364 | 2.945635473 | HSP90AB1 | . |
| 0 | 0 | 2.458525059 | 2.649946583 | HSP90AA1 | . |
| 0 | 0 | 2.445324097 | 2.62521349 | RTN4R | . |
| 0 | 0 | 1.472009292 | 1.55798874 | DNAJB1;HS | . |
| 0 | 0 | 2.38137125 | 2.506381484 | HSP90AB1 | . |
| 0 | 0 | 2.332546335 | 2.416781612 | NCK2 | . |
| 0 | 0 | 1.637284701 | 1.695722933 | NCK2;HSPA | . |
| 0 | 0 | 2.2742344 | 2.311075981 | HMOX1 | . |
| 0 | 0 | 2.240612592 | 2.250785797 | TLR7 | . |
| 0 | 0 | 2.207960644 | 2.192703795 | HSP90AA1 | . |
| 0 | 0 | 2.176237187 | 2.13672242 | TIGAR | . |
| 0 | 0 | 2.155584671 | 2.100518402 | RTN4R | . |
| 0 | 0 | 2.155584671 | 2.100518402 | HSP90AA1 | . |
| 0 | 0 | 2.125322997 | 2.047816732 | HSPA1A | . |
| 0 | 0 | 2.057878819 | 1.93187272 | HMOX1 | . |
| 0 | 0 | 1.994539939 | 1.82493501 | HSPD1 | . |
| 0 | 0 | 1.934942596 | 1.726090697 | NECAB2 | . |
| 0 | 0 | 1.902441378 | 1.672930531 | HSPA8 | . |
| 0 | 0 | 1.871001424 | 1.622016378 | HSPA1A | . |
| 0 | 0 | 1.283887932 | 1.094891243 | RBM38;DN | . |
| 0 | 0 | 1.833116279 | 1.561342902 | HSPA8 | . |
| 0 | 0 | 1.825720374 | 1.549585883 | NECAB2 | . |
| 0 | 0 | 1.782553615 | 1.481544922 | HSP90AB1 | . |
| 0 | 0 | 1.761717352 | 1.449060791 | PPID | . |
| 0 | 0 | 1.728038613 | 1.397057518 | HSP90AA1 | . |
| 0 | 0 | 1.7214548 | 1.386964822 | PPID | . |
| 0 | 0 | 1.67671863 | 1.319031544 | NECAB2 | . |
| 0 | 0 | 1.67671863 | 1.319031544 | HSPA8 | . |
| 0 | 0 | 1.658240647 | 1.291304729 | PTGES3 | . |
| 0 | 0 | 1.549940875 | 1.132836209 | HSP90AB1 | . |
| 0 | 0 | 1.528894765 | 1.102864807 | P4HA1 | . |
| 0 | 0 | 1.528894765 | 1.102864807 | HSPA8 | . |
| 0 | 0 | 1.513476051 | 1.081082426 | PEA15 | . |
| 0 | 0 | 1.498360656 | 1.059873836 | HSPA8 | . |
| 0 | 0 | 1.4594695 | 1.005975865 | HSP90AA1 | . |
| 0 | 0 | 1.199475066 | 0.819135574 | NCK2;HSPA | . |
| 0 | 0 | 1.422516844 | 0.955676438 | HSPA1A | . |
| 0 | 0 | 1.362096494 | 0.875404064 | BAG3 | . |
| 0 | 0 | 1.306523622 | 0.803804844 | TIGAR | . |

| | | | | | |
|---|---|---|---|---|---|
| 0 | 0 | 1.284048477 | 0.775474977 | HSPD1 | . |
| 0 | 0 | 1.237866532 | 0.718428501 | PDIA4 | . |
| 0 | 0 | 1.084364767 | 0.540706331 | PPID | . |
| 0 | 0 | 0.979104927 | 0.430252723 | HSPA8 | . |
| 0 | 0 | 0.957913368 | 0.409224137 | HMOX1 | . |
| 0 | 0 | 0.914305849 | 0.367287721 | TLR7 | . |
| 0 | 0 | 0.826731408 | 0.288744043 | HSPA8 | . |
| 0 | 0 | 0.80626703 | 0.253607823 | DNAJB1;N( | . |
| 0 | 0 | 0.720483005 | 0.204370285 | HSP90AB1 | . |
| 0 | 0 | 0.488423374 | 0.067554304 | NCK2 | . |
| 0 | 0 | 0.369377877 | 0.026081151 | NCK2 | . |
| 0 | 0 | 0.376237203 | 0.013312489 | HSPA8;HSF | . |

**s were highlighted)**

| Term | Overlap | P-value | Adjusted P-value | Old P-value |
|---|---|---|---|---|
| Protein processing in endoplasmic reticulum | 10/171 | 3.09E-12 | 1.67E-10 | 0 |
| Antigen processing and presentation | 28642 | 1.81E-08 | 4.90E-07 | 0 |
| Lipid and atherosclerosis | 7/215 | 4.11E-07 | 7.12E-06 | 0 |
| Estrogen signaling pathway | 6/137 | 5.27E-07 | 7.12E-06 | 0 |
| Legionellosis | 20911 | 7.39E-06 | 7.98E-05 | 0 |
| Measles | 4/139 | 2.44E-04 | 0.002199886 | 0 |
| Longevity regulating pathway | 3/102 | 0.001465377 | 0.011304339 | 0 |
| Toxoplasmosis | 3/112 | 0.001915685 | 0.012930875 | 0 |
| Prion disease | 4/273 | 0.003001999 | 0.018011991 | 0 |
| Fluid shear stress and atherosclerosis | 3/139 | 0.003532696 | 0.019076561 | 0 |
| Spliceosome | 3/150 | 0.00437269 | 0.021465934 | 0 |
| Necroptosis | 3/159 | 0.005142913 | 0.023143109 | 0 |
| Chemical carcinogenesis | 3/239 | 0.015567685 | 0.06466577 | 0 |
| Endocytosis | 3/252 | 0.01790492 | 0.064893655 | 0 |
| IL-17 signaling pathway | 34366 | 0.018190096 | 0.064893655 | 0 |
| Prostate cancer | 35462 | 0.019295932 | 0.064893655 | 0 |
| Progesterone-mediated oocyte maturation | 2/100 | 0.020429484 | 0.064893655 | 0 |
| Th17 cell differentiation | 2/107 | 0.023180005 | 0.069540014 | 0 |
| MAPK signaling pathway | 3/294 | 0.026731012 | 0.075972351 | 0 |
| Influenza A | 2/172 | 0.054976005 | 0.141230001 | 0 |
| Biosynthesis of unsaturated fatty acids | 44588 | 0.057768136 | 0.141230001 | 0 |
| Fatty acid elongation | 44588 | 0.057768136 | 0.141230001 | 0 |
| NOD-like receptor signaling pathway | 2/181 | 0.060153519 | 0.141230001 | 0 |
| Fructose and mannose metabolism | 12055 | 0.070155536 | 0.157849957 | 0 |
| Ferroptosis | 14977 | 0.086424772 | 0.180896828 | 0 |
| Type I diabetes mellitus | 15707 | 0.090448414 | 0.180896828 | 0 |
| Porphyrin and chlorophyll metabolism | 15707 | 0.090448414 | 0.180896828 | 0 |
| Salmonella infection | 2/249 | 0.103960387 | 0.187911436 | 0 |
| Arginine and proline metabolism | 18264 | 0.104395242 | 0.187911436 | 0 |
| Vibrio cholerae infection | 18264 | 0.104395242 | 0.187911436 | 0 |
| Pathways in cancer | 3/531 | 0.111084024 | 0.193501203 | 0 |
| Mineral absorption | 21916 | 0.123957374 | 0.206002577 | 0 |
| Arachidonic acid metabolism | 22282 | 0.125890464 | 0.206002577 | 0 |
| Central carbon metabolism in cancer | 25569 | 0.143101727 | 0.227279213 | 0 |
| Thyroid hormone synthesis | 27395 | 0.152519968 | 0.235316522 | 0 |
| RNA degradation | 28856 | 0.159981653 | 0.23997248 | 0 |
| ErbB signaling pathway | 31048 | 0.171053944 | 0.249646297 | 0 |
| PI3K-Akt signaling pathway | 2/354 | 0.18279147 | 0.259756299 | 0 |
| T cell receptor signaling pathway | 1/104 | 0.205183503 | 0.276997729 | 0 |
| Toll-like receptor signaling pathway | 1/104 | 0.205183503 | 0.276997729 | 0 |
| HIF-1 signaling pathway | 1/109 | 0.213934262 | 0.281767077 | 0 |
| Cellular senescence | 1/156 | 0.291720968 | 0.374248274 | 0 |
| Wnt signaling pathway | 1/166 | 0.307273296 | 0.374248274 | 0 |
| Hepatocellular carcinoma | 1/168 | 0.310343467 | 0.374248274 | 0 |

| | | | | |
|---|---|---|---|---|
| Tight junction | 1/169 | 0.311873561 | 0.374248274 | 0 |
| Tuberculosis | 1/180 | 0.328487172 | 0.380832613 | 0 |
| Axon guidance | 1/182 | 0.331465423 | 0.380832613 | 0 |
| Neutrophil extracellular trap formation | 1/189 | 0.341787991 | 0.38451149 | 0 |
| Pathogenic Escherichia coli infection | 1/197 | 0.353394548 | 0.389455216 | 0 |
| Coronavirus disease | 1/232 | 0.40186473 | 0.434013908 | 0 |
| Shigellosis | 1/246 | 0.420242152 | 0.444962279 | 0 |
| MicroRNAs in cancer | 1/310 | 0.497462585 | 0.516595761 | 0 |
| Alzheimer disease | 1/369 | 0.55968767 | 0.570247814 | 0 |
| Pathways of neurodegeneration | 1/475 | 0.653114095 | 0.653114095 | 0 |

| Old Adjusted P-value | Odds Ratio | Combined Score | Genes |
|---|---|---|---|
| 0 | 36.16185605 | 958.3708381 | DNAJA1;DNAJB1;HSPA8;HSP90AA1;HSP90AB1;HSPH1;H |
| 0 | 43.60526316 | 777.2496125 | HSPA8;HSP90AA1;HSP90AB1;HSPA1L;HSPA4;HSPA1A |
| 0 | 17.96205821 | 264.1123097 | HSPA8;HSP90AA1;HSP90AB1;HSPA1L;HSPA4;HSPD1;HSI |
| 0 | 23.89513861 | 345.4104961 | HSPA8;HSP90AA1;HSP90AB1;HSPA1L;FKBP4;HSPA1A |
| 0 | 37.55283019 | 443.6960181 | HSPA8;HSPA1L;HSPD1;HSPA1A |
| 0 | 14.68222222 | 122.1057896 | HSPA8;HSPA1L;TLR7;HSPA1A |
| 0 | 14.67627494 | 95.77212406 | HSPA8;HSPA1L;HSPA1A |
| 0 | 13.32311479 | 83.37178811 | HSPA8;HSPA1L;HSPA1A |
| 0 | 7.318587361 | 42.50984668 | STIP1;HSPA8;HSPA1L;HSPA1A |
| 0 | 10.66355811 | 60.20318423 | HSP90AA1;HSP90AB1;HMOX1 |
| 0 | 9.860129418 | 53.5639389 | HSPA8;HSPA1L;HSPA1A |
| 0 | 9.287054409 | 48.94403594 | HSP90AA1;HSP90AB1;PPID |
| 0 | 6.114096734 | 25.4502821 | HSP90AA1;HSP90AB1;PTGES3 |
| 0 | 5.791066706 | 23.29560684 | HSPA8;HSPA1L;HSPA1A |
| 0 | 10.2815735 | 41.19701054 | HSP90AA1;HSP90AB1 |
| 0 | 9.955388471 | 39.30248946 | HSP90AA1;HSP90AB1 |
| 0 | 9.649173955 | 37.54277574 | HSP90AA1;HSP90AB1 |
| 0 | 9.002721088 | 33.89043064 | HSP90AA1;HSP90AB1 |
| 0 | 4.944681921 | 17.90929615 | HSPA8;HSPA1L;HSPA1A |
| 0 | 5.542296919 | 16.07741891 | DNAJB1;TLR7 |
| 0 | 17.82647585 | 50.82895022 | ACOT7 |
| 0 | 17.82647585 | 50.82895022 | ACOT7 |
| 0 | 5.261239691 | 14.78858366 | HSP90AA1;HSP90AB1 |
| 0 | 14.47965116 | 38.47302036 | TIGAR |
| 0 | 11.57906977 | 28.35113154 | HMOX1 |
| 0 | 11.02657807 | 26.49659809 | HSPD1 |
| 0 | 11.02657807 | 26.49659809 | HMOX1 |
| 0 | 3.799691537 | 8.601534035 | HSP90AA1;HSP90AB1 |
| 0 | 9.448030375 | 21.34849709 | P4HA1 |
| 0 | 9.448030375 | 21.34849709 | PDIA4 |
| 0 | 2.692350333 | 5.916354752 | HSP90AA1;HSP90AB1;HMOX1 |
| 0 | 7.842727631 | 16.37418424 | HMOX1 |
| 0 | 7.711627907 | 15.98113875 | PTGES3 |
| 0 | 6.70273003 | 13.03144454 | TIGAR |
| 0 | 6.248271527 | 11.74962315 | PDIA4 |
| 0 | 5.926654741 | 10.86175725 | HSPD1 |
| 0 | 5.50166113 | 9.714702878 | NCK2 |
| 0 | 2.652056277 | 4.50692906 | HSP90AA1;HSP90AB1 |
| 0 | 4.482501693 | 7.099612841 | NCK2 |
| 0 | 4.482501693 | 7.099612841 | TLR7 |
| 0 | 4.273901809 | 6.590726271 | HMOX1 |
| 0 | 2.970892723 | 3.66001364 | PPID |
| 0 | 2.789429175 | 3.291575837 | CACYBP |
| 0 | 2.755744325 | 3.2244293 | HMOX1 |

| | | | |
|---|---|---|---|
| 0 | 2.739202658 | 3.191602316 | HSPA4 |
| 0 | 2.56944264 | 2.860451269 | HSPD1 |
| 0 | 2.540794038 | 2.805625525 | NCK2 |
| 0 | 2.445324097 | 2.62521349 | TLR7 |
| 0 | 2.344565733 | 2.438747281 | NCK2 |
| 0 | 1.985804893 | 1.810338655 | TLR7 |
| 0 | 1.871001424 | 1.622016378 | PPID |
| 0 | 1.478663355 | 1.032454406 | HMOX1 |
| 0 | 1.237866532 | 0.718428501 | PPID |
| 0 | 0.955843391 | 0.407192573 | PPID |

SPA1L;HSPA4L;PDIA4;HSPA1A

**Supplementary File S6: Comprehensive details of the results of functional enrichment analysis**

| BP | Term | Overlap | P-value |
|---|---|---|---|
| | positive regulation of transcription, DNA-templated (GO:0045893) | 68/1183 | 2.89E-14 |
| | positive regulation of transcription by RNA polymerase II (GO:0045944) | 54/908 | 4.93E-12 |
| | regulation of transcription by RNA polymerase II (GO:0006357) | 95/2206 | 5.01E-12 |
| | regulation of transcription, DNA-templated (GO:0006355) | 93/2244 | 7.43E-11 |
| | histone H3-K4 monomethylation (GO:0097692) | 44720 | 2.24E-09 |
| | histone H3-K4 methylation (GO:0051568) | 44834 | 7.06E-09 |
| | histone H3-K4 dimethylation (GO:0044648) | 44688 | 8.09E-08 |
| | negative regulation of transcription, DNA-templated (GO:0045892) | 46/948 | 1.16E-07 |
| | regulation of myeloid cell differentiation (GO:0045637) | 25143 | 1.61E-07 |
| | histone H3-K4 trimethylation (GO:0080182) | 44726 | 2.15E-07 |
| | positive regulation of nucleic acid-templated transcription (GO:1903508 | 30/511 | 4.56E-07 |
| | negative regulation of transcription by RNA polymerase II (GO:0000122) | 35/684 | 1.26E-06 |
| | regulation of megakaryocyte differentiation (GO:0045652) | 19968 | 1.68E-06 |
| | peptidyl-lysine trimethylation (GO:0018023) | 44772 | 2.30E-06 |
| | regulation of cellular macromolecule biosynthetic process (GO:2000112 | 27/468 | 2.41E-06 |
| | regulation of nucleic acid-templated transcription (GO:1903506) | 25/430 | 4.98E-06 |
| | peptidyl-lysine dimethylation (GO:0018027) | 44700 | 3.64E-05 |
| | regulation of intracellular signal transduction (GO:1902531) | 23/437 | 5.65E-05 |
| | histone lysine methylation (GO:0034968) | 12571 | 6.76E-05 |
| | regulation of gene expression (GO:0010468) | 42/1079 | 9.07E-05 |
| | cardiac cell development (GO:0055006) | 44664 | 1.18E-04 |
| | epiboly involved in wound healing (GO:0090505) | 44667 | 2.86E-04 |
| | phosphorylation (GO:0016310) | 20/400 | 3.33E-04 |
| | posttranscriptional regulation of gene expression (GO:0010608) | 16954 | 3.81E-04 |
| | skeletal myofibril assembly (GO:0014866) | 44628 | 4.76E-04 |
| | tRNA transcription (GO:0009304) | 44628 | 4.76E-04 |
| | regulation of cellular component organization (GO:0051128) | 10/139 | 7.20E-04 |
| | negative regulation of nucleic acid-templated transcription (GO:190350 | 21/464 | 8.74E-04 |
| | regulation of androgen receptor signaling pathway (GO:0060765) | 13271 | 8.79E-04 |
| | regulation of p38MAPK cascade (GO:1900744) | 13636 | 9.99E-04 |
| | monocarboxylic acid transport (GO:0015718) | 20972 | 0.001215459 |
| | regulation of transforming growth factor beta receptor signaling pathwa | 8/100 | 0.001223062 |
| | regulation of autophagy (GO:0010506) | 13/231 | 0.001257346 |
| | transcription by RNA polymerase II (GO:0006366) | 16/320 | 0.001280045 |
| | negative regulation of homotypic cell-cell adhesion (GO:0034111) | 44631 | 0.001338196 |
| | negative regulation of cellular macromolecule biosynthetic process (GO: | 23/547 | 0.00135002 |
| | cardiac muscle cell differentiation (GO:0055007) | 44675 | 0.001459879 |
| | wound healing, spreading of cells (GO:0044319) | 44675 | 0.001459879 |
| | positive regulation of p38MAPK cascade (GO:1900745) | 44675 | 0.001459879 |
| | regulation of alcohol biosynthetic process (GO:1902930) | 44632 | 0.001756589 |
| | negative regulation of gene silencing by miRNA (GO:0060965) | 44632 | 0.001756589 |
| | regulation of neurogenesis (GO:0050767) | 22798 | 0.001886051 |
| | protein phosphorylation (GO:0006468) | 21/496 | 0.001982124 |
| | regulation of actomyosin structure organization (GO:0110020) | 44633 | 0.002248187 |

| | | |
|---|---|---|
| microtubule bundle formation (GO:0001578) | 16558 | 0.002441803 |
| transforming growth factor beta receptor signaling pathway (GO:00071 | 32690 | 0.002692384 |
[truncated: 353,879 more chars]
